# Rh‐Catalyzed Chemodivergent Parallel Kinetic Resolution and Desymmetrization of Enynes and Dienynes with Acrylamides

**DOI:** 10.1002/anie.9965825

**Published:** 2026-03-15

**Authors:** Shintaro Hamada, Julong Jiang, Yu Sato, Takashi Yamazaki, Satoshi Maeda, Ken Tanaka

**Affiliations:** ^1^ Department of Chemical Science and Engineering Institute of Science Tokyo Meguro‐ku Tokyo Japan; ^2^ Institute for Chemical Reaction Design and Discovery (WPI‐ICReDD) Hokkaido University Sapporo Hokkaido Japan; ^3^ Department of Chemistry, Faculty of Science Hokkaido University Sapporo Hokkaido Japan

**Keywords:** 3D molecules, desymmetrization, enynes, fluorinated tertiary stereocenter, parallel kinetic resolution, rhodium

## Abstract

Catalytic enantioselective synthesis of structurally diverse 3D molecules remains a central challenge in organic chemistry. Intermolecular chemodivergent parallel kinetic resolution (PKR) of racemic substrates through C─C bond formation using a single catalyst to yield distinct products is rare. Here, we report Rh‐catalyzed chemodivergent PKR of racemic 1,6‐enynes with α‐fluoroacrylamides or mixtures of different acrylamides, proceeding via [2+2+2] cycloaddition and C–H alkylation to afford structurally and stereochemically distinct products with high selectivity under mild conditions. Additionally, substituent‐dependent enantioselective desymmetrization of symmetric dienynes was achieved. Computational studies revealed that, depending on the stereochemistry of the 1,6‐enyne, steric repulsion between the ligand and the enyne favors a compact C–H activation transition state, whereas its absence favors a C═C insertion transition state involving a smaller bond dissociation energy. These findings establish a versatile catalytic platform for the selective generation of 3D molecular complexity, advancing diversity‐oriented synthesis from racemic or symmetric precursors.

## Graphical abstract

Rh‐catalyzed chemodivergent parallel kinetic resolution of racemic 1,6‐enynes with α‐fluoroacrylamides or mixtures of different acrylamides proceeded via [2+2+2] cycloaddition and C–H alkylation to afford structurally and stereochemically distinct products with high selectivity under mild conditions. Furthermore, substituent‐dependent enantioselective desymmetrization of symmetric dienynes was also achieved.

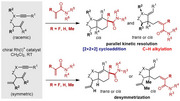

## 1 Introduction

The development of structurally diverse 3D molecular libraries, particularly those rich in sp^3^‐hybridized carbon centers, is a major goal in medicinal chemistry due to their potential to yield novel bioactive compounds [1]. To meet this challenge, a variety of catalytic enantioselective reactions have been established to efficiently access single enantiomers with central chirality, which serve as key intermediates for constructing chiral 3D molecules. Alternatively, catalytic kinetic resolution (KR) of racemic 3D compounds can provide both the transformed product and the remaining starting material in enantiomerically enriched forms, though each is limited to a maximum theoretical yield of 50% [2]. In contrast, parallel kinetic resolution (PKR) with a single catalyst allows both enantiomers of a racemate to undergo different reaction pathways in one operation, greatly increasing the structural diversity of the resulting chiral 3D products [3, 4, 5, 6, 7, 8]. Consequently, catalytic PKR has emerged as a promising strategy for the efficient synthesis of diverse chiral molecular libraries.

**FIGURE 1:**
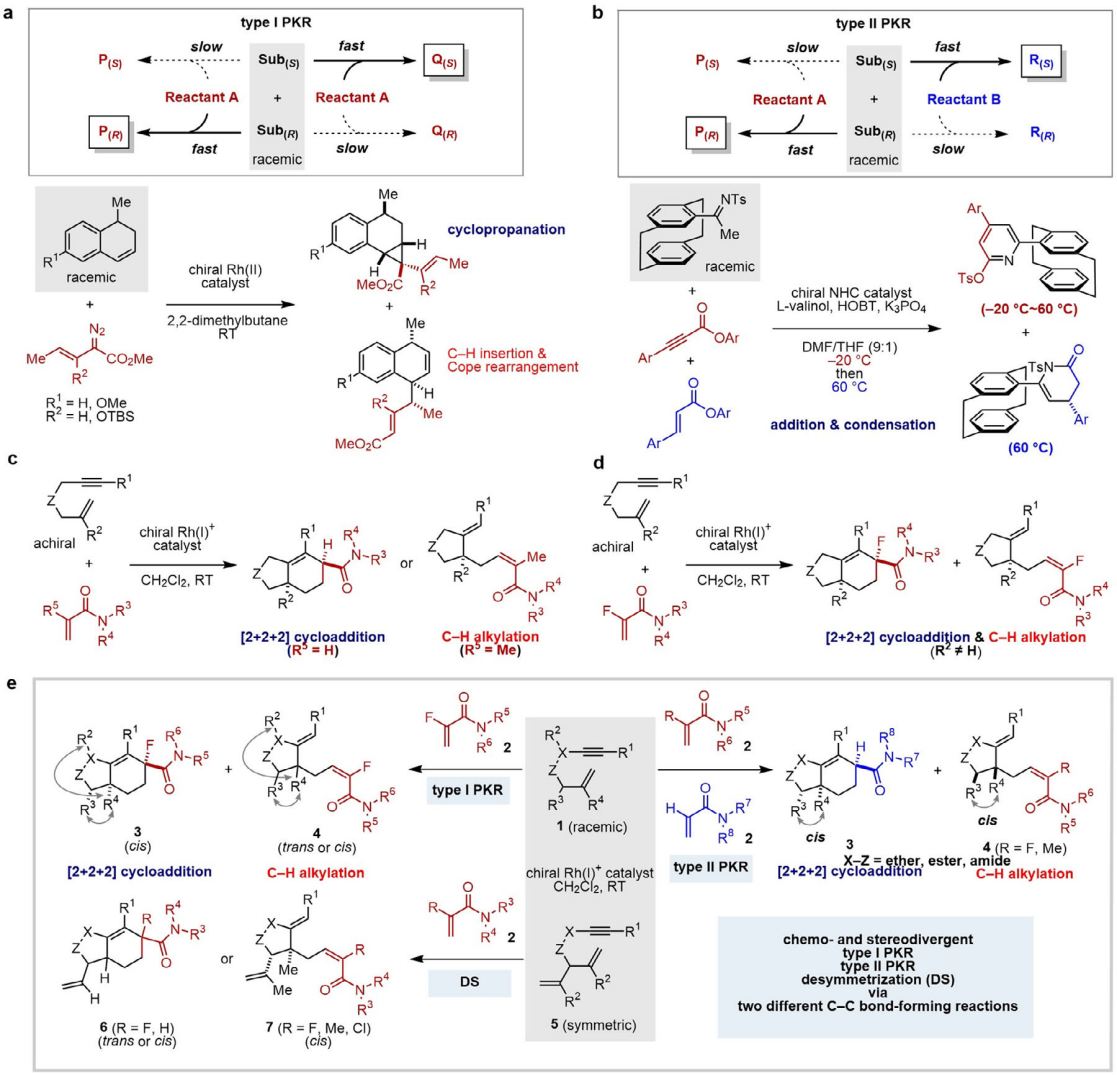
Catalytic enantioselective parallel kinetic resolution (PKR) via intermolecular C─C bond‐forming reactions. (a) Type I PKR via two different intermolecular C─C bond‐forming reactions (Davies in 2005 and 2010, refs [19] and [20]). (b) Type II PKR via the same intermolecular C─C bond‐forming reactions (Jin in 2024, ref [22]). (c) Rh‐catalyzed enantioselective [2+2+2] cycloaddition and C–H alkylation of 1,6‐enynes with acrylamide derivatives (our group in 2012 and 2024, refs [23] and [30], respectively). (d) Rh‐catalyzed enantioselective [2+2+2] cycloaddition and C–H alkylation of 1,6‐enynes with α‐fluoroacrylamides (our group in 2025, ref [34]). (e) Rh‐catalyzed chemo‐ and stereodivergent type I and II PKR and desymmetrization (DS) of 1,6‐enynes with acrylamide derivatives via two different intermolecular C─C bond‐forming reactions (this work). Sub = substrate, NHC = *N*‐heterocyclic carbene, HOBT = 1‐hydroxybenzotriazole.

Among various PKR strategies, intermolecular C─C bond‐forming PKR enables structural diversification by altering the reaction partners [9, 10, 11, 12, 13, 14, 15, 16, 17, 18]. In a more advanced approach, intermolecular C─C bond‐forming chemodivergent PKR represents an even more attractive strategy for the structural diversification of chiral 3D molecules. This strategy can be categorized into two types: type I, which affords two distinct products **P_(_ *_R_*_)_** and **Q_(_ *_S_*_)_** from racemic substrates **Sub_(_ *_R_*_)_** and **Sub_(_ *_S_*_)_** reacting with **Reactant A** (Figure 1a, top) [19, 20] and type II, which affords two distinct products **P_(_ *_R_*_)_** and **R_(_ *_S_*_)_** from racemic substrates **Sub_(_ *_R_*_)_** and **Sub_(_ *_S_*_)_** reacting with a mixture of **Reactant A** and **Reactant B** (Figure 1b, top) [21, 22]. Both generate chemically distinct products from one or two distinct achiral coupling partners, respectively. Despite their potential, only one example of each type has been reported to date. In 2005, Davies reported a type I PKR via Rh(II)‐catalyzed cyclopropanation and C–H insertion/Cope rearrangement, while the method was applicable to only a limited number of examples (Figure 1a, bottom) [19, 20]. More recently, in 2024, Jin reported a type II PKR, while the method employs the same *N*‐heterocyclic carbene (NHC)‐catalyzed addition/condensation and requires chiral and achiral additives [L‐valinol and 1‐hydroxybenzotriazole (HOBT), respectively] and a stepwise increase in reaction temperature for attaining better chemoselectivity (Figure 1b, bottom) [22].

Enantioselective transition‐metal‐catalyzed [2+2+2] cycloadditions of achiral 1,6‐enynes with alkenes have been reported as a valuable approach for synthesizing chiral 3D cyclic compounds [23, 24, 25, 26, 27, 28]. In 2012, we reported enantioselective [2+2+2] cycloaddition [29] with strongly coordinating acrylamides, yielding chiral cyclohexene derivatives (Figure 1c) [23]. In 2024, we found that acrylamides bearing alkyl or phenyl substituents at the α‐position undergo enantioselective C–H alkylation [30, 31, 32, 33] instead of cycloaddition (Figure 1c) [30]. Subsequently, we discovered that α‐fluoroacrylamides undergo both enantioselective [2+2+2] cycloaddition and C–H alkylation when the alkene moiety of the enyne is 1,1‐disubstituted (Figure 1d) [34]. Here we report the type I PKR of racemic 1,6‐enynes **1** with α‐fluoroacrylamides **2** using a chiral cationic Rh(I) catalyst at room temperature via intermolecular chemodivergent [14, 22, 35, 36, 37, 38] as well as stereodivergent [12, 18, 39, 40, 41, 42, 43, 44, 45, 46, 47, 48, 49] C─C bond‐forming reactions, affording two chemically and stereochemically distinct products. One enantiomer forms medicinally valuable [2+2+2] cycloaddition product **3** featuring a fluorinated tertiary stereocenter [50, 51] and *cis* configuration, while the other undergoes C–H alkylation to give product **4** with either *trans* or *cis* configuration (Figure 1e, top left). Additionally, the chemodivergent type II PKR using two different acrylamide derivatives also proceeds with high selectivity (Figure 1e, top right). We further reveal that using symmetric dienynes **5**, bearing two identical alkene moieties, undergo two distinct enantioselective desymmetrization reactions [52, 53, 54, 55, 56, 57, 58], delivering products **6** or **7** with different diastereoselectivities depending on the alkene substituent (Figure 1e, bottom left).

## 2 Results and Discussion

**FIGURE 2:**
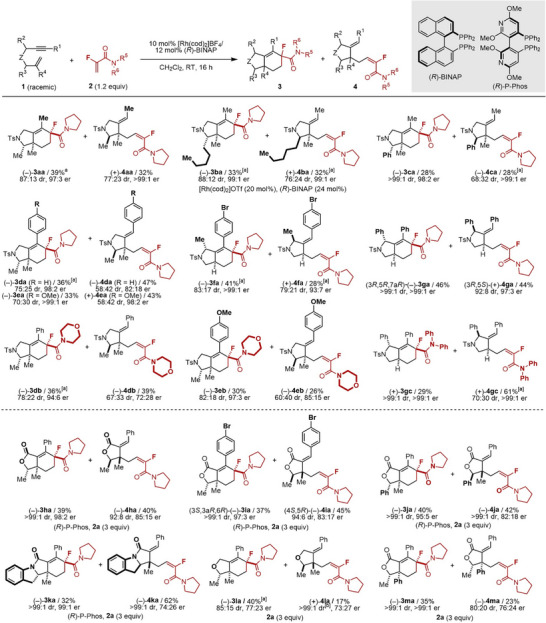
Rh‐catalyzed enantioselective PKR (type I) of racemic 1,6‐enynes with α‐fluoroacrylamides. Cited yields and dr values are of isolated products. Structures and er values of major diastereomers are shown. **1** (0.05–0.10 mmol), **2** (0.06–0.15 mmol), [Rh(cod)_2_]BF_4_ or [Rh(cod)_2_]OTf (0.005–0.020 mmol), ligand (0.006–0.024 mmol), and CH_2_Cl_2_ (1.0–2.0 mL) were used. [a] Two diastereomers were isolated separately. [b] A trace amount (ca. <2%) of another diastereomer was detected in a crude reaction mixture. er = enantiomeric ratio, dr = diastereomeric ratio.

In our previous report, the reactions of 1,6‐enynes with α‐fluoroacrylamides afforded both [2+2+2] cycloaddition and C–H alkylation products [34]. This observation led us to hypothesize that type I PKR could occur when using racemic 1,6‐enynes bearing central chirality (Figure 2, top). Thus, we screened a series of axially chiral biaryl bisphosphine ligands in the reaction of racemic tosylamide‐linked 1,6‐enyne **1a** bearing allylic substituents with α‐fluoroacrylamide **2a** (Table S1), which revealed that type I PKR proceeds efficiently using (*R*)‐BINAP, wherein the *S*‐ and *R*‐enantiomers of **1a** gave the cycloadduct **3aa** and the C–H alkylation product **4aa**, respectively, in good yields and stereoselectivity. NOESY analysis confirmed that **3aa** and **4aa** have *cis* and *trans* relationships between the R^3^ and R^4^ methyl groups, respectively, supporting chemo‐ and stereodivergent PKR. For **1b** (R^3^ = pentyl), defluorination was observed, but using Rh(cod)_2_OTf/(*R*)‐BINAP suppressed this side reaction, affording **3ba** and **4ba**. 1,6‐Enyne **1c** (R^3^ = Ph) gave **3ca** with excellent stereoselectivity. The reaction of **1d** and **1e** (R^1^ = aryl) with **2a** afforded **3da/3ea** and **4da**/**4ea** with high enantioselectivity but lower diastereoselectivity than that using **1a** (R^1^ = Me). We also explored racemic 1,6‐enynes with propargylic substituents. 1,6‐Enyne **1f** (R^2^ = Me) with **2a** afforded **3fa** in high yield and stereoselectivity, along with **4fa** in moderate yield but good stereoselectivity. For **1g** (R^2^ = Ph), both **3ga** and **4ga** were obtained in excellent yields and stereoselectivity. For the amide substituents, the use of morpholine amide **2b** improved the diastereoselectivity for **3db** and **3eb**. Using *N,N*‐diphenylamide **2c**, PKR also occurred; however, **4gc** was preferentially formed, giving a moderate yield of **3gc** and reduced diastereoselectivity for **4gc**. X‐ray crystallographic analysis of (–)‐**3ga** and (+)‐**4ga** revealed their absolute configurations as (3*R*,5*R*,7a*R*) and (3*R*,5*S*), respectively (Figures S2 and S3), confirming chemo‐ and stereodivergent PKR for propargyl‐substituted 1,6‐enynes.

We further evaluated the effect of the linker moieties of the 1,6‐enynes (Figure 2, bottom). Ester‐linked 1,6‐enynes **1h**–**1j** required 3 equiv of **2a** but provided excellent yields and stereoselectivity when (*R*)‐P‐Phos was used (ligand screening: Table S2). Amide‐linked 1,6‐enyne **1k**, bearing an indole unit, also underwent PKR to give tetracyclic (**3ka**) and tricyclic (**4ka**) products using (*R*)‐P‐Phos. Ether‐linked 1,6‐enyne **1l** afforded **3la** and **4la** using 3 equiv of **2a** in moderate yield and enantioselectivity, but with improved diastereoselectivity relative to the other linker variants. For **1m** (R^2^ = Ph), mainly one enantiomer reacted, resulting in products **3ma** and **4ma**, which had identical absolute configurations, as confirmed by the reaction using (+)‐**1m** (Figure S1). X‐ray crystal structure analyses of (–)‐**3ia** and (±)‐**4ia** determined their absolute configuration to be (3*S*,3a*R*,6R) (Figure S4) and (4*S*,5*R*) (Figure S5), indicating *cis* selectivity, which contrasts with the outcomes observed for tosylamide‐linked 1,6‐enynes. NOESY spectra of **3ka**, **4ka**, **3la**, and **4la** consistently showed *cis* configurations of major products.

**FIGURE 3:**
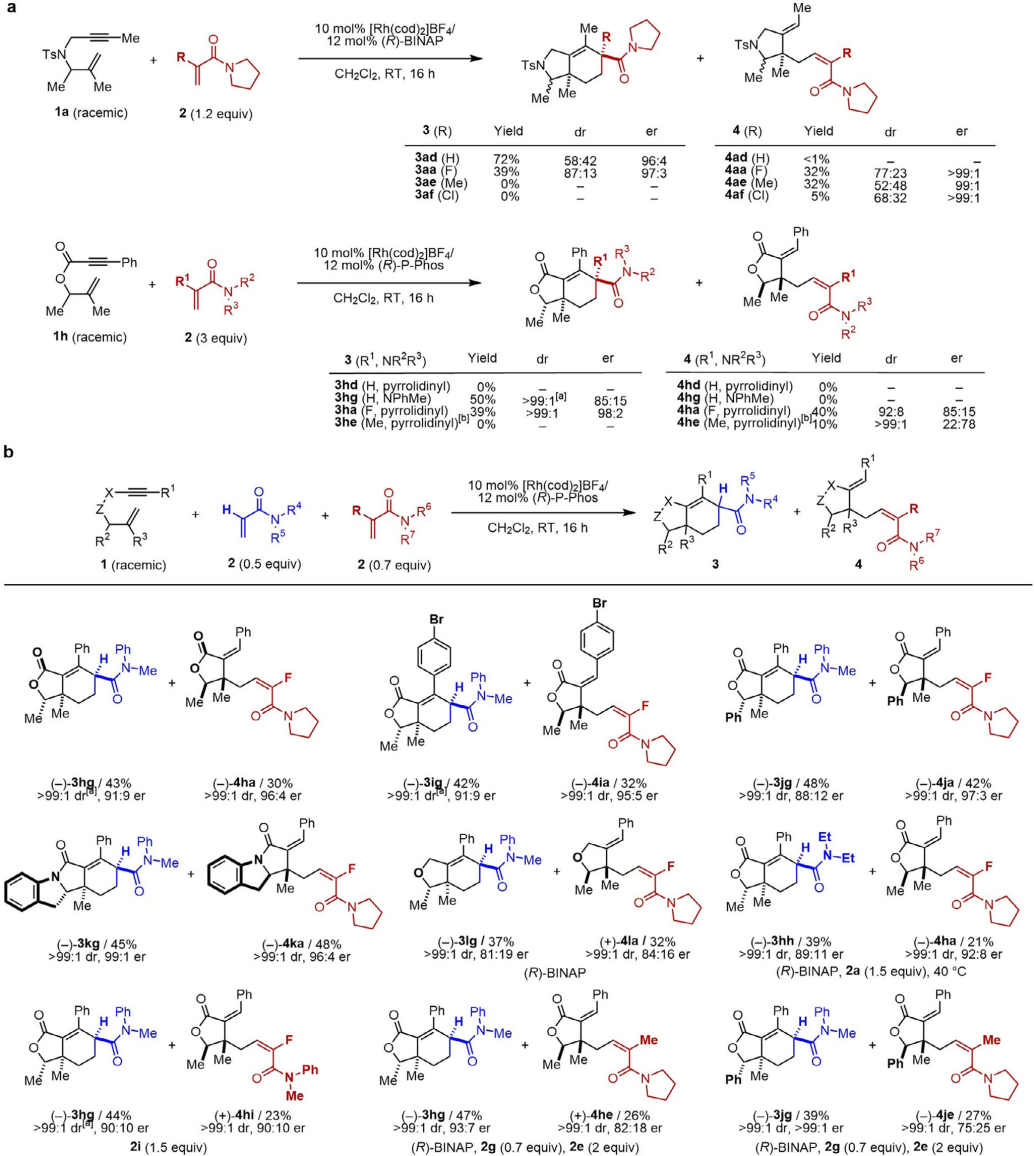
Rh‐catalyzed enantioselective PKR (type II) of racemic 1,6‐enynes with two different acrylamide derivatives. (a) Substituent effect at α‐position of acrylamides; (b) PKR (type II) using two different acrylamide derivatives. Cited yields and dr values are of isolated products. Structures and er values of major diastereomers are shown. **1** (0.10 mmol), **2** (0.05–0.20 mmol), [Rh(cod)_2_]BF_4_ (0.010 mmol), ligand (0.012 mmol), and CH_2_Cl_2_ (2.0 mL) were used. [a] A trace amount (ca. <2%) of another diastereomer was detected in a crude reaction mixture. [b] (*R*)‐BINAP was used instead of (*R*)‐P‐Phos.

We next examined the type II PKR because substituents in the acrylamides markedly change the chemoselectivity between [2+2+2] cycloaddition and C–H alkylation. The reactions of racemic tosylamide‐linked 1,6‐enyne **1a** with acrylamides bearing H (**2d**), F (**2a**), Me (**2e**), or Cl (**2f**) at the α‐position were conducted at room temperature using the Rh(I)^+^/(*R*)‐BINAP catalyst to examine the influence of α‐substituents on chemo‐, diastereo‐, and enantioselectivity. Thus, we conducted the reactions of racemic tosylamide‐linked 1,6‐enyne **1a** with acrylamides bearing H (**2d**), F (**2a**), Me (**2e**), or Cl (**2f**) at the α‐position using the Rh(I)^+^/(*R*)‐BINAP or (*R*)‐P‐Phos catalyst at room temperature (Figure 3a, top). When using **2d**, [2+2+2] cycloaddition exclusively proceeded to afford **3ad** in high yield and enantioselectivity, albeit with low diastereoselectivity. In contrast, using **2e** or **2f** resulted in the exclusive formation of C–H alkylation products **4ae** or **4af** with low diastereoselectivity. We also conducted the reactions of racemic ester‐linked 1,6‐enyne **1h** with acrylamides **2d**, **2g, 2a**, and **2e** (Figure 3a, bottom). Although no reaction was observed with pyrrolidine‐derived acrylamide **2d**, presumably due to its high coordination ability, the use of *N*‐methylaniline‐derived acrylamide **2g** exclusively led to the [2+2+2] cycloaddition, affording **3hg** in 50% yield with high diastereoselectivity, albeit in moderate enantioselectivity. In contrast, the use of **2e** resulted in the exclusive formation of C–H alkylation product **4he** with excellent diastereoselectivity, albeit with moderate enantioselectivity. In both 1,6‐enynes **1a** and **1h**, α‐fluoroacrylamide **2a** stereoselectively affords **3aa**/**3ha** and **4aa**/**4ha** via type I PKR.

On the basis of these results, racemic ester‐linked 1,6‐enyne **1h**, rather than racemic tosylamide‐linked 1,6‐enyne **1a**, was identified as a suitable substrate for the type II PKR. Accordingly, we succeeded in the type II PKR of racemic **1h** with a mixture of acrylamides **2g** and **2a** using the Rh(I)^+^/(*R*)‐P‐Phos catalyst at room temperature, affording **3hg** and **4ha** in 43% and 30% yields, respectively, with high stereoselectivity (Figure 3b). Similarly, racemic ester‐linked 1,6‐enynes **1i** and **1j,** amide‐linked 1,6‐enyne **1k**, and ether‐linked 1,6‐enyne **1l** were suitable substrates for the type II PKR. Particularly, **1k** reacted with **2g** and **2a** to give **3kg** and **4ka** with the highest chemo‐ and stereoselectivities. Furthermore, diethylamine‐derived acrylamide **2h** and *N*‐methylaniline‐derived α‐fluoroacrylamide **2i** could equally be employed instead of *N*‐methylaniline‐derived acrylamide **2g** and pyrrolidine‐derived α‐fluoroacrylamide **2a**, respectively. Finally, the combination of **2g** and methacrylamide **2e** was examined, which revealed that the desired type II PKR of racemic ester‐linked 1,6‐enynes **1h** and **1j** proceeded to give **3hg**/**3je** and **4hg**/**4je** with excellent diastereoselectivity; however, enantioselectivity of C–H alkylation products **4hg**/**4je** was low.

**FIGURE 4:**
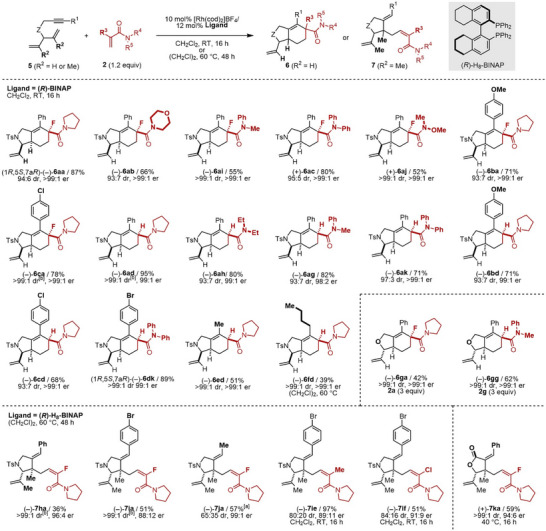
Rh‐catalyzed enantioselective DS of symmetric dienynes with acrylamide derivatives. Cited yields and dr values are of isolated products. Structures and er values of major diastereomers are shown. **1** (0.05–0.10 mmol), **2** (0.06–0.15 mmol), [Rh(cod)_2_]BF_4_ (0.005–0.010 mmol), ligand (0.006–0.012 mmol), and CH_2_Cl_2_ (1.0–2.0 mL) or (CH_2_Cl)_2_ (1.0 mL) were used. [a] Two diastereomers were isolated separately. [b] A trace amount (ca. < 2%) of another diastereomer was detected in a crude reaction mixture.

We have previously reported that the alkene substituents on 1,6‐enynes affect the reaction outcomes with α‐fluoroacrylamides [34]. In the absence of an alkene substituent, only the cycloadduct is obtained, whereas in its presence, both the cycloadduct and the C–H alkylation product are formed (Figure 1d). On the basis of this finding and the present PKR results, we anticipated that symmetric dienynes **5**, bearing two identical alkene units [53, 54, 55, 56, 57, 58], would undergo an unprecedented substituent‐dependent, chemodivergent, and enantioselective desymmetrization to afford cycloadducts **6** or the C–H alkylation products **7** (Figure 4). Gratifyingly, tosylamide‐linked dienyne **5a** reacted with **2a** at room temperature using (*R*)‐BINAP to afford cycloadduct **6aa** in high yield with excellent enantio‐ and diastereoselectivity. Other α‐fluoroacrylamides **2b**, **2i**, **2c**, and **2j** were also compatible, affording **6ab**, **6ai**, **6ac**, and **6aj** with high stereoselectivity, although in slightly reduced yields. Dienynes bearing electron‐donating (**5b**) or ‐withdrawing (**5c**) aryl groups at the alkyne terminus gave **6ba** and **6ca** smoothly. X‐ray crystallographic analysis of (–)‐**6aa** confirmed its absolute configuration as (1*R*,5*S*,7a*R*) (Figure S6), indicating preferential formation of the *trans*‐isomer. In contrast, **5g** afforded *cis*‐isomer **6ga**; this linker‐dependent diastereomeric inversion is consistent with the results in the Rh‐catalyzed Pauson‐Khand‐type reaction [57, 58]. This desymmetrization strategy was also applied to non‐fluorinated acrylamides **2d**, **2h**, **2g**, and **2k**, affording **6ad**, **6ah**, **6ag**, and **6ak** in high yields with excellent stereoselectivity. Dienynes **5b**–**5d** with various aryl substituents reacted efficiently, while alkyl‐substituted dienynes **5e** (Me) and **5f** (Bu) gave moderate yields but retained excellent stereoselectivity. X‐ray crystallographic analysis of (–)‐**6dk** showed the same (1*R*,5*S*,7a*R*) configuration as **6aa** (Figure S7). Ether‐linked dienyne **5g** also provided **6ga** and **6gg** with excellent stereoselectivity using 3 equiv of **2a** and **2g**.

In contrast, tosylamide‐linked dienynes **5h**–**5j** with methyl‐substituted alkenes underwent enantioselective C–H alkylation via desymmetrization at 60°C using (*R*)‐H_8_‐BINAP. Dienynes **5h** and **5i** (R^1^ = Ph and 4‐Br‐C_6_H_4_) reacted with **2a** to give **7ha** and **7ia**, respectively, with moderate yields and good stereoselectivity, while **5j** (R^1^ = Me) showed lower diastereoselectivity. This method was extended to methacrylamide **2e** and α‐chloroacrylamide **2f**, producing **7ie** and **7if**, respectively, at room temperature. Ester‐linked dienyne **5k** provided **7ka** with high stereoselectivity at 40°C. NOESY analysis of **7ia** confirmed a *cis* relationship of the product.

**FIGURE 5:**
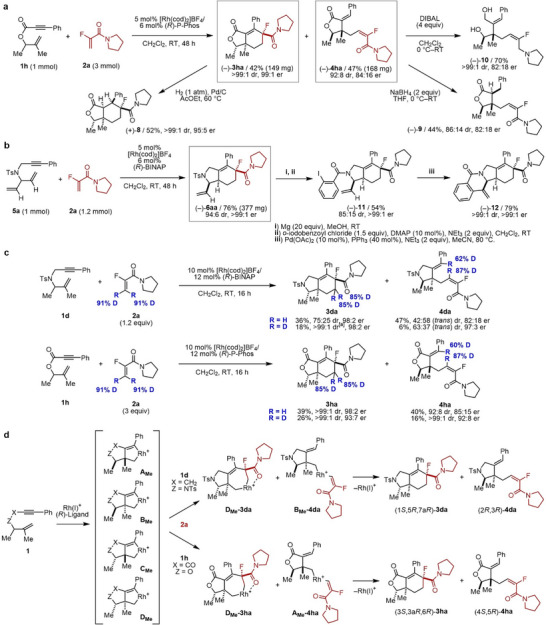
Synthetic applications (a and b) and experimental mechanistic studies (c and d). (a) Synthetic applications for PKR; (b) synthetic applications for DS; (c) deuterium‐labeling studies; (d) putative reaction mechanisms. Cited yields and dr values are of isolated products. Structures and er values of major diastereomers are shown. DIBAL = diisobutylaluminium hydride, DMAP = 4‐dimethylaminopyridine. [a] A trace amount (ca. <2%) of another diastereomer was detected in a crude reaction mixture.

To demonstrate the synthetic utility, we explored preparative‐scale reactions and subsequent transformations. A 1 mmol‐scale PKR of 1,6‐enyne **1h** with **2a** under 5 mol% Rh catalyst afforded (–)‐**3ha** and (–)‐**4ha** in high yields and stereoselectivity (Figure 5a). Hydrogenation of (–)‐**3ha** using a Pd/C catalyst afforded cyclohexane (+)‐**8** bearing five contiguous stereocenters in 52% yield as a single diastereomer. Reduction of (–)‐**4ha** with NaBH_4_ provided the corresponding alkene‐reduced product (–)‐**9** in 44% yield. In contrast, treatment with DIBAL induced ring‐opening and amide reduction, affording linear aminodiol (–)‐**10** in good yield. Dienyne **5a** was also applicable on a 1 mmol scale, producing (–)‐**6aa** in high yield and excellent stereoselectivity (Figure 5b). Deprotection of the tosyl group followed by amidation afforded (–)‐**11**, which underwent a Pd‐catalyzed intramolecular Heck reaction to give tetracyclic product (–)‐**12**.

**FIGURE 6:**
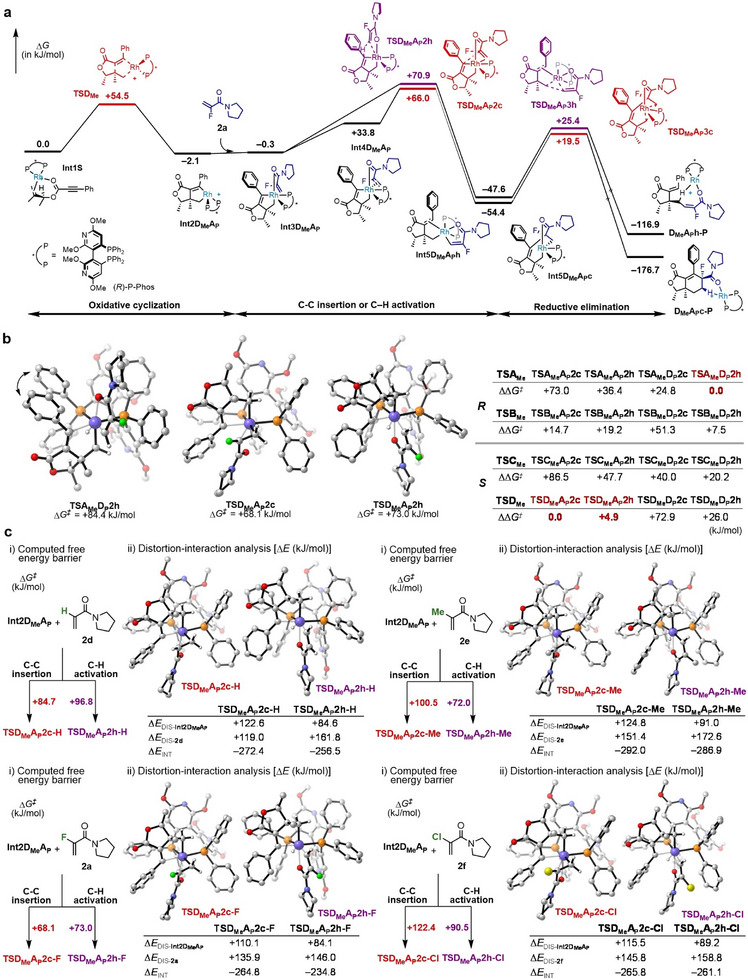
Theoretical mechanistic studies. (a) Computed free energy profile of PKR reaction of 1,6‐enyne **1h** and acrylamide **2a** through rhodacycle **D_Me_A_P_**, which is derived from (*S*)‐**1h**, at the B3LYP‐D3/Def2‐TZVPPD/IEFPCM(DCM)//B3LYP‐D3/Def2‐SVP/IEFPCM(DCM) level of theory; (b) ΔΔ*G* ^≠^ values computed using the most stable TS conformers, from which relative rate constants can be derived; SC‐AFIR (GFN‐xTB) used for conformational sampling of C–C insertion/C–H activation TSs, followed by re‐optimization at DFT level [same as (a)]; (c) Influence of α‐substituents on acrylamides, calculated at the B3LYP‐D3/Def2‐TZVPPD/IEFPCM(DCM)//B3LYP‐D3/Def2‐SVP/IEFPCM(DCM) level of theory.

To gain mechanistic insight into the present PKR reactions, deuterium labeling studies were conducted using deuterated α‐fluoroacrylamide **2a‐D_2_** (Figure 5c). Reactions of 1,6‐enynes **1d** and **1h** with **2a‐D_2_** showed more pronounced yield decreases for the C–H alkylation products **4da‐D_2_** (6%) and **4ha‐D_2_** (16%) compared to those of the [2+2+2] cycloaddition products **3da‐D_2_** (18%) and **3ha‐D_2_** (26%). Notably, the stereoselectivities of both pathways were generally improved. Furthermore, the yields of the C–H alkylation products **4da‐D_2_** and **4ha‐D_2_** were markedly reduced, indicating a primary isotope effect favoring C–H cleavage over C–D cleavage. These results suggest that C–H cleavage is the rate‐determining step. The yields of the [2+2+2] cycloadducts **3da‐D_2_** and **3ha‐D_2_** were also slightly reduced, indicating a secondary isotope effect. In these reactions, the bulky 1,6‐enyne **1d** was recovered unreacted, but the reaction with **1h** significantly increased the yield of the homo‐[2+2+2] cycloadduct (NMR yield < 1% using **2a** and 19% using **2a‐D_2_**), indicating that the coordination ability of **2a‐D_2_** to Rh is lower than that of **2a**. Putative reaction mechanisms are shown in Figure 5d (see Figure 6 for detailed analysis using DFT calculations). The five‐membered rhodacycle generated from racemic 1,6‐enyne **1** and a Rh(I)^+^/(*R*)‐biaryl bisphosphine complex exists as four isomers, **A_Me_**, **B_Me_**, **C_Me_**, and **D_Me_**, depending on the configuration of the Me groups. For tosylamide‐linked 1,6‐enyne **1d**, formation of (1*S*,5*R*,7a*R*)‐**3da** and (2*R*,3*R*)‐**4da** indicated that **D_Me_‐3da** [23] and **B_Me_‐4da** [30] are appropriate intermediates and thus, **D_Me_** and **B_Me_** are appropriate rhodacycles, respectively. In contrast, for ester‐linked 1,6‐enyne **1h**, only isomers **D_Me_** and **A_Me_** yield products (3*S*,3a*R*,6*R*)‐**3ha** and (4*S*,5*R*)‐**4ha**, respectively, through intermediates **D_Me_‐3ha** and **A_Me_‐4ha**.

Extensive efforts have been made to elucidate the mechanisms and origins of enantioselectivity in the cyclization reactions of 1,6‐enynes, including the Rh‐catalyzed Pauson–Khand reaction, the Rh‐catalyzed [2+2+2] cycloaddition with alkynes [59, 60, 61, 62, 63], and Co‐catalyzed C–H alkylation [64]. However, a comprehensive conformational search using the single‐component AFIR (SC‐AFIR) method, as well as mechanistic studies on the Rh‐catalyzed [2+2+2] cycloaddition with alkenes, have not yet been conducted. Thus, we performed the comprehensive computational study on the Rh(I)^+^/(*R*)‐P‐Phos‐catalyzed PKR of ester‐linked 1,6‐enyne (*R*)‐**1h** or (*S*)‐**1h** and α‐fluoroacrylamide **2a** using the AFIR method [65] (Figures 6a and S9–S12). The computed free energy profile through rhodacycle **D_Me_**, formed from (*S*)‐**1h** (Figures 5d and S8), represents the most energetically favorable pathway (Figure 6a). It begins with coordination of (*S*)‐**1h** to Rh(I)^+^/(*R*)‐P‐Phos, yielding **Int1S**. The subsequent oxidative cyclization proceeds quickly with a barrier of only +54.5 kJ/mol. Notably, our calculations indicate that the cyclization step is a fast, reversible equilibrium. The positions of the P atom attached to the Rh center in the rhodacycle yield four isomers: **A_P_**, **B_P_**, **C_P_**, and **D_P_** (Figure S8). Among them, only **A_P_** and **D_P_** are suitable for the subsequent reactions, as coordination of the amide oxygen is crucial when the C═C moiety of the incoming **2a** reacts with the sp^2^‐C atom. Here, intermediate **Int2D_Me_A_P_**, which possesses two vacant coordination sites, immediately interacts with the incoming **2a**, which coordinates firmly to the Rh(III) center through its nucleophilic carbonyl oxygen and C═C bond. The reaction path then bifurcates at this stage: one leads to C–C insertion through **TSD_Me_A_P_2c**, and the other leads to C–H activation via **TSD_Me_A_P_2h**. Here, the final letter indicates whether it is C–C insertion (**c**) or C–H activation (**h**). In this case, **TSD_Me_A_P_2c** is 4.9 kJ/mol lower in energy than **TSD_Me_A_P_2h**. It is clear from the energy profile that neither insertion nor C–H activation is reversible under room temperature, therefore serving as the enantio‐determining step. The corresponding intermediates **Int5D_Me_A_P_c** and **Int5D_Me_A_P_h** subsequently undergo reductive elimination to yield the two distinct products (3*S*,3a*R*,6*R*)‐**3ha** and (4*S*,5*R*)‐**4ha** observed experimentally (Figure 3a). In contrast, for (*R*)‐**1h**, the most energetically favorable pathway via rhodacycle **A_Me_** reveals that the C–H activation transition state **TSA_Me_D_P_2h** is substantially lower in energy (24.8 kJ/mol) than the C–C insertion transition state **TSA_Me_D_P_2c**, yielding (4*R*,5*S*)‐**4ha** observed experimentally (Figure S9, bottom).

As observed in Path **D_Me_A_P_**, the second step (either insertion or C–H activation) is irreversible and determines the enantioselectivity. Accordingly, we performed conformational sampling of the relevant transition states using the SC‐AFIR method at the GFN‐xTB [66] level of theory. The lowest‐energy conformers were further optimized at the density functional theory (DFT) level (see caption of Figure 6 for detailed methods). Considering the spatial arrangement of the two methyl groups and the diphosphine ligand, as well as the mechanistic divergence between C─C bond formation and C–H activation, a total of 16 possible transition states were identified and subjected to conformational analysis to afford optimized structures, revealing strong π–π interactions within (*R*)‐P‐Phos ligand (Figures 6b, S13, and S14, and Tables S10 and S11). In this context, the energy difference between **TS2c** and **TS2h** substantially influences the reaction kinetics, affecting the ratio of the resulting products. Given racemic **1h**, the (*R*)‐**1h** has eight different pathways, with the C–H activation pathway via **TSA_Me_D_P_2h** identified as the most favored (Figure S9, bottom, and Table S10 and Figure S13). For (*S*)‐**1h**, the pathways through **TSD_Me_A_P_2c** and **TSD_Me_A_P_2h** are competitive, with ΔΔ*G* ^≠^ values of 0.0 and +4.9 kJ/mol, respectively (Table S11 and Figure S14). Thus, it is evident that the C–C insertion pathway for **1h** yields enantiopure **3ha**, whereas the C–H activation pathway yields **4ha** as a mixture of enantiomers, which is in close agreement with the experimental result (**3ha**: 98:2 er, **4ha**: 85:15 er, Figure 3a).

To summarize the computational results obtained using 1,6‐enyne (*R*)‐**1h** or (*S*)‐**1h**, α‐fluoroacrylamide **2a**, and the Rh(I)^+^/(*R*)‐P‐Phos catalyst, after the formation of the rhodacyclopentene intermediates that avoid steric repulsion between the methyl group at the chiral center of **1h** and the ligand's equatorial phenyl group, the following trends were observed. When steric repulsion exists between the ligand's equatorial phenyl group and the phenyl group of (*R*)‐**1h**, as indicated by the double arrow in **TSA_Me_D_P_2h (**Figure 6b), the compact C–H activation transition state **TSA_Me_D_P_2h** (Figure 6a), in which **2a** approaches the Rh center from the sterically less demanding terminal position rather than across the C = C bond, is favored. In contrast, when there is no steric repulsion between the ligand's equatorial phenyl group and the phenyl group of (*S*)‐**1h**, the C═C insertion transition state **TSD_Me_A_P_2c** (Figure 6a), which involves a smaller bond dissociation energy than that of the C–H activation pathway, becomes more favorable.

The substituents on the acrylamides also markedly influence the chemoselectivity, and thus additional calculations were performed for the reactions of intermediate **Int2D_Me_A_P_** with acrylamide **2** bearing various α‐substituents (Figures 6c and S15). Replacing H (**2d**) with Me (**2e**) markedly suppresses C–C insertion, likely due to steric hindrance, as supported by distortion‐interaction analysis. In contrast, replacing F (**2a**) with Cl (**2f**) suppressed the C–C insertion pathway and favored C–H activation. Distortion–interaction analysis revealed that the sum of Δ*E*_DIS_ values for each transition state is larger for the C–C insertion pathway, indicating that, from the standpoint of distortion energy, C–H activation is more favorable. On the other hand, comparison of Δ*E*_INT_ values shows that, when fluoroacrylamide **2a** is employed, there is a substantial difference in stabilization energy between **TSD_Me_A_P_2c‐F** (−264.8 kJ/mol) and **TSD_Me_A_P_2h‐F** (−234.8 kJ/mol). This difference likely accounts for the lower activation energy observed for the C–C insertion pathway with **2a**. Collectively, these results suggest that the observed selectivity originates from the unique electronic properties of fluorine, demonstrating that the electronic effect of the α‐substituent plays a crucial role in governing the chemoselectivity of the reaction. The difference in chemoselectivity among the α‐substituents underlies the realization of type II PKR.

## 3 Conclusion

In summary, we have developed chemodivergent PKR reactions of racemic 1,6‐enynes using a single chiral cationic Rh(I) catalyst. This transformation proceeds via two distinct intermolecular C─C bond‐forming pathways, [2+2+2] cycloaddition and C–H alkylation, by employing either a single α‐fluoroacrylamide or two different acrylamide derivatives, affording structurally and stereochemically distinct products with high selectivity under mild conditions. Mechanistic investigations reveal that both chemo‐ and stereoselectivity underlying type I and type II PKR reactions originate from (i) divergent transformations of a common rhodacyclopentene intermediate, governed by the substituent attached to the chiral center of the 1,6‐enyne, and (ii) difference in chemoselectivity among the α‐substituents of the acrylamides. Furthermore, Rh‐catalyzed enantioselective desymmetrization of symmetric dienynes bearing two identical alkenes was achieved in substituent‐dependent, chemodivergent manners, delivering diastereomerically distinct products. These results establish a versatile catalytic platform for the selective generation of 3D molecular complexity, opening new avenues for diversity‐oriented synthesis using racemic or achiral precursors. Whereas previous examples of PKR have largely relied on serendipity, the mechanistic factors governing chemoselectivity revealed in this study may inform the rational design and development of future PKR reactions.

## Conflicts of Interest

The authors declare no conflicts of interest.

## Supporting information



Materials and methods, synthetic experiments, computational studies, NMR spectra, chiral HPLC charts, references, Figures S1–S15, and Tables S1–S11. Deposition numbers 2480941 for (3*R*,5*R*,7a*S*)‐(–)‐**3ga**, 2480945 for (3*R*,5*S*)‐(+)‐**4ga**, 2480942 for (3*S*,3a*R*,6*R*)‐(–)‐**3ia**, 2480939 for (±)‐**4ia**, 2480946 for (1*R*,5*S*,7a*R*)‐(–)‐**6aa**, and 2480943 for (1*R*,5*S*,7a*R*)‐(–)‐**6dk** contain the supplementary crystallographic data for this paper. These data can be obtained free of charge via www.ccdc.cam.ac.uk/data_request/cif or data_request@ccdc.cam.ac.uk. by emailing.
**Supporting File 1**: anie71834‐sup‐0001‐SuppMat.pdf.

**Supporting File 2**: anie71834‐sup‐0002‐SuppMat.xyz.

## Data Availability

The data that supports the findings of this study are available in the Supporting Information of this article.
