# Supplementary material for: Rh‐Catalyzed Chemodivergent Parallel Kinetic Resolution and Desymmetrization of Enynes and Dienynes with Acrylamides
Source: Angew Chem Int Ed Engl. 2026 Mar 15;65(17):e9965825. doi: 10.1002/anie.9965825 (PMC13098305; doi:10.1002/anie.9965825)
Supplement: Supplementary file 1 — Materials and methods, synthetic experiments, computational studies, NMR spectra, chiral HPLC charts, references, Figures S1–S15, and Tables S1–S11. Deposition numbers 2480941 for (3R,5R,7aS)‐(–)‐3ga, 2480945 for (3R,5S)‐(+)‐4ga, 2480942 for (3S,3aR,6R)‐(–)‐3ia, 2480939 for (±)‐4ia, 2480946 for (1R,5S,7aR)‐(–)‐6aa, and 2480943 for (1R,5S,7aR)‐(–)‐6dk contain the supplementary crystallographic data for this paper. These data can be obtained free of charge via www.ccdc.cam.ac.uk/data_request/cif or data_request@ccdc.cam.ac.uk. by emailing. Supporting File 1: anie71834‐sup‐0001‐SuppMat.pdf. [file ANIE-65-e9965825-s002.pdf]

Table of Contents

1. General Information **S2**
1.1. General Experimental Information S2
1.2. General Analytical Information S2

2. Synthetic Experiments **S3**
2.1. Synthesis of Substrates S3
2.1.1. Synthesis of 1,6-Enynes S3
2.1.2. Synthesis of Acrylamides S13
2.2. Optimization of Reaction Conditions S14
2.3. Rh-Catalyzed Enantioselective PKR (type I) of Racemic 1,6-Enynes with α-Fluoroacrylamides S16
2.4. Rh-Catalyzed Enantioselective PKR (type II) of Racemic 1,6-Enynes with Two Different Acrylamide Derivatives S35
2.4.1. Substituent Effect at α-Position of Acrylamides S35
2.4.2. PKR (type II) Using Two Different Acrylamide Derivatives S39
2.5. Rh-Catalyzed Enantioselective Desymmetrization of Achiral Dienynes with Acrylamide Derivatives S46
2.6. Synthetic Applications S59
2.6.1. Synthetic Applications for Parallel Kinetic Resolution S59
2.6.2. Synthetic Applications for Desymmetrization S62
2.7. Experimental Mechanistic Studies S65
2.7.1. Deuterium-Labeling Studies S65

3. Single-Crystal X-Ray Diffraction Analysis **S68**

4. Theoretical Calculations **S81**
4.1. Computational Methods S81
4.2. Computational Energies of All Optimized Structures S83
4.3. Computational Studies for Reaction Pathways (Path A_{Me}, B_{Me}, C_{Me}, and D_{Me}) S85
4.4. Results of Conformational Sampling of Transition States of C–C Insertion and C–H Activation Steps S89
4.5. Influence of α-Substituents on Acrylamides S91

5. References **S92**

6. ^{1}H, ^{13}C, and ^{19}F NMR Spectra **S94**

7. Chiral HPLC Charts **S258–S346**

1. General Information

1.1. General Experimental Information

Anhydrous and degassed CH_2Cl_2 (No. 041-32345, Wako) and $(CH_2Cl)_2$ (No. 28450-5, Aldrich) were used as received. Other solvents used in this work were dried over molecular sieves 4Å or 3Å (Wako) before use. Et_3N for the synthesis of substrates was dried over KOH (Kanto Chemicals) before use.

$[Rh(cod)_2]BF_4$ was synthesized from $[RhCl(cod)]_2$ according to the published literature.[1] (*R*)-H_8-BINAP, (*R*)-tol-BINAP, (*R*)-xyl-BINAP, and (*R*)-Segphos were obtained from Takasago International Corporation. Methyl 2-fluoroacrylate was purchased from BLD Pharmatech Ltd. Lipase PS "Amano" IM was obtained from Amano Enzyme Inc. All other reagents were purchased from TCI Chemicals, Wako Pure Chemical Industries, Sigma-Aldrich, and Kanto Chemicals and used as received.

Silica gel column chromatography was performed using silica gel [Silica Gel 60 N (spherical, neutral), Kanto Chemicals] and JIS (Japanese Industrial Standards) special grade solvents. Silica gel preparative thin layer chromatography (PTLC) was performed using silica gel (Wakogel® B-5F) and JIS special grade solvents.

All reactions were carried out under an atmosphere of argon (Ar) or nitrogen (N_2) in oven-dried glassware with magnetic stirring.

All racemic products for chiral HPLC analysis were synthesized using (*rac*)-BINAP as a ligand.

1.2. General Analytical Information

^{1}H, ^{13}C, and ^{19}F NMR data were collected on Bruker AVANCE III HD 400 at ambient temperature. All ^{1}H NMR experiments are reported in δ units, parts per million (ppm), and were measured relative to the signal for residual chloroform (7.26 ppm). All ^{13}C NMR spectra are reported in ppm relative to deuterochloroform (77.0 ppm), and were obtained with ^{1}H decoupling. All ^{19}F NMR spectra are reported in ppm relative to benzotrifluoride (–64.0 ppm), and were obtained with ^{1}H decoupling. HRMS data were obtained on a Bruker micrOTOF Focus II. Melting points were determined on a Mettler MP30.

Chiral HPLC analyses were carried out on a Jasco LC-2000Plus Series system using Daicel CHIRALPAK® and CHIRALCEL® columns (internal diameter 4.6 mm, column length 250 mm, and particle size 3 or 5 μm). Optical rotation data were obtained on a Jasco P-2200 digital polarimeter with the sodium D line (589 nm) at ambient temperature.

2. Synthetic Experiments

2.1. Synthesis of Substrates

2.1.1. Synthesis of 1,6-Enynes

Substrates **5a**,[2] **5b**,[3] **5e**,[2] **S1a**,[4] **S1b**,[5] **S1c**,[6] **S1d**,[7] **S2a**,[8] **S2b**,[9] **S2c**,[10] **S2d**,[11] **S3b**,[12] **S5**,[13] **S7a**,[2] **S7b**,[14] **S7c**,[15] **S8a**,[3] and **S8b**[15] were synthesized according to the literature.

***N*-(But-2-yn-1-yl)-4-methyl-*N*-(3-methylbut-3-en-2-yl)benzenesulfonamide (1a)**

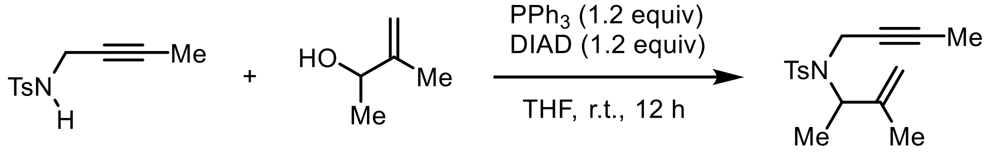

S1a **S2a** **1a**

Diisopropyl azodicarboxylate (DIAD, 1.213 g, 6.00 mmol) was added to a solution of 3-methylbut-3-en-2-ol (**S2a**, 430.7 mg, 5.00 mmol), *N*-(but-2-yn-1-yl)-4-methyl-benzenesulfonamide (**S1a**, 1.117 g, 5.00 mmol), and PPh_3 (1.574 g, 6.00 mmol) in THF (25.0 mL) under N_2. After stirring at room temperature for 12 h, the reaction mixture was concentrated. The residue was dissolved in EtOAc and washed with water and brine. The obtained organic layers were dried over Na_2SO_4, filtered, and concentrated. The residue was stirred in *n*-hexane/EtOAc = 10:1 (20.0 mL) for 30 min and filtered, and the filtrate was concentrated. The crude product was purified by silica gel column chromatography (eluent: *n*-hexane/EtOAc = 4:1) to furnish **1a** (427.7 mg, 1.47 mmol, 29% yield).

White solid; mp 56.8–58.6 °C; ^{1}H NMR (400 MHz, $CDCl_3$) δ 7.85–7.82 (m, 2H), 7.28 (d, J = 8.0 Hz, 2H), 5.02–5.00 (m, 1H), 4.93 (s, 1H), 4.42 (q, J = 6.9 Hz, 1H), 4.09 (dq, J = 18.2, 2.3 Hz, 1H), 3.66 (dq, J = 18.2, 2.4 Hz, 1H), 2.42 (s, 3H), 1.682–1.676 (m, 3H), 1.63 (t, J = 2.4 Hz, 3H), 1.19 (d, J = 7.0 Hz, 3H); ^{13}C NMR (101 MHz, $CDCl_3$) δ 144.1, 142.9, 138.3, 129.0, 127.6, 113.6, 79.9, 75.1, 57.2, 32.2, 21.4, 21.1, 16.0, 3.3; HRMS (ESI) calcd for $C_{16}H_{21}NNaO_2S$ $[M+Na]^+$ 314.1185, found 314.1181.

***N*-(But-2-yn-1-yl)-4-methyl-*N*-(2-methyloct-1-en-3-yl)benzenesulfonamide (1b)**

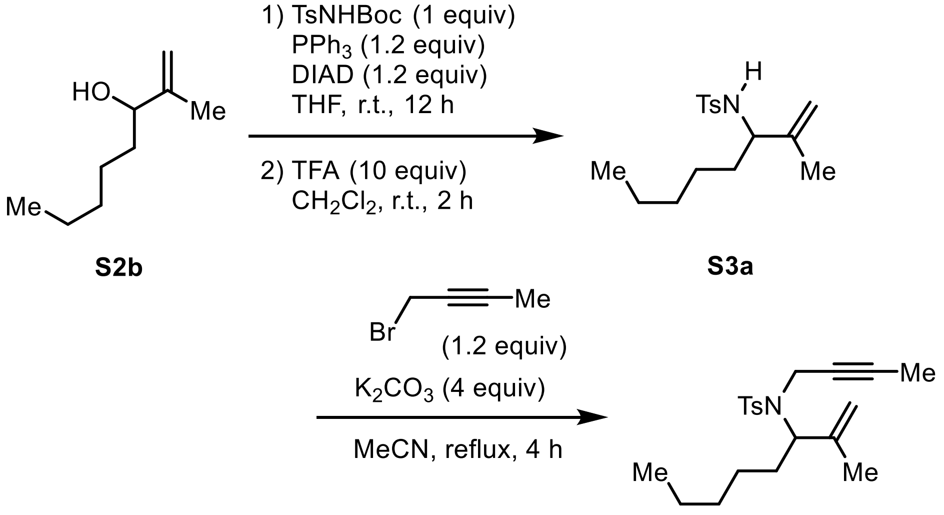

1b

Diisopropyl azodicarboxylate (DIAD, 1.213 g, 6.00 mmol) was added to a solution of 2-methyloct-1-en-3-ol (**S2b**, 712.2 mg, 5.00 mmol), TsNHBoc (1.357 g, 5.00 mmol), and PPh_3 (1.574 g, 6.00 mmol) in THF (25.0 mL) under N_2. After stirring at room temperature for 12 h, the reaction

mixture was concentrated, and the residue was dissolved in EtOAc and washed with water and brine. The organic layers were dried over Na_2SO_4, filtered, and concentrated. The residue was stirred in *n*-hexane/EtOAc = 10:1 (20.0 mL) and filtered, and the filtrate was concentrated. To a solution of the crude product in CH_2Cl_2 (12.5 mL) was added trifluoroacetic acid (TFA, 3.83 mL, 50.0 mmol) at room temperature. After stirring at room temperature for 2 h, the reaction was quenched with saturated aqueous $NaHCO_3$, and the aqueous layer was extracted with EtOAc three times. The combined organic layers were washed with water and brine, dried over Na_2SO_4, and concentrated. The crude product was purified by silica gel column chromatography (eluent: *n*-hexane/EtOAc = 4:1) to furnish *N*-(2-methyloct-1-en-3-yl)-(4-methylbenzene)sulfonamide **(S3a,** 824.4 mg, 0.279 mmol, 56% yield).

Yellow oil; ^{1}H NMR (400 MHz, $CDCl_3$) δ 7.73–7.70 (m, 2H), 7.26 (d, *J* = 7.9 Hz, 2H), 4.74–4.73 (m, 1H), 4.72–4.70 (m, 1H), 4.70–4.40 (br m, 1H), 3.70 (q, *J* = 7.3 Hz, 1H), 2.42 (s, 3H), 1.49 (dd, *J* = 1.3, 0.8 Hz, 3H), 1.47–1.41 (m, 2H), 1.27–1.13 (m, 6H), 0.83 (t, *J* = 6.9 Hz, 3H); ^{13}C NMR (101 MHz, $CDCl_3$) δ 143.4, 143.1, 137.9, 129.4, 127.2, 113.3, 59.6, 33.9, 31.3, 25.2, 22.4, 21.5, 17.4, 13.9; HRMS (ESI) calcd for $C_{16}H_{25}NNaO_2S$ $[M+Na]^+$ 318.1498, found 318.1492.

1-Bromobut-2-yne (498.7 mg, 3.75 mmol) was added to a MeCN (12.5 mL) solution of K_2CO_3 (1.382 g, 10.0 mmol) and **S3a** (0.739 g, 2.50 mmol) at room temperature, and the mixture was refluxed for 4 h. The reaction was quenched with aqueous NH_4Cl, and the crude mixture was extracted with EtOAc, washed with brine, dried over Na_2SO_4, and concentrated. The residue was purified by silica gel column chromatography (eluent: *n*-hexane/EtOAc = 4:1) to furnish **1b** (821.6 mg, 2.36 mmol, 95% yield).

Yellow oil; ^{1}H NMR (400 MHz, $CDCl_3$) δ 7.82 (d, *J* = 8.3 Hz, 2H), 7.26 (d, *J* = 8.0 Hz, 2H), 5.02 (q, *J* = 1.2 Hz, 1H), 4.92 (s, 1H), 4.25 (t, *J* = 7.4 Hz, 1H), 4.10 (dq, *J* = 18.3, 2.3 Hz, 1H), 3.69 (dq, *J* = 18.4, 2.4 Hz, 1H), 2.42 (s, 3H), 1.68 (s, 3H), 1.52–1,49 (m, 1H), 1.32–1.24 (m, 1H), 1.21–1.11 (m, 6H), 1.63 (t, *J* = 2.4 Hz, 3H), 0.81 (t, *J* = 6.9 Hz, 3H); ^{13}C NMR (101 MHz, $CDCl_3$) δ 142.8, 142.6, 138.4, 128.9, 127.7, 113.9, 79.5, 75.0, 62.1, 32.3, 31.6, 30.3, 26.7, 22.4, 21.8, 21.4, 13.8, 3.2; HRMS (ESI) calcd for $C_{20}H_{29}NNaO_2S$ $[M+Na]^+$ 370.1811, found 370.1815.

N-(But-2-yn-1-yl)-4-methyl-*N*-(2-methyl-1-phenylprop-2-en-1-yl)benzenesulfonamide (1c)

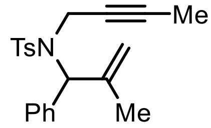

1c (649.5 mg, 1.84 mmol, 92% yield) was prepared from 4-methyl-*N*-(2-methyl-1-phenylprop-2-en-1-yl)benzenesulfonamide (**S3b**, 602.8 mg, 2.00 mmol) and 1-bromobut-2-yne (399.0 mg, 3.00 mmol) according to the procedure for **1b**.

Pale yellow oil; ^{1}H NMR (400 MHz, $CDCl_3$) δ 7.74 (d, *J* = 8.3 Hz, 2H), 7.23–7.20 (m, 5H), 7.13–7.10 (m, 2H), 5.54 (s, 1H), 5.12 (q, *J* = 1.3 Hz, 1H), 4.93 (s, 1H), 3.98 (q, *J* = 2.3 Hz, 1H), 3.97 (q, *J* = 2.3 Hz, 1H), 2.40 (s, 3H), 1.69 (s, 3H), 1.51 (t, *J* = 2.4 Hz, 3H); ^{13}C NMR (101 MHz, $CDCl_3$) δ 142.8, 142.3, 137.9, 136.6, 129.0, 128.9, 128.1, 127.63, 127.58, 115.8, 79.9, 74.4, 66.4, 35.0, 21.41, 21.37, 3.2; HRMS (ESI) calcd for $C_{21}H_{23}NNaO_2S$ $[M+Na]^+$ 376.1342, found 376.1341.

4-Methyl-*N*-(3-methylbut-3-en-2-yl)-*N*-(3-phenylprop-2-yn-1-yl)benzenesulfonamide (1d)

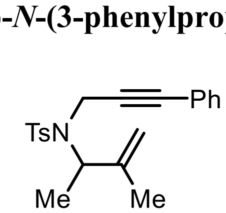

1d (526.6 mg, 1.49 mmol, 30% yield) was prepared from 4-methyl-*N*-(3-phenylprop-2-yn-1-yl)-benzenesulfonamide (**S1b**, 1.427 g, 5.00 mmol) and **S2a** (430.7 mg, 5.00 mmol) according to the procedure for **1a**.

White solid; mp 114.5–116.5 °C; ^{1}H NMR (400 MHz, $CDCl_3$) δ 7.87 (d, *J* = 8.3 Hz, 2H), 7.30–7.24 (m, 3H), 7.20 (d, *J* = 8.0 Hz, 2H), 7.19–7.15 (m, 2H), 5.06 (q, *J* = 1.3 Hz, 1H), 5.00 (s, 1H), 4.52 (q, *J* = 6.8 Hz, 1H), 4.39 (d, *J* = 18.6 Hz, 1H), 3.95 (d, *J* = 18.6 Hz, 1H), 2.35 (s, 3H), 1.75 (s, 3H), 1.27 (d, *J* = 7.0 Hz, 3H); ^{13}C NMR (101 MHz, $CDCl_3$) δ 144.1, 143.0, 138.3, 131.3, 129.3, 128.2, 128.1, 127.7, 122.8, 114.0, 85.5, 84.1, 57.4, 32.6, 21.4, 21.3, 16.2; HRMS (ESI) calcd for $C_{21}H_{23}NNaO_2S$ $[M+Na]^+$ 376.1342, found 376.1342.

***N*-{3-(4-Methoxyphenyl)prop-2-yn-1-yl}-4-methyl-*N*-(3-methylbut-3-en-2-yl)-benzenesulfonamide (1e)**

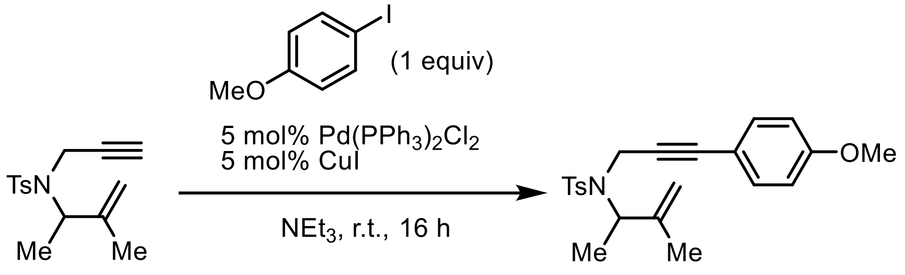

S4 **1e**

4-Methyl-*N*-(3-methylbut-3-en-2-yl)-*N*-(prop-2-yn-1-yl)benzenesulfonamide (**S4**, 147.9 mg, 0.533 mmol, 36% yield) was prepared from 4-methyl-*N*-(prop-2-yn-1-yl)benzenesulfonamide (**S1c**, 314.4 mg, 1.50 mmol) and **S2a** (129.2 mg, 1.50 mmol) according to the procedure for **1a**.

Coloress oil; ^{1}H NMR (400 MHz, $CDCl_3$) δ 7.83 (d, *J* = 8.3 Hz, 2H), 7.28 (d, *J* = 8.0 Hz, 2H), 5.04–4.96 (m, 1H), 4.96 (s, 1H), 4.41 (q, *J* = 6.9 Hz, 1H), 4.14 (dd, *J* = 18.4, 2.4 Hz, 1H), 3.73 (dd, *J* = 18.5, 2.5 Hz, 1H), 2.42 (s, 3H), 2.10 (t, *J* = 2.5 Hz, 1H), 1.66 (s, 3H), 1.22 (d, *J* = 6.9 Hz, 3H); ^{13}C NMR (101 MHz, $CDCl_3$) δ 143.7, 143.2, 137.9, 129.3, 127.6, 114.1, 80.0, 72.2, 57.3, 31.7, 21.4, 21.2, 16.1; HRMS (ESI) calcd for $C_{15}H_{19}NNaO_2S$ $[M+Na]^+$ 300.1029, found 370.1039.

To a solution of 4-iodoanisole (140.4 mg, 0.600 mol) and **S4** (138.7 mg, 0.500 mol) in Et_3N (5.0 mL) were added $PdCl_2(PPh_3)_2$ (14.6 mg, 0.0250 mmol) and CuI (4.8 mg, 0.025 mmol) under Ar. The mixture was stirred at room temperature for 16 h. The resulting mixture was filtered and concentrated. The crude product was purified by silica gel column chromatography (eluent: *n*-hexane/EtOAc = 4:1) to furnish **1e** (149.1 mg, 0.389 mmol, 78% yield).

Yellow solid; mp 99.4–100.9 °C; ^{1}H NMR (400 MHz, $CDCl_3$) δ 7.86 (d, *J* = 8.3 Hz, 2H), 7.20 (d, *J* = 8.0 Hz, 2H), 7.13–7.10 (m, 2H), 6.81–6.77 (m, 2H), 5.05 (q, *J* = 1.2 Hz, 1H), 4.99 (s, 1H), 4.50 (q, *J* = 6.9 Hz, 1H), 4.37 (d, *J* = 18.5 Hz, 1H), 3.94 (d, *J* = 18.5 Hz, 1H), 3.80 (s, 3H), 2.36 (s, 3H), 1.74 (s, 3H), 1.26 (d, *J* = 7.0 Hz, 3H); ^{13}C NMR (101 MHz, $CDCl_3$) δ 159.5, 144.1, 143.0, 138.3, 132.7, 129.3, 127.6, 114.9, 113.9, 113.8, 84.05, 83.95, 57.3, 55.3, 32.7, 21.4, 21.3, 16.1; HRMS (ESI) calcd for $C_{22}H_{25}NNaO_3S$ $[M+Na]^+$ 406.1447, found 406.1432.

***N*-{4-(4-Bromophenyl)but-3-yn-2-yl}-4-methyl-*N*-(prop-2-en-1-yl)benzenesulfonamide (1f)**

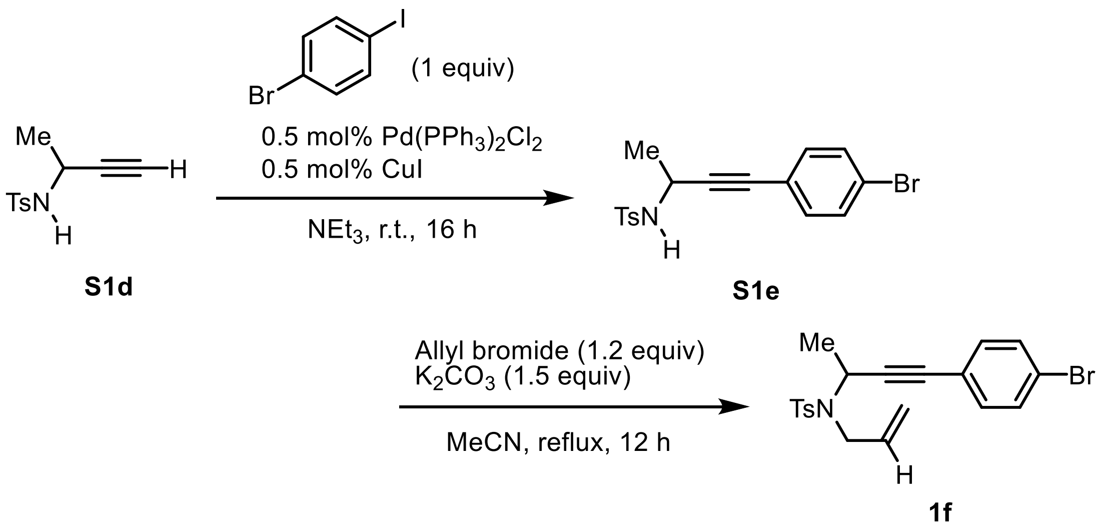

To a solution of 1-bromo-4-iodobenzene (1.698 g, 6.00 mmol) and *N*-(but-3-yn-2-yl)-4-methylbenzenesulfonamide (**S1d,** 1.117 g, 5.00 mmol) in Et_3N (10.0 mL) were added $PdCl_2(PPh_3)_2$ (17.6 mg, 0.0250 mmol) and CuI (4.8 mg, 0.0250 mmol) under Ar, and the mixture was stirred at room temperature for 16 h. The reaction mixture was filtered and concentrated, and the crude product was purified by silica gel column chromatography (eluent: *n*-hexane/EtOAc = 2:1) to furnish **S1e** (1.608 g, 4.25 mmol, 85% yield).

White solid; mp 123.5-125.0 °C; ^{1}H NMR (400 MHz, $CDCl_3$) δ 7.82–7.79 (m, 2H), 7.37–7.34 (m, 2H), 7.24 (d, *J* = 8.0 Hz, 2H), 6.93–6.90 (m, 2H), 4.85 (d, *J* = 9.0 Hz, 1H), 4.41 (dq, *J* = 9.0, 7.0 Hz, 1H), 2.33 (s, 3H), 1.49 (d, *J* = 7.0 Hz, 3H); ^{13}C NMR (101 MHz, $CDCl_3$) δ 143.5, 137.4, 132.9, 131.3, 129.5, 127.5, 122.6, 121.1, 89.2, 82.7, 41.9, 23.4, 21.4; HRMS (ESI) calcd for $C_{17}H_{16}BrNNaO_2S$ $[M+Na]^+$ 399.9977, found 399.9971.

Allyl bromide (435.5 mg, 3.60 mmol) was added to a solution of K_2CO_3 (621.9 mg, 4.50 mmol) and **S1e** (1.135 g, 3.00 mmol) in MeCN (10.0 mL) at room temperature, and the mixture was refluxed for 12 h. The reaction was quenched with aqueous NH_4Cl and extracted with EtOAc three times. The cobined organic layers were washed with brine, dried over Na_2SO_4 and concentrated. The residue was purified by silica gel column chromatography (eluent: *n*-hexane/EtOAc = 4:1) to furnish **1f** (820.3 mg, 1.96 mmol, 65% yield).

White solid; mp 82.4-83.9 °C; ^{1}H NMR (400 MHz, $CDCl_3$) δ 7.76 (d, *J* = 8.3 Hz, 2H), 7.37 (d, *J* = 8.6 Hz, 2H), 7.26 (d, *J* = 8.0 Hz, 2H), 6.94 (d, *J* = 8.5 Hz, 2H), 5.95 (dddd, *J* = 17.1, 10.3, 6.8, 5.0 Hz, 1H), 5.29 (ddd, *J* = 17.1, 3.1, 1.4 Hz, 1H), 5.15 (ddd, *J* = 10.2, 2.8, 1.3 Hz, 1H), 5.08 (q, *J* = 7.1 Hz, 1H), 3.98 (ddt, *J* = 16.6, 4.4, 1.8 Hz, 1H), 3.76 (ddt, *J* = 16.5, 6.7, 1.3 Hz, 1H), 2.36 (s, 3H), 1.50 (d, *J* = 7.1 Hz, 3H); ^{13}C NMR (101 MHz, $CDCl_3$) δ 143.3, 136.3, 135.8, 132.8, 131.4, 129.4, 127.7, 122.6, 121.1, 117.0, 87.9, 83.9, 47.5, 46.9, 22.4, 21.4; HRMS (ESI) calcd for $C_{20}H_{20}BrNNaO_2S$ $[M+Na]^+$ 440.0290, found 440.0296.

***N*-Allyl-*N*-(1,3-diphenylprop-2-yn-1-yl)-4-methylbenzenesulfonamide (1g)**

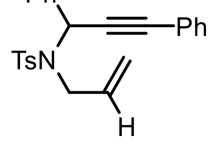

1g (470.9 mg, 1.17 mmol, 23% yield) was prepared from 1,3-diphenylprop-2-yn-1-ol (1.041 g, 5.00 mmol) according to the procedure for **1f**.

White solid; mp 97.9–98.4 °C; ^{1}H NMR (400 MHz, CDCl3) δ 7.85 (d, *J* = 8.3 Hz, 2H), 7.65 (d, *J* = 7.2 Hz, 2H), 7.39–7.26 (m, 8H), 7.19–7.16 (m, 2H), 6.29 (s, 1H), 5.53–5.43 (m, 1H), 4.86 (ddd, *J* = 17.1, 2.8, 1.4 Hz, 1H), 4.80–4.76 (m, 1H), 3.82–3.77 (m, 1H), 3.74–3.68 (m, 1H), 2.37 (s,

3H); ^{13}C NMR (101 MHz, $CDCl_3$) δ 143.4, 136.4, 136.3, 134.4, 131.5, 129.5, 128.6, 128.35, 128.33, 128.31, 128.2, 127.9, 122.1, 116.9, 88.4, 83.6, 54.1, 48.1, 21.4; HRMS (ESI) calcd for $C_{25}H_{23}NNaO_2S$ $[M+Na]^+$ 424.1342, found 424.1340.

3-Methylbut-3-en-2-yl 3-phenylpropiynoate (1h)

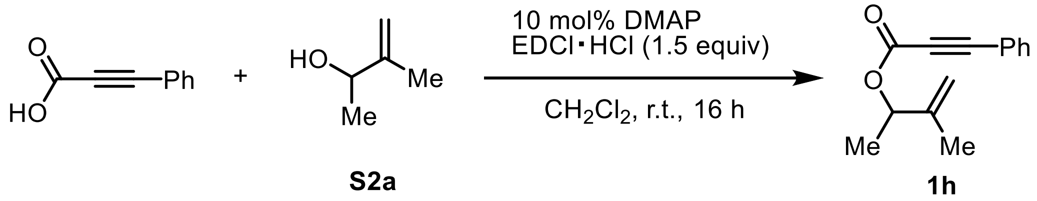

To a solution of 3-phenylpropynoic acid (1.461 g, 10.0 mmol) in CH_2Cl_2 (50.0 mL) were added alcohol **S2a** (861.3 mg, 10.0 mmol), EDCI·HCl (2.876 g, 15.0 mmol) and DMAP (122.1 mg, 1.00 mmol) at 0 °C, and the mixture was stirred at room temperature for 16 h, The reaction was quenched with aqueous NH_4Cl, and the crude mixture was extracted with EtOAc, washed with brine, dried over Na_2SO_4, and concentrated. The crude product was purified by sílica gel column chromatography (eluent: *n*-hexane/EtOAc = 4:1) to furnish **1h** (631.3 mg, 2.95 mmol, 29% yield).

Colorless oil; ^{1}H NMR (400 MHz, $CDCl_3$) δ 7.60–7.57 (m, 2H), 7.46–7.42 (m, 1H), 7.38–7.34 (m, 2H), 5.43 (q, J = 6.5 Hz, 1H), 5.05 (s, 1H), 4.93–4.91 (m, 1H), 1.80 (s, 3H), 1.42 (d, J = 6.6 Hz, 3H); ^{13}C NMR (101 MHz, $CDCl_3$) δ 153.3, 143.6, 132.9, 130.5, 128.5, 119.7, 112.5, 86.0, 80.9, 75.4, 18.9, 18.2; HRMS (ESI) calcd for $C_{14}H_{14}NaO_2$ $[M+Na]^+$ 237.0886, found 237.0883.

3-Methylbut-3-en-2-yl 3-(4-bromophenyl)propynoate (1i)

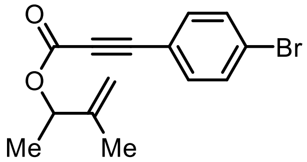

1i (370.0 mg, 1.26 mmol, 25% yield) was prepared from 3-(4-bromophenyl)propynoic acid (1.125 g, 5.00 mmol) and **S2a** (516.8 mg, 6.00 mmol) according to the procedure for **1h**.

Colorless oil; ^{1}H NMR (400 MHz, $CDCl_3$) δ 7.53–7.49 (m, 2H), 7.44–7.42 (m, 2H), 5.43 (q, J = 6.5 Hz, 1H), 5.05 (s, 1H), 4.93–4.91 (m, 1H), 1.79 (s, 3H), 1.41 (d, J = 6.6 Hz, 3H); ^{13}C NMR (101 MHz, $CDCl_3$) δ 153.0, 143.4, 134.2, 131.9, 125.3, 118.6, 112.5, 84.6, 81.7, 75.5, 18.9, 18.1; HRMS (ESI) calcd for $C_{14}H_{13}BrNaO_2$ $[M+Na]^+$ 314.9991, found 315.0008.

2-Methyl-1-phenylprop-2-en-1-yl 3-phenylpropynoate (1j)

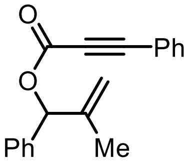

1j (304.1 mg, 1.10 mmol, 22% yield) was prepared from 3-phenylpropynoic acid (730.7 mg, 5.00 mmol) and 2-methyl-1-phenylprop-2-en-1-ol (**S2c**, 741.0 mg, 5.00 mmol) according to the procedure for **1h**.

Pale yellow oil; ^{1}H NMR (400 MHz, $CDCl_3$) δ 7.61–7.58 (m, 2H), 7.47–7.30 (m, 8H), 6.30 (s, 1H), 5.21–5.20 (m, 1H), 5.06–5.04 (m, 1H), 1.70 (s, 3H); ^{13}C NMR (101 MHz, $CDCl_3$) δ 153.0, 142.2, 137.5, 133.0, 130.6, 128.52, 128.46, 128.3, 127.2, 119.6, 113.3, 86.6, 80.7, 80.0, 18.8; HRMS (ESI) calcd for $C_{19}H_{16}NaO_2$ $[M+Na]^+$ 299.1043, found 299.1037.

3-Phenyl-1-{2-(prop-1-en-2-yl)indolin-1-yl}prop-2-yn-1-one (1k)

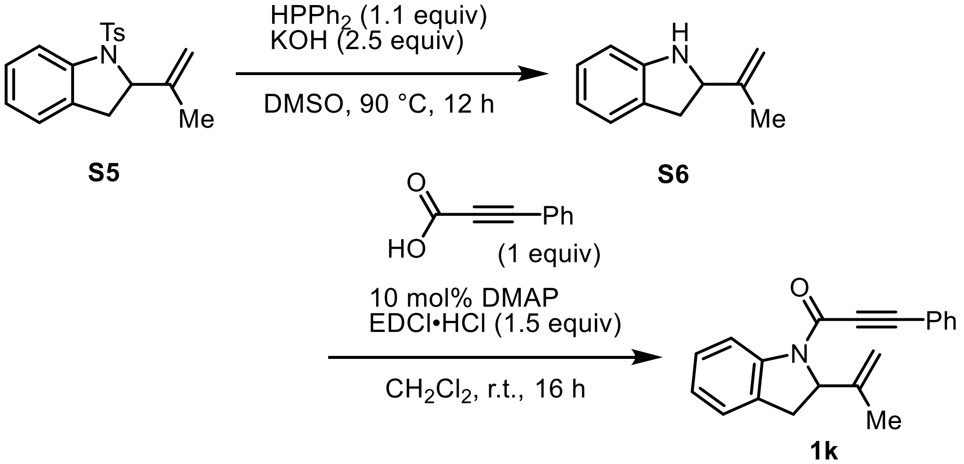

1k

To a mixture of *N*-(4-methylbenzensulfonyl)-2,3-dihydro-2-(prop-1-en-2-yl)indole (**S5**, 270.0 mg, 0.862 mmol) and potassium hydroxide (120.8 mg, 2.15 mmol) in DMSO (10.0 mL) was added $HPPh_2$ (176.5 mg, 0.948 mmol), and the mixture was stirred at 90 °C for 1 h. The reaction was quenched with water, and the crude mixture was extracted with EtOAc, washed with brine, dried over Na_2SO_4, and concentrated. The crude product was purified by alumina column chromatography (eluent: *n*-hexane/EtOAc = 1:1) to furnish 2-(prop-1-en-2-yl)indoline (**S6**, 48% NMR yield) which was used in the following step without further purification in spite of a small amount of unidentified impurities. The spectroscopic data are consistent with the literature reported.[16]

^{1}H NMR (400 MHz, $CDCl_3$) δ 7.05 (d, *J* = 7.2 Hz, 1H), 7.01 (t, *J* = 7.6 Hz, 1H), 6.67 (td, *J* = 7.4, 0.8 Hz, 1H), 6.61 (d, *J* = 7.7 Hz, 1H), 5.003–4.999 (m, 1H), 4.81–4.80 (m, 1H), 4.37 (t, *J* = 9.0 Hz, 1H), 3.84 (s, 1H), 3.18 (dd, *J* = 15.5, 9.2 Hz, 1H), 2.84 (dd, *J* = 15.6, 9.0 Hz, 1H), 1.77 (s, 3H).

To a solution of 3-phenylpropiolic acid (58.5 mg, 0.400 mmol) in CH_2Cl_2 (2.0 mL) were added **S6**, EDCI·HCl (115.0 mg, 0.600 mmol), and DMAP (4.9 mg, 0.040 mmol) at 0 °C, and the mixture was stirred at room temperature for 16 h. The reaction was quenched with aqueous NH_4Cl and extracted with EtOAc three times. The combined organic layers were washed with brine, dried over Na_2SO_4, and concentrated. The crude product was purified by silica gel column chromatography (eluent: *n*-hexane/EtOAc = 4:1) to furnish **1k** (71.8 mg, 0.250 mmol, 29% yield in 2 steps).

White solid; mp 98.4–99.4 °C; ^{1}H NMR (400 MHz, $CDCl_3$) δ 8.21 (d, *J* = 8.0 Hz, 1H), 7.56–7.53 (m, 2H), 7.45–7.35 (m, 3H), 7.25 (t, *J* = 7.8 Hz, 1H), 7.18 (d, *J* = 7.3 Hz, 1H), 7.06 (td, *J* = 11.1, 0.9 Hz, 1H), 5.19 (dd, *J* = 10.1, 2.1 Hz, 1H), 4.93 (s, 1H), 4.88 (s, 1H), 3.59 (dd, *J* = 16.1, 10.2 Hz, 1H), 2.91 (dd, *J* = 16.1, 2.2 Hz, 1H), 1.74 (s, 3H); ^{13}C NMR (101 MHz, $CDCl_3$) δ 152.0, 144.7, 142.2, 132.4, 130.7, 130.1, 128.6, 127.7, 124.73, 124.70, 120.6, 117.3, 111.4, 89.4, 83.1, 65.4, 34.9, 18.2; HRMS (ESI) calcd for $C_{20}H_{17}NNaO$ $[M+Na]^+$ 310.1202, found 310.1194.

[3-{(3-Methylbut-3-en-2-yl)oxy}prop-1-yn-1-yl]benzene (1l)

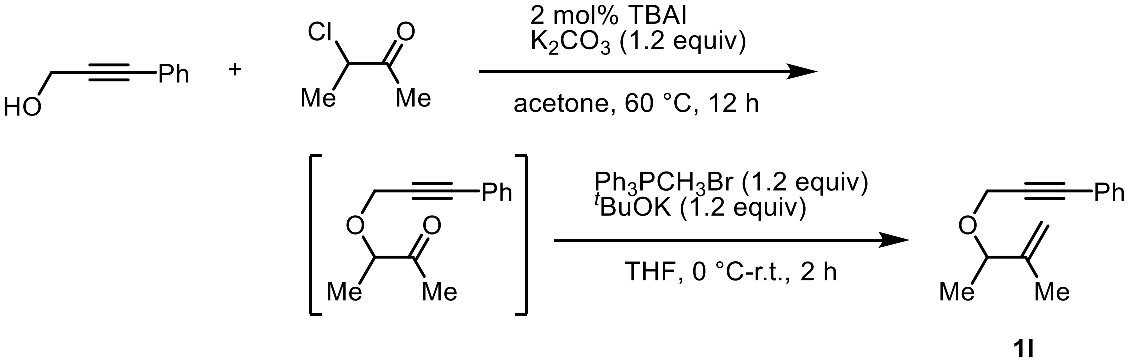

1l

A solution of 3-chlorobutan-2-one (1.00 mL, 10.0 mmol) in acetone (5.0 mL) was added to a solution of 3-phenylprop-2-yn-1-ol (1.27 mL, 10.0 mmol), K_2CO_3 (1.659 g, 12.0 mmol), tetrabutylammonium iodide (TBAI, 73.9 mg, 0.200 mmol), and KI (83.0 mg, 0.500 mmol) in acetone (15.0 mL) under N_2. After stirring at 60 ºC for 12 h, the reaction mixture was filtered, and the filtrate was concentrated. The crude product was passed through silica gel column chromatography (eluent: *n*-hexane/EtOAc = 4:1), and the obtained product was used in the following step without further purification.

To a mixture of Ph_3PCH_3Br (4.287 g, 12.0 mmol) and ^{t}BuOK (1.347 g, 12.0 mmol) in THF (15.0 mL) was added a solution of the product above in THF (5.0 mL) at 0 °C, and the mixture was stirred at room temperature for 2 h. The reaction was quenched with aqueous NH_4Cl, and the mixture was extracted with EtOAc three times. The combined organic layers were washed with water and brine, dried over Na_2SO_4, and concentrated. The crude product was purified by silica gel column chromatography (eluent: *n*-hexane/EtOAc = 4:1) to furnish **1l** (603.4 mg, 3.01 mmol, 30% yield).

Pale yellow oil; ^{1}H NMR (400 MHz, $CDCl_3$) δ 7.50–7.45 (m, 2H), 7.35–7.31 (m, 3H) 5.04–5.03 (m, 1H), 4.98–4.96 (m, 1H), 4.36 (d, *J* = 15.6 Hz, 1H), 4.21 (d, *J* = 15.7 Hz, 1H), 4.16 (q, *J* = 6.3 Hz, 1H), 1.75 (dd, *J* = 1.4, 1.0 Hz, 3H), 1.34 (d, *J* = 6.5 Hz, 3H); ^{13}C NMR (101 MHz, $CDCl_3$) δ 145.1, 131.7, 128.24, 128.18, 122.8, 113.2, 85.6, 85.5, 78.4, 55.9, 19.9, 16.4; HRMS (ESI) calcd for $C_{14}H_{16}NaO$ $[M+Na]^+$ 233.1093, found 233.1092.

3-Phenylbut-3-en-2-yl 3-phenylpropynoate (1m)

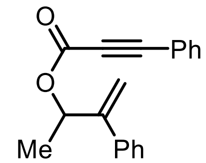

1m (189.4 mg, 0.685 mmol, 23% yield) was prepared from 3-phenylpropynoic acid (438.4 mg, 3.00 mmol) and 3-phenylbut-3-en-2-ol (**S2d**, 444.6 mg, 3.00 mmol) according to the procedure for **1h**.

White solid; mp 46.3–47.7 °C; ^{1}H NMR (400 MHz, $CDCl_3$) δ 7.59–7.57 (m, 2H), 7.45–7.41 (m, 3H), 7.37–7.28 (m, 5H), 5.94 (q, *J* = 6.5 Hz, 1H), 5.43 (s, 1H), 5.36 (s, 1H), 1.44 (d, *J* = 6.5 Hz, 1H); ^{13}C NMR (101 MHz, $CDCl_3$) δ 153.3, 148.5, 139.0, 133.0, 130.6, 128.5, 128.4, 127.9, 126.9, 119.6, 113.7, 86.3, 80.8, 73.4, 20.0; HRMS (ESI) calcd for $C_{19}H_{16}NaO_2$ $[M+Na]^+$ 299.1043, found 299.1043.

***N*-{3-(4-Chlorophenyl)prop-2-yn-1-yl}-4-methyl-*N*-(penta-1,4-dien-3-yl)-benzenesulfonamide (5c)**

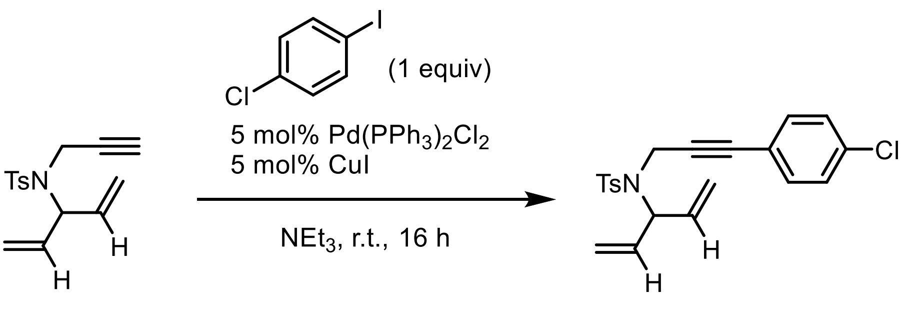

S7a **5c**

5c (472.6 mg, 1.23 mmol, 82% yield) was prepared from 4-methyl-*N*-(penta-1,4-dien-3-yl)-*N*-(prop-2-yn-1-yl)benzenesulfonamide (**S7a**, 413.0 mg, 1.50 mmol) and 1-chloro-4-iodobenzene (429.2 mg, 1.80 mmol) according to the procedure for **1e**.

Yellow oil; ^{1}H NMR (400 MHz, $CDCl_3$) δ 7.81 (d, *J* = 8.3 Hz, 2H), 7.56–7.53 (m, 2H), 7.20 (d, *J* = 8.0 Hz, 2H), 7.10–7.07 (m, 2H), 5.93 (ddd, *J* = 17.1, 10.5, 6.0 Hz, 2H), 5.25 (dt, *J* = 10.5,

1.3 Hz, 2H), 5.23 (dt, J = 17.2, 1.3 Hz, 2H), 5.09–5.05 (m, 1H), 4.26 (s, 2H), 2.35 (s, 3H); ^{13}C NMR (101 MHz, $CDCl_3$) δ 143.2, 137.7, 134.5, 134.3, 132.6, 129.2, 128.5, 127.8, 121.0, 118.9, 86.2, 83.4, 62.0, 34.2, 21.4; HRMS (ESI) calcd for $C_{21}H_{20}ClNNaO_2S$ [M+Na]$^+$ 408.0795, found 408.0814.

_N_-{3-(4-Bromophenyl)prop-2-yn-1-yl}-4-methyl-_N_-(penta-1,4-dien-3-yl)-benzenesulfonamide (5d)

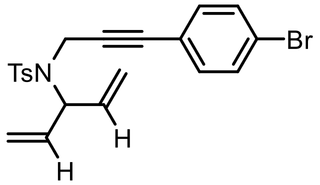

5d (1.022 g, 2.37 mmol, 79% yield) was prepared from **S7a** (826.1 mg, 3.00 mmol) and 1-bromo-4-iodobenzene (1.019 g, 3.60 mmol) according to the procedure for **1e**.

Yellow oil; ^{1}H NMR (400 MHz, $CDCl_3$) δ 7.80 (d, J = 8.3 Hz, 2H), 7.41–7.3 (m, 2H), 7.20 (d, J = 8.0 Hz, 2H), 7.03–7.00 (m, 2H), 5.93 (ddd, J = 17.1, 10.5, 6.0 Hz, 2H), 5.25 (dt, J = 10.5, 1.3 Hz, 2H), 5.23 (dt, J = 17.2, 1.3 Hz, 2H), 5.09–5.05 (m, 1H), 4.26 (s, 2H), 2.35 (s, 3H); ^{13}C NMR (101 MHz, $CDCl_3$) δ 143.2, 137.7, 134.5, 132.7, 131.4, 129.2, 127.7, 122.5, 121.5, 118.8, 86.4, 83.4, 62.0, 34.2, 21.4; HRMS (ESI) calcd for $C_{21}H_{20}BrNNaO_2S$ [M+Na]$^+$ 452.0290, found 452.0290.

_N_-(Hept-2-yn-1-yl)-4-methyl-_N_-(penta-1,4-dien-3-yl)benzenesulfonamide (5f)

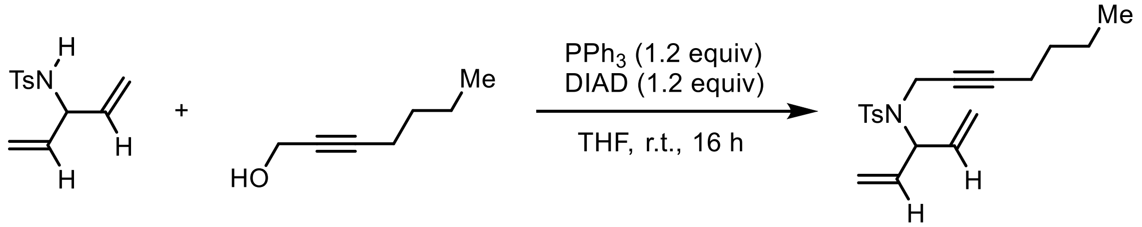

S8a **5f**

5f (204.1 mg, 0.616 mmol, 21% yield) was prepared from 4-methyl-_N_-(penta-1,4-dien-3-yl)benzenesulfonamide (**S8a**, 712.0 mg, 3.00 mmol) and hept-2-yn-1-ol (336.3 mg, 3.00 mmol) according to the procedure for **1a**.

Coloress oil; ^{1}H NMR (400 MHz, $CDCl_3$) δ 7.79 (d, J = 8.3 Hz, 2H), 7.25 (d, J = 8.0 Hz, 2H), 5.91 (ddd, J = 17.1, 10.5, 6.1 Hz, 2H), 5.20 (dt, J = 10.4, 1.3 Hz, 2H), 5.18 (dt, J = 17.2, 1.4 Hz, 2H), 5.00–4.96 (m, 1H), 4.04 (t, J = 2.2 Hz, 2H), 2.41 (s, 3H), 1.99 (tt, J = 10.5, 2.2 Hz, 2H), 1.36–1.23 (m, 4H), 0.86 (t, J = 7.1 Hz, 3H); ^{13}C NMR (101 MHz, $CDCl_3$) δ 142.9, 137.9, 134.8, 129.0, 127.8, 118.5, 85.0, 75.6, 62.1, 34.0, 30.3, 21.8, 21.4, 18.2, 13.5; HRMS (ESI) calcd for $C_{19}H_{25}NNaO_2S$ [M+Na]$^+$ 354.1498, found 354.1518.

Penta-1,4-dien-3-yl 3-phenylpropynoate (5h)

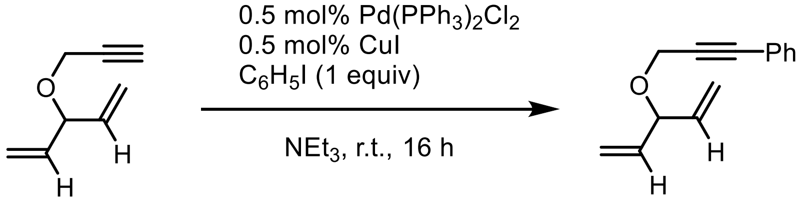

S7b **5g**

To a solution of iodobenzene (1.020 g, 5.00 mmol) and **S7b** (610.8 mg, 5.00 mmol) in Et_3N (10.0 mL) were added $PdCl_2(PPh_3)_2$ (17.6 mg, 0.0250 mmol) and CuI (4.8 mg, 0.0250 mmol) under Ar,

and the mixture was stirred at room temperature for 16 h. The reaction mixture was filtered and concentrated, and the crude product was purified by silica gel column chromatography (eluent: *n*-hexane/EtOAc = 2:1) to furnish **5g** (414.4 mg, 2.09 mmol, 42% yield). The spectroscopic data are consistent with the literature reported.[2]

Colorless oil; ^{1}H NMR (400 MHz, $CDCl_3$) δ 7.47–7.42 (m, 2H), 7.33–7.29 (m, 3H), 5.83 (ddd, *J* = 17.2, 10.4, 6.8 Hz, 2H), 5.36–5.25 (m, 4H), 4.38 (s, 2H); ^{13}C NMR (101 MHz, $CDCl_3$) δ 137.1, 131.8, 128.8, 128.3, 122.9, 117.5, 86.0, 85.7, 80.6, 55.9.

N-(2,4-Dimethylpenta-1,4-dien-3-yl)-4-methyl-*N*-(3-phenylprop-2-yn-1-yl)-benzenesulfonamide (5h)

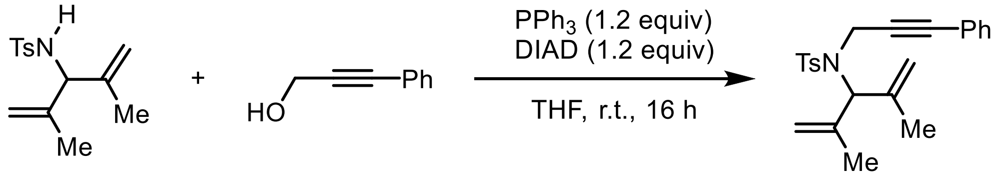

5h (526.3 mg, 1.39 mmol, 46% yield) was prepared from 4-methyl-*N*-(penta-1,4-dien-3-yl)benzenesulfonamide (**S8b**, 796.1 mg, 3.00 mmol) and hept-2-yn-1-ol (396.5 mg, 3.00 mmol) according to the procedure for **1a**.

Yellow oil; ^{1}H NMR (400 MHz, $CDCl_3$) δ 7.85 (d, *J* = 8.3 Hz, 2H), 7.28–7.23 (m, 3H), 7.19–7.13 (m, 4H), 5.10–5.08 (m, 2H), 4.964–4.957 (m, 2H), 4.80 (s, 1H), 4.31 (s, 2H), 2.33 (s, 3H), 1.71 (s, 6H); ^{13}C NMR (101 MHz, $CDCl_3$) δ 143.0, 141.3, 138.0, 131.4, 129.1, 128.10, 128.08, 127.7, 122.8, 115.5, 85.2, 83.8, 67.6, 34.7, 21.9, 21.4; HRMS (ESI) calcd for $C_{23}H_{25}NNaO_2S$ $[M+Na]^+$ 402.1498, found 402.1494.

N-{3-(4-Bromophenyl)prop-2-yn-1-yl}-4-methyl-*N*-(2,4-dimethylpenta-1,4-dien-3-yl)-benzenesulfonamide (5i)

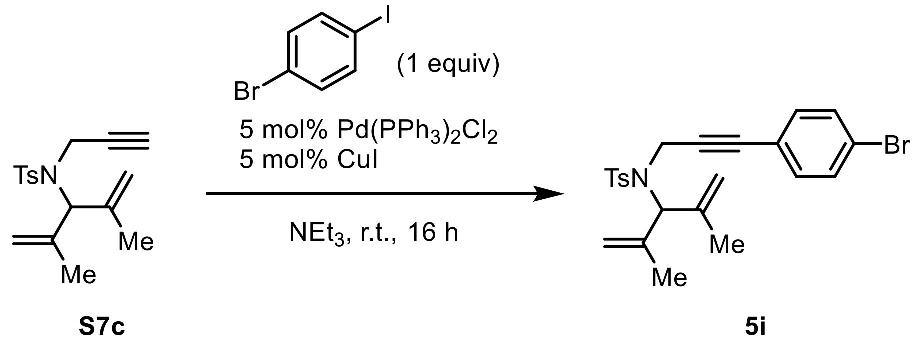

5i (248.4 mg, 0.542 mmol, 90% yield) was prepared from 4-methyl-*N*-(2,4-dimethylpenta-1,4-dien-3-yl)-*N*-(prop-2-yn-1-yl)benzenesulfonamide (**S7c**, 182.1 mg, 0.600 mmol) and 1-bromo-4-iodobenzene (169.7 mg, 0.600 mmol) according to the procedure for **1e**.

White solid; mp 86.4–87.9 °C; ^{1}H NMR (400 MHz, $CDCl_3$) δ 7.82 (d, *J* = 8.3 Hz, 2H), 7.39–7.36 (m, 2H), 7.18 (d, *J* = 8.3 Hz, 2H), 7.02–6.99 (m, 2H), 5.08 (s, 2H), 4.94 (s, 2H), 4.81 (s, 1H), 4.28 (s, 2H), 2.33 (s, 3H), 1.70 (s, 6H); ^{13}C NMR (101 MHz, $CDCl_3$) δ 143.0, 141.1, 137.9, 132.7, 131.3, 129.0, 127.6, 122.3, 121.6, 115.4, 86.4, 82.6, 67.5, 34.6, 21.8, 21.3; HRMS (ESI) calcd for $C_{23}H_{24}BrNNaO_2S$ $[M+Na]^+$ 480.0603, found 480.0603.

***N*-(But-2-yn-1-yl)-4-methyl-*N*-(2,4-dimethylpenta-1,4-dien-3-yl)benzenesulfonamide (5j)**

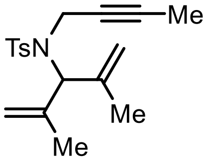

5j (224.7 mg, 0.708 mmol, 35% yield) was prepared from 4-methyl-*N*-(penta-1,4-dien-3-yl)benzenesulfonamide (**S8b**, 530.7 mg, 2.00 mmol) and 2-butyne-1-ol (140.2 mg, 2.00 mmol) according to the procedure for **1a**.

White solid; mp 53.8–55.3 °C; ^{1}H NMR (400 MHz, $CDCl_3$) δ 7.81 (d, *J* = 8.3 Hz, 2H), 7.25 (d, *J* = 7.8 Hz, 2H), 5.04–5.02 (m, 2H), 4.88–4.87 (m, 2H), 4.74 (s, 1H), 4.01 (q, *J* = 2.3 Hz, 2H), 2.41 (s, 3H), 1.67 (s, 6H), 1.61 (t, *J* = 2.4 Hz, 3H); ^{13}C NMR (101 MHz, $CDCl_3$) δ 142.9, 141.3, 138.2, 128.9, 127.8, 115.3, 79.8, 74.9, 67.6, 34.4, 21.8, 21.5, 3.3; HRMS (ESI) calcd for $C_{18}H_{23}NNaO_2S$ $[M+Na]^+$ 340.1342, found 340.1342.

2,4-Dimethylpenta-1,4-dien-3-yl 3-phenylpropynoate (5k)

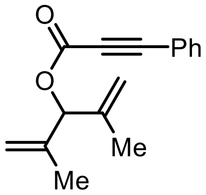

5k (631.3 mg, 2.95 mmol, 29% yield) was prepared from 3-phenylpropynoic acid (1.461 g, 10.0 mmol) and 2,4-dimethylpentadien-3-ol (861.3 mg, 10.0 mmol) according to the procedure for **1h**.

Coloress oil; ^{1}H NMR (400 MHz, $CDCl_3$) δ 7.60–7.58 (m, 2H), 7.45–7.42 (m, 1H), 7.39–7.35 (m, 2H), 5.65 (s, 1H), 5.12–5.11 (m, 2H), 5.03–5.02 (m, 2H), 1.73 (s, 6H); ^{13}C NMR (101 MHz, $CDCl_3$) δ 152.9, 140.5, 133.0, 130.6, 128.5, 119.7, 113.9, 86.3, 81.6, 80.7, 18.3; HRMS (ESI) calcd for $C_{16}H_{16}NaO_2$ $[M+Na]^+$ 263.1043, found 263.1043.

2.1.2. Synthesis of Acrylamides

Substrates **2a**,[17] **2b**,[17] **2c**,[17] **2d**,[18] **2e**,[19] **2g**,[20] **2h**,[21] **2i**,[17] **2j**,[17] and **2k**[22] were synthesized according to the literature.

2-Chloro-1-(pyrrolidin-1-yl)prop-2-en-1-one (2f)

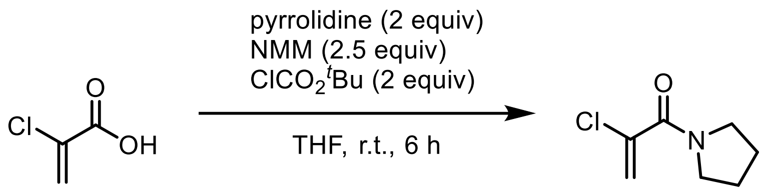

To a solution of 2-chloroacrylic acid (319.5 mg, 3.00 mmol) in THF (5.0 mL) was added a mixture of *N*-methylmorpholine (NMM, 0.82 mL, 7.5 mmol) and isobutyl chloroformate (0.79 mL, 6.0 mmol) in THF (12.0 mL), and the reaction mixture was stirred at room temperature for 0.5 h. Then, pyrrolidine (0.50 mL, 6.0 mmol) was added, and mixture was stirred at room temperature for 6 h. The reaction was quenched with aqueous NH_4Cl and extracted with EtOAc three times. The combined organic layers were washed with brine, dried over Na_2SO_4, and concentrated. The residue was purified by silica gel column chromatography (eluent: *n*-hexane/EtOAc = 4:1) to furnish **2f** (105.4 mg, 0.661 mmol, 22% yield).

Colorless oil; ^{1}H NMR (400 MHz, $CDCl_3$) δ 5.71 (d, J = 2.0 Hz, 1H), 5.65 (d, J = 2.0 Hz, 1H), 3.55–3.52 (m, 4H), 1.95–1.92 (m, 4H); ^{13}C NMR (101 MHz, $CDCl_3$) δ 163.6, 133.8, 117.8, 48.4, 46.1, 26.1, 24.2; HRMS (ESI) calcd for $C_7H_{10}ClNNaO$ $[M+Na]^+$ 182.0343, found 182.0343.

2.2. Optimization of Reaction Conditions

General Procedure for Optimization of Reaction Conditions Using 1a (Table S1): Ligand (0.012 mmol) and [Rh(cod)$_2$]BF$_4$ (4.1 mg, 0.010 mmol) were dissolved in CH_2Cl_2 (2.0 mL) in a Schlenk tube, and the mixture was stirred at room temperature for 10 min. After introduction of H_2 and stirring at room temperature for 30 min, the resulting mixture was concentrated to dryness. The residue was dissolved in CH_2Cl_2 (1.0 mL), followed by the addition of a solution of **1a** (29.1 mg, 0.100 mmol) and **2a** (17.2 mg, 0.120 mmol) in CH_2Cl_2 (1.0 mL). The mixture was stirred at room temperature for 16 h, then passed through short-path silica gel column chromatography to remove the Rh complex and concentrated. The crude product was further purified by silica gel PTLC twice (eluent: *n*-hexane/EtOAc = 1:1 and CH_2Cl_2/EtOAc = 10:1) to furnish **3aa** and **4aa**.

Table S1. Optimization of reaction conditions using **1a**.[a,b]

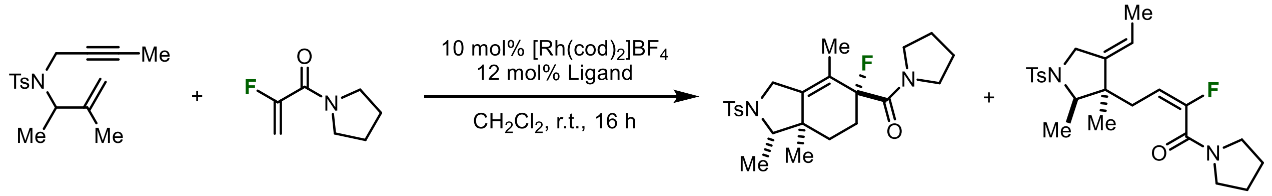

1a **2a** (1.2 equiv) **3aa** **4aa**

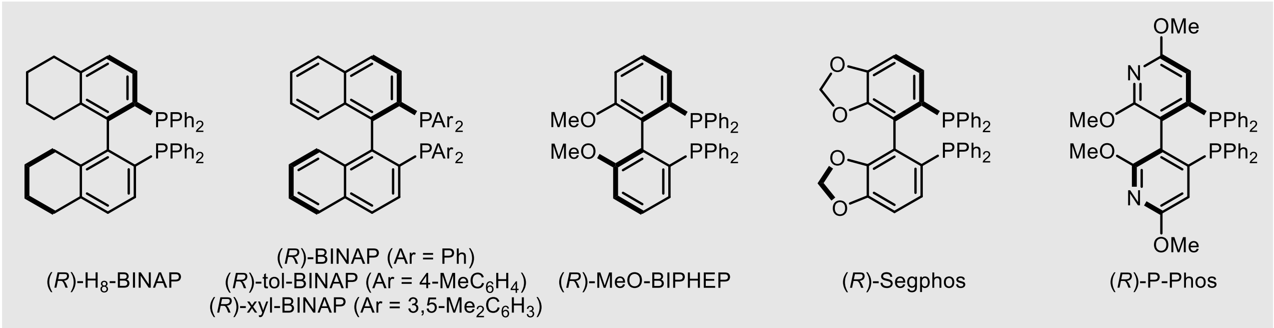

Entry	Ligand	**3aa** % yield[c]	**3aa** d.r.	**3aa** e.r.	**4aa** % yield[c]	**4aa** d.r.	**4aa** e.r.
1	(*R*)-H$_8$-BINAP	18	87:13	97:3	51	68:32	96:4
**2**	**(*R*)-BINAP**	**39**	**87:13**	**97:3**	**32**	**78:22**	**>99:1**
3	(*R*)-tol-BINAP	9	87:13	99:1	23	78:22	99:1
4	(*R*)-xyl-BINAP	<1	–	–	<1	–	–
5[d]	(*R*)-xyl-BINAP	12	>99:1	99:1	<1	–	–
6	(*R*)-MeO-BIPHEP	35	69:31	99:1	30	58:42	99:1
7	(*R*)-Segphos	35	64:36	>99:1	36	63:37	89:11
8	(*R*)-P-Phos	48	61:39	98:2	28	69:31	97:3

[a] Stereochemistry and e.r. values of major diastereomers are shown. [b] **1a** (0.10 mmol), **2a** (0.12 mmol), [Rh(cod)$_2$]BF$_4$ (0.010 mmol), ligand (0.012 mmol), and CH_2Cl_2 (2.0 mL) were used. [c] Isolated yields. [d] Reaction was conducted at 60 °C in $(CH_2Cl)_2$.

General Procedure for Optimization of Reaction Conditions Using 1h (Table S2): Ligand (0.006 mmol) and [Rh(cod)$_2$]BF$_4$ (2.1 mg, 0.005 mmol) were dissolved in CH_2Cl_2 (2.0 mL) in a Schlenk tube, and the mixture was stirred at room temperature for 10 min. After introduction of H_2 and stirring at room temperature for 30 min, the resulting mixture was concentrated to dryness. The residue was dissolved in CH_2Cl_2 (0.5 mL), followed by the addition of a solution of **1h** (10.7 mg, 0.0500 mmol) and **2a** (21.5 mg, 0.150 mmol) in CH_2Cl_2 (0.5 mL). The mixture was stirred at room temperature for 16 h, then passed through short-path silica gel column chromatography to remove the Rh complex and concentrated. The crude product was further purified by silica gel PTLC twice (eluent: *n*-hexane/EtOAc = 1:1 and CH_2Cl_2/EtOAc = 10:1) to furnish **3ha** and **4ha**.

Table S2. Optimization of reaction conditions using **1h**.[a,b]

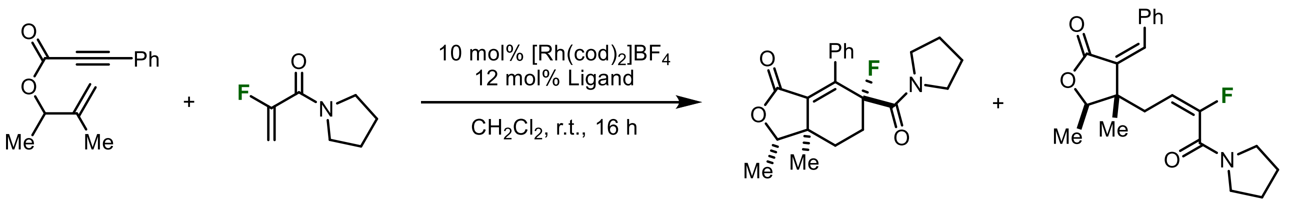

1h **2a** (3.0 equiv) **3ha** **4ha**

Entry	Ligand	**3ha** % yield[c]	**3ha** d.r.	**3ha** e.r.	**4ha** % yield[c]	**4ha** d.r.	**4ha** e.r.
1	(*R*)-H$_8$-BINAP	<1	–	–	<1	–	–
2	(*R*)-BINAP	32	>99:1	97:3	29	>99:1	58:42
3	(*R*)-MeO-BIPHEP	13	>99:1	97:3	27	>99:1	75:25
4	(*R*)-Segphos	14	>99:1	98:2	49	94:6	77:23
**5**	**(*R*)-P-Phos**	**39**	**>99:1**	**98:2**	**40**	**92:8**	**85:15**

[a] Stereochemistry and e.r. values of major diastereomers are shown. [b] **1a** (0.10 mmol), **2a** (0.12 mmol), [Rh(cod)$_2$]BF$_4$ (0.010 mmol), ligand (0.012 mmol), and CH_2Cl_2 (2.0 mL) were used. [c] Isolated yields.

2.3. Rh-Catalyzed Enantioselective PKR (type I) of Racemic 1,6-Enynes with α-Fluoroacrylamides

General Procedure for Rh-Catalyzed Enantioselective PKR (type I) Using 1,6-Enyne 1 (Figure 2): (*R*)-BINAP (7.5 mg, 0.012 mmol) and $[Rh(cod)_2]BF_4$ (4.1 mg, 0.010 mmol) were dissolved in CH_2Cl_2 (2.0 mL) in a Schlenk tube, and the mixture was stirred at room temperature for 10 min. After introduction of H_2 and stirring at room temperature for 30 min, the resulting mixture was concentrated to dryness. The residue was dissolved in CH_2Cl_2 (1.0 mL), followed by the addition of a solution of **1** (0.100 mmol) and **2** (0.120 mmol) in CH_2Cl_2 (1.0 mL) was added, and the mixture was stirred at room temperature for 16 h, then passed through short-path silica gel column chromatography to remove the Rh complex and concentrated. The crude product was further purified by silica gel PTLC twice (eluent: *n*-hexane/EtOAc = 1:1 and CH_2Cl_2/EtOAc = 10:1) to furnish **3** and **4**.

Reaction using 1a and 2a: 3aa [16.9 mg, 0.0388 mmol, 39% yield, 87:13 d.r., 97:3 e.r. (major)] and **4aa** [13.9 mg, 0.0320 mmol, 32% yield, 77:23 d.r., >99:1 e.r. (major)] were obtained from **1a** (29.1 mg, 0.100 mmol) and **2a** (17.2 mg, 0.120 mmol). Two diastereomers of **3aa** were isolated separately (major diastereomer: 14.6 mg, 0.0336 mmol, 34% yield, 97:3 e.r.; minor diastereomer: 2.3 mg, 0.0053 mmol, 5% yield), whereas two diastereomers of **4aa** were obtained as a mixture.

(–)-{(1*S*,5*R*,7a*R*)-5-Fluoro-1,4,7a-trimethyl-2-(4-methylphenyl)-2,3,5,6,7,7a-hexahydro-1*H*-isoindol-5-yl}(pyrrolidin-1-yl)methanone [(–)-3aa (major diastereomer)]

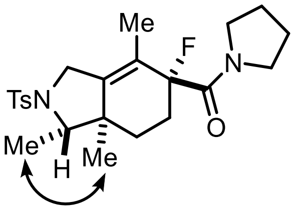

The relative configuration was determined by the NOESY experiment. Colorless oil; $[\alpha]^{25}_{D}$ –57.9 (*c* 0.34, $CHCl_3$, 97:3 e.r.); ^{1}H NMR (400 MHz, $CDCl_3$) δ 7.69–7.66 (m, 2H), 7.31 (d, *J* = 7.9 Hz, 2H), 4.16–4.10 (m 1H), 3.76 (ddd, *J* = 14.9, 6.2, 0.8 Hz, 1H), 3.70–3.66 (m, 2H), 3.50–3.38 (m, 2H), 2.72 (q, *J* = 6.4 Hz, 1H), 2.43 (s, 3H), 2.17–2.09 (m, 2H), 2.00–1.69 (m, 4H), 1.65–1.58 (m, 1H), 1.53–1.49 (m, 1H), 1.48–1.47 (m, 3H), 1.33 (d, *J* = 6.4 Hz, 3H), 1.11 (s, 3H); ^{13}C NMR (101 MHz, $CDCl_3$) δ 169.4 (d, *J* = 27.2 Hz), 143.6, 141.2 (d, *J* = 7.0 Hz), 133.6, 129.9, 127.5, 123.1 (d, *J* = 20.3 Hz), 97.3 (d, *J* = 183.6 Hz), 65.9, 50.6 (d, *J* = 1.2 Hz), 47.7, 47.1 (d, *J* = 16.5 Hz), 43.9 (d, *J* = 2.4 Hz), 30.8 (d, *J* = 6.6 Hz), 30.5 (d, *J* = 23.6 Hz), 26.8 (d, *J* = 5.2 Hz), 23.1, 21.5, 18.1 (d, *J* = 3.3 Hz), 14.1, 13.6 (d, *J* = 2.0 Hz); ^{19}F NMR (377 MHz, $CDCl_3$) δ –147.3; HRMS (ESI) calcd for $C_{23}H_{31}FN_2NaO_3S$ $[M+Na]^+$ 457.1932, found 457.1922; CHIRALPAK AD-H, *n*-hexane/*i*-PrOH = 98:2, 1.0 mL/min, retention times: 63.4 min (major isomer) and 82.3 min (minor isomer).

Minor diastereomer: Colorless oil; ^{1}H NMR (400 MHz, $CDCl_3$) δ 7.75 (d, *J* = 8.3 Hz, 2H), 7.29 (d, *J* = 8.0 Hz, 2H), 3.99 (dd, *J* = 13.5, 4.7 Hz, 1H), 3.90–3.84 (m, 1H), 3.74 (q, *J* = 6.7 Hz, 1H), 3.71–3.64 (m, 2H), 3.56–3.42 (m, 2H), 2.42 (s, 3H), 2.18–2.01 (m, 3H), 1.99–1.70 (m, 5H), 1.26 (s, 3H), 1.12 (d, *J* = 6.6 Hz, 3H), 0.82 (s, 3H); ^{19}F NMR (377 MHz, $CDCl_3$) δ –149.6.

(+)-(*E*)-4-{(2*R*,3*R*)-4-(*Z*)-Ethylidene-2,3-dimethyl-1-(4-methylphenyl)-pyrrolidin-3-yl}-2-fluoro-1-(pyrrolidin-1-yl)but-2-en-1-one [(+)-4aa]

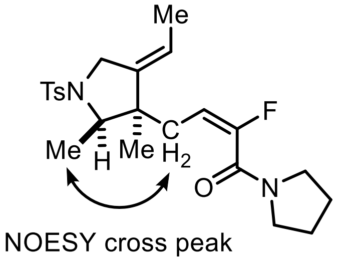

The following experimental data were acquired using a mixture of isomers [78:22 d.r., >99:1 e.r. (major)]. The relative configuration was determined by the NOESY experiment. Colorless oil; $[\alpha]^{25}_{D}$ +6.4 (*c* 0.50, $CHCl_3$); **major diastereomer:** ^{1}H NMR (400 MHz, $CDCl_3$) δ 7.72 (d, *J* = 8.2 Hz, 2H), 7.31 (d, *J* = 8.0 Hz, 2H), 5.48 (dt, *J* = 23.6, 8.0 Hz, 1H), 5.28–5.19 (m, 1H), 4.02 (d, *J* = 14.4 Hz, 1H), 3.79 (d, *J* = 13.8 Hz, 1H), 3.56–3.47 (m, 4H), 3.19 (q, *J* = 6.5 Hz, 1H), 2.49 (ddd, *J* = 15.1, 7.7, 1.6 Hz, 1H), 2.42 (s, 3H), 2.36 (dd, *J* = 15.1, 8.3 Hz, 1H), 1.96–1.84 (m, 4H), 1.55 (d, *J* = 6.8 Hz, 3H), 1.21 (d, *J* = 6.5 Hz, 3H), 0.78 (s, 3H); **partial protons of minor diastereomer:** ^{1}H NMR (400 MHz, $CDCl_3$) δ 7.74 (d, *J* = 8.4 Hz, 2H), 7.30 (d, *J* = 4.7 Hz, 2H), 3.98 (d, *J* = 15.2 Hz, 1H), 3.64 (q, *J* = 6.5 Hz, 1H), 2.41 (s, 3H), 2.17 (dd, *J* = 14.7, 8.8 Hz, 1H), 2.02 (ddd, *J* = 14.8, 7.8, 1.6 Hz, 1H), 1.57 (d, *J* = 6.9 Hz, 3H), 1.08 (d, *J* = 6.6 Hz, 3H), 0.94 (s, 3H); ^{13}C NMR (101 MHz, $CDCl_3$) δ 160.3 (d, *J* = 32.7 Hz), 152.1 (d, *J* = 261.4 Hz), 143.3, 143.2, 140.81, 140.80, 136.2, 134.9, 129.6, 127.4, 127.3, 117.3, 116.5, 112.6 (d, *J* = 18.7 Hz), 111.6 (d, *J* = 18.0 Hz), 65.0, 63.6, 49.0, 48.5 (d, *J* = 2.1 Hz), 47.9 (d, *J* = 1.7 Hz), 47.6, 47.2, 47.14, 47.06, 46.2, 46.1, 34.1 (d, *J* = 5.6 Hz), 29.5 (d, *J* = 5.9 Hz), 26.23 (d, *J* = 3.2 Hz), 26.17 (d, *J* = 2.9 Hz), 23.83, 23.78, 23.0, 21.5, 18.7, 17.2, 15.9, 14.3, 14.1; ^{19}F NMR (377 MHz, $CDCl_3$) δ –113.2 (major), –113.0 (minor); HRMS (ESI) calcd for $C_{23}H_{31}FN_2NaO_3S$ $[M+Na]^+$ 457.1932, found 457.1956; CHIRALPAK IG-3, *n*-hexane/*i*-PrOH = 80:20, 1.0 mL/min, retention times: 40.4 min (major isomer) and 55.5 min (minor isomer).

Reaction using 1b and 2a: 3ba [16.2 mg, 0.0330 mmol, 33% yield, 88:12 d.r., 99:1 e.r. (major)] and **4ba** [15.6 mg, 0.0318 mmol, 32% yield, 76:24 d.r., 99:1 e.r. (major)] were obtained from **1b** (34.8 mg, 0.100 mmol) and **2a** (17.2 mg, 0.120 mmol), using (*R*)-BINAP (14.9 mg, 0.0240 mmol) and $[Rh(cod)_2]OTf$ (9.4 mg, 0.020 mmol) in CH_2Cl_2 (2.0 mL). Two diastereomers of **3ba** were isolated separately (major diastereomer: 14.1 mg, 0.0287 mmol, 29% yield, 99:1 e.r.; minor diastereomer: 2.1 mg, 0.0043 mmol, 4% yield), and two diastereomers of **4ba** were isolated separately (major diastereomer: 11.6 mg, 0.0236 mmol, 24% yield, 99:1 e.r.; minor diastereomer: 4.0 mg, 0.0082 mmol, 8% yield).

(–)-{(1*S*,5*R*,7a*R*)-5-Fluoro-4,7a-dimethyl-2-(4-methylphenyl)-1-pentyl-2,3,5,6,7,7a-hexahydro-1*H*-isoindol-5-yl}(pyrrolidin-1-yl)methanone [(–)-3ba (major diastereomer)]

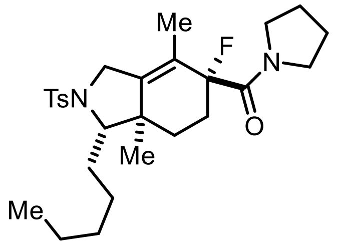

Colorless oil; $[\alpha]^{25}_{D}$ –6.4 (*c* 0.79, $CHCl_3$, 99:1 e.r.); ^{1}H NMR (400 MHz, $CDCl_3$) δ 7.67 (d, *J* = 8.2 Hz, 2H), 7.31 (d, *J* = 8.0 Hz, 2H), 4.09 (ddd, *J* = 14.9, 8.0, 1.4 Hz, 1H), 3.96–3.91 (m, 1H), 3.70–3.65 (m, 2H), 3.51–3.39 (m, 2H), 2.83 (dd, *J* = 9.6, 3.8 Hz, 1H), 2.43 (s, 3H), 2.31–2.20 (m, 1H), 2.15–2.03 (m, 2H), 2.02–1.60 (m, 7H), 1.46 (s, 3H), 1.31–1.23 (m, 6H), 1.17 (s, 3H), 0.89–0.86 (m, 3H); ^{13}C NMR (101 MHz, $CDCl_3$) δ 169.2 (d, *J* = 27.4 Hz), 143.5, 141.1 (d, *J* = 6.8 Hz), 134.4, 129.8, 127.4, 123.0 (d, *J* = 20.3 Hz), 97.1 (d, *J* = 183.2 Hz), 70.9, 50.7 (d, *J* = 1.1 Hz), 47.7, 47.1 (d, *J* = 16.6 Hz), 44.3 (d, *J* = 2.3 Hz), 32.6 (d, *J* = 6.7 Hz), 32.2, 30.4 (d, *J* = 23.2 Hz), 29.7,

26.9 (d, J = 5.2 Hz), 26.3, 23.1, 22.5, 21.5, 18.4 (d, J = 3.2 Hz), 14.0, 13.6 (d, J = 2.4 Hz); ^{19}F NMR (377 MHz, $CDCl_3$) δ –148.2; HRMS (ESI) calcd for $C_{27}H_{39}FN_2NaO_3S$ $[M+Na]^+$ 513.2558, found 513.2571; CHIRALPAK AD-H, *n*-hexane/*i*-PrOH = 95:5, 1.0 mL/min, retention times: 14.7 min (major isomer) and 16.5 min (minor isomer).

Minor diastereomer: Colorless oil; ^{1}H NMR (400 MHz, $CDCl_3$) δ 7.75 (d, J = 8.2 Hz, 2H), 7.29 (d, J = 7.9 Hz, 2H), 3.95–3.93 (m, 1H), 3.71–3.67 (m, 2H), 3.54–3.46 (m, 2H), 2.42 (s, 3H), 2.18–1.73 (m, 7H), 1.53 (s, 3H), 1.47–1.30 (m, 3H), 1.25–1.15 (m, 8H), 0.86–0.83 (m, 6H); ^{19}F NMR (377 MHz, $CDCl_3$) δ –148.3.

(+)-(*E*)-4-{(2*R*,3*R*,*Z*)-4-Ethylidene-3-methyl-1-(4-methylphenyl)pyrrolidin-3-yl}-2-pentyl-2-fluoro-1-(pyrrolidin-1-yl)but-2-en-1-one [(+)-4ba (major diastereomer)]

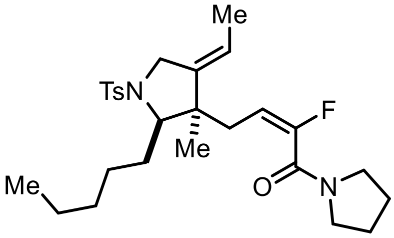

Colorless oil; $[\alpha]^{25}_D$ +2.9 (c 0.74, $CHCl_3$, 99:1 e.r.); ^{1}H NMR (400 MHz, $CDCl_3$) δ 7.74 (d, J = 8.3 Hz, 2H), 7.29 (d, J = 8.0 Hz, 2H), 5.52 (ddd, J = 23.5, 8.1, 7.3 Hz, 1H), 5.21 (qt, J = 9.1, 2.4 Hz, 1H), 3.97 (ddd, J = 14.3, 2.6, 1.6 Hz, 1H), 3.90 (ddd, J = 14.4, 2.1, 1.3 Hz, 1H), 3.65–3.62 (m, 1H), 3.58–3.49 (m, 4H), 2.65 (dd, J = 15.5, 7.9 Hz, 1H), 2.41 (s, 3H), 2.41–2.34 (m, 1H), 1.96–1.84 (m, 4H), 1.53 (d, J = 6.7 Hz, 3H), 1.50–1.09 (m, 8H), 0.84 (t, J = 7.0 Hz, 3H), 0.69 (s, 3H); ^{13}C NMR (101 MHz, $CDCl_3$) δ 160.2 (d, J = 32.4 Hz), 152.2 (d, J = 262.3 Hz), 143.12 (d, J = 0.8 Hz), 143.06, 136.8, 129.5, 127.3, 115.0, 112.9 (d, J = 18.4 Hz), 68.9, 48.1, 47.5 (d, J = 1.5 Hz), 47.2 (d, J = 10.3 Hz), 46.3, 32.1, 31.7, 29.7 (d, J = 5.9 Hz), 26.2 (d, J = 3.3 Hz), 25.7, 25.6, 23.7, 22.6, 21.5, 14.09, 14.06; ^{19}F NMR (377 MHz, $CDCl_3$) δ –113.3; HRMS (ESI) calcd for $C_{27}H_{39}FN_2NaO_3S$ $[M+Na]^+$ 513.2558, found 513.2567; CHIRALPAK IF-3, *n*-hexane/*i*-PrOH = 90:10, 1.0 mL/min, retention times: 37.7 min (major isomer) and 22.1 min (minor isomer).

Minor diastereomer: Colorless oil; ^{1}H NMR (400 MHz, $CDCl_3$) δ 7.74 (d, J = 8.2 Hz, 2H), 7.29 (d, J = 8.0 Hz, 2H), 5.28 (ddd, J = 23.3, 9.0, 7.4 Hz, 1H), 5.22–5.17 (m, 1H), 3.97–3.92 (m, 1H), 3.88–3.84 (m, 1H), 3.76 (t, J = 5.7 Hz, 1H), 3.53–3.46 (m, 4H), 2.40 (s, 3H), 2.15 (dd, J = 14.7, 9.0 Hz, 1H), 1.95–1.86 (m, 5H), 1.29–1.12 (m, 9H), 0.98 (s, 3H), 0.88–0.81 (m, 2H), 0.83 (t, J = 6.9 Hz, 3H); ^{19}F NMR (377 MHz, $CDCl_3$) δ –112.9.

Reaction using 1c and 2a: 3ca [14.1 mg, 0.0284 mmol, 28% yield, >99:1 d.r., 98:2 e.r. (major)] and **4ca** [13.8 mg, 0.0278 mmol, 28% yield, 68:32 d.r., >99:1 e.r. (major)] were obtained from **1c** (35.4 mg, 0.100 mmol) and **2a** (17.2 mg, 0.120 mmol). Two diastereomers of **4ca** were isolated separately (major diastereomer: 9.2 mg, 0.0185 mmol, 19% yield, >99:1 e.r.; minor diastereomer: 4.6 mg, 0.0093 mmol, 9% yield).

(–)-{(1*S*,5*R*,7a*R*)-5-Fluoro-4,7a-dimethyl-2-(4-methylphenyl)-1-phenyl-2,3,5,6,7,7a-hexahydro-1*H*-isoindol-5-yl}(pyrrolidin-1-yl)methanone [(–)-3ca]

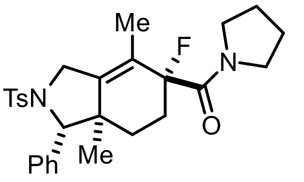

White solid; mp 52.5–53.9 °C; $[\alpha]^{25}_D$ –14.4 (c 1.03, $CHCl_3$, 98:2 e.r.); ^{1}H NMR (400 MHz, $CDCl_3$) δ 7.67 (br s, 1H), 7.52 (d, J = 8.2 Hz, 2H), 7.28–7.26 (m, 5H), 6.96 (br s, 1H), 4.27 (ddd, J = 15.0, 8.1, 1.4 Hz, 1H), 4.10 (dd, J = 14.5, 5.5 Hz, 1H), 3.86 (s, 1H), 3.69–3.64 (m, 2H), 3.53–3.40 (m, 2H), 2.42 (s, 3H), 2.13–2.06 (m, 1H), 2.04–1.71 (m, 6H), 1.50 (s, 3H), 1.36–1.31 (m,

1H), 0.91 (s, 3H); ^{13}C NMR (101 MHz, $CDCl_3$) δ 169.2 (d, J = 27.1 Hz), 143.7, 140.5 (d, J = 6.8 Hz), 135.9, 132.7, 129.7, 127.9, 127.7 (2C), 127.4, 123.8 (d, J = 20.4 Hz), 97.3 (d, J = 183.7 Hz), 75.4, 50.6 (d, J = 1.0 Hz), 47.7, 47.1 (d, J = 16.6 Hz), 45.4 (d, J = 2.4 Hz), 31.1 (d, J = 6.6 Hz), 30.2 (d, J = 23.5 Hz), 26.8 (d, J = 5.2 Hz), 23.1, 21.6, 19.9 (d, J = 3.0 Hz), 13.6 (d, J = 2.3 Hz); ^{19}F NMR (377 MHz, $CDCl_3$) δ –147.6; HRMS (ESI) calcd for $C_{28}H_{33}FN_2NaO_3S$ $[M+Na]^+$ 519.2088, found 519.2066; CHIRALPAK AD-H, *n*-hexane/*i*-PrOH = 90:10, 1.0 mL/min, retention times: 11.5 min (major isomer) and 13.6 min (minor isomer).

(–)-(*E*)-4-{(2*R*,3*R*,*Z*)-4-Ethylidene-3-methyl-2-phenyl-1-(4-methylphenyl)pyrrolidin-3-yl}-2-fluoro-1-(pyrrolidin-1-yl)but-2-en-1-one [(–)-4ca (major diastereomer)]

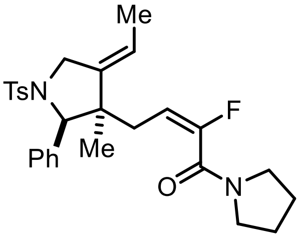

White solid; mp 82.3–84.3 °C; $[\alpha]^{25}_D$ –2.0 (*c* 1.38, $CHCl_3$, >99:1 e.r.); ^{1}H NMR (400 MHz, $CDCl_3$) δ 7.34 (d, J = 8.3 Hz, 2H), 7.18–7.05 (m, 3H), 7.06 (d, J = 7.9 Hz, 2H), 6.86 (d, J = 6.7 Hz, 2H), 5.42 (ddd, J = 23.2, 9.3, 7.1 Hz, 1H), 5.22 (qt, J = 6.8, 2.4 Hz, 1H), 4.64 (s, 1H), 4.18 (ddd, J = 13.7, 2.5, 1.6 Hz, 1H), 4.12 (ddd, J = 13.7, 2.0, 1.1 Hz, 1H), 3.58–3.48 (m, 4H), 2.56 (dd, J = 14.7, 9.3 Hz, 1H), 2.37–2.31 (m, 1H), 2.34 (s, 3H), 1.97–1.86 (m, 4H), 1.66 (d, J = 6.8 Hz, 3H), 0.60 (s, 3H); ^{13}C NMR (101 MHz, $CDCl_3$) δ 160.2 (d, J = 32.7 Hz), 152.6 (d, J = 262.3 Hz), 142.7, 140.8, 139.5, 136.0, 129.1, 128.0, 127.5, 127.3, 127.1, 118.1, 111.3 (d, J = 18.0 Hz), 72.3, 49.8 (d, J = 2.0 Hz), 49.0, 47.2 (d, J = 8.8 Hz), 46.1, 35.8 (d, J = 5.8 Hz), 26.1 (d, J = 2.9 Hz), 23.8, 21.4, 19.5, 14.5; ^{19}F NMR (377 MHz, $CDCl_3$) δ –112.5; HRMS (ESI) calcd for $C_{28}H_{33}FN_2NaO_3S$ $[M+Na]^+$ 519.2088, found 519.2071; CHIRALPAK IF-3, *n*-hexane/*i*-PrOH = 90:10, 1.0 mL/min, retention times: 74.6 min (major isomer) and 47.1 min (minor isomer).

Minor diastereomer: White solid; ^{1}H NMR (400 MHz, $CDCl_3$) δ 7.36–7.33 (m, 2H), 7.19–7.13 (m, 3H), 7.08 (d, J = 7.9 Hz, 2H), 7.05–7.03 (m, 2H), 5.34–5.23 (m, 2H), 4.44 (s, 1H), 4.21 (ddd, J = 14.0, 2.4, 1.5 Hz, 1H), 4.10 (ddd, J = 14.0, 2.1, 1.2 Hz, 1H), 3.46–3.32 (m, 4H), 2.61 (dd, J = 15.2, 8.9 Hz, 1H), 2.34 (s, 3H), 1.90–1.79 (m, 5H), 1.62 (d, J = 6.8 Hz, 3H), 1.08 (s, 3H); ^{13}C NMR (101 MHz, $CDCl_3$) δ 160.0 (d, J = 32.7 Hz), 151.8 (d, J = 261.3 Hz), 142.8, 142.1, 138.5, 135.3, 129.1, 128.1, 128.0, 127.5, 127.2, 116.4, 112.2 (d, J = 18.8 Hz), 73.3, 49.1, 48.9 (d, J = 1.8 Hz), 47.0 (d, J = 10.0 Hz), 46.1, 30.0 (d, J = 6.1 Hz), 26.1 (d, J = 3.3 Hz), 25.8, 23.7, 21.4, 14.3; ^{19}F NMR (377 MHz, $CDCl_3$) δ –113.5.

Reaction using 1d and 2a: 3da [18.1 mg, 0.0364 mmol, 36% yield, 75:25 d.r., 98:2 e.r. (major)] and **4da** [23.2 mg, 0.0467 mmol, 47% yield, 58:42 d.r., 82:18 e.r. (major)] were obtained from **1d** (35.4 mg, 0.100 mmol) and **2a** (17.2 mg, 0.120 mmol). Two diastereomers of **3da** were isolated separately (major diastereomer: 13.5 mg, 0.0272 mmol, 27% yield, 98:2 e.r.; minor diastereomer: 4.6 mg, 0.0093 mmol, 9% yield), whereas two diastereomers of **4da** were obtained as a mixture.

(–)-{(1*S*,5*R*,7a*R*)-5-Fluoro-1,7a-dimethyl-2-(4-methylphenyl)-4-phenyl-2,3,5,6,7,7a-hexahydro-1*H*-isoindol-5-yl}(pyrrolidin-1-yl)methanone [(–)-3da (major diastereomer)]

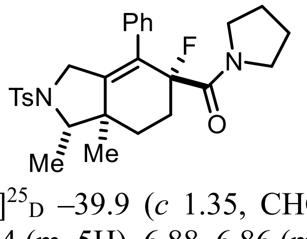

White solid; mp 62.9–64.6 °C; $[\alpha]^{25}_D$ –39.9 (*c* 1.35, $CHCl_3$, 98:2 e.r.); ^{1}H NMR (400 MHz, $CDCl_3$) δ 7.54–7.51 (m, 2H), 7.31–7.24 (m, 5H), 6.88–6.86 (m, 2H), 3.95 (dd, J = 15.7, 8.7 Hz, 1H),

3.43 (dd, J = 15.7, 6.1 Hz, 1H), 3.38–3.32 (m, 1H), 3.30–3.20 (m, 2H), 2.83 (q, J = 6.4 Hz, 1H), 2.73–2.66 (m, 1H), 2.46 (s, 3H), 2.33 (dt, J = 13.5, 3.6 Hz, 1H), 2.15 (dtd, J = 23.7, 13.8, 3.9 Hz, 1H), 1.97 (td, J = 20.0, 3.6 Hz, 1H), 1.67–1.51 (m, 4H), 1.38 (d, J = 6.4 Hz, 3H), 1.37–1.30 (m, 1H), 1.24 (s, 3H); ^{13}C NMR (101 MHz, $CDCl_3$) δ 169.4 (d, J = 27.4 Hz), 145.2 (d, J = 6.7 Hz), 143.6, 136.1, 133.2, 129.8, 129.24 (d, J = 19.5 Hz), 129.20 (d, J = 1.2 Hz), 128.2, 127.8, 127.5, 96.3 (d, J = 186.4 Hz), 66.2, 51.1, 47.7, 46.8 (d, J = 17.2 Hz), 43.9 (d, J = 2.4 Hz), 31.8 (d, J = 6.5 Hz), 31.5 (d, J = 24.4 Hz), 26.5 (d, J = 5.6 Hz), 22.9, 21.6, 18.3 (d, J = 3.3 Hz), 14.5; ^{19}F NMR (377 MHz, $CDCl_3$) δ –142.8; HRMS (ESI) calcd for $C_{28}H_{33}FN_2NaO_3S$ $[M+Na]^+$ 519.2088, found 519.2110; CHIRALPAK AD-H, *n*-hexane/*i*-PrOH = 90:10, 1.0 mL/min, retention times: 16.3 min (major isomer) and 12.9 min (minor isomer).

Minor diastereomer: Colorless oil; ^{1}H NMR (400 MHz, $CDCl_3$) δ 7.70–7.67 (m, 2H), 7.30–7.28 (m, 5H), 7.06–7.03 (m, 2H), 3.78 (q, J = 6.3 Hz, 1H), 3.75 (dd, J = 14.5, 8.1 Hz, 1H), 3.60 (dd, J = 14.6, 5.0 Hz, 1H), 3.48–3.33 (m, 3H), 2.92–2.84 (m, 1H), 2.47–2.41 (m, 1H), 2.41 (s, 3H), 2.31 (dt, J = 13.4, 3.6 Hz, 1H), 2.19–2.06 (m, 1H), 1.72–1.57 (m, 3H), 1.54–1.38 (m, 2H), 1.23 (d, J = 6.6 Hz, 3H), 0.87 (s, 3H). ^{13}C NMR (101 MHz, $CDCl_3$) δ 169.3 (d, J = 27.1 Hz), 144.9 (d, J = 6.3 Hz), 143.1, 136.4, 136.3, 131.7 (d, J = 20.5 Hz), 129.6, 129.1 (d, J = 0.6 Hz), 128.3, 127.8, 127.1, 96.2 (d, J = 186.1 Hz), 66.0, 47.9, 47.8, 46.9 (d, J = 17.1 Hz), 44.2 (d, J = 2.3 Hz), 31.4 (d, J = 23.4 Hz), 28.2 (d, J = 7.0 Hz), 26.6 (d, J = 5.5 Hz), 24.4 (d, J = 3.2 Hz), 23.0, 21.5, 18.9; ^{19}F NMR (377 MHz, $CDCl_3$) δ –144.8.

(–)-(*E*)-4-{(2*R*,3*R*)-4-(*Z*)-Benzylidene-2,3-dimethyl-1-(4-methylphenyl)-pyrrolidin-3-yl}-2-fluoro-1-(pyrrolidin-1-yl)but-2-en-1-one [(–)-4da]

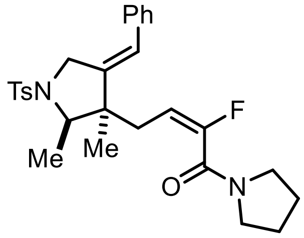

The following experimental data were acquired using a mixture of isomers [58:42 d.r., 82:18 e.r. (major)]. Colorless oil; $[\alpha]^{25}_D$ –4.0 (*c* 2.37, $CHCl_3$); **major diastereomer:** ^{1}H NMR (400 MHz, $CDCl_3$) δ 7.69 (d, J = 8.3 Hz, 2H), 7.37–7.33 (m, 2H), 7.30–7.23 (m, 3H), 7.16–7.13 (m, 2H), 6.17 (t, J = 2.3 Hz, 1H), 5.56 (dt, J = 23.6, 8.1 Hz, 1H), 4.37 (dd, J = 15.0, 2.3 Hz, 1H), 4.15 (dd, J = 15.0, 2.4 Hz, 1H), 3.48–3.38 (m, 4H), 3.22 (q, J = 6.5 Hz, 1H), 2.69 (ddd, J = 14.9, 7.9, 1.5 Hz, 1H), 2.51 (ddd, J = 14.9, 8.3, 0.9 Hz, 1H), 2.40 (s, 3H), 1.88–1.74 (m, 4H), 1.26 (d, J = 6.5 Hz, 3H), 0.95 (s, 3H); **partial protons of minor diastereomer:** ^{1}H NMR (400 MHz, $CDCl_3$) δ 7.76 (d, J = 8.3 Hz, 2H), 6.19 (t, J = 2.2 Hz, 1H), 5.32 (dt, J = 23.4, 8.3 Hz, 1H), 4.27 (dd, J = 14.6, 2.7 Hz, 1H), 4.21 (dd, J = 14.6, 2.1 Hz, 1H), 3.69 (q, J = 6.5 Hz, 1H), 2.40 (s, 3H), 2.31–2.19 (m, 2H), 1.10 (s, 3H), 1.08 (d, J = 6.6 Hz, 3H); ^{13}C NMR (101 MHz, $CDCl_3$) δ 160.2 (d, J = 32.2 Hz), 160.1 (d, J = 32.8 Hz), 152.5 (d, J = 262.6 Hz), 152.3 (d, J = 262.3 Hz), 143.4, 143.3, 141.99, 141.97, 136.3, 136.21, 136.19, 134.8, 129.67, 129.66, 128.5, 128.4, 127.4, 127.2, 127.12, 127.09, 122.9, 122.1, 112.4 (d, J = 19.1 Hz), 111.3 (d, J = 18.6 Hz), 63.7, 62.5, 50.4, 50.0 (d, J = 2.3 Hz), 49.5 (d, J = 1.9 Hz), 48.8, 47.14 (d, J = 10.2 Hz), 47.06 (d, J = 9.4 Hz), 46.2, 46.1, 34.1 (d, J = 6.0 Hz), 29.6 (d, J = 6.1 Hz), 26.02 (d, J = 3.0 Hz), 25.99 (d, J = 2.3 Hz), 23.7, 23.6, 22.8, 21.5, 18.6, 17.3, 15.8; ^{19}F NMR (377 MHz, $CDCl_3$) δ –112.2 (major), –112.4 (minor); HRMS (ESI) calcd for $C_{28}H_{33}FN_2NaO_3S$ $[M+Na]^+$ 519.2088, found 519.2088; CHIRALPAK IF-3, *n*-hexane/*i*-PrOH = 90:10, 1.0 mL/min, retention times: 50.0 min (major isomer) and 39.9 min (minor isomer).

Reaction using 1e and 2a: 3ea [17.4 mg, 0.0330 mmol, 33% yield, 70:30 d.r., >99:1 e.r. (major)] and **4ea** [22.6 mg, 0.0320 mmol, 43% yield, 58:42 d.r., 98:2 e.r. (major)] were obtained from **1e** (38.3 mg, 0.100 mmol) and **2a** (17.2 mg, 0.120 mmol). Two diastereomers of **3ea** and **4ea** were obtained as a mixture, respectively.

(–)-{(1*S*,5*R*,7a*R*)-5-Fluoro-4-(4-methoxyphenyl)-1,7a-dimethyl-2-(4-methylphenyl)-2,3,5,6,7,7a-hexahydro-1*H*-isoindol-5-yl}(pyrrolidin-1-yl)methanone [(–)-3ea]

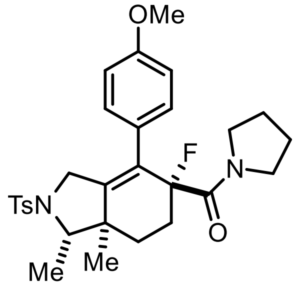

The following experimental data were acquired using a mixture of isomers [70:30 d.r., >99:1 e.r. (major)]. White solid; mp 72.4–74.0 °C; $[\alpha]^{25}_{D}$ –64.4 (*c* 1.74, $CHCl_3$); **major diastereomer:** ^{1}H NMR (400 MHz, $CDCl_3$) δ 7.53 (d, *J* = 8.2 Hz, 2H), 7.38–7.24 (m, 2H), 6.84–6.79 (m, 4H), 3.95 (dd, *J* = 15.6, 8.6 Hz, 1H), 3.82 (s, 3H), 3.75 (dd, *J* = 14.5, 7.9 Hz, 1H), 3.47–3.22 (m, 4H), 2.84–2.77 (m, 2H), 2.46 (s, 3H), 2.33–2.26 (m, 1H), 2.21–2.07 (m, 1H), 1.94 (td, *J* = 13.5, 3.7 Hz, 1H), 1.73–1.56 (m, 3H), 1.46–1.40 (m, 1H), 1.38 (d, *J* = 6.4 Hz, 3H), 1.22 (s, 3H); **partial protons of minor diastereomer:** ^{1}H NMR (400 MHz, $CDCl_3$) δ 7.69 (d, *J* = 8.3 Hz, 2H), 6.98–6.95 (m, 2H), 3.61 (dd, *J* = 14.5, 5.0 Hz, 1H), 3.03–2.95 (m, 1H), 2.41 (s, 3H), 0.86 (s, 3H); ^{13}C NMR (101 MHz, $CDCl_3$) δ 169.5 (d, *J* = 27.4 Hz), 169.3 (d, *J* = 27.2 Hz), 159.11, 159.09, 145.3 (d, *J* = 6.7 Hz), 144.8 (d, *J* = 6.3 Hz), 143.5, 143.1, 136.4, 133.3, 130.3 (d, *J* = 1.1 Hz), 130.2 (d, *J* = 0.6 Hz), 129.8, 129.6, 128.9 (d, *J* = 19.7 Hz), 128.4 (d, *J* = 22.0 Hz), 127.5, 127.0, 113.71, 113.65, 96.4 (d, *J* = 185.9 Hz), 66.2, 66.0, 55.3, 55.2, 51.13, 51.12, 47.9, 47.8, 47.7, 46.9 (d, *J* = 17.0 Hz), 46.8 (d, *J* = 17.2 Hz), 44.2 (d, *J* = 2.4 Hz), 43.9 (d, *J* = 2.4 Hz), 31.6 (d, *J* = 22.3 Hz), 31.5 (d, *J* = 24.8 Hz), 28.0 (d, *J* = 7.1 Hz), 26.7 (d, *J* = 5.5 Hz), 26.6 (d, *J* = 5.5 Hz), 24.4 (d, *J* = 3.4 Hz), 23.0, 22.9, 21.6, 21.5, 18.9, 18.30, 18.27, 14.4; ^{19}F NMR (377 MHz, $CDCl_3$) δ –142.9 (major), –145.0 (minor); HRMS (ESI) calcd for $C_{29}H_{35}FN_2NaO_4S$ $[M+Na]^+$ 549.2194, found 549.2176; CHIRALPAK AD-H, *n*-hexane/*i*-PrOH = 90:10, 1.0 mL/min, retention times: 15.3 min (major isomer) and 16.5 min (minor isomer).

(+)-(*E*)-2-Fluoro-4-{(2*R*,3*R*)-4-(*Z*)-4-methoxybenzylidene-2,3-dimethyl-1-(4-methylphenyl)-pyrrolidin-3-yl}-1-(pyrrolidin-1-yl)but-2-en-1-one [(+)-4ea]

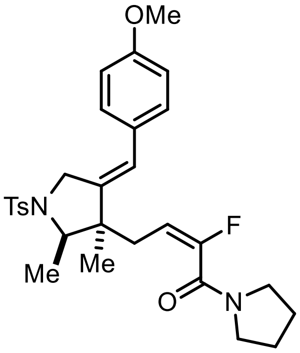

The following experimental data were acquired using a mixture of isomers [58:42 d.r., 98:2 e.r. (major)]. Pale yellow oil; $[\alpha]^{25}_{D}$ +1.4 (*c* 2.26, $CHCl_3$); **major diastereomer:** ^{1}H NMR (400 MHz, $CDCl_3$) δ 7.70 (d, *J* = 8.2 Hz, 2H), 7.30–7.26 (m, 2H), 7.10–7.07 (m, 2H), 6.88 (d, *J* = 8.4 Hz, 2H), 6.10 (t, *J* = 2.3 Hz, 1H), 5.55 (dt, *J* = 23.4, 8.2 Hz, 1H), 4.35 (dd, *J* = 14.9, 2.3 Hz, 1H), 4.11 (dd, *J* = 14.9, 2.4 Hz, 1H), 3.823 (s, 3H), 3.50–3.42 (m, 4H), 3.19 (q, *J* = 6.5 Hz, 1H), 2.67 (ddd, *J* = 15.0, 8.0, 1.7 Hz, 1H), 2.53–2.42 (m, 1H), 2.40 (s, 3H), 1.94–1.76 (m, 4H), 1.25 (d, *J* = 6.4 Hz, 3H), 0.93 (s, 3H); **partial protons of minor diastereomer:** ^{1}H NMR (400 MHz, $CDCl_3$) δ 7.76 (d, *J* = 8.3 Hz, 2H), 6.12 (t, *J* = 2.2 Hz, 1H), 5.32 (dt, *J* = 23.4, 8.3 Hz, 1H), 4.24 (dd, *J* = 14.4, 2.6 Hz, 1H), 4.19 (dd, *J* = 14.4, 2.1 Hz, 1H), 3.819 (s, 3H), 3.70–3.65 (m, 1H), 2.29–2.18 (m, 2H), 1.08 (s, 3H), 1.07 (d, *J* = 6.4 Hz, 3H); ^{13}C NMR (101 MHz, $CDCl_3$) δ 160.3 (d, *J* = 32.6 Hz), 160.2

(d, *J* = 32.9 Hz), 158.7, 158.6, 152.4 (d, *J* = 262.5 Hz), 152.2 (d, *J* = 262.0 Hz), 143.4, 143.2, 139.64, 139.63, 136.4, 134.8, 129.67, 129.66, 129.01, 128.98, 127.4, 127.2, 122.4, 121.5, 113.9, 112.5 (d, *J* = 19.0 Hz), 111.4 (d, *J* = 18.2 Hz), 63.8, 62.5, 55.3, 50.5, 49.9 (d, *J* = 2.1 Hz,), 49.4 (d, *J* = 1.9 Hz), 48.8, 47.2 (d, *J* = 8.4 Hz), 47.1 (d, *J* = 7.3 Hz), 46.2, 46.1, 34.2 (d, *J* = 5.8 Hz), 29.6 (d, *J* = 6.1 Hz), 26.04 (d, *J* = 3.1 Hz), 26.01 (d, *J* = 2.7 Hz), 23.73, 23.66, 22.8, 21.5, 19.1, 18.6, 17.3, 15.8; ^{19}F NMR (377 MHz, $CDCl_3$) δ –112.6 (major), –112.3 (minor); HRMS (ESI) calcd for $C_{29}H_{35}FN_2NaO_4S$ [M+Na]$^+$ 549.2194, found 549.2176; CHIRALPAK IF-3, *n*-hexane/*i*-PrOH = 95:5, 1.0 mL/min, retention times: 213.1 min (major isomer) and 185.4 min (minor isomer).

Reaction using 1f and 2a: 3fa [11.6 mg, 0.0207 mmol, 41% yield, 83:17 d.r., >99:1 e.r. (major)] and **4fa** [7.9 mg, 0.014 mmol, 28% yield, 79:21 d.r., 93:7 e.r. (major)] were obtained from **1f** (20.9 mg, 0.0500 mmol) and **2a** (8.6 mg, 0.0600 mmol). Two diastereomers of **3fa** were isolated separately (major diastereomer: 9.7 mg, 0.0173 mmol, 34% yield, >99:1 e.r.; minor diastereomer: 1.9 mg, 0.0034 mmol, 7% yield), and two diastereomers of **4fa** were isolated separately (major diastereomer: 6.2 mg, 0.0110 mmol, 22% yield, 93:7 e.r.; minor diastereomer: 1.7 mg, 0.0030 mmol, 6% yield).

(–)-{(3*R*,5*R*,7a*R*)-4-(4-Bromophenyl)-5-fluoro-3-methyl-2-(4-methylphenyl)-2,3,5,6,7,7a-hexahydro-1*H*-isoindol-5-yl}(pyrrolidin-1-yl)methanone [(–)-3fa (major diastereomer)]

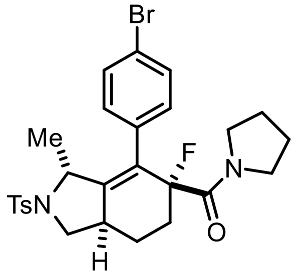

Colorless oil; [α]$^{25}_{D}$ –80.0 (*c* 1.14, $CHCl_3$, >99:1 e.r.); ^{1}H NMR (400 MHz, $CDCl_3$) δ 7.60 (d, *J* = 8.3 Hz, 2H), 7.41 (d, *J* = 8.5 Hz, 2H), 7.33 (d, *J* = 7.9 Hz, 2H), 6.78 (d, *J* = 7.7 Hz, 2H), 3.85–3.78 (m, 2H), 3.43–3.28 (m, 2H), 3.20–3.14 (m, 1H), 2.96–2.89 (m, 2H), 2.60 (dd, *J* = 10.9, 8.6 Hz, 1H), 2.48 (s, 3H), 2.36–2.32 (m, 1H), 2.02–1.80 (m, 3H), 1.72–1.49 (m, 4H), 1.21 (d, *J* = 6.5 Hz, 3H); ^{13}C NMR (101 MHz, $CDCl_3$) δ 168.5 (d, *J* = 27.2 Hz), 147.5 (d, *J* = 6.5 Hz), 143.7, 134.4, 133.3, 131.9, 131.3, 129.7, 128.8 (d, *J* = 19.1 Hz), 127.7, 122.0, 96.7 (d, *J* = 186.3 Hz), 57.6 (d, *J* = 1.2 Hz), 54.5, 47.6, 46.8 (d, *J* = 16.7 Hz), 36.7 (d, *J* = 2.7 Hz), 33.6 (d, *J* = 23.6 Hz), 26.6 (d, *J* = 5.3 Hz), 24.3 (d, *J* = 7.1 Hz), 22.9 (d, *J* = 3.2 Hz), 22.8, 21.6; ^{19}F NMR (377 MHz, $CDCl_3$) δ –145.9; HRMS (ESI) calcd for $C_{27}H_{30}BrFN_2NaO_3S$ [M+Na]$^+$ 583.1037, found 583.1030; CHIRALPAK AD-H, *n*-hexane/*i*-PrOH = 90:10, 1.0 mL/min, retention times: 9.5 min (major isomer) and 11.1 min (minor isomer).

Minor diastereomer: Colorless oil; ^{1}H NMR (400 MHz, $CDCl_3$) δ 7.71 (d, *J* = 8.2 Hz, 2H), 7.45–7.42 (m, 2H), 7.31 (d, *J* = 8.0 Hz, 2H), 6.95 (d, *J* = 8.4 Hz, 2H), 4.62–4.55 (m, 1H), 3.83 (dd, *J* = 11.5, 7.8 Hz, 1H), 3.52–3.35 (m, 3H), 2.98–2.89 (m, 2H), 2.44 (s, 3H), 2.39–2.30 (m, 2H), 1.96–1.81 (m, 3H), 1.78–1.67 (m, 3H), 1.65–1.59 (m, 1H), 0.85 (d, *J* = 6.7 Hz, 3H); ^{19}F NMR (377 MHz, $CDCl_3$) δ –144.1.

(+)-(*E*)-4-[(3*R*,5*S*)-4-{(*Z*)-4-Bromobenzylidene}-3,5-dimethyl-1-(4-methylphenyl)pyrrolidin-3-yl]-2-fluoro-1-(pyrrolidin-1-yl)but-2-en-1-one [(+)-4fa (major diastereomer)]

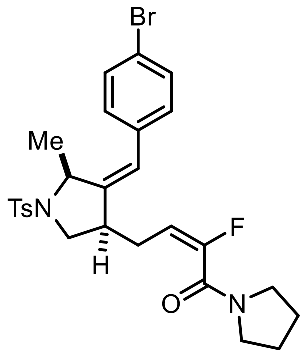

Colorless oil; $[\alpha]^{25}_{D}$ +40.2 (*c* 0.98, $CHCl_3$, 93:7 e.r.); ^1H NMR (400 MHz, $CDCl_3$) δ 7.67 (d, *J* = 8.3 Hz, 2H), 7.46 (d, *J* = 8.5 Hz, 2H), 7.26 (d, *J* = 7.9 Hz, 2H), 7.02 (d, *J* = 8.5 Hz, 2H), 6.10 (s, 1H), 5.55–5.46 (m, 1H), 4.75 (ddd, *J* = 13.2, 6.5, 1.9 Hz, 1H), 3.56–3.45 (m, 5H), 3.29 (dd, *J* = 11.0, 5.1 Hz, 1H), 2.70–2.58 (m, 3H), 2.41 (s, 3H), 1.91–1.84 (m, 4H), 1.35 (d, *J* = 6.6 Hz, 3H); ^{13}C NMR (101 MHz, $CDCl_3$) δ 160.1 (d, *J* = 32.0 Hz), 152.4 (d, *J* = 264.0 Hz), 146.2, 143.6, 135.1, 134.9, 131.7, 129.71, 129.69, 127.5, 122.3, 121.0, 113.2 (d, *J* = 18.3 Hz), 57.3, 50.6, 47.2 (d, *J* = 10.1 Hz), 46.4, 44.6 (d, *J* = 2.6 Hz), 29.6 (d, *J* = 6.4 Hz), 26.2 (d, *J* = 3.3 Hz), 23.7, 21.5, 20.7; ^{19}F NMR (377 MHz, $CDCl_3$) δ –113.4; HRMS (ESI) calcd for $C_{27}H_{30}BrFN_2NaO_3S$ $[M+Na]^+$ 583.1037, found 583.1034; CHIRALPAK ID-3, *n*-hexane/*i*-PrOH = 90:10, 1.0 mL/min, retention times: 34.5 min (major isomer) and 21.5 min (minor isomer).

Minor diastereomer: Colorless oil; ^1H NMR (400 MHz, $CDCl_3$) δ 7.62 (d, *J* = 8.2 Hz, 2H), 7.46–7.43 (m, 2H), 7.23 (d, *J* = 8.0 Hz, 2H), 6.95 (d, *J* = 8.5 Hz, 2H), 6.05 (s, 1H), 5.48 (dt, *J* = 22.6, 8.1 Hz, 1H), 4.61 (q, *J* = 6.4 Hz, 1H), 3.66 (dd, *J* = 9.2, 7.8 Hz, 1H), 3.58–3.49 (m, 3H), 3.09–3.00 (m, 1H), 2.81–2.74 (m, 2H), 2.40 (s, 3H), 2.48–2.29 (m, 1H), 1.94–1.87 (m, 5H), 1.37 (d, *J* = 6.5 Hz, 3H); ^{19}F NMR (377 MHz, $CDCl_3$) δ –113.5.

Reaction using 1g and 2a: 3ga [12.5 mg, 0.0229 mmol, 46% yield, >99:1 d.r., >99:1 e.r. (major)] and **4ga** [11.9 mg, 0.0218 mmol, 44% yield, 92:8 d.r., 97:3 e.r. (major)] were obtained from **1g** (20.1 mg, 0.0500 mmol) and **2a** (8.6 mg, 0.0600 mmol). Two diastereomers of **4ga** were obtained as a mixture.

(–)-{(3*R*,5*R*,7a*R*)-5-Fluoro-3,4-diphenyl-2-(4-methylphenyl)-2,3,5,6,7,7a-hexahydro-1*H*-isoindol-5-yl}(pyrrolidin-1-yl)methanone [(–)-3ga]

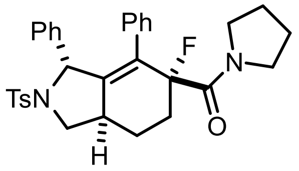

The absolute configuration was determined by X-ray crystallographic analysis (Figure S2). White solid; mp 147.1–147.9 °C; $[\alpha]^{25}_{D}$ –17.9 (*c* 1.28, $CHCl_3$, >99:1 e.r.); ^1H NMR (400 MHz, $CDCl_3$) δ 7.51 (d, *J* = 8.2 Hz, 2H), 7.28–7.24 (m, 3H), 7.20–7.07 (m, 5H), 6.75–6.73 (m, 2H), 6.67–6.65 (m, 2H), 4.85 (dd, *J* = 4.8, 1.3 Hz, 1H), 3.97 (t, *J* = 8.2 Hz, 1H), 3.35–3.09 (m, 4H), 2.96 (dd, *J* = 10.6, 8.6 Hz, 1H), 2.70–2.62 (m, 1H), 2.45 (s, 3H), 2.38 (dt, *J* = 13.0, 3.3 Hz, 1H), 2.17–2.06 (m, 2H), 1.89 (dtd, *J* = 22.1, 13.3, 4.6 Hz, 1H), 1.62–1.44 (m, 3H), 1.35–1.25 (m, 1H); ^{13}C NMR (101 MHz, $CDCl_3$) δ 169.0 (d, *J* = 27.2 Hz), 146.2 (d, *J* = 6.7 Hz), 143.5, 140.9 (d, *J* = 3.0 Hz), 135.1, 134.2, 131.3 (d, *J* = 18.9 Hz), 130.2, 129.6, 127.94, 127.88, 127.66, 127.65, 127.2 (2C), 96.7 (d, *J* = 187.2 Hz), 65.0 (d, *J* = 1.0 Hz), 54.2, 47.6, 46.7 (d, *J* = 16.9 Hz), 38.0 (d, *J* = 2.7 Hz), 33.9 (d, *J* = 23.8 Hz), 26.5 (d, *J* = 5.4 Hz), 25.2 (d, *J* = 6.9 Hz), 22.8, 21.6; ^{19}F NMR (377 MHz, $CDCl_3$) δ –145.9; HRMS (ESI) calcd for $C_{32}H_{33}FN_2NaO_3S$ $[M+Na]^+$ 567.2088, found 567.2080; CHIRALPAK AD-H,

n-hexane/*i*-PrOH = 90:10, 1.0 mL/min, retention times: 15.3 min (major isomer) and 26.9 min (minor isomer).

(+)-(*E*)-4-{(3*R*,5*S*)-4-(*Z*)-Benzylidene-3-methyl-1-(4-methylphenyl)-5-phenyl-pyrrolidin-3-yl}-2-fluoro-1-(pyrrolidin-1-yl)but-2-en-1-one [(+)-4ga]

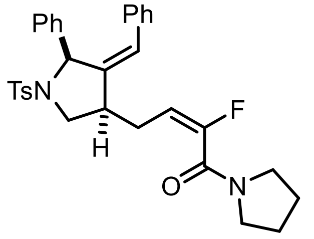

The following experimental data were acquired using a mixture of isomers [92:8 d.r., 97:3 e.r. (major)]. The absolute configuration was determined by X-ray crystallographic analysis (Figure S3). White solid; mp 152.8–153.9 °C; $[\alpha]^{25}_{D}$ +8.0 (*c* 1.19, $CHCl_3$); **major diastereomer:** ^1H NMR (400 MHz, $CDCl_3$) δ 7.48–7.43 (m, 4H), 7.32–7.20 (m, 6H), 7.05–7.00 (m, 4H), 6.32 (s, 1H), 5.85 (s, 1H), 5.43 (dt, *J* = 22.5, 8.3 Hz, 1H), 3.87 (dd, *J* = 12.7, 8.4 Hz, 1H), 3.47–3.43 (m, 4H), 2.97 (dd, *J* = 12.8, 8.0 Hz, 1H), 2.66–2.48 (m, 2H), 2.36 (s, 3H), 2.39–2.31 (m, 1H), 1.86–1.80 (m, 4H); **partial protons of minor diastereomer:** ^1H NMR (400 MHz, $CDCl_3$) δ 6.26 (s, 1H), 5.70 (s, 1H), 3.63 (dd, *J* = 10.6, 8.6 Hz, 1H), 3.60–3.53 (m, 4H), 3.11 (dd, *J* = 10.3, 8.6 Hz, 1H); ^{13}C NMR (101 MHz, $CDCl_3$) δ 160.0 (d, *J* = 32.3 Hz), 152.1 (d, *J* = 263.6 Hz), 143.3, 141.0, 138.0, 136.1, 135.6, 129.5, 128.48, 128.46, 128.0, 127.9, 127.8, 127.4, 127.3, 127.0, 113.3 (d, *J* = 18.1 Hz), 64.9, 50.4, 47.1 (d, *J* = 9.7 Hz), 46.2, 44.7 (d, *J* = 2.5 Hz), 30.0 (d, *J* = 6.2 Hz), 26.1 (d, *J* = 3.2 Hz), 23.7, 21.5; ^{19}F NMR (377 MHz, $CDCl_3$) δ –113.8 (major), –113.4 (minor); HRMS (ESI) calcd for $C_{32}H_{33}FN_2NaO_3S$ $[M+Na]^+$ 567.2088, found 567.2058; CHIRALPAK IG-3, *n*-hexane/*i*-PrOH = 70:30, 1.0 mL/min, retention times: 60.9 min (major isomer) and 54.9 min (minor isomer).

Reaction using 1d and 2b: 3db [18.6 mg, 0.0363 mmol, 36% yield, 78:22 d.r., 94:6 e.r. (major)] and **4db** [19.9 mg, 0.0388 mmol, 39% yield, 67:33 d.r., 72:28 e.r. (major)] were obtained from **1d** (35.4 mg, 0.100 mmol) and **2b** (19.1 mg, 0.120 mmol). Two diastereomers of **3db** were isolated separately (major diastereomer: 14.4 mg, 0.0281 mmol, 28% yield, 94:6 e.r.; minor diastereomer: 4.2 mg, 0.0082 mmol, 8% yield), whereas two diastereomers of **4db** were obtained as a mixture.

(–)-{(1*S*,5*R*,7a*R*)-5-Fluoro-1,7a-dimethyl-2-(4-methylphenyl)-4-phenyl-2,3,5,6,7,7a-hexahydro-1*H*-isoindol-5-yl}(morpholino)methanone [(–)-3db]

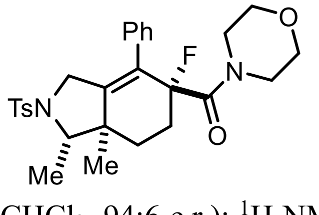

Colorless oil; $[\alpha]^{25}_{D}$ –31.7 (*c* 1.72, $CHCl_3$, 94:6 e.r.); ^1H NMR (400 MHz, $CDCl_3$) δ 7.54 (d, *J* = 8.2 Hz, 2H), 7.31–7.28 (m, 5H), 6.89–6.87 (m, 2H), 3.91 (dd, *J* = 15.6, 9.1 Hz, 1H), 3.59–3.55 (m, 1H), 3.51–3.46 (m, 2H), 3.42–3.32 (m, 4H), 3.21–3.18 (m, 1H), 2.87–2.81 (m, 2H), 2.47 (s, 3H), 2.33 (dt, *J* = 13.6, 3.8 Hz, 1H), 2.18 (dtd, *J* = 23.3, 13.5, 4.0 Hz, 1H), 1.85 (td, *J* = 13.5, 4.1 Hz, 1H), 1.66 (dt, *J* = 13.0, 3.7 Hz, 1H), 1.39 (d, *J* = 6.4 Hz, 3H), 1.23 (s, 3H); ^{13}C NMR (101 MHz, $CDCl_3$) δ 169.3 (d, *J* = 24.4 Hz), 145.5 (d, *J* = 6.8 Hz), 143.6, 135.9, 133.2, 129.8, 129.5 (d, *J* = 0.9 Hz), 129.1 (d, *J* = 19.2 Hz), 128.5, 128.0, 127.5, 97.7 (d, *J* = 187.3 Hz), 66.6 (d, *J* = 27.7 Hz), 66.1, 51.1 (d, *J* = 1.7 Hz), 46.7, 46.5, 43.9 (d, *J* = 2.5 Hz), 43.7, 32.0 (d, *J* = 24.7 Hz), 31.4 (d, *J* = 6.5 Hz), 21.6, 18.4 (d, *J* = 3.3 Hz), 14.5; ^{19}F NMR (377 MHz, $CDCl_3$) δ –138.5; HRMS (ESI) calcd for $C_{28}H_{33}FN_2NaO_4S$ $[M+Na]^+$ 535.2037, found 535.2036; CHIRALPAK AD-H, *n*-hexane/*i*-PrOH = 90:10, 1.0 mL/min, retention times: 21.6 min (major isomer) and 16.7 min (minor isomer).

Minor diastereomer: Colorless oil, ^1H NMR (400 MHz, $CDCl_3$) δ 7.68 (d, *J* = 8.2 Hz, 2H),

7.33–7.28 (m, 5H), 7.09–7.07 (m, 2H), 3.78 (q, *J* = 6.6 Hz, 1H), 3.73 (dd, *J* = 14.6, 8.1 Hz, 1H), 3.80–3.60 (m, 3H), 3.51–3.41 (m, 6H), 3.08–3.05 (m, 1H), 2.41 (s, 3H), 2.34–2.11 (m, 3H), 1.22 (d, *J* = 6.6 Hz, 3H), 0.86 (s, 3H); ^{19}F NMR (377 MHz, $CDCl_3$) δ –140.7.

(–)-(*E*)-4-{(2*R*,3*R*)-4-(*Z*)-Benzylidene-2,3-dimethyl-1-(4-methylphenyl)-pyrrolidin-3-yl}-2-fluoro-1-morpholinobut-2-en-1-one [(–)-4db]

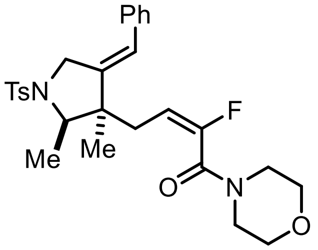

The following experimental data were acquired using a mixture of isomers [67:33 d.r., 72:28 e.r. (major)]. Colorless oil; $[\alpha]^{25}_{D}$ –5.9 (*c* 1.59, $CHCl_3$); **major diastereomer:** ^{1}H NMR (400 MHz, $CDCl_3$) δ 7.76 (d, *J* = 8.3 Hz, 2H), 7.36 (t, *J* = 7.5 Hz, 2H), 7.38–7.24 (m, 3H), 7.16 (d, *J* = 6.7 Hz, 2H), 6.20 (t, *J* = 2.2 Hz, 1H), 5.37 (dt, *J* = 22.3, 8.3 Hz, 1H), 4.28 (dd, *J* = 14.6, 2.8 Hz, 1H), 4.19 (dd, *J* = 14.6, 2.0 Hz, 1H), 3.73 (q, *J* = 6.6 Hz, 1H), 3.69–3.60 (br m, 6H), 3.46–3.36 (m, 2H), 2.41 (s, 3H), 2.16 (dd, *J* = 14.7, 8.5 Hz, 1H), 2.07 (ddd, *J* = 14.6, 7.9, 1.7 Hz, 1H), 1.11 (s, 3H), 1.06 (d, *J* = 6.5 Hz, 3H); **partial protons of minor diastereomer:** ^{1}H NMR (400 MHz, $CDCl_3$) δ 7.69 (d, *J* = 8.2 Hz, 2H), 7.14 (d, *J* = 6.5 Hz, 2H), 6.16 (t, *J* = 2.3 Hz, 1H), 5.56 (dt, *J* = 22.5, 8.1 Hz, 1H), 4.37 (dd, *J* = 15.1, 2.3 Hz, 1H), 4.13 (dd, *J* = 15.1, 2.4 Hz, 1H), 3.18 (q, *J* = 6.5 Hz, 1H), 2.51 (ddd, *J* = 14.8, 8.0, 1.3 Hz, 1H), 2.40 (s, 3H), 2.32 (ddd, *J* = 14.7, 8.2, 0.9 Hz, 1H), 1.27 (d, *J* = 6.5 Hz, 3H), 0.96 (s, 3H); ^{13}C NMR (101 MHz, $CDCl_3$) δ 160.6 (d, *J* = 31.6 Hz), 160.5 (d, *J* = 31.8 Hz), 151.5 (d, *J* = 262.0 Hz), 151.4 (d, *J* = 261.7 Hz), 143.5, 143.3, 141.8, 141.7, 136.6, 136.11, 136.06, 134.6, 129.7, 128.6, 128.4, 127.4, 127.3, 127.2, 123.2, 122.4, 111.7 (d, *J* = 17.5 Hz), 110.8 (d, *J* = 17.0 Hz), 66.8 (d, *J* = 29.6 Hz), 66.7, 63.7, 62.5, 50.5, 49.9 (d, *J* = 2.1 Hz), 49.3 (d, *J* = 1.9 Hz), 48.6, 46.8 (d, *J* = 5.4 Hz), 42.4, 42.3, 34.4 (d, *J* = 6.0 Hz), 29.7 (d, *J* = 6.3 Hz), 22.7, 21.5, 18.6, 17.2, 15.7; ^{19}F NMR (377 MHz, $CDCl_3$) δ –109.1 (major), –109.2 (minor); HRMS (ESI) calcd for $C_{28}H_{33}FN_2NaO_4S$ $[M+Na]^+$ 535.2037, found 535.2051; CHIRALPAK IF-3, *n*-hexane/*i*-PrOH = 90:10, 1.0 mL/min, retention times: 48.3 min (major isomer) and 44.0 min (minor isomer).

Reaction using 1e and 2b: 3eb [8.1 mg, 0.015 mmol, 30% yield, 82:18 d.r., 97:3 e.r. (major)] and **4eb** [7.0 mg, 0.013 mmol, 26% yield, 60:40 d.r., 85:15 e.r. (major)] were obtained from **1e** (19.2 mg, 0.0500 mmol) and **2b** (9.5 mg, 0.0600 mmol). Two diastereomers of **3eb** and **4eb** were obtained as a mixture, respectively.

(–)-{(1*S*,5*R*,7a*R*)-5-Fluoro-4-(4-methoxyphenyl)-1,7a-dimethyl-2-(4-methylphenyl)-2,3,5,6,7,7a-hexahydro-1*H*-isoindol-5-yl}(morpholino)methanone [(–)-3eb]

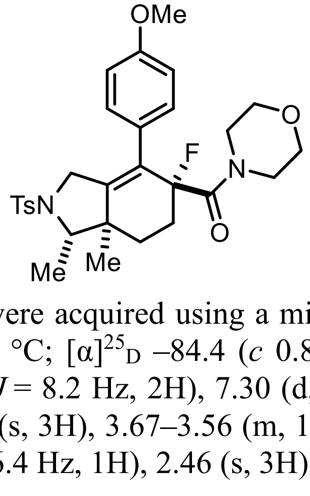

The following experimental data were acquired using a mixture of isomers [82:18 d.r., 97:3 e.r. (major)]. White solid; mp 75.0–76.3 °C; $[\alpha]^{25}_{D}$ –84.4 (*c* 0.81, $CHCl_3$); **major diastereomer:** ^{1}H NMR (400 MHz, $CDCl_3$) δ 7.54 (d, *J* = 8.2 Hz, 2H), 7.30 (d, *J* = 8.0 Hz, 2H), 6.85–6.78 (m, 4H), 3.92 (dd, *J* = 15.7, 8.9 Hz, 1H), 3.83 (s, 3H), 3.67–3.56 (m, 1H), 3.53–3.36 (m, 6H), 3.26–3.17 (m, 1H), 3.01–2.97 (m, 1H), 2.82 (q, *J* = 6.4 Hz, 1H), 2.46 (s, 3H), 2.33–2.12 (m, 2H), 1.82 (td, *J* = 13.3,

4.0 Hz, 1H), 1.64 (dt, J = 13.0, 3.6 Hz, 1H), 1.38 (d, J = 6.4 Hz, 3H), 1.22 (s, 3H); **partial protons of minor diastereomer:** ^{1}H NMR (400 MHz, $CDCl_3$) δ 7.69 (d, J = 8.3 Hz, 2H), 6.99 (d, J = 8.5 Hz, 2H), 3.81 (s, 3H), 2.41 (s, 3H), 0.86 (s, 3H); ^{13}C NMR (101 MHz, $CDCl_3$) δ 169.4 (d, J = 24.4 Hz), 159.3, 159.2, 145.5 (d, J = 6.8 Hz), 144.8 (d, J = 6.6 Hz), 143.6, 143.2, 136.3, 133.3, 130.7 (d, J = 0.9 Hz), 130.5, 129.7, 129.6, 128.8 (d, J = 19.2 Hz), 128.1, 127.5, 127.1, 113.8, 97.8 (d, J = 186.8 Hz), 66.8, 66.5, 66.1, 65.8, 55.3, 55.2, 51.1 (d, J = 1.4 Hz), 47.8, 46.7 (d, J = 20.1 Hz), 44.2 (d, J = 2.0 Hz), 43.9 (d, J = 2.4 Hz), 43.7, 31.9 (d, J = 24.5 Hz), 31.3 (d, J = 6.3 Hz), 27.7 (d, J = 6.9 Hz), 24.5 (d, J = 3.2 Hz), 21.6, 21.5, 18.9, 18.4 (d, J = 3.3 Hz), 14.5; ^{19}F NMR (377 MHz, $CDCl_3$) δ –138.6 (major), –140.9 (minor); HRMS (ESI) calcd for $C_{29}H_{35}FN_2NaO_5S$ $[M+Na]^+$ 565.2143, found 565.2138; CHIRALPAK ID-3, *n*-hexane/*i*-PrOH = 70:30, 1.0 mL/min, retention times: 27.1 min (major isomer) and 30.5 min (minor isomer).

(–)-(*E*)-2-Fluoro-4-[(2*R*,3*R*)-4-{(*Z*)-4-methoxybenzylidene}-2,3-dimethyl-1-(4-methylphenyl)pyrrolidin-3-yl]-1-morpholinobut-2-en-1-one [(–)-4eb]

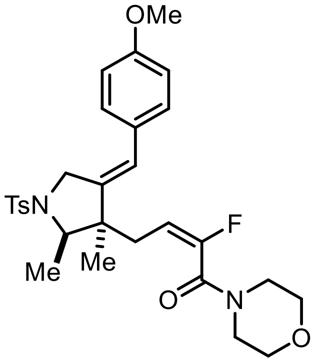

The following experimental data were acquired using a mixture of isomers [60:40 d.r., 85:15 e.r. (major)]. White solid; mp 53.3–55.1 °C; $[\alpha]^{25}_{D}$ –7.3 (*c* 0.70, $CHCl_3$); **major diastereomer:** ^{1}H NMR (400 MHz, $CDCl_3$) δ 7.76 (d, J = 8.3 Hz, 2H), 7.30 (d, J = 7.8 Hz, 2H), 7.11–7.08 (m, 2H), 6.91–6.87 (m, 2H), 6.13 (t, J = 2.2 Hz, 1H), 5.36 (dt, J = 22.4, 8.3 Hz, 1H), 4.24 (dd, J = 14.4, 2.8 Hz, 1H), 4.17 (dd, J = 14.5, 2.0 Hz, 1H), 3.82 (s, 3H), 3.74–3.62 (m, 7H), 3.47–3.36 (m, 2H), 2.41 (s, 3H), 2.13 (dd, J = 14.6, 8.8 Hz, 1H), 2.05 (ddd, J = 14.6, 7.8, 1.8 Hz, 1H), 1.09 (s, 3H), 1.05 (d, J = 6.5 Hz, 3H); **partial protons of minor diastereomer:** ^{1}H NMR (400 MHz, $CDCl_3$) δ 7.69 (d, J = 8.3 Hz, 2H), 6.09 (t, J = 2.3 Hz, 1H), 5.56 (dt, J = 22.5, 8.1 Hz, 1H), 4.34 (dd, J = 15.0, 2.3 Hz, 1H), 4.10 (dd, J = 14.9, 2.4 Hz, 1H), 3.83 (s, 3H), 3.15 (q, J = 6.5 Hz, 1H), 2.49 (ddd, J = 14.8, 8.0, 1.3 Hz, 1H), 2.40 (s, 3H), 2.30 (ddd, J = 14.8, 8.3, 0.8 Hz, 1H), 1.27 (d, J = 6.5 Hz, 3H), 0.94 (s, 3H); ^{13}C NMR (101 MHz, $CDCl_3$) δ 160.6 (d, J = 31.9 Hz), 158.78, 158.76, 151.4 (d, J = 261.8 Hz), 143.5, 143.3, 139.5, 139.3, 136.6, 134.6, 129.70, 129.69, 128.9, 128.8, 127.4, 127.2, 122.7, 121.8, 114.0, 111.8 (d, J = 17.6 Hz), 110.9 (d, J = 16.8 Hz), 67.0, 66.7, 63.7, 62.6, 55.3, 50.5, 49.8 (d, J = 2.0 Hz), 49.2 (d, J = 1.9 Hz), 48.6, 46.8 (d, J = 2.6 Hz), 42.3 (d, J = 8.1 Hz), 34.4 (d, J = 5.6 Hz), 29.7 (d, J = 6.6 Hz), 22.7, 21.5, 18.6, 17.2, 15.7; ^{19}F NMR (377 MHz, $CDCl_3$) δ –109.26 (major), –109.32 (minor); HRMS (ESI) calcd for $C_{29}H_{35}FN_2NaO_5S$ $[M+Na]^+$ 565.2143, found 565.2136; CHIRALPAK IE-3, *n*-hexane/*i*-PrOH = 80:20, 1.0 mL/min, retention times: 102.1 min (major isomer) and 92.6 min (minor isomer).

Reaction using 1g and 2c: **3gc** [9.4 mg, 0.015 mmol, 29% yield, >99:1 d.r., >99:1 e.r. (major)] and **4gc** [19.6 mg, 0.0305 mmol, 61% yield, 70:30 d.r., >99:1 e.r. (major)] were obtained from **1g** (20.1 mg, 0.0500 mmol) and **2c** (14.5 mg, 0.0600 mmol), purified by silica gel PTLC (eluent: *n*-hexane/EtOAc = 1:1) according to the general procedure. Two diastereomers of **4gc** were isolated separately (major diastereomer: 14.2 mg, 0.0221 mmol, 44% yield, >99:1 e.r.; minor diastereomer: 5.4 mg, 0.0084 mmol, 17% yield).

(+)-(3*R*,5*R*,7a*R*)-5-Fluoro-2-(4-methylphenyl)-*N*,*N*,3,4-tetraphenyl-2,3,5,6,7,7a-hexahydro-1*H*-isoindole-5-carboxamide [(+)-3gc]

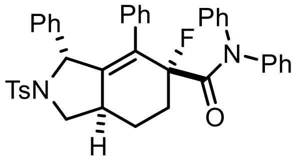

White solid; mp 197.6 °C (decomp.); $[\alpha]^{25}_{D}$ +2.7 (*c* 0.94, $CHCl_3$, >99:1 e.r.); ^{1}H NMR (400 MHz, $CDCl_3$) δ 7.57 (d, *J* = 8.3 Hz, 2H), 7.44–7.40 (m, 1H), 7.33–7.28 (m, 4H), 7.16–7.03 (m, 9H), 6.76–6.69 (m, 8H), 4.97 (dd, *J* = 4.6, 1.1 Hz, 1H), 4.00 (t, *J* = 8.1 Hz, 1H), 3.11–3.02 (m, 1H), 2.93 (dd, *J* = 10.6, 8.6 Hz, 1H), 2.53 (dt, *J* = 13.2, 3.1 Hz, 1H), 2.45 (s, 3H), 2.09–2.03 (m, 2H), 1.95–1.82 (m, 1H); ^{13}C NMR (101 MHz, $CDCl_3$) δ 170.9 (d, *J* = 24.7 Hz), 145.8 (d, *J* = 6.6 Hz), 143.6, 140.7 (d, *J* = 3.1 Hz), 135.2, 133.9, 131.2, 131.0, 130.9, 129.7, 128.8, 128.1, 128.0, 127.9, 127.8, 127.2, 127.1, 126.9, 126.8, 96.7 (d, *J* = 194.5 Hz), 64.6 (d, *J* = 1.1 Hz), 54.4, 37.6 (d, *J* = 2.5 Hz), 34.8 (d, *J* = 23.7 Hz), 24.7 (d, *J* = 6.2 Hz), 21.6; ^{19}F NMR (377 MHz, $CDCl_3$) δ –145.9; HRMS (ESI) calcd for $C_{40}H_{35}FN_2NaO_3S$ $[M+Na]^+$ 665.2245, found 665.2222; CHIRALPAK AD-H, *n*-hexane/*i*-PrOH = 95:5, 1.0 mL/min, retention times: 30.4 min (major isomer) and 37.6 min (minor isomer).

(+)-(*E*)-4-{(3*R*,5*S*)-4-(*Z*)-Benzylidene-3-methyl-1-(4-methylphenyl)-5-phenylpyrrolidin-3-yl}-2-fluoro-*N*,*N*-diphenylbut-2-enamide [(+)-4gc (major diastereomer)]

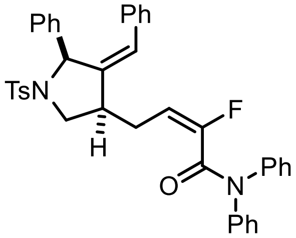

White solid; mp 73.2–74.7 °C; $[\alpha]^{25}_{D}$ +2.5 (*c* 1.42, $CHCl_3$, >99:1 e.r.); ^{1}H NMR (400 MHz, $CDCl_3$) δ 7.49–7.47 (m, 2H), 7.44 (d, *J* = 8.3 Hz, 2H), 7.32–7.21 (m, 12H), 7.10–7.08 (m, 4H), 7.04–7.01 (m, 4H), 6.30 (s, 1H), 5.86 (s, 1H), 5.32 (dt, *J* = 21.3, 8.2 Hz, 1H), 3.83 (dd, *J* = 12.8, 8.5 Hz, 1H), 2.92 (dd, *J* = 13.0, 8.0 Hz, 1H), 2.64–2.56 (m, 1H), 2.52–2.45 (m, 1H), 2.36 (s, 3H), 2.35–2.27 (m, 1H); ^{13}C NMR (101 MHz, $CDCl_3$) δ 161.7 (d, *J* = 30.8 Hz), 151.5 (d, *J* = 264.0 Hz), 143.3, 141.8, 140.9, 138.0, 136.0, 135.5, 129.5, 129.2, 128.5, 128.0, 127.90, 127.88, 127.5, 127.3, 127.2, 127.1, 126.6 (2C), 114.4 (d, *J* = 17.3 Hz), 64.8, 50.4, 44.7 (d, *J* = 2.4 Hz), 30.4 (d, *J* = 5.6 Hz), 21.5; ^{19}F NMR (377 MHz, $CDCl_3$) δ –110.9; HRMS (ESI) calcd for $C_{40}H_{35}FN_2NaO_3S$ $[M+Na]^+$ 665.2245, found 665.2231; CHIRALPAK ID-3, *n*-hexane/*i*-PrOH = 95:5, 1.0 mL/min, retention times: 229.5 min (major isomer) and 300.8 min (minor isomer).

Minor diastereomer: White solid; ^{1}H NMR (400 MHz, $CDCl_3$) δ 7.42–7.36 (m, 8H), 7.31–7.27 (m, 5H), 7.25–7.20 (m, 7H), 6.98–6.93 (m, 4H), 6.25 (s, 1H), 5.73 (s, 1H), 5.26 (dt, *J* = 21.3, 7.9 Hz, 1H), 3.62 (dd, *J* = 10.1, 8.4 Hz, 1H), 3.09 (dd, *J* = 10.1, 8.7 Hz, 1H), 3.03–2.96 (m, 1H), 2.81–2.73 (m, 1H), 2.41–2.32 (m, 1H), 2.28 (s, 3H); ^{19}F NMR (377 MHz, $CDCl_3$) δ –110.5.

Reaction using 1h and 2a: 3ha [7.0 mg, 0.020 mmol, 39% yield, >99:1 d.r., 98:2 e.r. (major)] and **4ha** [7.2 mg, 0.020 mmol, 40% yield, 92:8 d.r., 85:15 e.r. (major)] were obtained from **1h** (10.7 mg, 0.0500 mmol) and **2a** (21.5 mg, 0.150 mmol), using (*R*)-P-Phos (3.9 mg, 0.0060 mmol) and $[Rh(cod)_2]BF_4$ (2.1 mg, 0.0050 mmol) in CH_2Cl_2 (1.0 mL). Two diastereomers of **4ha** were obtained as a mixture.

(–)-(3*S*,3a*R*,6*R*)-6-Fluoro-3,3a-dimethyl-7-phenyl-6-(pyrrolidine-1-carbonyl)-3a,4,5,6-tetrahydroisobenzofuran-1(3*H*)-one [(–)-3ha]

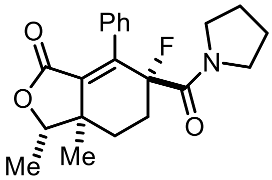

White solid; mp 107.3–109.0 °C; $[\alpha]^{25}_{D}$ –54.9 (*c* 0.70, $CHCl_3$, 98:2 e.r.); ^{1}H NMR (400 MHz, $CDCl_3$) δ 7.34–7.30 (m, 3H), 7.16–7.13 (m, 2H), 4.31 (q, *J* = 6.5 Hz, 1H), 3.43–3.28 (m, 3H), 2.88–2.80 (m, 1H), 2.46–2.37 (m, 2H), 2.35–2.21 (m, 1H), 1.82–1.77 (m, 1H), 1.72–1.54 (m, 3H), 1.49–1.43 (m, 1H), 1.35 (d, *J* = 6.5 Hz, 3H), 1.25 (s, 3H); ^{13}C NMR (101 MHz, $CDCl_3$) δ 168.3 (d, *J* = 25.4 Hz), 167.5, 142.0 (d, *J* = 19.4 Hz), 137.6 (d, *J* = 5.3 Hz), 133.5, 128.9, 128.2, 127.7, 96.4 (d, *J* = 191.3 Hz), 83.2, 47.8, 46.7 (d, *J* = 17.0 Hz), 42.4 (d, *J* = 2.1 Hz), 31.3 (d, *J* = 24.5 Hz), 31.0 (d, *J* = 7.0 Hz), 26.6 (d, *J* = 5.4 Hz), 22.8, 18.4 (d, *J* = 3.1 Hz), 13.6; ^{19}F NMR (377 MHz, $CDCl_3$) δ –150.7; HRMS (ESI) calcd for $C_{21}H_{24}FNNaO_3$ $[M+Na]^+$ 380.1632, found 380.1633; CHIRALPAK AD-H, *n*-hexane/*i*-PrOH = 90:10, 1.0 mL/min, retention times: 9.9 min (major isomer) and 21.1 min (minor isomer).

(–)-(4*S*,5*R*)-3-(*Z*)-Benzylidene-4-{(*E*)-3-fluoro-4-oxo-4-(pyrrolidin-1-yl)but-2-en-1-yl}-4,5-dimethyldihydrofuran-2(3*H*)-one [(–)-4ha]

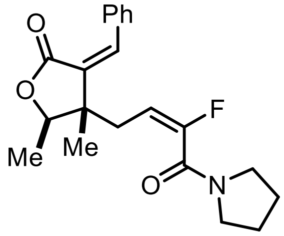

The following experimental data were acquired using a mixture of isomers [92:8 d.r., 85:15 e.r. (major)]. White solid; mp 109.0–110.3 °C; $[\alpha]^{25}_{D}$ –7.4 (*c* 1.27, $CHCl_3$); **major diastereomer:** ^{1}H NMR (400 MHz, $CDCl_3$) δ 7.87–7.85 (m, 2H), 7.39–7.29 (m, 3H), 6.74 (s, 1H), 5.54 (dt, *J* = 22.9, 8.5 Hz, 1H), 4.49 (q, *J* = 6.5 Hz, 1H), 3.50–3.33 (m, 4H), 2.78–2.65 (m, 2H), 1.84–1.64 (m, 4H), 1.31 (d, *J* = 6.5 Hz, 3H), 1.23 (s, 3H); **partial protons of minor diastereomer:** ^{1}H NMR (400 MHz, $CDCl_3$) δ 6.65 (s, 1H), 4.29 (q, *J* = 6.5 Hz, 1H), 1.41 (d, *J* = 6.6 Hz, 3H), 1.26 (s, 3H); ^{13}C NMR (101 MHz, $CDCl_3$) δ 168.6, 168.4, 159.8 (d, *J* = 31.9 Hz), 152.9 (d, *J* = 265.2 Hz), 139.1, 138.5, 133.2, 132.3, 130.8, 130.7, 129.6, 129.5, 128.02, 127.96, 110.73 (d, *J* = 19.3 Hz), 110.69 (d, *J* = 19.9 Hz), 81.8, 80.0, 47.7 (d, *J* = 2.3 Hz), 47.4 (d, *J* = 2.2 Hz), 47.0 (d, *J* = 9.9 Hz), 46.1, 46.0, 34.8 (d, *J* = 6.1 Hz), 30.1 (d, *J* = 6.3 Hz), 25.8 (d, *J* = 3.2 Hz), 25.6 (d, *J* = 3.2 Hz), 23.5, 23.4, 21.43, 21.35, 16.5, 13.5; ^{19}F NMR (377 MHz, $CDCl_3$) δ –110.8 (major), –110.6 (minor); HRMS (ESI) calcd for $C_{21}H_{24}FNNaO_3$ $[M+Na]^+$ 380.1632, found 380.1641; CHIRALPAK IG-3, *n*-hexane/*i*-PrOH = 90:10, 1.0 mL/min, retention times: 61.1 min (major isomer) and 53.1 min (minor isomer).

Reaction using 1i and 2a: 3ia [8.0 mg, 0.018 mmol, 37% yield, >99:1 d.r., 97:3 e.r. (major)] and **4ia** [9.6 mg, 0.022 mmol, 45% yield, 94:6 d.r., 83:17 e.r. (major)] were obtained from **1i** (14.7 mg, 0.0500 mmol) and **2a** (21.5 mg, 0.150 mmol) using (*R*)-P-Phos (3.9 mg, 0.0060 mmol) and $[Rh(cod)_2]BF_4$ (2.1 mg, 0.0050 mmol) in CH_2Cl_2 (1.0 mL). Two diastereomers of **4ia** were obtained as a mixture.

(–)-(3*S*,3a*R*,6*R*)-7-(4-Bromophenyl)-6-fluoro-3,3a-dimethyl-6-(pyrrolidine-1-carbonyl)-3a,4,5,6-tetrahydroisobenzofuran-1(3*H*)-one [(–)-3ia]

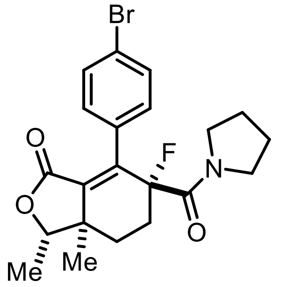

The absolute configuration was determined by X-ray crystallographic analysis (Figure S4). White solid; mp 155.8–157.2 °C; $[\alpha]^{25}_{D}$ –56.7 (*c* 4.31, $CHCl_3$, 97:3 e.r.); ^1H NMR (400 MHz, $CDCl_3$) δ 7.44 (d, *J* = 8.5 Hz, 2H), 7.05 (d, *J* = 7.9 Hz, 2H), 4.30 (q, *J* = 6.5 Hz, 1H), 3.50–3.29 (m, 3H), 3.07–2.99 (m, 1H), 2.41–2.26 (m, 3H), 1.80–1.59 (m, 5H), 1.35 (d, *J* = 6.5 Hz, 3H), 1.24 (s, 3H); ^{13}C NMR (101 MHz, $CDCl_3$) δ 167.8 (d, *J* = 25.3 Hz), 167.4, 140.9 (d, *J* = 19.8 Hz), 138.0 (d, *J* = 5.7 Hz), 132.4, 130.9, 130.8, 122.6, 96.4 (d, *J* = 191.6 Hz), 83.2, 47.9, 46.8 (d, *J* = 16.7 Hz), 42.5 (d, *J* = 2.0 Hz), 30.9 (d, *J* = 24.1 Hz), 30.5 (d, *J* = 7.1 Hz), 26.6 (d, *J* = 5.3 Hz), 22.8, 18.3 (d, *J* = 2.9 Hz), 13.5; ^{19}F NMR (377 MHz, $CDCl_3$) δ –151.5; HRMS (ESI) calcd for $C_{21}H_{23}BrFNNaO_3$ $[M+Na]^+$ 458.0738, found 458.0761; CHIRALPAK AD-H, *n*-hexane/*i*-PrOH = 90:10, 1.0 mL/min, retention times: 9.9 min (major isomer) and 25.1 min (minor isomer).

(–)-(4*S*,5*R*)-3-(*Z*)-4-Bromobenzylidene-4-{(*E*)-3-fluoro-4-oxo-4-(pyrrolidin-1-yl)but-2-en-1-yl}-4,5-dimethyldihydrofuran-2(3*H*)-one [(–)-4ia]

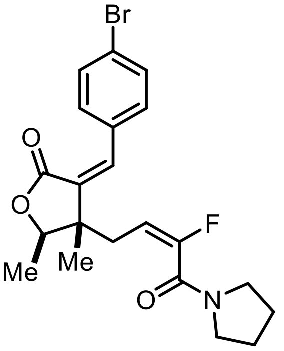

The relative configuration was determined by X-ray crystallographic analysis of (±)-**4ia** (Figure S5). The following experimental data were acquired using a mixture of isomers [94:6 d.r., 83:17 e.r. (major)]. White solid; mp 103.6–105.3 °C; $[\alpha]^{25}_{D}$ –11.0 (*c* 2.26, $CHCl_3$); **major diastereomer:** ^1H NMR (400 MHz, $CDCl_3$) δ 7.74 (d, *J* = 8.4 Hz, 2H), 7.50 (d, *J* = 8.6 Hz, 2H), 6.67 (s, 1H), 5.52 (dt, *J* = 22.9, 8.5 Hz, 1H), 4.50 (q, *J* = 6.5 Hz, 1H), 3.50–3.41 (m, 4H), 2.77–2.65 (m, 2H), 1.89–1.73 (m, 4H), 1.32 (d, *J* = 6.5 Hz, 3H), 1.23 (s, 3H); **partial protons of minor diastereomer:** ^1H NMR (400 MHz, $CDCl_3$) δ 7.53 (d, *J* = 8.4 Hz, 2H), 1.41 (d, *J* = 6.6 Hz, 3H); ^{13}C NMR (101 MHz, $CDCl_3$) δ 168.4, 159.9 (d, *J* = 32.2 Hz), 153.1 (d, *J* = 265.4 Hz), 137.8, 133.4, 132.4, 132.2, 131.3, 124.0, 111.0 (d, *J* = 19.3 Hz), 80.3, 47.6 (d, *J* = 2.2 Hz), 47.2 (d, *J* = 10.2 Hz), 46.3, 34.9 (d, *J* = 6.1 Hz), 26.1 (d, *J* = 3.4 Hz), 23.6, 21.5, 16.7; ^{19}F NMR (377 MHz, $CDCl_3$) δ –110.6 (major), –110.4 (minor); HRMS (ESI) calcd for $C_{21}H_{23}BrFNNaO_3$ $[M+Na]^+$ 458.0738, found 458.0758; CHIRALPAK IG-3, *n*-hexane/*i*-PrOH = 90:10, 1.0 mL/min, retention times: 64.1 min (major isomer) and 56.2 min (minor isomer).

Reaction using 1j and 2a: 3ja [8.4 mg, 0.020 mmol, 40% yield, >99:1 d.r., 95:5 e.r. (major)] and **4ja** [8.8 mg, 0.021 mmol, 42% yield, >99:1 d.r., 82:18 e.r. (major)] were obtained from **1j** (27.7 mg, 0.0500 mmol) and **2a** (21.5 mg, 0.150 mmol), using (*R*)-P-Phos (3.9 mg, 0.0060 mmol) and $[Rh(cod)_2]BF_4$ (2.1 mg, 0.0050 mmol) in CH_2Cl_2 (1.0 mL).

(–)-(3*S*,3a*R*,6*R*)-6-Fluoro-3a-methyl-3,7-diphenyl-6-(pyrrolidine-1-carbonyl)-3a,4,5,6-tetrahydroisobenzofuran-1(3*H*)-one [(–)-3ja]

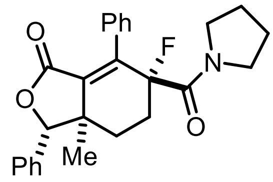

Colorless oil; $[\alpha]^{25}_D$ –25.5 (*c* 0.88, $CHCl_3$, 95:5 e.r.); ^1H NMR (400 MHz, $CDCl_3$) δ 7.41–7.31 (m, 8H), 7.20–7.18 (m, 2H), 5.30 (s, 1H), 3.47–3.34 (m, 3H), 2.90–2.75 (m, 2H), 2.45 (dt, *J* = 13.9, 3.5 Hz, 1H), 2.24 (dtd, *J* = 23.1, 14.1, 3.9 Hz, 1H), 1.89 (dt, *J* = 12.9, 3.2 Hz, 1H), 1.74–1.57 (m, 3H), 1.52–1.46 (m, 1H), 0.94 (s, 3H); ^{13}C NMR (101 MHz, $CDCl_3$) δ 168.3 (d, *J* = 25.3 Hz), 167.2, 142.8 (d, *J* = 19.7 Hz), 137.3 (d, *J* = 5.2 Hz), 134.8, 133.4, 128.9, 128.44, 128.35, 128.2, 127.8, 125.5, 96.4 (d, *J* = 191.6 Hz), 87.5, 47.9, 46.8 (d, *J* = 16.9 Hz), 44.1 (d, *J* = 2.0 Hz), 31.6 (d, *J* = 7.1 Hz), 31.1 (d, *J* = 24.4 Hz), 26.6 (d, *J* = 5.4 Hz), 22.9, 19.6 (d, *J* = 2.8 Hz); ^{19}F NMR (377 MHz, $CDCl_3$) δ –150.5; HRMS (ESI) calcd for $C_{26}H_{26}FNNaO_3$ $[M+Na]^+$ 442.1789, found 442.1795; CHIRALPAK AD-H, *n*-hexane/*i*-PrOH = 90:10, 1.0 mL/min, retention times: 21.8 min (major isomer) and 65.2 min (minor isomer).

(–)-(4*S*,5*R*)-3-(*Z*)-Benzylidene-4-{(*E*)-3-fluoro-4-oxo-4-(pyrrolidin-1-yl)but-2-en-1-yl}-4-methyl-5-phenyldihydrofuran-2(3*H*)-one [(–)-4ja]

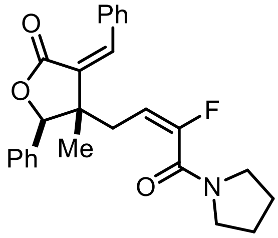

Colorless oil; $[\alpha]^{25}_D$ –18.6 (*c* 0.84, $CHCl_3$, 82:18 e.r.); ^1H NMR (400 MHz, $CDCl_3$) δ 7.93–7.90 (m, 2H), 7.44–7.32 (m, 6H), 7.25–7.22 (m, 2H), 6.74 (s, 1H), 5.66 (dt, *J* = 22.9, 8.4 Hz, 1H), 5.38 (s, 1H), 3.55–3.42 (m, 4H), 2.90 (ddd, *J* = 14.9, 8.4, 1.2 Hz, 1H), 2.86 (ddd, *J* = 14.9, 8.4, 1.3 Hz, 1H), 1.86–1.64 (m, 4H), 0.87 (s, 3H); ^{13}C NMR (101 MHz, $CDCl_3$) δ 168.8, 160.0 (d, *J* = 32.2 Hz), 153.4 (d, *J* = 265.8 Hz), 140.1, 136.7, 133.3, 131.5, 131.0, 129.9, 128.4 (2C), 128.2, 126.3, 110.9 (d, *J* = 19.3 Hz), 85.1, 49.0 (d, *J* = 2.2 Hz), 47.2 (d, *J* = 10.1 Hz), 46.4, 36.2 (d, *J* = 6.0 Hz), 26.0 (d, *J* = 3.3 Hz), 23.8, 23.6; ^{19}F NMR (377 MHz, $CDCl_3$) δ –110.1; HRMS (ESI) calcd for $C_{26}H_{26}FNNaO_3$ $[M+Na]^+$ 442.1789, found 442.1786; CHIRALPAK IG-3, *n*-hexane/*i*-PrOH = 80:20, 1.0 mL/min, retention times: 50.1 min (major isomer) and 43.1 min (minor isomer).

Reaction using 1k and 2a: 3ka [6.9 mg, 0.016 mmol, 32% yield, >99:1 d.r., 99:1 e.r. (major)] and **4ka** [13.3 mg, 0.0309 mmol, 62% yield, >99:1 d.r., 74:26 e.r. (major)] were obtained from **1k** (14.4 mg, 0.0500 mmol) and **2a** (21.5 mg, 0.150 mmol), using (*R*)-P-Phos (3.9 mg, 0.0060 mmol) and $[Rh(cod)_2]BF_4$ (2.1 mg, 0.0050 mmol) in CH_2Cl_2 (1.0 mL).

(–)-(8*R*,10a*R*,10b*S*)-8-Fluoro-10a-methyl-7-phenyl-8-(pyrrolidine-1-carbonyl)-8,9,10,10a, 10b,11-hexahydro-6*H*-isoindolo[2,1-a]indol-6-one [(–)-3ka]

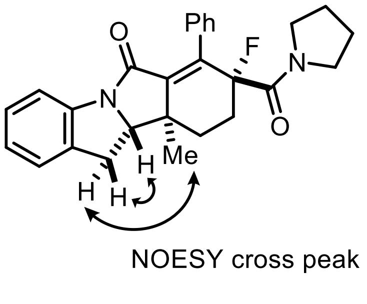

The relative configuration was determined by the NOESY experiment. White solid; mp 84.4–86.2 °C; $[\alpha]^{25}_{D}$ –2.4 (*c* 0.84, $CHCl_3$, 99:1 e.r.); ^{1}H NMR (400 MHz, $CDCl_3$) δ 7.57 (d, *J* = 7.8 Hz, 1H), 7.35–7.31 (m, 3H), 7.22–7.12 (m, 4H), 6.98 (td, *J* = 11.2, 1.0 Hz, 1H), 4.51 (t, *J* = 9.4 Hz, 1H), 3.45–3.31 (m, 3H), 3.18 (dd, *J* = 16.2, 9.3 Hz, 1H), 3.00 (dd, *J* = 16.3, 9.5 Hz, 1H), 2.80–2.72 (m, 1H), 2.67 (td, *J* = 13.6, 3.5 Hz, 1H), 2.47 (dt, *J* = 13.8, 3.6 Hz, 1H), 2.28 (dtd, *J* = 24.1, 14.0, 3.9 Hz, 1H), 1.93 (dt, *J* = 12.9, 3.4 Hz, 1H), 1.70–1.55 (m, 3H), 1.47–1.39 (m, 1H), 1.32 (s, 3H); ^{13}C NMR (101 MHz, $CDCl_3$) δ 168.9 (d, *J* = 26.0 Hz), 162.6, 145.3 (d, *J* = 5.8 Hz), 139.5, 138.5 (d, *J* = 19.2 Hz), 134.3, 133.0, 129.3, 127.9, 127.8, 127.6, 125.3, 124.1, 114.5, 96.4 (d, *J* = 190.0 Hz), 69.7, 47.9, 46.8 (d, *J* = 17.1 Hz), 42.8 (d, *J* = 2.2 Hz), 32.6 (d, *J* = 6.9 Hz), 31.5 (d, *J* = 24.7 Hz), 27.8, 26.6 (d, *J* = 5.5 Hz), 22.9, 20.6 (d, *J* = 3.0 Hz); ^{19}F NMR (377 MHz, $CDCl_3$) δ –148.9; HRMS (ESI) calcd for $C_{27}H_{27}FN_2NaO_2$ $[M+Na]^+$ 453.1949, found 453.1955; CHIRALPAK OD-H, *n*-hexane/*i*-PrOH = 80:20, 1.0 mL/min, retention times: 8.4 min (major isomer) and 16.4 min (minor isomer).

(–)-(1*S*,9a*R*)-2-(*Z*)-Benzylidene-1-{(*E*)-3-fluoro-4-oxo-4-(pyrrolidin-1-yl)but-2-en-1-yl}-1-methyl-1,2,9,9a-tetrahydro-3*H*-pyrrolo[1,2-a]indol-3-one [(–)-4ka]

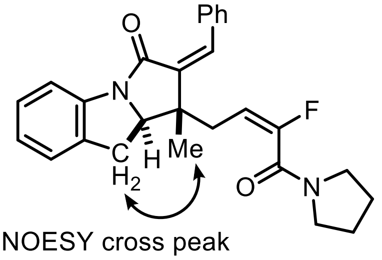

The relative configuration was determined by the NOESY experiment. White solid; mp 56.8–58.4 °C; $[\alpha]^{25}_{D}$ –46.3 (*c* 1.23, $CHCl_3$, 74:26 e.r.); ^{1}H NMR (400 MHz, $CDCl_3$) δ 7.85–7.83 (m, 2H), 7.73–7.71 (m, 1H), 7.39–7.34 (m, 2H), 7.32–7.28 (m, 1H), 7.22–7.19 (m, 2H), 7.05–7.01 (m, 1H), 6.63 (s, 1H), 5.67 (ddd, *J* = 23.1, 9.1, 7.7 Hz, 1H), 4.70 (dd, *J* = 9.9, 9.2 Hz, 1H), 3.59–3.51 (m, 4H), 3.14 (dd, *J* = 15.9, 10.0 Hz, 1H), 3.05 (dd, *J* = 16.0, 9.1 Hz, 1H), 2.95 (dd, *J* = 14.6, 9.1 Hz, 1H), 2.77 (ddd, *J* = 14.6, 7.7, 2.2 Hz, 1H), 1.94–1.81 (m, 4H), 1.28 (s, 3H); ^{13}C NMR (101 MHz, $CDCl_3$) δ 163.2, 160.1 (d, *J* = 32.3 Hz), 152.7 (d, *J* = 264.5 Hz), 142.8, 139.5, 134.3, 134.2, 133.8, 130.4, 128.6, 127.9, 127.7, 125.3, 124.4, 114.9, 112.1 (d, *J* = 18.9 Hz), 65.7, 47.2 (d, *J* = 10.0 Hz), 46.7 (d, *J* = 2.2 Hz), 46.4, 33.4 (d, *J* = 6.0 Hz), 29.4, 26.1 (d, *J* = 3.4 Hz), 24.7, 23.7; ^{19}F NMR (377 MHz, $CDCl_3$) δ –111.6; HRMS (ESI) calcd for $C_{27}H_{27}FN_2NaO_2$ $[M+Na]^+$ 453.1949, found 453.1967; CHIRALPAK IF-3, *n*-hexane/*i*-PrOH = 70:30, 1.0 mL/min, retention times: 29.4 min (major isomer) and 11.7 min (minor isomer).

Reaction using 1l and 2a: 3la [14.0 mg, 0.040 mmol, 40% yield, 85:15 d.r., 77:23 e.r. (major)] and **4la** [6.0 mg, 0.0174 mmol, 17% yield, >99:1 d.r., 73:27 e.r. (major)] were obtained from **1l** (20.0 mg, 0.100 mmol) and **2a** (42.9 mg, 0.300 mmol), using (*R*)-BINAP (7.5 mg, 0.0120 mmol), purified by silica gel PTLC twice (eluent: *n*-hexane/EtOAc = 2:1 and CH_2Cl_2/EtOAc = 20:1). Two diastereomers of **3la** were isolated separately (major diastereomer: 11.8 mg, 0.0344 mmol, 34% yield, 77:23 e.r.; minor diastereomer: 2.2 mg, 0.0064 mmol, 6% yield). A trace amount (ca. <2%) of another diastereomer of **4la** was detected in a crude reaction mixture.

(–)-{(1*S*,5*R*,7a*R*)-5-Fluoro-1,7a-dimethyl-4-phenyl-1,3,5,6,7,7a-hexahydroisobenzofuran-5-yl}(pyrrolidin-1-yl)methanone [(–)-3la (major diastereomer)]

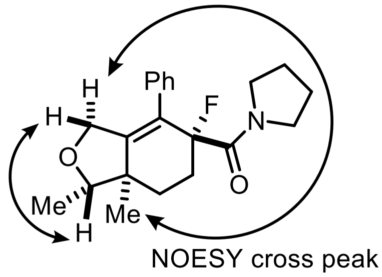

The relative configuration was determined by the NOESY experiment.

Colorless oil; $[\alpha]^{25}_{D}$ –23.7 (*c* 1.44, $CHCl_3$, 77:23 e.r.); ^{1}H NMR (400 MHz, $CDCl_3$) δ 7.29–7.26 (m, 3H), 7.10–7.08 (m, 2H), 4.27 (dd, *J* = 14.4, 8.5 Hz, 1H), 3.95 (dd, *J* = 14.4, 6.8 Hz, 1H), 3.63 (q, *J* = 6.3 Hz, 1H), 3.48–3.32 (m, 3H), 2.84–2.76 (m, 1H), 2.45–2.40 (m, 1H), 2.33–2.13 (m, 2H), 1.70–1.60 (m, 4H), 1.45–1.36 (m, 1H), 1.19 (d, *J* = 6.3 Hz, 3H), 1.15 (s, 3H); ^{13}C NMR (101 MHz, $CDCl_3$) δ 170.1 (d, *J* = 27.7 Hz), 151.7 (d, *J* = 6.5 Hz), 136.7, 129.3 (d, *J* = 1.3 Hz), 128.2, 127.7 (d, *J* = 19.6 Hz), 127.5, 96.8 (d, *J* = 186.2 Hz), 83.7, 67.5 (d, *J* = 1.5 Hz), 47.8, 46.8 (d, *J* = 17.2 Hz), 42.9 (d, *J* = 2.4 Hz), 32.0 (d, *J* = 24.3 Hz), 31.1 (d, *J* = 6.5 Hz), 26.6 (d, *J* = 5.6 Hz), 22.9, 17.2 (d, *J* = 3.5 Hz), 13.7; ^{19}F NMR (377 MHz, $CDCl_3$) δ –143.2; HRMS (ESI) calcd for $C_{21}H_{26}FNNaO_2$ $[M+Na]^+$ 366.1840, found 366.1835; CHIRALPAK AD-H, *n*-hexane/*i*-PrOH = 90:10, 1.0 mL/min, retention times: 5.1 min (major isomer) and 7.6 min (minor isomer).

Minor diastereomer: Colorless oil; ^{1}H NMR (400 MHz, $CDCl_3$) δ 7.31–7.24 (m, 3H), 7.10–7.08 (m, 2H), 4.27 (dd, *J* = 14.4, 8.5 Hz, 1H), 3.95 (dd, *J* = 14.4, 6.8 Hz, 1H), 3.63 (q, *J* = 6.3 Hz, 1H), 3.48–3.32 (m, 3H), 2.84–2.76 (m, 1H), 2.45–2.40 (m, 1H), 2.33–2.13 (m, 2H), 1.71–1.58 (m, 4H), 1.45–1.35 (m, 1H), 1.19 (d, *J* = 6.3 Hz, 3H), 1.15 (s, 3H); ^{19}F NMR (377 MHz, $CDCl_3$) δ –143.3.

(+)-(*E*)-4-{(2*R*,3*S*)-4-(*Z*)-Benzylidene-2,3-dimethyltetrahydrofuran-3-yl}-2-fluoro-1-(pyrrolidin-1-yl)but-2-en-1-one [(+)-4la (major diastereomer)]

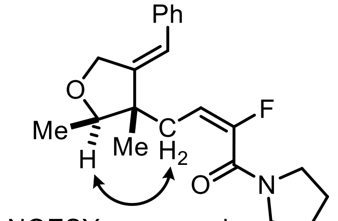

The relative configuration was determined by the NOESY experiment.

Colorless oil; $[\alpha]^{25}_{D}$ +1.8 (*c* 0.30, $CHCl_3$, 73:27 e.r.); ^{1}H NMR (400 MHz, $CDCl_3$) δ 7.37–7.32 (m, 2H), 7.23–7.19 (m, 1H), 7.14 (d, *J* = 7.2 Hz, 2H), 6.17 (t, *J* = 2.5 Hz, 1H), 5.59 (ddd, *J* = 23.7, 8.7, 7.9 Hz, 1H), 4.75 (dd, *J* = 14.4, 2.3 Hz, 1H), 4.58 (dd, *J* = 14.4, 2.7 Hz, 1H), 3.85 (q, *J* = 6.4 Hz, 1H), 3.50–3.44 (m, 4H), 2.70 (dd, *J* = 15.0, 8.7 Hz, 1H), 2.61 (ddd, *J* = 15.0, 7.8, 2.0 Hz, 1H), 1.83–1.73 (m, 4H), 1.17 (d, *J* = 6.4 Hz, 3H), 1.08 (s, 3H); ^{13}C NMR (101 MHz, $CDCl_3$) δ 160.4 (d, *J* = 32.8 Hz), 152.2 (d, *J* = 261.7 Hz), 148.3, 137.2, 128.5, 128.0, 126.6, 120.2, 112.4 (d, *J* = 18.2 Hz), 80.3, 69.2, 49.0 (d, *J* = 1.9 Hz), 47.1 (d, *J* = 9.6 Hz), 46.2, 33.0 (d, *J* = 5.8 Hz), 26.0 (d, *J* = 3.2 Hz), 23.7, 21.5, 15.0; ^{19}F NMR (377 MHz, $CDCl_3$) δ –113.1; HRMS (ESI) calcd for $C_{21}H_{26}FNNaO_2$ $[M+Na]^+$ 366.1840, found 366.1839; CHIRALPAK IG-3, *n*-hexane/*i*-PrOH = 90:10, 1.0 mL/min, retention times: 39.7 min (major isomer) and 28.9 min (minor isomer).

Reaction using 1m and 2a: 3ma [7.4 mg, 0.018 mmol, 35% yield, >99:1 d.r., >99:1 e.r. (major)] and **4ma** [4.9 mg, 0.012 mmol, 23% yield, 80:20 d.r., 76:24 e.r. (major)] were obtained from **1m** (27.7 mg, 0.0500 mmol) and **2a** (21.5 mg, 0.150 mmol), using (*R*)-BINAP (3.7 mg, 0.0600 mmol). Two diastereomers of **4ma** were obtained as a mixture.

(–)-(3*S*,3a*S*,6*R*)-6-Fluoro-3-methyl-3a,7-diphenyl-6-(pyrrolidine-1-carbonyl)-3a,4,5,6-tetrahydroisobenzofuran-1(3*H*)-one [(–)-3ma]

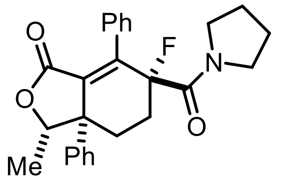

Colorless oil; $[\alpha]^{25}_{D}$ –71.0 (*c* 0.83, $CHCl_3$, >99:1 e.r.); ^{1}H NMR (400 MHz, $CDCl_3$) δ 7.42–7.32 (m, 6H), 7.31–7.25 (m, 4H), 4.57 (q, *J* = 6.5 Hz, 1H), 3.43–3.28 (m, 3H), 2.85–2.70 (m, 2H), 2.58 (dt, *J* = 13.2, 3.2 Hz, 1H), 2.33 (dt, *J* = 13.6, 3.0 Hz, 1H), 1.85 (dtd, *J* = 24.5, 13.9, 3.2 Hz, 1H), 1.71–1.51 (m, 3H), 1.49–1.39 (m, 1H), 1.11 (d, *J* = 6.5 Hz, 3H); ^{13}C NMR (101 MHz, $CDCl_3$) δ 168.5, 168.1 (d, *J* = 29.1 Hz), 144.2 (d, *J* = 18.6 Hz), 138.7 (d, *J* = 2.6 Hz), 136.5 (d, *J* = 5.4 Hz), 133.6, 128.9, 128.6, 128.34, 128.26, 127.8, 127.5, 96.3 (d, *J* = 192.5 Hz), 83.6, 51.1 (d, *J* = 2.2 Hz), 47.8, 46.7 (d, *J* = 17.0 Hz), 31.8 (d, *J* = 3.7 Hz), 31.7 (d, *J* = 21.9 Hz), 26.5 (d, *J* = 5.5 Hz), 22.8, 16.3; ^{19}F NMR (377 MHz, $CDCl_3$) δ –151.1; HRMS (ESI) calcd for $C_{26}H_{26}FNNaO_3$ $[M+Na]^+$ 442.1789, found 442.1789; CHIRALPAK AD-H, *n*-hexane/*i*-PrOH = 80:20, 1.0 mL/min, retention times: 7.0 min (major isomer) and 18.3 min (minor isomer).

(–)-(4*R*,5*R*)-3-(*Z*)-Benzylidene-4-{(*E*)-3-fluoro-4-oxo-4-(pyrrolidin-1-yl)but-2-en-1-yl}-5-methyl-4-phenyldihydrofuran-2(3*H*)-one [(–)-4ma]

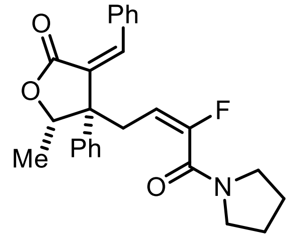

The following experimental data were acquired using a mixture of isomers [80:20 d.r., 76:24 e.r. (major)]. Colorless oil; $[\alpha]^{25}_{D}$ –15.7 (*c* 0.53, $CHCl_3$); **major diastereomer:** ^{1}H NMR (400 MHz, $CDCl_3$) δ 7.92–7.87 (m, 2H), 7.42–7.35 (m, 8H), 6.80 (s, 1H), 5.62 (ddd, *J* = 23.1, 8.8, 7.4 Hz, 1H), 4.67 (q, *J* = 6.5 Hz, 1H), 3.53–3.31 (m, 5H), 3.23 (ddd, *J* = 15.0, 7.4, 2.3 Hz, 1H), 1.82–1.58 (m, 4H), 0.93 (d, *J* = 6.5 Hz, 3H); **partial protons of minor diastereomer:** ^{1}H NMR (400 MHz, $CDCl_3$) δ 6.61 (s, 1H), 2.98 (ddd, *J* = 15.4, 7.7, 1.6 Hz, 1H), 1.35 (d, *J* = 6.6 Hz, 3H); ^{13}C NMR (101 MHz, $CDCl_3$) δ 168.8, 168.5, 160.0 (d, *J* = 32.2 Hz), 153.3 (d, *J* = 265.7 Hz), 143.4, 142.0, 140.7, 133.3, 131.2, 131.1, 130.04, 129.99, 128.9, 128.5, 128.28, 128.25, 128.22, 128.20, 127.5, 127.4, 111.5 (d, *J* = 20.2 Hz), 83.7, 81.3, 55.7 (d, *J* = 2.1 Hz), 47.1 (d, *J* = 10.8 Hz), 46.4, 46.3, 33.6 (d, *J* = 6.1 Hz), 25.9 (d, *J* = 3.5 Hz), 23.6, 23.5, 18.8, 13.4; ^{19}F NMR (377 MHz, $CDCl_3$) δ –110.0 (major), –110.4 (minor); HRMS (ESI) calcd for $C_{26}H_{26}FNNaO_3$ $[M+Na]^+$ 442.1789, found 442.1786; CHIRALPAK IG-3, *n*-hexane/*i*-PrOH = 90:10, 1.0 mL/min, retention times: 58.5 min (major isomer) and 104.6 min (minor isomer).

Synthesis of (+)-(*S*)-3-Phenylbut-3-en-2-yl 3-phenylpropynoate [(+)-1m]

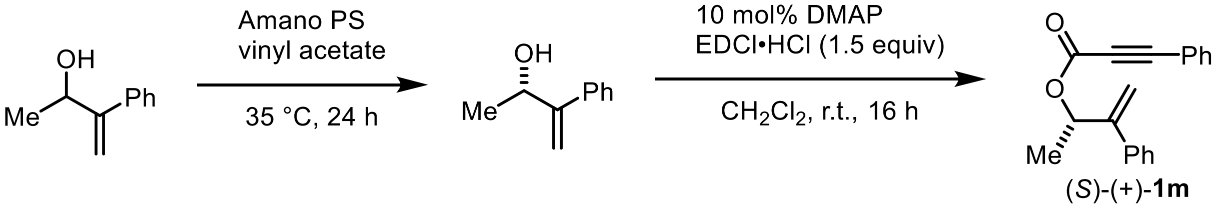

(*S*)-(+)-**1m**

3-Phenylbut-3-en-2-ol (741.0 mg, 5.00 mmol) was added to a suspension of Lipase PS "Amano" IM (3.7050 g) in vinyl acetate (10.0 mL) at room temperature, and the resultant mixture was stirred for 24 h at 35 °C. Then the reaction mixture was filtered, and the filtrate was concentrated. The

resulting crude product was purified by silica gel column chromatography (eluent: *n*-hexane/EtOAc = 4:1) to furnish 3-phenylbut-3-en-2-ol (247.0 mg, 1.67 mmol, 33% yield) as a colorless oil.

To a solution of 3-phenylpropiolic acid (219.2 mg, 1.50 mmol) in CH_2Cl_2 (2.0 mL) were added obtained 3-phenylbut-3-en-2-ol (222.3 mg, 1.50 mmol), EDCI·HCl (431.3 mg, 2.25 mmol) and DMAP (18.3 mg, 0.150 mmol) at 0 °C, and the mixture was stirred at room temperature for 16 h, The reaction was quenched with aqueous NH_4Cl and extracted with EtOAc three times. The combined organic layers were washed with brine, dried over Na_2SO_4, and concentrated. The crude product was purified by silica gel column chromatography (eluent: *n*-hexane/EtOAc = 4:1), to furnish (+)-**1m** (133.5 mg, 0.483 mmol, 32% yield, >99:1 e.r.). The spectroscopic data were consistent with **1m**. The stereochemistry of the product obtained from the subsequent transformation suggests that the stereochemistry of the OH-containing carbon atom is *S*.

$[\alpha]^{25}_{D}$ +70.5° (*c* 1.86, $CHCl_3$, >99:1 e.r.); CHIRALCEL OD-H, *n*-hexane/*i*-PrOH = 99:1, 1.0 mL/min, retention times: 9.8 min (major isomer) and 25.3 min (minor isomer).

Reactions of (+)-1m with 2a

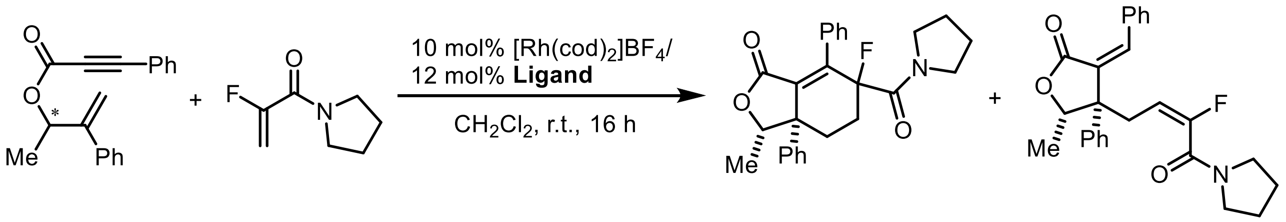

(*S*)-(+)-**1m** **2a** (3 equiv) (–)-**3ma** (–)-**4ma**

Ligand	(–)-**3ma**			(–)-**4ma**		
	% yield	d.r.	e.r.	% yield	d.r.	e.r.
(*R*)-BINAP	59	>99:1	>99:1	40	>99:1	>99:1
(*S*)-BINAP	0	–	–	20	63:37	>99:1

Figure S1. Reactions of (+)-**1m** with **2a** using (*R*)- and (*S*)-BINAP.

The above reactions were conducted according to the synthesis of **3ma** and **4ma**. When using (*R*)-BINAP as a ligand, both cycloaddition and hydroalkenylation proceeded smoothly to afford (–)-**3ma** and (–)-**4ma** in good yields with excellent enantio- and diastereoselectivities, while only **4ma** was obtained in low yield and diastereoselectivity when (*S*)-BINAP was used. These results indicate that only one enantiomer of the racemate is suitable for this PKR system in the presence of Rh/(*R*)-BINAP catalyst. Therefore, we conclude that the reaction of (±)-**1m** with **2a** resulted in the formation of **3ma** and **4ma**, both of which possess the same absolute configuration.

2.4. Rh-Catalyzed Enantioselective PKR (type II) of Racemic 1,6-Enynes with Two Different Acrylamide Derivatives

2.4.1. Substituent Effect at α-Position of Acrylamides (Figure 3a)

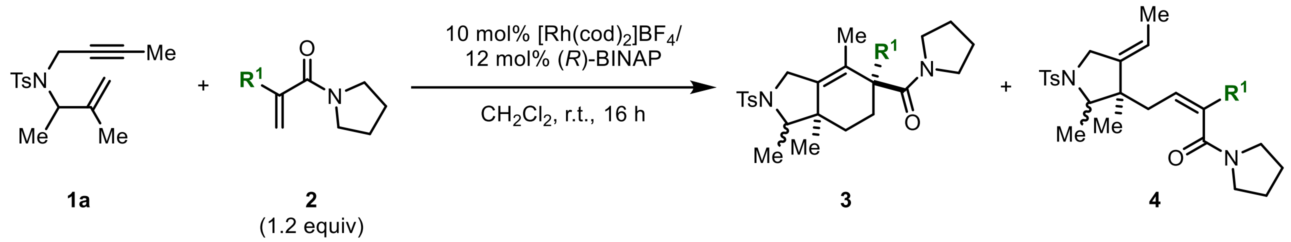

2 (R^1)	3			4		
	% yield	d.r.	e.r.	% yield	d.r.	e.r.
**2d** (H)	72	58:42	96:4	<1	–	–
**2a** (F)	39	87:13	97:3	32	78:22	>99:1
**2e** (Me)	0	–	–	32	52:48	99:1
**2f** (Cl)	0	–	–	5	68:32	>99:1

(*R*)-BINAP (3.7 mg, 0.0060 mmol) and [Rh(cod)$_2$]BF$_4$ (2.1 mg, 0.0050 mmol) were dissolved in CH_2Cl_2 (2.0 mL) in a Schlenk tube, and the mixture was stirred at room temperature for 10 min. After introduction of H_2 and stirring at room temperature for 30 min, the resulting mixture was concentrated to dryness. The residue was dissolved in CH_2Cl_2 (0.5 mL), followed by the addition of a solution of **1a** (15.0 mg, 0.050 mmol) and **2** (0.060 mmol) in CH_2Cl_2 (0.5 mL) The mixture was stirred at room temperature for 16 h, then passed through short-path silica gel column chromatography to remove the Rh complex and concentrated. The crude product was further purified by silica gel PTLC twice (eluent: *n*-hexane/EtOAc = 1:1 and CH_2Cl_2/EtOAc = 10:1) to furnish **3** and/or **4**.

(–)-Pyrrolidin-1-yl{(5*S*,7a*R*)-1,4,7a-trimethyl-2-(4-methylphenyl)-2,3,5,6,7,7a-hexahydro-1*H*-isoindol-5-yl}methanone [(–)-3ad]

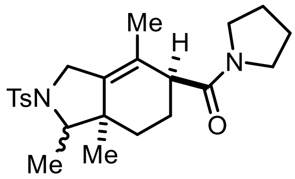

3ad [14.9 mg, 0.0358 mmol, 72% yield, 58:42 d.r., 96:4 e.r. (major)] was prepared from **2d** (8.1 mg, 0.060 mmol). Two diastereomers of **3ad** were obtained as a mixture.

The following experimental data were acquired using a mixture of isomers [58:42 d.r., 96:4 e.r. (major)]. Colorless oil; $[\alpha]^{25}_{D}$ –10.8 (*c* 1.49, $CHCl_3$); **major diastereomer:** ^{1}H NMR (400 MHz, $CDCl_3$) δ 7.69–7.66 (m, 2H), 7.30 (d, *J* = 7.8 Hz, 2H), 4.11 (ddd, *J* = 14.1, 2.6, 1.4 Hz, 1H), 3.82–3.78 (m, 1H), 3.55–3.34 (m, 4H), 3.05 (d, *J* = 7.8 Hz, 1H), 2.72 (q, *J* = 6.4 Hz, 1H), 2.42 (s, 3H), 2.03–1.79 (m, 5H), 1.71–1.65 (m, 1H), 1.47–1.43 (m, 1H), 1.43 (s, 3H), 1.39–1.34 (m, 1H), 1.35 (d, *J* = 6.4 Hz, 3H), 1.00 (s, 3H); **partial protons of minor diastereomer:** ^{1}H NMR (400 MHz, $CDCl_3$) δ 7.77–7.74 (m, 2H), 7.28 (d, *J* = 7.9 Hz, 2H), 4.01 (d, *J* = 13.1 Hz, 1H), 3.86 (ddd, *J* = 13.1, 2.4, 1.4 Hz, 1H), 3.75 (q, *J* = 6.7 Hz, 1H), 2.99 (d, *J* = 4.8 Hz, 1H), 2.41 (s, 3H), 1.50 (s, 3H), 1.23–1.19 (m, 1H), 1.14 (d, *J* = 6.6 Hz, 3H), 0.74 (s, 3H); ^{13}C NMR (101 MHz, $CDCl_3$) δ 172.7, 172.3, 143.4, 142.9, 136.9, 136.0, 135.9, 133.7, 129.8, 129.5, 127.4, 127.1, 124.4, 122.1, 66.2, 50.8, 47.2, 46.9, 46.83, 45.80, 45.7, 43.8, 43.5, 43.0, 42.8, 28.8, 26.3, 26.2, 25.6, 25.1, 24.37, 24.35, 22.9, 22.8, 21.51, 21.47, 18.7, 18.6, 18.1, 17.8, 14.7; HRMS (ESI) calcd for $C_{23}H_{32}N_2NaO_3S$ $[M+Na]^+$

439.2026, found 439.2026; CHIRALPAK IF-3, *n*-hexane/*i*-PrOH = 70:30, 1.0 mL/min, retention times for major diasteremer: 12.3 min (major isomer) and 18.2 min (minor isomer), retention times for minor diasteremer: 14.2 min (major isomer) and 20.3 min (minor isomer).

(+)-(*Z*)-4-{(3*R*,*Z*)-4-Ethylidene-2,3-dimethyl-1-(4-methylphenyl)pyrrolidin-3-yl}-2-methyl-1-(pyrrolidin-1-yl)but-2-en-1-one [(+)-4ae]

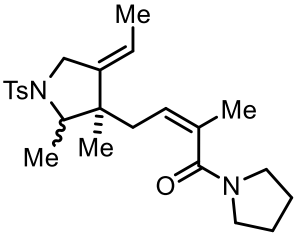

4ae [6.9 mg, 0.0169 mmol, 32% yield, 52:48 d.r., 99:1 e.r. (major)] was prepared from **2e** (8.1 mg, 0.060 mmol). Two diastereomers of **4ae** were obtained as a mixture.

The following experimental data were acquired using a mixture of isomers [52:48 d.r., 99:1 e.r. (major)]. Colorless oil; $[\alpha]^{25}_{D}$ +7.7 (*c* 1.46, $CHCl_3$); **major diastereomer:** ^{1}H NMR (400 MHz, $CDCl_3$) δ 7.71 (d, *J* = 8.1 Hz, 2H), 7.31 (d, *J* = 4.8 Hz, 2H), 5.27–5.23 (m, 1H), 5.16–5.11 (m, 1H), 3.98–3.94 (m, 1H), 3.81–3.80 (m, 1H), 3.32–3.28 (m, 1H), 3.51–3.44 (m, 2H), 3.24 (q, *J* = 6.5 Hz, 1H), 3.19–3.09 (m, 1H), 2.42 (s, 3H), 2.09 (ddd, *J* = 14.8, 7.3, 1.0 Hz, 1H), 1.96–1.85 (m, 7H), 1.77–1.64 (m, 1H), 1.54 (d, *J* = 6.8 Hz, 3H), 1.19 (d, *J* = 6.5 Hz, 3H), 0.73 (s, 3H); **partial protons of minor diastereomer:** ^{1}H NMR (400 MHz, $CDCl_3$) δ 7.74 (d, *J* = 8.3 Hz, 2H), 7.29 (d, *J* = 4.8 Hz, 2H), 5.22–5.19 (m, 1H), 5.07–5.03 (m, 1H), 4.02–3.98 (m, 1H), 3.77–3.76 (m, 1H), 3.65 (q, *J* = 6.5 Hz, 1H), 3.51–3.44 (m, 2H), 3.32–3.28 (m, 1H), 2.41 (s, 3H), 1.81 (d, *J* = 1.0 Hz, 3H), 1.58 (d, *J* = 6.8 Hz, 3H), 1.04 (d, *J* = 6.5 Hz, 3H), 0.90 (s, 3H); ^{13}C NMR (101 MHz, $CDCl_3$) δ 170.2, 170.1, 143.3, 143.0, 141.2, 140.9, 136.5, 135.8, 135.5, 134.9, 129.6, 129.5, 127.3, 127.2, 123.7, 117.2, 116.0, 64.9, 63.9, 48.7, 48.4, 47.6, 47.5, 46.9, 46.7, 44.9, 44.8, 38.4, 33.1, 26.0, 25.9, 24.43, 24.40, 23.36, 21.48, 21.47, 20.1, 20.0, 18.8, 17.1, 16.1, 14.3, 14.1; HRMS (ESI) calcd for $C_{24}H_{34}N_2NaO_3S$ $[M+Na]^+$ 453.2182, found 453.2181; CHIRALPAK IG-3, *n*-hexane/*i*-PrOH = 80:20, 1.0 mL/min, retention times for major diastereomer: 43.1 min (major isomer) and 88.5 min (minor isomer), retention times for minor diastereomer: 57.0 min (major isomer) and 37.3 min (minor isomer).

(+)-(*E*)-2-Chloro-4-{(3*R*,*Z*)-4-ethylidene-2,3-dimethyl-1-(4-methylphenyl)pyrrolidin-3-yl}-1-(pyrrolidin-1-yl)but-2-en-1-one [(+)-4af]

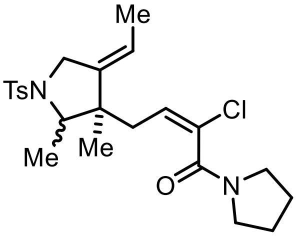

4af [2.2 mg, 0.0049 mmol, 5% yield, 68:32 d.r., >99:1 e.r. (major)] was prepared from **1a** (29.1 mg, 0.100 mmol) and **2f** (19.2 mg, 0.120 mmol). Two diastereomers of **4af** were obtained as a mixture.

The following experimental data were acquired using a mixture of isomers [68:32 d.r., >99:1 e.r. (major)]. Colorless oil; $[\alpha]^{25}_{D}$ +1.7 (*c* 0.19, $CHCl_3$); **major diastereomer:** ^{1}H NMR (400 MHz, $CDCl_3$) δ 7.73 (d, *J* = 8.3 Hz, 2H), 7.33–7.30 (m, 2H), 5.57 (t, *J* = 7.9 Hz, 1H), 5.27–5.22 (m, 1H), 3.97–3.92 (m, 1H), 3.83–3.78 (m, 1H), 3.65 (q, *J* = 6.5 Hz, 1H), 3.53–3.30 (m, 4H), 2.42 (s, 3H), 1.96–1.90 (m, 5H), 1.85 (dd, *J* = 14.7, 7.5 Hz, 1H), 1.60–1.58 (m, 3H), 1.04 (d, *J* = 6.6 Hz, 3H), 0.94 (s, 3H); **partial protons of minor diastereomer:** ^{1}H NMR (400 MHz, $CDCl_3$) δ 7.71 (d, *J* = 8.1 Hz, 2H), 5.77 (t, *J* = 7.7 Hz, 1H), 4.03–3.92 (m, 1H), 3.13 (q, *J* = 6.5 Hz, 1H), 2.43 (s, 3H), 2.25

(dd, J = 14.8, 7.5 Hz, 1H), 2.08 (dd, J = 14.9, 8.0 Hz, 1H), 1.22 (d, J = 6.5 Hz, 3H), 0.79 (s, 3H); ^{13}C NMR (101 MHz, $CDCl_3$) δ 162.9, 162.8, 143.5, 143.3, 140.403, 140.399, 136.4, 134.6, 129.68, 129.66, 128.0, 127.6, 127.5, 127.2, 125.7, 117.9, 116.7, 64.9, 63.8, 49.1, 48.4, 47.8, 47.4, 47.3, 47.2, 45.6, 45.5, 38.3, 33.4, 29.69, 29.65, 25.94, 25.89, 24.3, 22.8, 21.53, 21.51, 18.7, 16.9, 15.7, 14.3, 14.1; HRMS (ESI) calcd for $C_{23}H_{31}ClN_2NaO_3S$ $[M+Na]^+$ 473.1636, found 473.1648; CHIRALPAK IG-3, *n*-hexane/*i*-PrOH = 70:30, 1.0 mL/min, retention times for major diastereomer: 51.1 min (major isomer) and 22.3 min (minor isomer), retention times for minor diastereomer: 27.4 min (major isomer) and 46.3 min (minor isomer).

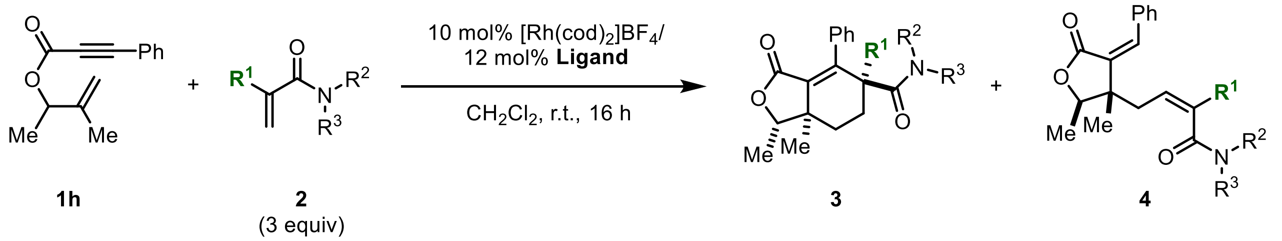

1h + **2** (3 equiv) → **3** + **4**

**2** (R[1], NR[2]R[3])	Ligand	**3**			**4**		
		% yield	d.r.	e.r.	% yield	d.r.	e.r.
**2d** (H, pyrrolidinyl)	(*R*)-P-Phos	0	–	–	0	–	–
**2g** (H, NPhMe)	(*R*)-P-Phos	50	>99:1	85:15	0	–	–
**2a** (F, pyrrolidinyl)	(*R*)-P-Phos	39	>99:1	98:2	40	92:8	85:15
**2e** (Me, pyrrolidinyl)	(*R*)-BINAP	0	–	–	10	>99:1	22:78

Ligand (0.0060 mmol) and $[Rh(cod)_2]BF_4$ (2.1 mg, 0.0050 mmol) were dissolved in CH_2Cl_2 (2.0 mL) in a Schlenk tube, and the mixture was stirred at room temperature for 10 min. After introduction of H_2 and stirring at room temperature for 30 min, the resulting mixture was concentrated to dryness. The residue was dissolved in CH_2Cl_2 (0.5 mL), followed by the addition of a solution of **1h** (10.7 mg, 0.050 mmol) and **2** (0.150 mmol) in CH_2Cl_2 (0.5 mL) The mixture was stirred at room temperature for 16 h, then passed through short-path silica gel column chromatography to remove the Rh complex and concentrated. The crude product was further purified by silica gel PTLC twice (eluent: *n*-hexane/EtOAc = 1:1 and CH_2Cl_2/EtOAc = 10:1) to furnish **3** and/or **4**.

(–)-(1*S*,5*S*,7a*R*)-*N*,1,7a-Trimethyl-3-oxo-*N*,4-diphenyl-1,3,5,6,7,7a-hexahydroisobenzofuran-5-carboxamide [(–)-3hg (major diastereomer)]

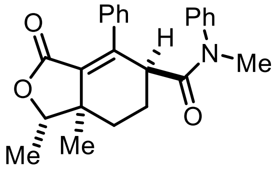

3hg [9.4 mg, 0.0250 mmol, 50% yield, >99:1 d.r., 85:15 e.r. (major)] was prepared from **2g** (24.2 mg, 0.150 mmol) using (*R*)-P-Phos (3.9 mg, 0.0060 mmol). A trace amount (ca. <2%) of another diastereomer of **3hg** was detected in a crude reaction mixture.

White solid; mp 98.4–99.6 °C; $[\alpha]^{25}_D$ –41.5 (*c* 0.81, $CHCl_3$, 85:15 e.r.); ^{1}H NMR (400 MHz, $CDCl_3$) δ 7.36–7.26 (m, 6H), 7.00–6.98 (m, 2H), 6.63 (br s, 2H), 4.33 (q, J = 6.5 Hz, 1H), 3.47 (d, J = 6.3 Hz, 1H), 3.05 (s, 3H), 2.11 (td, J = 13.1, 3.7 Hz, 1H), 1.98–1.92 (m, 1H), 1.88–1.79 (m, 1H), 1.61 (dt, J = 12.5, 3.3Hz, 1H), 1.36 (d, J = 6.5 Hz, 3H), 1.02 (s, 3H); ^{13}C NMR (101 MHz, $CDCl_3$) δ 171.9, 168.6, 144.7, 143.5, 137.4, 133.6, 129.7, 128.0, 127.93, 127.89, 127.8, 127.4, 83.3, 42.7, 42.3, 37.2, 27.5, 23.4, 18.6, 13.8; HRMS (ESI) calcd for $C_{24}H_{25}NNaO_3$ $[M+Na]^+$ 398.1727,

found 398.1714; CHIRALPAK OD-H, *n*-hexane/*i*-PrOH = 90:10, 1.0 mL/min, retention times: 12.7 min (major isomer) and 16.9 min (minor isomer).

(–)-(4*S*,5*R*)-3-{(*Z*)-Benzylidene}-4,5-dimethyl-4-[(*Z*)-3-methyl-4-oxo-4-{pyrrolidin-1-yl}but-2-en-1-yl]dihydrofuran-2(3*H*)-one [(–)-4he]

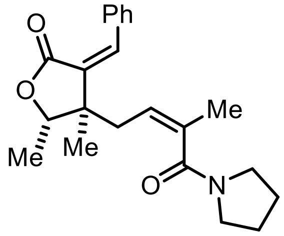

4he (1.8 mg, 0.0051 mmol, 10% yield, >99:1 d.r., 78:22 e.r.) was prepared from **2e** (20.9 mg, 0.150 mmol), using (*R*)-BINAP (3.7 mg, 0.0060 mmol).

Colorless oil; $[\alpha]^{25}_{D}$ –5.2 (*c* 0.53, $CHCl_3$, 78:22 e.r.); ^1H NMR (400 MHz, $CDCl_3$) δ 7.88–7.85 (m, 2H), 7.41–7.35 (m, 3H), 6.72 (s, 1H), 5.35–5.30 (m, 1H), 4.45 (q, J = 6.5 Hz, 1H), 5.52–3.49 (m, 2H), 3.29–3.26 (m, 2H), 2.33–2.21 (m, 2H), 1.92–1.83 (m, 7H), 1.28 (d, J = 6.5 Hz, 3H), 1.19 (s, 3H); ^{13}C NMR (101 MHz, $CDCl_3$) δ 169.9, 168.7, 139.0, 137.4, 133.5, 133.0, 130.8, 129.6, 128.1, 122.4, 80.4, 47.3, 46.9, 45.0, 39.3, 25.9, 24.4, 21.2, 20.0, 16.6; HRMS (ESI) calcd for $C_{22}H_{27}NNaO_3$ $[M+Na]^+$ 376.1883, found 376.1877; CHIRALPAK OD-H, *n*-hexane/*i*-PrOH = 90:10, 1.0 mL/min, retention times: 20.1 min (major isomer) and 18.0 min (minor isomer).

2.4.2. PKR (type II) Using Two Different Acrylamide Derivatives

General Procedure for Rh-Catalyzed Enantioselective PKR (type II) (Figure 3b): (*R*)-P-Phos (7.7 mg, 0.012 mmol) and [Rh(cod)$_2$]BF$_4$ (4.1 mg, 0.010 mmol) were dissolved in CH_2Cl_2 (2.0 mL) in a Schlenk tube, and the mixture was stirred at room temperature for 10 min. After introduction of H_2 and stirring at room temperature for 30 min, the resulting mixture was concentrated to dryness. The residue was dissolved in CH_2Cl_2 (1.0 mL), followed by the addition of a solution of **1** (0.100 mmol), α-unsubstituted acrylamide **2** (0.0500 mmol) and α-substituted acrylamide **2** (0.0700 mmol) in CH_2Cl_2 (1.0 mL) was added, and the mixture was stirred at room temperature for 16 h, then passed through short-path silica gel column chromatography to remove the Rh complex and concentrated. The crude product was further purified by silica gel PTLC twice (eluent: *n*-hexane/EtOAc = 1:1 and CH_2Cl_2/EtOAc = 20:1) to furnish **3** and **4**.

Reaction using 1h, 2g and 2a: 3hg [16.2 mg, 0.0431 mmol, 43% yield, >99:1 d.r., 91:9 e.r. (major)] and **4ha** (10.7 mg, 0.0299 mmol, 30% yield, >99:1 d.r., 96:4 e.r.) were obtained from **1h** (21.4 mg, 0.100 mmol), **2g** (8.1 mg, 0.0500 mmol) and **2a** (10.0 mg, 0.0700 mmol). A trace amount (ca. <2%) of another diastereomer of **3hg** was detected in a crude reaction mixture.

(–)-(1*S*,5*S*,7a*R*)-*N*,1,7a-Trimethyl-3-oxo-*N*,4-diphenyl-1,3,5,6,7,7a-hexahydroisobenzofuran-5-carboxamide [(–)-3hg (major diastereomer)]

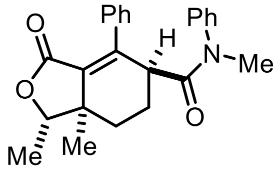

CHIRALPAK OD-H, *n*-hexane/*i*-PrOH = 90:10, 1.0 mL/min, retention times: 12.6 min (major isomer) and 16.9 min (minor isomer).

(–)-(4*S*,5*R*)-3-(*Z*)-Benzylidene-4-{(*E*)-3-fluoro-4-oxo-4-(pyrrolidin-1-yl)but-2-en-1-yl}-4,5-dimethyldihydrofuran-2(3*H*)-one [(–)-4ha]

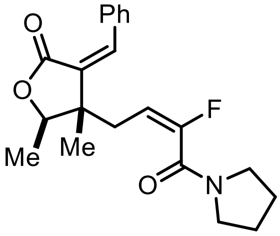

CHIRALPAK IG-3, *n*-hexane/*i*-PrOH = 90:10, 1.0 mL/min, retention times: 61.8 min (major isomer) and 53.7 min (minor isomer).

Reaction using 1i, 2g and 2a: 3ig [19.0 mg, 0.0418 mmol, 42% yield, >99:1 d.r., 91:9 e.r. (major)] and **4ia** (13.8 mg, 0.0317 mmol, 32% yield, >99:1 d.r., 95:5 e.r.) were obtained from **1i** (29.3 mg, 0.100 mmol), **2g** (8.1 mg, 0.0500 mmol) and **2a** (10.0 mg, 0.0700 mmol), purified by silica gel PTLC twice (eluent: *n*-hexane/EtOAc = 1:1 and *n*-hexane/Et_2O = 4:1). A trace amount (ca. <2%) of another diastereomer of **3ig** was detected in a crude reaction mixture.

(–)-(1*S*,5*S*,7a*R*)-4-(4-Bromophenyl)-*N*,1,7a-trimethyl-3-oxo-*N*-phenyl-1,3,5,6,7,7a-hexahydroisobenzofuran-5-carboxamide [(–)-3ig (major diastereomer)]

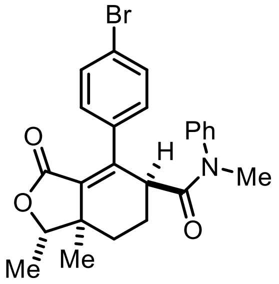

White solid; mp 180.4–182.2 °C; $[\alpha]^{25}_{D}$ –108.3 (*c* 0.95, $CHCl_3$, 91:9 e.r.); ^1H NMR (400 MHz, $CDCl_3$) δ 7.48–7.45 (m, 2H), 7.38–7.33 (m, 3H), 6.90–6.87 (m, 2H), 6.72 (br s, 2H), 4.31 (q, J = 6.5 Hz, 1H), 3.46 (d, J = 6.4 Hz, 1H), 3.07 (s, 3H), 2.06 (td, J = 13.2, 3.6 Hz, 1H), 1.98–1.92 (m, 1H), 1.83 (tdd, J = 14.1, 7.0, 3.4 Hz, 1H), 1.61 (dt, J = 12.2, 3.3 Hz, 1H), 1.36 (d, J = 6.5 Hz, 3H), 1.01 (s, 3H); ^{13}C NMR (101 MHz, $CDCl_3$) δ 171.6, 168.5, 143.5, 143.3, 136.3, 134.3, 131.1, 129.9, 129.7, 128.2, 127.3, 122.2, 83.4, 42.50, 42.46, 37.3, 27.4, 23.4, 18.5, 13.8; HRMS (ESI) calcd for $C_{24}H_{24}BrNNaO_3$ $[M+Na]^+$ 476.0832, found 476.0821; CHIRALPAK OD-H, *n*-hexane/*i*-PrOH = 95:5, 1.0 mL/min, retention times: 18.2 min (major isomer) and 27.4 min (minor isomer).

(–)-(4*S*,5*R*)-3-(*Z*)-4-Bromobenzylidene-4-{(*E*)-3-fluoro-4-oxo-4-(pyrrolidin-1-yl)but-2-en-1-yl}-4,5-dimethyldihydrofuran-2(3*H*)-one [(–)-4ia]

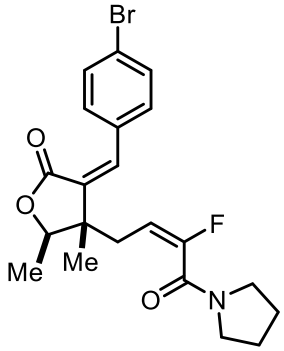

CHIRALPAK IG-3, *n*-hexane/*i*-PrOH = 90:10, 1.0 mL/min, retention times: 64.8 min (major isomer) and 56.9 min (minor isomer).

Reaction using 1j, 2g and 2a: 3jg (20.8 mg, 0.0475 mmol, 48% yield, >99:1 d.r., 88:12 e.r.) and **4ja** (17.8 mg, 0.0424 mmol, 42% yield, >99:1 d.r., 97:3 e.r.) were obtained from **1j** (27.6 mg, 0.100 mmol), **2g** (8.1 mg, 0.0500 mmol) and **2a** (10.0 mg, 0.0700 mmol). T

(–)-(1*S*,5*S*,7a*R*)-*N*,7a-Dimethyl-3-oxo-*N*,1,4-triphenyl-1,3,5,6,7,7a-hexahydroisobenzofuran-5-carboxamide [(–)-3jg]

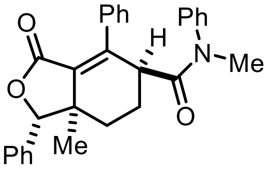

Colorless oil; $[\alpha]^{25}_{D}$ –43.3 (*c* 1.04, $CHCl_3$, 88:12 e.r.); ^1H NMR (400 MHz, $CDCl_3$) δ 7.38–7.30 (m, 11H), 7.05–7.03 (m, 2H), 6.65 (br s, 2H), 5.31 (s, 1H), 3.51 (d, J = 6.6 Hz, 1H), 3.08 (s, 3H), 2.46 (td, J = 13.1, 3.6 Hz, 1H), 1.98 (dt, J = 14.3, 3.2 Hz, 1H), 1.79 (tdd, J = 14.0, 7.0, 3.4 Hz, 1H), 1.71 (dt, J = 12.3, 3.3 Hz, 1H), 0.72 (s, 3H); ^{13}C NMR (101 MHz, $CDCl_3$) δ 171.9, 168.2, 145.6, 143.5, 137.3, 135.5, 133.3, 129.7, 128.3, 128.05, 128.04, 127.97, 127.94, 127.86, 127.4, 125.6, 87.6, 44.2, 42.8, 37.2, 28.0, 23.3, 19.8; HRMS (ESI) calcd for $C_{29}H_{27}NNaO_3$ $[M+Na]^+$ 460.1883, found 460.1871; CHIRALPAK ID-3, *n*-hexane/*i*-PrOH = 70:30, 1.0 mL/min, retention times: 19.0 min

(major isomer) and 75.2 min (minor isomer).

(–)-(4*S*,5*R*)-3-(*Z*)-Benzylidene-4-{(*E*)-3-fluoro-4-oxo-4-(pyrrolidin-1-yl)but-2-en-1-yl}-4-methyl-5-phenyldihydrofuran-2(3*H*)-one [(–)-4ja]

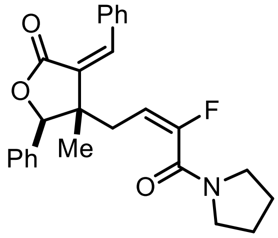

CHIRALPAK IG-3, *n*-hexane/*i*-PrOH = 80:20, 1.0 mL/min, retention times: 49.9 min (major isomer) and 43.4 min (minor isomer).

Reaction using 1k, 2g and 2a: 3kg (20.0 mg, 0.0446 mmol, 45% yield, >99:1 d.r., 99:1 e.r.) and **4ka** (20.5 mg, 0.0476 mmol, 48% yield, >99:1 d.r., 96:4 e.r.) were obtained from **1k** (28.7 mg, 0.100 mmol), **2g** (8.1 mg, 0.0500 mmol) and **2a** (10.0 mg, 0.0700 mmol).

(–)-(8*S*,10a*R*,10b*S*)-*N*,10a-Dimethyl-6-oxo-*N*,7-diphenyl-8,9,10,10a,10b,11-hexahydro-6*H*-isoindolo[2,1-a]indole-8-carboxamide [(–)-3kg]

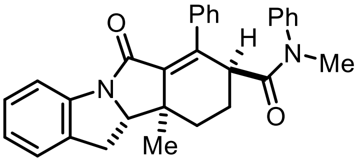

White solid; mp 165.9–167.5 °C; $[\alpha]^{25}_{D}$ –67.0 (*c* 1.00, $CHCl_3$, 99:1 e.r.); ^1H NMR (400 MHz, $CDCl_3$) δ 7.60 (d, J = 7.8 Hz, 1H), 7.36–7.29 (m, 6H), 7.18–7.11 (m, 2H), 7.08–7.03 (m, 2H), 6.97 (td, J = 7.5, 1.0 Hz, 1H), 6.62 (br s, 2H), 4.53 (t, J = 9.3 Hz, 1H), 3.45 (d, J = 6.6 Hz, 1H), 3.16 (dd, J = 16.3, 9.1 Hz, 1H), 3.07 (s, 3H), 3.02 (dd, J = 16.3, 9.7 Hz, 1H), 2.39 (td, J = 13.1, 3.8 Hz, 1H), 1.95 (dt, J = 14.3, 3.2 Hz, 1H), 1.84 (tdd, J = 14.0, 7.0, 3.5 Hz, 1H), 1.75 (dt, J = 12.4, 3.3 Hz, 1H), 1.07 (s, 3H); ^{13}C NMR (101 MHz, $CDCl_3$) δ 172.5, 163.9, 143.7, 141.0, 140.6, 140.0, 138.3, 133.0, 129.6, 128.2, 127.9, 127.8, 127.6, 127.54, 127.47, 125.2, 123.6, 114.3, 69.8, 43.0, 42.2, 37.2, 29.3, 27.9, 23.4, 20.7; HRMS (ESI) calcd for $C_{30}H_{28}N_2NaO_2$ $[M+Na]^+$ 471.2043, found 471.2030; CHIRALPAK OD-H, *n*-hexane/*i*-PrOH = 90:10, 1.0 mL/min, retention times: 13.4 min (major isomer) and 41.2 min (minor isomer).

(–)-(1*S*,9a*R*)-2-(*Z*)-Benzylidene-1-{(*E*)-3-fluoro-4-oxo-4-(pyrrolidin-1-yl)but-2-en-1-yl}-1-methyl-1,2,9,9a-tetrahydro-3*H*-pyrrolo[1,2-a]indol-3-one [(–)-4ka]

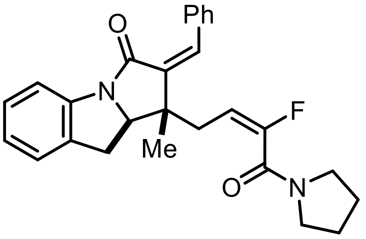

CHIRALPAK IF-3, *n*-hexane/*i*-PrOH = 70:30, 1.0 mL/min, retention times: 33.4 min (major isomer) and 12.3 min (minor isomer).

Reaction using 1l, 2g and 2a: 3lg (13.5 mg, 0.0373 mmol, 37% yield, >99:1 d.r., 81:19 e.r.) and **4la** (10.9 mg, 0.0317 mmol, 32% yield, >99:1 d.r., 84:16 e.r.) were obtained from **1l** (20.0 mg, 0.100 mmol), **2g** (8.1 mg, 0.0500 mmol) and **2a** (10.0 mg, 0.0700 mmol), using (*R*)-BINAP (7.5 mg, 0.0120 mmol), purified by silica gel PTLC twice (eluent: *n*-hexane/EtOAc = 3:1 and CH_2Cl_2/EtOAc = 20:1).

(–)-(1*S*,5*S*,7a*R*)-*N*,1,7a-Trimethyl-*N*,4-diphenyl-1,3,5,6,7,7a-hexahydroisobenzofuran-5-carboxamide [(–)-3lg]

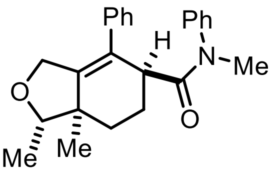

White solid; mp 54.9–56.3 °C; $[\alpha]^{25}_D$ –40.9 (*c* 0.68, $CHCl_3$, 81:19 e.r.); ^{1}H NMR (400 MHz, $CDCl_3$) δ 7.33–7.23 (m, 6H), 7.01–6.99 (m, 2H), 6.60 (br s, 2H), 4.24 (dd, *J* = 13.3, 2.8 Hz, 1H), 4.12 (dd, *J* = 13.3, 2.1 Hz, 1H), 3.66 (q, *J* = 6.3 Hz, 1H), 3.24–3.23 (m, 1H), 3.13 (s, 3H), 1.99–1.76 (m, 3H), 1.46 (dt, *J* = 12.1, 3.2 Hz, 1H), 1.19 (d, *J* = 6.3 Hz, 3H), 0.92 (s, 3H); ^{13}C NMR (101 MHz, $CDCl_3$) δ 174.2, 144.9, 144.1, 140.7, 129.5, 128.3, 128.2, 127.6, 127.4, 127.2, 126.9, 83.8, 67.5, 42.2, 41.0, 37.3, 27.7, 23.7, 17.6, 14.2; HRMS (ESI) calcd for $C_{24}H_{27}NNaO_2$ $[M+Na]^+$ 384.1934, found 384.1934; CHIRALPAK OD-H, *n*-hexane/*i*-PrOH = 95:5, 1.0 mL/min, retention times: 7.2 min (major isomer) and 31.1 min (minor isomer).

(+)-(*E*)-4-{(2*R*,3*S*)-4-(*Z*)-Benzylidene-2,3-dimethyltetrahydrofuran-3-yl}-2-fluoro-1-(pyrrolidin-1-yl)but-2-en-1-one [(+)-4la]

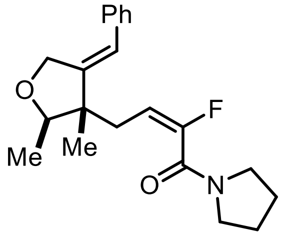

CHIRALPAK IG-3, *n*-hexane/*i*-PrOH = 90:10, 1.0 mL/min, retention times: 40.2 min (major isomer) and 29.4 min (minor isomer).

Reaction using 1h, 2h and 2a: 3hh (13.4 mg, 0.0392 mmol, 39% yield, >99:1 d.r., 89:11 e.r.) and **4ha** (7.5 mg, 0.0210 mmol, 21% yield, >99:1 d.r., 92:8 e.r.) were obtained from **1h** (21.4 mg, 0.100 mmol), **2h** (6.4 mg, 0.0500 mmol) and **2a** (26.9 mg, 0.150 mmol), using (*R*)-BINAP (7.5 mg, 0.0120 mmol) at 40 °C.

(–)-(1*S*,5*S*,7a*R*)-*N*,*N*-Diethyl-1,7a-dimethyl-3-oxo-4-phenyl-1,3,5,6,7,7a-hexahydroisobenzofuran-5-carboxamide [(–)-3hh]

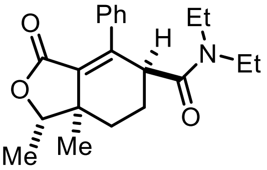

White solid; mp 123.0–125.4 °C; $[\alpha]^{25}_D$ –78.4 (*c* 0.67, $CHCl_3$, 89:11 e.r.); ^{1}H NMR (400 MHz, $CDCl_3$) δ 7.31–7.26 (m, 3H), 7.15–7.13 (m, 2H), 4.31 (q, *J* = 6.5 Hz, 1H), 3.73–3.72 (m, 1H), 3.37–3.29 (m, 1H), 3.15–3.00 (m, 3H), 2.15–2.04 (m, 2H), 1.94–1.85 (m, 1H), 1.64–1.61 (m, 1H), 1.37 (d, *J* = 6.5 Hz, 3H), 1.12 (s, 3H), 0.95 (t, *J* = 7.2 Hz, 3H), 0.87 (t, *J* = 7.1 Hz, 3H); ^{13}C NMR (101 MHz, $CDCl_3$) δ 171.2, 168.7, 144.9, 137.5, 133.7, 127.9 (2C), 127.8, 83.3, 42.4, 42.3, 41.9, 40.7, 27.5, 23.6, 18.8, 14.6, 13.8, 12.6; HRMS (ESI) calcd for $C_{21}H_{27}NNaO_3$ $[M+Na]^+$ 364.1883, found 364.1901; CHIRALPAK IE-3, *n*-hexane/*i*-PrOH = 80:20, 1.0 mL/min, retention times: 48.4 min (major isomer) and 76.0 min (minor isomer).

(–)-(4*S*,5*R*)-3-(*Z*)-Benzylidene-4-{(*E*)-3-fluoro-4-oxo-4-(pyrrolidin-1-yl)but-2-en-1-yl}-4,5-dimethyldihydrofuran-2(3*H*)-one [(–)-4ha]

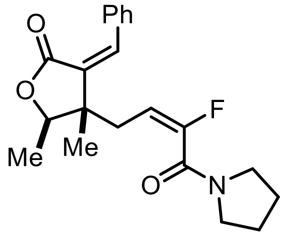

CHIRALPAK IG-3, *n*-hexane/*i*-PrOH = 90:10, 1.0 mL/min, retention times: 62.3 min (major isomer) and 53.9 min (minor isomer).

Reaction using 1h, 2g and 2i: 3hg [16.4 mg, 0.0437 mmol, 44% yield, >99:1 d.r., 90:10 e.r. (major)] and **4hi** (9.0 mg, 0.0229 mmol, 23% yield, >99:1 d.r., 90:10 e.r.) were obtained from **1h** (21.4 mg, 0.100 mmol), **2g** (8.1 mg, 0.0500 mmol) and **2i** (26.9 mg, 0.150 mmol). A trace amount (ca. <2%) of another diastereomer of **3hg** was detected in a crude reaction mixture.

(–)-(1*S*,5*S*,7a*R*)-*N*,1,7a-Trimethyl-3-oxo-*N*,4-diphenyl-1,3,5,6,7,7a-hexahydroisobenzofuran-5-carboxamide [(–)-3hg (major diastereomer)]

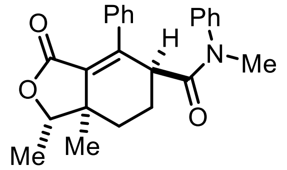

CHIRALPAK OD-H, *n*-hexane/*i*-PrOH = 90:10, 1.0 mL/min, retention times: 12.6 min (major isomer) and 16.9 min (minor isomer).

(+)-(*E*)-4-{(2*R*,3*S*)-4-(*Z*)-Benzylidene-2,3-dimethyl-5-oxotetrahydrofuran-3-yl}-2-fluoro-*N*-methyl-*N*-phenylbut-2-enamide [(+)-4hi]

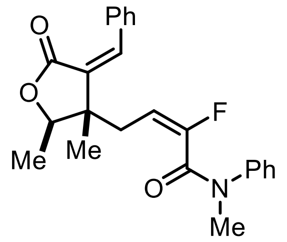

Colorless oil; $[\alpha]^{25}_{D}$ 11.4 (*c* 0.82, $CHCl_3$, 90:10 e.r.); ^1H NMR (400 MHz, $CDCl_3$) δ 7.86–7.83 (m, 2H), 7.40–7.29 (m, 6H), 7.14–7.12 (m, 2H), 6.73 (s, 1H), 5.36–5.31 (br m, 1H), 4.46 (q, J = 6.5 Hz, 1H), 3.33 (d, J = 1.1 Hz, 3H), 2.65–2.54 (m, 2H), 1.32 (d, J = 6.5 Hz, 3H), 1.23 (s, 3H); ^{13}C NMR (101 MHz, $CDCl_3$) δ 168.4, 161.6 (d, J = 30.4 Hz), 152.4 (d, J = 264.0 Hz), 142.6, 139.3, 133.3, 132.5, 130.8, 129.7, 129.4, 128.1, 127.7, 125.8 (d, J = 1.6 Hz), 110.7 (d, J = 18.3 Hz), 80.1, 47.3 (d, J = 1.9 Hz), 37.7, 34.9 (d, J = 5.2 Hz), 21.2, 16.6; ^{19}F NMR (377 MHz, $CDCl_3$) δ –106.3; HRMS (ESI) calcd for $C_{24}H_{24}FNNaO_3$ $[M+Na]^+$ 416.1632, found 416.1632; CHIRALPAK IE-3, *n*-hexane/*i*-PrOH = 80:20, 1.0 mL/min, retention times: 24.2 min (major isomer) and 26.9 min (minor isomer).

Reaction using 1h, 2g and 2e: 3hg (17.8 mg, 0.0474 mmol, 47% yield, >99:1 d.r., 93:7 e.r.) and **4he** (9.2 mg, 0.0260 mmol, 26% yield, >99:1 d.r., 82:18 e.r.) were obtained from **1h** (21.4 mg, 0.100 mmol), 2**g** (11.3 mg, 0.0700 mmol) and **2e** (27.8 mg, 0.200 mmol), using (*R*)-BINAP (7.5 mg, 0.0120 mmol), purified by silica gel PTLC twice (eluent: *n*-hexane/EtOAc = 1:1 and CH_2Cl_2/EtOAc = 10:1).

(–)-(1*S*,5*S*,7a*R*)-*N*,1,7a-Trimethyl-3-oxo-*N*,4-diphenyl-1,3,5,6,7,7a-hexahydroisobenzofuran-5-carboxamide [(–)-3hg (major diastereomer)]

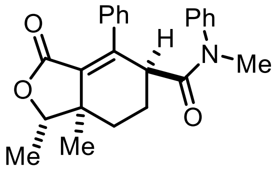

CHIRALPAK OD-H, *n*-hexane/*i*-PrOH = 90:10, 1.0 mL/min, retention times: 12.8 min (major isomer) and 17.0 min (minor isomer).

(+)-(4*S*,5*R*)-3-{(*Z*)-Benzylidene}-4,5-dimethyl-4-[(*Z*)-3-methyl-4-oxo-4-{pyrrolidin-1-yl}but-2-en-1-yl]dihydrofuran-2(3*H*)-one [(+)-4he]

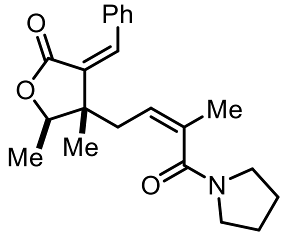

$[\alpha]^{25}_{D}$ +8.8 (*c* 0.71, $CHCl_3$, 82:18 e.r.); CHIRALPAK OD-H, *n*-hexane/*i*-PrOH = 90:10, 1.0 mL/min, retention times: 19.8 min (major isomer) and 17.9 min (minor isomer).

Reaction using 1j, 2g and 2e: 3jg (17.0 mg, 0.0388 mmol, 39% yield, >99:1 d.r., >99:1 e.r) and **4je** (11.4 mg, 0.0274 mmol, 27% yield, >99:1 d.r., 75:25 e.r.) were obtained from **1j** (27.6 mg, 0.100 mmol), **2g** (8.1 mg, 0.0700 mmol) and **2e** (27.8 mg, 0.200 mmol), using (*R*)-BINAP (7.5 mg, 0.0120 mmol), purified by silica gel PTLC twice (eluent: *n*-hexane/EtOAc = 1:1 and CH_2Cl_2/EtOAc = 10:1).

(–)-(1*S*,5*S*,7a*R*)-*N*,7a-Dimethyl-3-oxo-*N*,1,4-triphenyl-1,3,5,6,7,7a-hexahydroisobenzofuran-5-carboxamide [(–)-3jg]

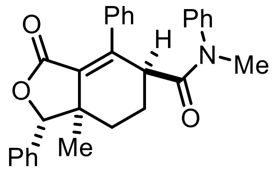

CHIRALPAK ID-3, *n*-hexane/*i*-PrOH = 70:30, 1.0 mL/min, retention times: 20.4 min (major isomer) and 74.0 min (minor isomer).

(–)-(4*S*,5*R*)-3-{(*Z*)-Benzylidene}-4-methyl-4-[(*Z*)-3-methyl-4-oxo-4-{pyrrolidin-1-yl}but-2-en-1-yl]-5-phenyldihydrofuran-2(3H)-one [(–)-4je]

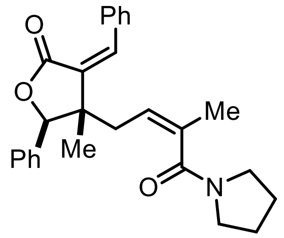

White solid; mp 146.5–147.8 °C; $[\alpha]^{25}_{D}$ –6.0 (*c* 0.60, $CHCl_3$, 75:25 e.r.); ^{1}H NMR (400 MHz, $CDCl_3$) δ 7.94–7.91 (m, 2H), 7.44–7.31 (m, 6H), 7.25–7.23 (m, 2H), 6.73 (s, 1H), 5.46–5.41 (m, 1H), 5.35 (s, 1H), 3.56–3.53 (m, 2H), 3.34–3.31 (m, 2H), 2.44–2.42 (m, 2H), 1.95–1.83 (m, 7H), 0.84 (s, 3H); ^{13}C NMR (101 MHz, $CDCl_3$) δ 169.9, 169.0, 139.8, 137.8, 136.7, 133.5, 132.1, 130.9, 129.7, 128.3 (2C), 128.2, 126.4, 122.4, 85.2, 48.7, 47.0, 45.1, 40.4, 25.9, 24.4, 23.6, 20.2; HRMS (ESI) calcd for $C_{27}H_{29}NNaO_3$ $[M+Na]^+$ 438.2040, found 438.2025; CHIRALPAK ID-3, *n*-hexane/*i*-

PrOH = 70:30, 1.0 mL/min, retention times: 76.8 min (major isomer) and 28.5 min (minor isomer).

2.5. Rh-Catalyzed Enantioselective Desymmetrization of Achiral Dienynes with Acrylamide Derivatives

General Procedure for Rh-Catalyzed Enantioselective Desymmetrization Using 1,6-Enynes 5a–5g (Figure 4): (*R*)-BINAP (3.7 mg, 0.0060 mmol) and [Rh(cod)$_2$]BF$_4$ (2.1 mg, 0.0050 mmol) were dissolved in CH_2Cl_2 (2.0 mL) in a Schlenk tube, and the mixture was stirred at room temperature for 10 min. After the introduction of H_2 and stirring at room temperature for 30 min, the resulting mixture was concentrated to dryness. The residue was dissolved in CH_2Cl_2 (0.5 mL), followed by the addition of a solution of **5** (0.0500 mmol) and **2** (0.0600 mmol) in CH_2Cl_2 (0.5 mL). The mixture was stirred at room temperature for 16 h, then passed through short-path silica gel column chromatography to remove the Rh complex and concentrated. The crude product was further purified by silica gel PTLC twice (eluent: *n*-hexane/EtOAc = 1:1 and CH_2Cl_2/EtOAc = 10:1) to furnish **6**.

(–)-{(1*R*,5*R*,7a*R*)-5-Fluoro-2-(4-methylphenyl)-4-phenyl-1-vinyl-2,3,5,6,7,7a-hexahydro-1*H*-isoindol-5-yl}(pyrrolidin-1-yl)methanone [(–)-6aa]

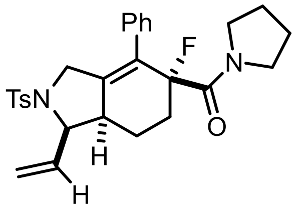

6aa [37.5 mg, 0.0867 mmol, 87% yield, 94:6 d.r., >99:1 e.r. (major)] was prepared from **5a** (35.2 mg, 0.100 mmol) and **2a** (17.2 mg, 0.120 mmol). Two diastereomers of **6aa** were obtained as a mixture. The absolute configuration was determined by X-ray crystallographic analysis (Figure S6).

The following experimental data were acquired using a mixture of isomers [94:6 d.r., >99:1 e.r. (major)]. White solid; mp 103.9–105.1 °C; $[\alpha]^{25}_{D}$ –2.3 (*c* 3.75, $CHCl_3$); **major diastereomer:** ^{1}H NMR (400 MHz, $CDCl_3$) δ 7.63 (d, *J* = 8.2 Hz, 2H), 7.30–7.28 (m, 3H), 7.24 (d, *J* = 7.9 Hz, 2H), 7.07–7.05 (m, 2H), 5.57 (ddd, *J* = 16.9, 10.0, 8.8 Hz, 1H), 5.26–5.21 (m, 1H), 5.17–5.14 (m, 1H), 4.55 (t, *J* = 8.4 Hz, 1H), 3.99 (ddd, *J* = 15.2, 7.3, 1.7 Hz, 1H), 3.58 (ddd, *J* = 15.2, 5.3, 1.7 Hz, 1H), 3.48–3.34 (m, 3H), 2.98–2.91 (m, 1H), 2.83–2.75 (m, 1H), 2.41 (s, 3H), 2.37 (dt, *J* = 12.8, 3.4 Hz, 1H), 2.17–1.88 (m, 2H), 1.78–1.49 (m, 5H); **partial protons of minor diastereomer:** ^{1}H NMR (400 MHz, $CDCl_3$) δ 7.72 (d, *J* = 8.4 Hz, 2H), 5.70 (ddd, *J* = 17.1, 10.1, 7.1 Hz, 1H), 4.62 (t, *J* = 8.4 Hz, 1H); ^{13}C NMR (101 MHz, $CDCl_3$) δ 169.1 (d, *J* = 27.0 Hz), 143.2, 141.7 (d, *J* = 6.4 Hz), 136.3, 136.1, 133.6, 132.1 (d, *J* = 20.9 Hz), 129.4, 129.0, 128.3, 127.8, 127.5, 118.4, 96.4 (d, *J* = 186.3 Hz), 65.7, 49.0, 47.8, 46.9 (d, *J* = 16.9 Hz), 43.1 (d, *J* = 2.5 Hz), 33.9 (d, *J* = 23.1 Hz), 26.6 (d, *J* = 5.4 Hz), 22.9, 21.9 (d, *J* = 7.4 Hz), 21.5; ^{19}F NMR (377 MHz, $CDCl_3$) δ –145.6 (major), –143.7 (minor); HRMS (ESI) calcd for $C_{28}H_{31}FN_2NaO_3S$ $[M+Na]^+$ 517.1932, found 517.1938; CHIRALPAK AD-H, *n*-hexane/*i*-PrOH = 95:5, 1.0 mL/min, retention times: 68.8 min (major isomer) and 64.3 min (minor isomer).

(–)-{(1*R*,5*R*,7a*R*)-5-Fluoro-2-(4-methylphenyl)-4-phenyl-1-vinyl-2,3,5,6,7,7a-hexahydro-1*H*-isoindol-5-yl}(morpholino)methanone [(–)-6ab]

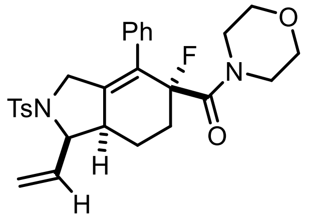

6ab [16.9 mg, 0.0331 mmol, 66% yield, 93:7 d.r., >99:1 e.r. (major)] was prepared from **5a** (17.6 mg, 0.0500 mmol) and **2b** (8.6 mg, 0.0600 mmol). Two diastereomers of **6ab** were obtained as a mixture.

The following experimental data were acquired using a mixture of isomers [93:7 d.r., >99:1 e.r. (major)]. Colorless oil; $[\alpha]^{25}_{D}$ –3.9 (*c* 1.69, $CHCl_3$); **major diastereomer:** ^{1}H NMR (400 MHz, $CDCl_3$) δ 7.64 (d, *J* = 8.3 Hz, 2H), 7.29–7.25 (m, 3H), 7.26–7.23 (m, 2H), 7.10–7.08 (m, 2H), 5.55 (ddd, *J* = 16.9, 10.0, 8.7 Hz, 1H), 5.27–5.22 (m, 1H), 5.19–5.16 (m, 1H), 4.55 (t, *J* = 8.3 Hz, 1H), 3.97 (ddd, *J* = 15.3, 7.4, 1.7 Hz, 1H), 3.65–3.58 (m, 3H), 3.51–3.40 (m, 5H), 3.12–3.06 (m, 1H), 2.83–2.76 (m, 1H), 2.41 (s, 3H), 2.40–2.35 (m, 1H), 2.08–1.86 (m, 2H), 1.81–1.75 (m, 1H); **partial protons of minor diastereomer:** ^{1}H NMR (400 MHz, $CDCl_3$) δ 7.74 (d, *J* = 8.4 Hz, 2H), 7.55–7.53 (m, 3H), 7.00–6.97 (m, 2H), 6.00 (ddd, *J* = 17.1, 10.1, 8.4 Hz, 1H); ^{13}C NMR (101 MHz, $CDCl_3$) δ 169.0 (d, *J* = 24.3 Hz), 143.3, 141.8 (d, *J* = 6.5 Hz), 136.2, 135.9 (d, *J* = 0.7 Hz), 133.5, 132.0 (d, *J* = 21.0 Hz), 129.5, 129.3, 128.5, 128.0, 127.5, 118.6, 97.7 (d, *J* = 186.9 Hz), 66.9, 66.6, 65.6, 49.0, 46.8 (d, *J* = 19.9 Hz), 43.9, 43.2 (d, *J* = 2.5 Hz), 34.2 (d, *J* = 22.7 Hz), 21.6 (d, *J* = 7.0 Hz), 21.5; ^{19}F NMR (377 MHz, $CDCl_3$) δ –141.4 (major), –139.3 (minor); HRMS (ESI) calcd for $C_{28}H_{31}FN_2NaO_4S$ $[M+Na]^+$ 533.1881, found 533.1897; CHIRALPAK AD-H, *n*-hexane/*i*-PrOH = 90:10, 1.0 mL/min, retention times: 29.5 min (major isomer) and 43.9 min (minor isomer).

(–)-(1*R*,5*R*,7a*R*)-5-Fluoro-*N*-methyl-2-(4-methylphenyl)-*N*,4-diphenyl-1-vinyl-2,3,5,6,7,7a-hexahydro-1*H*-isoindole-5-carboxamide [(–)-6ai]

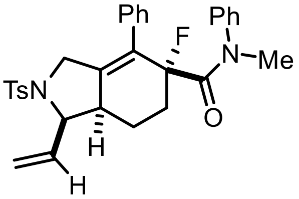

6ai (14.7 mg, 0.0277 mmol, 55% yield, >99:1 d.r., >99:1 e.r.) was prepared from **5a** (17.6 mg, 0.0500 mmol) and **2i** (10.8 mg, 0.0600 mmol).

Colorless oil; $[\alpha]^{25}_{D}$ –7.8 (*c* 1.47, $CHCl_3$, >99:1 e.r.); ^{1}H NMR (400 MHz, $CDCl_3$) δ 7.63 (d, *J* = 8.3 Hz, 2H), 7.36–7.33 (m, 3H), 7.23 (d, *J* = 7.9 Hz, 2H), 7.17 (br s, 3H), 7.03 (br s, 2H), 6.53 (br s, 2H), 5.67–5.58 (m, 1H), 5.28–5.19 (m, 2H), 4.55 (t, *J* = 8.4 Hz, 1H), 3.93 (dd, *J* = 15.4, 6.8 Hz, 1H), 3.63 (ddd, *J* = 15.2, 5.2, 1.6 Hz, 1H), 3.12 (d, *J* = 1.1 Hz, 3H), 2.74 (br s, 1H), 2.40 (s, 3H), 2.40–2.35 (m, 1H), 2.13–2.04 (m, 1H), 1.82–1.71 (m, 2H); ^{13}C NMR (101 MHz, $CDCl_3$) δ 170.2 (d, *J* = 24.3 Hz), 144.0, 143.2, 141.2, 136.3, 136.1, 133.6, 132.1 (d, *J* = 20.7 Hz), 129.4, 129.3, 128.7, 128.4, 127.8, 127.5, 127.1, 126.4 (d, *J* = 3.7 Hz), 118.4, 96.5 (d, *J* = 194.0 Hz), 65.8, 48.9, 43.0 (d, *J* = 2.2 Hz), 40.5, 34.8 (d, *J* = 21.5 Hz), 21.8 (d, *J* = 6.7 Hz), 21.5; ^{19}F NMR (377 MHz, $CDCl_3$) δ –138.4 (major), –136.8 (minor); HRMS (ESI) calcd for $C_{31}H_{31}FN_2NaO_3S$ $[M+Na]^+$ 553.1932, found 553.1955; CHIRALPAK AD-H, *n*-hexane/*i*-PrOH = 85:15, 1.0 mL/min, retention times: 10.2 min (major isomer) and 14.6 min (minor isomer).

(+)-(1*R*,5*R*,7a*R*)-5-Fluoro-2-(4-methylphenyl)-*N*,*N*,4-triphenyl-1-vinyl-2,3,5,6,7,7a-hexahydro-1*H*-isoindole-5-carboxamide [(+)-6ac]

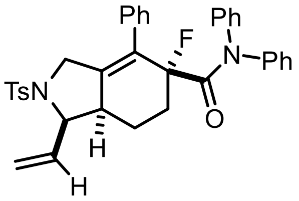

6ac [23.7 mg, 0.0400 mmol, 80% yield, 95:5 d.r., >99:1 e.r. (major)] was prepared from **5a** (17.6 mg, 0.0500 mmol) and **2c** (14.5 mg, 0.0600 mmol). Two diastereomers of **6ac** were obtained as a mixture.

The following experimental data were acquired using a mixture of isomers [95:5 d.r., >99:1 e.r. (major)]. White solid; mp 97.6–98.9 °C; $[\alpha]^{25}_{D}$ +8.1 (*c* 2.37, $CHCl_3$); **major diastereomer:** ^{1}H NMR (400 MHz, $CDCl_3$) δ 7.62 (d, *J* = 8.3 Hz, 2H), 7.44–7.40 (m, 3H), 7.24–7.12 (m, 10H), 6.81 (br s, 4H), 5.58 (ddd, *J* = 16.9, 10.0, 8.8 Hz, 1H), 5.26–5.21 (m, 1H), 5.12–5.10 (m, 1H), 4.55 (t, *J* =

8.4 Hz, 1H), 3.89 (ddd, J = 15.3, 7.4, 1.6 Hz, 1H), 3.72 (ddd, J = 15.3, 5.4, 1.7 Hz, 1H), 2.78–2.71 (m, 1H), 2.55 (dt, J = 13.0, 3.3 Hz, 1H), 2.40 (s, 3H), 2.14–2.04 (m, 1H), 1.98–1.85 (m, 1H), 1.79–1.73 (m, 1H); **partial protons of minor diastereomer:** ^{1}H NMR (400 MHz, $CDCl_3$) δ 7.54 (d, J = 8.2 Hz, 2H), 7.29 (d, J = 7.9 Hz, 2H), 7.01–6.99 (m, 2H), 6.00 (ddd, J = 17.1, 10.2, 8.4 Hz, 1H), 3.96 (ddd, J = 15.8, 7.5, 1.6 Hz, 1H), 3.58 (ddd, J = 15.8, 6.2, 1.9 Hz, 1H), 3.12 (d, J = 1.0 Hz, 1H), 3.03 (dd, J = 10.0, 8.5 Hz, 1H), 2.45 (s, 3H); ^{13}C NMR (101 MHz, $CDCl_3$) δ 170.9 (d, J = 25.0 Hz), 143.3, 141.7 (d, J = 6.3 Hz), 136.2, 136.0, 133.6, 131.9 (d, J = 20.0 Hz), 129.8, 129.4, 129.0, 128.5, 128.1, 127.8, 127.5, 127.2, 127.0, 118.5, 96.5 (d, J = 193.4 Hz), 65.9, 48.8, 43.0 (d, J = 2.4 Hz), 35.1 (d, J = 23.0 Hz), 21.9 (d, J = 6.5 Hz), 21.5; ^{19}F NMR (377 MHz, $CDCl_3$) δ –138.1 (major), –137.6 (minor); HRMS (ESI) calcd for $C_{36}H_{33}FN_2NaO_3S$ [M+Na]$^+$ 615.2088, found 615.2082; CHIRALPAK AD-H, *n*-hexane/*i*-PrOH = 90:10, 1.0 mL/min, retention times: 15.5 min (major isomer) and 13.1 min (minor isomer).

(+)-(1*R*,5*R*,7a*R*)-5-Fluoro-*N*-methoxy-*N*-methyl-2-(4-methylphenyl)-4-phenyl-1-vinyl-2,3,5,6,7,7a-hexahydro-1*H*-isoindole-5-carboxamide [(+)-6aj]

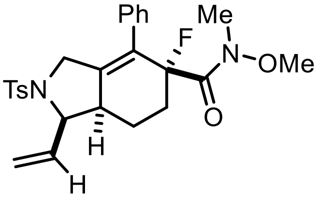

6aj (12.6 mg, 0.0260 mmol, 52% yield, >99:1 d.r., >99:1 e.r.) was prepared from **5a** (17.6 mg, 0.0500 mmol) and **2j** (8.0 mg, 0.0600 mmol).

Colorless oil; $[\alpha]^{25}_D$ +6.3 (*c* 1.26, $CHCl_3$, >99:1 e.r.); ^{1}H NMR (400 MHz, $CDCl_3$) δ 7.64 (d, J = 8.2 Hz, 2H), 7.33–7.28 (m, 3H), 7.24 (d, J = 7.9 Hz, 2H), 7.14–7.12 (m, 2H), 5.56 (ddd, J = 16.9, 10.0, 9.0 Hz, 1H), 5.26–5.21 (m, 1H), 5.17–5.14 (m, 1H), 4.52 (t, J = 8.4 Hz, 1H), 4.00 (ddd, J = 15.0, 6.5, 1.6 Hz, 1H), 3.69–3.64 (m, 1H), 3.56 (s, 3H), 3.11 (d, J = 1.8 Hz, 3H), 2.83–2.75 (m, 1H), 2.49 (br s, 1H), 2.41 (s, 3H), 2.06–1.93 (m, 1H), 1.72 (br s, 2H); ^{13}C NMR (101 MHz, $CDCl_3$) δ 143.2, 136.3, 133.7, 129.5, 129.3, 128.3, 127.7, 127.5, 118.6, 65.6, 48.9, 43.4, 21.5; HRMS (ESI) calcd for $C_{26}H_{29}FN_2NaO_4S^+$ [M+Na]$^+$ 507.1724, found 507.1727; CHIRALPAK AD-H, *n*-hexane/*i*-PrOH = 90:10, 1.0 mL/min, retention times: 25.2 min (major isomer) and 36.8 min (minor isomer).

(–)-{(1*R*,5*R*,7a*R*)-5-Fluoro-4-(4-methoxyphenyl)-2-(4-methylphenyl)-1-vinyl-2,3,5,6,7,7a-hexahydro-1*H*-isoindol-5-yl}(pyrrolidin-1-yl)methanone [(–)-6ba]

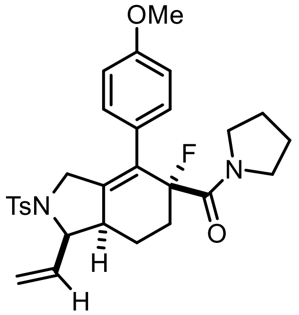

6ba [18.6 mg, 0.0355 mmol, 71% yield, 93:7 d.r., >99:1 e.r. (major)] was prepared from **5b** (19.1 mg, 0.0500 mmol) and **2a** (8.3 mg, 0.0600 mmol). Two diastereomers of **6ba** were obtained as a mixture.

The following experimental data were acquired using a mixture of isomers [93:7 d.r., >99:1 e.r. (major)]. White solid; mp 61.2–62.9 °C; $[\alpha]^{25}_D$ –2.5 (*c* 1.26, $CHCl_3$); **major diastereomer:** ^{1}H NMR (400 MHz, $CDCl_3$) δ 7.63 (d, J = 8.3 Hz, 2H), 7.24 (d, J = 8.0 Hz, 2H), 6.98 (d, J = 8.5 Hz, 2H), 6.82 (d, J = 8.8 Hz, 2H), 5.55 (ddd, J = 16.9, 10.0, 8.9 Hz, 1H), 5.25–5.20 (m, 1H), 5.16–5.14 (m, 1H), 4.54 (t, J = 8.4 Hz, 1H), 3.99 (ddd, J = 15.1, 7.2, 1.7 Hz, 1H), 3.81 (s, 3H), 3.59 (ddd, J = 15.1, 5.3, 1.6 Hz, 1H), 3.47–3.35 (m, 3H), 3.08–3.00 (m, 1H), 2.82–2.75 (m, 1H), 2.41 (s, 3H),

2.37–2.33 (m, 1H), 2.08–1.87 (m, 2H), 1.75–1.59 (m, 5H); **partial protons of minor diastereomer:** ^{1}H NMR (400 MHz, $CDCl_3$) δ 7.68 (d, *J* = 8.3 Hz, 2H), 4.61 (t, *J* = 8.6 Hz, 1H), 3.80 (s, 3H); ^{13}C NMR (101 MHz, $CDCl_3$) δ 169.3 (d, *J* = 26.9 Hz), 159.1, 143.2, 141.7 (d, *J* = 6.2 Hz), 136.3, 133.7, 131.8 (d, *J* = 21.1 Hz), 130.1, 129.4, 128.3, 127.5, 118.4, 113.8, 96.5 (d, *J* = 185.8 Hz), 65.8, 55.2, 49.0, 47.9, 46.9 (d, *J* = 16.8 Hz), 43.2 (d, *J* = 2.5 Hz), 33.9 (d, *J* = 23.2 Hz), 26.7 (d, *J* = 5.4 Hz), 23.0, 21.8 (d, *J* = 7.3 Hz), 21.5; ^{19}F NMR (377 MHz, $CDCl_3$) δ –145.7 (major), –143.7 (minor); HRMS (ESI) calcd for $C_{29}H_{33}FN_2NaO_4S$ [M+Na]$^+$ 547.2037, found 547.2034; CHIRALPAK AD-H, *n*-hexane/*i*-PrOH = 90:10, 1.0 mL/min, retention times: 29.0 min (major isomer) and 32.3 min (minor isomer).

(–)-{(1*R*,5*R*,7a*R*)-4-(4-Chlorophenyl)-5-fluoro-2-(4-methylphenyl)-1-vinyl-2,3,5,6,7,7a-hexahydro-1*H*-isoindol-5-yl}(pyrrolidin-1-yl)methanone [(–)-6ca (major diastereomer)]

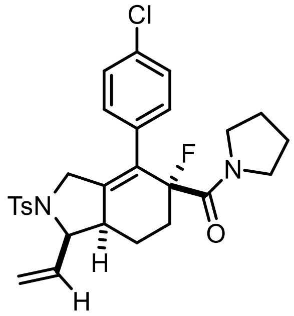

6ca [20.6 mg, 0.0389 mmol, 78% yield, >99:1 d.r., >99:1 e.r. (major)] was prepared from **5c** (19.3 mg, 0.0500 mmol) and **2a** (8.3 mg, 0.060 mmol). A trace amount (ca. <2%) of another diastereomer of **6ca** was detected in a crude reaction mixture.

White solid; mp 73.4–74.9 °C; $[\alpha]^{25}_D$ –1.2 (*c* 1.06, $CHCl_3$, >99:1 e.r.); ^{1}H NMR (400 MHz, $CDCl_3$) δ 7.63 (d, *J* = 8.3 Hz, 2H), 7.28 (d, *J* = 8.6 Hz, 2H), 7.24 (d, *J* = 8.0 Hz, 2H), 7.03 (d, *J* = 8.3 Hz, 2H), 5.54 (ddd, *J* = 16.9, 10.0, 8.9 Hz, 1H), 5.23 (ddd, *J* = 16.9, 1.5, 0.8 Hz, 1H), 5.17–5.14 (m, 1H), 4.56 (t, *J* = 8.4 Hz, 1H), 3.97 (ddd, *J* = 15.1, 7.1, 1.6 Hz, 1H), 3.55 (ddd, *J* = 15.1, 5.2, 1.7 Hz, 1H), 3.50–3.34 (m, 3H), 3.14–3.07 (m, 1H), 2.85–2.77 (m, 1H), 2.41 (s, 3H), 2.36–2.34 (m, 1H), 2.00–1.92 (m, 2H), 1.78–1.61 (m, 5H); ^{13}C NMR (101 MHz, $CDCl_3$) δ 168.8 (d, *J* = 26.9 Hz), 143.3, 142.2 (d, *J* = 6.2 Hz), 136.2, 134.5, 133.8, 133.5, 131.2 (d, *J* = 21.2 Hz), 130.6, 129.5, 128.6, 127.5, 118.6, 96.5 (d, *J* = 186.5 Hz), 65.7, 48.8, 47.9, 47.0 (d, *J* = 16.6 Hz), 43.3 (d, *J* = 2.5 Hz), 33.6 (d, *J* = 22.6 Hz), 26.7 (d, *J* = 5.3 Hz), 23.0, 21.6 (d, *J* = 7.3 Hz), 21.5; ^{19}F NMR (377 MHz, $CDCl_3$) δ –146.6; HRMS (ESI) calcd for $C_{28}H_{30}ClFN_2NaO_3S$ [M+Na]$^+$ 551.1542, found 551.1540; CHIRALPAK AD-H, *n*-hexane/*i*-PrOH = 95:5, 1.0 mL/min, retention times: 56.8 min (major isomer) and 61.5 min (minor isomer).

(–)-{(1*R*,5*S*,7a*R*)-2-(4-Methylphenyl)-4-phenyl-1-vinyl-2,3,5,6,7,7a-hexahydro-1*H*-isoindol-5-yl}(pyrrolidin-1-yl)methanone [(–)-6ad (major diastereomer)]

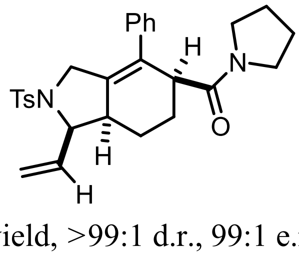

6ad [22.6 mg, 0.0474 mmol, 95% yield, >99:1 d.r., 99:1 e.r. (major)] was prepared from **5a** (17.6 mg, 0.0500 mmol) and **2d** (7.5 mg, 0.0600 mmol). A trace amount (ca. <2%) of another diastereomer of **6ad** was detected in a crude reaction mixture.

White solid; mp 105.7–107.4 °C; $[\alpha]^{25}_D$ –4.6 (*c* 2.26, $CHCl_3$, 99:1 e.r.); ^{1}H NMR (400 MHz, $CDCl_3$) δ 7.64 (d, *J* = 8.2 Hz, 2H), 7.29–7.27 (m, 1H), 7.25–7.21 (m, 4H), 7.05–7.03 (m, 2H), 5.65 (ddd, *J* = 17.0, 10.0, 9.1 Hz, 1H), 5.23 (ddd, *J* = 16.9, 1.6, 0.7 Hz, 1H), 5.15–5.13 (m, 1H), 4.55 (dd, *J* = 8.7, 8.0 Hz, 1H), 4.04 (dt, *J* = 14.2, 2.1 Hz, 1H), 3.72 (dt, *J* = 14.2, 1.6 Hz, 1H), 3.39–3.26

(m, 4H), 3.01–2.96 (m, 1H), 2.71–2.64 (m, 1H), 2.40 (s, 3H), 1.97–1.87 (m, 2H), 1.83–1.74 (m, 3H), 1.73–1.67 (m, 2H), 1.60–1.54 (m, 1H); ^{13}C NMR (101 MHz, $CDCl_3$) δ 172.0, 143.0, 140.5, 136.5, 136.1, 134.1, 131.1, 129.3, 128.4, 127.62, 127.55, 127.2, 118.1, 66.1, 49.1, 46.6, 45.8, 43.18, 43.15, 26.1, 26.0, 24.2, 21.5, 19.3; HRMS (ESI) calcd for $C_{28}H_{32}N_2NaO_3S$ $[M+Na]^+$ 499.2026, found 499.2024; CHIRALPAK AD-H, *n*-hexane/*i*-PrOH = 90:10, 1.0 mL/min, retention times: 53.6 min (major isomer) and 90.0 min (minor isomer).

(–)-(1*R*,5*S*,7a*R*)-*N*,*N*-Diethyl-2-(4-methylphenyl)-4-phenyl-1-vinyl-2,3,5,6,7,7a-hexahydro-1*H*-isoindole-5-carboxamide [(–)-6ah]

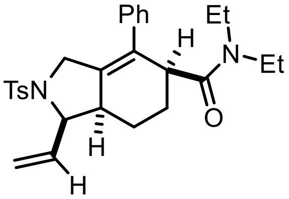

6ah [19.1 mg, 0.0399 mmol, 80% yield, 93:7 d.r., 99:1 e.r. (major)] was prepared from **5a** (17.6 mg, 0.0500 mmol) and **2h** (7.6 mg, 0.0600 mmol). Two diastereomers of **6ah** were obtained as a mixture.

The following experimental data were acquired using a mixture of isomers [93:7 d.r., 99:1 e.r. (major)]. Colorless oil; $[\alpha]^{25}_D$ –31.8 (*c* 1.91, $CHCl_3$); **major diastereomer:** ^{1}H NMR (400 MHz, $CDCl_3$) δ 7.64 (d, J = 8.3 Hz, 2H), 7.29–7.26 (m, 1H), 7.25–7.19 (m, 4H), 7.05–7.03 (m, 2H), 5.66 (ddd, J = 16.9, 10.0, 9.2 Hz, 1H), 5.23 (ddd, J = 16.9, 1.6, 0.6 Hz, 1H), 5.16–5.13 (m, 1H), 4.55 (dd, J = 8.8, 8.1 Hz, 1H), 4.05 (dt, J = 14.2, 2.1 Hz, 1H), 3.74 (dt, J = 14.2, 1.6 Hz, 1H), 3.43–3.41 (m, 1H), 3.39–3.29 (m, 1H), 3.25–3.02 (m, 3H), 2.70–2.65 (m, 1H), 2.39 (s, 3H), 1.91–1.79 (m, 3H), 1.59–1.54 (m, 1H) 0.99 (t, J = 7.0 Hz, 3H), 0.91 (t, J = 7.2 Hz, 3H); **partial protons of minor diastereomer:** ^{1}H NMR (400 MHz, $CDCl_3$) δ 7.55 (d, J = 8.2 Hz, 2H), 6.95–6.93 (m, 2H), 6.05 (ddd, J = 17.1, 10.2, 8.5 Hz, 1H), 3.58 (dt, J = 14.8, 2.1 Hz, 1H), 2.45 (s, 3H); ^{13}C NMR (101 MHz, $CDCl_3$) δ 172.7, 142.9, 140.4, 136.5, 135.9, 134.2, 131.4, 129.3, 128.3, 127.8, 127.5, 127.2, 118.1, 66.1, 49.0, 43.2, 42.2, 40.8, 40.7, 26.7, 21.4, 19.2, 14.6, 12.9; HRMS (ESI) calcd for $C_{28}H_{34}N_2NaO_3S$ $[M+Na]^+$ 501.2182, found 501.2181; CHIRALPAK AD-H, *n*-hexane/*i*-PrOH = 80:20, 1.0 mL/min, retention times: 7.6 min (major isomer) and 30.0 min (minor isomer).

(–)-(1*R*,5*S*,7a*R*)-*N*-Methyl-2-(4-methylphenyl)-*N*,4-diphenyl-1-vinyl-2,3,5,6,7,7a-hexahydro-1*H*-isoindole-5-carboxamide [(–)-6ag]

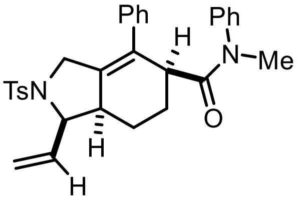

6ag [21.0 mg, 0.0410 mmol, 82% yield, 93:7 d.r., 98:2 e.r. (major)] was prepared from **5a** (17.6 mg, 0.0500 mmol) and **2g** (9.7 mg, 0.0600 mmol). Two diastereomers of **6ag** were obtained as a mixture.

The following experimental data were acquired using a mixture of isomers [93:7 d.r., 98:2 e.r. (major)]. White solid; mp 88.4–90.0 °C; $[\alpha]^{25}_D$ –67.8 (*c* 2.10, $CHCl_3$); **major diastereomer:** ^{1}H NMR (400 MHz, $CDCl_3$) δ 7.63 (d, J = 8.3 Hz, 2H), 7.32–7.28 (m, 3H), 7.24–7.21 (m, 5H) 6.95–6.93 (m, 2H), 6.61 (br s, 2H), 5.70 (ddd, J = 17.0, 10.0, 8.9 Hz, 1H), 5.28–5.24 (m, 1H), 5.21–5.18 (m, 1H), 4.54 (dd, J = 8.6, 8.1 Hz, 1H), 3.89 (dt, J = 14.4, 2.1 Hz, 1H), 3.75 (dt, J = 14.3, 1.7 Hz, 1H), 3.20–3.18 (m, 1H), 3.10 (s, 3H), 2.57–2.50 (m, 1H), 2.39 (s, 3H), 1.98–1.84 (m, 2H), 1.58–1.46 (m, 2H); **partial protons of minor diastereomer:** ^{1}H NMR (400 MHz, $CDCl_3$) δ 7.57 (d, J = 8.2 Hz, 2H), 6.83–6.81 (m, 2H), 6.02 (ddd, J = 17.2, 10.1, 8.4 Hz, 1H), 3.59 (dt, J = 14.8, 2.1 Hz, 1H), 3.04 (s, 3H), 2.47 (s, 3H); ^{13}C NMR (101 MHz, $CDCl_3$) δ 173.4, 143.9, 143.0, 140.2, 136.4,

135.9, 134.0, 131.3, 129.6, 129.3, 128.4, 127.9, 127.7, 127.5, 127.33, 127.26, 118.0, 66.1, 48.9, 43.0, 41.3, 37.4, 26.4, 21.4, 19.3; HRMS (ESI) calcd for $C_{31}H_{32}N_2NaO_3S$ [M+Na]$^+$ 535.2026, found 535.2020; CHIRALPAK AD-H, *n*-hexane/*i*-PrOH = 80:20, 1.0 mL/min, retention times: 10.7 min (major isomer) and 22.0 min (minor isomer).

(–)-(1*R*,5*S*,7a*R*)-*N*,*N*,4-Triphenyl-2-(4-methylphenyl)-1-vinyl-2,3,5,6,7,7a-hexahydro-1*H*-isoindole-5-carboxamide [(–)-6ak]

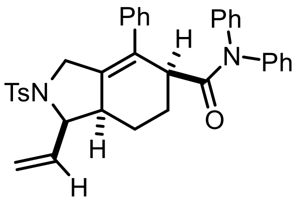

6ak [40.9 mg, 0.0712 mmol, 71% yield, 97:3 d.r., >99:1 e.r. (major)] was prepared from **5a** (35.2 mg, 0.100 mmol) and **2k** (26.8 mg, 0.120 mmol). Two diastereomers of **6ak** were obtained as a mixture.

The following experimental data were acquired using a mixture of isomers [97:3 d.r., >99:1 e.r (major)]. White solid; mp 102.6–104.2 °C; $[\alpha]^{25}_{D}$ –4.5 (*c* 4.09, $CHCl_3$); **major diastereomer:** ^{1}H NMR (400 MHz, $CDCl_3$) δ 7.63 (d, *J* = 8.3 Hz, 2H), 7.39–7.31 (m, 3H), 7.32–7.28 (m, 7H), 7.12–7.09 (br m, 1H), 7.04–7.02 (m, 2H), 6.98–6.96 (m, 2H), 6.73 (br s, 2H), 5.68 (ddd, *J* = 16.9, 9.9, 9.0 Hz, 1H), 5.27–5.22 (m, 1H), 5.14–5.11 (m, 1H), 4.55 (t, *J* = 8.3 Hz, 1H), 3.90–3.82 (m, 2H), 3.39–3.37 (m, 1H), 2.58–2.52 (m, 1H), 2.39 (s, 3H), 2.08–2.03 (m, 1H), 1.99–1.89 (m, 1H), 1.66–1.56 (m, 2H); **partial protons of minor diastereomer:** ^{1}H NMR (400 MHz, $CDCl_3$) δ 7.58 (d, *J* = 8.3 Hz, 2H), 6.03 (ddd, *J* = 17.2, 10.1, 8.4 Hz, 1H), 2.46 (s, 3H); ^{13}C NMR (101 MHz, $CDCl_3$) δ 173.7, 143.0, 142.7, 140.1, 136.4, 136.2, 134.0, 131.2, 129.6, 129.3, 128.9, 128.6, 128.5, 128.2, 127.82, 127.76, 127.49, 127.47, 126.5, 126.2, 118.0, 66.2, 48.8, 42.9, 42.1, 26.4, 21.5, 19.3; HRMS (ESI) calcd for $C_{36}H_{34}N_2NaO_3S$ [M+Na]$^+$ 597.2182, found 597.2173; CHIRALPAK AD-H, *n*-hexane/*i*-PrOH = 90:10, 1.0 mL/min, retention times: 15.5 min (major isomer) and 13.1 min (minor isomer).

(–)-{(1*R*,5*S*,7a*R*)-4-(4-Methoxyphenyl)-2-(4-methylphenyl)-1-vinyl-2,3,5,6,7,7a-hexahydro-1*H*-isoindol-5-yl}(pyrrolidin-1-yl)methanone [(–)-6bd]

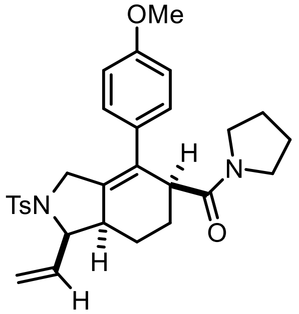

6bd [17.9 mg, 0.0353 mmol, 71% yield, 93:7 d.r., 99:1 e.r. (major)] was prepared from **5b** (19.1 mg, 0.0500 mmol) and **2d** (7.5 mg, 0.0600 mmol). Two diastereomers of **6bd** were obtained as a mixture.

The following experimental data were acquired using a mixture of isomers [93:7 d.r., 99:1 e.r. (major)]. Pale yellow solid; mp 110.0–111.7 °C; $[\alpha]^{25}_{D}$ –19.4 (*c* 1.79, $CHCl_3$); **major diastereomer:** ^{1}H NMR (400 MHz, $CDCl_3$) δ 7.65 (d, *J* = 8.3 Hz, 2H), 7.22 (d, *J* = 7.9 Hz, 2H), 6.96 (d, *J* = 8.8 Hz, 2H), 6.80 (d, *J* = 8.8 Hz, 2H), 5.64 (ddd, *J* = 16.9, 10.0, 9.1 Hz, 1H), 5.22 (ddd, *J* = 17.0, 1.6, 0.7 Hz, 1H), 5.14–5.12 (m, 1H), 4.53 (t, *J* = 8.4 Hz, 1H), 4.05 (dt, *J* = 14.2, 2.0 Hz, 1H), 3.80 (s, 3H), 3.72 (dt, *J* = 14.2, 1.6 Hz, 1H), 3.38 (t, *J* = 6.5 Hz, 2H), 3.40–3.29 (m, 2H), 3.08–3.03 (m, 1H), 2.69–2.63 (m, 1H), 2.39 (s, 3H), 1.93–1.86 (m, 2H), 1.84–1.69 (m, 6H); **partial protons of minor diastereomer:** ^{1}H NMR (400 MHz, $CDCl_3$) δ 7.55 (d, *J* = 8.3 Hz, 2H), 6.03 (ddd, *J* = 17.1, 10.1, 8.4 Hz, 1H), 3.81 (s, 3H), 2.44 (s, 3H); ^{13}C NMR (101 MHz, $CDCl_3$) δ 172.2,

158.6, 142.9, 136.5, 135.9, 134.2, 132.9, 130.6, 129.3, 128.7, 127.5, 118.0, 113.8, 66.1, 55.2, 49.1, 46.6, 45.8, 43.3, 43.2, 26.13, 26.05, 24.2, 21.5, 19.3; HRMS (ESI) calcd for $C_{29}H_{34}N_2NaO_4S$ $[M+Na]^+$ 529.2131, found 529.2126; CHIRALPAK AD-H, *n*-hexane/*i*-PrOH = 80:20, 1.0 mL/min, retention times: 17.2 min (major isomer) and 30.4 min (minor isomer).

(–)-{(1*R*,5*S*,7a*R*)-4-(4-Chlorophenyl)-2-(4-methylphenyl)-1-vinyl-2,3,5,6,7,7a-hexahydro-1*H*-isoindol-5-yl)(pyrrolidin-1-yl)methanone [(–)-6cd]

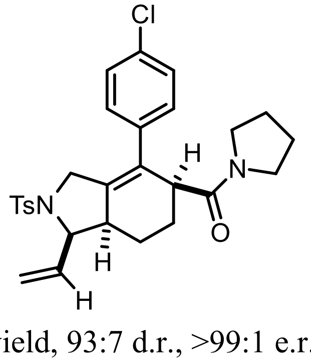

6cd [17.4 mg, 0.0340 mmol, 68% yield, 93:7 d.r., >99:1 e.r. (major)] was prepared from **5c** (19.3 mg, 0.0500 mmol) and **2d** (7.5 mg, 0.0600 mmol). Two diastereomers of **6cd** were obtained as a mixture.

The following experimental data were acquired using a mixture of isomers [93:7 d.r., >99:1 e.r. (major)]. White solid; mp 135.7 °C (decomp.); $[\alpha]^{25}_D$ –58.1 (*c* 1.74, $CHCl_3$); **major diastereomer:** ^1H NMR (400 MHz, $CDCl_3$) δ 7.64 (d, *J* = 8.3 Hz, 2H), 7.26–7.22 (m, 4H), 6.99 (d, *J* = 8.5 Hz, 2H), 5.62 (ddd, *J* = 16.9, 10.0, 9.1 Hz, 1H), 5.23 (ddd, *J* = 16.9, 1.5, 0.6 Hz, 1H), 5.15–5.13 (m, 1H), 4.55 (t, *J* = 8.4 Hz, 1H), 4.02 (dt, *J* = 14.2, 2.0 Hz, 1H), 3.67 (dt, *J* = 14.2, 1.6 Hz, 1H), 3.37 (t, *J* = 6.5 Hz, 2H), 3.34–3.00 (m, 2H), 3.10–3.04 (m, 1H), 2.70–2.65 (m, 1H), 2.40 (s, 3H), 1.92–1.66 (m, 8H); **partial protons of minor diastereomer:** ^1H NMR (400 MHz, $CDCl_3$) δ 7.55 (d, *J* = 8.2 Hz, 2H), 7.29 (d, *J* = 8.0 Hz, 2H), 6.90 (d, *J* = 8.5 Hz, 2H), 6.03 (ddd, *J* = 17.1, 10.1, 8.5 Hz, 2H), 3.51 (dt, *J* = 14.9, 2.0 Hz, 1H), 2.45 (s, 3H); ^{13}C NMR (101 MHz, $CDCl_3$) δ 171.7, 143.1, 139.0, 136.9, 136.4, 133.9, 133.1, 130.1, 129.3, 129.1, 128.7, 127.5, 118.2, 66.0, 48.9, 46.6, 45.8, 43.3, 43.2, 26.04, 26.03, 24.2, 21.5, 19.1; HRMS (ESI) calcd for $C_{28}H_{31}ClN_2NaO_3S$ $[M+Na]^+$ 533.1636, found 533.1638; CHIRALPAK AD-H, *n*-hexane/*i*-PrOH = 80:20, 1.0 mL/min, retention times: 16.8 min (major isomer) and 27.8 min (minor isomer).

(–)-(1*R*,5*S*,7a*R*)-4-(4-Bromophenyl)-2-(4-methylphenyl)-*N*,*N*-diphenyl-1-vinyl-2,3,5,6,7,7a-hexahydro-1*H*-isoindole-5-carboxamide [(–)-6dk]

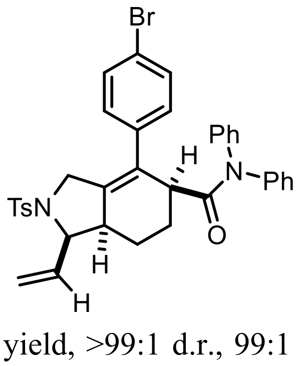

6dk (29.2 mg, 0.0447 mmol, 89% yield, >99:1 d.r., 99:1 e.r.) was prepared from **5d** (21.5 mg, 0.0500 mmol) and **2k** (13.4 mg, 0.0600 mmol). The absolute configuration was determined by X-ray crystallographic analysis (Figure S7).

White solid; mp 196.3 °C (decomp.); $[\alpha]^{25}_D$ –4.1 (*c* 2.92, $CHCl_3$, 99:1 e.r.); ^1H NMR (400 MHz, $CDCl_3$) δ 7.63 (d, *J* = 8.3 Hz, 2H), 7.50 (d, *J* = 8.5 Hz, 2H), 7.26–7.22 (m, 7H), 7.15–7.11 (br m, 1H), 7.00–6.99 (m, 2H), 6.93 (d, *J* = 8.5 Hz, 2H), 6.81 (br s, 2H), 5.63 (ddd, *J* = 16.9, 10.0, 9.1 Hz, 1H), 5.24 (ddd, *J* = 16.9, 1.5, 0.7 Hz, 1H), 5.14–5.11 (m, 1H), 4.55 (dd, *J* = 8.6, 8.1 Hz, 1H), 3.86 (dt, *J* = 14.5, 2.2 Hz, 1H), 3.76 (dt, *J* = 14.5, 1.7 Hz, 1H), 3.37–3.36 (m, 1H), 2.60–2.54 (m, 1H),

2.40 (s, 3H), 2.08–2.03 (m, 1H), 1.91–1.83 (m, 1H), 1.66–1.57 (m, 2H); ^{13}C NMR (101 MHz, $CDCl_3$) δ 173.4, 143.1, 139.1, 137.2, 136.4, 133.8, 131.7, 130.03, 129.97, 129.7, 129.4, 129.0, 128.6, 127.5, 126.4, 121.5, 118.3, 66.1, 48.7, 43.1, 42.1, 26.4, 21.5, 19.1; HRMS (ESI) calcd for $C_{36}H_{33}BrN_2NaO_3S$ [M+Na]$^+$ 675.1287, found 675.1287; CHIRALPAK IF-3, *n*-hexane/*i*-PrOH = 70:30, 1.0 mL/min, retention times: 9.7 min (major isomer) and 13.0 min (minor isomer).

(–)-{(1*R*,5*S*,7a*R*)-4-Methyl-2-(4-methylphenyl)-1-vinyl-2,3,5,6,7,7a-hexahydro-1*H*-isoindol-5-yl}(pyrrolidin-1-yl)methanone [(–)-6ed]

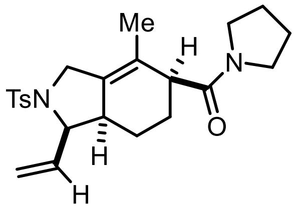

6ed (10.6 mg, 0.0256 mmol, 51% yield, >99:1 d.r., >99:1 e.r.) was prepared from **5e** (14.4 mg, 0.0500 mmol) and **2d** (7.5 mg, 0.0600 mmol).

Colorless oil; $[\alpha]^{25}_{D}$ –16.3 (*c* 1.06, $CHCl_3$, >99:1 e.r.); ^{1}H NMR (400 MHz, $CDCl_3$) δ 7.71 (d, *J* = 8.2 Hz, 2H), 7.25 (d, *J* = 8.5 Hz, 2H), 5.49 (ddd, *J* = 16.9, 9.9, 9.1 Hz, 1H), 5.21 (ddd, *J* = 16.9, 1.7, 0.6 Hz, 1H), 5.10–5.07 (m, 1H), 4.49 (dd, *J* = 8.9, 7.8 Hz, 1H), 4.09–4.05 (m, 1H), 3.94–3.90 (m, 1H), 3.55–3.45 (m, 3H), 3.44–3.35 (m, 1H), 3.02–3.01 (m, 1H), 2.57–2.50 (m, 1H), 2.41 (s, 3H), 2.04–1.77 (m, 4H), 1.73–1.65 (m, 1H), 1.63–1.56 (m, 2H), 1.52 (s, 3H), 1.49–1.45 (m, 1H); ^{13}C NMR (101 MHz, $CDCl_3$) δ 172.3, 143.0, 136.4, 133.9, 132.9, 129.3, 127.7, 124.9, 118.1, 66.4, 48.5, 46.8, 45.8, 43.12, 43.05, 26.2, 25.8, 24.3, 21.5, 19.6, 18.4; HRMS (ESI) calcd for $C_{23}H_{30}N_2NaO_3S$ [M+Na]$^+$ 437.1889, found 437.1876; CHIRALPAK IE-3, *n*-hexane/*i*-PrOH = 70:30, 1.0 mL/min, retention times: 46.6 min (major isomer) and 68.4 min (minor isomer).

(–)-{(1*R*,5*S*,7a*R*)-4-Butyl-2-(4-methylphenyl)-1-vinyl-2,3,5,6,7,7a-hexahydro-1*H*-isoindol-5-yl}(pyrrolidin-1-yl)methanone [(–)-6fd]

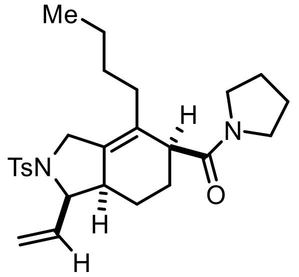

6fd (8.9 mg, 0.020 mmol, 39% yield, >99:1 d.r., >99:1 e.r.) was prepared from **5f** (16.6 mg, 0.0500 mmol) and **2d** (7.5 mg, 0.0600 mmol) in $(CH_2Cl)_2$ at 60 °C according to the general procedure procedure.

Colorless oil; $[\alpha]^{25}_{D}$ –32.5 (*c* 0.89, $CHCl_3$, >99:1 e.r.); ^{1}H NMR (400 MHz, $CDCl_3$) δ 7.71 (d, *J* = 8.3 Hz, 2H), 7.25 (d, *J* = 9.1 Hz, 2H), 5.49 (ddd, *J* = 16.9, 9.9, 9.4 Hz, 1H), 5.23–5.18 (m, 1H), 5.09–5.06 (m, 1H), 4.48 (dd, *J* = 9.0, 7.8 Hz, 1H), 4.07 (d, *J* = 13.5 Hz, 1H), 3.94 (d, *J* = 13.1 Hz, 1H), 3.50–3.34 (m, 4H), 3.16–3.15 (m, 1H), 2.59–2.55 (m, 1H), 2.41 (s, 3H), 2.04–1.79 (m, 6H), 1.71–1.55 (m, 3H), 1.48–1.43 (m, 1H), 1.36–1.20 (m, 4H), 0.88 (t, *J* = 6.9 Hz, 3H); ^{13}C NMR (101 MHz, $CDCl_3$) δ 172.4, 143.0, 136.5, 133.9, 133.1, 129.8, 129.3, 127.7, 118.1, 66.2, 48.2, 46.8, 45.8, 43.1, 40.4, 32.4, 30.4, 26.3, 25.9, 24.3, 22.7, 21.5, 19.3, 14.0; HRMS (ESI) calcd for $C_{26}H_{36}N_2NaO_3S$ [M+Na]$^+$ 479.2339, found 479.2360; CHIRALPAK AD-H, *n*-hexane/*i*-PrOH = 90:10, 1.0 mL/min, retention times: 8.7 min (major isomer) and 11.5 min (minor isomer).

(–)-{(1*R*,5*R*,7a*R*)-5-Fluoro-4-phenyl-1-vinyl-1,3,5,6,7,7a-hexahydroisobenzofuran-5-yl}-(pyrrolidin-1-yl)methanone [(–)-6ga]

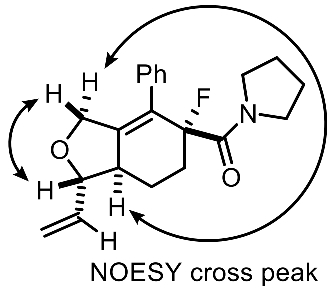

6ga (7.2 mg, 0.021 mmol, 42% yield, >99:1 d.r., >99:1 e.r.) was prepared from **5g** (9.9 mg, 0.050 mmol) and **2a** (21.5 mg, 0.150 mmol), purified by silica gel PTLC (eluent: *n*-hexane/EtOAc = 2:1) according to the general procedure. The relative configuration was determined by the NOESY experiment.

Colorless oil; $[\alpha]^{25}_{D}$ –27.7 (*c* 0.72, $CHCl_3$, >99:1 e.r.); ^{1}H NMR (400 MHz, $CDCl_3$) δ 7.33–7.25 (m, 3H), 7.14–7.11 (m, 2H), 5.91 (ddd, *J* = 17.3, 10.3, 7.1 Hz, 1H), 5.34 (ddd, *J* = 17.2, 1.4, 1.1 Hz, 1H), 5.24 (ddd, *J* = 10.3, 1.5, 0.8 Hz, 1H), 4.35 (ddd, *J* = 14.5, 7.9, 1.4 Hz, 1H), 4.06 (ddd, *J* = 14.5, 6.8, 2.0 Hz, 1H), 3.90 (dd, *J* = 10.1, 7.2 Hz, 1H), 3.49–3.33 (m, 3H), 2.87–2.80 (m, 1H), 2.54–2.44 (m, 2H), 2.17–1.92 (m, 3H), 1.73–1.58 (m, 3H), 1.48–1.38 (m, 1H); ^{13}C NMR (101 MHz, $CDCl_3$) δ 169.9 (d, *J* = 27.5 Hz), 147.1 (d, *J* = 6.5 Hz), 136.64, 136.58, 129.1 (d, *J* = 1.2 Hz), 128.6 (d, *J* = 19.9 Hz), 128.2, 127.6, 118.0, 97.0 (d, *J* = 186.4 Hz), 85.9, 68.7 (d, *J* = 1.4 Hz), 47.8, 46.9 (d, *J* = 17.1 Hz), 46.2 (d, *J* = 2.6 Hz), 35.0 (d, *J* = 23.9 Hz), 26.6 (d, *J* = 5.5 Hz), 23.7 (d, *J* = 6.7 Hz), 22.9; ^{19}F NMR (377 MHz, $CDCl_3$) δ –144.0; HRMS (ESI) calcd for $C_{21}H_{24}NNaO_2$ $[M+Na]^+$ 364.1683, found 364.1701; CHIRALPAK AD-H, *n*-hexane/*i*-PrOH = 95:5, 1.0 mL/min, retention times: 8.5 min (major isomer) and 11.7 min (minor isomer).

(–)-(1*S*,5*S*,7a*R*)-*N*-Methyl-*N*,4-diphenyl-1-vinyl-1,3,5,6,7,7a-hexahydroisobenzofuran-5-carboxamide [(–)-6gg]

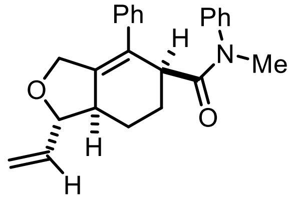

6gg (11.1 mg, 0.031 mmol, 62% yield, >99:1 d.r., >99:1 e.r.) was prepared from **5g** (9.9 mg, 0.0500 mmol) and **2g** (24.2 mg, 0.150 mmol), purified by silica gel PTLC (eluent: *n*-hexane/EtOAc = 2:1) according to the general procedure.

Colorless oil; $[\alpha]^{25}_{D}$ –40.0 (*c* 0.41, $CHCl_3$, >99:1 e.r.); ^{1}H NMR (400 MHz, $CDCl_3$) *δ* 7.34–7.28 (m, 3H), 7.27–7.25 (m, 3H), 7.03–7.01 (m, 2H), 6.64 (br s, 2H), 5.91 (ddd, *J* = 17.3, 10.3, 7.1 Hz, 1H), 5.35 (ddd, *J* = 17.2, 1.5, 1.1 Hz, 1H), 5.21 (ddd, *J* = 10.3, 1.6, 0.7 Hz, 1H), 4.30 (ddd, *J* = 13.5, 2.3, 1.7 Hz, 1H), 4.25 (dt, *J* = 13.5, 2.0 Hz, 1H), 3.93 (dd, *J* = 10.1, 7.2 Hz, 1H), 3.31–3.28 (m, 1H), 3.13 (s, 3H), 2.22–2.15 (m, 1H), 2.02–1.97 (m, 1H), 1.96–1.86 (m, 1H), 1.76 (ddt, *J* = 12.2, 5.5, 3.3 Hz, 1H), 1.57 (tdd, *J* = 13.5, 7.0, 3.2 Hz, 1H); ^{13}C NMR (101 MHz, $CDCl_3$) δ 174.1, 144.0, 140.6, 140.5, 137.4, 129.5, 128.3, 128.1, 127.9, 127.6, 127.4, 127.0, 117.5, 86.1, 68.7, 46.1, 41.0, 37.3, 27.0, 20.1; HRMS (ESI) calcd for $C_{24}H_{25}NNaO_2$ $[M+Na]^+$ 382.1777, found 382.1760; CHIRALPAK OD-H, *n*-hexane/*i*-PrOH = 90:10, 1.0 mL/min, retention times: 6.2 min (major isomer) and 12.1 min (minor isomer).

General Procedure for Rh-Catalyzed Enantioselective Desymmetrization Using 1,6-Enyne 5h–5k (Figure 4): (*R*)-H_8-BINAP (3.8 mg, 0.0060 mmol) and $[Rh(cod)_2]BF_4$ (2.1 mg, 0.0050 mmol) were dissolved in CH_2Cl_2 (2.0 mL) in a Schlenk tube, and the mixture was stirred at room temperature for 10 min. After the introduction of H_2 and stirring at room temperature for 30 min, the resulting mixture was concentrated to dryness. The residue was dissolved in $(CH_2Cl)_2$ (0.5 mL), followed by the addition of a solution of **5** (0.050 mmol) and **2** (0.060 mmol) in $(CH_2Cl)_2$ (0.5 mL). The mixture was stirred at 60 °C for 48 h, then passed through short-path silica gel column chromatography to remove the Rh complex and concentrated. The crude product was further purified by silica gel PTLC twice (eluent: *n*-hexane/EtOAc = 1:1 and CH_2Cl_2/EtOAc = 10:1) to furnish **7**.

(–)-(*E*)-4-{(2*R*,3*R*)-4-(*Z*)-Benzylidene-3-methyl-1-(4-methylphenyl)-2-(prop-1-en-2-yl)-pyrrolidin-3-yl}-2-fluoro-1-(pyrrolidin-1-yl)but-2-en-1-one [(–)-7ha (major diastereomer)]

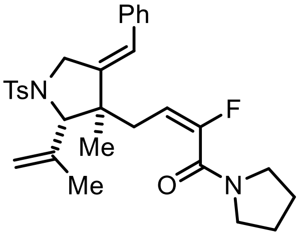

7ha [9.3 mg, 0.018 mmol, 36% yield, >99:1 d.r., 96:4 e.r. (major)] was prepared from **5h** (19.0 mg, 0.0500 mmol) and **2a** (8.6 mg, 0.0600 mmol). A trace amount (ca. <2%) of another diastereomer of **7ha** was detected in a crude reaction mixture.

Colorless oil; $[\alpha]^{25}_{D}$ –32.5 (*c* 0.93, $CHCl_3$, 96:4 e.r.); ^1H NMR (400 MHz, $CDCl_3$) δ 7.73 (d, *J* = 8.3 Hz, 2H), 7.38–7.34 (m 2H), 7.27–7.24 (m, 3H), 7.16 (d, *J* = 7.2 Hz, 2H), 6.19 (t, *J* = 2.1 Hz, 1H), 5.41 (ddd, *J* = 23.2, 9.2, 7.5 Hz, 1H), 4.87 (s, 1H), 4.82 (t, *J* = 1.5 Hz, 1H), 4.55 (dd, *J* = 14.5, 2.8 Hz, 1H), 4.17 (dd, *J* = 14.4, 1.9 Hz, 1H), 4.17 (s, 1H), 3.52–3.43 (m, 4H), 2.48 (dd, *J* = 14.5, 9.3 Hz, 1H), 2.40–2.34 (m, 1H), 2.39 (s, 3H), 1.88–1.75 (m, 4H), 1.21 (s, 3H), 1.06 (s, 3H); ^{13}C NMR (101 MHz, $CDCl_3$) δ 160.1 (d, *J* = 32.7 Hz), 152.8 (d, *J* = 263.1 Hz), 143.4, 143.1, 142.9, 136.6, 136.3, 129.4, 128.54, 128.45, 127.19, 127.15, 122.1, 115.8, 111.0 (d, *J* = 18.7 Hz), 73.2, 50.1, 49.7 (d, *J* = 2.1 Hz), 47.1 (d, *J* = 9.3 Hz), 46.2, 35.8 (d, *J* = 6.0 Hz), 26.0 (d, *J* = 3.1 Hz), 23.8, 21.5, 18.6, 18.2; ^{19}F NMR (377 MHz, $CDCl_3$) δ –111.7; HRMS (ESI) calcd for $C_{30}H_{35}FN_2NaO_3S$ $[M+Na]^+$ 545.2245, found 545.2225; CHIRALPAK IG-3, *n*-hexane/*i*-PrOH = 70:30, 1.0 mL/min, retention times: 26.3 min (major isomer) and 33.7 min (minor isomer).

(–)-(*E*)-4-[(2*R*,3*R*)-4-{(*Z*)-4-Bromobenzylidene}-3-methyl)-1-(4-methylphenyl)-2-(prop-1-en-2-ylpyrrolidin-3-yl]-2-fluoro-1-(pyrrolidin-1-yl)but-2-en-1-one [(–)-7ia (major diastereomer)]

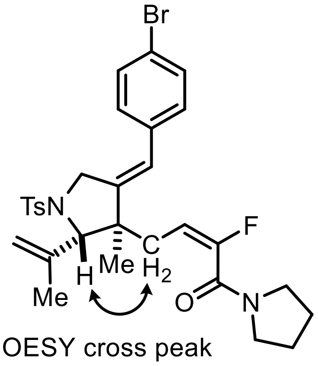

7ia [15.3 mg, 0.0254 mmol, 51% yield, >99:1 d.r., 88:12 e.r. (major)] was prepared from **5i** (22.9 mg, 0.0500 mmol) and **2a** (8.6 mg, 0.0600 mmol). The relative configuration was determined by the NOESY experiment. A trace amount (ca. <2%) of another diastereomer of **7ia** was detected in a crude reaction mixture.

White solid; mp 131.6 °C (decomp.); $[\alpha]^{25}_{D}$ –20.2 (*c* 1.35, $CHCl_3$, 88:12 e.r.); ^1H NMR (400 MHz, $CDCl_3$) δ 7.72 (d, *J* = 8.3 Hz, 2H), 7.48 (d, *J* = 8.4 Hz, 2H), 7.25 (d, *J* = 7.9 Hz, 2H), 7.02 (d, *J* = 8.4 Hz, 2H), 6.13 (t, *J* = 2.2 Hz, 1H), 5.40 (ddd, *J* = 23.1, 9.0, 7.7 Hz, 1H), 4.86 (s, 1H), 4.82 (t, *J* = 1.5 Hz, 1H), 4.52 (dd, *J* = 14.5, 2.8 Hz, 1H), 4.18 (s, 1H), 4.08 (dd, *J* = 14.5, 1.9 Hz, 1H), 3.55–3.45 (m, 4H), 2.47 (dd, *J* = 14.4, 9.1 Hz, 1H), 2.39 (s, 3H), 2.40–2.34 (m, 1H), 1.93–1.78 (m, 4H), 1.19 (s, 3H), 1.05 (s, 3H); ^{13}C NMR (101 MHz, $CDCl_3$) δ 160.1 (d, *J* = 32.6 Hz), 152.9 (d, *J* = 263.4 Hz), 144.1, 143.24, 143.19, 136.5, 135.1, 131.7, 129.9, 129.5, 127.1, 121.2, 121.0, 115.9, 110.8 (d, *J* = 18.7 Hz), 73.0, 50.0, 49.8 (d, *J* = 2.1 Hz), 47.2 (d, *J* = 9.2 Hz), 46.2, 35.8 (d, *J* = 5.9 Hz), 26.1 (d, *J* = 3.0 Hz), 23.8, 21.5, 18.5, 18.2; ^{19}F NMR (377 MHz, $CDCl_3$) δ –111.6; HRMS (ESI) calcd for $C_{30}H_{34}BrFN_2NaO_3S$ $[M+Na]^+$ 623.1350, found 623.1350; CHIRALPAK IF-3, *n*-hexane/*i*-PrOH = 90:10, 1.0 mL/min, retention times: 57.6 min (major isomer) and 43.7 min (minor isomer).

(–)-(*E*)-4-{(2*S*,3*R*,*Z*)-4-Ethylidene-3-methyl-1-(4-methylphenyl)-2-(prop-1-en-2-yl)-pyrrolidin-3-yl}-2-fluoro-1-(pyrrolidin-1-yl)but-2-en-1-one [(–)-7ja (major diastereomer)]

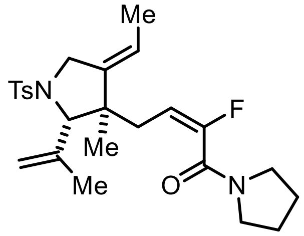

7ja [13.1 mg, 0.0285 mmol, 57% yield, 65:35 d.r., 99:1 e.r. (major)] was prepared from **5j** (15.9 mg, 0.0500 mmol) and **2a** (8.6 mg, 0.0600 mmol). Two diastereomers of **7ja** were isolated separately (major diastereomer: 8.5 mg, 0.0185 mmol, 37% yield, 99:1 e.r.; minor diastereomer: 4.6 mg, 0.0100 mmol, 20% yield).

Colorless oil; $[\alpha]^{25}_{D}$ –7.0 (*c* 0.50, $CHCl_3$, 99:1 e.r.); ^1H NMR (400 MHz, $CDCl_3$) δ 7.72 (d, *J* = 8.2 Hz, 2H), 7.27 (d, *J* = 7.7 Hz, 2H), 5.33 (ddd, *J* = 23.3, 9.3, 7.1 Hz, 1H), 5.21 (qt, *J* = 11.3, 2.5 Hz, 1H), 4.84–4.83 (m, 1H), 4.82–4.80 (m, 1H), 4.12 (s, 1H), 4.07–4.02 (m, 1H), 4.00–3.95 (m, 1H), 3.55–3.47 (m, 4H), 2.41 (s, 3H), 2.36 (dd, *J* = 14.6, 9.3 Hz, 1H), 2.12 (ddd, *J* = 14.6, 7.1, 2.7 Hz, 1H), 1.96–1.86 (m, 4H), 1.58 (dt, *J* = 6.8, 1.3 Hz, 3H), 1.28 (s, 3H), 0.89 (s, 3H); ^{13}C NMR (101 MHz, $CDCl_3$) δ 160.2 (d, *J* = 32.8 Hz), 152.6 (d, *J* = 262.1 Hz), 143.7, 143.0, 141.7, 136.5, 129.4, 127.2, 116.5, 115.4, 111.2 (d, *J* = 18.2 Hz), 74.3, 48.9, 48.2 (d, *J* = 2.0 Hz), 47.1 (d, *J* = 8.8 Hz), 46.1, 35.8 (d, *J* = 5.7 Hz), 26.1 (d, *J* = 2.8 Hz), 23.8, 21.5, 18.6, 18.3, 14.3; ^{19}F NMR (377 MHz, $CDCl_3$) δ –112.5; HRMS (ESI) calcd for $C_{25}H_{33}FN_2NaO_3S$ $[M+Na]^+$ 483.2088, found 483.2080; CHIRALPAK IE-3, *n*-hexane/*i*-PrOH = 80:20, 1.0 mL/min, retention times: 44.7 min (major isomer) and 50.2 min (minor isomer).

Minor diastereomer: Colorless oil; ^1H NMR (400 MHz, $CDCl_3$) δ 7.74–7.70 (m, 2H), 7.28 (d, *J* = 8.0 Hz, 2H), 5.58 (ddd, *J* = 23.7, 9.5, 6.4 Hz, 1H), 5.29–5.23 (m, 1H), 4.97 (s, 1H), 4.87–4.85 (m, 1H), 4.10–3.98 (m, 3H), 3.55–3.48 (m, 4H), 2.78 (dd, *J* = 15.3, 9.5 Hz, 1H), 2.41 (s, 3H), 2.18–2.11 (m, 1H), 1.94–1.85 (m, 4H), 1.54 (d, *J* = 6.8 Hz, 3H), 1.38 (s, 3H), 0.89 (s, 3H); ^{13}C NMR (101 MHz, $CDCl_3$) δ 160.3 (d, *J* = 32.5 Hz), 152.3 (d, *J* = 252.6 Hz), 143.4, 143.1, 142.9, 136.0, 129.4, 127.3, 116.4, 115.0, 113.1 (d, *J* = 18.9 Hz), 74.9, 49.0, 47.4 (d, *J* = 1.8 Hz), 47.1 (d, *J* = 10.2 Hz), 46.3, 29.4 (d, *J* = 51.0 Hz), 26.3 (d, *J* = 3.4 Hz), 26.2, 23.7, 21.5, 18.4, 14.2; ^{19}F NMR (377 MHz, $CDCl_3$) δ –113.5.

(–)-(*Z*)-4-[(2*S*,3*R*)-4-{(*Z*)-4-Bromobenzylidene}-3-methyl-1-(4-methylphenyl)-2-(prop-1-en-2-yl)pyrrolidin-3-yl]-2-methyl-1-(pyrrolidin-1-yl)but-2-en-1-one [(–)-7ie]

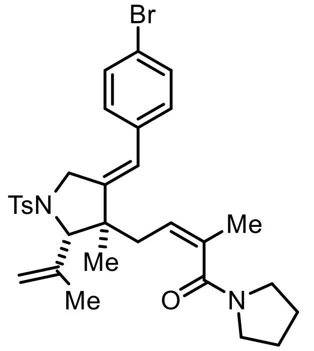

7ie [29.1 mg, 0.0487 mmol, 97% yield, 80:20 d.r., 89:11 e.r. (major)] was prepared from **5i** (22.9 mg, 0.0500 mmol) and **2e** (8.1 mg, 0.0600 mmol) in CH_2Cl_2 at room temperature for 16 h according to the general procedure. Two diastereomers of **7ie** were obtained as a mixture.

The following experimental data were acquired using a mixture of isomers [80:20 d.r., 89:11 e.r. (major)]. White solid; mp 69.8–71.2 °C; $[\alpha]^{25}_{D}$ –1.3 (*c* 2.91, $CHCl_3$); **major diastereomer:** ^1H NMR (400 MHz, $CDCl_3$) δ 7.71 (d, *J* = 8.3 Hz, 2H), 7.49–7.45 (m, 2H), 7.24 (d, *J* = 8.0 Hz, 2H), 7.05 (d, *J* = 8.4 Hz, 2H), 6.11 (t, *J* = 1.9 Hz, 1H), 5.23–5.19 (m, 1H), 4.85 (s, 1H), 4.79 (quint, *J* = 1.4 Hz, 1H), 4.51 (dd, *J* = 14.4, 2.8 Hz, 1H), 4.20 (s, 1H), 4.00 (dd, *J* = 14.4, 1.8 Hz, 1H), 3.58–3.43 (m, 3H), 3.33–3.21 (m, 1H), 2.38 (s, 3H), 2.14–2.01 (m, 2H), 1.94–1.87 (m, 4H), 1.85 (s, 3H), 1.12 (s, 3H), 1.03 (s, 3H); **partial protons of minor diastereomer:** ^1H NMR (400 MHz, $CDCl_3$) δ 7.68 (d, *J* = 8.4 Hz, 2H), 7.00 (d, *J* = 8.5 Hz, 2H), 6.16 (t, *J* = 2.1 Hz, 1H), 5.39 (ddd, *J* = 15.1, 9.0, 1.5 Hz, 1H), 4.99 (s, 1H), 4.90 (quint, *J* = 1.4 Hz, 1H), 4.39 (dd, *J* = 14.6, 2.6 Hz, 1H), 4.12 (dd, *J* = 14.6, 1.9 Hz, 1H), 4.05 (s, 1H), 2.39 (s, 3H), 1.32 (s, 3H), 1.02 (s, 3H); ^{13}C NMR (101 MHz, $CDCl_3$) δ 170.1, 170.0, 145.1, 144.2, 143.3, 143.09, 143.07, 142.9, 136.7, 136.6, 136.1, 135.6, 135.3, 131.61, 131.57, 130.0, 129.5, 129.4, 127.2, 127.1, 123.7, 122.7, 121.1, 121.0, 119.8, 116.8, 116.0, 73.8, 73.3, 50.0, 49.8, 49.6, 48.8, 46.94, 46.91, 45.0, 44.9, 40.0, 33.5, 25.93, 25.88, 24.5, 24.4, 21.5, 20.2, 20.1, 18.6, 18.5, 18.3; HRMS (ESI) calcd for $C_{31}H_{37}BrN_2NaO_3S$ $[M+Na]^+$ 619.1600, found 619.1632; CHIRALPAK IG-3, *n*-hexane/*i*-PrOH = 80:20, 1.0 mL/min, retention times: 60.8 min (major isomer) and 35.0 min (minor isomer).

(–)-(*E*)-4-[(2*S*,3*R*)-4-{(*Z*)-4-Bromobenzylidene}-3-methyl-1-(4-methylphenyl)-2-(prop-1-en-2-yl)pyrrolidin-3-yl]-2-chloro-1-(pyrrolidin-1-yl)but-2-en-1-one [(–)-7if]

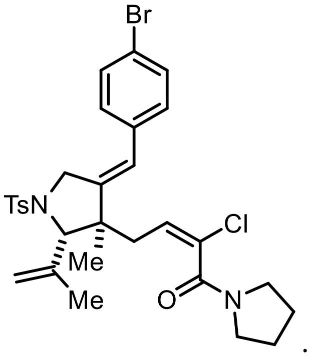

7if [15.8 mg, 0.0256 mmol, 51% yield, 84:16 d.r., 91:9 e.r. (major)] was prepared from **5i** (22.9 mg, 0.0500 mmol) and **2f** (9.6 mg, 0.0600 mmol) in CH_2Cl_2 at room temperature for 16 h according to the general procedure. Two diastereomers of **7if** were obtained as a mixture.

The following experimental data were acquired using a mixture of isomers [84:16 d.r., 91:9 e.r. (major)]. White solid; mp 87.2–89.0 °C; $[\alpha]^{25}_{D}$ –23.2 (*c* 1.58, $CHCl_3$); **major diastereomer:** ^1H NMR (400 MHz, $CDCl_3$) δ 7.70 (d, *J* = 8.2 Hz, 2H), 7.50–7.46 (m, 2H), 7.25 (d, *J* = 8.1 Hz, 2H), 7.04 (d, *J* = 8.4 Hz, 2H), 6.15 (t, *J* = 2.1 Hz, 1H), 5.72 (t, *J* = 8.0 Hz, 1H), 4.86 (s, 1H), 4.81 (m, 1H), 4.52 (dd, *J* = 14.6, 2.8 Hz, 1H), 4.19 (s, 1H), 4.01 (dd, *J* = 14.6, 1.9 Hz, 1H), 3.60–3.38 (m,

4H), 2.39 (s, 3H), 2.24 (d, J = 8.0 Hz, 2H), 1.98–1.91 (m, 4H), 1.13 (s, 3H), 1.07 (s, 3H); **partial protons of minor diastereomer:** ^{1}H NMR (400 MHz, $CDCl_3$) δ 7.00 (d, J = 8.4 Hz, 2H), 5.92 (q, J = 5.1 Hz, 1H), 5.03 (s, 1H), 4.92 (m, 1H), 4.42 (dd, J = 14.6, 2.6 Hz, 1H), 4.13 (dd, J = 14.6, 2.0 Hz, 1H), 4.08 (s, 1H), 2.52 (dd, J = 15.0, 9.8 Hz, 1H), 2.13 (dd, J = 14.9, 5.6 Hz, 1H), 1.31 (s, 3H), 1.05 (s, 3H); ^{13}C NMR (101 MHz, $CDCl_3$) δ 162.8, 162.7, 144.6, 143.5, 143.4, 143.3, 142.8, 142.7, 136.5, 135.6, 135.1, 135.0, 131.69, 131.65, 130.0, 129.9, 129.50, 129.45, 128.0, 127.2, 127.1, 126.7, 126.6, 126.2, 121.5, 121.3, 119.9, 117.2, 116.3, 73.5, 73.2, 49.9, 49.8, 49.6, 48.6, 47.4, 47.3, 45.6, 39.8, 33.5, 26.1, 26.0, 25.9, 24.33, 24.28, 21.5, 18.5, 18.4, 18.2; HRMS (ESI) calcd for $C_{30}H_{34}BrClN_2NaO_3S$ $[M+Na]^+$ 639.1054, found 639.1078; CHIRALPAK ID-3, *n*-hexane/*i*-PrOH = 70:30, 1.0 mL/min, retention times: 39.5 min (major isomer) and 19.3 min (minor isomer).

(+)-(4*R*,5*R*)-3-(*Z*)-Benzylidene-4-{(*E*)-3-fluoro-4-oxo-4-(pyrrolidin-1-yl)but-2-en-1-yl}-4-methyl-5-(prop-1-en-2-yl)dihydrofuran-2(3*H*)-one [(+)-7ka]

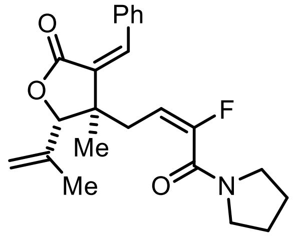

7ka (11.4 mg, 0.0297 mmol, 59% yield, >99:1 d.r., 94:6 e.r.) was prepared from **5k** (12.0 mg, 0.0500 mmol) and **2a** (8.6 mg, 0.0600 mmol) at 40 °C for 16 h according to the general procedure.

Colorless oil; $[\alpha]^{25}_{D}$ +12.8 (*c* 1.14, $CHCl_3$, 94:6 e.r.); ^{1}H NMR (400 MHz, CDCl3) δ 7.87–7.85 (m, 2H), 7.41–7.33 (m, 3H), 6.72 (s, 1H), 5.56 (ddd, J = 22.9, 8.8, 8.1 Hz, 1H), 5.06–5.05 (m, 1H), 5.03–5.02 (m, 1H), 4.75 (s, 1H), 3.49–3.36 (m, 4H), 2.83 (ddd, J = 14.6, 8.9, 0.5 Hz, 1H), 2.69 (ddd, J = 14.5, 8.1, 1.7 Hz, 1H), 1.80–1.69 (m, 6H), 1.66–1.59 (m, 1H) 1.21 (s, 3H); ^{13}C NMR (101 MHz, $CDCl_3$) δ 168.7, 160.0 (d, J = 32.2 Hz), 153.4 (d, J = 266.0 Hz), 141.1, 138.9, 133.3, 131.9, 130.9, 129.7, 128.2, 115.8, 110.6 (d, J = 19.2 Hz), 87.5, 47.7 (d, J = 2.3 Hz), 47.1 (d, J = 9.9 Hz), 46.3, 37.3 (d, J = 6.0 Hz), 25.9 (d, J = 3.2 Hz), 23.6, 21.8, 18.7; ^{19}F NMR (377 MHz, $CDCl_3$) δ –110.2; HRMS (ESI) calcd for $C_{23}H_{26}FNNaO_3$ $[M+Na]^+$ 406.1789, found 406.1794; CHIRALPAK AD-H, *n*-hexane/*i*-PrOH = 90:10, 1.0 mL/min, retention times: 11.4 min (major isomer) and 14.3 min (minor isomer).

2.6. Synthetic Applications

2.6.1. Synthetic Applications for Parallel Kinetic Resolution (Figure 5a)

Preparative-Scale Reaction of 1h with 2a Using 5 mol% Rh Catalyst

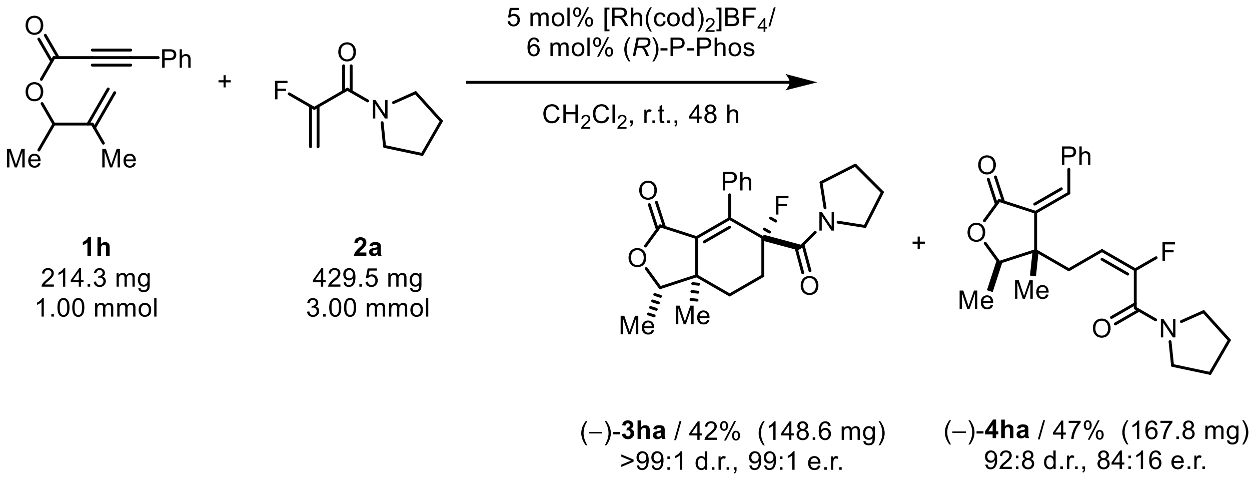

(*R*)-P-Phos (38.7 mg, 0.0600 mmol) and [Rh(cod)$_2$]BF$_4$ (20.5 mg, 0.0500 mmol) were dissolved in CH_2Cl_2 (2.0 mL) in a Schlenk tube, and the mixture was stirred at room temperature for 10 min. After introduction of H_2 and stirring at room temperature for 30 min, the resulting mixture was concentrated to dryness. The residue was dissolved in CH_2Cl_2 (8.0 mL) where a solution of **1h** (21.4 mg, 1.00 mmol) and **2a** (43.0 mg, 3.00 mmol) in CH_2Cl_2 (2.0 mL) was added, and the mixture was stirred at room temperature for 48 h. The resulting mixture was passed through short-path silica gel column chromatography to remove the Rh complex and concentrated. The residue was purified by silica gel column chromatography (eluent: *n*-hexane/EtOAc = 1:1) and silica gel PTLC (eluent: CH_2Cl_2/EtOAc = 10:1) to furnish (–)-**3ha** (148.6 mg, 0.416 mmol, 42% yield, >99:1 d.r., 99:1 e.r.) and (–)-**4ha** [167.8 mg, 0.470 mmol, 47% yield, 92:8 d.r., 84:16 e.r. (major)].

Hydrogenation of (–)-3ha

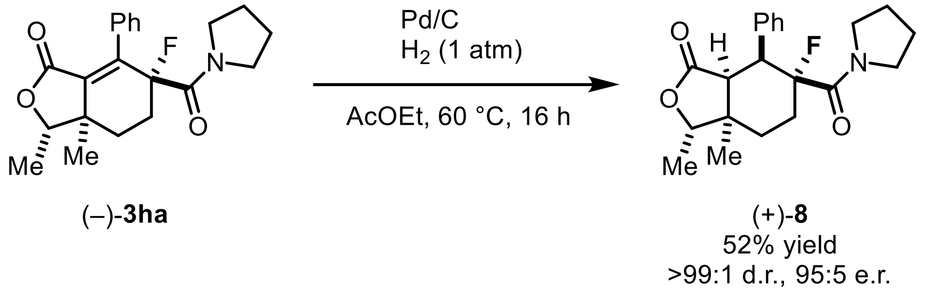

To a solution of (–)-**3ha** (17.9 mg, 0.0500 mmol, >99:1 d.r., 99:1 e.r.) in EtOAc (2.0 mL) was added Pd/C (53.2 mg, 30 wt% Pd) in a Schlenk tube where H_2 (1 atm) was introduced. After stirring at room temperature for 16 h, the reaction mixture was filtered, concentrated, and purified by silica gel PTLC (eluent: CH_2Cl_2/Et_2O = 20:1) to furnish (+)-**8** (9.4 mg, 0.026 mmol, 52% yield, >99:1 d.r., 95:5 e.r.).

(+)-(3*S*,3a*R*,6*R*,7*S*,7a*S*)-6-Fluoro-3,3a-dimethyl-7-phenyl-6-(pyrrolidine-1-carbonyl)-hexahydroisobenzofuran-1(3*H*)-one [(+)-8]

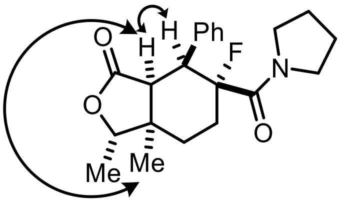

The relative configuration was determined by the NOESY experiment.

White solid; mp 168.1–169.8 °C; $[\alpha]^{25}_{D}$ +1.4 (*c* 0.79, $CHCl_3$, 95:5 e.r.); ^{1}H NMR (400 MHz, $CDCl_3$) δ 7.63–7.60 (m, 2H), 7.32–7.25 (m, 3H), 4.30 (q, *J* = 6.6 Hz, 1H), 3.51–3.41 (m, 2H), 3.35 (dd, *J* = 15.6, 6.1 Hz, 1H), 3.30–3.22 (m, 1H), 2.76 (d, *J* = 6.1 Hz, 1H), 2.69–2.60 (m, 2H), 2.40 (ddt, *J* = 13.9, 7.3, 4.7 Hz, 1H), 1.96 (dddd, *J* = 18.7, 14.0, 12.4, 4.7 Hz, 1H), 1.71–1.58 (m, 4H), 1.53–1.45 (m, 1H), 1.26 (s, 3H), 1.23 (d, *J* = 6.6 Hz, 3H); ^{13}C NMR (101 MHz, $CDCl_3$) δ 173.6, 168.6 (d, *J* = 24.9 Hz), 135.8 (d, *J* = 1.0 Hz), 131.2, 127.9, 127.8, 96.6 (d, *J* = 190.0 Hz), 81.6 (d, *J* = 1.9 Hz), 49.3 (d, *J* = 8.2 Hz), 48.5 (d, *J* = 24.1 Hz), 47.9, 46.7 (d, *J* = 17.1 Hz), 40.8 (d, *J* = 1.1 Hz), 32.8 (d, *J* = 22.7 Hz), 32.2 (d, *J* = 8.1 Hz), 26.9 (d, *J* = 5.1 Hz), 22.9, 20.2, 16.0; ^{19}F NMR (377 MHz, $CDCl_3$) δ –143.1; HRMS (ESI) calcd for $C_{21}H_{26}FNNaO_3$ $[M+Na]^+$ 382.1789, found 382.1772; CHIRALPAK IG-3, *n*-hexane/*i*-PrOH = 70:30, 1.0 mL/min, retention times: 20.5 min (major isomer) and 25.7 min (minor isomer).

Reduction of (–)-4ha with NaBH4

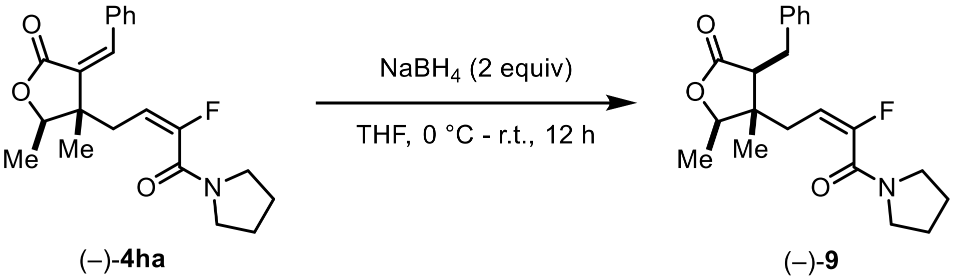

To a solution of (–)-**4ha** (17.9 mg, 0.0500 mmol, 92:8 d.r., 84:16 e.r.) in THF (1.0 mL) was added $NaBH_4$ (3.8 mg, 0.10 mmol) at 0 °C. After stirring at room temperature for 12 h, the reaction was quenched with water at 0 °C and extracted with EtOAc three times. The combined organic layers were washed with brine, dried over Na_2SO_4, filtered, and concentrated under. The residue was purified by silica gel PTLC (eluent: *n*-hexane/EtOAc = 2:1) to furnish (–)-**9** [7.9 mg, 0.022 mmol, 44% yield, 86:14 d.r., 82:18 e.r. (major)].

(–)-(4*S*,5*R*)-3-Benzyl-4-{(*E*)-3-fluoro-4-oxo-4-(pyrrolidin-1-yl)but-2-en-1-yl}-4,5-dimethyldihydrofuran-2(3*H*)-one [(–)-9]

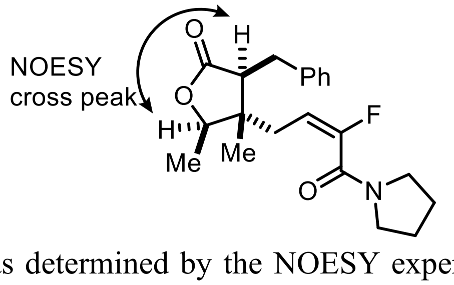

The relative configuration was determined by the NOESY experiment. Two diastereomers of **9** were obtained as a mixture.

The following experimental data were acquired using a mixture of isomers [86:14 d.r., 82:18 e.r. (major)]. White solid; mp 105.9 °C (decomp.); $[\alpha]^{25}_{D}$ –58.6 (*c* 0.79, $CHCl_3$); **major diastereomer:**

^{1}H NMR (400 MHz, $CDCl_3$) δ 7.30–7.29 (m, 4H), 7.24–7.19 (m, 1H), 5.29 (ddd, *J* = 23.2, 9.1, 8.4 Hz, 1H), 4.33 (q, *J* = 6.4 Hz, 1H), 3.50–3.45 (m, 4H), 3.16 (dd, *J* = 14.4, 6.0 Hz, 1H), 2.82 (dd, *J* = 7.5, 6.1 Hz, 1H), 2.75–2.60 (m, 1H), 2.38 (dd, *J* = 14.5, 9.3 Hz, 1H), 2.15 (ddd, *J* = 14.6, 8.3, 1.8 Hz, 1H), 1.94–1.82 (m, 4H), 1.30 (d, *J* = 6.4 Hz, 3H), 1.01 (s, 3H); **partial protons of minor diastereomer:** ^{1}H NMR (400 MHz, $CDCl_3$) δ 5.48 (ddd, *J* = 22.9, 8.7, 7.9 Hz, 1H), 4.47 (q, *J* = 6.7 Hz, 1H), 3.57–3.52 (m, 4H), 2.52 (ddd, *J* = 15.0, 7.8, 1.9 Hz, 1H), 1.24 (d, *J* = 6.7 Hz, 3H), 0.84 (s, 3H); ^{13}C NMR (101 MHz, $CDCl_3$) δ 177.3, 177.2, 159.7 (d, *J* = 32.3 Hz), 152.4 (d, *J* = 264.8 Hz), 139.3, 139.0, 128.8, 128.6, 126.6, 126.5, 111.3 (d, *J* = 19.0 Hz), 80.6, 80.5, 51.0, 50.0, 47.2 (d, *J* = 10.4 Hz), 46.3, 44.8, 31.4 (d, *J* = 6.1 Hz), 31.0, 26.2 (d, *J* = 3.4 Hz), 23.74, 23.65, 20.7, 15.9, 14.8, 13.9; ^{19}F NMR (377 MHz, $CDCl_3$) δ –110.8 (major), –110.9 (minor); HRMS (ESI) calcd for $C_{21}H_{26}FNNaO_3$ $[M+Na]^+$ 382.1789, found 382.1799; CHIRALPAK ID-3, *n*-hexane/*i*-PrOH = 70:30, 1.0 mL/min, retention times: 19.7 min (major isomer) and 16.5 min (minor isomer).

Reduction of (–)-4ha with DIBAL

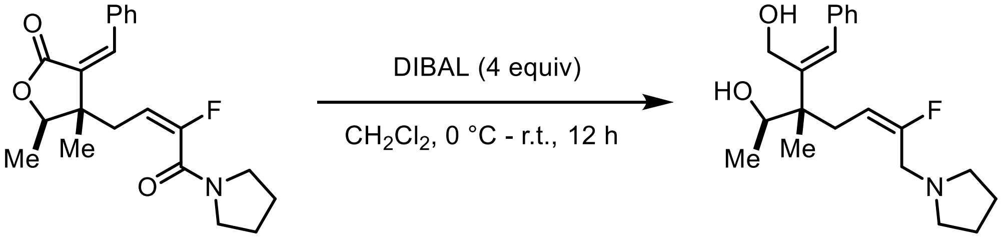

(–)-**4ha**

(–)-**10**
70% yield
>99:1 d.r., 82:18 e.r.

To a solution of (–)-**4ha** (17.9 mg, 0.0500 mmol, 92:8 d.r., 84:16 e.r.) in CH_2Cl_2 (2.0 mL) was added DIBAL (1M in *n*-hexane, 0.20 mL, 0.20 mmol) at 0 °C. After stirring at room temperature for 12 h, the reaction was quenched with aqueous Rochelle salt at 0 °C and extracted with CH_2Cl_2 three times. The combined organic layers were washed with brine, dried over Na_2SO_4, filtered, and concentrated. The residue was purified by silica gel PTLC (eluent: EtOAc) to furnish (–)-**10** (12.2 mg, 0.0351 mmol, 70% yield, >99:1 d.r., 82:18 e.r.).

(–)-(3*S*,4*R*)-2-(*Z*)-Benzylidene-3-{(*E*)-3-fluoro-4-(pyrrolidin-1-yl)but-2-en-1-yl}-3-methylpentane-1,4-diol [(–)-10]

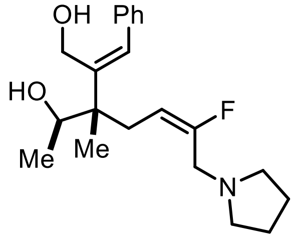

Colorless oil; $[\alpha]^{25}_{D}$ –25.3 (*c* 1.22, $CHCl_3$, 82:18 e.r.); ^{1}H NMR (400 MHz, $CDCl_3$) δ 7.43 (d, *J* = 7.1 Hz, 2H), 7.37–7.32 (m, 2H), 7.26–7.22 (m, 1H), 6.65 (s, 1H), 5.39 (ddd, *J* = 20.5, 12.2, 5.1 Hz, 1H), 4.20 (d, *J* = 12.3 Hz, 1H), 4.14 (d, *J* = 12.3 Hz, 1H), 3.90 (dd, *J* = 29.7, 13.5 Hz, 1H), 3.64 (q, *J* = 6.4 Hz, 1H), 2.99–2.92 (m, 1H), 2.75–2.64 (m, 5H), 1.88–1.77 (m, 6H), 1.18 (s, 3H), 1.11 (d, *J* = 6.5 Hz, 3H); ^{13}C NMR (101 MHz, $CDCl_3$) δ 158.8 (d, *J* = 249.7 Hz), 143.3, 137.9, 132.0, 129.3, 128.1, 126.7, 106.3 (d, *J* = 21.1 Hz), 70.5, 57.3, 53.9, 52.5 (d, *J* = 31.9 Hz), 48.1 (d, *J* = 2.2 Hz), 32.7 (d, *J* = 7.7 Hz), 23.3, 22.0, 18.0; ^{19}F NMR (377 MHz, $CDCl_3$) δ –95.0; HRMS (ESI) calcd for $C_{21}H_{31}FNO_2$ $[M+H]^+$ 348.2333, found 348.2337; CHIRALPAK AD-H, *n*-hexane/*i*-PrOH = 95:5, 1.0 mL/min, retention times: 8.9 min (major isomer) and 7.0 min (minor isomer).

2.6.2. Synthetic Applications for Desymmetrization (Figure 5b)

Preparative-Scale Reaction of 5a with 2a Using 5 mol% Rh Catalyst

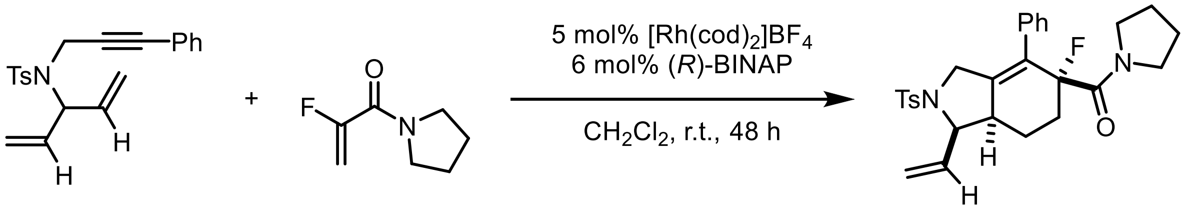

5a
351.5 mg
1.00 mmol

2a
171.8 mg
1.20 mmol

(–)-**6aa** / 76% (377.1 mg)
94:6 d.r., >99:1 e.r.

(*R*)-BINAP (37.5 mg, 0.0600 mmol) and [Rh(cod)$_2$]BF$_4$ (20.5 mg, 0.0500 mmol) were dissolved in CH_2Cl_2 (2.0 mL) in a Schlenk tube, and the mixture was stirred at room temperature for 10 min. After introduction of H_2 and stirring at room temperature for 30 min, the resulting mixture was concentrated to dryness. The residue was dissolved in CH_2Cl_2 (8.0 mL), followed by the addition of a solution of **5a** (351.5 mg, 1.00 mmol) and **2a** (171.8 mg, 1.20 mmol) in CH_2Cl_2 (2.0 mL). The mixture was stirred at room temperature for 48 h, then passed through short-path silica gel column chromatography to remove the Rh complex and concentrated. The crude product was further by silica gel column chromatography (eluent: *n*-hexane/EtOAc = 1:1) and silica gel PTLC (eluent: CH_2Cl_2/EtOAc = 10:1) to furnish (–)-**6aa** [377.1 mg, 0.762 mmol, 76% yield, 94:6 d.r., >99:1 e.r. (major)].

Detosylation of (–)-6aa Followed by Acylation

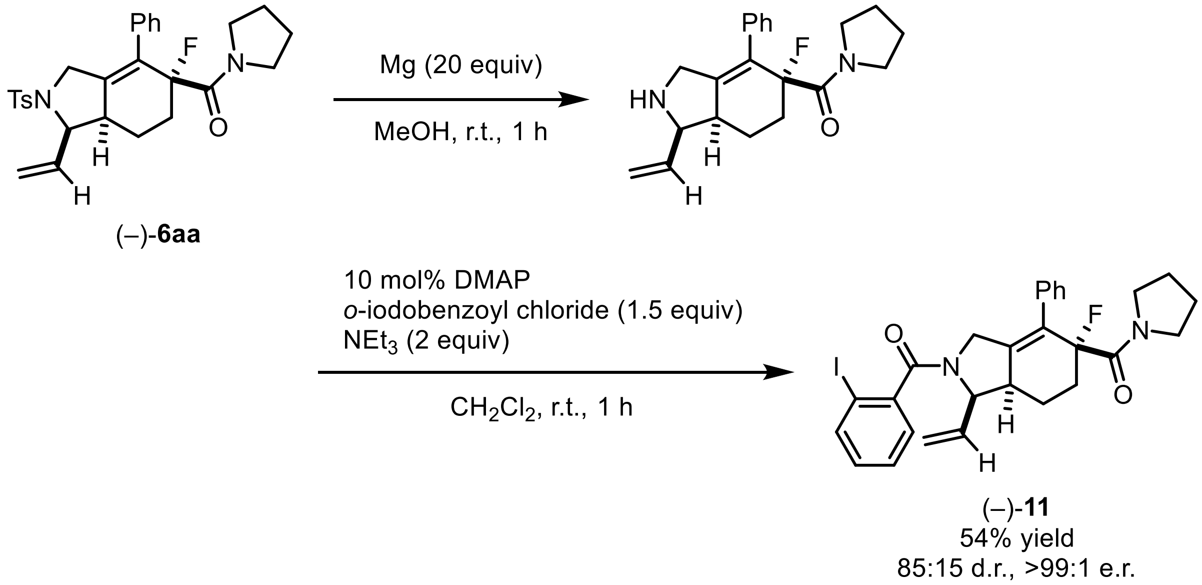

(–)-**11**
54% yield
85:15 d.r., >99:1 e.r.

To a solution of (–)-**6aa** (49.5 mg, 0.100 mmol, 94:6 d.r., >99:1 e.r.) in MeOH (4.0 mL) was added Mg powder (48.0 mg, 2.00 mmol) at room temperature. After sonication for 1 h, the reaction was quenched with aqueous NH_4Cl and extracted with EtOAc three times. The combined organic layers were washed with brine, dried over Na_2SO_4, filtered, and concentrated. The crude amine was used in the following reaction without further purification.

To a solution of the crude amine, NEt_3 (0.28 mL, 0.20 mmol), and DMAP (1.2 mg, 0.010 mmol) in CH_2Cl_2 (5.0 mL) was added *o*-iodobenzoyl chloride (40.0 mg, 0.150 mmol) at room temperature. After stirring at room temperature for 16 h, the reaction was quenched with aqueous NH_4Cl and extracted with CH_2Cl_2 three times. The combined organic layers were washed with brine, dried over Na_2SO_4, filtered, and concentrated. The residue was purified by silica gel PTLC (eluent: eluent: *n*-hexane/EtOAc = 1:2) to furnish (–)-**11** [31.0 mg, 0.0543 mmol, 54% yield, 85:15 d.r., >99:1 e.r.

(major)].

(–)-{(1*R*,5*R*,7a*R*)-5-Fluoro-2-(2-iodobenzoyl)-4-phenyl-1-vinyl-2,3,5,6,7,7a-hexahydro-1*H*-isoindol-5-yl}(pyrrolidin-1-yl)methanone [(–)-11]

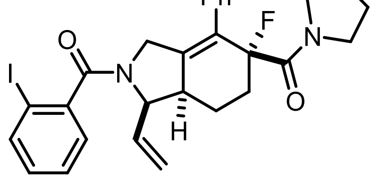

Two diastereomers of **11** were obtained as a mixture. The following experimental data were acquired using a mixture of isomers [85:15 d.r., >99:1 e.r. (major)]. Colorless oil; $[\alpha]^{25}_{D}$ –5.9 (*c* 1.55, $CHCl_3$); **major diastereomer:** ^1H NMR (400 MHz, $CDCl_3$) δ 7.81 (dd, *J* = 8.0, 0.8 Hz, 1H), 7.34–7.28 (m, 4H), 7.17–7.13 (m, 3H), 7.06 (ddd, *J* = 7.9, 7.5, 1.7 Hz, 1H), 5.68 (ddd, *J* = 17.0, 10.2, 8.2 Hz, 1H), 5.06 (d, *J* = 10.3 Hz, 1H), 4.53 (d, *J* = 17.0 Hz, 1H), 4.38 (ddd, *J* = 17.8, 7.8, 1.6 Hz, 1H), 4.09 (t, *J* = 7.9 Hz, 1H), 3.88 (ddd, *J* = 17.8, 5.3, 1.2 Hz, 1H), 3.50–3.36 (m, 3H), 3.23–3.15 (m, 1H), 3.01–2.90 (m, 1H), 2.47–2.41 (m, 1H), 2.20–1.98 (m, 2H), 1.73–1.59 (m, 4H), 1.58–1.49 (m, 1H); **partial protons of minor diastereomer:** ^1H NMR (400 MHz, $CDCl_3$) δ 7.73 (d, *J* = 7.9 Hz, 1H), 6.99 (ddd, *J* = 7.9, 7.5, 1.7 Hz, 1H), 5.84 (ddd, *J* = 17.0, 10.1, 8.2 Hz, 1H), 5.42 (d, *J* = 16.7 Hz, 1H), 5.34 (d, *J* = 10.8 Hz, 1H), 5.13 (t, *J* = 8.1 Hz, 1H), 1.82–1.75 (m, 4H); ^{13}C NMR (101 MHz, $CDCl_3$) δ 169.3 (d, *J* = 27.2 Hz), 169.0, 168.4, 142.7, 142.6, 141.6 (d, *J* = 6.5 Hz), 141.2 (d, *J* = 6.4 Hz), 139.1, 138.7, 136.2, 136.1, 133.5, 132.6, 132.3 (d, *J* = 20.8 Hz), 132.0 (d, *J* = 20.5 Hz), 130.24, 130.16, 129.1, 128.5, 128.3, 128.2, 127.84, 127.75, 127.6, 126.8, 118.7, 117.7, 96.6 (d, *J* = 186.1 Hz), 96.4 (d, *J* = 186.0 Hz), 92.6, 91.9, 65.5, 62.0, 49.8, 47.94, 47.85, 47.8, 46.9 (d, *J* = 17.0 Hz), 46.8 (d, *J* = 16.9 Hz), 43.5 (d, *J* = 2.3 Hz), 42.2 (d, *J* = 2.5 Hz), 34.1 (d, *J* = 23.2 Hz), 34.0 (d, *J* = 23.1 Hz), 26.6 (d, *J* = 5.4 Hz), 22.9, 22.0 (d, *J* = 7.1 Hz), 21.7 (d, *J* = 7.1 Hz); ^{19}F NMR (377 MHz, $CDCl_3$) δ –145.1 (major), –145.5 (minor); HRMS (ESI) calcd for $C_{28}H_{28}FIN_2NaO_2$ $[M+Na]^+$ 593.1072, found 593.1084; CHIRALPAK ID-3, *n*-hexane/*i*-PrOH = 70:30, 1.0 mL/min, retention times: 16.6 min (major isomer) and 27.1 min (minor isomer).

Intramolecular Heck Reaction of (–)-11

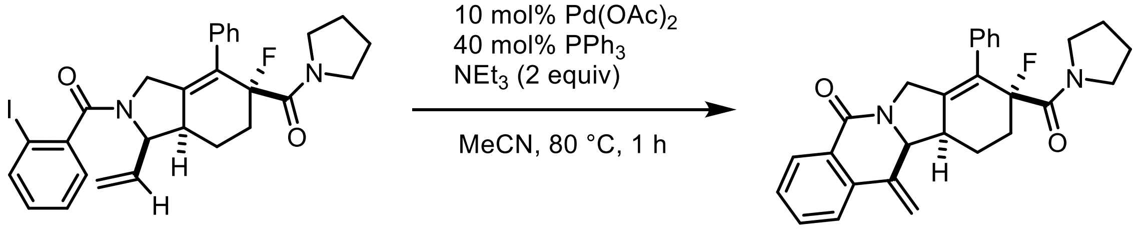

(–)-**11**

(–)-**12**
79% yield
>99:1 d.r., >99 e.r.

To a solution of (–)-**11** (31.0 mg, 0.0540 mmol, 85:15 d.r., >99:1 e.r.), $Pd(OAc)_2$ (1.2 mg, 0.0050 mmol), and PPh_3 (5.2 mg, 0.020 mmol) in MeCN (2.0 mL) was added NEt_3 (11.0 mg, 0.109 mmol) at room temperature. After stirring at 80 °C for 1 h, the reaction was allowed to cool to room temperature. The reaction mixture was filtered and concentrated. The residue was purified by silica gel PTLC (eluent: eluent: *n*-hexane/EtOAc = 1:1) to furnish (–)-**12** (19.0 mg, 0.0429 mmol, 79% yield, >99:1 d.r., >99:1 e.r.).

(–)-(9*R*,11a*R*,11b*S*)-9-Fluoro-12-methylene-8-phenyl-9-(pyrrolidine-1-carbonyl)-9,10,11,11a,11b,12-hexahydroisoindolo[2,1-b]isoquinolin-5(7*H*)-one [(–)-12]

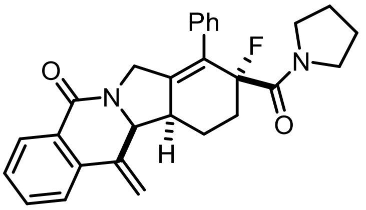

White solid; mp 173.7 °C (decomp.); $[\alpha]^{25}_{D}$ –229.3 (*c* 0.95, $CHCl_3$, >99:1 e.r.); ^1H NMR (400 MHz, $CDCl_3$) δ 8.13 (dd, *J* = 7.8, 1.1 Hz, 1H), 7.70 (dd, *J* = 8.0, 0.7 Hz, 1H), 7.47 (ddd, *J* = 7.9, 7.3, 1.5 Hz, 1H), 7.37 (dt, *J* = 11.4, 1.1 Hz, 1H), 7.33–7.25 (m, 3H), 7.14–7.11 (m, 2H), 6.06 (d, *J* = 2.7 Hz, 1H), 5.29 (d, *J* = 2.5 Hz, 1H), 5.06 (dt, *J* = 8.7, 2.5 Hz, 1H), 4.74 (dd, *J* = 15.3, 3.4 Hz, 1H), 3.71 (ddd, *J* = 15.4, 9.8, 1.6 Hz, 1H), 3.37–3.21 (m, 3H), 3.14 (dt, *J* = 12.3, 7.7 Hz, 1H), 2.95–2.87 (m, 1H), 2.72 (dddd, *J* = 15.5, 14.3, 6.0, 2.9 Hz, 1H), 2.29–2.23 (m, 1H), 2.07 (dddd, *J* = 25.4, 14.2, 11.2, 5.4 Hz, 1H), 1.67–1.47 (m, 4H), 1.11–1.00 (m, 1H); ^{13}C NMR (101 MHz, $CDCl_3$) δ 168.8 (d, *J* = 26.2 Hz), 161.2, 145.4 (d, *J* = 6.7 Hz), 136.6, 135.7, 134.3, 132.1, 131.5 (d, *J* = 17.0 Hz), 129.1 (d, *J* = 1.2 Hz), 128.7, 128.5, 128.2, 127.8, 126.5, 122.4, 112.2, 97.0 (d, *J* = 186.5 Hz), 61.6, 48.6 (d, *J* = 2.4 Hz), 47.4, 47.0 (d, *J* = 15.0 Hz), 43.9 (d, *J* = 1.6 Hz), 34.3 (d, *J* = 25.9 Hz), 26.3 (d, *J* = 4.7 Hz), 24.4 (d, *J* = 4.4 Hz), 22.8; ^{19}F NMR (377 MHz, $CDCl_3$) δ –134.1; HRMS (ESI) calcd for $C_{28}H_{27}FN_2NaO_2$ $[M+Na]^+$ 465.1949, found 465.1949; CHIRALPAK IF-3, *n*-hexane/*i*-PrOH = 70:30, 1.0 mL/min, retention times: 14.0 min (major isomer) and 24.6 min (minor isomer).

2.7. Experimental Mechanistic Studies

2.7.1 Deuterium-Labeling Studies (Figure 5c)

Reaction of 1,6-Enyne 1d with α-Fluoroacrylamide-D$_2$

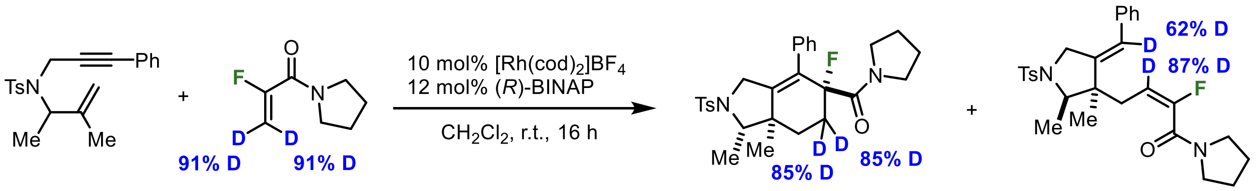

1d | **2a**-D$_2$ (1.2 equiv) | (–)-**3da**-D$_2$ 18%, 88:12 d.r., 98:2 e.r. | (–)-**4da**-D$_2$ 6%, 63:37 d.r., 97:3 e.r.

(*R*)-BINAP (3.7 mg, 0.0060 mmol) and [Rh(cod)$_2$]BF$_4$ (2.1 mg, 0.0050 mmol) were dissolved in CH_2Cl_2 (2.0 mL) in a Schlenk tube, and the mixture was stirred at room temperature for 10 min. After introduction of H_2 and stirring at room temperature for 30 min, the resulting mixture was concentrated to dryness. The residue was dissolved in CH_2Cl_2 (0.5 mL) where a solution of **1d** (17.7 mg, 0.0500 mmol) and **2a**-D$_2$ (1 M in CH_2Cl_2, 0.60 mL, 0.060 mmol) was added, and the mixture was stirred at room temperature for 16 h. The resulting mixture was passed through short-path silica gel column chromatography to remove the Rh complex and concentrated. The residue was purified by silica gel PTLC twice (eluent: *n*-hexane/EtOAc = 1:1 and CH_2Cl_2/EtOAc = 10:1) to furnish **3da**-D$_2$ [4.4 mg, 0.0088 mmol, 18% yield, >99:1 d.r., 98:2 e.r. (major)] and **4da-**D$_2$ [1.6 mg, 0.0032 mmol, 6% yield, 63:37 d.r., 97:3 e.r. (major)]. Two diastereomers of **4da**-D$_2$ were obtained as a mixture. A trace amount (ca. <2%) of another diastereomer of **3da**-D$_2$ was detected in a crude reaction mixture.

(–)-{(1*S*,5*R*,7a*R*)-5-Fluoro-1,7a-dimethyl-2-(4-methylphenyl)-4-phenyl-2,3,5,6,7,7a-hexahydro-1*H*-isoindol-5-yl-6,6-*d*$_2$}(pyrrolidin-1-yl)methanone [(–)-3da-D$_2$ (major diastereomer)]

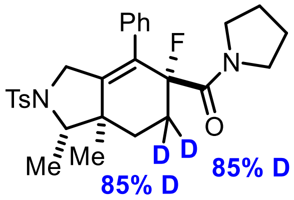

White solid; mp 102.4–103.9 °C; $[\alpha]^{25}_{D}$ –17.2 (*c* 0.44, $CHCl_3$, 98:2 e.r.); ^{1}H NMR (400 MHz, $CDCl_3$) δ 7.52 (d, *J* = 8.3 Hz, 2H), 7.29–7.25 (m, 5H), 6.88–6.86 (m, 2H), 3.95 (dd, *J* = 15.7, 8.7 Hz, 1H), 3.43 (dd, *J* = 15.7, 6.1 Hz, 1H), 3.38–3.32 (m, 1H), 3.30–3.20 (m, 2H), 2.83 (q, *J* = 6.4 Hz, 1H), 2.73–2.65 (m, 1H), 2.46 (s, 3H), 2.32–2.30 (m, 0.15H), 2.19–2.08 (m, 0.15H), 1.65–1.53 (m, 6H), 1.38 (d, *J* = 6.4 Hz, 3H), 1.23 (s, 3H); ^{13}C NMR (101 MHz, $CDCl_3$) δ 169.4 (d, *J* = 27.4 Hz), 145.2 (d, *J* = 6.7 Hz), 143.6, 136.1, 133.2, 129.8, 129.214 (d, *J* = 19.6 Hz), 129.209 (d, *J* = 1.3 Hz), 128.2, 127.8, 127.5, 96.2 (d, *J* = 186.2 Hz), 66.2, 51.1 (d, *J* = 1.5 Hz), 47.7, 46.8 (d, *J* = 17.2 Hz), 43.8 (d, *J* = 2.3 Hz), 31.7–31.6 (m, 2C), 26.5 (d, *J* = 5.5 Hz), 22.9, 21.6, 18.3 (d, *J* = 3.3 Hz), 14.5; HRMS (ESI) calcd for $C_{28}H_{31}D_2FN_2NaO_3S$ $[M+Na]^+$ 521.2214, found 521.2212; CHIRALPAK AD-H, *n*-hexane/*i*-PrOH = 95:5, 1.0 mL/min, retention times: 32.7 min (major isomer) and 25.1 min (minor isomer).

(–)-(*E*)-4-{(2*S*,3*R*,*Z*)-2,3-Dimethyl-1-(4-methylphenyl)-4-(phenylmethylene-*d*)pyrrolidin-3-yl}-2-fluoro-1-(pyrrolidin-1-yl)but-2-en-1-one-3-*d* [(–)-4da-D$_2$]

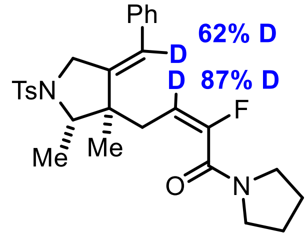

The following experimental data were acquired using a mixture of isomers [63:37 d.r., 97:3 e.r. (major)]. Colorless oil; $[\alpha]^{25}_{D}$ –6.9 (*c* 0.16, $CHCl_3$); **major diastereomer:** ^1H NMR (400 MHz, $CDCl_3$) δ 7.76 (d, *J* = 8.3 Hz, 2H), 7.37–7.33 (m, 2H), 7.30–7.26 (m, 2H), 7.25–7.22 (m, 1H), 7.17–7.13 (m, 2H), 6.19 (t, *J* = 2.3 Hz, 0.38H), 5.32 (dt, *J* = 23.4, 8.4 Hz, 0.13H), 4.27 (d, *J* = 14.6 Hz, 1H), 4.22 (d, *J* = 14.6 Hz, 1H), 3.76–3.65 (m, 1H), 3.48–3.41 (m, 4H), 2.40 (s, 3H), 2.29–2.20 (m, 2H), 1.86–1.75 (m, 4H), 1.10 (s, 3H), 1.08 (d, *J* = 6.5 Hz, 3H); **partial protons of minor diastereomer:** ^1H NMR (400 MHz, $CDCl_3$) δ 7.70 (d, *J* = 8.2 Hz, 2H), 6.17 (t, *J* = 2.5 Hz, 0.38H), 5.55 (dt, *J* = 24.0, 7.8 Hz, 0.13H), 4.37 (d, *J* = 15.0 Hz, 1H), 4.15 (d, *J* = 15.0 Hz, 1H), 3.22 (q, *J* = 6.5 Hz, 1H), 2.69 (d, *J* = 14.8 Hz, 1H), 2.51 (d, *J* = 15.0 Hz, 1H), 1.26 (d, *J* = 6.4 Hz, 3H), 0.95 (s, 3H); HRMS (ESI) calcd for $C_{28}H_{31}D_2FN_2NaO_3S$ $[M+Na]^+$ 521.2214, found 521.2212; CHIRALPAK IF-3, *n*-hexane/*i*-PrOH = 90:10, 1.0 mL/min, retention times: 51.9 min (major isomer) and 42.4 min (minor isomer).

Reaction of 1,6-Enyne 1h with α-Fluoroacrylamide-D$_2$

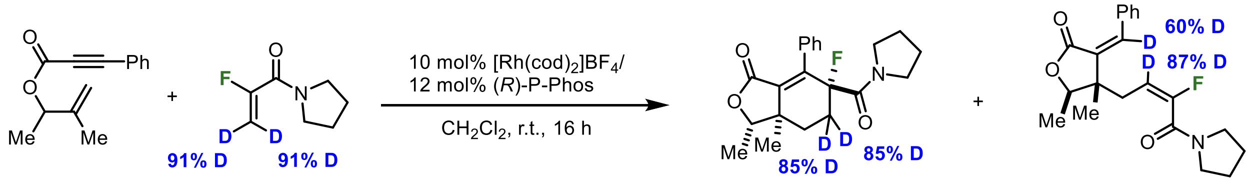

1h | **2a**-D$_2$ (3 equiv) | (–)-**3ha**-D$_2$ 26%, >99:1 d.r., 93:7 e.r. | (+)-**4ha**-D$_2$ 16%, >99:1 d.r., 92:8 e.r.

(*R*)-P-Phos (3.9 mg, 0.0060 mmol) and $[Rh(cod)_2]BF_4$ (2.1 mg, 0.0050 mmol) were dissolved in CH_2Cl_2 (2.0 mL) in a Schlenk tube, and the mixture was stirred at room temperature for 10 min. After introduction of H_2 and stirring at room temperature for 30 min, the resulting mixture was concentrated to dryness. The residue was dissolved in CH_2Cl_2 (0.5 mL) where a solution of **1h** (10.7 mg, 0.0500 mmol) and **2a**-D$_2$ (1 M in CH_2Cl_2, 1.50 mL, 0.150 mmol) was added, and the mixture was stirred at room temperature for 16 h. The resulting mixture was passed through short-path silica gel column chromatography to remove the Rh complex and concentrated. The residue was purified by silica gel PTLC twice (eluent: *n*-hexane/EtOAc = 1:1 and CH_2Cl_2/EtOAc = 10:1) to furnish **3ha**-D$_2$ (4.6 mg, 0.013 mmol, 26% yield, >99:1 d.r., 93:7 e.r.) and **4ha**-D$_2$ (2.9 mg, 0.0082 mmol, 16% yield, >99:1 d.r., 92:8 e.r.).

(–)-(3*S*,3a*R*,6*R*)-6-Fluoro-3,3a-dimethyl-7-phenyl-6-(pyrrolidine-1-carbonyl)-3a,4,5,6-tetrahydroisobenzofuran-1(3*H*)-one-5,5-*d*$_2$ [(–)-3ha-D$_2$]

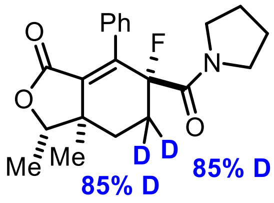

Colorless oil; $[\alpha]^{25}_{D}$ –116.6 (*c* 0.46, $CHCl_3$, 93:7 e.r.); ^1H NMR (400 MHz, $CDCl_3$) δ 7.34–7.30 (m, 3H), 7.16–7.13 (m, 2H), 4.30 (q, *J* = 6.5 Hz, 1H), 3.43–3.28 (m, 3H), 2.87–2.80 (m, 1H), 2.44–2.39 (m, 1.15H), 2.31–2.21 (m, 0.15H), 1.79 (d, *J* = 13.5 Hz, 1H), 1.72–1.54 (m, 3H), 1.51–1.43 (m,

1H), 1.35 (d, J = 6.5 Hz, 3H), 1.25 (s, 3H); ^{13}C NMR (101 MHz, $CDCl_3$) δ 168.3 (d, J = 25.2 Hz), 167.5, 142.0 (d, J = 19.4 Hz), 137.7 (d, J = 5.3 Hz), 133.5, 128.9, 128.2, 127.7, 96.3 (d, J = 191.5 Hz), 83.2, 47.8, 46.7 (d, J = 16.9 Hz), 42.4 (d, J = 1.9 Hz), 31.0–30.8 (m, 2C), 26.6 (d, J = 5.4 Hz), 22.8, 18.4 (d, J = 3.0 Hz), 13.6; HRMS (ESI) calcd for $C_{21}H_{22}D_2FNNaO_3$ $[M+Na]^+$ 382.1758, found 382.1775; CHIRALPAK AD-H, *n*-hexane/*i*-PrOH = 90:10, 1.0 mL/min, retention times: 9.7 min (major isomer) and 20.5 min (minor isomer).

(+)-(4*R*,5*S*,*Z*)-4-{(*E*)-3-Fluoro-4-oxo-4-(pyrrolidin-1-yl)but-2-en-1-yl-2-*d*}-4,5-dimethyl-3-(phenylmethylene-*d*)dihydrofuran-2(3*H*)-one [(+)-4ha-D$_2$]

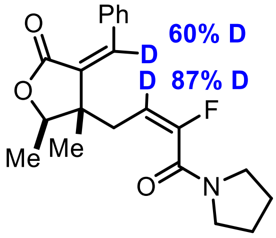

Colorless oil; $[\alpha]^{25}_D$ +0.6 (c 0.29, $CHCl_3$, 92:8 e.r.); ^{1}H NMR (400 MHz, $CDCl_3$) δ 7.88–7.85 (m, 3H), 7.40–7.34 (m, 2H), 6.73 (s, 0.40H), 5.54 (dt, J = 22.9, 8.5 Hz, 0.13H), 4.49 (q, J = 6.5 Hz, 1H), 3.51–3.39 (m, 4H), 2.75 (d, J = 14.6 Hz, 1H), 2.68 (d, J = 14.5 Hz, 1H), 1.84–1.65 (m, 4H), 1.32 (d, J = 6.5 Hz, 3H), 1.23 (s, 3H); ^{13}C NMR (101 MHz, $CDCl_3$) δ 168.6, 160.0 (d, J = 32.2 Hz), 153.1 (d, J = 265.2 Hz), 139.3, 133.3 (d, J = 5.6 Hz), 132.4 (d, J = 8.9 Hz), 130.9 (d, J = 2.7 Hz), 129.7 (d, J = 2.4 Hz), 128.2, 111.1–110.6 (m), 80.2, 47.5 (d, J = 1.5 Hz), 47.1 (d, J = 10.1 Hz), 46.3, 34.9 (d, J = 6.0 Hz), 26.0 (d, J = 3.3 Hz), 23.6, 21.5, 16.7; HRMS (ESI) calcd for $C_{21}H_{22}D_2FNNaO_3$ $[M+Na]^+$ 382.1758, found 382.1772; CHIRALPAK IG-3, *n*-hexane/*i*-PrOH = 90:10, 1.0 mL/min, retention times: 60.9 min (major isomer) and 52.7 min (minor isomer).

3. Single-Crystal X-Ray Diffraction Analysis

Single crystal X-ray diffraction data for **3ga**, **4ga, 3ia** and **6dk** were collected using a XtaLAB Mini II diffractometer equipped with a Hybrid Pixel Array detector using graphite monochromatized Mo-Kα (0.71073 Å) radiation. Data for (±)-**4ia** and **6aa** were collected using a Rigaku XtaLAB Synergy diffractometer equipped with a HyPix-6000HE Hybrid Photon Counting (HPC) detector with graphite-monochromatized Mo-Kα (0.71073 Å) radiation. The initial structure was solved by an intrinsic phasing method using SHELXT-2018/2[23] software and refined by a full matrix least-squares method using SHELXL-2018/3[24] software. All hydrogen atoms were located at geometrically calculated positions and included in least-squares calculations using riding models.

Details of the crystal data and the summaries of the intensity data collection parameters for **3ga**, **4ga**, **3ia**, (±)-**4ia**, **6aa** and **6dk** are listed in Tables S3–S8 and structures are shown in Figures S2–S7. Single crystals suitable for X-ray analyses were grown by vapor diffusion of a Et_2O/*n*-hexane solution of **3ga**, **3ia** and **6aa**, a Et_2O/CH_2Cl_2/*n*-hexane solution of **4ga** and (±)-**4ia** and a THF/*n*-hexane solution of **6dk**.

Crystallographic data have been, deposited with the Cambridge Crystallographic Data Centre: Deposition code CCDC 2480941 (**3ga**), CCDC 2480945 (**4ga**), CCDC 2480942 (**3ia**), CCDC 2480939 [(±)-**4ia**], CCDC 2480946 (**6aa**) and CCDC 2480943 (**6dk**). They contain the supplementary crystallographic data for this paper. The data can be obtained free of charge from The Cambridge Crystallographic Data Centre via www.ccdc.cam.ac.uk/structures.

Table S3. Crystal data and data collection parameters of (3*R*,5*R*,7a*S*)-(–)-**3ga**.

Empirical formula	$C_{32}H_{33}FN_2O_3S$
Formula weight	544.66
Temperature/K	298.15
Crystal system	orthorhombic
Space group	$P2_12_12_1$
a/Å	9.6437(3)
b/Å	11.1387(4)
c/Å	26.8019(9)
α /°	90
β /°	90
γ /°	90
V/Å^3	2879.01(17)
Z	4
ρ_{calc}/g cm^{-3}	1.257
μ/mm^{-1}	0.154
F(000)	1152
Crystal size/mm^3	0.2 × 0.2 × 0.04
Radiation	Mo Kα (λ = 0.71073)
2θ range for data collection/°	4.488 to 61.23
Index ranges	$-13 \leq h \leq 12$, $-15 \leq k \leq 15$, $-38 \leq l \leq 37$
Reflections collected	29631
Independent reflections	8382 [R_{int} = 0.0435, R_{sigma} = 0.0717]
Data/restraints/parameters	8382/0/353
Goodness-of-fit on F^2	1.081
Final R indexes [$I \geq 2\sigma(I)$]	R_1 = 0.0772, wR_2 = 0.1136
Final R indexes [all data]	R_1 = 0.1307, wR_2 = 0.1276
Largest diff. peak/hole.e. Å^{-3}	0.23/-0.32
Absolute structure parameter	-0.04(3)

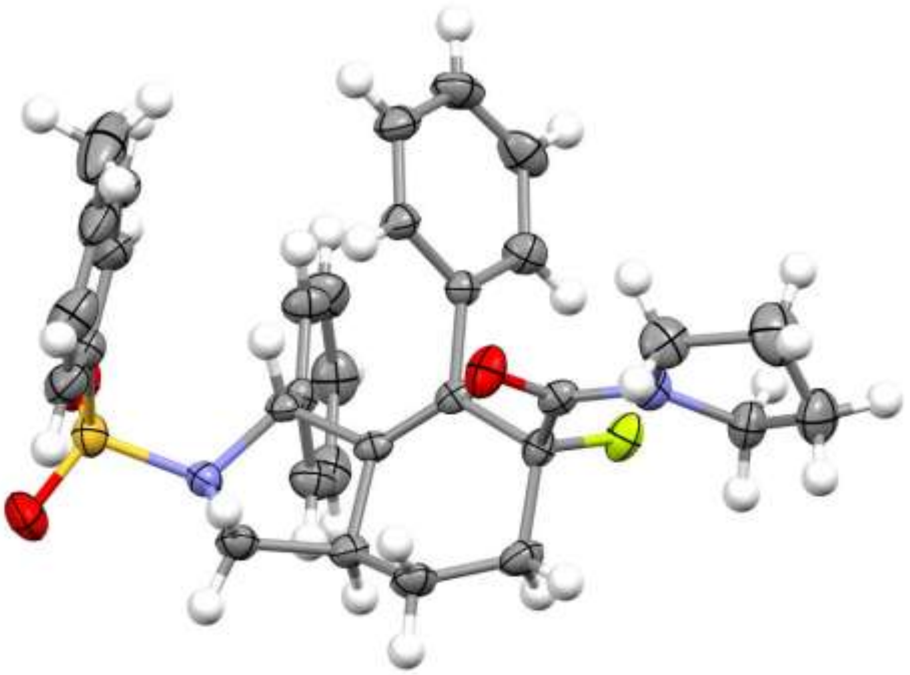

Figure S2. X-ray crystal structure of (3*R*,5*R*,7a*S*)-(–)-**3ga**, showing thermal ellipsoids at the 50% probability level.

Table S4. Crystal data and data collection parameters of (3*R*,5*S*)-(+)-**4ga**.

Empirical formula	$C_{32}H_{33}FN_2O_3S$
Formula weight	544.66
Temperature/K	298.15
Crystal system	orthorhombic
Space group	$P2_12_12_1$
a/Å	12.9217(3)
b/Å	13.6593(3)
c/Å	16.1068(4)
α /°	90
β /°	90
γ /°	90
V/Å^3	2842.87(12)
Z	4
ρ_{calc}/g cm^{-3}	1.273
μ/mm^{-1}	0.156
F(000)	1152
Crystal size/mm^3	0.5 × 0.3 × 0.1
Radiation	Mo Kα (λ = 0.71073)
2θ range for data collection/°	5.022 to 61.042
Index ranges	$-18 \leq h \leq 18$, $-19 \leq k \leq 19$, $-23 \leq l \leq 22$
Reflections collected	28110
Independent reflections	8387 [R_{int} = 0.0246, R_{sigma} = 0.0292]
Data/restraints/parameters	8387/0/353
Goodness-of-fit on F^2	1.018
Final R indexes [$I \geq 2\sigma(I)$]	R_1 = 0.0423, wR_2 = 0.0959
Final R indexes [all data]	R_1 = 0.0600, wR_2 = 0.1031
Largest diff. peak/hole.e. Å^{-3}	0.24/-0.19
Absolute structure parameter	-0.012(19)

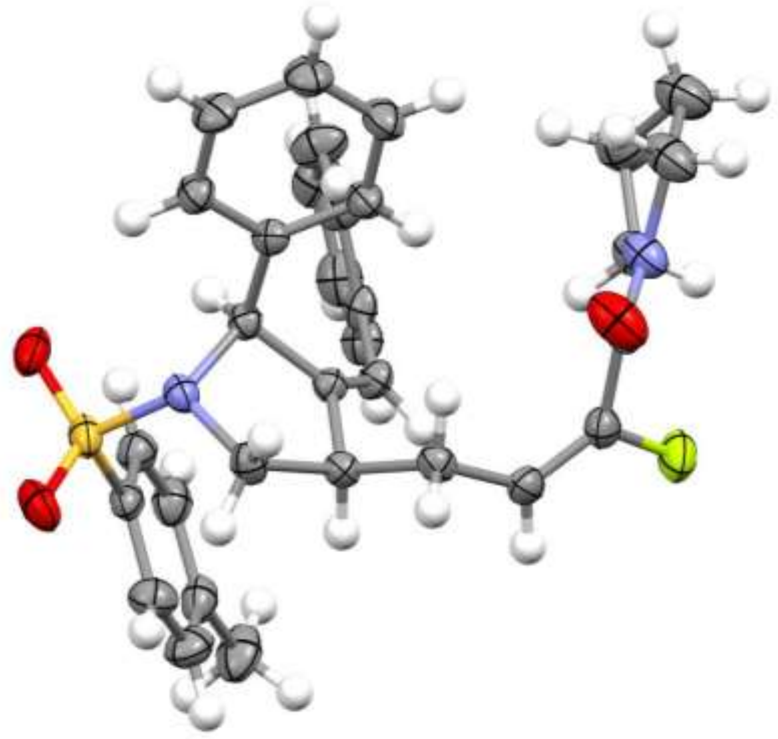

Figure S3. X-ray crystal structure of (3*R*,5*S*)-(+)-**4ga**, showing thermal ellipsoids at the 50% probability level.

Table S5. Crystal data and data collection parameters of (3*S*,3a*R*,6*R*)-(–)-**3ia**.

Empirical formula	$C_{21}H_{23}BrFNO_3$
Formula weight	436.31
Temperature/K	298
Crystal system	orthorhombic
Space group	$P2_12_12_1$
a/Å	9.1217(3)
b/Å	12.6660(5)
c/Å	17.2201(5)
α /°	90
β /°	90
γ /°	90
V/Å^3	1989.53(12)
Z	4
ρ_{calc}/g cm^{-3}	1.457
μ/mm^{-1}	2.096
F(000)	896
Crystal size/mm^3	0.56 × 0.37 × 0.35
Radiation	Mo Kα (= 0.71073)
2θ range for data collection/°	5.054 to 61.004
Index ranges	$-12 \leq h \leq 13$, $-17 \leq k \leq 17$, $-24 \leq l \leq 24$
Reflections collected	18206
Independent reflections	5806 [R_{int} = 0.0273, R_{sigma} = 0.0325]
Data/restraints/parameters	5806/0/246
Goodness-of-fit on F^2	1.061
Final R indexes [$I \geq 2\sigma(I)$]	R_1 = 0.0504, wR_2 = 0.1000
Final R indexes [all data]	R_1 = 0.0717, wR_2 = 0.1073
Largest diff. peak/hole.e. Å^{-3}	0.59/-0.39
Absolute structure parameter	-0.009(3)

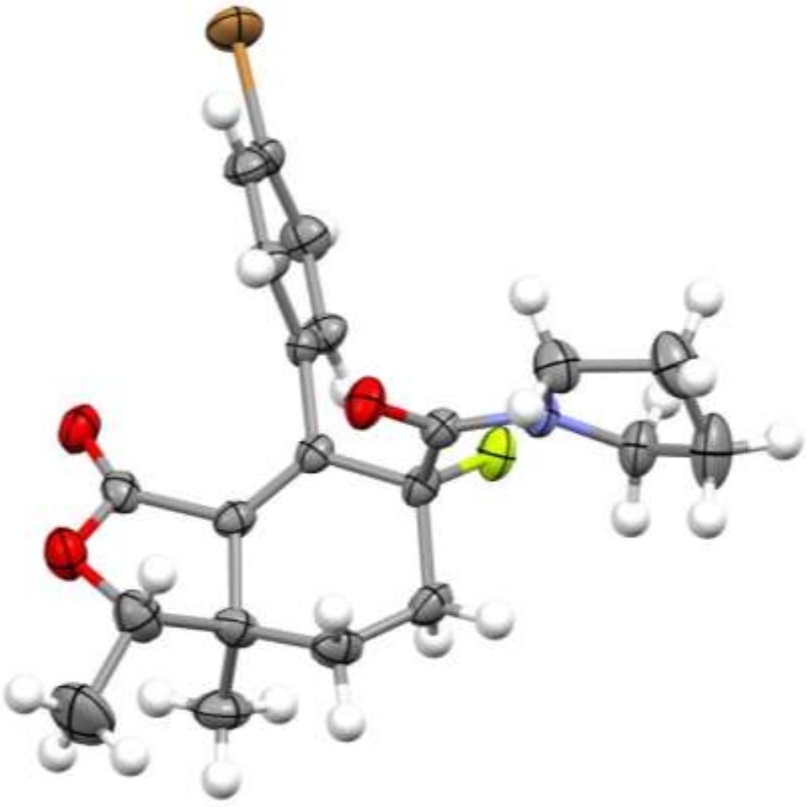

Figure S4. X-ray crystal structure of (3*S*,3a*R*,6*R*)-(–)-**3ia**, showing thermal ellipsoids at the 50% probability level.

Table S6. Crystal data and data collection parameters of (±)-**4ia**.

Empirical formula	$C_{21}H_{23}BrFNO_3$
Formula weight	436.31
Temperature/K	100.00(10)
Crystal system	monoclinic
Space group	$P2_1$/n
a/Å	14.3084(5)
b/Å	6.7116(2)
c/Å	21.1170(7)
α /°	90
β /°	102.754(3)
γ /°	90
V/Å^3	1977.88(11)
Z	4
ρ_{calc}/g cm^{-3}	1.465
μ/mm^{-1}	2.108
F(000)	896
Crystal size/mm^3	0.25 × 0.12 × 0.11
Radiation	Mo Kα (λ = 0.71073)
2θ range for data collection/°	5.838 to 60.066
Index ranges	$-18 \le h \le 18$, $-8 \le k \le 8$, $-27 \le l \le 20$
Reflections collected	14212
Independent reflections	5181 [R_{int} = 0.0228, R_{sigma} = 0.0332]
Data/restraints/parameters	5181/0/246
Goodness-of-fit on F^2	1.064
Final R indexes [$I \ge 2\sigma(I)$]	R_1 = 0.0352, wR_2 = 0.0760
Final R indexes [all data]	R_1 = 0.0521, wR_2 = 0.0859
Largest diff. peak/hole.e. Å^{-3}	0.65/-0.88

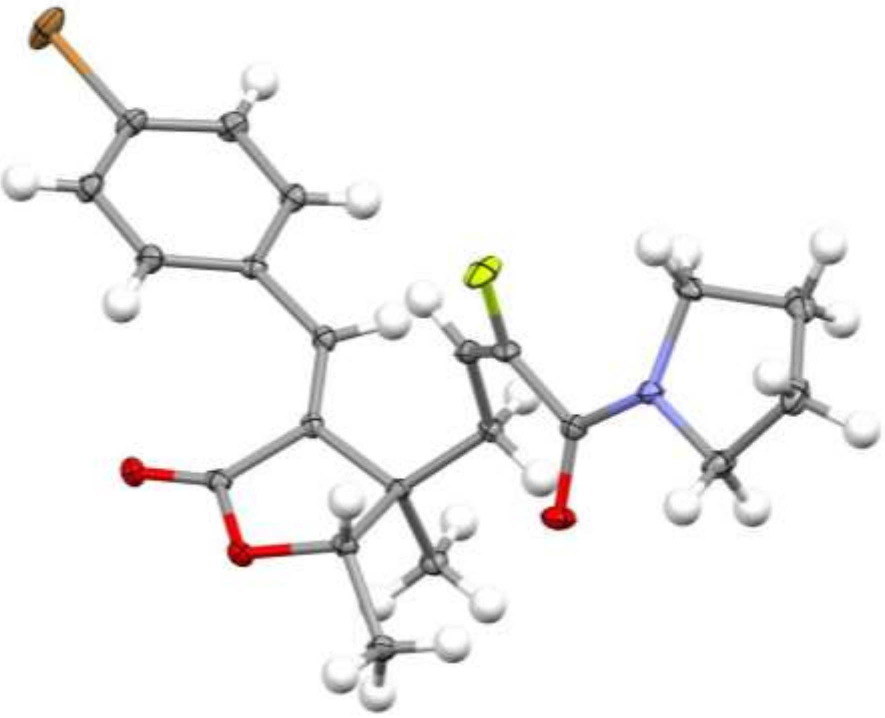

Figure S5. X-ray crystal structure of (±)-**4ia**, showing thermal ellipsoids at the 50% probability level.

Table S7. Crystal data and data collection parameters of (1*R*,5*S*,7a*R*)-(–)-**6aa**.

Empirical formula	$C_{28}H_{31}FN_2O_3S$
Formula weight	494.61
Temperature/K	100.00(10)
Crystal system	monoclinic
Space group	$P2_1$
a/Å	11.3197(2)
b/Å	8.25270(10)
c/Å	26.6741(4)
α /°	90
β /°	100.021(2)
γ /°	90
V/Å^3	2453.83(7)
Z	4
ρ_{calc}/g cm^{-3}	1.339
μ/mm^{-1}	0.173
F(000)	1048
Crystal size/mm^3	0.3 × 0.2 × 0.16
Radiation	Mo Kα (λ = 0.71073)
2θ range for data collection/°	4.21 to 62.78
Index ranges	$-15 \leq h \leq 15$, $-11 \leq k \leq 10$, $-34 \leq l \leq 38$
Reflections collected	40485
Independent reflections	13355 [R_{int} = 0.0283, R_{sigma} = 0.0367]
Data/restraints/parameters	13355/1/633
Goodness-of-fit on F^2	1.034
Final R indexes [$I \geq 2\sigma(I)$]	R_1 = 0.0355, wR_2 = 0.0825
Final R indexes [all data]	R_1 = 0.0421, wR_2 = 0.0875
Largest diff. peak/hole.e. Å^{-3}	0.31/-0.42
Absolute structure parameter	0.011(18)

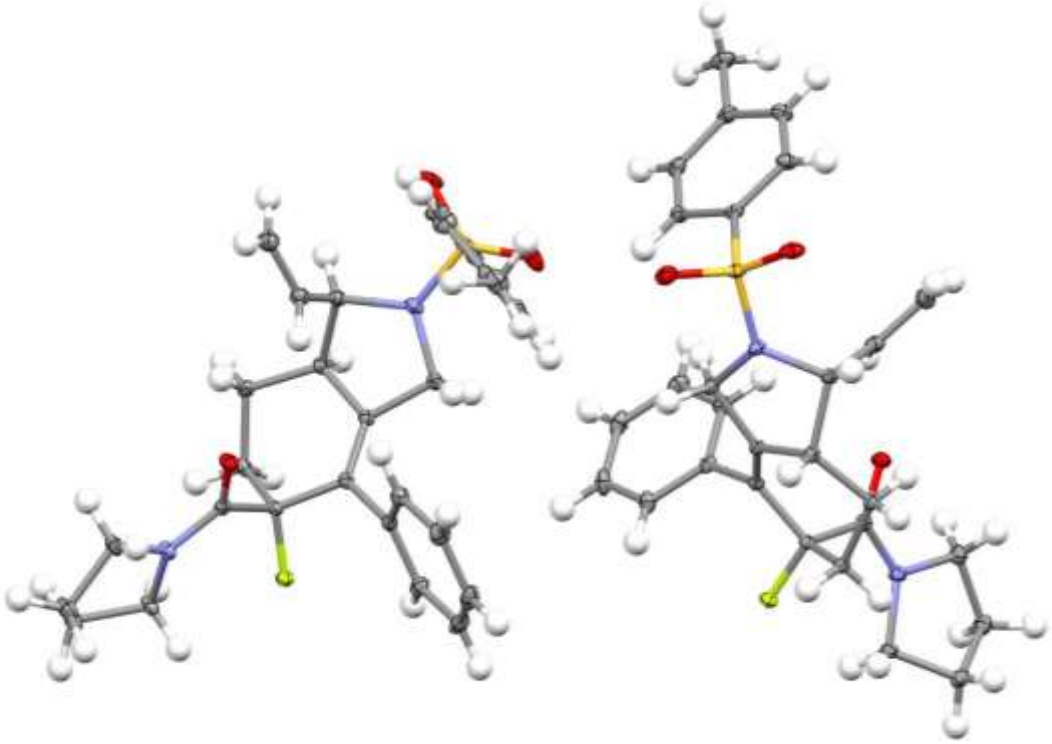

Figure S6. X-ray crystal structures of (1*R*,5*S*,7a*R*)-(–)-**6aa**, showing thermal ellipsoids at the 50% probability level.

Table S8. Crystal data and data collection parameters of (1*R*,5*S*,7a*R*)-(–)-**6dk**.

Empirical formula	$C_{44}H_{49}BrN_2O_5S$
Formula weight	797.82
Temperature/K	293.15
Crystal system	monoclinic
Space group	$P2_1$
a/Å	11.8761(5)
b/Å	14.8615(4)
c/Å	12.4403(5)
α /°	90
β /°	110.310(4)
γ /°	90
V/Å^3	2059.16(14)
Z	2
ρ_{calc}/g cm^{-3}	1.287
μ/mm^{-1}	1.095
F(000)	836
Crystal size/mm^3	0.6 × 0.4 × 0.2
Radiation	Mo Kα (λ = 0.71073)
2θ range for data collection/°	4.086 to 58.252
Index ranges	$-16 \le h \le 15$, $-20 \le k \le 20$, $-17 \le l \le 16$
Reflections collected	30023
Independent reflections	10982 [R_{int} = 0.0589, R_{sigma} = 0.0770]
Data/restraints/parameters	10982/1/479
Goodness-of-fit on F^2	1.014
Final R indexes [$I \geq 2\sigma(I)$]	R_1 = 0.0633, wR_2 = 0.1302
Final R indexes [all data]	R_1 = 0.1204, wR_2 = 0.1500
Largest diff. peak/hole.e. Å^{-3}	0.53/-0.26
Absolute structure parameter	-0.002(6)

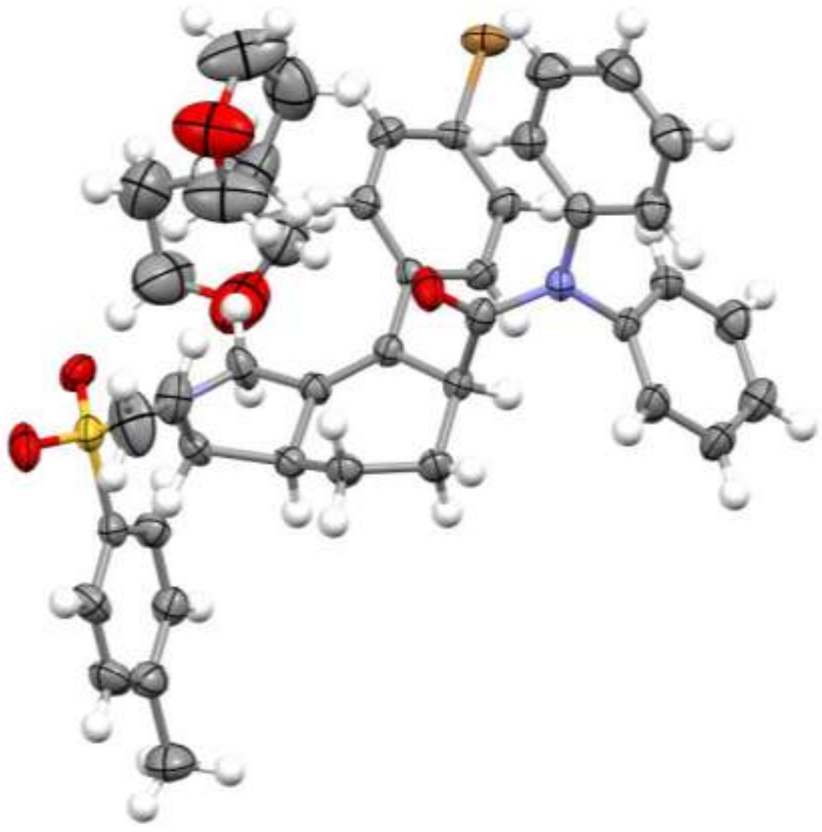

Figure S7. X-ray crystal structure of (1*R*,5*S*,7a*R*)-(–)-**6dk**, showing thermal ellipsoids at the 50% probability level.

4. Theoretical Calculations

4.1. Computational Methods

All the calculations were performed at the DFT level of theory with the B3LYP hybrid functional[25,26] as implemented in Gaussian 16.[27]. To describe the dispersion properly, an explicit dispersion correction term (D3) developed by Grimme and co-workers,[28,29] was also employed in the DFT calculations. The Ahlrichs-type Def2-SVP basis sets[30,31] along with the associated ECP were used for C, H, O, N, F, P, Cl, and Rh atoms in both the geometry optimization and the corresponding frequency calculations.

Due to the evident π–π stacking interaction found in all transition states, to achieve a considerably high accuracy, the large basis sets with diffuse function, Def2-TZVPPD,[30,31] was then employed for the single-point calculations. Solvation effects were introduced using the IEF-PCM method[32,33] with dichloromethane as the solvent. Notably, the solvent accessible surface (SAS) was used in both the optimization and frequency calculations while a better solvent excluded surface (SES) was then adopted during the single-point calculations.

The artificial force induced reaction (AFIR) method[34–37] was applied to explore all the pathways with accessible barriers. Because the transition states involved in this reaction are conformationally flexible, the transition-state (TS) conformation sampling calculations were conducted to ensure that the transition states discussed in this article adopt the most stable conformation. This sampling calculation was realized using the SC-AFIR method at the semi-empirical GFN-xTB[38] level of theory as implemented in ORCA 4.0 software package,[39] and a collision energy of 50 kJ/mol was applied to all the pendant functional groups for any possible conformational change.

For the Rh-catalyzed cyclization step (i.e., **TSAMe**, **TSBMe**. **TSCMe**, and **TSDMe**), 185, 186, 166, and 166 possible conformations were identified, respectively. For the subsequent enantio-determining step, an extensive conformational search was also conducted, yielding tens of conformers for each of the 16 transition states. Specifically, the number of conformers identified for each TS is as follows: **TSAMeAP2c** (45), **TSAMeAP2h** (39), **TSAMeDP2c** (82), **TSAMeDP2h** (78), **TSBMeAP2c** (74), **TSBMeAP2h** (69), **TSBMeDP2c** (84), **TSBMeDP2h** (88), **TSCMeAP2c** (57), **TSCMeAP2h** (44), **TSCMeDP2c** (104), **TSCMeDP2h** (77), **TSDMeAP2c** (36), **TSDMeAP2h** (148), **TSDMeDP2c** (159), and **TSDMeDP2h** (86).

For those conformers having lowest GFN-xTB energies, further optimizations were subsequently conducted at the DFT level of theory as what was discussed previously. All the minima and transition states were fully optimized without any constraints. An intrinsic reaction coordinate (IRC) calculation[40] was performed for each transition state to confirm it connecting to the correct reactant and product. The free energies were computed at 298.15 K and 1 atm. All the geometries shown in this article are visualized by the CYLview software.[41]

In this study, structural isomers of the Rh complexes in all reaction intermediates and transition states after first cyclization step were differentiated as follows. Each intermediate and transition state is designated as **Int/TSX$_{Me}$Y$_P$c** and **Int/TSX$_{Me}$Y$_P$h** where **X$_{Me}$** and **Y$_P$** serve as classification letters. The **X$_{Me}$** (**A$_{Me}$–D$_{Me}$**) indicates the stereochemistry of the two methyl groups on the rhodacycles, while the **Y$_P$** (**A$_P$–D$_P$**) denotes to the isomerism of the Rh complex based on the positions of two P atoms (Figure S8). The lowercase letter of **c** or **h** specifies whether this species arises from a C-C bond insertion reaction or a C-H activation reaction at the second step, respectively.

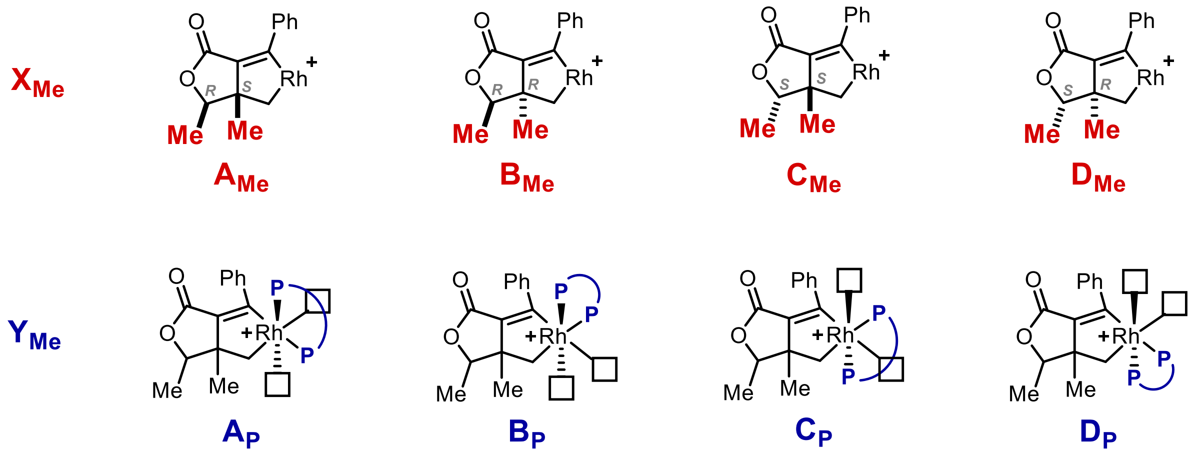

Figure S8. Classification of isomers of intermediate and transition state structures.

4.2. Computational Energies of All Optimized Structures

Table S9. Sums of thermal free energies (*G*) and electronic energies (*E*) for optimized structures.

	**G_{corr} (hartree) B3LYP-D3/Def2-SVP/PCM(DCM)**	***E* (hartree) B3LYP-D3/Def2-TZVPPD/PCM(DCM)**	***G* (hartree) B3LYP-D3/Def2-TZVPPD/PCM(DCM)**
**Int1S**	0.784791	–3365.684823	–3364.900032
**Int1R**	0.785402	–3365.681785	–3364.896383
**TSA$_{Me}$**	0.790869	–3365.663364	–3364.872495
**TSB$_{Me}$**	0.790569	–3365.667209	–3364.876640
**TSC$_{Me}$**	0.789776	–3365.661914	–3364.872138
**TSD$_{Me}$**	0.789625	–3365.668892	–3364.879267
**Int2A$_{Me}$A$_{P}$**	0.789704	–3365.693620	–3364.903916
**Int2A$_{Me}$D$_{P}$**	0.791386	–3365.687194	–3364.895808
**Int2B$_{Me}$A$_{P}$**	0.789391	–3365.690599	–3364.901208
**Int2B$_{Me}$D$_{P}$**	0.788835	–3365.692298	–3364.903463
**Int2C$_{Me}$A$_{P}$**	0.789699	–3365.692596	–3364.902897
**Int2C$_{Me}$D$_{P}$**	0.791164	–3365.684867	–3364.893703
**Int2D$_{Me}$A$_{P}$**	0.790137	–3365.690971	–3364.900834
**Int2D$_{Me}$D$_{P}$**	0.788560	–3365.692861	–3364.904301
**2a**	0.129302	–502.799224	–502.669922
**Int3A$_{Me}$A$_{P}$**	0.948546	–3868.526582	–3867.578036
**Int3A$_{Me}$D$_{P}$**	0.949048	–3868.528469	–3867.579421
**Int3B$_{Me}$A$_{P}$**	0.948653	–3868.524957	–3867.576304
**Int3B$_{Me}$D$_{P}$**	0.949477	–3868.521148	–3867.571671
**Int3C$_{Me}$A$_{P}$**	0.950524	–3868.525615	–3867.575091
**Int3C$_{Me}$D$_{P}$**	0.949137	–3868.524999	–3867.575862
**Int3D$_{Me}$A$_{P}$**	0.949870	–3868.519922	–3867.570052
**Int4D$_{Me}$A$_{P}$**	0.950825	–3868.507906	–3867.557081
**Int3D$_{Me}$D$_{P}$**	0.949358	–3868.520658	–3867.571300
**TSA$_{Me}$A$_{P}$2c**	0.954020	–3868.472087	–3867.518067
**TSA$_{Me}$A$_{P}$2h**	0.946817	–3868.478725	–3867.531908
**TSA$_{Me}$D$_{P}$2c**	0.953439	–3868.491257	–3867.537818
**TSA$_{Me}$D$_{P}$2h**	0.948192	–3868.495433	–3867.547241
**TSB$_{Me}$A$_{P}$2c**	0.952514	–3868.491279	–3867.538765
**TSB$_{Me}$A$_{P}$2h**	0.949001	–3868.485846	–3867.536845
**TSB$_{Me}$D$_{P}$2c**	0.954044	–3868.475747	–3867.521703
**TSB$_{Me}$D$_{P}$2h**	0.946919	–3868.485295	–3867.538376
**TSC$_{Me}$A$_{P}$2c**	0.955715	–3868.471924	–3867.516209
**TSC$_{Me}$A$_{P}$2h**	0.947420	–3868.478411	–3867.530991
**TSC$_{Me}$D$_{P}$2c**	0.954191	–3868.488854	–3867.534663
**TSC$_{Me}$D$_{P}$2h**	0.947420	–3868.489633	–3867.542213
**TSD$_{Me}$A$_{P}$2c**	0.952529	–3868.497342	–3867.544813
**TSD$_{Me}$A$_{P}$2h**	0.949053	–3868.492009	–3867.542956
**TSD$_{Me}$D$_{P}$2c**	0.954105	–3868.474602	–3867.520497
**TSD$_{Me}$D$_{P}$2h**	0.946835	–3868.485214	–3867.538379
**Int5A$_{Me}$A$_{P}$c**	0.952096	–3868.537244	–3867.585148
**Int5A$_{Me}$A$_{P}$h**	0.951985	–3868.521877	–3867.569892
**Int5A$_{Me}$D$_{P}$c**	0.952630	–3868.534133	–3867.581503
**Int5A$_{Me}$D$_{P}$h**	0.951604	–3868.533565	–3867.581553
**Int5B$_{Me}$A$_{P}$c**	0.951350	–3868.526930	–3867.575580

	G_{corr} (hartree) B3LYP-D3/Def2-SVP/PCM(DCM)	*E* (hartree) B3LYP-D3/Def2-TZVPPD/PCM(DCM)	*G* (hartree) B3LYP-D3/Def2-TZVPPD/PCM(DCM)
**Int5B$_{Me}$A$_P$h**	0.951604	–3868.514513	–3867.562909
**Int5B$_{Me}$D$_P$c**	0.952383	–3868.534778	–3867.582395
**Int5B$_{Me}$D$_P$h**	0.949007	–3868.526626	–3867.577619
**Int5C$_{Me}$A$_P$c**	0.950504	–3868.536259	–3867.585755
**Int5C$_{Me}$A$_P$h**	0.951644	–3868.518620	–3867.566976
**Int5C$_{Me}$D$_P$c**	0.954129	–3868.530024	–3867.575895
**Int5C$_{Me}$D$_P$h**	0.952139	–3868.531678	–3867.579539
**Int5D$_{Me}$A$_P$c**	0.952804	–3868.543471	–3867.590667
**Int5D$_{Me}$A$_P$h**	0.949417	–3868.537499	–3867.588082
**Int5D$_{Me}$D$_P$c**	0.951141	–3868.535578	–3867.584437
**Int5D$_{Me}$D$_P$h**	0.948199	–3868.527670	–3867.579471
**TSD$_{Me}$A$_P$3c**	0.947479	–3868.509968	–3867.562489
**TSD$_{Me}$A$_P$3h**	0.949879	–3868.510168	–3867.560289
**D$_{Me}$A$_P$c-P**	0.951855	–3868.589127	–3867.637272
**D$_{Me}$A$_P$h-P**	0.948281	–3868.562780	–3867.614499
**2d**	0.138165	–403.520023	–403.381858
**2e**	0.163733	–442.851402	–442.687669
**2f**	0.127052	–863.142875	–863.015823
**Int3D$_{Me}$A$_P$-H**	0.958985	–3769.252548	–3768.293563
**Int3D$_{Me}$A$_P$-Me**	0.983687	–3808.580688	–3807.597001
**Int3D$_{Me}$A$_P$-Cl**	0.945437	–4228.876261	–4227.930824
**TSD$_{Me}$A$_P$2c-H**	0.961445	–3769.222711	–3768.261266
**TSD$_{Me}$A$_P$2h-H**	0.958189	–3769.214863	–3768.256674
**TSD$_{Me}$A$_P$2c-Me**	0.989667	–3808.548362	–3807.558695
**TSD$_{Me}$A$_P$2h-Me**	0.981697	–3808.551258	–3807.569561
**TSD$_{Me}$A$_P$2c-Cl**	0.951332	–4228.835548	–4227.884216
**TSD$_{Me}$A$_P$2h-Cl**	0.943273	–4228.839629	–4227.896356
**Int5D$_{Me}$A$_P$c-H**	0.961985	–3769.254755	–3768.292770
**Int5D$_{Me}$A$_P$h-H**	0.960856	–3769.253670	–3768.292814
**Int5D$_{Me}$A$_P$c-Me**	0.989538	–3808.579313	–3807.589775
**Int5D$_{Me}$A$_P$h-Me**	0.987451	–3808.584455	–3807.597004
**Int5D$_{Me}$A$_P$c-Cl**	0.951514	–4228.873965	–4227.922451
**Int5D$_{Me}$A$_P$h-Cl**	0.946731	–4228.863005	–4227.916274

4.3. Computational Studies for Reaction Pathways (Path A$_{Me}$, B$_{Me}$, C$_{Me}$, and D$_{Me}$)

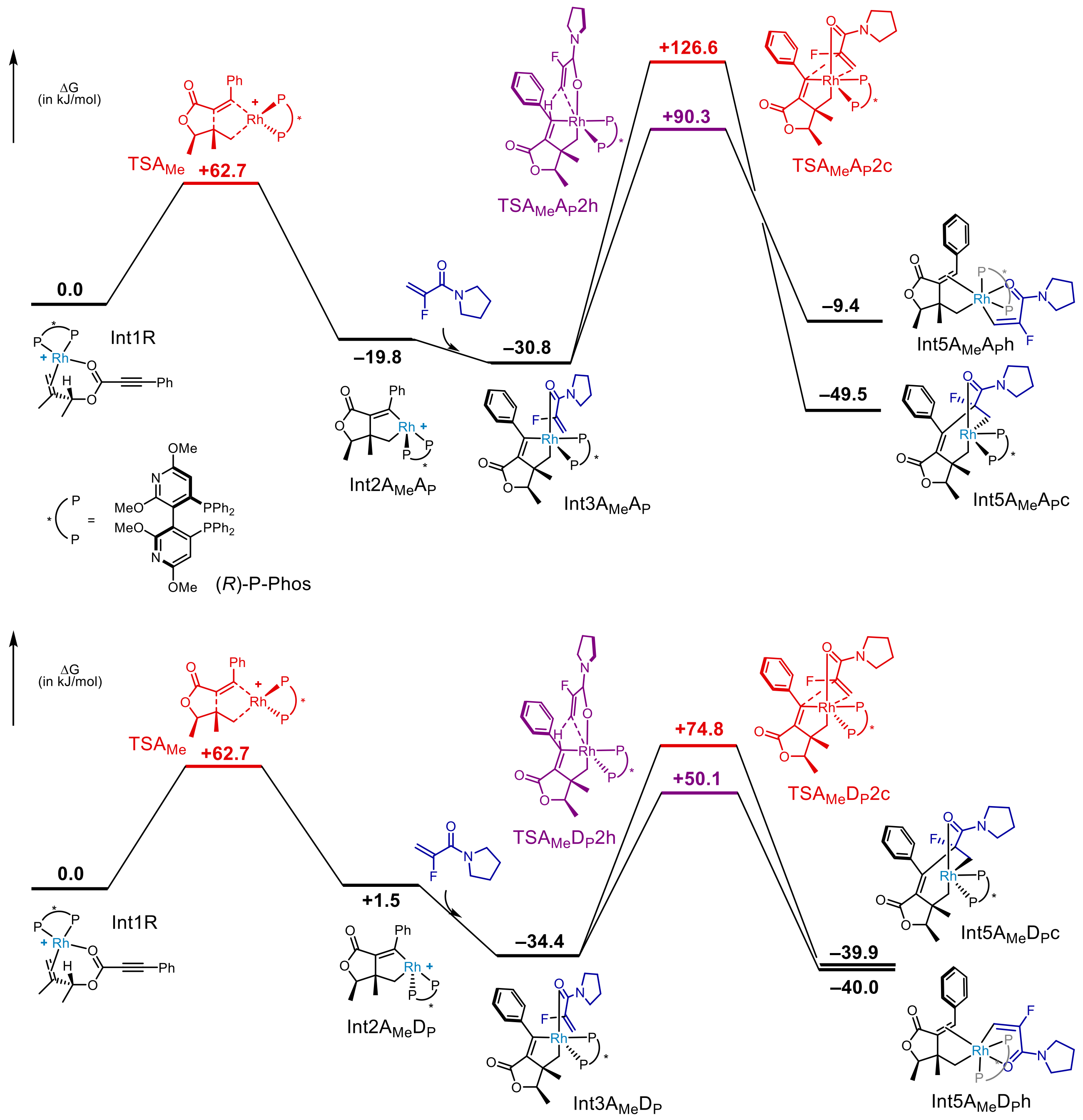

Figure S9. Computed free energy profiles at the B3LYP-D3/Def2-TZVPPD/IEFPCM(DCM)//B3LYP-D3/Def2-SVP/IEFPCM(DCM) level of theory for Path **A$_{Me}$**, which is derived from (*R*)-**1h** (unit is given in kJ/mol).

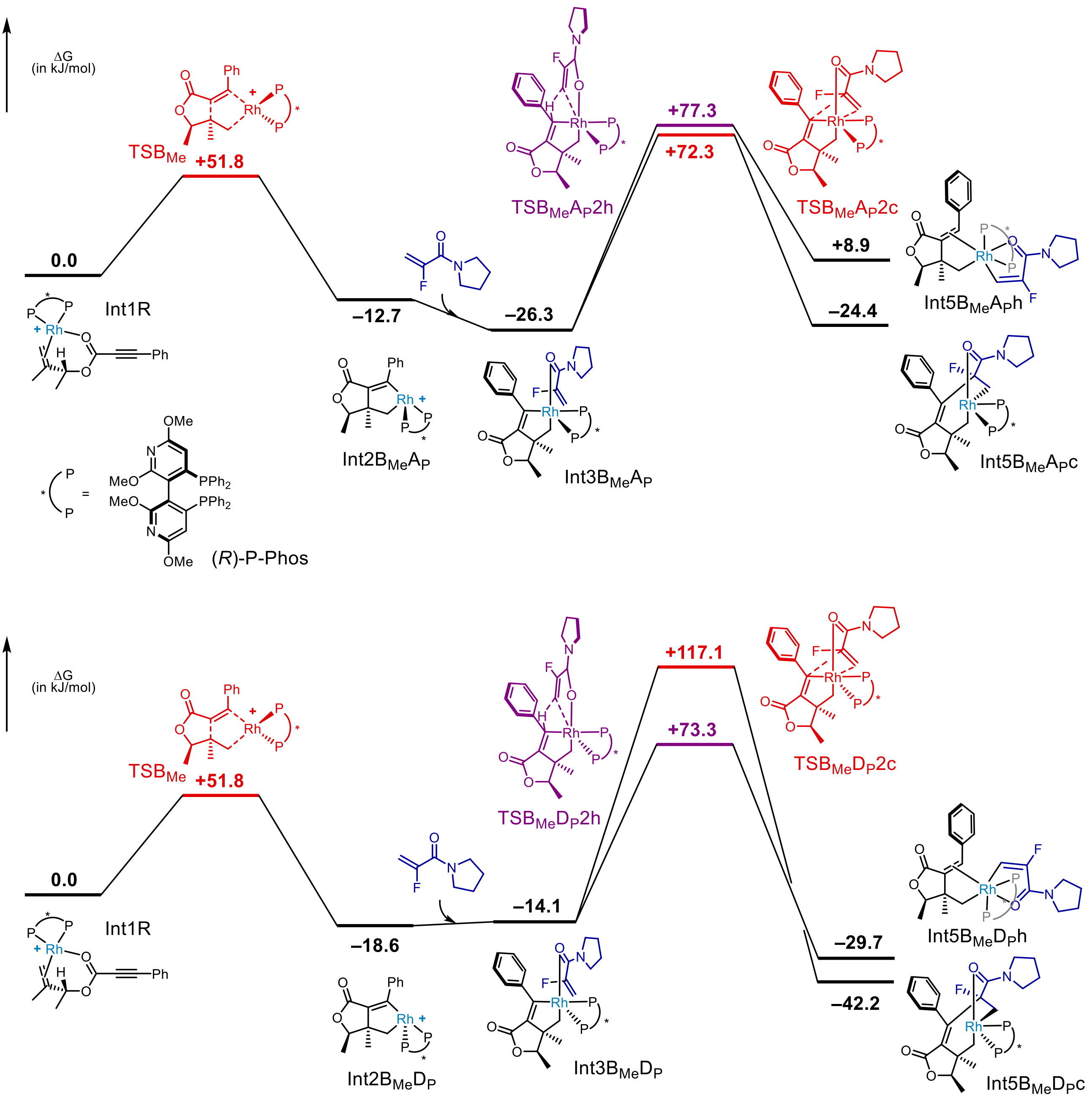

Figure S10. Computed free energy profiles at the B3LYP-D3/Def2-TZVPPD/IEFPCM(DCM)// B3LYP-D3/Def2-SVP/IEFPCM(DCM) level of theory for Path **B$_{Me}$**, which is derived from (*R*)-**1h** (unit is given in kJ/mol).

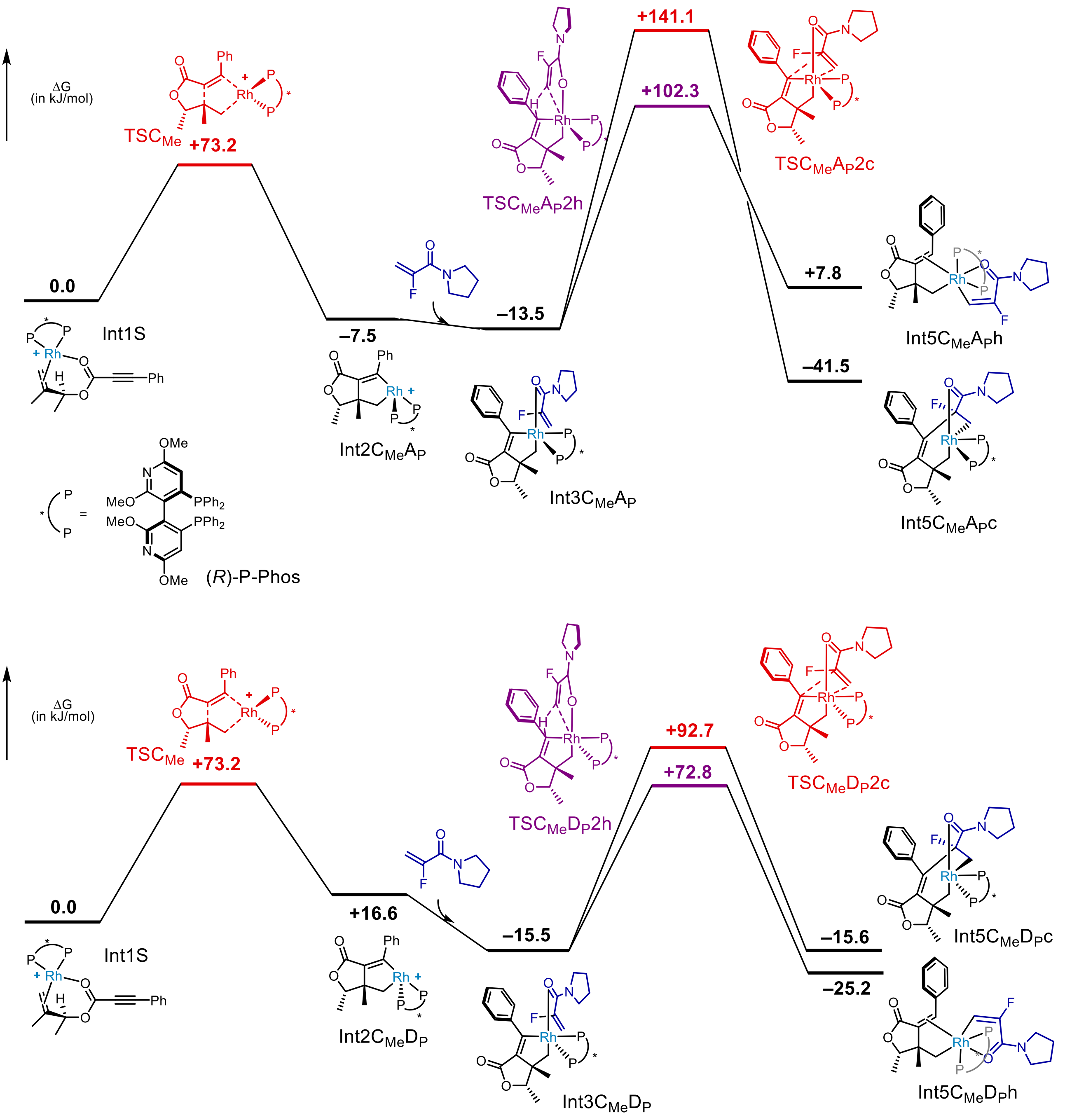

Figure S11. Computed free energy profiles at the B3LYP-D3/Def2-TZVPPD/IEFPCM(DCM)// B3LYP-D3/Def2-SVP/IEFPCM(DCM) level of theory for Path **C$_{Me}$**, which is derived from (*S*)-**1h** (unit is given in kJ/mol).

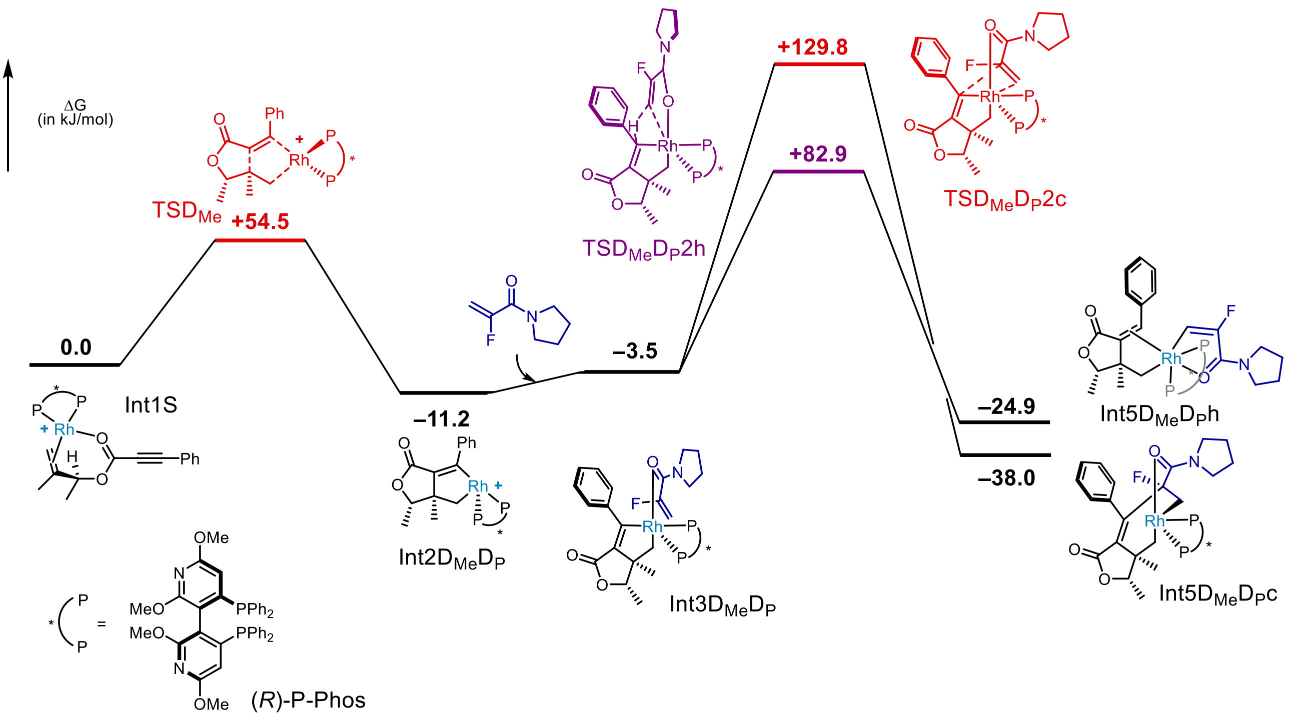

Figure S12. Computed free energy profiles at the B3LYP-D3/Def2-TZVPPD/IEFPCM(DCM)// B3LYP-D3/Def2-SVP/IEFPCM(DCM) level of theory for Path **D$_{Me}$D$_P$**, which is derived from (*S*)-**1h** (unit is given in kJ/mol).

4.4. Results of Conformational Sampling of Transition States of C–C Insertion and C–H Activation Steps

Table S10. Effective free energy barriers ($\Delta G^{\neq}$) and relative barrier differences ($\Delta\Delta G^{\neq}$) for all eight transition states, which is derived from (*R*)-**1h**, in enantio-determining step (unit in kJ/mol).

TS	TSA$_{Me}$A$_P$2c	TSA$_{Me}$A$_P$2h	TSA$_{Me}$D$_P$2c	TSA$_{Me}$D$_P$2h	TSB$_{Me}$A$_P$2c	TSB$_{Me}$A$_P$2h	TSB$_{Me}$D$_P$2c	TSB$_{Me}$D$_P$2h
$\Delta G^{\neq}$	+157.4	+120.8	+109.2	**+84.4**	+99.1	+103.6	+135.7	+91.9
$\Delta\Delta G^{\neq}$	+73.0	+36.4	+24.8	**0.0**	+14.7	+19.2	+51.3	+7.5

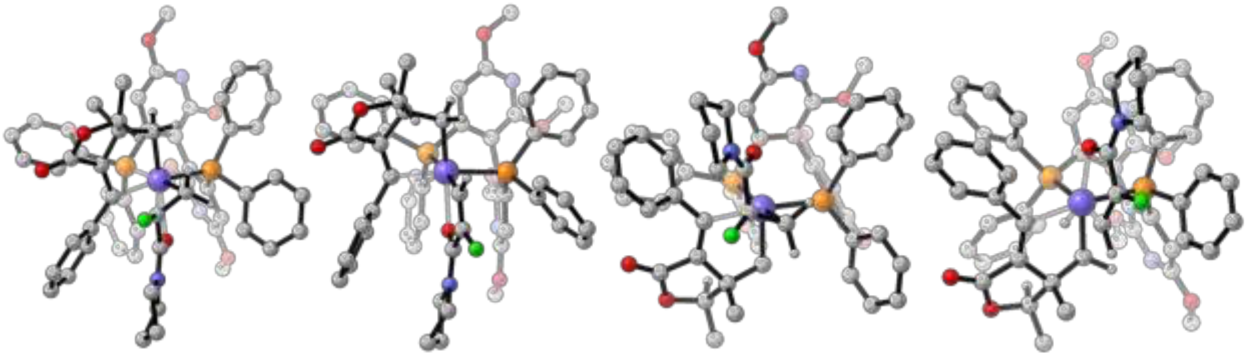

TSA$_{Me}$A$_P$2c **TSA$_{Me}$A$_P$2h** **TSA$_{Me}$D$_P$2c** **TSA$_{Me}$D$_P$2h**

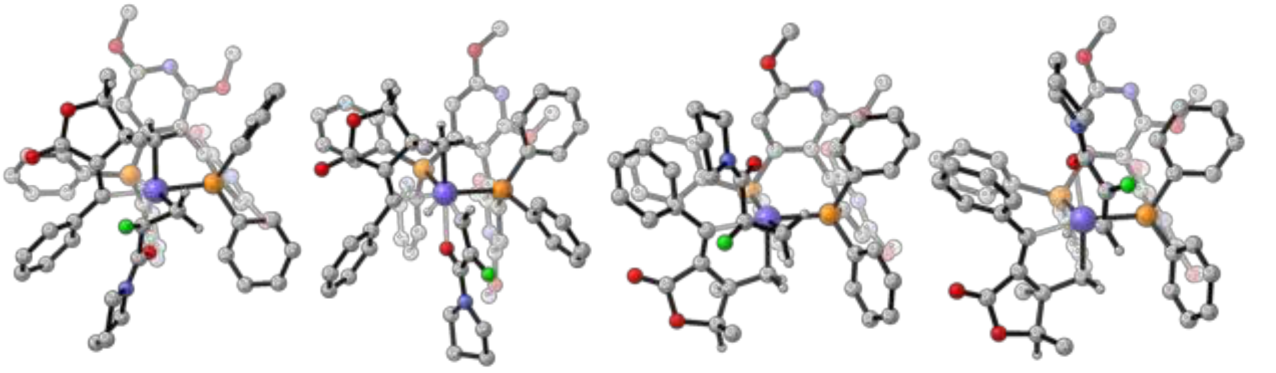

TSB$_{Me}$A$_P$2c **TSB$_{Me}$A$_P$2h** **TSB$_{Me}$D$_P$2c** **TSB$_{Me}$D$_P$2h**

Figure S13. Structures of all eight transition states, which is derived from (*R*)-**1h**, in enantio-determining step.

Table S11. Effective free energy barriers ($\Delta G^{\neq}$) and relative barrier differences ($\Delta\Delta G^{\neq}$) for all eight transition states, which is derived from (*S*)-**1h**, in enantio-determining step (unit in kJ/mol).

TS	TSC$_{Me}$A$_{P}$2c	TSC$_{Me}$A$_{P}$2h	TSC$_{Me}$D$_{P}$2c	TSC$_{Me}$D$_{P}$2h	TSD$_{Me}$A$_{P}$2c	TSD$_{Me}$A$_{P}$2h	TSD$_{Me}$D$_{P}$2c	TSD$_{Me}$D$_{P}$2h
$\Delta G^{\neq}$	+154.6	+115.8	+108.1	+88.3	**+68.1**	**+73.0**	+141.0	+94.1
$\Delta\Delta G^{\neq}$	+86.5	+47.7	+40.0	+20.2	**0.0**	**+4.9**	+72.9	+26.0

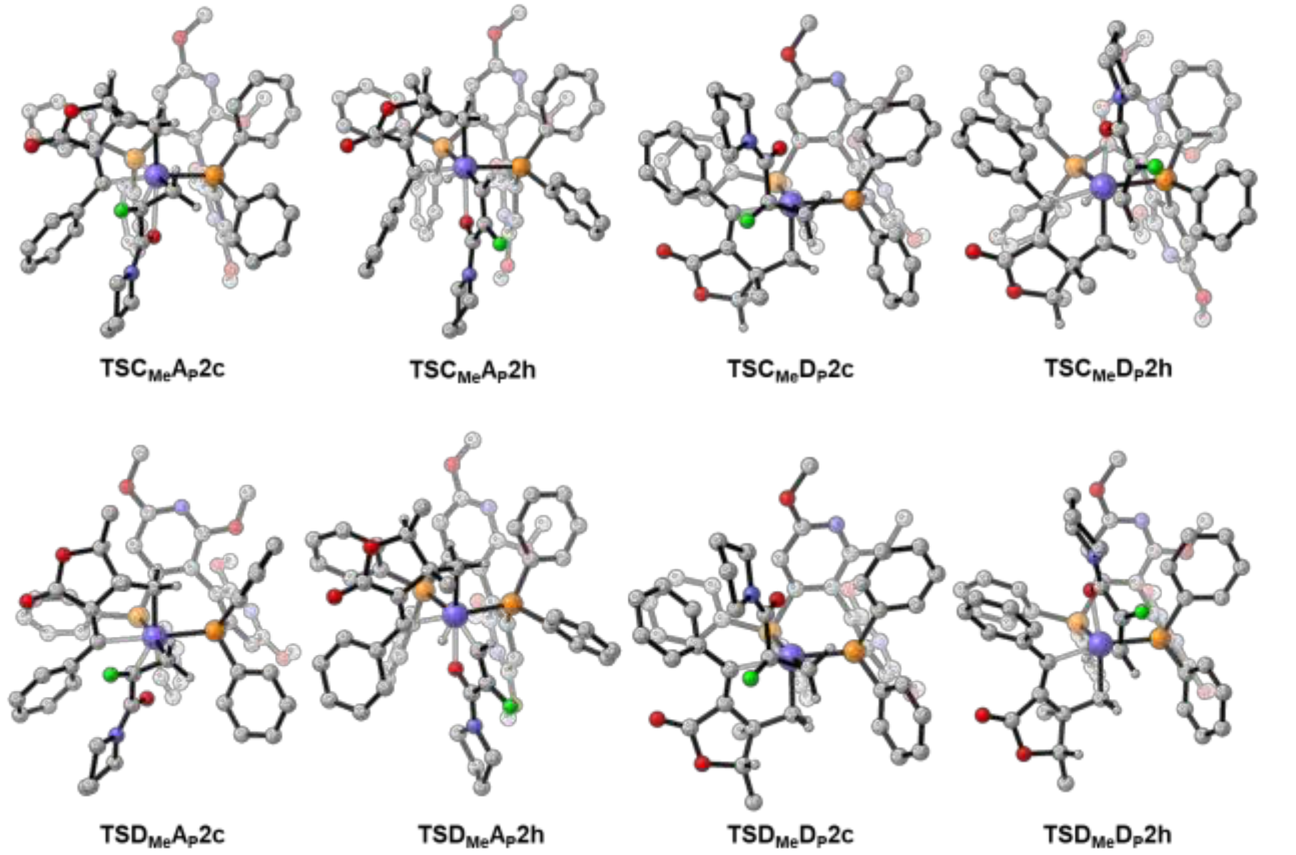

Figure S14. Structures of all eight transition states, which is derived from (*S*)-**1h**, in enantio-determining step.

4.5. Influence of α-Substituents on Acrylamides

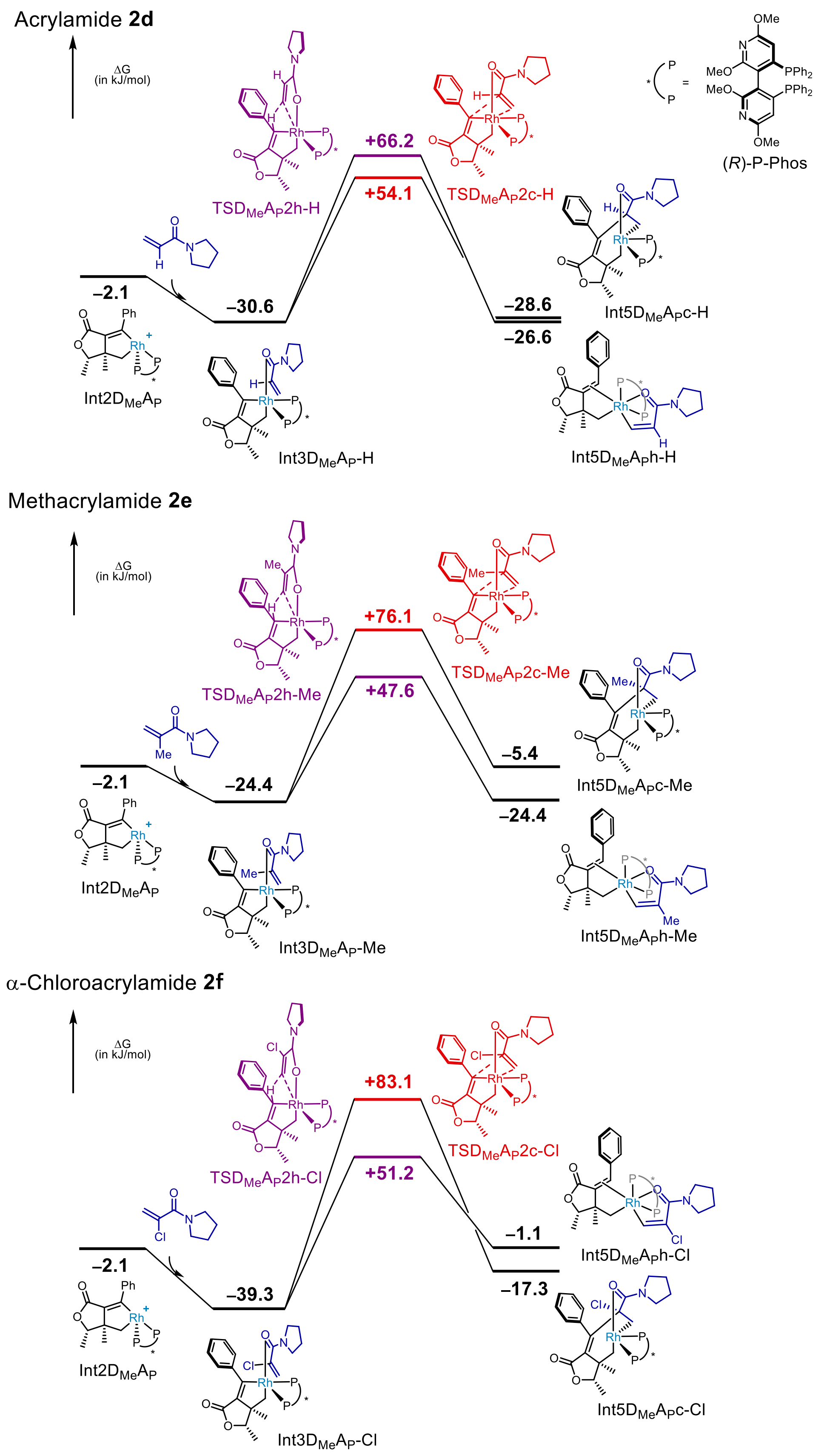

Figure S15. Computed free energy profiles at the B3LYP-D3/Def2-TZVPPD/IEFPCM(DCM)//B3LYP-D3/Def2-SVP/IEFPCM(DCM) level of theory for reactions of intermediate **Int2D$_{Me}$A$_P$** with acrylamides **2d**–**f** bearing various α-substituents (unit is given in kJ/mol).

5. References

1 B. J. Lee, A. R. Ickes, A. K. Gupta, S. C. Ensign, T. D. Ho, A. Tarasewicz, E. P. Vanable, G. D. Kortman, K. L. Hull, *Org. Lett.* **2022**, *24*, 5513–5518.

2 N. Jeong, D. H. Kim, J. H. Choi, *Chem. Commun.* **2004**, 1134–1135.

3 H. Jullien, D. Brissy, R. Sylvain, P. Retailleau, J. Naubron, S. Gladiali, A. Marinetti, *Adv. Synth. Catal.* **2011**, *353*, 1109–1124.

4 R. Liu, D. Yang, F. Chang, L. Giordano, G. Liu, A. Tenaglia, *Asian J. Org. Chem.* **2019**, *8*, 2011–2016.

5 J. H. Park, Y. Cho, Y. K. Chung, *Angew. Chem. Int. Ed.* **2010**, *49*, 5138–5141.

6 Q. Wu, J. Hu, X. Ren, J. S. Zhou, *Chem. Eur. J.* **2011**, *17*, 11553–11558.

7 F. Kramm, J. Teske, F. Ullwer, W. Frey, B. Plietker, *Angew. Chem. Int. Ed.* **2018**, *57*, 13335–13338.

8 Y. Mu, F. W. W. Hartrampf, E. C. Yu, K. E. Lounsbury, R. R. Schrock, F. Romiti, A. H. Hoveyda, *Nat. Chem.* **2022**, *14*, 640–649.

9 A. Lefevre, R. Guillot, C. Kouklovsky, G. Vincent, *Org. Lett.* **2024**, *26*, 7403–7407.

10 K. Miura, D. Wang, A. Hosomi, *J. Am. Chem. Soc.* **2005**, *127*, 9366–9367.

11 A. Kolleth, M. Cattoen, S. Arseniyadis, J. Cossy, *Chem. Commun.* **2013**, *49*, 9338–9340.

12 X. Sun, X. Dong, Y. Yang, J. Fu, Y. Wang, Z. Li, Y. Liu, H. Liu, *Org. Biomol. Chem.* **2021**, *19*, 2676–2680.

13 R. C. Larock, T. R. Hightower, L. A. Hasvold, K. P. Peterson, *J. Org. Chem.* **1996**, *61*, 3584–3585.

14 M. Virolleaud, O. Piva, *Eur. J. Org. Chem.* **2007**, 1606–1612.

15 K. Burgess, M. J. Ohlmeyer, *J. Org. Chem.* **1991**, *56*, 1027–1036.

16 A. B. Smith, L. Kürti, A. H. Davulcu, *Org. Lett.* **2006**, *8*, 2167–2170.

17 S. Hamada, Y. Sato, Y. Komiya, K. Tanaka, *Org. Lett.* **2025**, *27*, 12172–12177.

18 T. D. Svejstrup, W. Zawodny, J. J. Douglas, D. Bidgeli, N. S. Sheikh, D. Leonori, *Chem. Commun.* **2016**, *52*, 12302–12305.

19 W. Yu, Y. Luo, L. Yan, D. Liu, Z. Wang, P. Xu, *Angew. Chem. Int. Ed.* **2019**, *58*, 10941–10945.

20 X. Yi, S. Lei, W. Liu, F. Che, C. Yu, X. Liu, Z. Wang, X. Zhou, Y. Zhang, *Org. Lett.* **2020**, *22*, 4583–4587.

21 N. Kuhnert, A. Le-Gresley, *Org. Biomol. Chem.* **2005**, *3*, 2175–2182.

22 L. A. Bateman, T. B. Nguyen, A. M. Roberts, D. K. Miyamoto, W.-M. Ku, T. R. Huffman, Y. Petri, M. J. Heslin, C. M. Contreras, C. F. Skibola, J. A. Olzmann, D. K. Nomura, *Chem. Commun.* **2017**, *53*, 7234–7237.

23 G. M. Sheldrick, *Acta Cryst.* **2015**, *A71*, 3–8.

24 G. M. Sheldrick, *Acta Cryst.* **2015**, *C71*, 3–8.

25 A. D. Becke, *J. Chem. Phys.* **1993**, *98*, 1372–1377.

26 C. Lee, W. Yang, R. G. Parr, *Phys. Rev. B* **1988**, *37*, 785–789.

27 Gaussian 16, Revision C.01, M. J. Frisch, G. W. Trucks, H. B. Schlegel, G. E. Scuseria, M. A. Robb, J. R. Cheeseman, G. Scalmani, V. Barone, G. A. Petersson, H. Nakatsuji, X. Li, M. Caricato, A. V. Marenich, J. Bloino, B. G. Janesko, R. Gomperts, B. Mennucci, H. P. Hratchian, J. V. Ortiz, A. F. Izmaylov, J. L. Sonnenberg, D. Williams-Young, F. Ding, F. Lipparini, F. Egidi, J. Goings, B. Peng, A. Petrone, T. Henderson, D. Ranasinghe, V. G.

Zakrzewski, J. Gao, N. Rega, G. Zheng, W. Liang, M. Hada, M. Ehara, K. Toyota, R. Fukuda, J. Hasegawa, M. Ishida, T. Nakajima, Y. Honda, O. Kitao, H. Nakai, T. Vreven, K. Throssell, J. A. Montgomery, Jr., J. E. Peralta, F. Ogliaro, M. J. Bearpark, J. J. Heyd, E. N. Brothers, K. N. Kudin, V. N. Staroverov, T. A. Keith, R. Kobayashi, J. Normand, K. Raghavachari, A. P. Rendell, J. C. Burant, S. S. Iyengar, J. Tomasi, M. Cossi, J. M. Millam, M. Klene, C. Adamo, R. Cammi, J. W. Ochterski, R. L. Martin, K. Morokuma, O. Farkas, J. B. Foresman and D. J. Fox, Gaussian, Inc., Wallingford CT, **2016**.

28 S. Grimme, J. Antony, S. Ehrlich, H. Krieg, *J. Chem. Phys.* **2010**, *132*, 154104.

29 S. Grimme, S. Ehrlich, L. Goerigk, *J. Comput. Chem.* **2011**, *32*, 1456–1465.

30 F. Weigend, R. Ahlrichs, *Phys. Chem. Chem. Phys.* **2005**, *7*, 3297–3305.

31 F. Weigend, *Phys. Chem. Chem. Phys.* **2006**, *8*, 1057–1065.

32 J. Tomasi, B. Mennucci, R. Cammi, *Chem. Rev.* **2005**, *105*, 2999–3094.

33 G. Scalmani, M. J. Frisch, *J. Chem. Phys.* **2010**, *132*, 114110.

34 S. Maeda, K. Ohno, K. Morokuma, *Phys. Chem. Chem. Phys.* **2013**, *15*, 3683–3701.

35 S. Maeda, Y. Harabuchi, M. Takagi, T. Taketsugu, K. Morokuma, *Chem. Rec.* **2016**, *16*, 2232–2248.

36 S. Maeda, Y. Harabuchi, M. Takagi, K. Saita, K. Suzuki, T. Ichino, Y. Sumiya, K. Sugiyama, Y. Ono, *J. Comput. Chem.* **2018**, *39*, 233–251.

37 S. Maeda, Y. Harabuchi, *Wiley Interdiscip. Rev.: Comput. Mol. Sci.* **2021**, *11*, e1538.

38 S. Grimme, C. Bannwarth, P. Shushkov, *J. Chem. Theory Comput.* **2017**, *13*, 1989–2009.

39 F. Neese, "Software update: the ORCA program system, version 4.0" *Wiley Interdiscip. Rev.: Comput. Mol. Sci.* **2018**, *8*, e1327.

40 K. Fukui, *Acc. Chem. Res.* **1981**, *14*, 363–368.

41 CYLview, 1.0b; C. Y. Legault, Université de Sherbrooke, **2009**.

6. ^{1}H, ^{13}C, and ^{19}F NMR Spectra

6.1. Synthesis of Substrates

N-(But-2-yn-1-yl)-4-methyl-*N*-(3-methylbut-3-en-2-yl)benzenesulfonamide (1a)

^{1}H NMR ($CDCl_3$, 400 MHz)

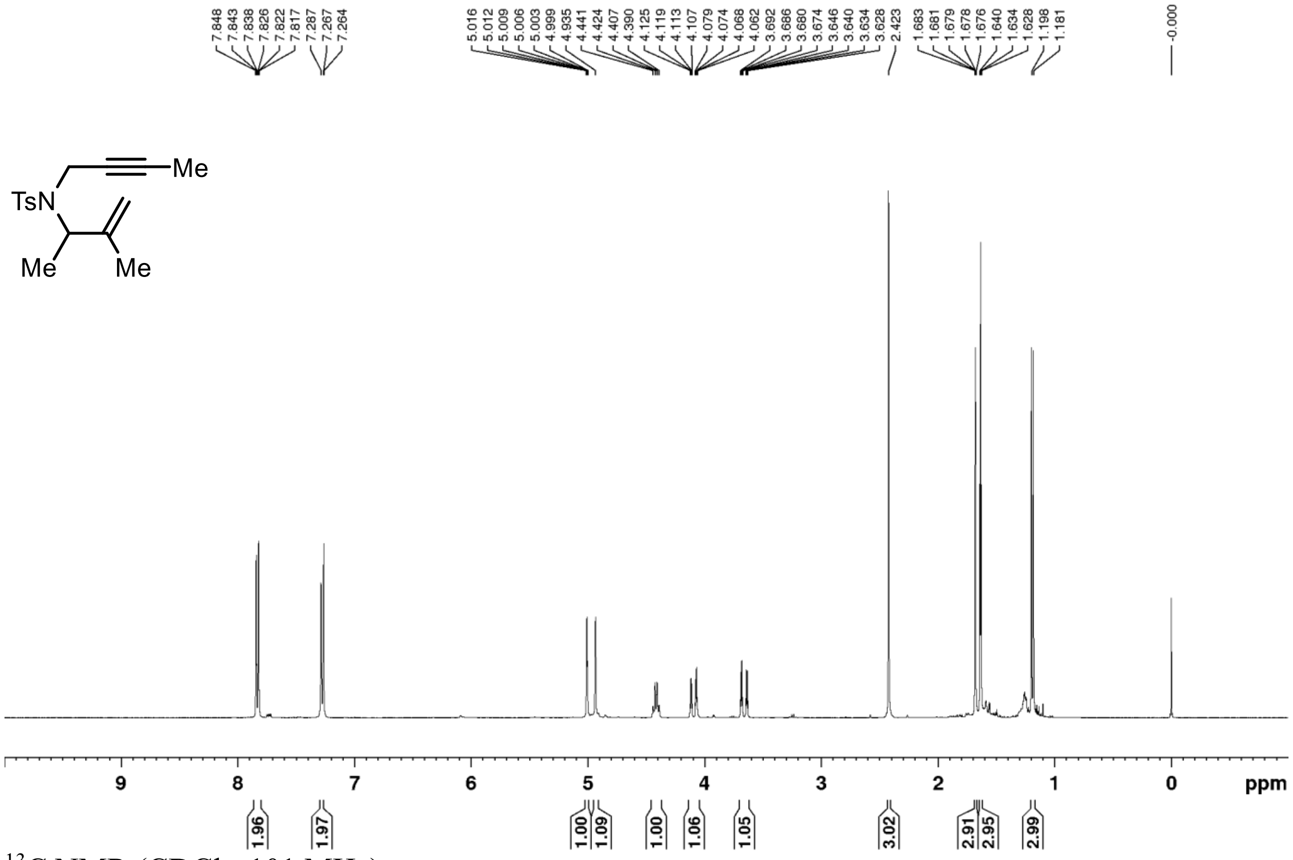

^{13}C NMR ($CDCl_3$, 101 MHz)

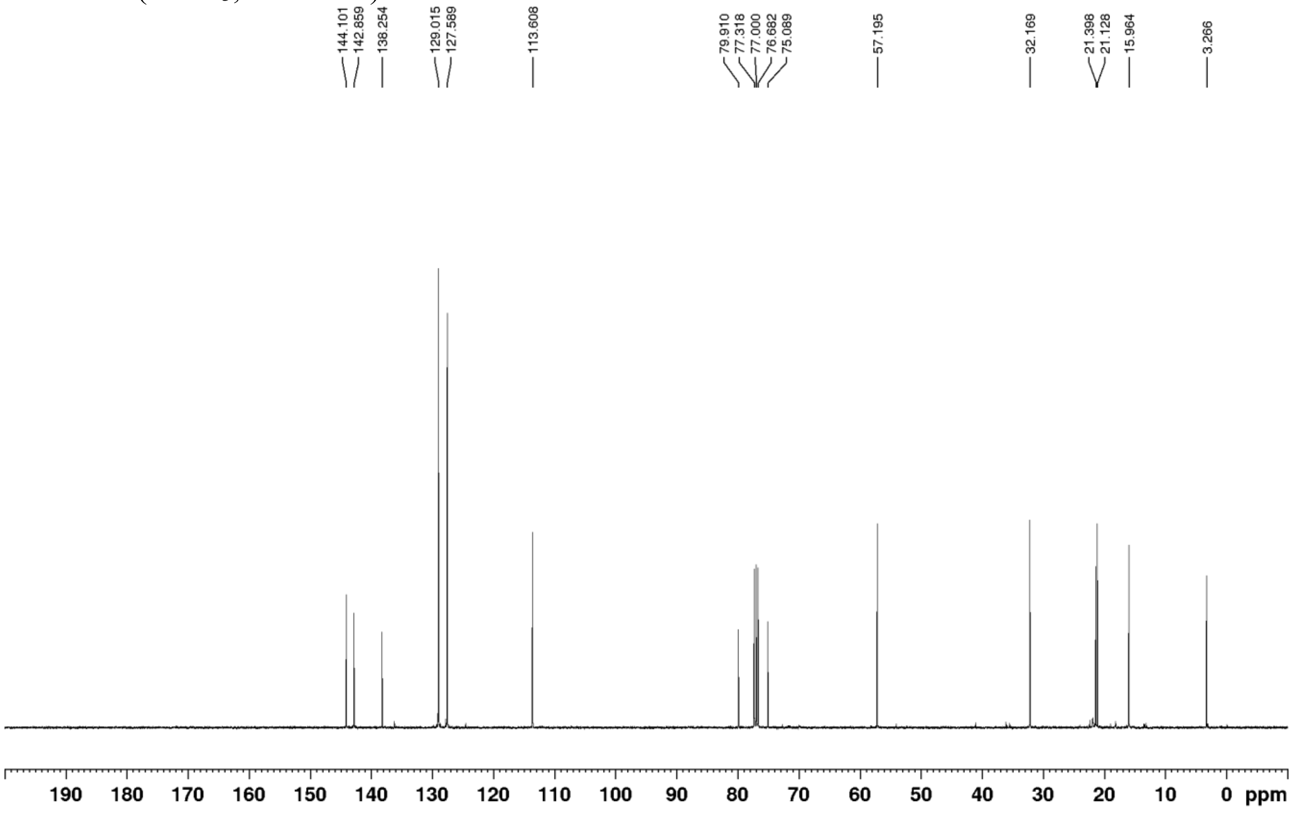

***N*-(2-methyloct-1-en-3-yl)-(4-methylbenzene)sulfonamide (S3a)**

^{1}H NMR ($CDCl_3$, 400 MHz)

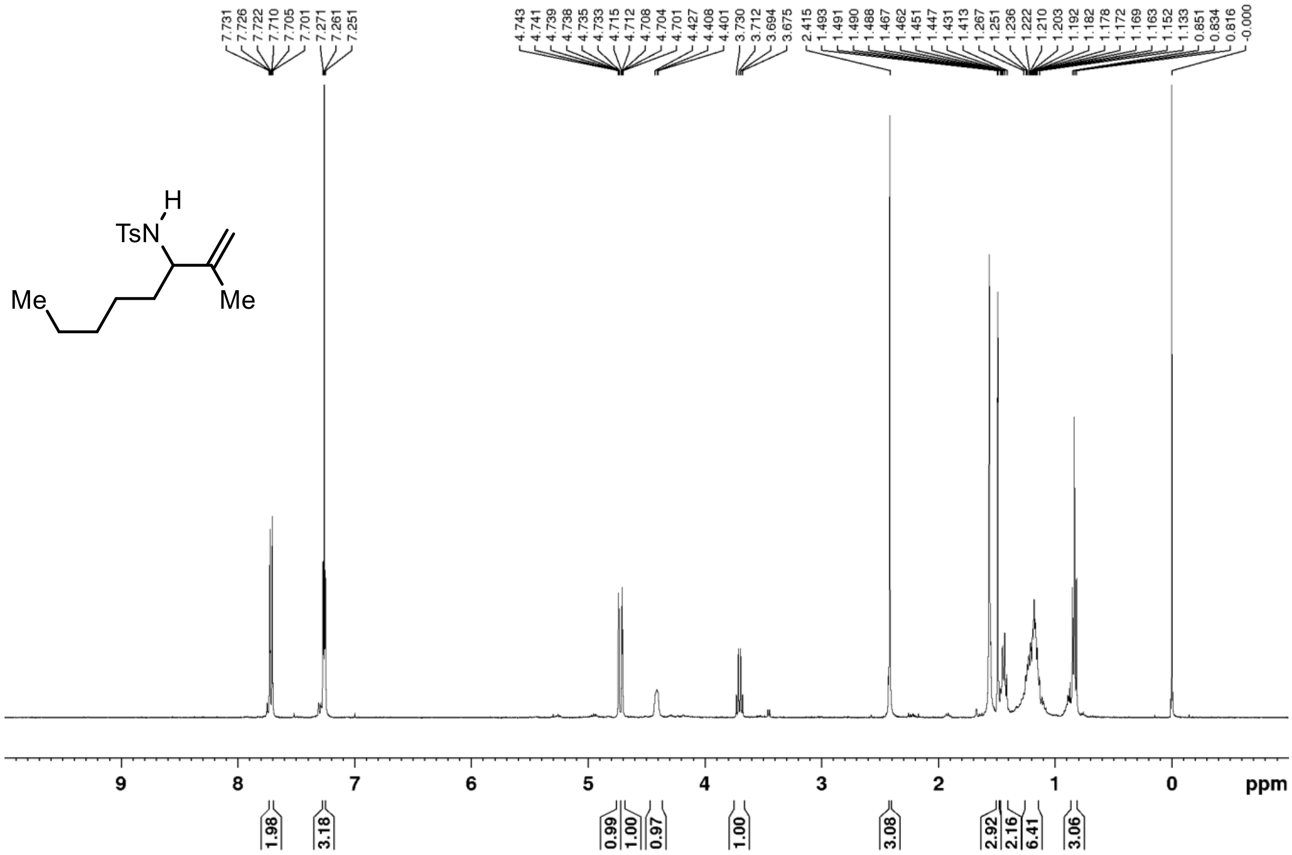

^{13}C NMR ($CDCl_3$, 101 MHz)

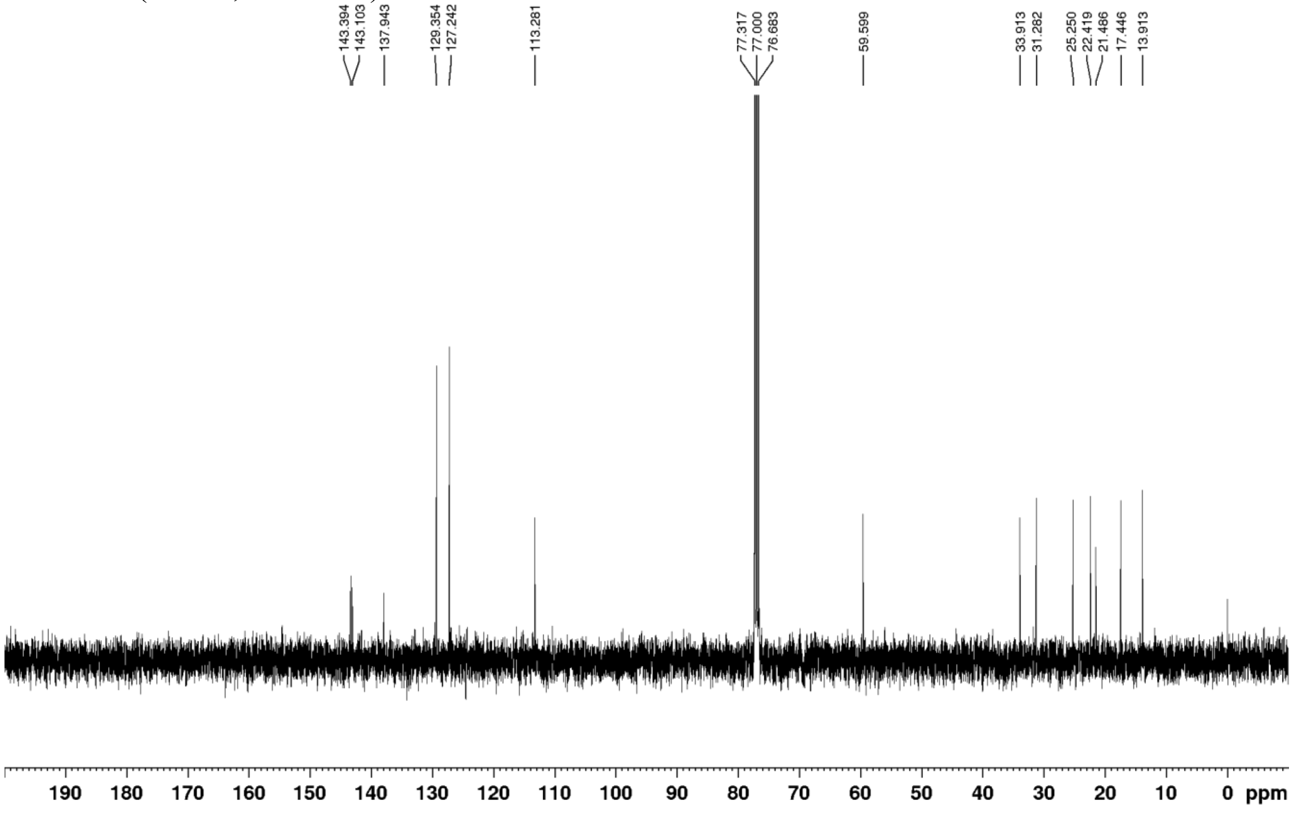

N-(But-2-yn-1-yl)-4-methyl-N-(2-methyloct-1-en-3-yl)benzenesulfonamide (1b)

^{1}H NMR ($CDCl_3$, 400 MHz)

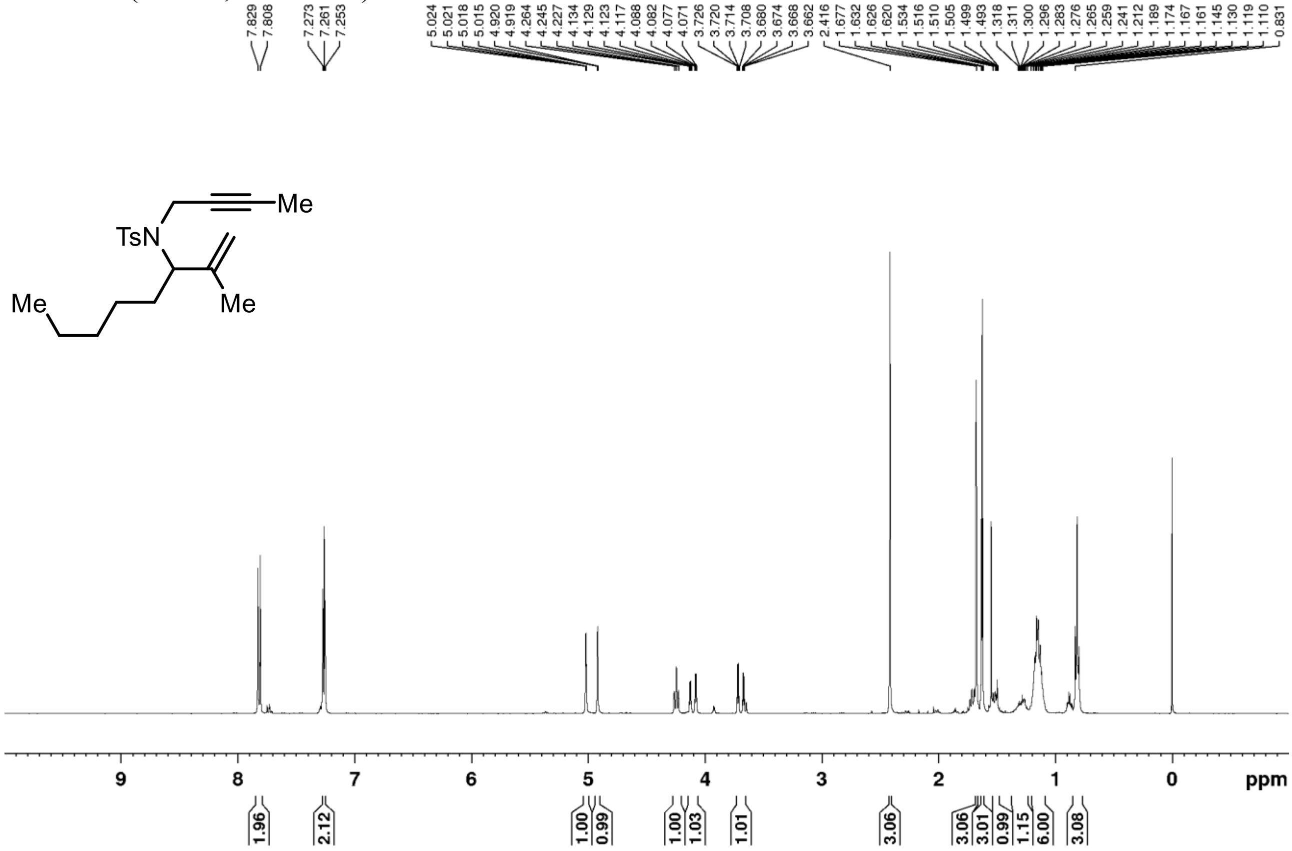

^{13}C NMR ($CDCl_3$, 101 MHz)

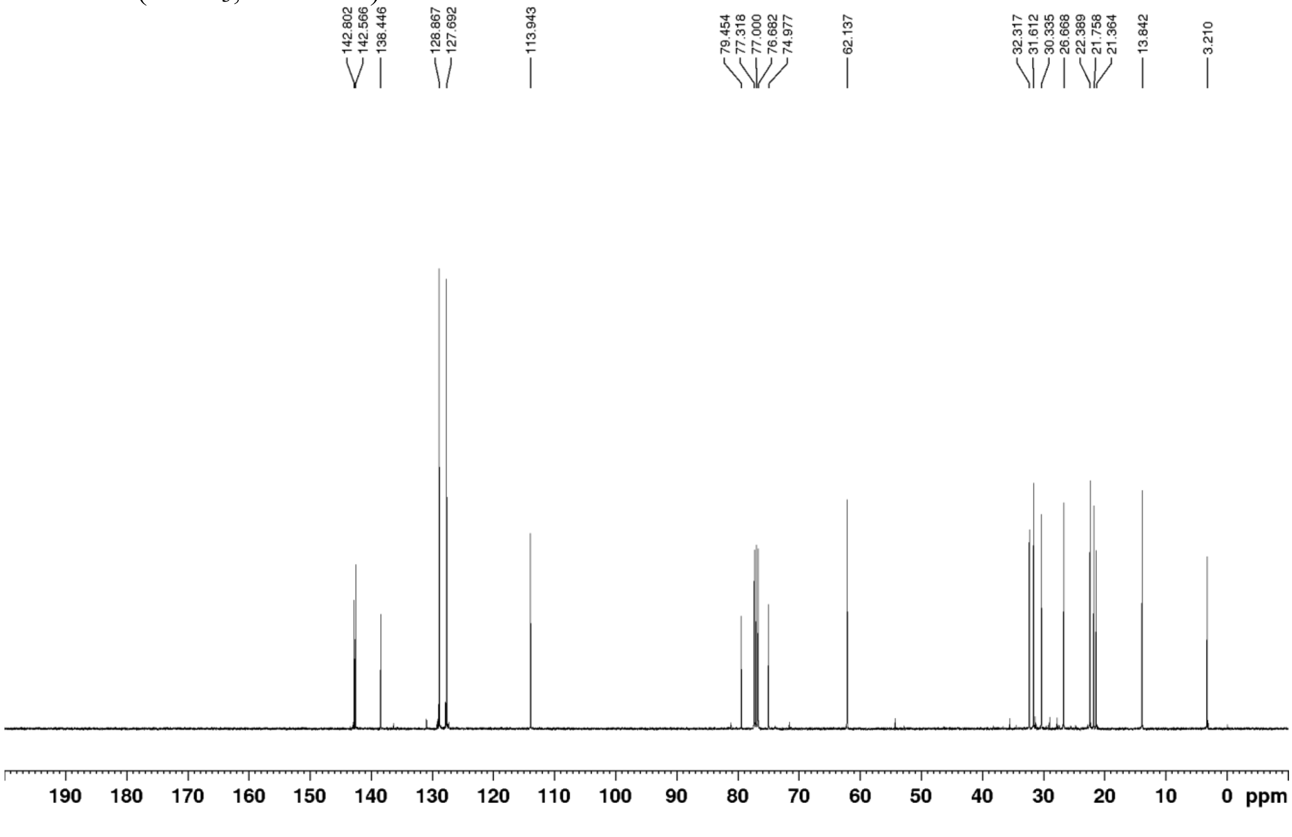

_N_-(But-2-yn-1-yl)-4-methyl-_N_-(2-methyl-1-phenylprop-2-en-1-yl)benzenesulfonamide (1c)

^{1}H NMR ($CDCl_3$, 400 MHz)

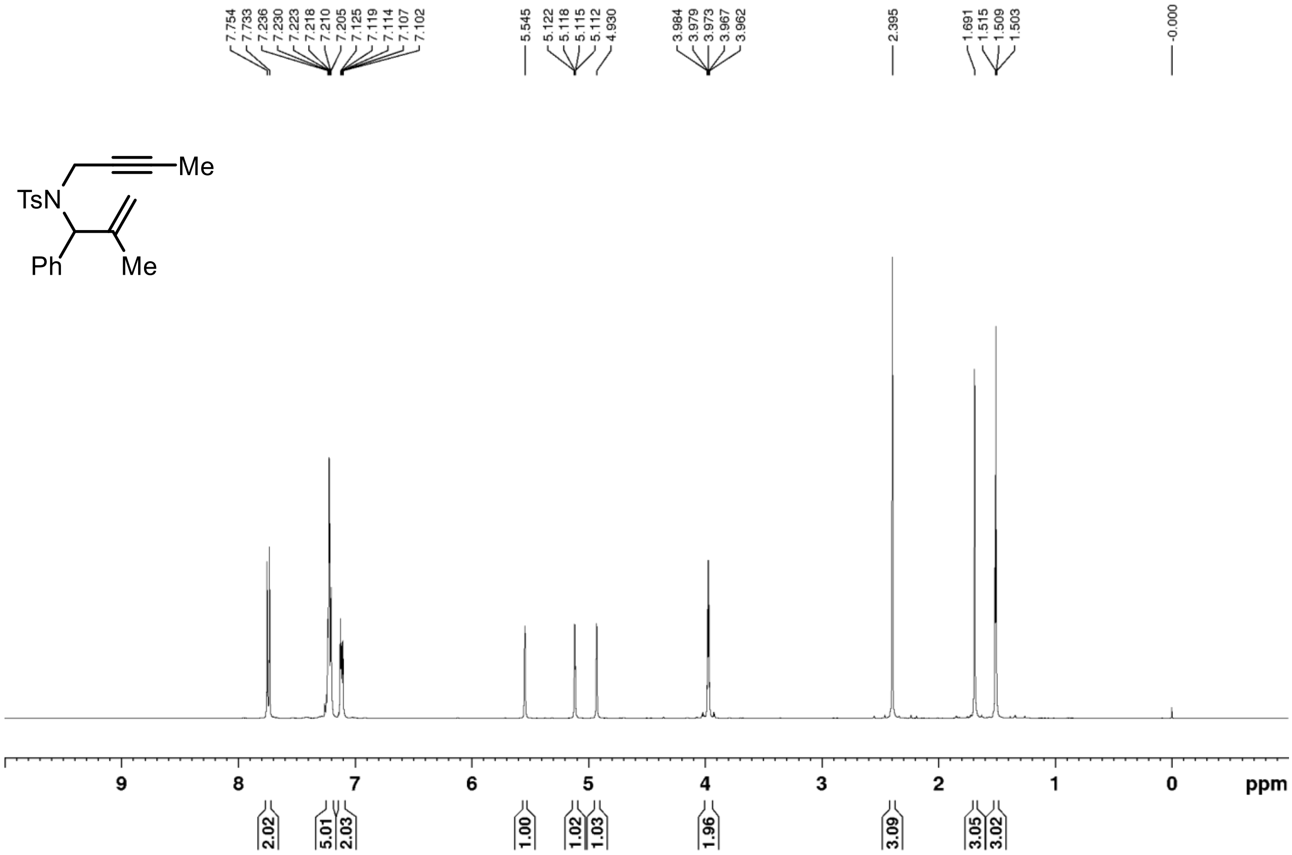

^{13}C NMR ($CDCl_3$, 101 MHz)

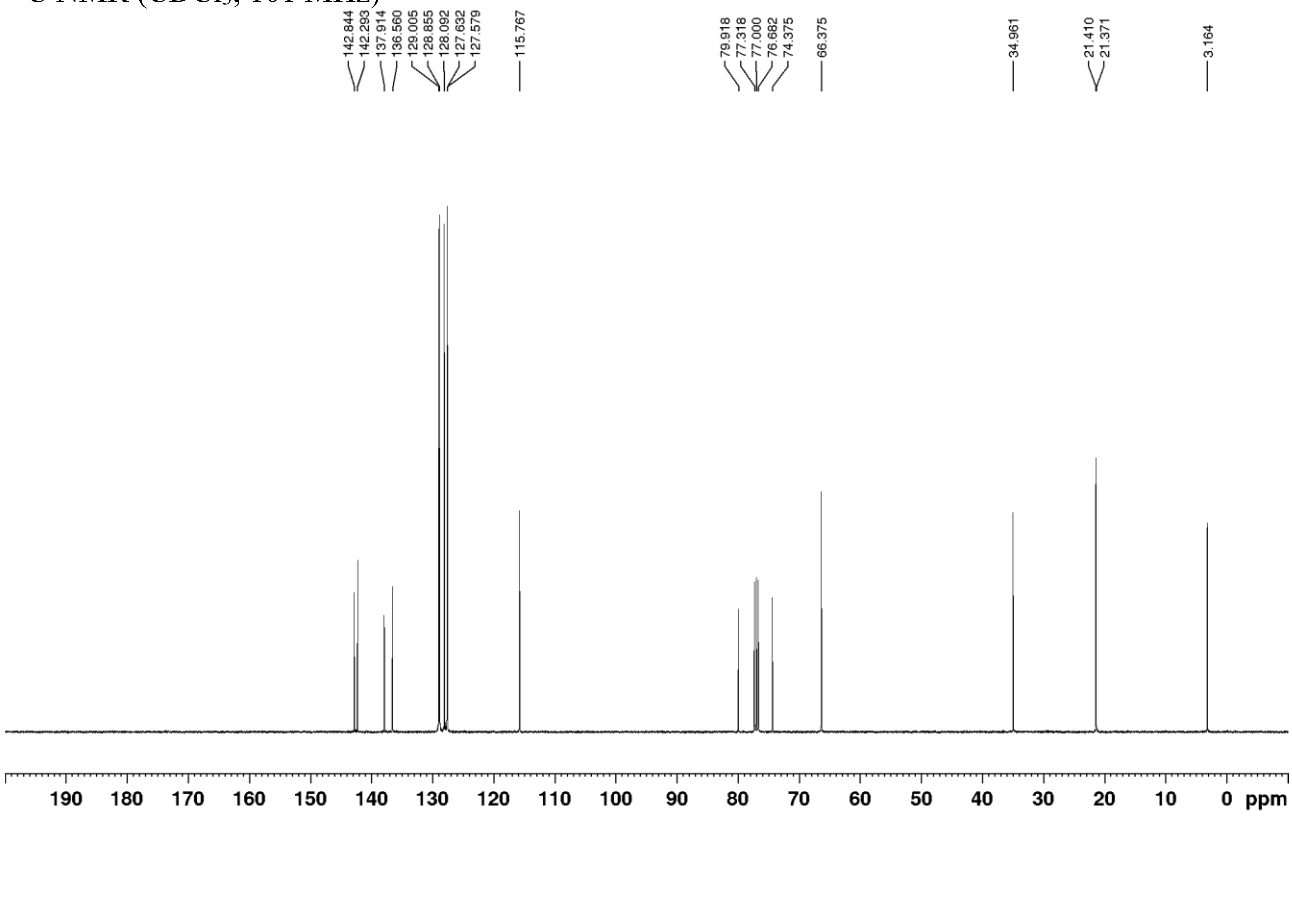

4-Methyl-*N*-(3-methylbut-3-en-2-yl)-*N*-(3-phenylprop-2-yn-1-yl)benzenesulfonamide (1d)

^{1}H NMR (CDCl$_3$, 400 MHz)

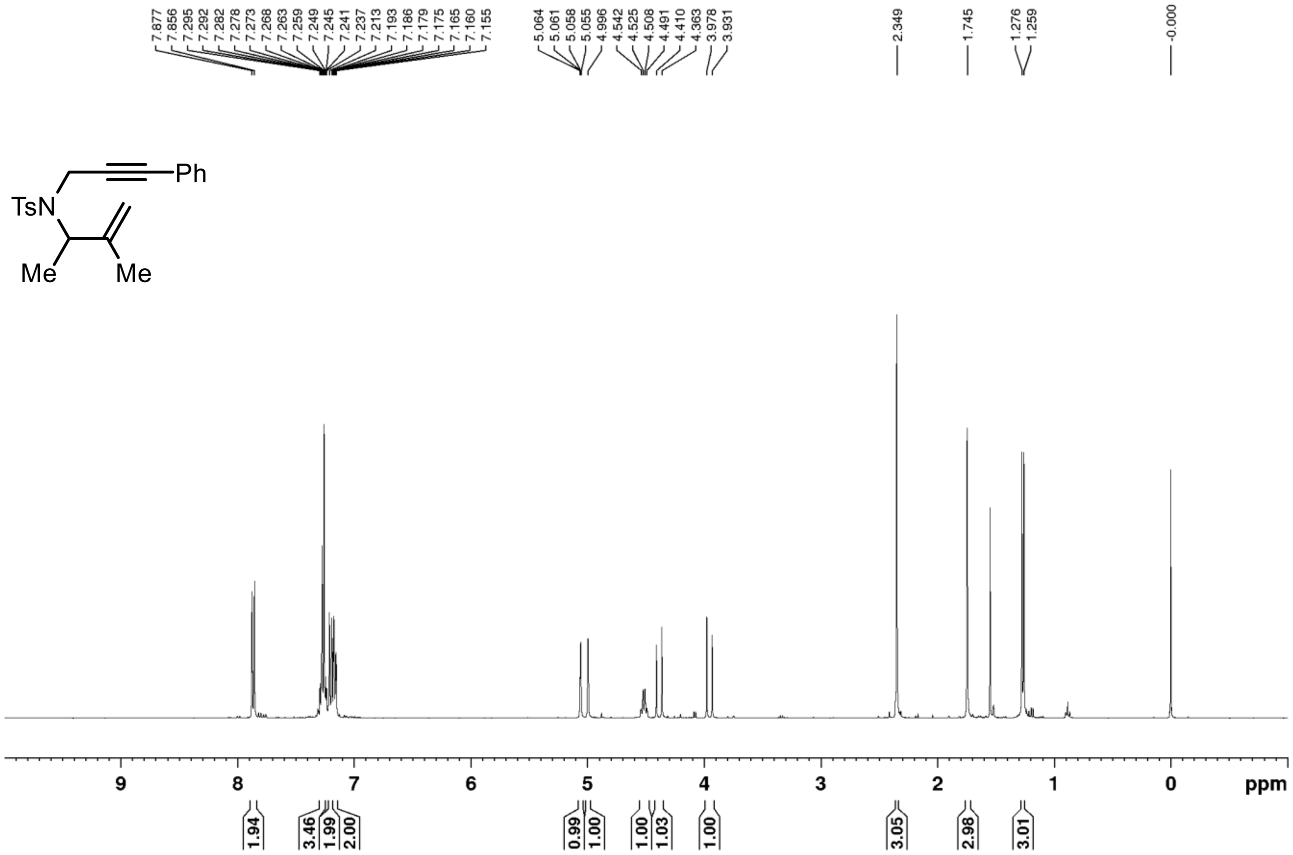

^{13}C NMR (CDCl$_3$, 101 MHz)

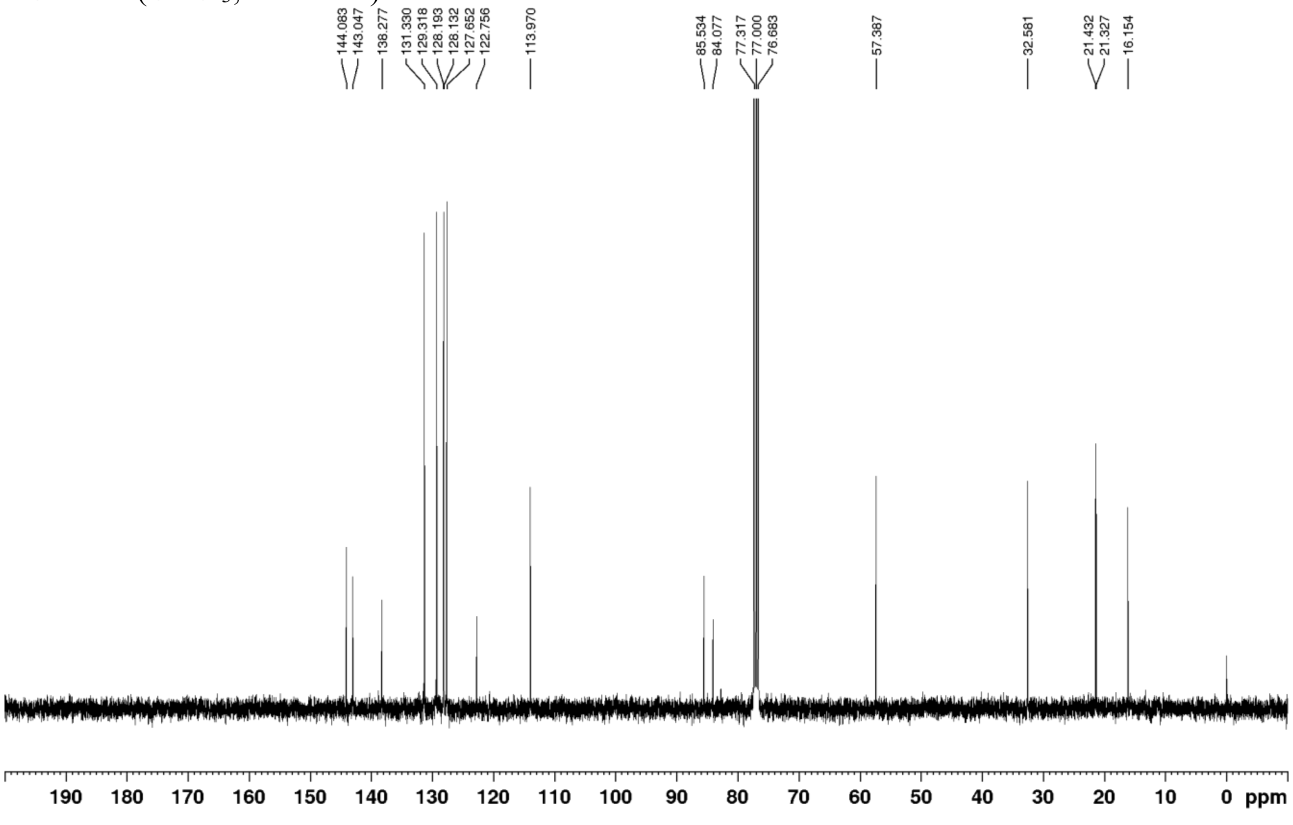

4-Methyl-*N*-(3-methylbut-3-en-2-yl)-*N*-(prop-2-yn-1-yl)benzenesulfonamide (S4)

^{1}H NMR ($CDCl_3$, 400 MHz)

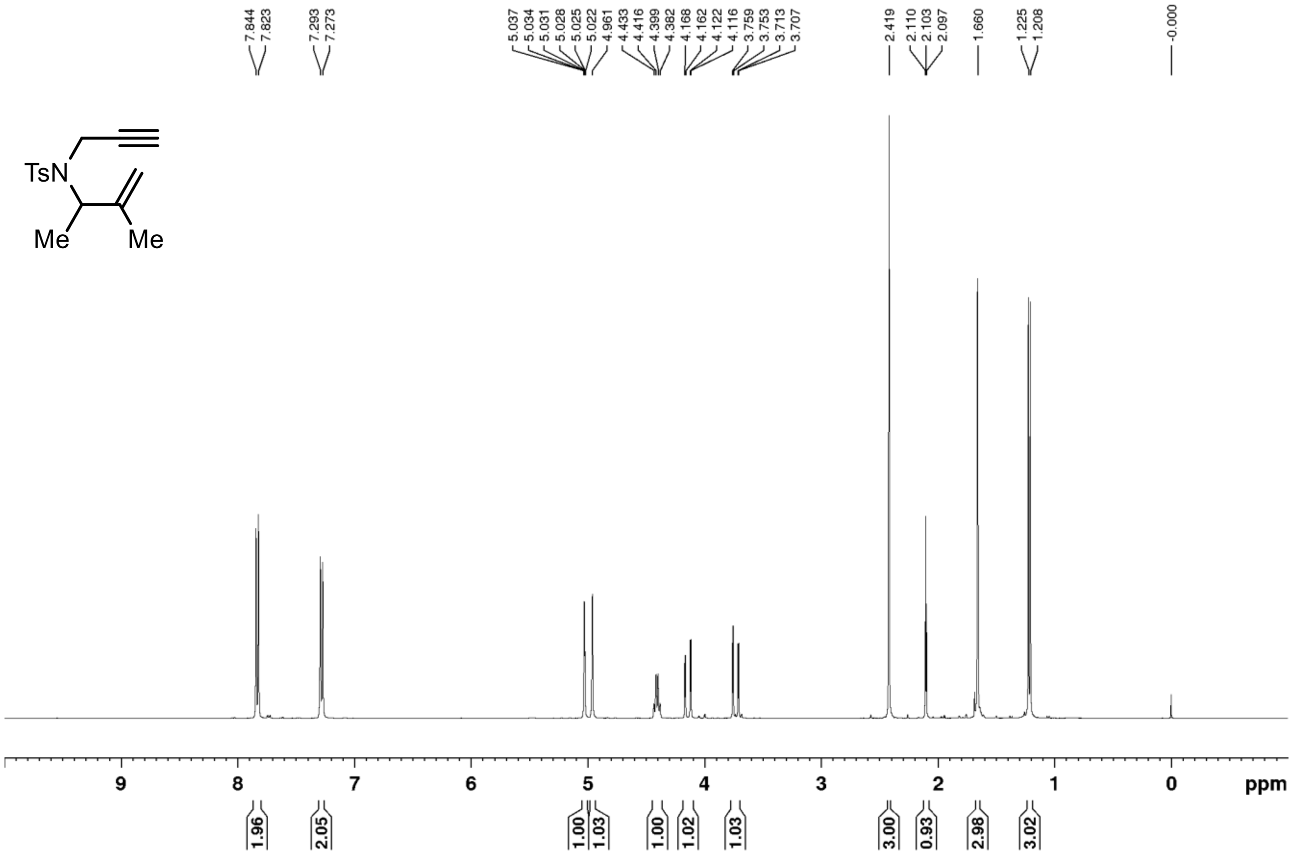

^{13}C NMR ($CDCl_3$, 101 MHz)

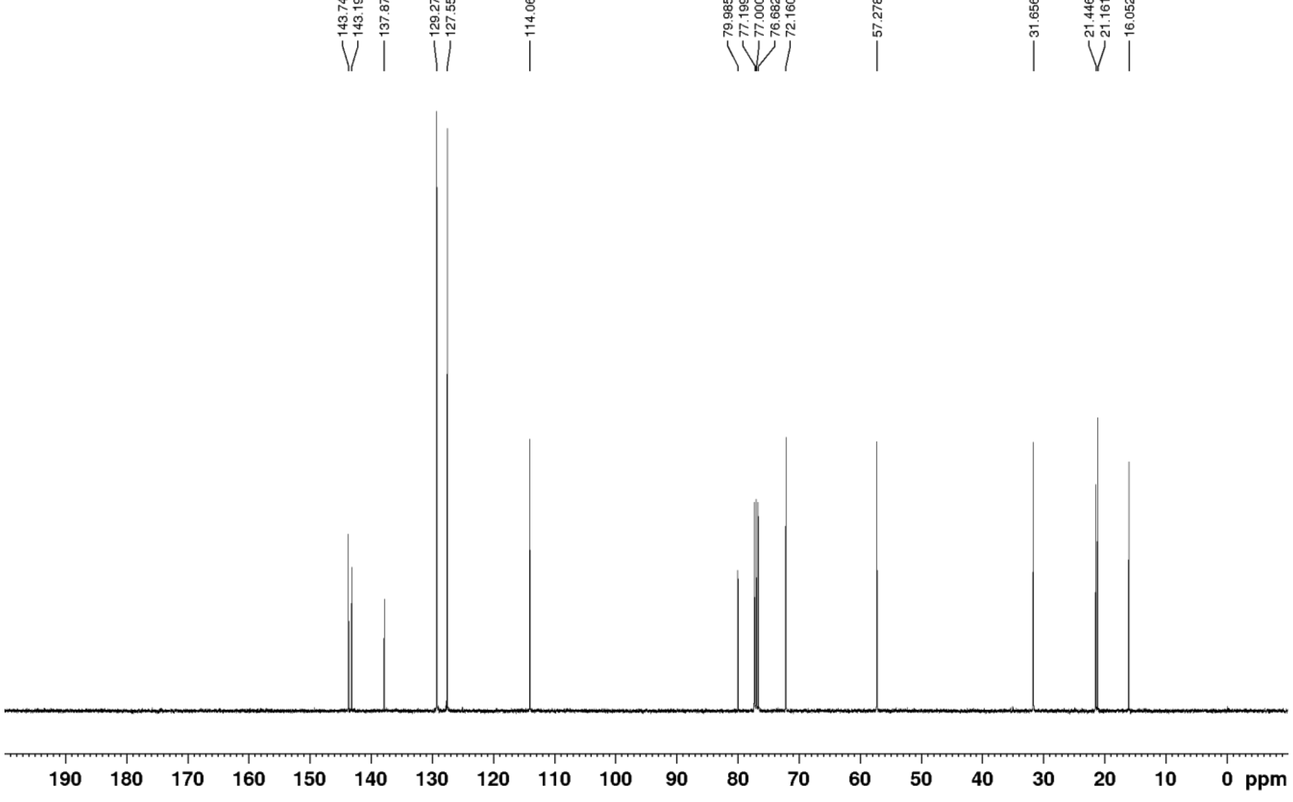

***N*-{3-(4-Methoxyphenyl)prop-2-yn-1-yl}-4-methyl-*N*-(3-methylbut-3-en-2-yl) benzenesulfonamide (1e)**

^{1}H NMR ($CDCl_3$, 400 MHz)

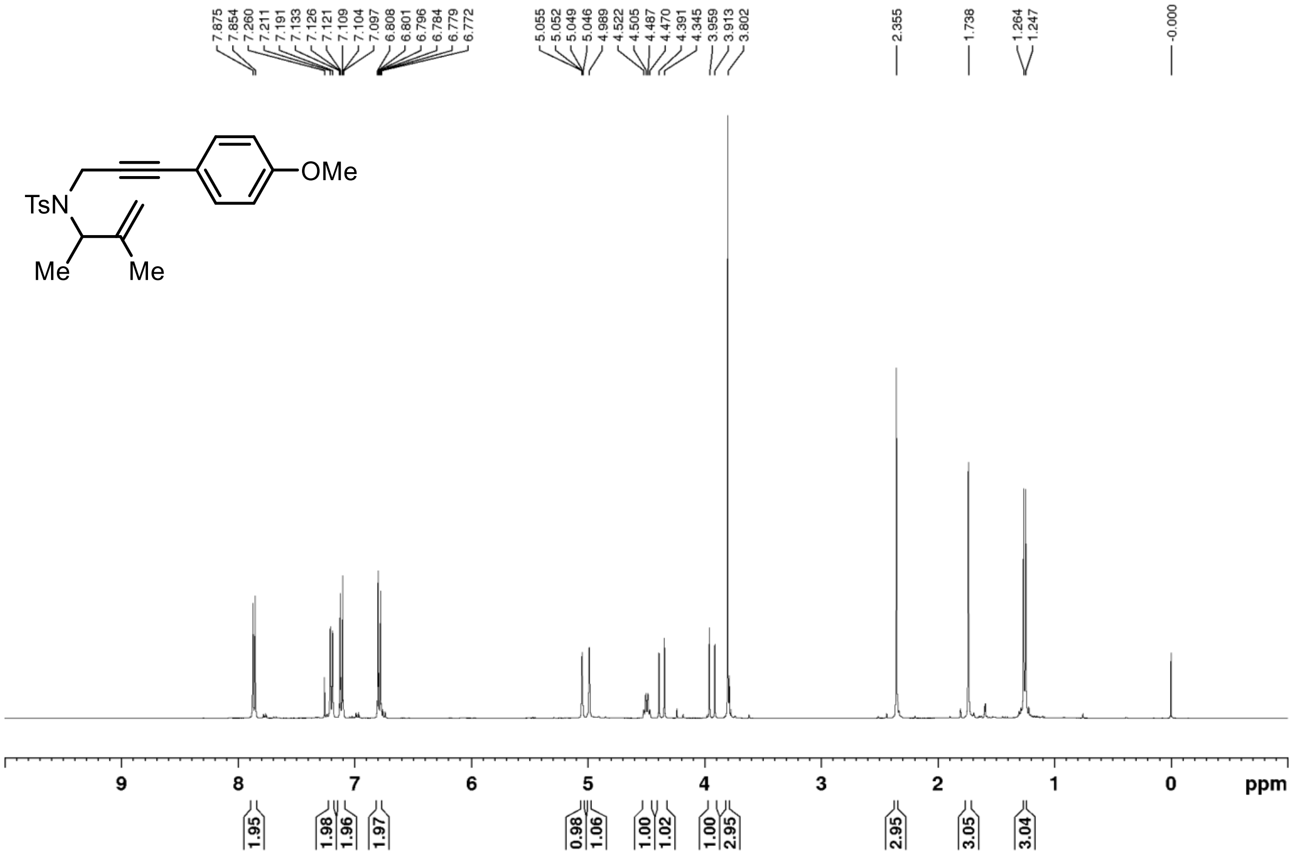

^{13}C NMR ($CDCl_3$, 101 MHz)

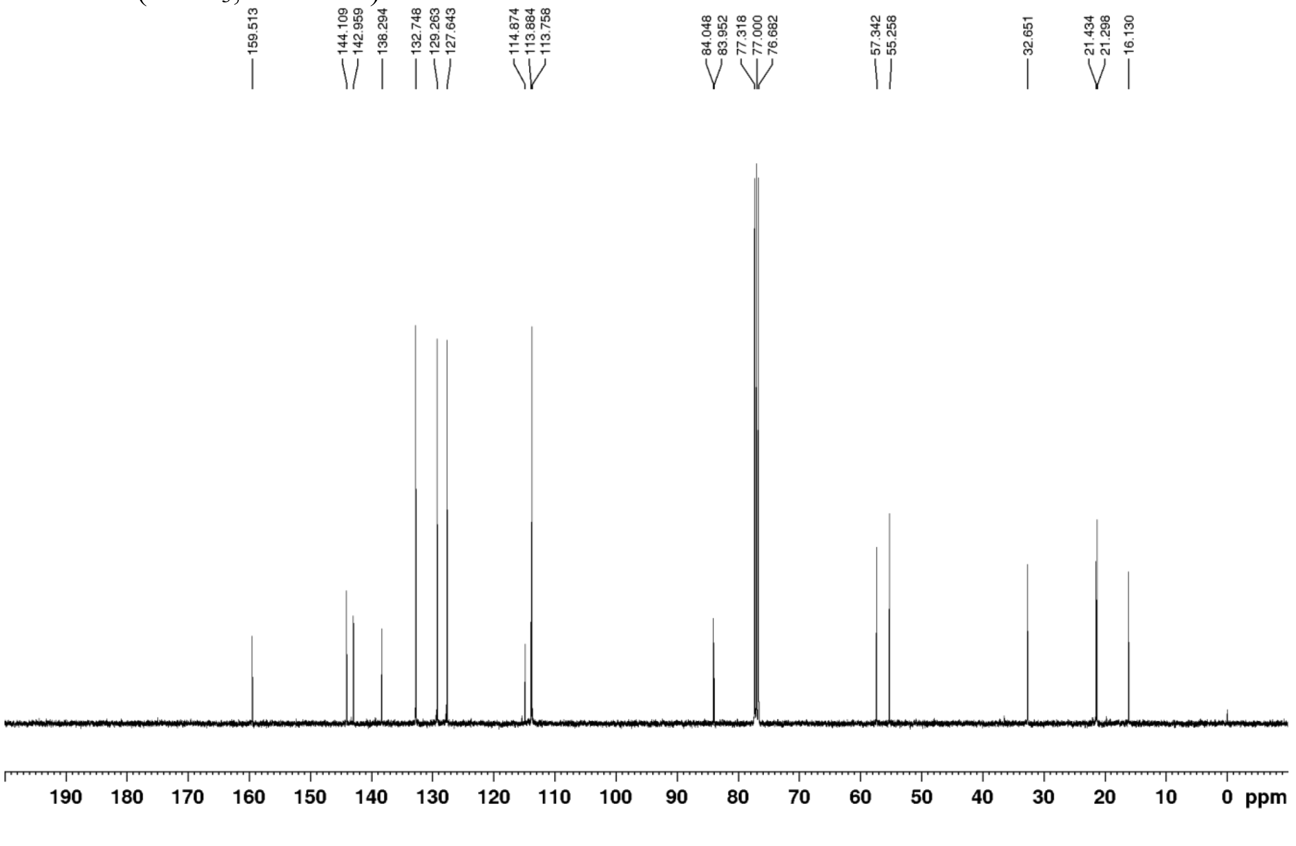

***N*-(But-3-yn-2-yl)-4-methylbenzenesulfonamide (S1e)**

^{1}H NMR ($CDCl_3$, 400 MHz)

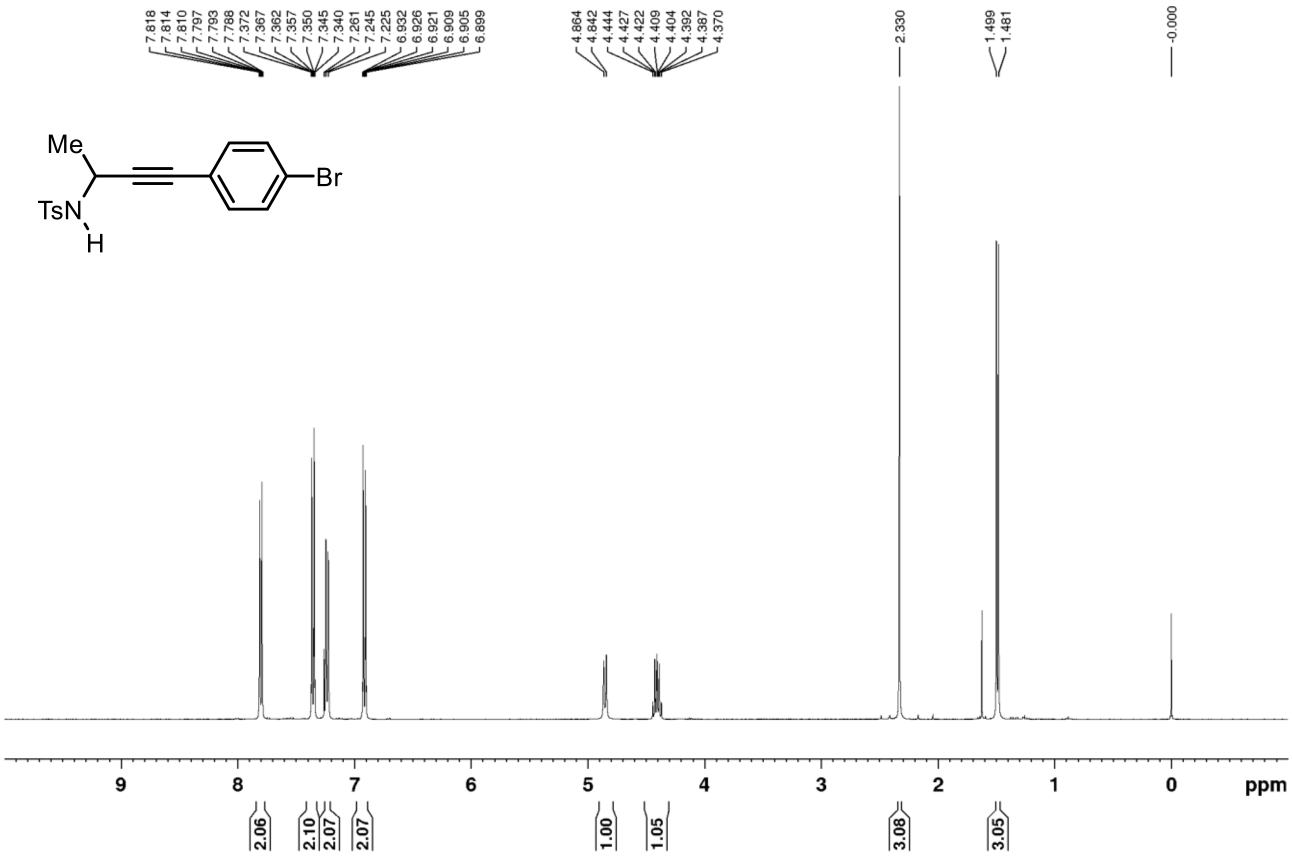

^{13}C NMR ($CDCl_3$, 101 MHz)

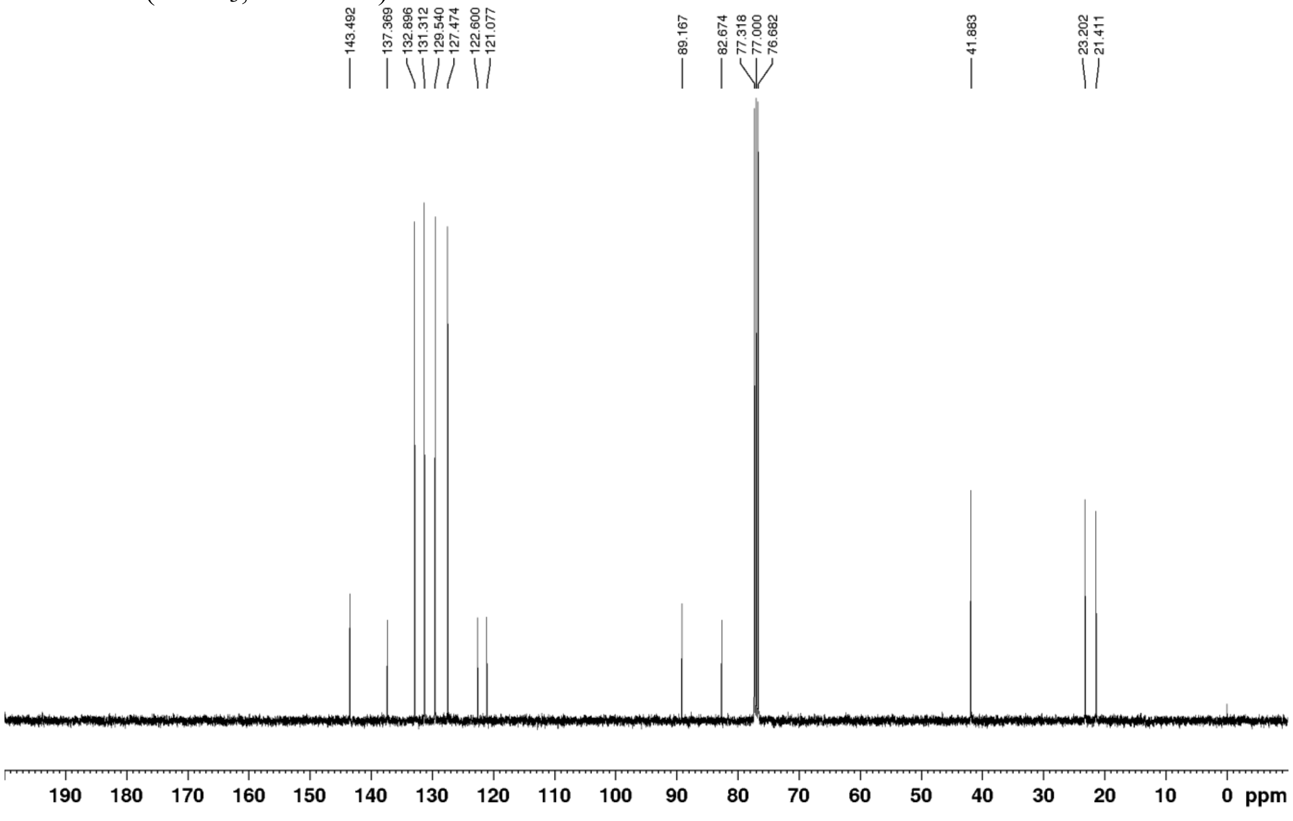

N-{4-(4-Bromophenyl)but-3-yn-2-yl}-4-methyl-N-(prop-2-en-1-yl)benzenesulfonamide (1f)

^{1}H NMR (CDCl$_3$, 400 MHz)

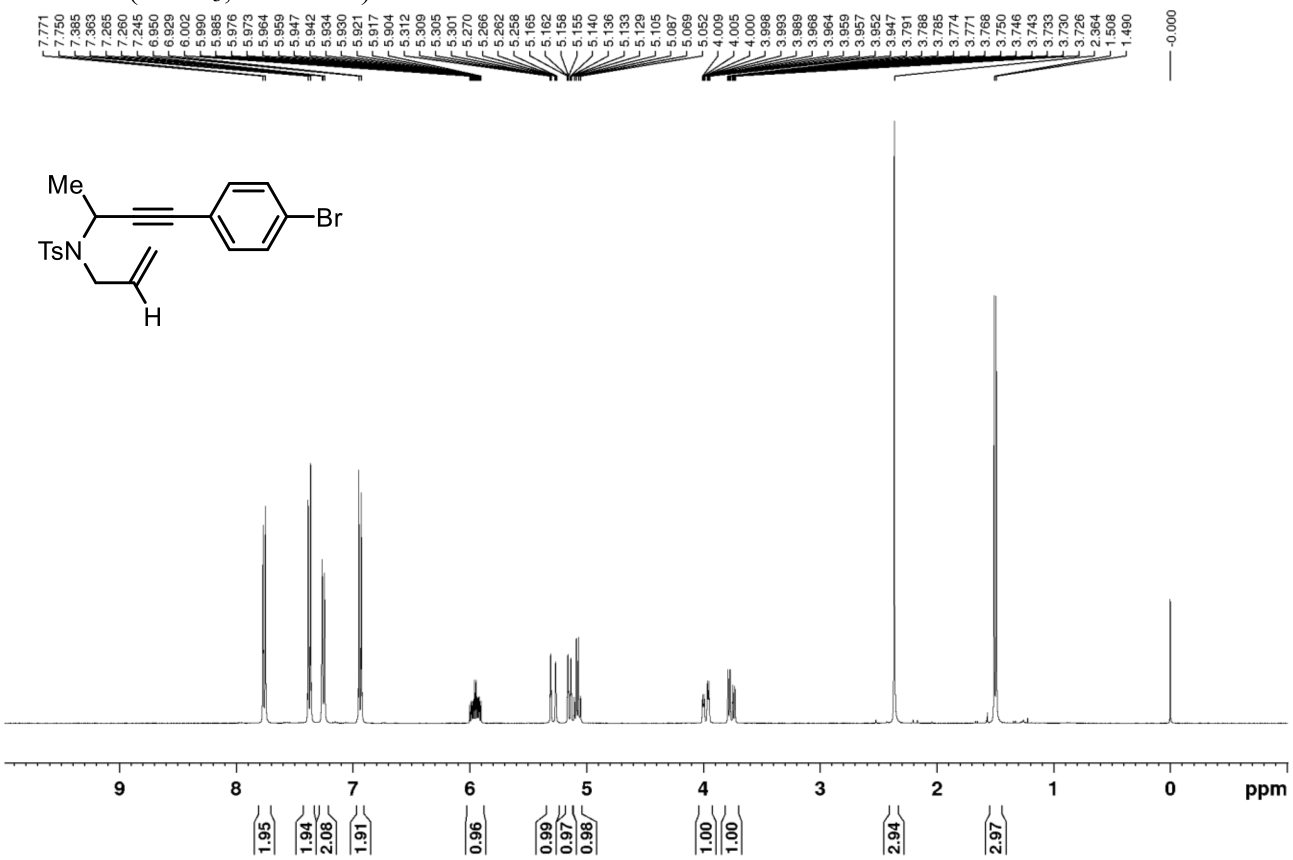

^{13}C NMR (CDCl$_3$, 101 MHz)

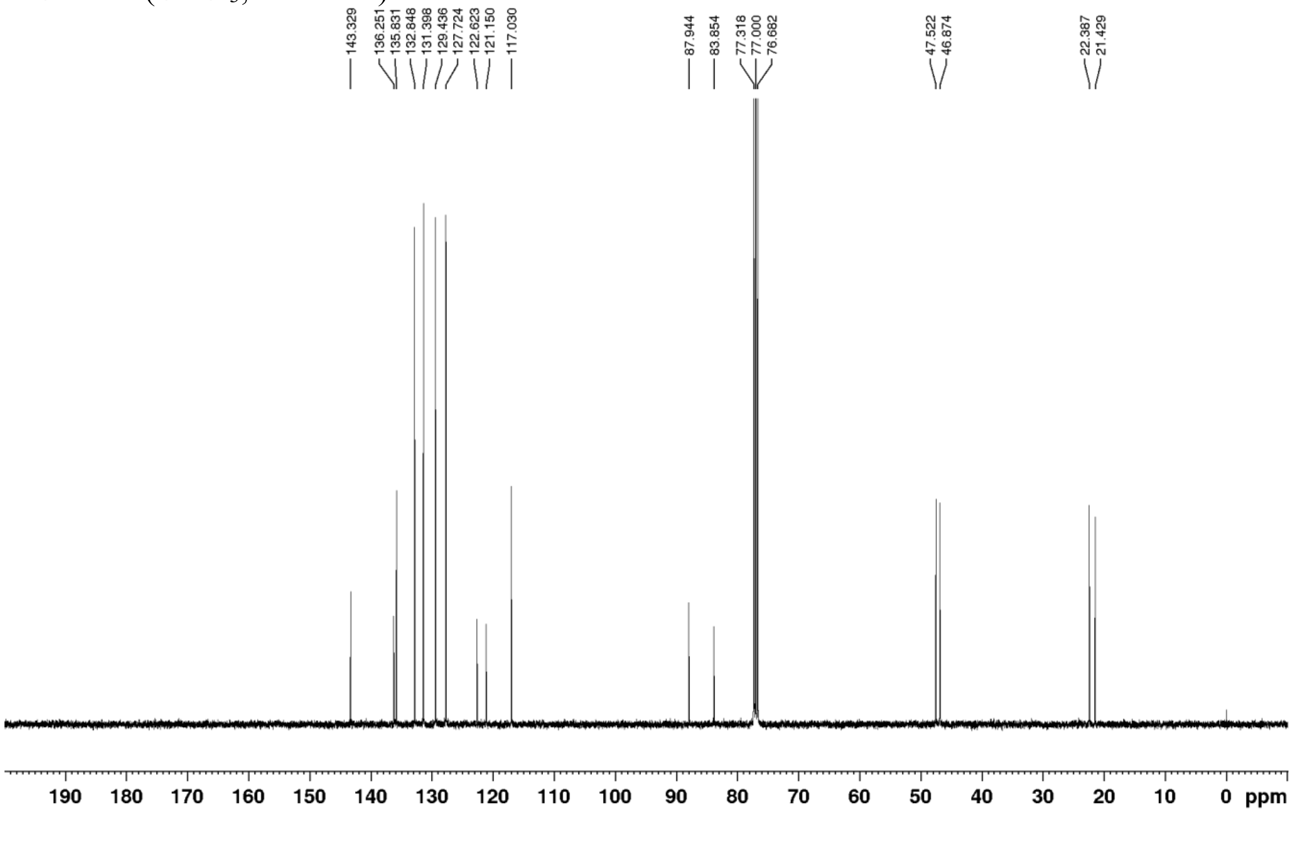

N-Allyl-N-(1,3-diphenylprop-2-yn-1-yl)-4-methylbenzenesulfonamide (1g)

^{1}H NMR ($CDCl_3$, 400 MHz)

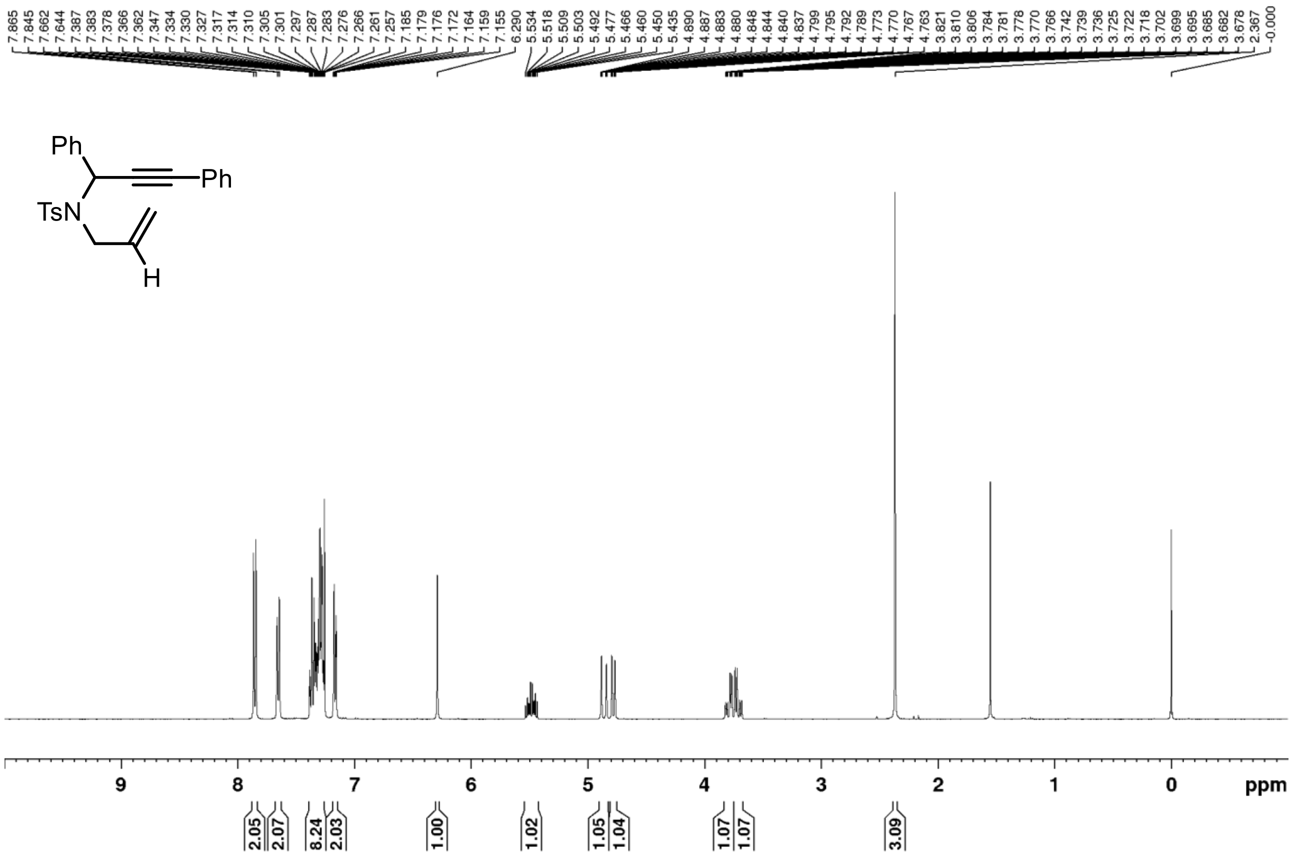

^{13}C NMR ($CDCl_3$, 101 MHz)

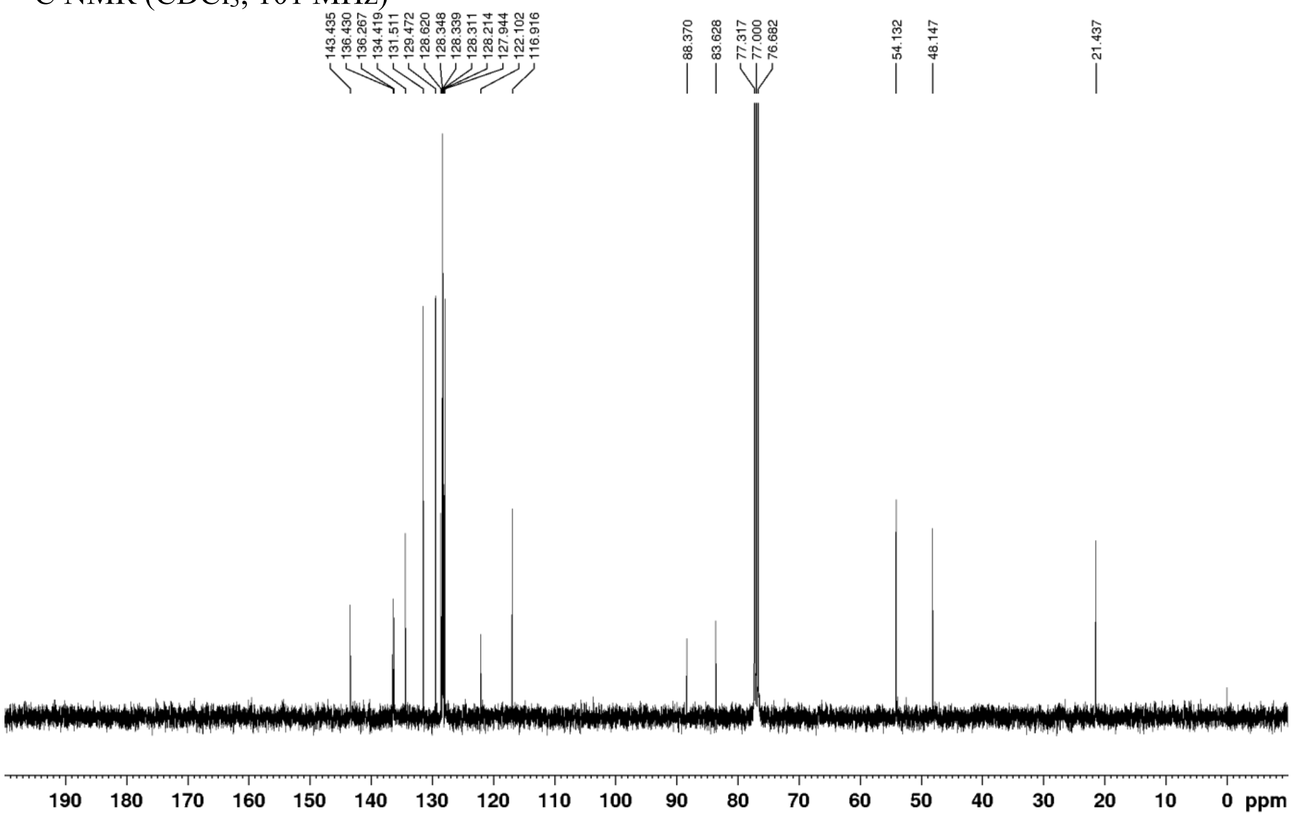

3-Methylbut-3-en-2-yl 3-phenylpropiynoate (1h)

^{1}H NMR ($CDCl_3$, 400 MHz)

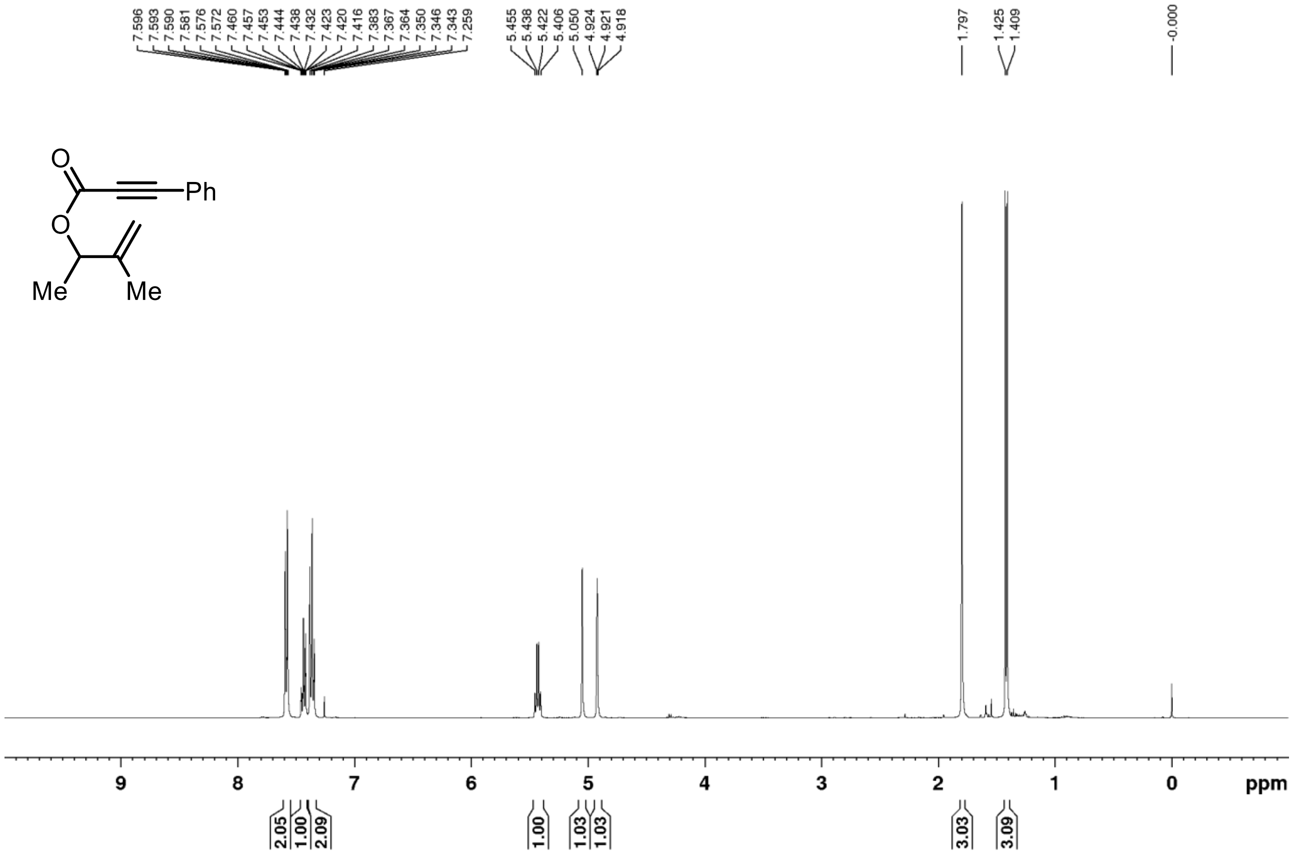

^{13}C NMR ($CDCl_3$, 101 MHz)

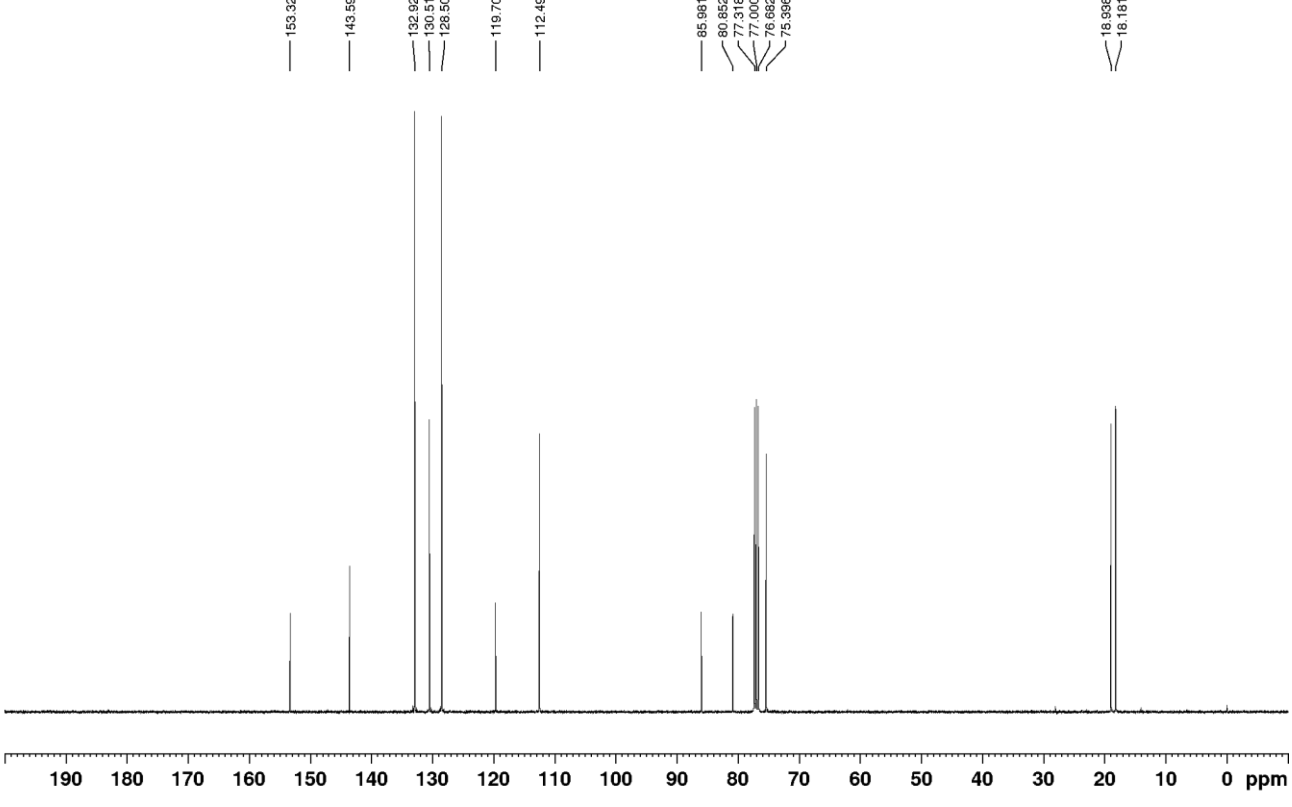

3-Methylbut-3-en-2-yl 3-(4-bromophenyl)propynoate (1i)

^{1}H NMR ($CDCl_3$, 400 MHz)

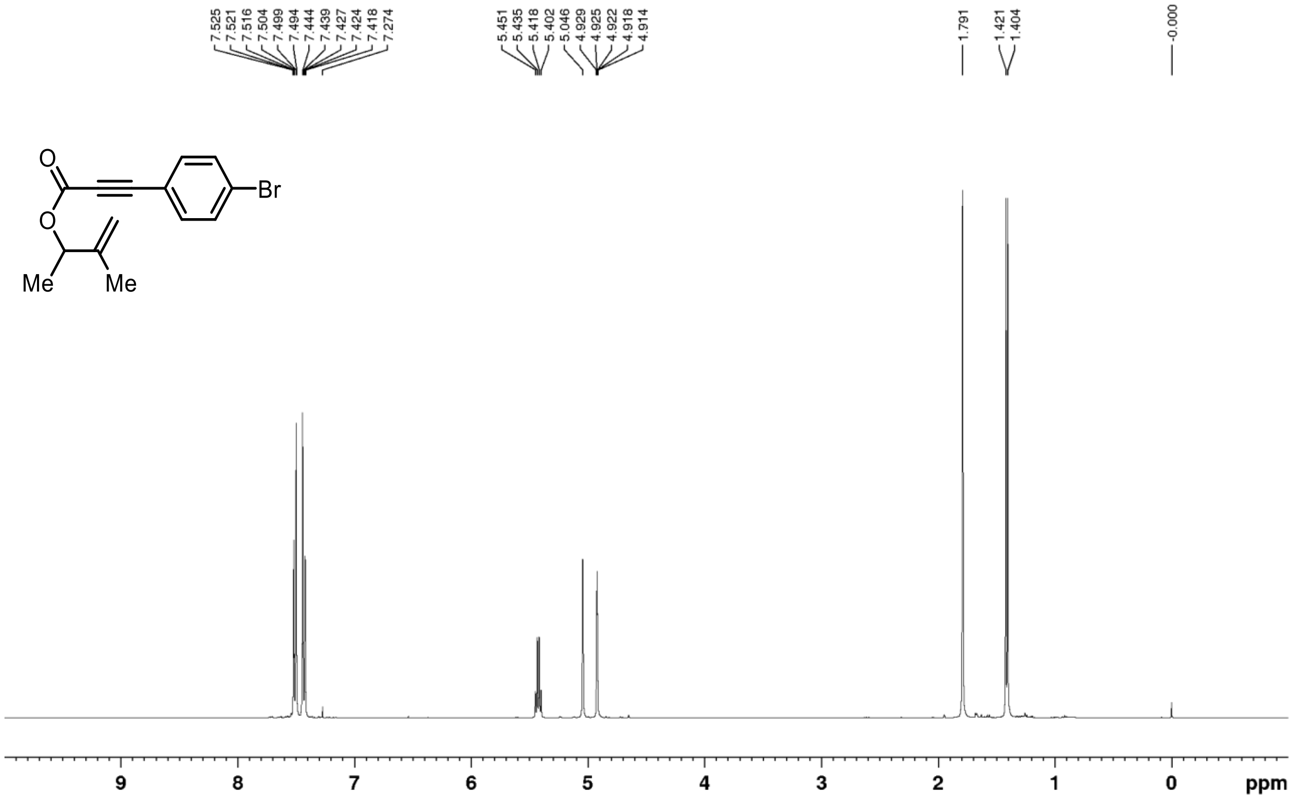

^{13}C NMR ($CDCl_3$, 101 MHz)

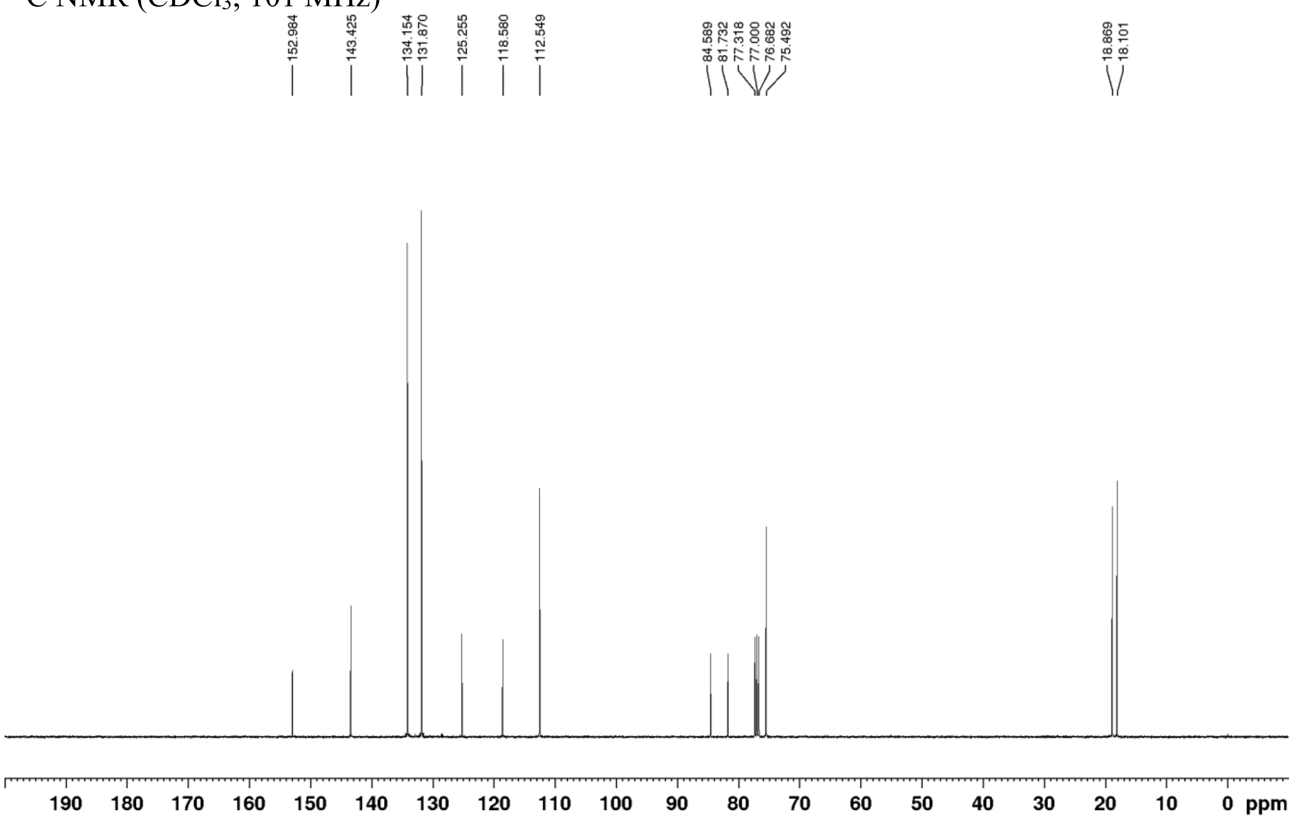

2-Methyl-1-phenylprop-2-en-1-yl 3-phenylpropynoate (1j)

^{1}H NMR ($CDCl_3$, 400 MHz)

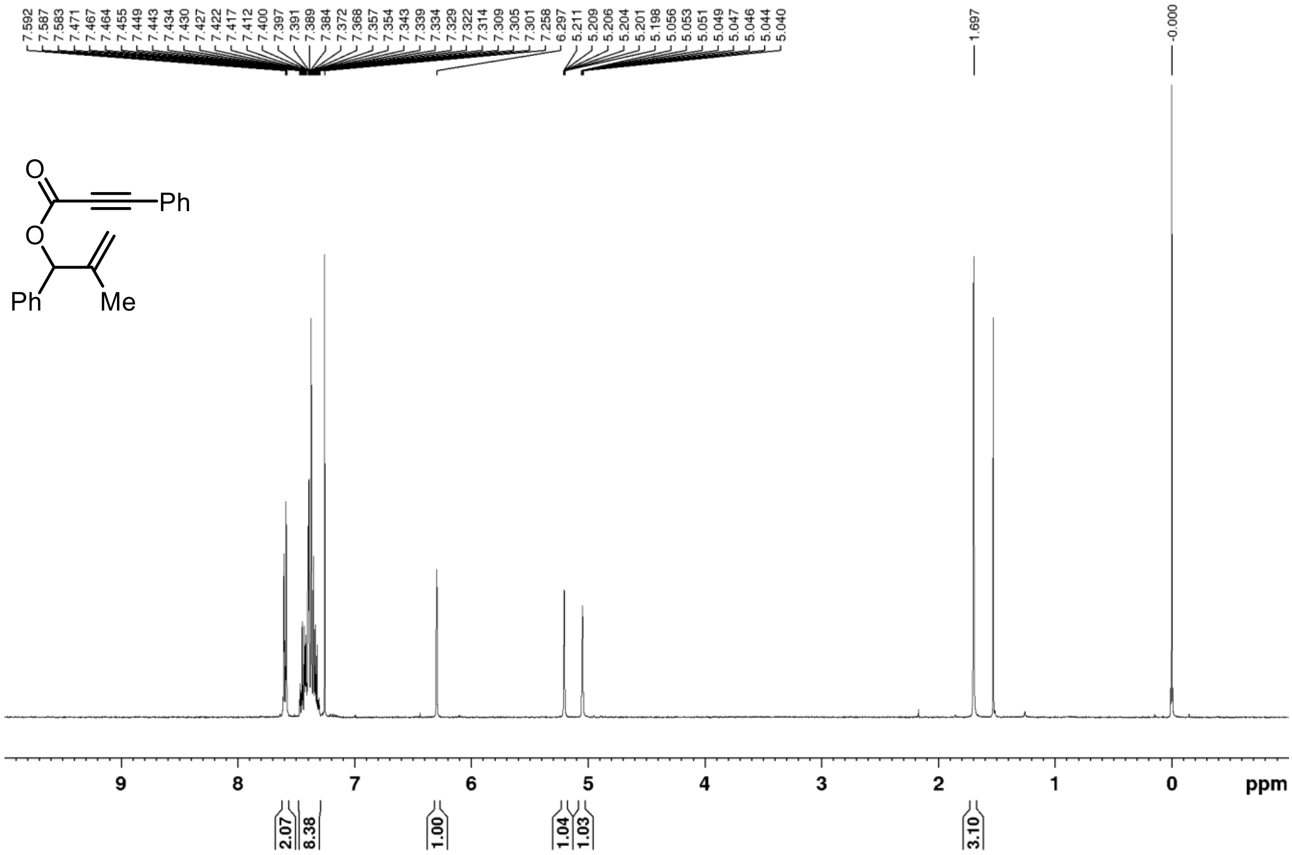

^{13}C NMR ($CDCl_3$, 101 MHz)

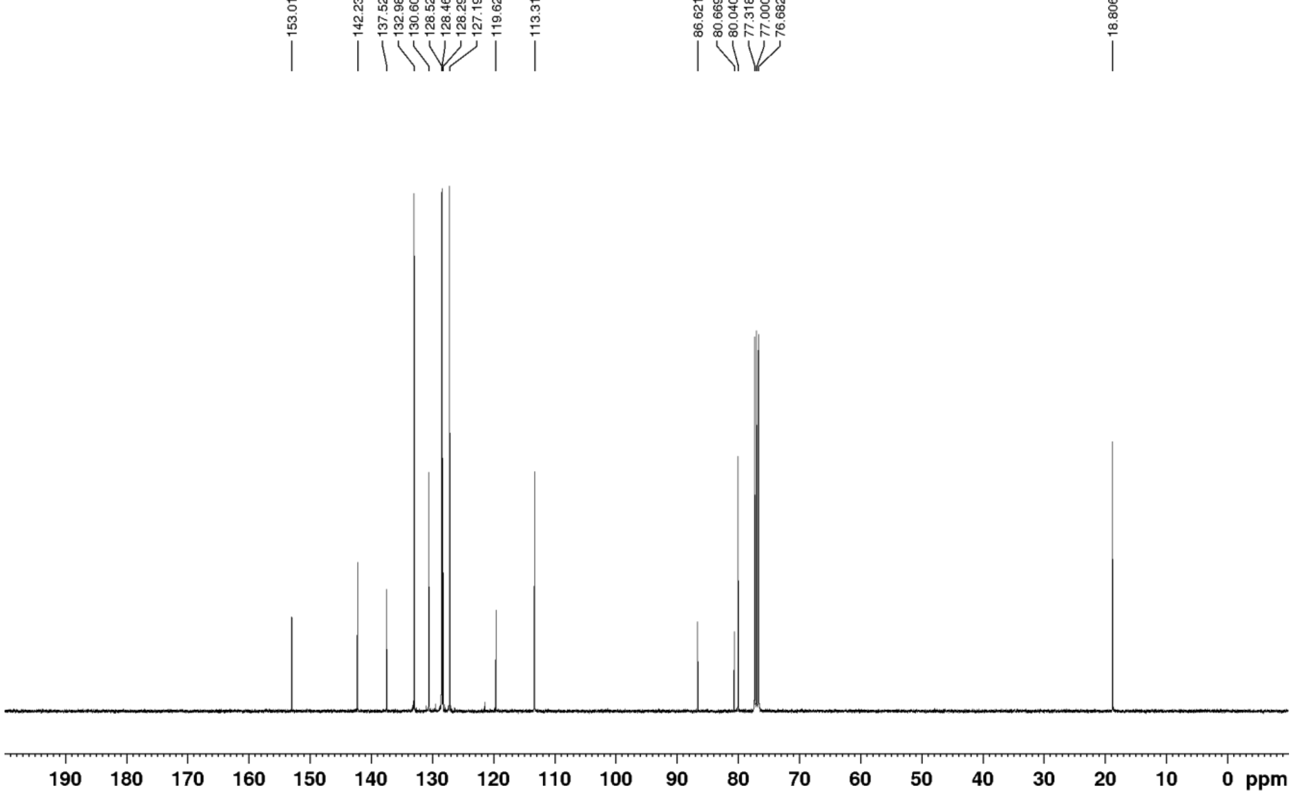

3-Phenyl-1-{2-(prop-1-en-2-yl)indolin-1-yl}prop-2-yn-1-one (1k)

^{1}H NMR ($CDCl_3$, 400 MHz)

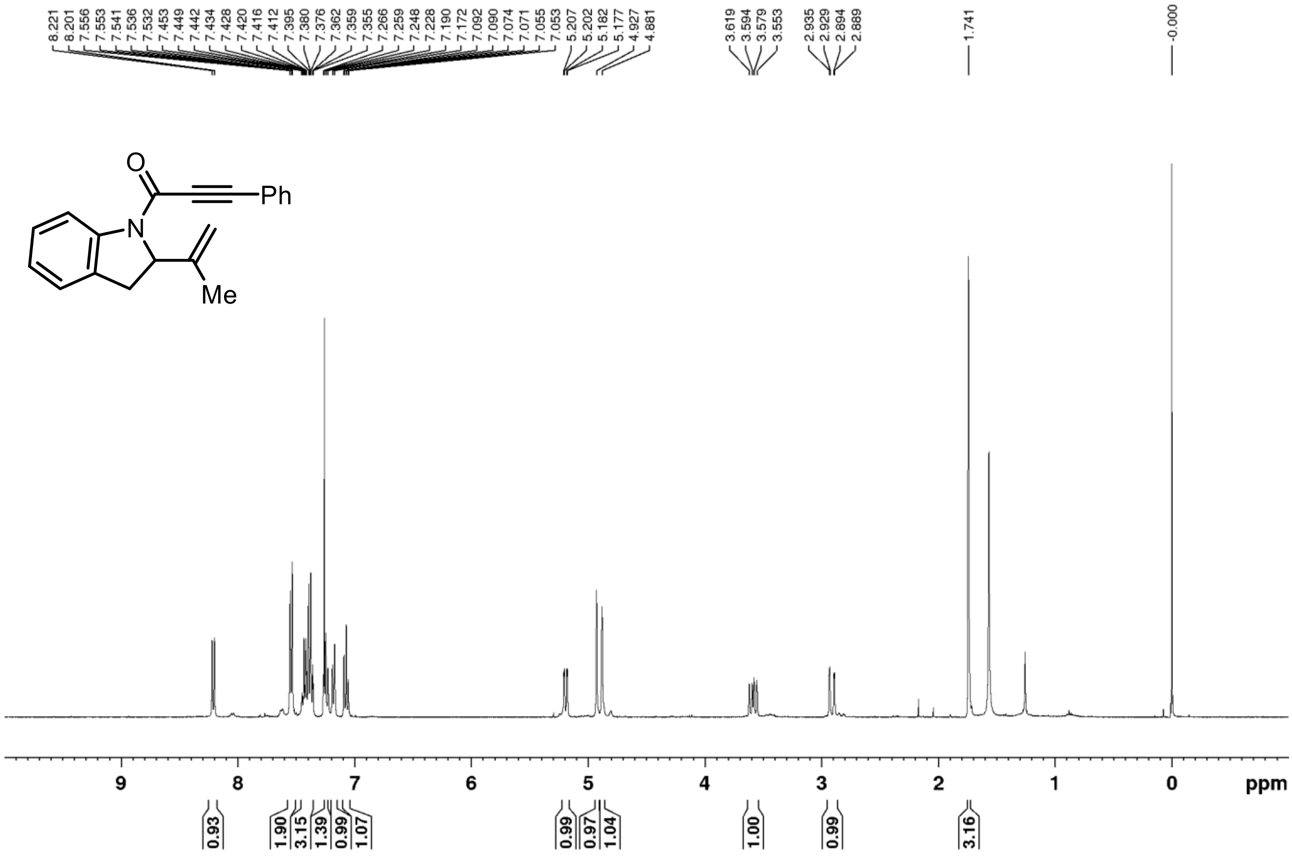

^{13}C NMR ($CDCl_3$, 101 MHz)

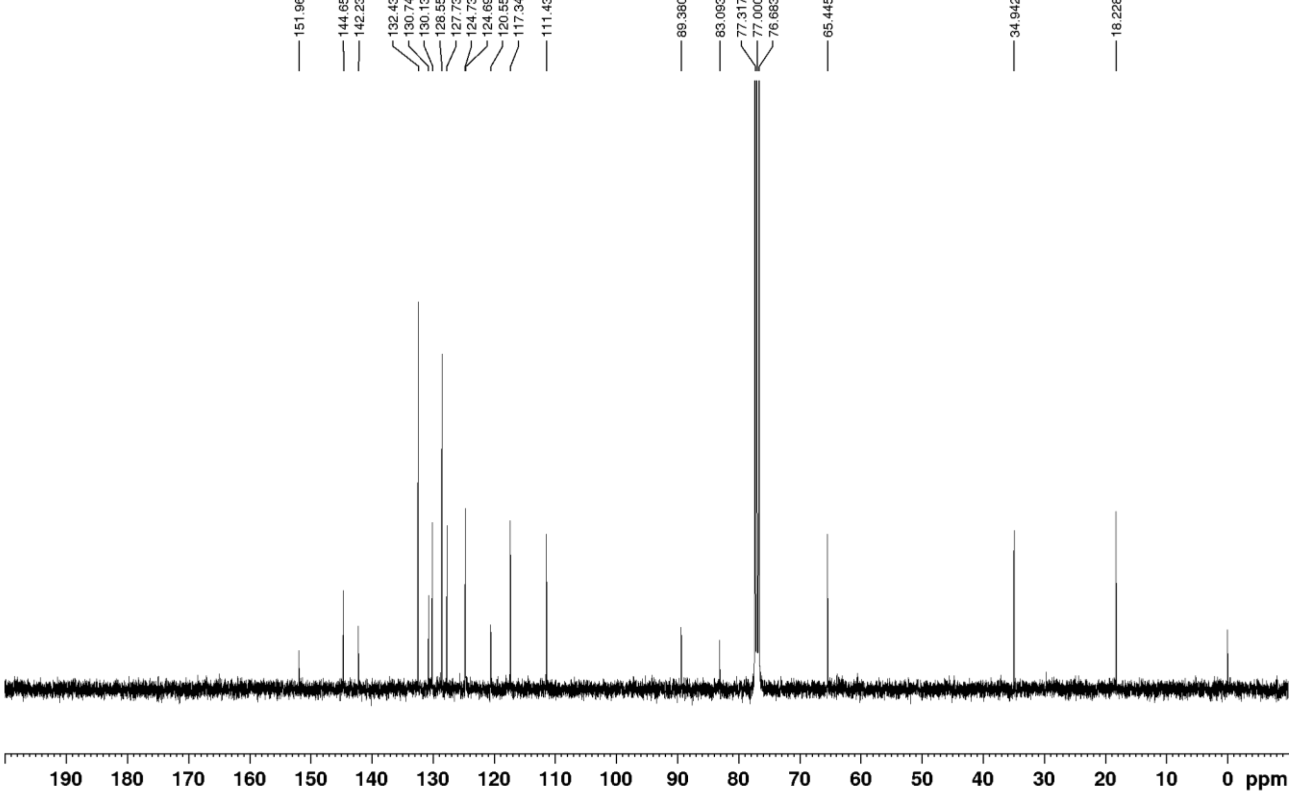

[3-{(3-Methylbut-3-en-2-yl)oxy}prop-1-yn-1-yl]benzene (1l)

^{1}H NMR ($CDCl_3$, 400 MHz)

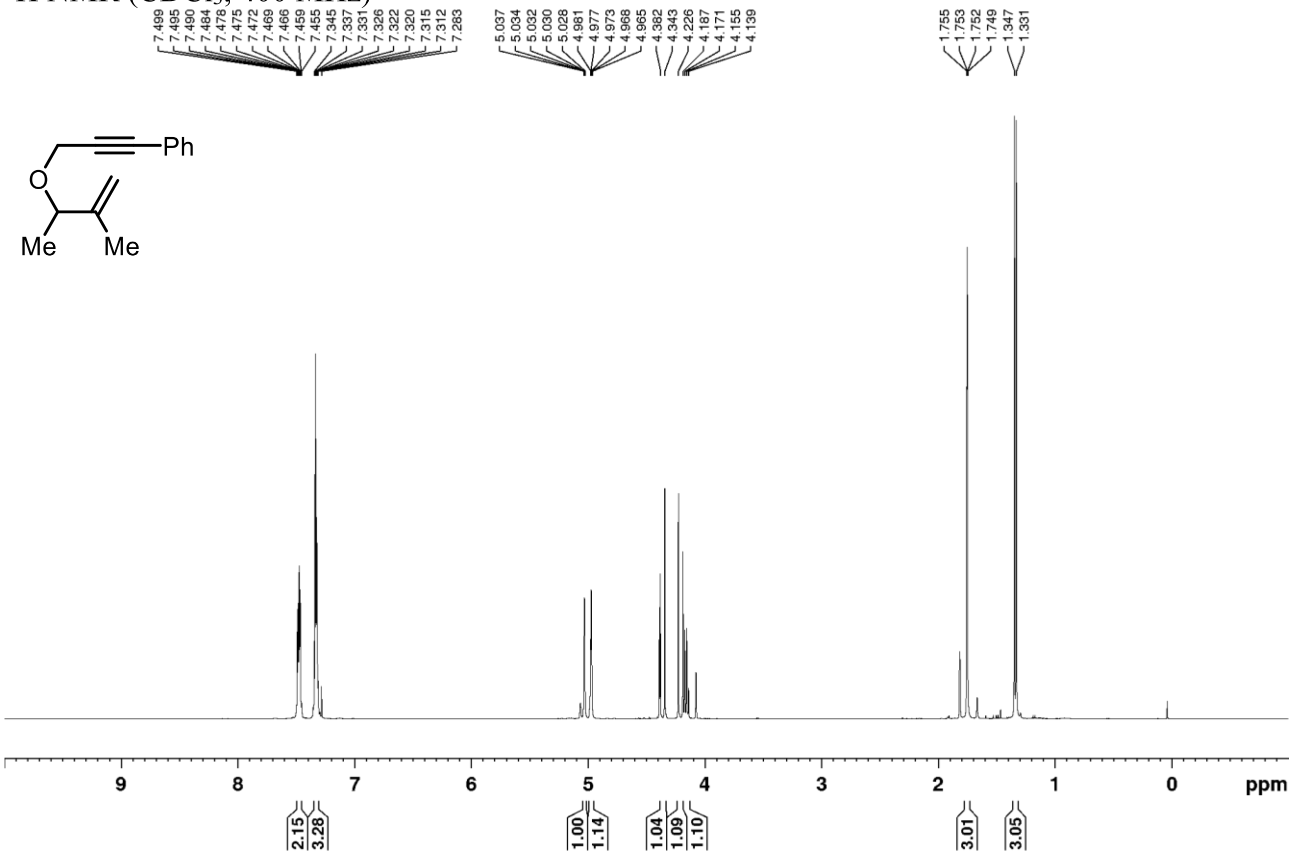

^{13}C NMR ($CDCl_3$, 101 MHz)

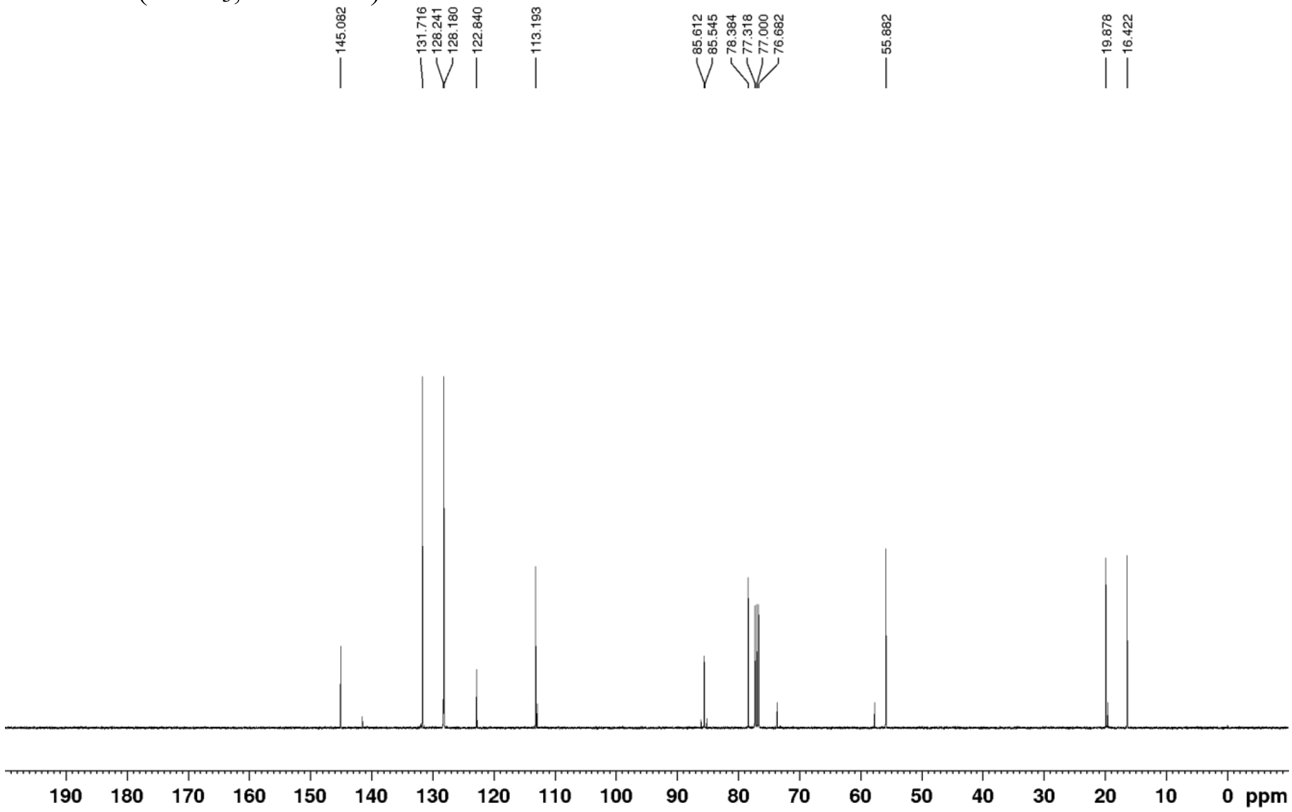

3-Phenylbut-3-en-2-yl 3-phenylpropynoate (1m)

^{1}H NMR ($CDCl_3$, 400 MHz)

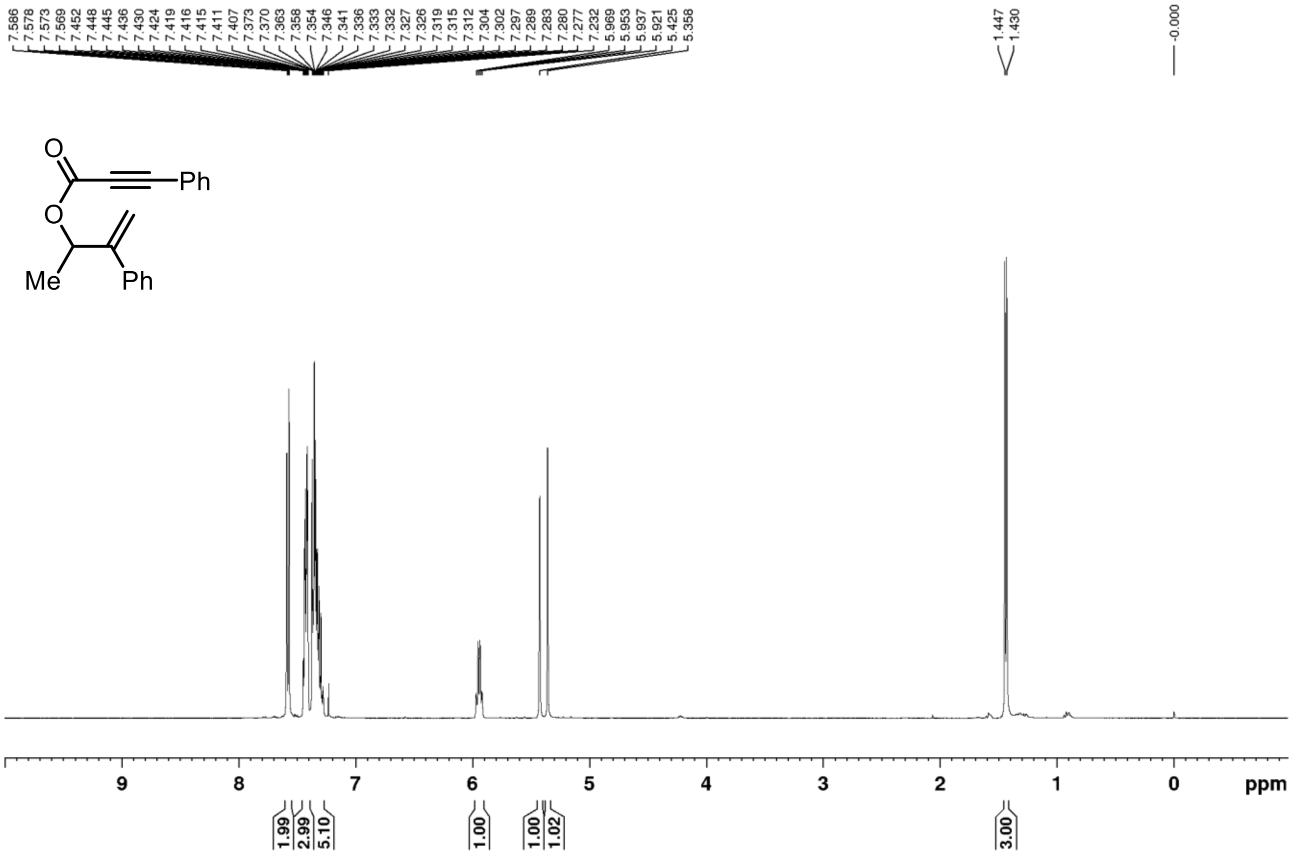

^{13}C NMR ($CDCl_3$, 101 MHz)

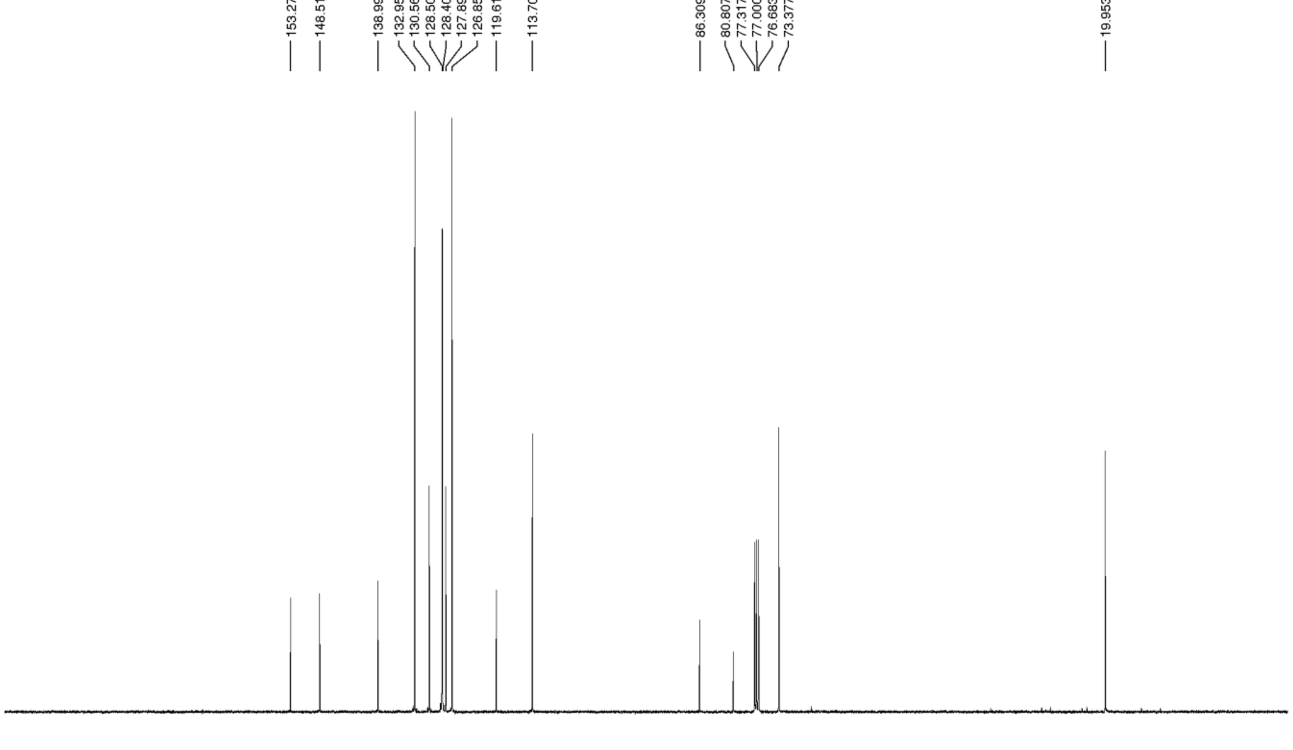

N-{3-(4-Chlorophenyl)prop-2-yn-1-yl}-4-methyl-N-(penta-1,4-dien-3-yl) benzenesulfonamide (5c)

^{1}H NMR ($CDCl_3$, 400 MHz)

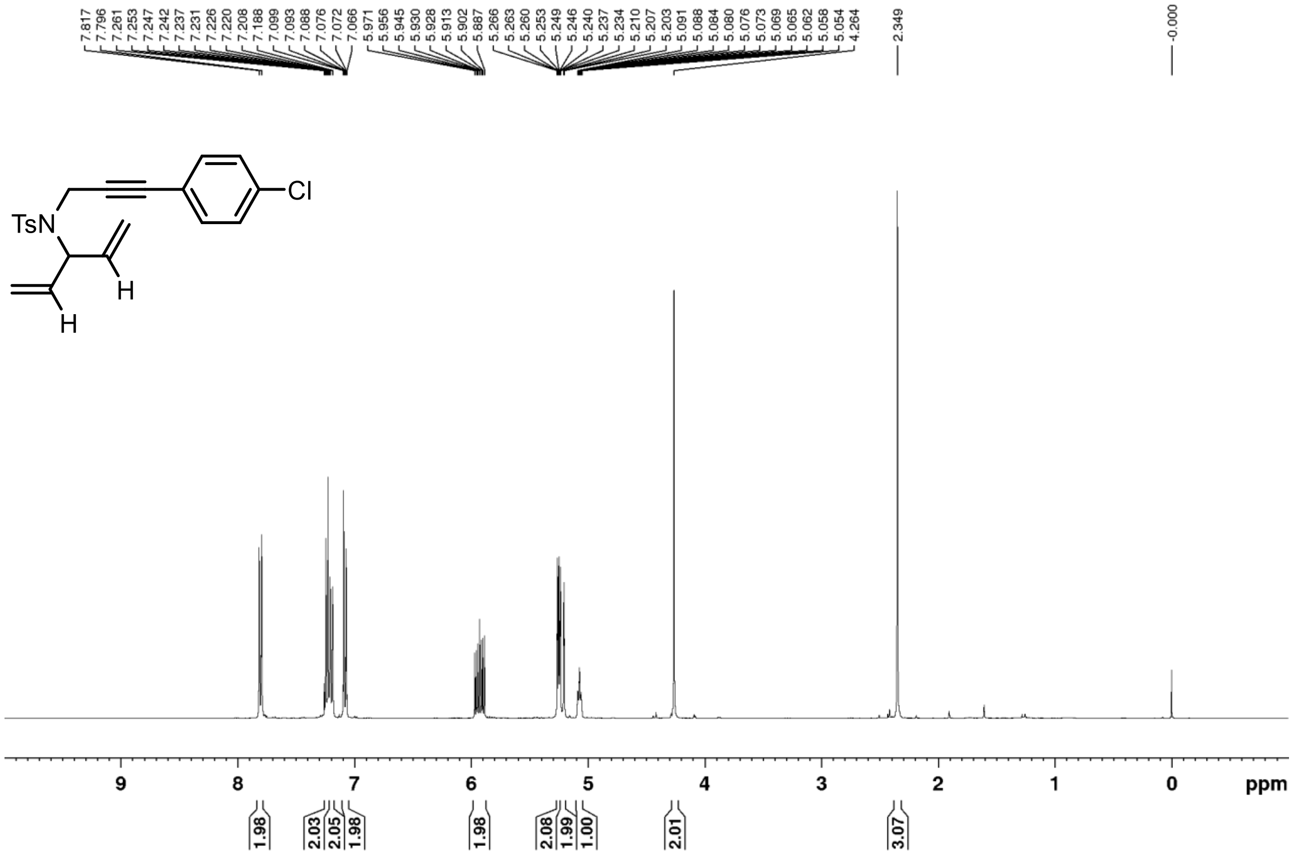

^{13}C NMR ($CDCl_3$, 101 MHz)

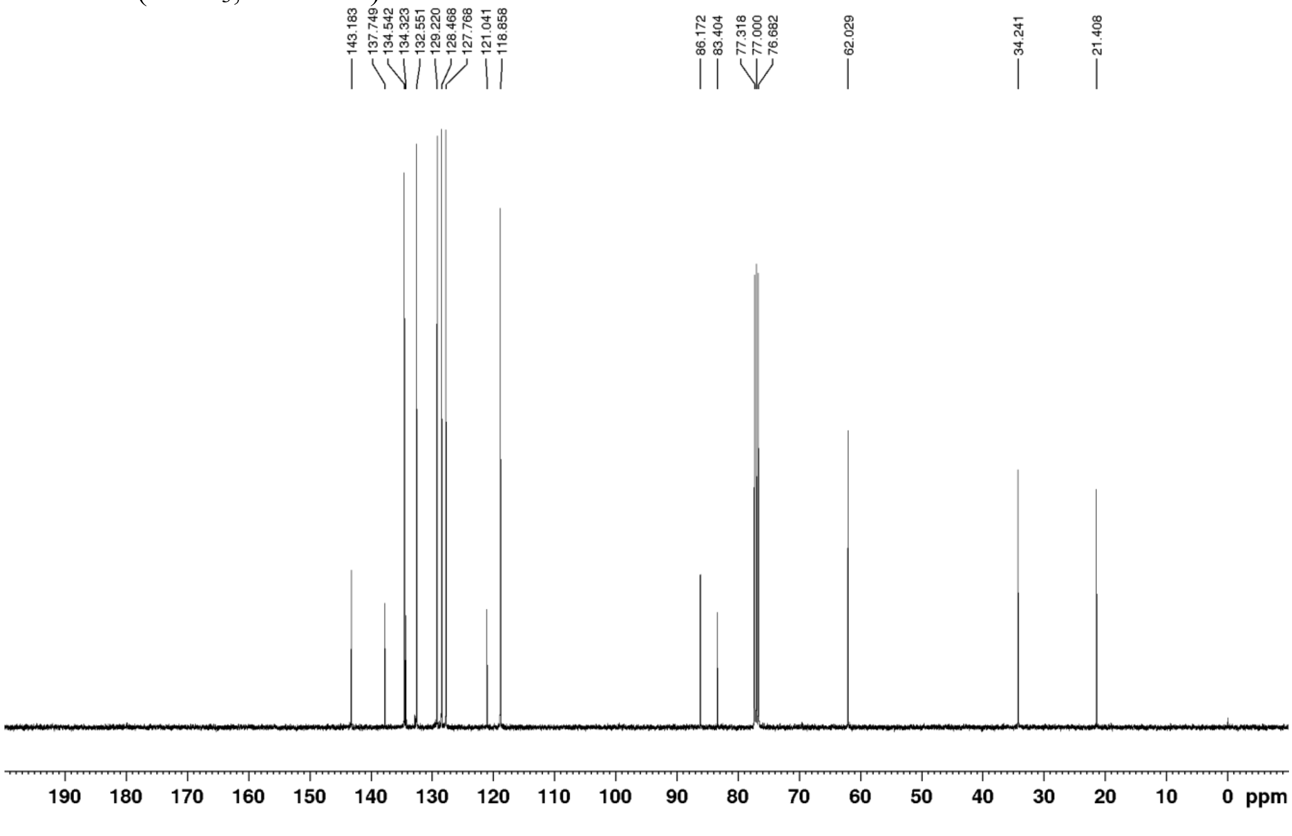

N-{3-(4-Bromophenyl)prop-2-yn-1-yl}-4-methyl-N-(penta-1,4-dien-3-yl) benzenesulfonamide (5d)

^{1}H NMR ($CDCl_3$, 400 MHz)

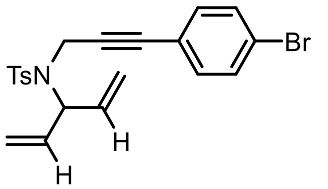

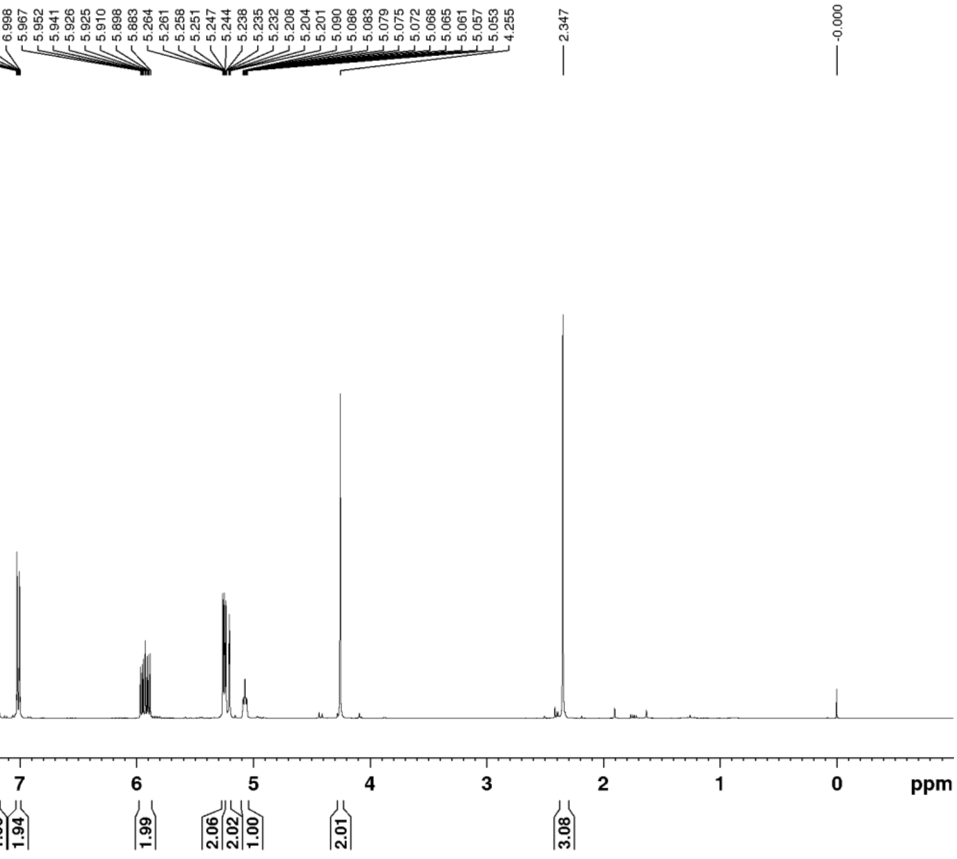

^{13}C NMR ($CDCl_3$, 101 MHz)

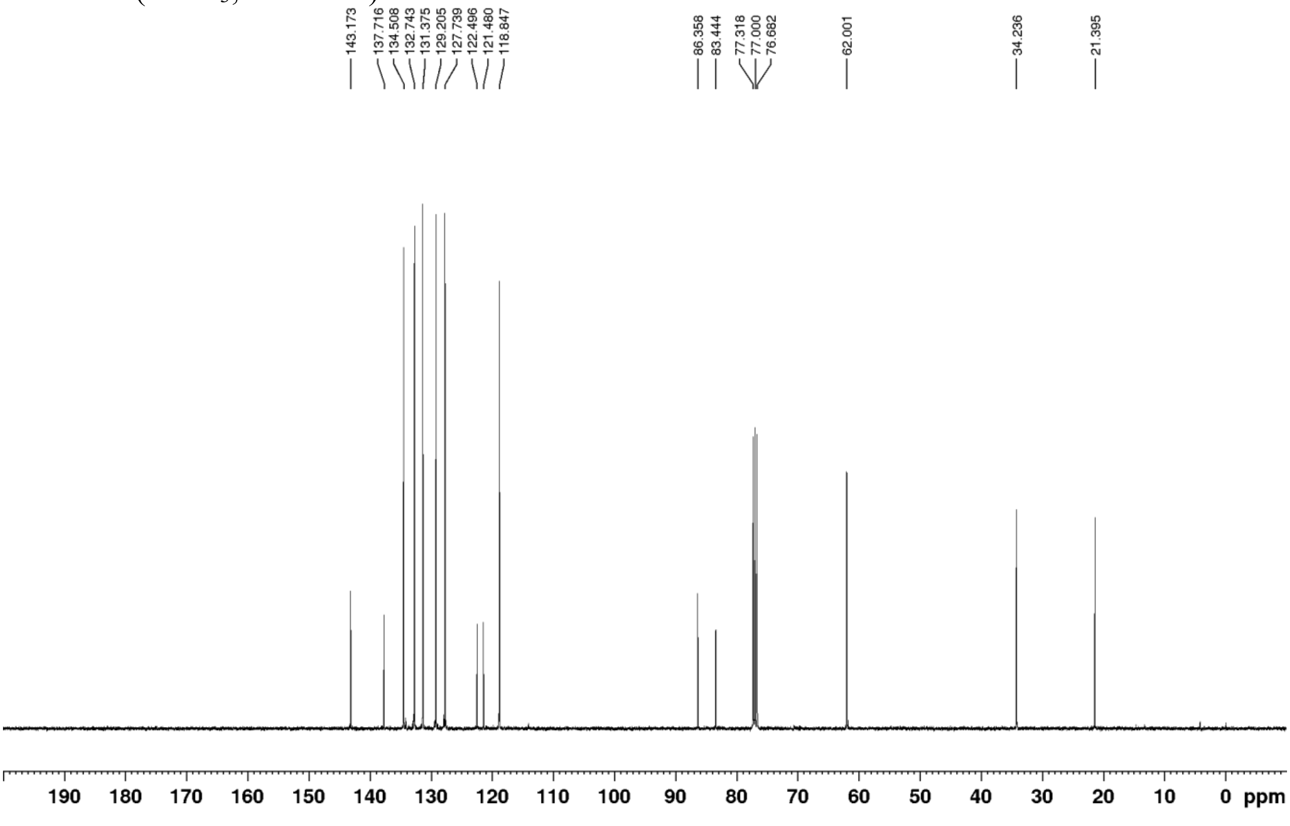

N-(Hept-2-yn-1-yl)-4-methyl-N-(penta-1,4-dien-3-yl)benzenesulfonamide (5f)

^{1}H NMR ($CDCl_3$, 400 MHz)

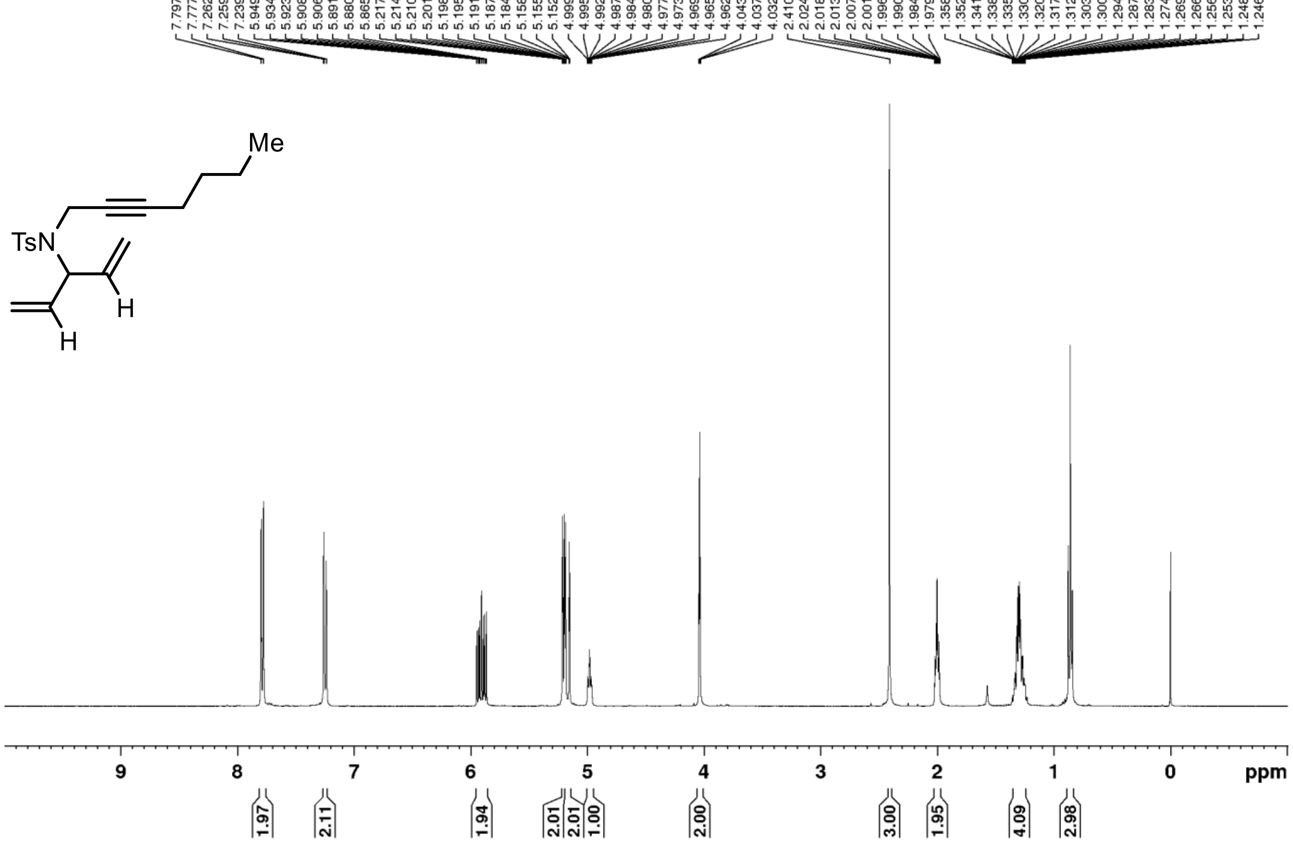

^{13}C NMR ($CDCl_3$, 101 MHz)

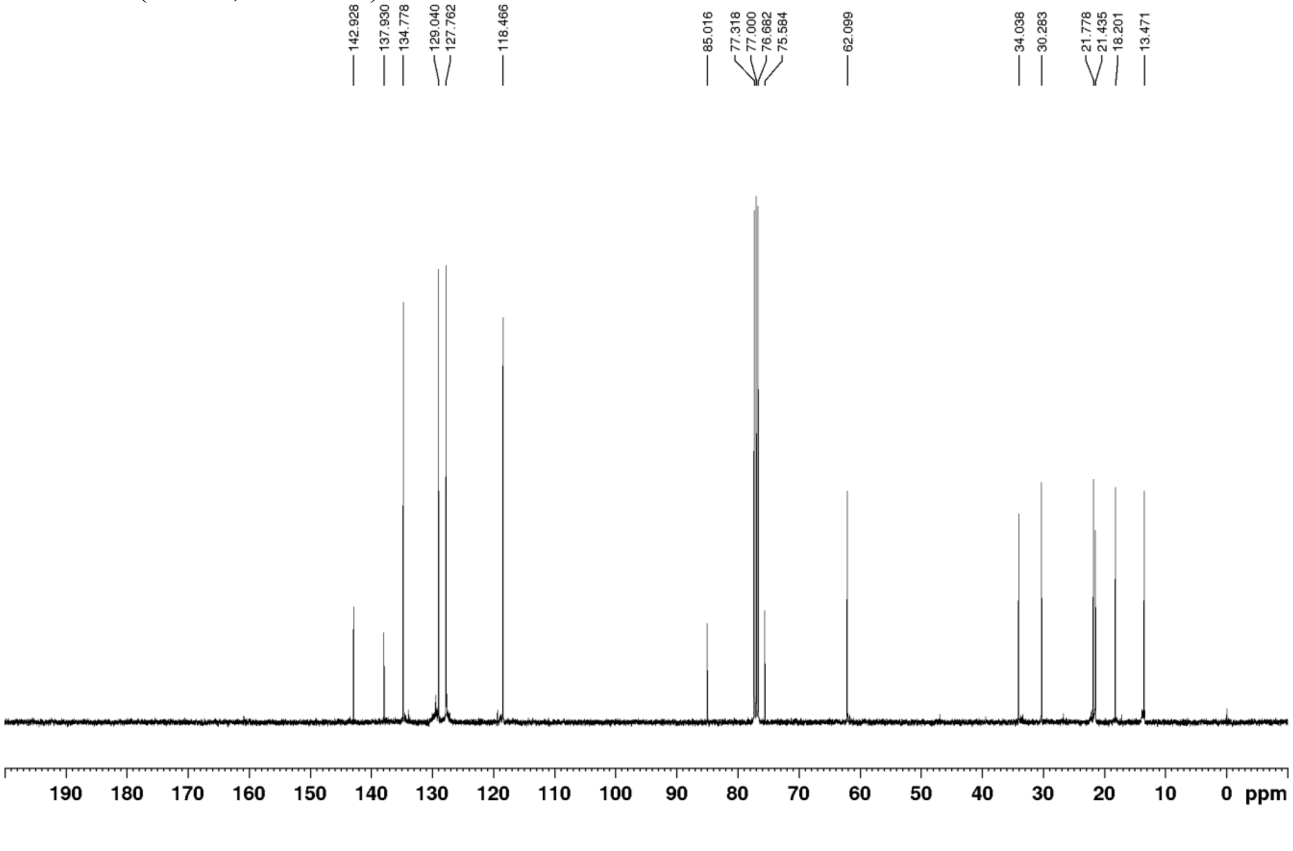

_N_-(2,4-Dimethylpenta-1,4-dien-3-yl)-4-methyl-_N_-(3-phenylprop-2-yn-1-yl) benzenesulfonamide (5h)

^{1}H NMR ($CDCl_3$, 400 MHz)

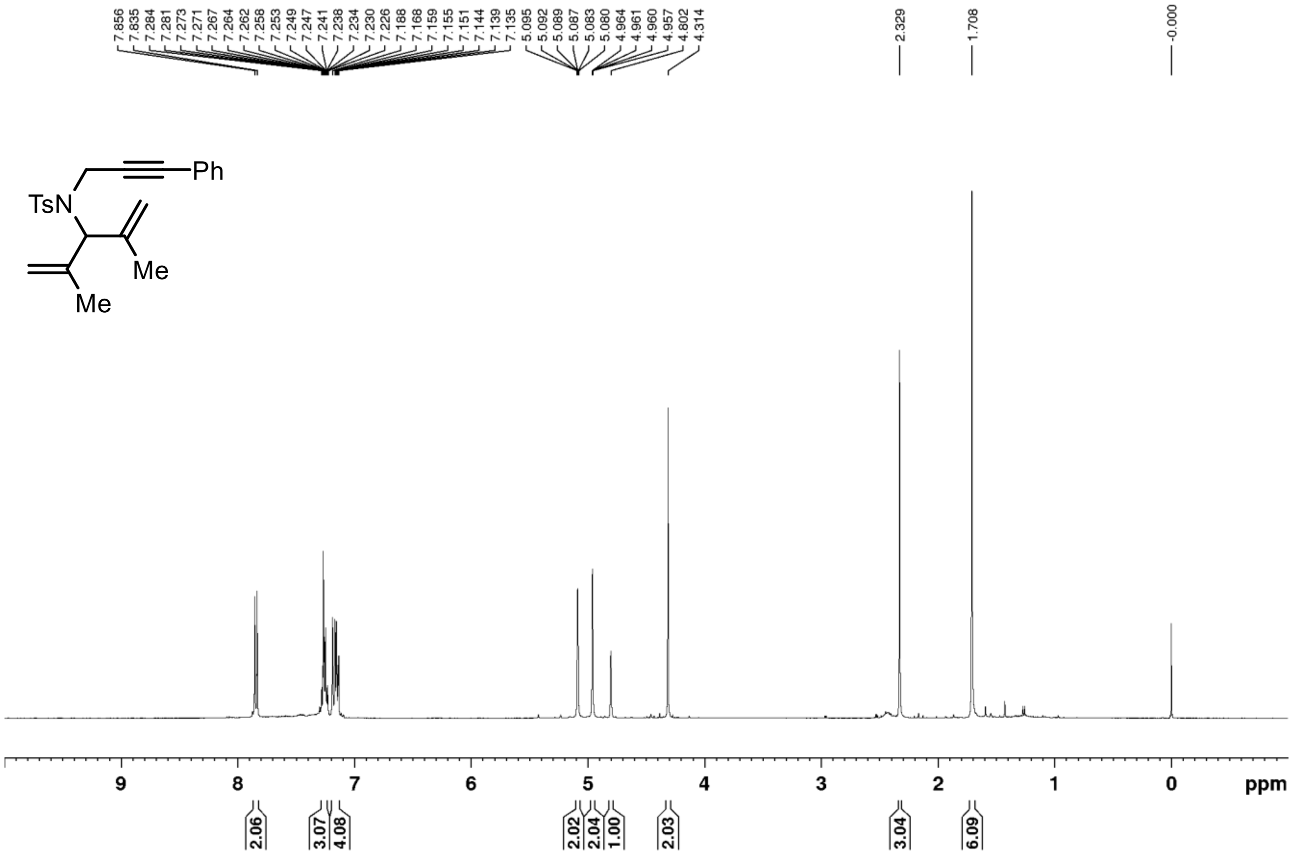

^{13}C NMR ($CDCl_3$, 101 MHz)

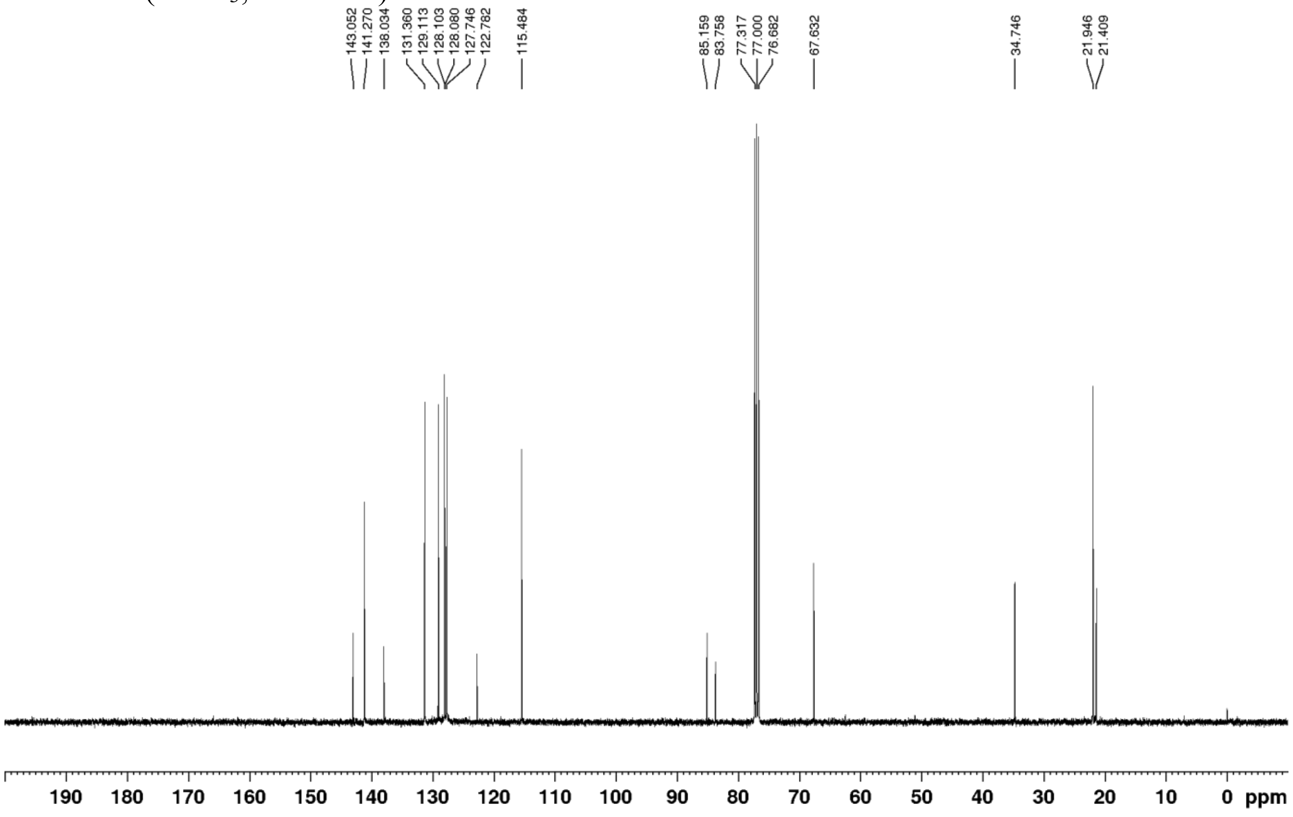

***N*-{3-(4-Bromophenyl)prop-2-yn-1-yl}-4-methyl-*N*-(2,4-dimethylpenta-1,4-dien-3-yl)-benzenesulfonamide (5i)**

^{1}H NMR (CDCl$_3$, 400 MHz)

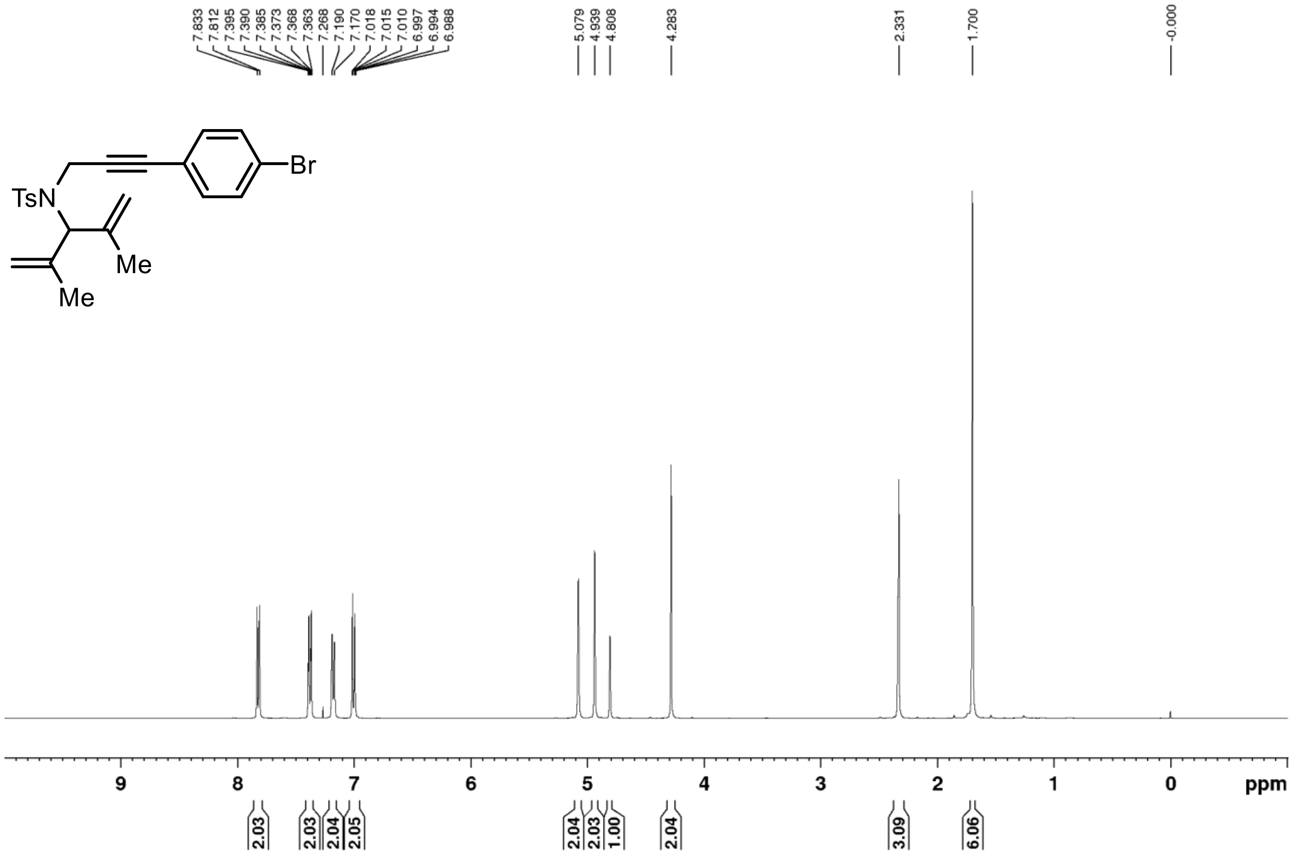

^{13}C NMR (CDCl$_3$, 101 MHz)

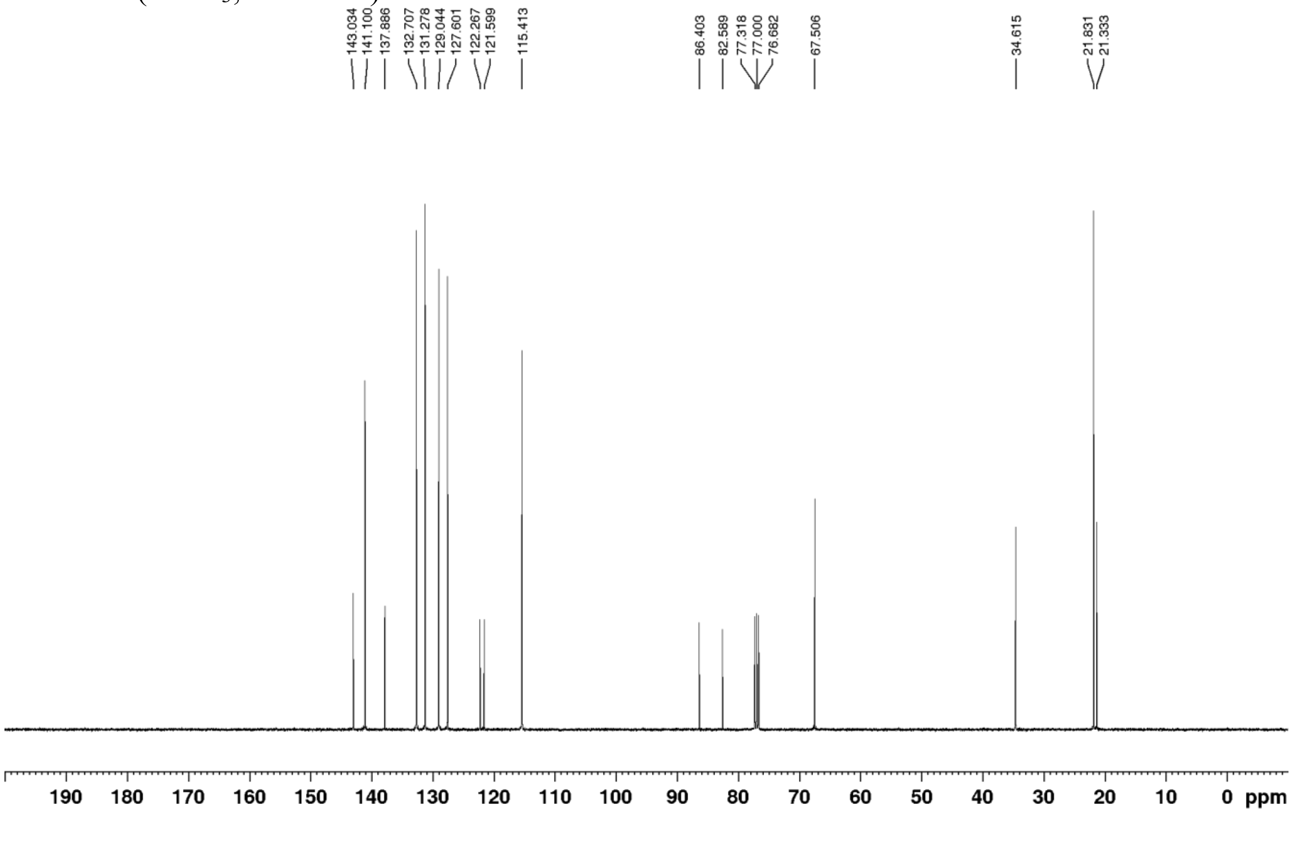

_N_-(But-2-yn-1-yl)-4-methyl-_N_-(2,4-dimethylpenta-1,4-dien-3-yl)-benzenesulfonamide (5j)

^{1}H NMR ($CDCl_3$, 400 MHz)

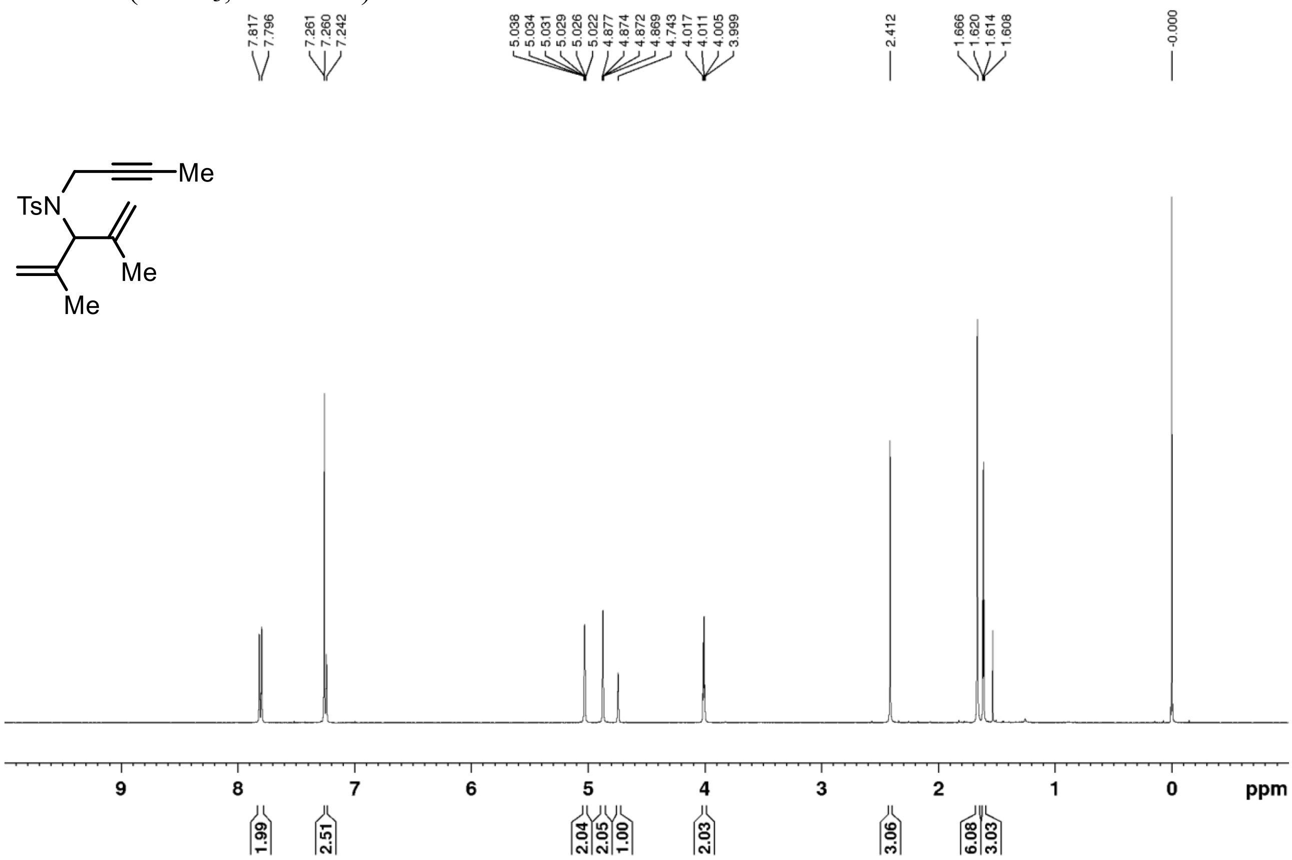

^{13}C NMR ($CDCl_3$, 101 MHz)

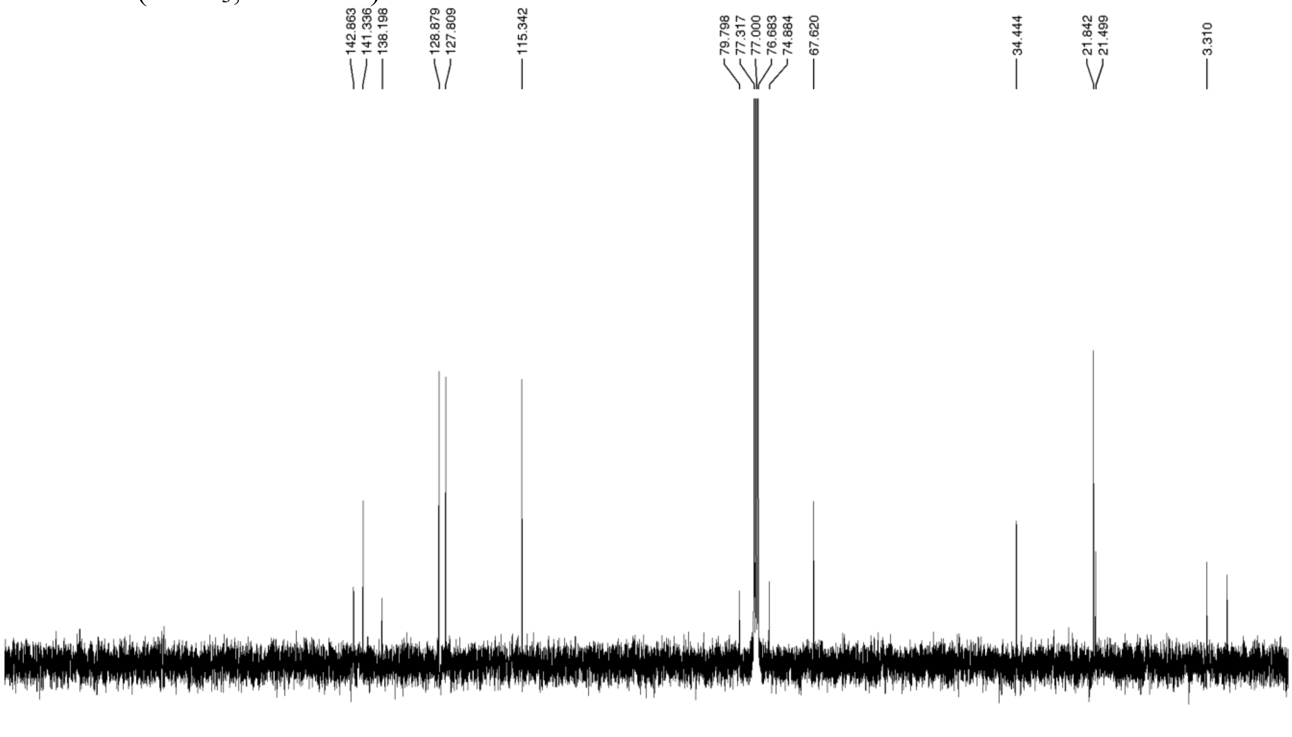

2,4-Dimethylpenta-1,4-dien-3-yl 3-phenylpropynoate (5k)

^{1}H NMR ($CDCl_3$, 400 MHz)

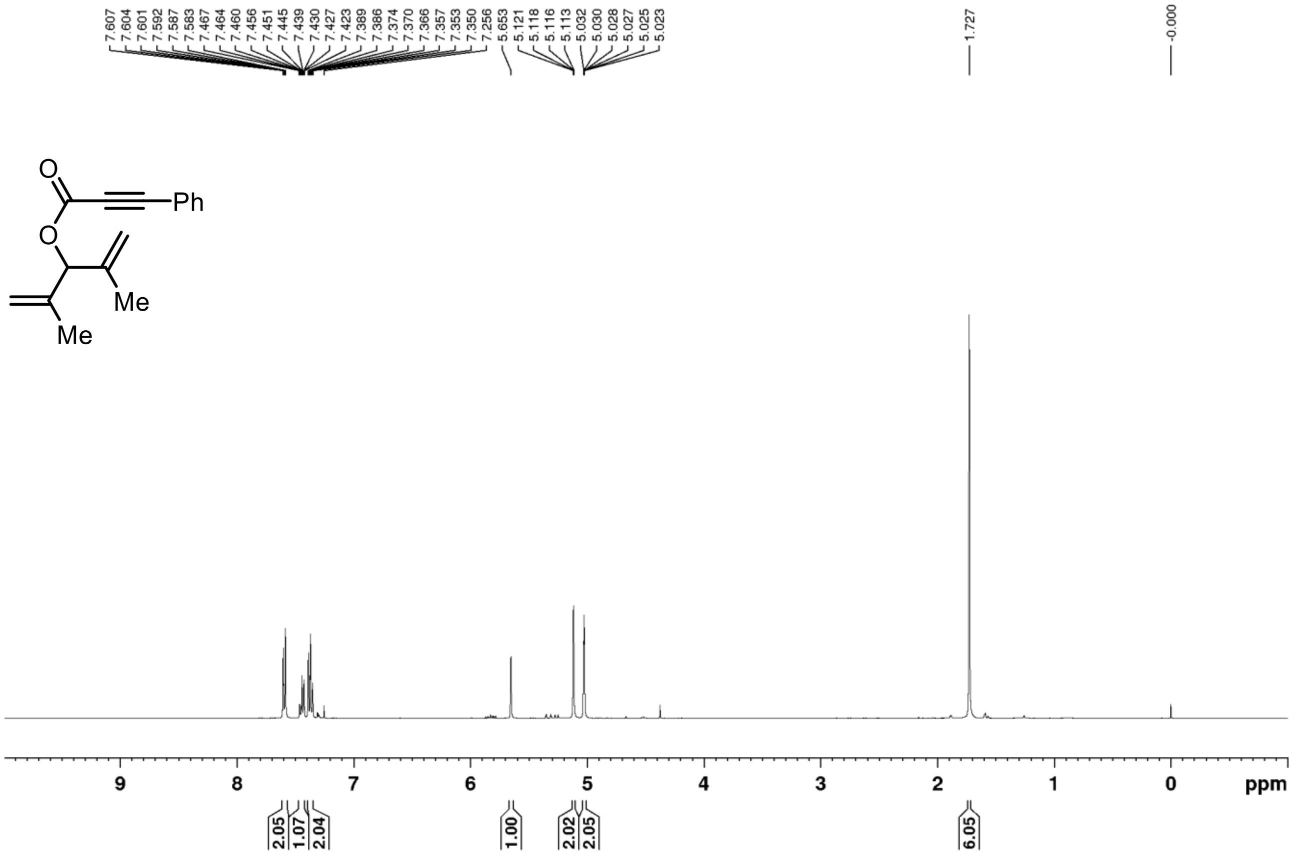

^{13}C NMR ($CDCl_3$, 101 MHz)

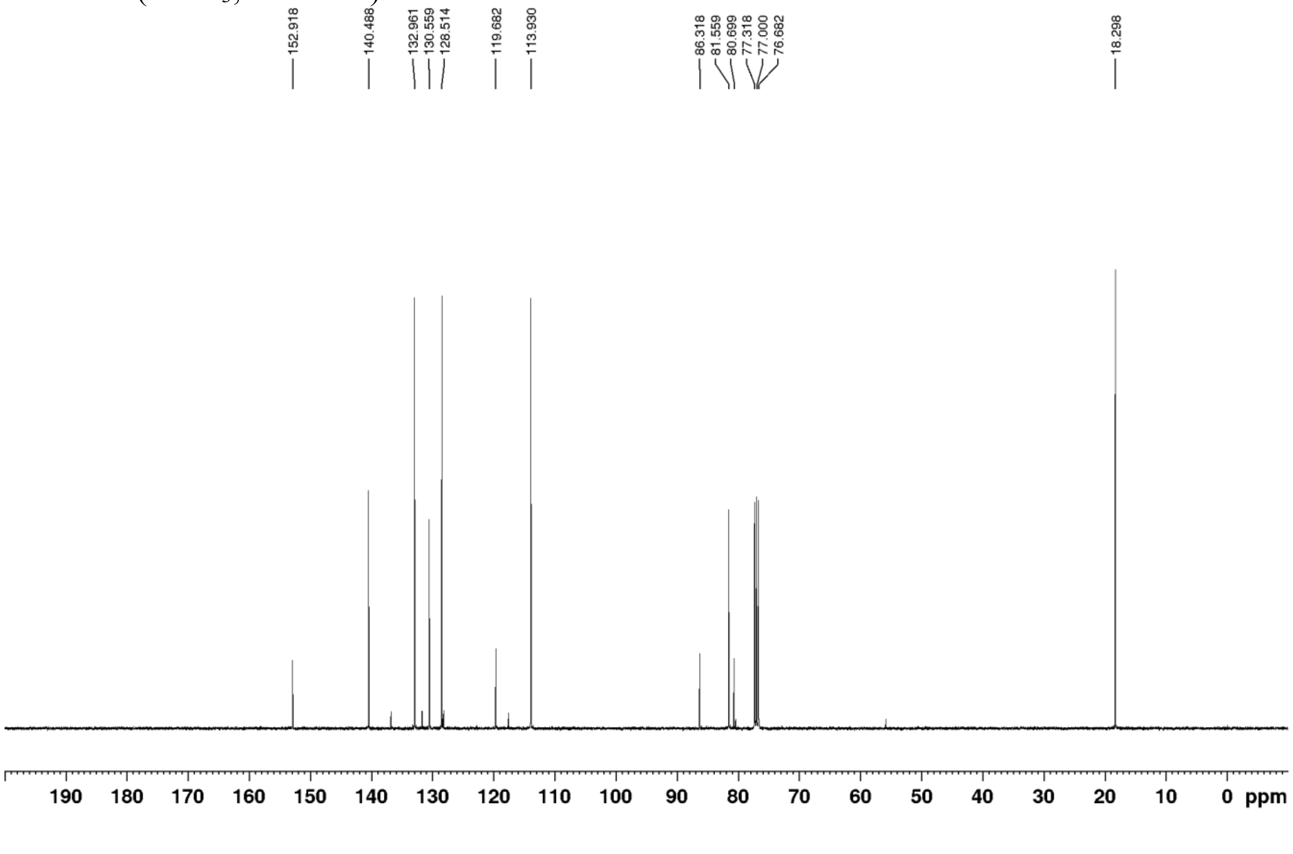

2-Chloro-1-(pyrrolidin-1-yl)prop-2-en-1-one (2f)

^{1}H NMR ($CDCl_3$, 400 MHz)

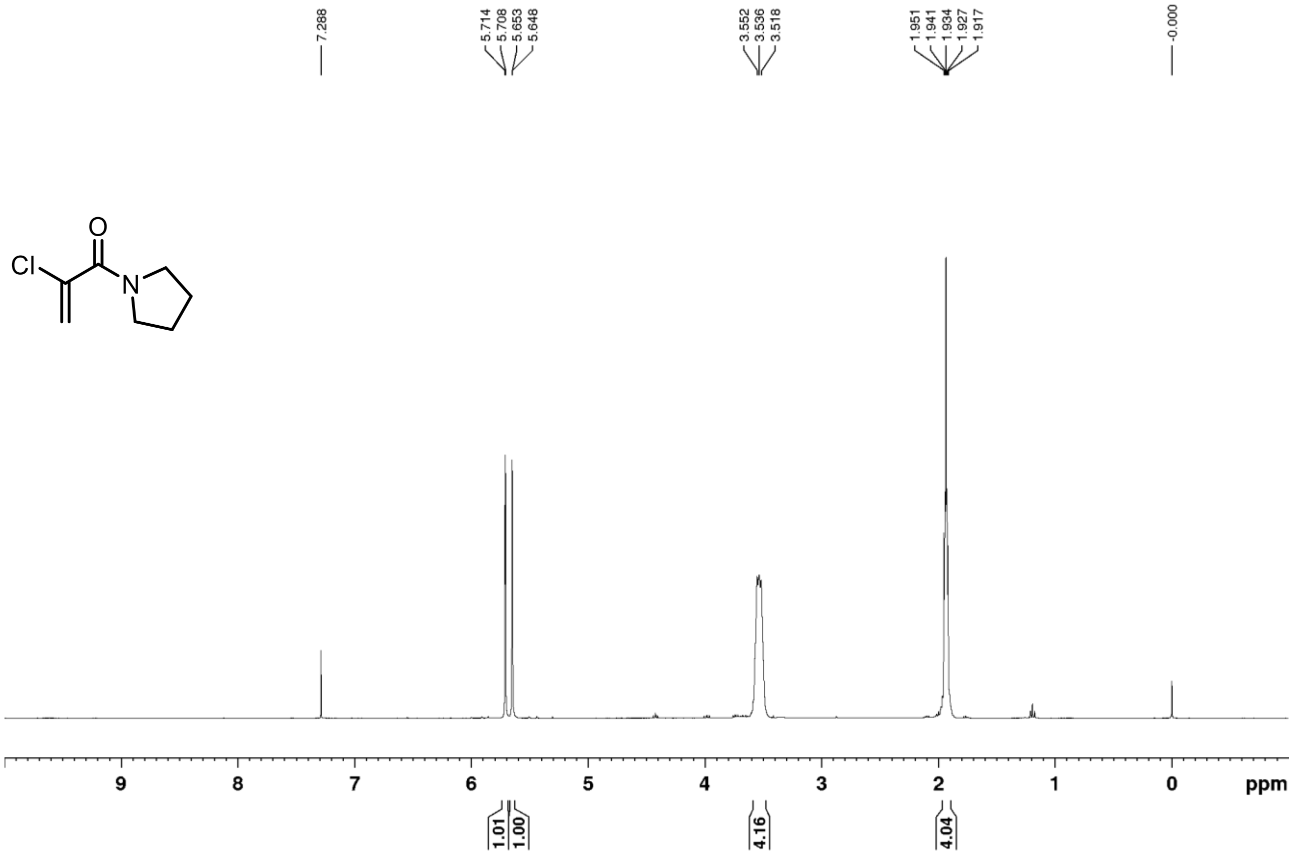

^{13}C NMR ($CDCl_3$, 101 MHz)

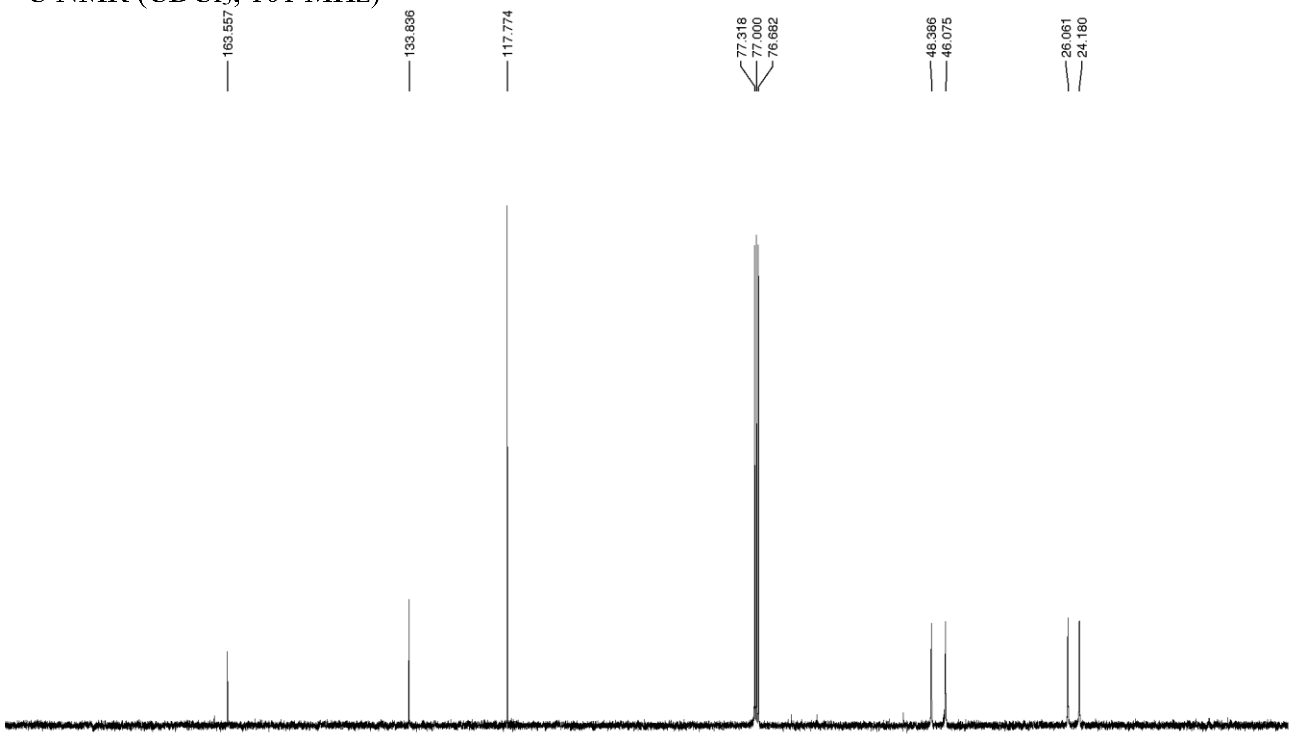

6.2. Rh-Catalyzed Enantioselective PKR (type I) of Racemic 1,6-Enynes with α-Fluoroacrylamides (Figure 2)

{(1*S*,5*R*,7a*R*)-5-Fluoro-1,4,7a-trimethyl-2-(4-methylphenyl)-2,3,5,6,7,7a-hexahydro-1*H*-isoindol-5-yl}(pyrrolidin-1-yl)methanone [3aa (major diastereomer)]

^{1}H NMR (CDCl$_3$, 400 MHz)

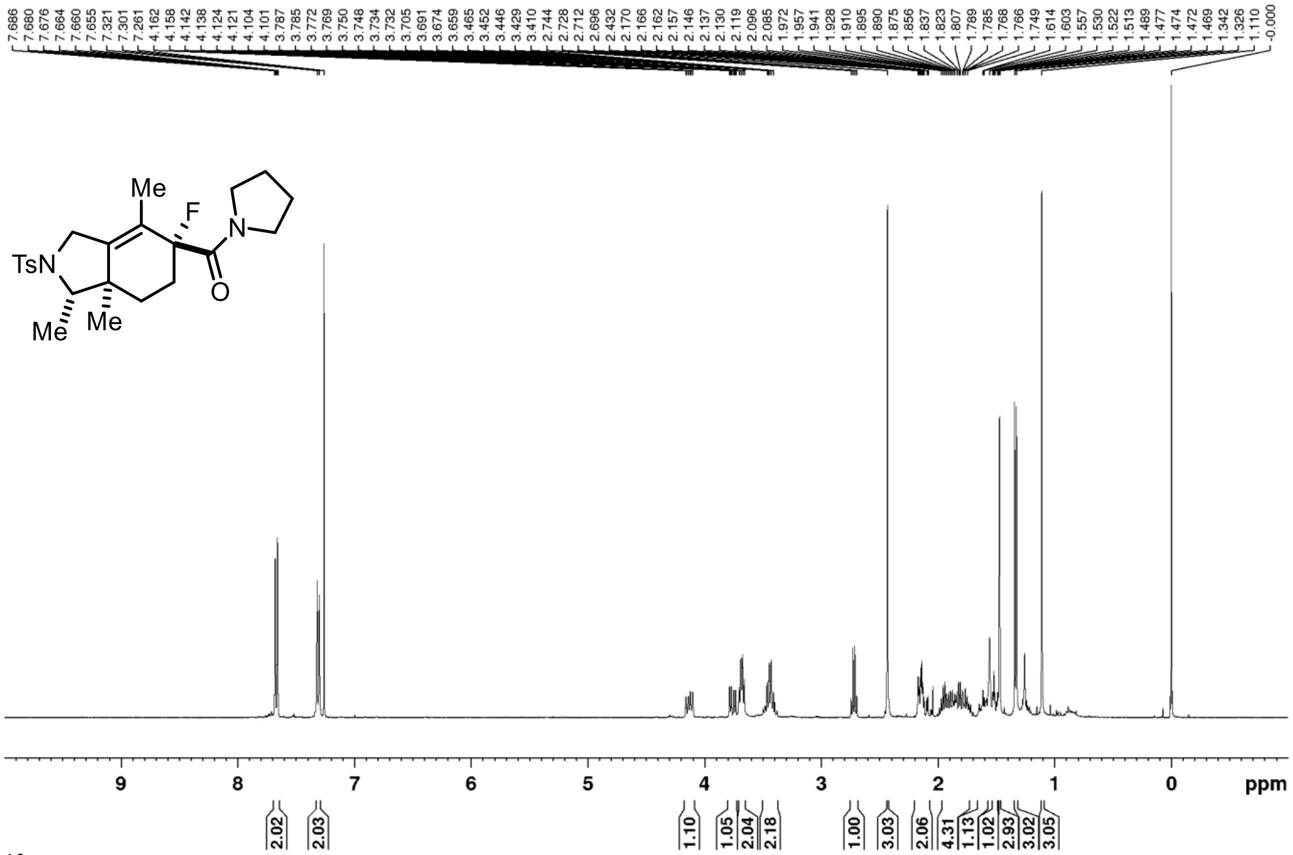

^{13}C NMR (CDCl$_3$, 101 MHz)

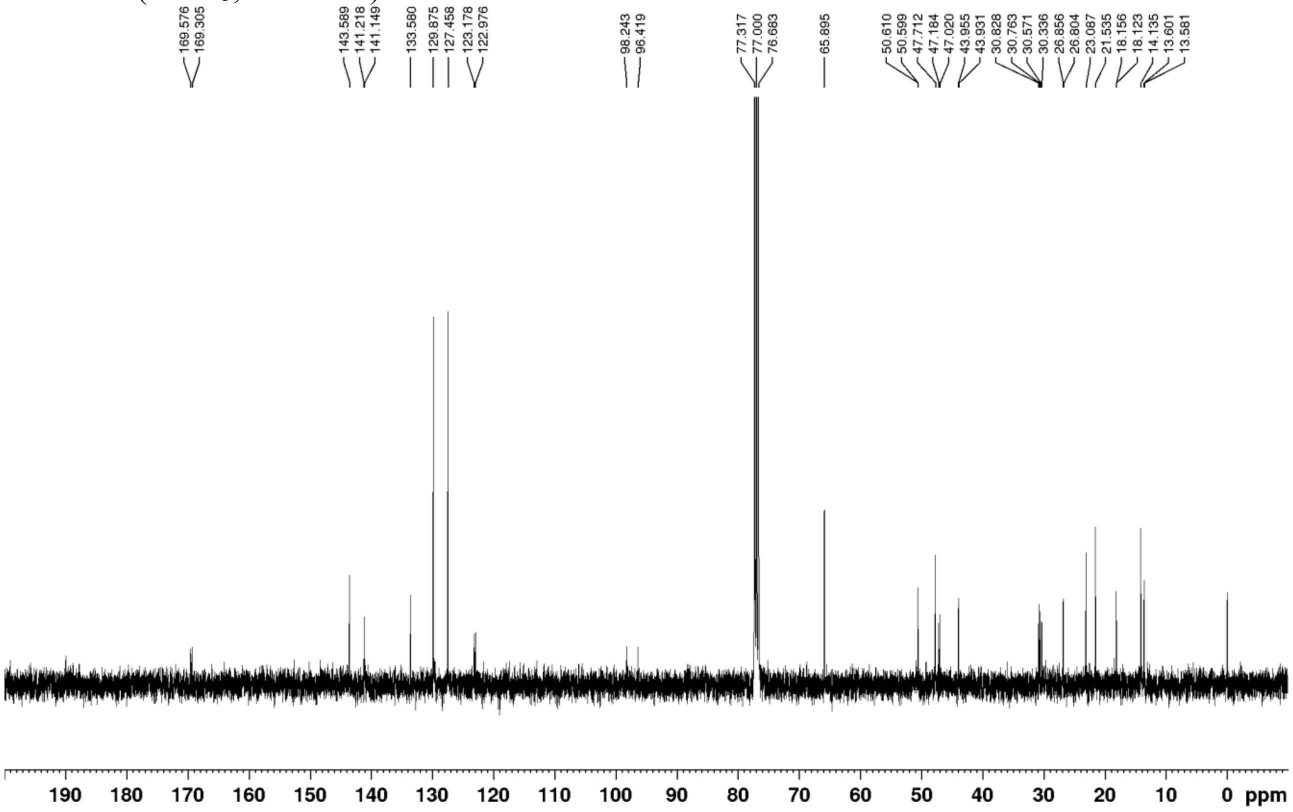

^{19}F NMR ($CDCl_3$, 377 MHz)

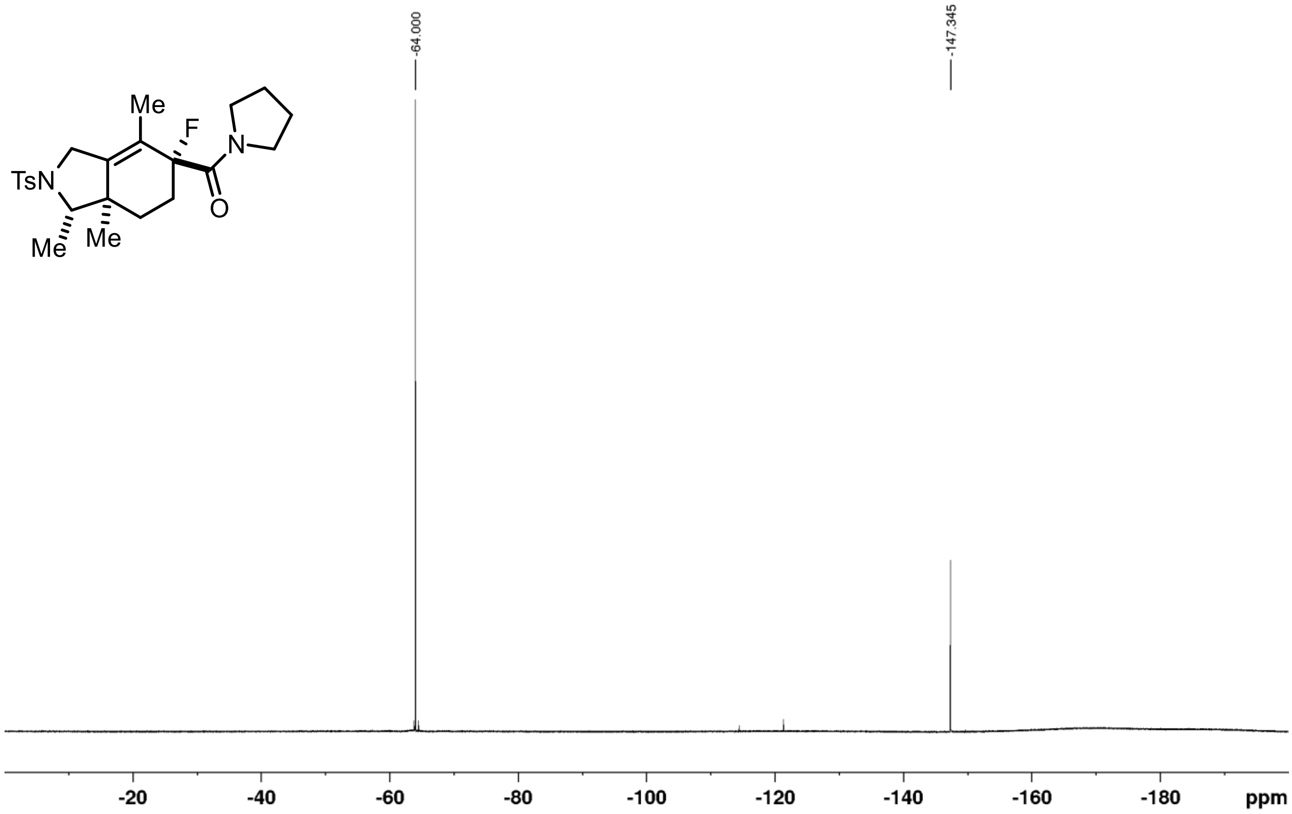

NOESY ($CDCl_3$, 400 MHz)

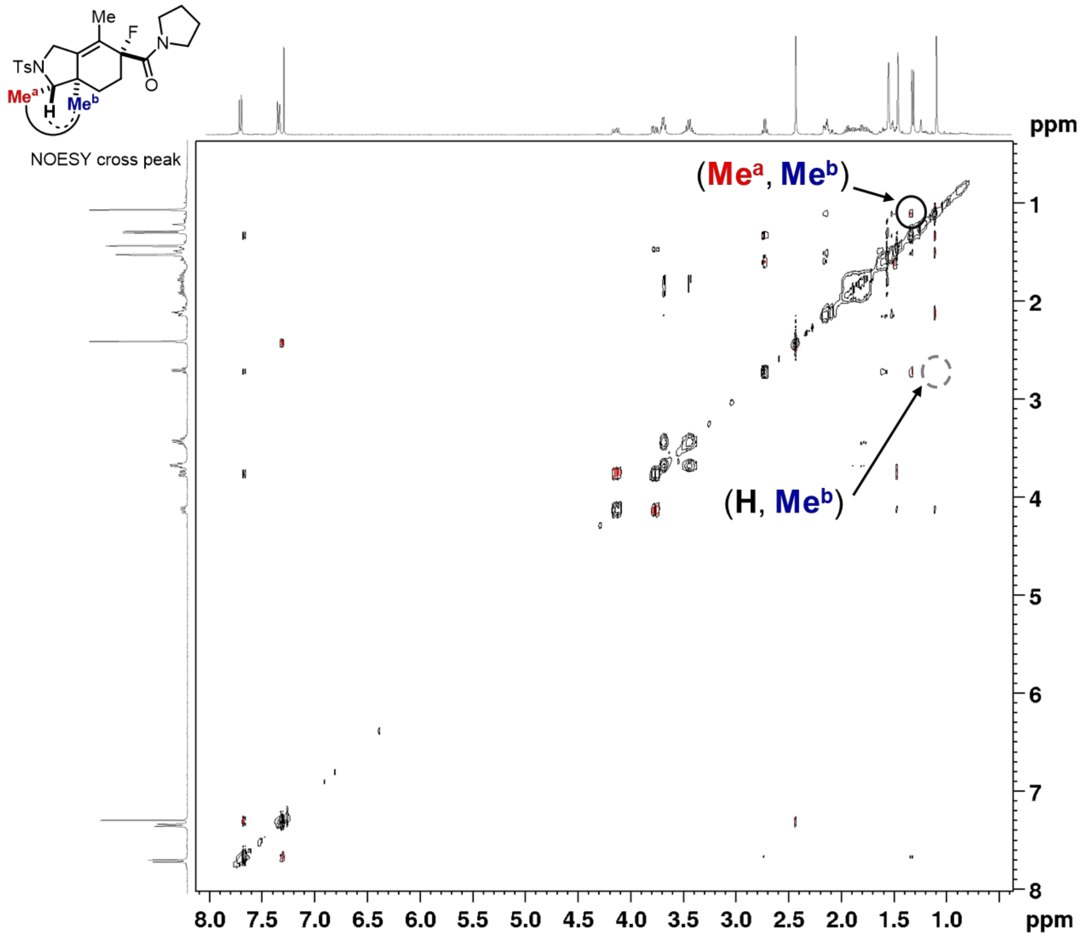

{5-Fluoro-1,4,7a-trimethyl-2-(4-methylphenyl)-2,3,5,6,7,7a-hexahydro-1*H*-isoindol-5-yl}(pyrrolidin-1-yl)methanone [3aa (minor diastereomer)]

^{1}H NMR ($CDCl_3$, 400 MHz)

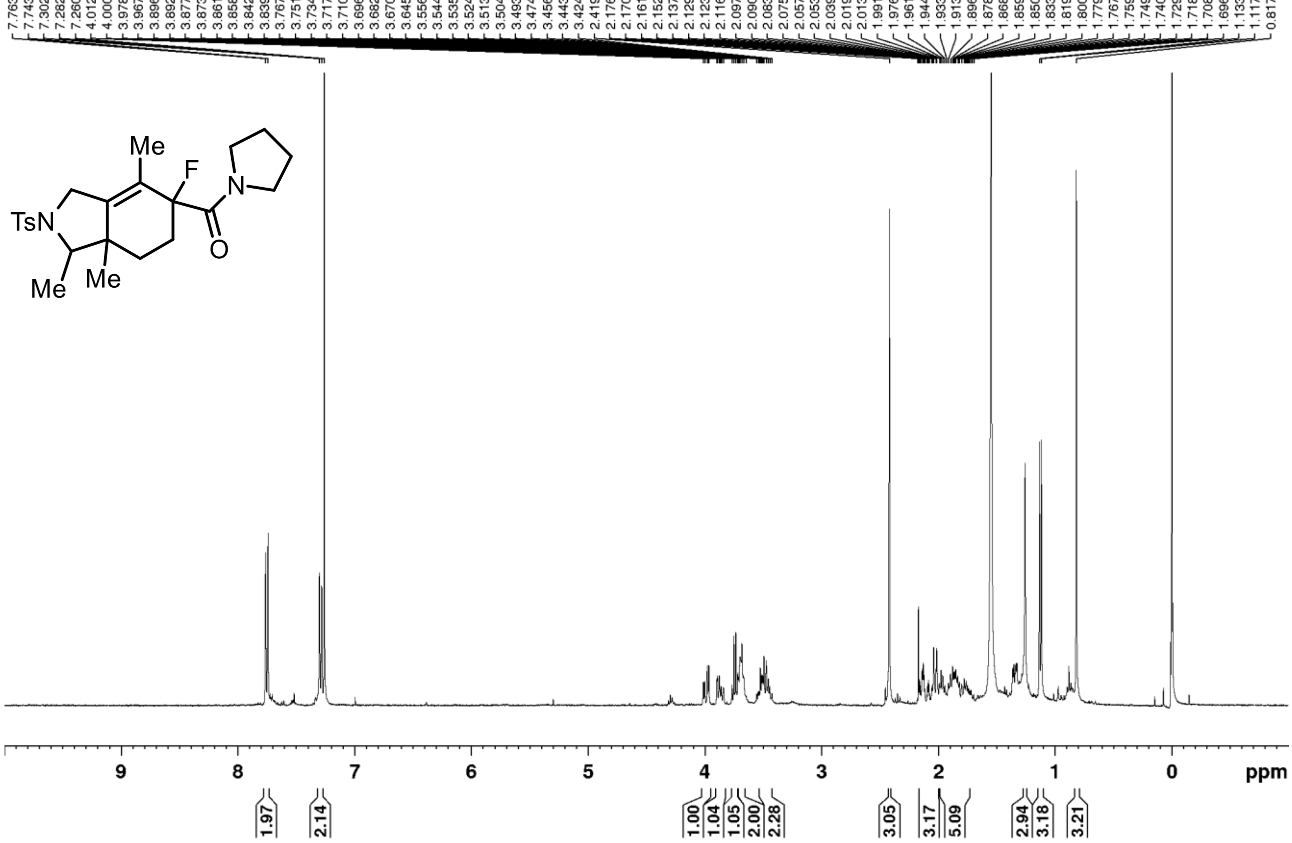

^{19}F NMR ($CDCl_3$, 377 MHz)

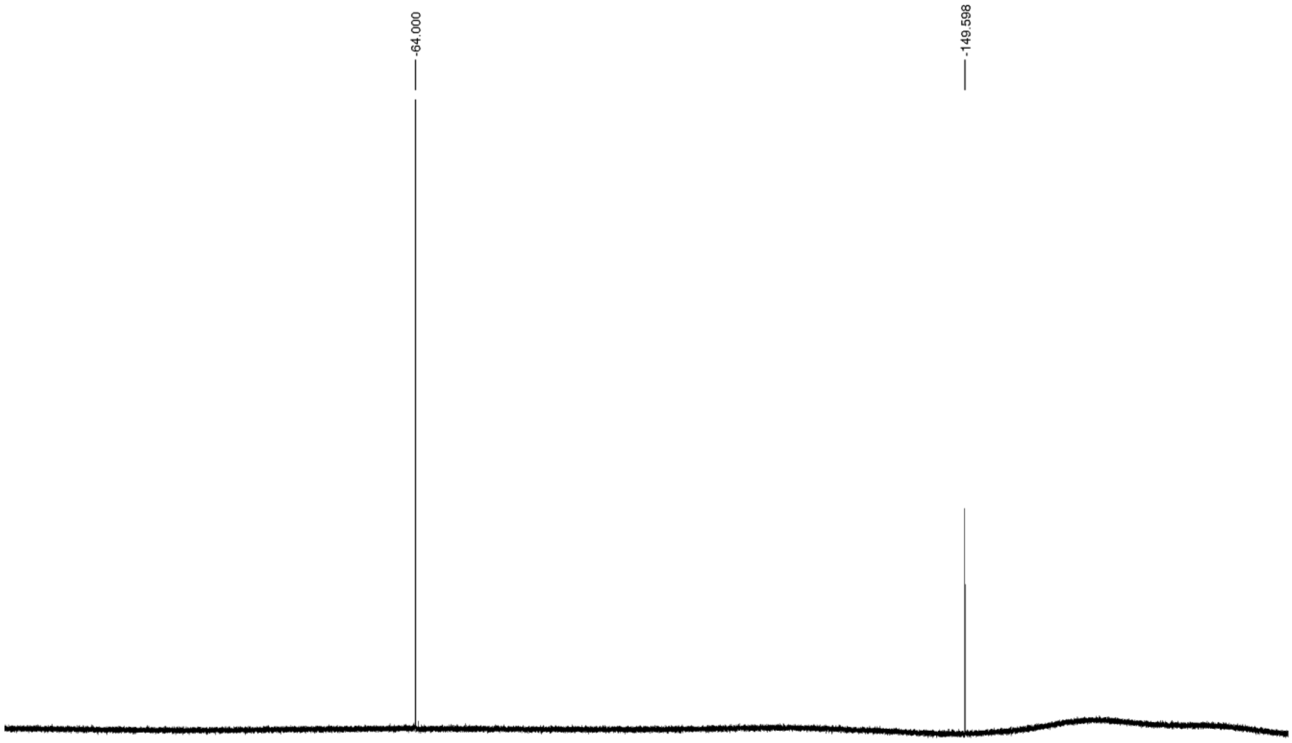

(*E*)-4-{(2*R*,3*R*,*Z*)-4-Ethylidene-2,3-dimethyl-1-(4-methylphenyl)pyrrolidin-3-yl}-2-fluoro-1-(pyrrolidin-1-yl)but-2-en-1-one (4aa)

Diastereomer mixture (78:22 d.r.)

^{1}H NMR (CDCl$_3$, 400 MHz)

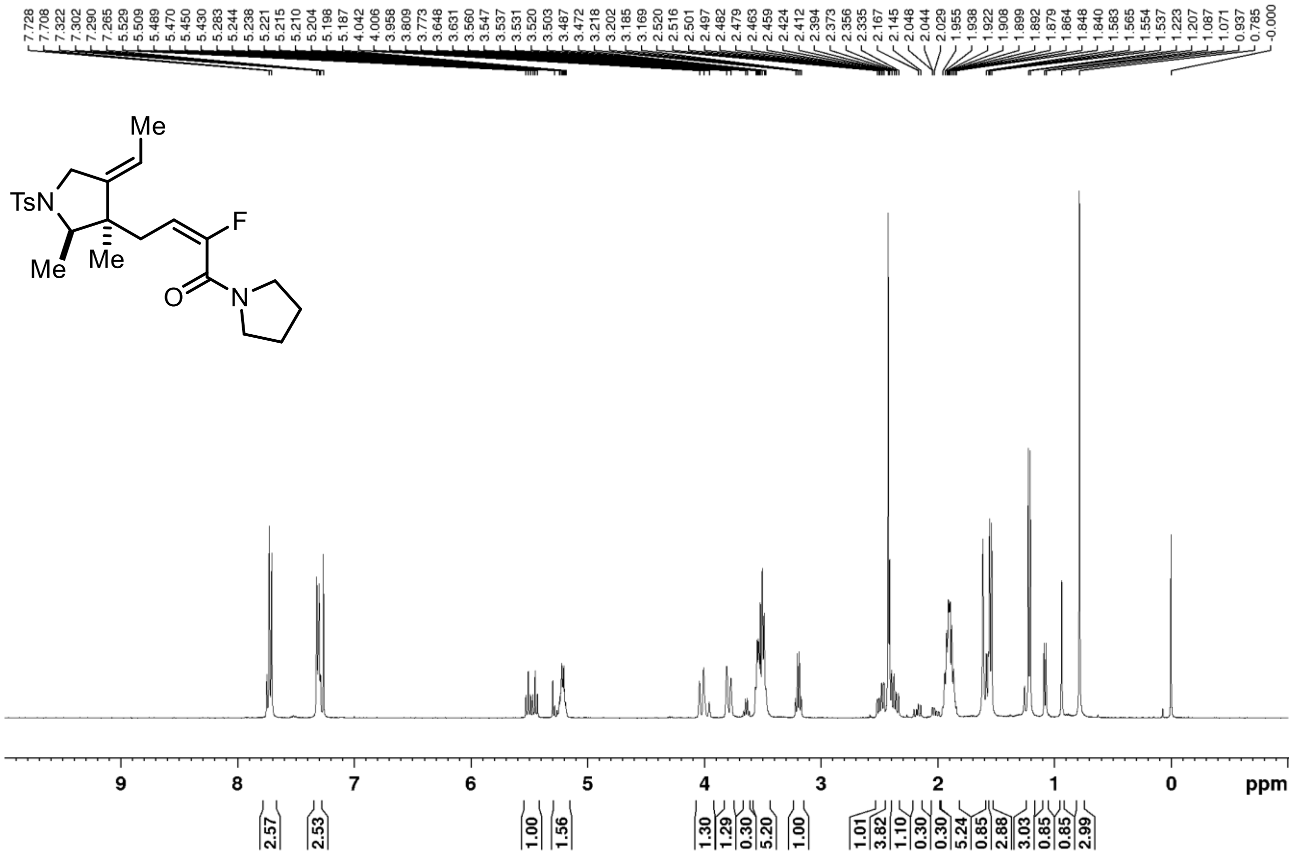

^{13}C NMR (CDCl$_3$, 101 MHz)

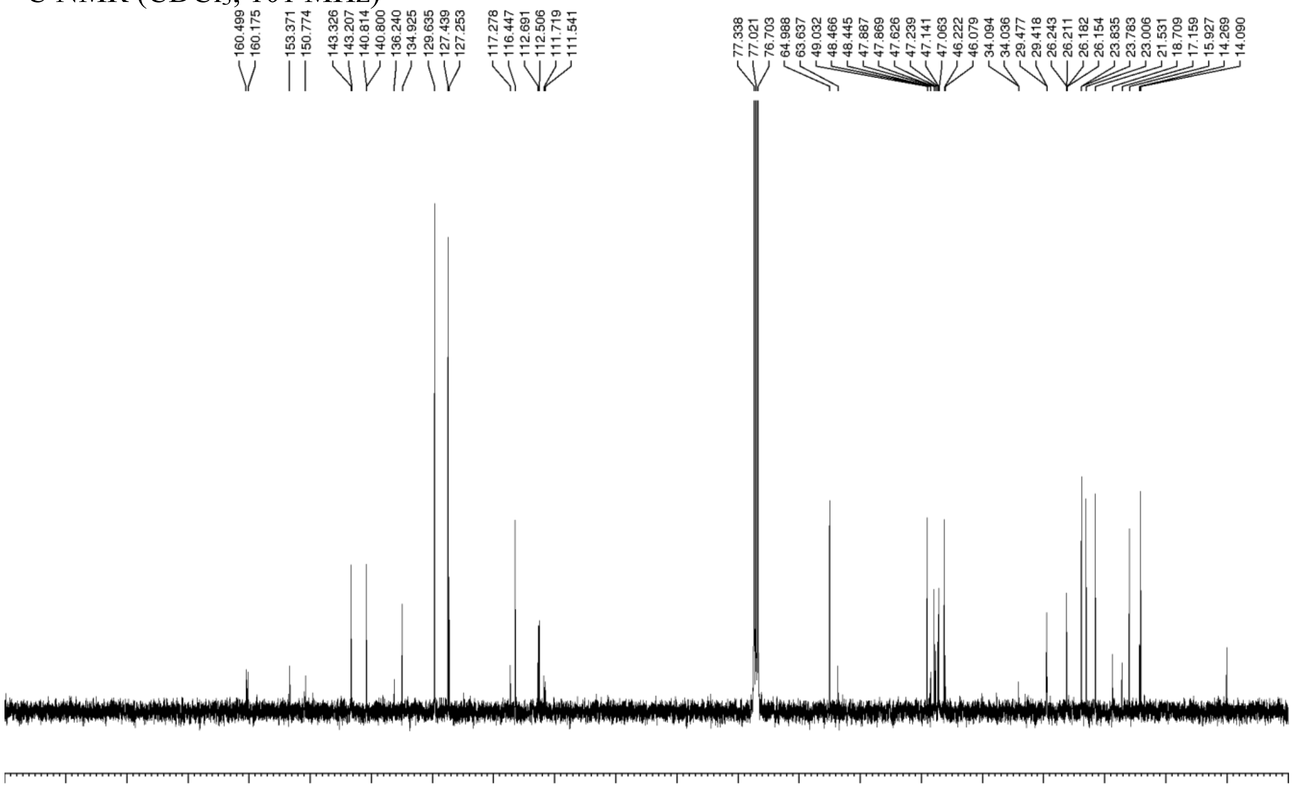

^{19}F NMR ($CDCl_3$, 377 MHz)

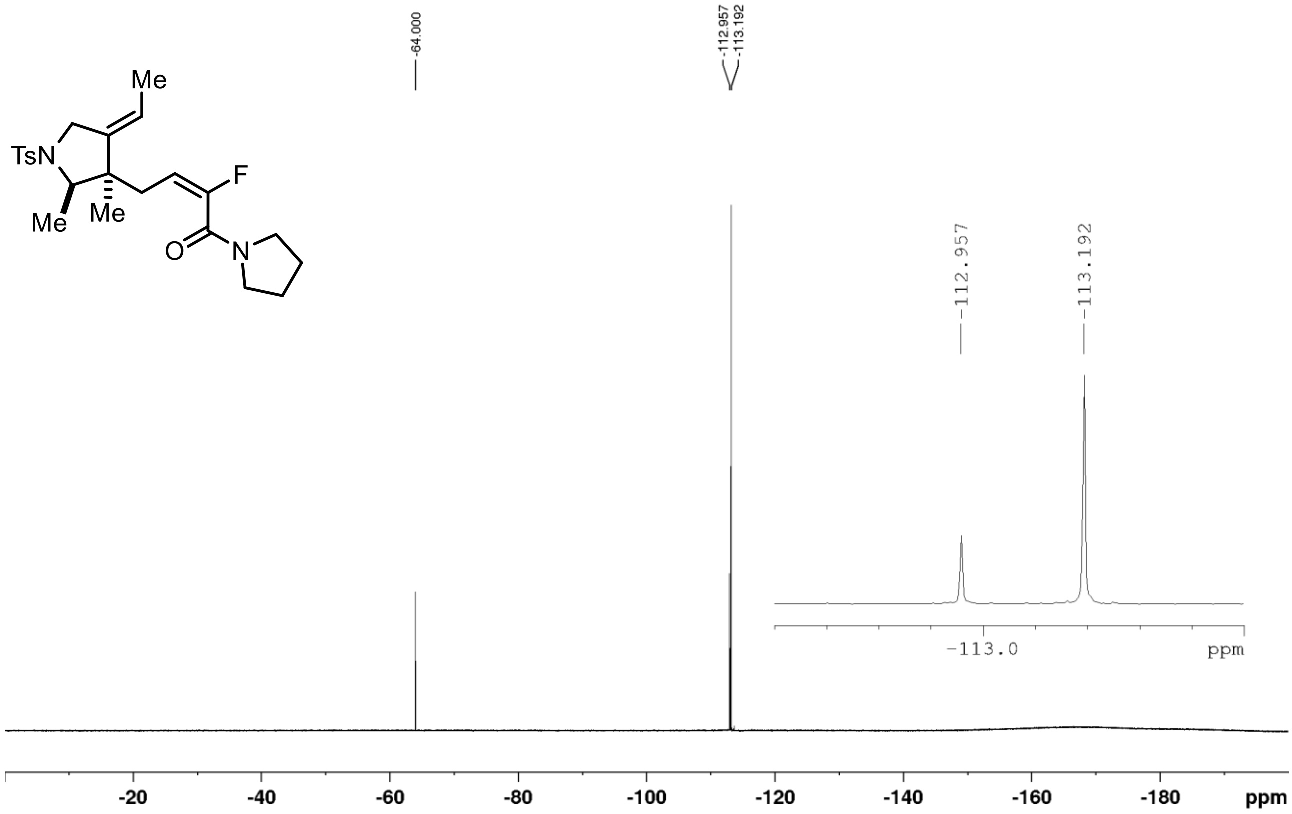

NOESY ($CDCl_3$, 400 MHz)

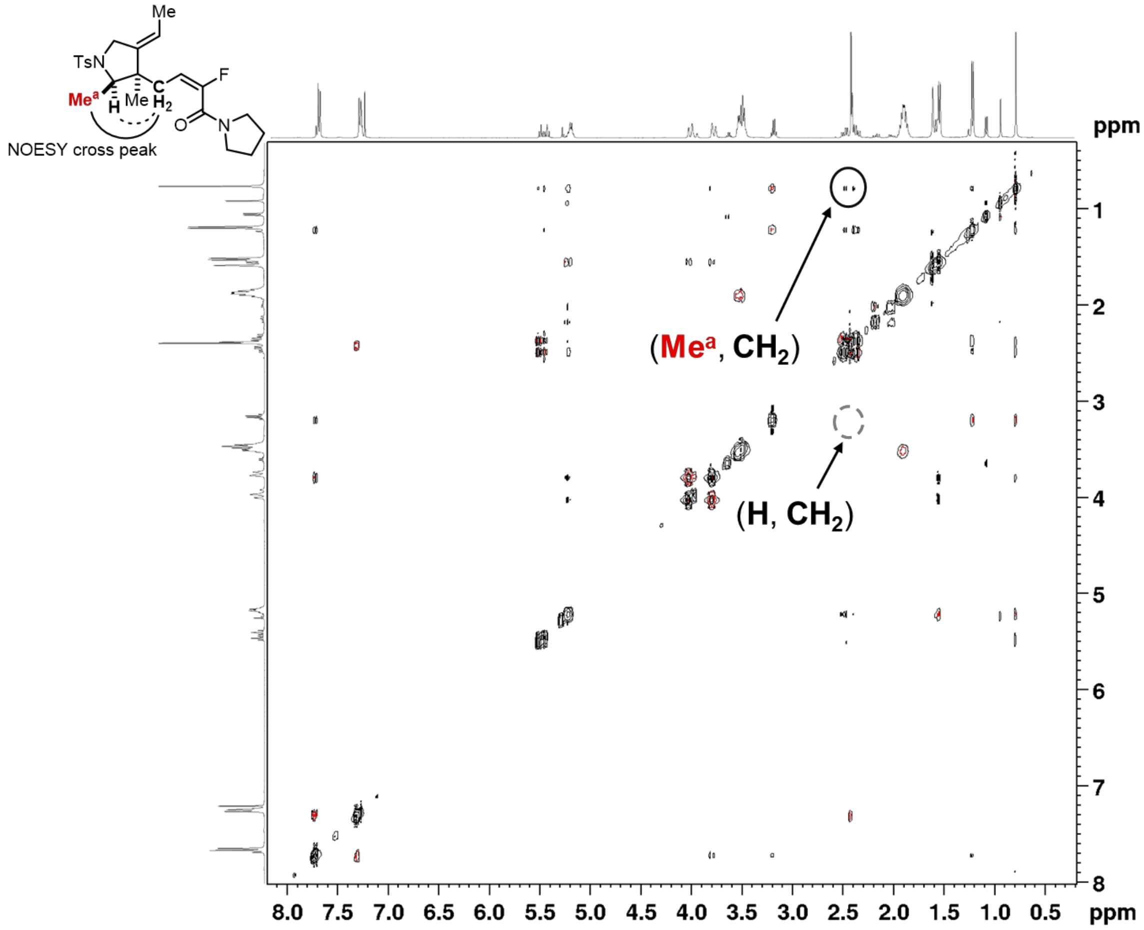

{(1*S*,5*R*,7a*R*)-5-Fluoro-4,7a-dimethyl-2-(4-methylphenyl)-1-pentyl-2,3,5,6,7,7a-hexahydro-1*H*-isoindol-5-yl}(pyrrolidin-1-yl)methanone [3ba (major diastereomer)]

^{1}H NMR ($CDCl_3$, 400 MHz)

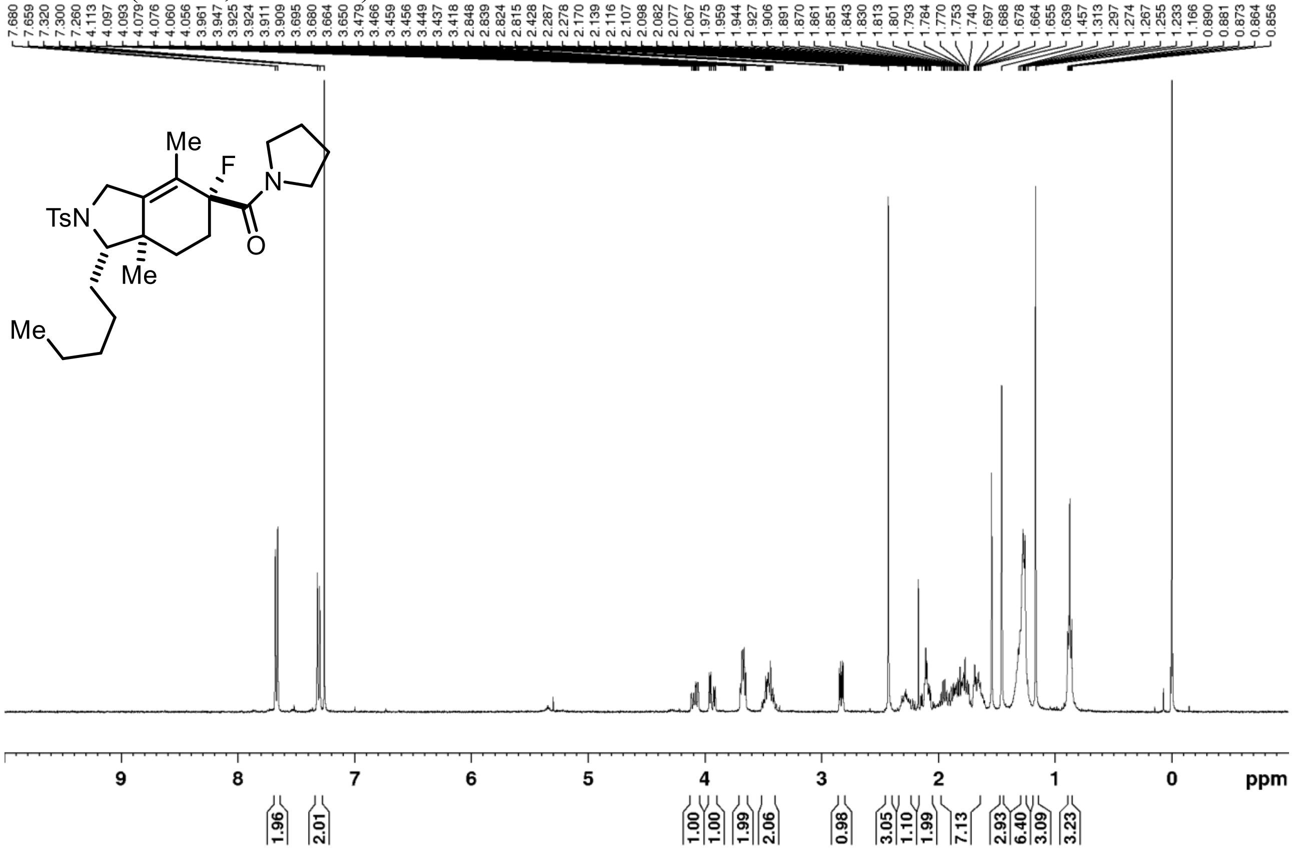

^{13}C NMR ($CDCl_3$, 101 MHz)

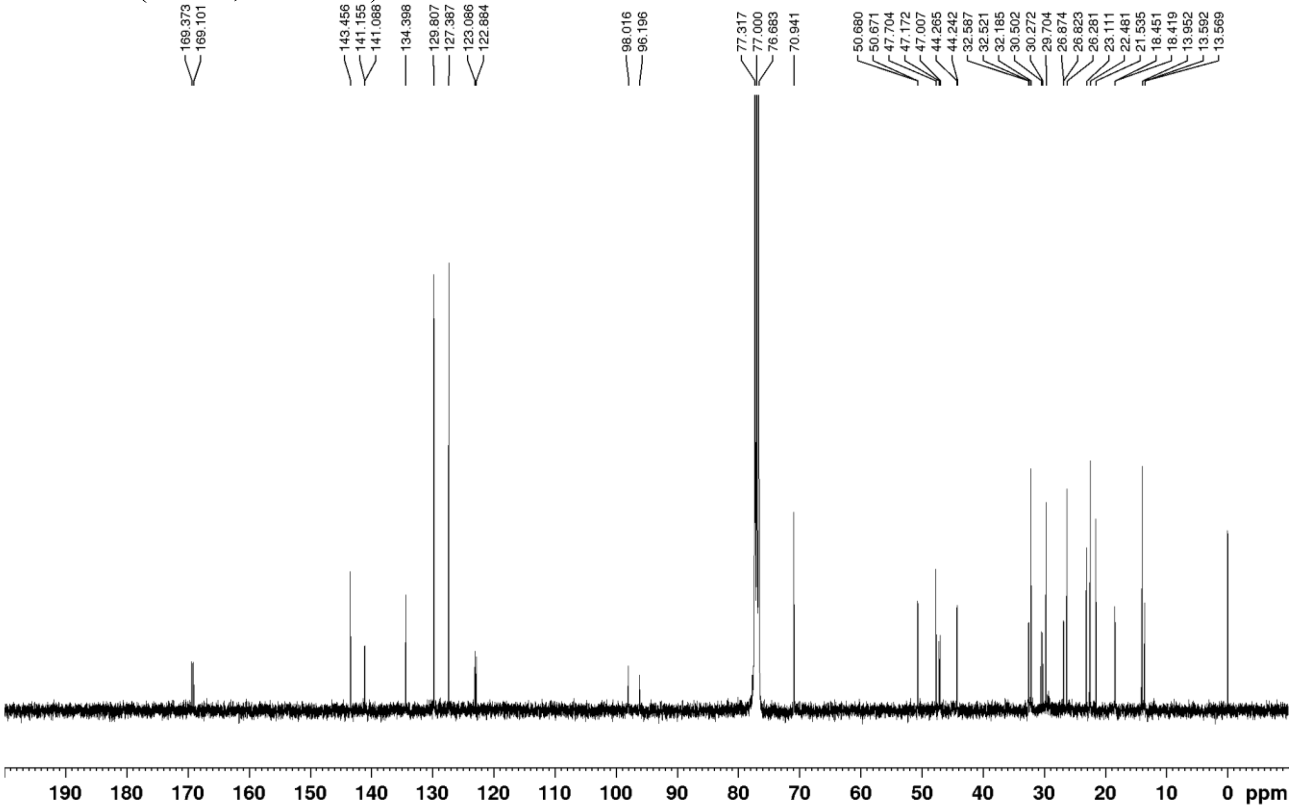

^{19}F NMR ($CDCl_3$, 377 MHz)

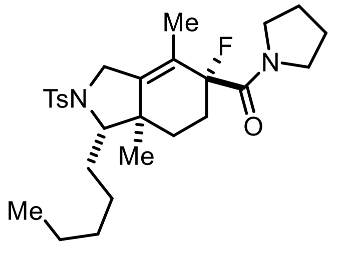

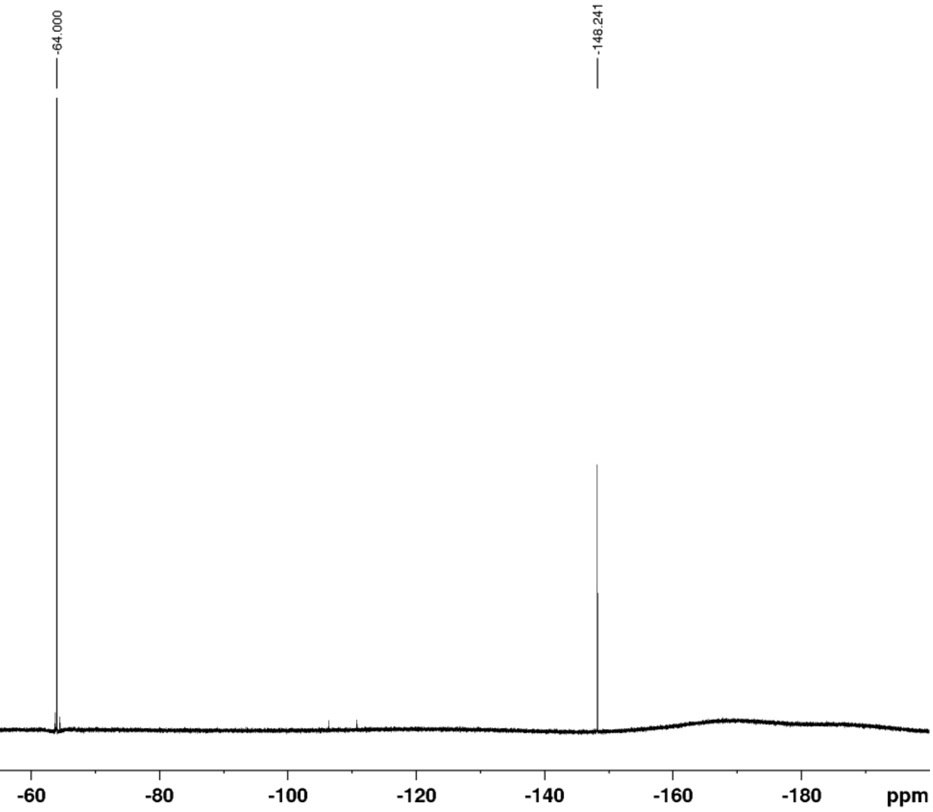

{5-Fluoro-4,7a-dimethyl-2-(4-methylphenyl)-1-pentyl-2,3,5,6,7,7a-hexahydro-1*H*-isoindol-5-yl}(pyrrolidin-1-yl)methanone [3ba (minor diastereomer)]

^{1}H NMR ($CDCl_3$, 400 MHz)

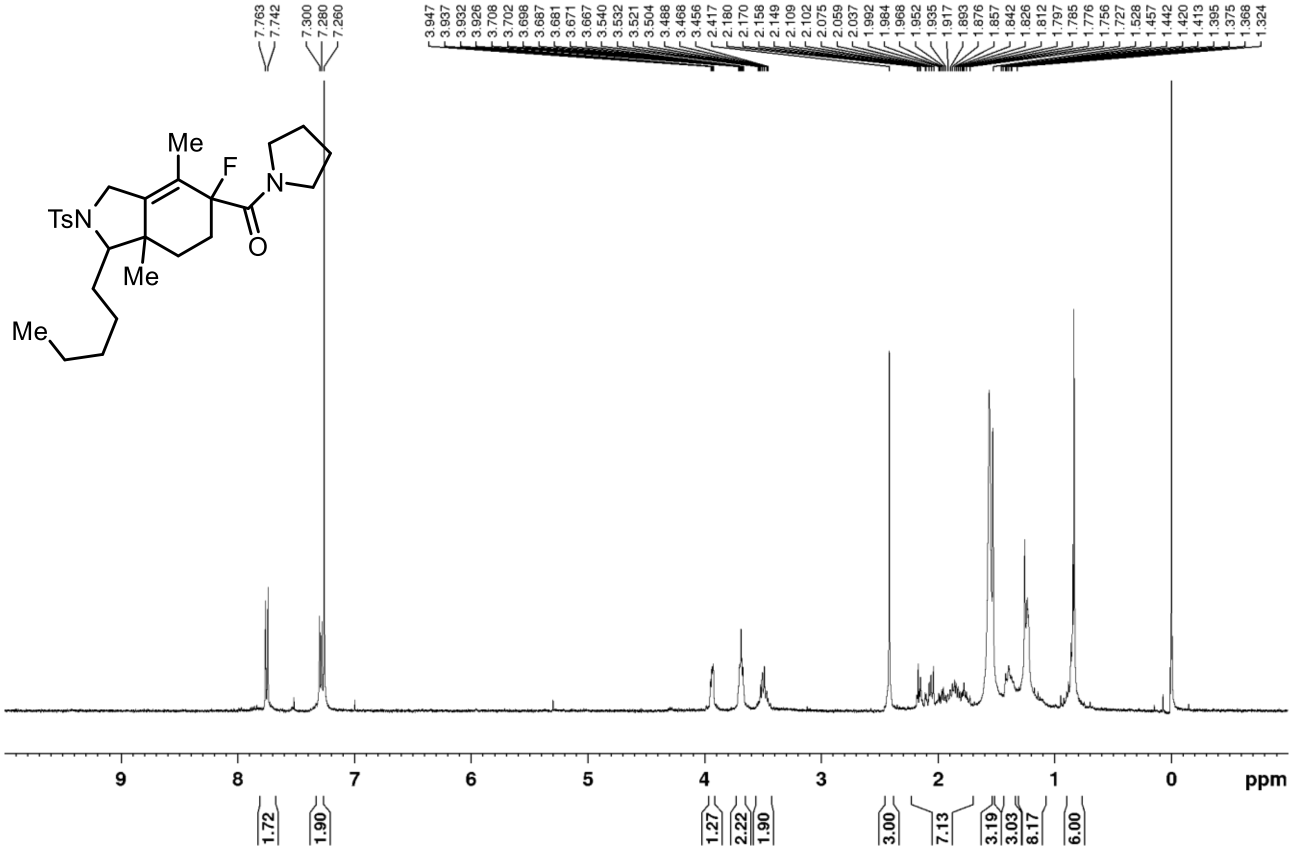

^{19}F NMR ($CDCl_3$, 377 MHz)

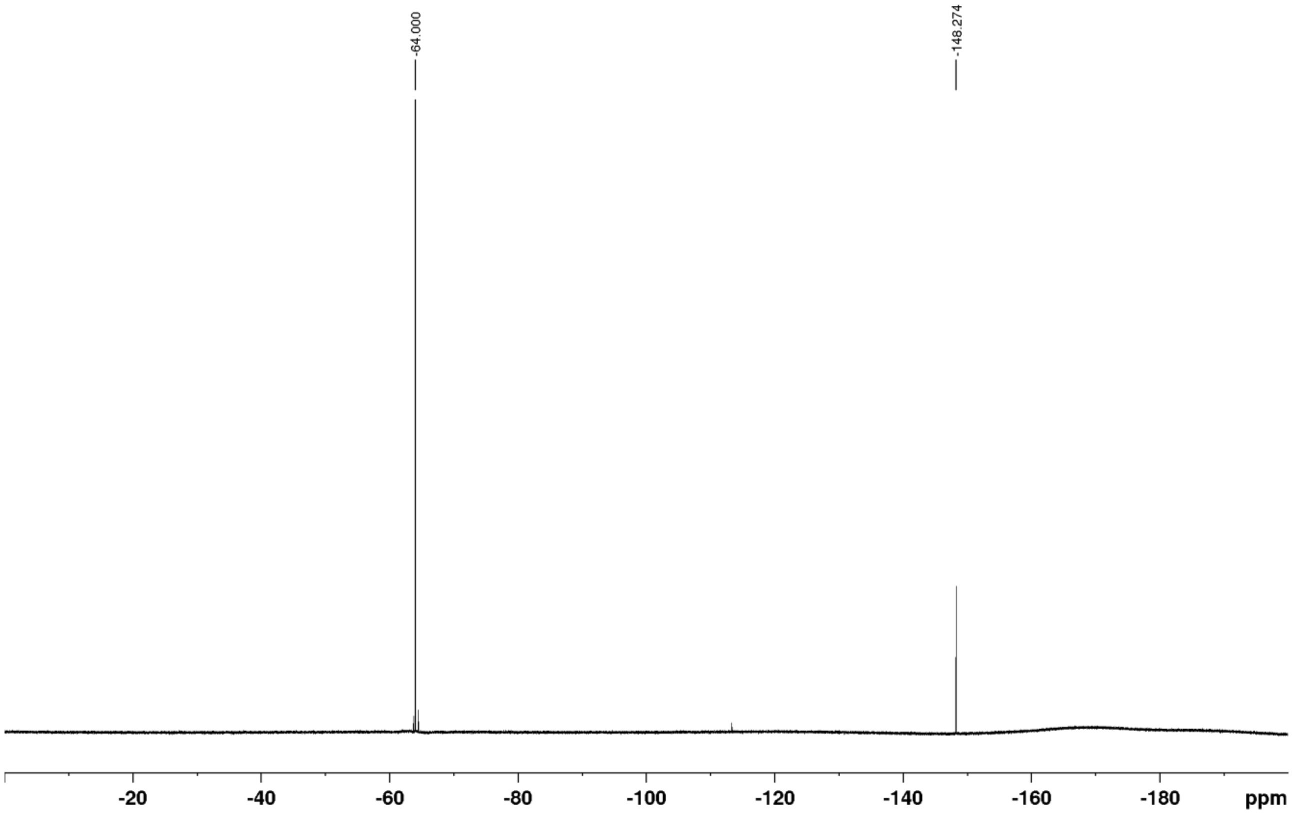

(*E*)-4-{(2*R*,3*R*,*Z*)-4-Ethylidene-3-methyl-1-(4-methylphenyl)pyrrolidin-3-yl}-2-pentyl-2-fluoro-1-(pyrrolidin-1-yl)but-2-en-1-one [4ba (major diastereomer)]

^{1}H NMR (CDCl$_3$, 400 MHz)

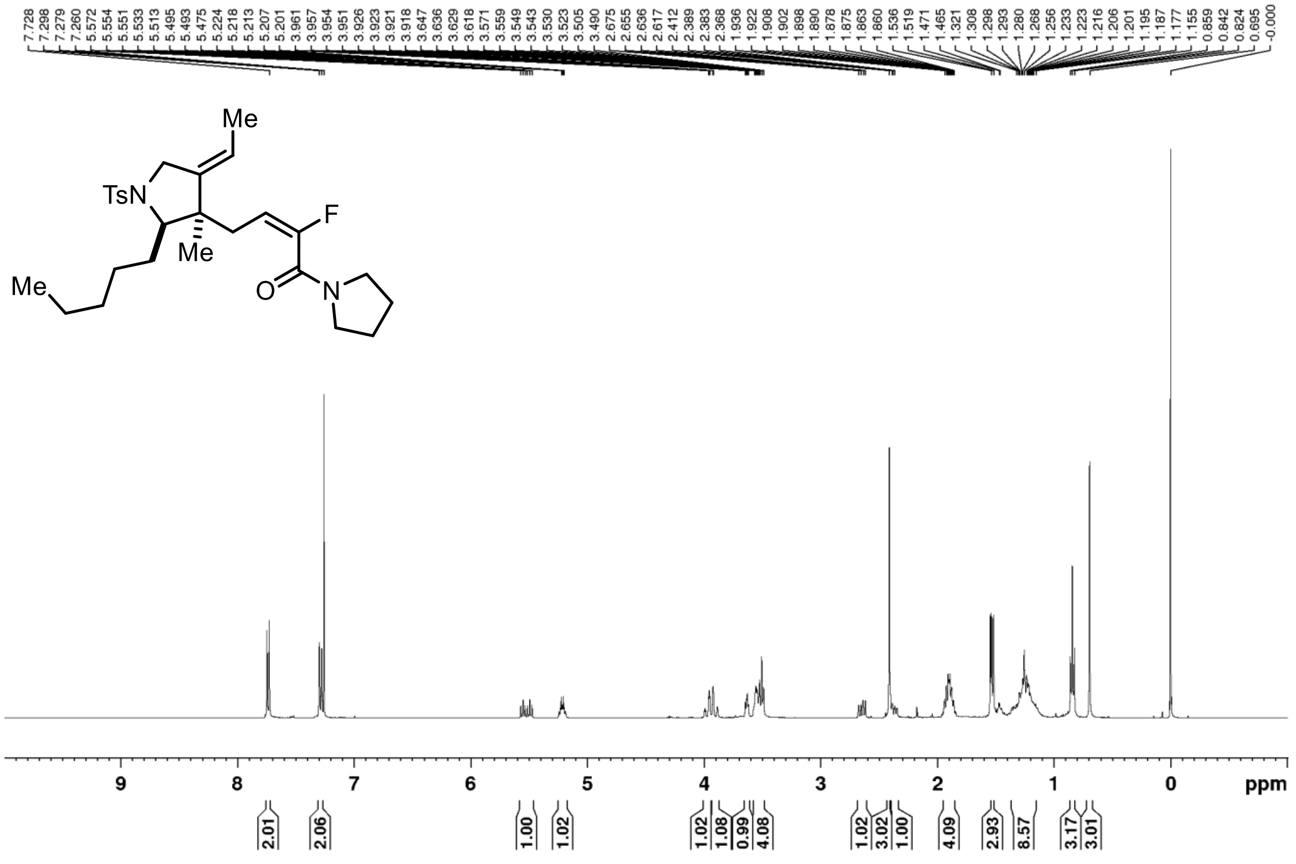

^{13}C NMR (CDCl$_3$, 101 MHz)

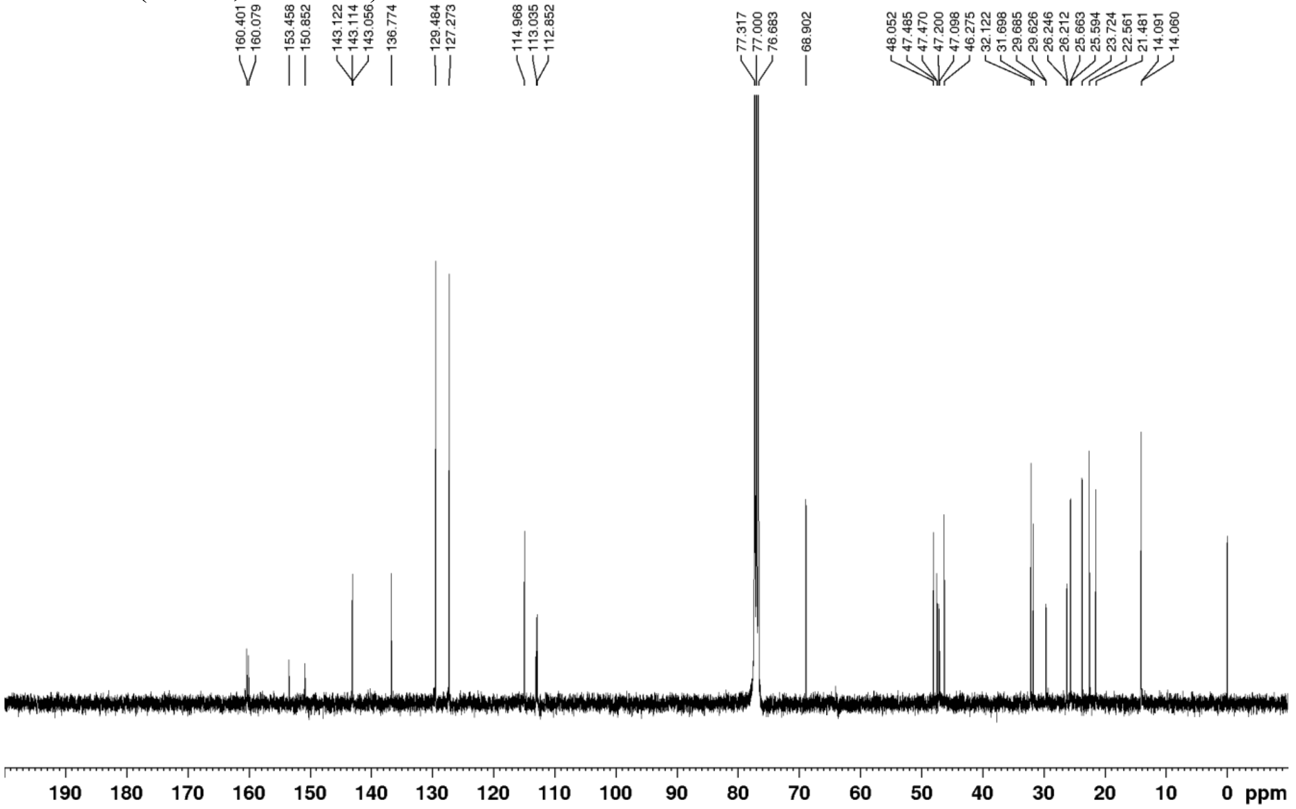

^{19}F NMR ($CDCl_3$, 377 MHz)

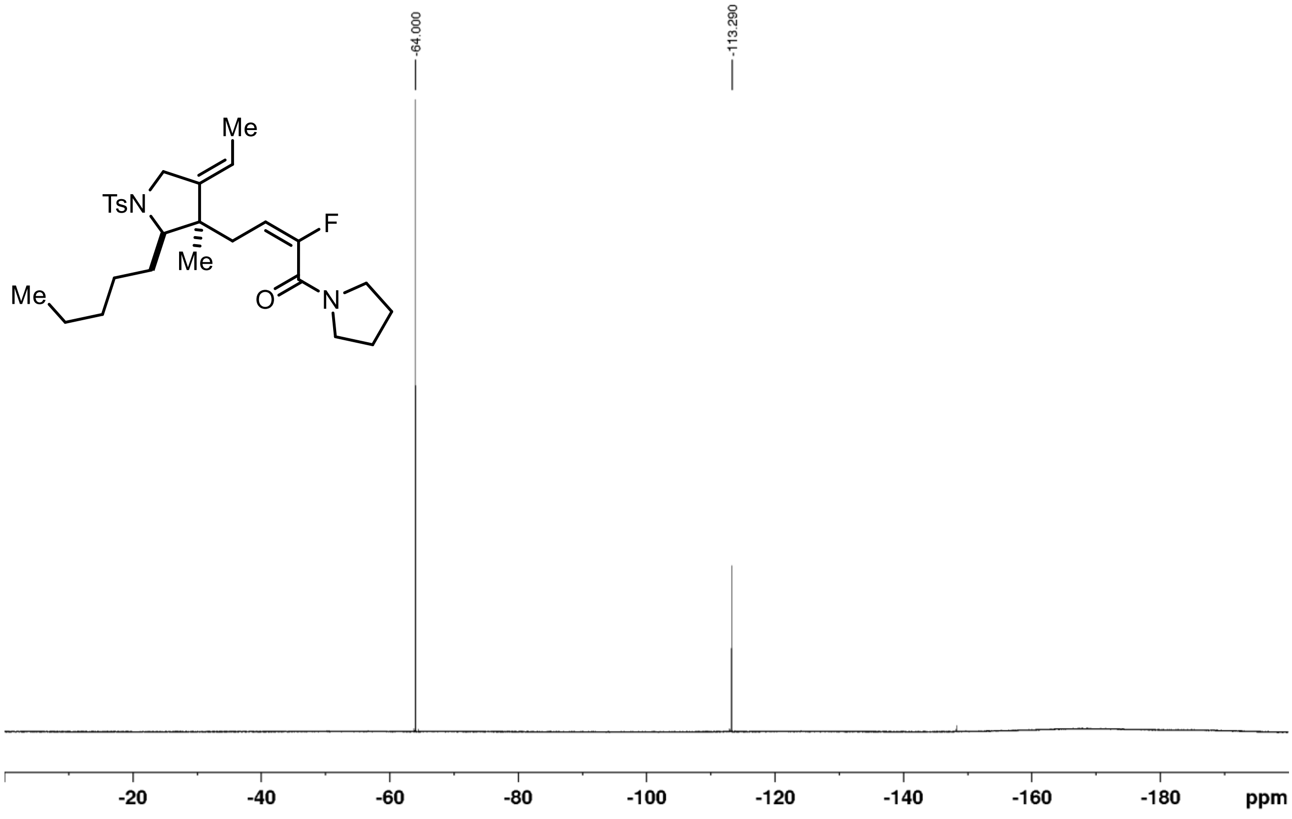

(*E*)-4-{(*Z*)-4-Ethylidene-3-methyl-1-(4-methylphenyl)pyrrolidin-3-yl}-2-pentyl-2-fluoro-1-(pyrrolidin-1-yl)but-2-en-1-one [4ba (minor diastereomer)]

^{1}H NMR ($CDCl_3$, 400 MHz)

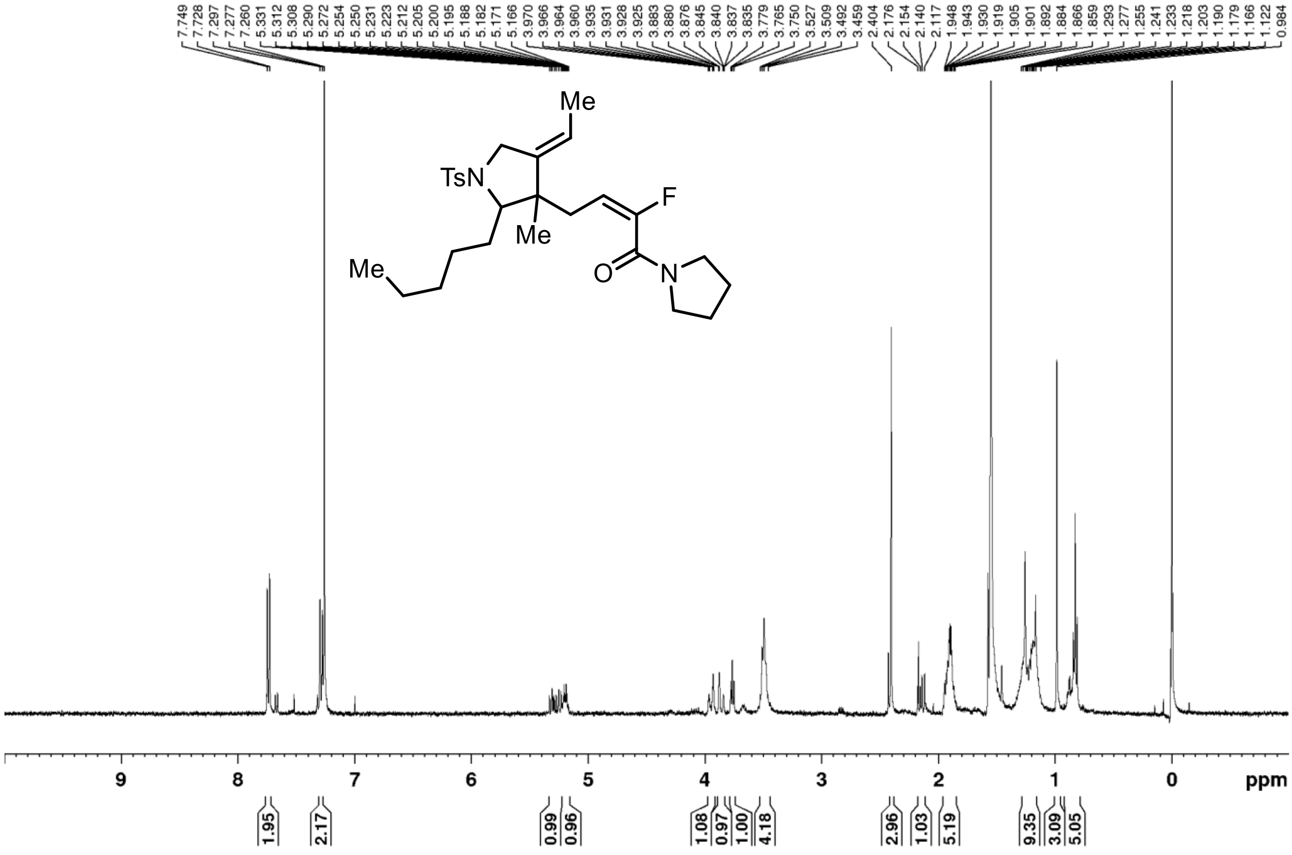

^{19}F NMR ($CDCl_3$, 377 MHz)

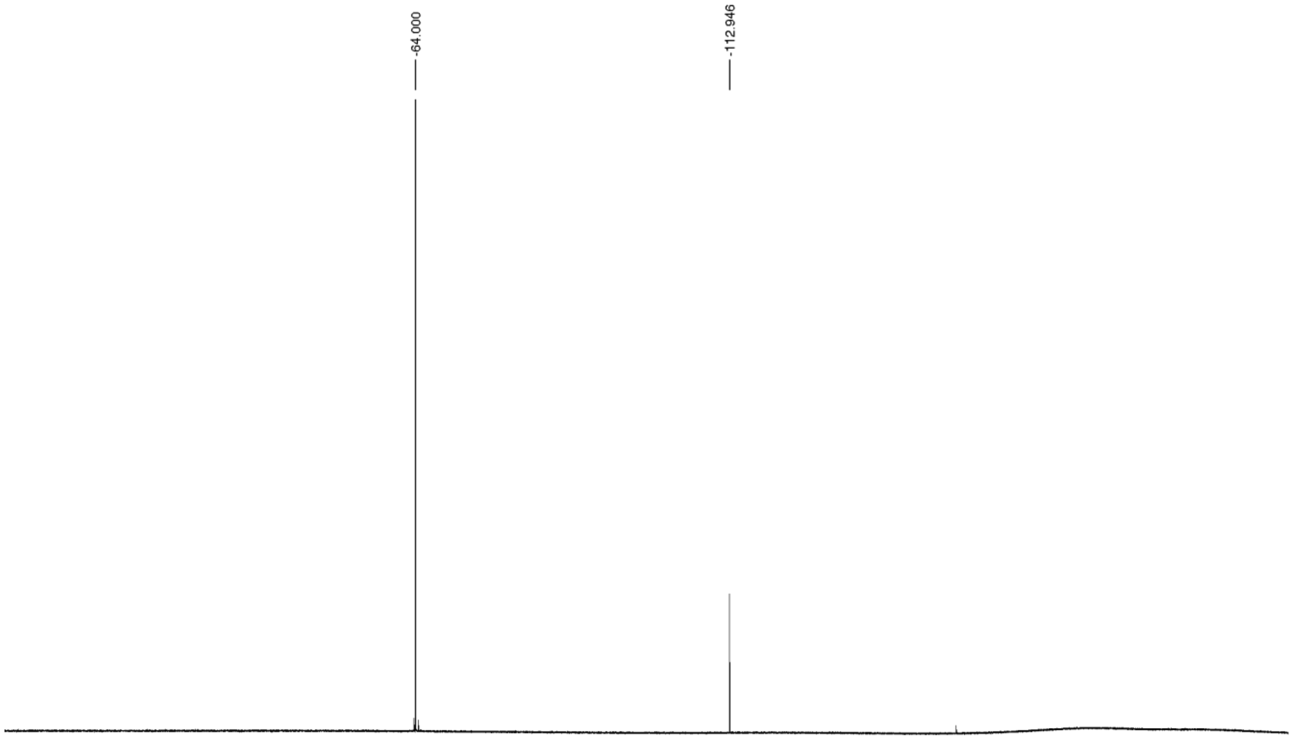

{(1*S*,5*R*,7a*R*)-5-Fluoro-4,7a-dimethyl-2-(4-methylphenyl)-1-phenyl-2,3,5,6,7,7a-hexahydro-1*H*-isoindol-5-yl}(pyrrolidin-1-yl)methanone (3ca)

^{1}H NMR ($CDCl_3$, 400 MHz)

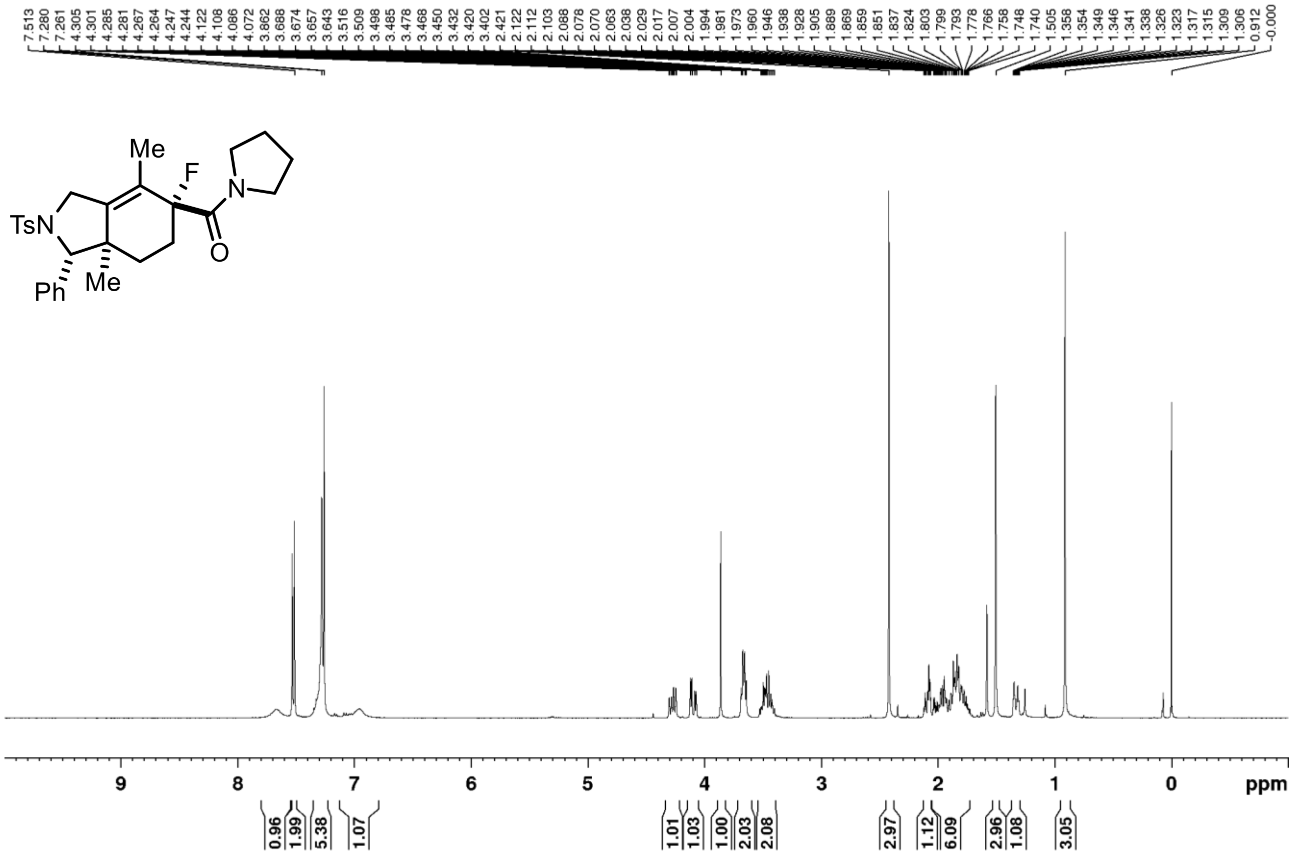

^{13}C NMR ($CDCl_3$, 101 MHz)

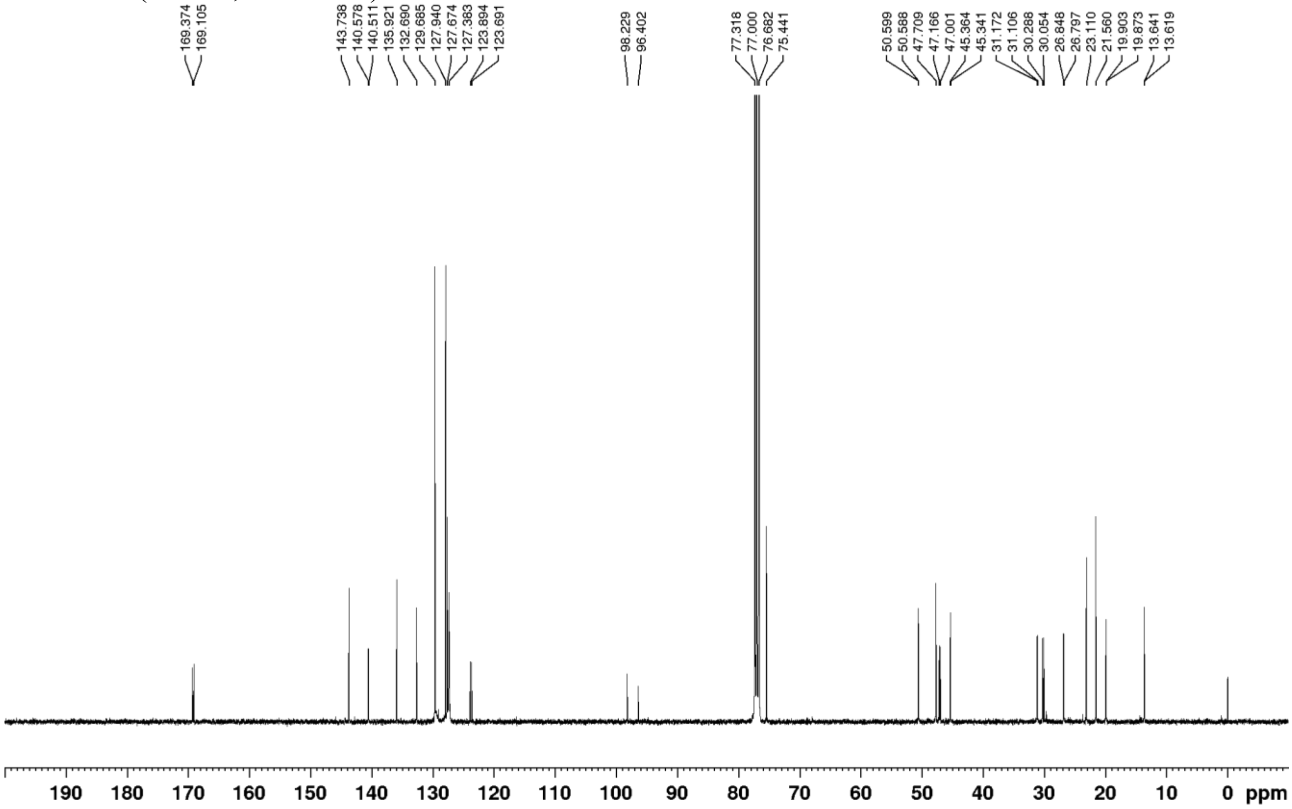

^{19}F NMR ($CDCl_3$, 377 MHz)

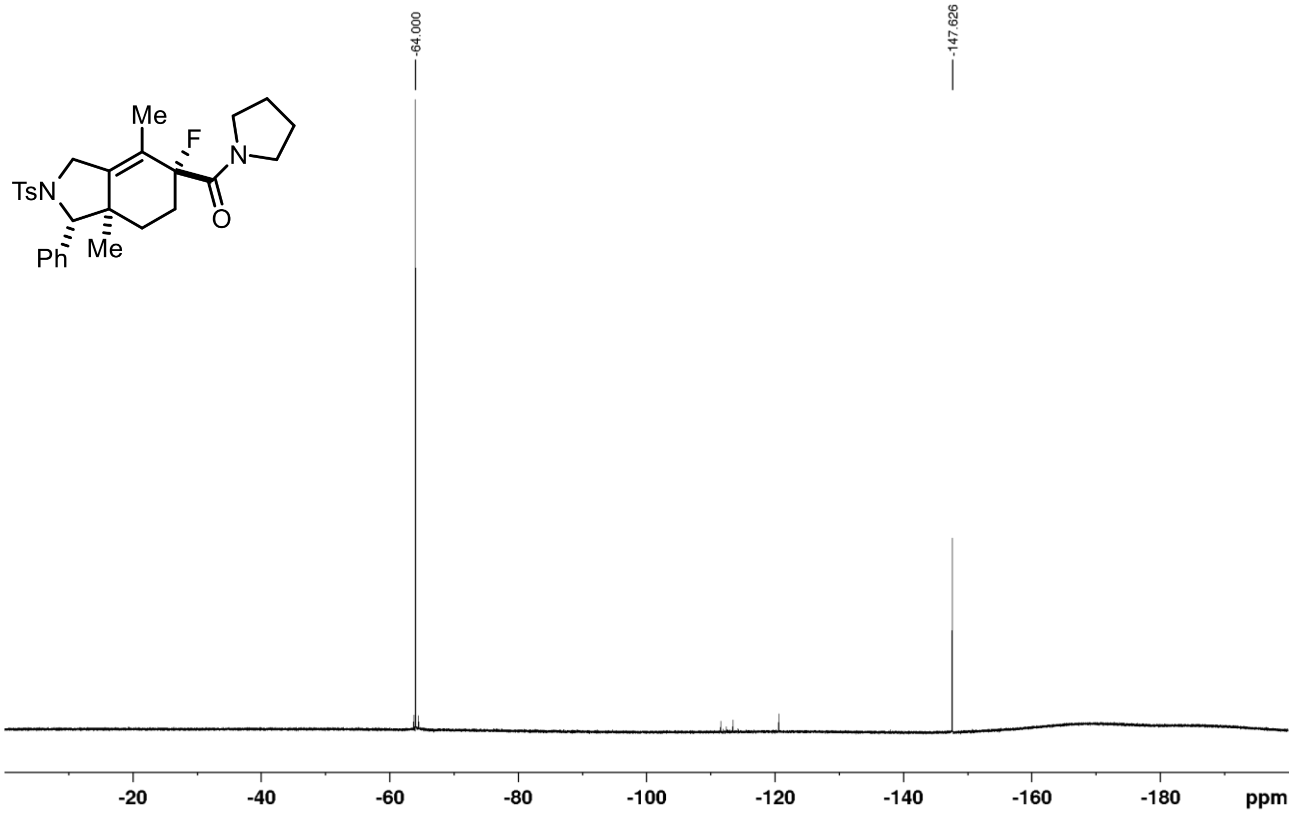

(*E*)-4-{(2*R*,3*R*,*Z*)-4-Ethylidene-3-methyl-2-phenyl-1-(4-methylphenyl)pyrrolidin-3-yl}-2-fluoro-1-(pyrrolidin-1-yl)but-2-en-1-one [4ca (major diastereomer)]

^{1}H NMR ($CDCl_3$, 400 MHz)

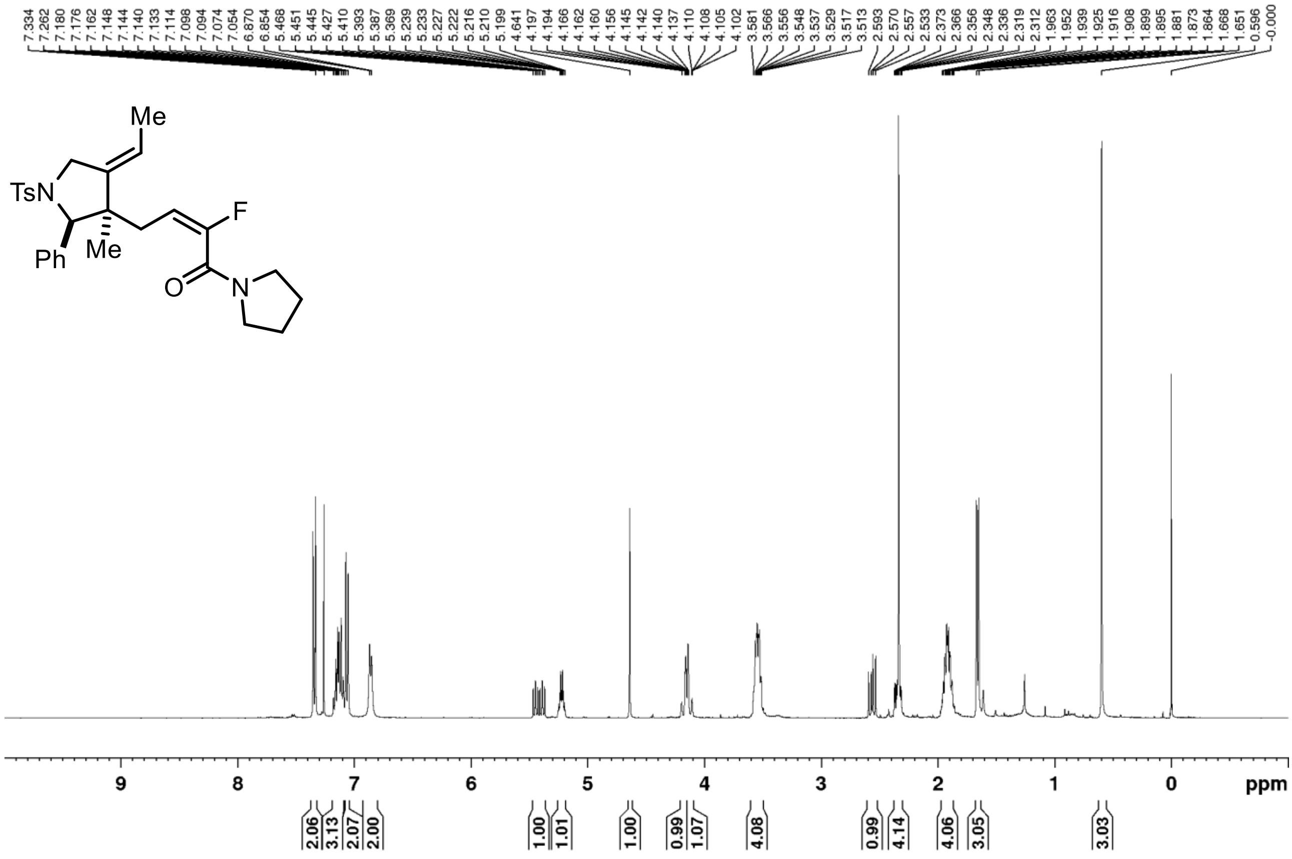

^{13}C NMR ($CDCl_3$, 101 MHz)

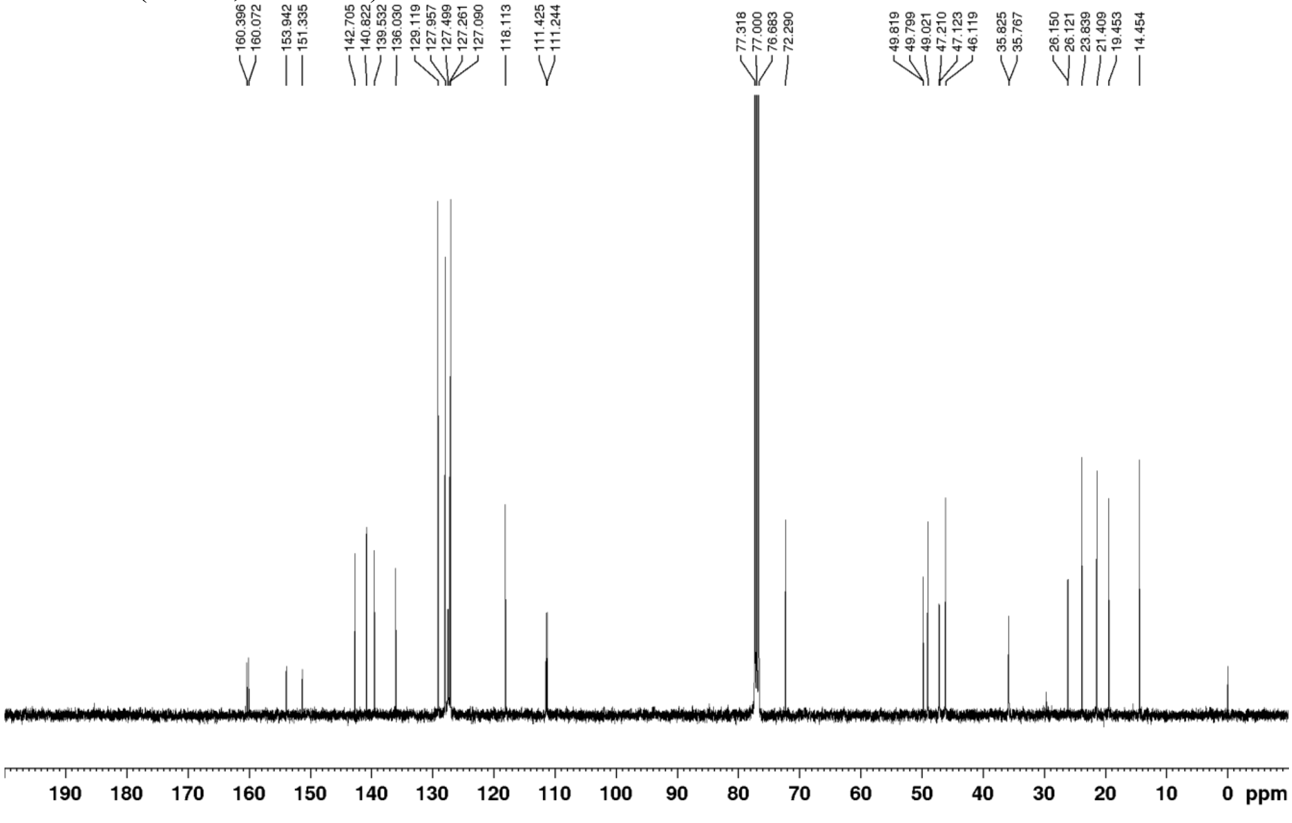

^{19}F NMR ($CDCl_3$, 377 MHz)

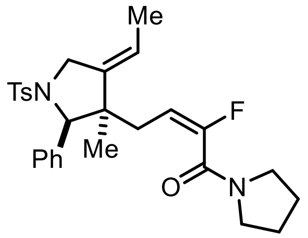

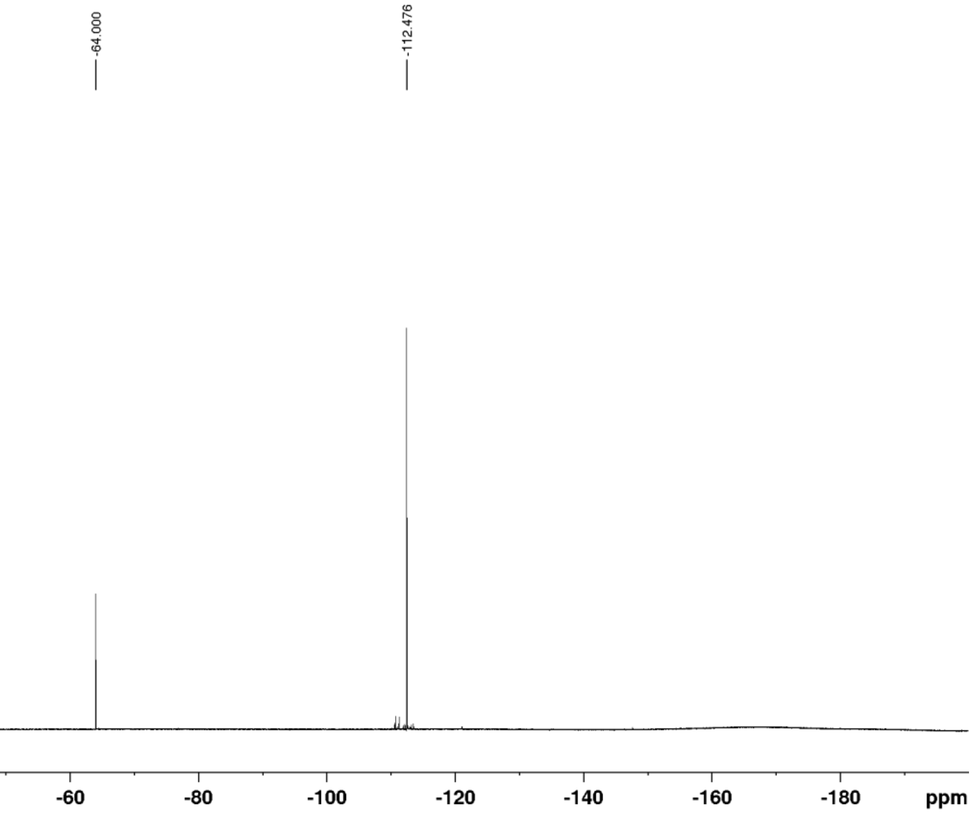

(*E*)-4-{(*Z*)-4-Ethylidene-3-methyl-2-phenyl-1-(4-methylphenyl)pyrrolidin-3-yl}-2-fluoro-1-(pyrrolidin-1-yl)but-2-en-1-one [4ca (minor diastereomer)]

^{1}H NMR ($CDCl_3$, 400 MHz)

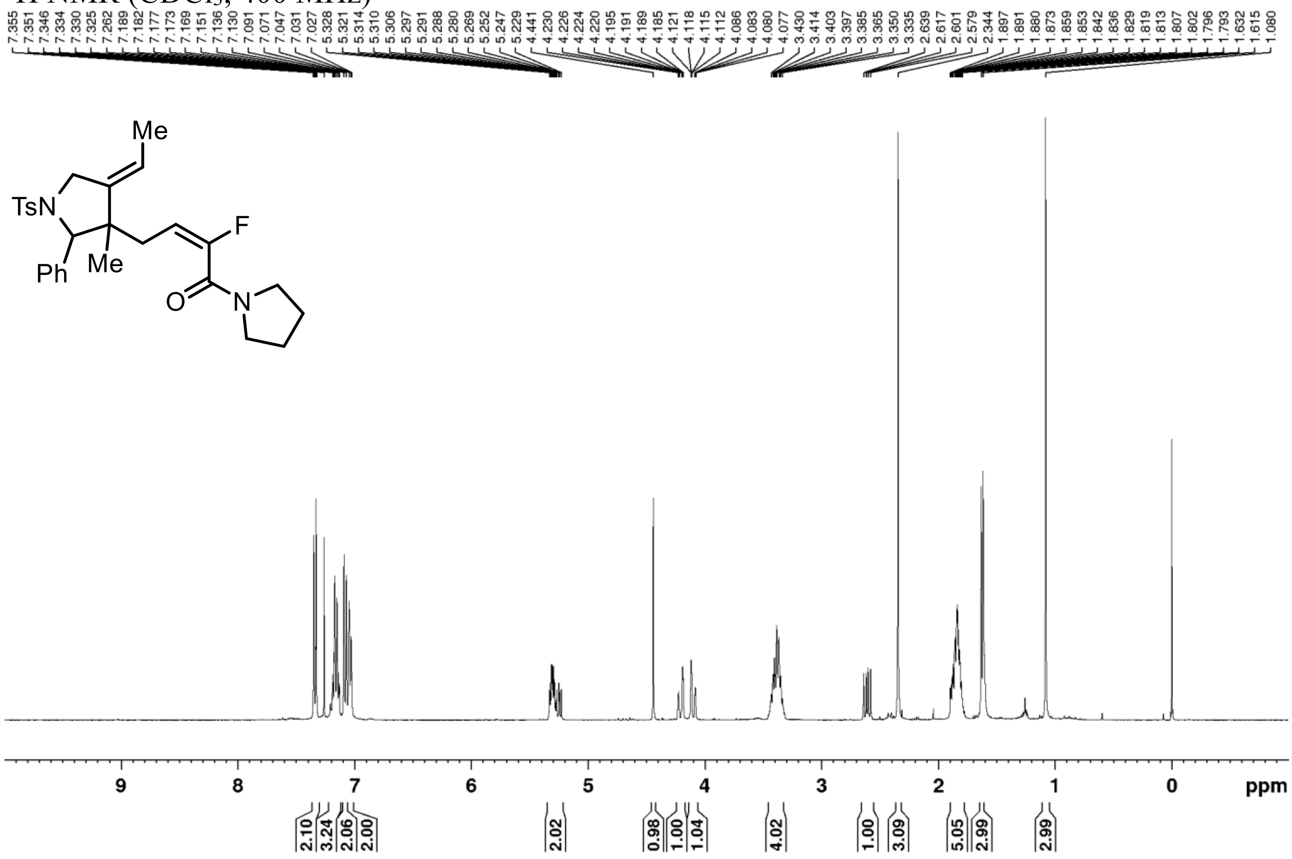

^{13}C NMR ($CDCl_3$, 101 MHz)

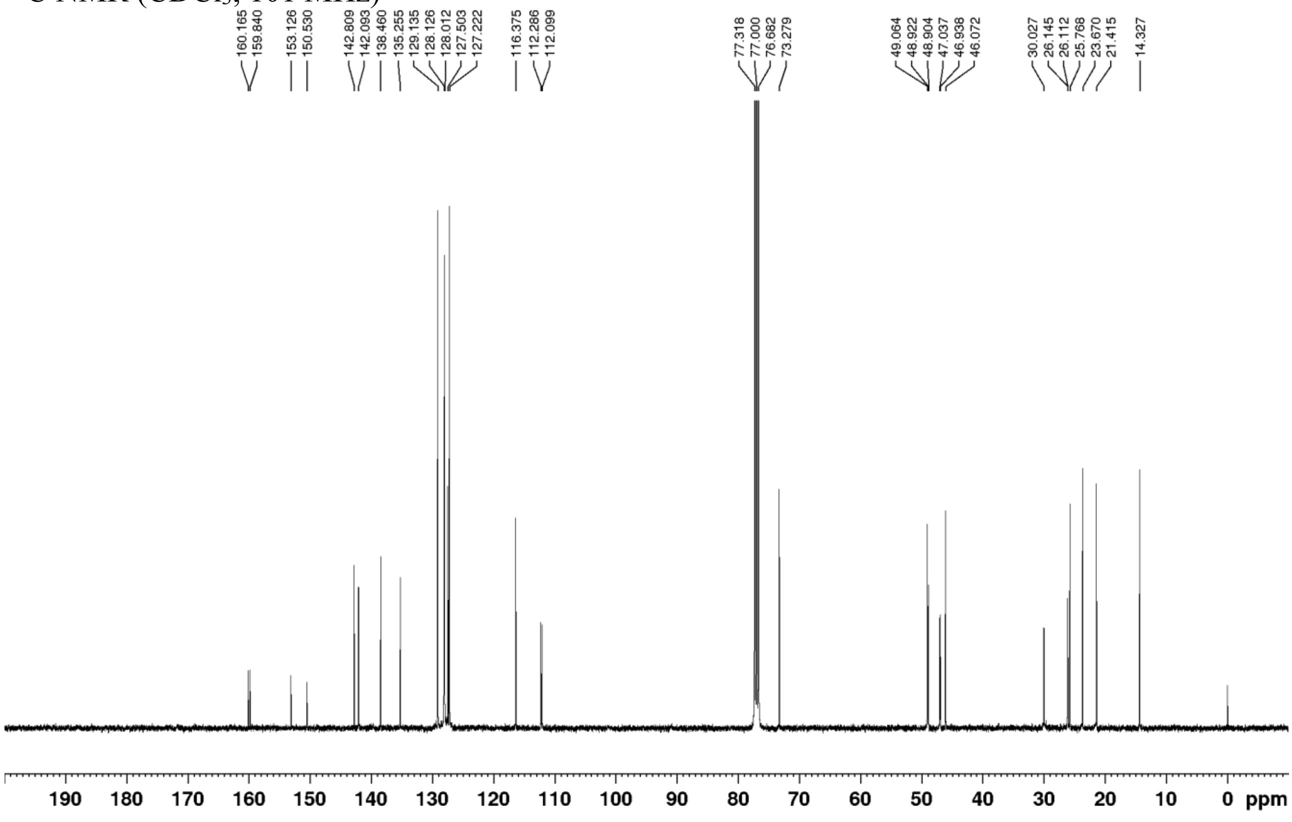

^{19}F NMR ($CDCl_3$, 377 MHz)

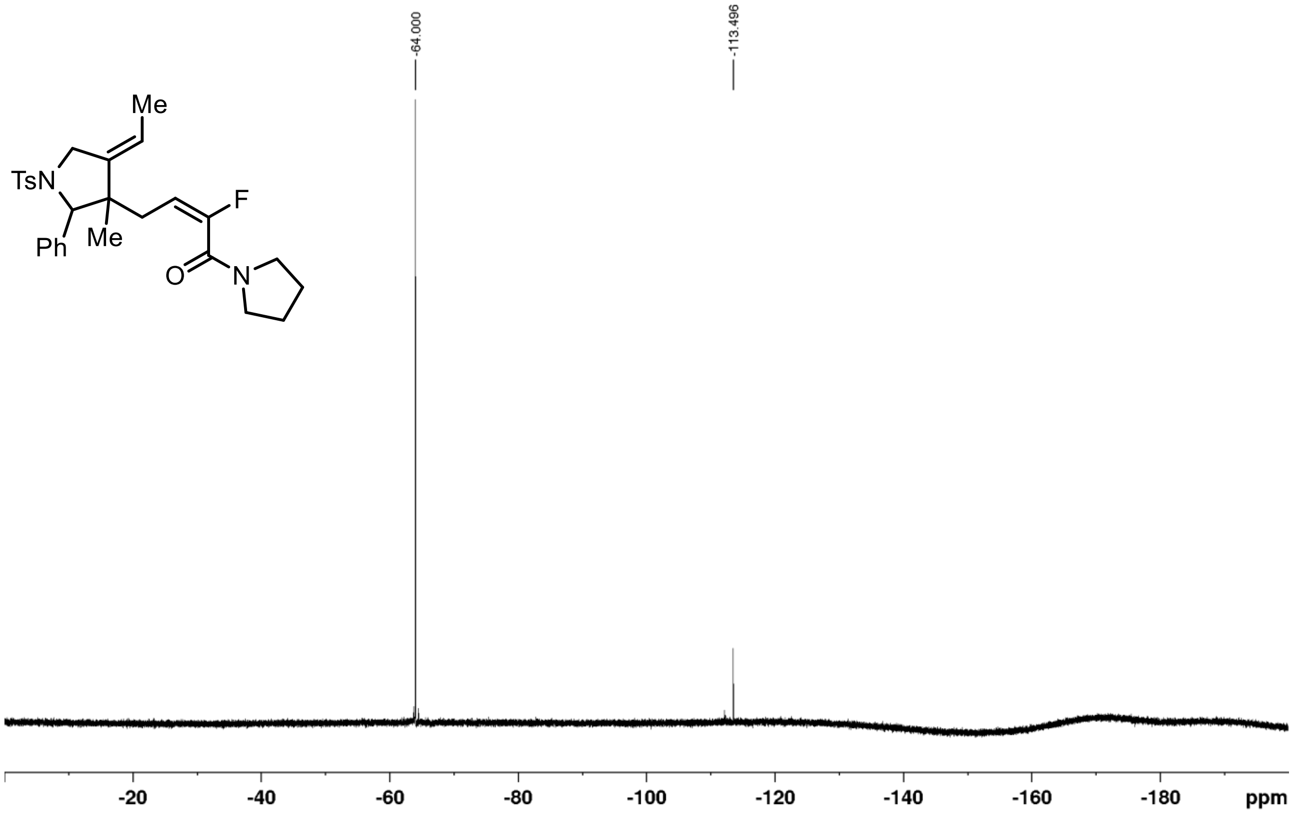

{(1*S*,5*R*,7a*R*)-5-Fluoro-1,7a-dimethyl-2-(4-methylphenyl)-4-phenyl-2,3,5,6,7,7a-hexahydro-1*H*-isoindol-5-yl}(pyrrolidin-1-yl)methanone [3da (major diastereomer)]

^{1}H NMR ($CDCl_3$, 400 MHz)

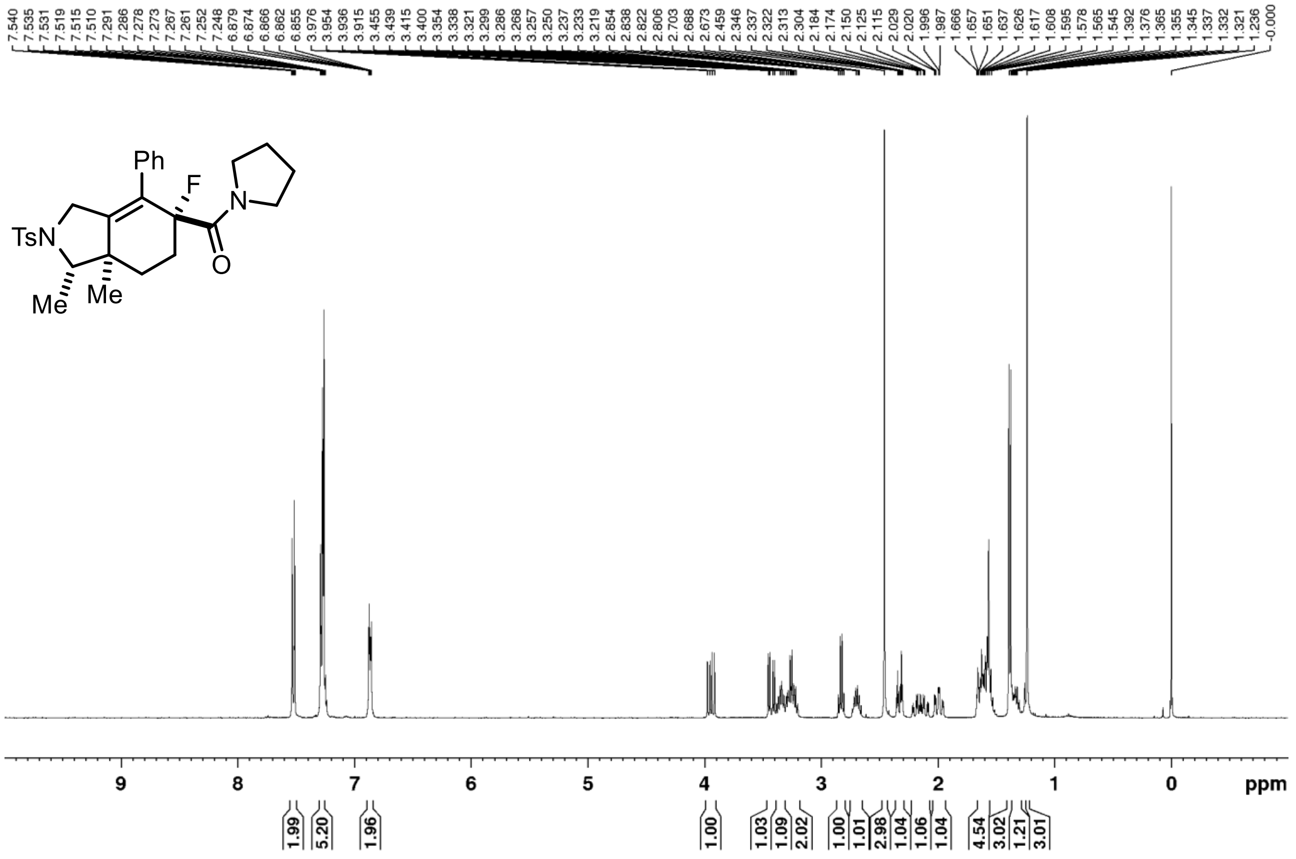

^{13}C NMR ($CDCl_3$, 101 MHz)

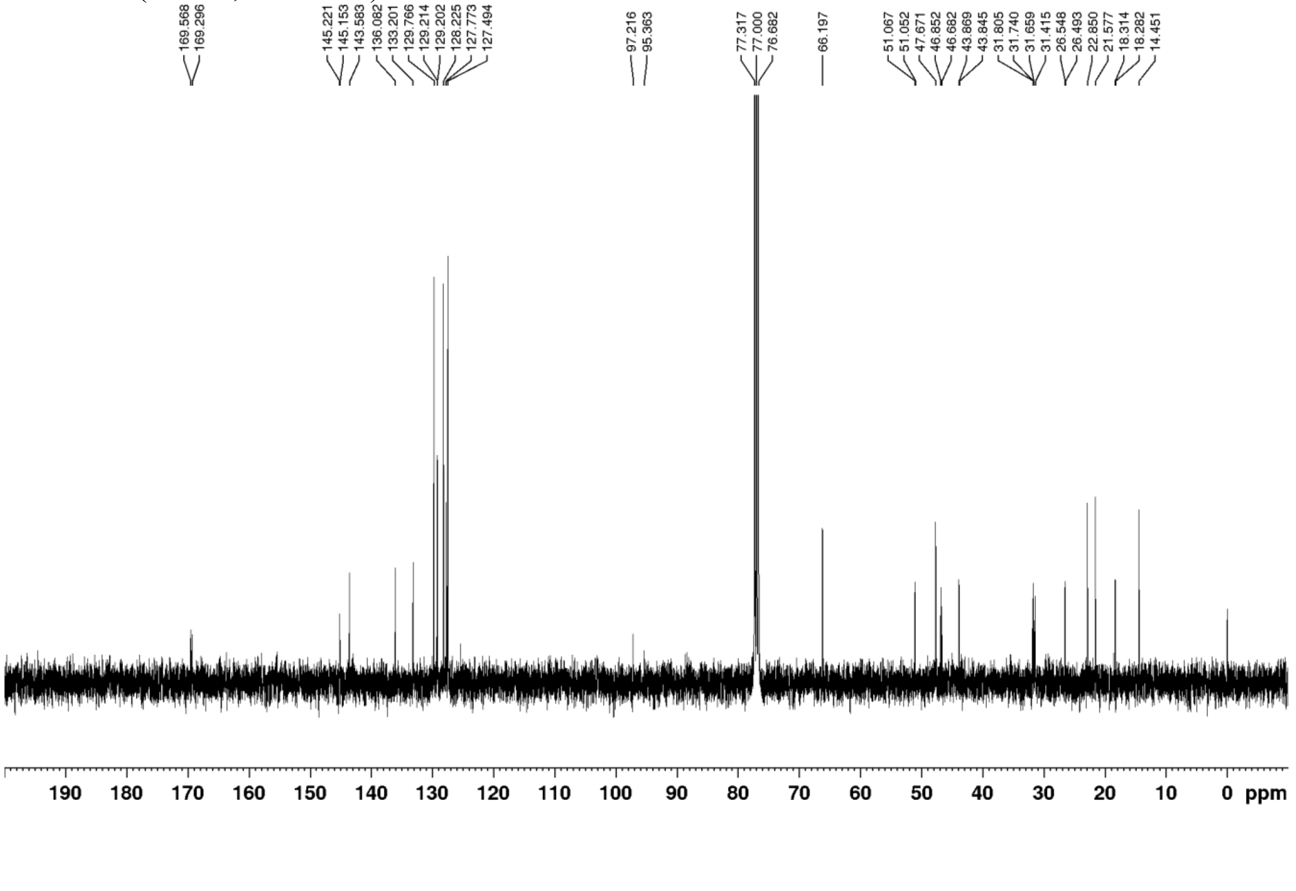

^{19}F NMR ($CDCl_3$, 377 MHz)

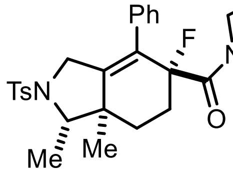

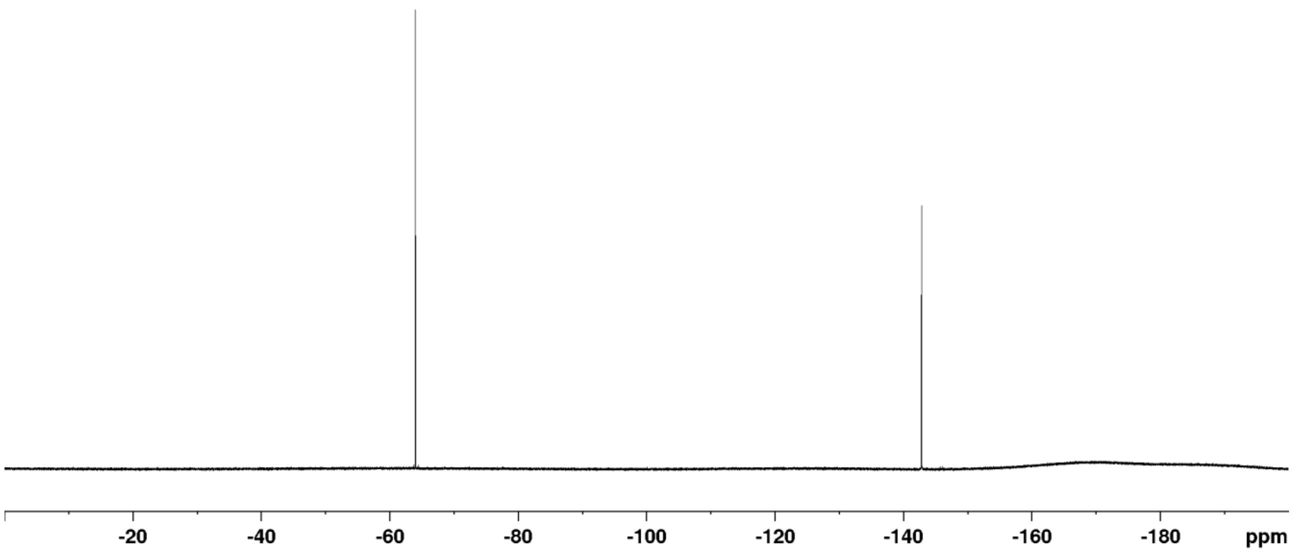

{5-Fluoro-1,7a-dimethyl-2-(4-methylphenyl)-4-phenyl-2,3,5,6,7,7a-hexahydro-1*H*-isoindol-5-yl}(pyrrolidin-1-yl)methanone [3da (minor diastereomer)]

^{1}H NMR (CDCl$_3$, 400 MHz)

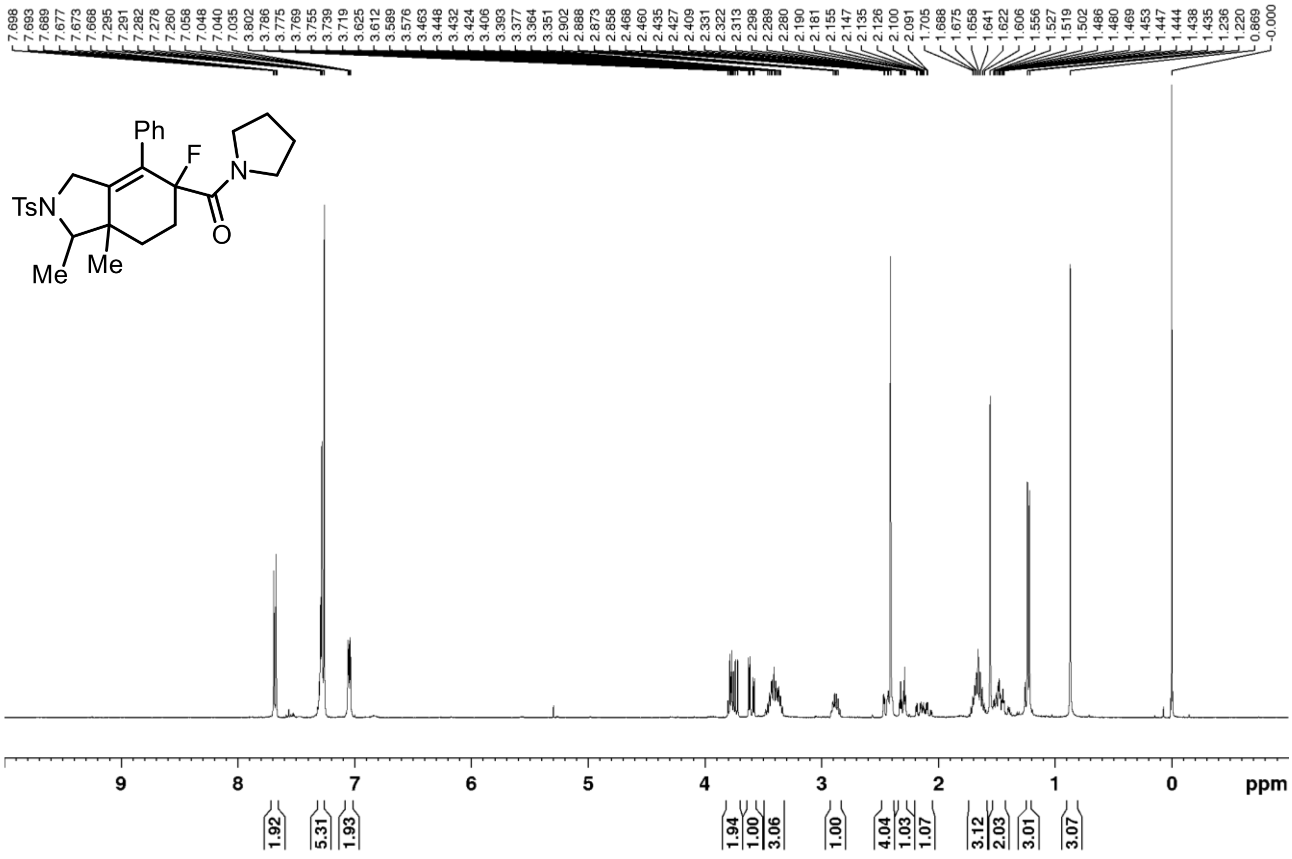

^{13}C NMR (CDCl$_3$, 101 MHz)

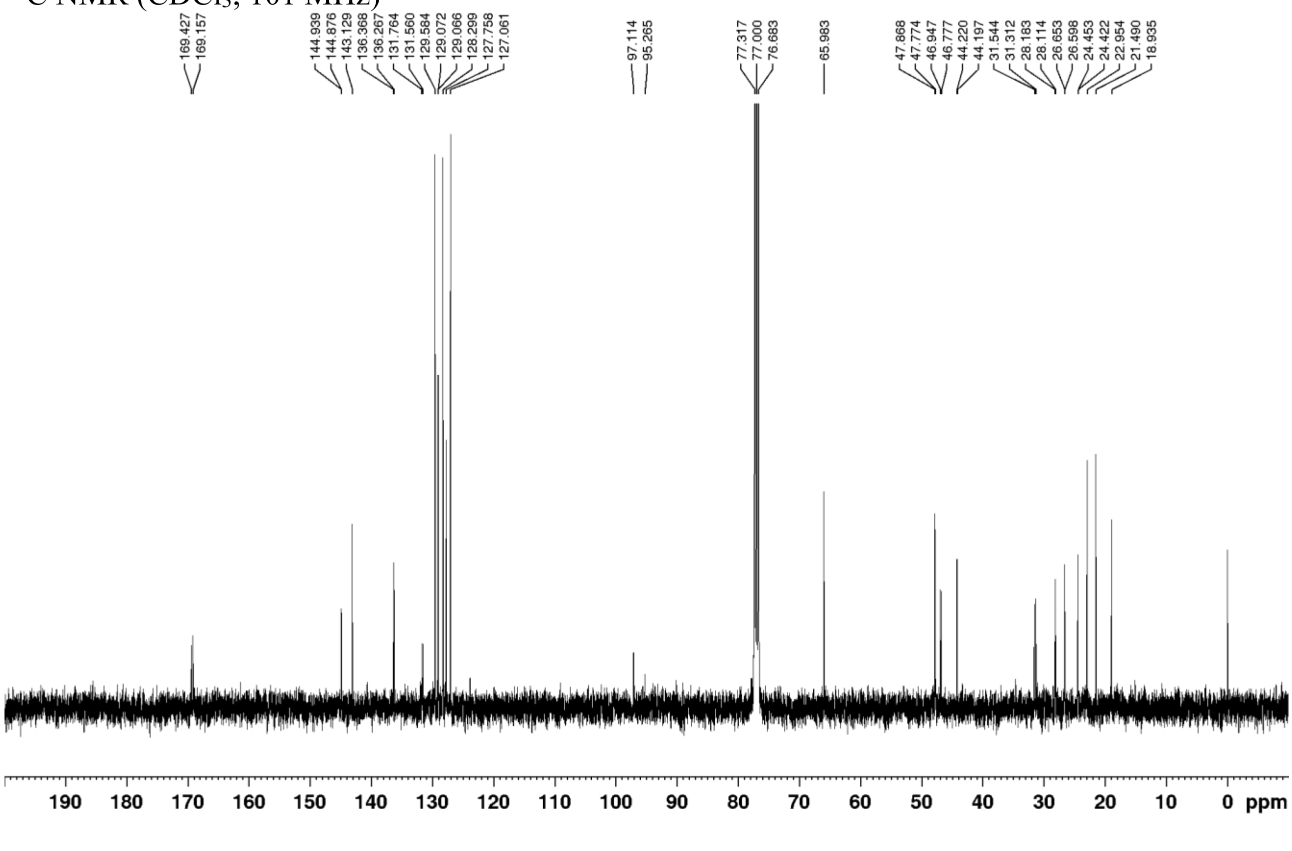

^{19}F NMR ($CDCl_3$, 377 MHz)

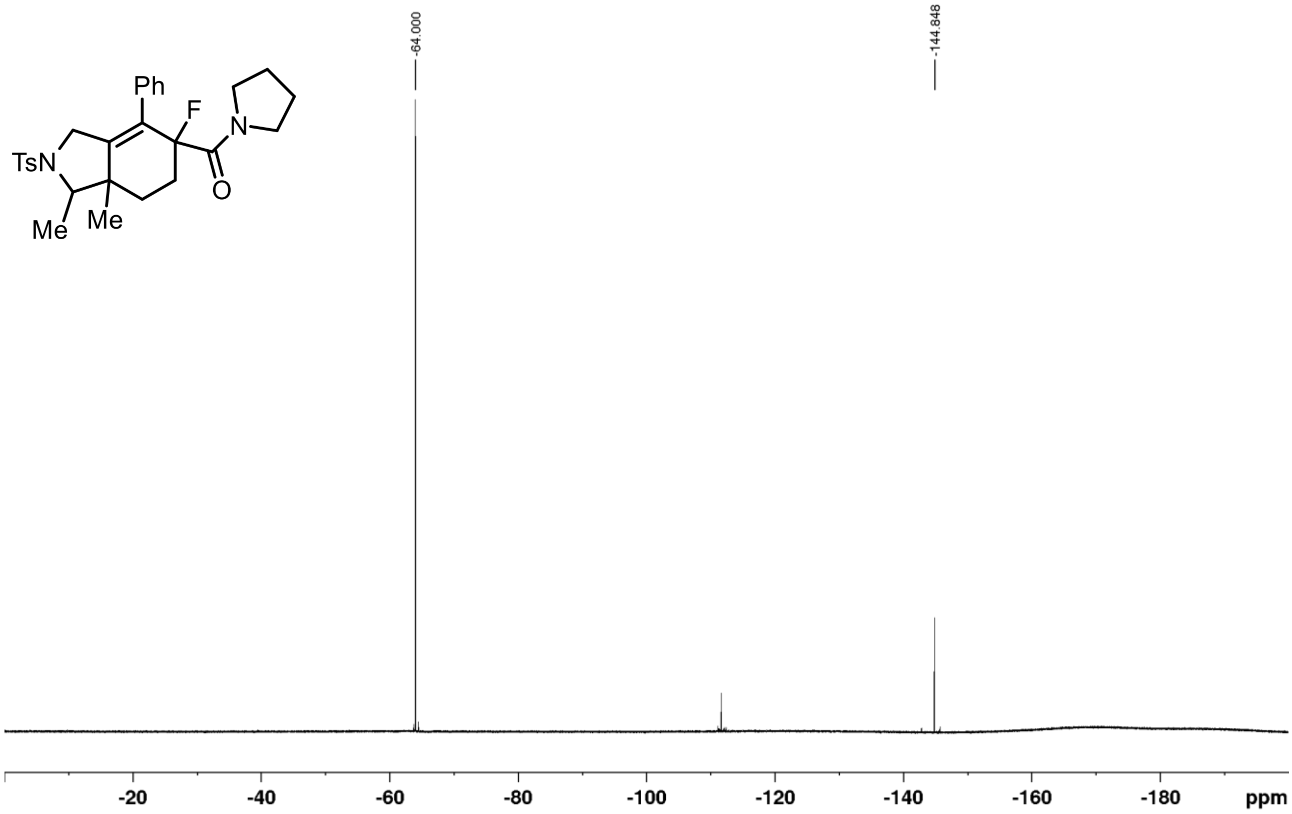

(*E*)-4-{(2*R*,3*R*)-4-(*Z*)-Benzylidene-2,3-dimethyl-1-(4-methylphenyl)-pyrrolidin-3-yl}-2-fluoro-1-(pyrrolidin-1-yl)but-2-en-1-one (4da)

Diastereomer mixture (58:42 d.r.)

^{1}H NMR ($CDCl_3$, 400 MHz)

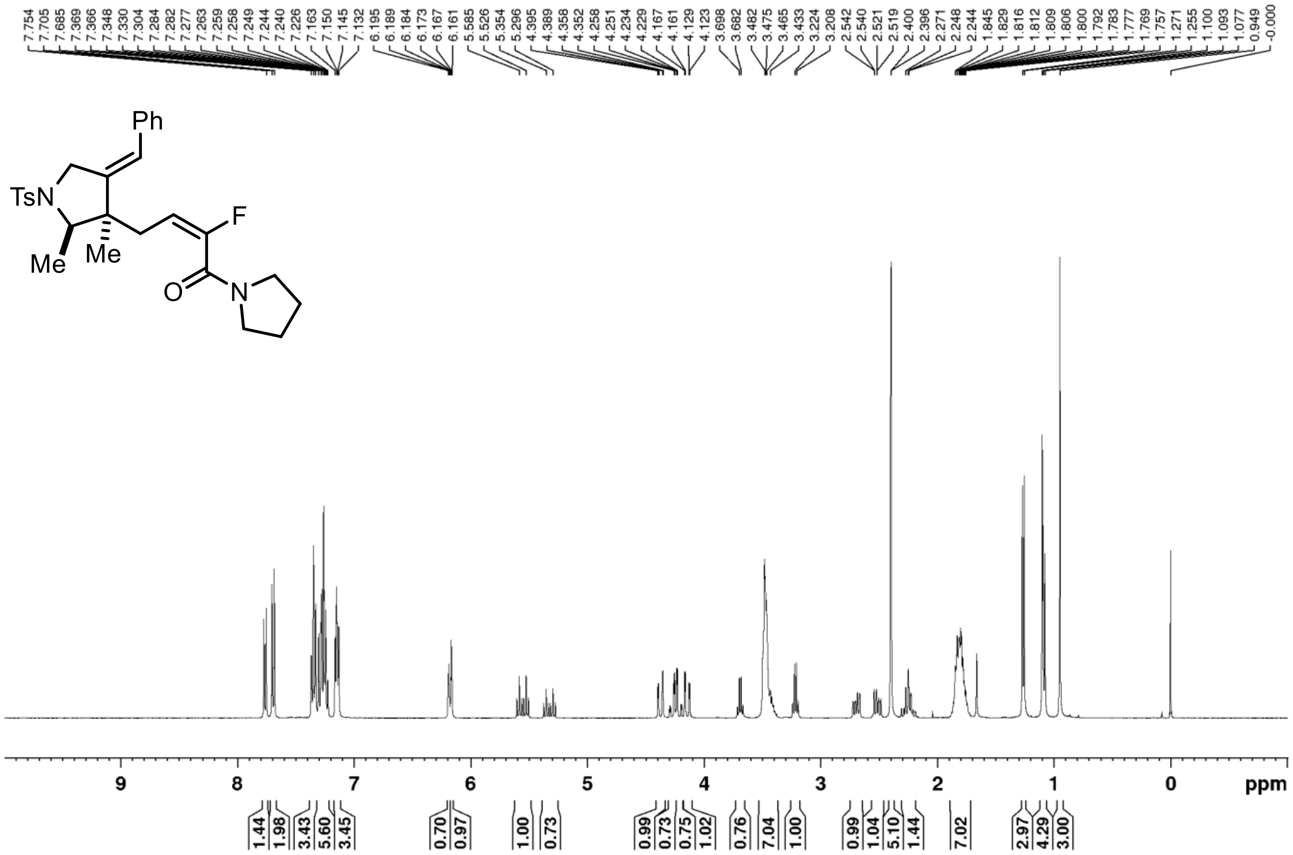

^{13}C NMR ($CDCl_3$, 101 MHz)

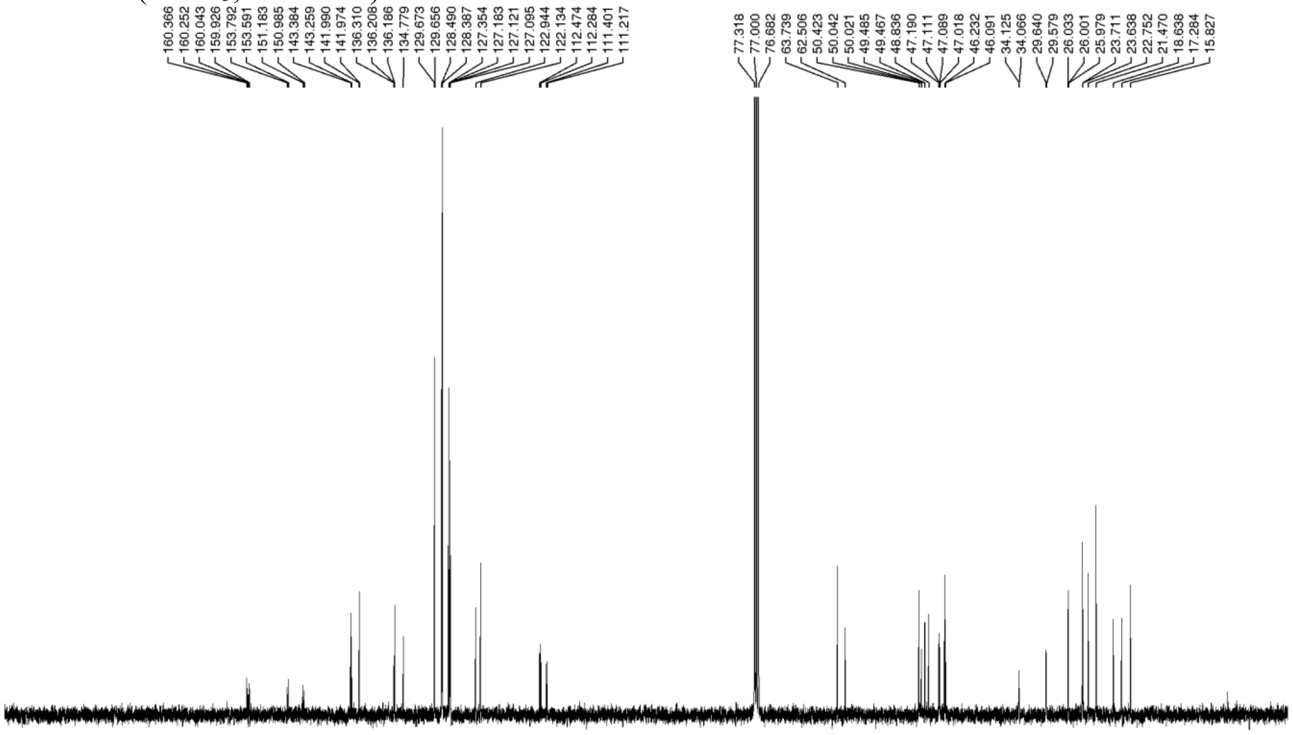

^{19}F NMR ($CDCl_3$, 377 MHz)

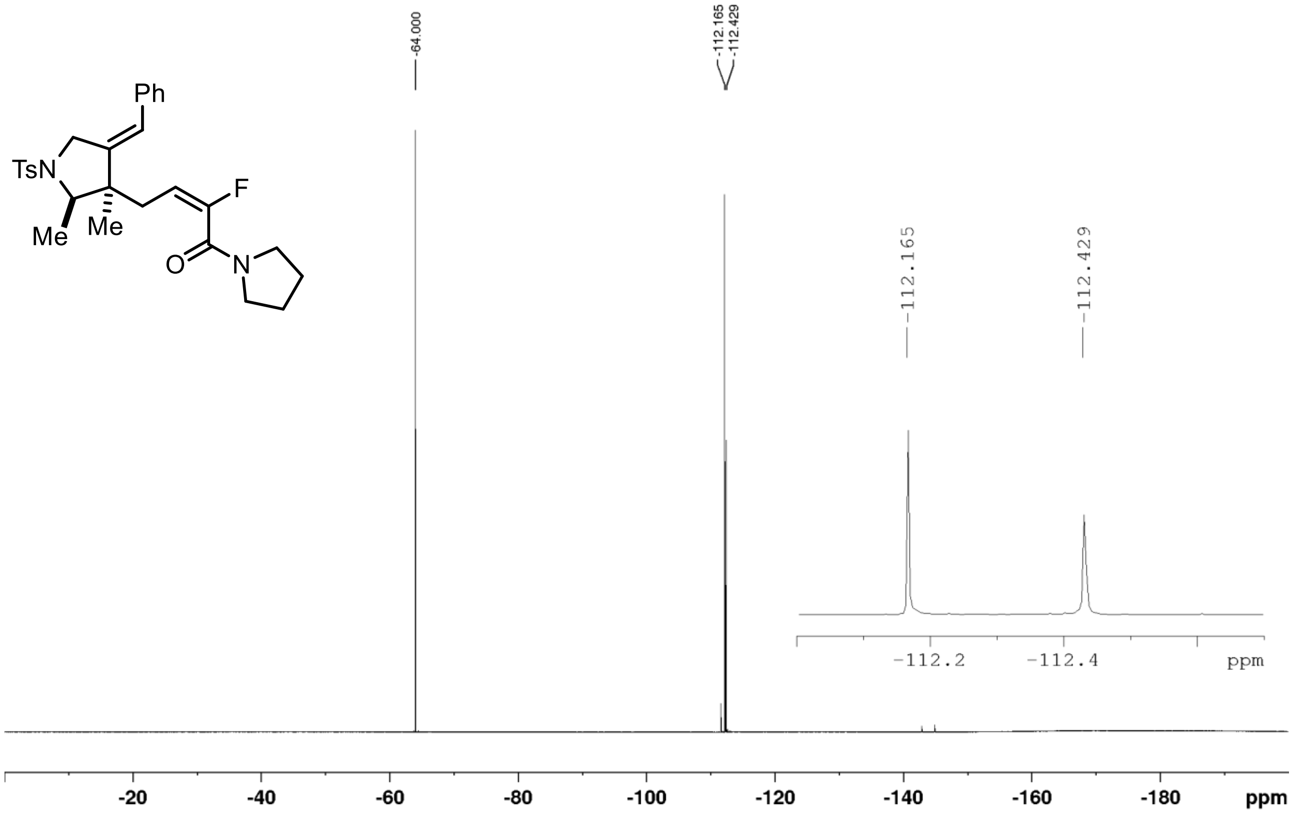

{(1*S*,5*R*,7a*R*)-5-Fluoro-4-(4-methoxyphenyl)-1,7a-dimethyl-2-(4-methylphenyl)-2,3,5,6,7,7a-hexahydro-1*H*-isoindol-5-yl}(pyrrolidin-1-yl)methanone (3ea)

Diastereomer mixture (70:30 d.r.)

^{1}H NMR ($CDCl_3$, 400 MHz)

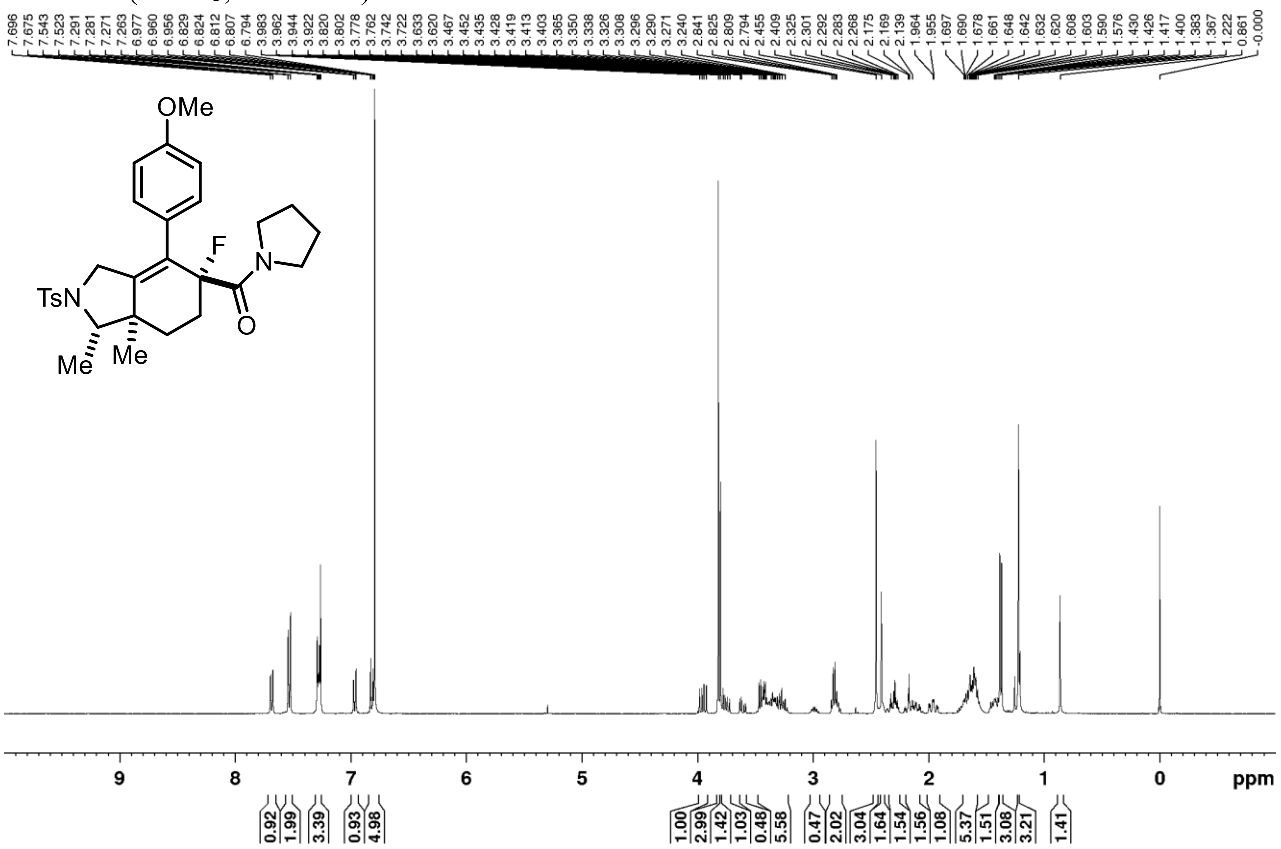

^{13}C NMR ($CDCl_3$, 101 MHz)

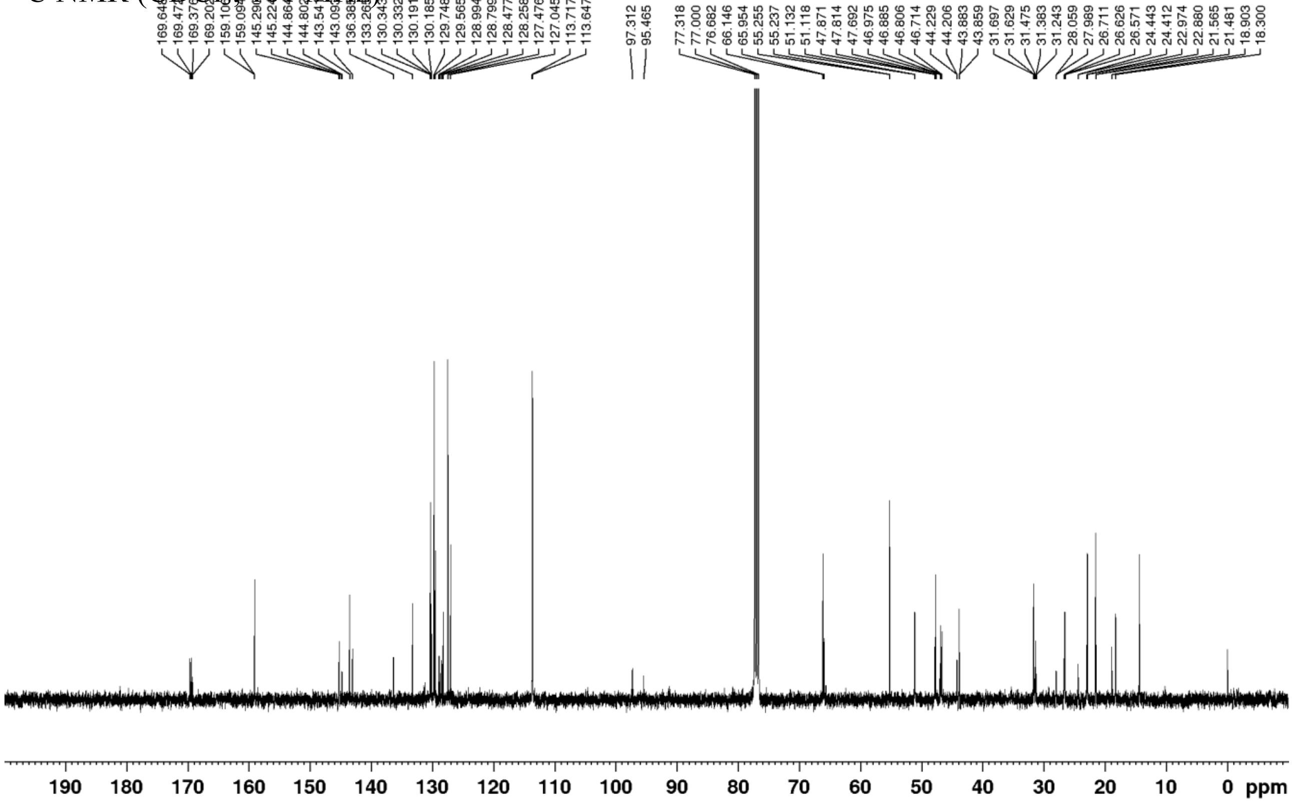

^{19}F NMR ($CDCl_3$, 377 MHz)

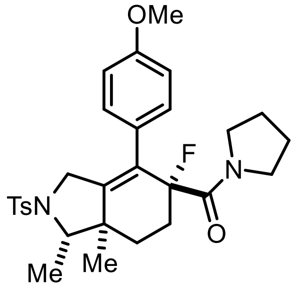

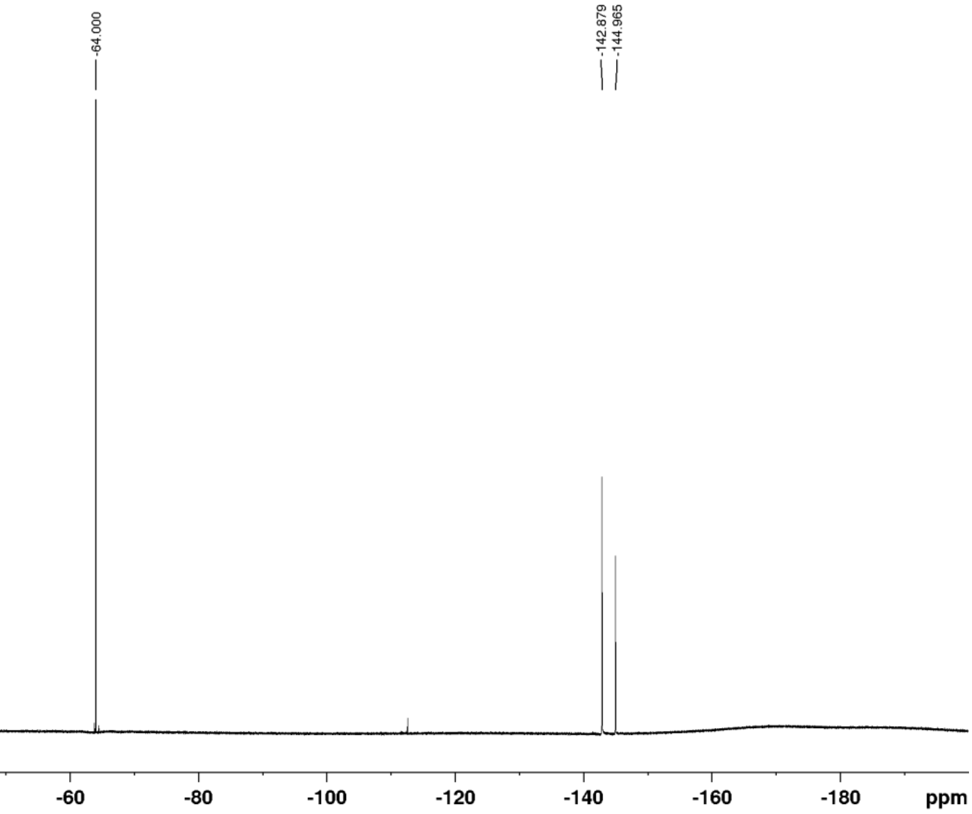

(*E*)-2-Fluoro-4-{(2*R*,3*R*)-4-(*Z*)-4-methoxybenzylidene-2,3-dimethyl-1-(4-methylphenyl) pyrrolidin-3-yl}-1-(pyrrolidin-1-yl)but-2-en-1-one (4ea)

Diastereomer mixture (58:42 d.r.)

^{1}H NMR (CDCl$_3$, 400 MHz)

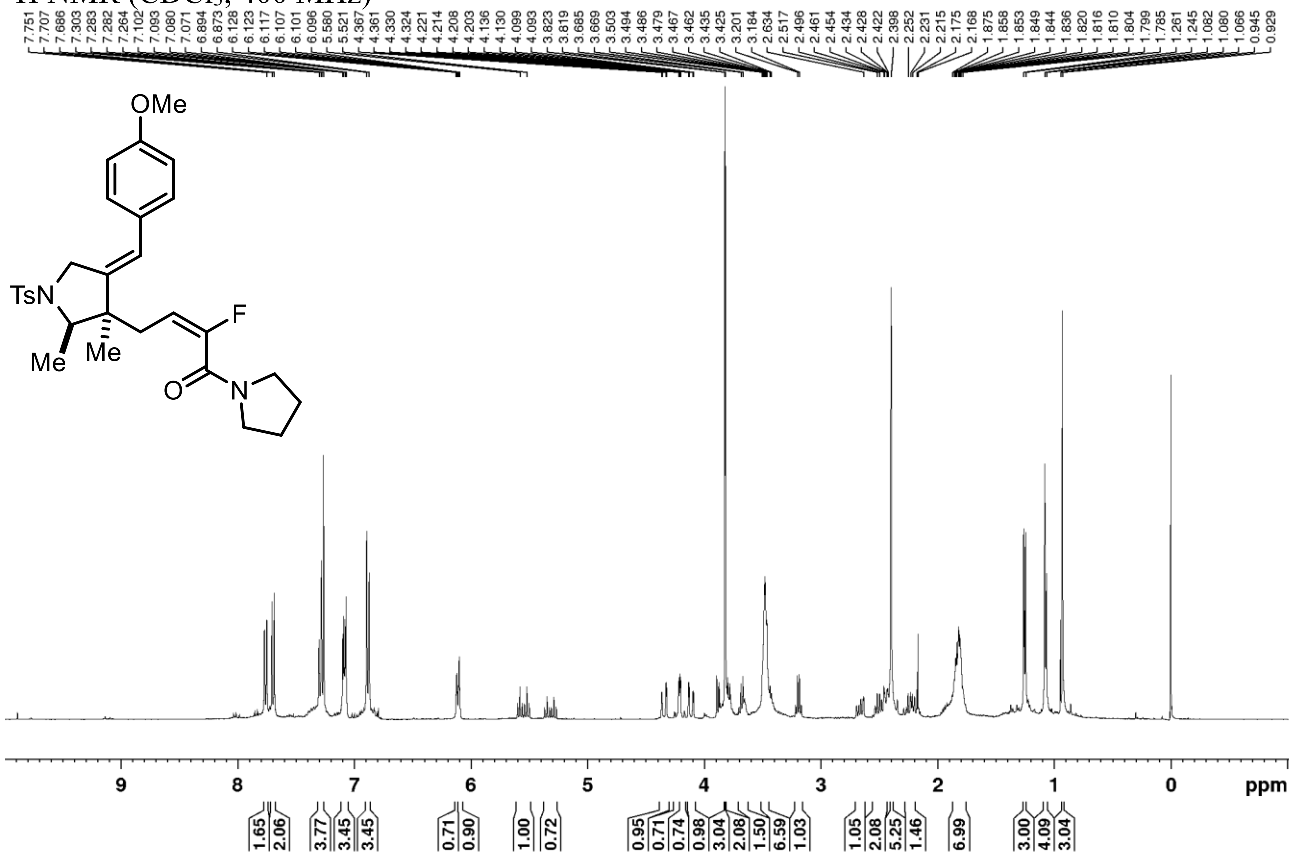

^{13}C NMR (CDCl$_3$, 101 MHz)

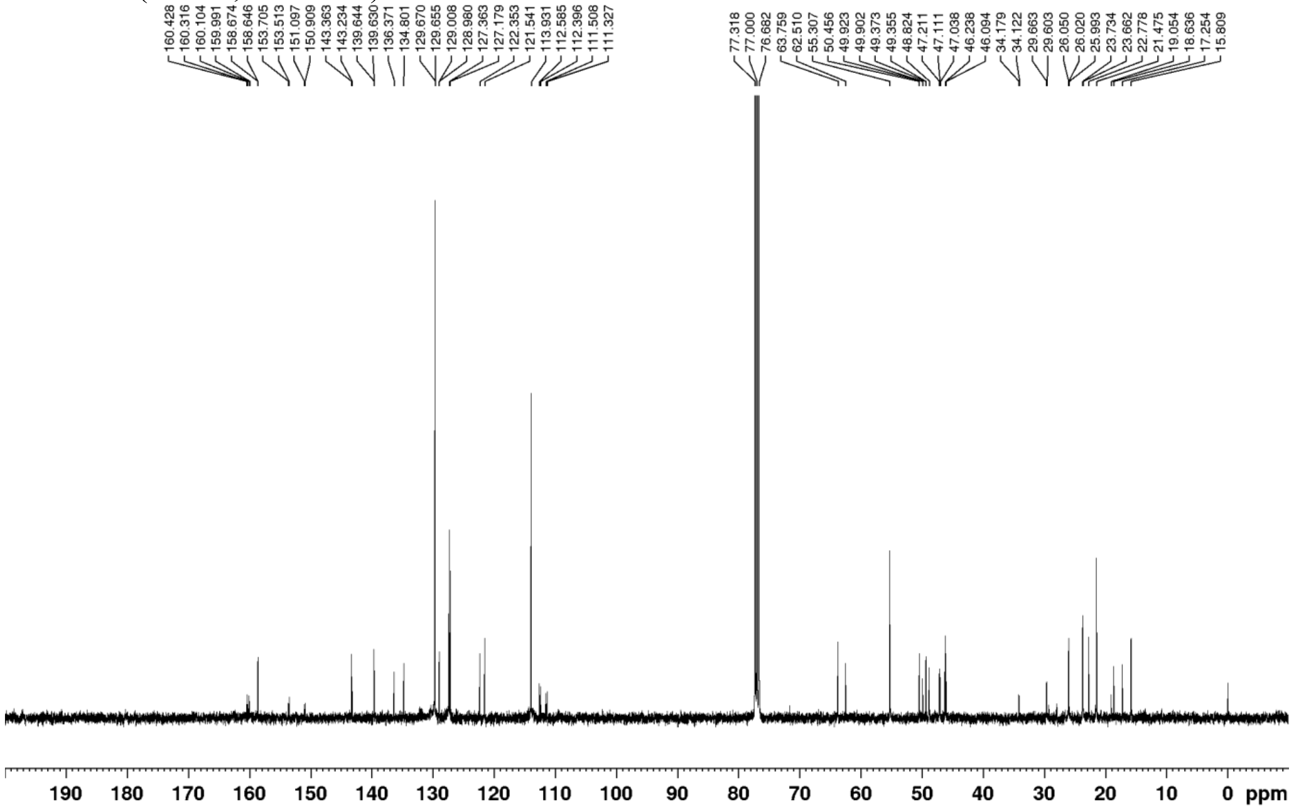

^{19}F NMR ($CDCl_3$, 377 MHz)

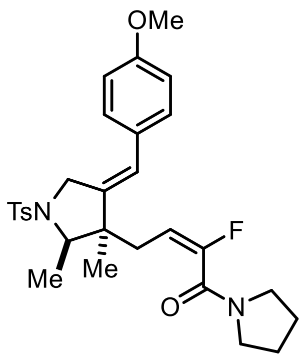

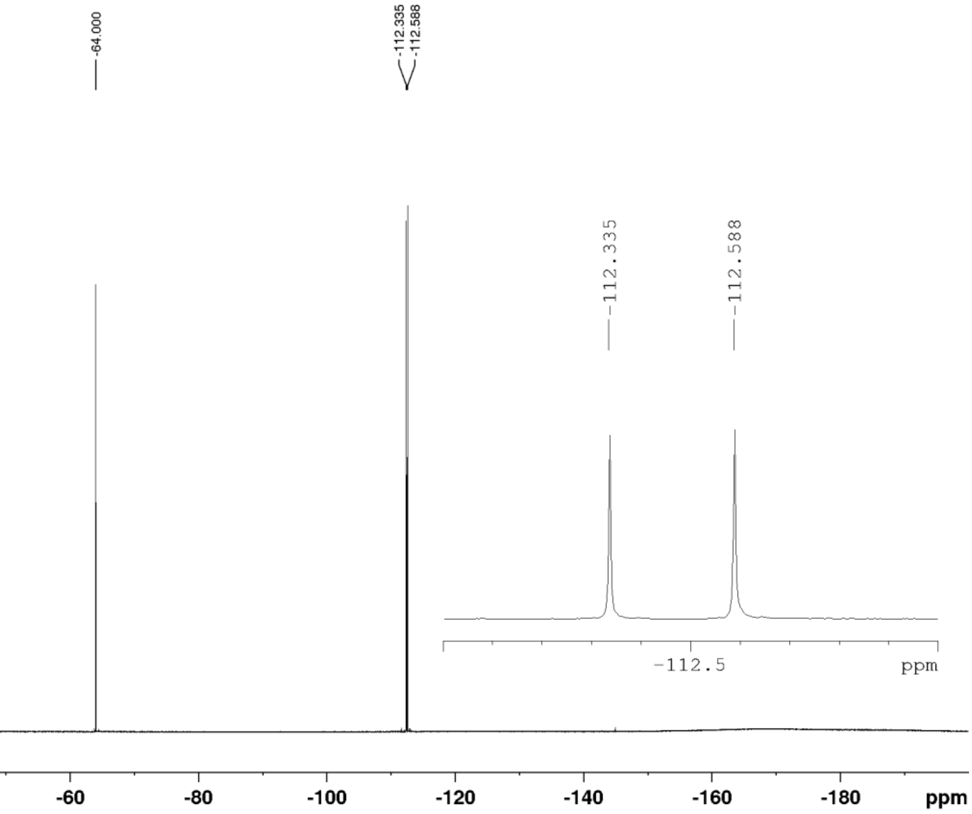

{(3*R*,5*R*,7a*R*)-4-(4-Bromophenyl)-5-fluoro-3-methyl-2-(4-methylphenyl)-2,3,5,6,7,7a-hexahydro-1*H*-isoindol-5-yl}(pyrrolidin-1-yl)methanone [3fa (major diastereomer)]

^{1}H NMR ($CDCl_3$, 400 MHz)

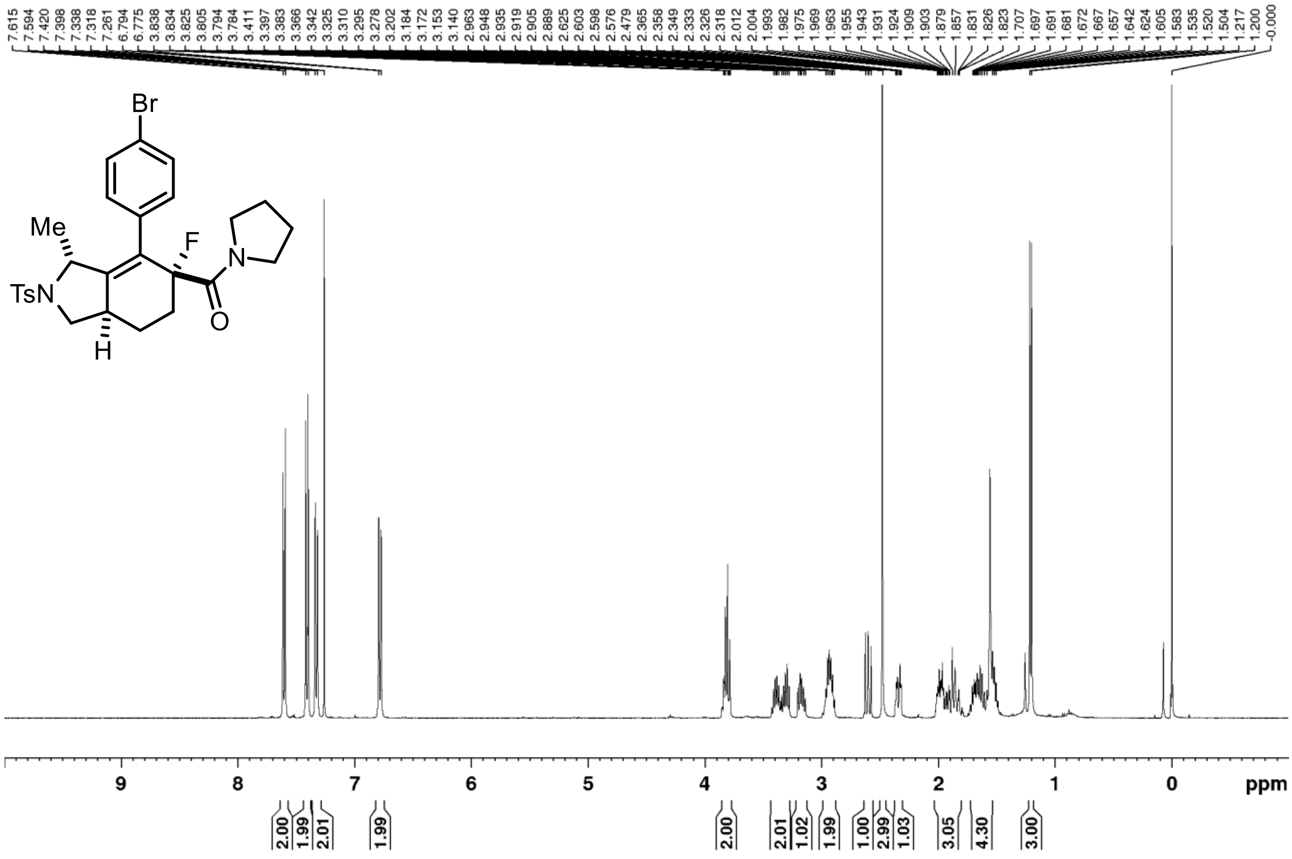

^{13}C NMR ($CDCl_3$, 101 MHz)

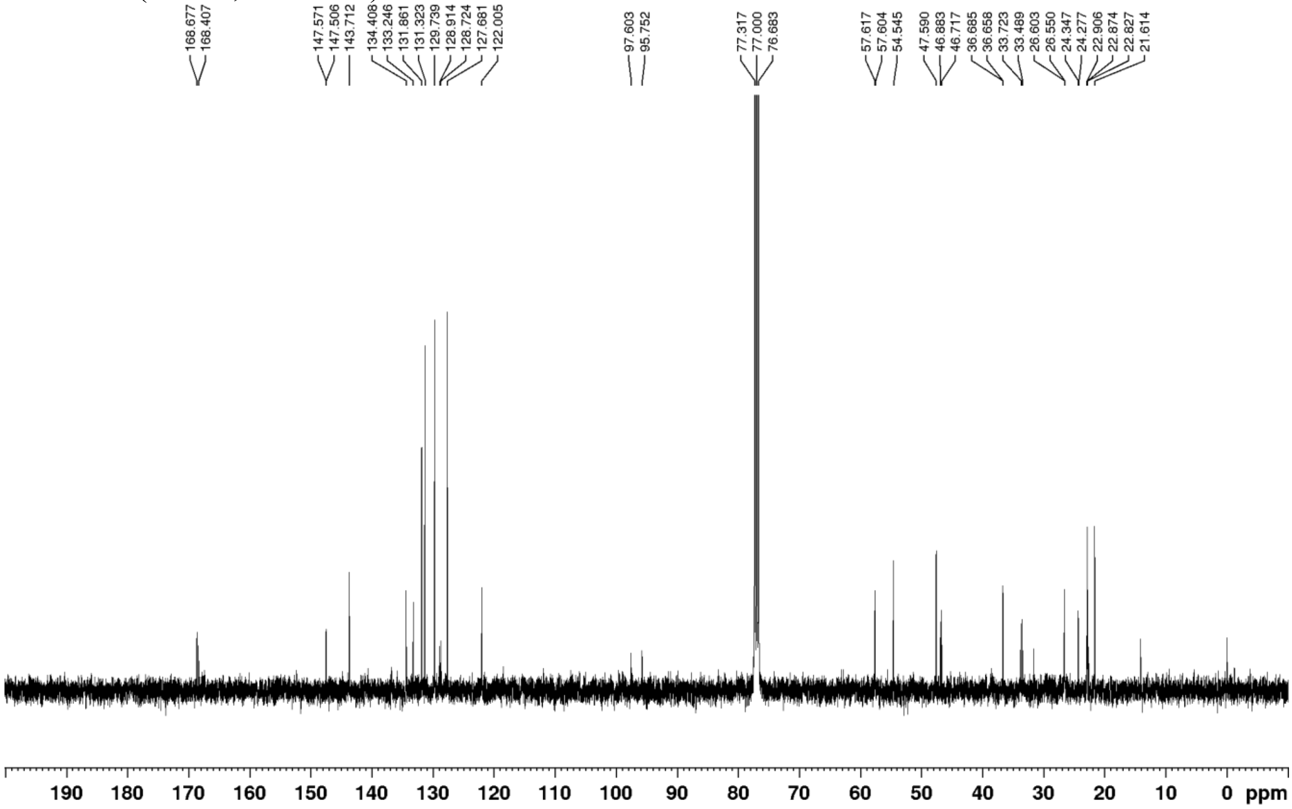

^{19}F NMR ($CDCl_3$, 377 MHz)

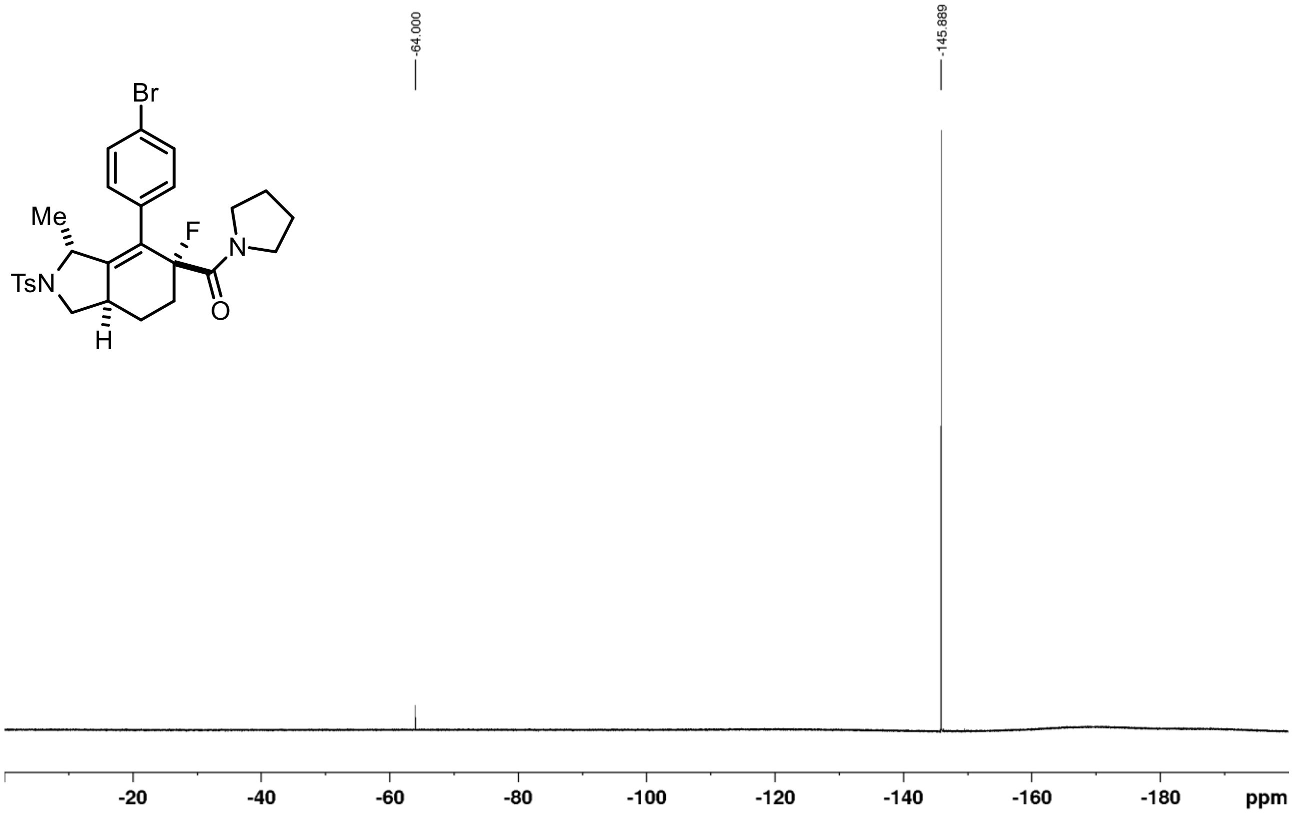

{4-(4-Bromophenyl)-5-fluoro-3-methyl-2-(4-methylphenyl)-2,3,5,6,7,7a-hexahydro-1*H*-isoindol-5-yl}(pyrrolidin-1-yl)methanone [3fa (minor diastereomer)]

^{1}H NMR (CDCl$_3$, 400 MHz)

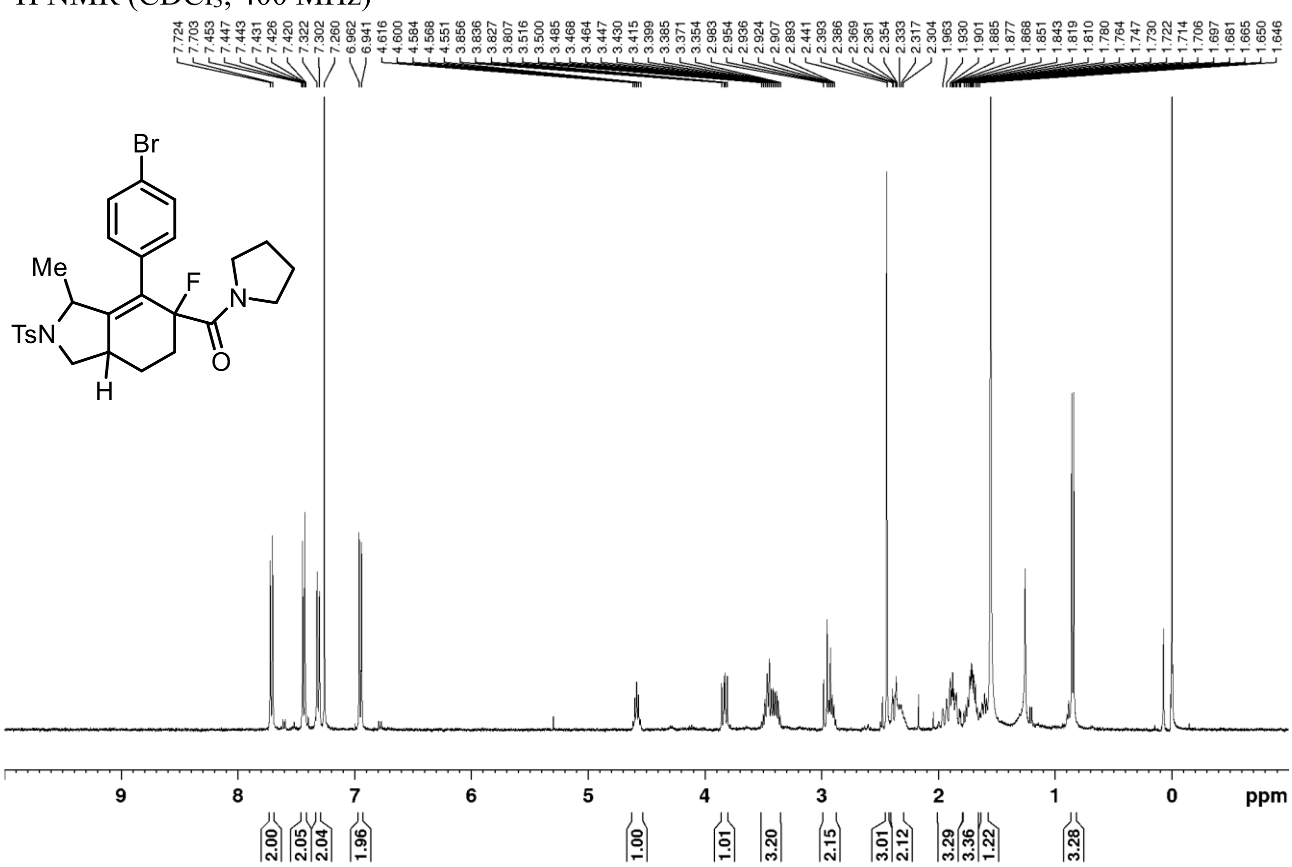

^{19}F NMR (CDCl$_3$, 377 MHz)

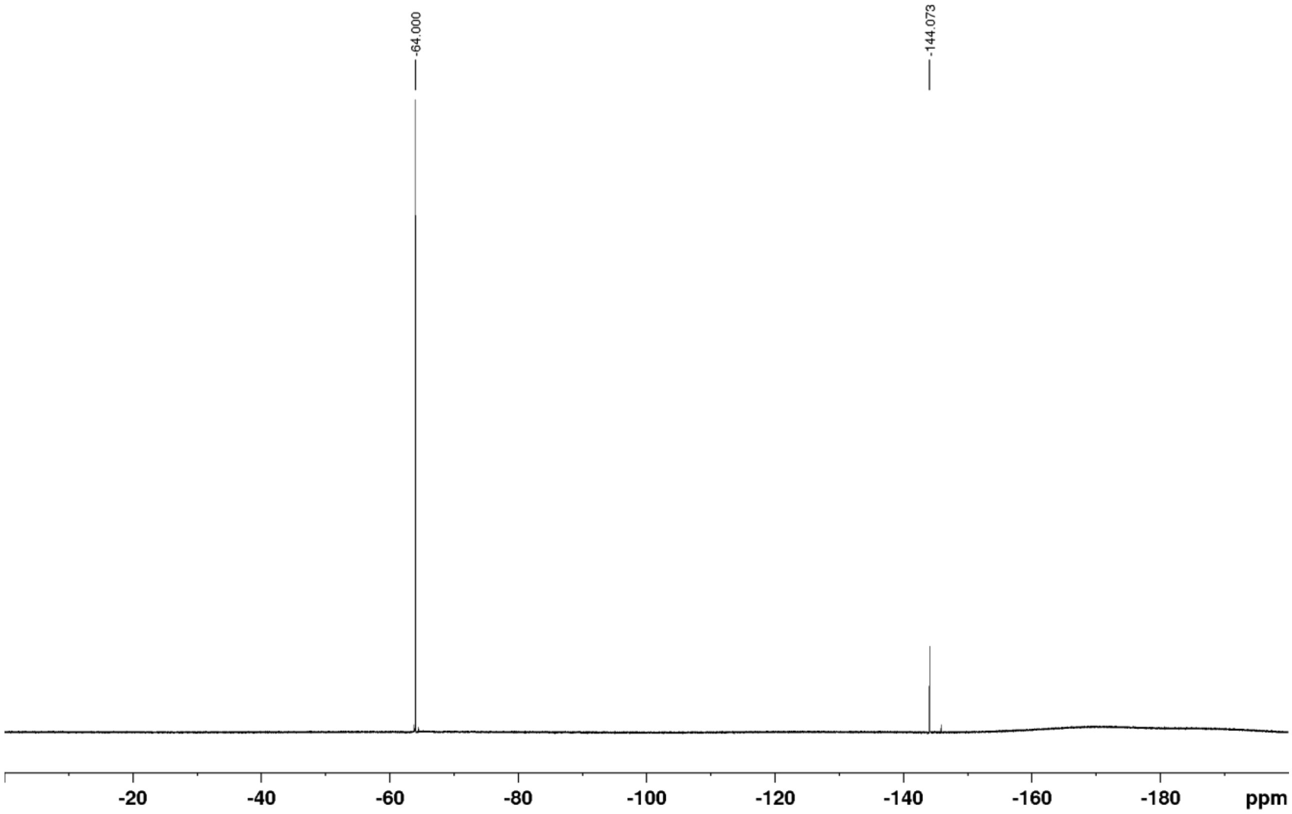

(*E*)-4-[(3*R*,5*S*)-4-{(*Z*)-4-Bromobenzylidene}-3,5-dimethyl-1-(4-methylphenyl)pyrrolidin-3-yl]-2-fluoro-1-(pyrrolidin-1-yl)but-2-en-1-one [4fa (major diastereomer)]

^{1}H NMR ($CDCl_3$, 400 MHz)

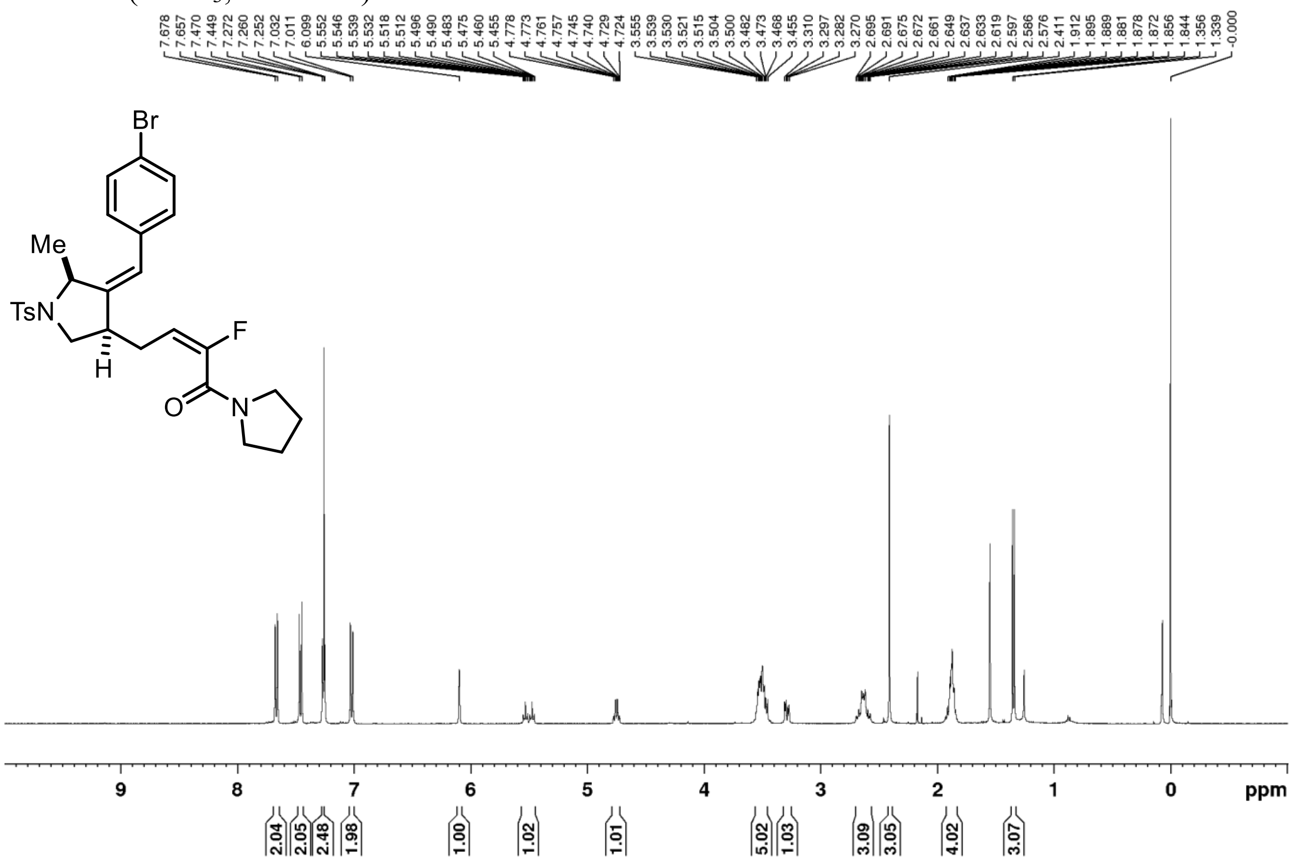

^{13}C NMR ($CDCl_3$, 101 MHz)

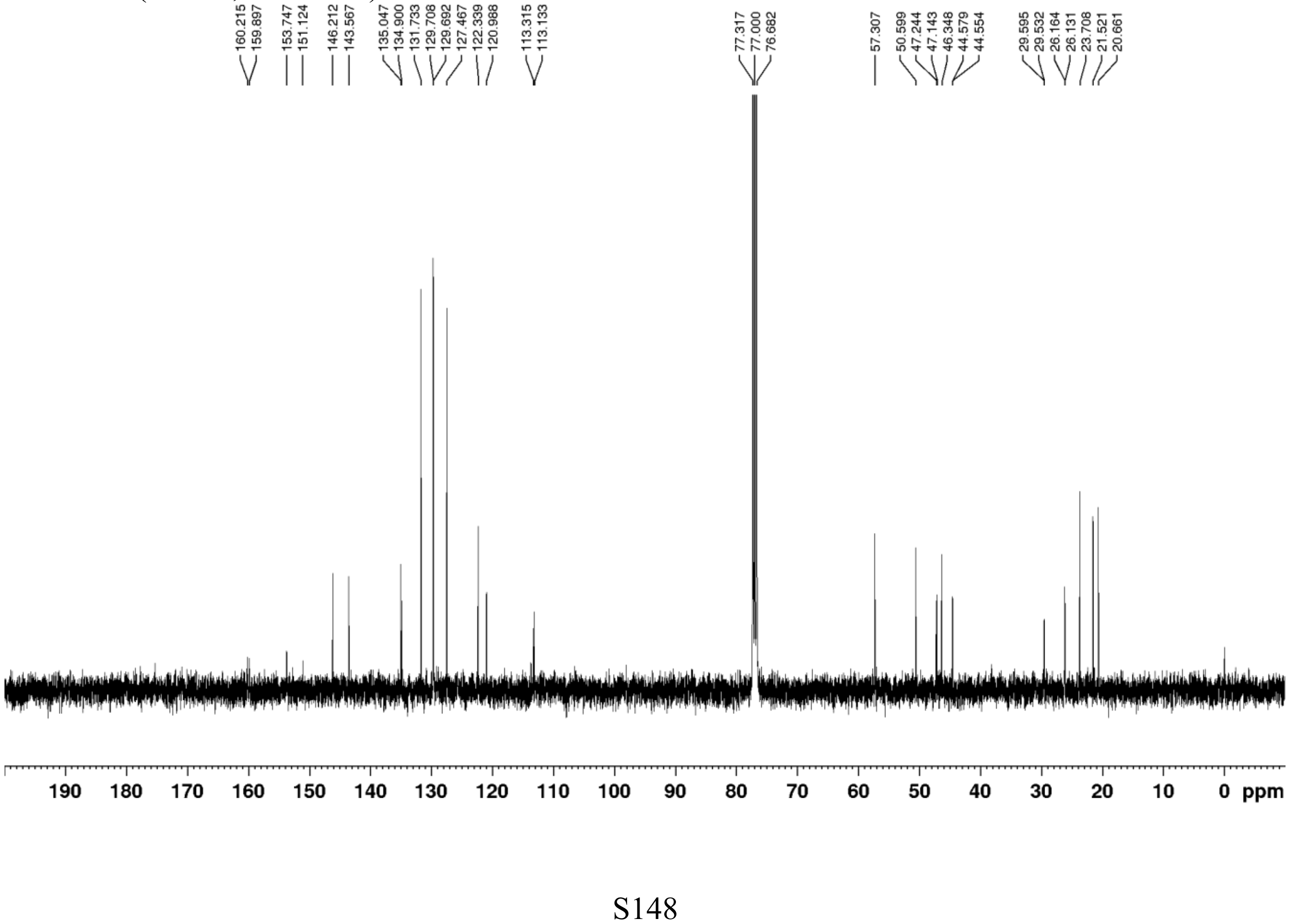

^{19}F NMR ($CDCl_3$, 377 MHz)

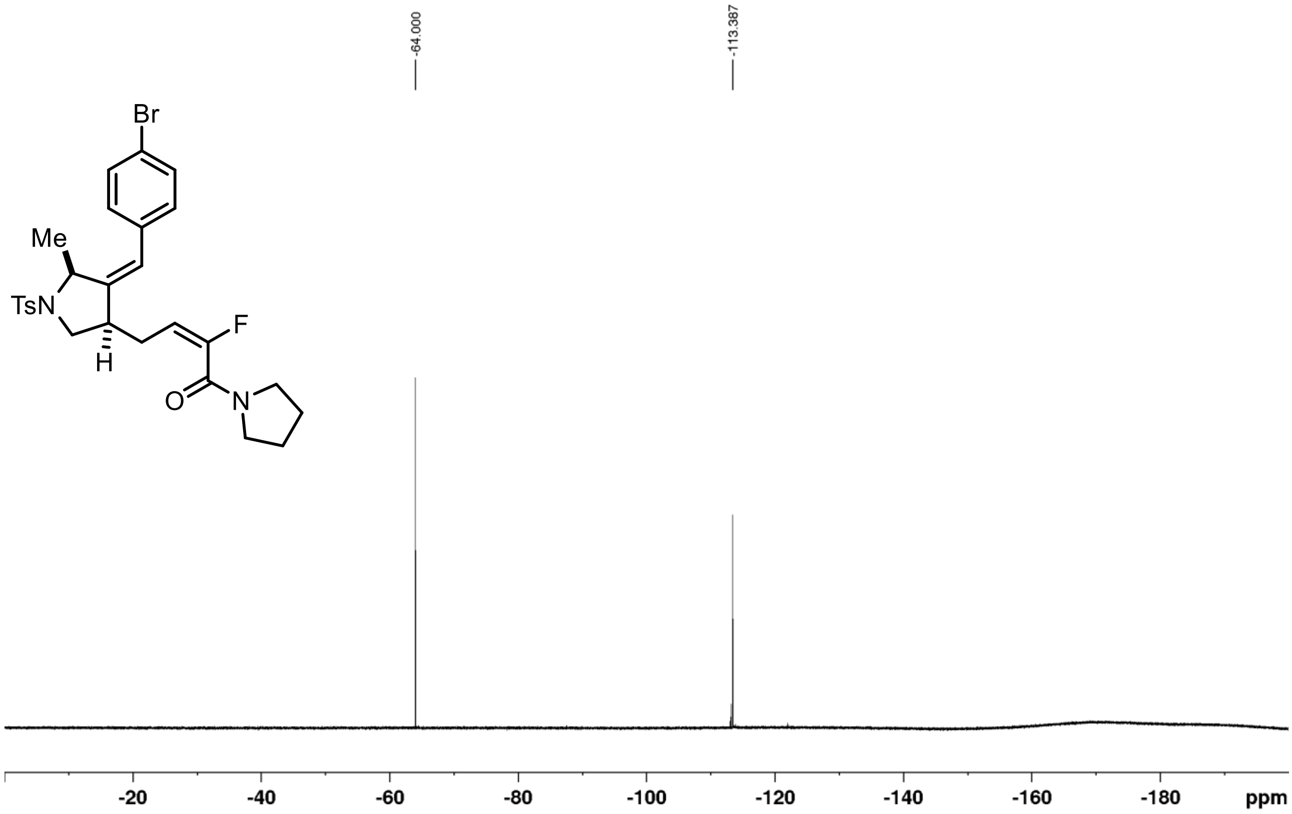

(*E*)-4-[4-{(*Z*)-4-Bromobenzylidene}-3,5-dimethyl-1-(4-methylphenyl)pyrrolidin-3-yl]-2-fluoro-1-(pyrrolidin-1-yl)but-2-en-1-one [4fa (minor diastereomer)]

^{1}H NMR (CDCl$_3$, 400 MHz)

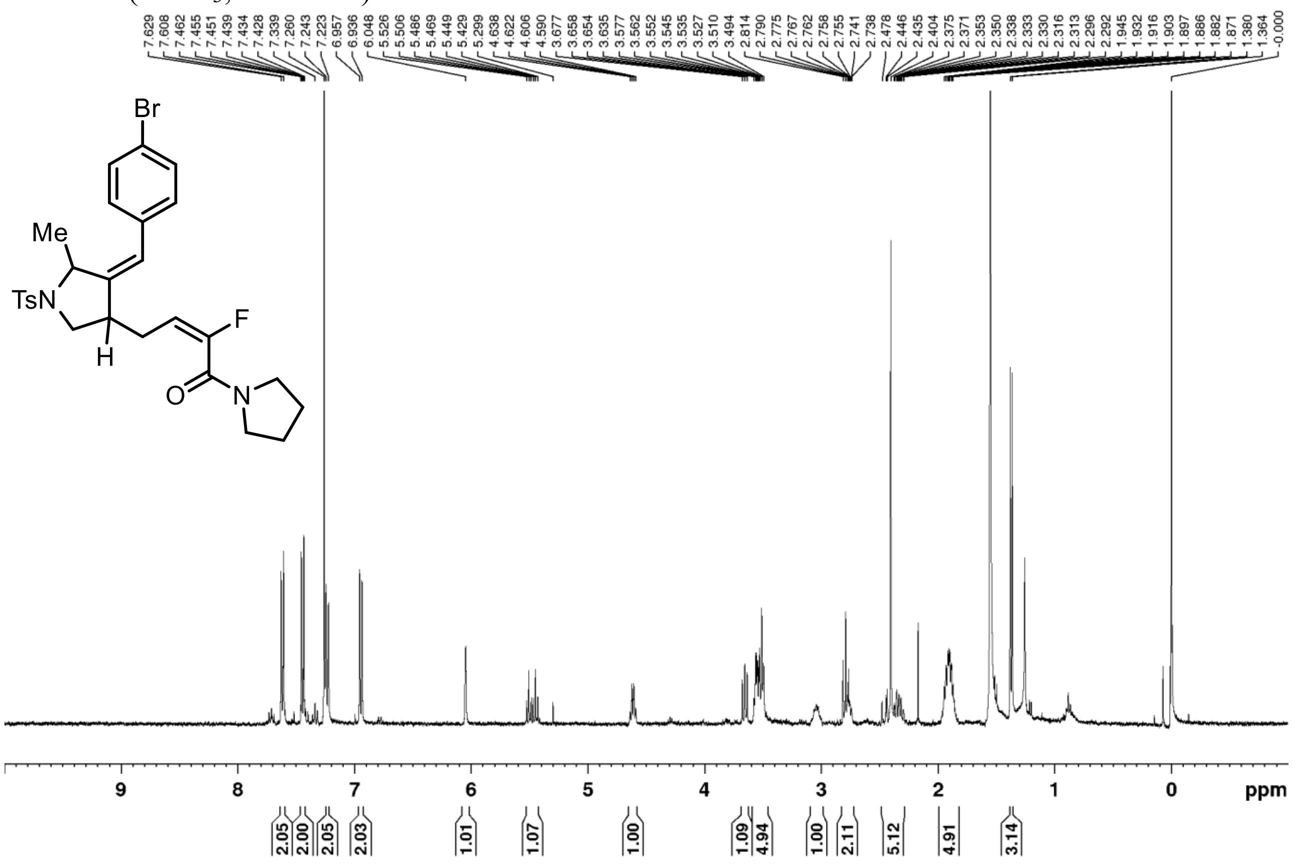

^{19}F NMR (CDCl$_3$, 377 MHz)

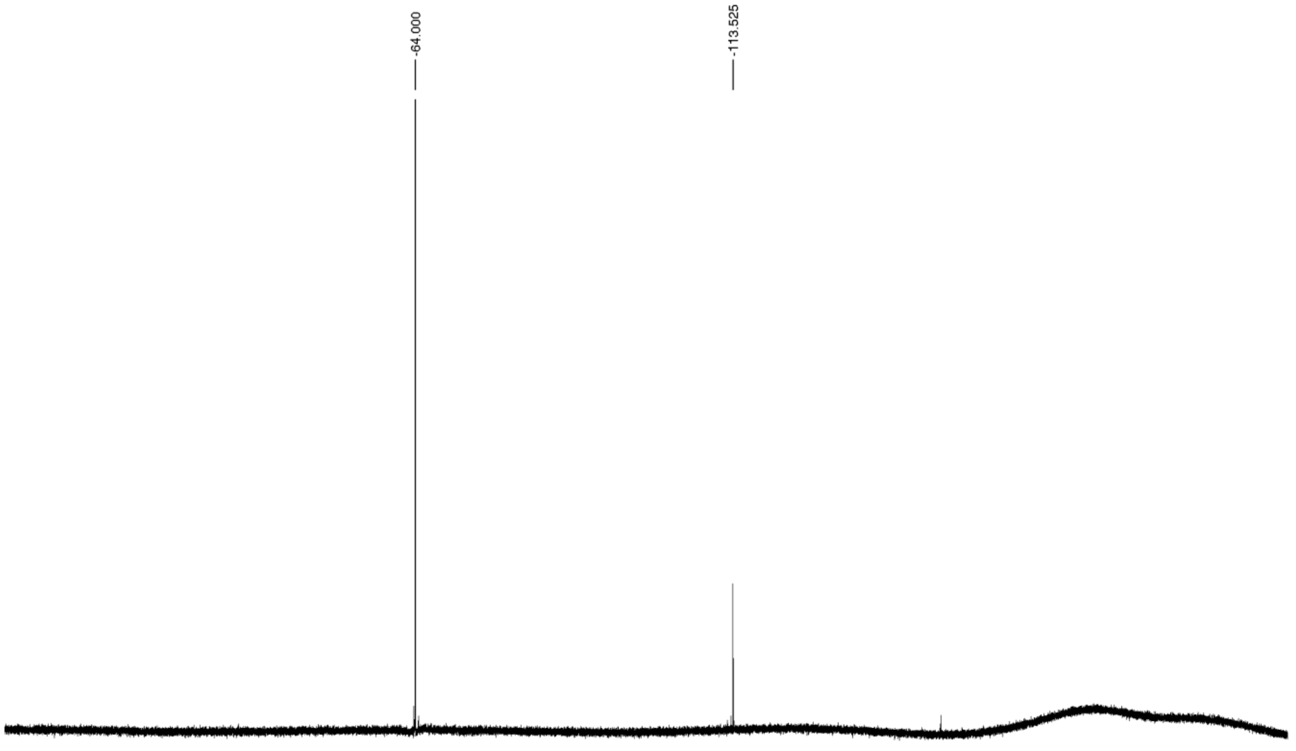

{(3*R*,5*R*,7a*R*)-5-Fluoro-3,4-diphenyl-2-(4-methylphenyl)-2,3,5,6,7,7a-hexahydro-1*H*-isoindol-5-yl}(pyrrolidin-1-yl)methanone (3ga)

^{1}H NMR ($CDCl_3$, 400 MHz)

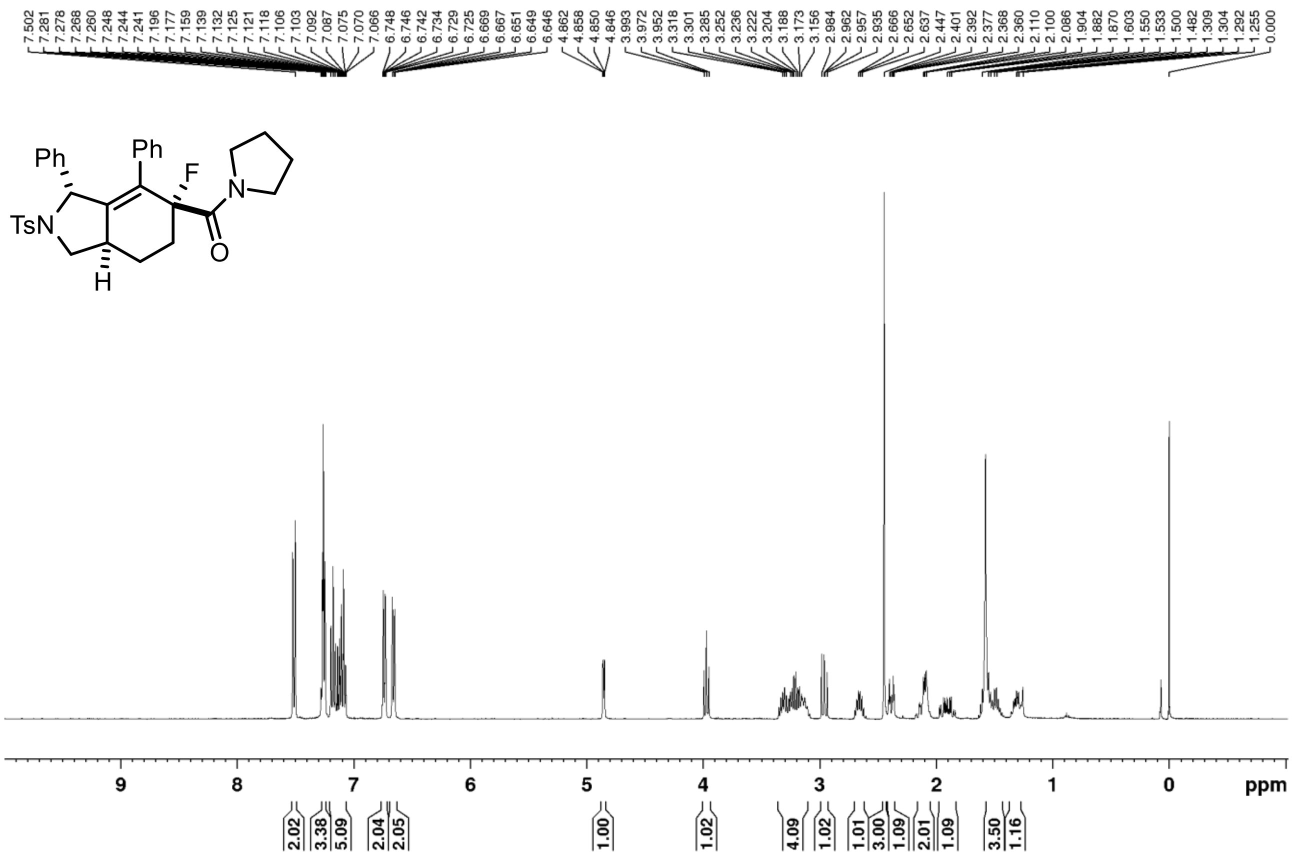

^{13}C NMR ($CDCl_3$, 101 MHz)

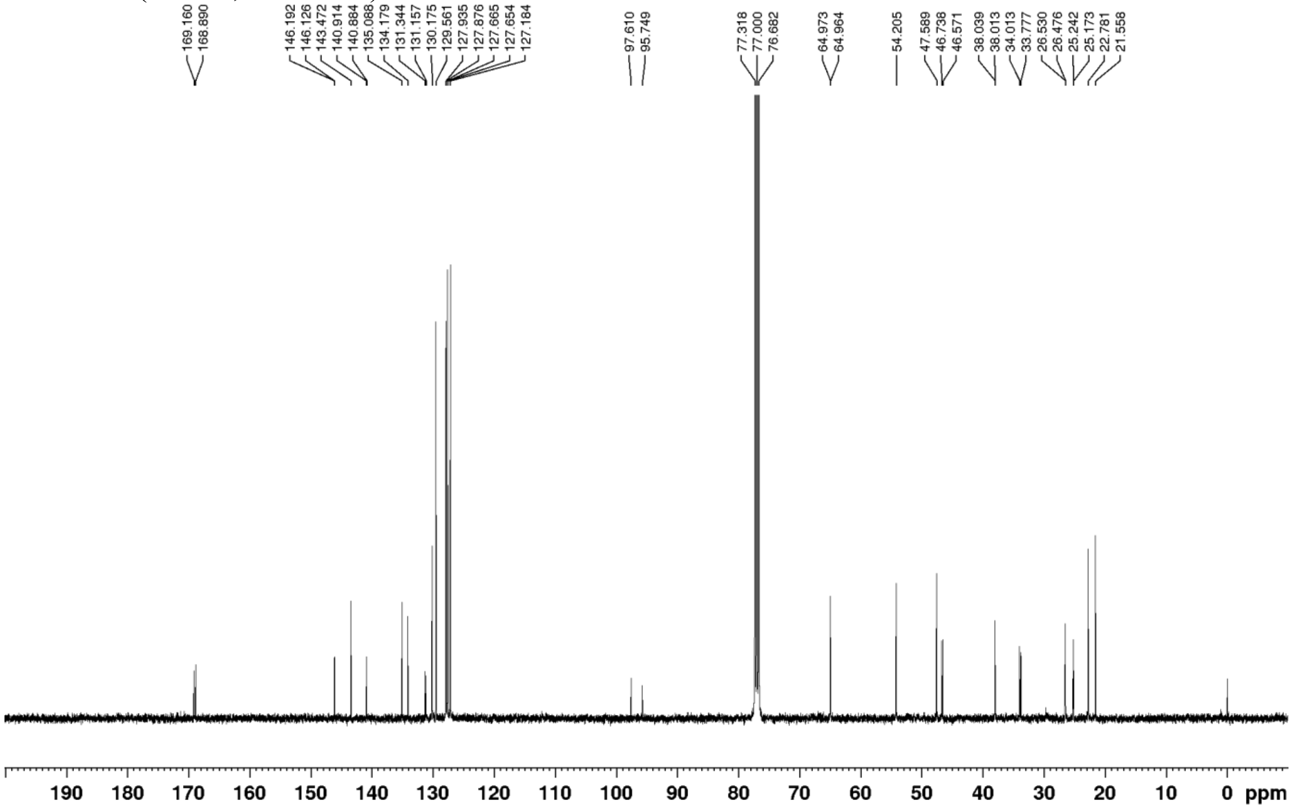

^{19}F NMR ($CDCl_3$, 377 MHz)

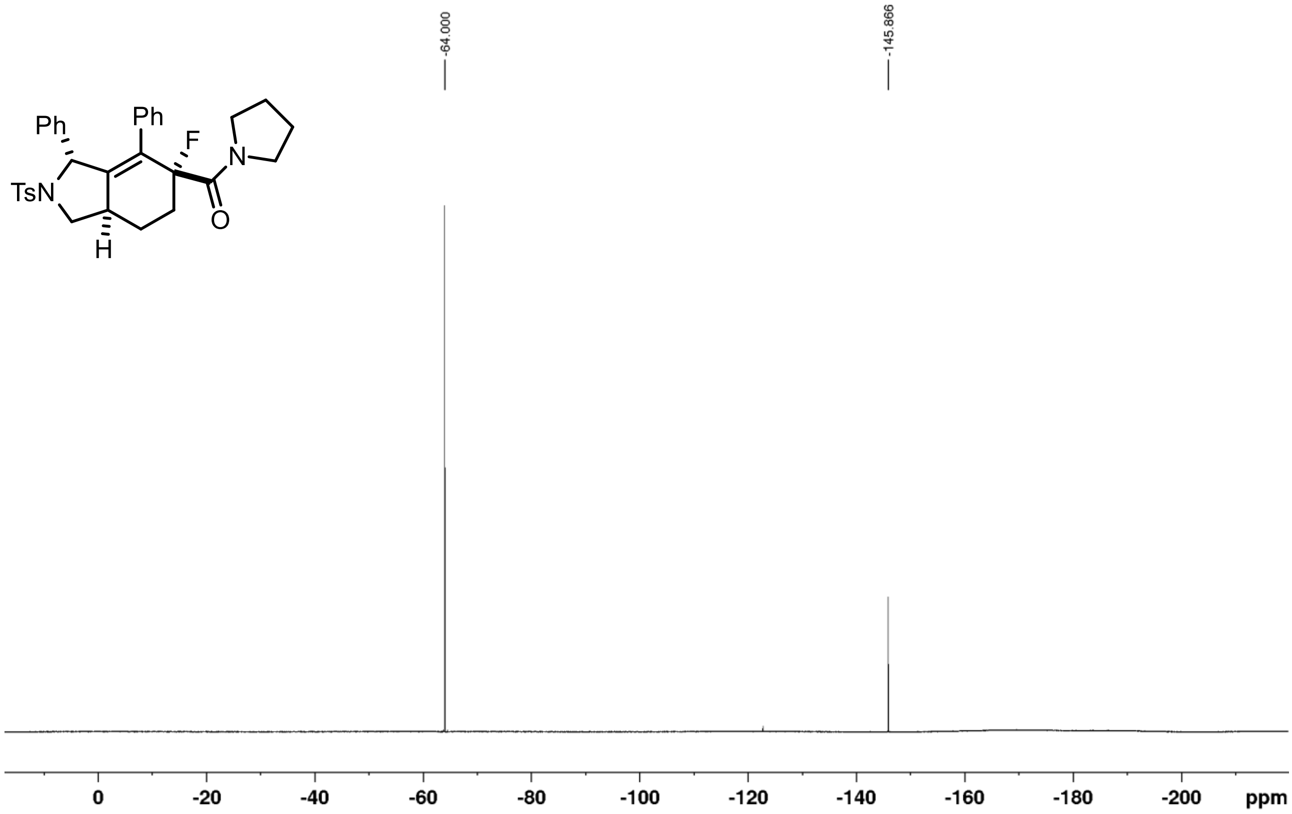

(*E*)-4-{(3*R*,5*S*)-4-(*Z*)-Benzylidene-3-methyl-1-(4-methylphenyl)-5-phenyl-pyrrolidin-3-yl}-2-fluoro-1-(pyrrolidin-1-yl)but-2-en-1-one (4ga)

Diastereomer mixture (92:8 d.r.)

^{1}H NMR ($CDCl_3$, 400 MHz)

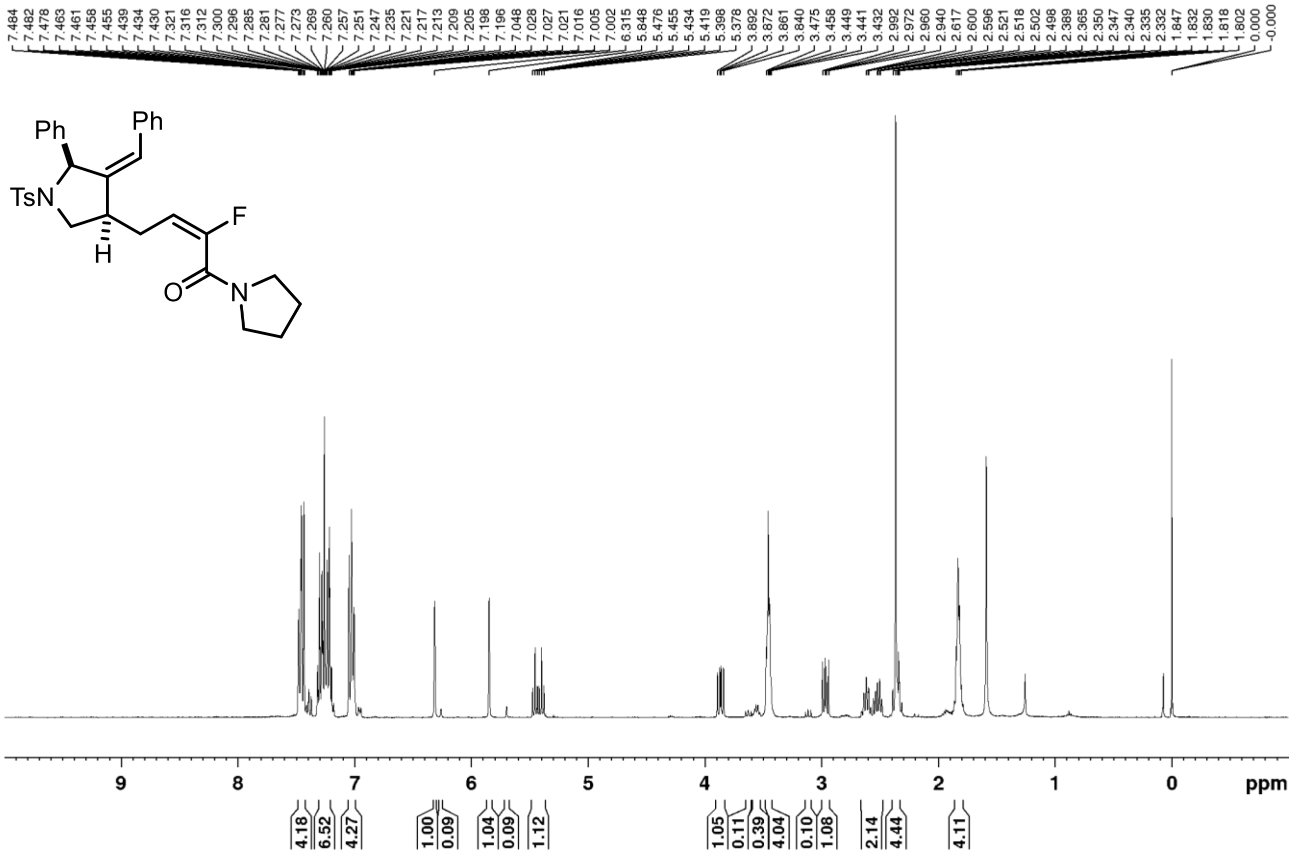

^{13}C NMR ($CDCl_3$, 101 MHz)

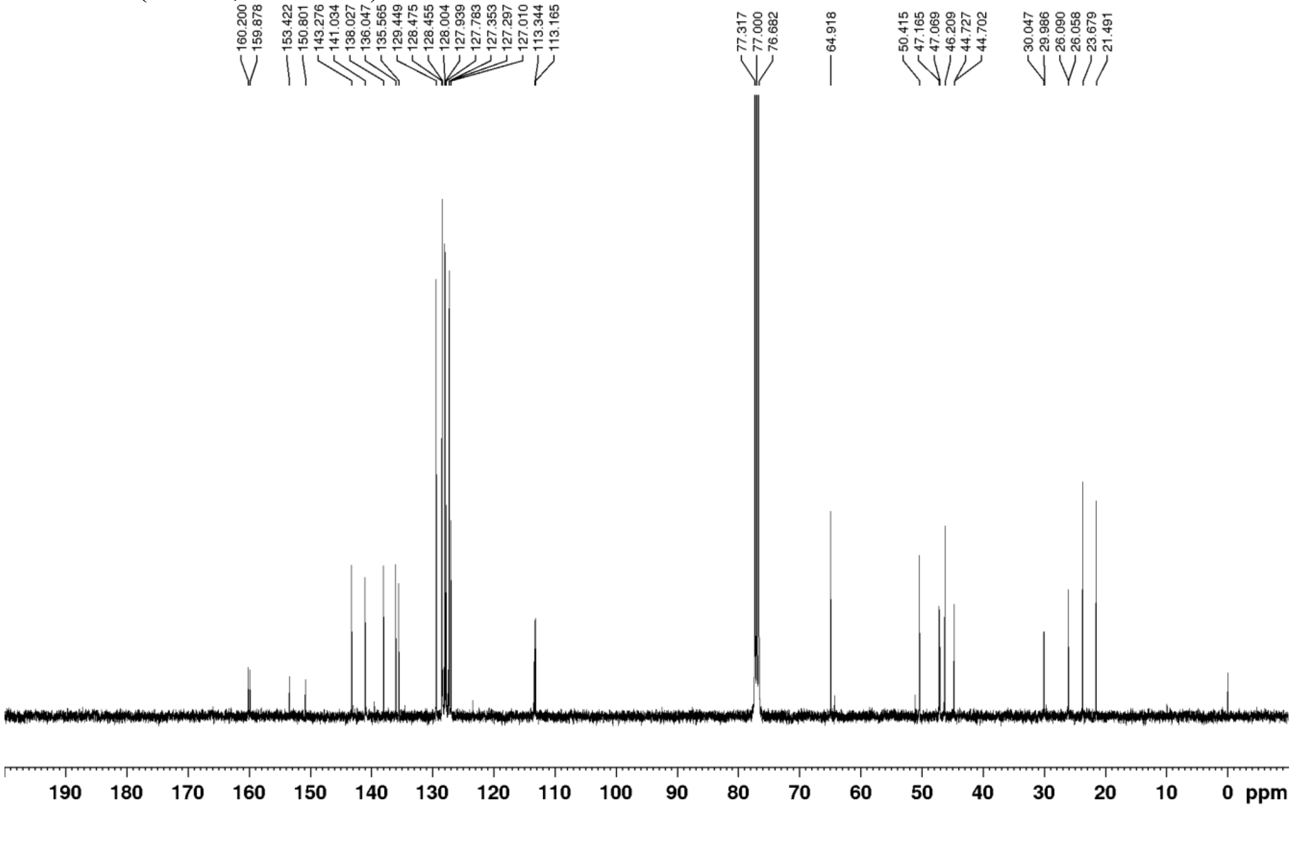

^{19}F NMR ($CDCl_3$, 377 MHz)

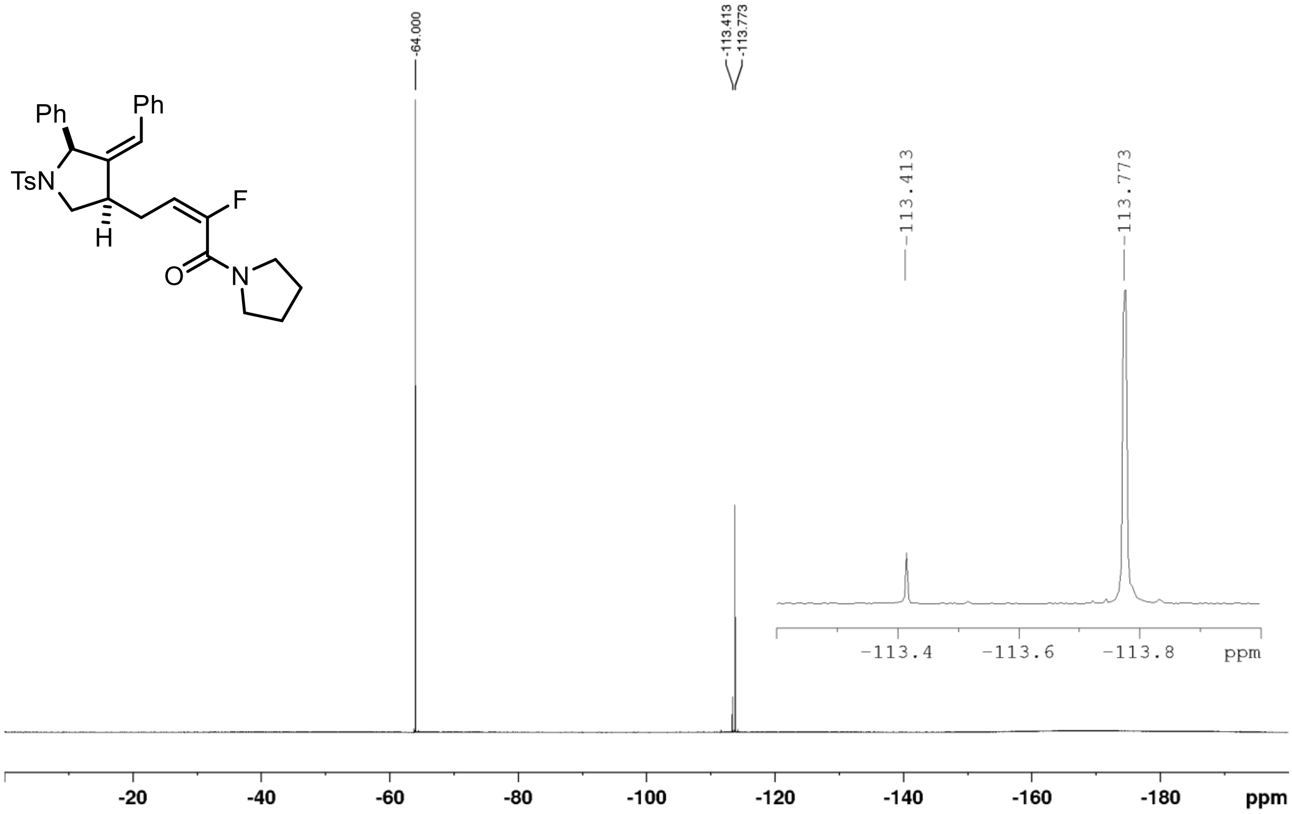

{(1*S*,5*R*,7a*R*)-5-Fluoro-1,7a-dimethyl-2-(4-methylphenyl)-4-phenyl-2,3,5,6,7,7a-hexahydro-1*H*-isoindol-5-yl}(morpholino)methanone [3db (major diastereomer)]

^{1}H NMR ($CDCl_3$, 400 MHz)

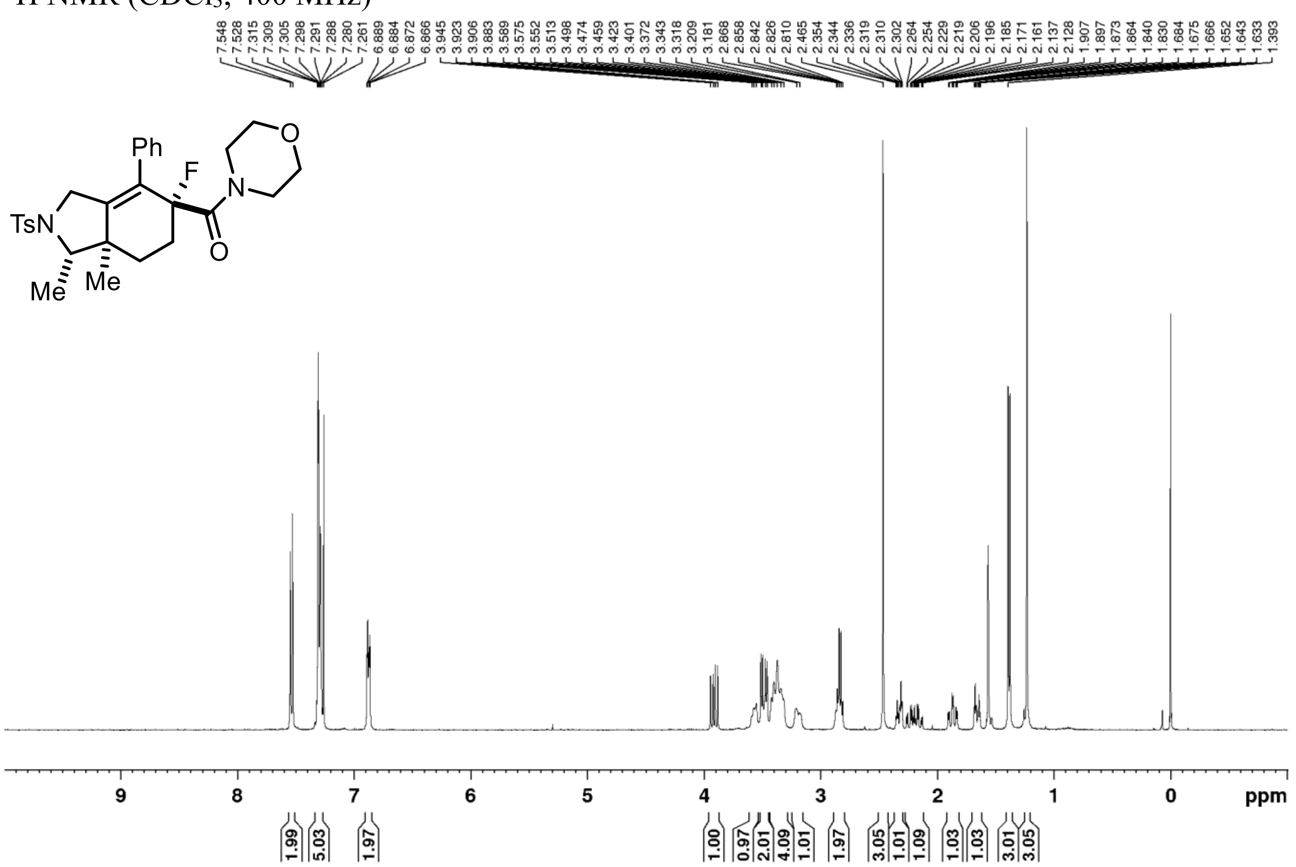

^{13}C NMR ($CDCl_3$, 101 MHz)

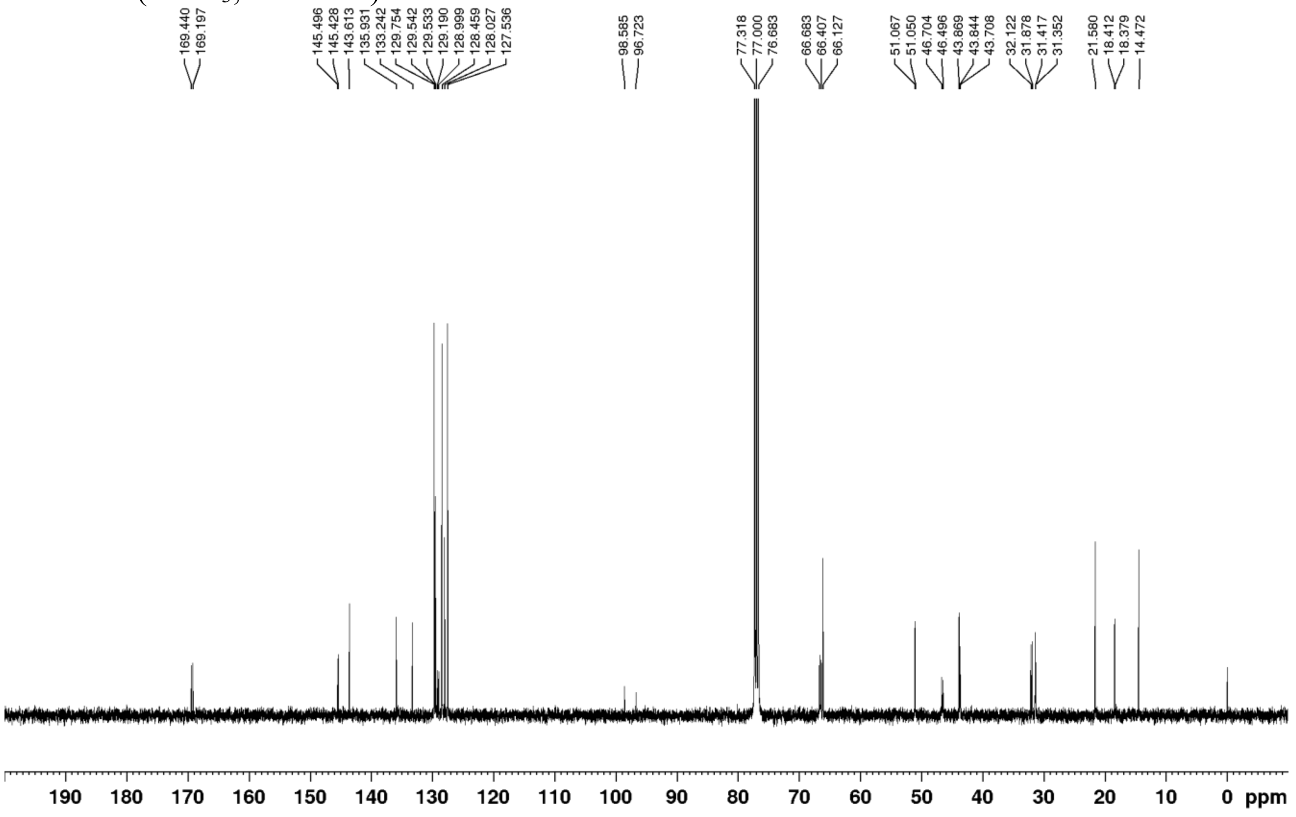

^{19}F NMR ($CDCl_3$, 377 MHz)

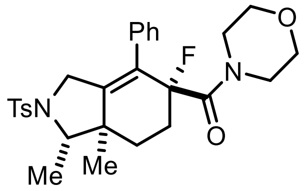

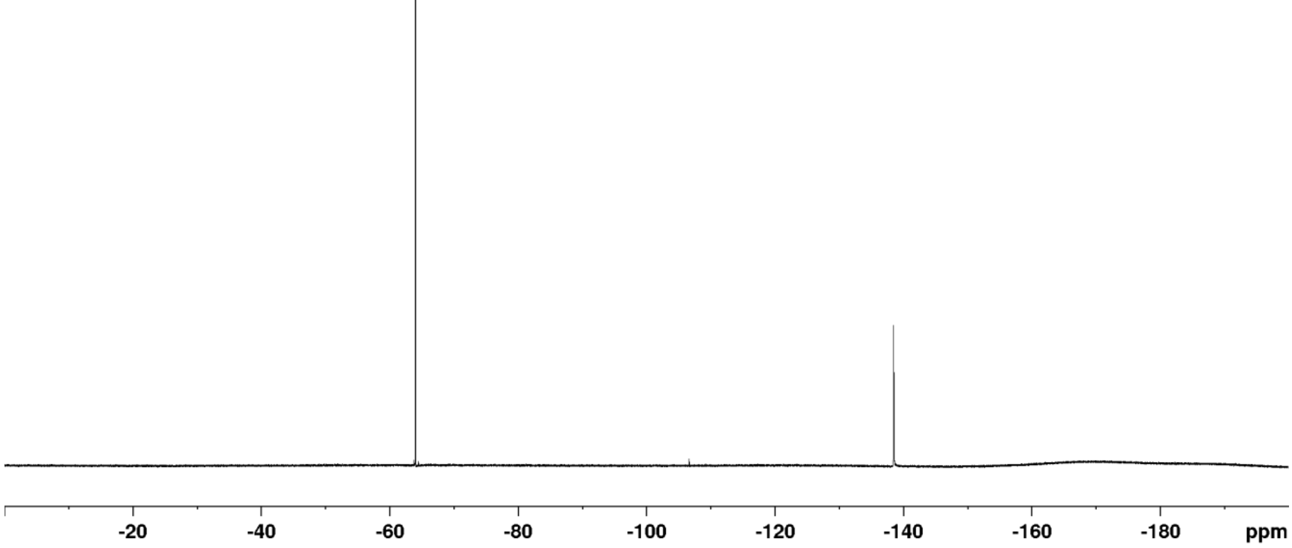

{5-Fluoro-1,7a-dimethyl-2-(4-methylphenyl)-4-phenyl-2,3,5,6,7,7a-hexahydro-1*H*-isoindol-5-yl}(morpholino)methanone [3db (minor diastereomer)]

^{1}H NMR ($CDCl_3$, 400 MHz)

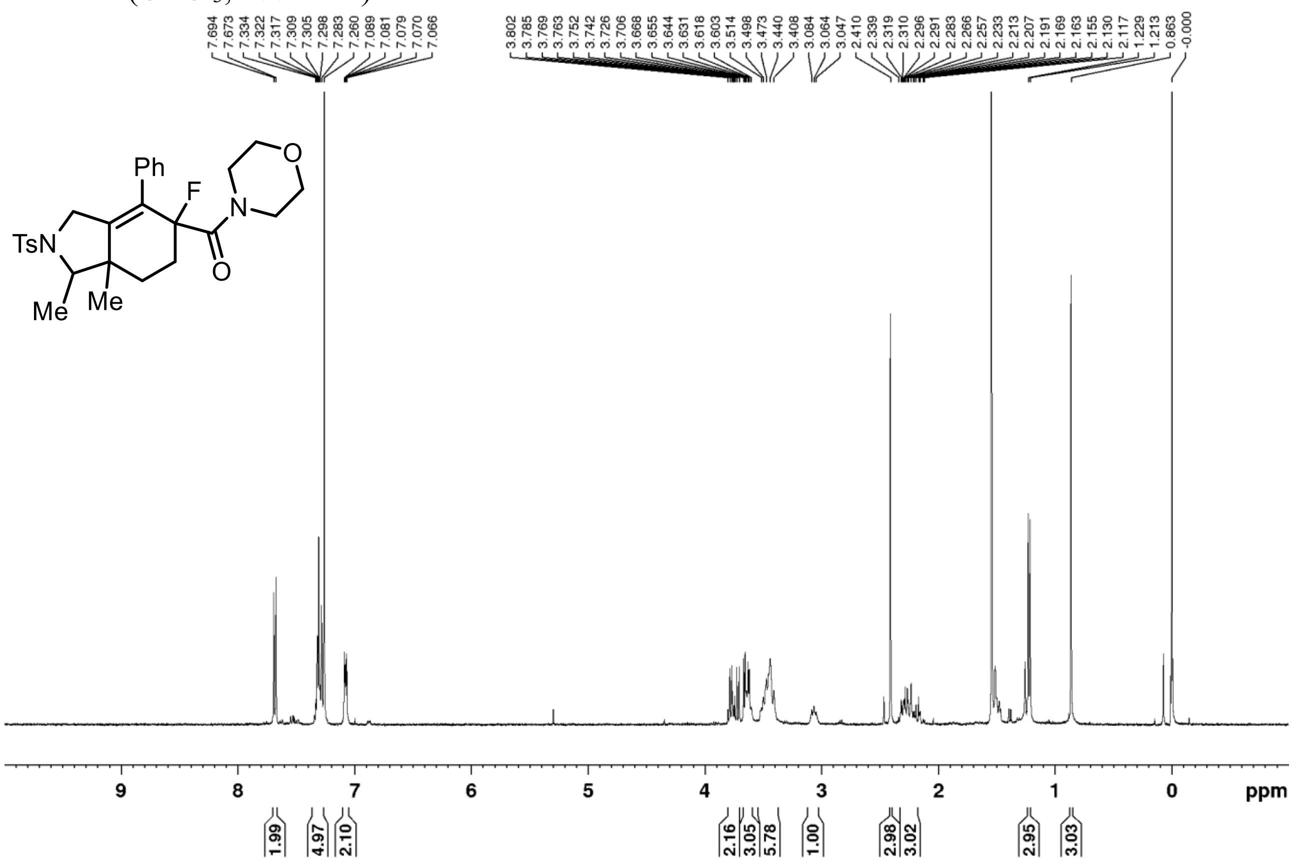

^{19}F NMR ($CDCl_3$, 377 MHz)

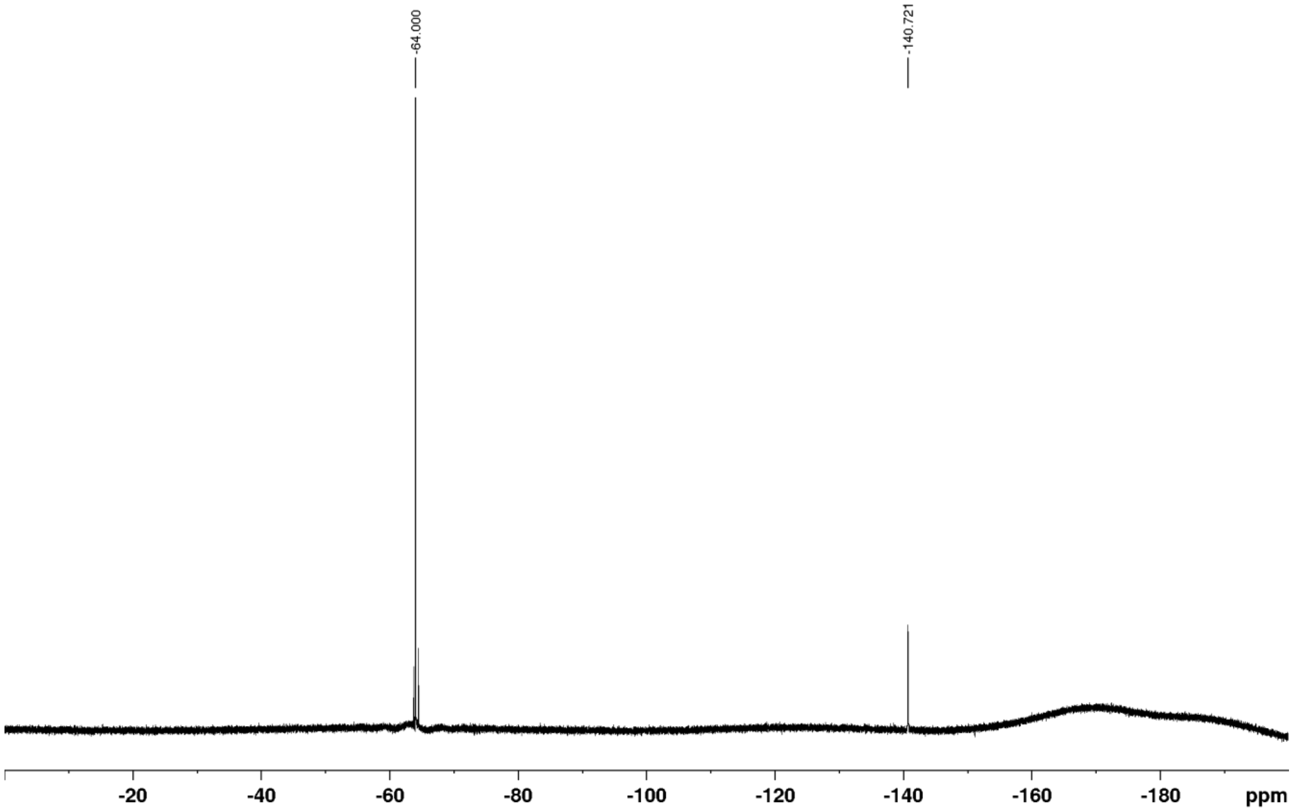

(*E*)-4-{(2*R*,3*R*)-4-(*Z*)-Benzylidene-2,3-dimethyl-1-(4-methylphenyl)-pyrrolidin-3-yl}-2-fluoro-1-morpholinobut-2-en-1-one (4db)

Diastereomer mixture (67:33 d.r.)

^{1}H NMR (CDCl$_3$, 400 MHz)

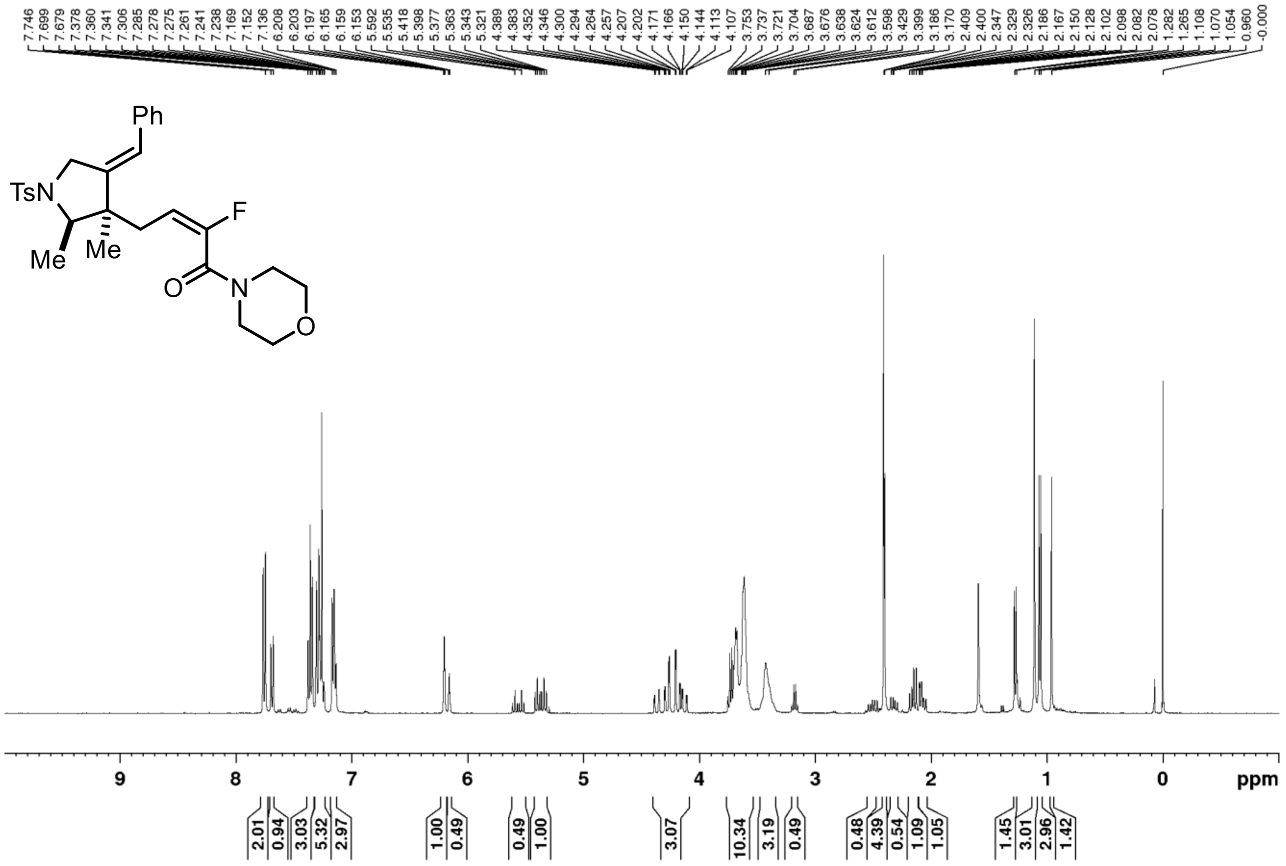

^{13}C NMR (CDCl$_3$, 101 MHz)

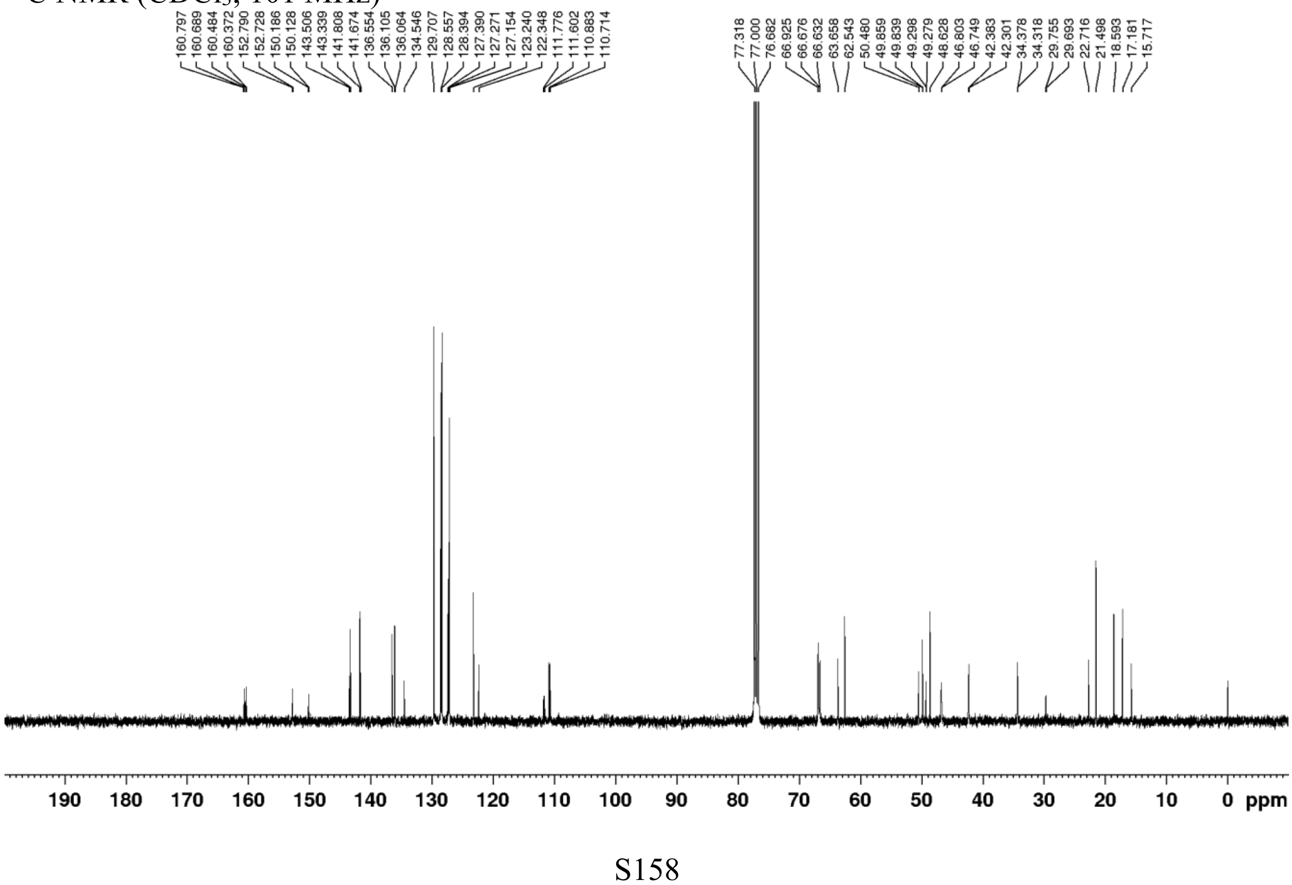

^{19}F NMR ($CDCl_3$, 377 MHz)

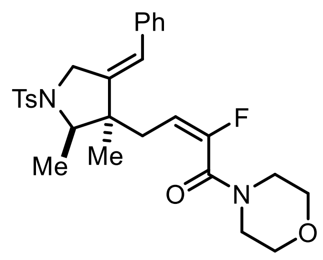

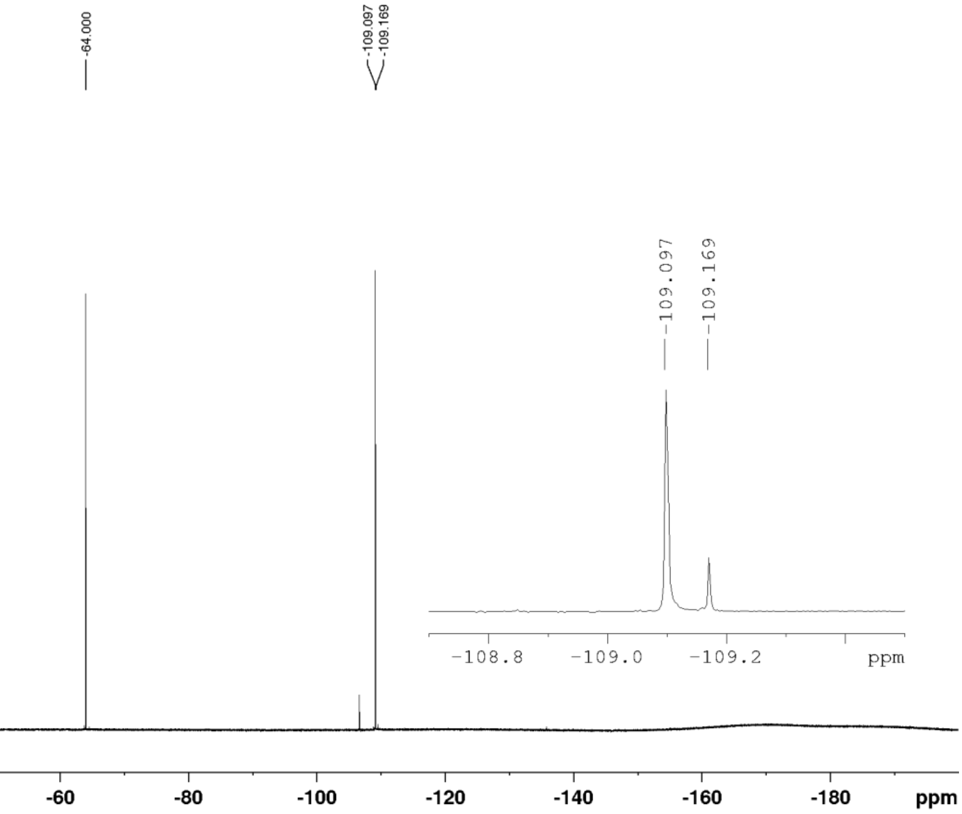

{(1*S*,5*R*,7a*R*)-5-Fluoro-4-(4-methoxyphenyl)-1,7a-dimethyl-2-(4-methylphenyl)-2,3,5,6,7,7a-hexahydro-1*H*-isoindol-5-yl}(morpholino)methanone (3eb)

Diastereomer mixture (82:18 d.r.)

^{1}H NMR ($CDCl_3$, 400 MHz)

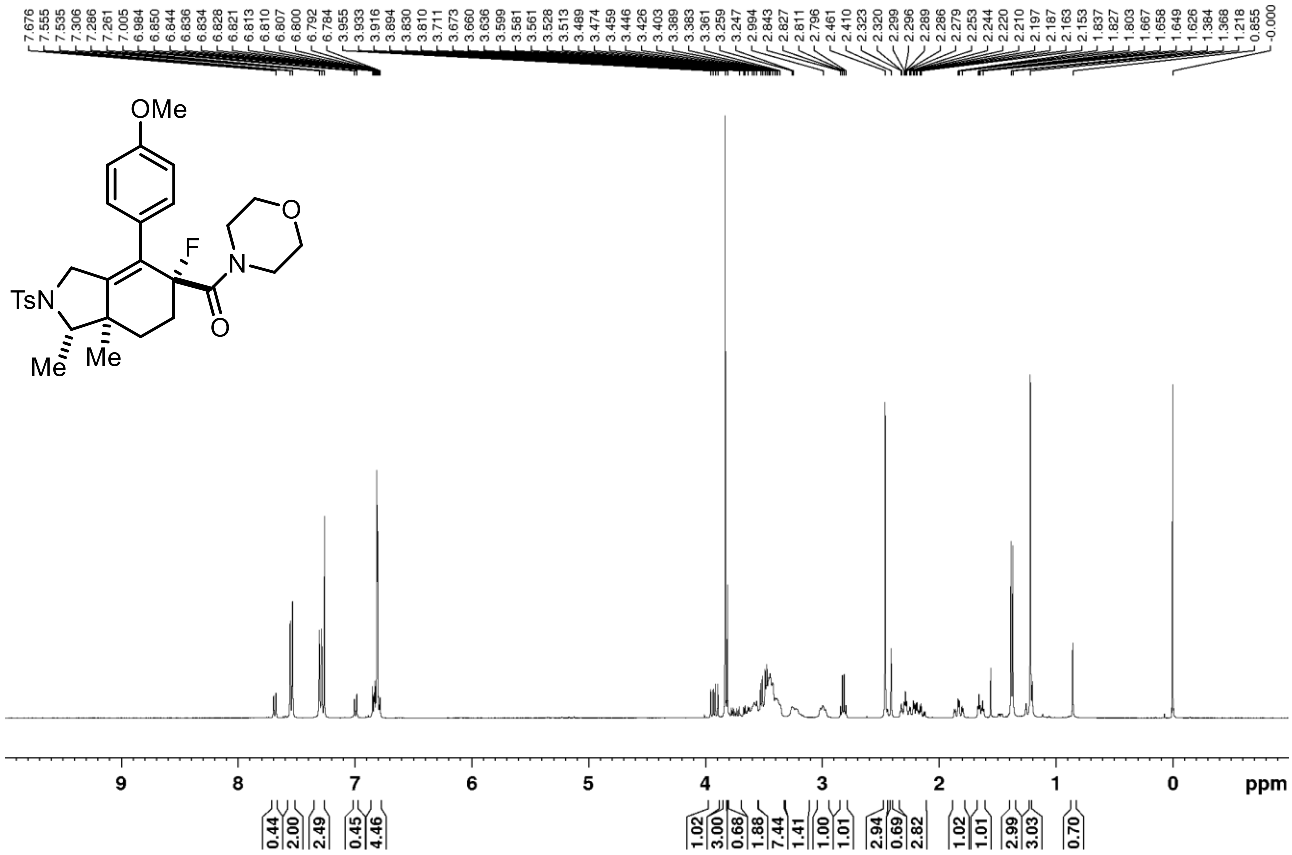

^{13}C NMR ($CDCl_3$, 101 MHz)

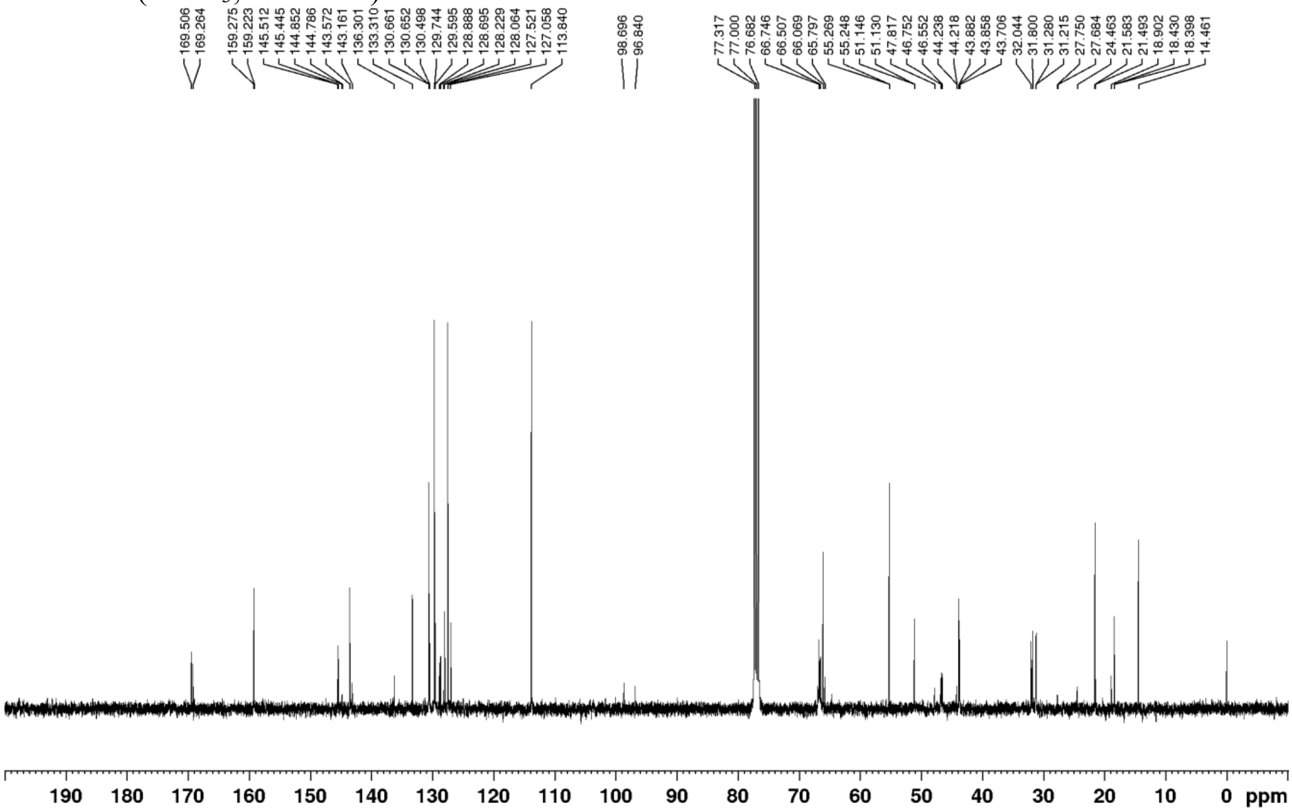

^{19}F NMR (CDCl$_3$, 377 MHz)

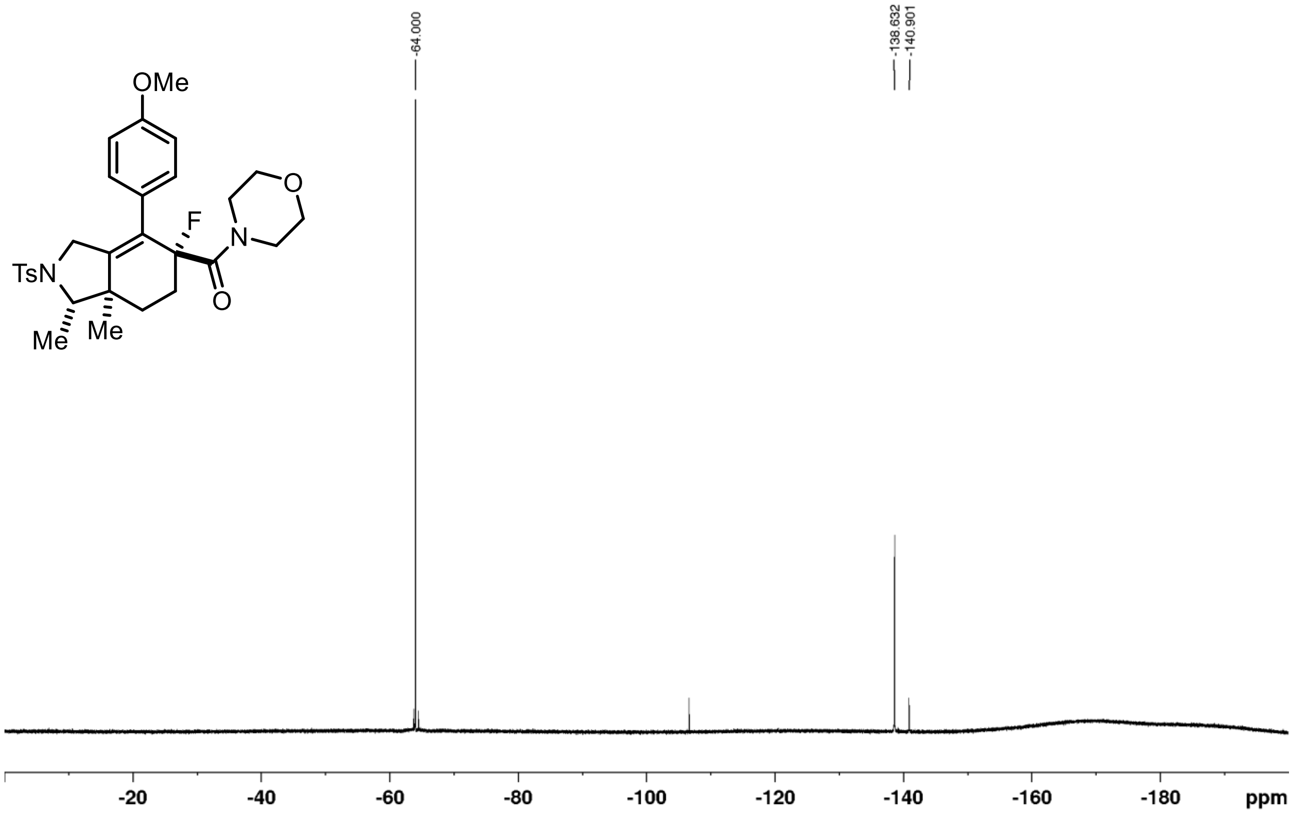

(*E*)-2-Fluoro-4-[(2*R*,3*R*)-4-{(*Z*)-4-methoxybenzylidene}-2,3-dimethyl-1-(4-methylphenyl) pyrrolidin-3-yl]-1-morpholinobut-2-en-1-one (4eb)

Diastereomer mixture (60:40 d.r.)

^{1}H NMR ($CDCl_3$, 400 MHz)

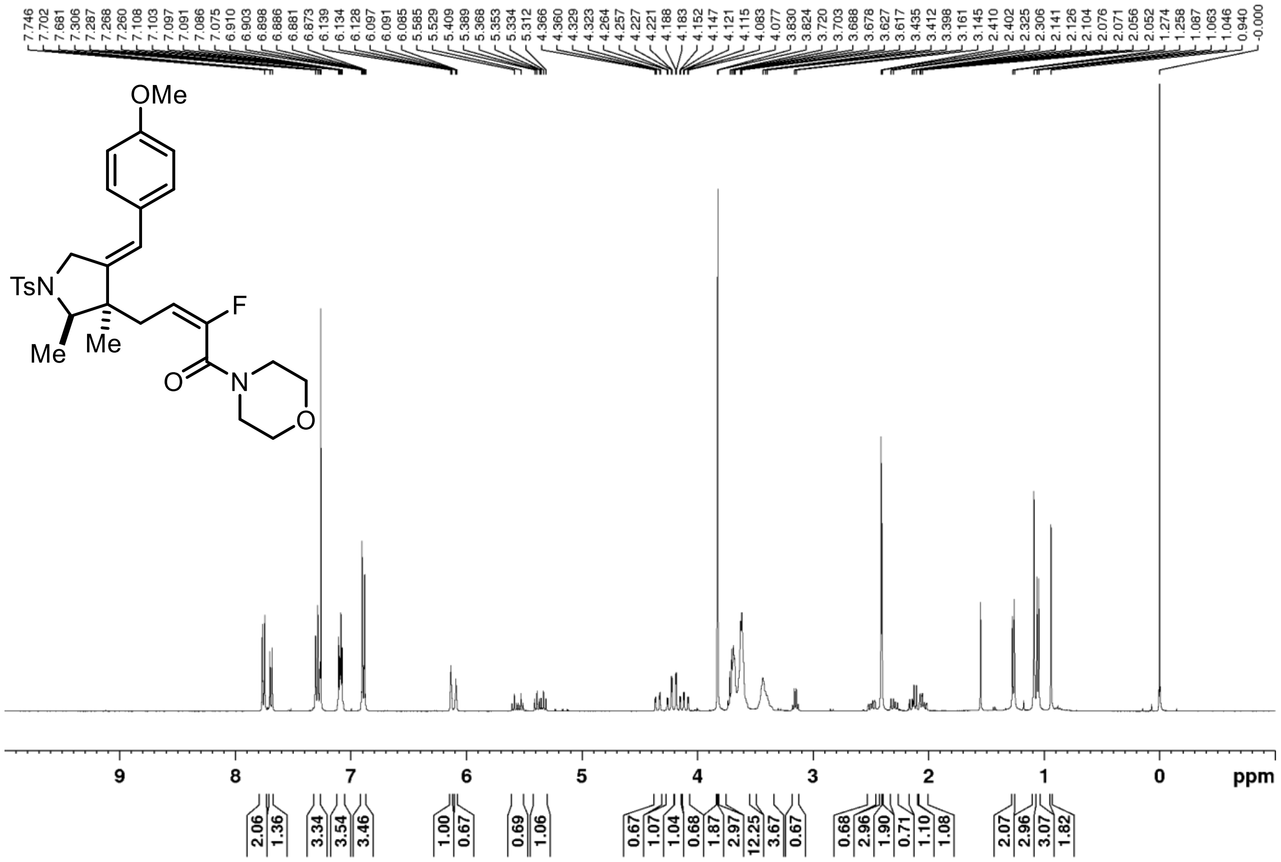

^{13}C NMR ($CDCl_3$, 101 MHz)

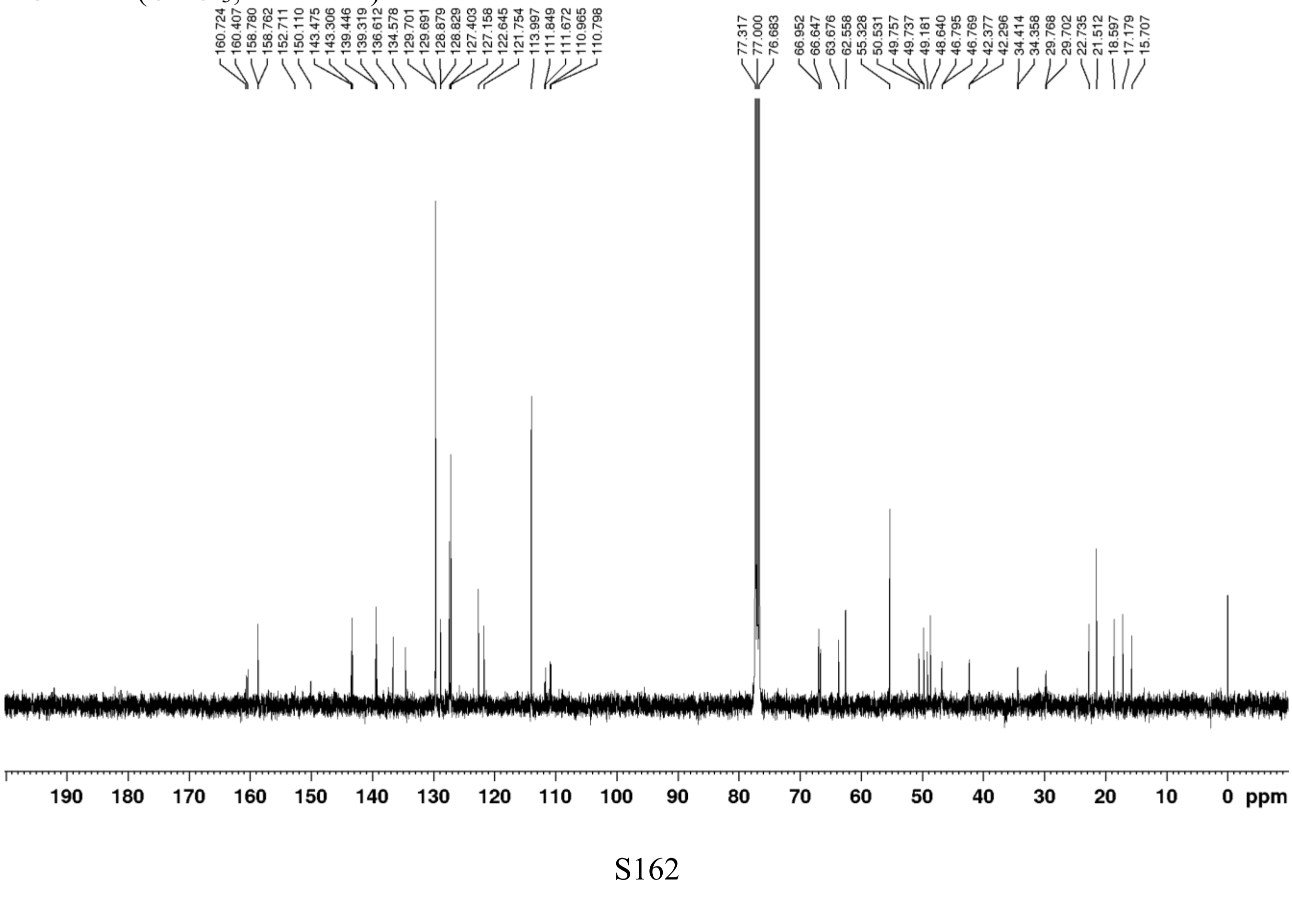

^{19}F NMR ($CDCl_3$, 377 MHz)

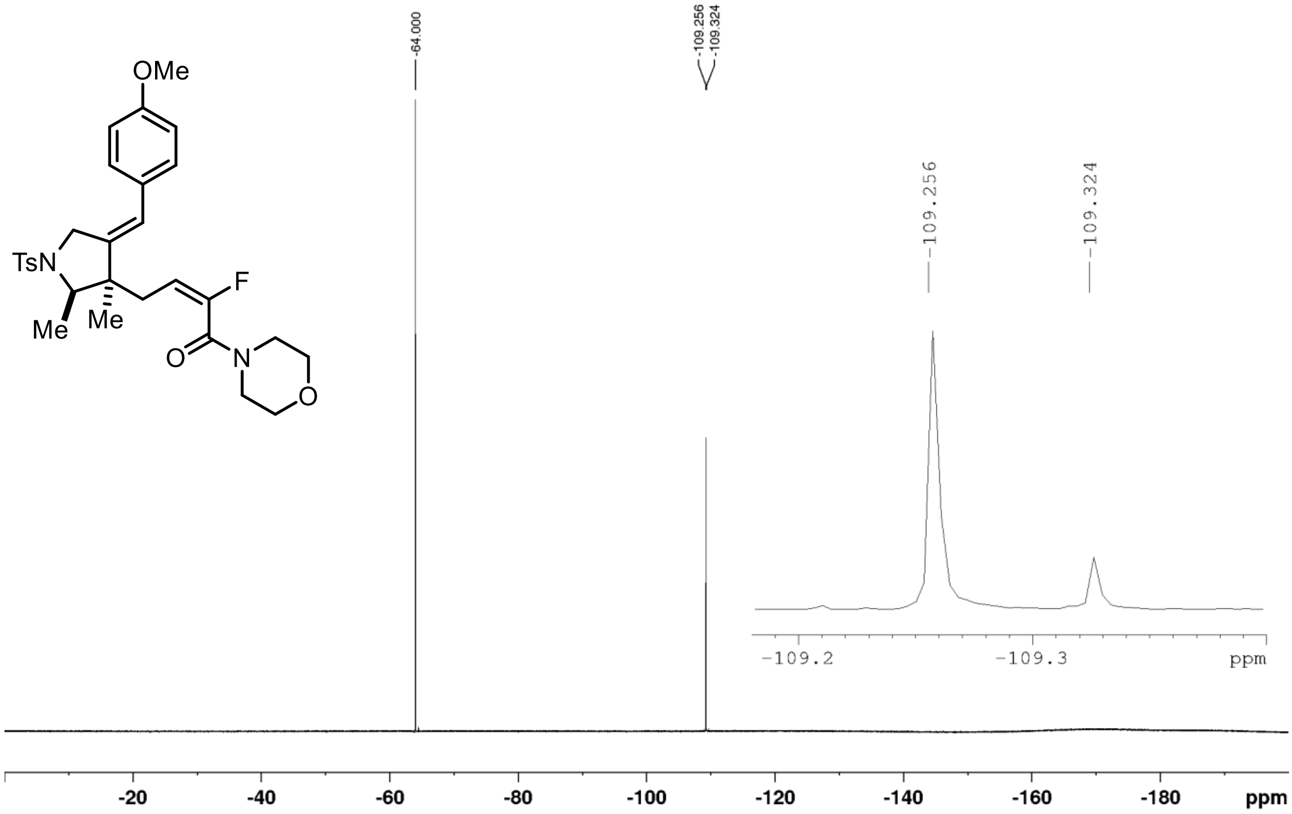

(3*R*,5*R*,7a*R*)-5-Fluoro-2-(4-methylphenyl)-*N*,*N*,3,4-tetraphenyl-2,3,5,6,7,7a-hexahydro-1*H*-isoindole-5-carboxamide (3gc)

^{1}H NMR ($CDCl_3$, 400 MHz)

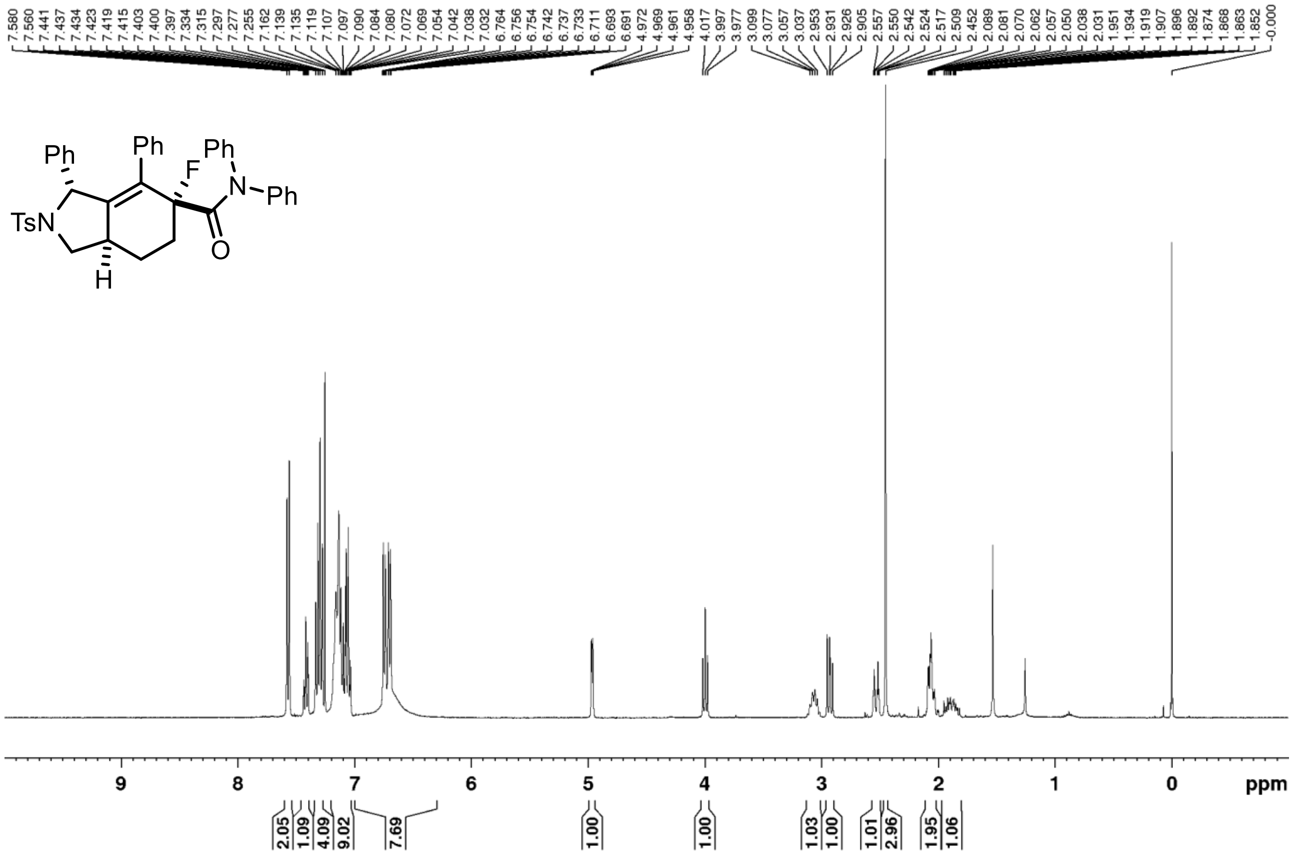

^{13}C NMR ($CDCl_3$, 101 MHz)

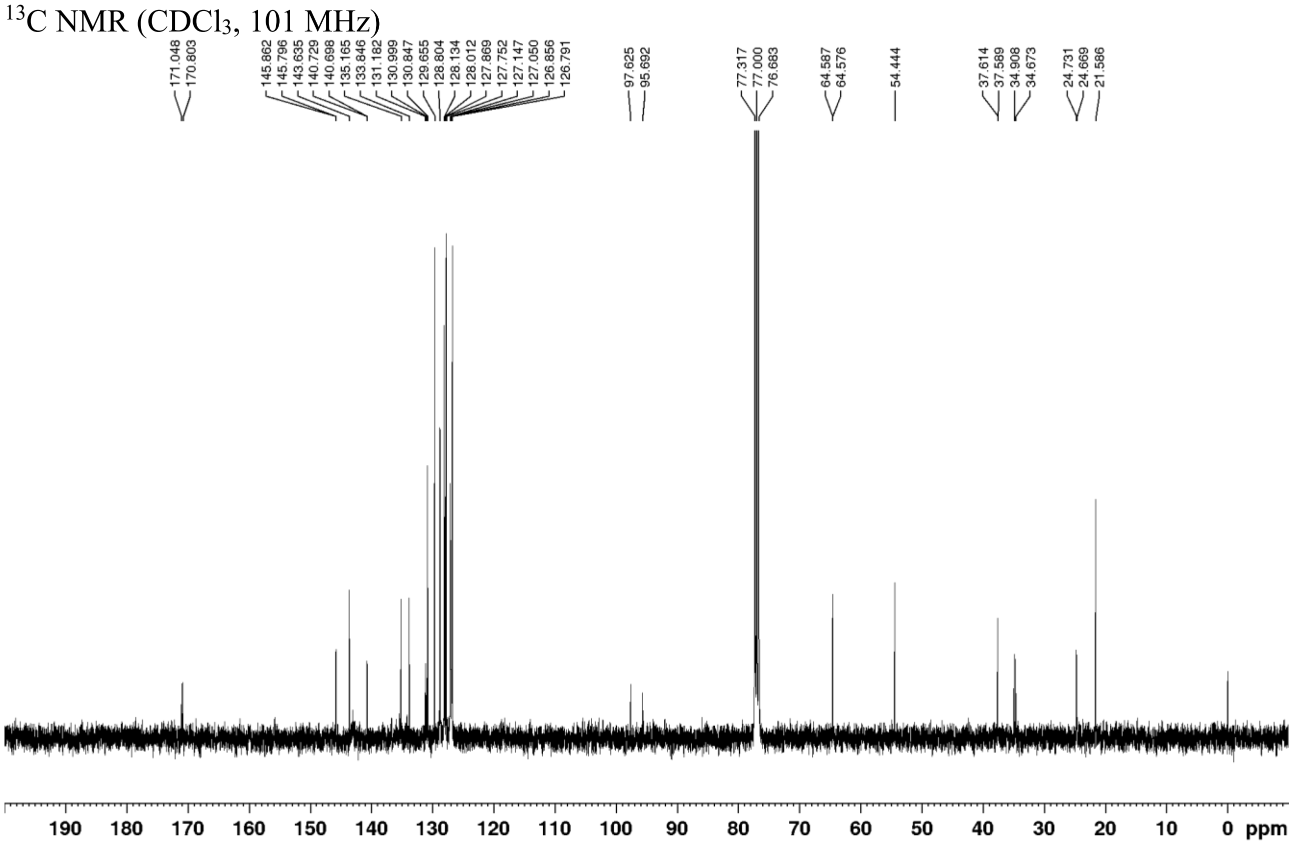

^{19}F NMR ($CDCl_3$, 377 MHz)

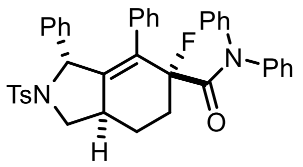

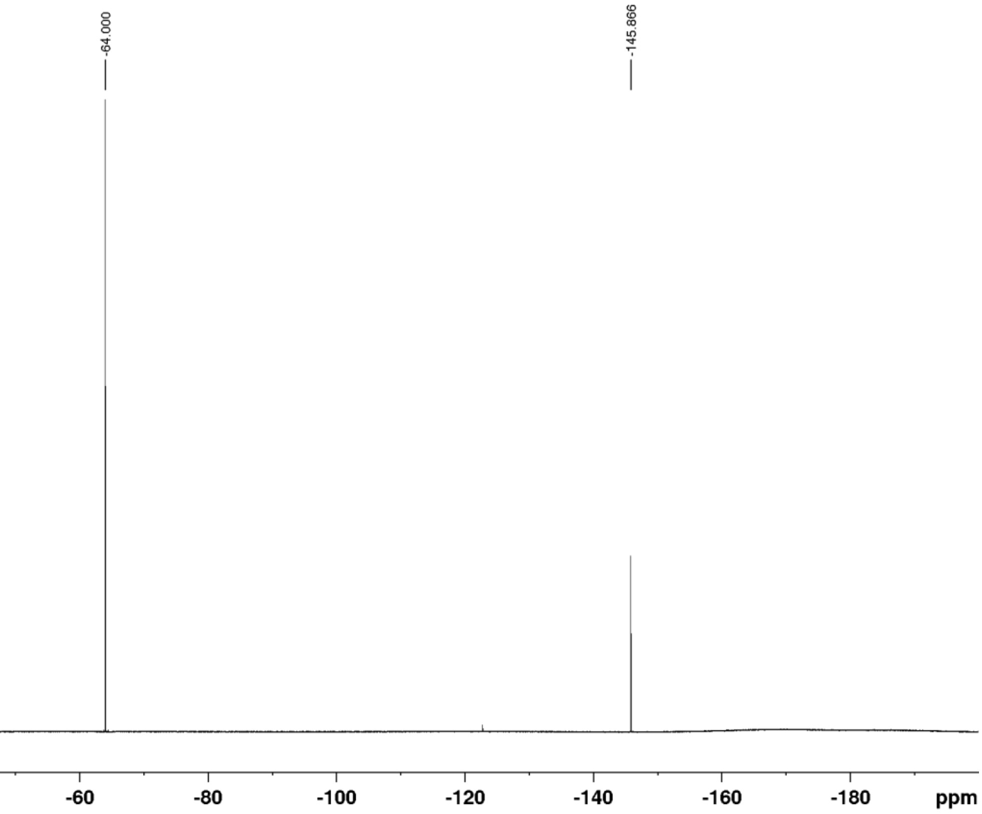

(*E*)-4-{(3*R*,5*S*)-4-(*Z*)-Benzylidene-3-methyl-1-(4-methylphenyl)-5-phenylpyrrolidin-3-yl}-2-fluoro-*N*,*N*-diphenylbut-2-enamide [4gc (major diastereomer)]

^{1}H NMR ($CDCl_3$, 400 MHz)

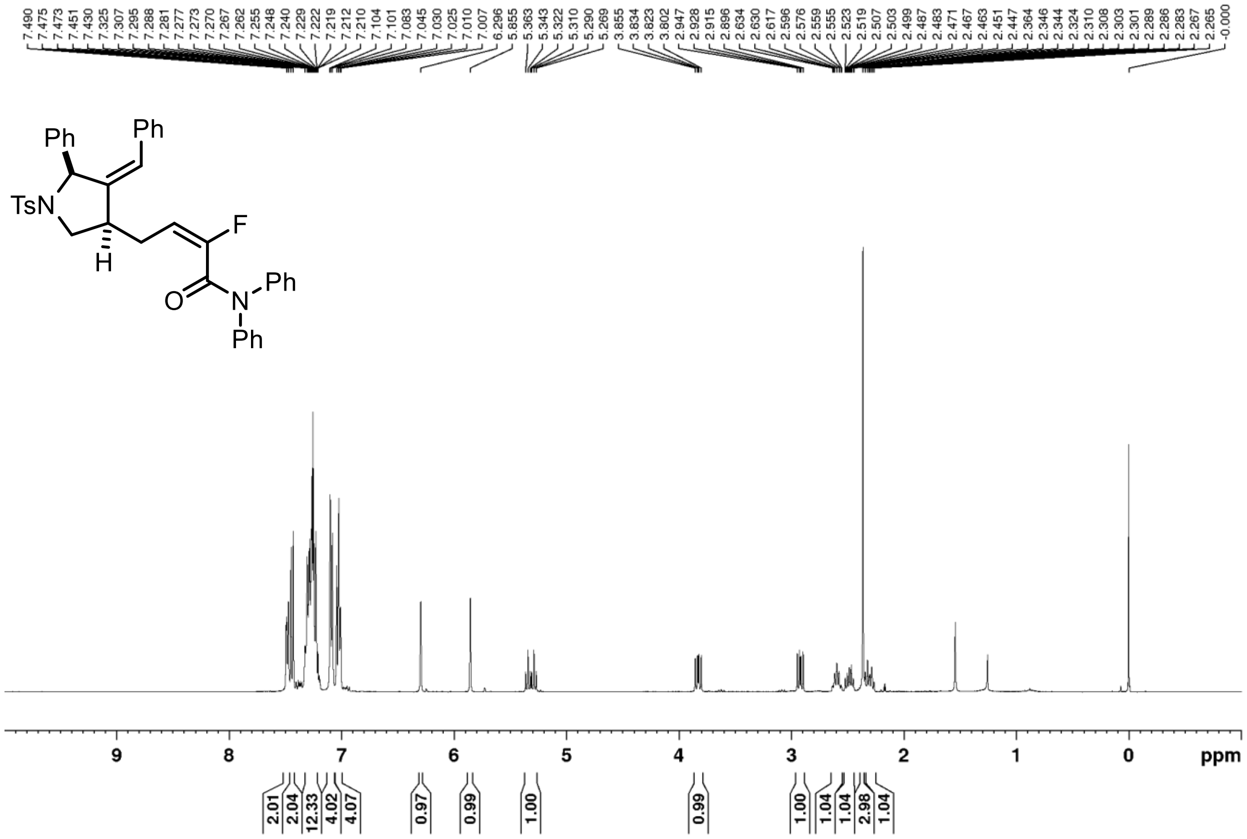

^{13}C NMR ($CDCl_3$, 101 MHz)

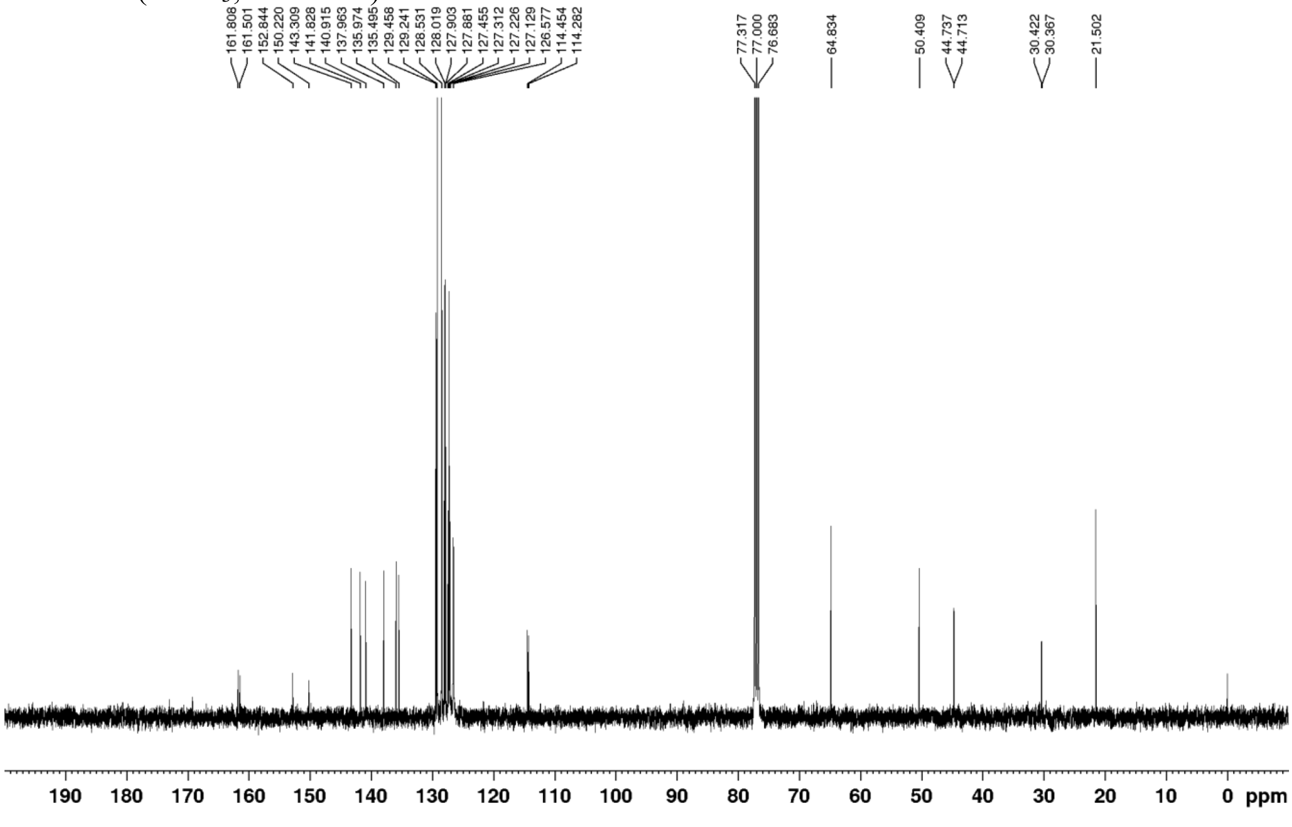

^{19}F NMR (CDCl$_3$, 377 MHz)

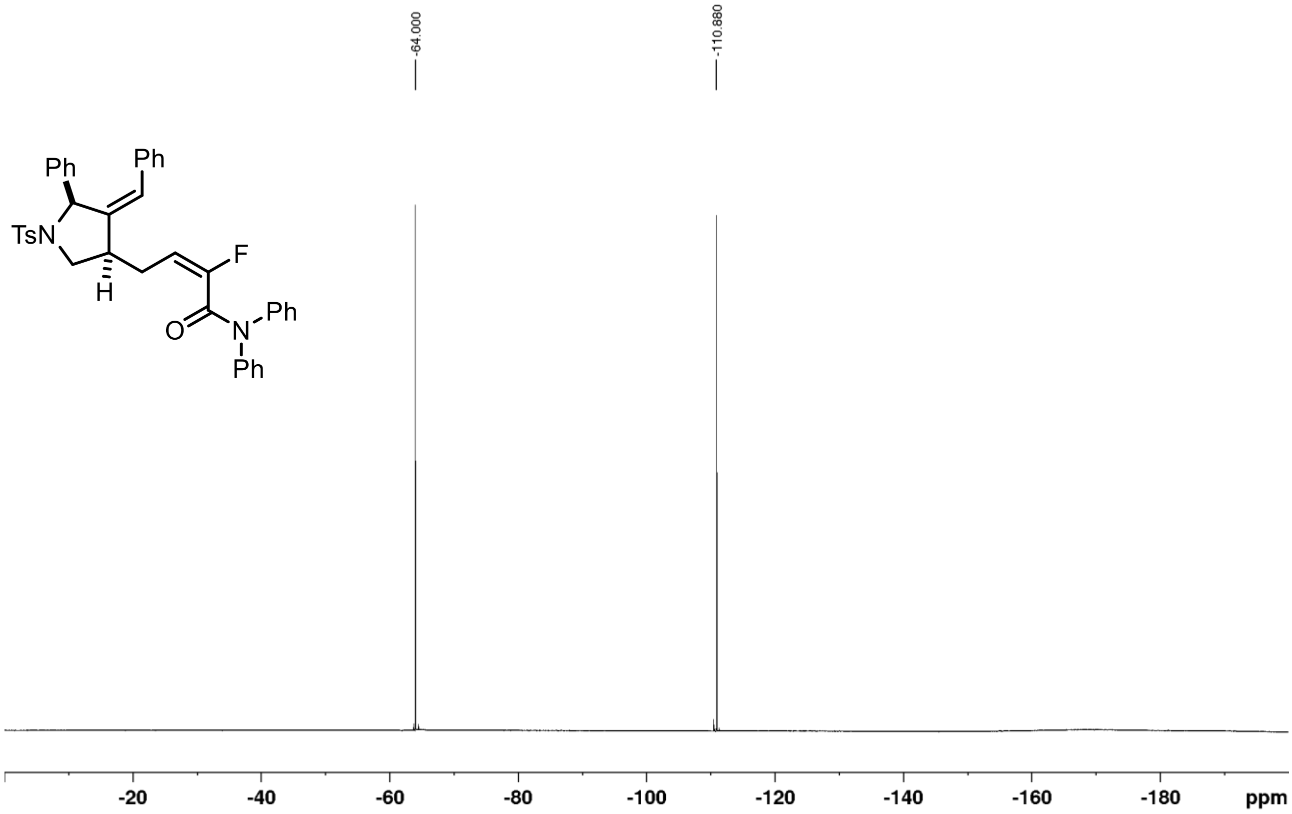

(*E*)-4-{4-(*Z*)-Benzylidene-3-methyl-1-(4-methylphenyl)-5-phenylpyrrolidin-3-yl}-2-fluoro-*N*,*N*-diphenylbut-2-enamide [4gc (minor diastereomer)]

^{1}H NMR (CDCl$_3$, 400 MHz)

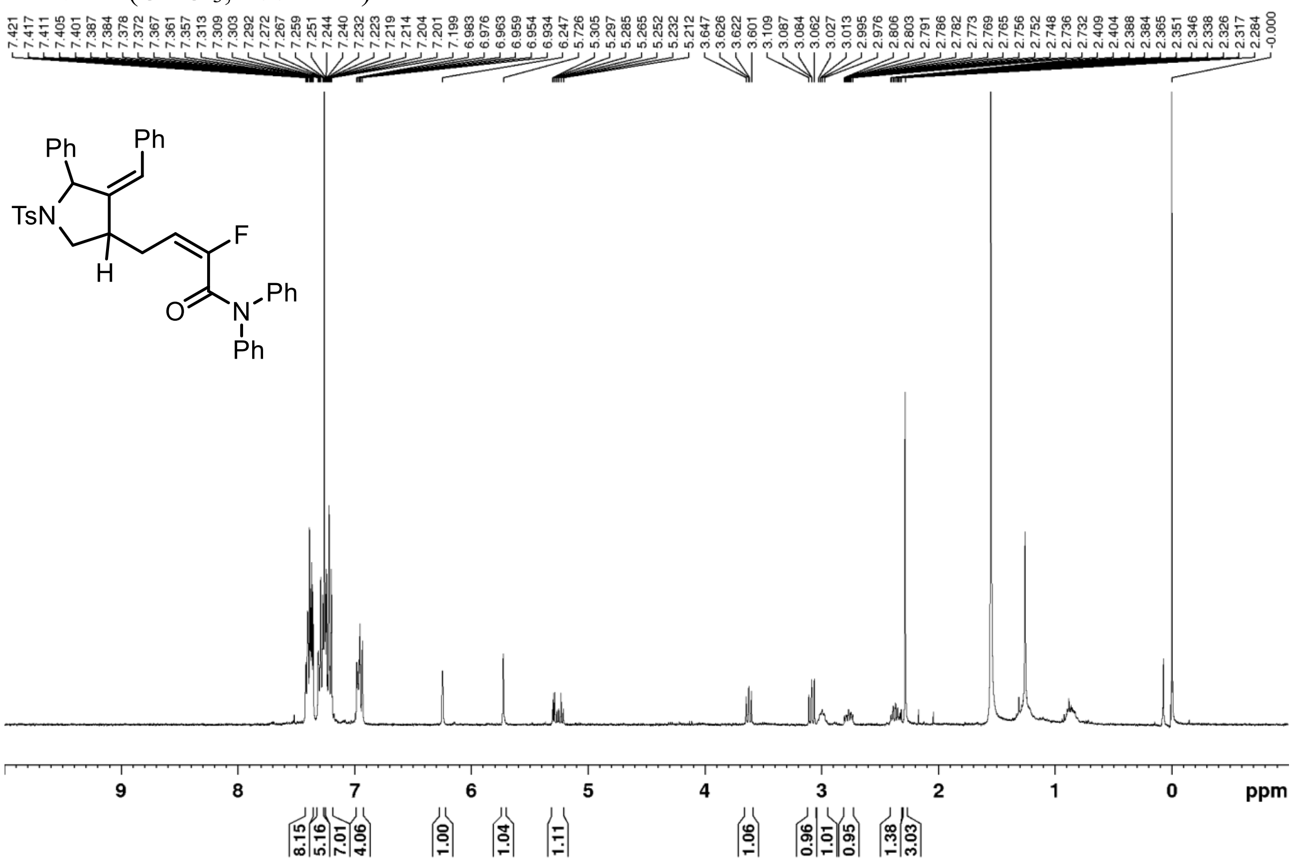

^{19}F NMR (CDCl$_3$, 377 MHz)

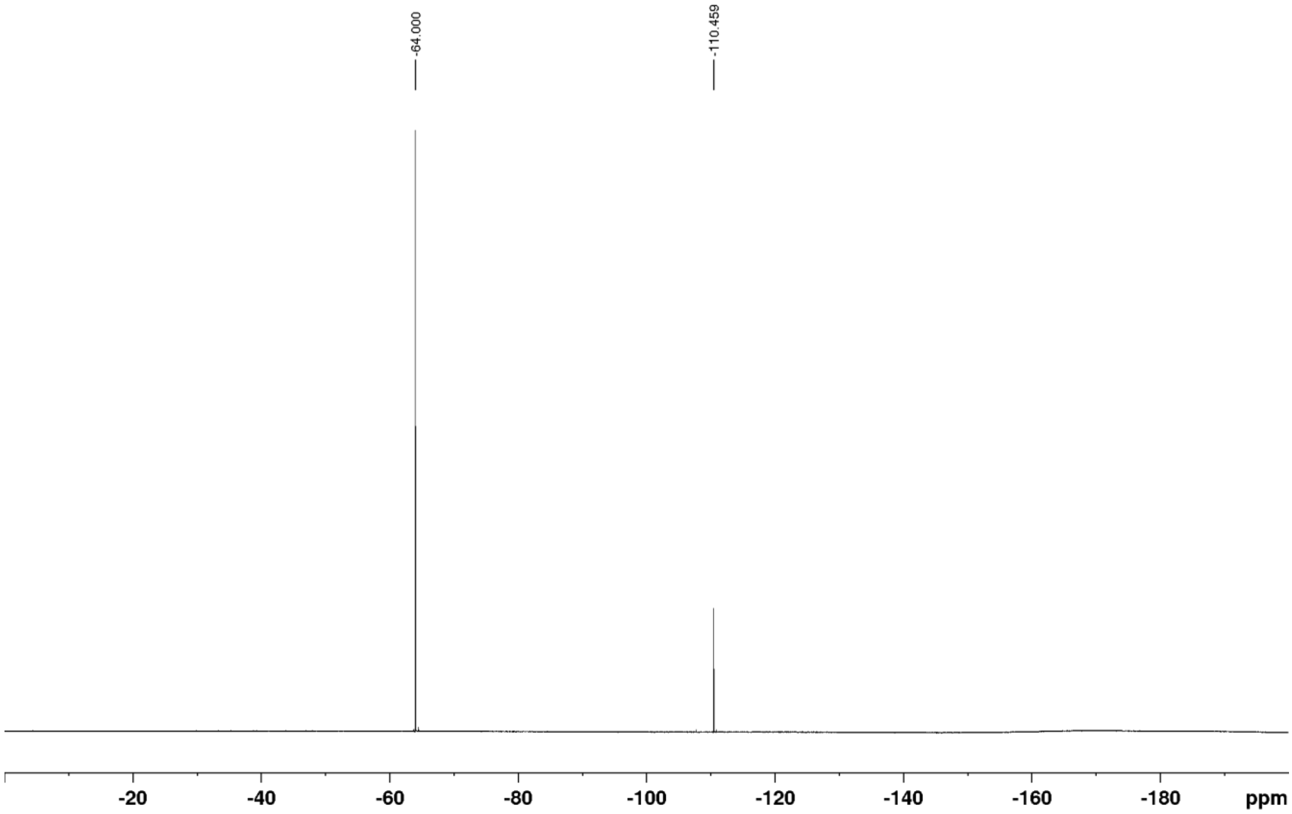

(3*S*,3a*R*,6*R*)-6-Fluoro-3,3a-dimethyl-7-phenyl-6-(pyrrolidine-1-carbonyl)-3a,4,5,6-tetrahydroisobenzofuran-1(3*H*)-one (3ha)

^{1}H NMR (CDCl$_3$, 400 MHz)

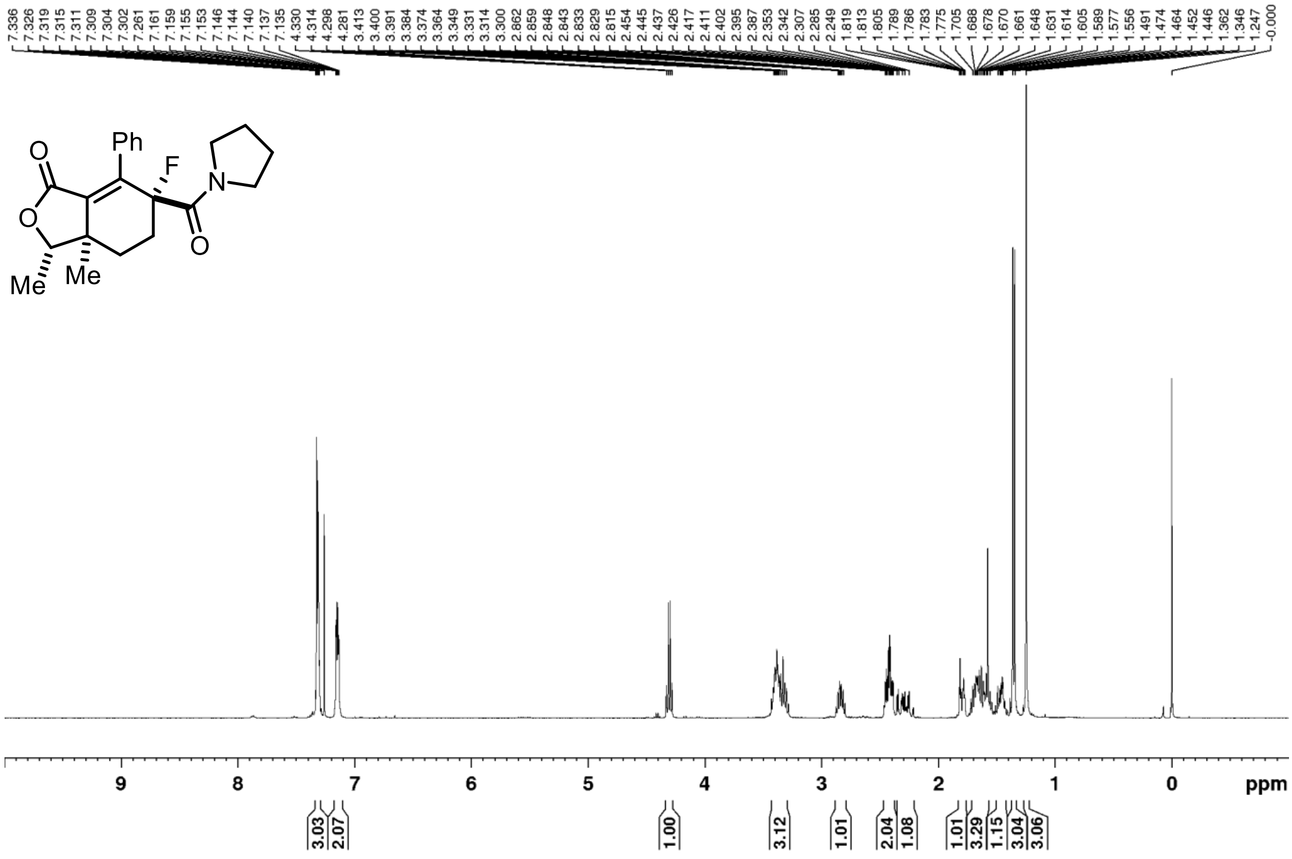

^{13}C NMR (CDCl$_3$, 101 MHz)

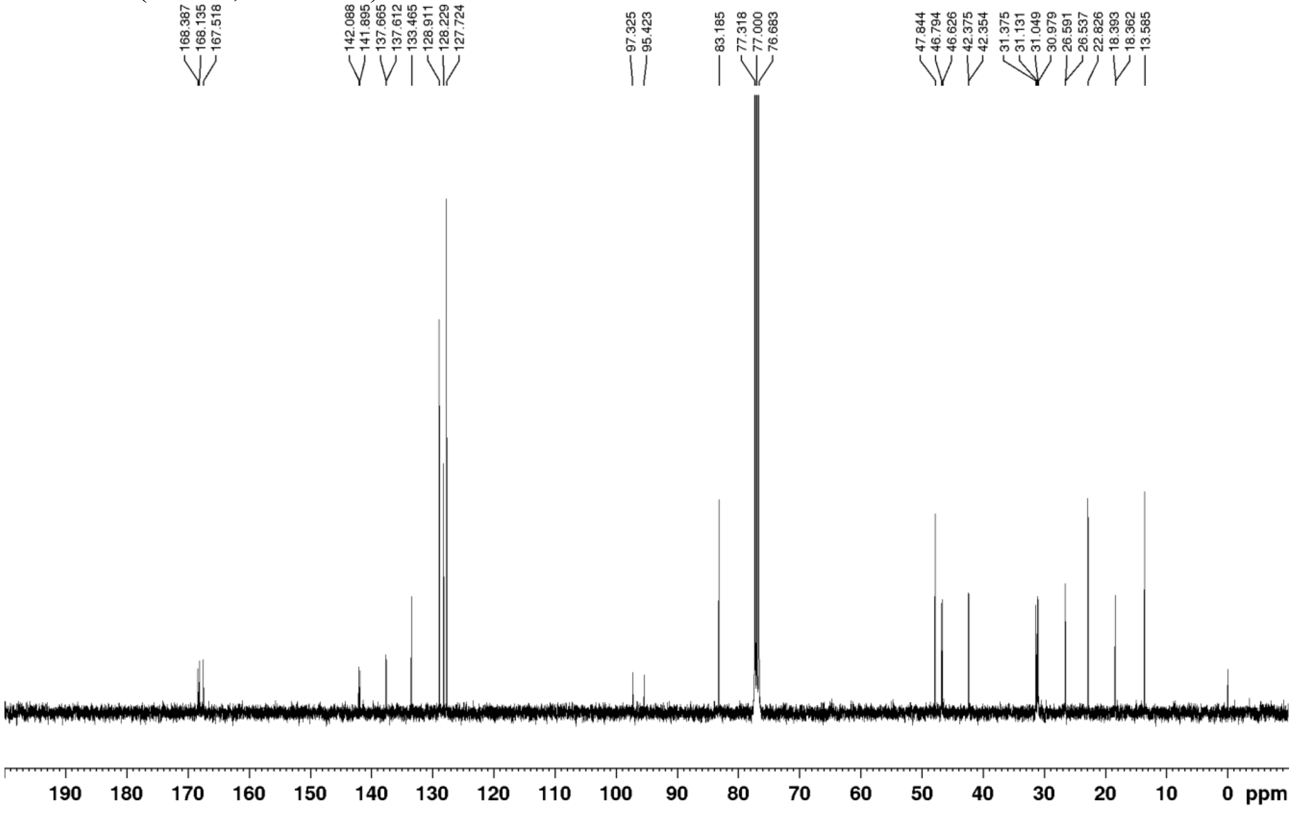

^{19}F NMR ($CDCl_3$, 377 MHz)

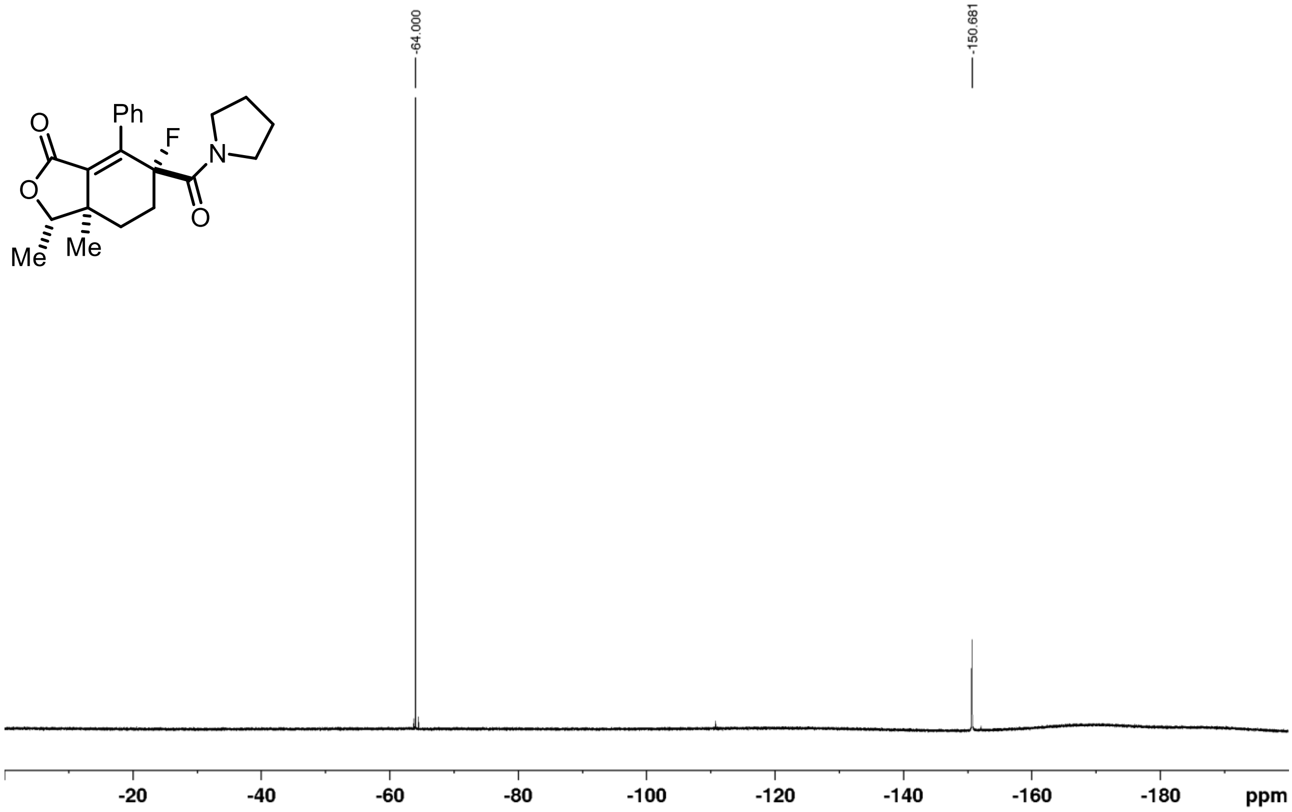

(4*S*,5*R*)-3-(*Z*)-Benzylidene-4-{(*E*)-3-fluoro-4-oxo-4-(pyrrolidin-1-yl)but-2-en-1-yl}-4,5-dimethyldihydrofuran-2(3*H*)-one (4ha)

Diastereomer mixture (92:8 d.r.)

^{1}H NMR (CDCl$_3$, 400 MHz)

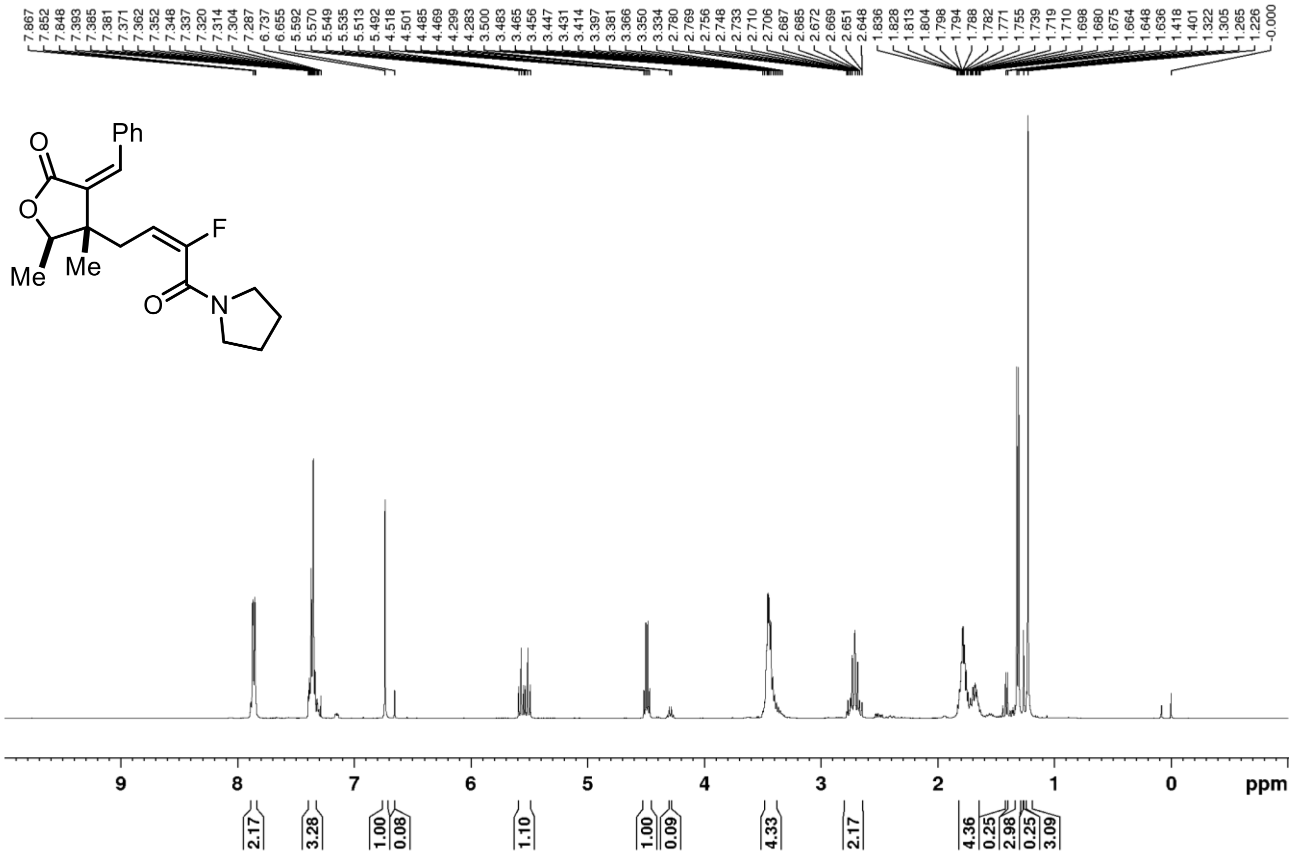

^{13}C NMR (CDCl$_3$, 101 MHz)

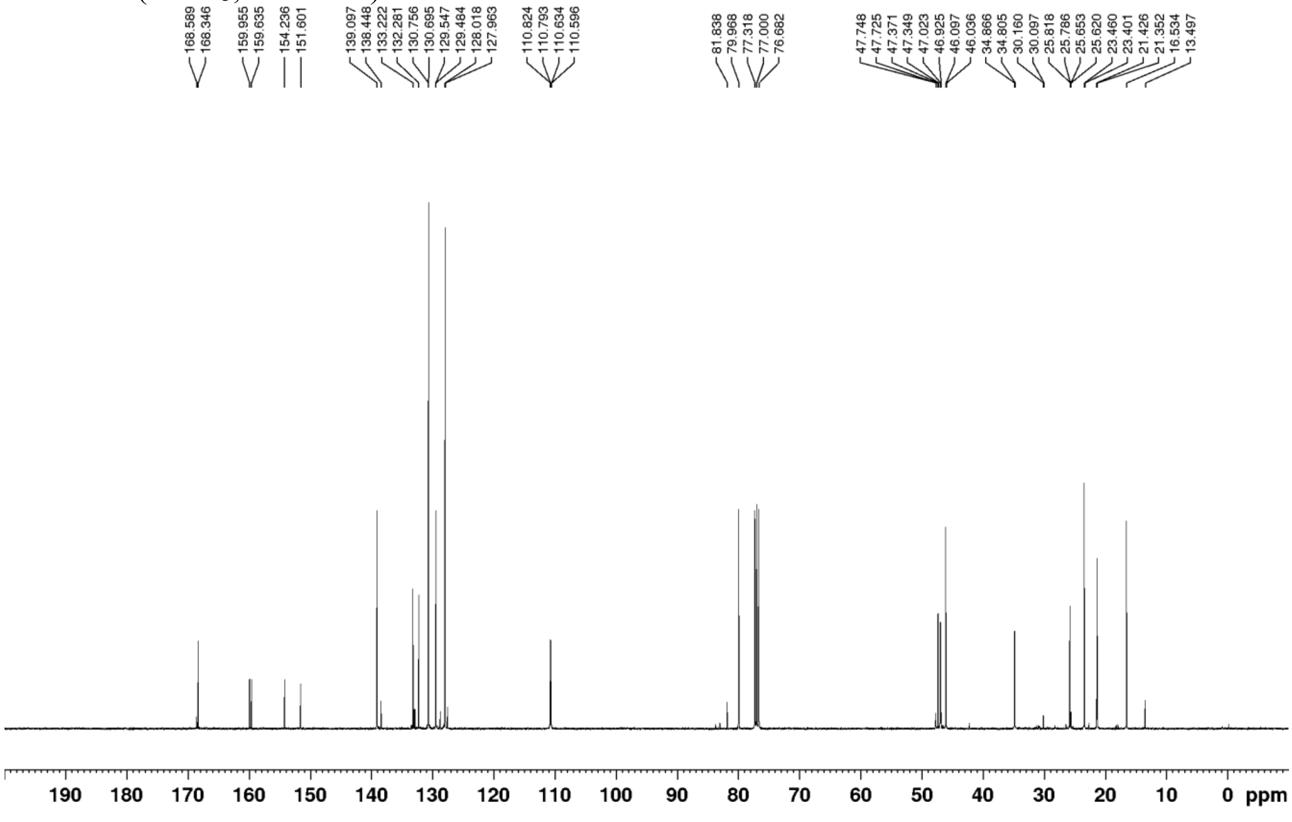

^{19}F NMR ($CDCl_3$, 377 MHz)

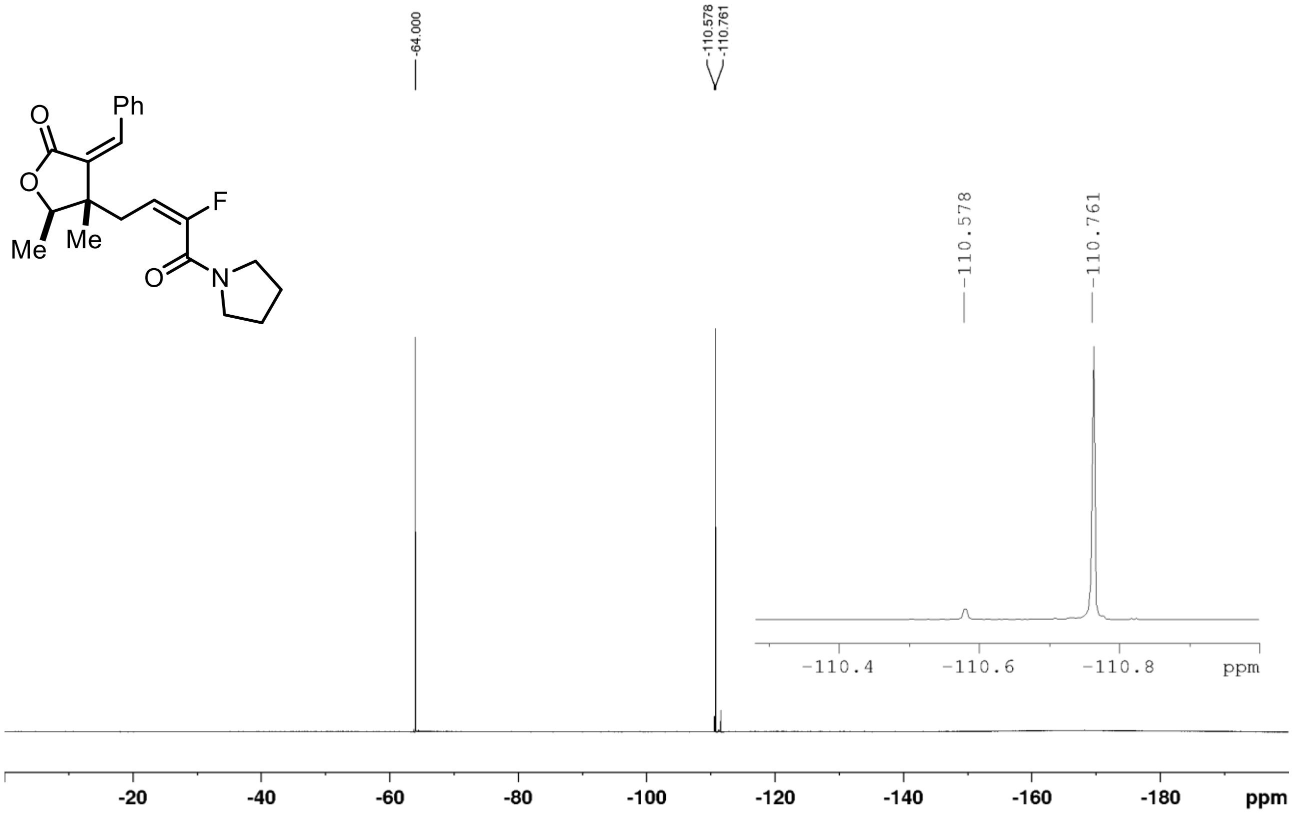

(3*S*,3a*R*,6*R*)-7-(4-Bromophenyl)-6-fluoro-3,3a-dimethyl-6-(pyrrolidine-1-carbonyl)-3a,4,5,6-tetrahydroisobenzofuran-1(3*H*)-one (3ia)

^{1}H NMR (CDCl$_3$, 400 MHz)

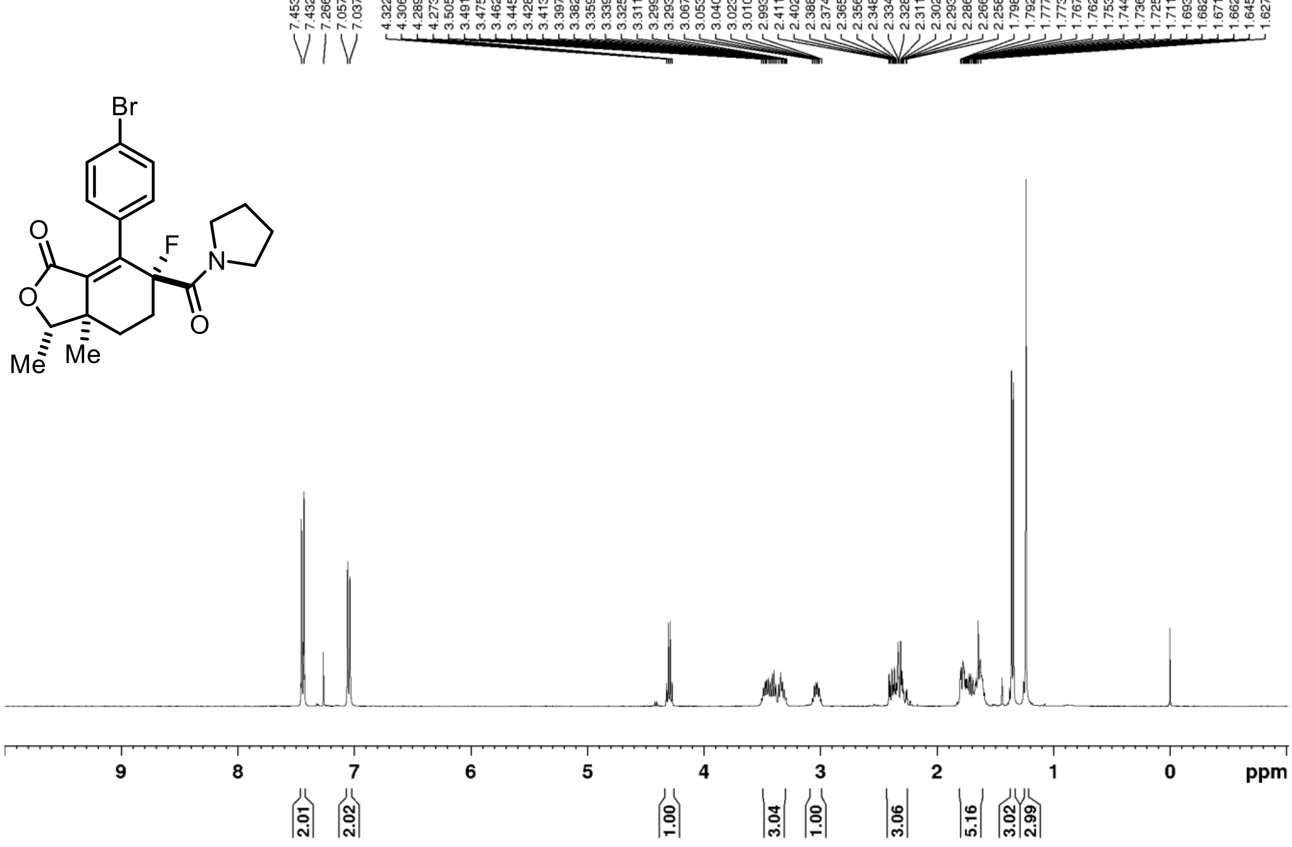

^{13}C NMR (CDCl$_3$, 101 MHz)

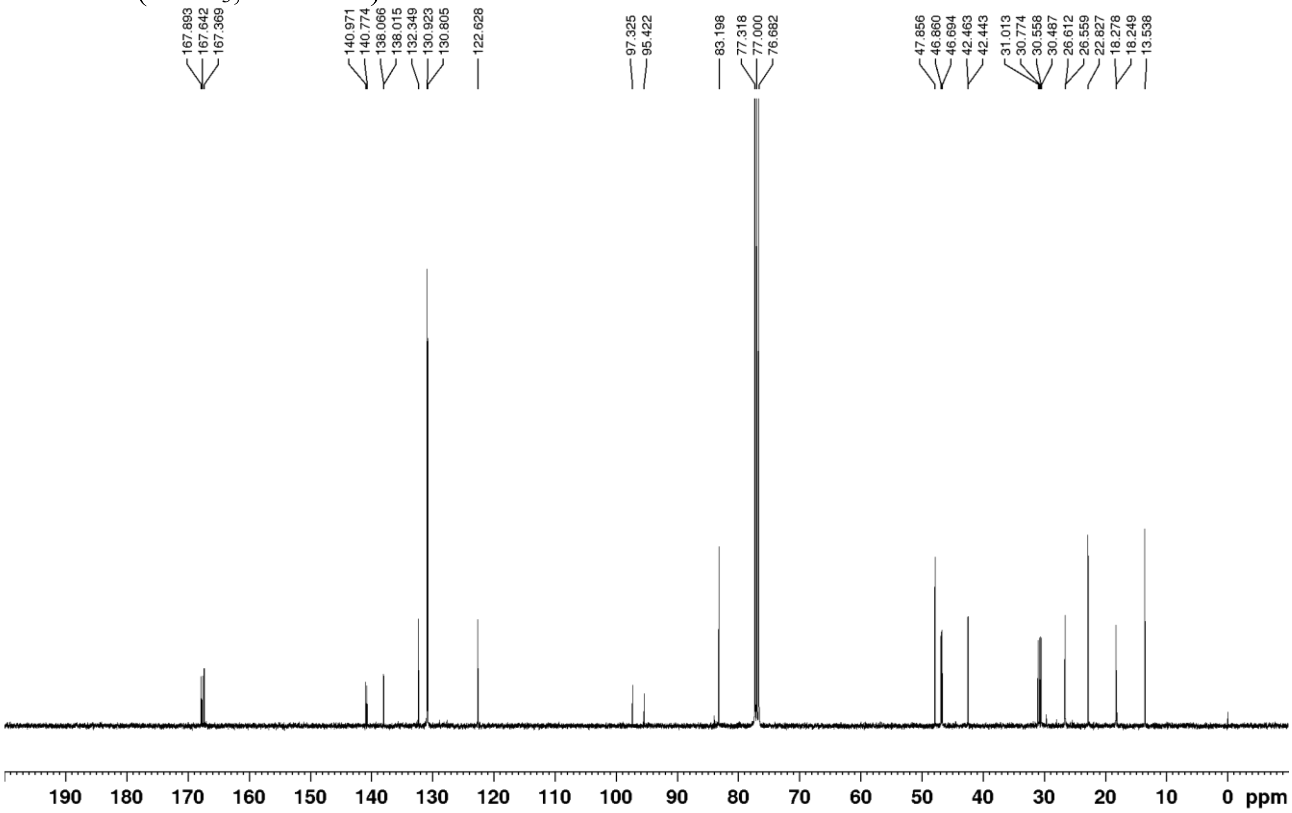

^{19}F NMR ($CDCl_3$, 377 MHz)

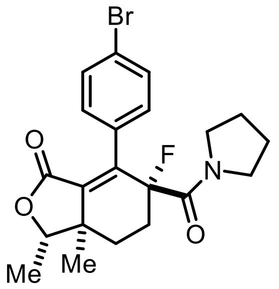
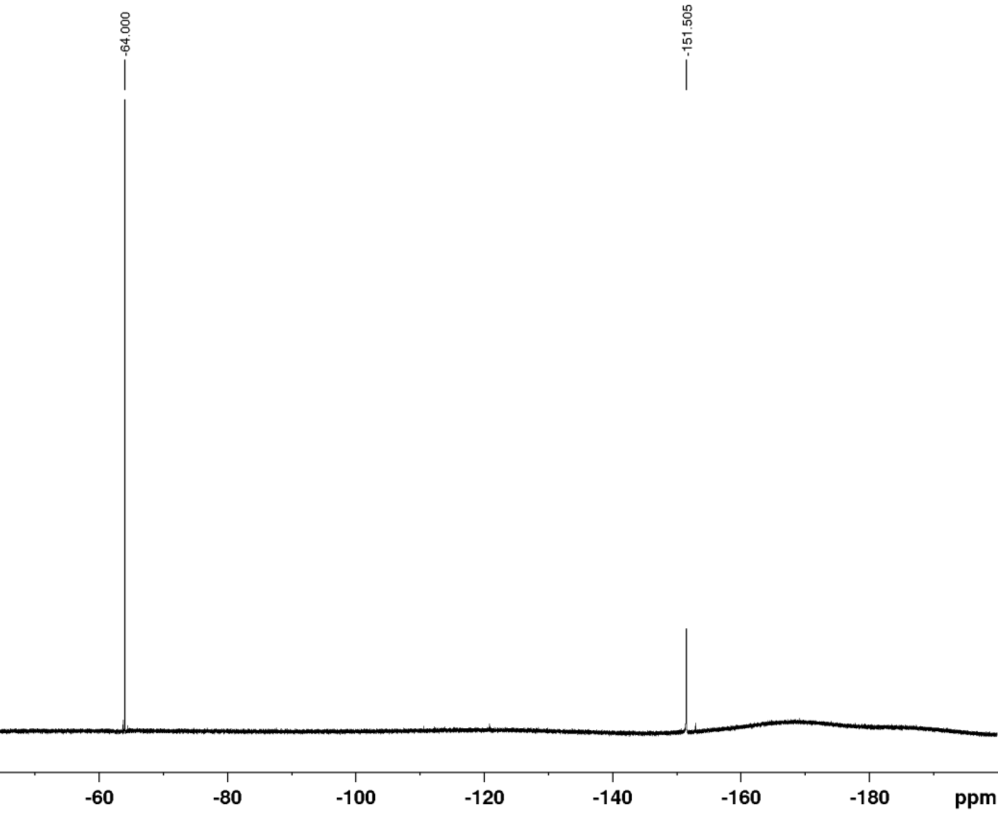

(4*S*,5*R*)-3-(*Z*)-4-Bromobenzylidene-4-{(*E*)-3-fluoro-4-oxo-4-(pyrrolidin-1-yl)but-2-en-1-yl}-4,5-dimethyldihydrofuran-2(3*H*)-one (4ia)

Diastereomer mixture (94:6 d.r.)

^{1}H NMR ($CDCl_3$, 400 MHz)

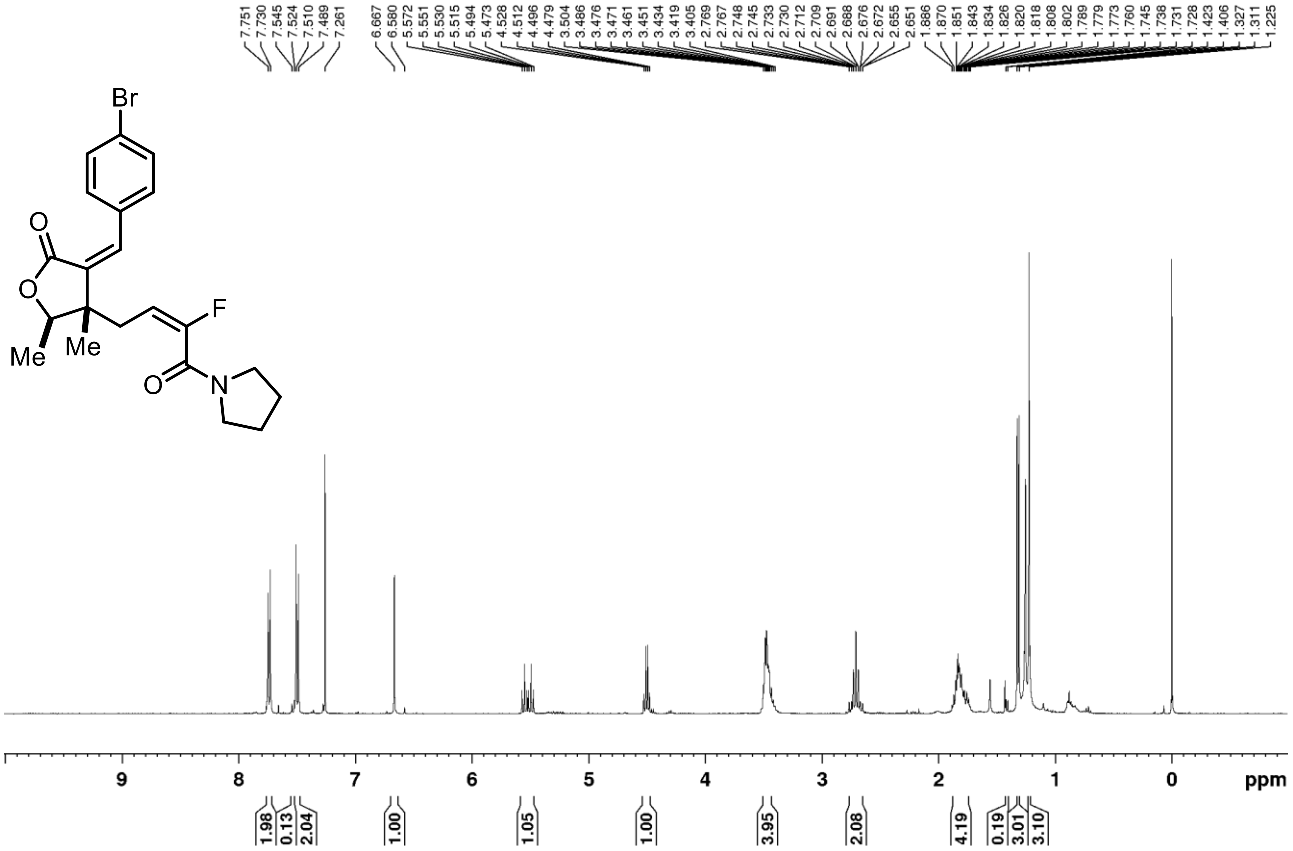

^{13}C NMR ($CDCl_3$, 101 MHz)

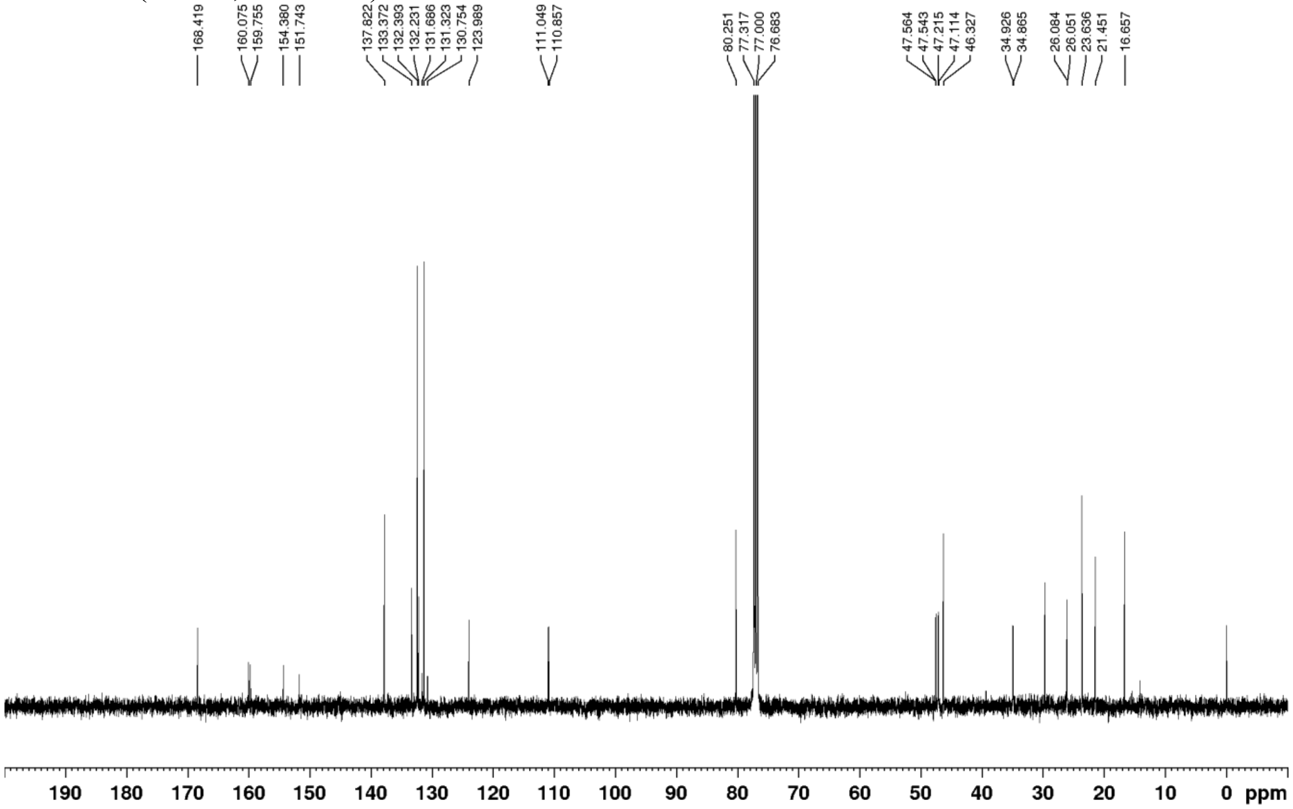

^{19}F NMR ($CDCl_3$, 377 MHz)

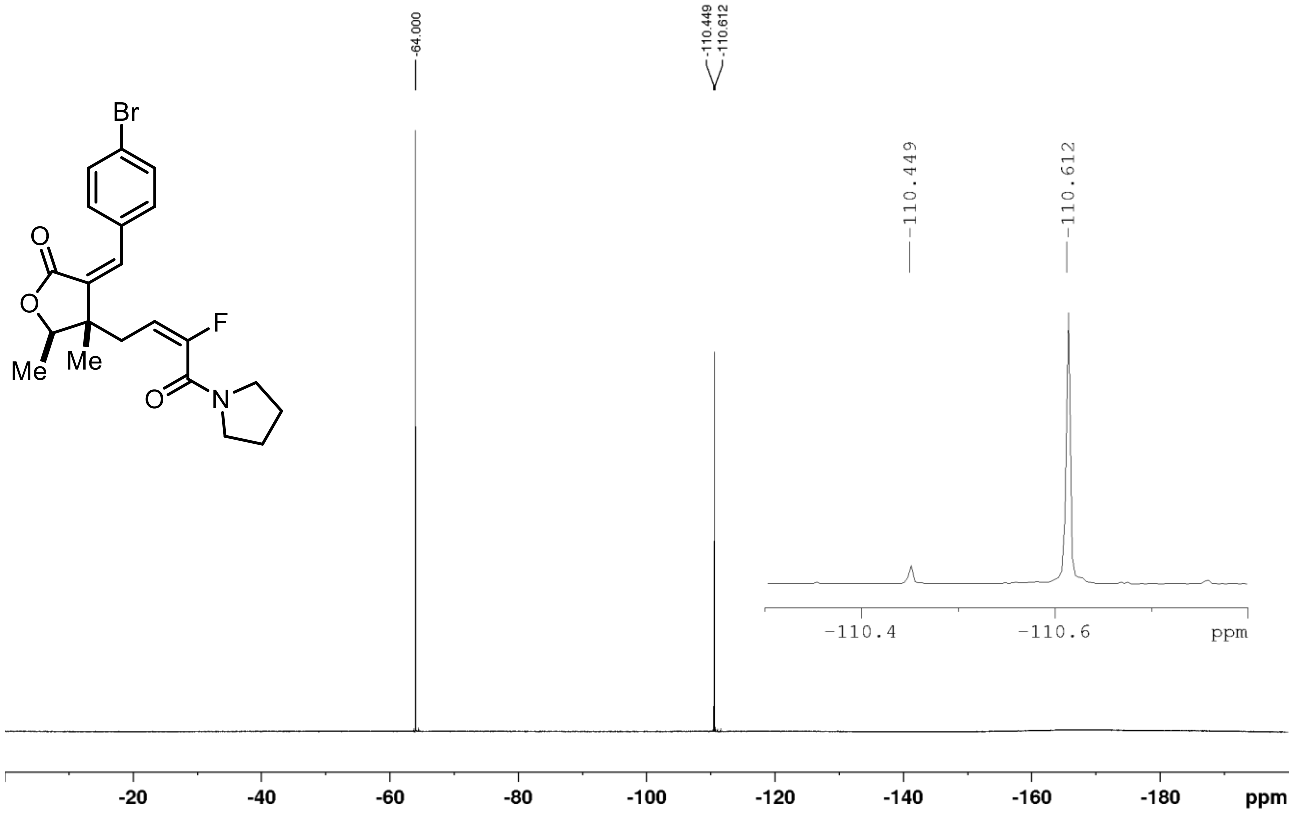

(3*S*,3a*R*,6*R*)-6-Fluoro-3a-methyl-3,7-diphenyl-6-(pyrrolidine-1-carbonyl)-3a,4,5,6-tetrahydroisobenzofuran-1(3*H*)-one (3ja)

^{1}H NMR (CDCl$_3$, 400 MHz)

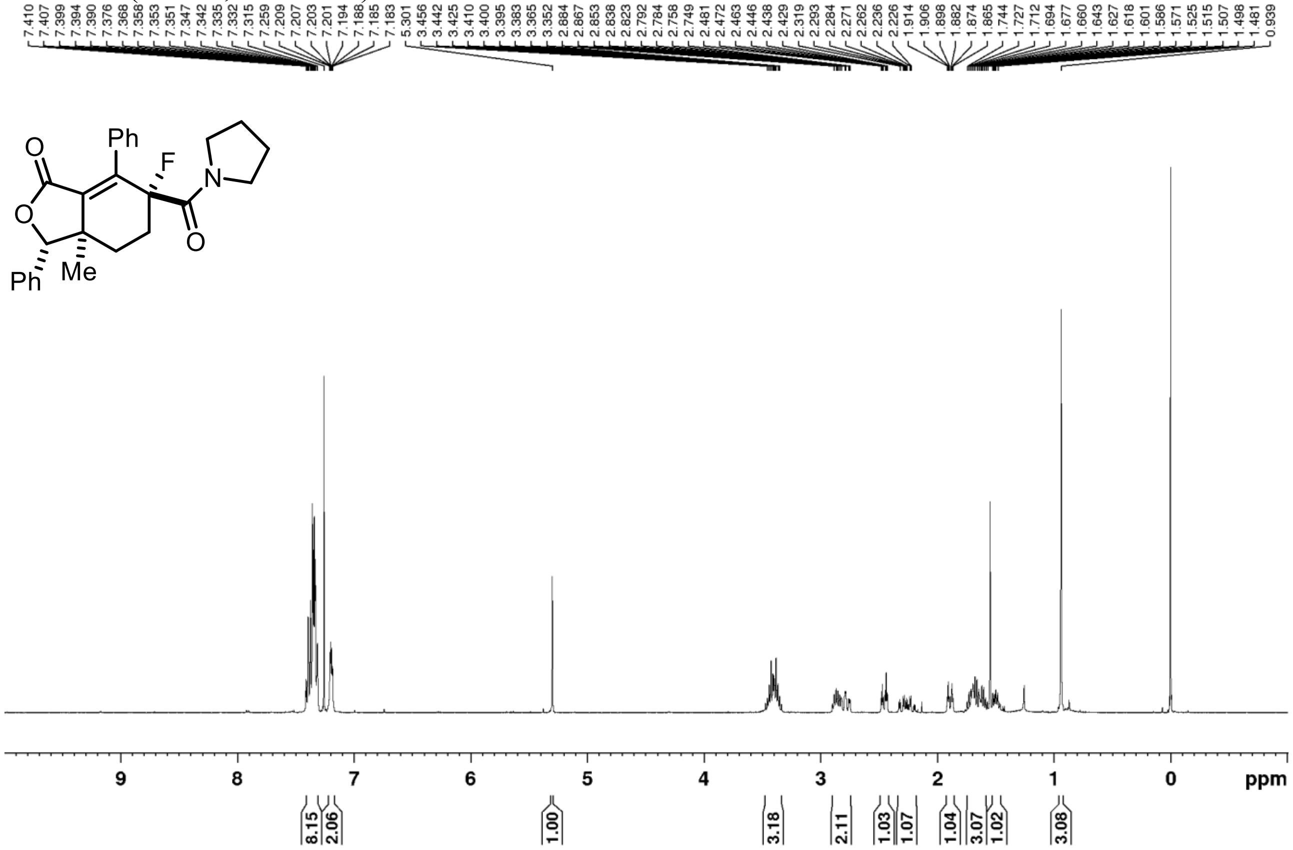

^{13}C NMR (CDCl$_3$, 101 MHz)

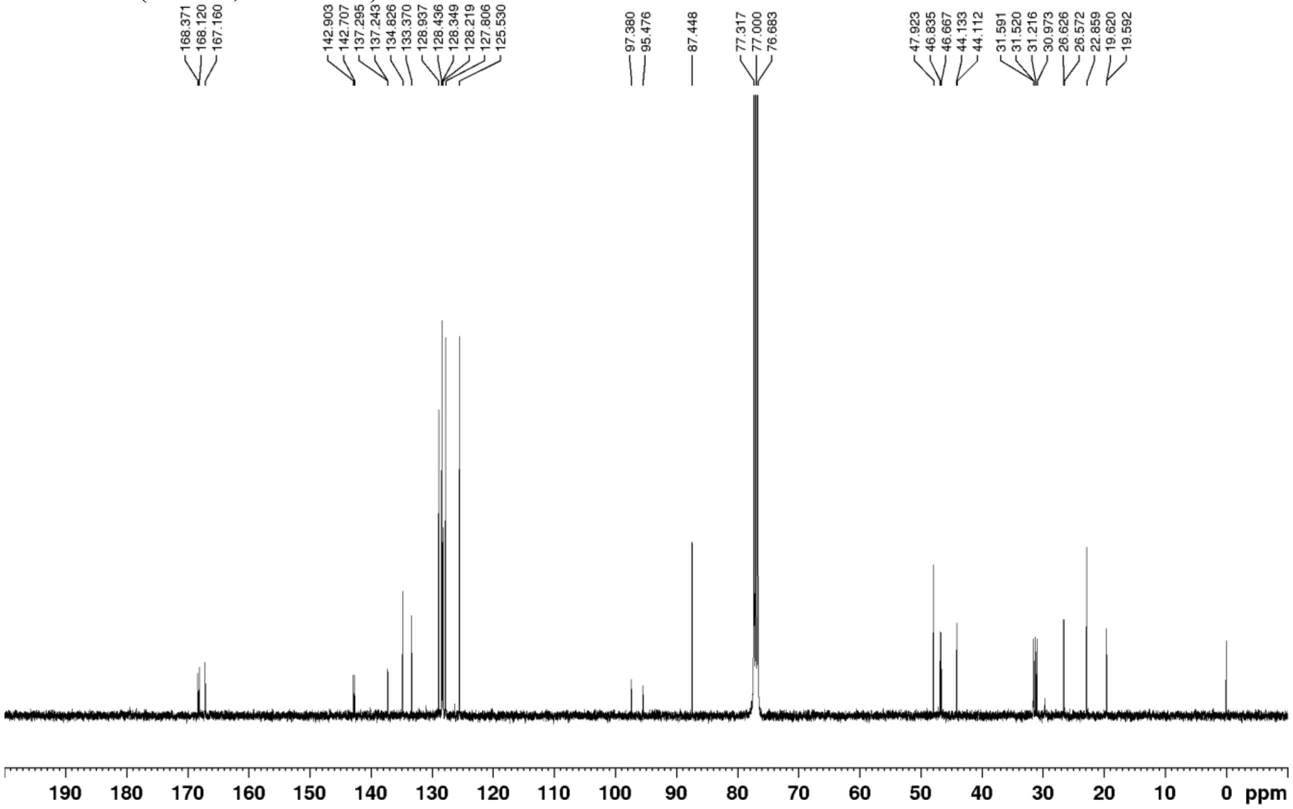

^{19}F NMR ($CDCl_3$, 377 MHz)

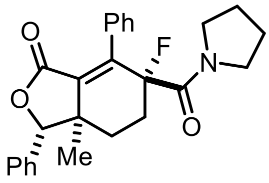

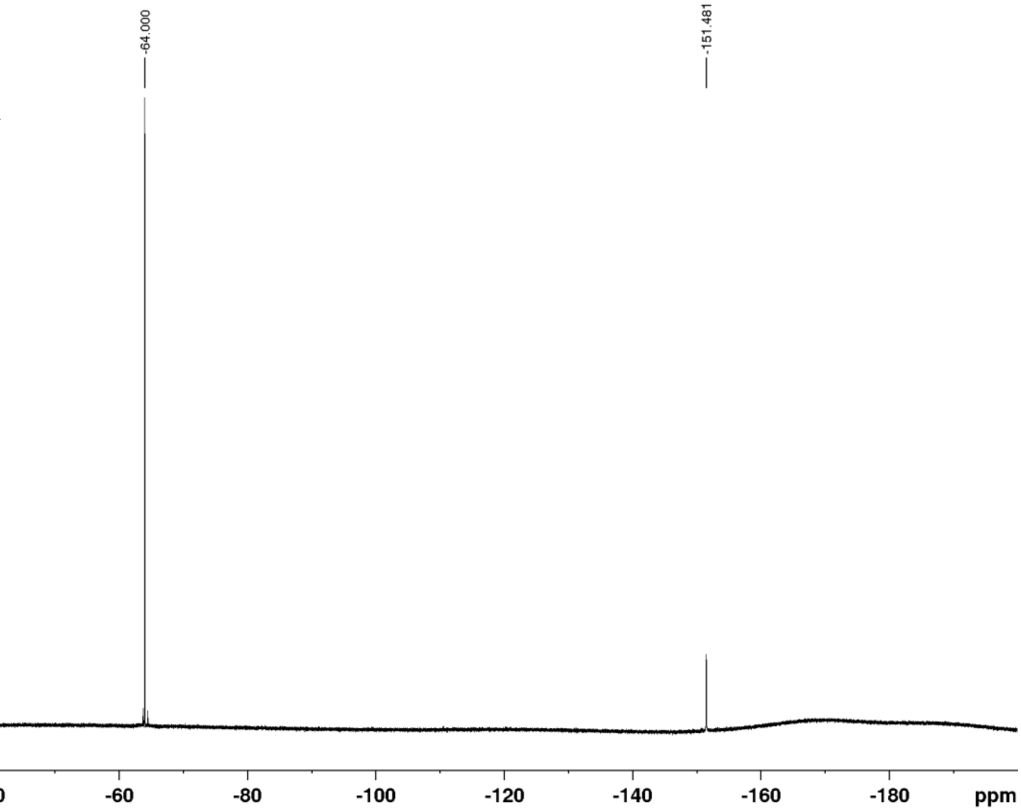

(4*S*,5*R*)-3-(*Z*)-Benzylidene-4-{(*E*)-3-fluoro-4-oxo-4-(pyrrolidin-1-yl)but-2-en-1-yl}-4-methyl-5-phenyldihydrofuran-2(3*H*)-one (4ja)

^{1}H NMR (CDCl$_3$, 400 MHz)

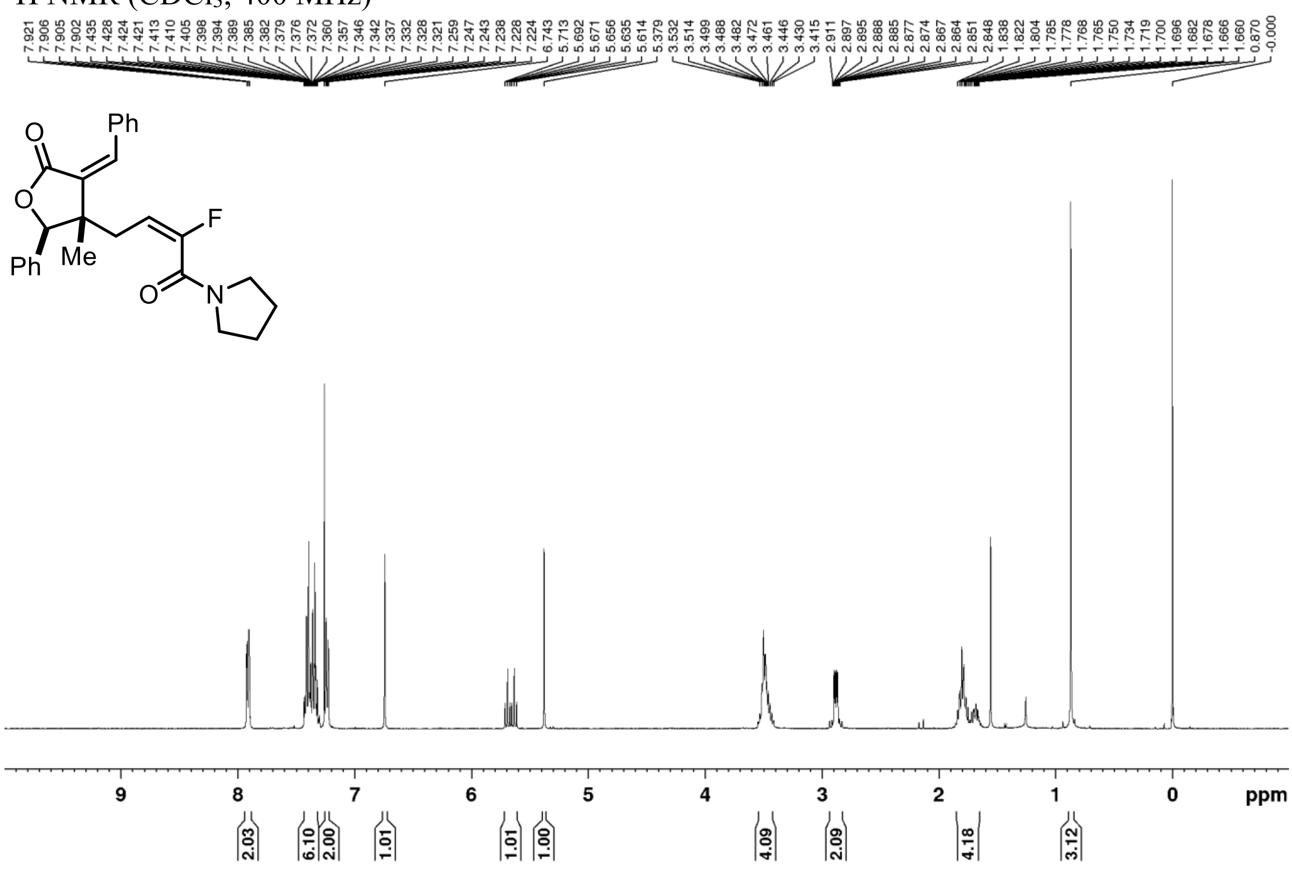

^{13}C NMR (CDCl$_3$, 101 MHz)

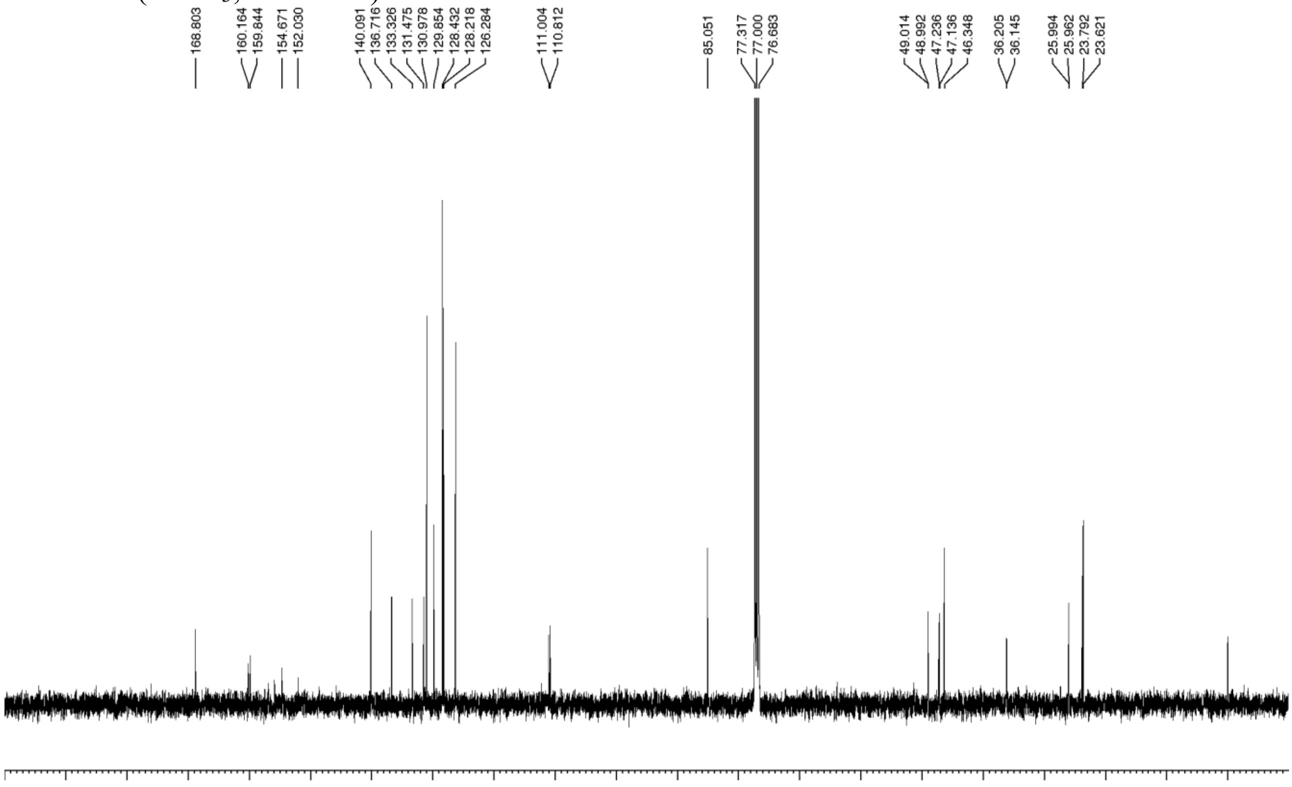

^{19}F NMR ($CDCl_3$, 377 MHz)

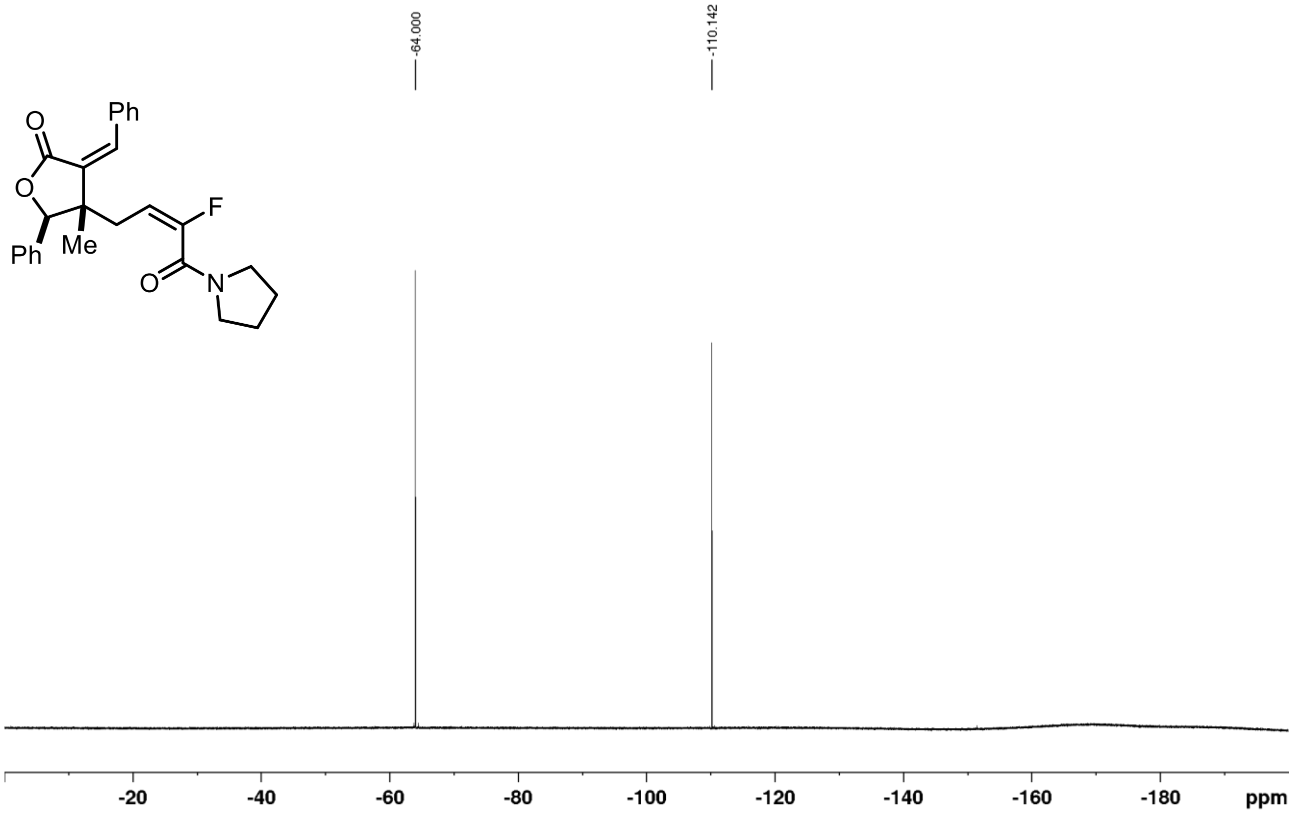

(8*R*,10a*R*,10b*S*)-8-Fluoro-10a-methyl-7-phenyl-8-(pyrrolidine-1-carbonyl)-8,9,10,10a, 10b,11-hexahydro-6*H*-isoindolo[2,1-a]indol-6-one (3ka)

^{1}H NMR ($CDCl_3$, 400 MHz)

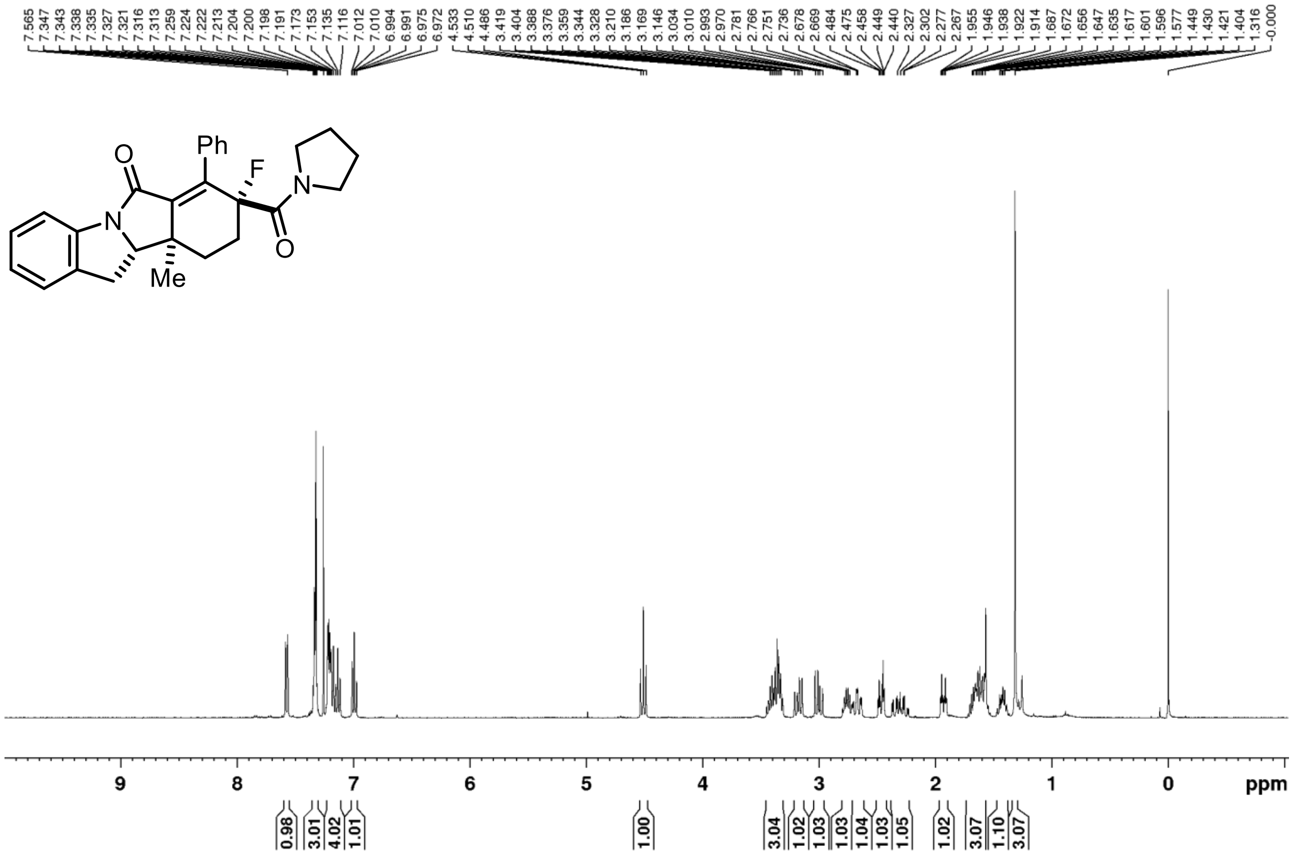

^{13}C NMR ($CDCl_3$, 101 MHz)

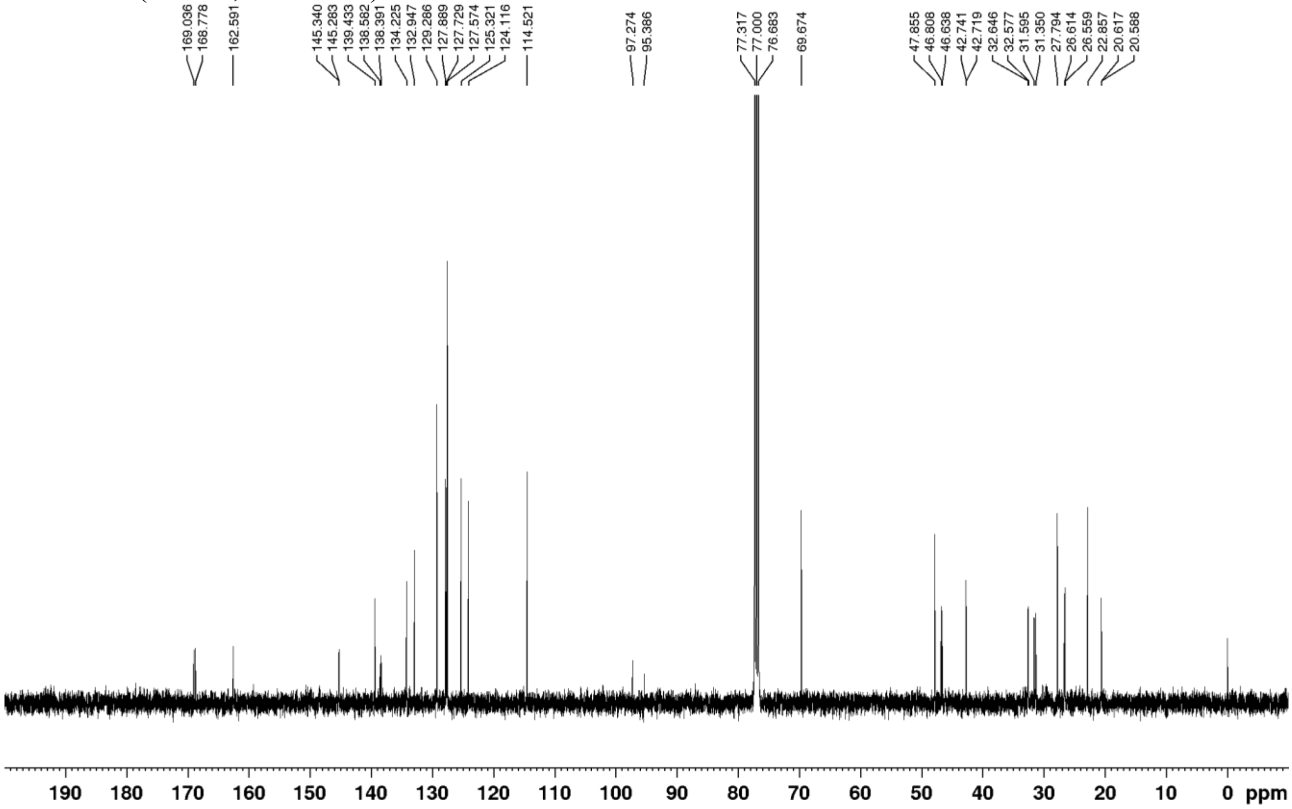

^{19}F NMR ($CDCl_3$, 377 MHz)

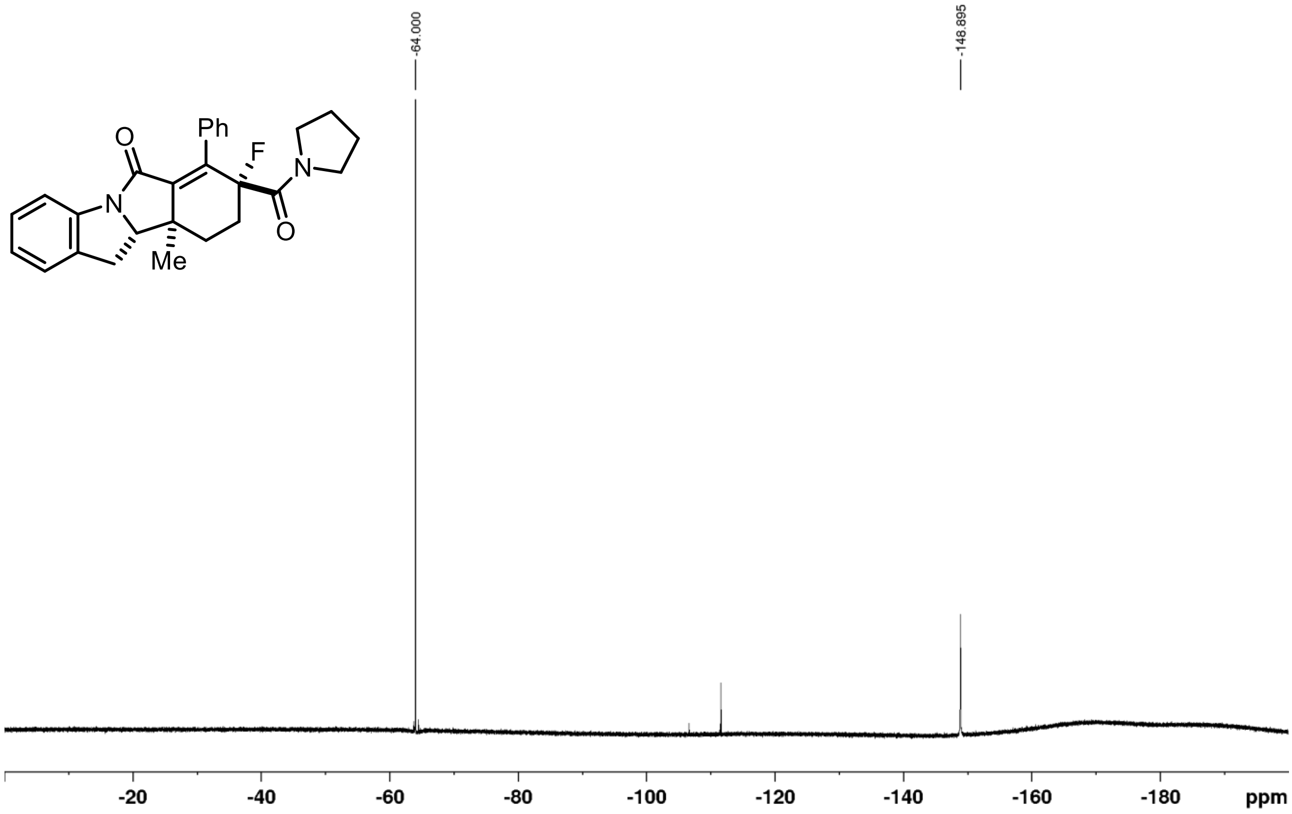

NOESY ($CDCl_3$, 400 MHz)

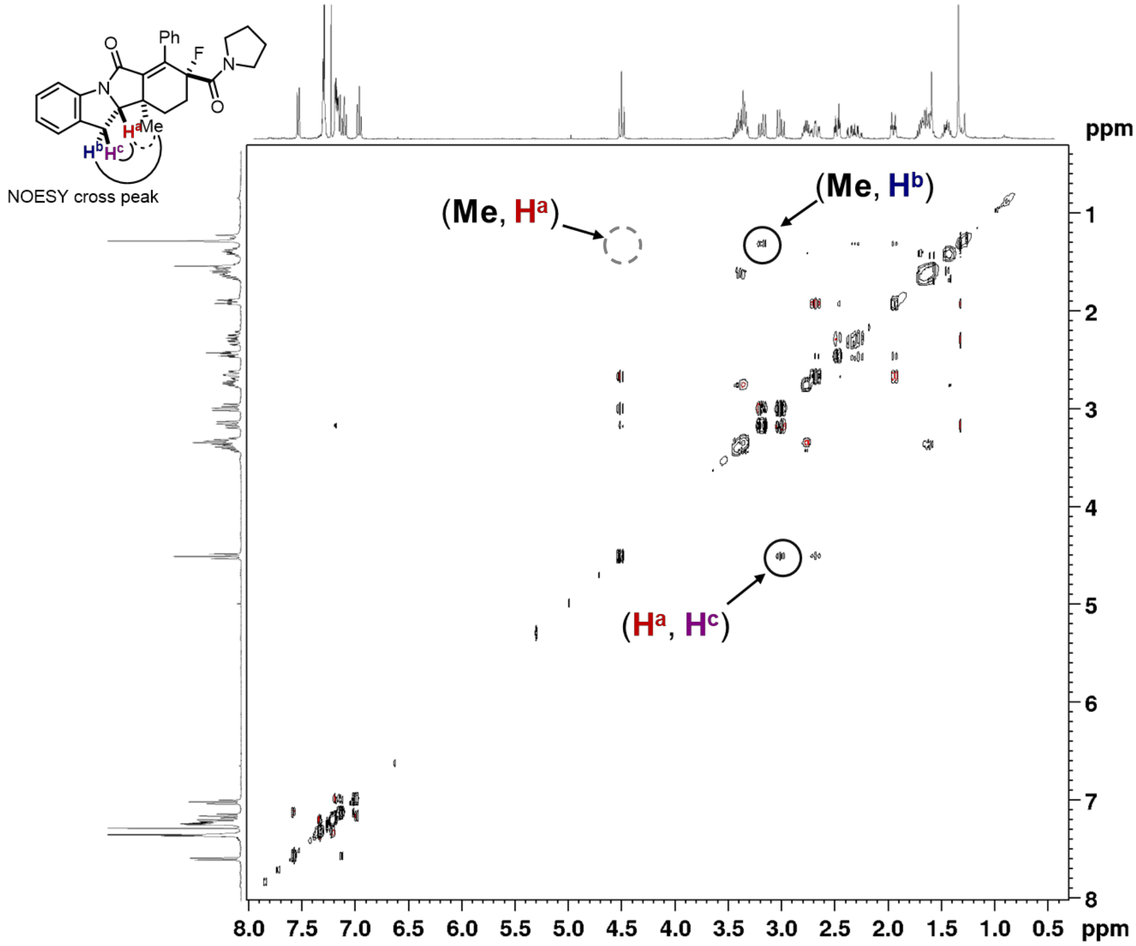

(1*S*,9a*R*)-2-(*Z*)-Benzylidene-1-{(*E*)-3-fluoro-4-oxo-4-(pyrrolidin-1-yl)but-2-en-1-yl}-1-methyl-1,2,9,9a-tetrahydro-3*H*-pyrrolo[1,2-a]indol-3-one (4ka)

^{1}H NMR (CDCl$_3$, 400 MHz)

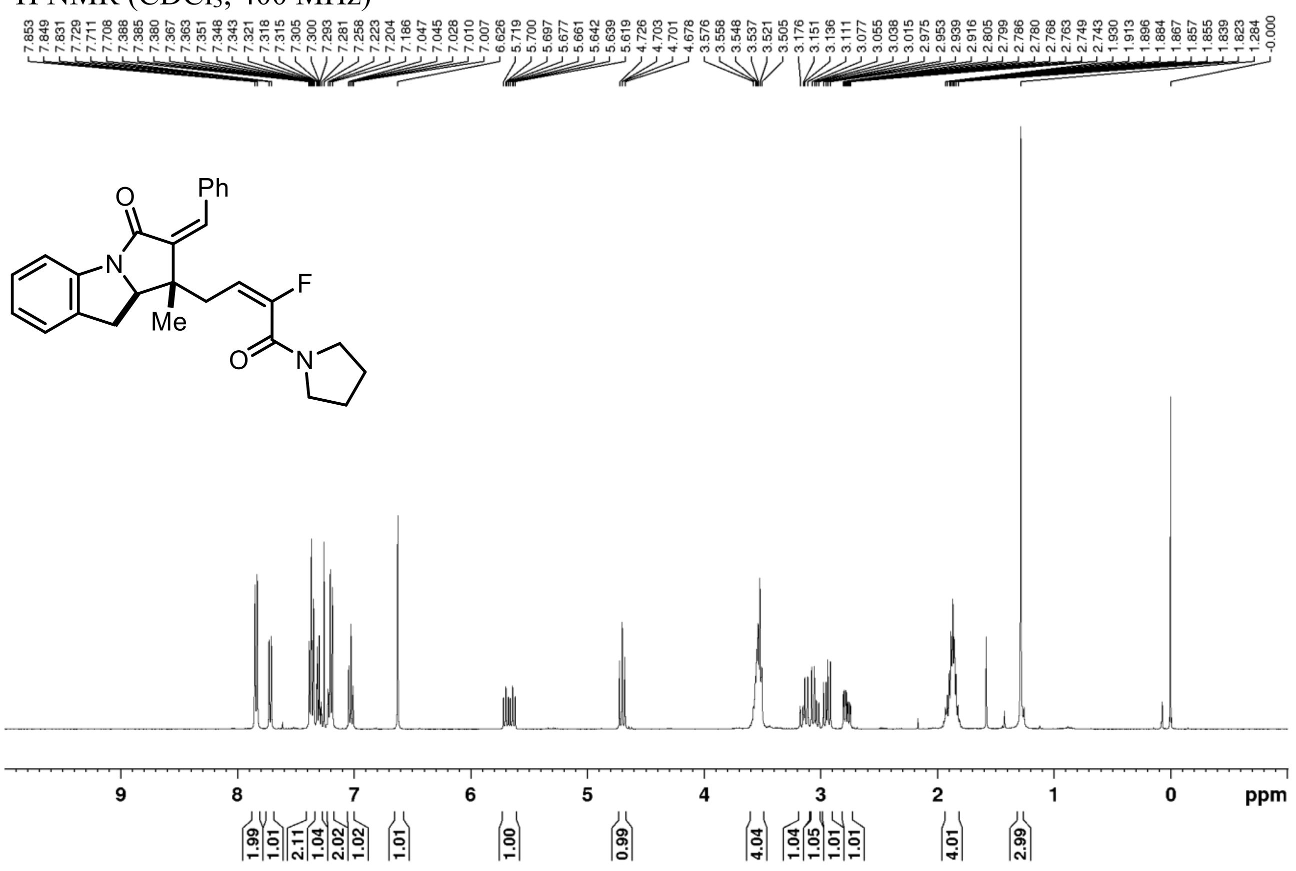

^{13}C NMR (CDCl$_3$, 101 MHz)

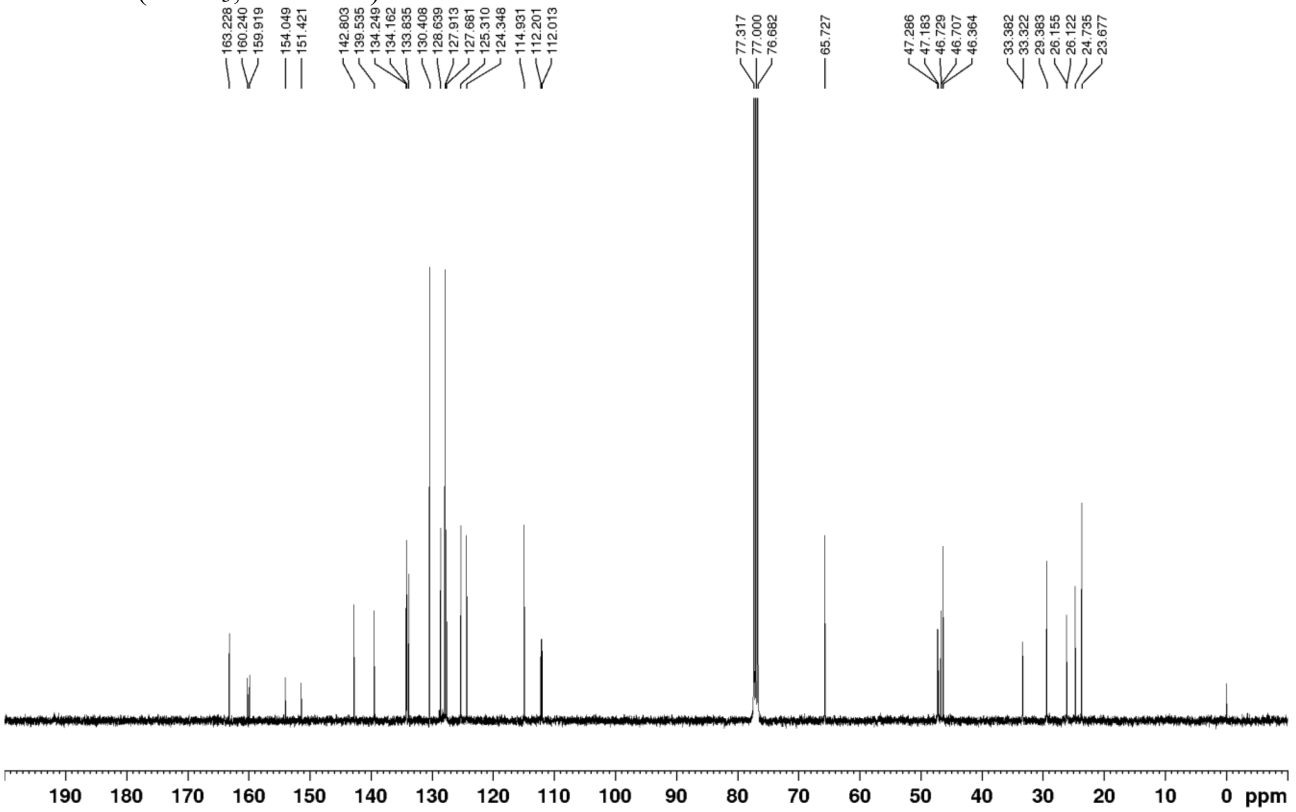

^{19}F NMR ($CDCl_3$, 377 MHz)

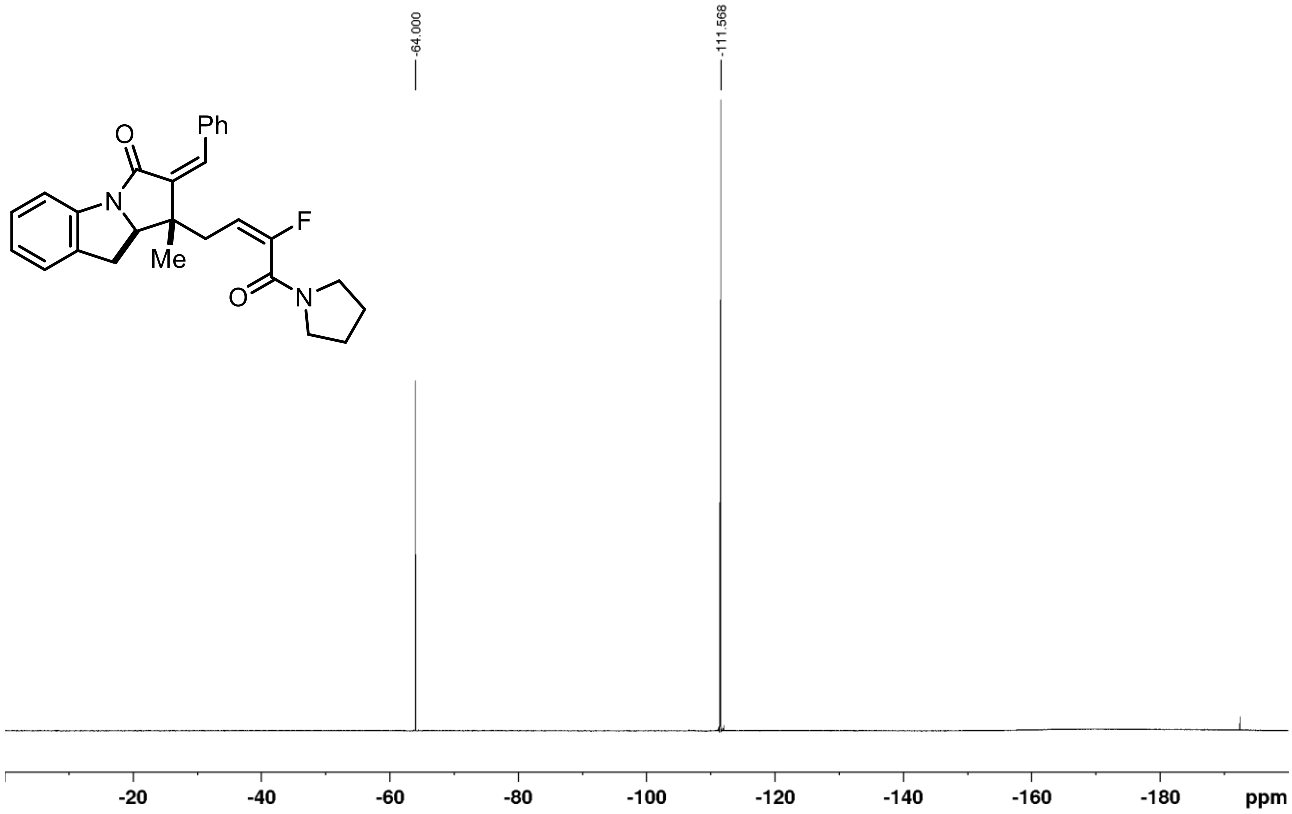

NOESY ($CDCl_3$, 400 MHz)

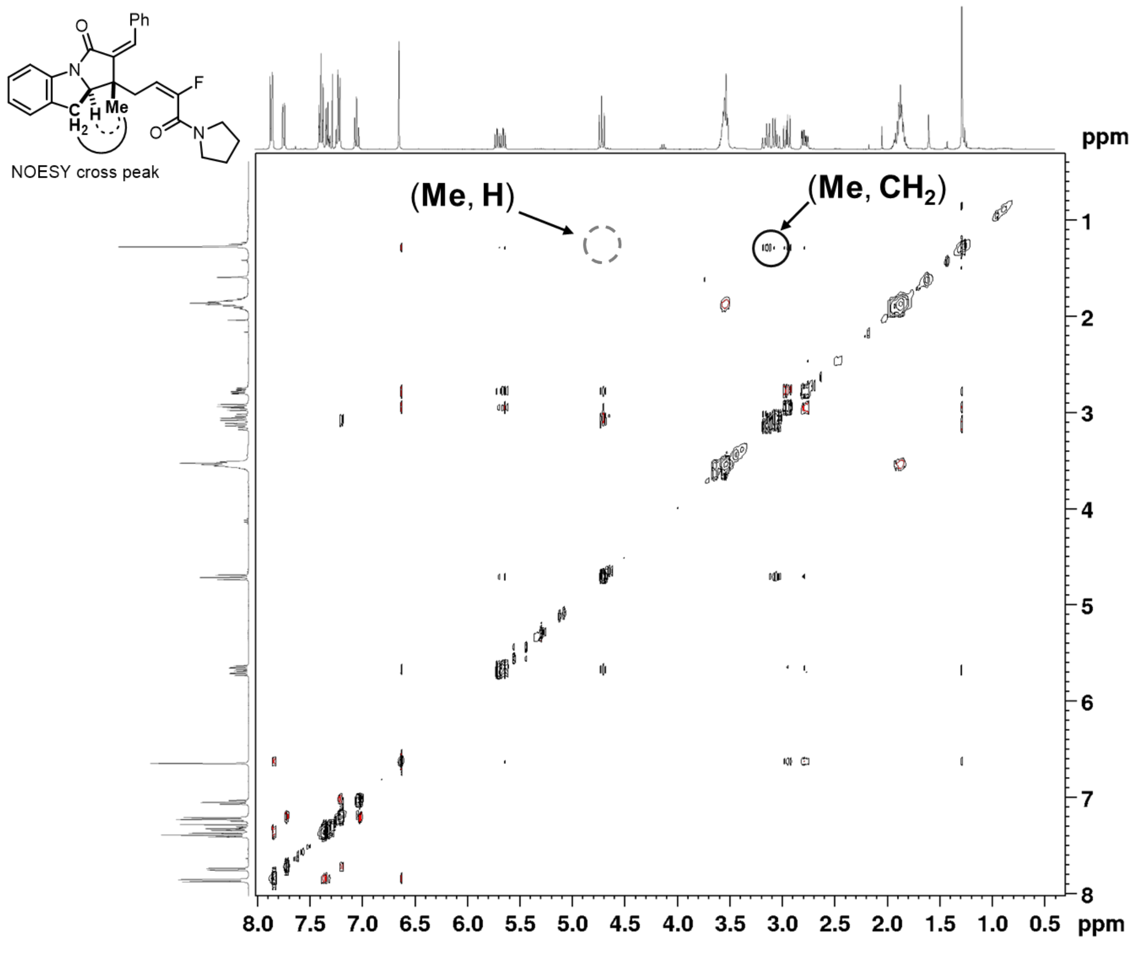

{(1*S*,5*R*,7a*R*)-5-Fluoro-1,7a-dimethyl-4-phenyl-1,3,5,6,7,7a-hexahydroisobenzofuran-5-yl}(pyrrolidin-1-yl)methanone [3la (major diastereomer)]

^{1}H NMR ($CDCl_3$, 400 MHz)

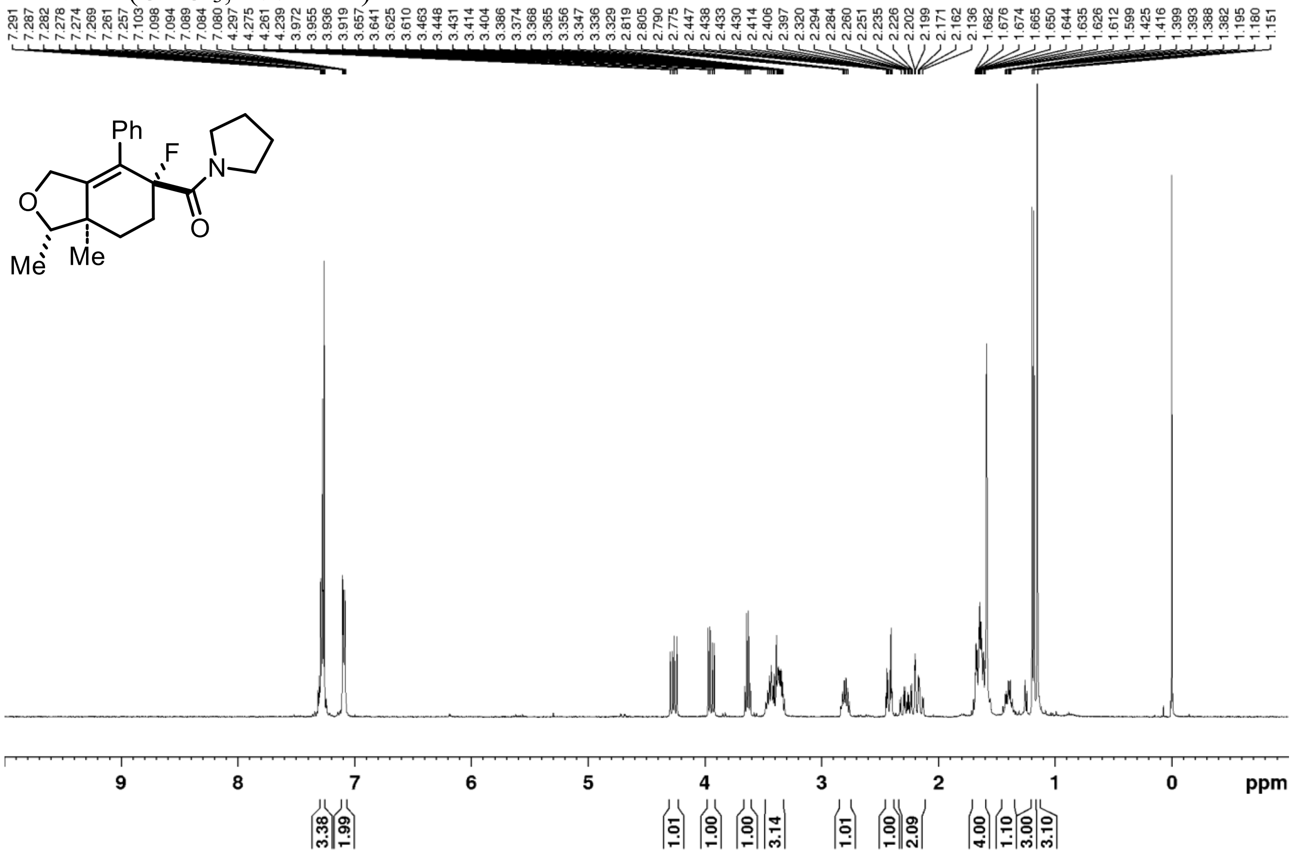

^{13}C NMR ($CDCl_3$, 101 MHz)

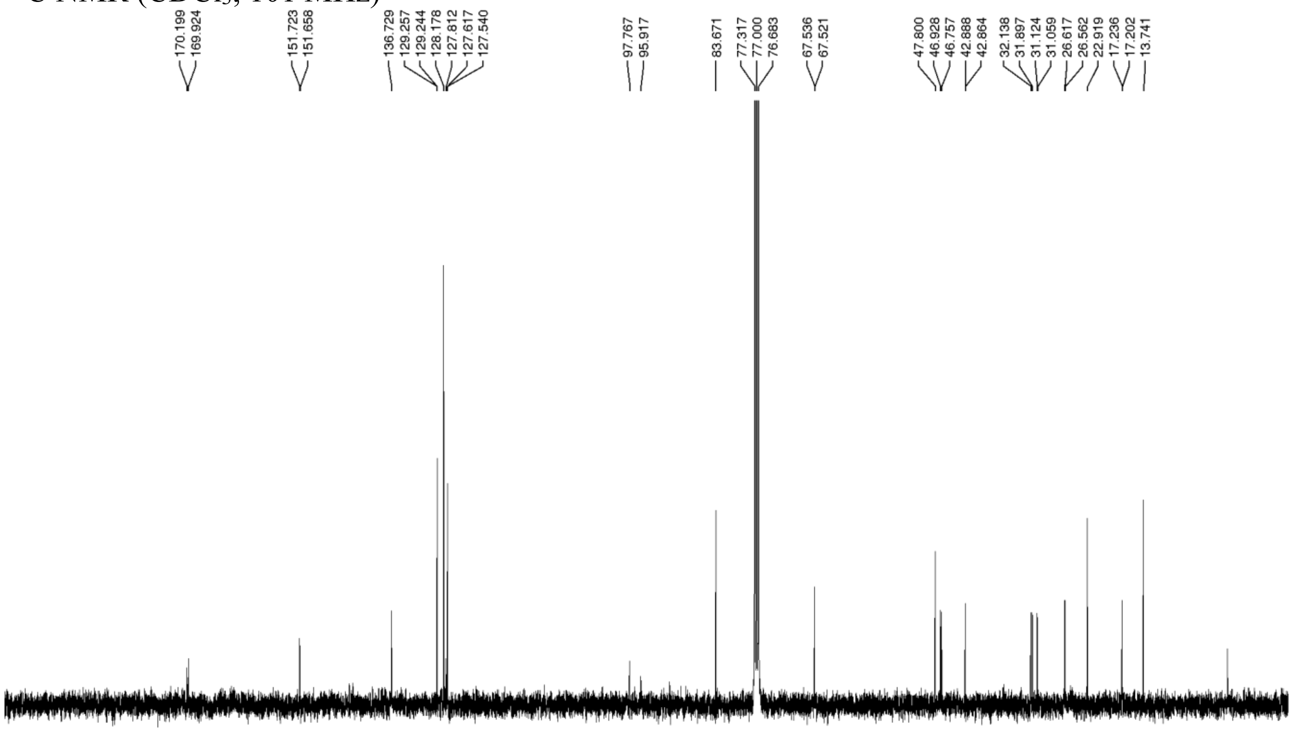

^{19}F NMR ($CDCl_3$, 377 MHz)

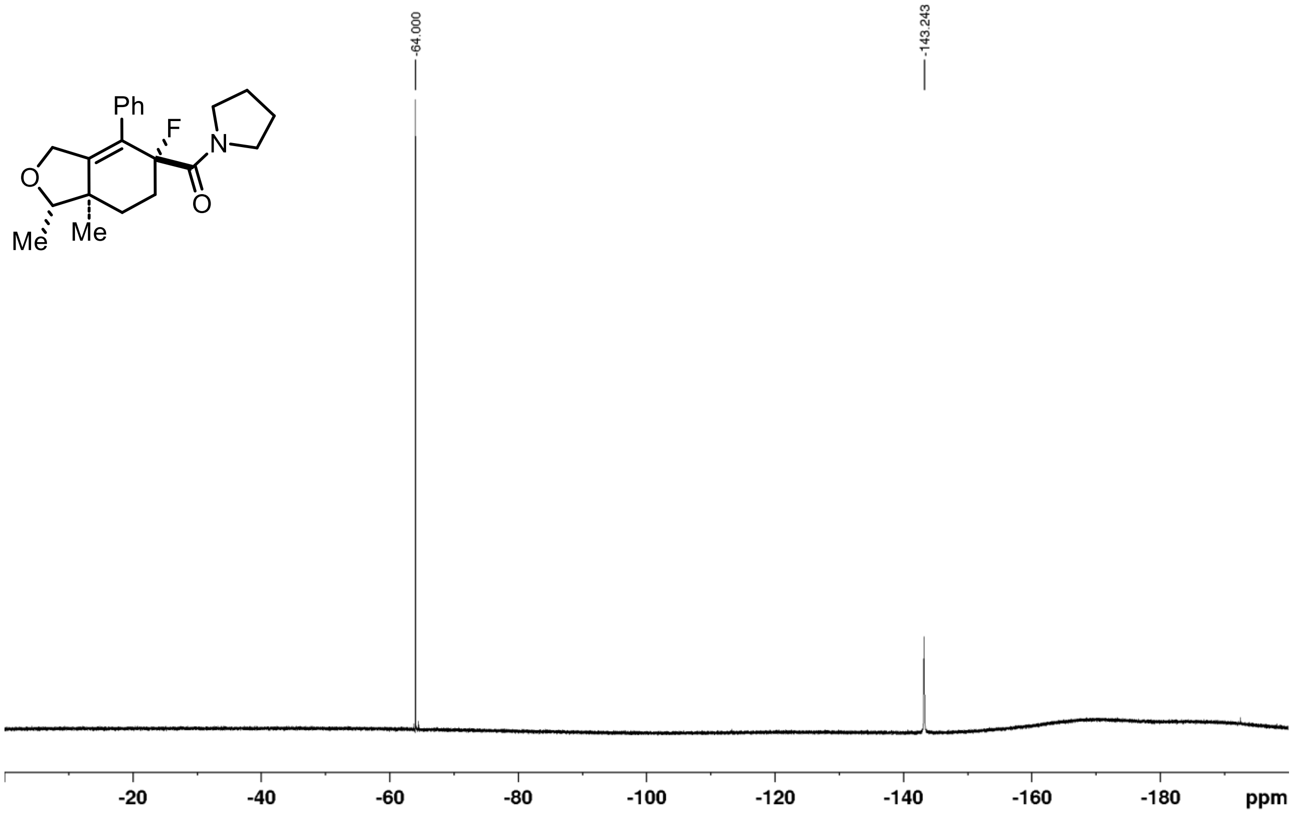

NOESY ($CDCl_3$, 400 MHz)

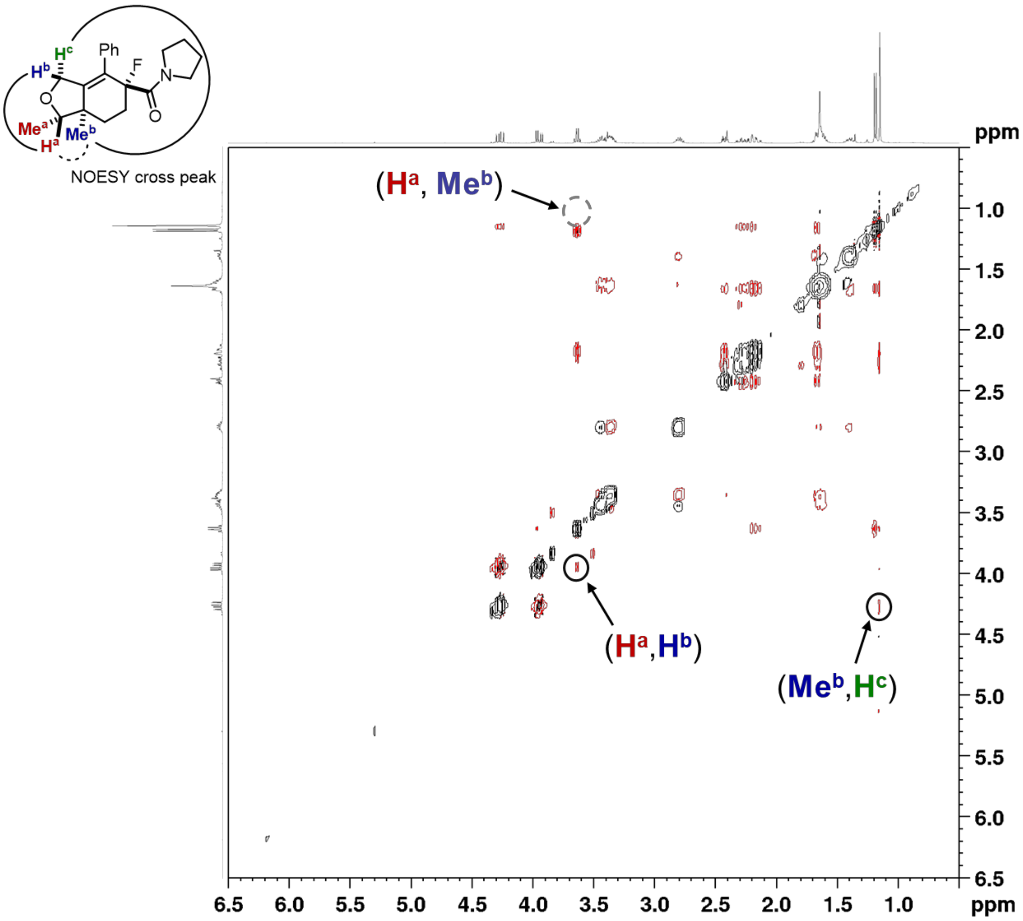

{5-Fluoro-1,7a-dimethyl-4-phenyl-1,3,5,6,7,7a-hexahydroisobenzofuran-5-yl}(pyrrolidin-1-yl)methanone [3la (minor diastereomer)]

^{1}H NMR ($CDCl_3$, 400 MHz)

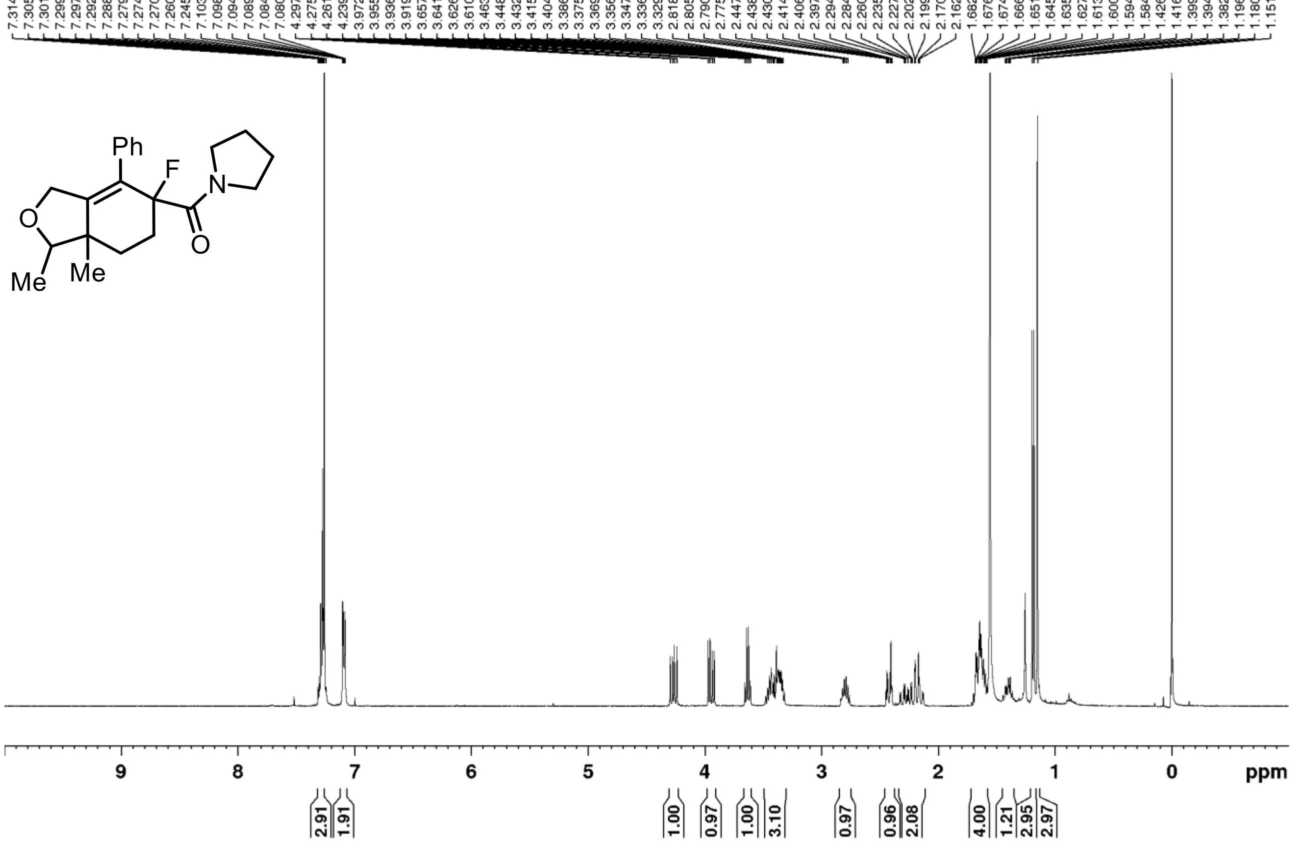

^{19}F NMR ($CDCl_3$, 377 MHz)

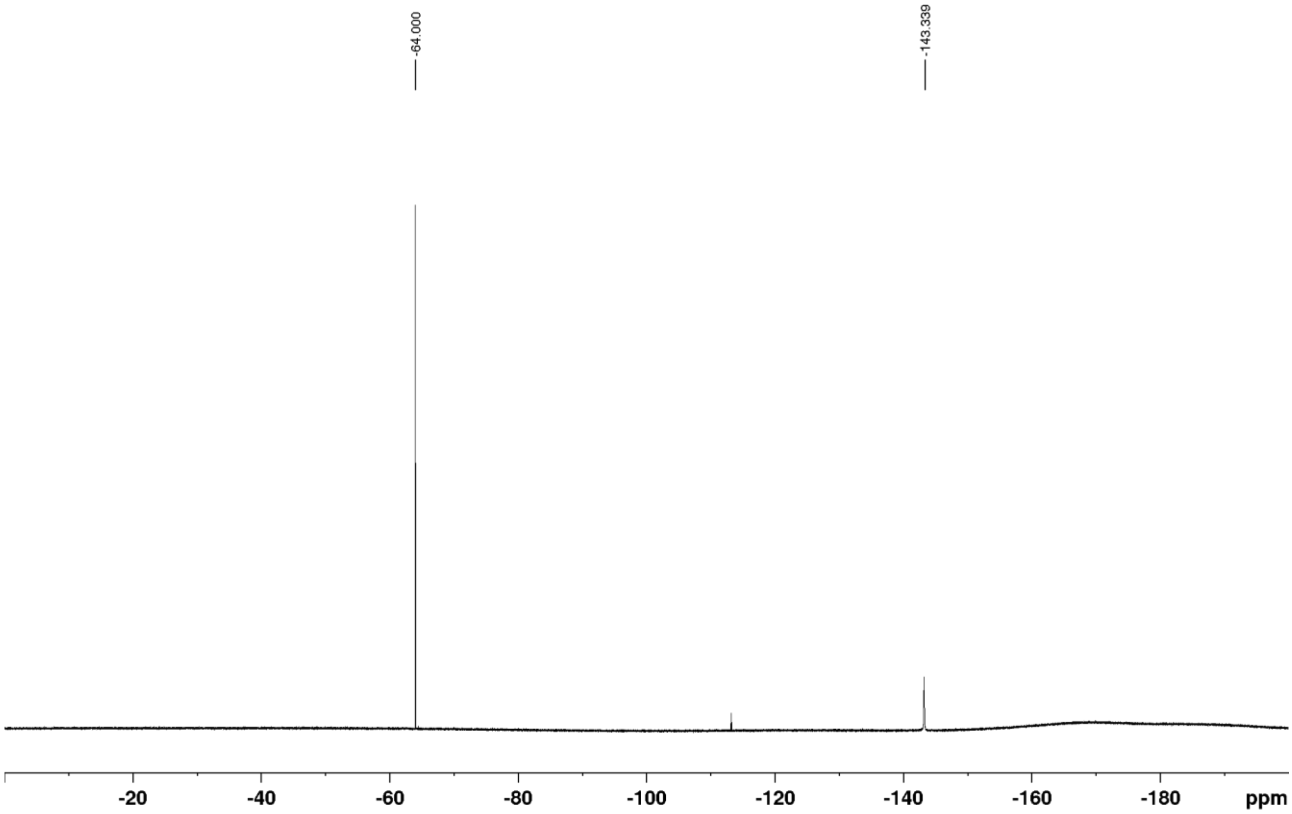

(*E*)-4-{(2*R*,3*S*)-4-(*Z*)-Benzylidene-2,3-dimethyltetrahydrofuran-3-yl}-2-fluoro-1-(pyrrolidin-1-yl)but-2-en-1-one [4la (major diastereomer)]

^{1}H NMR ($CDCl_3$, 400 MHz)

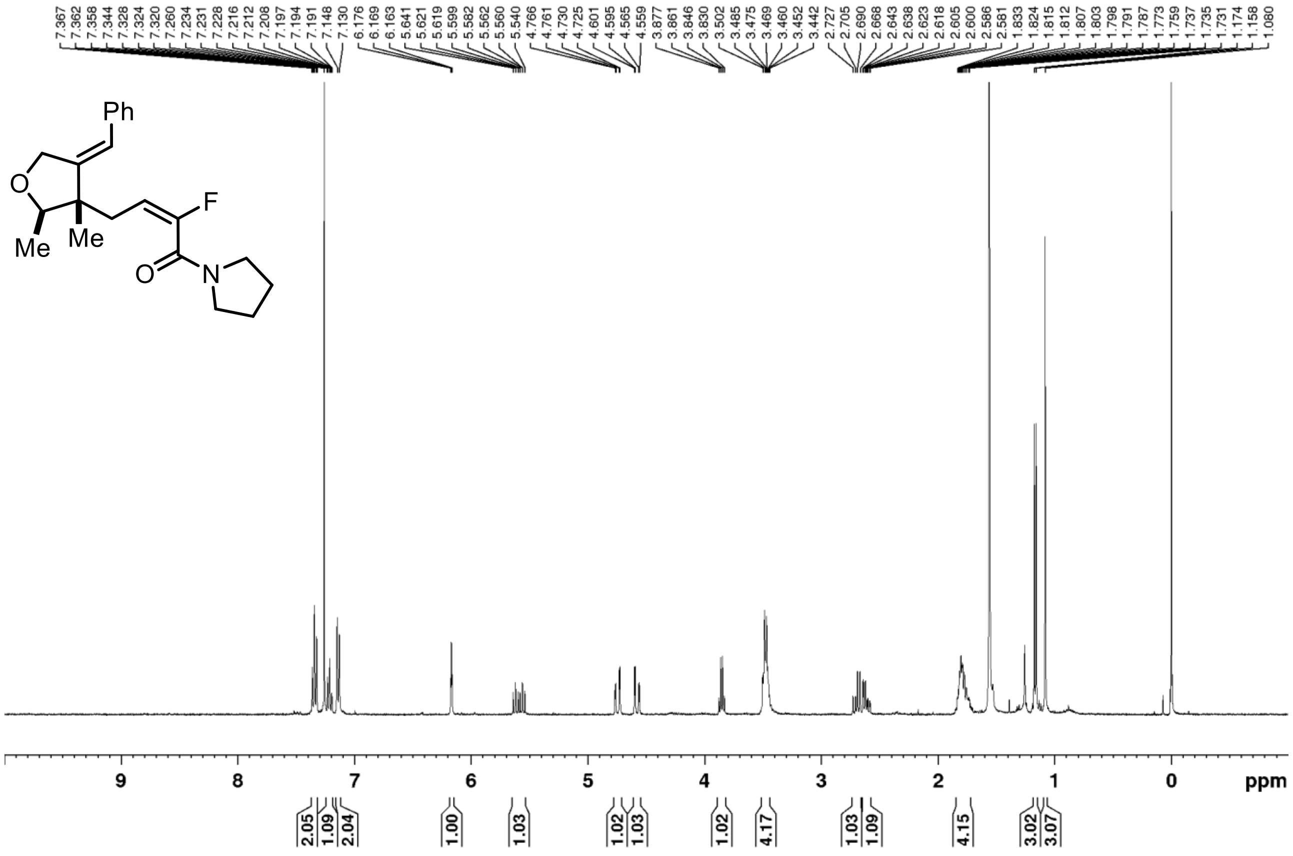

^{13}C NMR ($CDCl_3$, 101 MHz)

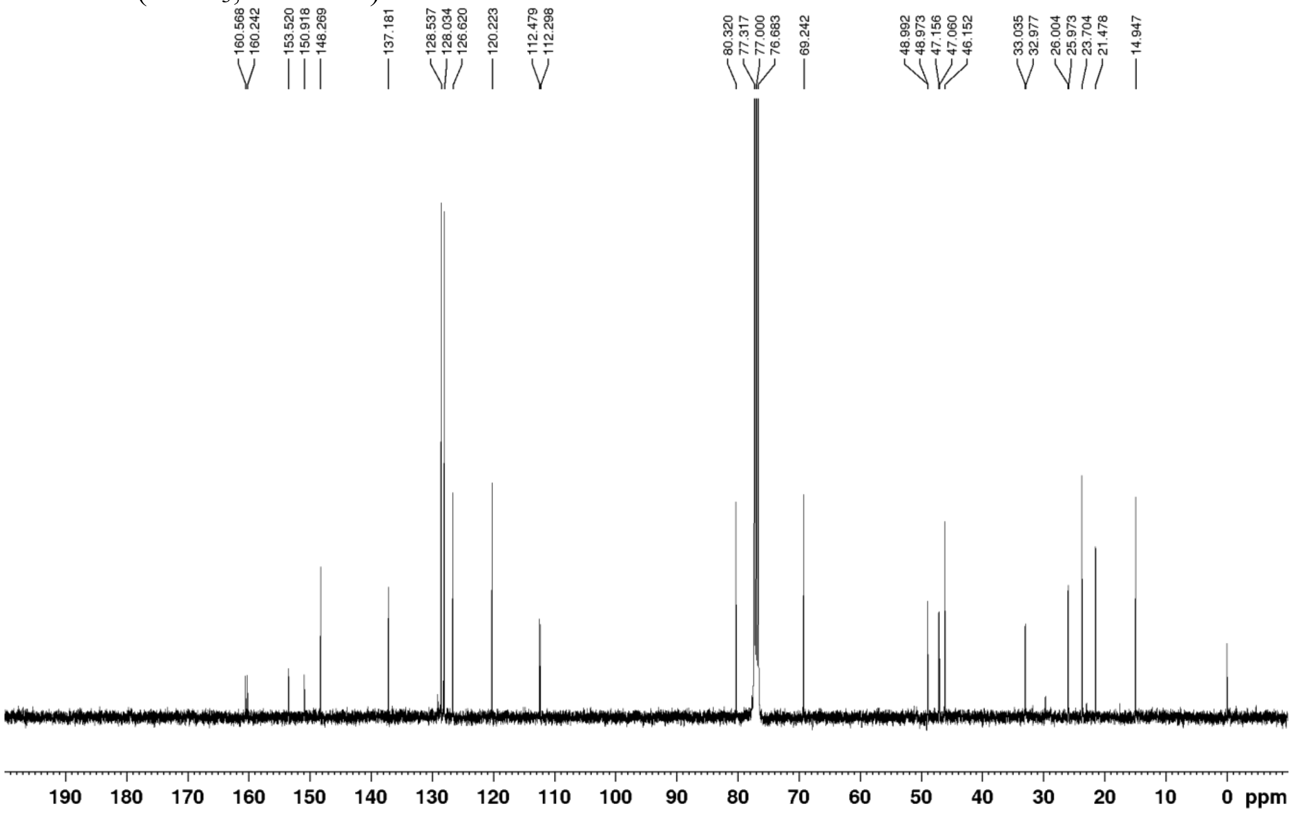

^{19}F NMR (CDCl$_3$, 377 MHz)

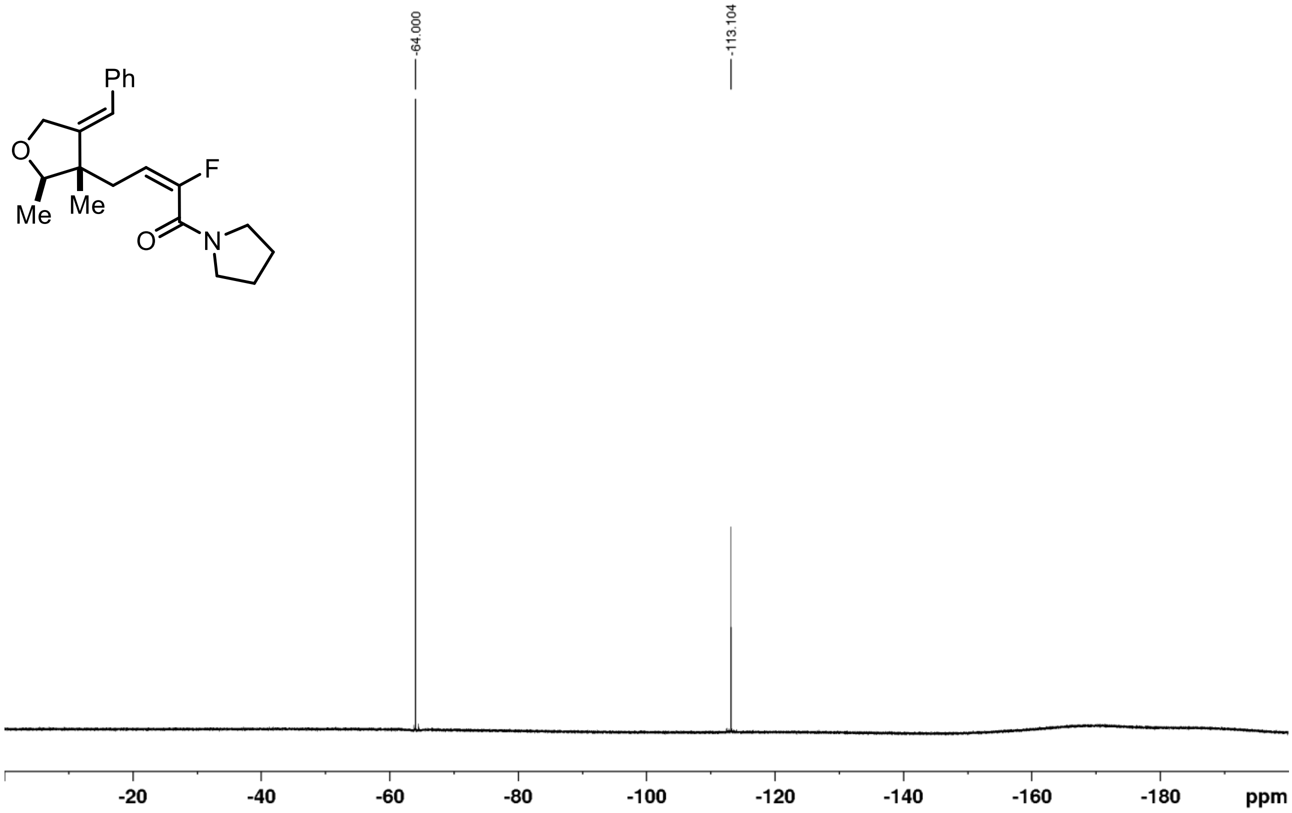

NOESY (CDCl$_3$, 400 MHz)

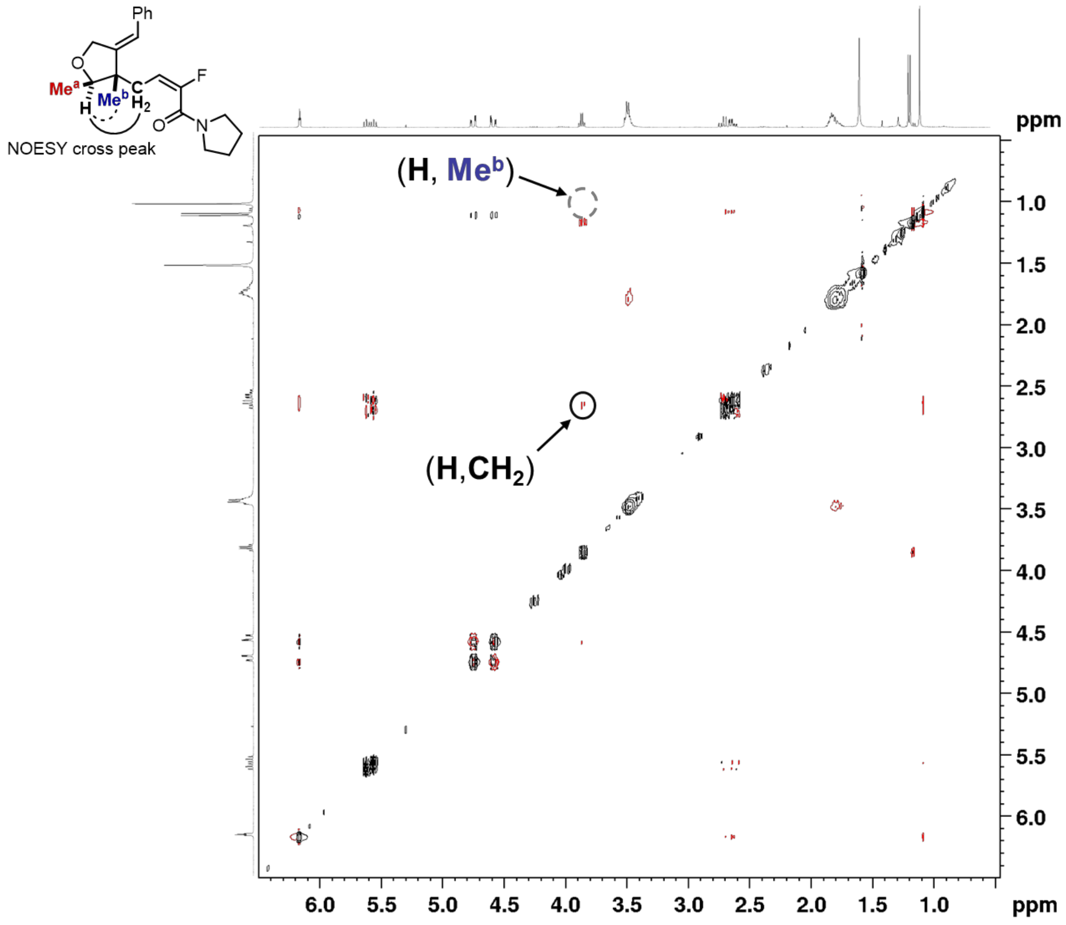

(3*S*,3a*S*,6*R*)-6-Fluoro-3-methyl-3a,7-diphenyl-6-(pyrrolidine-1-carbonyl)-3a,4,5,6-tetrahydroisobenzofuran-1(3*H*)-one (3ma)

^{1}H NMR (CDCl$_3$, 400 MHz)

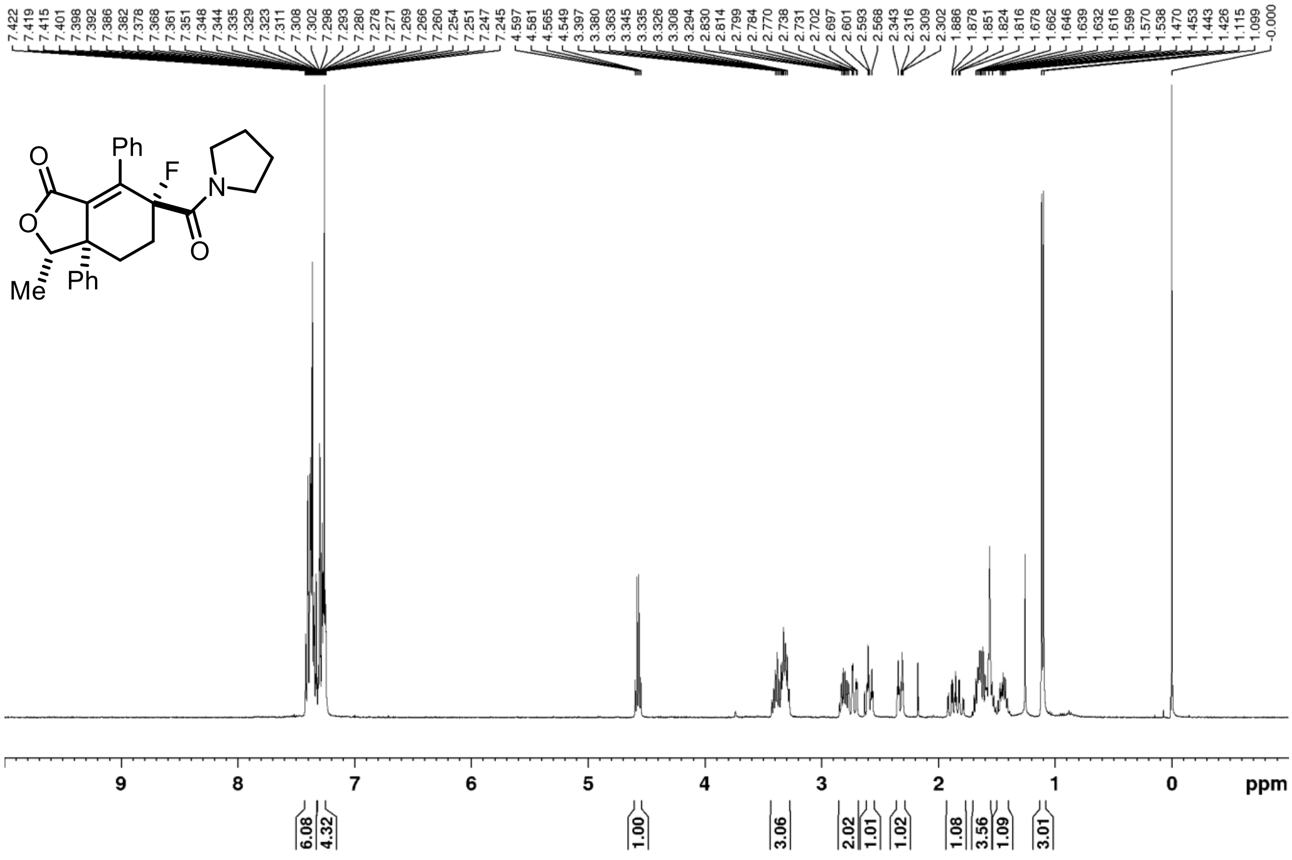

^{13}C NMR (CDCl$_3$, 101 MHz)

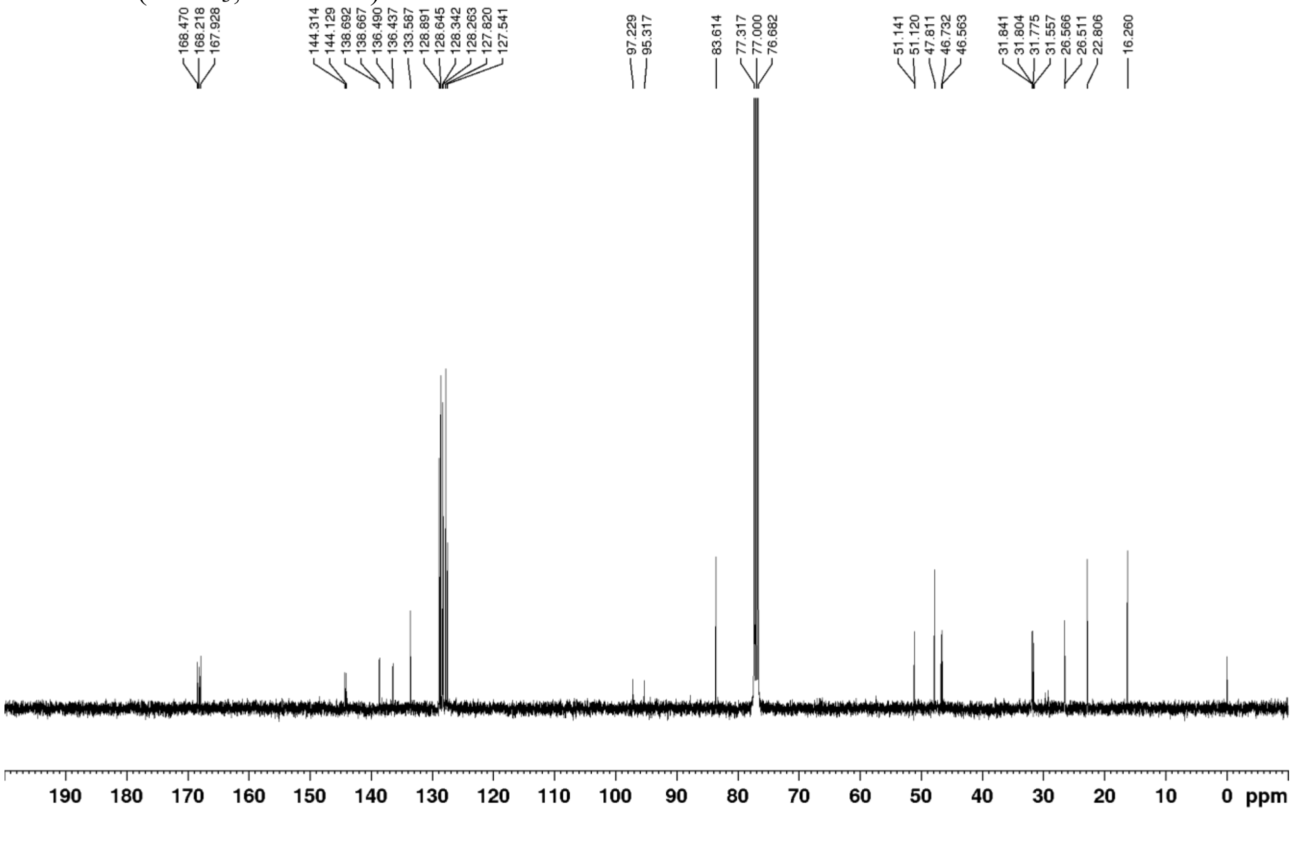

^{19}F NMR (CDCl$_3$, 377 MHz)

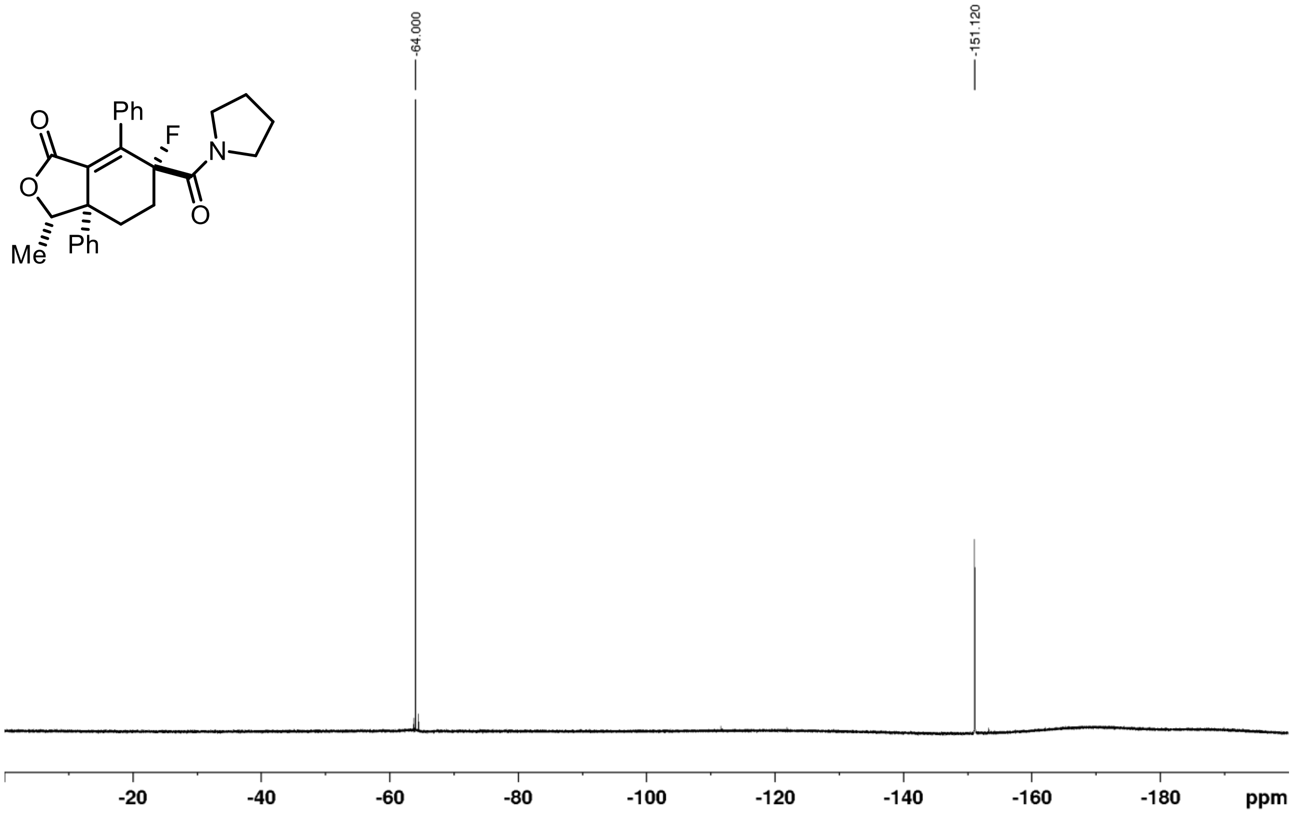

(4*R*,5*R*)-3-(*Z*)-Benzylidene-4-{(*E*)-3-fluoro-4-oxo-4-(pyrrolidin-1-yl)but-2-en-1-yl}-5-methyl-4-phenyldihydrofuran-2(3*H*)-one (4ma)

Diastereomer mixture (80:20 d.r.)

^{1}H NMR (CDCl$_3$, 400 MHz)

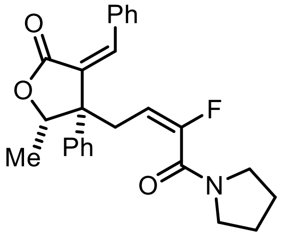

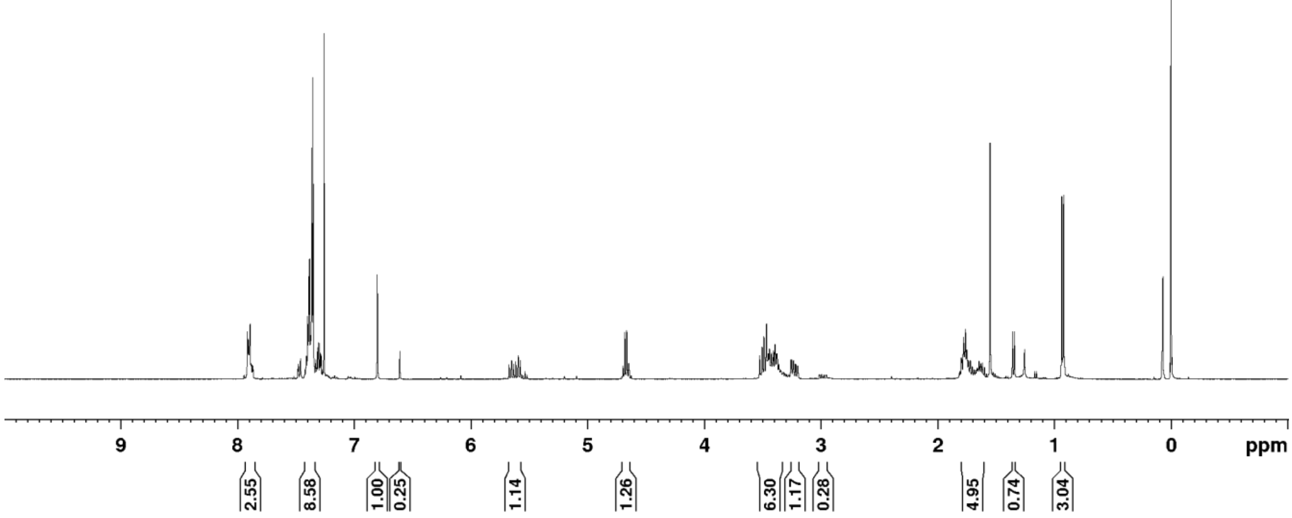

^{13}C NMR (CDCl$_3$, 101 MHz)

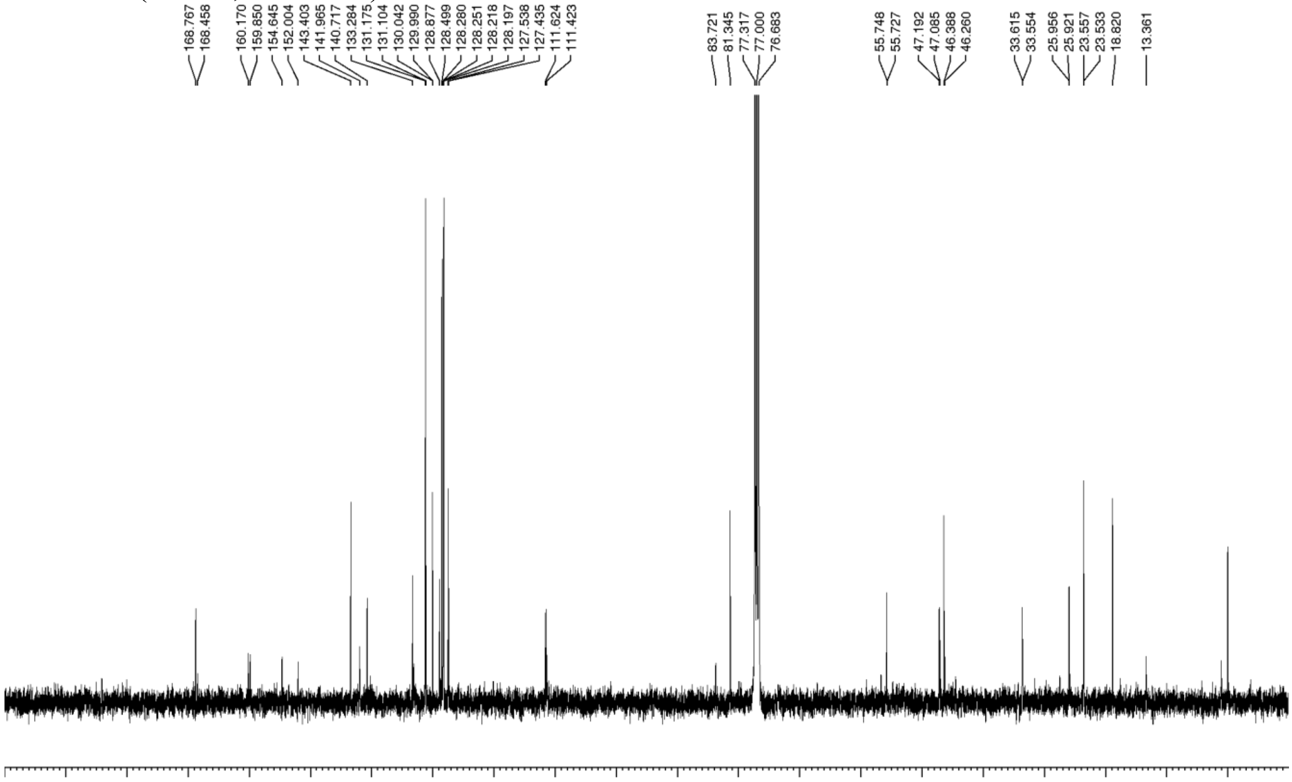

^{19}F NMR ($CDCl_3$, 377 MHz)

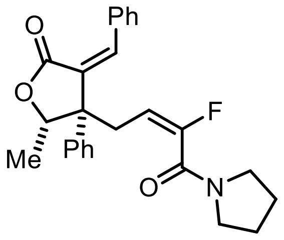

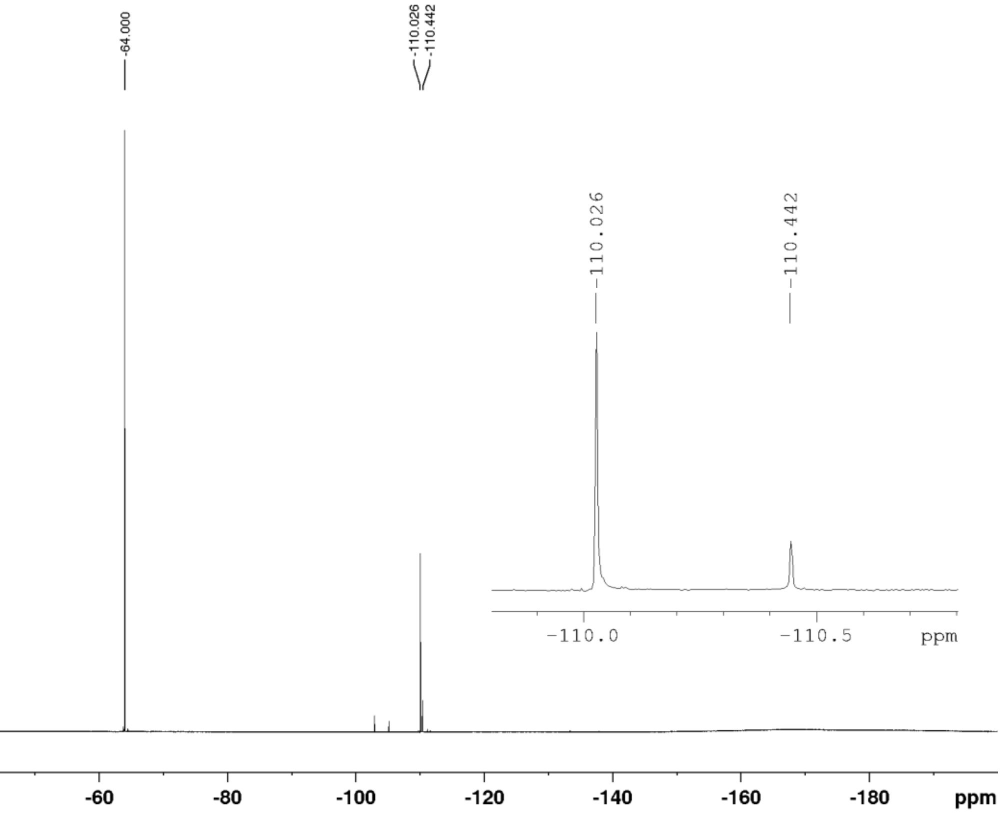

6.3. Rh-Catalyzed Enantioselective PKR (type II) of Racemic 1,6-Enynes with Two Different Acrylamide Derivatives
6.3.1. Substituent Effect at α-Position of Acrylamides (Figure 3a)

Pyrrolidin-1-yl{(5*S*,7a*R*)-1,4,7a-trimethyl-2-(4-methylphenyl)-2,3,5,6,7,7a-hexahydro-1*H*-isoindol-5-yl}methanone (3ad) Diastereomer mixture (58:42 d.r.)

^{1}H NMR (CDCl$_3$, 400 MHz)

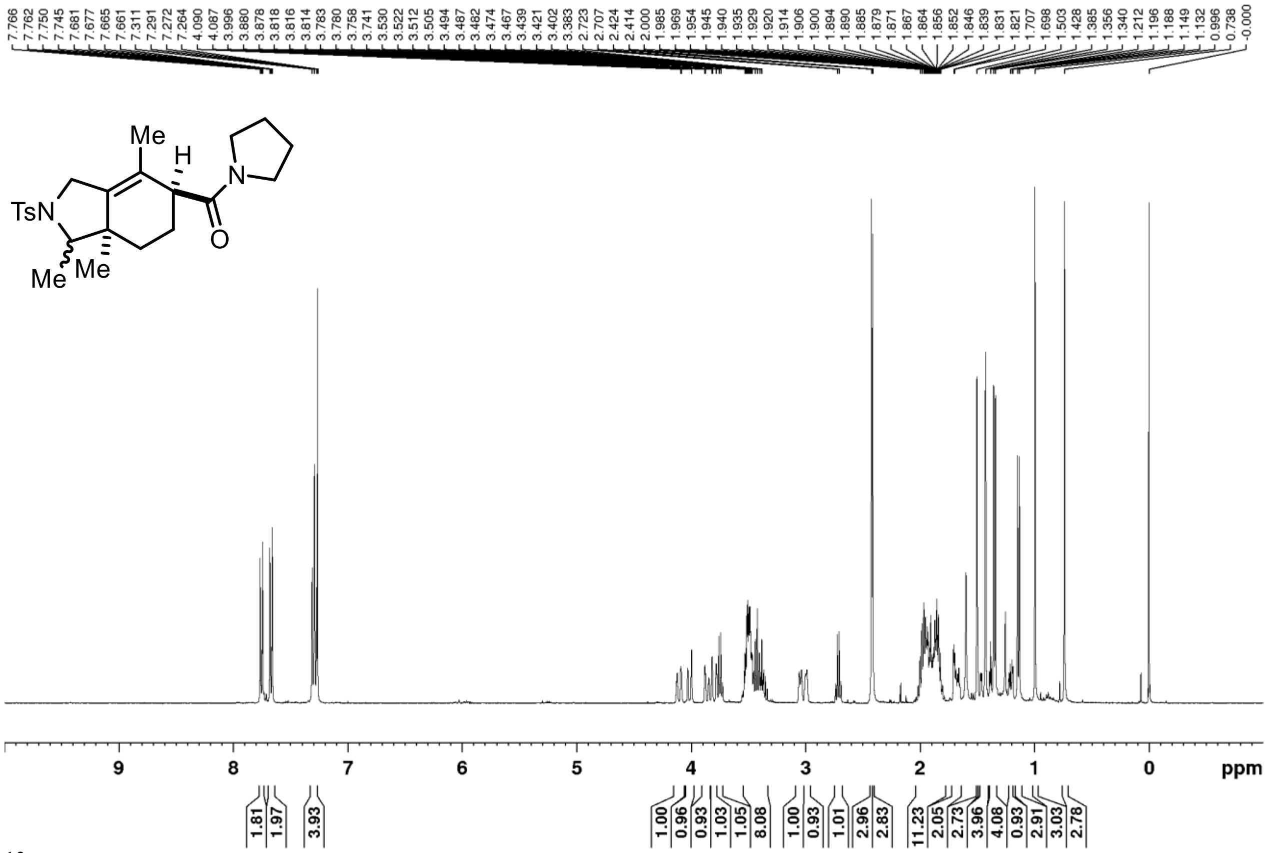

^{13}C NMR (CDCl$_3$, 101 MHz)

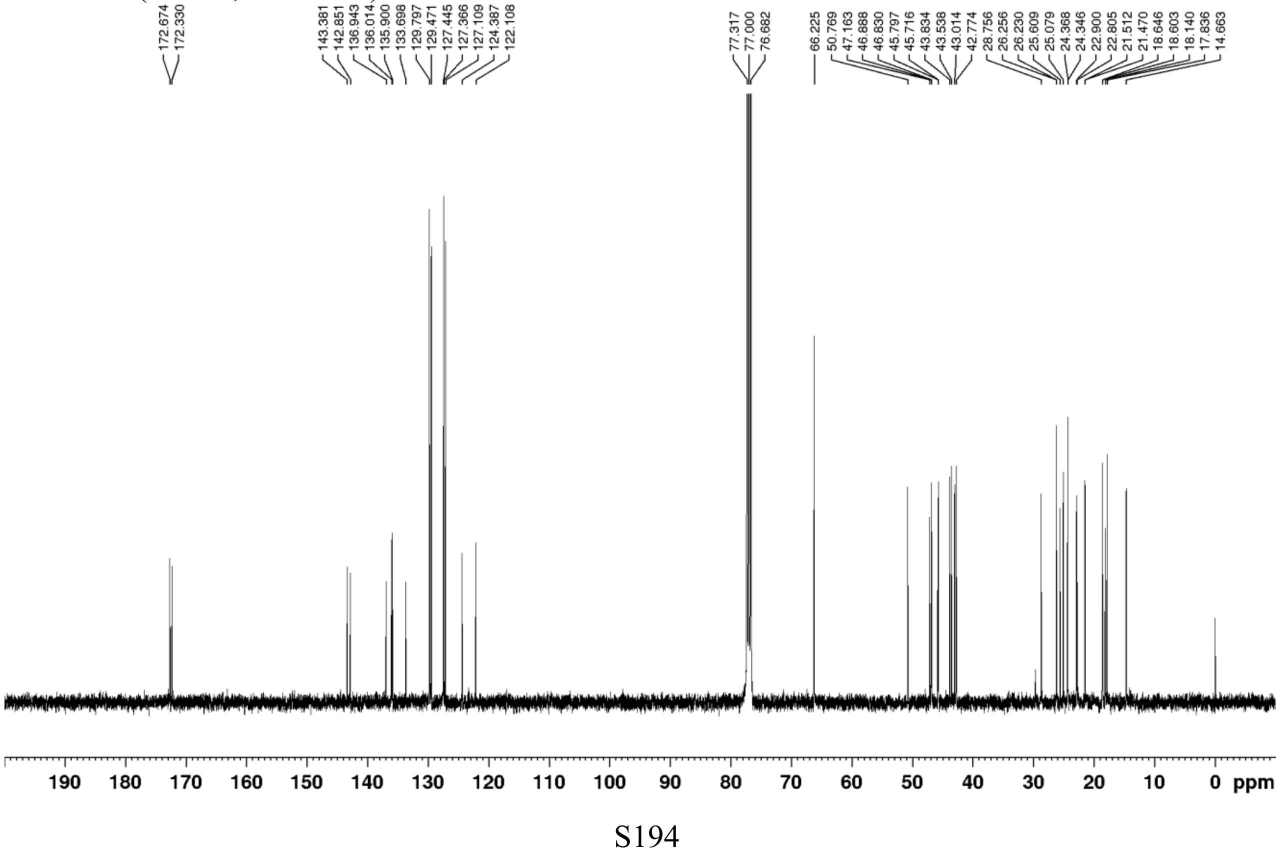

(*Z*)-4-{(3*R*,*Z*)-4-Ethylidene-2,3-dimethyl-1-(4-methylphenyl)pyrrolidin-3-yl}-2-methyl-1-(pyrrolidin-1-yl)but-2-en-1-one (4ae)

Diastereomer mixture (52:48 d.r.)

^{1}H NMR ($CDCl_3$, 400 MHz)

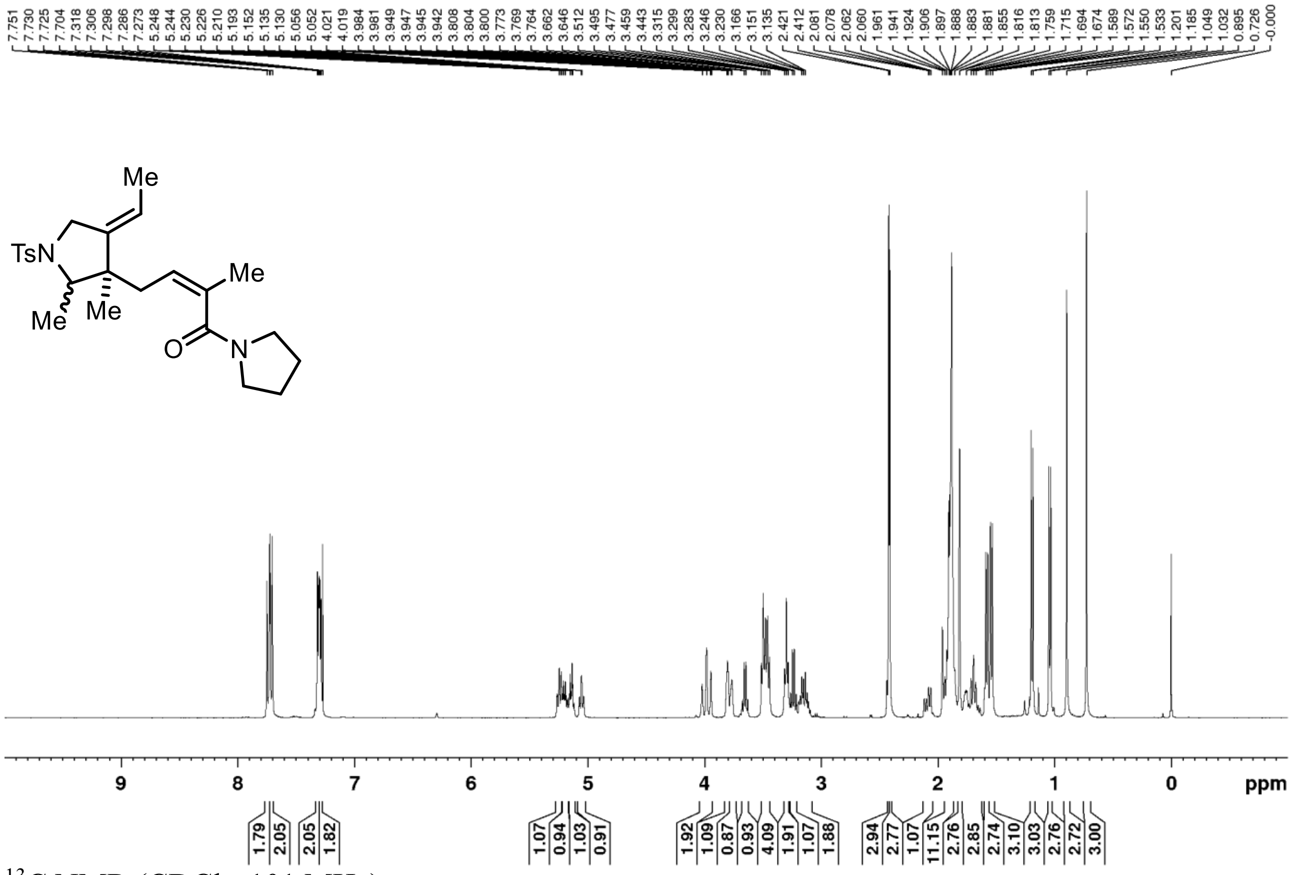

^{13}C NMR ($CDCl_3$, 101 MHz)

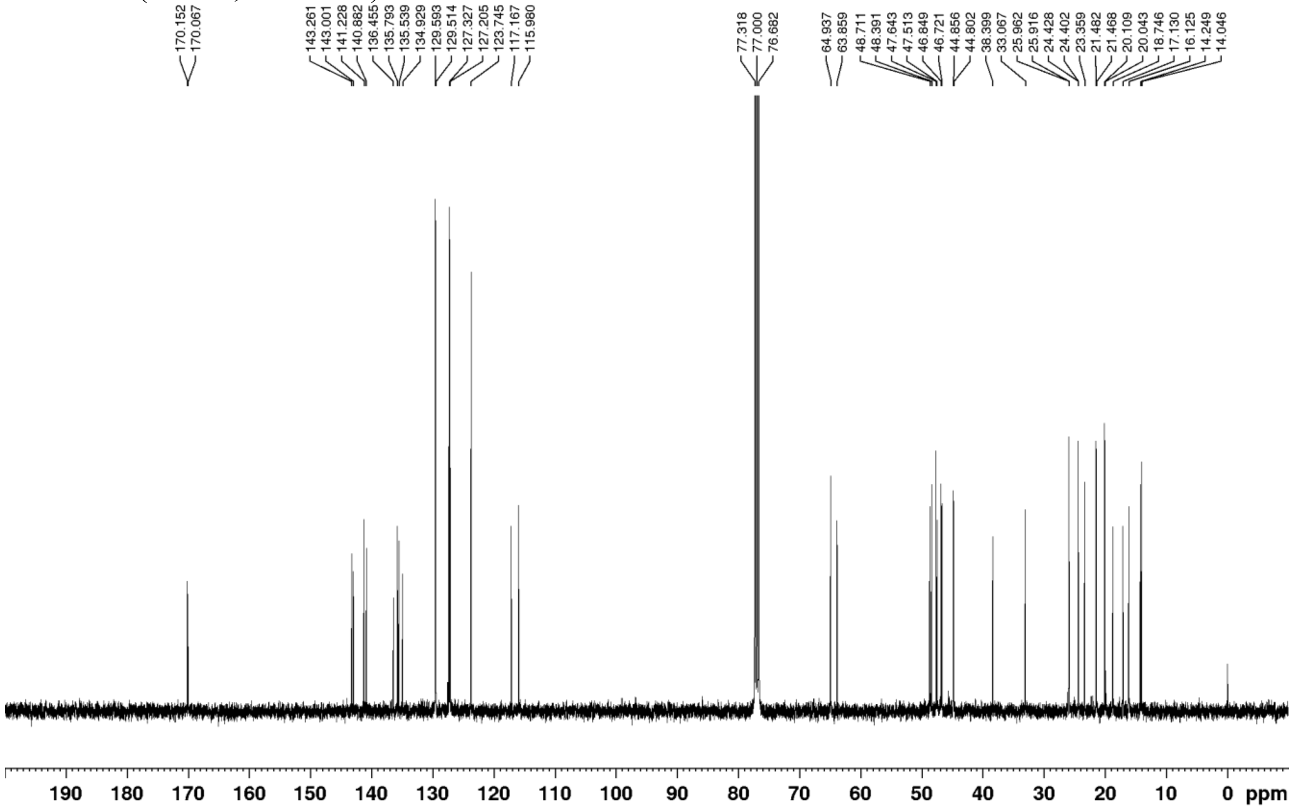

(*E*)-2-Chloro-4-{(3*R*,*Z*)-4-ethylidene-2,3-dimethyl-1-(4-methylphenyl)pyrrolidin-3-yl}-1-(pyrrolidin-1-yl)but-2-en-1-one (4af)

Diastereomer mixture (68:32 d.r.)

^{1}H NMR ($CDCl_3$, 400 MHz)

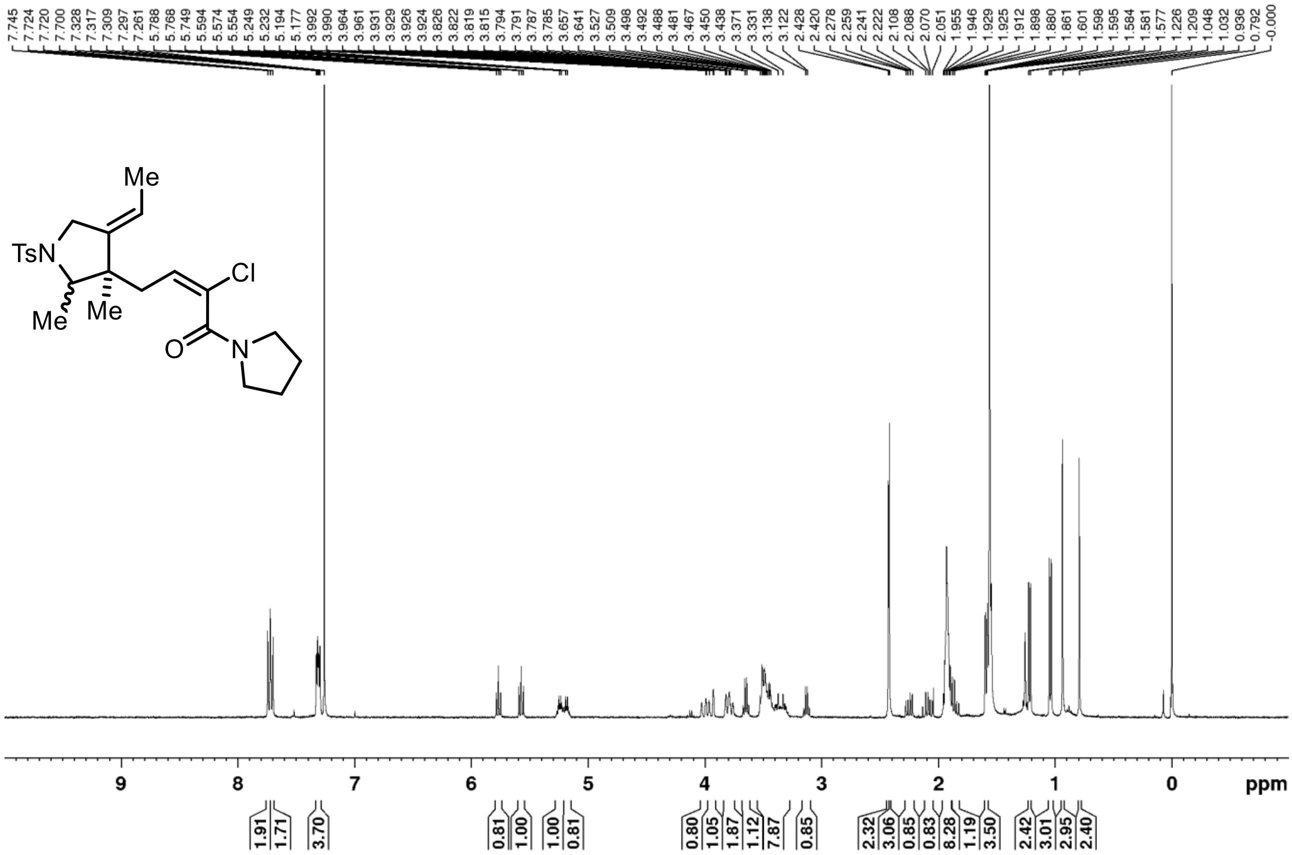

^{13}C NMR ($CDCl_3$, 101 MHz)

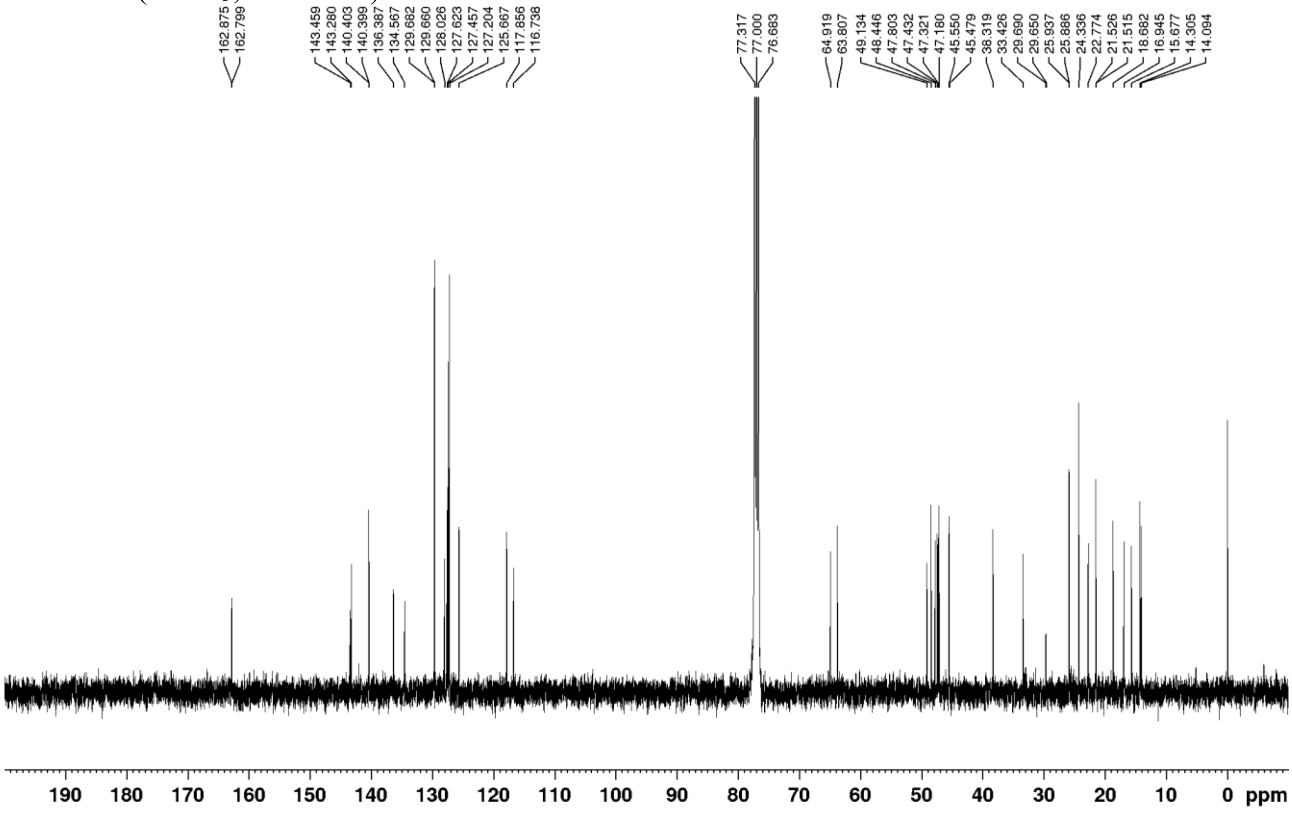

(–)-(1*S*,5*R*,7a*R*)-5-Fluoro-*N*,1,7a-trimethyl-3-oxo-*N*,4-diphenyl-1,3,5,6,7,7a-hexahydroisobenzofuran-5-carboxamide [3hg (major diastereomer)]

^{1}H NMR (CDCl$_3$, 400 MHz)

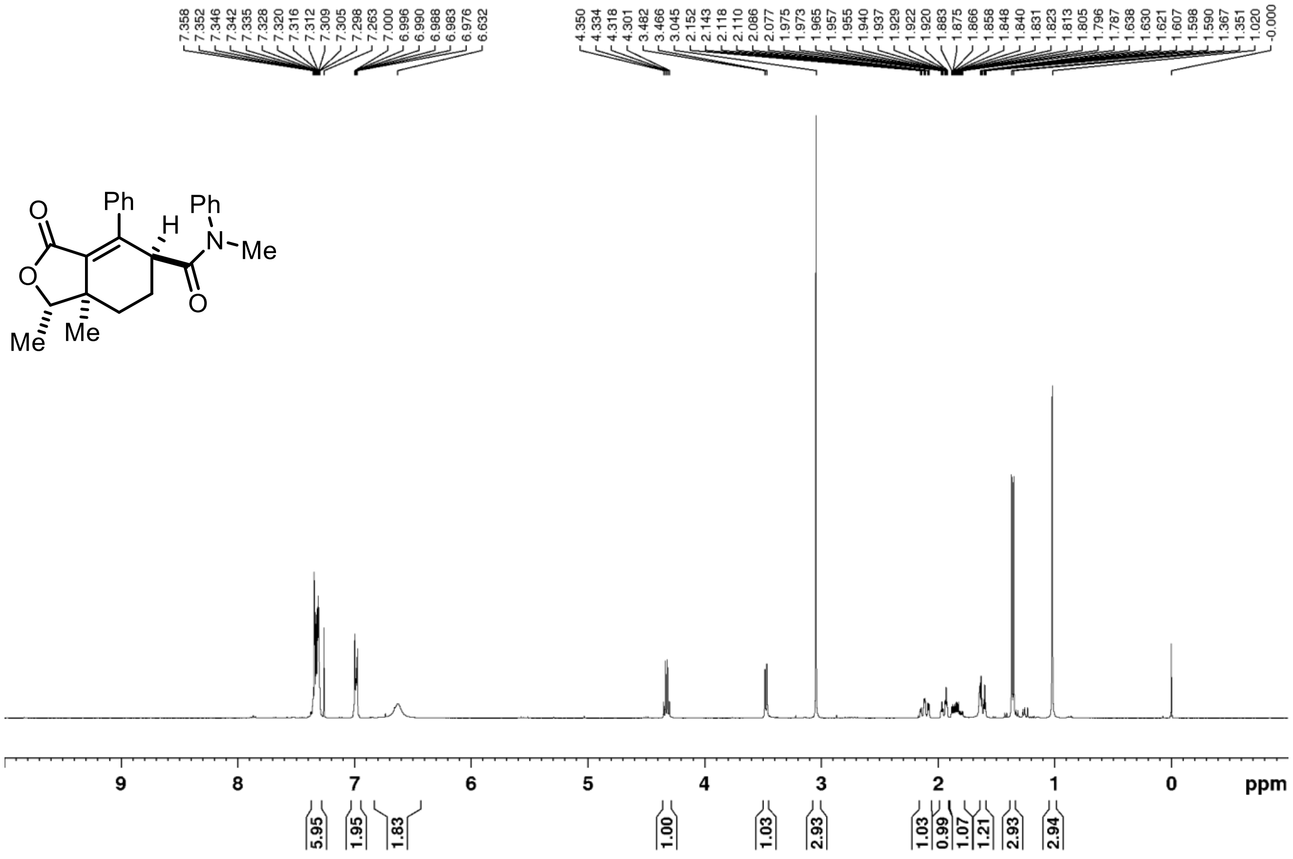

^{13}C NMR (CDCl$_3$, 101 MHz)

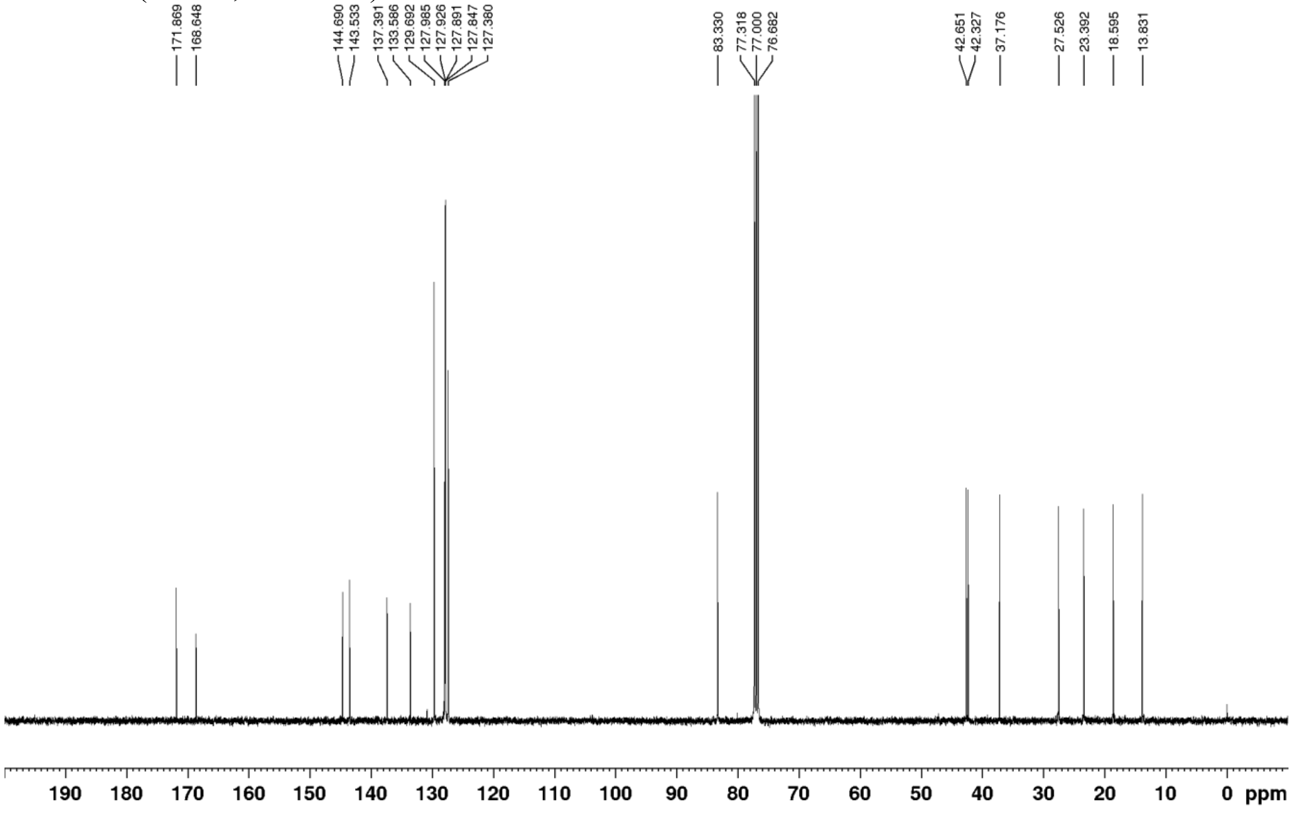

(–)-(4*S*,5*R*)-3-{(*Z*)-Benzylidene}-4,5-dimethyl-4-[(*Z*)-3-methyl-4-oxo-4-{pyrrolidin-1-yl}but-2-en-1-yl]dihydrofuran-2(3*H*)-one (4he)

^{1}H NMR ($CDCl_3$, 400 MHz)

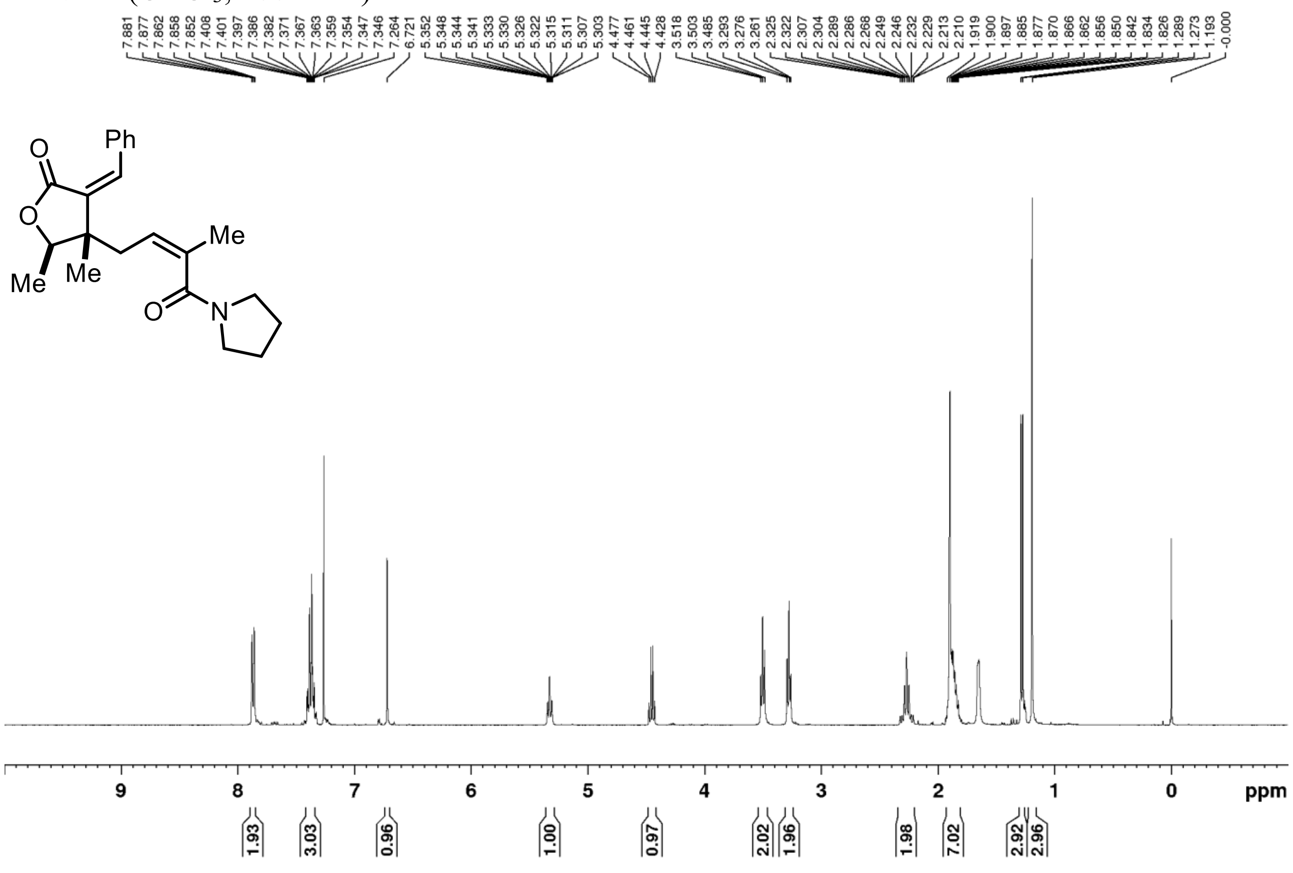

^{13}C NMR ($CDCl_3$, 101 MHz)

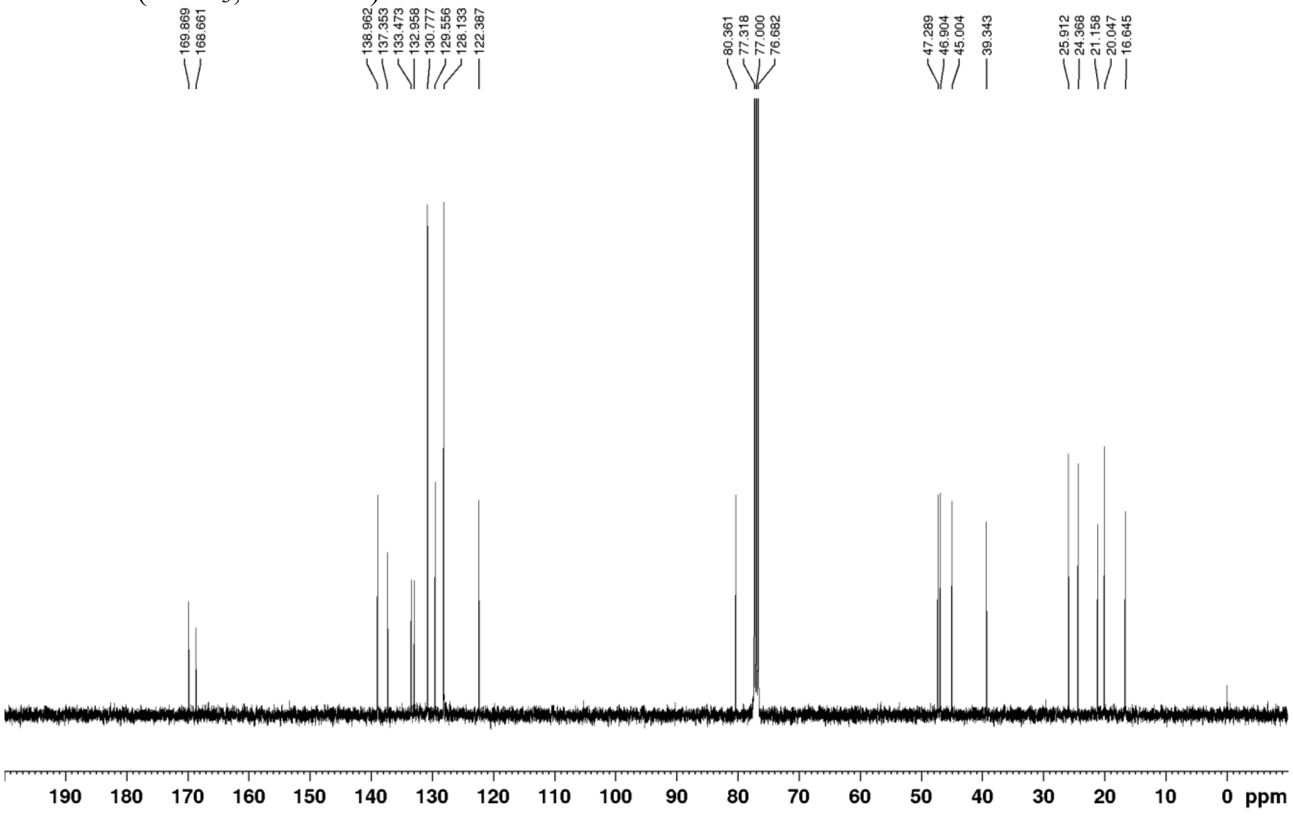

6.3.2. PKR (type II) Using Two Different Acrylamide Derivatives (Figure 3b)

(–)-(1*S*,5*S*,7a*R*)-4-(4-Bromophenyl)-*N*,1,7a-trimethyl-3-oxo-*N*-phenyl-1,3,5,6,7,7a-hexahydroisobenzofuran-5-carboxamide [3ig (major diastereomer)]

^{1}H NMR ($CDCl_3$, 400 MHz)

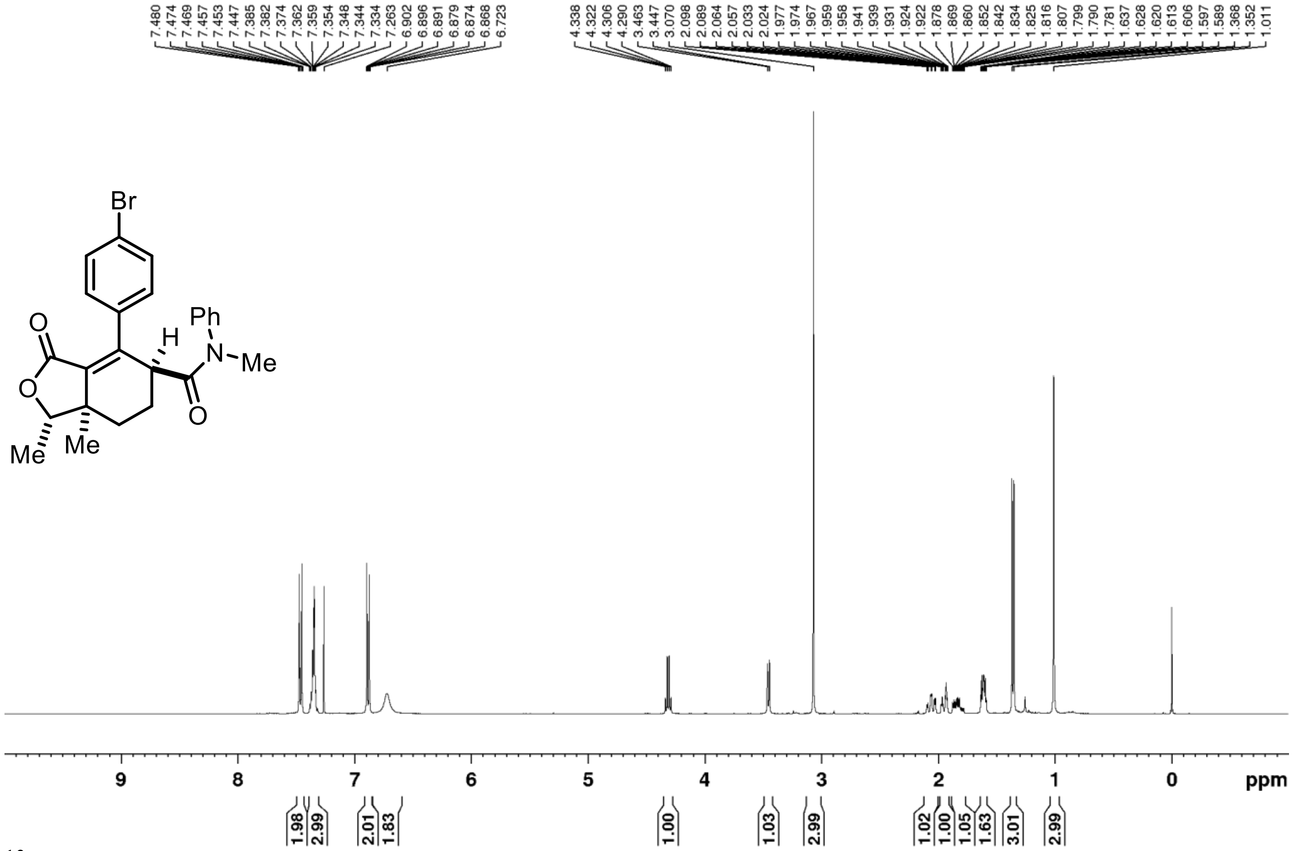

^{13}C NMR ($CDCl_3$, 101 MHz)

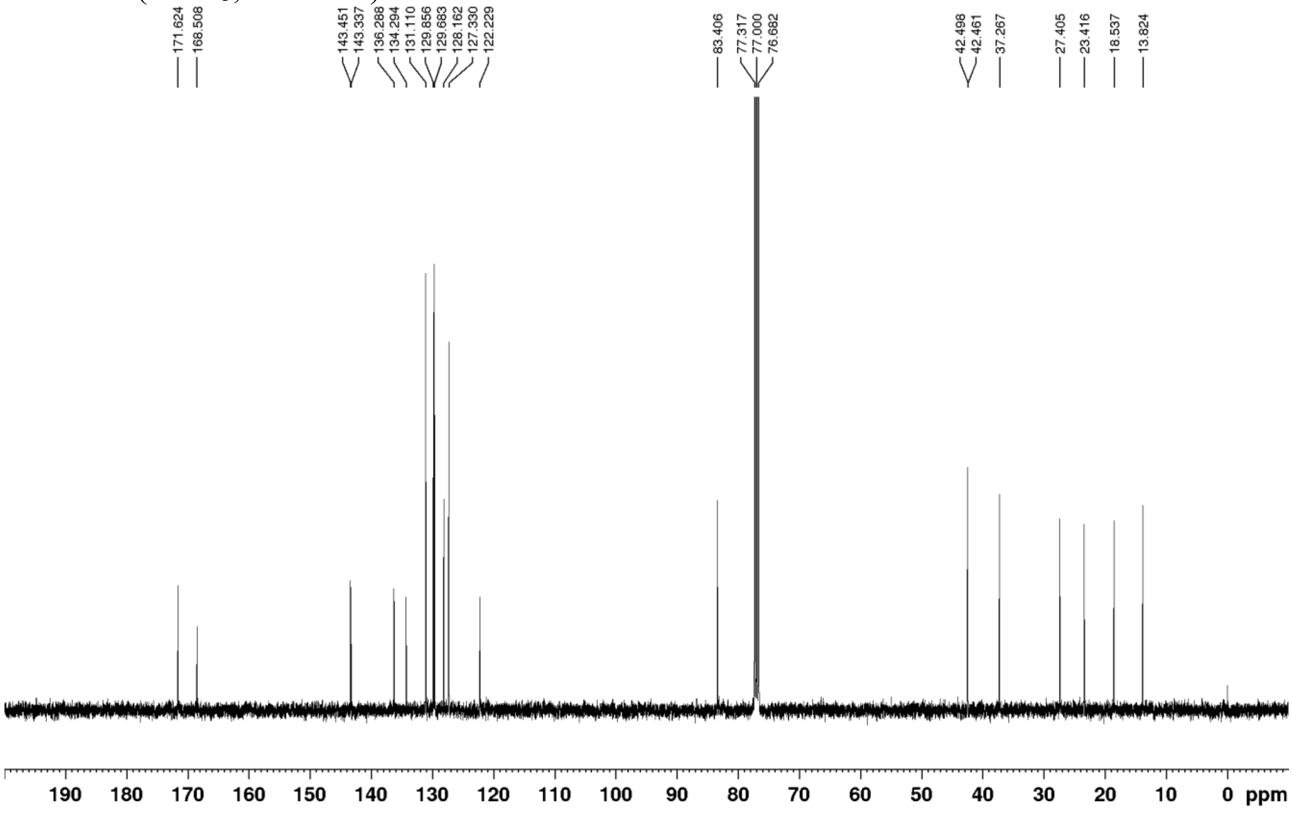

(–)-(1*S*,5*S*,7a*R*)-*N*,7a-Dimethyl-3-oxo-*N*,1,4-triphenyl-1,3,5,6,7,7a-hexahydroisobenzofuran-5-carboxamide (3jg)

^{1}H NMR ($CDCl_3$, 400 MHz)

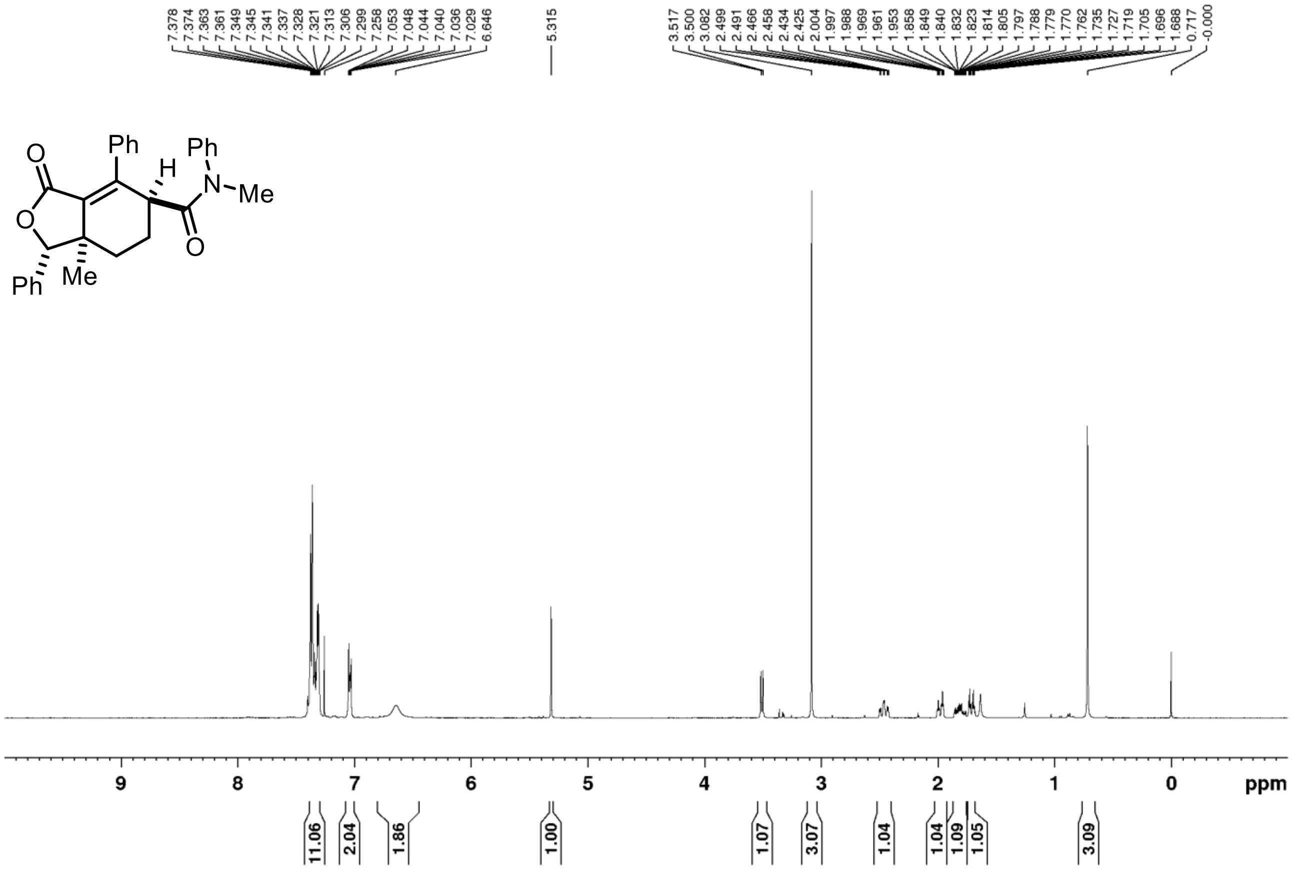

^{13}C NMR ($CDCl_3$, 101 MHz)

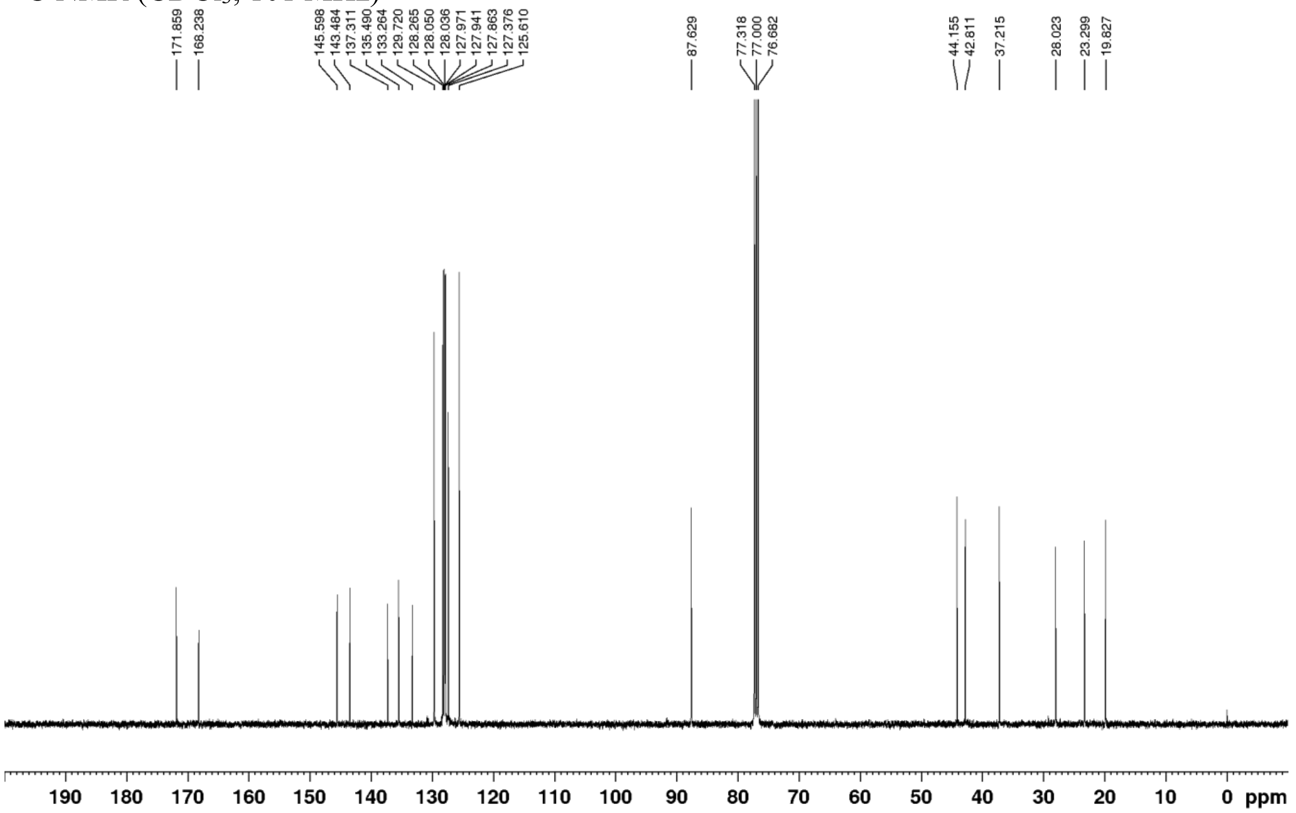

(–)-(8*S*,10a*R*,10b*S*)-*N*,10a-Dimethyl-6-oxo-*N*,7-diphenyl-8,9,10,10a,10b,11-hexahydro-6*H*-isoindolo[2,1-a]indole-8-carboxamide (3kg)

^{1}H NMR ($CDCl_3$, 400 MHz)

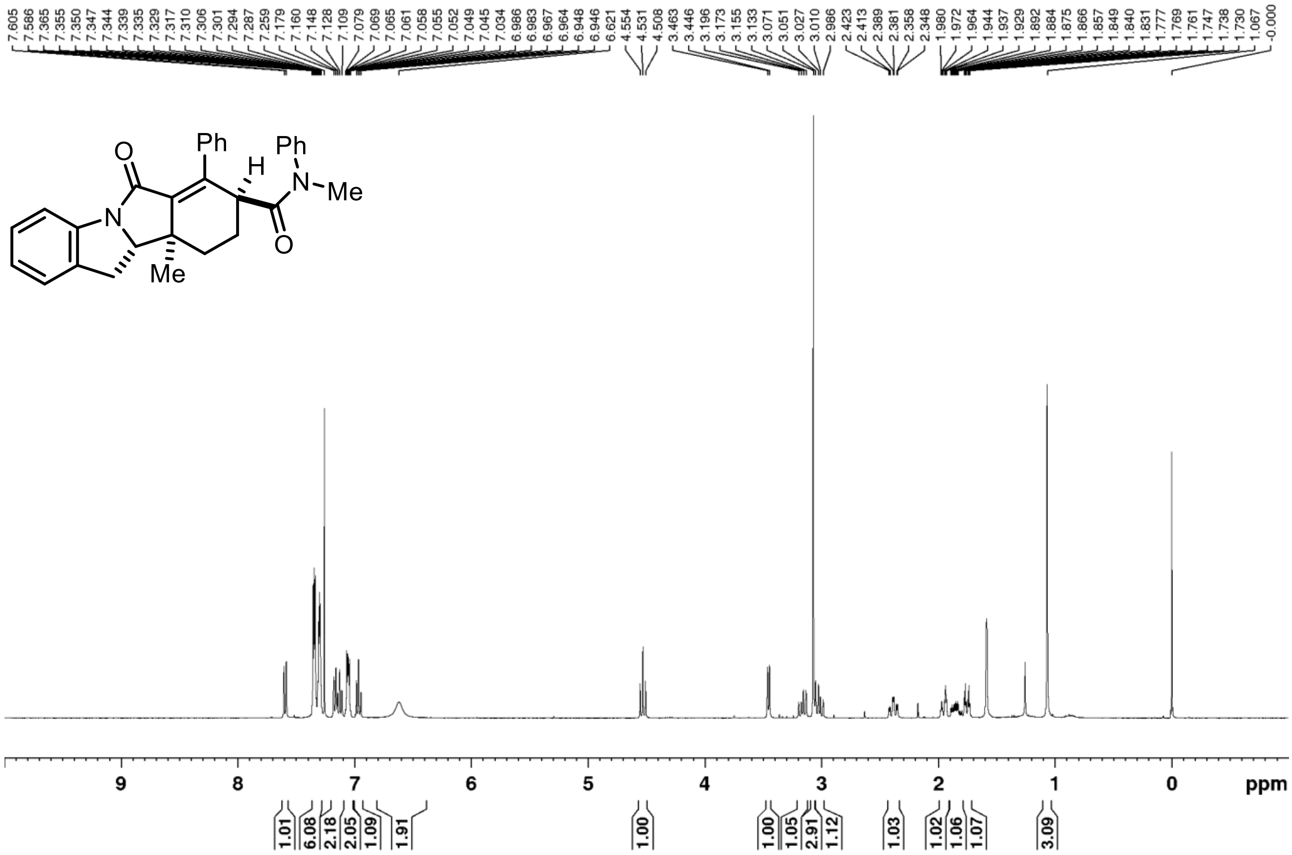

^{13}C NMR ($CDCl_3$, 101 MHz)

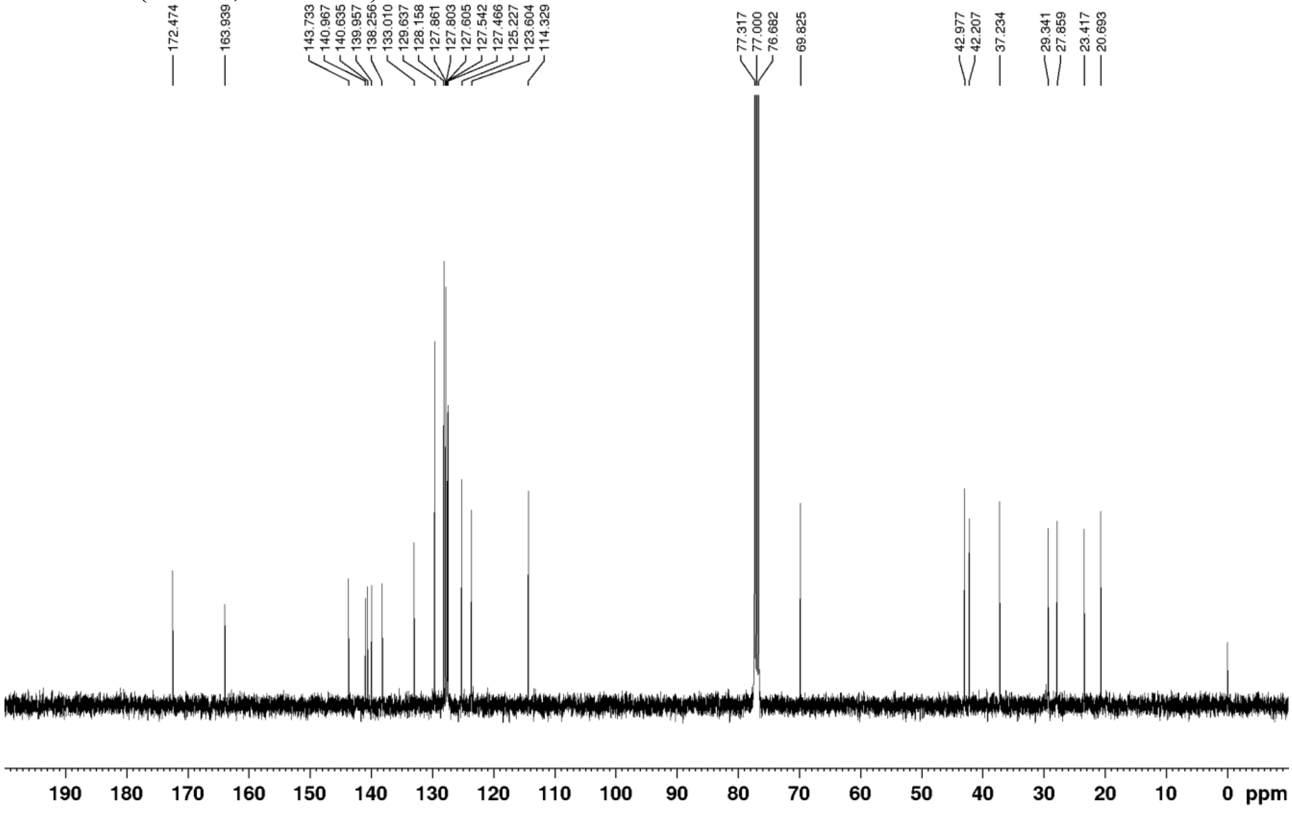

(–)-(1*S*,5*S*,7a*R*)-*N*,1,7a-trimethyl-*N*,4-diphenyl-1,3,5,6,7,7a-hexahydroisobenzofuran-5-carboxamide (3lg)

^{1}H NMR ($CDCl_3$, 400 MHz)

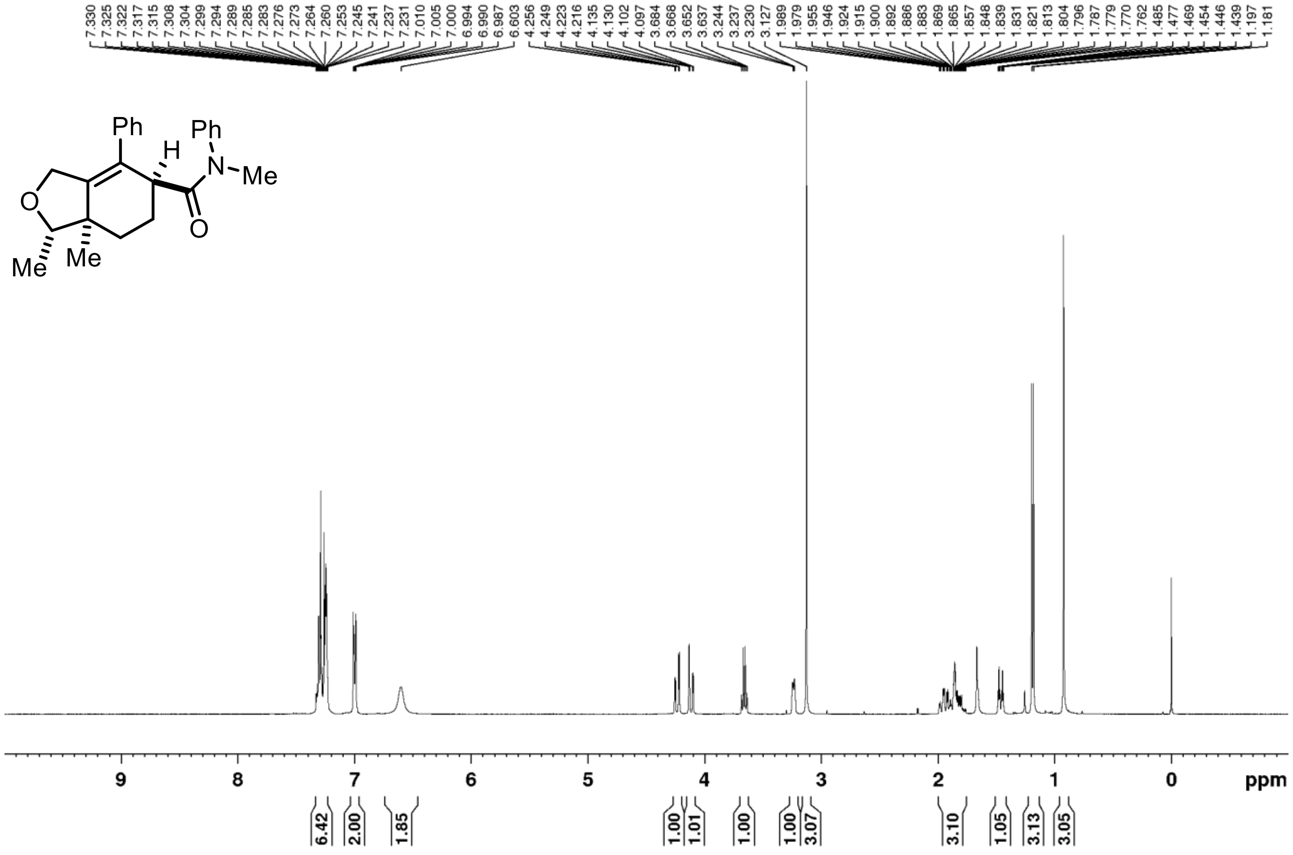

^{13}C NMR ($CDCl_3$, 101 MHz)

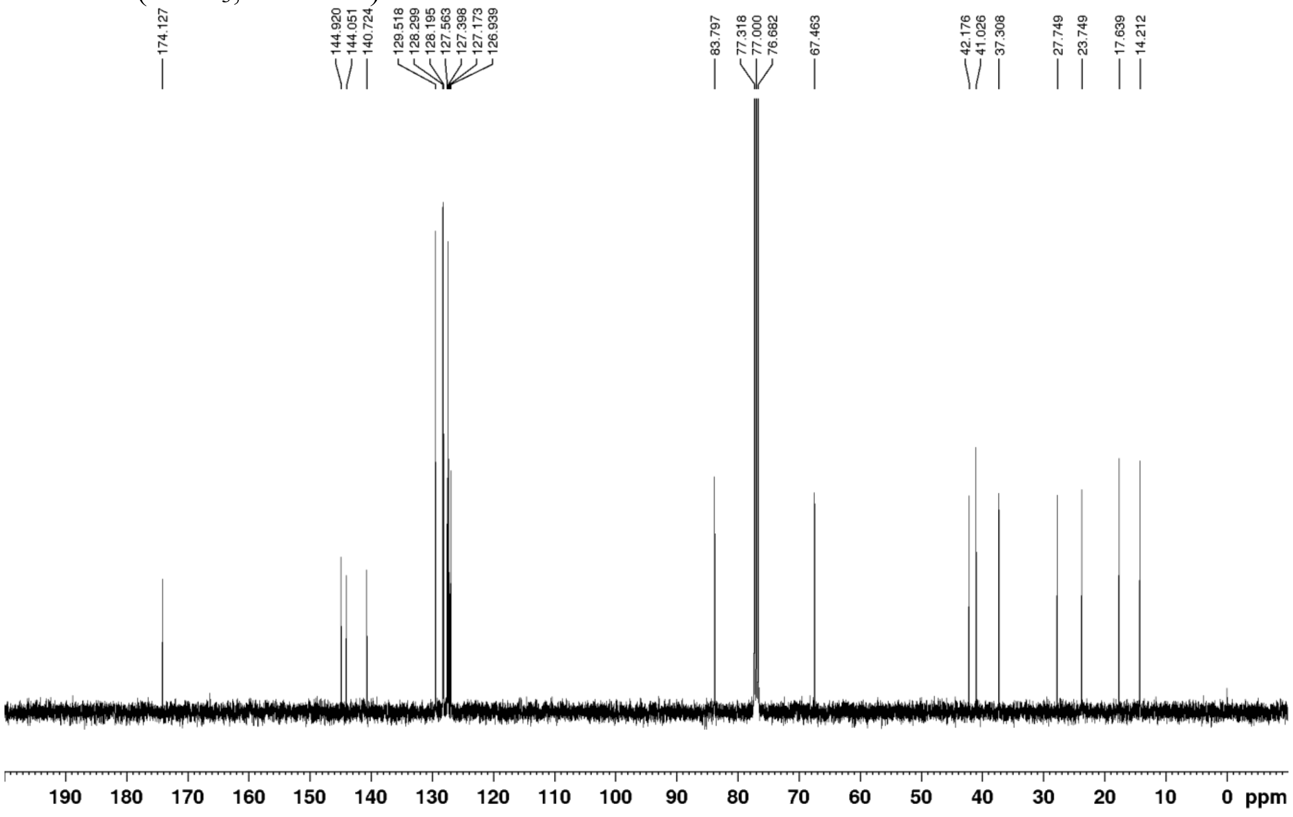

(–)-(1*S*,5*S*,7a*R*)-*N*,*N*-Diethyl-1,7a-dimethyl-3-oxo-4-phenyl-1,3,5,6,7,7a-hexahydroisobenzofuran-5-carboxamide (3hh)

^{1}H NMR ($CDCl_3$, 400 MHz)

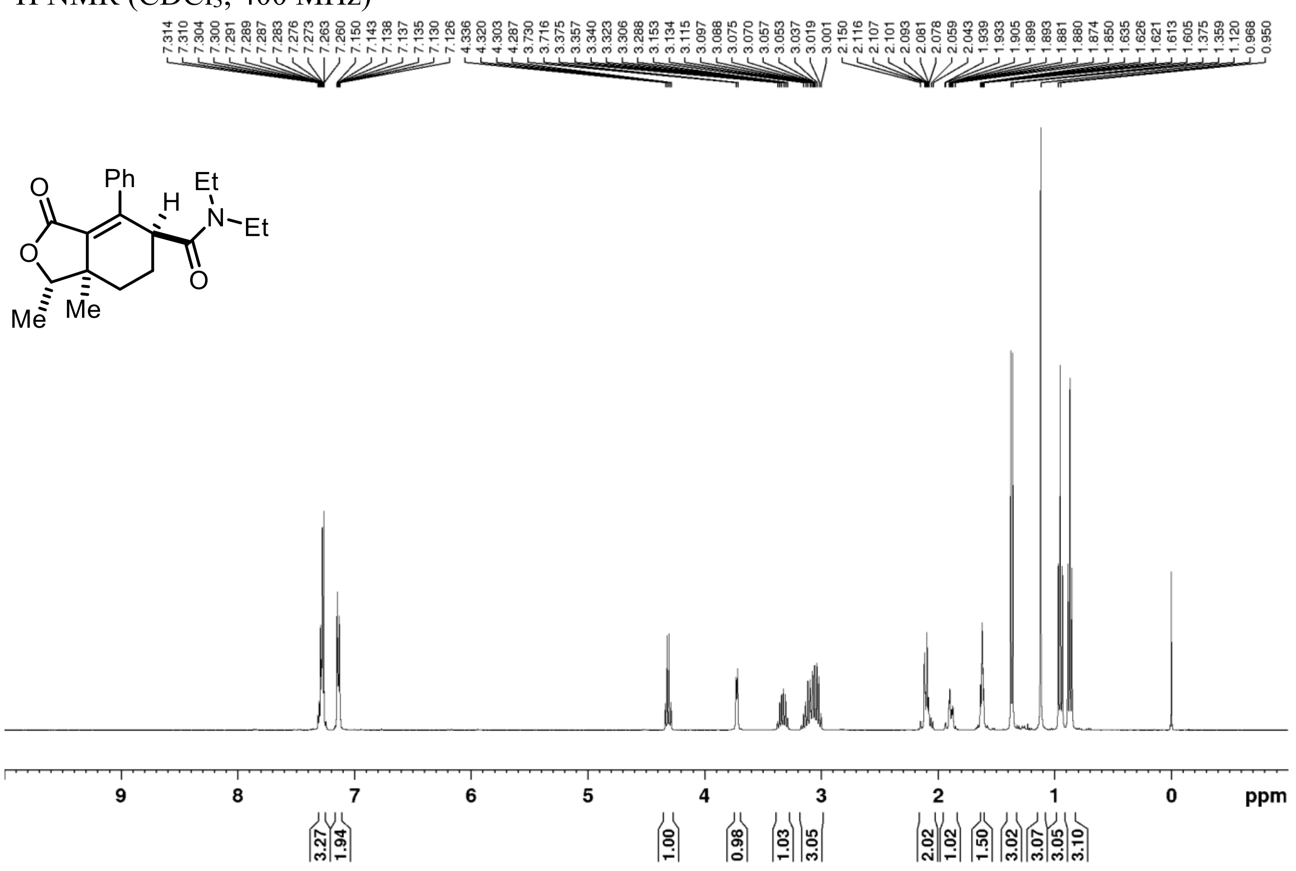

^{13}C NMR ($CDCl_3$, 101 MHz)

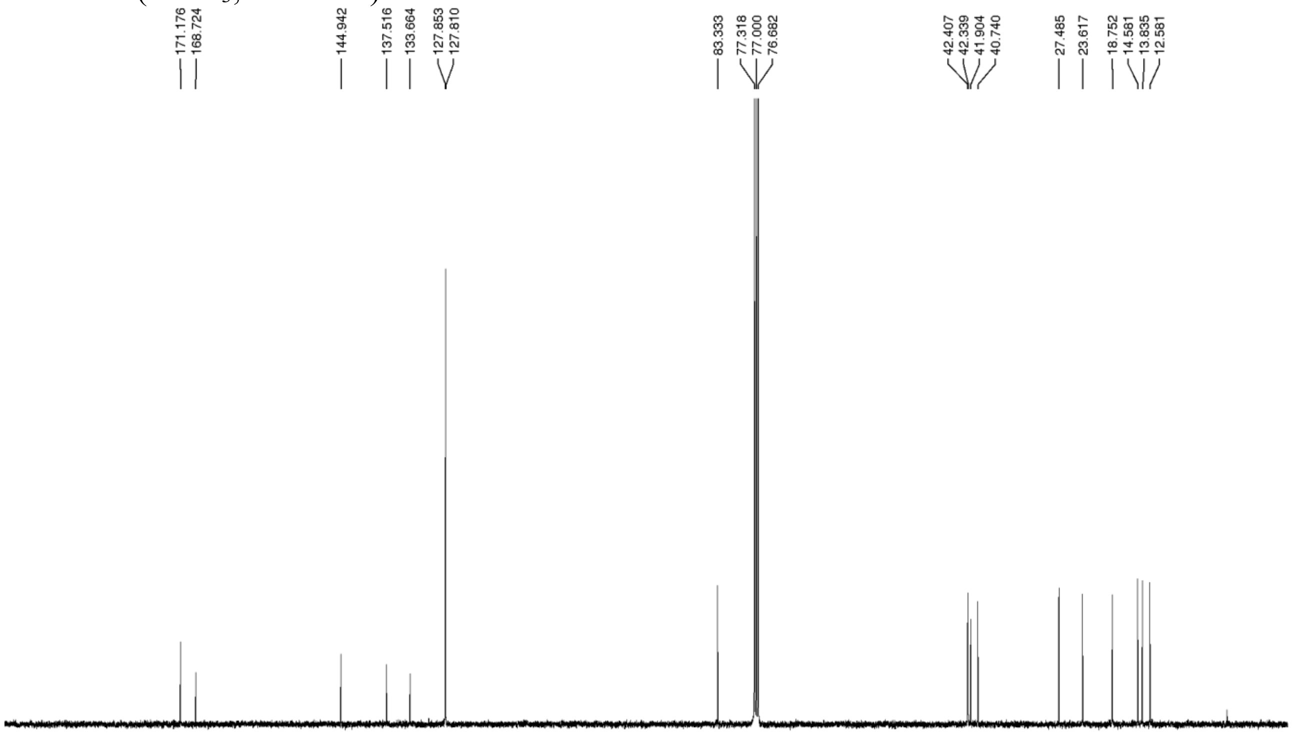

(–)-(*E*)-4-{(2*R*,3*S*)-4-(*Z*)-Benzylidene-2,3-dimethyl-5-oxotetrahydrofuran-3-yl}-2-fluoro-*N*-methyl-*N*-phenylbut-2-enamide (4hi)

^{1}H NMR (CDCl$_3$, 400 MHz)

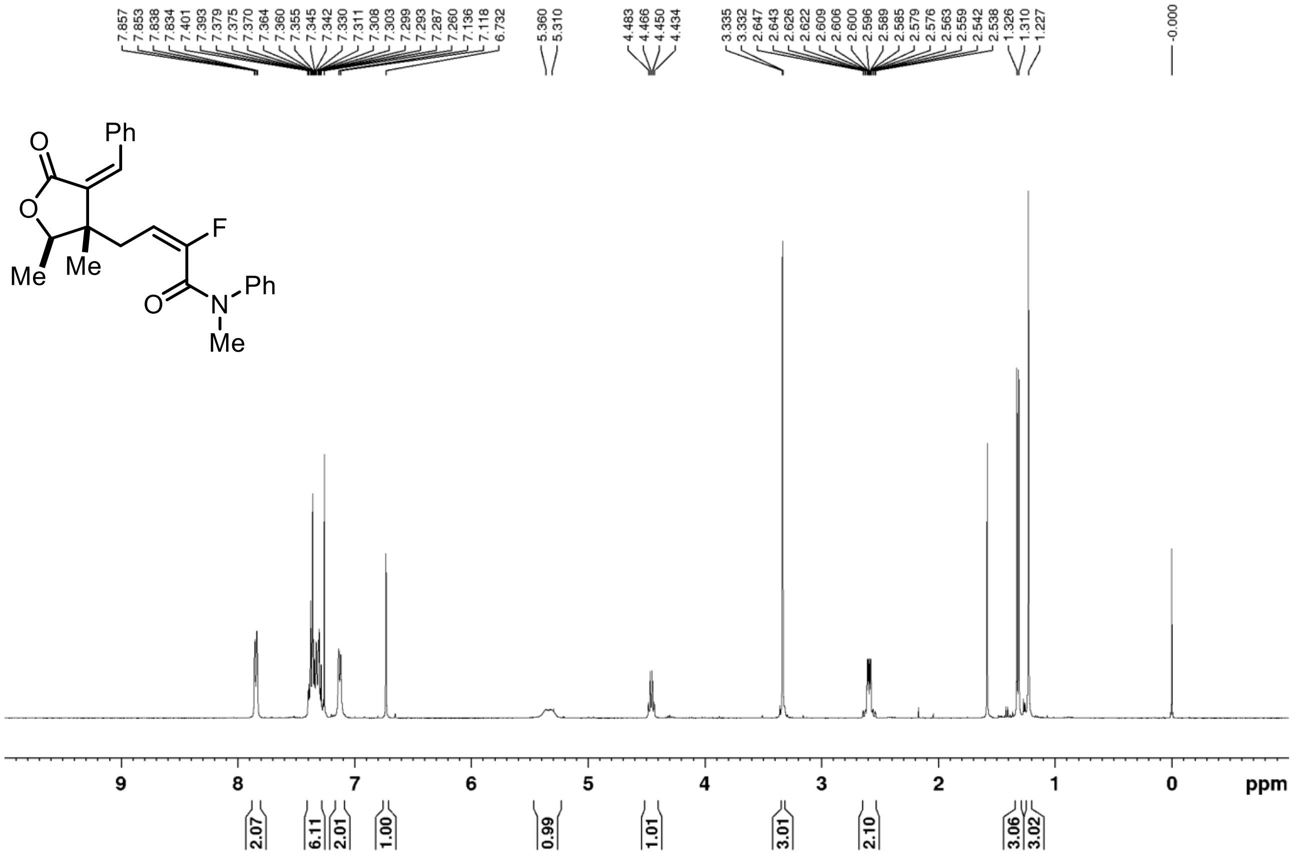

^{13}C NMR (CDCl$_3$, 101 MHz)

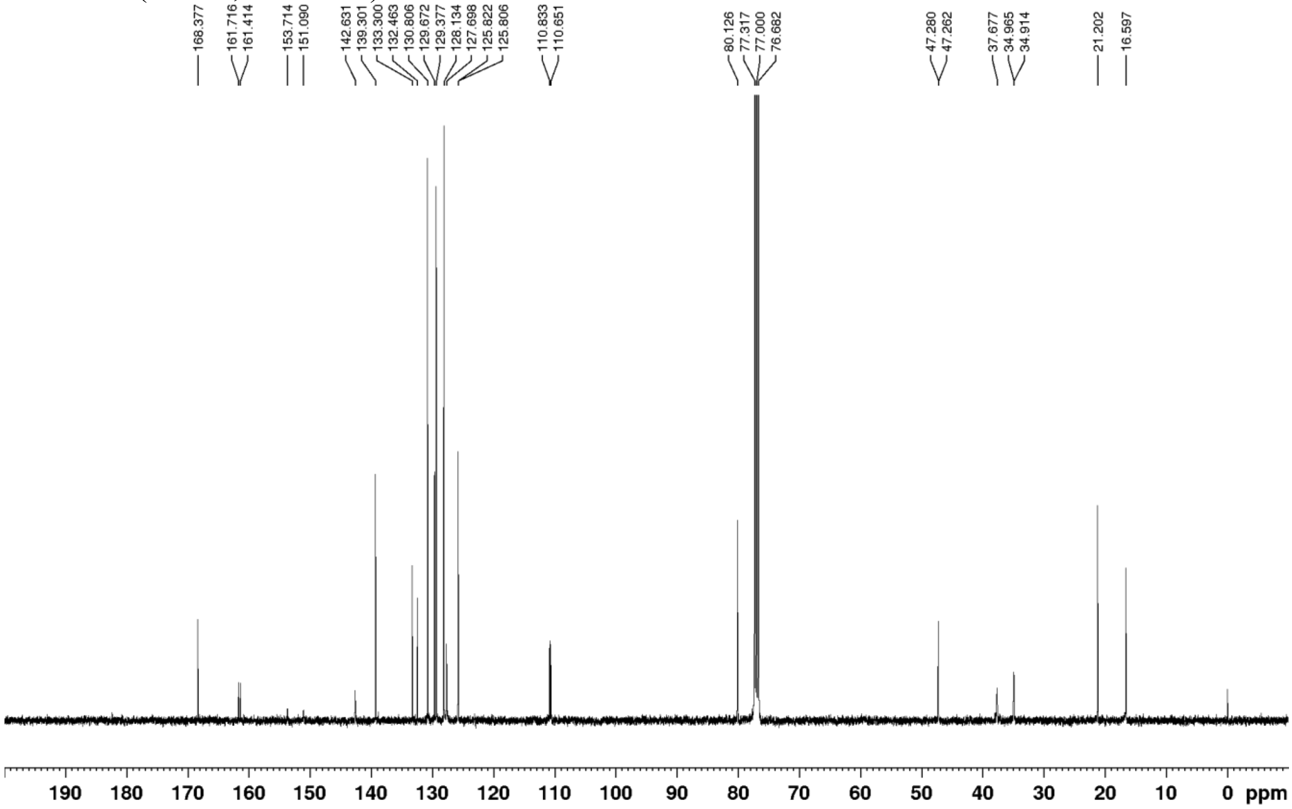

^{19}F NMR ($CDCl_3$, 377 MHz)

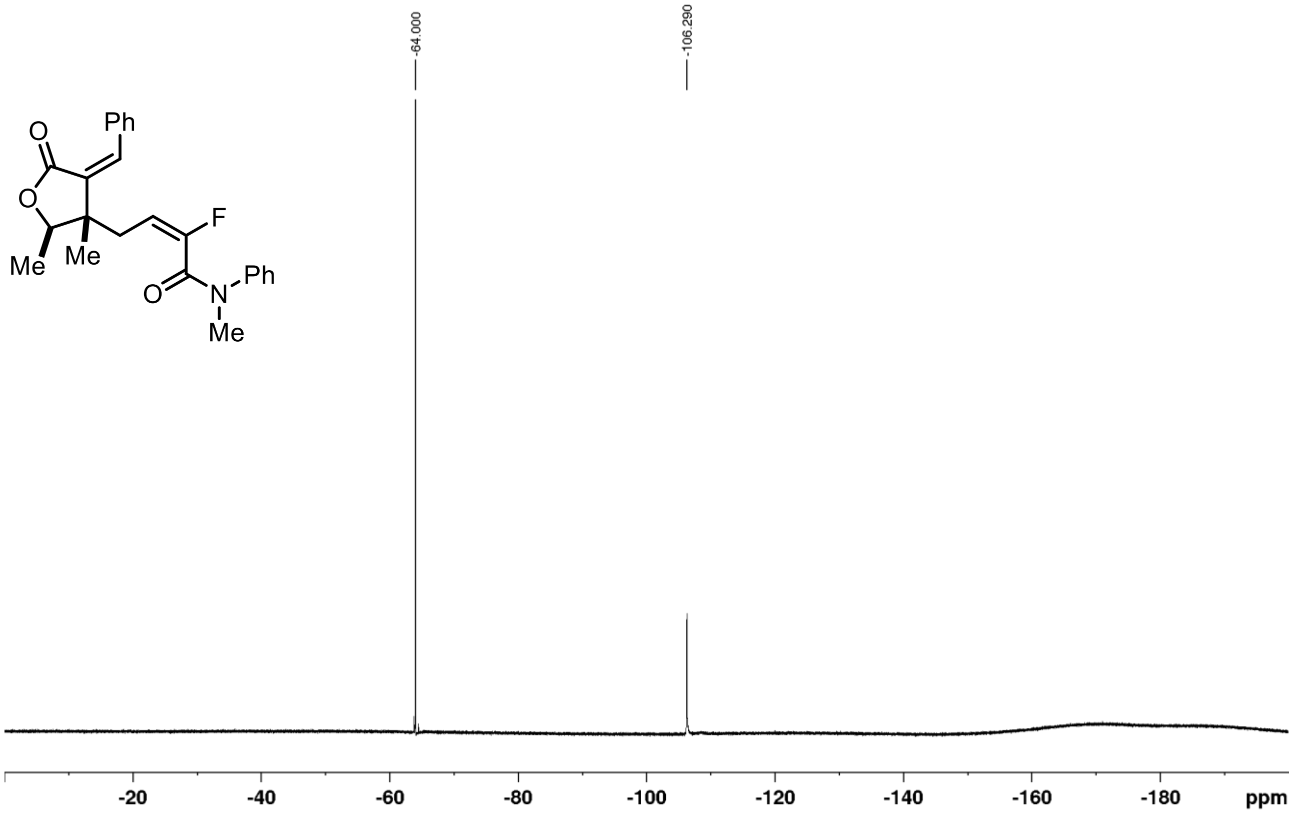

(–)-(4*S*,5*R*)-3-{(*Z*)-Benzylidene}-4-methyl-4-[(*Z*)-3-methyl-4-oxo-4-{pyrrolidin-1-yl}but-2-en-1-yl]-5-phenyldihydrofuran-2(3H)-one (4je)

^{1}H NMR (CDCl$_3$, 400 MHz)

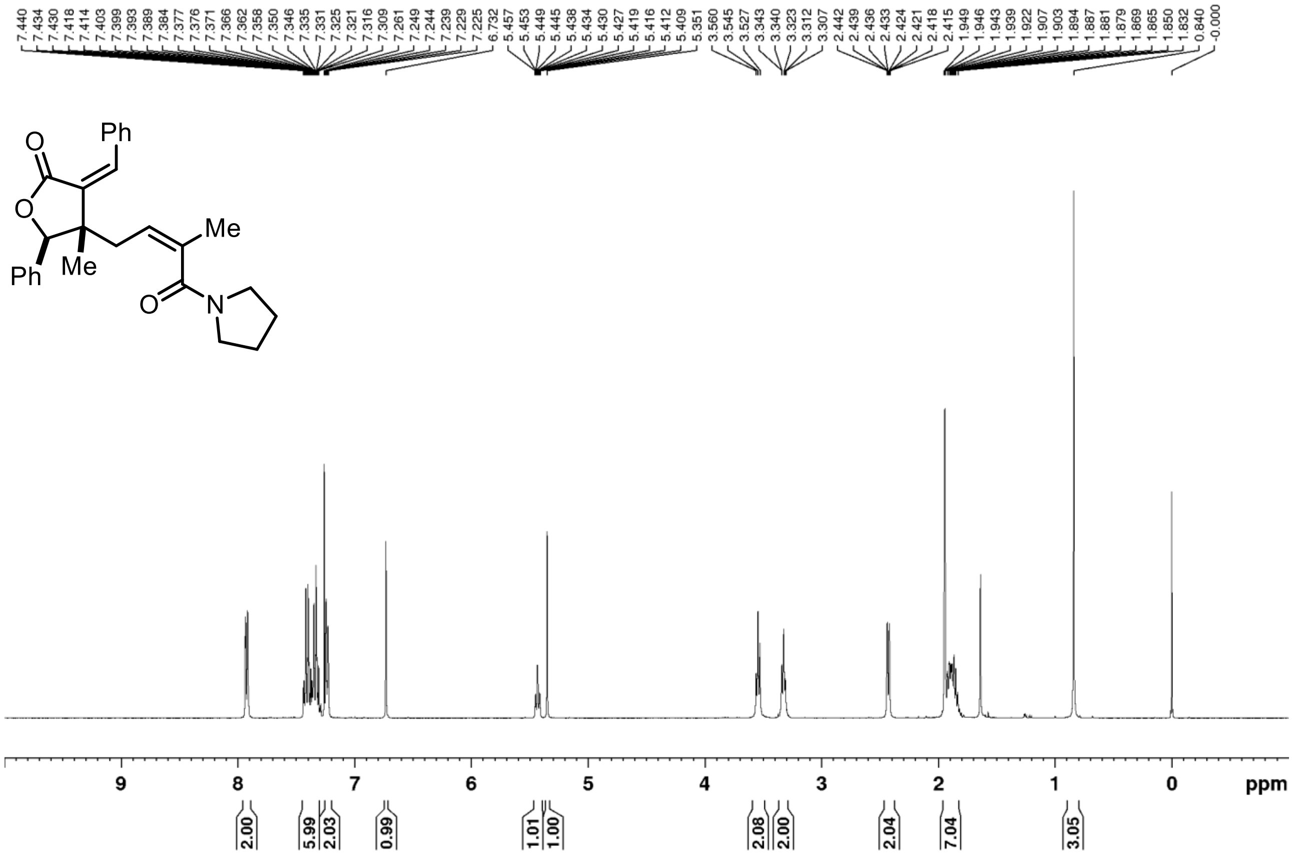

^{13}C NMR (CDCl$_3$, 101 MHz)

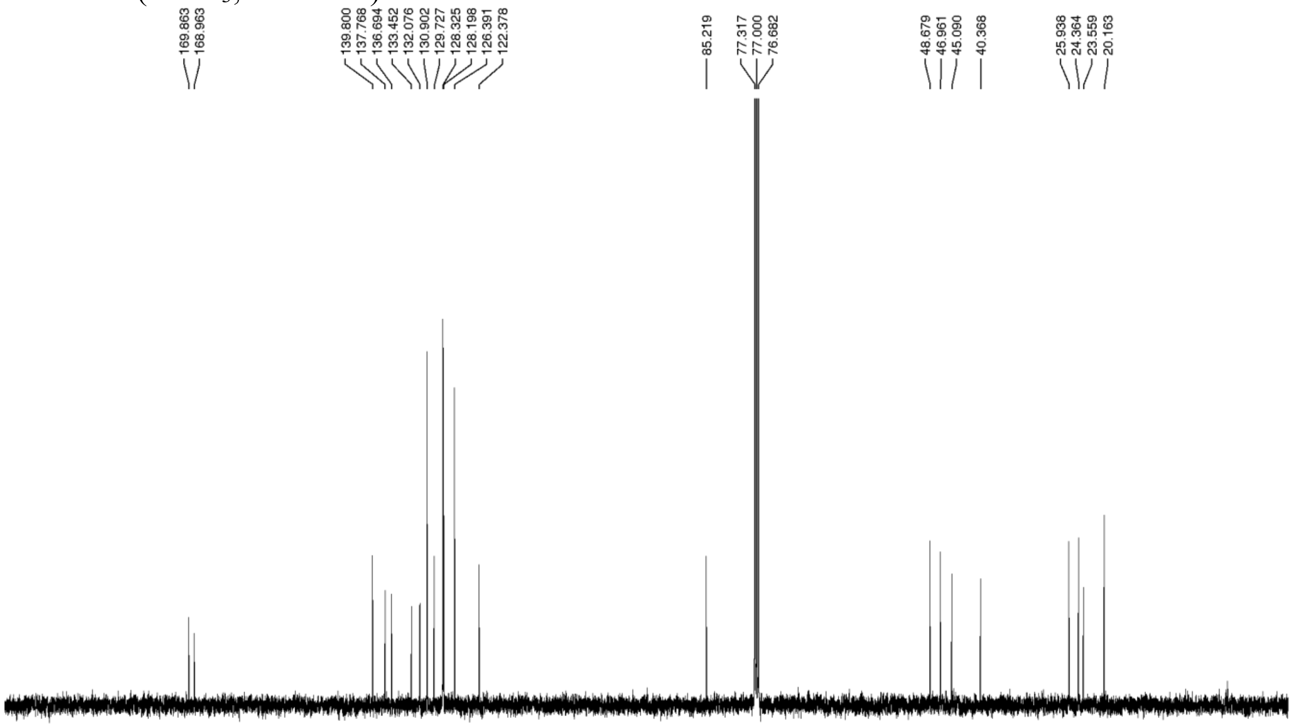

6.4. Rh-Catalyzed Enantioselective Desymmetrization of Achiral Dienynes with Acrylamide Derivatives (Figure 4)

{(1*R*,5*R*,7a*R*)-5-Fluoro-2-(4-methylphenyl)-4-phenyl-1-vinyl-2,3,5,6,7,7a-hexahydro-1*H*-isoindol-5-yl}(pyrrolidin-1-yl)methanone (6aa) Diastereomer mixture (94:6 d.r.)

^{1}H NMR ($CDCl_3$, 400 MHz)

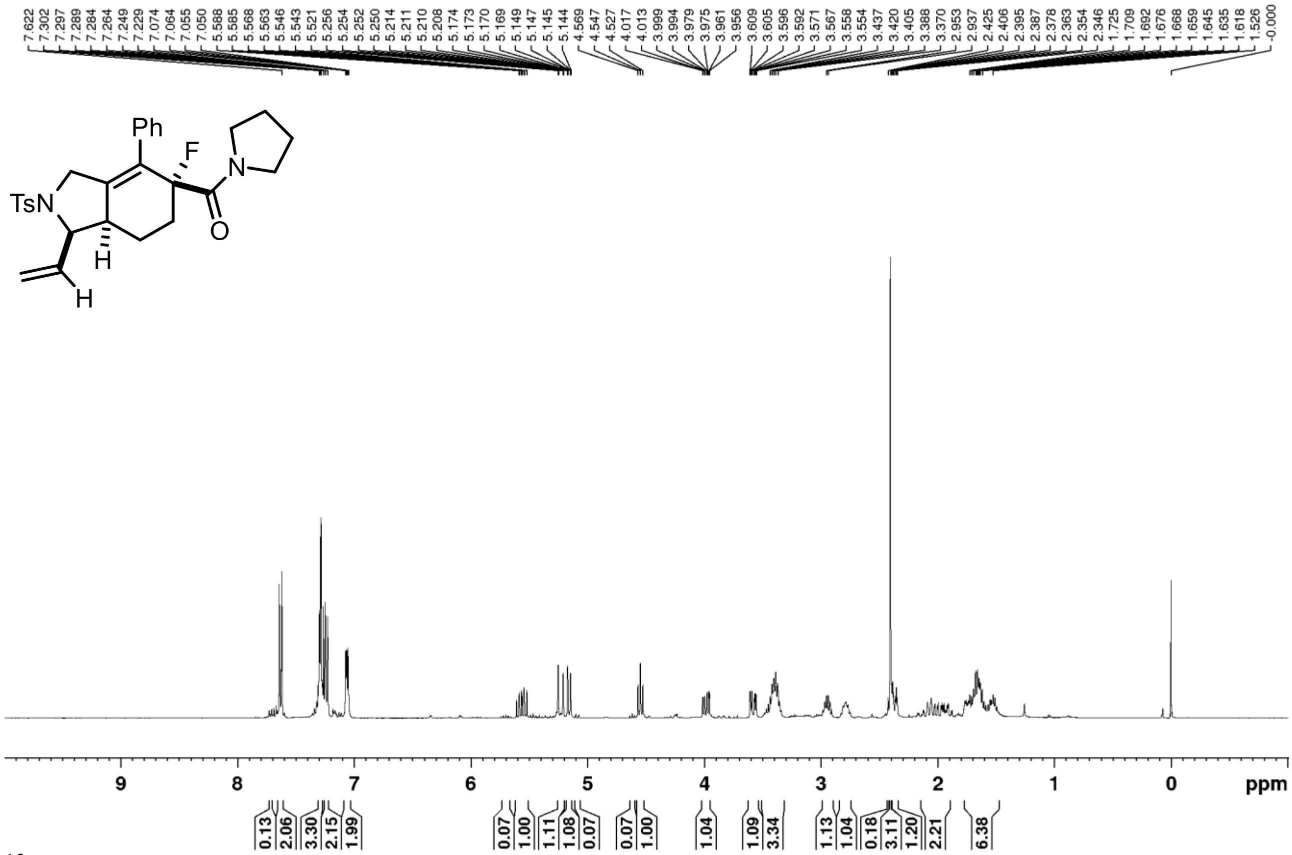

^{13}C NMR ($CDCl_3$, 101 MHz)

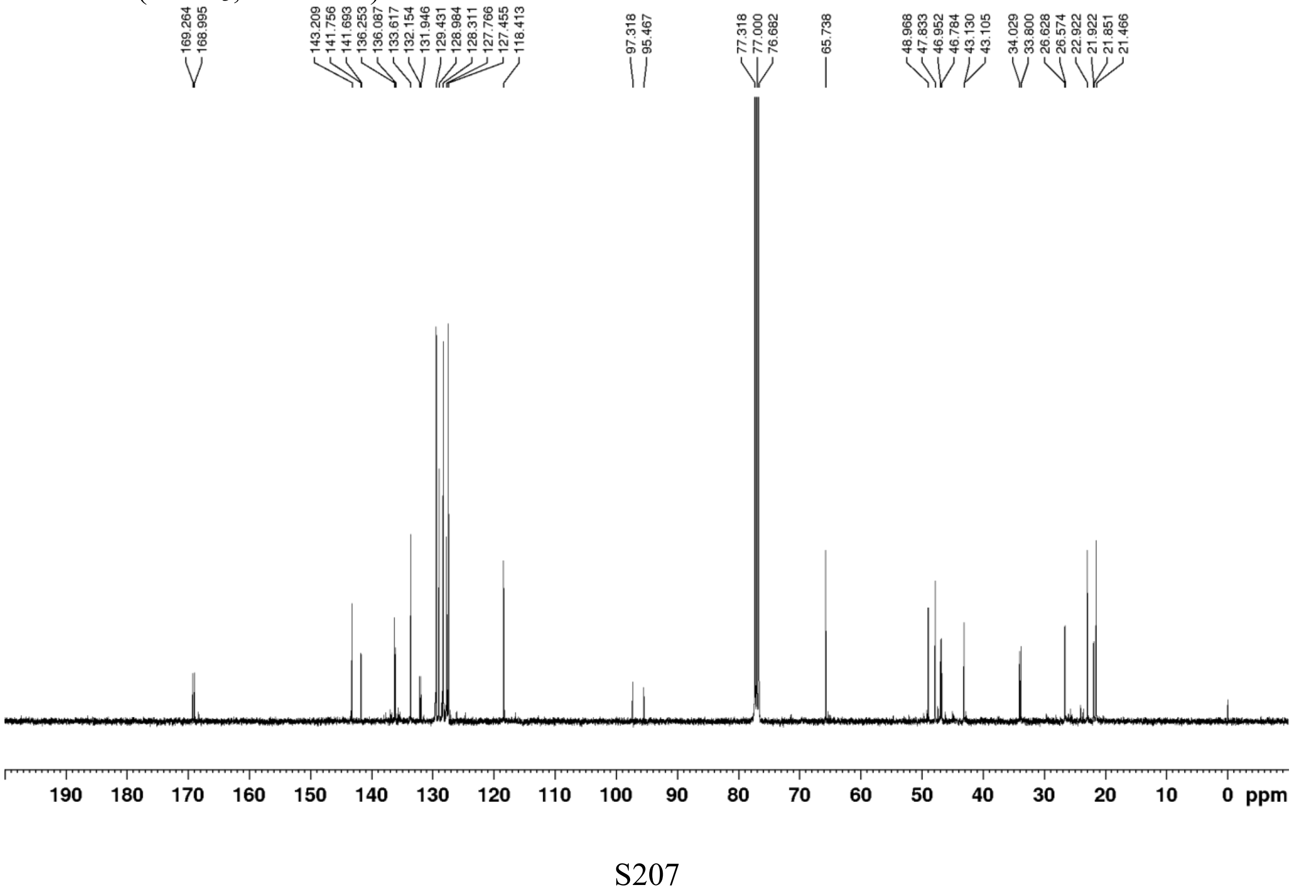

^{19}F NMR ($CDCl_3$, 377 MHz)

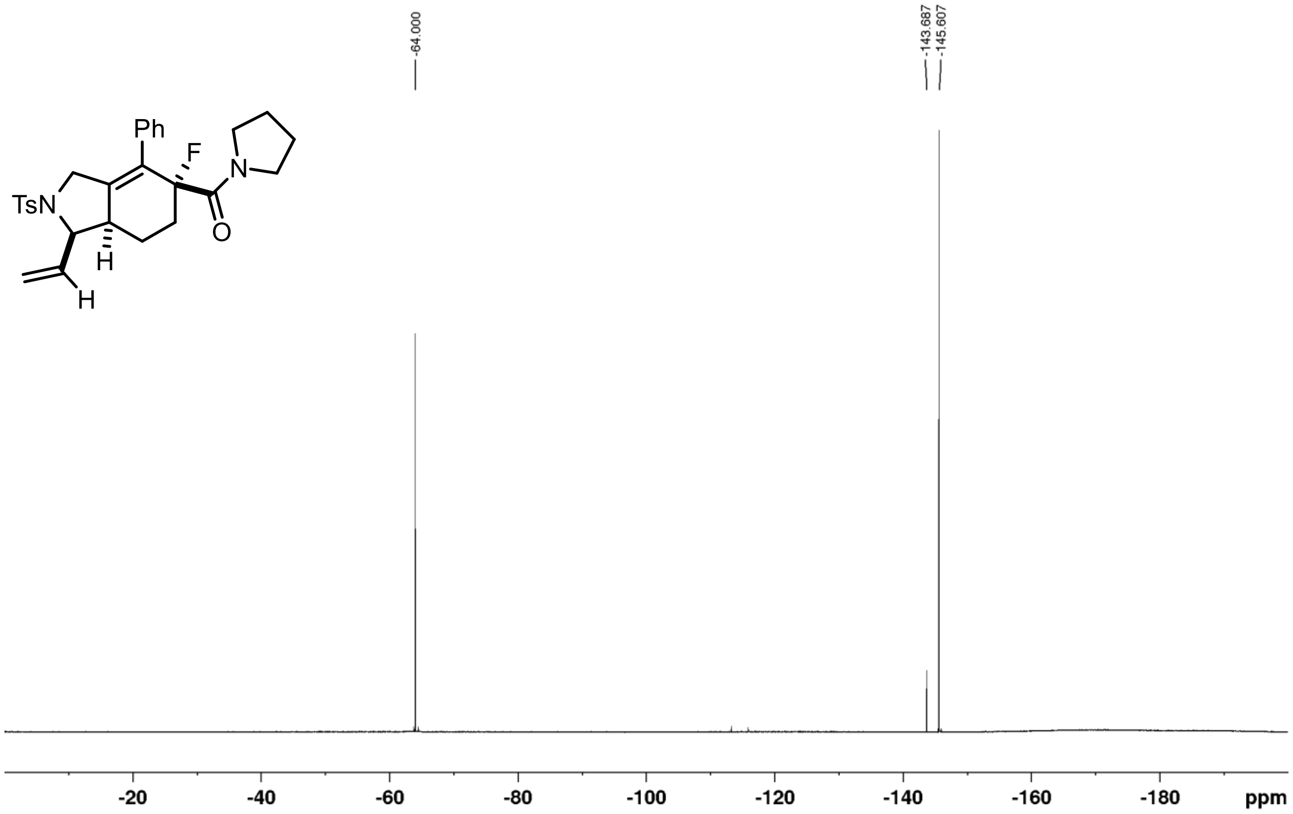

{(1*R*,5*R*,7a*R*)-5-Fluoro-2-(4-methylphenyl)-4-phenyl-1-vinyl-2,3,5,6,7,7a-hexahydro-1*H*-isoindol-5-yl}(pyrrolidin-1-yl)methanone (6ab)

Diastereomer mixture (93:7 d.r.)

^{1}H NMR ($CDCl_3$, 400 MHz)

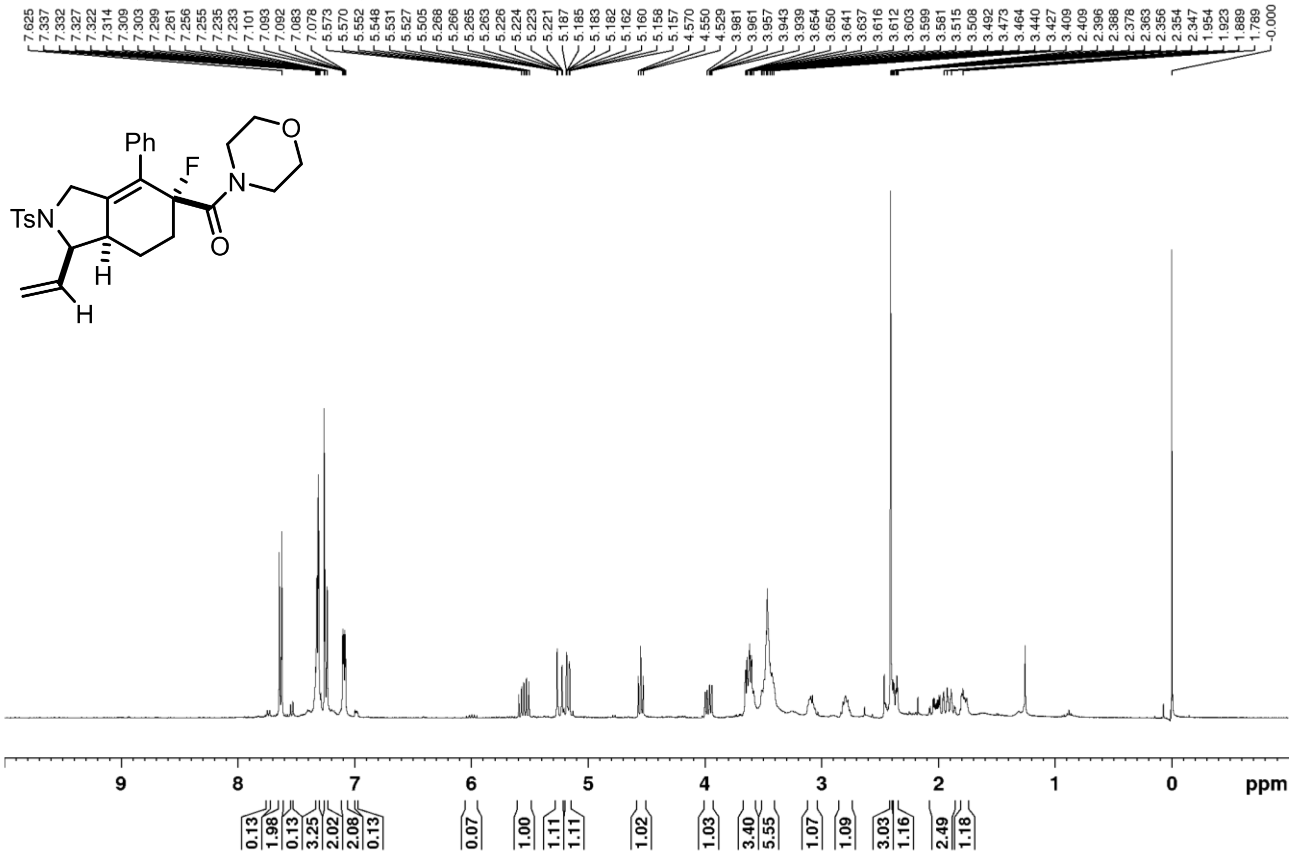

^{13}C NMR ($CDCl_3$, 101 MHz)

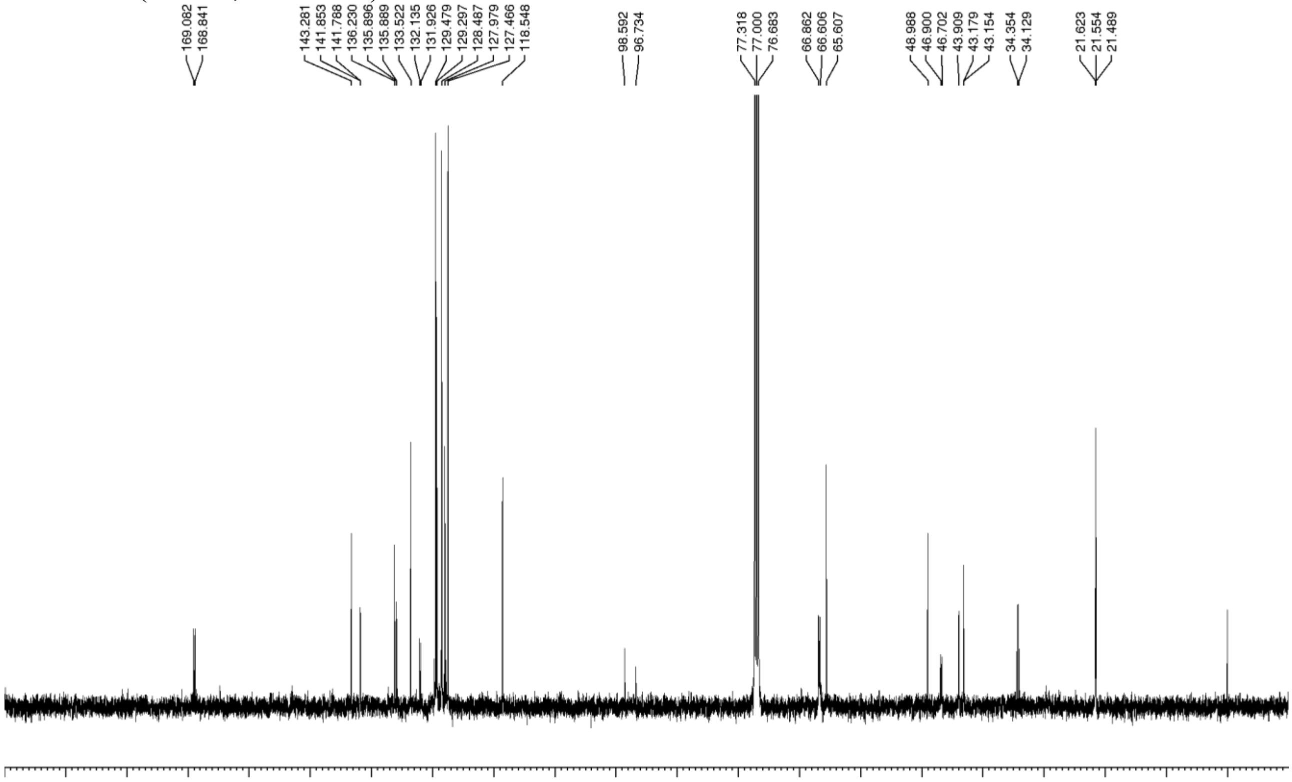

^{19}F NMR ($CDCl_3$, 377 MHz)

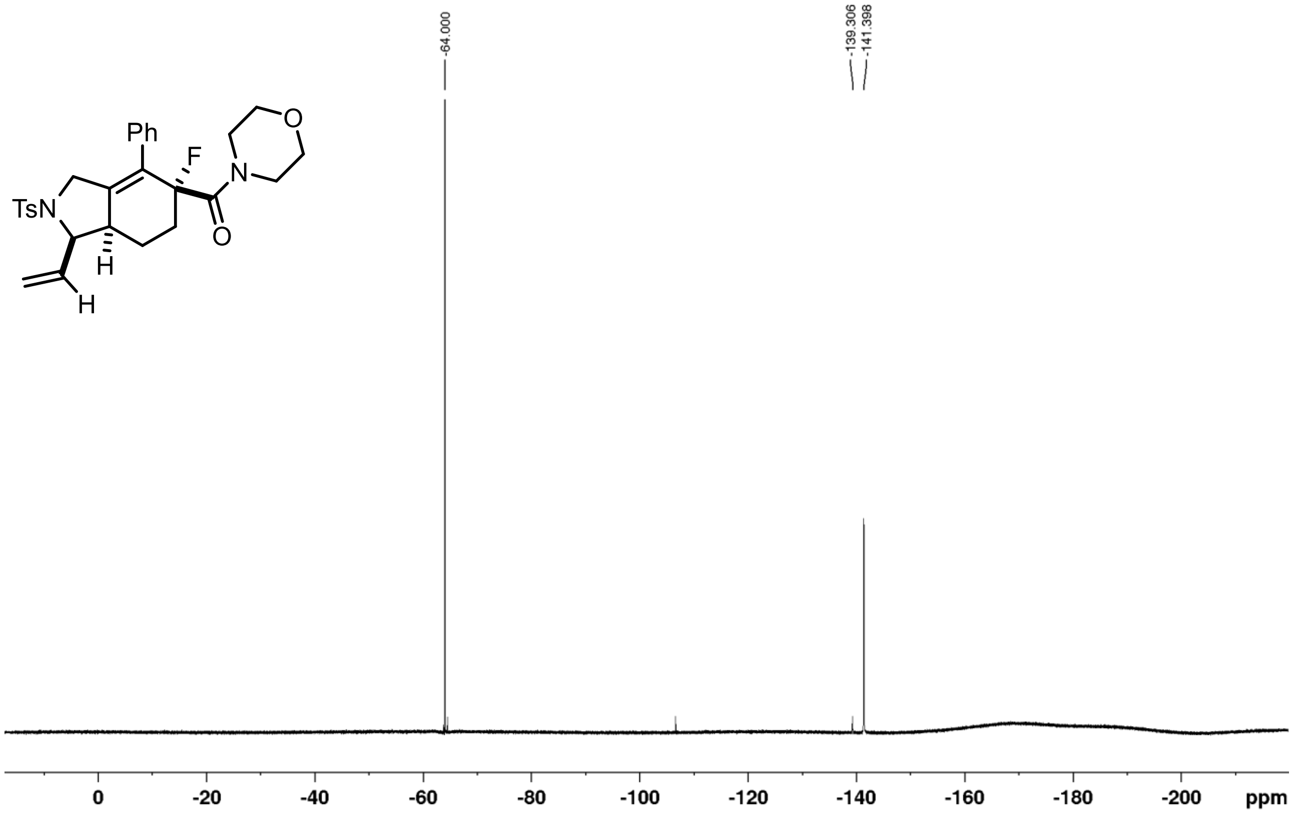

(1*R*,5*R*,7a*R*)-5-Fluoro-*N*-methyl-2-(4-methylphenyl)-*N*,4-diphenyl-1-vinyl-2,3,5,6,7,7a-hexahydro-1*H*-isoindole-5-carboxamide (6ai)

^{1}H NMR (CDCl$_3$, 400 MHz)

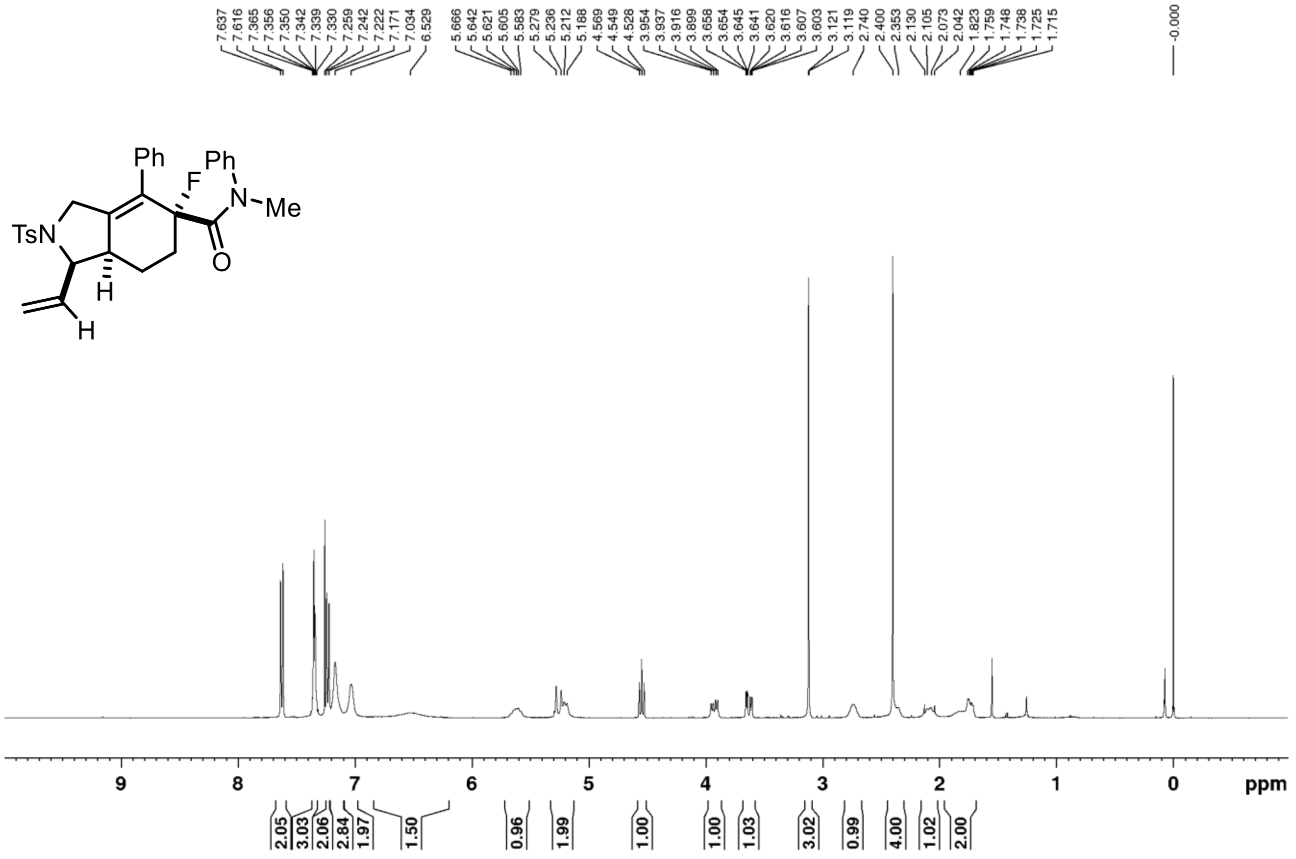

^{13}C NMR (CDCl$_3$, 101 MHz)

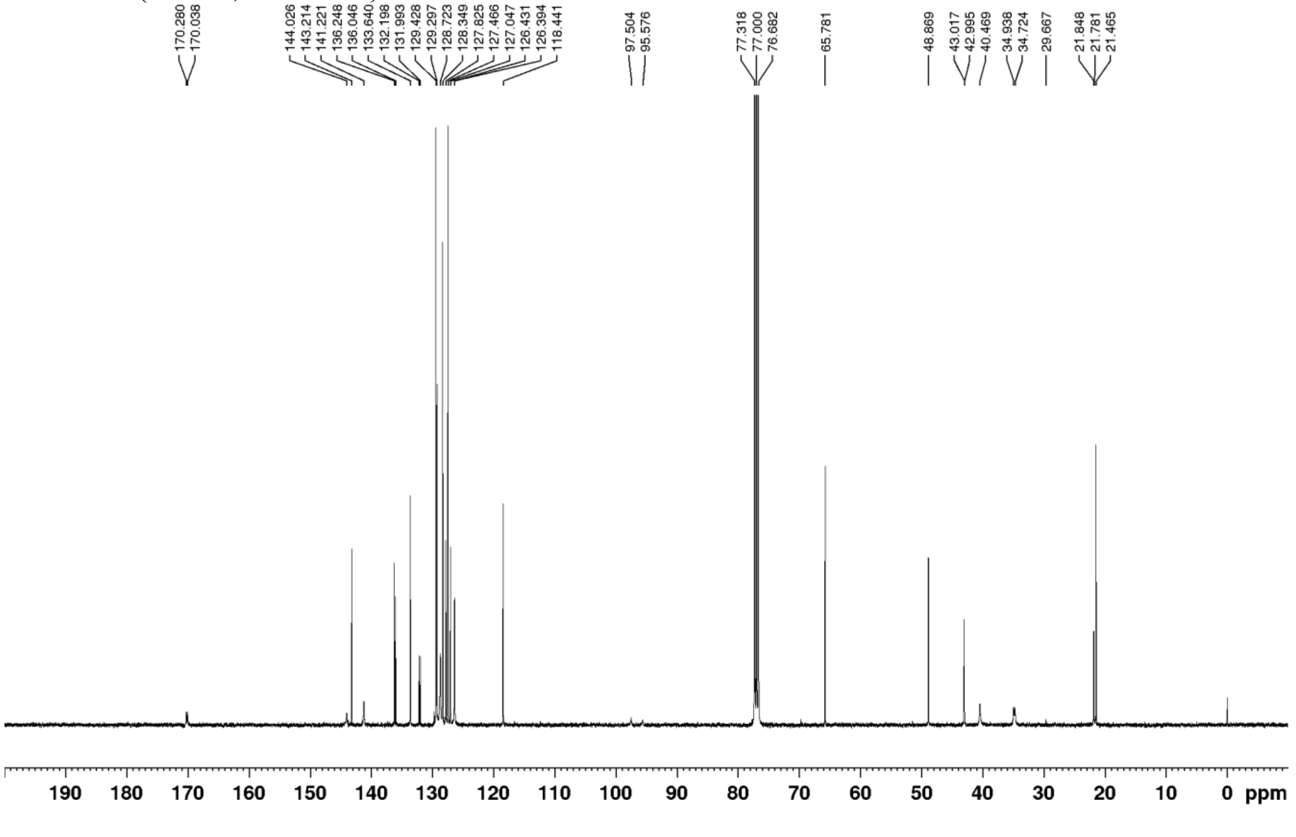

^{19}F NMR ($CDCl_3$, 377 MHz)

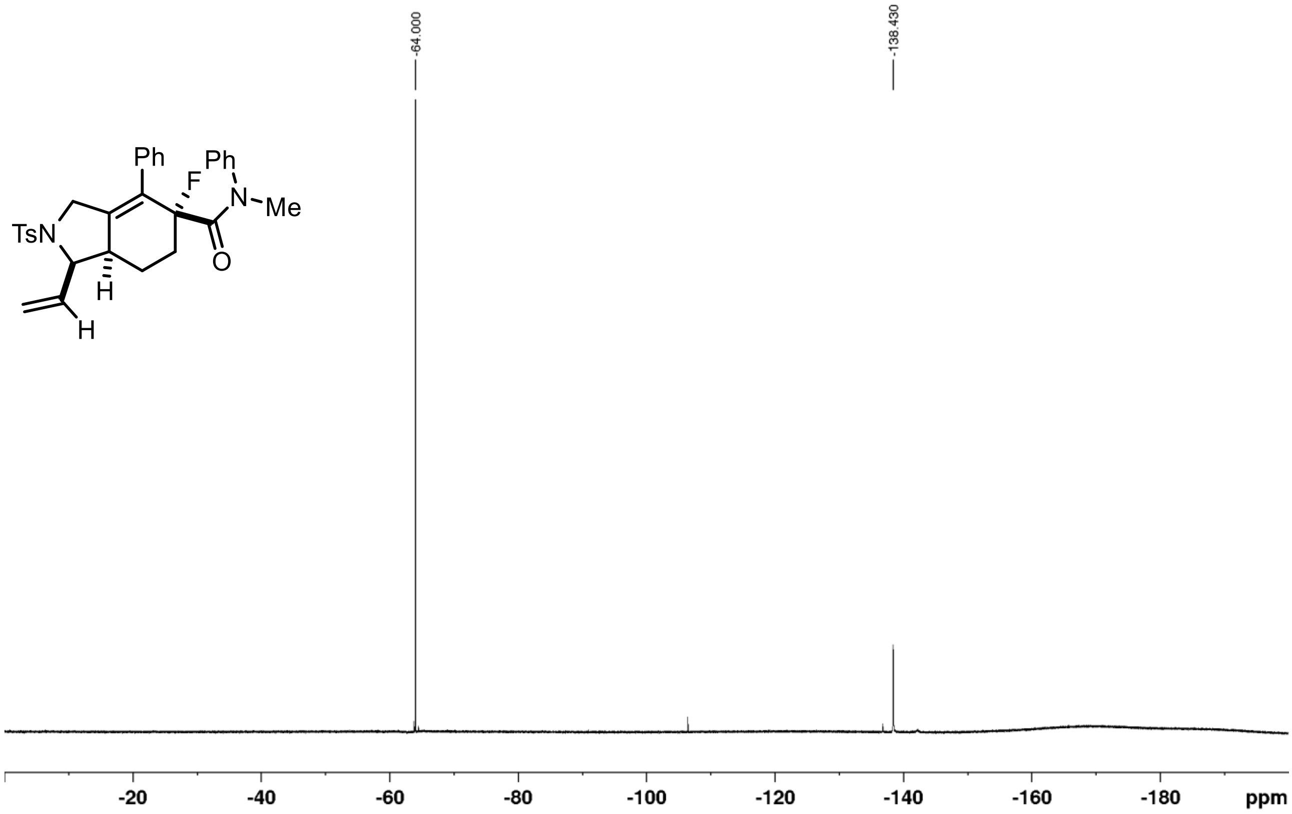

(1*R*,5*R*,7a*R*)-5-Fluoro-2-(4-methylphenyl)-*N*,*N*,4-triphenyl-1-vinyl-2,3,5,6,7,7a-hexahydro-1*H*-isoindole-5-carboxamide (6ac)

Diastereomer mixture (95:5 d.r.)

^{1}H NMR ($CDCl_3$, 400 MHz)

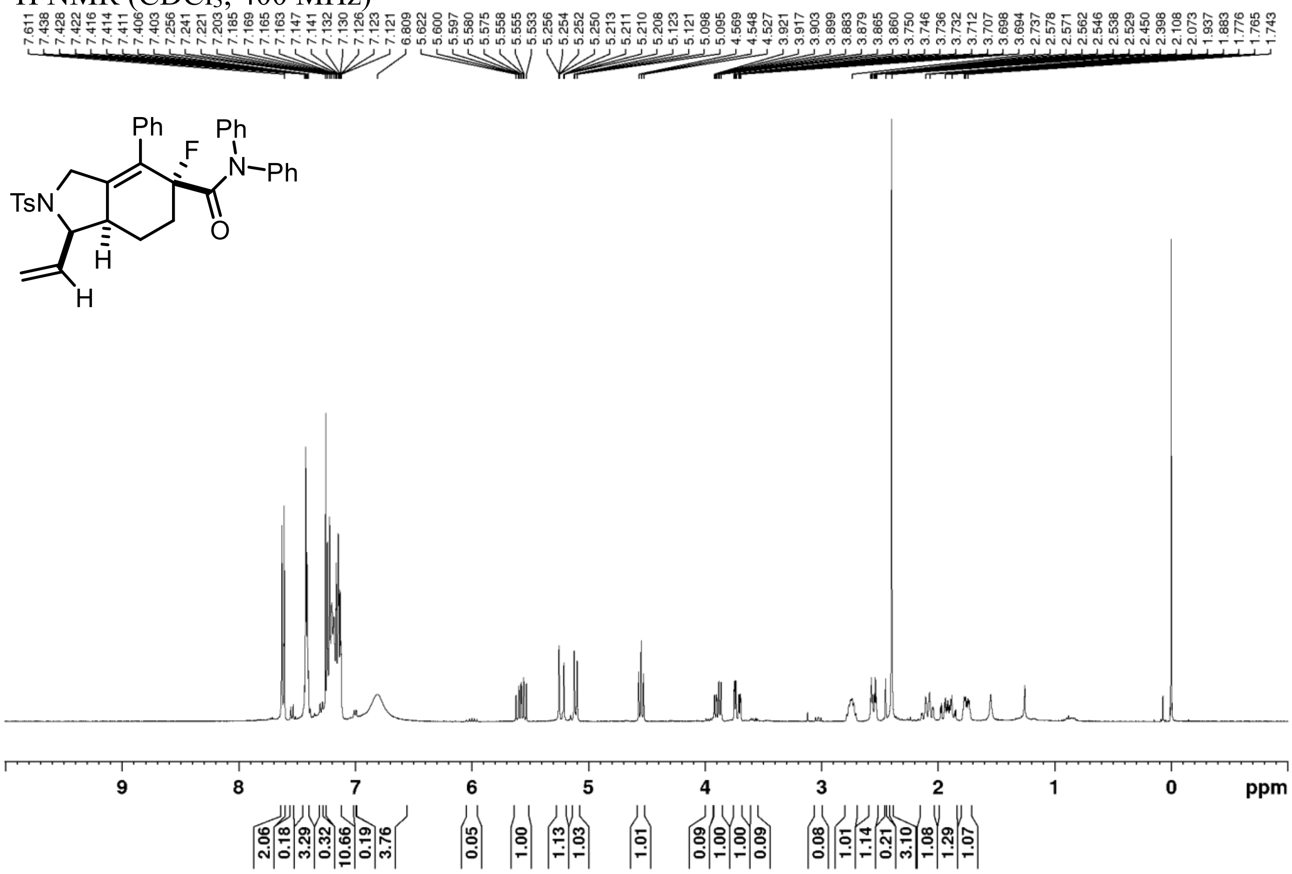

^{13}C NMR ($CDCl_3$, 101 MHz)

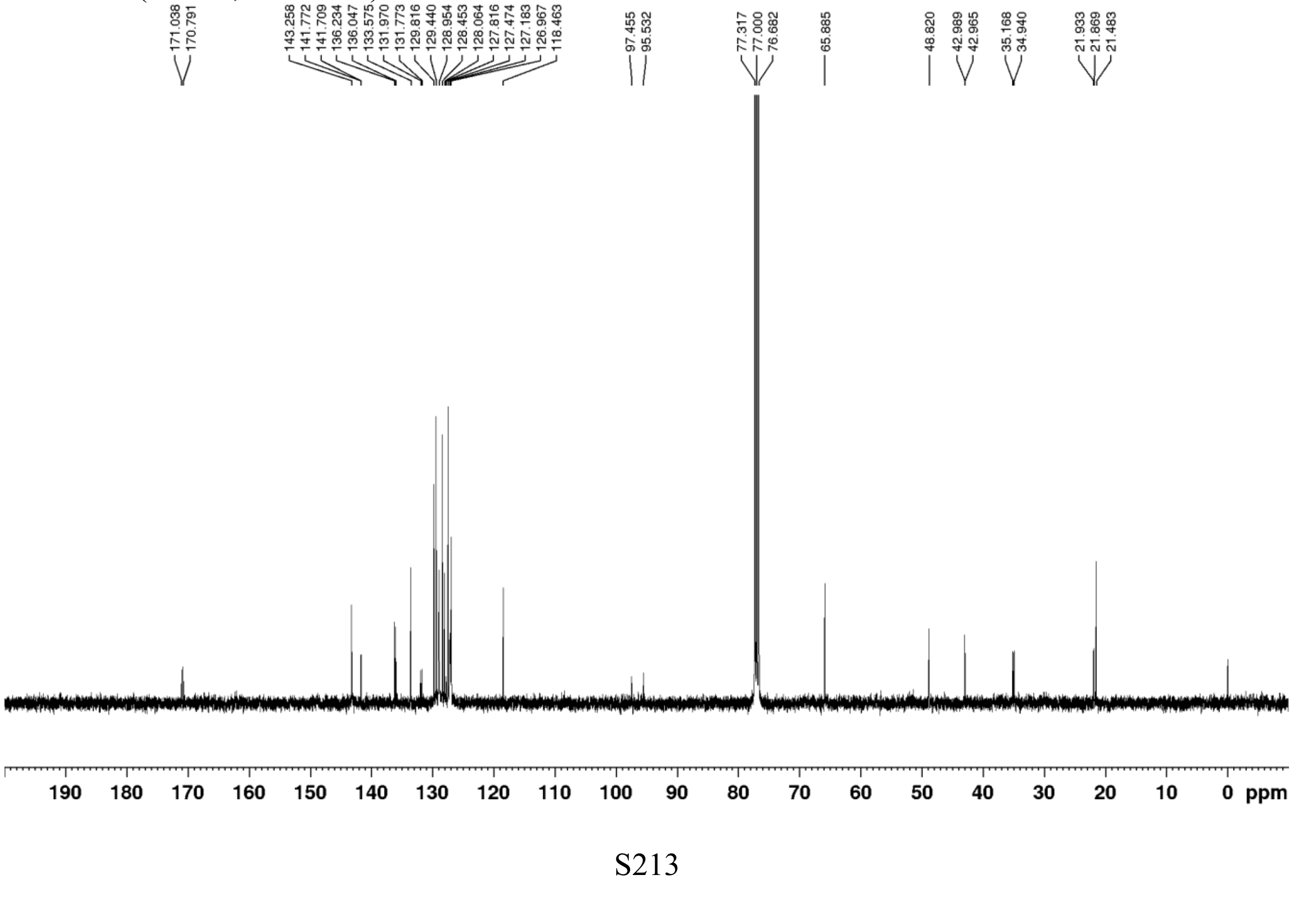

^{19}F NMR ($CDCl_3$, 377 MHz)

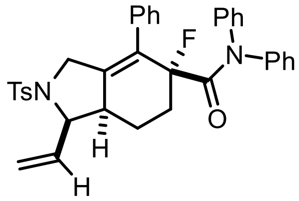

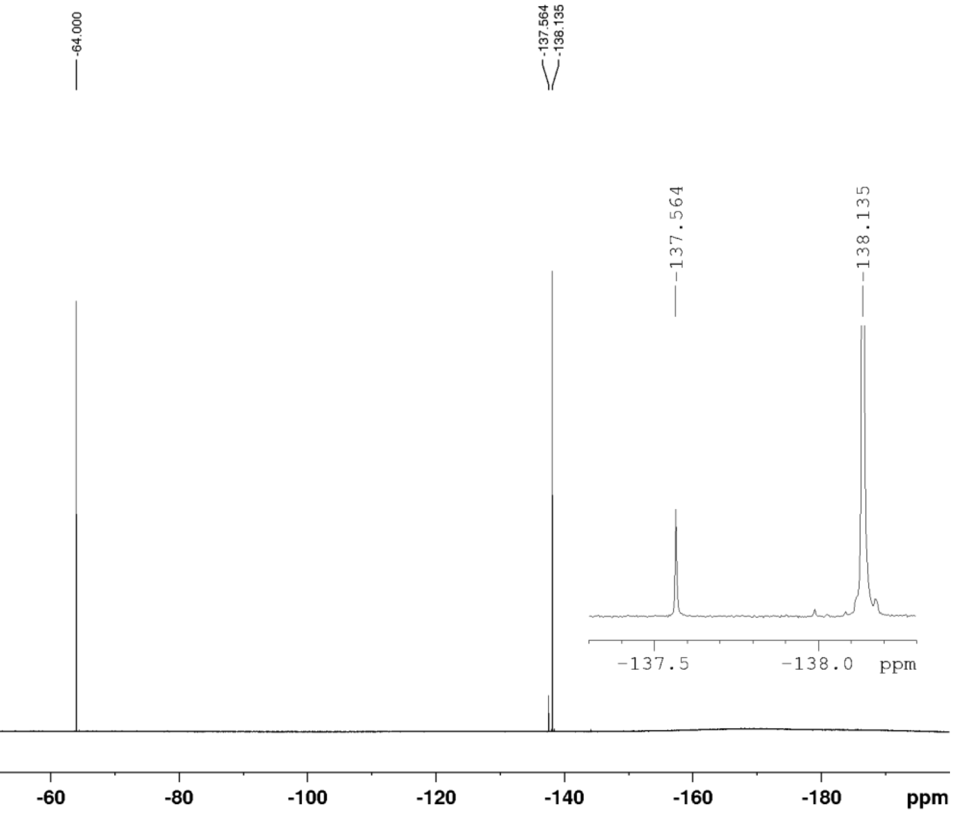

(1*R*,5*R*,7a*R*)-5-Fluoro-*N*-methoxy-*N*-methyl-2-(4-methylphenyl)-4-phenyl-1-vinyl-2,3,5,6,7,7a-hexahydro-1*H*-isoindole-5-carboxamide (6aj)

^{1}H NMR ($CDCl_3$, 400 MHz)

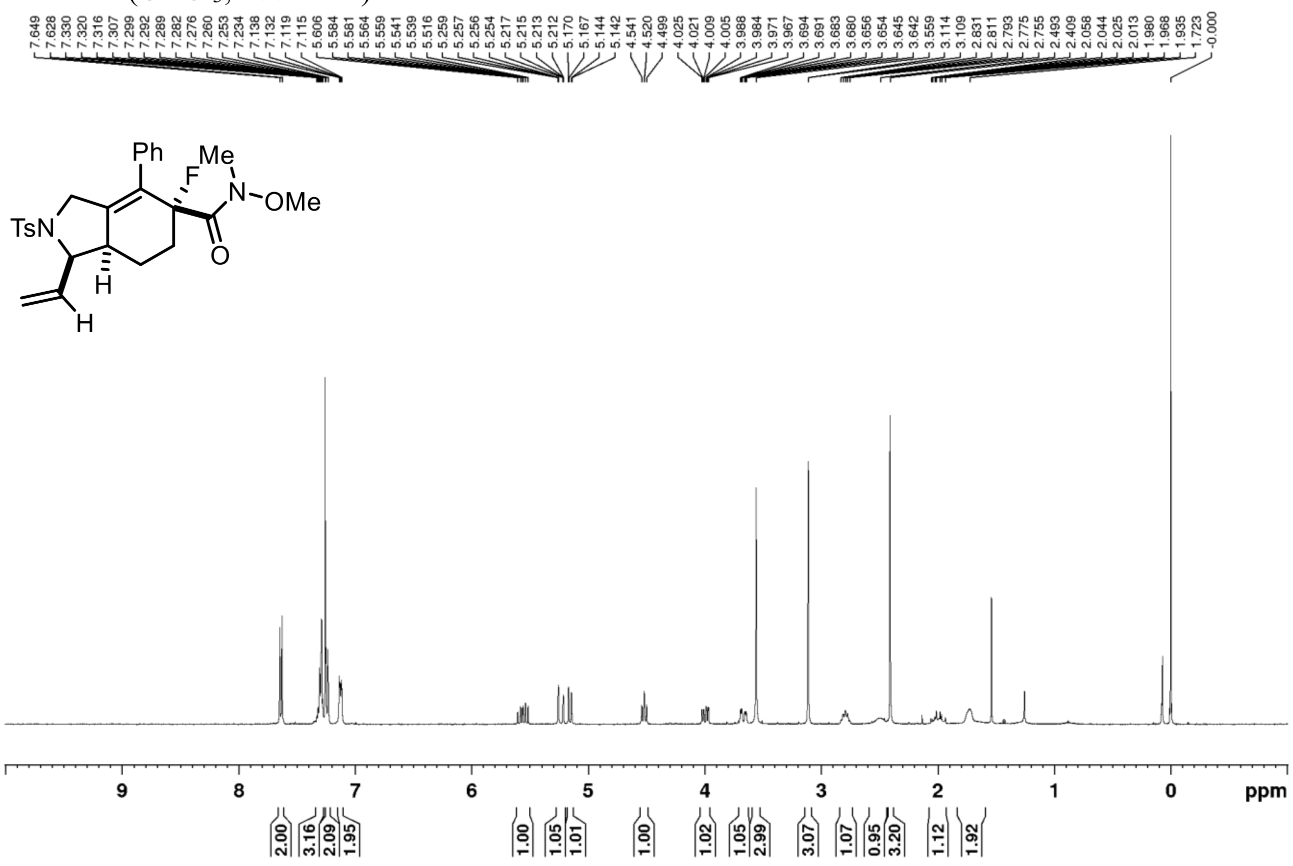

^{13}C NMR ($CDCl_3$, 101 MHz)

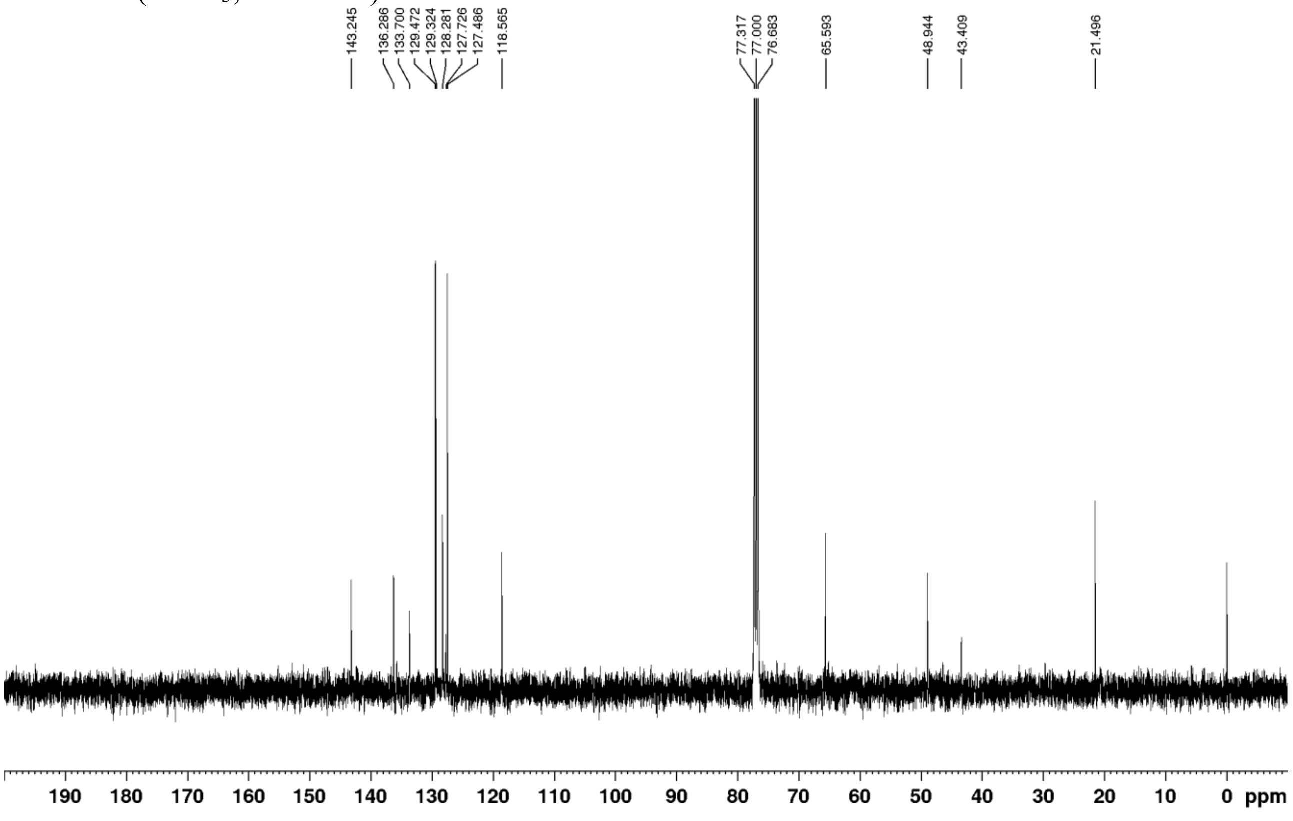

{(1*R*,5*R*,7a*R*)-5-Fluoro-4-(4-methoxyphenyl)-2-(4-methylphenyl)-1-vinyl-2,3,5,6,7,7a-hexahydro-1*H*-isoindol-5-yl}(pyrrolidin-1-yl)methanone (6ba)

Diastereomer mixture (93:7 d.r.)

^{1}H NMR ($CDCl_3$, 400 MHz)

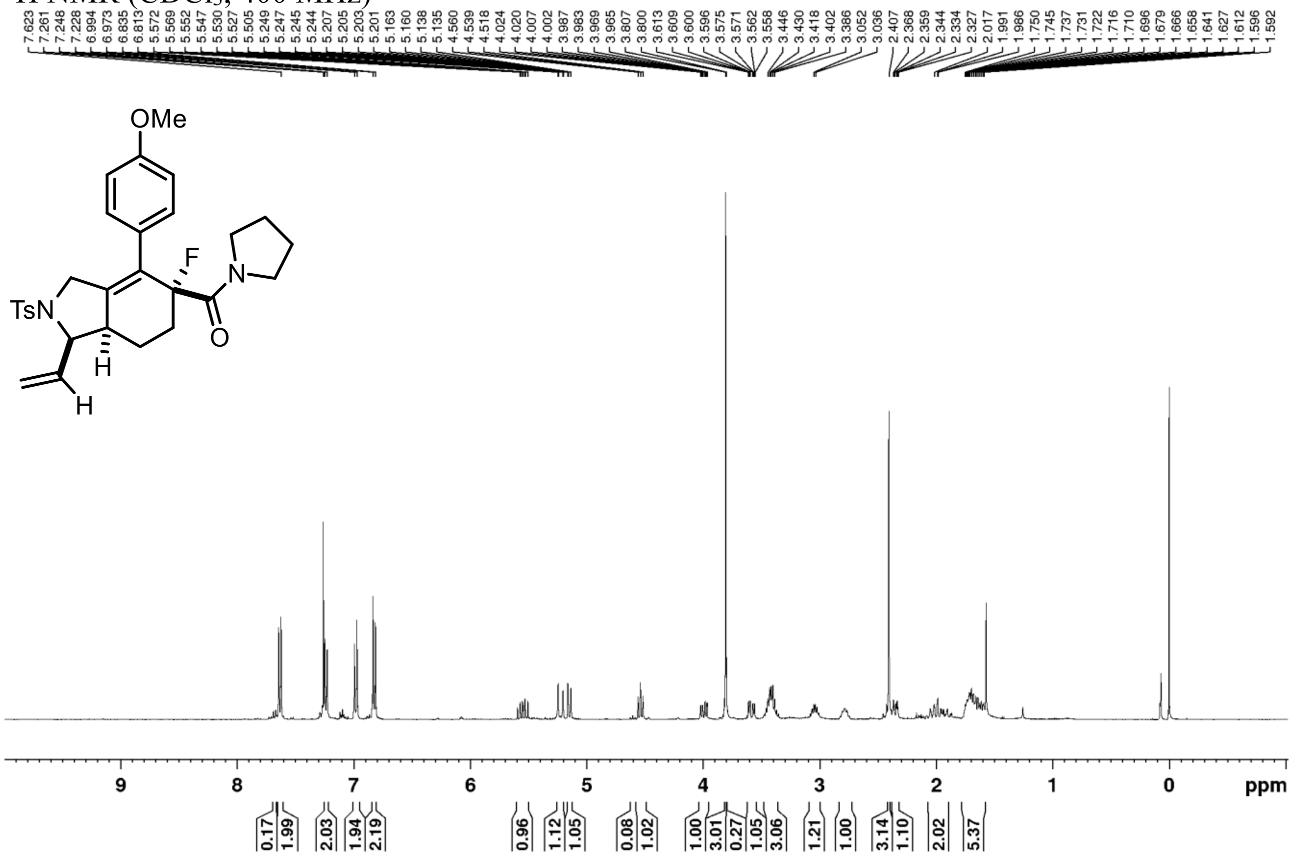

^{13}C NMR ($CDCl_3$, 101 MHz)

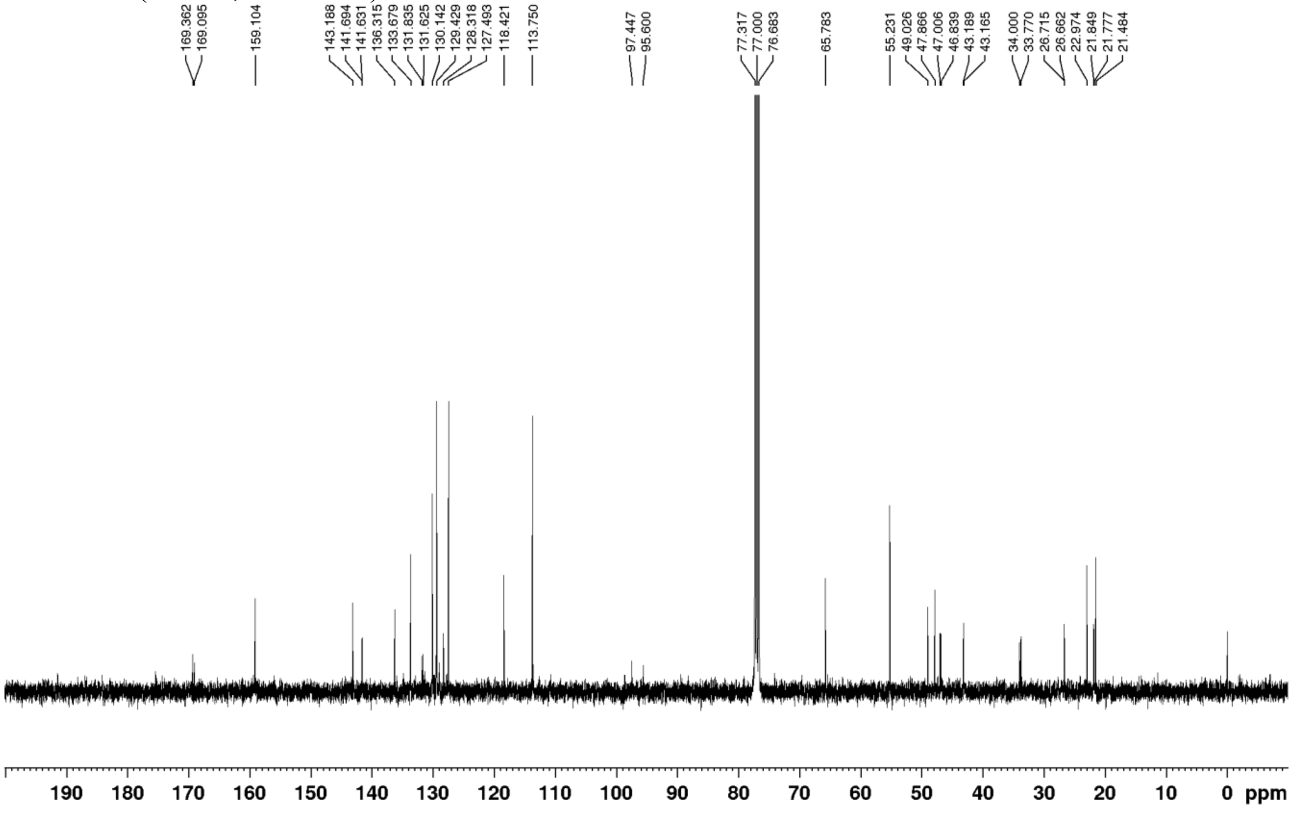

^{19}F NMR ($CDCl_3$, 377 MHz)

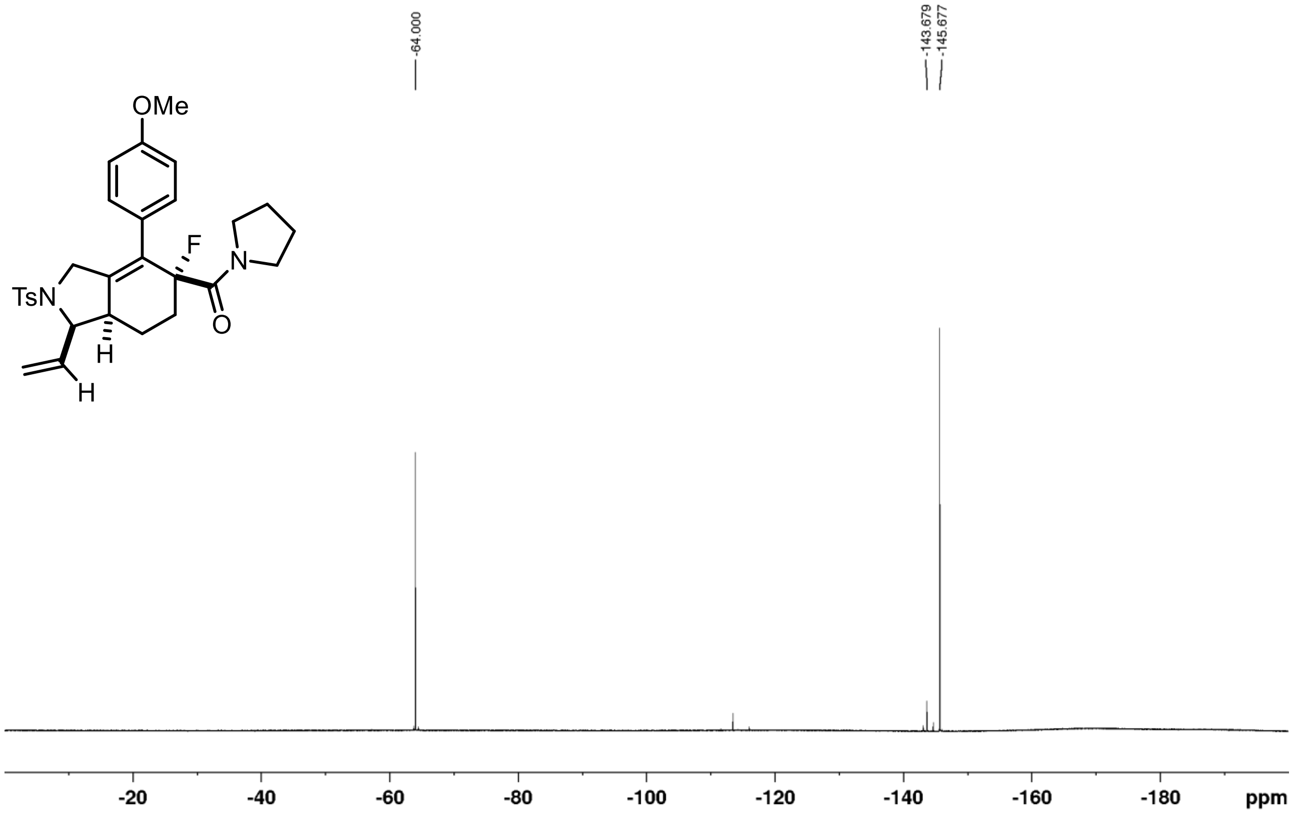

{(1*R*,5*R*,7a*R*)-4-(4-Chlorophenyl)-5-fluoro-2-(4-methylphenyl)-1-vinyl-2,3,5,6,7,7a-hexahydro-1*H*-isoindol-5-yl}(pyrrolidin-1-yl)methanone [6ca (major diastereomer)]

^{1}H NMR ($CDCl_3$, 400 MHz)

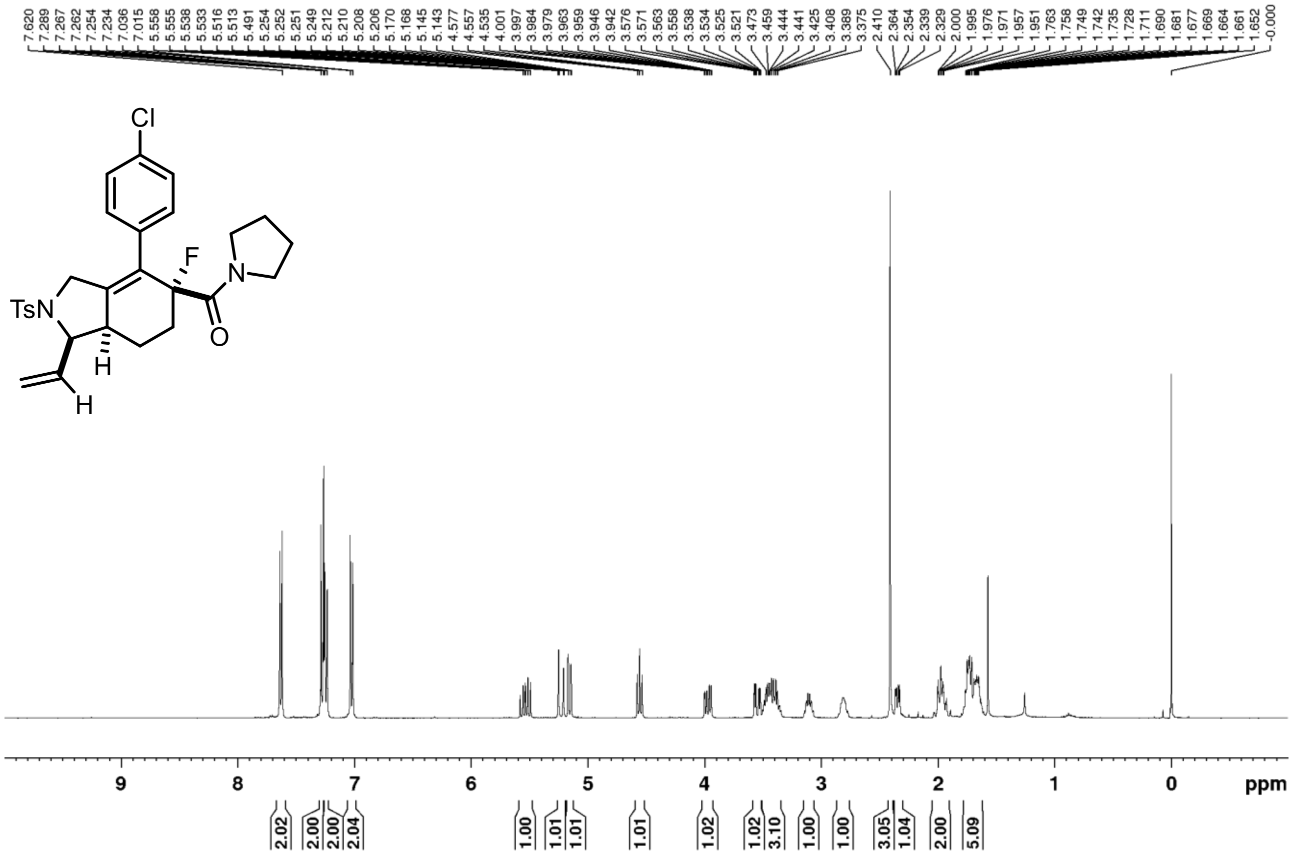

^{13}C NMR ($CDCl_3$, 101 MHz)

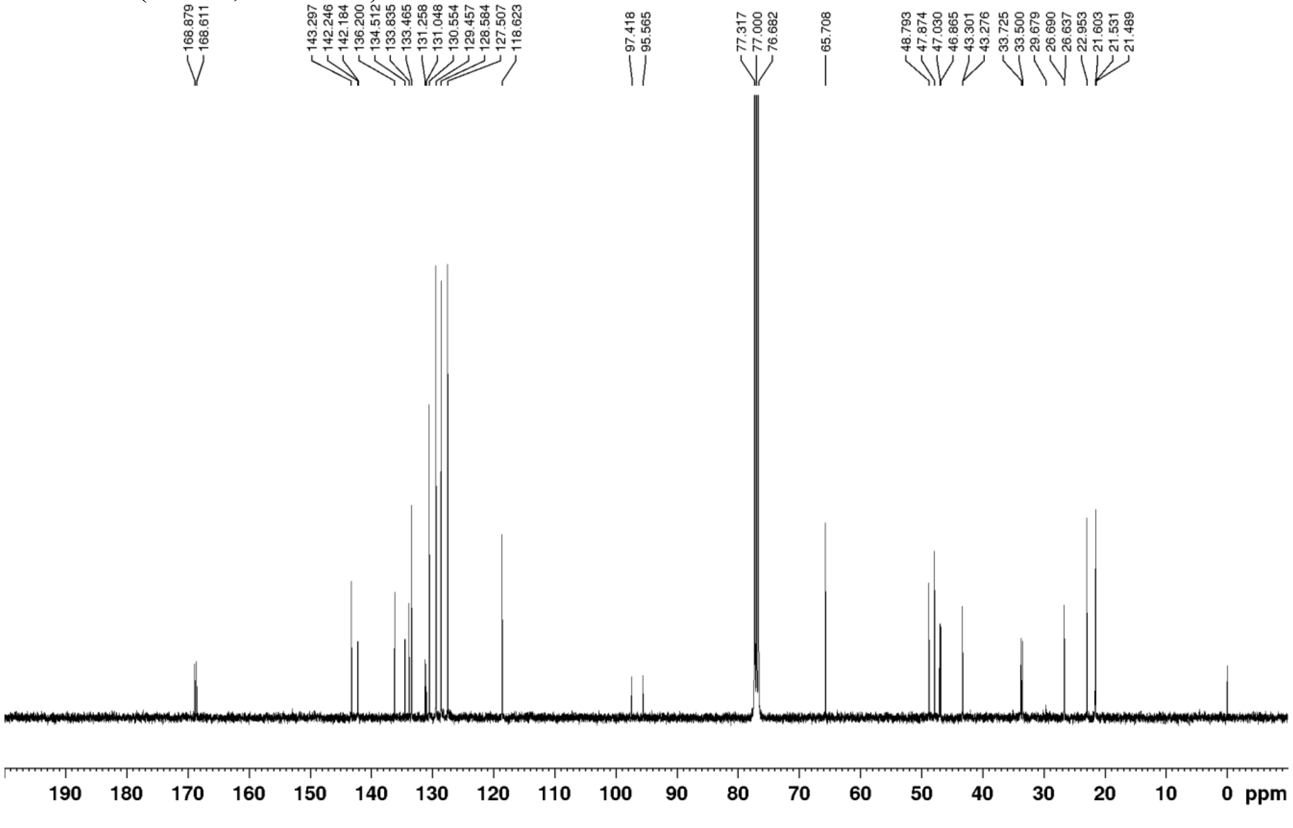

^{19}F NMR ($CDCl_3$, 377 MHz)

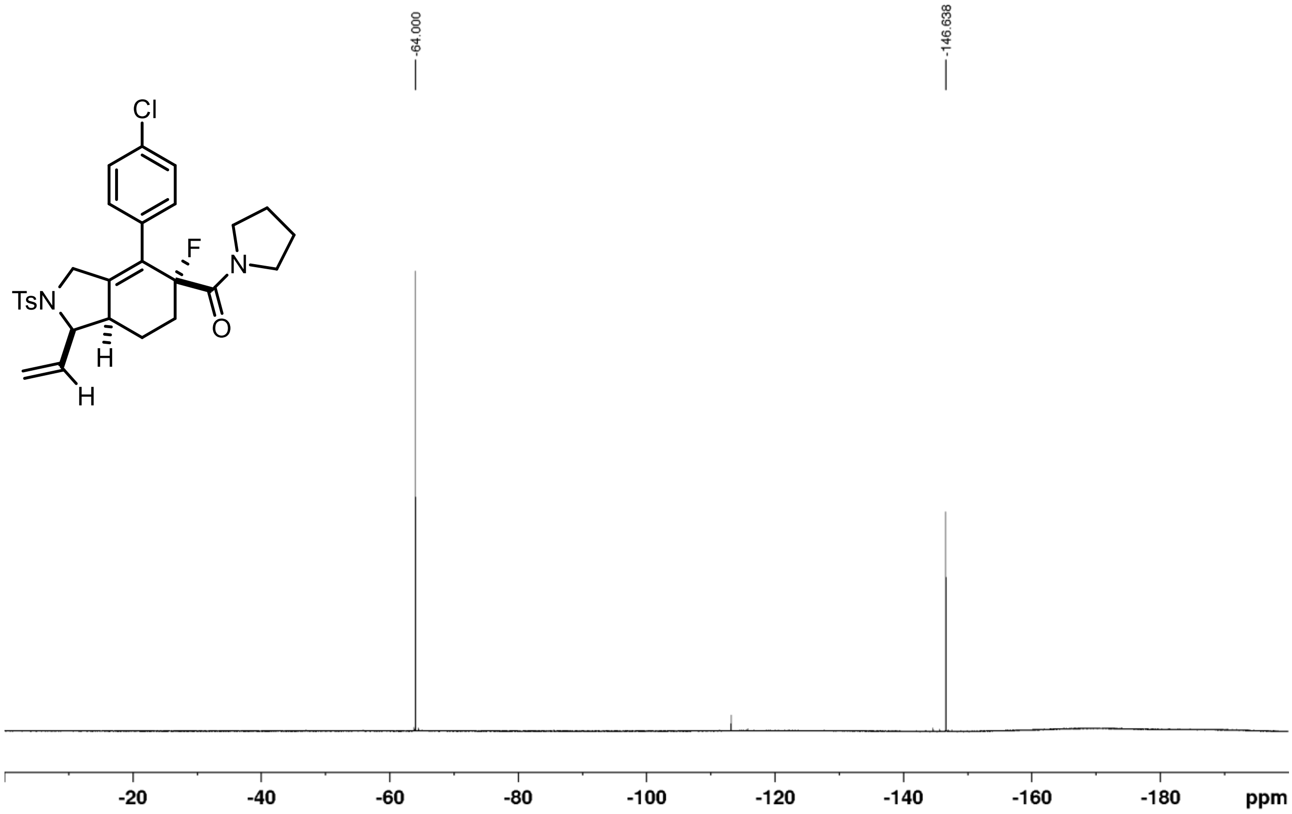

{(1*R*,5*S*,7a*R*)-2-(4-Methylphenyl)-4-phenyl-1-vinyl-2,3,5,6,7,7a-hexahydro-1*H*-isoindol-5-yl}(pyrrolidin-1-yl)methanone [6ad (major diastereomer)]

^{1}H NMR ($CDCl_3$, 400 MHz)

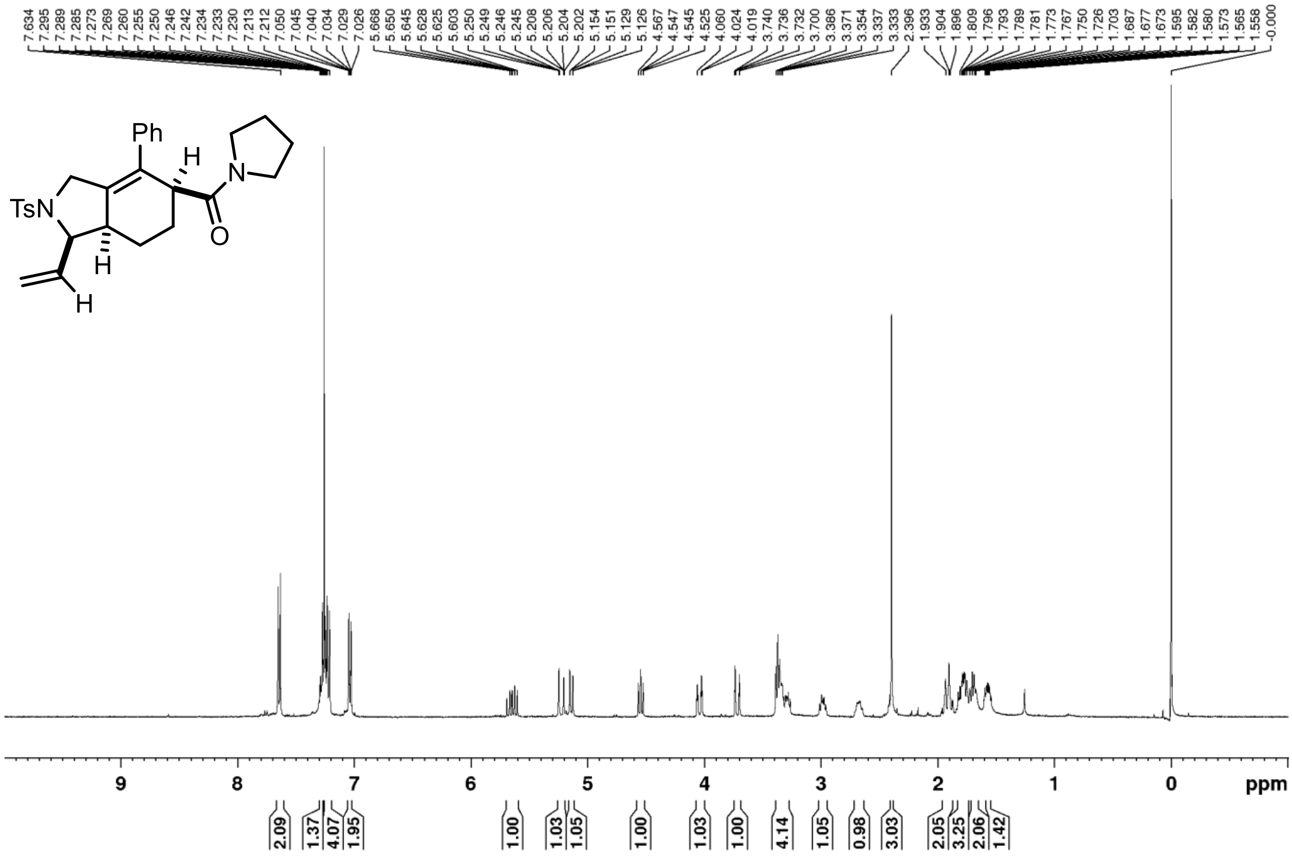

^{13}C NMR ($CDCl_3$, 101 MHz)

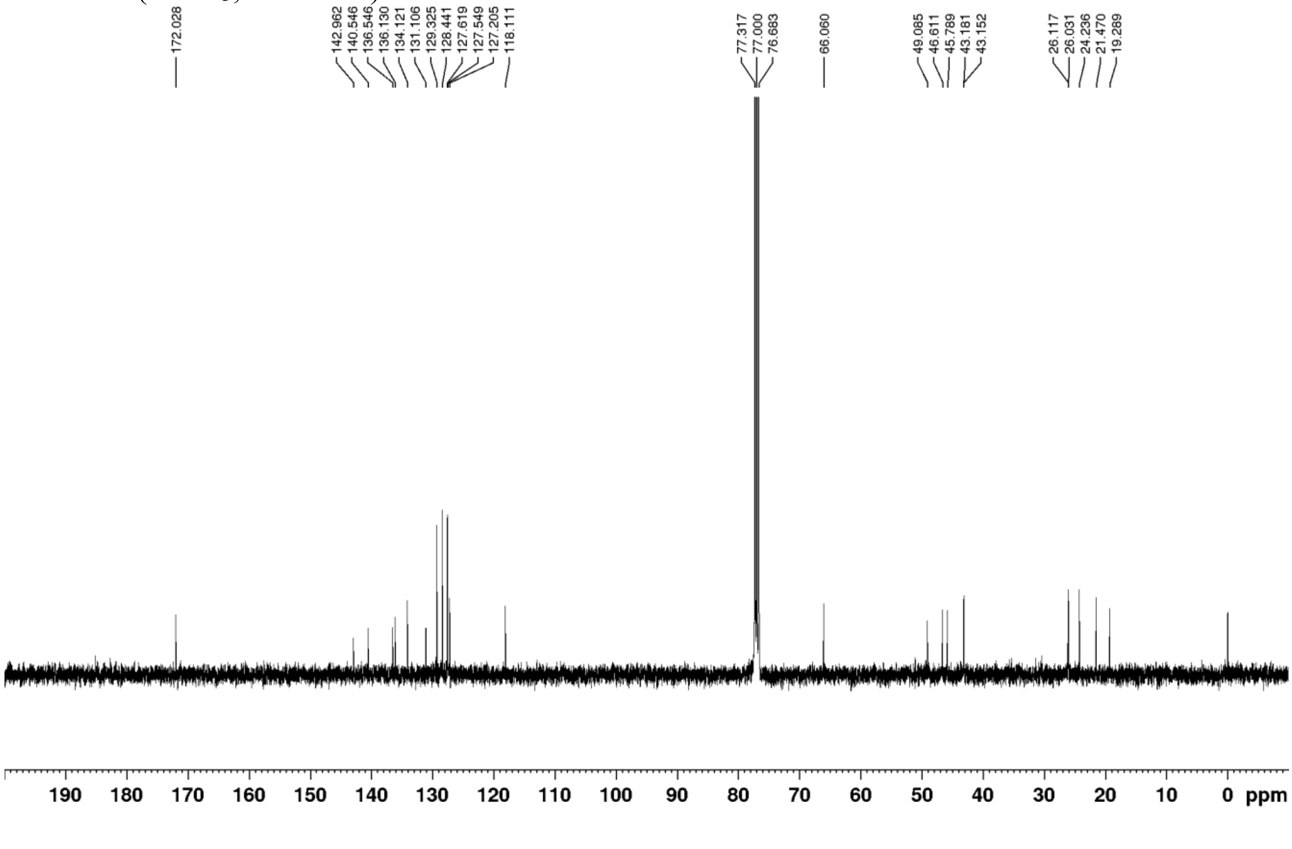

(1*R*,5*S*,7a*R*)-*N*,*N*-Diethyl-2-(4-methylphenyl)-4-phenyl-1-vinyl-2,3,5,6,7,7a-hexahydro-1*H*-isoindole-5-carboxamide (6ah)

Diastereomer mixture (93:7 d.r.)

^{1}H NMR ($CDCl_3$, 400 MHz)

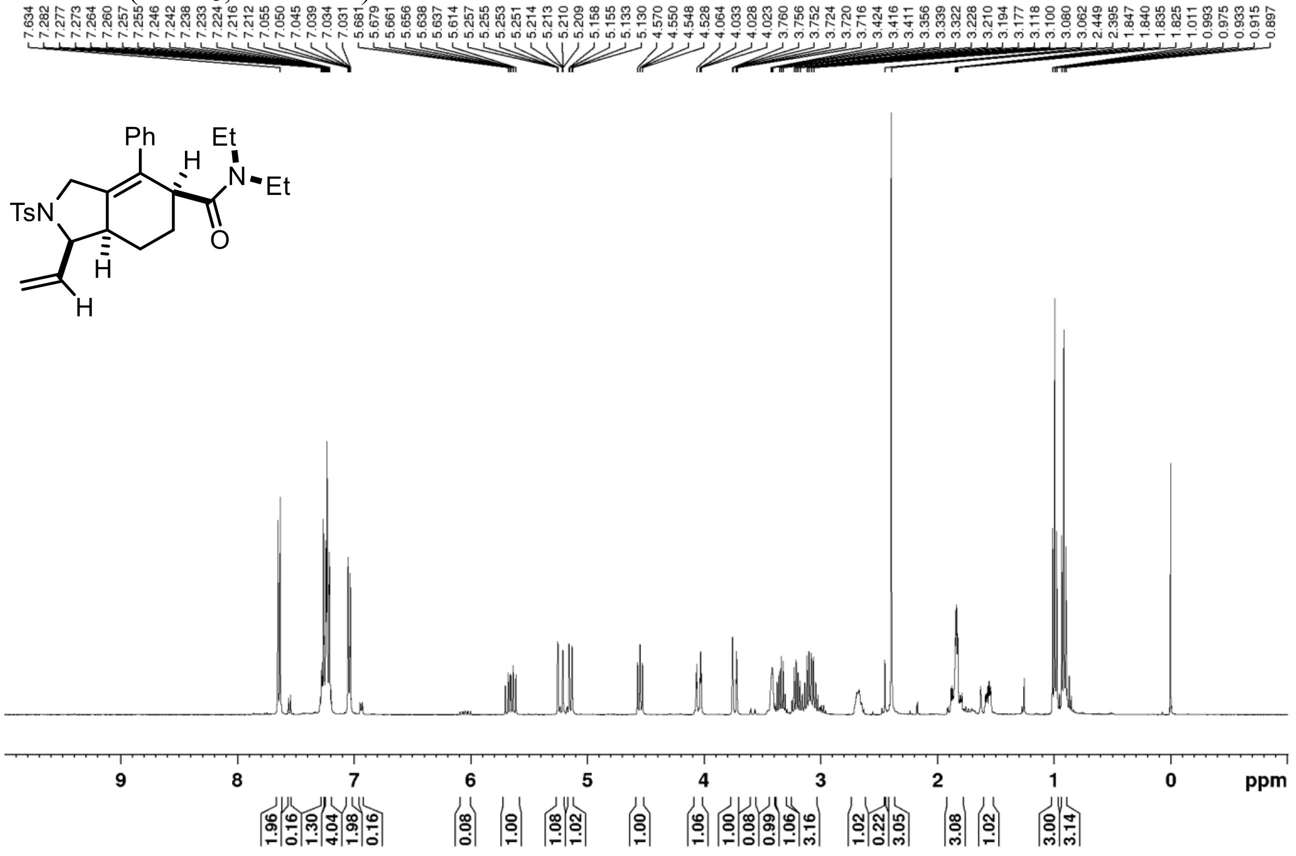

^{13}C NMR ($CDCl_3$, 101 MHz)

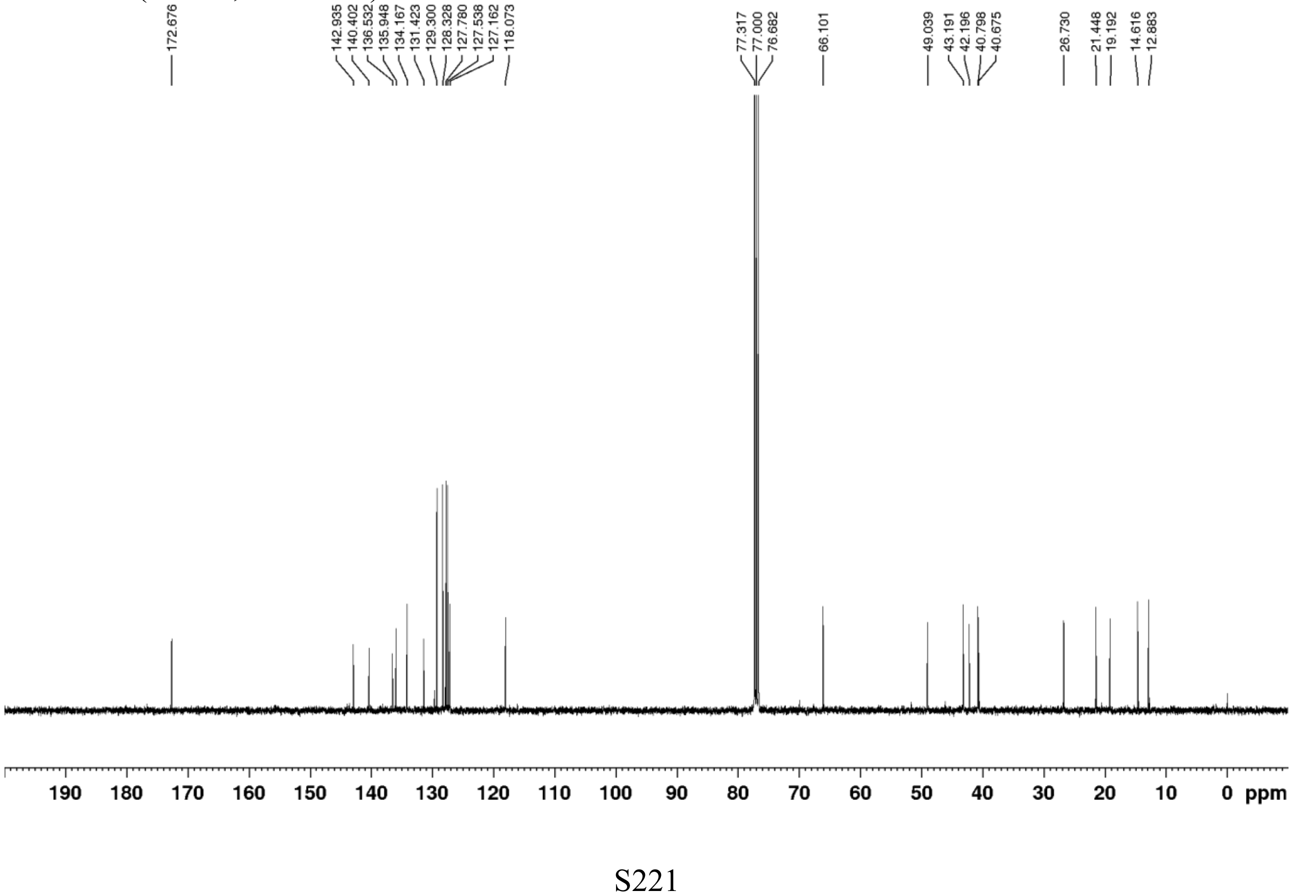

(1*R*,5*S*,7a*R*)-*N*-Methyl-2-(4-methylphenyl)-*N*,4-diphenyl-1-vinyl-2,3,5,6,7,7a-hexahydro-1*H*-isoindole-5-carboxamide (6ag)

Diastereomer mixture (93:7 d.r.)

^{1}H NMR (CDCl$_3$, 400 MHz)

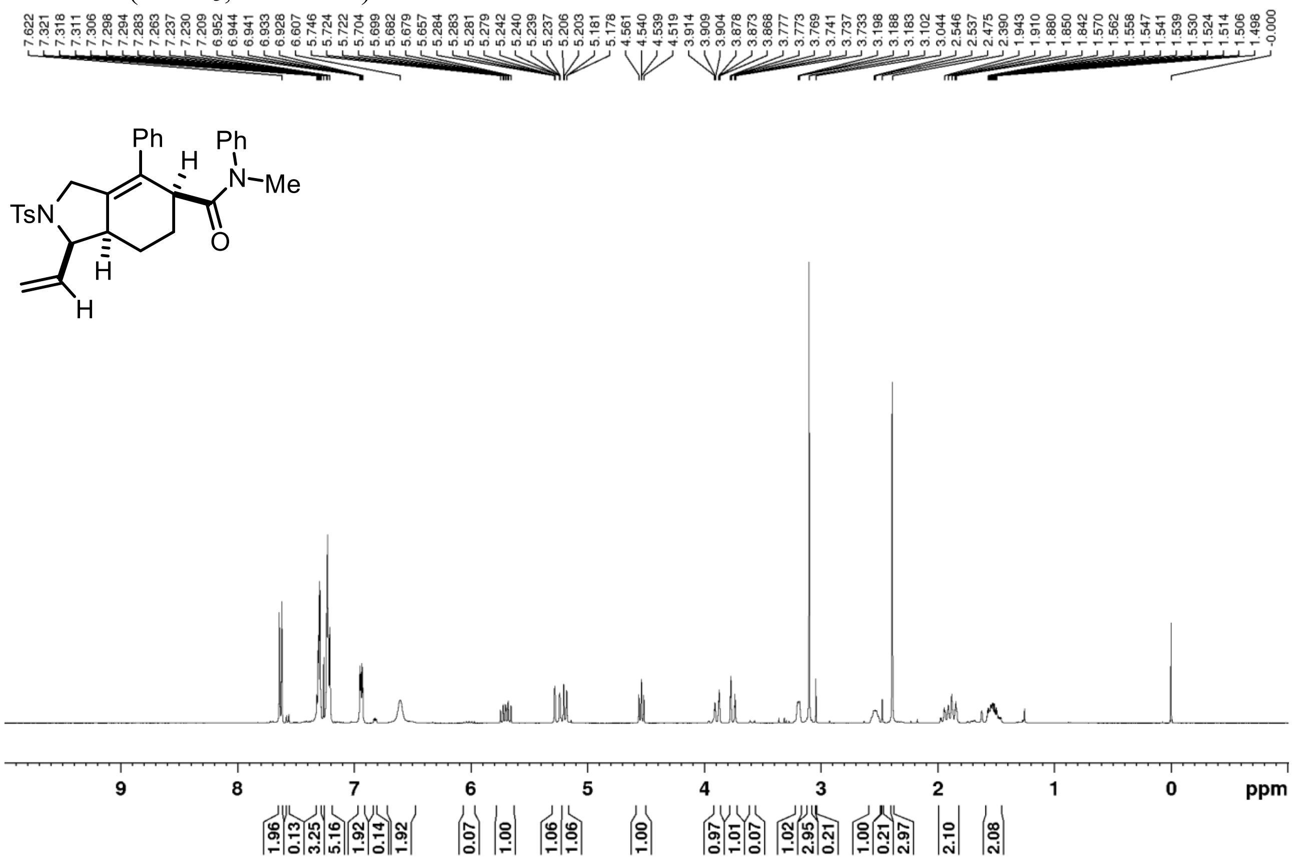

^{13}C NMR (CDCl$_3$, 101 MHz)

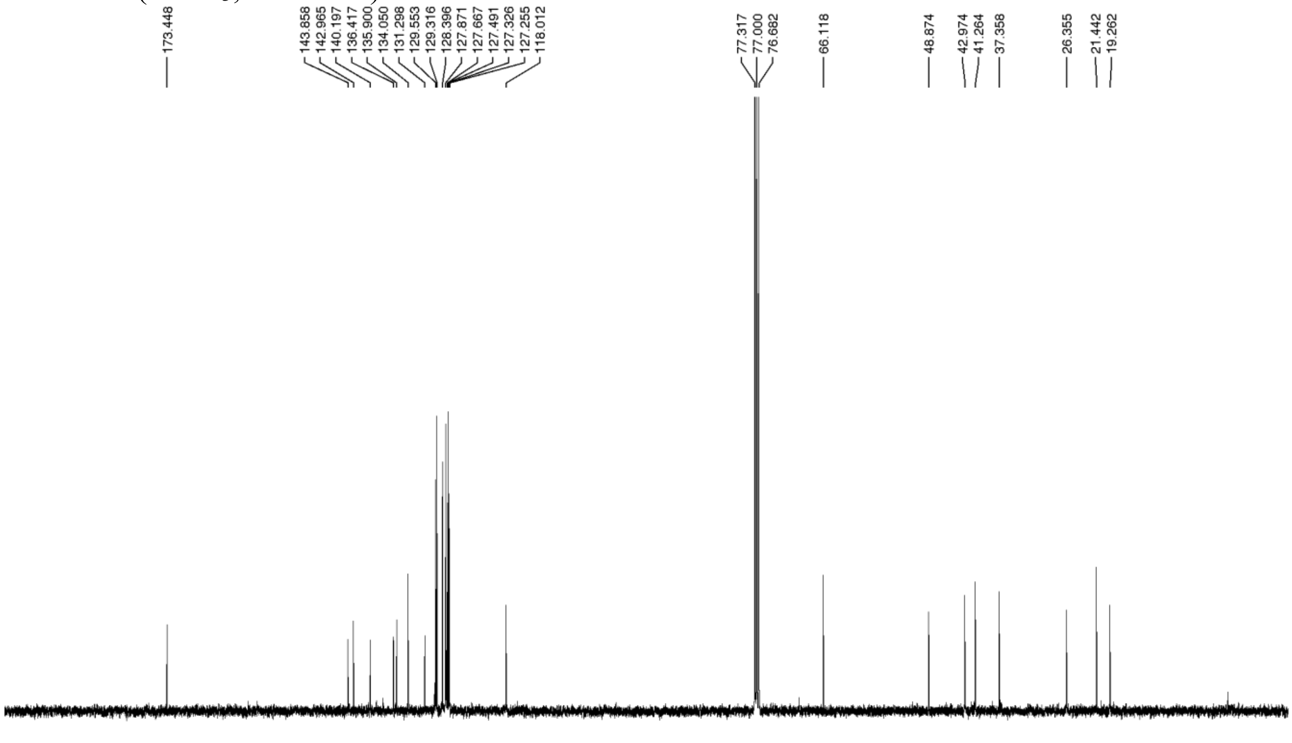

(1*R*,5*S*,7a*R*)-*N*,*N*,4-Triphenyl-2-(4-methylphenyl)-1-vinyl-2,3,5,6,7,7a-hexahydro-1*H*-isoindole-5-carboxamide (6ak)

Diastereomer mixture (97:3 d.r.)

^{1}H NMR (CDCl$_3$, 400 MHz)

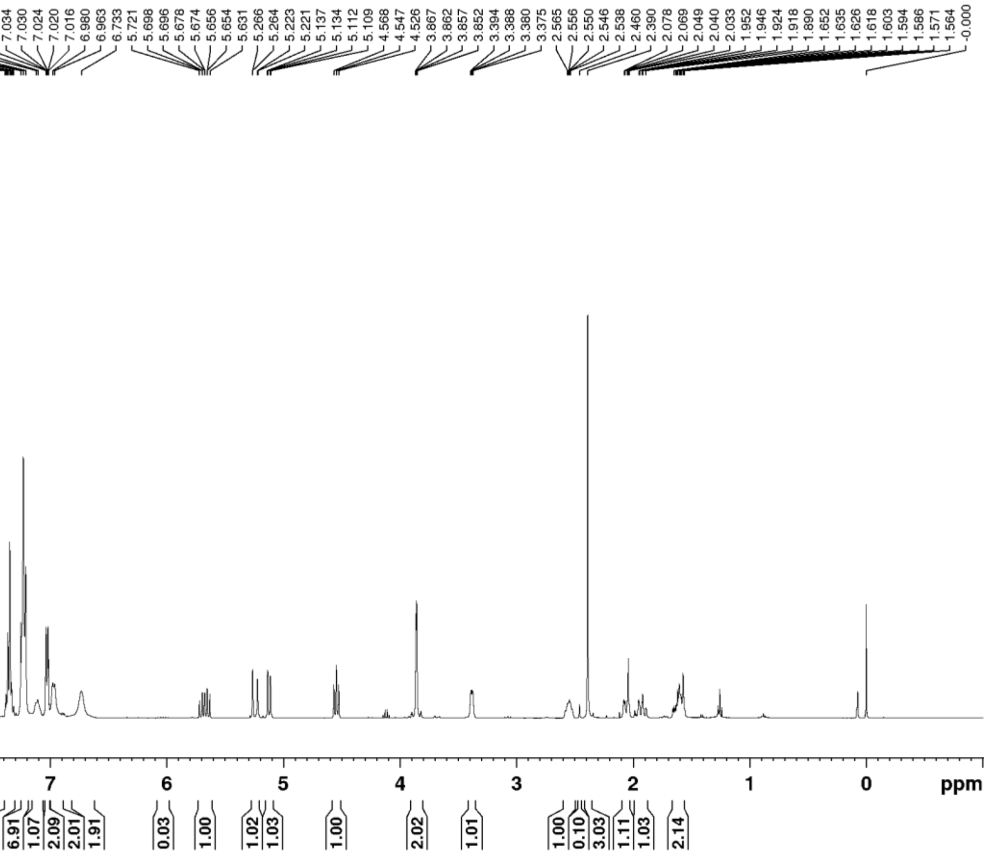

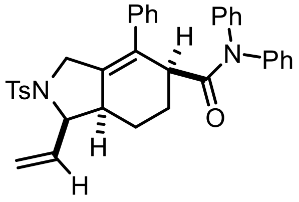

^{13}C NMR (CDCl$_3$, 101 MHz)

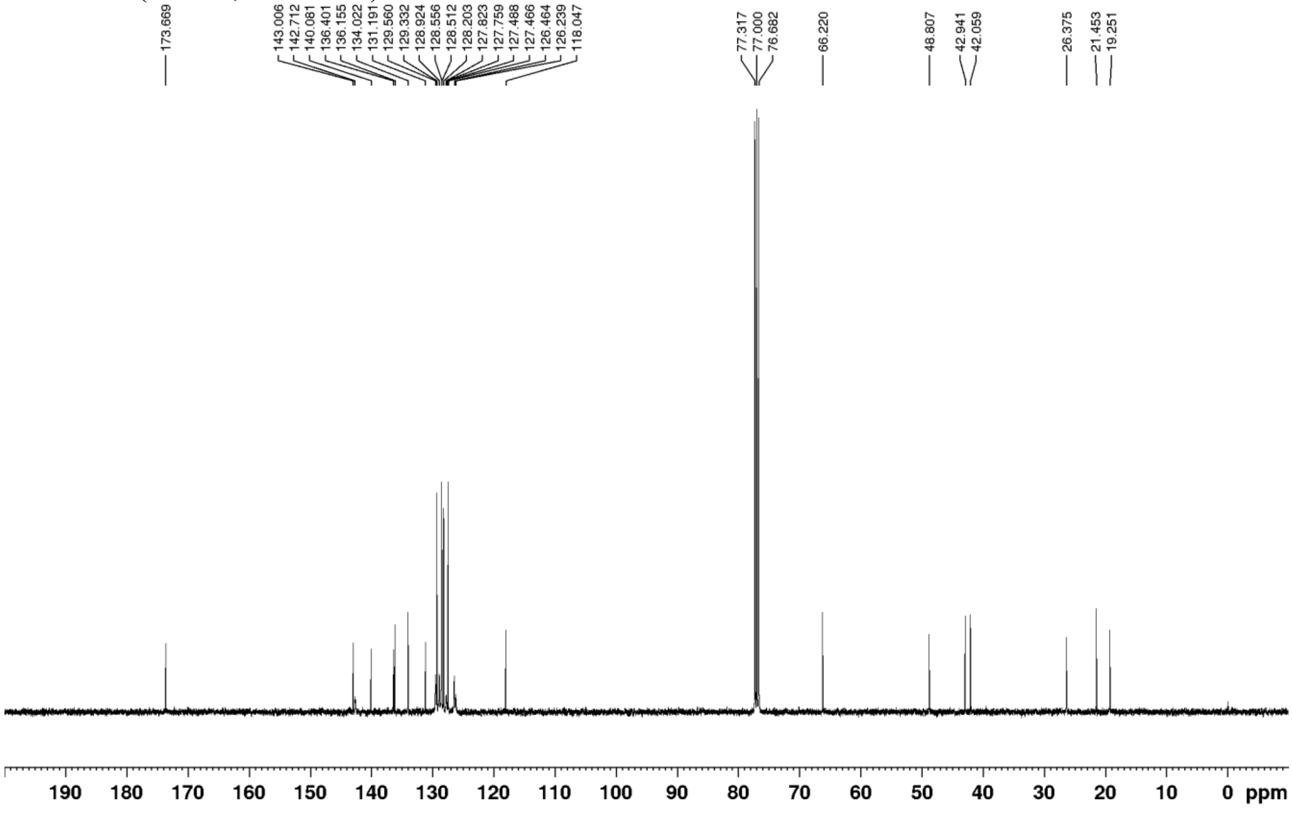

{(1*R*,5*S*,7a*R*)-4-(4-Methoxyphenyl)-2-(4-methylphenyl)-1-vinyl-2,3,5,6,7,7a-hexahydro-1*H*-isoindol-5-yl}(pyrrolidin-1-yl)methanone (6bd)

Diastereomer mixture (93:7 d.r.)

^{1}H NMR ($CDCl_3$, 400 MHz)

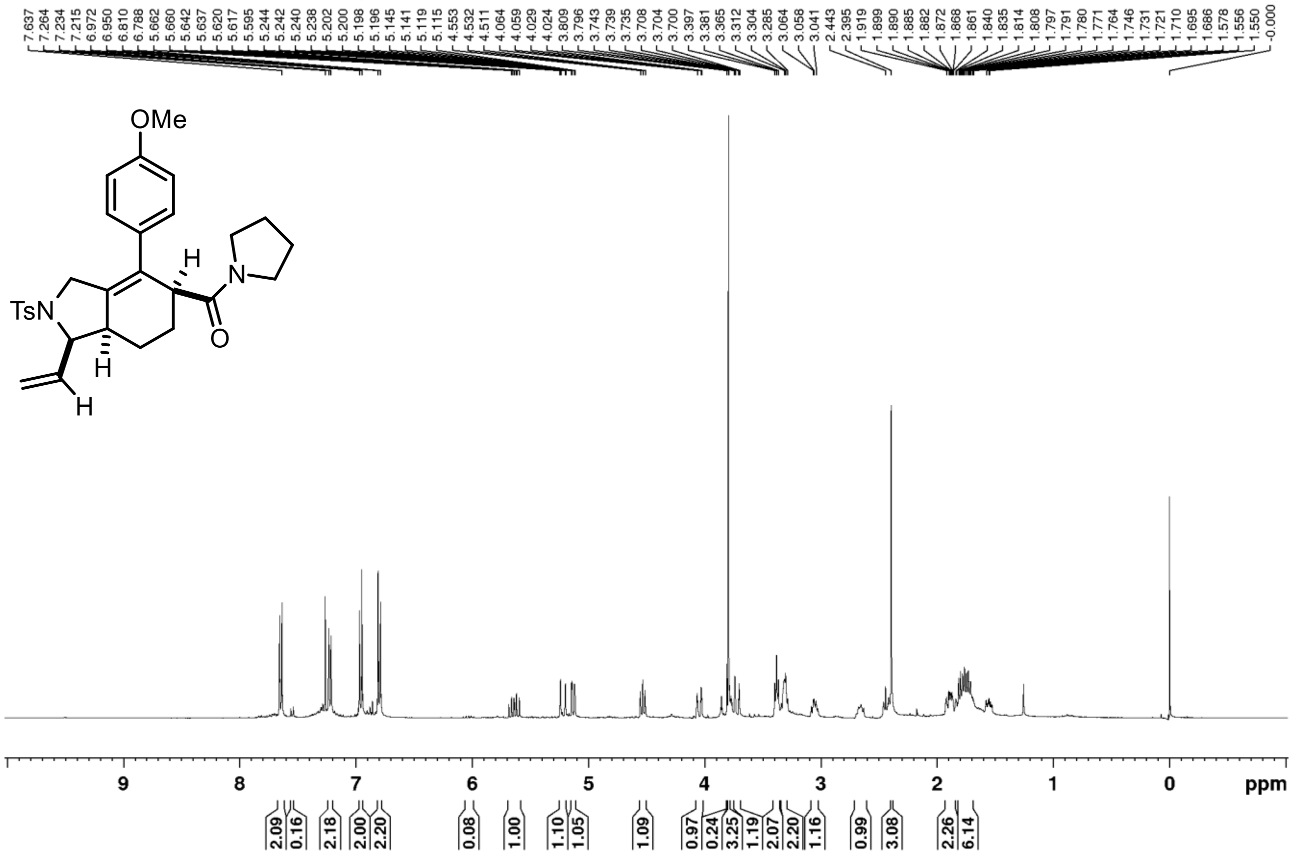

^{13}C NMR ($CDCl_3$, 101 MHz)

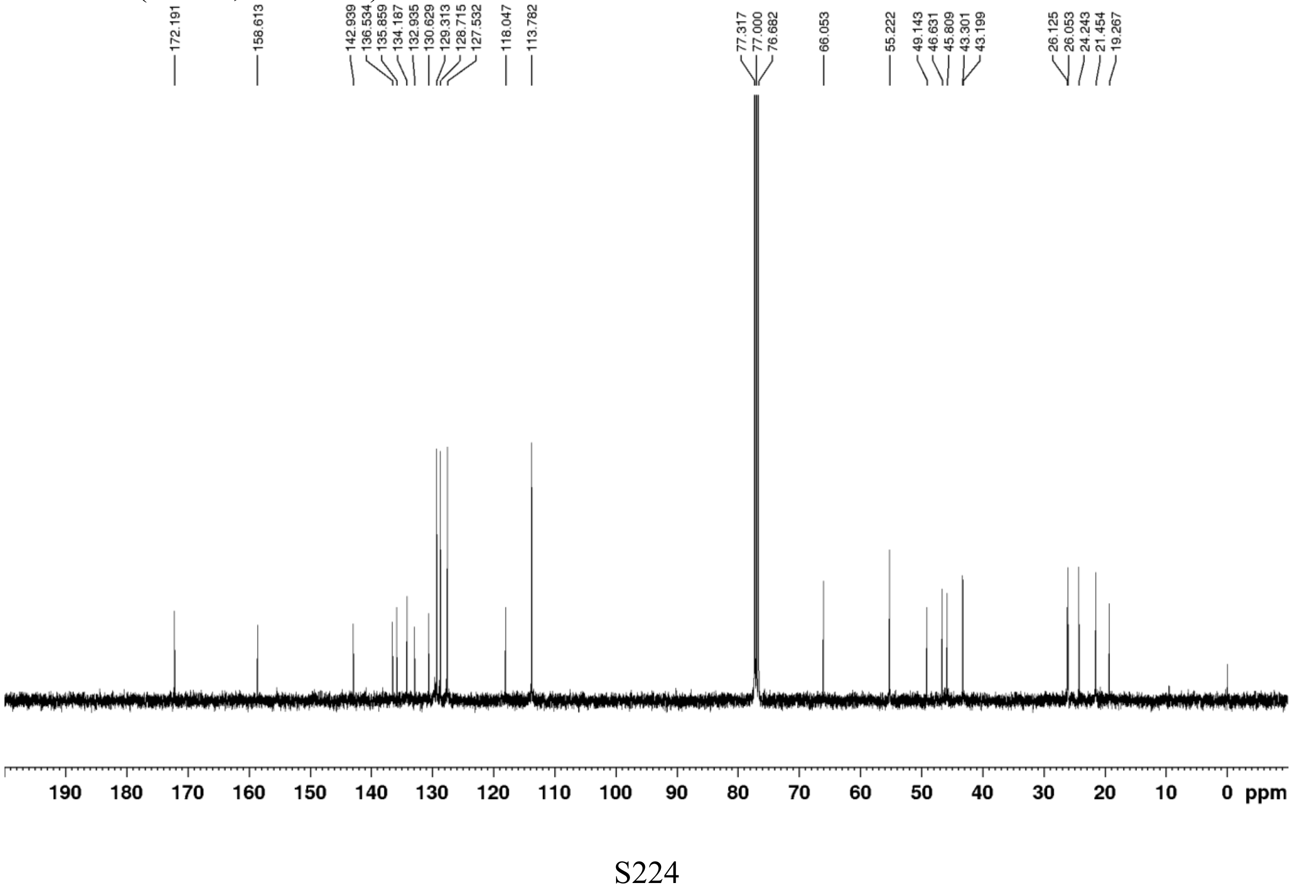

{(1*R*,5*S*,7a*R*)-4-(4-Chlorophenyl)-2-(4-methylphenyl)-1-vinyl-2,3,5,6,7,7a-hexahydro-1*H*-isoindol-5-yl)(pyrrolidin-1-yl)methanone (6cd)

Diastereomer mixture (93:7 d.r.)

^{1}H NMR (CDCl$_3$, 400 MHz)

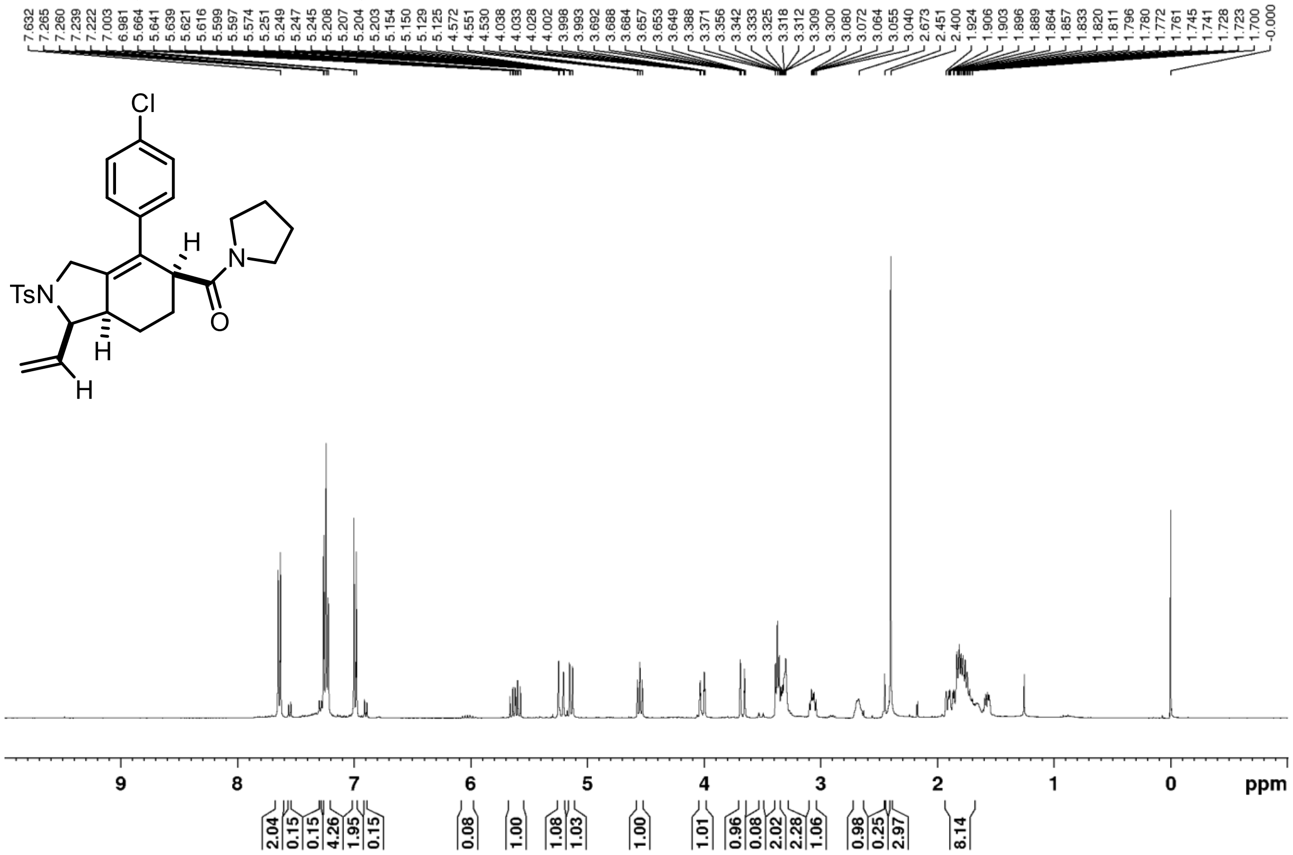

^{13}C NMR (CDCl$_3$, 101 MHz)

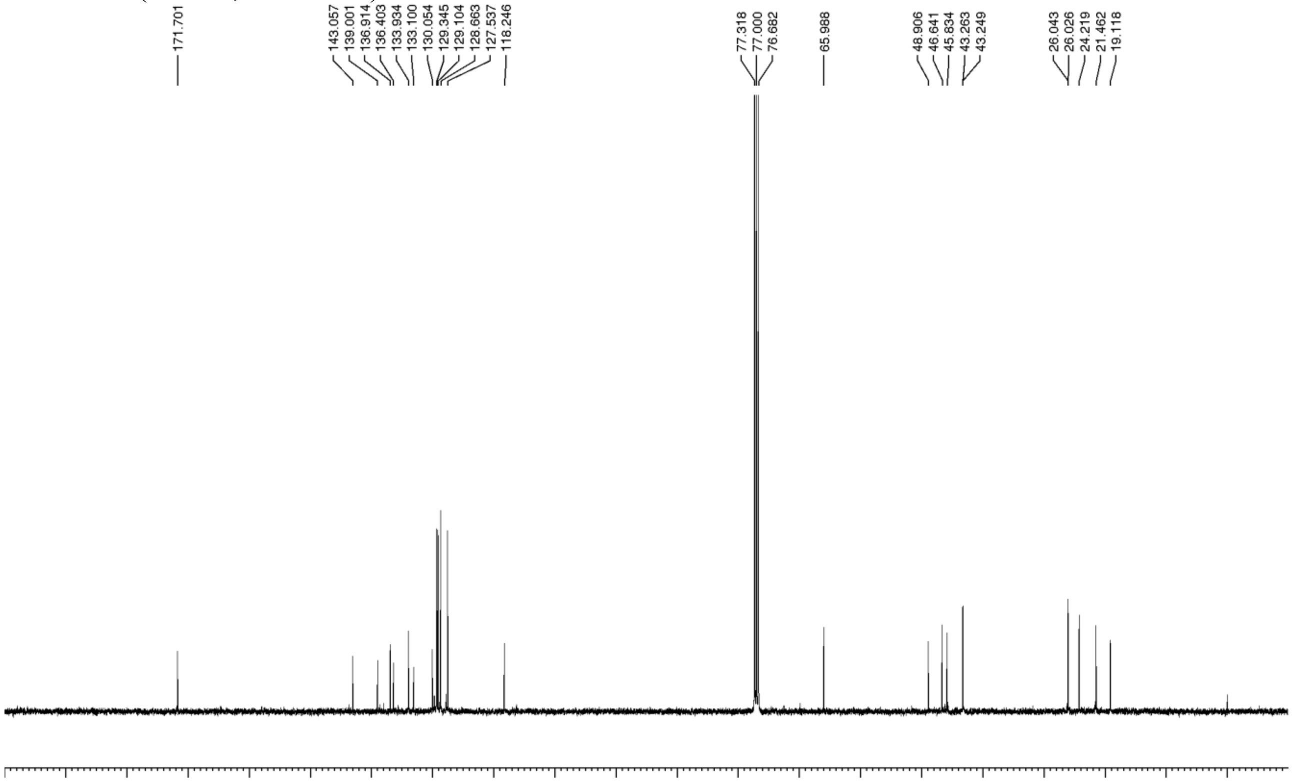

(1*R*,5*S*,7a*R*)-4-(4-Bromophenyl)-2-(4-methylphenyl)-*N*,*N*-diphenyl-1-vinyl-2,3,5,6,7,7a-hexahydro-1*H*-isoindole-5-carboxamide (6dk)

^{1}H NMR (CDCl$_3$, 400 MHz)

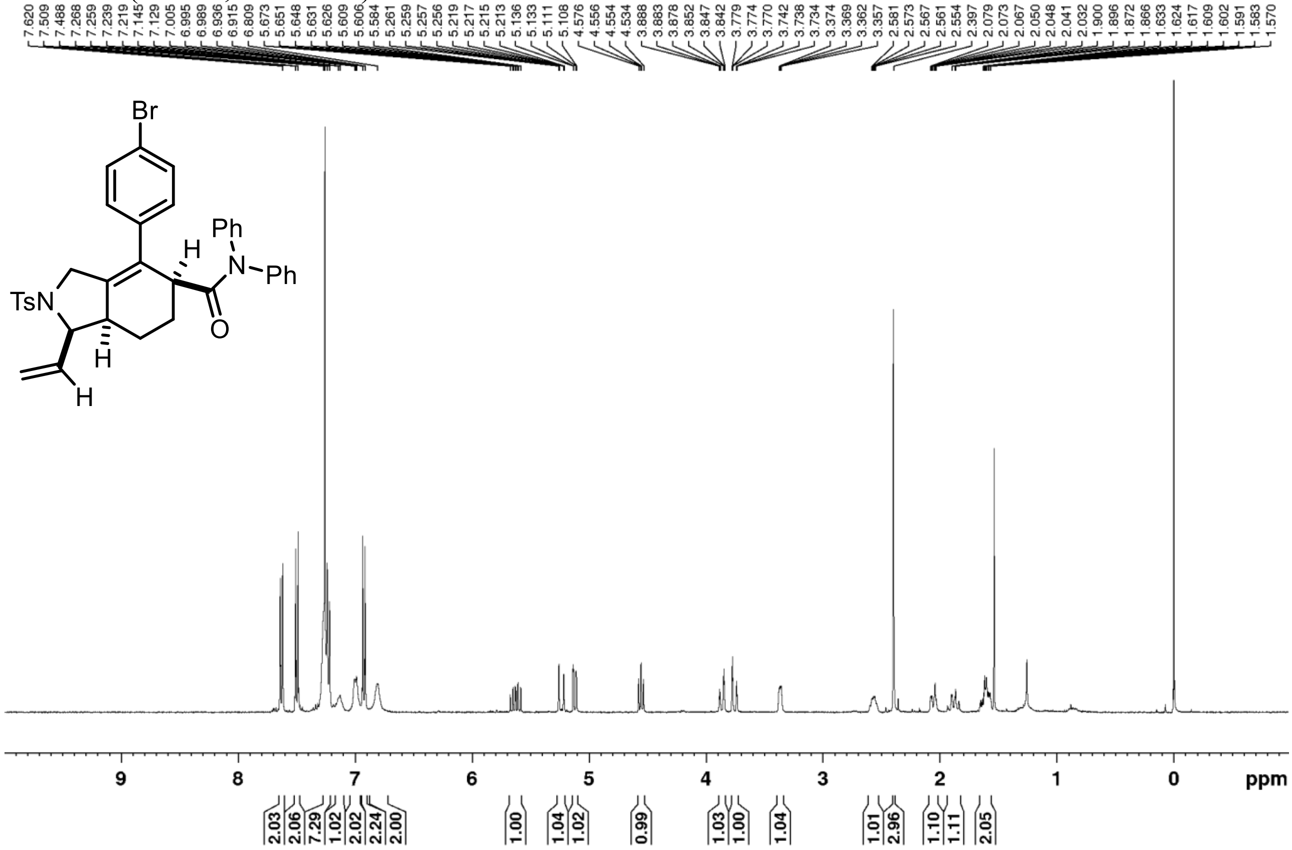

^{13}C NMR (CDCl$_3$, 101 MHz)

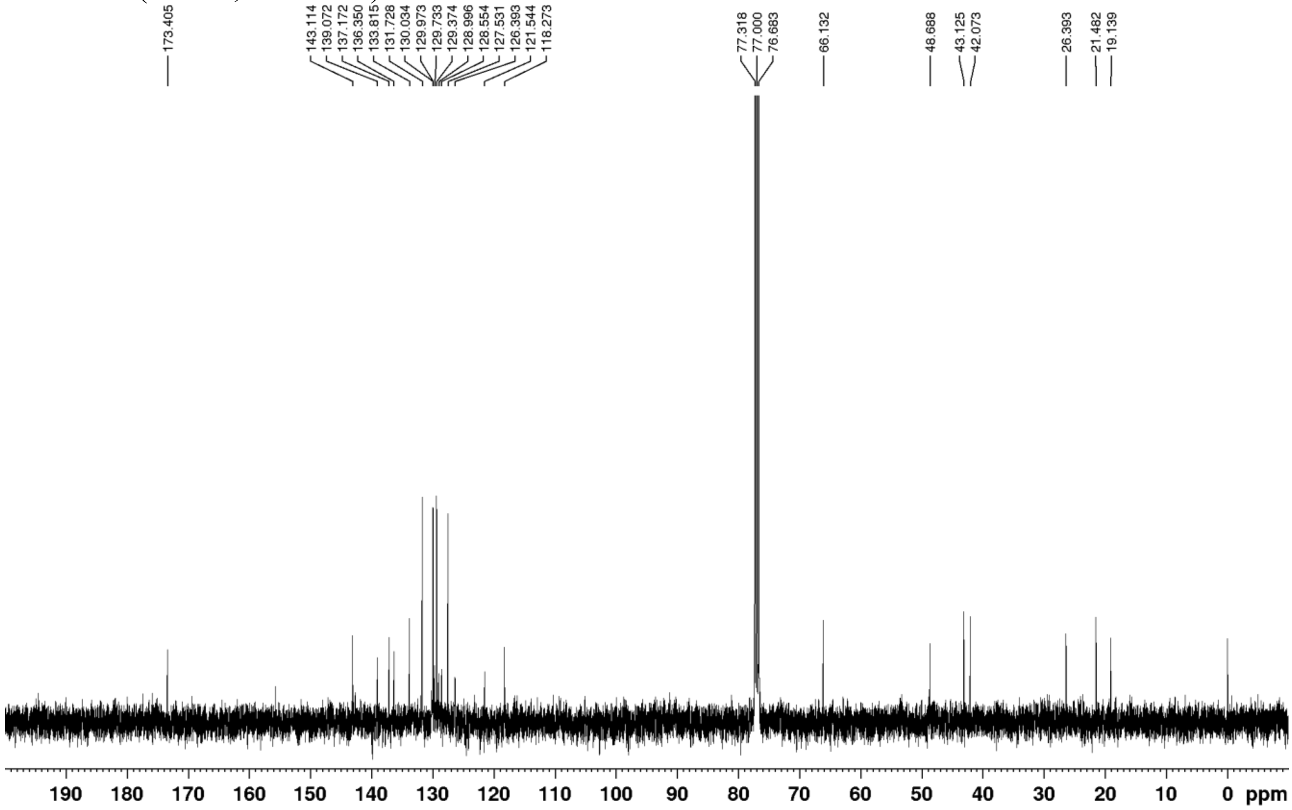

{(1*R*,5*S*,7a*R*)-4-Methyl-2-(4-methylphenyl)-1-vinyl-2,3,5,6,7,7a-hexahydro-1*H*-isoindol-5-yl}(pyrrolidin-1-yl)methanone (6ed)

^{1}H NMR ($CDCl_3$, 400 MHz)

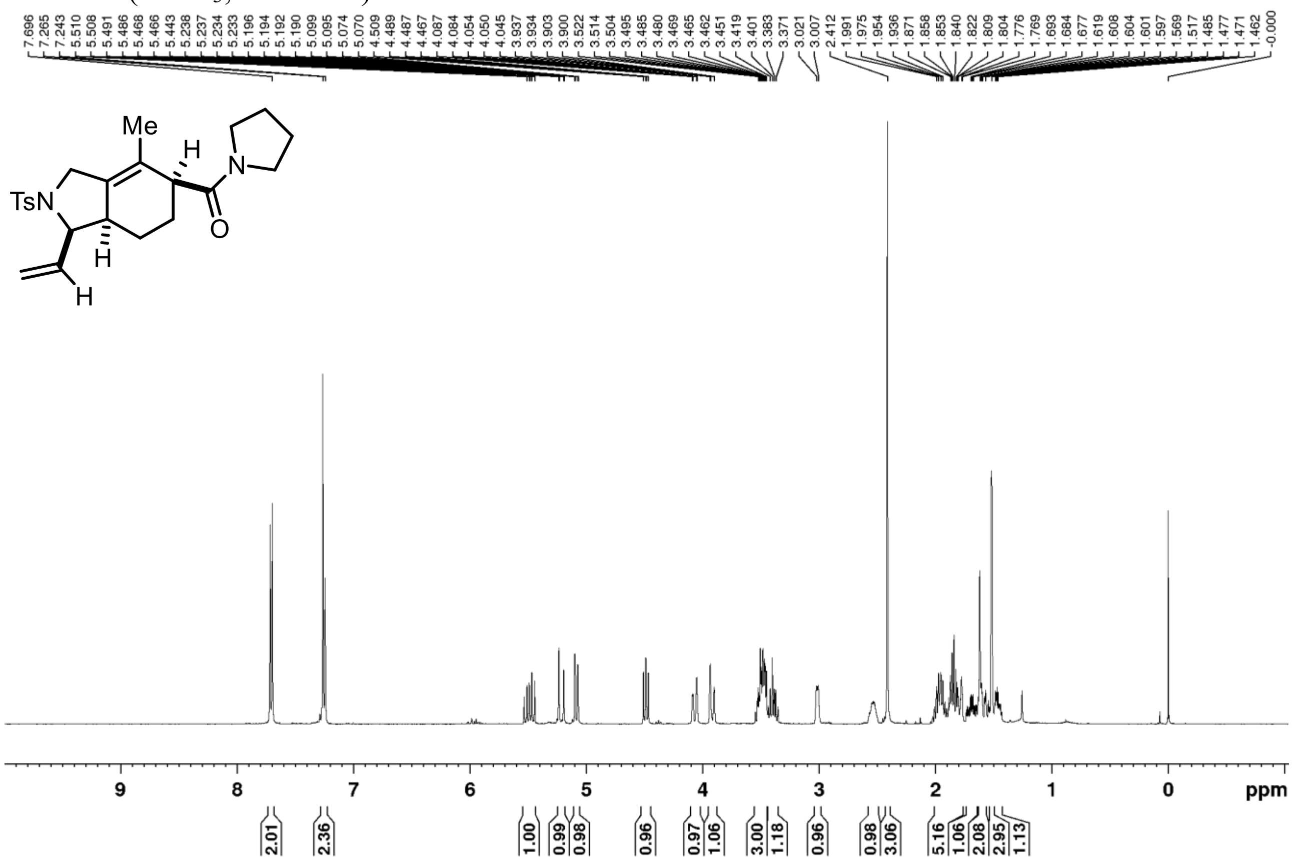

^{13}C NMR ($CDCl_3$, 101 MHz)

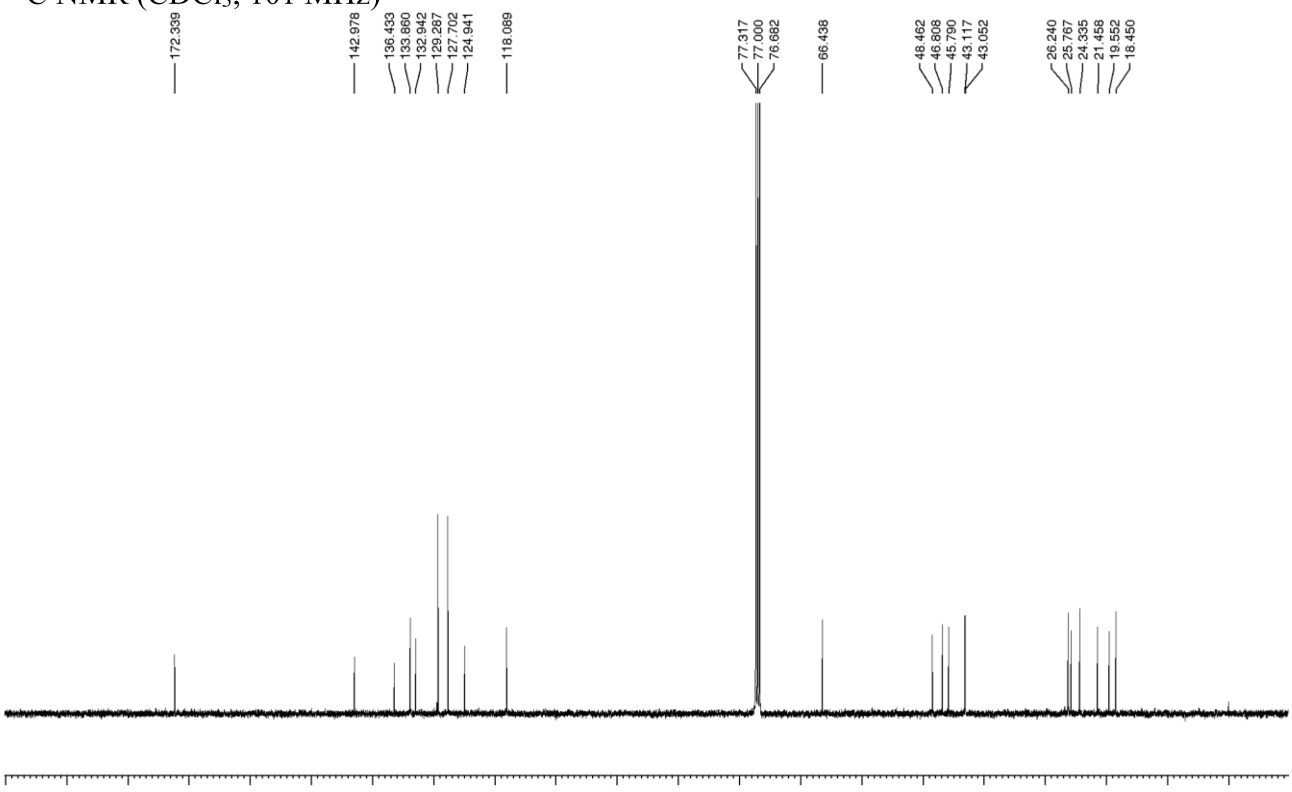

{(1*R*,5*S*,7a*R*)-4-Butyl-2-(4-methylphenyl)-1-vinyl-2,3,5,6,7,7a-hexahydro-1*H*-isoindol-5-yl}(pyrrolidin-1-yl)methanone (6fd)

^{1}H NMR (CDCl$_3$, 400 MHz)

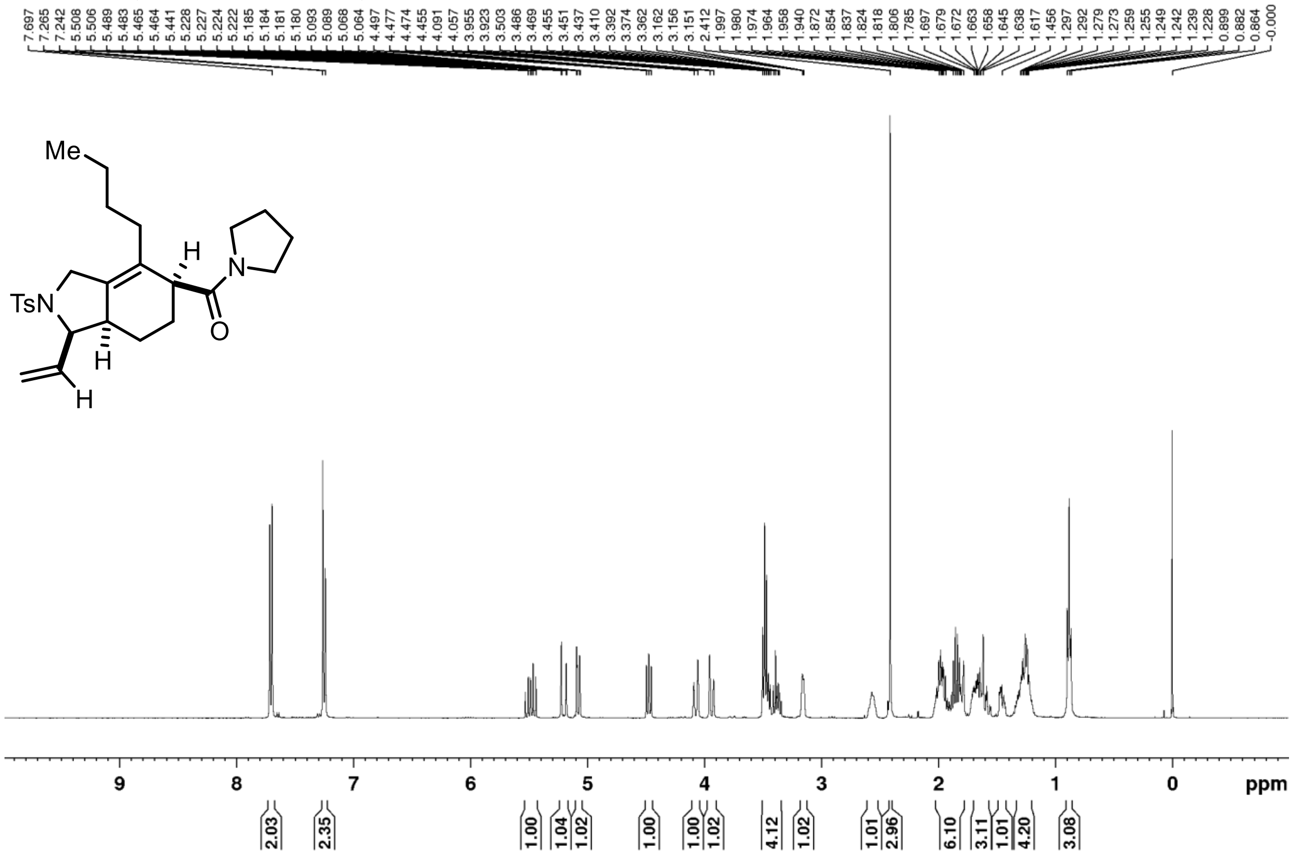

^{13}C NMR (CDCl$_3$, 101 MHz)

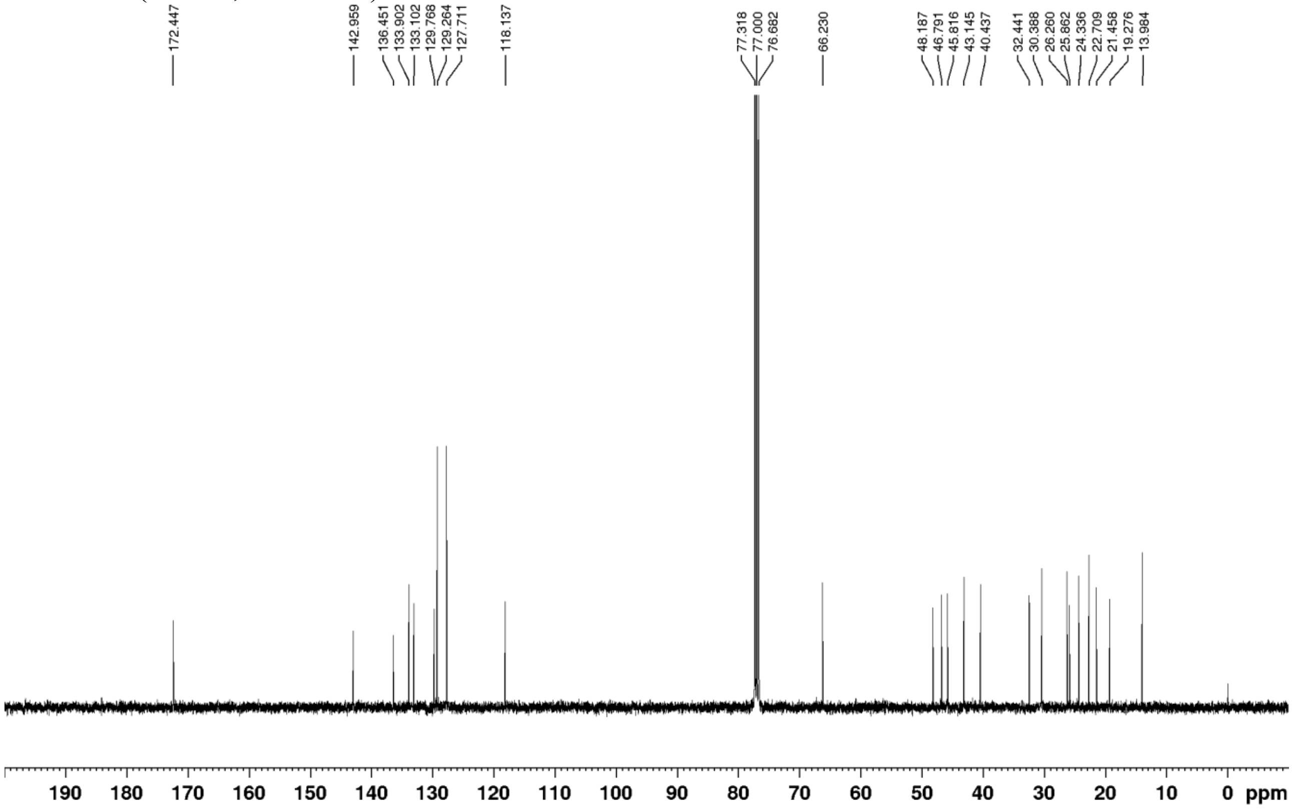

{(1*R*,5*R*,7a*R*)-5-Fluoro-4-phenyl-1-vinyl-1,3,5,6,7,7a-hexahydroisobenzofuran-5-yl}(pyrrolidin-1-yl)methanone (6ga)

^{1}H NMR ($CDCl_3$, 400 MHz)

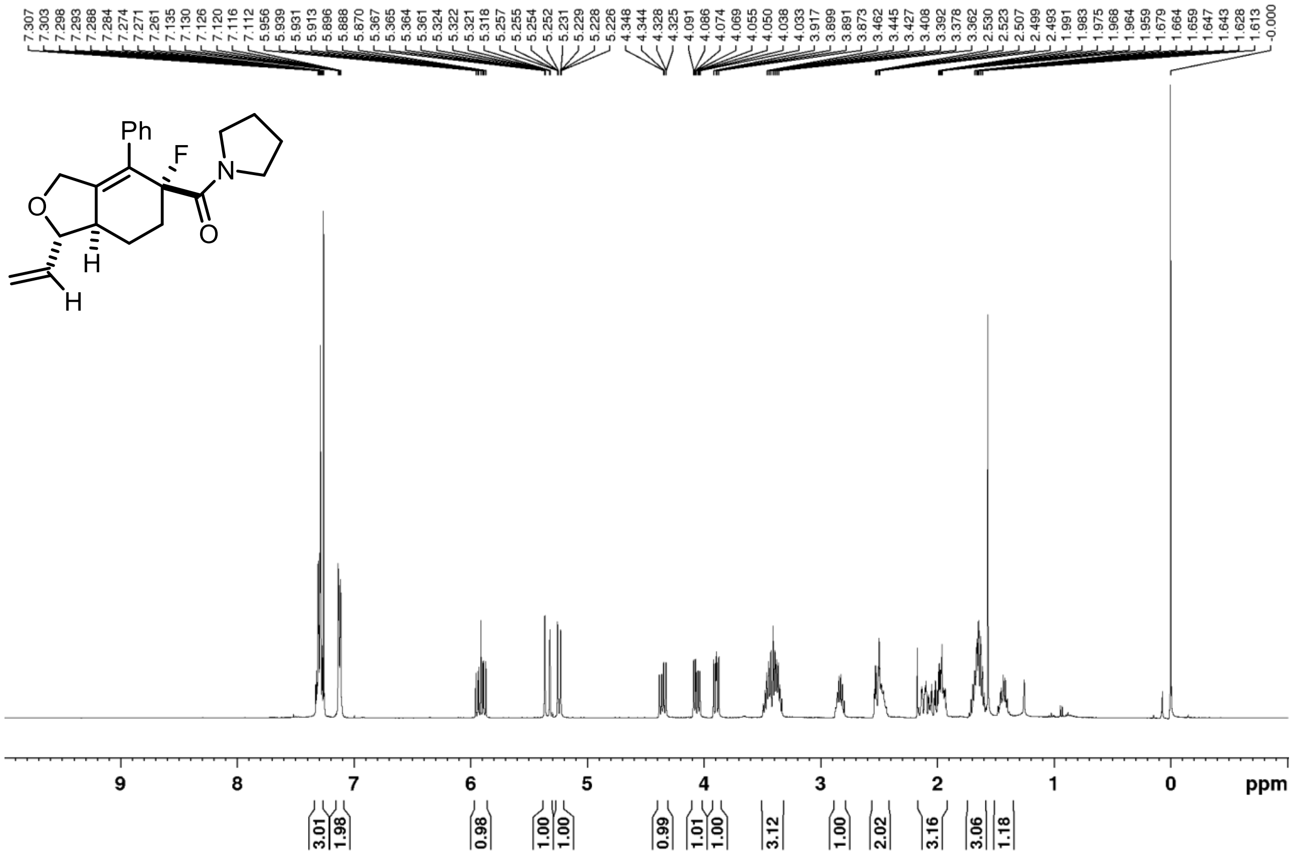

^{13}C NMR ($CDCl_3$, 101 MHz)

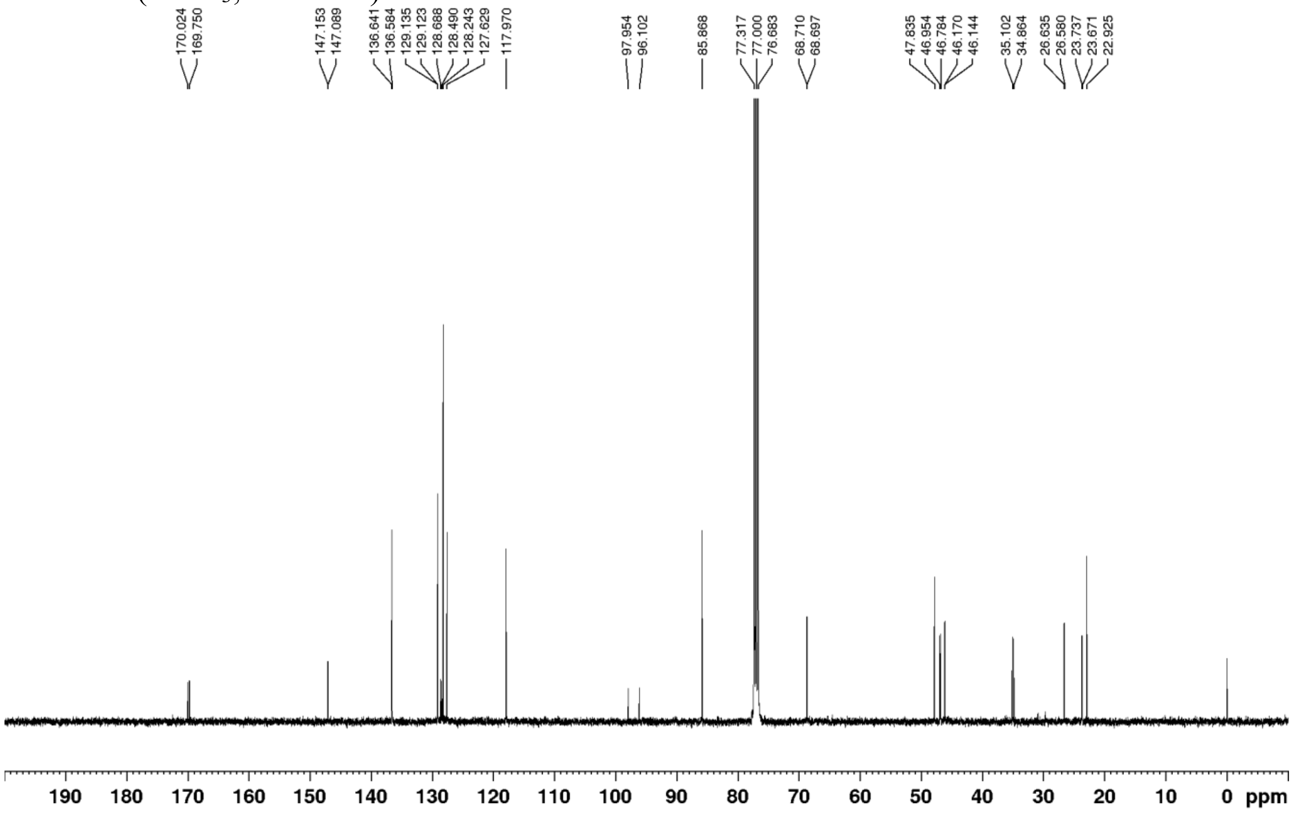

^{19}F NMR (CDCl$_3$, 377 MHz)

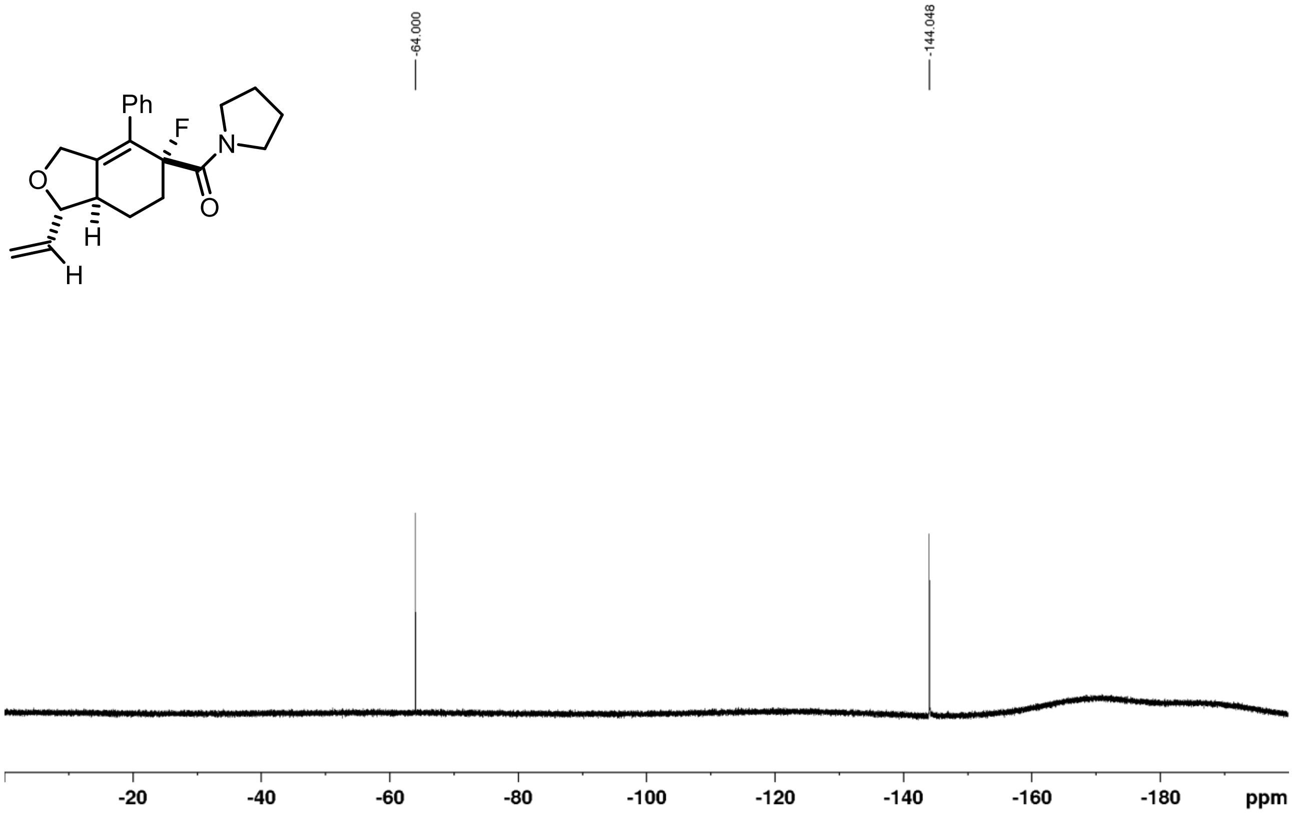

NOESY (CDCl$_3$, 400 MHz)

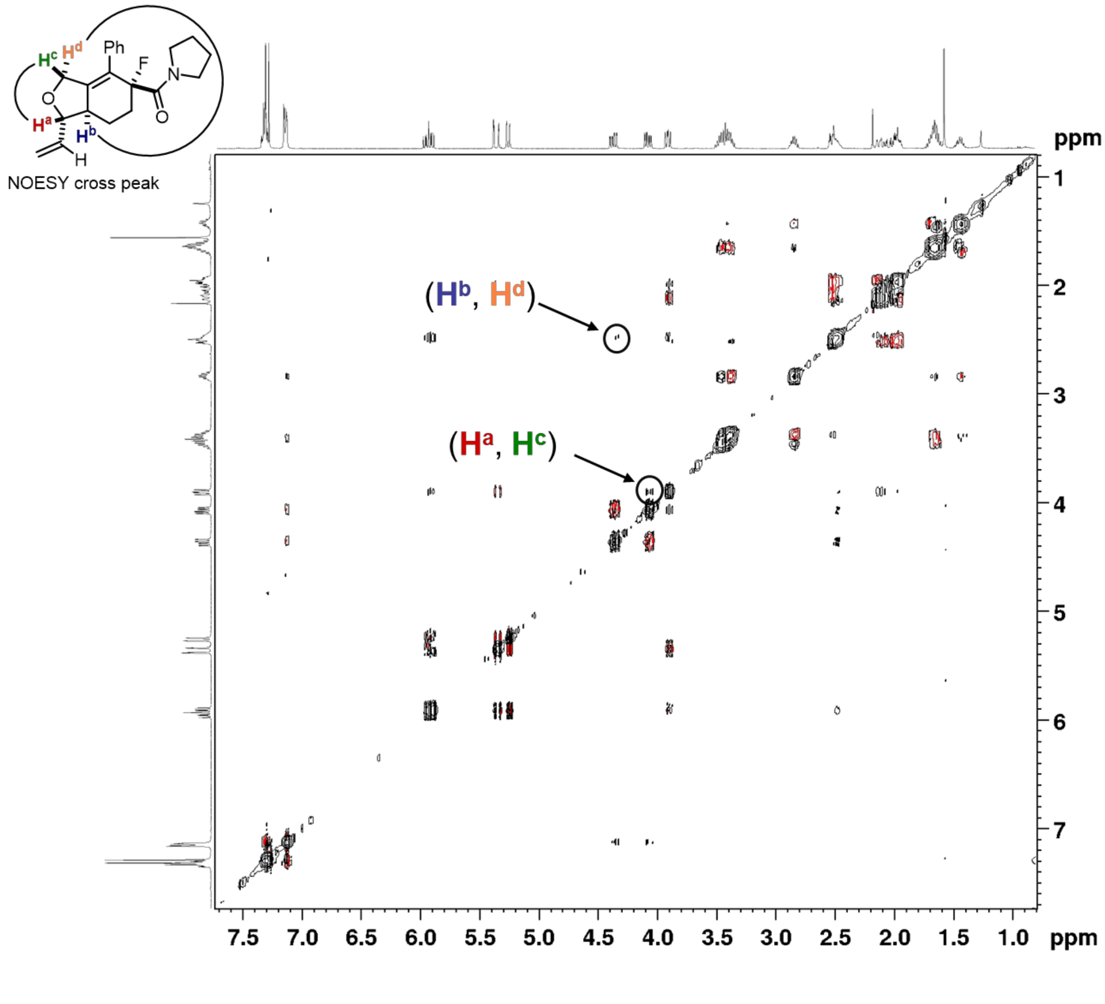

(–)-(1*S*,5*S*,7a*R*)-*N*-Methyl-*N*,4-diphenyl-1-vinyl-1,3,5,6,7,7a-hexahydroisobenzofuran-5-carboxamide (6gg)

^{1}H NMR ($CDCl_3$, 400 MHz)

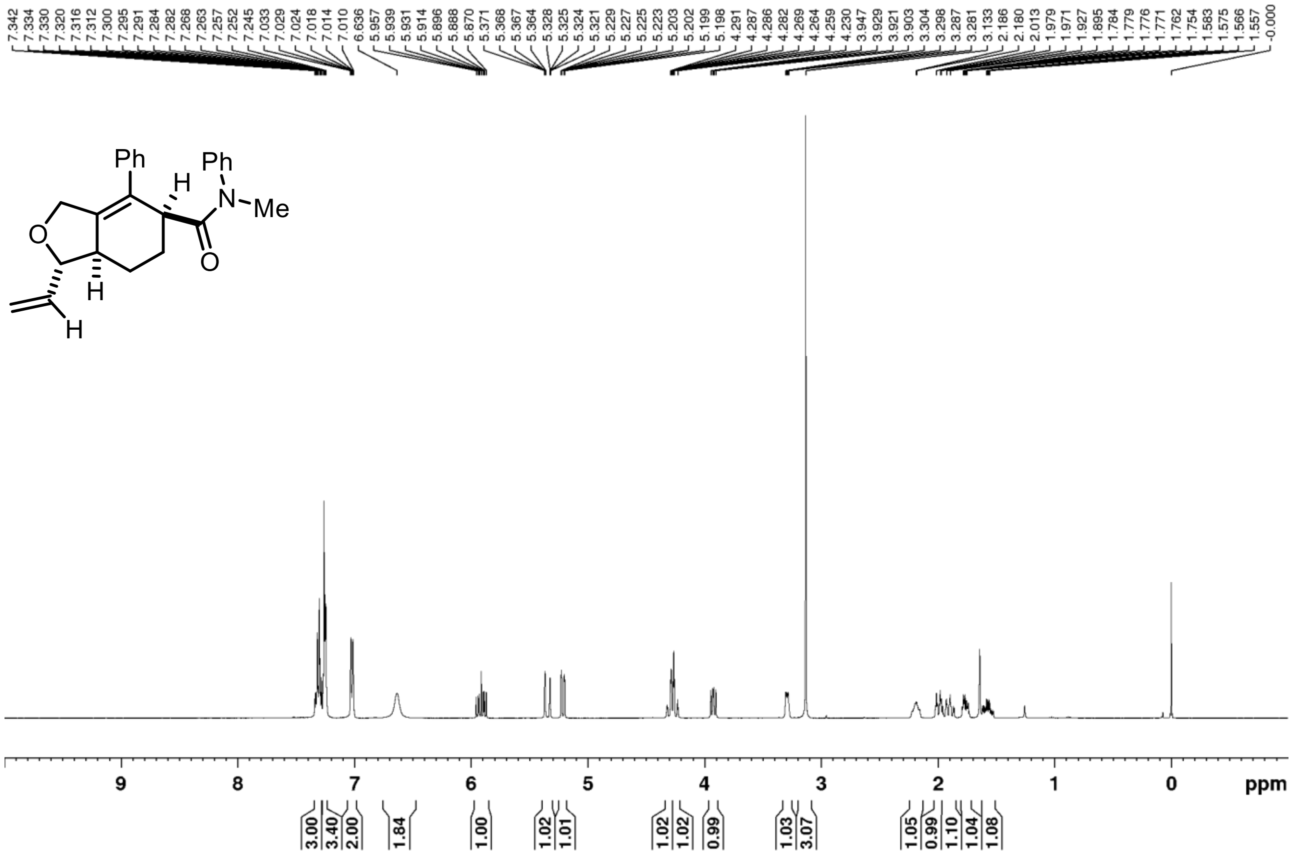

^{13}C NMR ($CDCl_3$, 101 MHz)

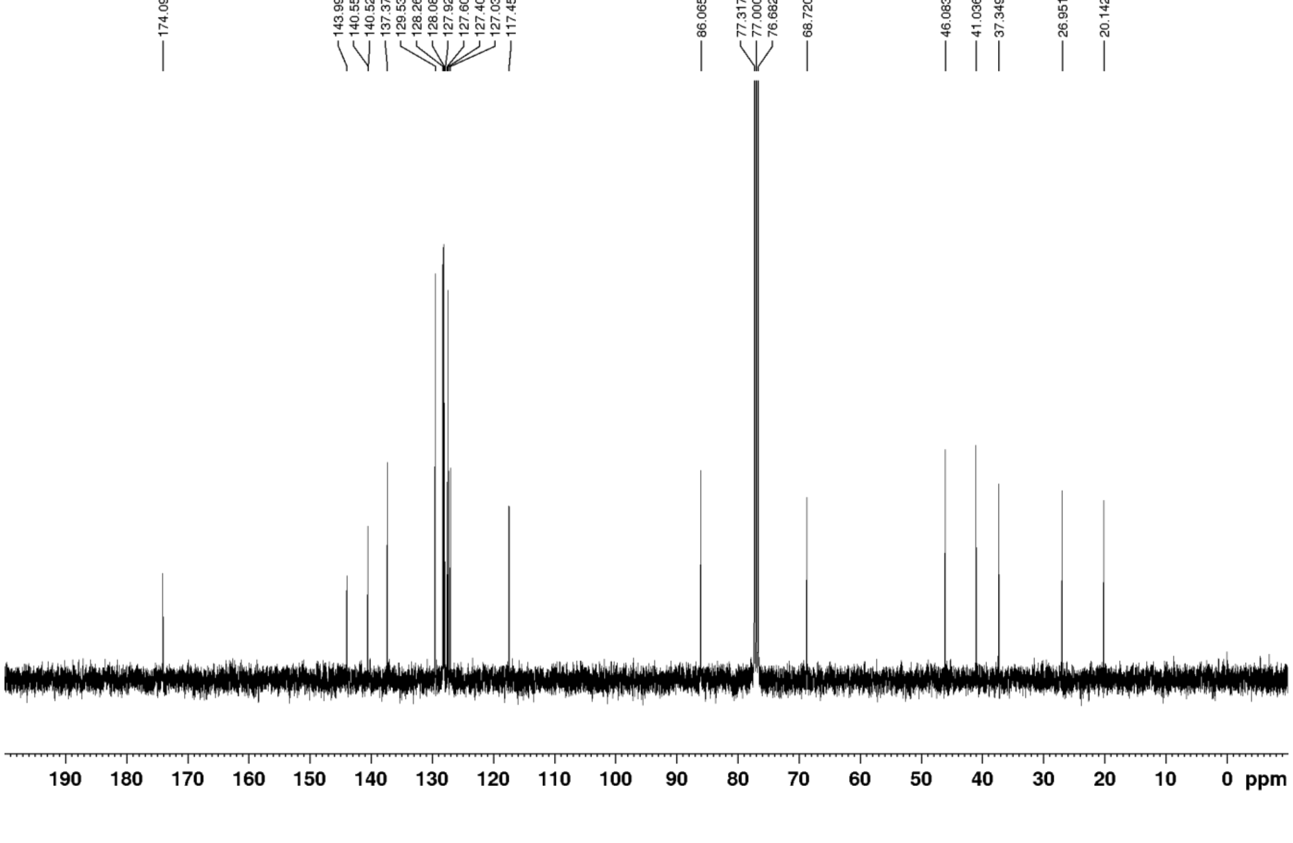

(*E*)-4-{(2*R*,3*R*)-4-(*Z*)-Benzylidene-3-methyl-1-(4-methylphenyl)-2-(prop-1-en-2-yl) pyrrolidin-3-yl}-2-fluoro-1-(pyrrolidin-1-yl)but-2-en-1-one [7ha (major diastereomer)]

^{1}H NMR ($CDCl_3$, 400 MHz)

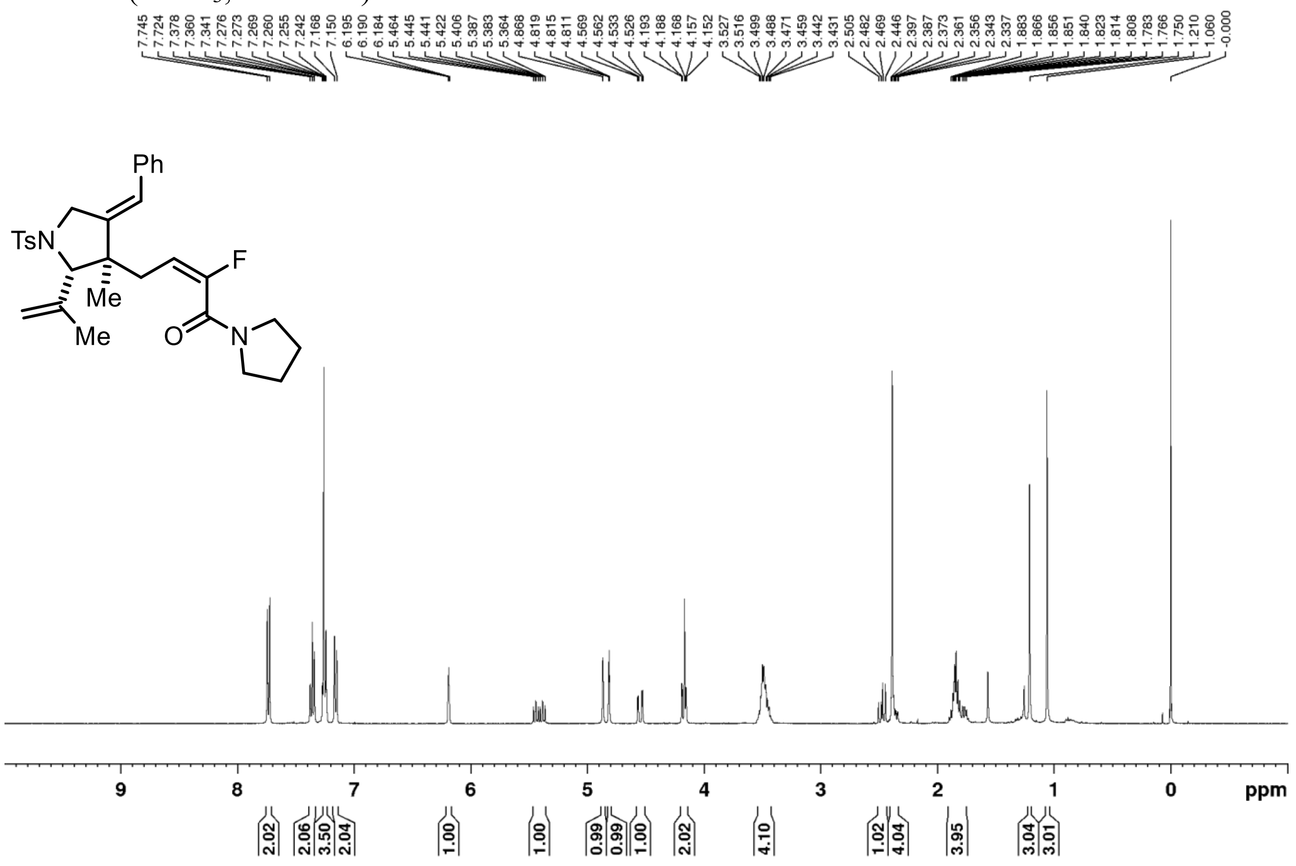

^{13}C NMR ($CDCl_3$, 101 MHz)

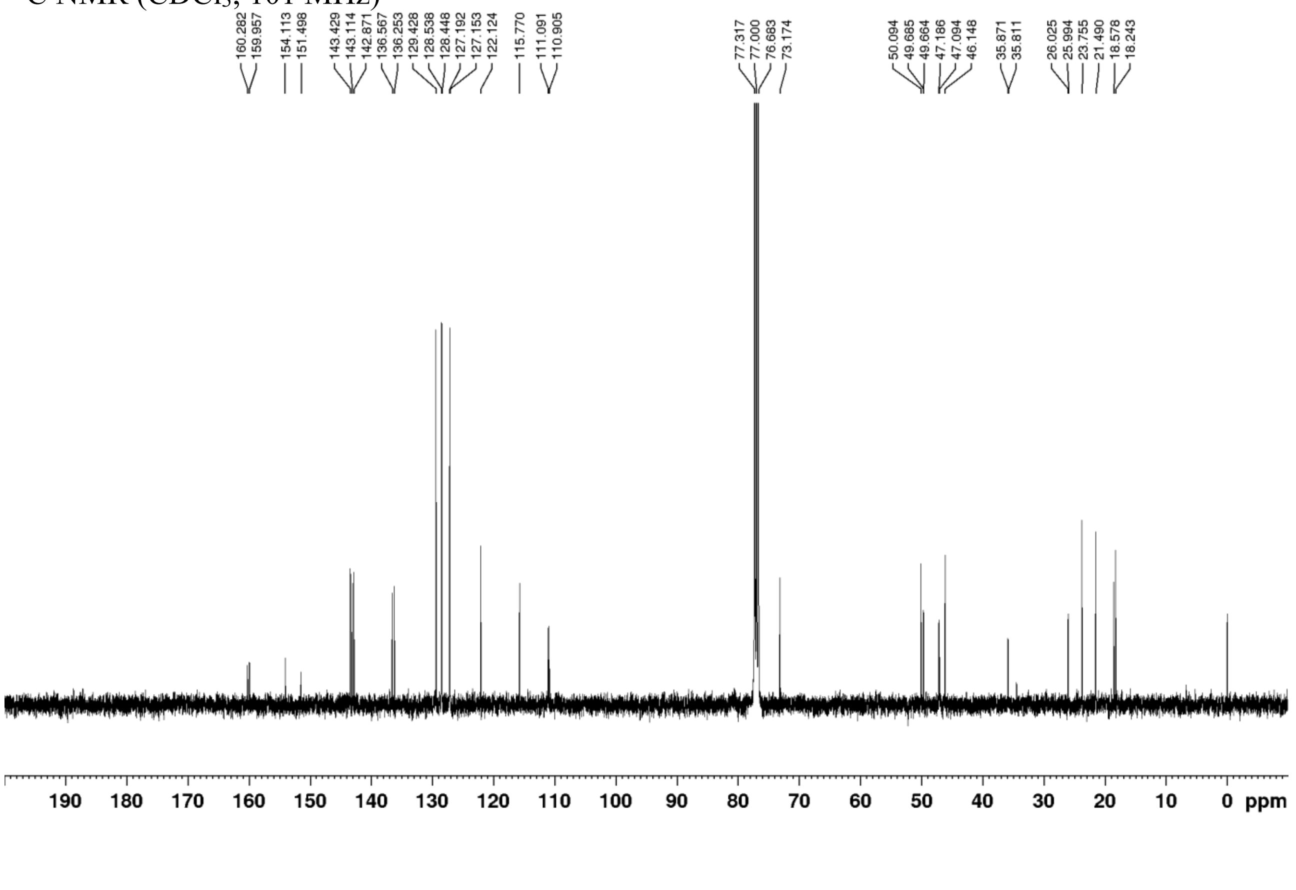

^{19}F NMR ($CDCl_3$, 377 MHz)

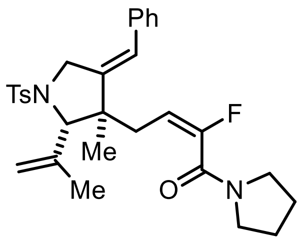

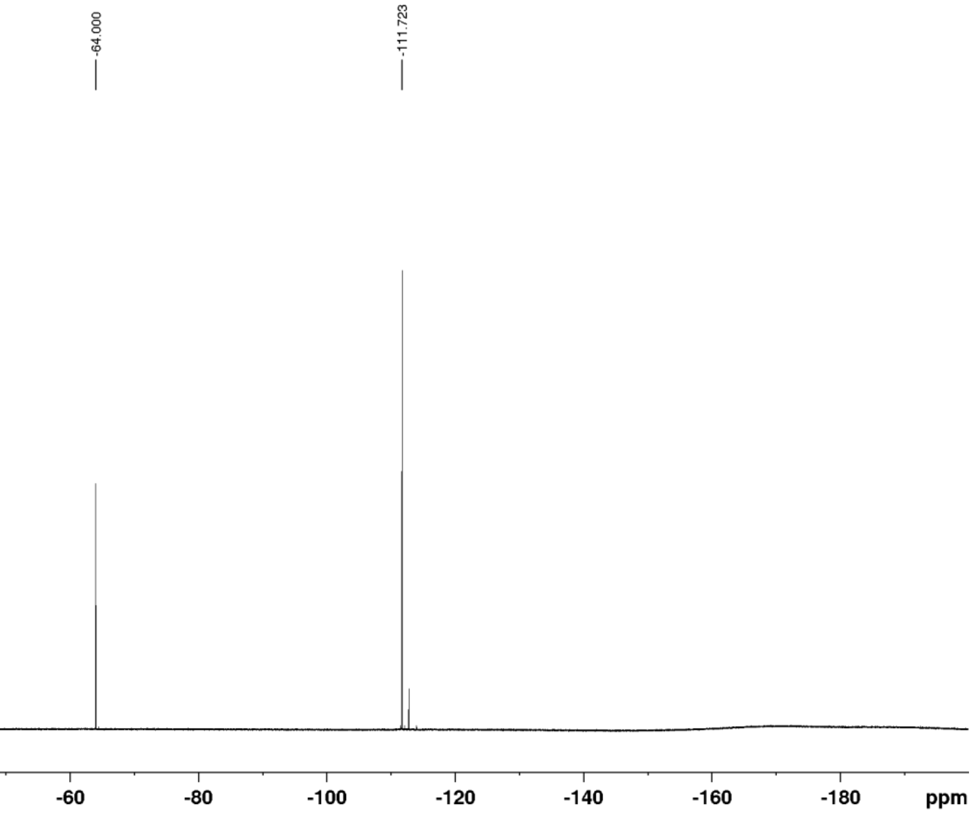

(*E*)-4-[(2*R*,3*R*)-4-{(*Z*)-4-Bromobenzylidene}-3-methyl)-1-(4-methylphenyl)-2-(prop-1-en-2-ylpyrrolidin-3-yl]-2-fluoro-1-(pyrrolidin-1-yl)but-2-en-1-one [7ia (major diastereomer)]

^{1}H NMR (CDCl$_3$, 400 MHz)

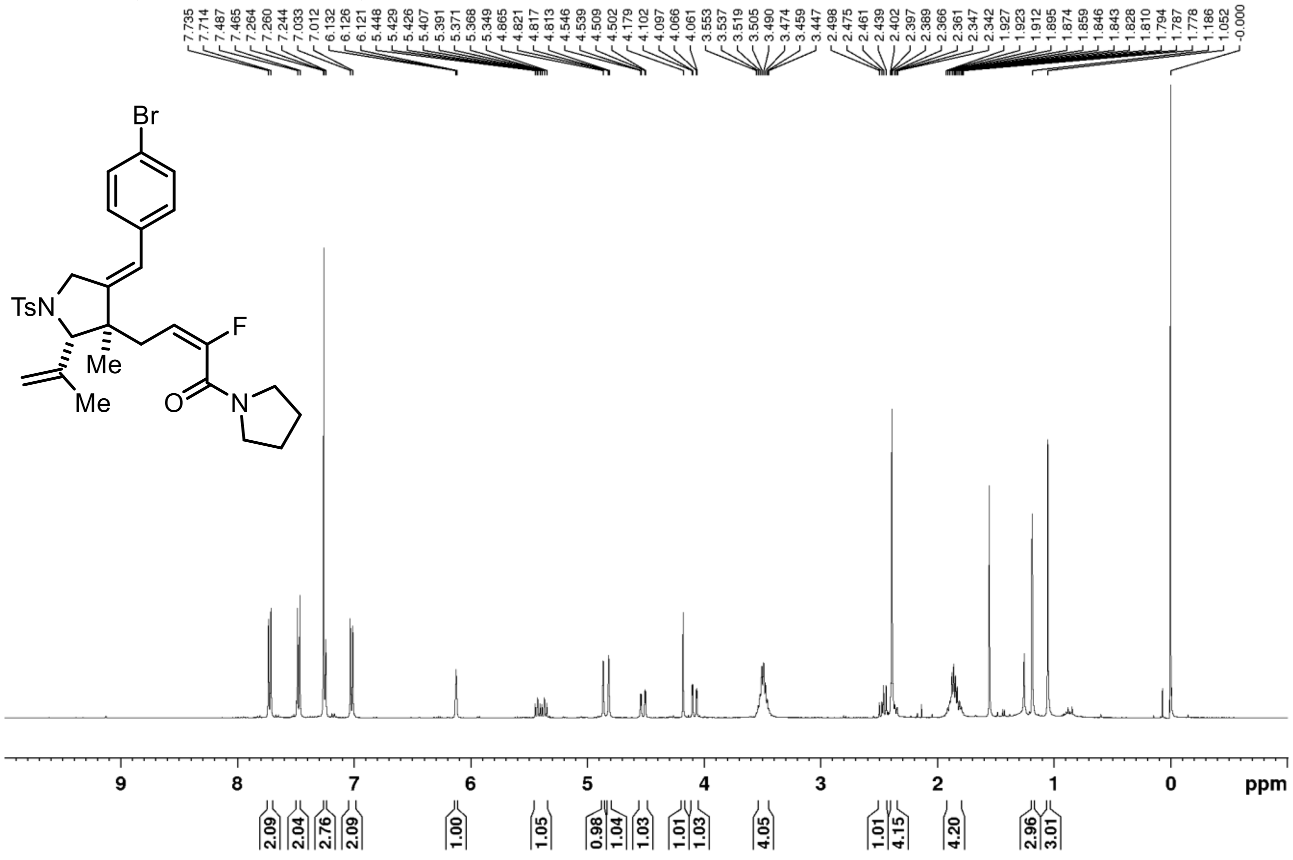

^{13}C NMR (CDCl$_3$, 101 MHz)

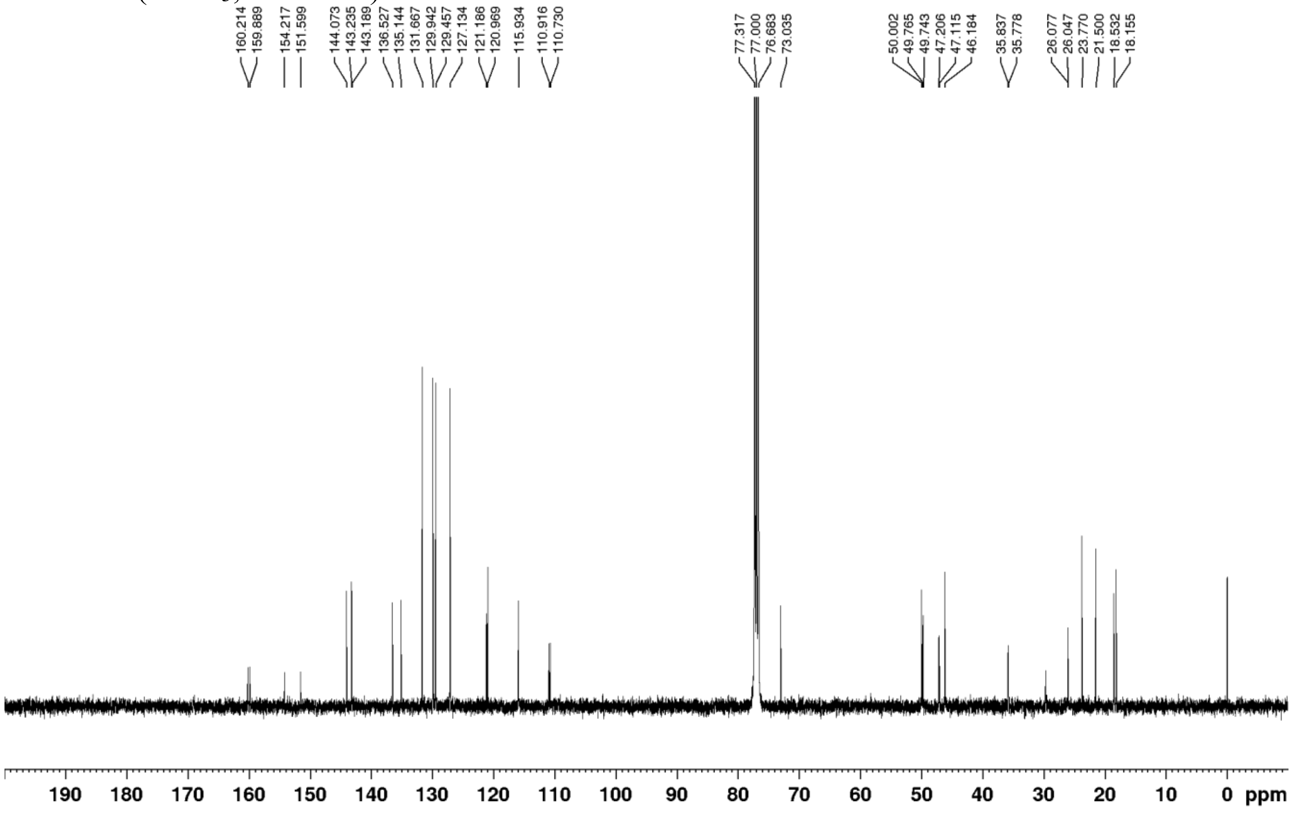

^{19}F NMR ($CDCl_3$, 377 MHz)

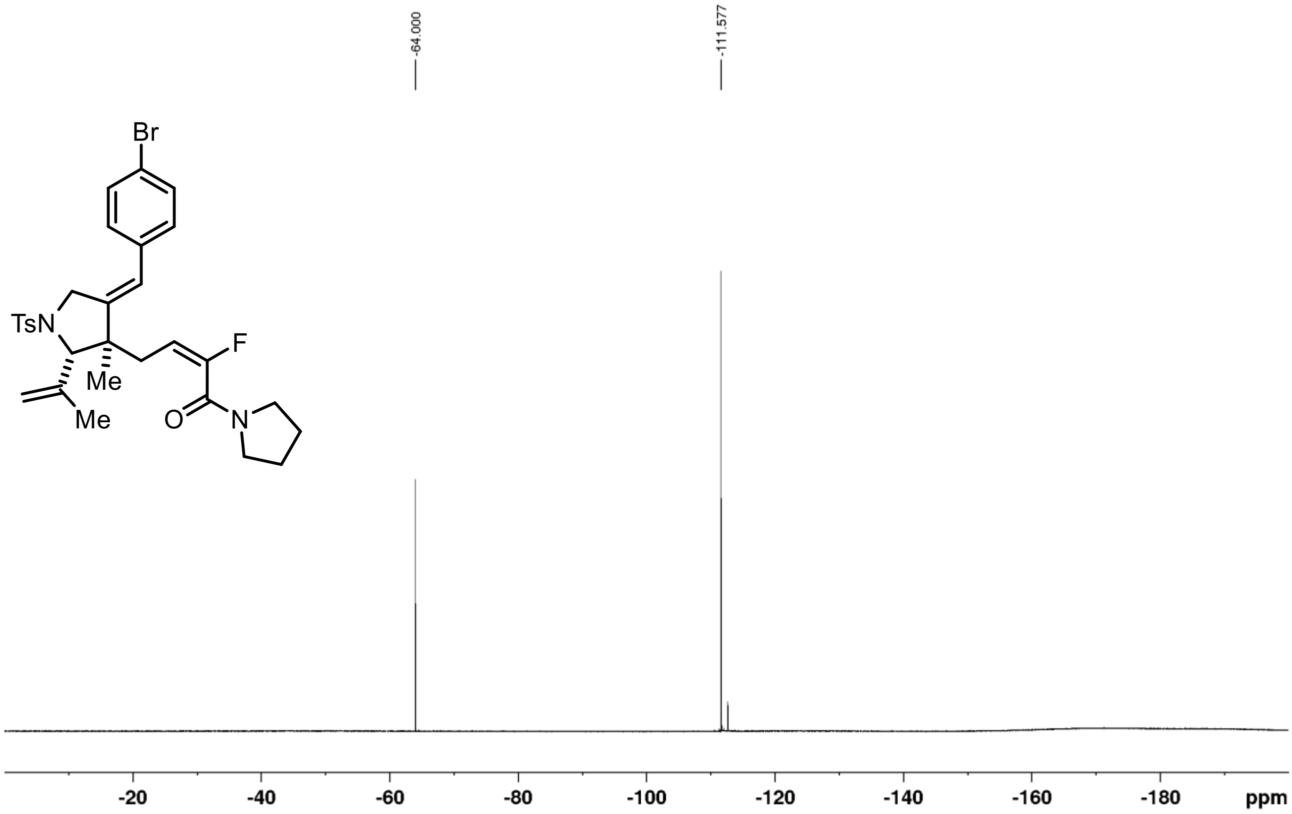

NOESY ($CDCl_3$, 400 MHz)

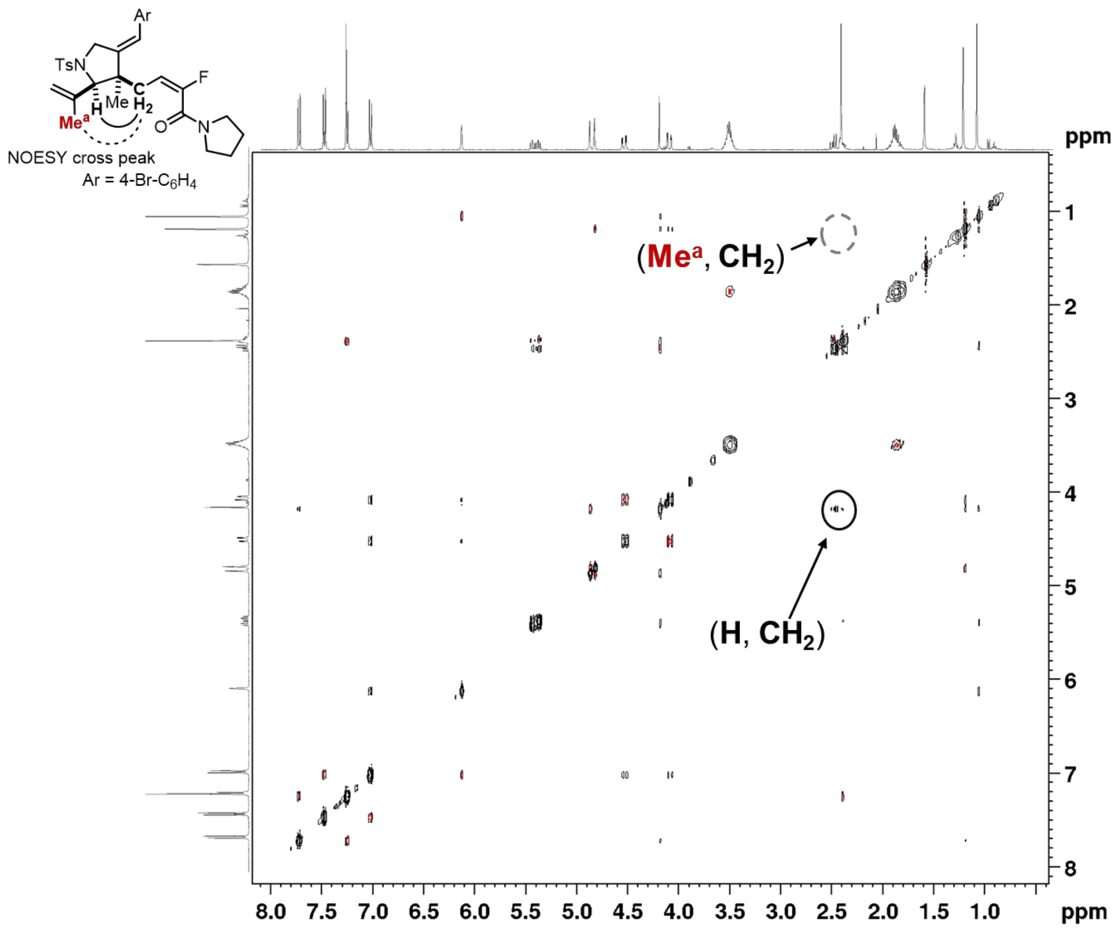

(*E*)-4-{(2*S*,3*R*,*Z*)-4-Ethylidene-3-methyl-1-(4-methylphenyl)-2-(prop-1-en-2-yl) pyrrolidin-3-yl}-2-fluoro-1-(pyrrolidin-1-yl)but-2-en-1-one [7ja (major diastereomer)]

^{1}H NMR ($CDCl_3$, 400 MHz)

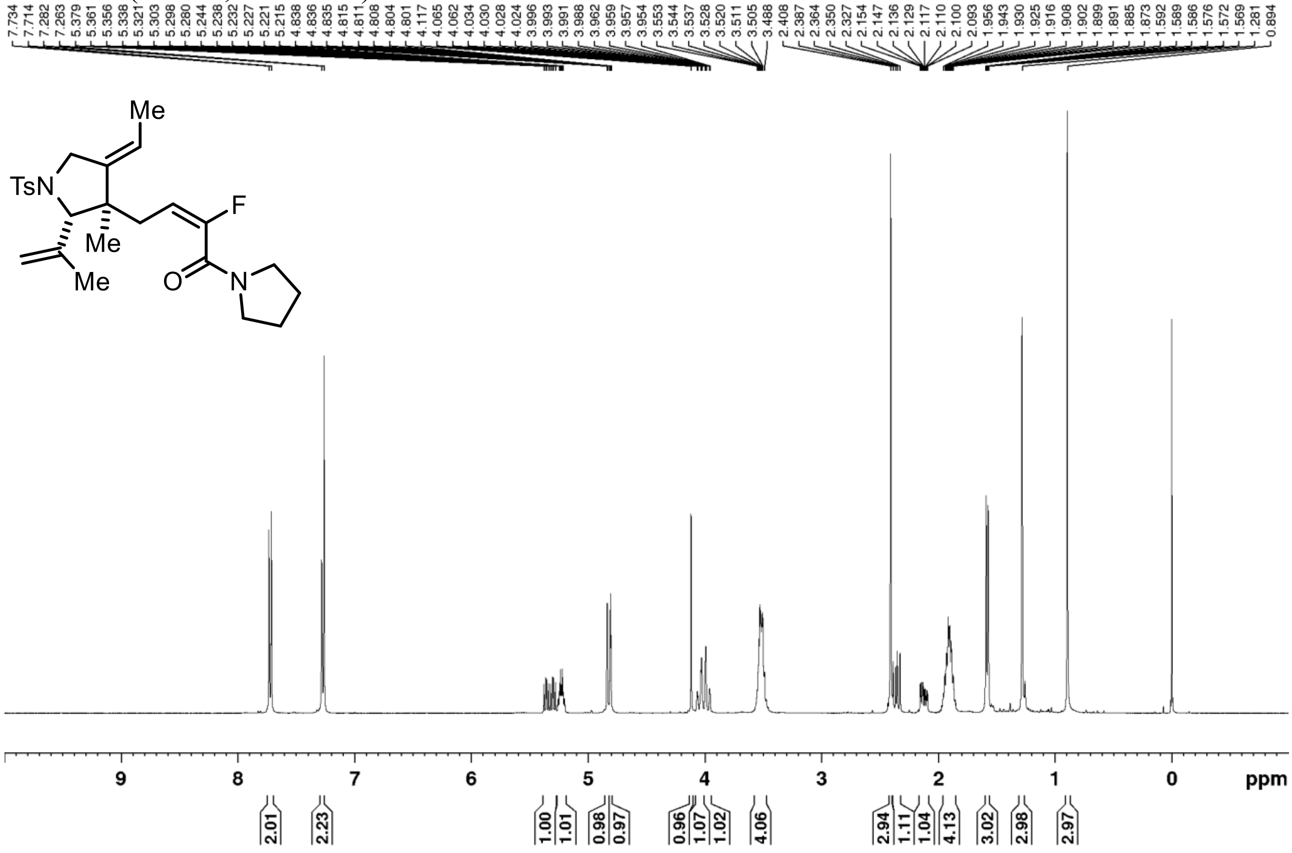

^{13}C NMR ($CDCl_3$, 101 MHz)

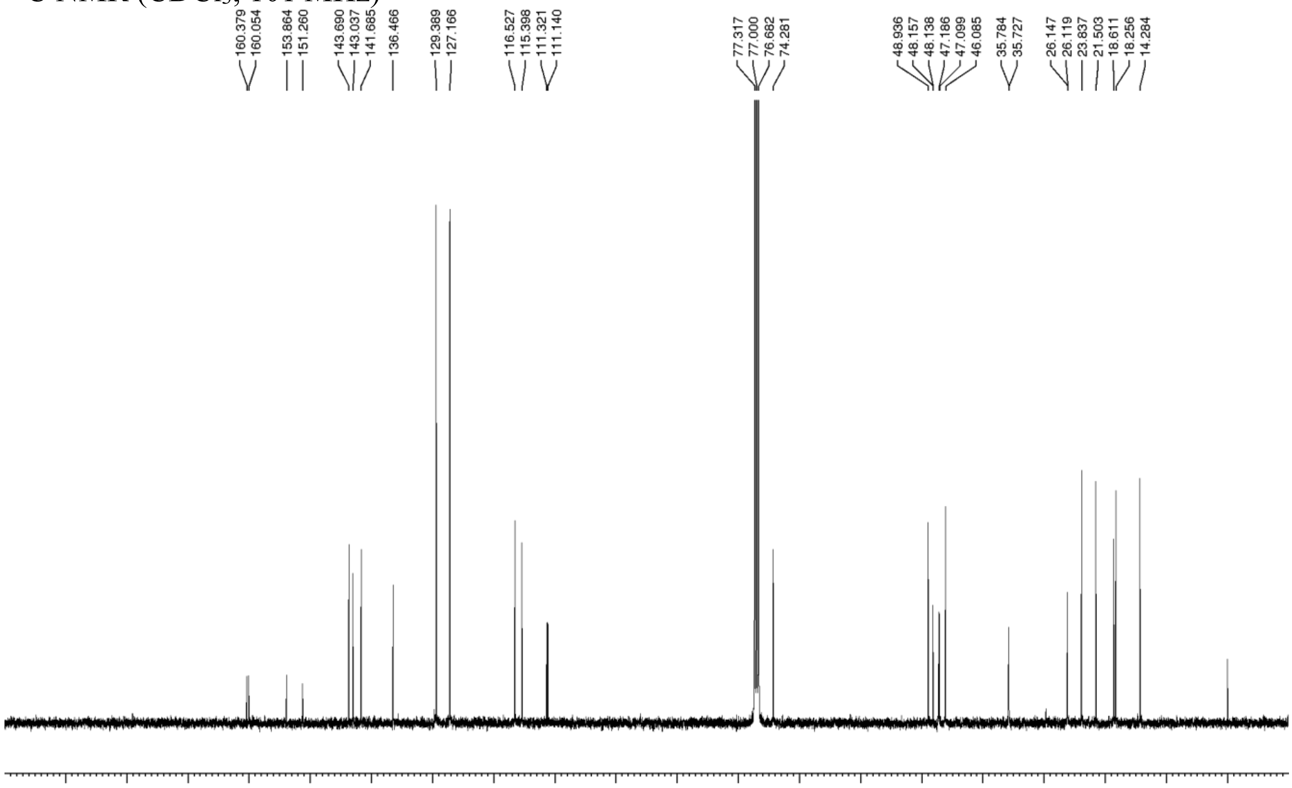

^{19}F NMR ($CDCl_3$, 377 MHz)

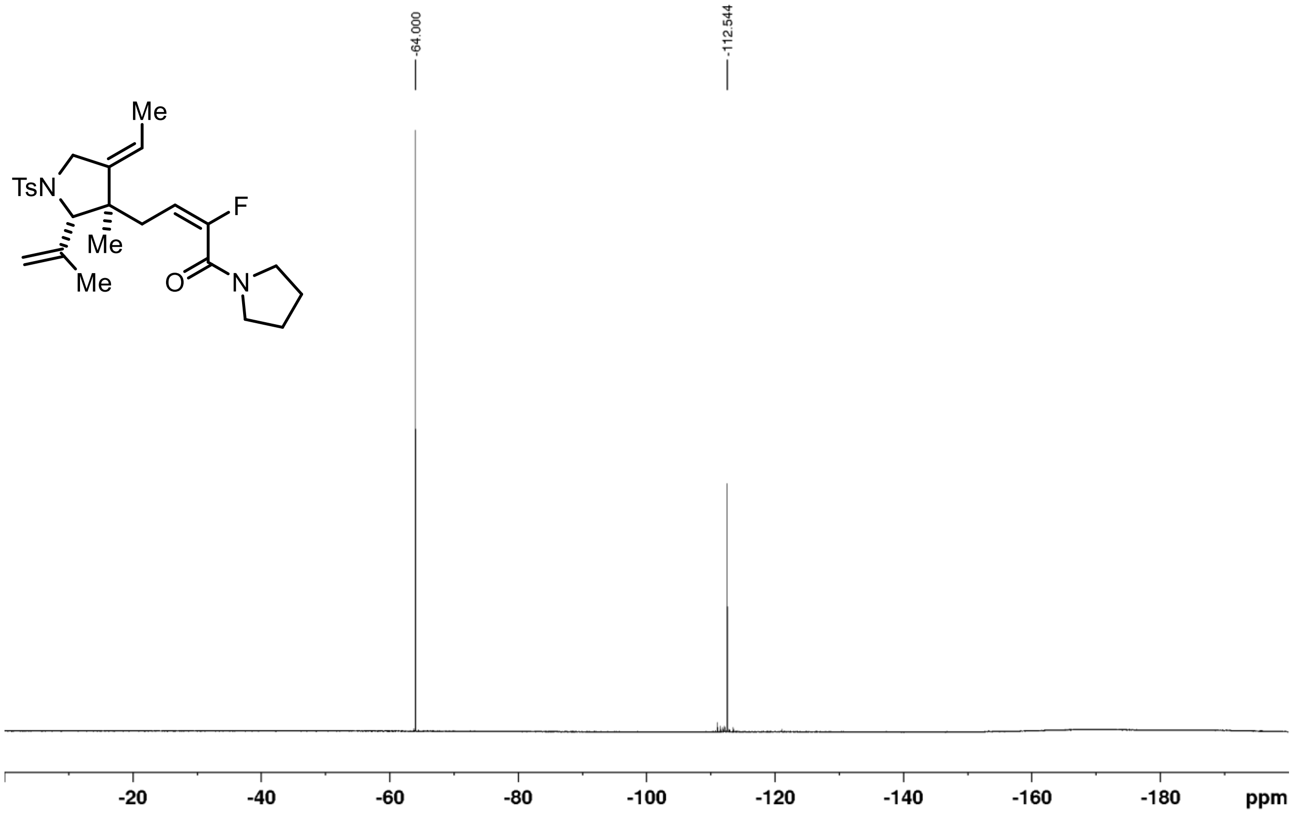

(*E*)-4-{(*Z*)-4-Ethylidene-3-methyl-1-(4-methylphenyl)-2-(prop-1-en-2-yl)pyrrolidin-3-yl}-2-fluoro-1-(pyrrolidin-1-yl)but-2-en-1-one [7ja (minor diastereomer)]

^{1}H NMR (CDCl$_3$, 400 MHz)

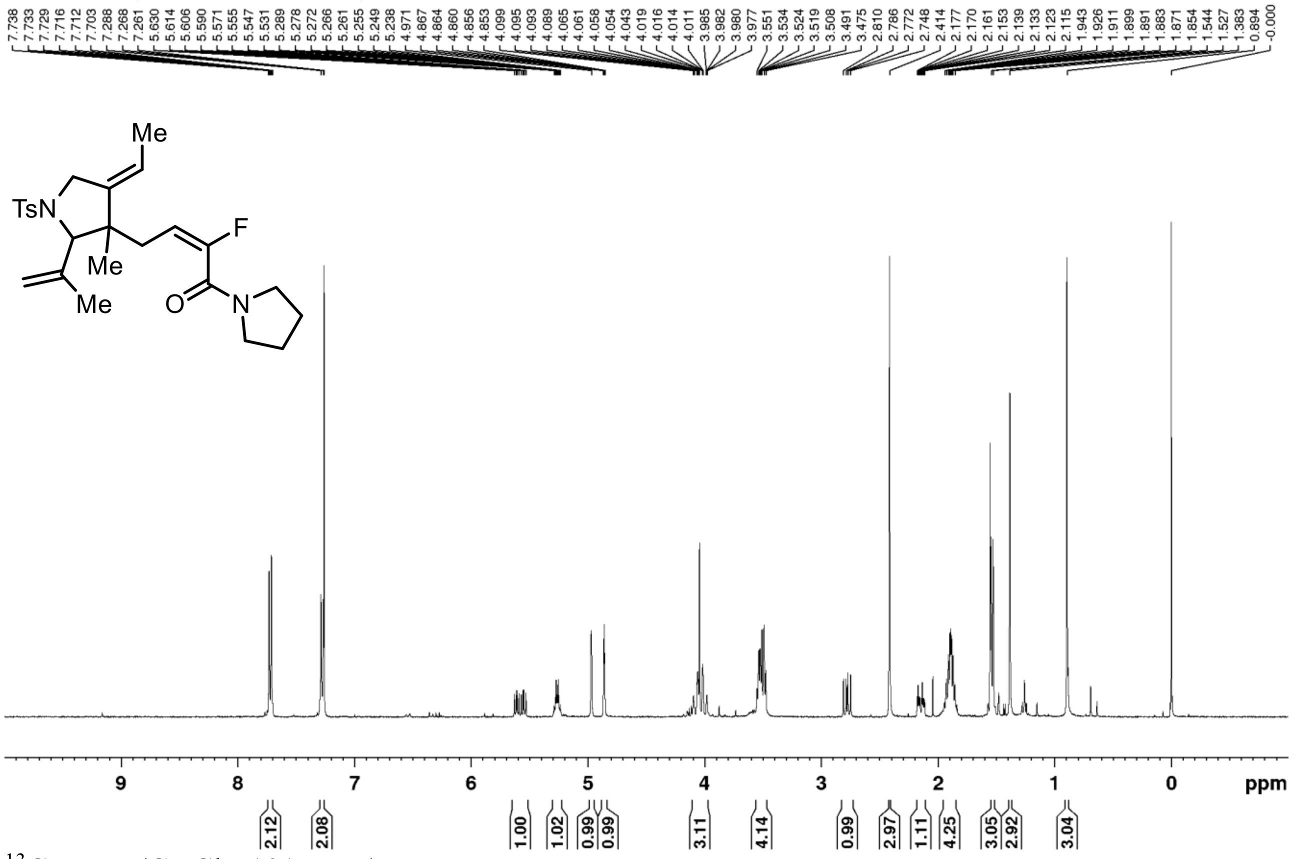

^{13}C NMR (CDCl$_3$, 101 MHz)

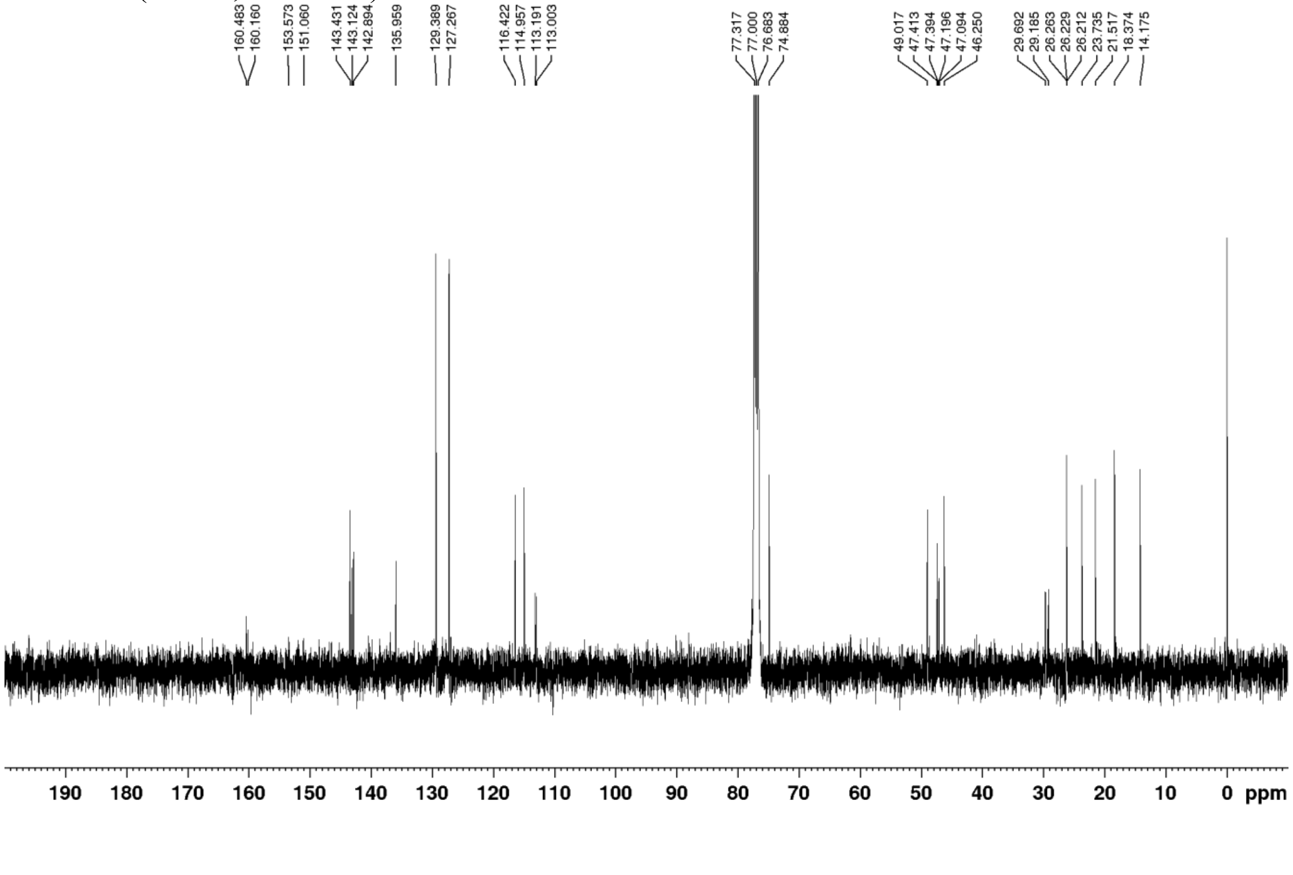

^{19}F NMR ($CDCl_3$, 377 MHz)

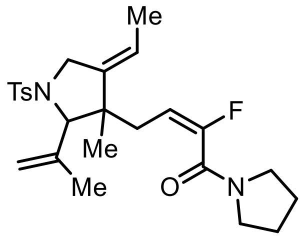

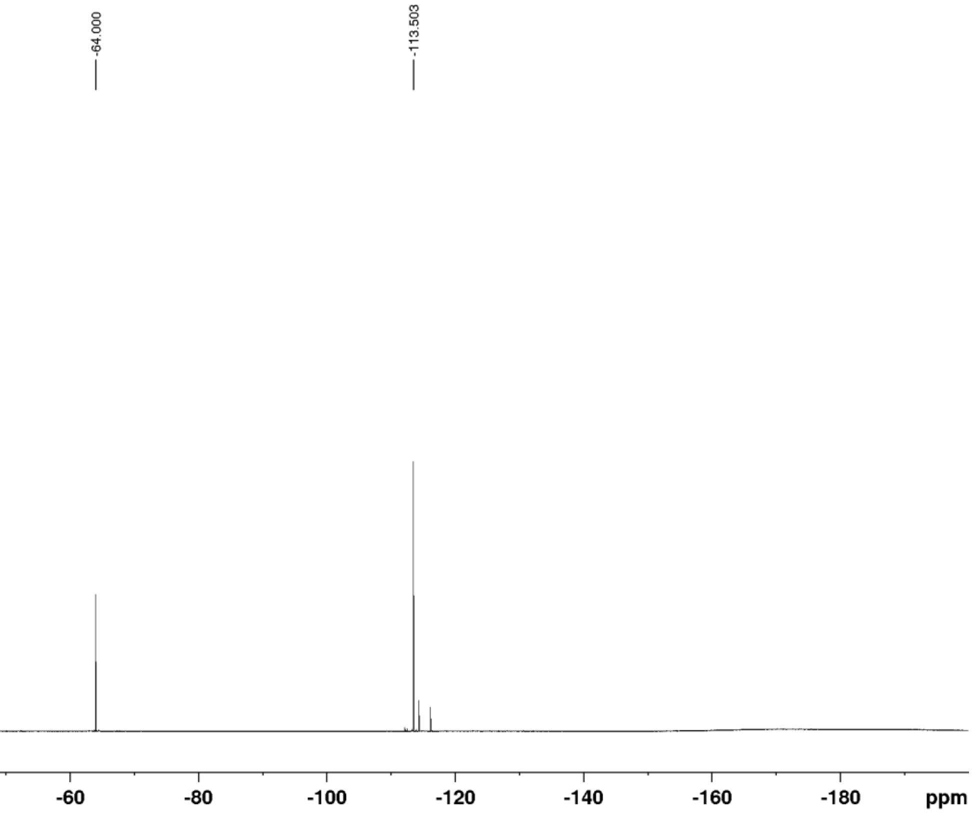

(*Z*)-4-[(2*S*,3*R*)-4-{(*Z*)-4-Bromobenzylidene}-3-methyl-1-(4-methylphenyl)-2-(prop-1-en-2-yl)pyrrolidin-3-yl]-2-methyl-1-(pyrrolidin-1-yl)but-2-en-1-one (7ie)

Diastereomer mixture (79:21 d.r.)

^{1}H NMR (CDCl$_3$, 400 MHz)

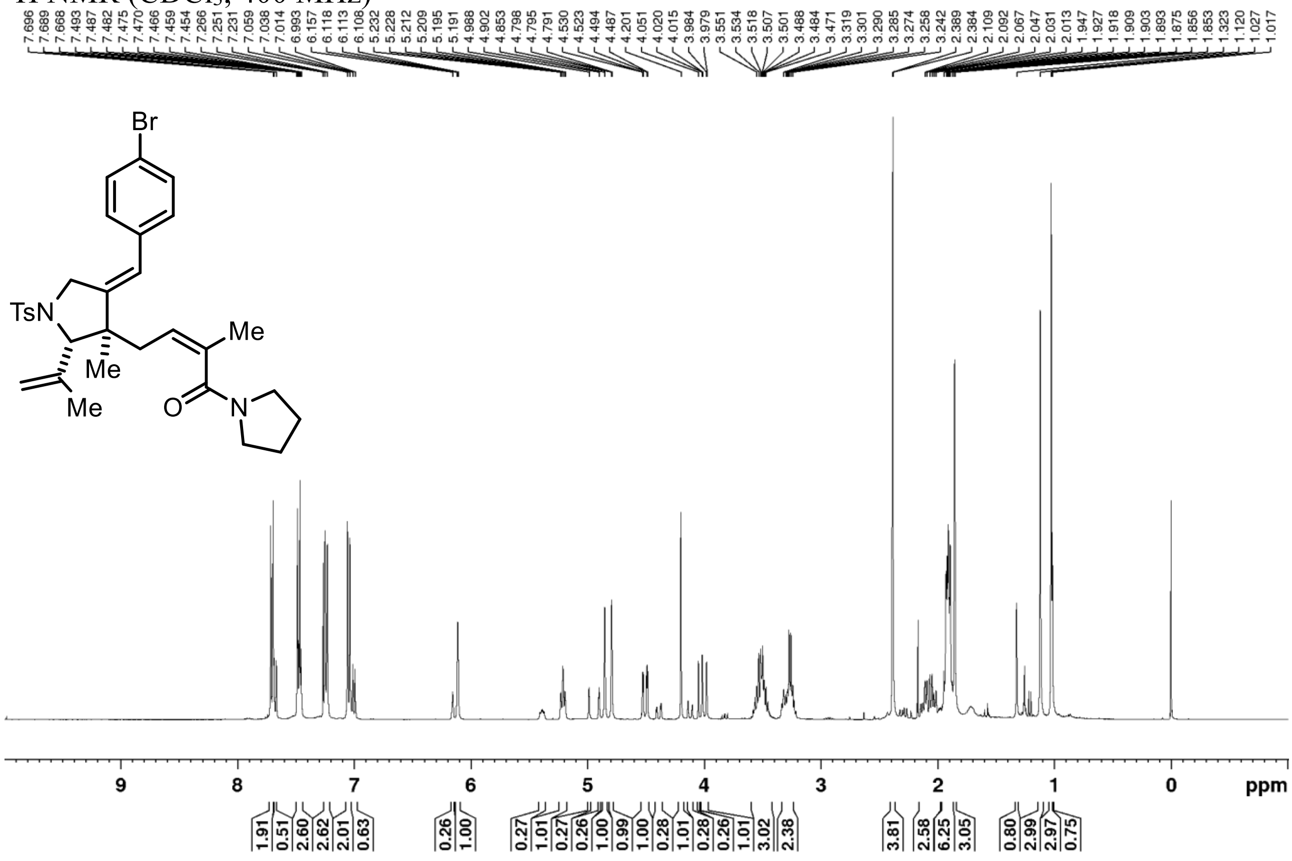

^{13}C NMR (CDCl$_3$, 101 MHz)

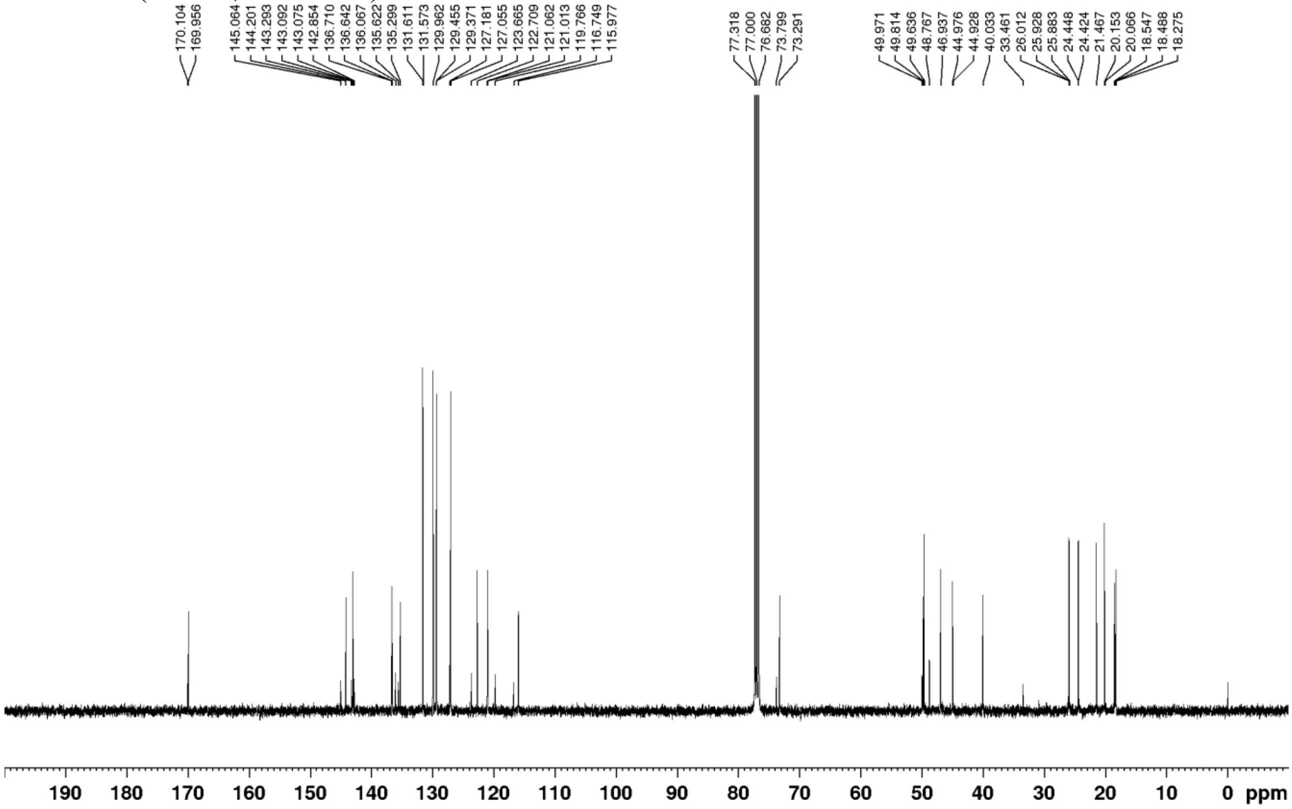

(*E*)-4-[(2*S*,3*R*)-4-{(*Z*)-4-Bromobenzylidene}-3-methyl-1-(4-methylphenyl)-2-(prop-1-en-2-yl)pyrrolidin-3-yl]-2-chloro-1-(pyrrolidin-1-yl)but-2-en-1-one (7if)

Diastereomer mixture (84:16 d.r.)

^{1}H NMR ($CDCl_3$, 400 MHz)

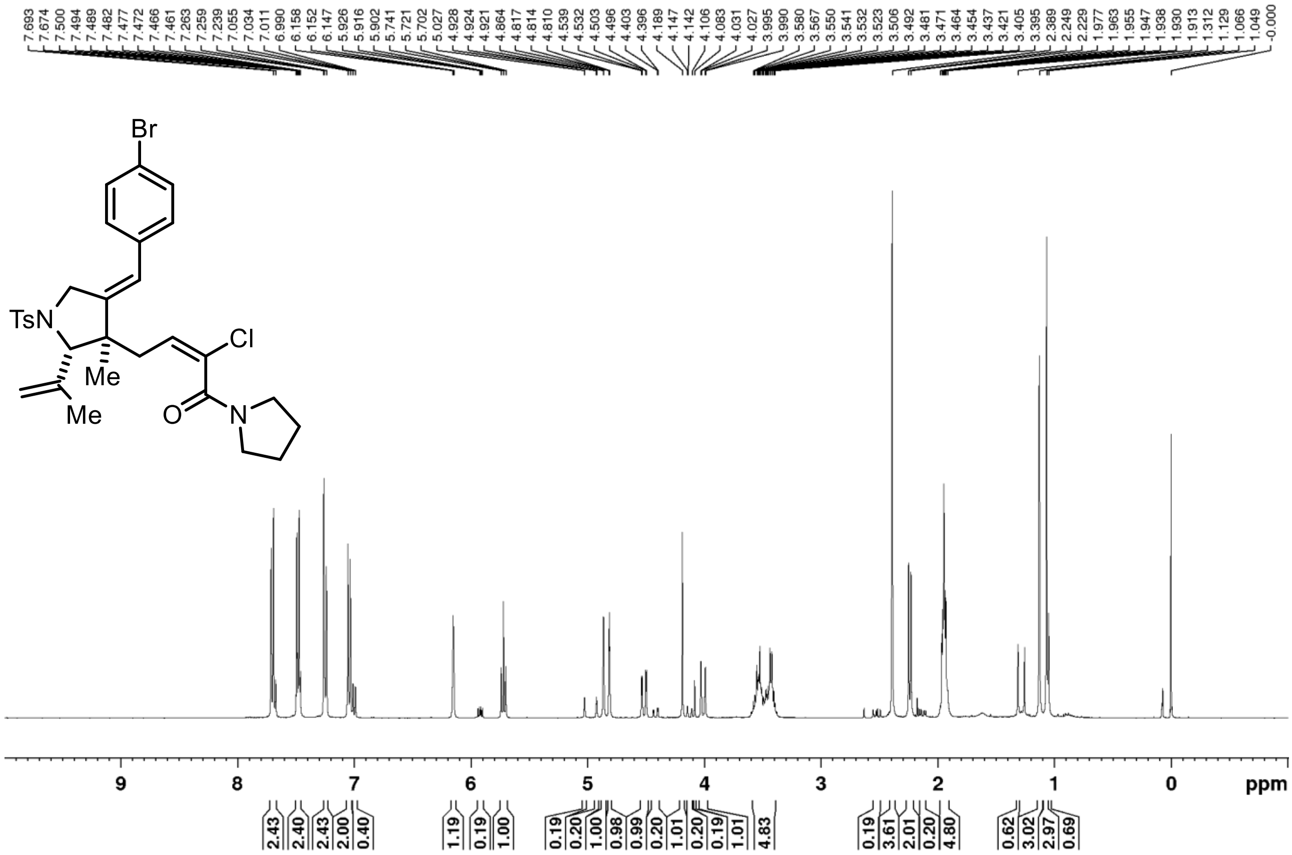

^{13}C NMR ($CDCl_3$, 101 MHz)

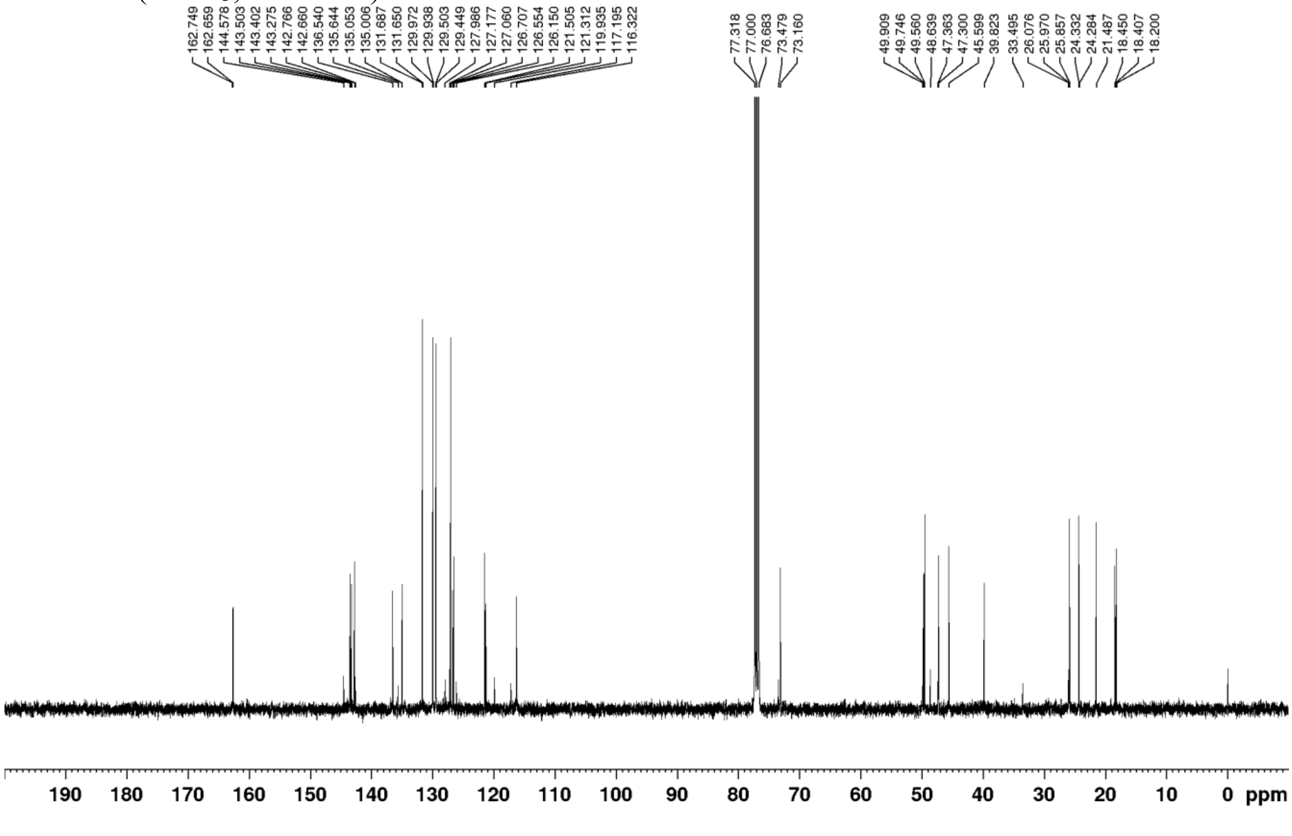

(4*R*,5*R*)-3-(*Z*)-Benzylidene-4-{(*E*)-3-fluoro-4-oxo-4-(pyrrolidin-1-yl)but-2-en-1-yl}-4-methyl-5-(prop-1-en-2-yl)dihydrofuran-2(3*H*)-one (7ka)

^{1}H NMR (CDCl$_3$, 400 MHz)

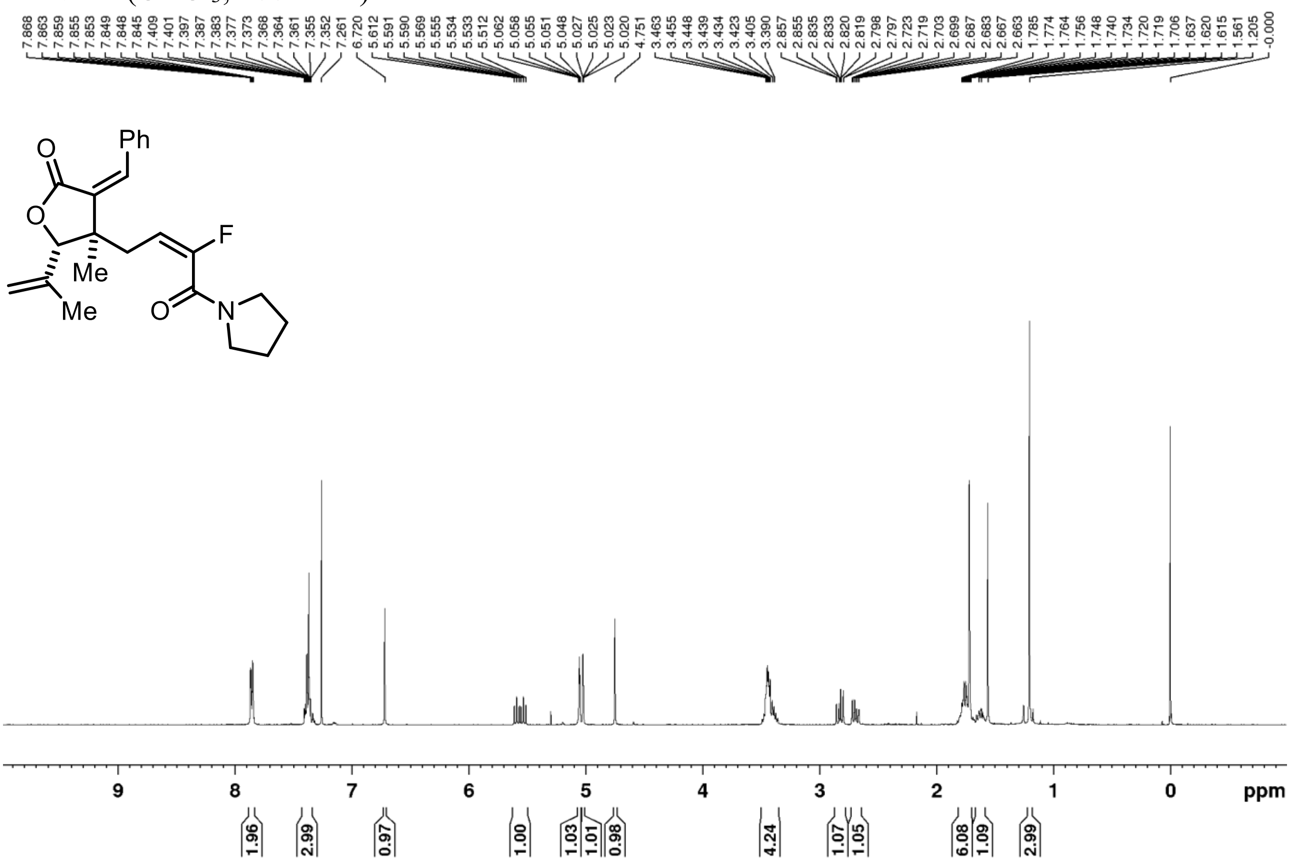

^{13}C NMR (CDCl$_3$, 101 MHz)

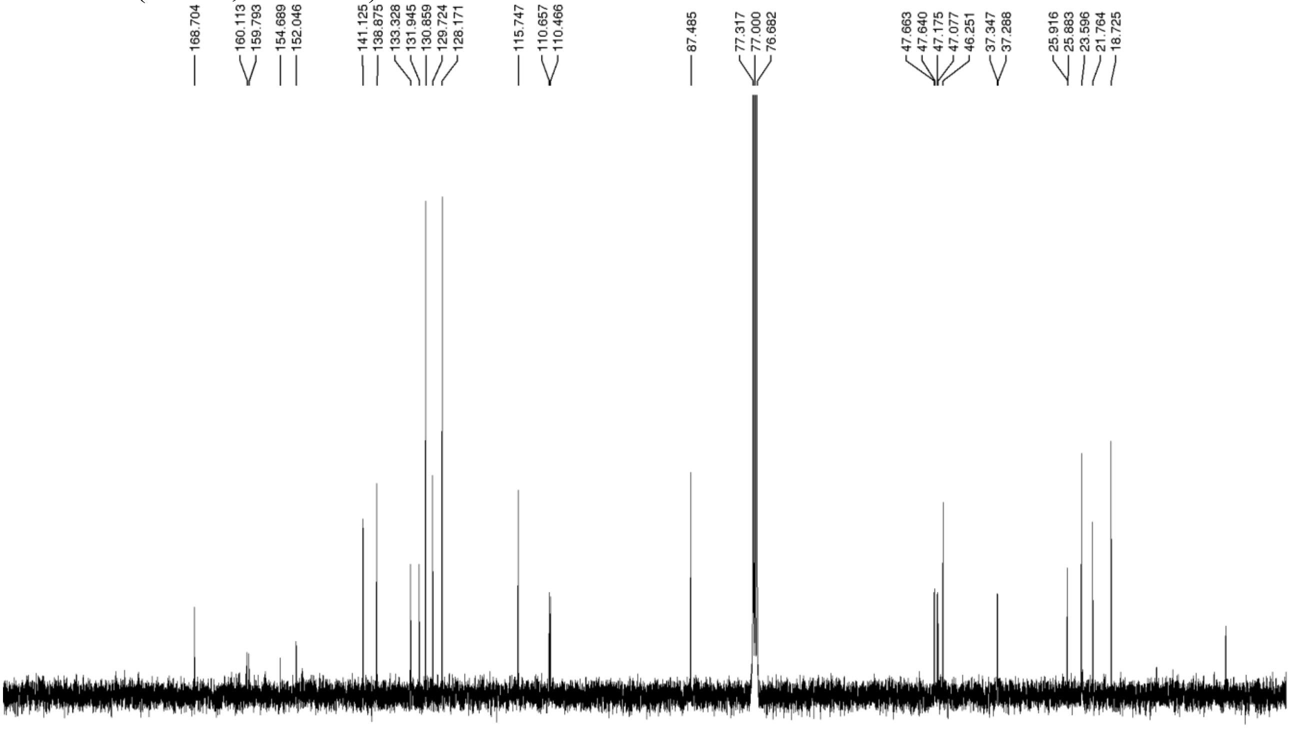

^{19}F NMR ($CDCl_3$, 377 MHz)

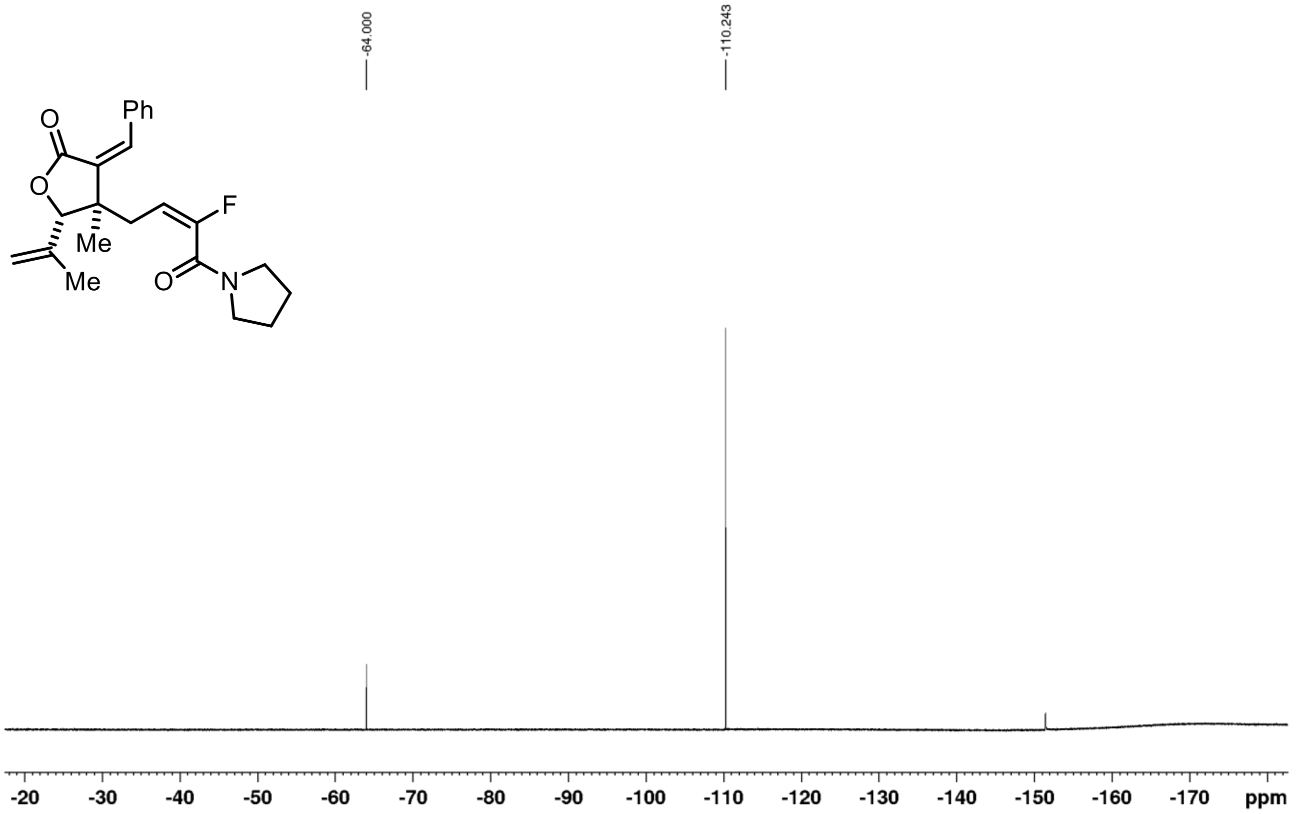

6.5. Synthetic Applications (Figures 5a, 5b)

(3*S*,3a*R*,6*R*,7*S*,7a*S*)-6-Fluoro-3,3a-dimethyl-7-phenyl-6-(pyrrolidine-1-carbonyl) hexahydroisobenzofuran-1(3*H*)-one (8)

^{1}H NMR ($CDCl_3$, 400 MHz)

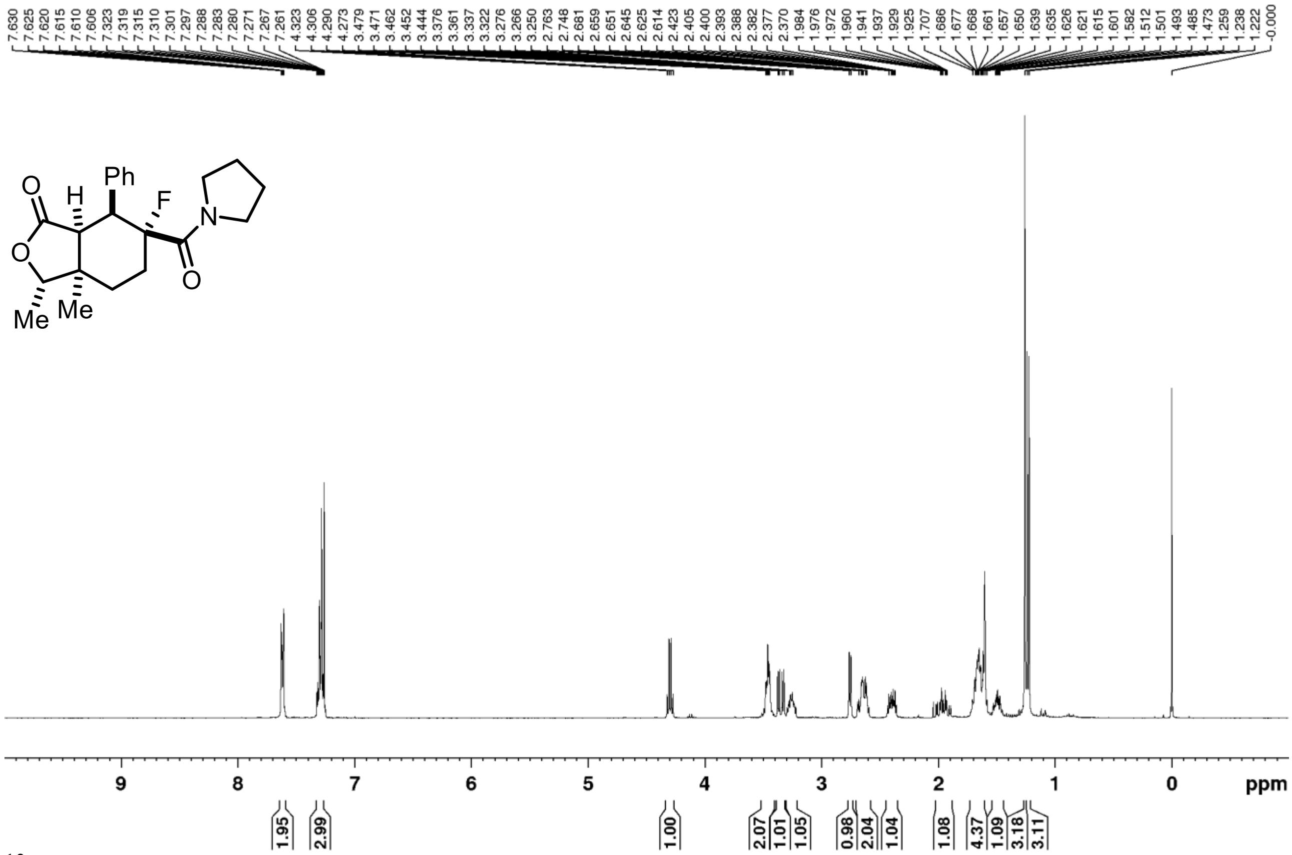

^{13}C NMR ($CDCl_3$, 101 MHz)

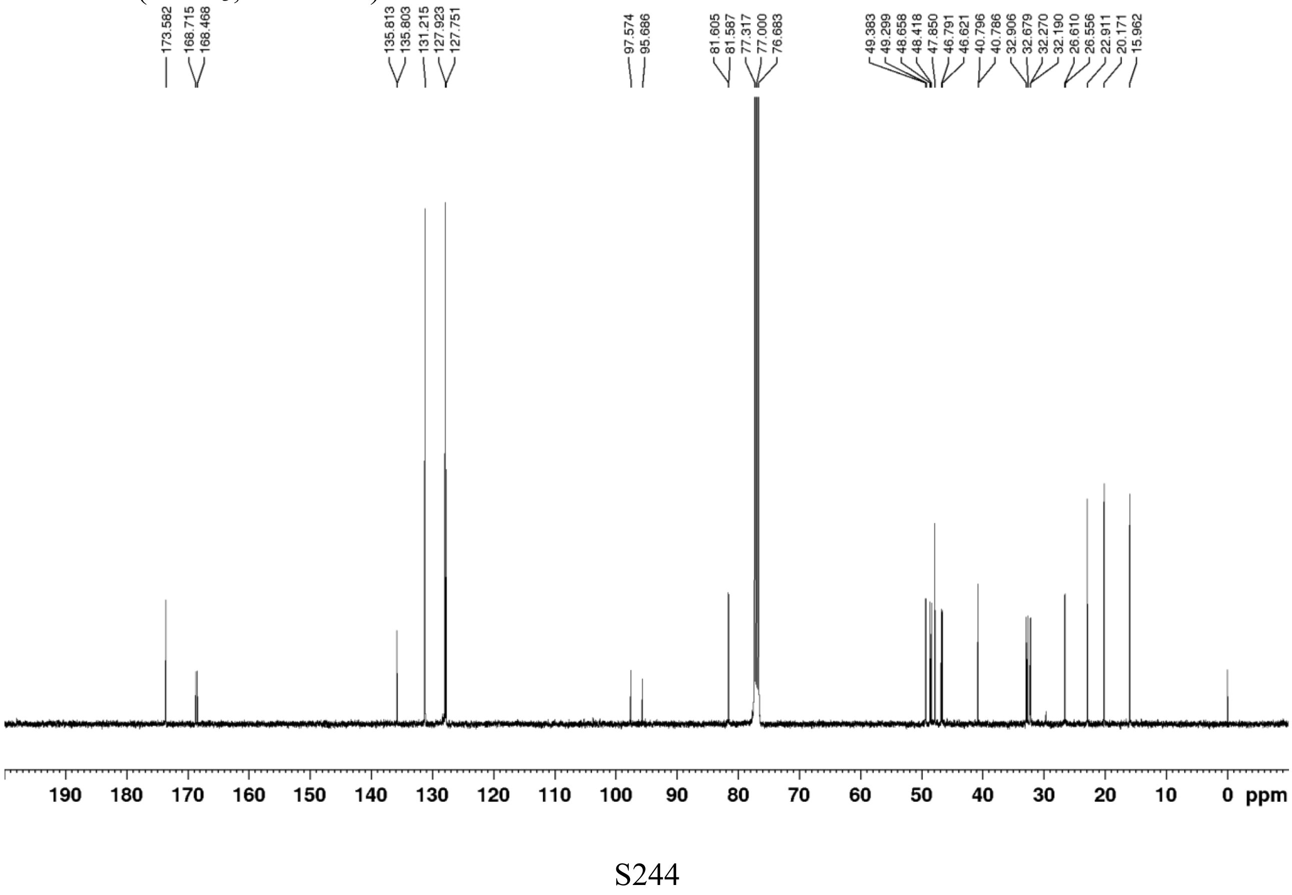

^{19}F NMR (CDCl$_3$, 377 MHz)

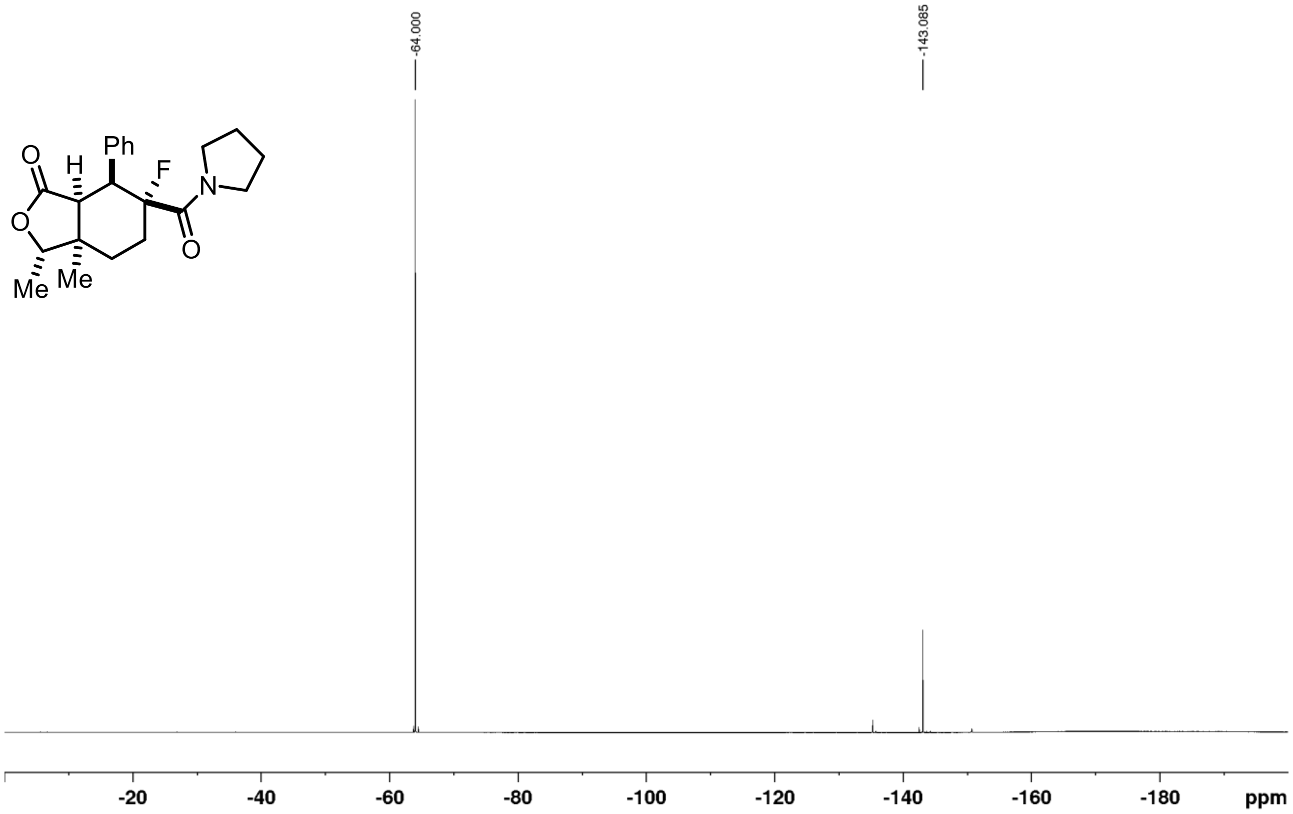

NOESY (CDCl$_3$, 400 MHz)

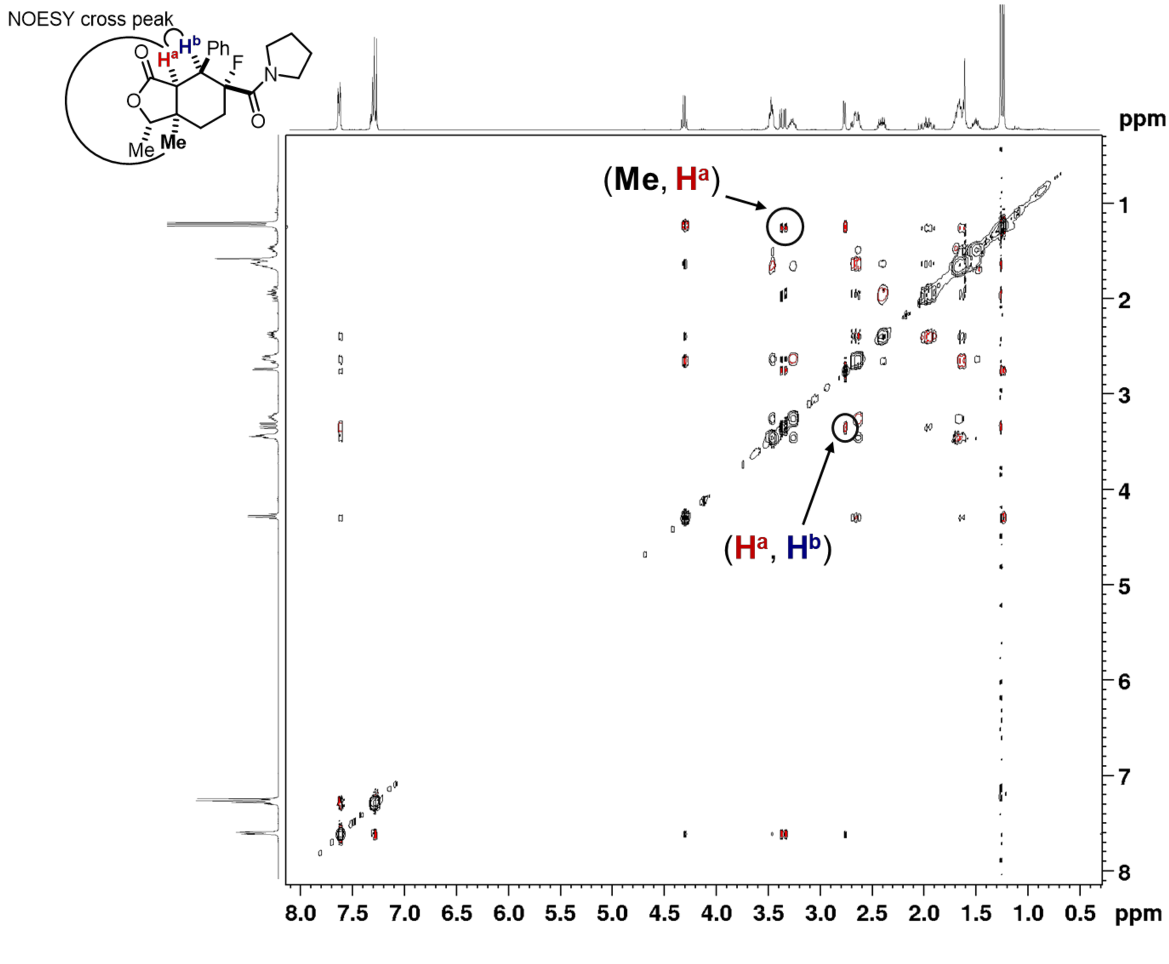

(4*S*,5*R*)-3-Benzyl-4-{(*E*)-3-fluoro-4-oxo-4-(pyrrolidin-1-yl)but-2-en-1-yl}-4,5-dimethyldihydrofuran-2(3*H*)-one (9)

Diastereomer mixture (86:14 d.r.)

^{1}H NMR ($CDCl_3$, 400 MHz)

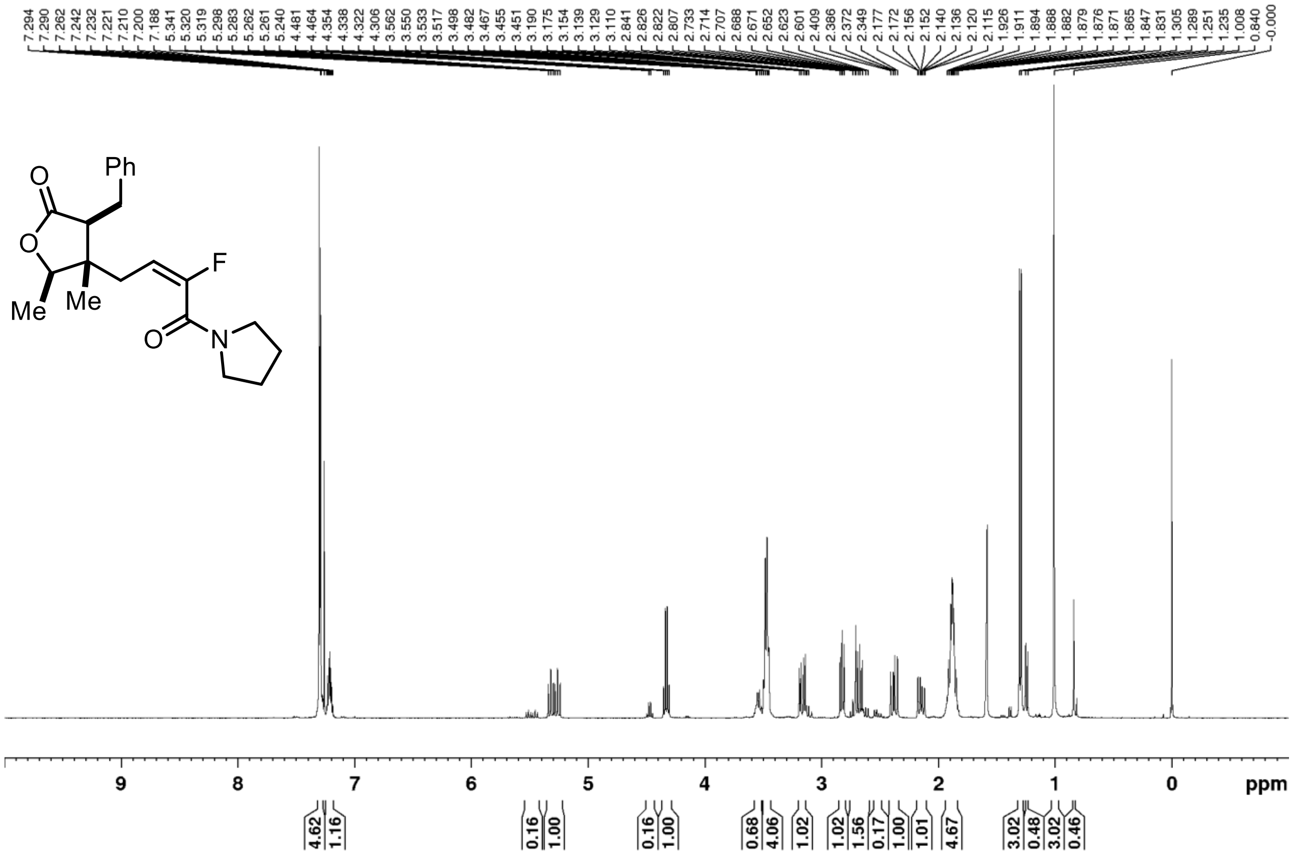

^{13}C NMR ($CDCl_3$, 101 MHz)

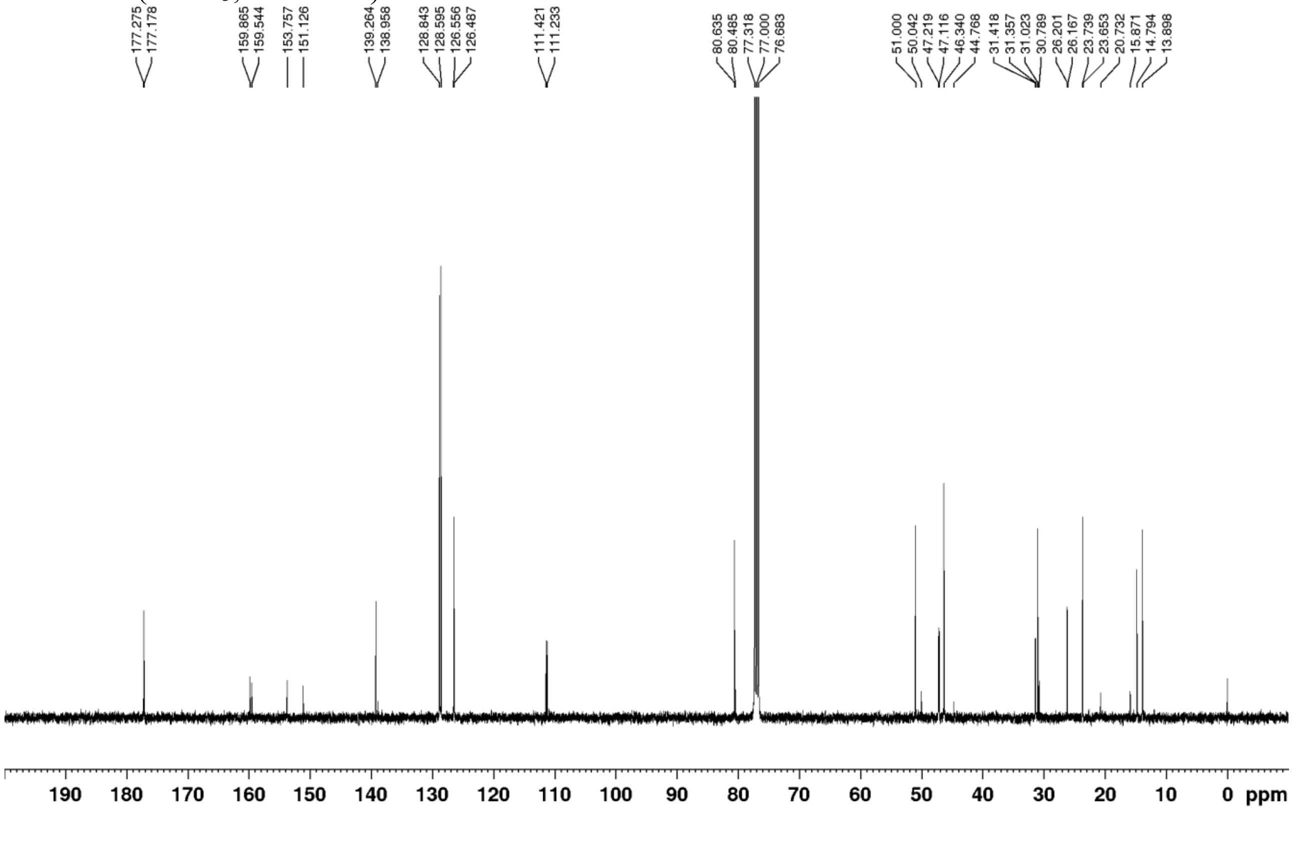

^{19}F NMR ($CDCl_3$, 377 MHz)

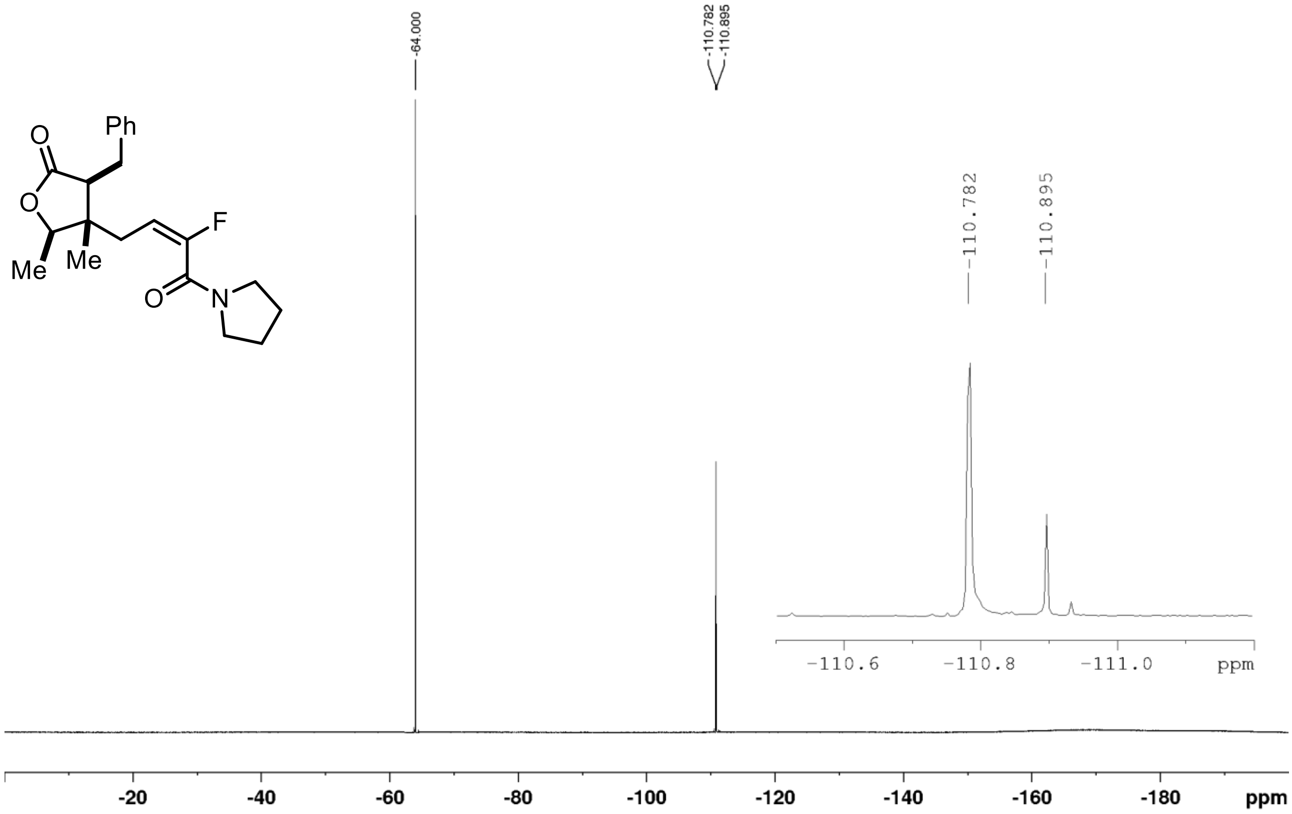

NOESY ($CDCl_3$, 400 MHz)

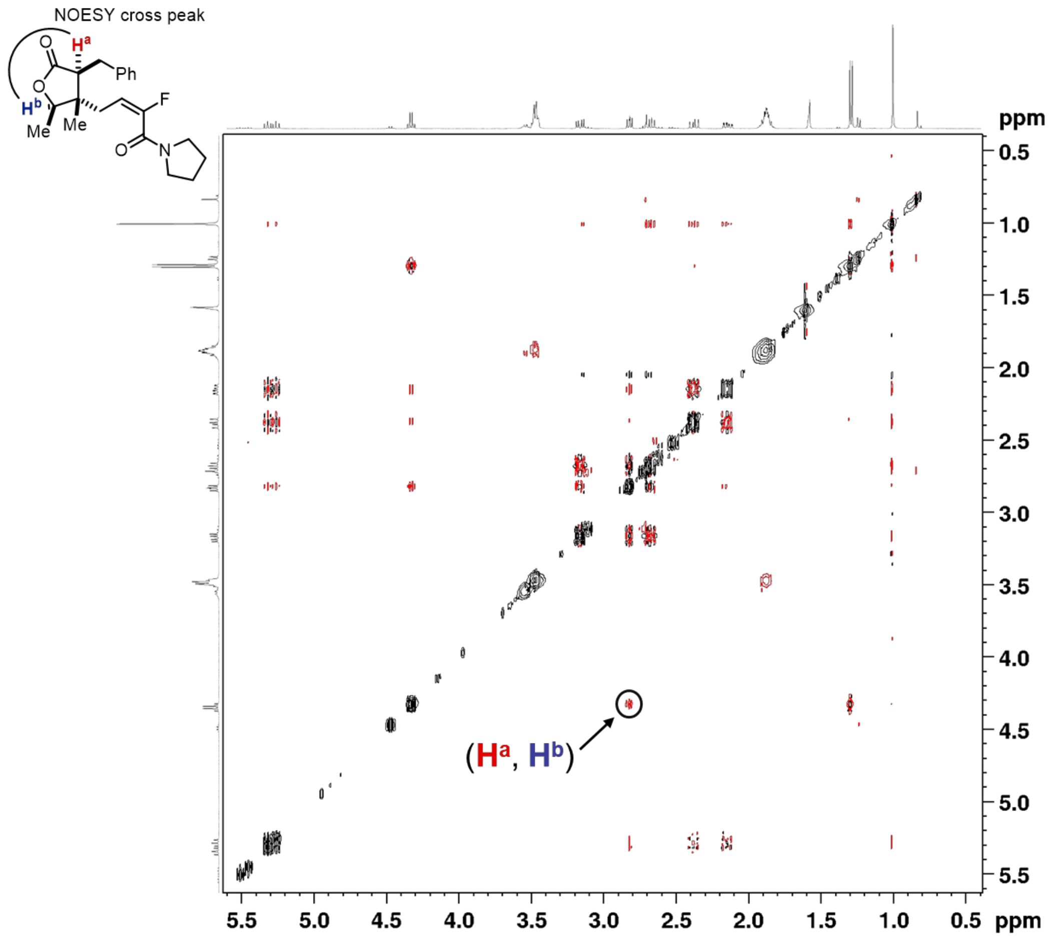

(3*S*,4*R*)-2-(*Z*)-Benzylidene-3-{(*E*)-3-fluoro-4-(pyrrolidin-1-yl)but-2-en-1-yl}-3-methylpentane-1,4-diol (10)

^{1}H NMR ($CDCl_3$, 400 MHz)

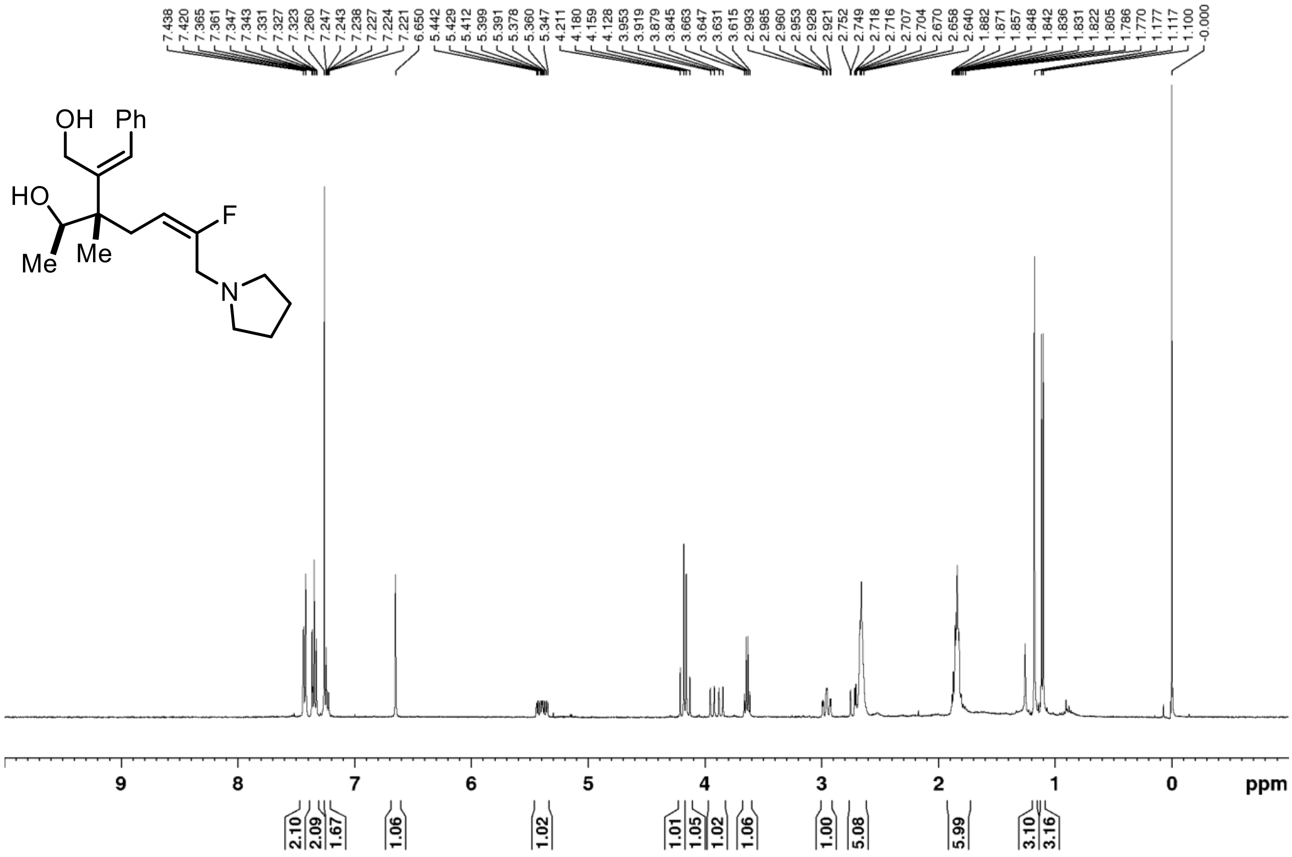

^{13}C NMR ($CDCl_3$, 101 MHz)

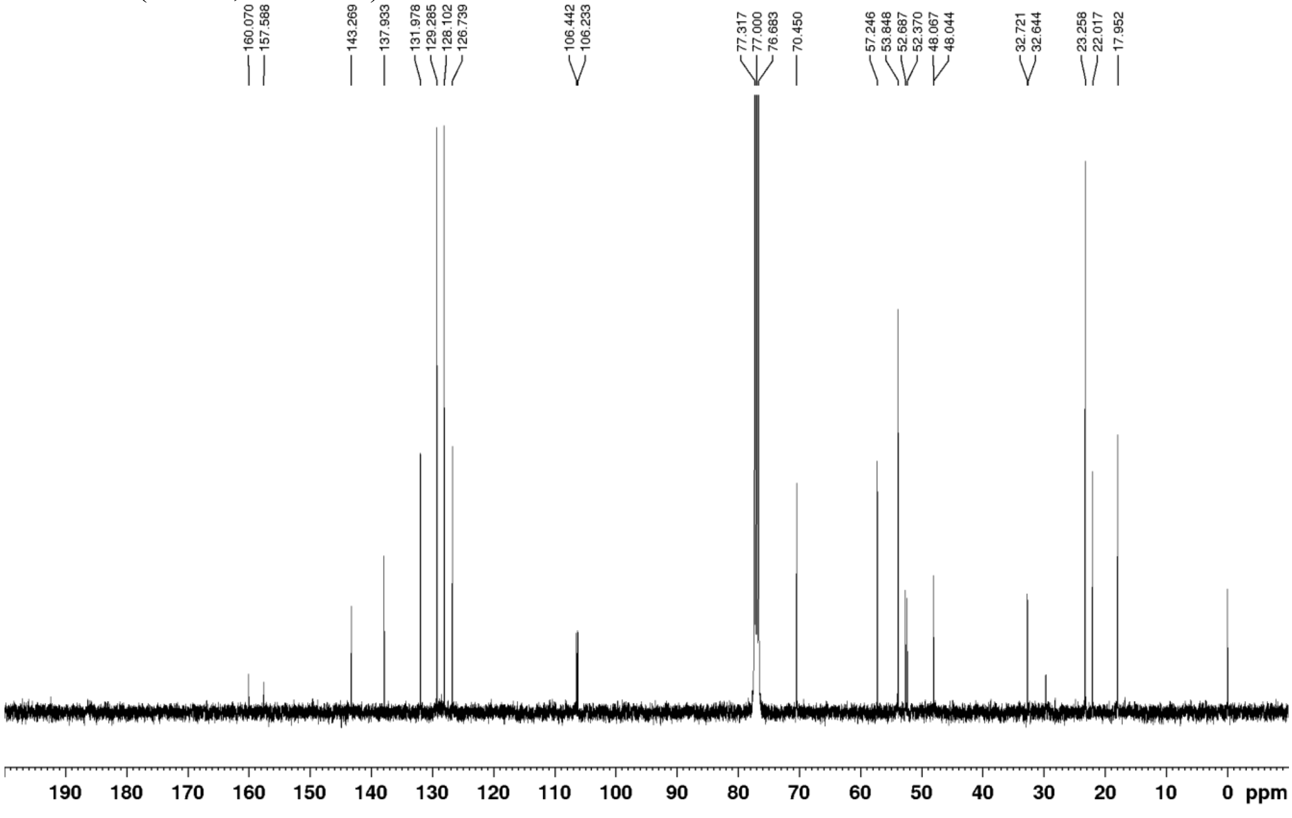

^{19}F NMR ($CDCl_3$, 377 MHz)

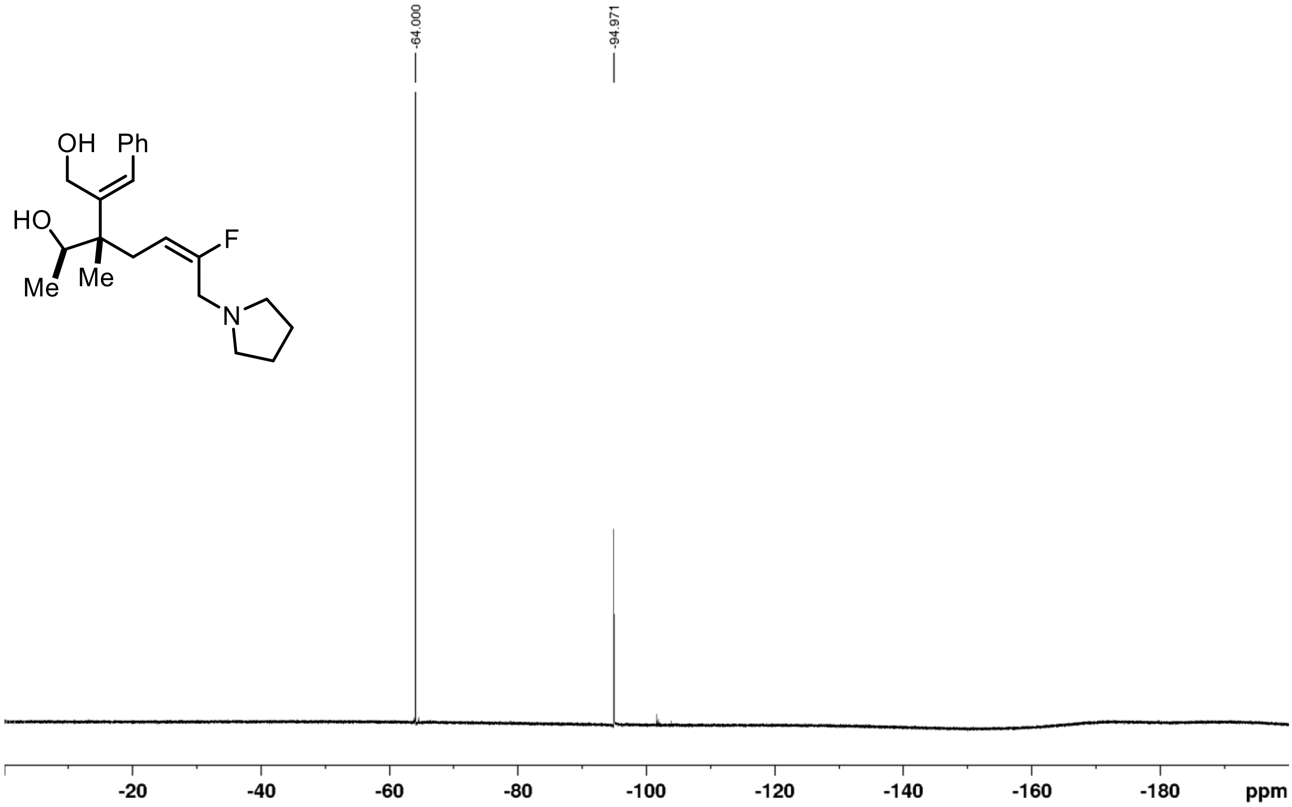

{(1*R*,5*R*,7a*R*)-5-Fluoro-2-(2-iodobenzoyl)-4-phenyl-1-vinyl-2,3,5,6,7,7a-hexahydro-1*H*-isoindol-5-yl}(pyrrolidin-1-yl)methanone (11)

Diastereomer mixture (85:15 d.r.)

^{1}H NMR ($CDCl_3$, 400 MHz)

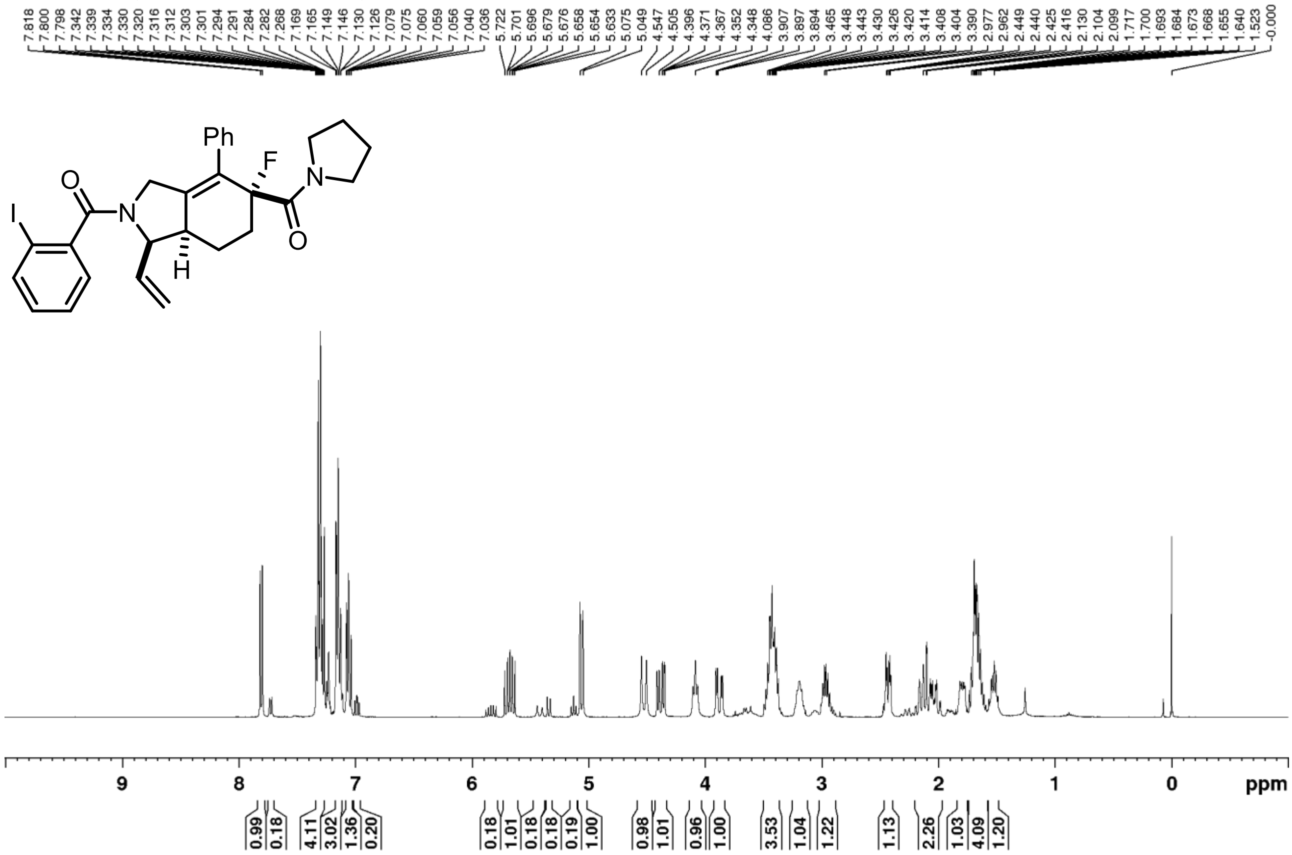

^{13}C NMR ($CDCl_3$, 101 MHz)

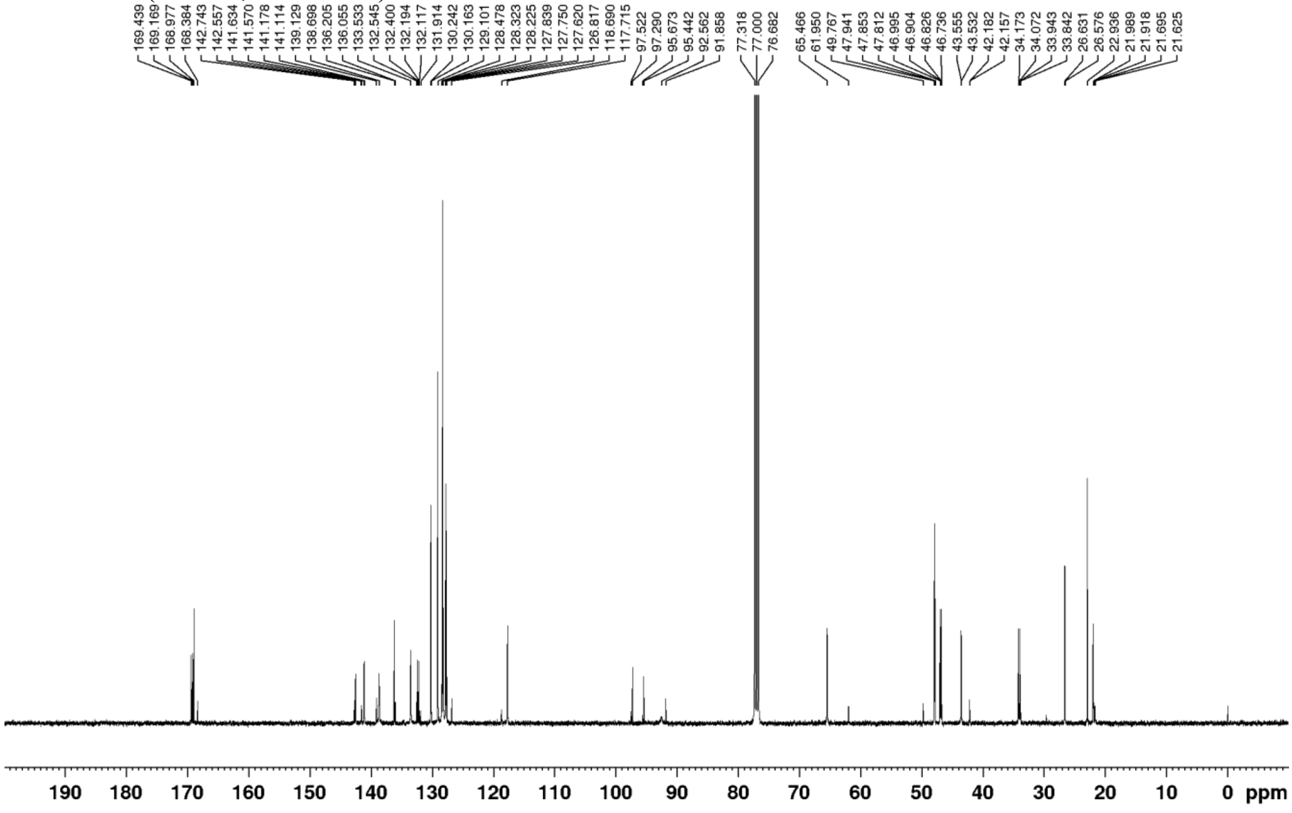

^{19}F NMR ($CDCl_3$, 377 MHz)

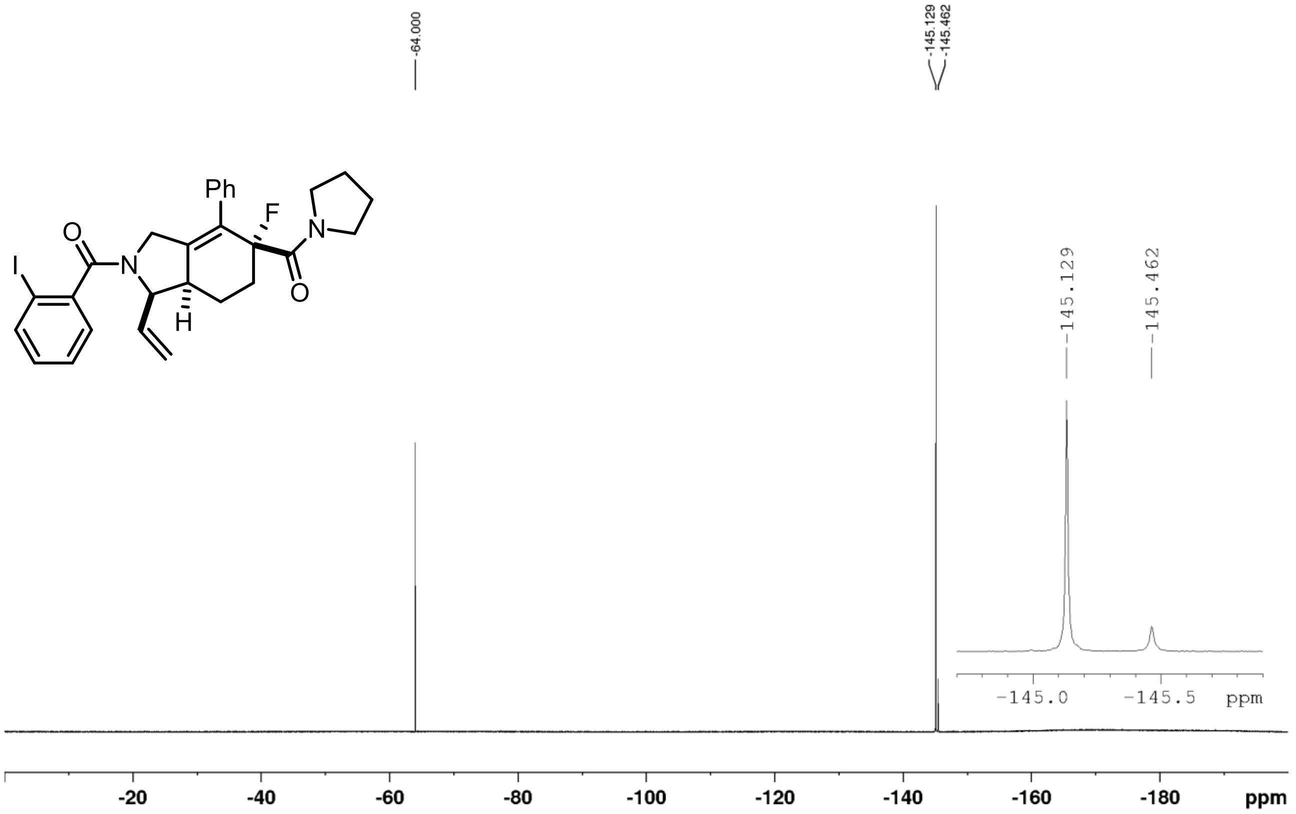

(9*R*,11a*R*,11b*S*)-9-Fluoro-12-methylene-8-phenyl-9-(pyrrolidine-1-carbonyl)-9,10,11,11a,11b,12-hexahydroisoindolo[2,1-b]isoquinolin-5(7*H*)-one (12)

^{1}H NMR (CDCl$_3$, 400 MHz)

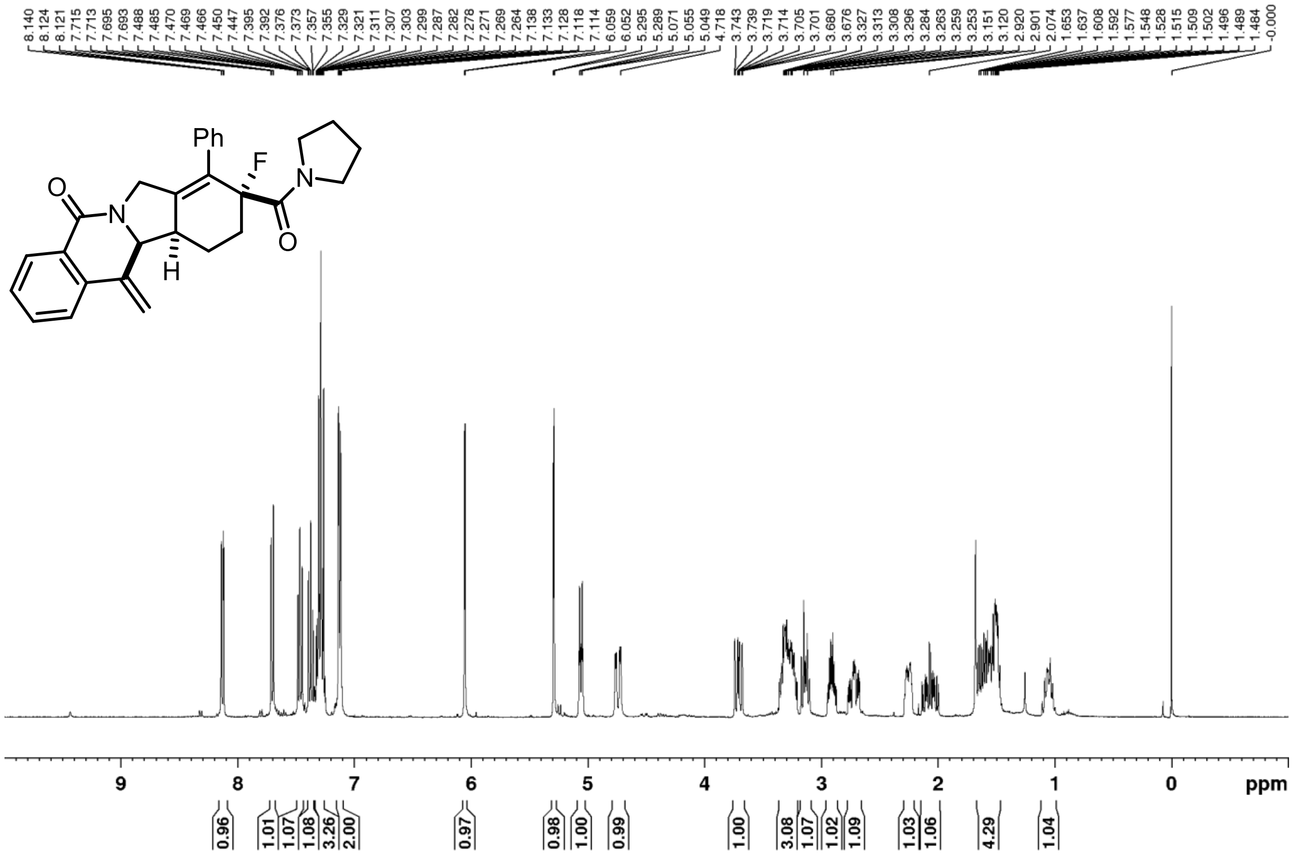

^{13}C NMR (CDCl$_3$, 101 MHz)

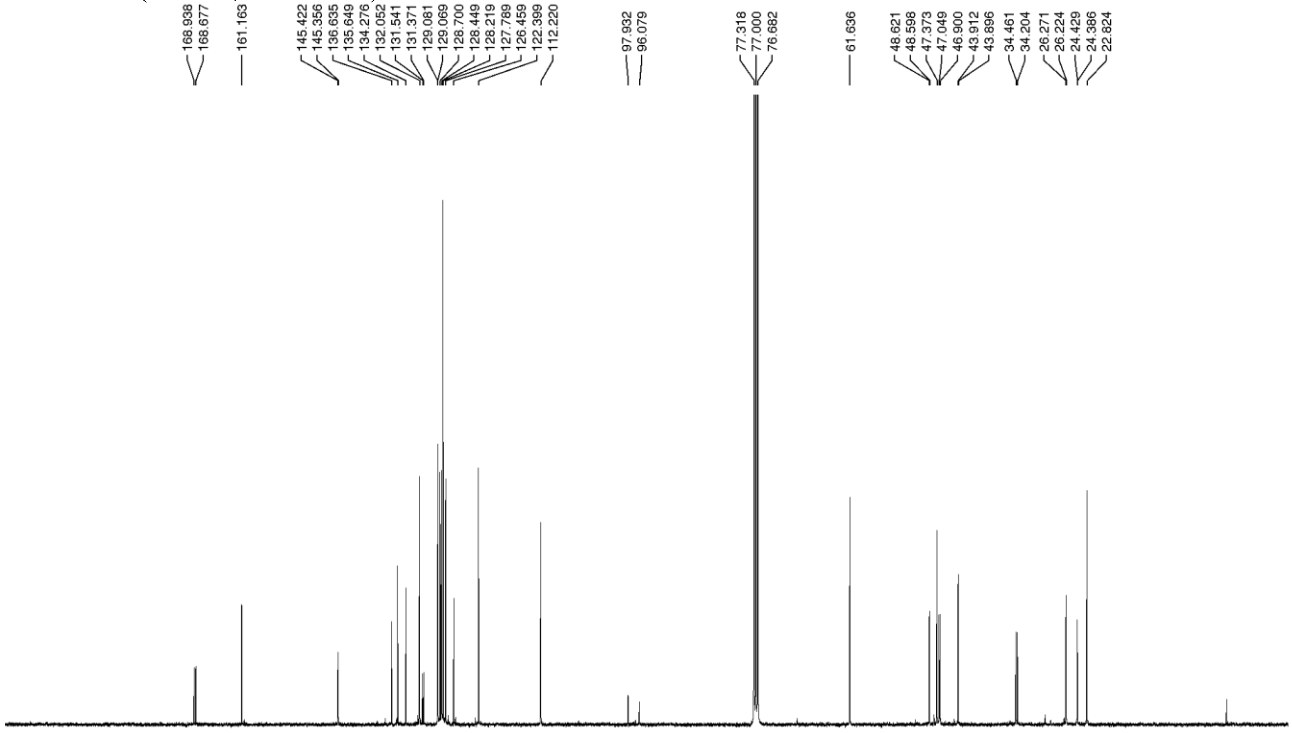

^{19}F NMR ($CDCl_3$, 377 MHz)

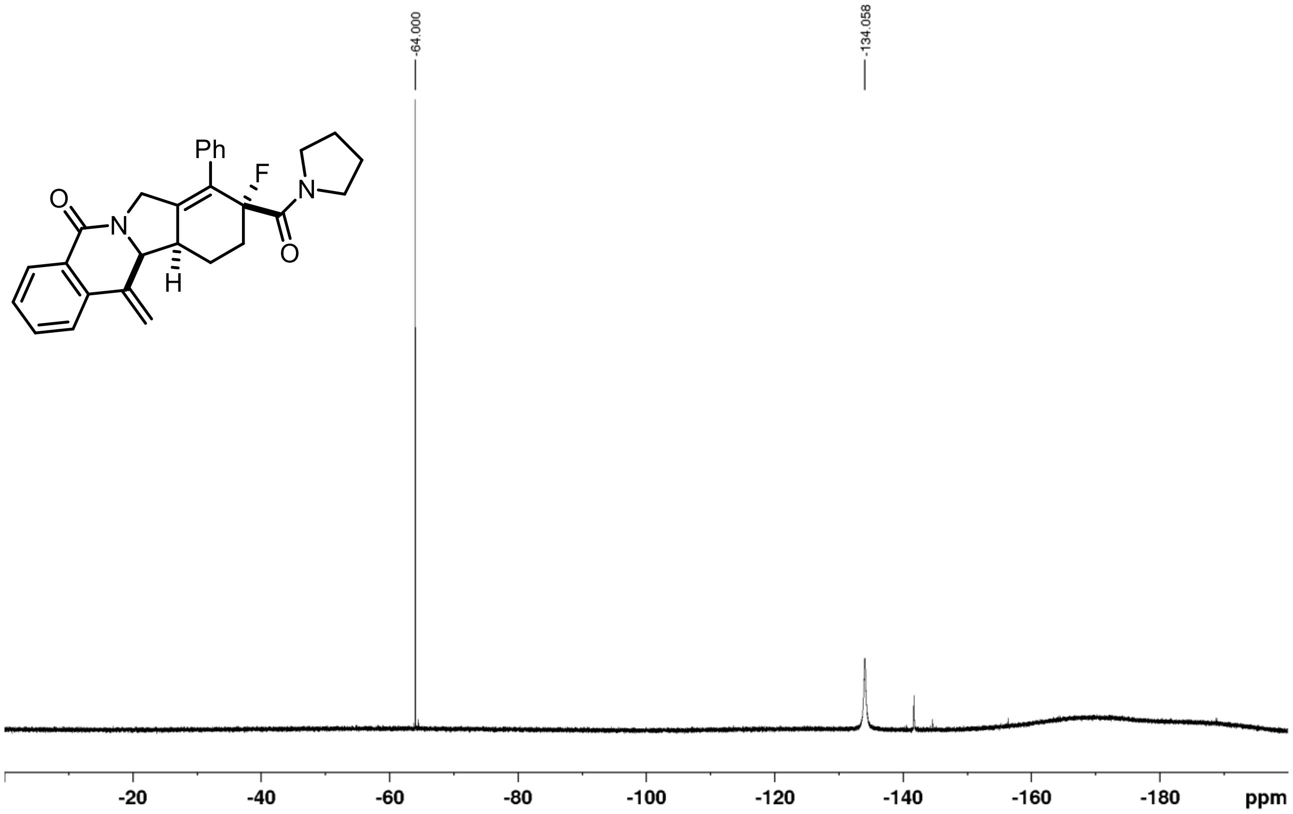

6.6. Experimental Mechanistic Studies (Figure 5c)

{(1*S*,5*R*,7a*R*)-5-Fluoro-1,7a-dimethyl-2-(4-methylphenyl)-4-phenyl-2,3,5,6,7,7a-hexahydro-1*H*-isoindol-5-yl-6,6-d_2}(pyrrolidin-1-yl)methanone [3da-D_2 (major diastereomer)]

^{1}H NMR ($CDCl_3$, 400 MHz)

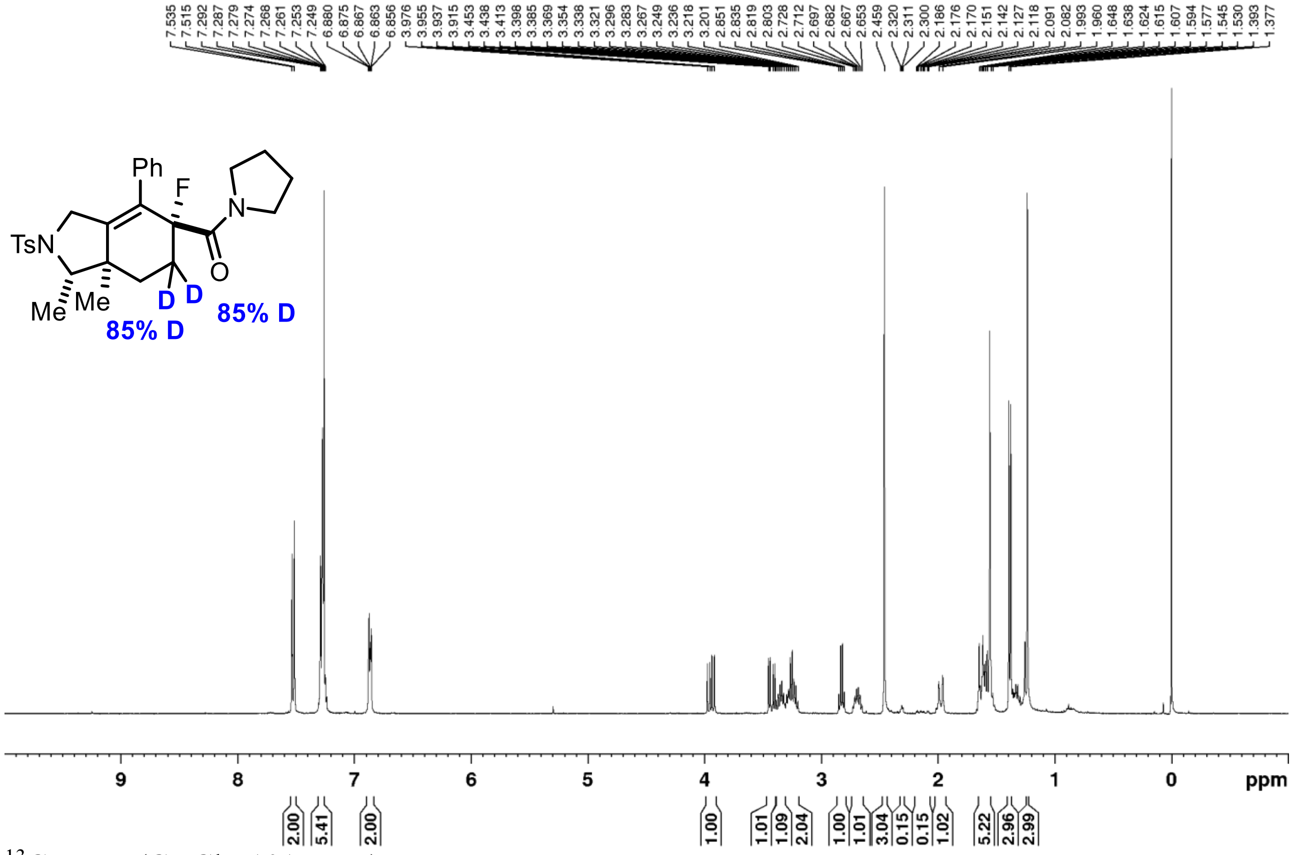

^{13}C NMR ($CDCl_3$, 101 MHz)

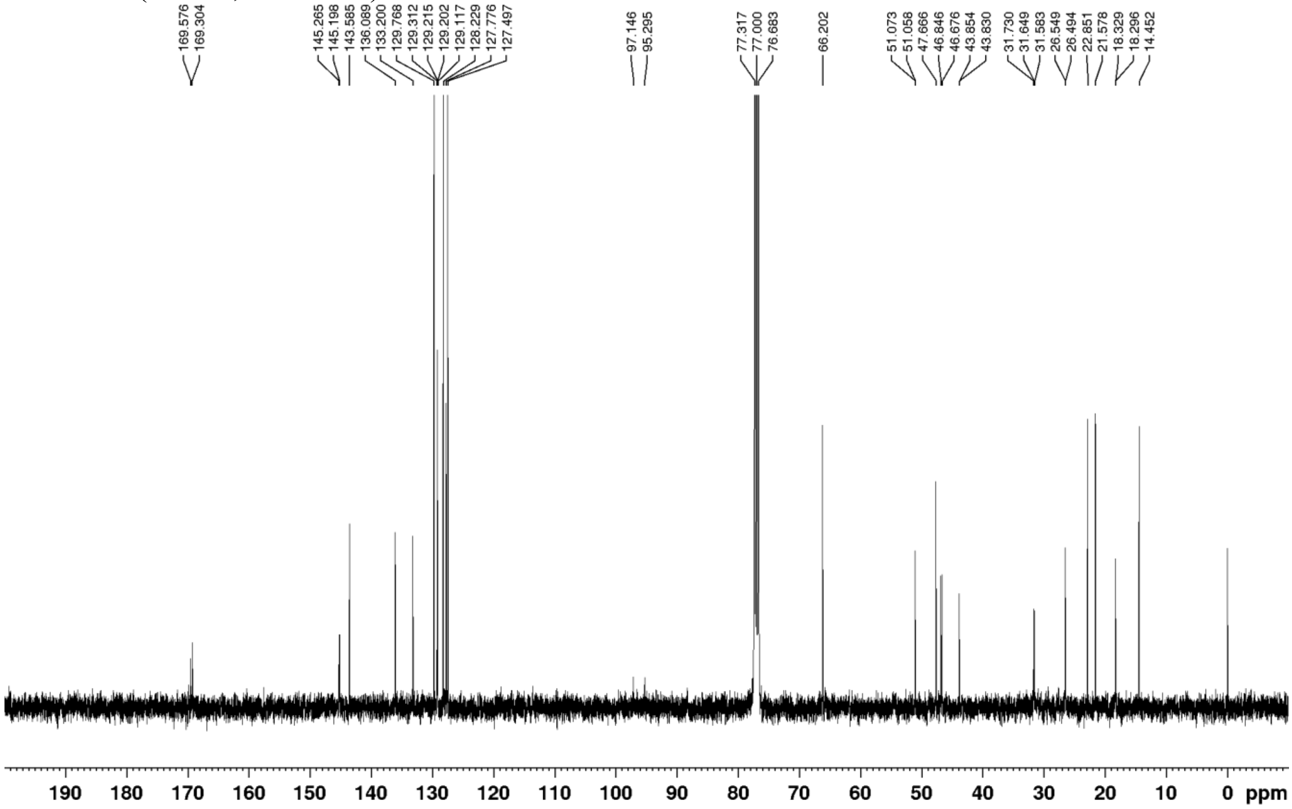

(*E*)-4-{(2*S*,3*R*,*Z*)-2,3-Dimethyl-1-(4-methylphenyl)-4-(phenylmethylene-*d*)pyrrolidin-3-yl}-2-fluoro-1-(pyrrolidin-1-yl)but-2-en-1-one-3-*d* (4da-D_2)

Diastereomer mixture (63:37 d.r.)

^{1}H NMR ($CDCl_3$, 400 MHz)

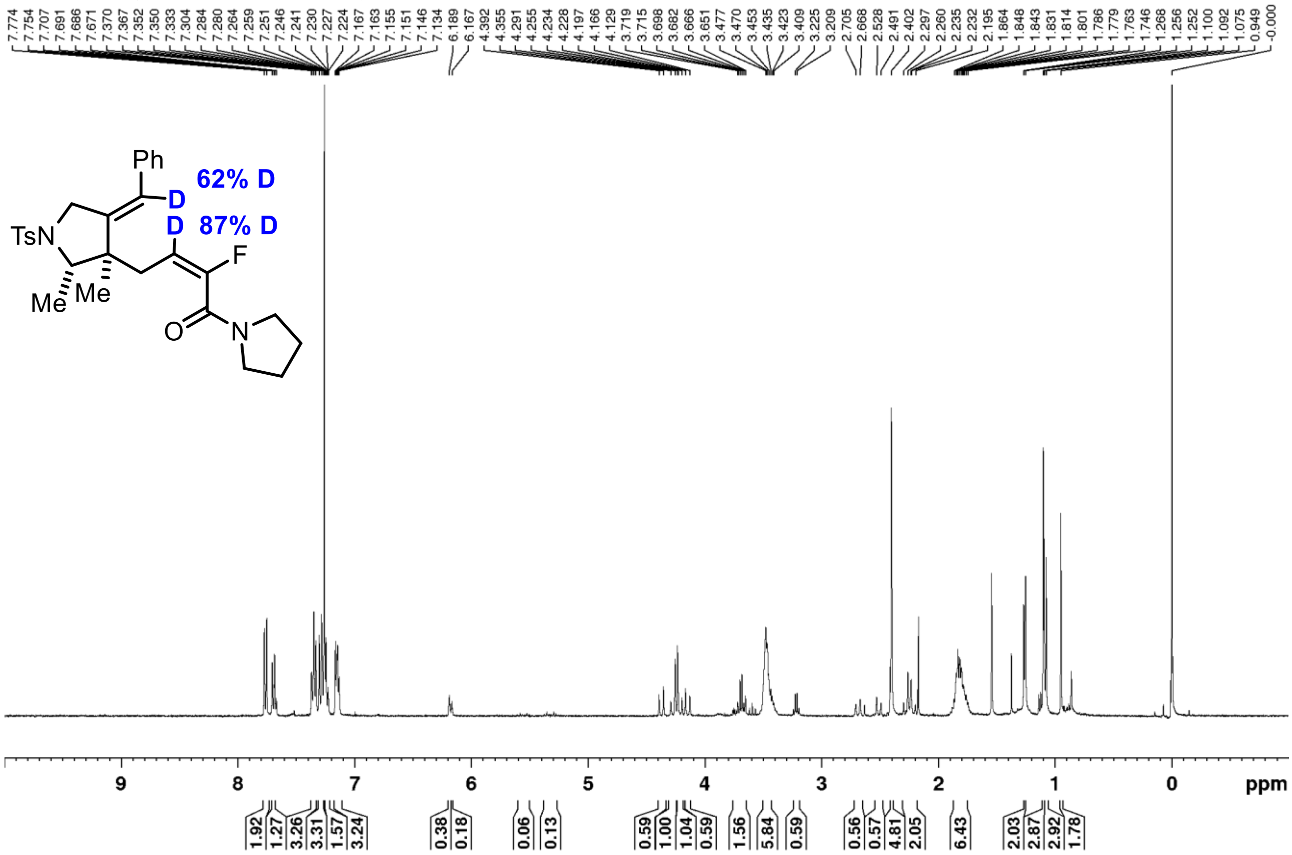

(3*S*,3a*R*,6*R*)-6-Fluoro-3,3a-dimethyl-7-phenyl-6-(pyrrolidine-1-carbonyl)-3a,4,5,6-tetrahydroisobenzofuran-1(3*H*)-one-5,5-d_2 (3ha-D_2)

^{1}H NMR ($CDCl_3$, 400 MHz)

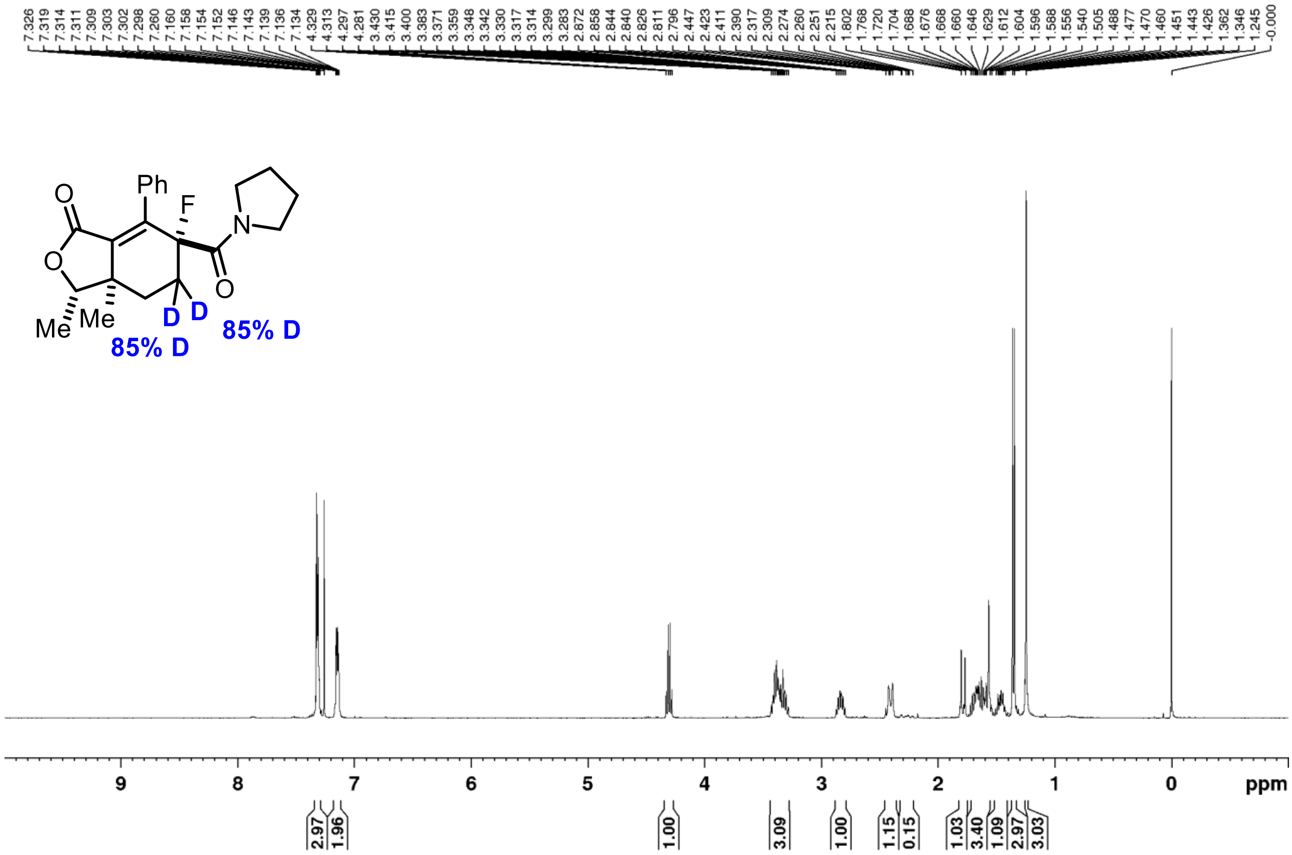

^{13}C NMR ($CDCl_3$, 101 MHz)

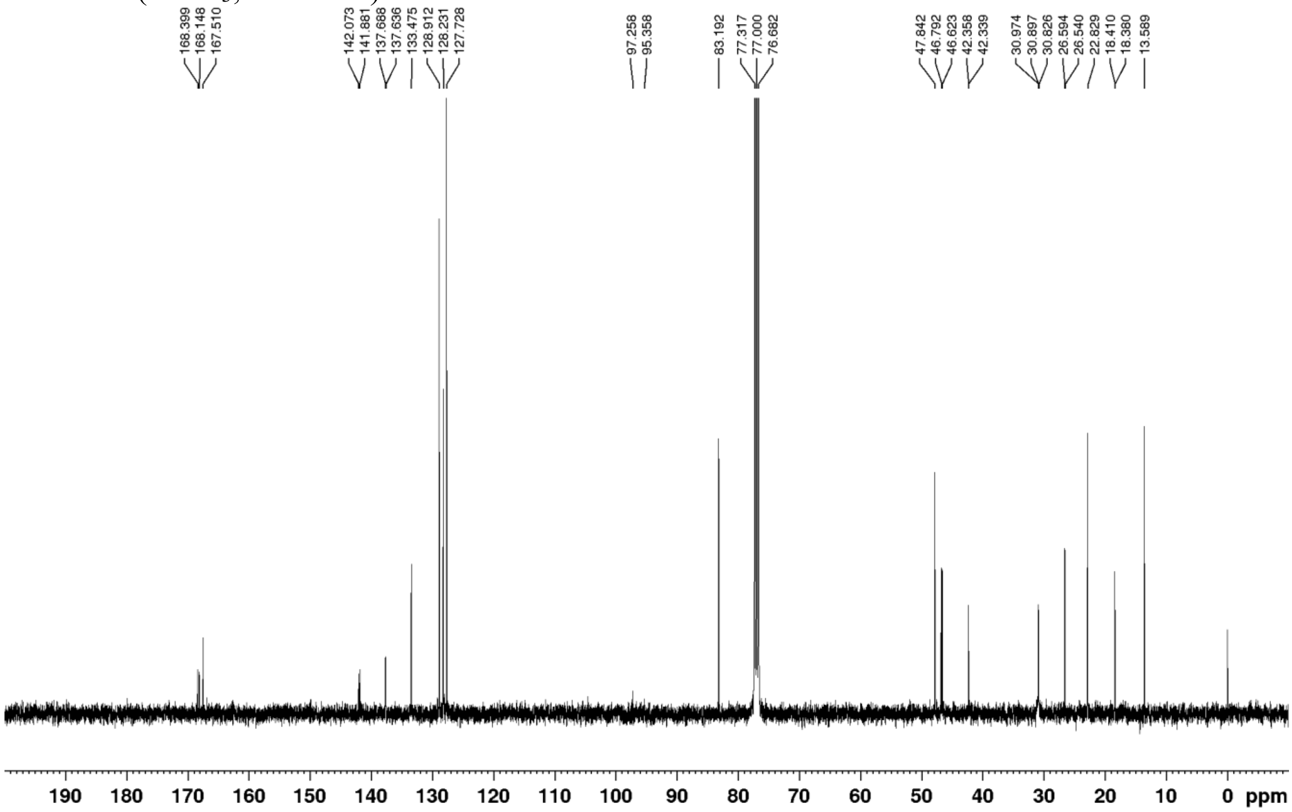

(4*R*,5*S*,*Z*)-4-{(*E*)-3-Fluoro-4-oxo-4-(pyrrolidin-1-yl)but-2-en-1-yl-2-*d*}-4,5-dimethyl-3-(phenylmethylene-*d*)dihydrofuran-2(3*H*)-one (4ha-D_2)

^{1}H NMR ($CDCl_3$, 400 MHz)

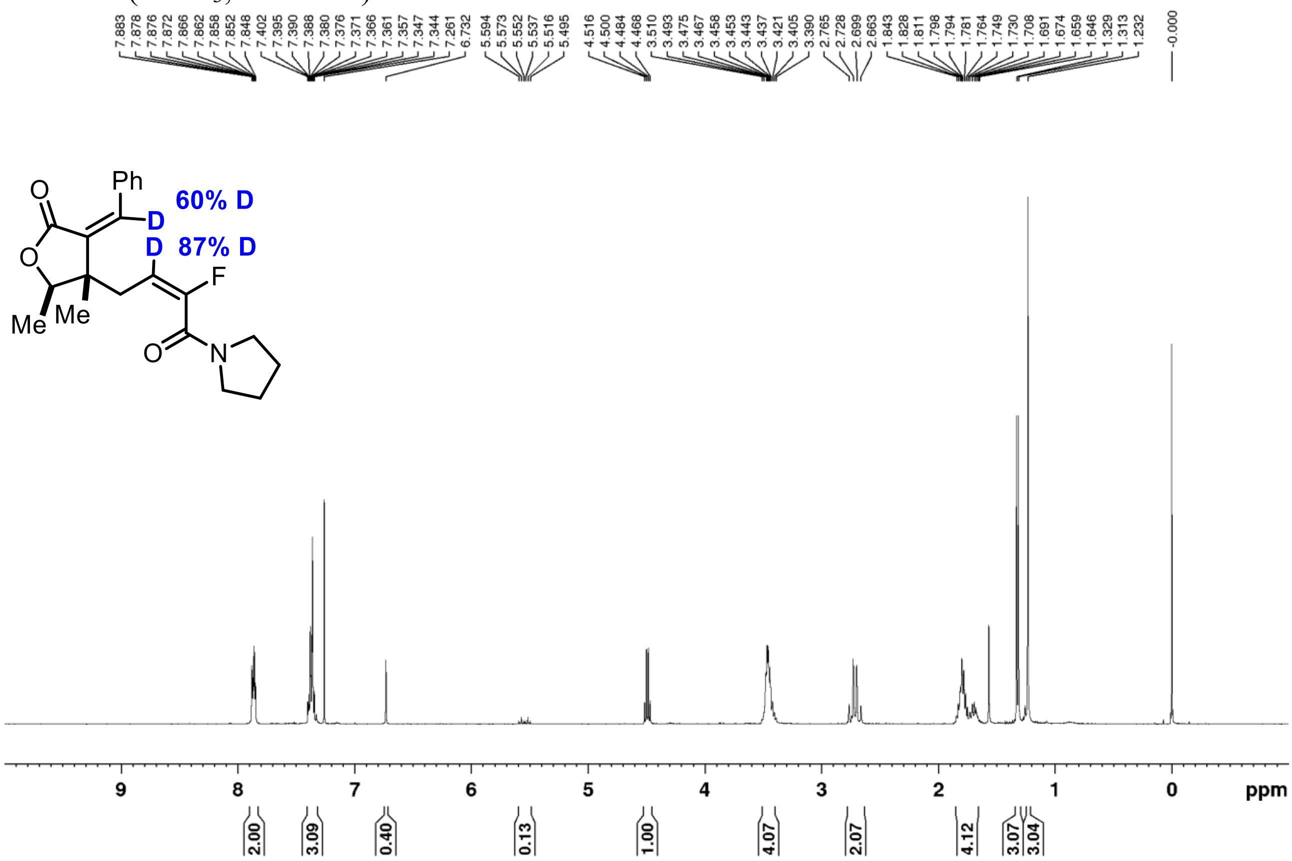

^{13}C NMR ($CDCl_3$, 101 MHz)

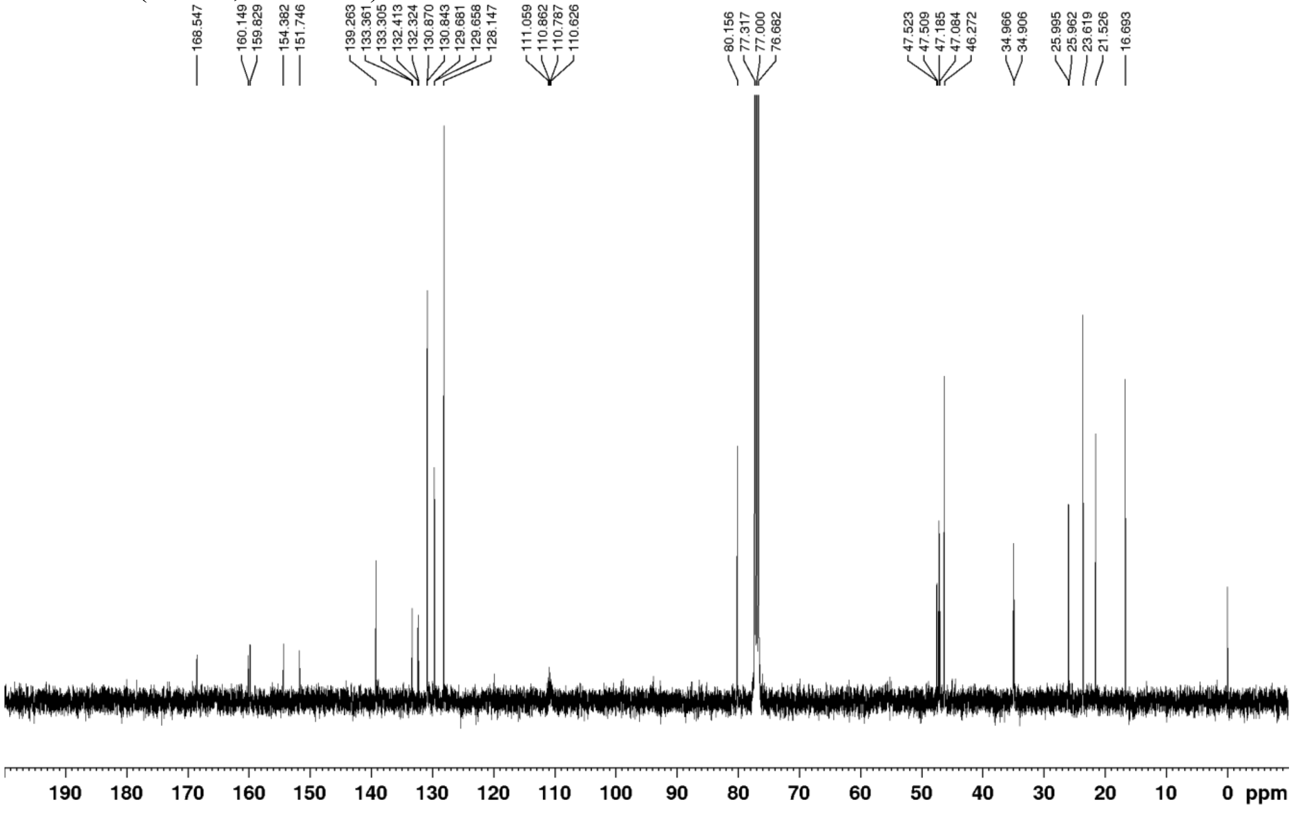

7. Chiral HPLC Charts

7.1. Rh-Catalyzed Enantioselective PKR (type I) of Racemic 1,6-Enynes with α-Fluoroacrylamides (Figure 2)

(–)-{(1*S*,5*R*,7a*R*)-5-Fluoro-1,4,7a-trimethyl-2-(4-methylphenyl)-2,3,5,6,7,7a-hexahydro-1*H*-isoindol-5-yl}(pyrrolidin-1-yl)methanone [(–)-3aa (major diastereomer)]

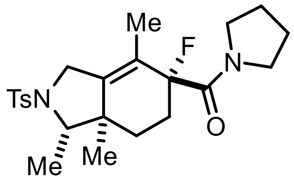

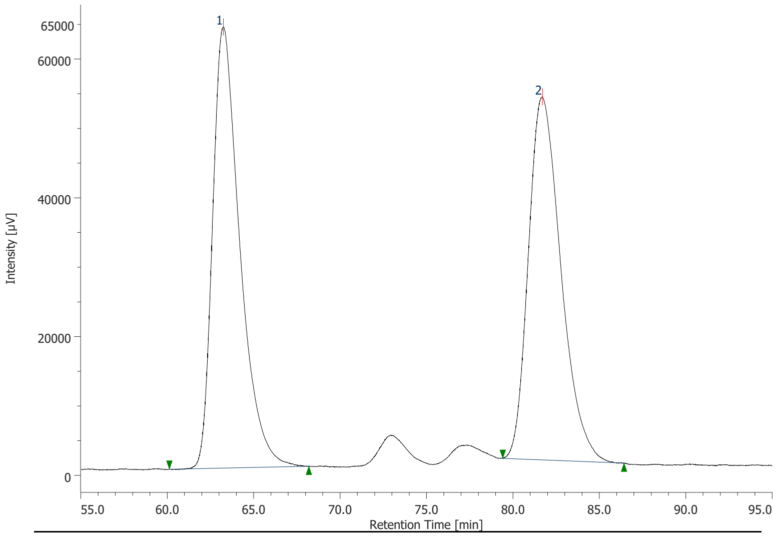

Peak No.	Retention Time (min)	Area (%)
1	63.250	50.405
2	81.700	49.595

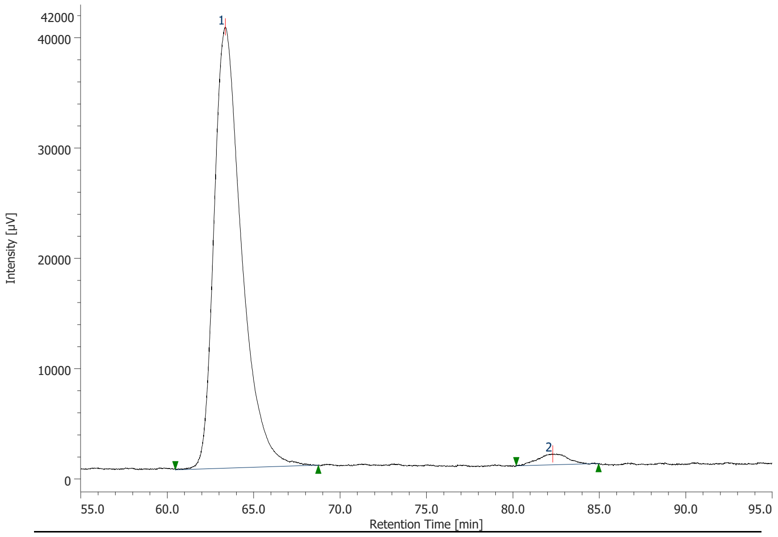

Peak No.	Retention Time (min)	Area (%)
1	63.367	97.410
2	82.300	2.590

(+)-(*E*)-4-{(2*R*,3*R*,*Z*)-4-Ethylidene-2,3-dimethyl-1-(4-methylphenyl)pyrrolidin-3-yl}-2-fluoro-1-(pyrrolidin-1-yl)but-2-en-1-one [(+)-4aa (major diastereomer)]

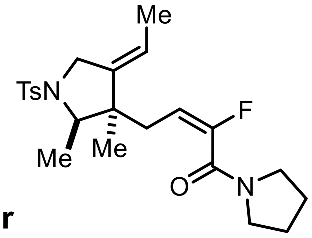

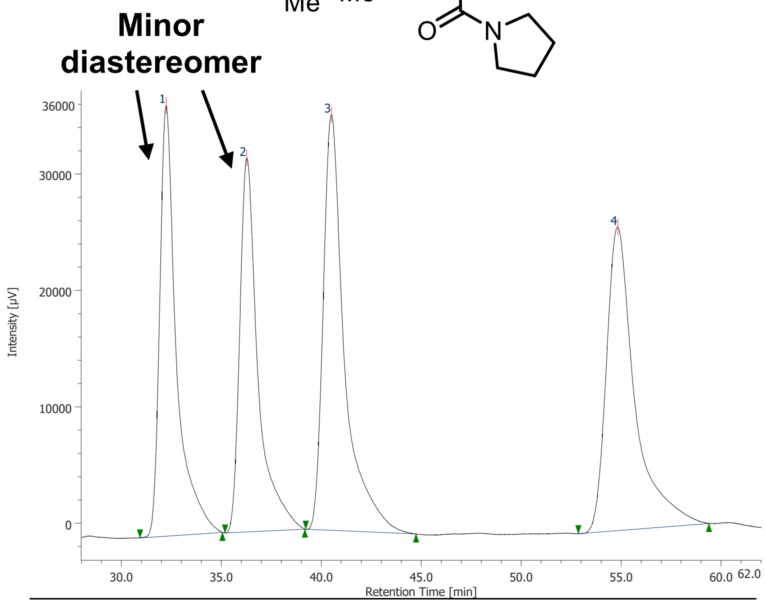

Peak No.	Retention Time (min)	Area (%)
1	32.242	22.857
2	36.275	21.629
**3**	**40.508**	**28.053**
**4**	**54.833**	**27.461**

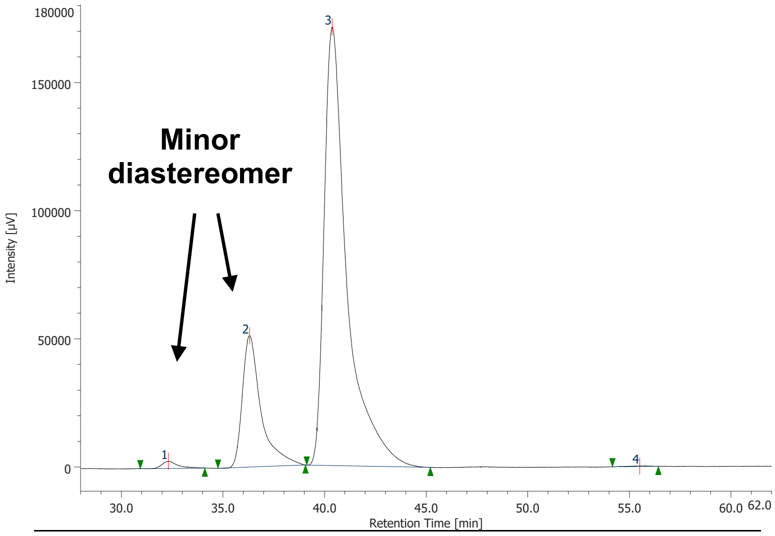

Peak No.	Retention Time (min)	Area (%)
1	32.317	0.917
2	36.308	19.413
**3**	**40.375**	**79.569**
**4**	**55.500**	**0.102**

(–)-{(1*S*,5*R*,7a*R*)-5-Fluoro-4,7a-dimethyl-2-(4-methylphenyl)-1-pentyl-2,3,5,6,7,7a-hexahydro-1*H*-isoindol-5-yl}(pyrrolidin-1-yl)methanone [(–)-3ba (major diastereomer)]

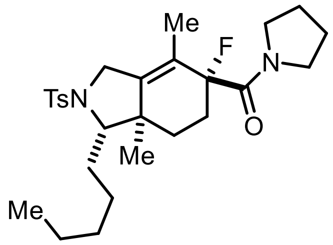

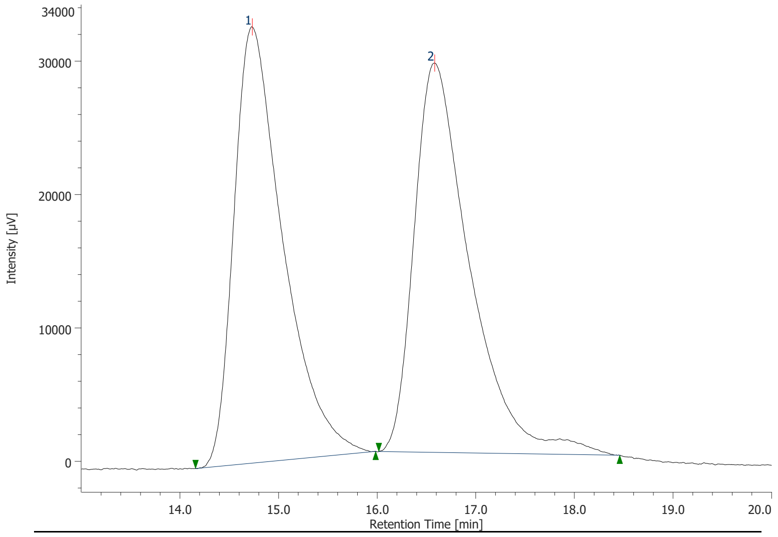

Peak No.	Retention Time (min)	Area (%)
1	14.733	49.738
2	16.583	50.262

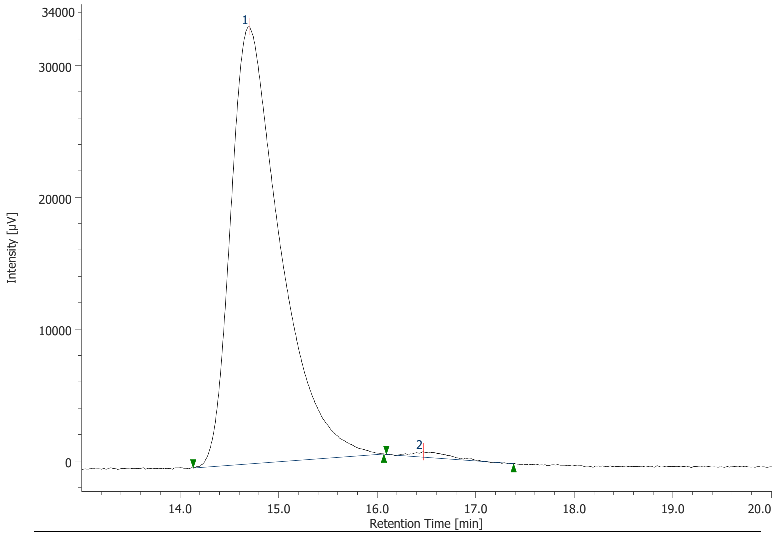

Peak No.	Retention Time (min)	Area (%)
1	14.700	99.057
2	16.467	0.943

(+)-(*E*)-4-{(2*R*,3*R*,*Z*)-4-Ethylidene-3-methyl-1-(4-methylphenyl)pyrrolidin-3-yl}-2-pentyl-2-fluoro-1-(pyrrolidin-1-yl)but-2-en-1-one [(+)-4ba (major diastereomer)]

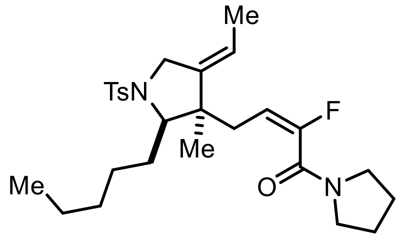

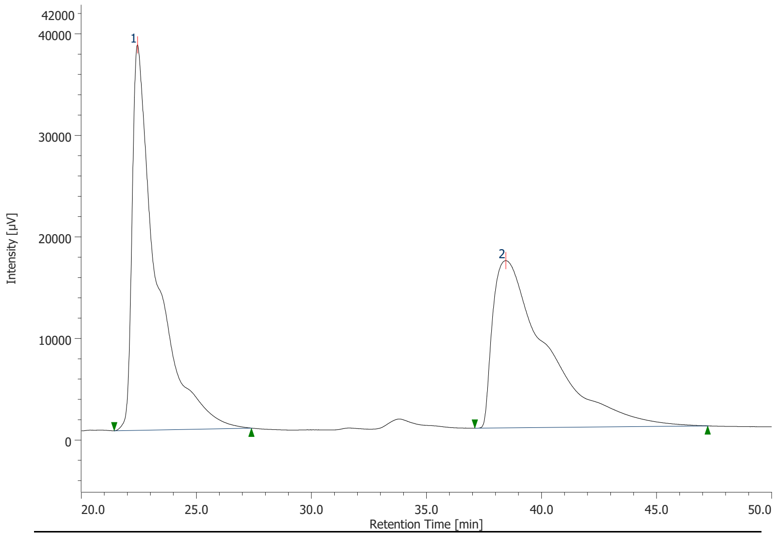

Peak No.	Retention Time (min)	Area (%)
1	22.442	50.493
2	38.442	49.507

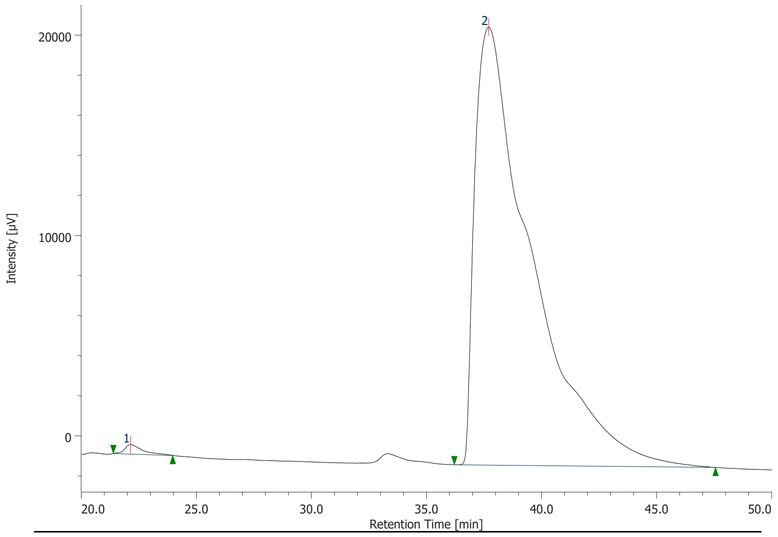

Peak No.	Retention Time (min)	Area (%)
1	22.142	0.684
2	37.700	99.316

(–)-{(1*S*,5*R*,7a*R*)-5-Fluoro-4,7a-dimethyl-2-(4-methylphenyl)-1-phenyl-2,3,5,6,7,7a-hexahydro-1*H*-isoindol-5-yl}(pyrrolidin-1-yl)methanone [(–)-3ca]

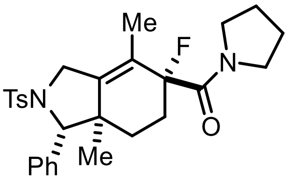

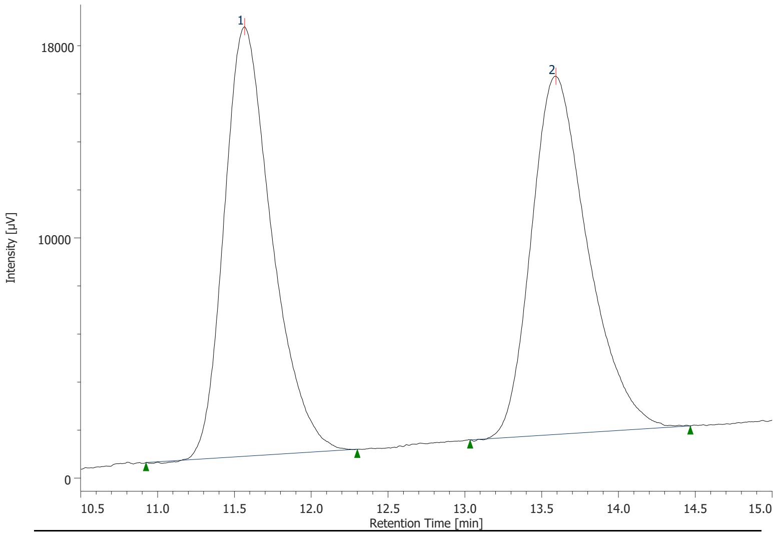

Peak No.	Retention Time (min)	Area (%)
1	11.567	50.290
2	13.592	49.710

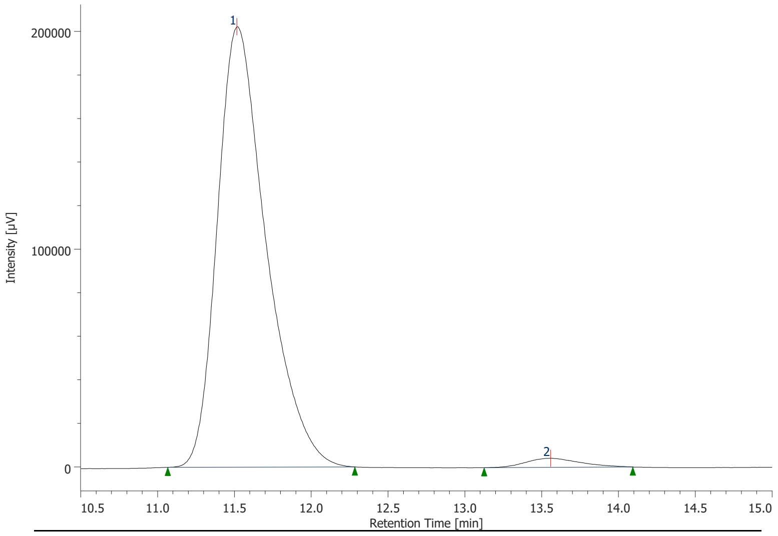

Peak No.	Retention Time (min)	Area (%)
1	11.517	97.815
2	13.558	2.185

(–)-(*E*)-4-{(2*R*,3*R*,*Z*)-4-Ethylidene-3-methyl-2-phenyl-1-(4-methylphenyl)pyrrolidin-3-yl}-2-fluoro-1-(pyrrolidin-1-yl)but-2-en-1-one [(–)-4ca (major diastereomer)]

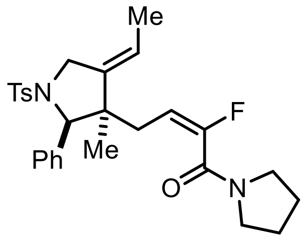

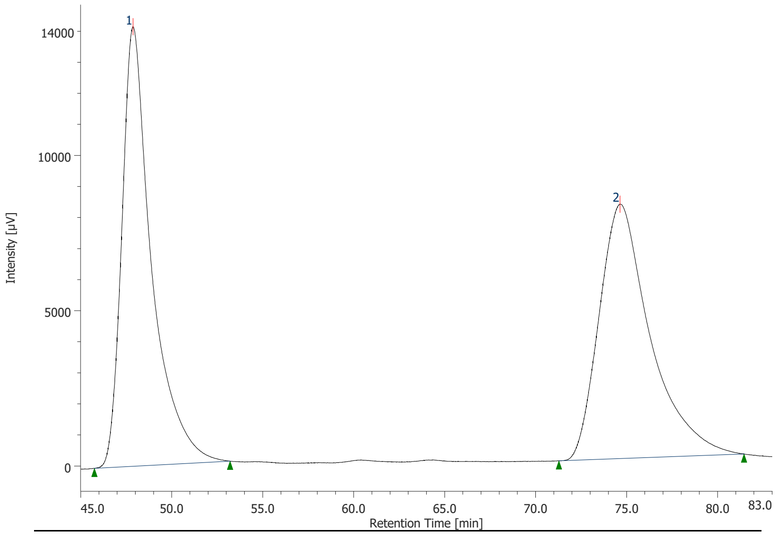

Peak No.	Retention Time (min)	Area (%)
1	47.875	50.197
2	74.633	49.803

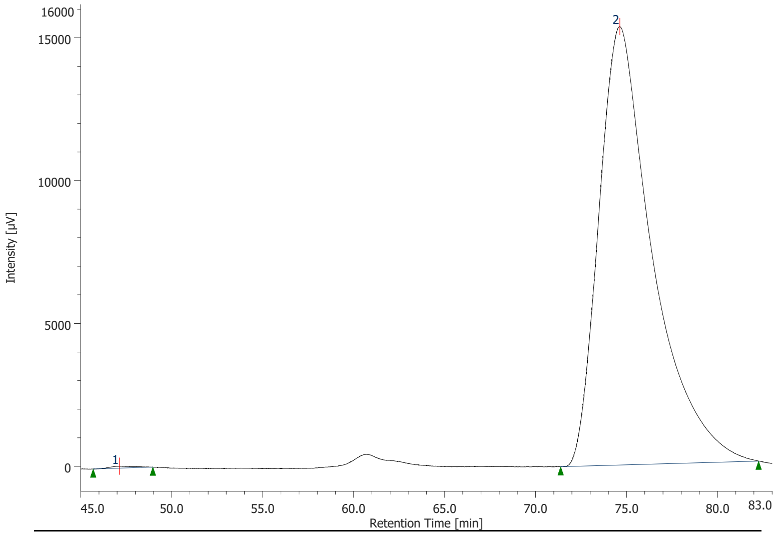

Peak No.	Retention Time (min)	Area (%)
1	47.117	0.192
2	74.617	99.808

(–)-{(1*S*,5*R*,7a*R*)-5-Fluoro-1,7a-dimethyl-2-(4-methylphenyl)-4-phenyl-2,3,5,6,7,7a-hexahydro-1*H*-isoindol-5-yl}(pyrrolidin-1-yl)methanone [(–)-3da (major diastereomer)]

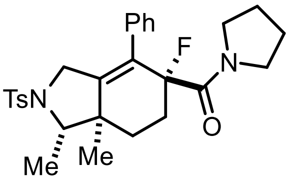

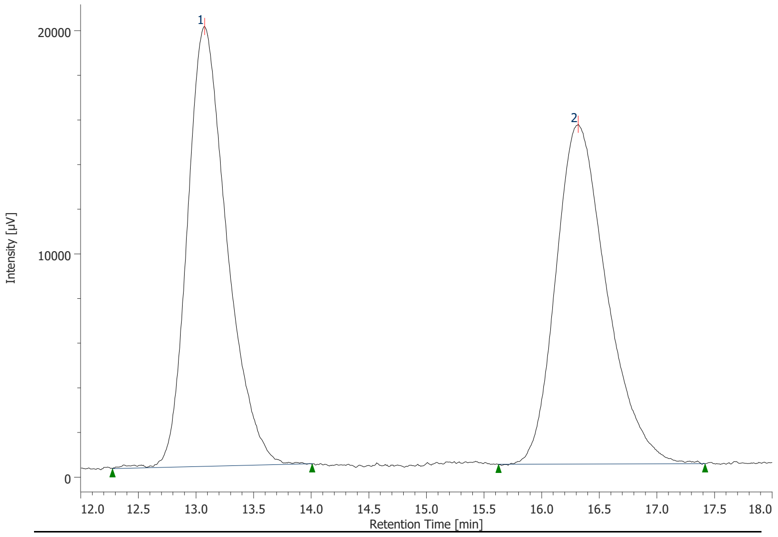

Peak No.	Retention Time (min)	Area (%)
1	13.075	50.236
2	16.317	49.764

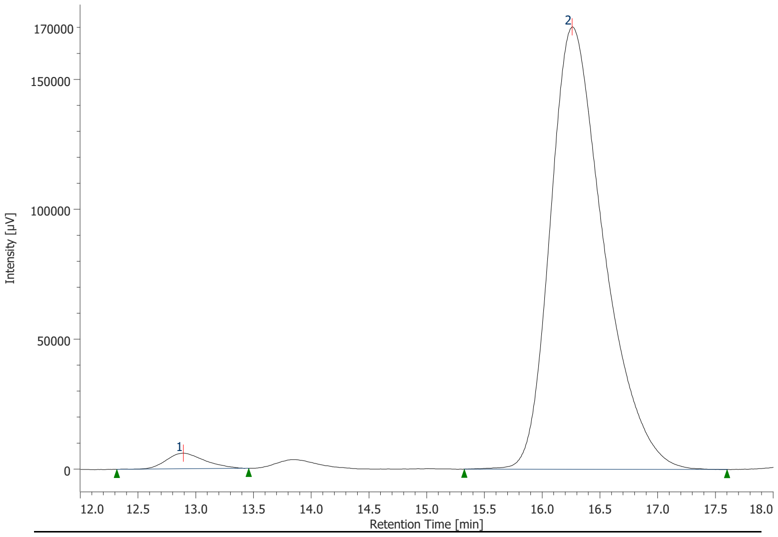

Peak No.	Retention Time (min)	Area (%)
1	12.892	2.434
2	16.258	97.566

(–)-(*E*)-4-{(2*R*,3*R*)-4-(*Z*)-Benzylidene-2,3-dimethyl-1-(4-methylphenyl)pyrrolidin-3-yl}-2-fluoro-1-(pyrrolidin-1-yl)but-2-en-1-one [(–)-4da (major diastereomer)]

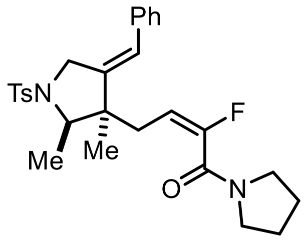

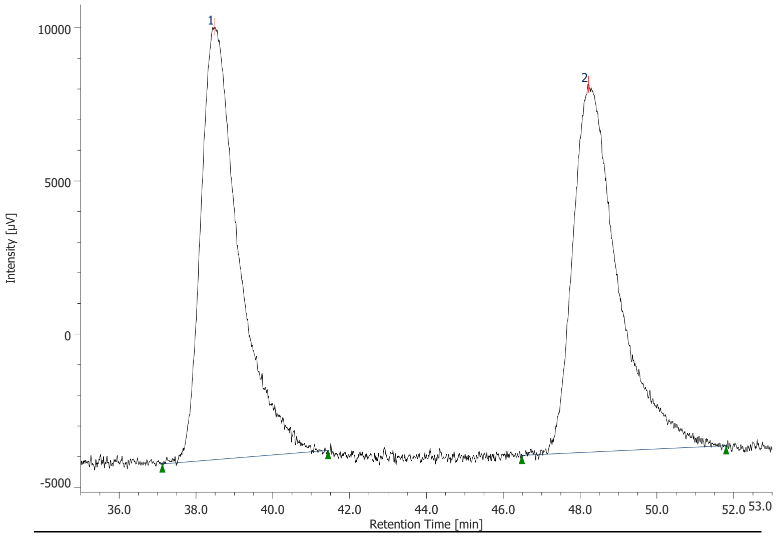

Peak No.	Retention Time (min)	Area (%)
1	38.492	49.909
2	48.217	50.091

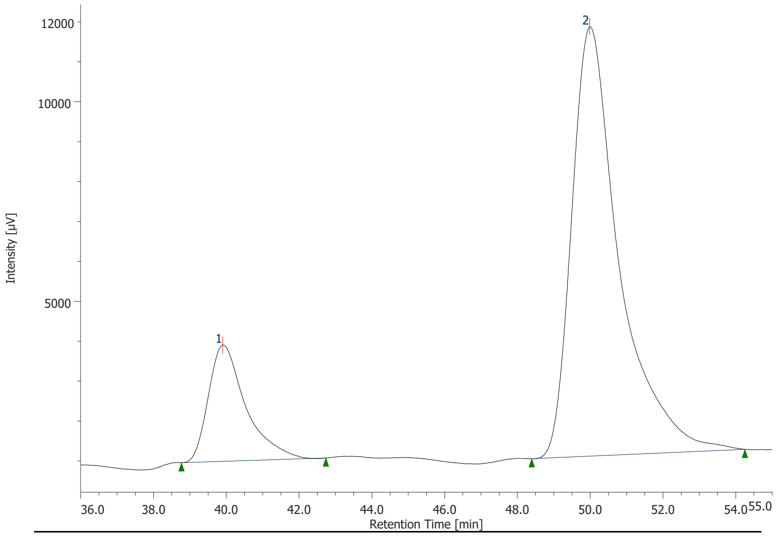

Peak No.	Retention Time (min)	Area (%)
1	39.900	17.536
2	49.983	82.464

(–)-{(1*S*,5*R*,7a*R*)-5-Fluoro-4-(4-methoxyphenyl)-1,7a-dimethyl-2-(4-methylphenyl)-2,3,5,6,7,7a-hexahydro-1*H*-isoindol-5-yl}(pyrrolidin-1-yl)methanone [(–)-3ea (major diastereomer)]

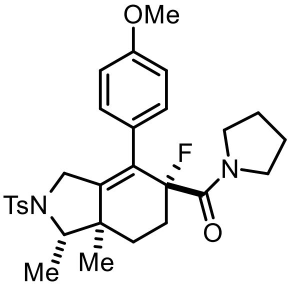

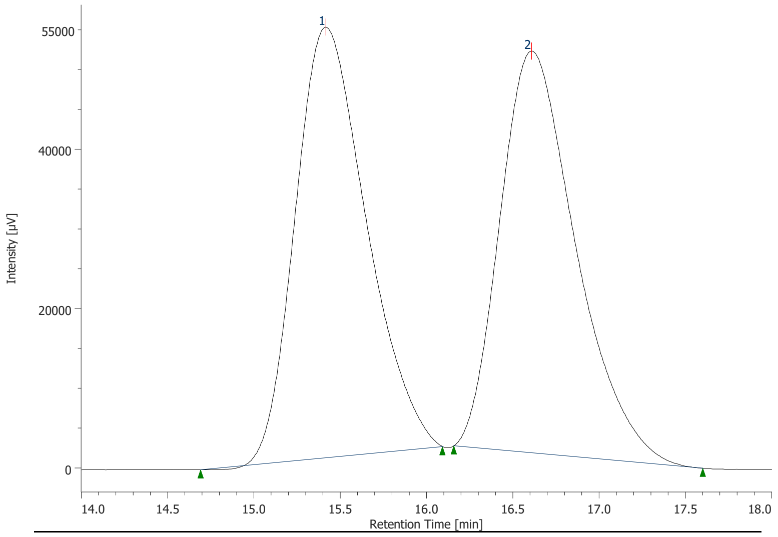

Peak No.	Retention Time (min)	Area (%)
1	15.417	50.114
2	16.608	49.886

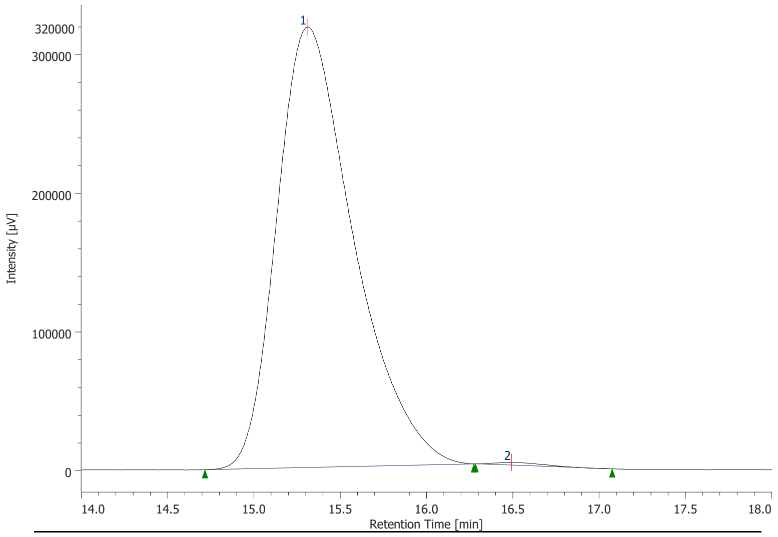

Peak No.	Retention Time (min)	Area (%)
1	15.308	99.618
2	16.492	0.382

(+)-(*E*)-2-Fluoro-4-{(2*R*,3*R*)-4-(*Z*)-4-methoxybenzylidene-2,3-dimethyl-1-(4-methylphenyl) pyrrolidin-3-yl}-1-(pyrrolidin-1-yl)but-2-en-1-one [(+)-4ea (major diastereomer)]

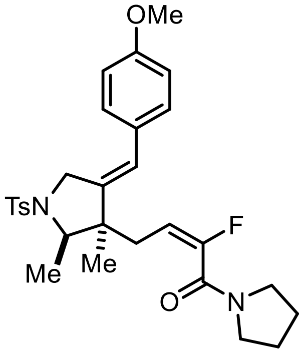

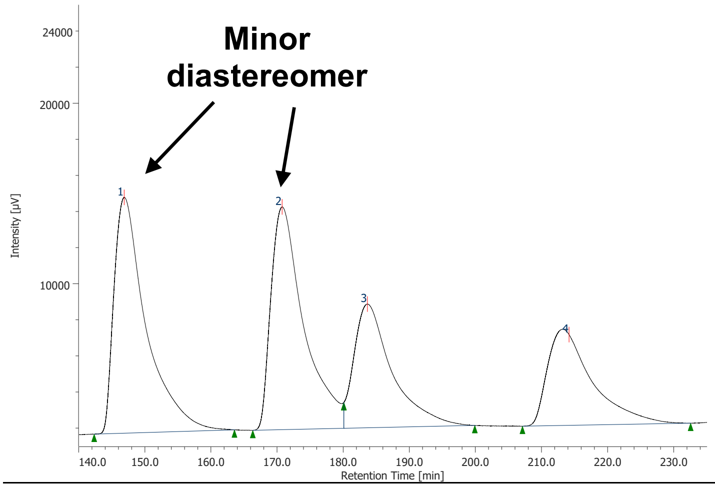

Peak No.	Retention Time (min)	Area (%)
1	146.875	31.662
2	170.767	30.729
**3**	**183.667**	**19.672**
**4**	**214.150**	**17.937**

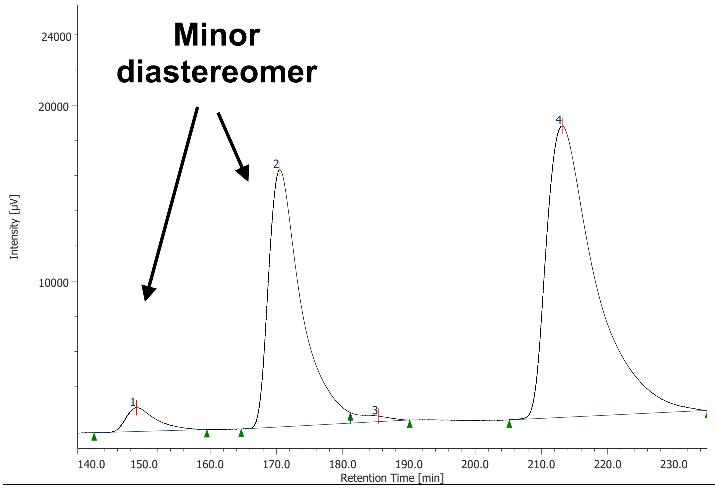

Peak No.	Retention Time (min)	Area (%)
1	148.875	3.041
2	170.517	35.756
**3**	**185.417**	**1.035**
**4**	**213.133**	**60.168**

(–)-{(3*R*,5*R*,7a*R*)-4-(4-Bromophenyl)-5-fluoro-3-methyl-2-(4-methylphenyl)-2,3,5,6,7,7a-hexahydro-1*H*-isoindol-5-yl}(pyrrolidin-1-yl)methanone [(–)-3fa (major diastereomer)]

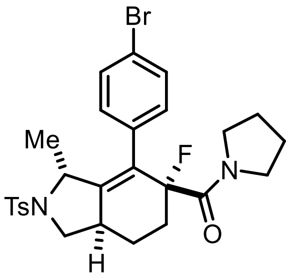

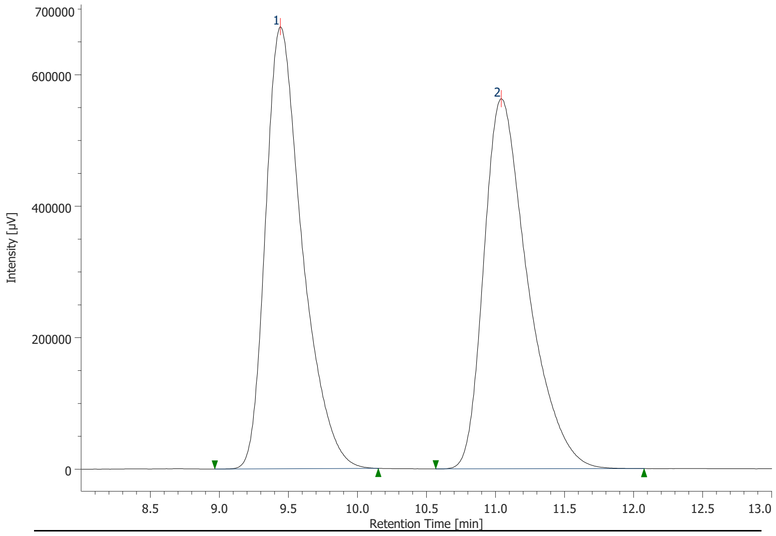

Peak No.	Retention Time (min)	Area (%)
1	9.442	49.884
2	11.042	50.116

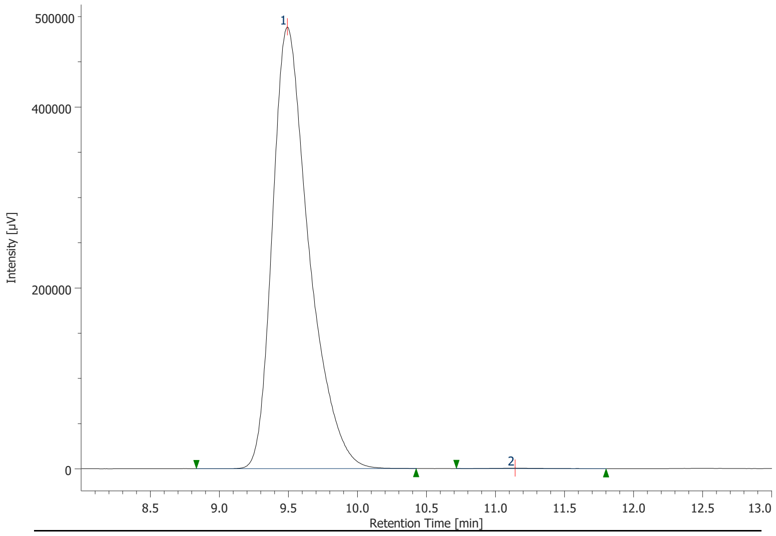

Peak No.	Retention Time (min)	Area (%)
1	9.492	99.884
2	11.142	0.116

(+)-(*E*)-4-[(3*R*,5*S*)-4-{(*Z*)-4-Bromobenzylidene}-3,5-dimethyl-1-(4-methylphenyl)pyrrolidin-3-yl]-2-fluoro-1-(pyrrolidin-1-yl)but-2-en-1-one [(+)-4fa (major diastereomer)]

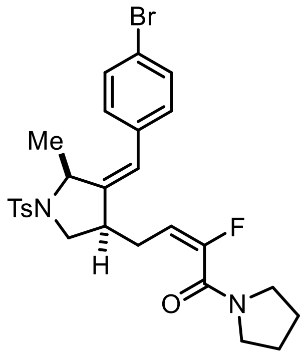

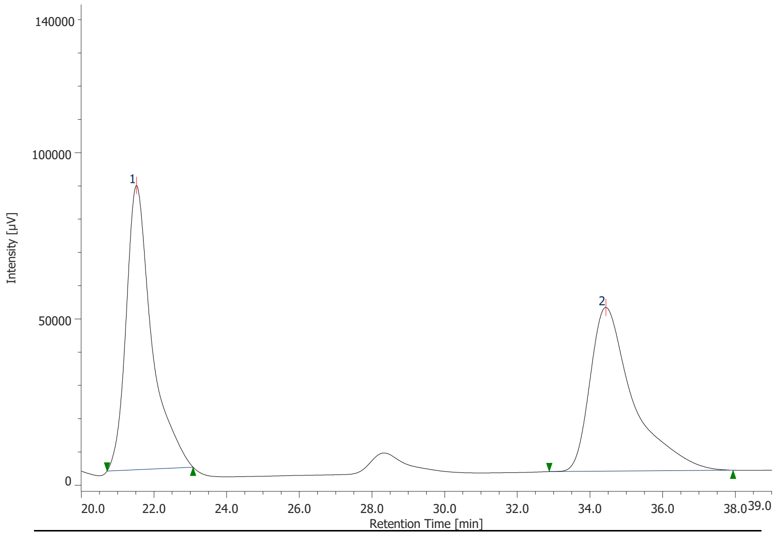

Peak No.	Retention Time (min)	Area (%)
1	21.517	50.864
2	34.433	49.136

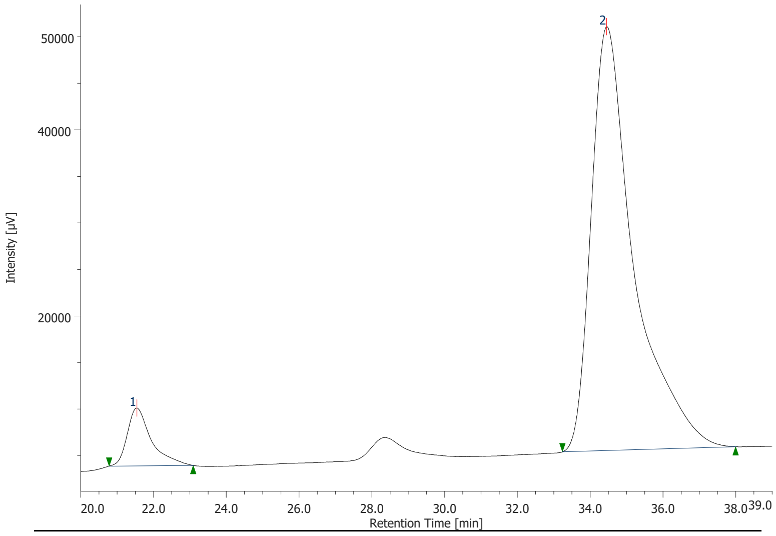

Peak No.	Retention Time (min)	Area (%)
1	21.542	7.153
2	34.450	92.847

(–)-{(3*R*,5*R*,7a*R*)-5-Fluoro-3,4-diphenyl-2-(4-methylphenyl)-2,3,5,6,7,7a-hexahydro-1*H*-isoindol-5-yl}(pyrrolidin-1-yl)methanone [(–)-3ga]

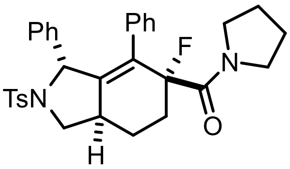

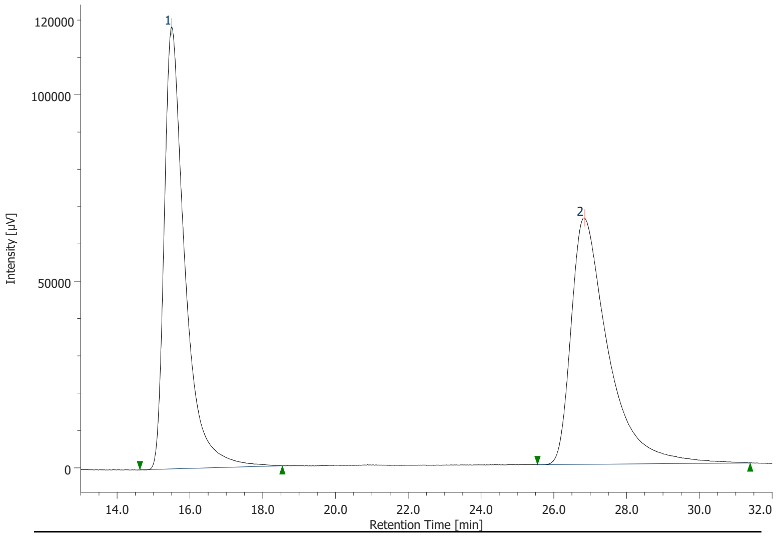

Peak No.	Retention Time (min)	Area (%)
1	15.500	50.059
2	26.833	49.941

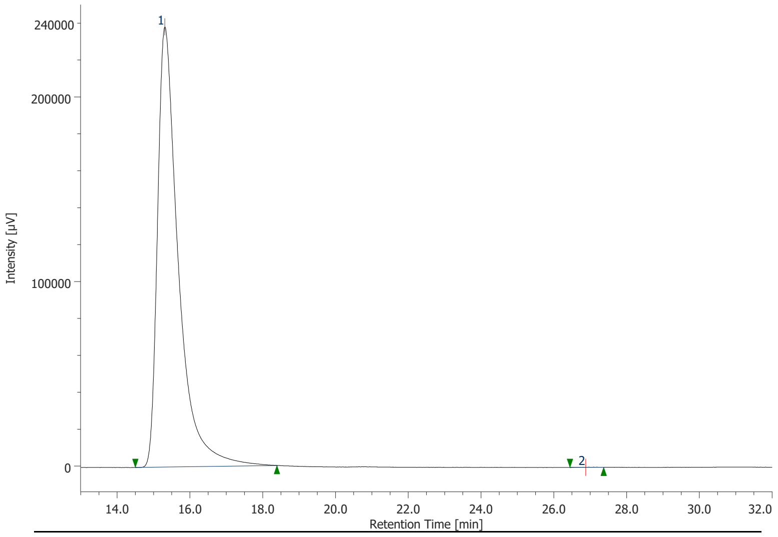

Peak No.	Retention Time (min)	Area (%)
1	15.308	99.965
2	26.875	0.035

(+)-(*E*)-4-{(3*R*,5*S*)-4-(*Z*)-Benzylidene-3-methyl-1-(4-methylphenyl)-5-phenylpyrrolidin-3-yl}-2-fluoro-1-(pyrrolidin-1-yl)but-2-en-1-one [(+)-4ga (major diastereomer)]

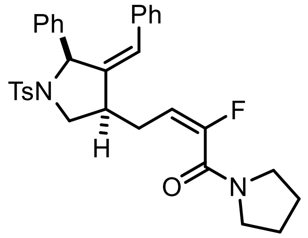

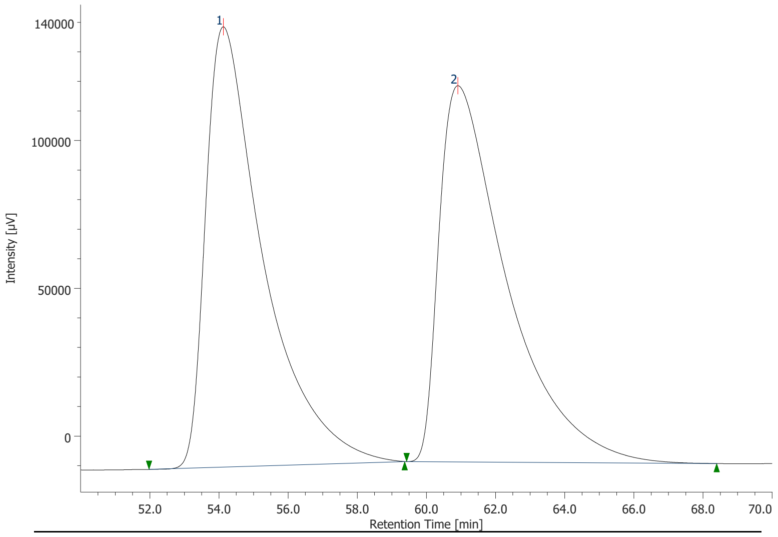

Peak No.	Retention Time (min)	Area (%)
1	54.125	49.852
2	60.908	50.148

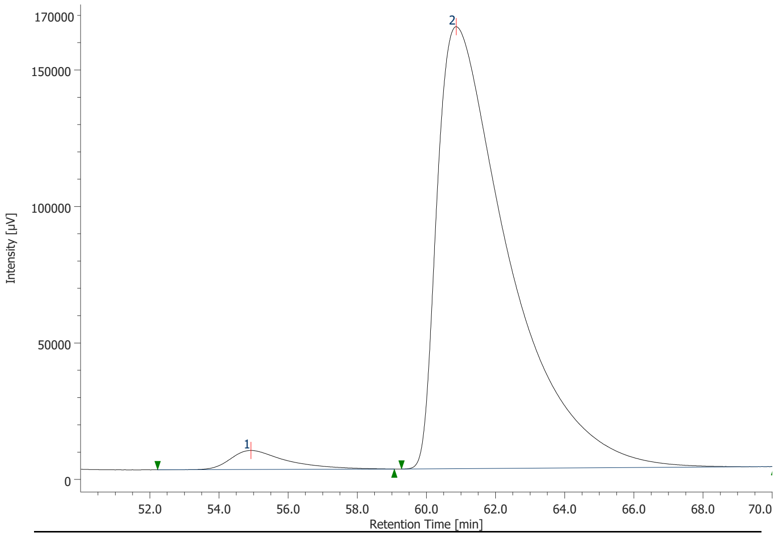

Peak No.	Retention Time (min)	Area (%)
1	54.925	3.271
2	60.858	96.729

(–)-{(1*S*,5*R*,7a*R*)-5-Fluoro-1,7a-dimethyl-2-(4-methylphenyl)-4-phenyl-2,3,5,6,7,7a-hexahydro-1*H*-isoindol-5-yl}(morpholino)methanone [(–)-3db (major diastereomer)]

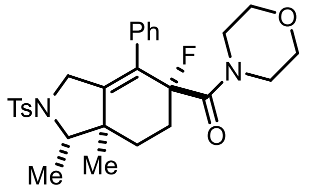

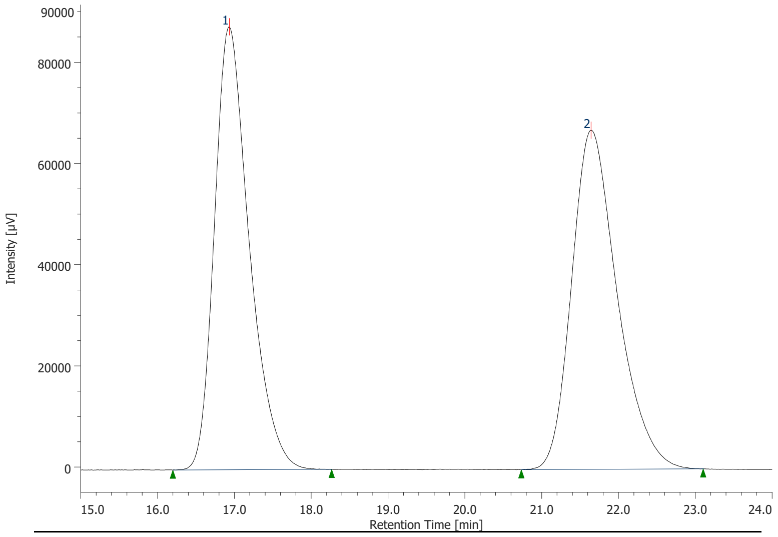

Peak No.	Retention Time (min)	Area (%)
1	16.933	49.929
2	21.642	50.071

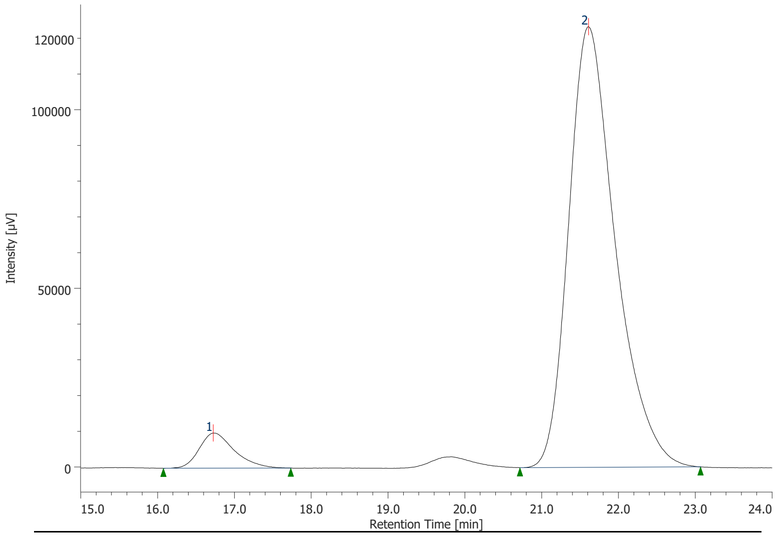

Peak No.	Retention Time (min)	Area (%)
1	16.725	5.647
2	21.608	94.353

(-)-(*E*)-4-{(2*R*,3*R*)-4-(*Z*)-Benzylidene-2,3-dimethyl-1-(4-methylphenyl)pyrrolidin-3-yl}-2-fluoro-1-morpholinobut-2-en-1-one [(–)-4db (major diastereomer)]

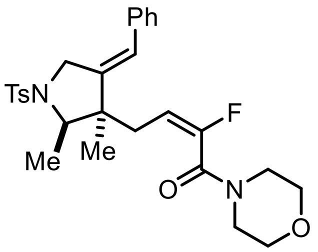

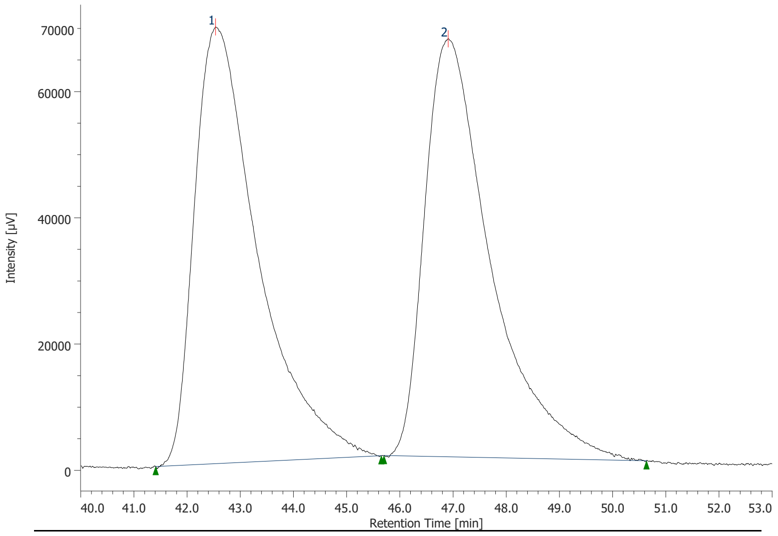

Peak No.	Retention Time (min)	Area (%)
1	42.533	50.038
2	46.908	49.962

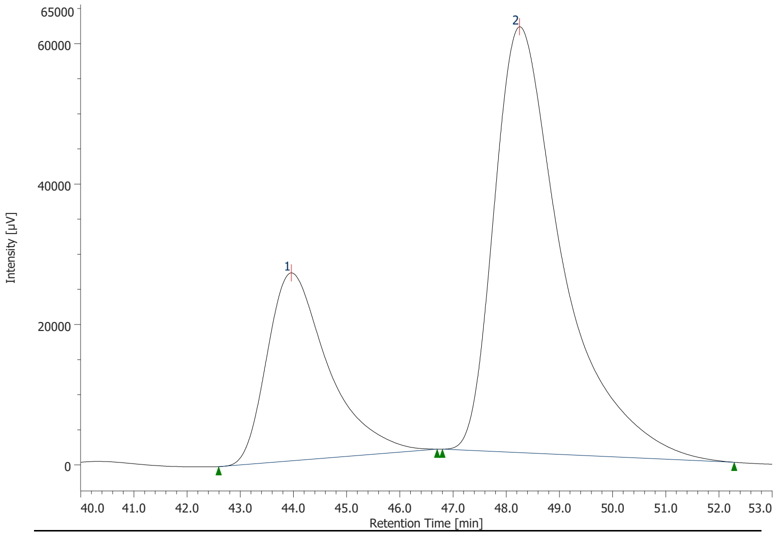

Peak No.	Retention Time (min)	Area (%)
1	43.958	28.298
2	48.250	71.702

(–)-{(1*S*,5*R*,7a*R*)-5-Fluoro-4-(4-methoxyphenyl)-1,7a-dimethyl-2-(4-methylphenyl)-2,3,5,6,7,7a-hexahydro-1*H*-isoindol-5-yl}(morpholino)methanone [(–)-3eb (major diastereomer)]

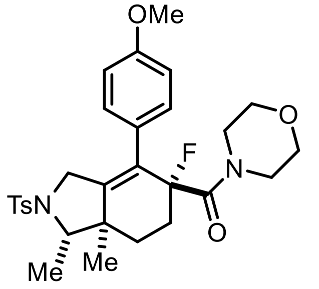

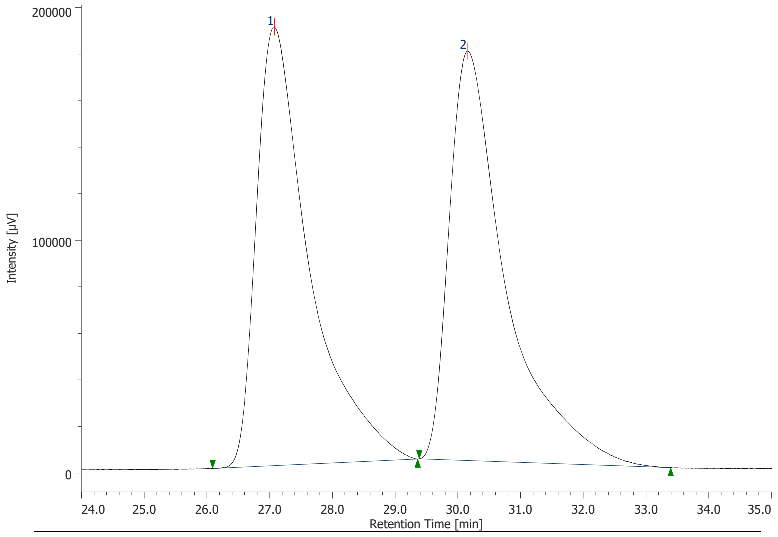

Peak No.	Retention Time (min)	Area (%)
1	27.075	50.063
2	30.150	49.937

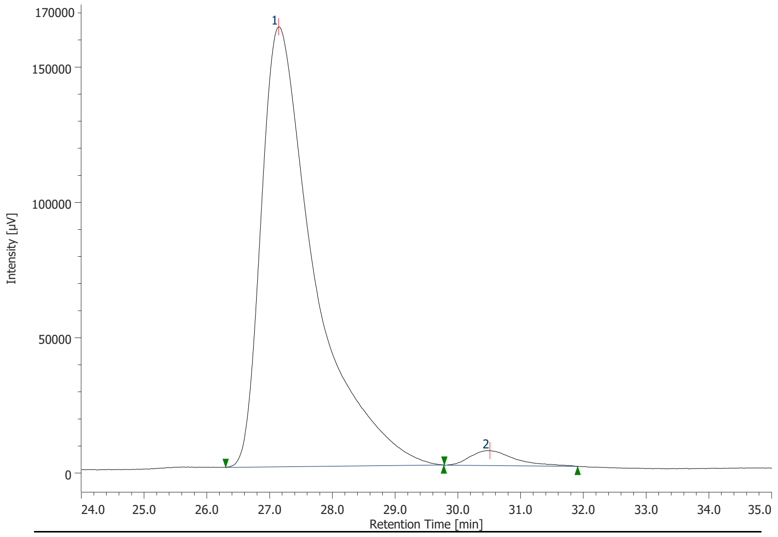

Peak No.	Retention Time (min)	Area (%)
1	27.142	97.241
2	30.508	2.759

(–)-(*E*)-2-Fluoro-4-[(2*R*,3*R*)-4-{(*Z*)-4-methoxybenzylidene}-2,3-dimethyl-1-(4-methylphenyl)pyrrolidin-3-yl]-1-morpholinobut-2-en-1-one [(–)-4eb (major diastereomer)]

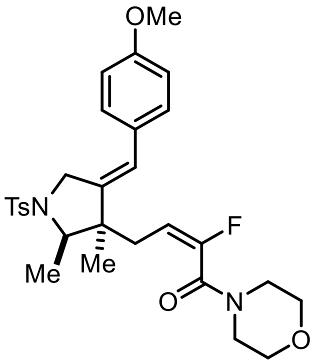

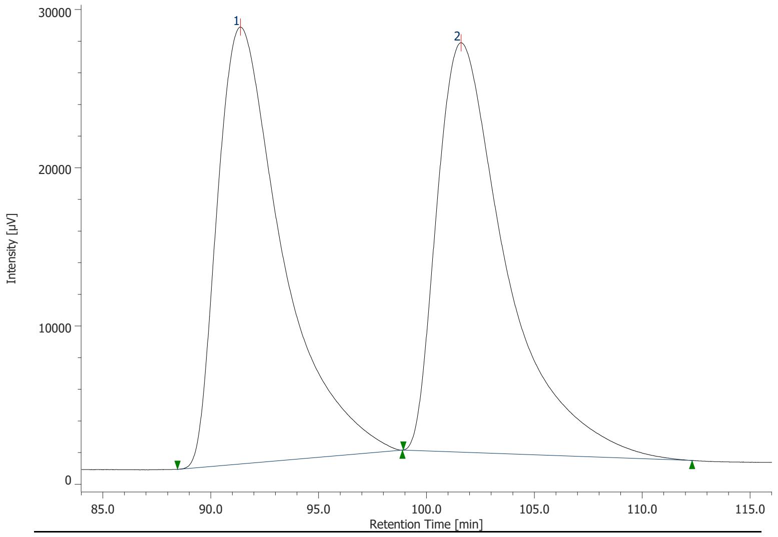

Peak No.	Retention Time (min)	Area (%)
1	91.375	50.030
2	101.600	49.970

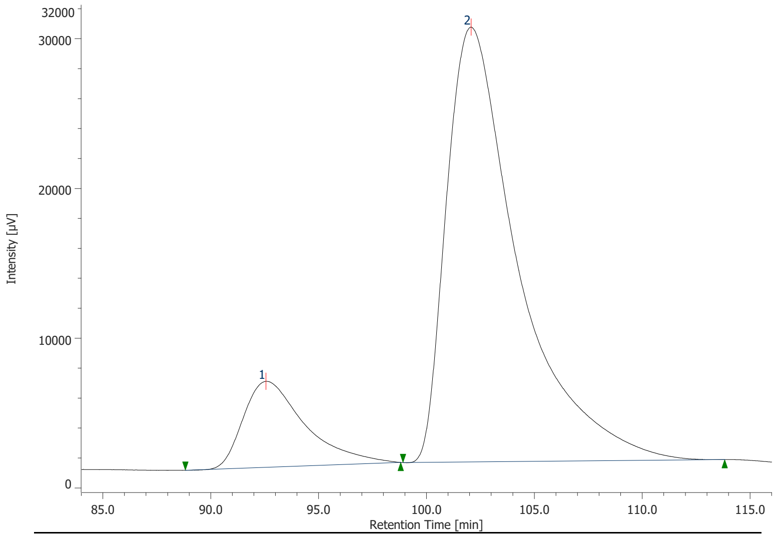

Peak No.	Retention Time (min)	Area (%)
1	92.558	14.543
2	102.067	85.457

(+)-(3*R*,5*R*,7a*R*)-5-Fluoro-2-(4-methylphenyl)-*N*,*N*,3,4-tetraphenyl-2,3,5,6,7,7a-hexahydro-1*H*-isoindole-5-carboxamide [(+)-3gc]

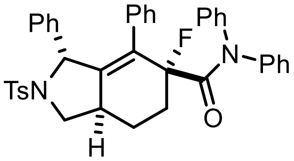

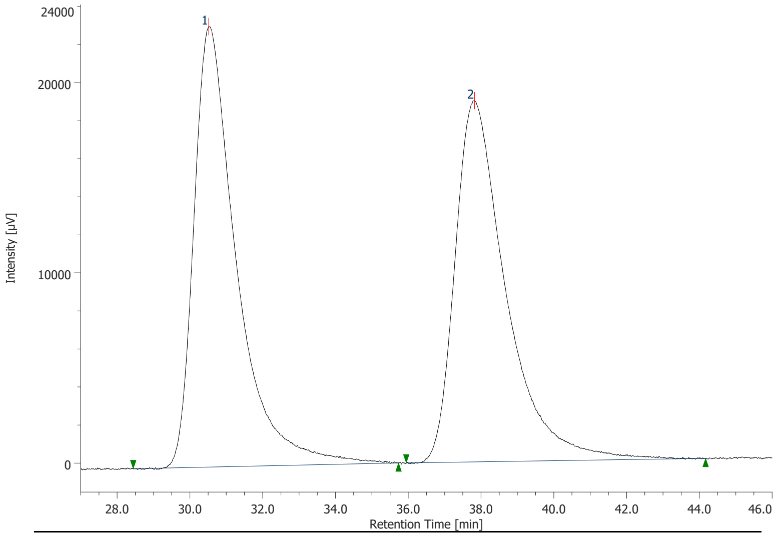

Peak No.	Retention Time (min)	Area (%)
1	30.517	50.486
2	37.817	49.514

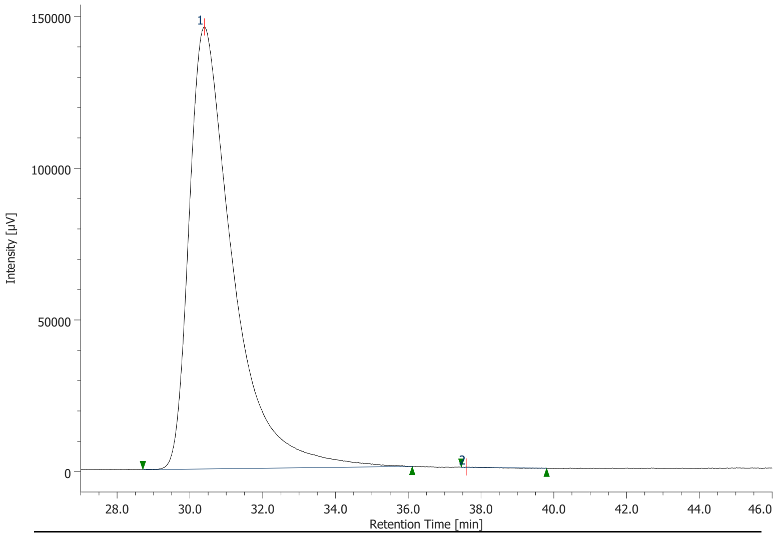

Peak No.	Retention Time (min)	Area (%)
1	30.392	99.990
2	37.592	0.010

(+)-(*E*)-4-{(3*R*,5*S*)-4-(*Z*)-Benzylidene-3-methyl-1-(4-methylphenyl)-5-phenylpyrrolidin-3-yl}-2-fluoro-*N*,*N*-diphenylbut-2-enamide [(+)-4gc (major diastereomer)]

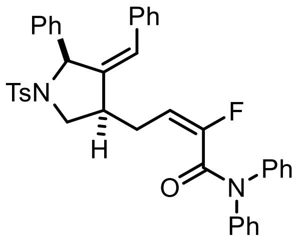

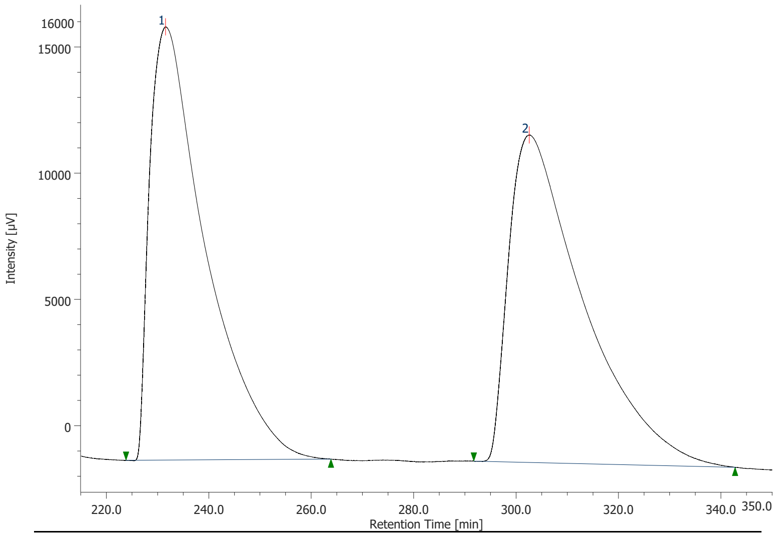

Peak No.	Retention Time (min)	Area (%)
1	230.875	49.797
2	301.567	50.203

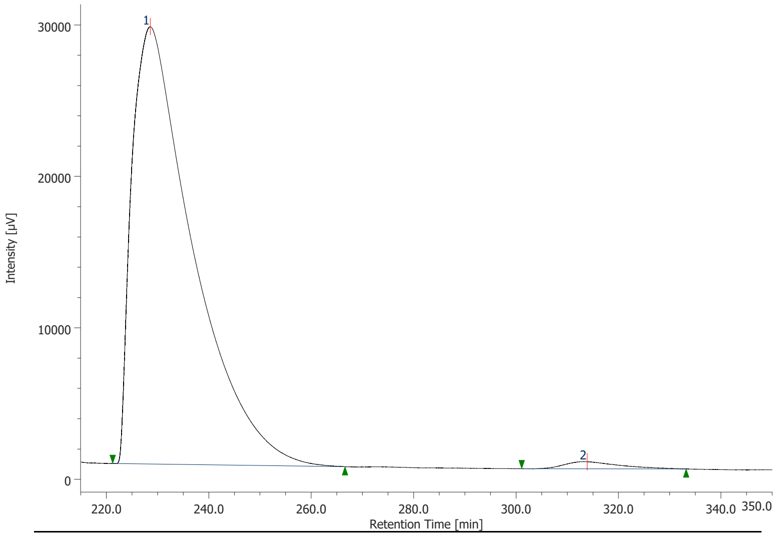

Peak No.	Retention Time (min)	Area (%)
1	229.458	99.983
2	300.817	0.017

(–)-(3*S*,3a*R*,6*R*)-6-Fluoro-3,3a-dimethyl-7-phenyl-6-(pyrrolidine-1-carbonyl)-3a,4,5,6-tetrahydroisobenzofuran-1(3*H*)-one [(–)-3ha]

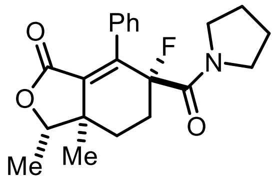

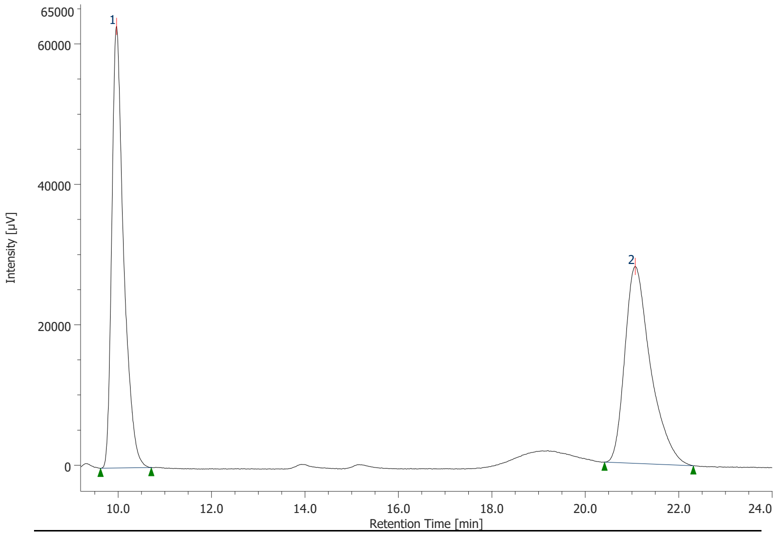

Peak No.	Retention Time (min)	Area (%)
1	9.967	50.781
2	21.067	49.219

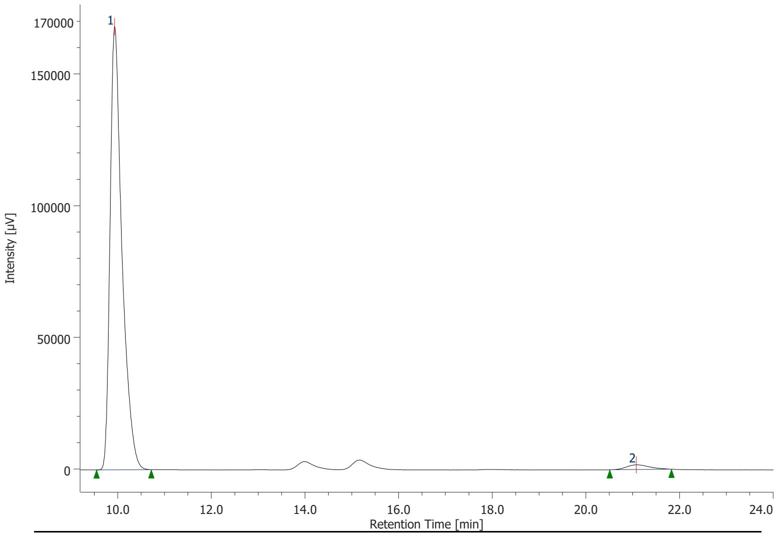

Peak No.	Retention Time (min)	Area (%)
1	9.933	97.987
2	21.075	2.013

(–)-(4*S*,5*R*)-3-(*Z*)-Benzylidene-4-{(*E*)-3-fluoro-4-oxo-4-(pyrrolidin-1-yl)but-2-en-1-yl}-4,5-dimethyldihydrofuran-2(3*H*)-one [(–)-4ha (major diastereomer)]

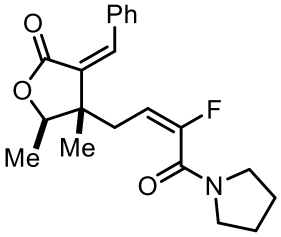

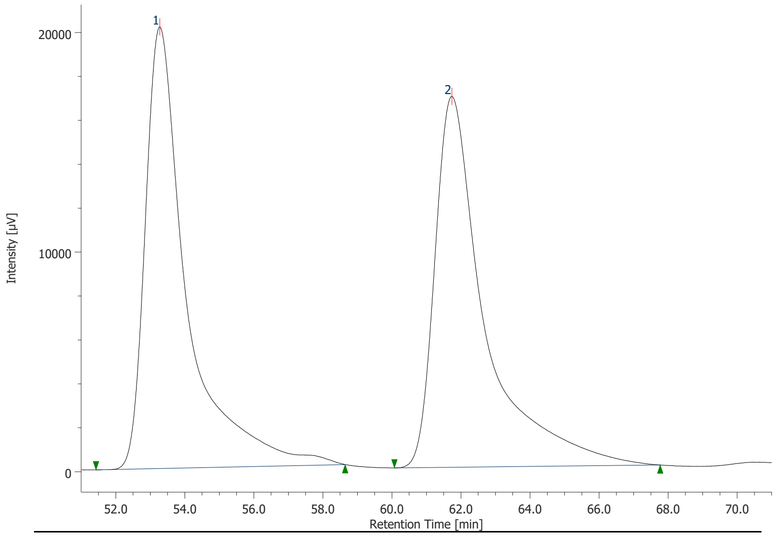

Peak No.	Retention Time (min)	Area (%)
1	53.275	50.651
2	61.733	49.349

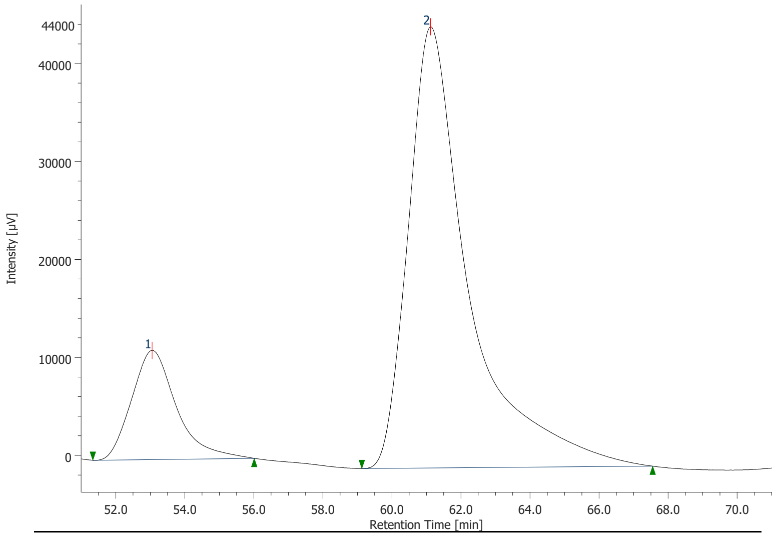

Peak No.	Retention Time (min)	Area (%)
1	53.050	15.472
2	61.117	84.528

(–)-(3*S*,3a*R*,6*R*)-7-(4-Bromophenyl)-6-fluoro-3,3a-dimethyl-6-(pyrrolidine-1-carbonyl)-3a,4,5,6-tetrahydroisobenzofuran-1(3*H*)-one [(–)-3ia]

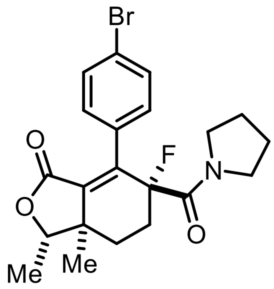

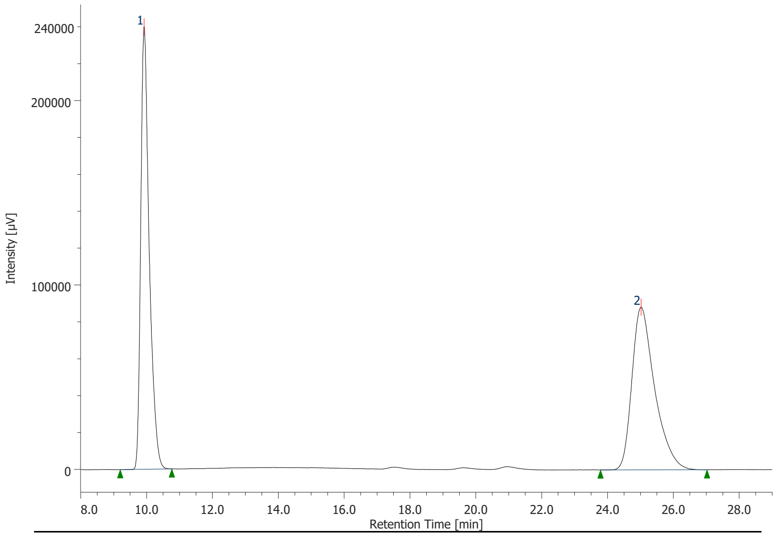

Peak No.	Retention Time (min)	Area (%)
1	9.925	50.355
2	25.017	49.645

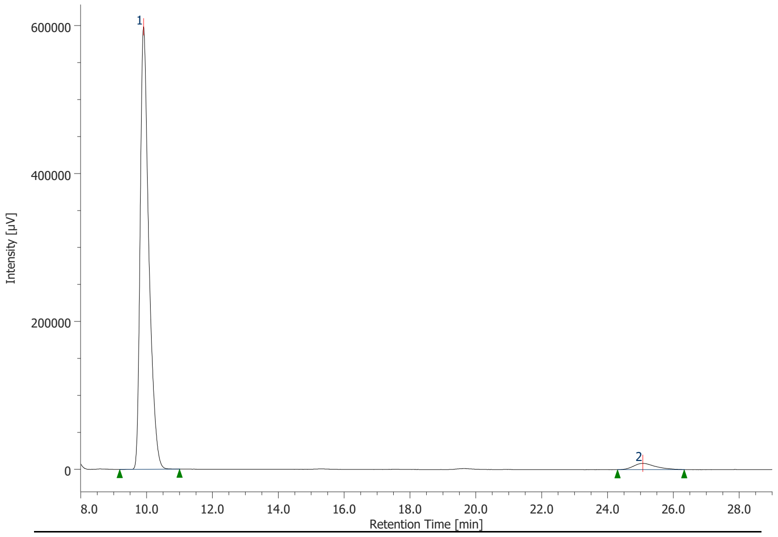

Peak No.	Retention Time (min)	Area (%)
1	9.908	96.546
2	25.067	3.454

(–)-(4*S*,5*R*)-3-(*Z*)-4-Bromobenzylidene-4-{(*E*)-3-fluoro-4-oxo-4-(pyrrolidin-1-yl)but-2-en-1-yl}-4,5-dimethyldihydrofuran-2(3*H*)-one [(–)-4ia (major diastereomer)]

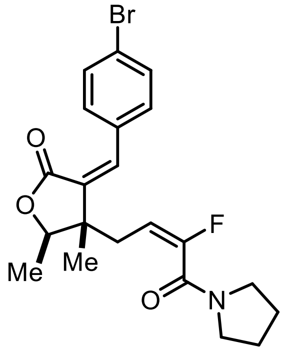

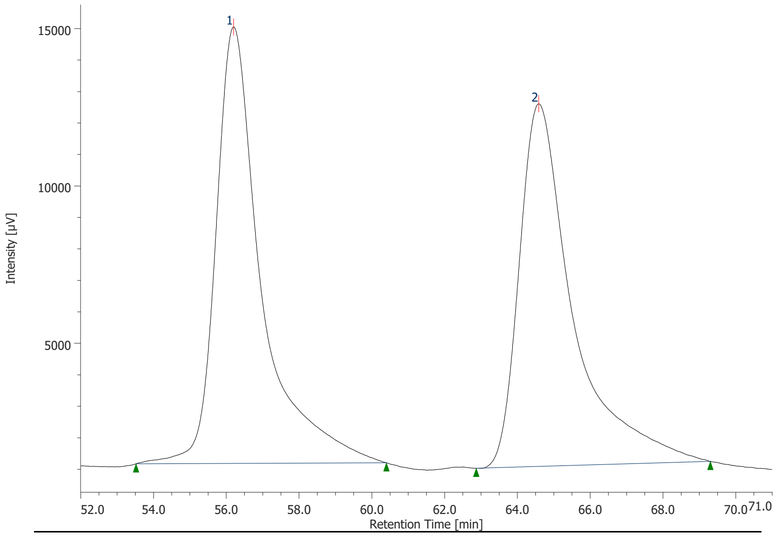

Peak No.	Retention Time (min)	Area (%)
1	56.200	50.494
2	64.583	49.506

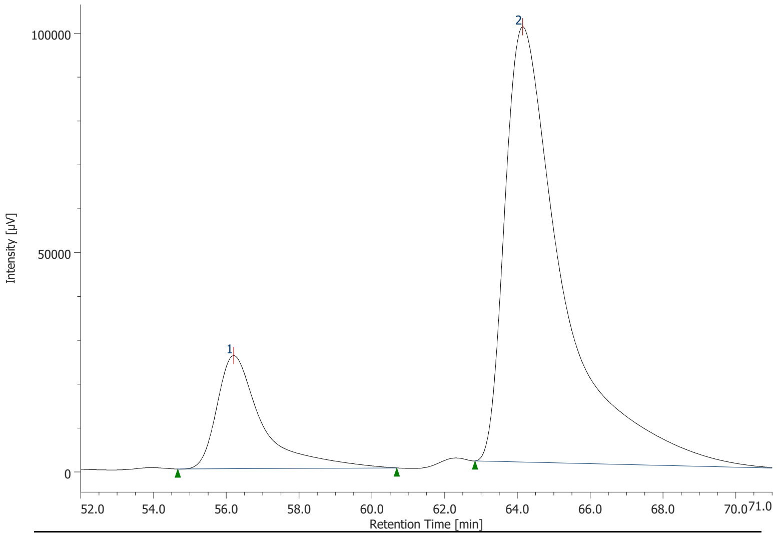

Peak No.	Retention Time (min)	Area (%)
1	56.200	16.819
2	64.142	83.181

(–)-(3*S*,3a*R*,6*R*)-6-Fluoro-3a-methyl-3,7-diphenyl-6-(pyrrolidine-1-carbonyl)-3a,4,5,6-tetrahydroisobenzofuran-1(3*H*)-one [(–)-3ja]

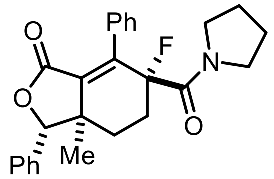

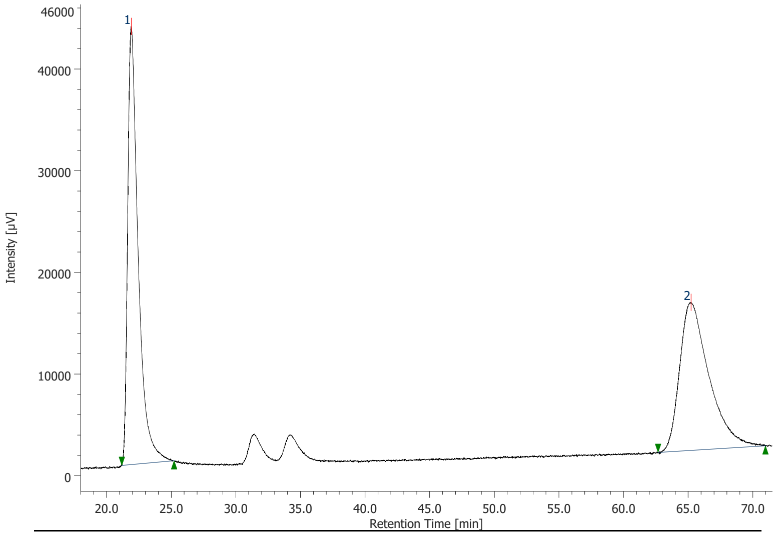

Peak No.	Retention Time (min)	Area (%)
1	21.908	51.472
2	65.206	48.528

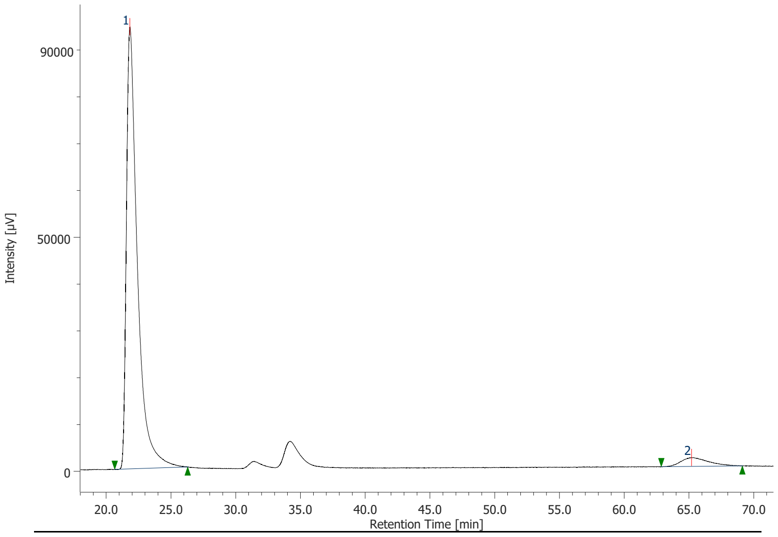

Peak No.	Retention Time (min)	Area (%)
1	21.833	95.216
2	65.183	4.784

(–)-(4*S*,5*R*)-3-(*Z*)-Benzylidene-4-{(*E*)-3-fluoro-4-oxo-4-(pyrrolidin-1-yl)but-2-en-1-yl}-4-methyl-5-phenyldihydrofuran-2(3*H*)-one [(–)-4ja]

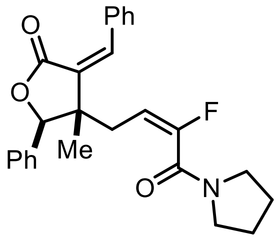

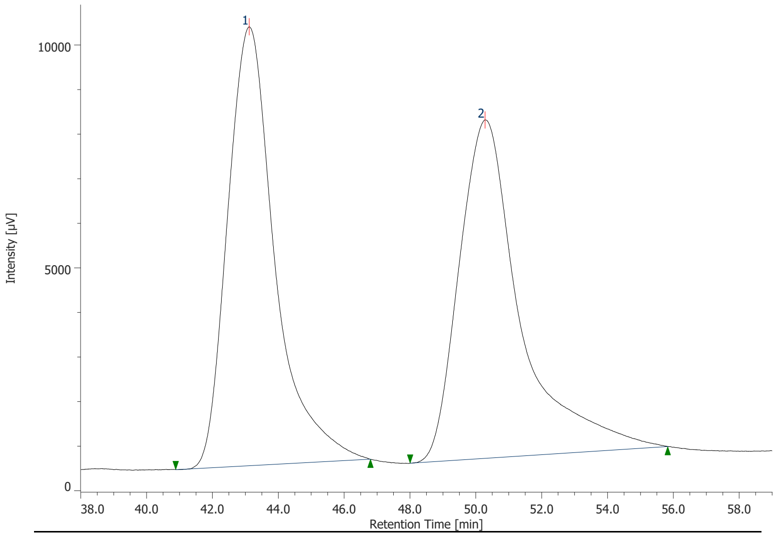

Peak No.	Retention Time (min)	Area (%)
1	43.117	50.055
2	50.275	49.945

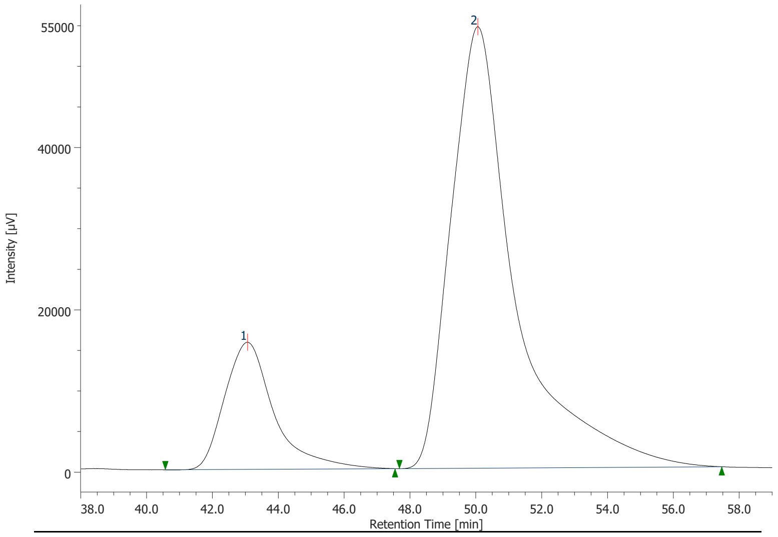

Peak No.	Retention Time (min)	Area (%)
1	43.067	18.075
2	50.058	81.925

(–)-(8*R*,10a*R*,10b*S*)-8-Fluoro-10a-methyl-7-phenyl-8-(pyrrolidine-1-carbonyl)-8,9,10,10a,10b,11-hexahydro-6*H*-isoindolo[2,1-a]indol-6-one [(–)-3ka]

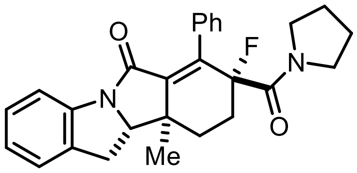

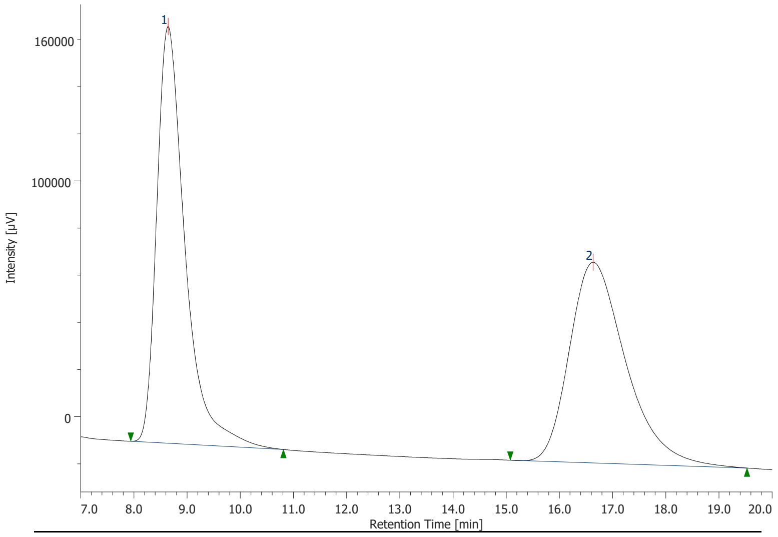

Peak No.	Retention Time (min)	Area (%)
1	8.642	51.055
2	16.633	48.945

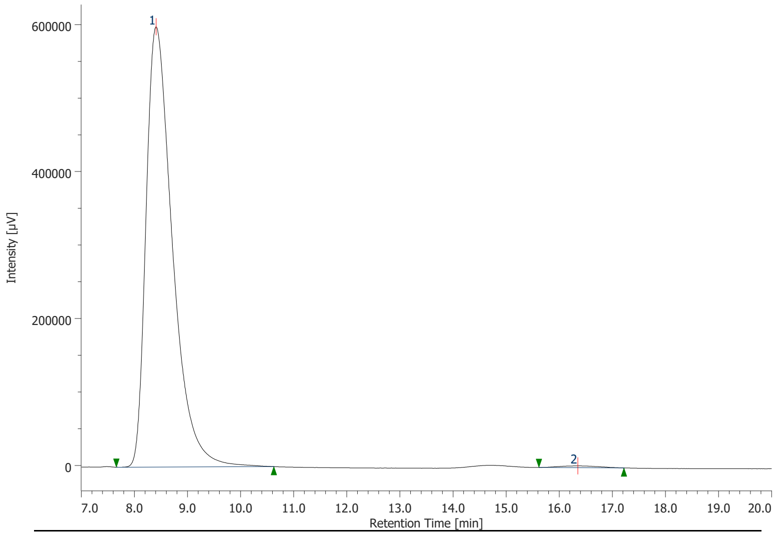

Peak No.	Retention Time (min)	Area (%)
1	8.408	99.389
2	16.350	0.611

(–)-(1*S*,9a*R*)-2-(*Z*)-Benzylidene-1-{(*E*)-3-fluoro-4-oxo-4-(pyrrolidin-1-yl)but-2-en-1-yl}-1-methyl-1,2,9,9a-tetrahydro-3*H*-pyrrolo[1,2-a]indol-3-one [(–)-4ka]

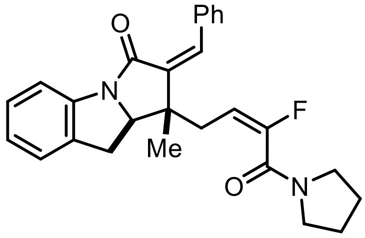

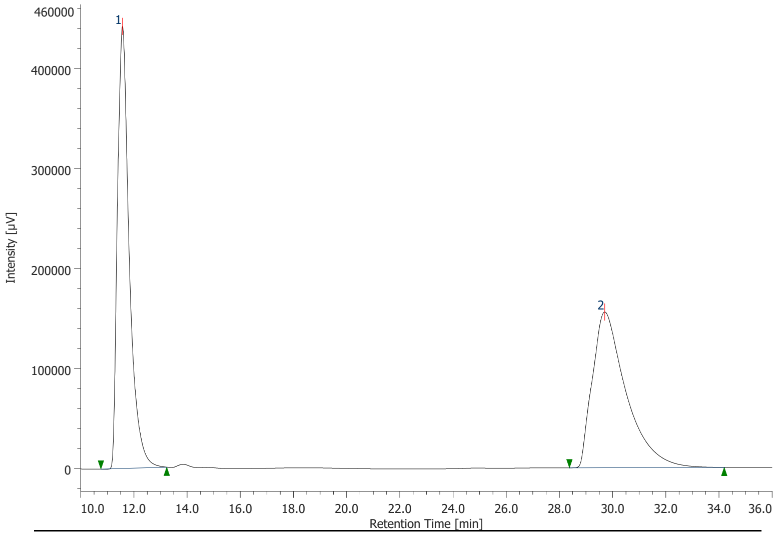

Peak No.	Retention Time (min)	Area (%)
1	11.567	49.950
2	29.700	50.050

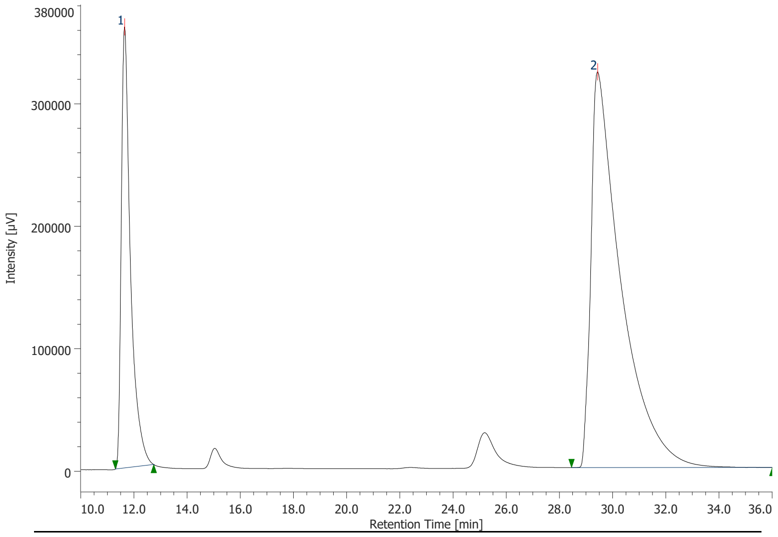

Peak No.	Retention Time (min)	Area (%)
1	11.650	25.585
2	29.433	74.415

(–)-{(1*S*,5*R*,7a*R*)-5-Fluoro-1,7a-dimethyl-4-phenyl-1,3,5,6,7,7a-hexahydroisobenzofuran-5-yl}(pyrrolidin-1-yl)methanone [(–)-3la (major diastereomer)]

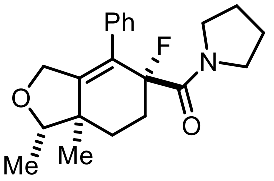

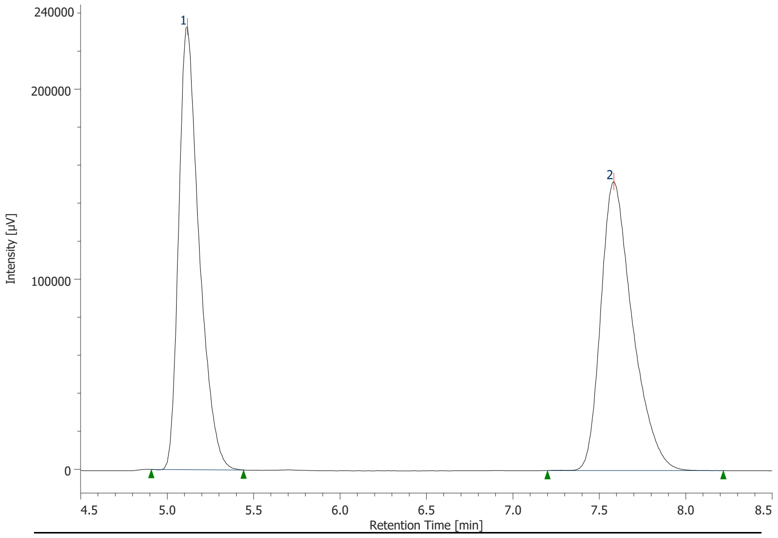

Peak No.	Retention Time (min)	Area (%)
1	5.117	49.720
2	7.583	50.280

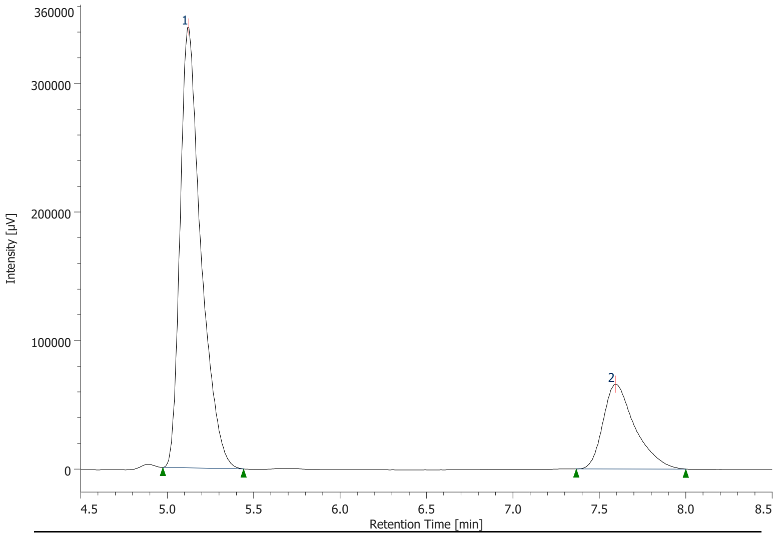

Peak No.	Retention Time (min)	Area (%)
1	5.125	77.322
2	7.592	22.678

(+)-(*E*)-4-{(2*R*,3*S*)-4-(*Z*)-Benzylidene-2,3-dimethyltetrahydrofuran-3-yl}-2-fluoro-1-(pyrrolidin-1-yl)but-2-en-1-one [(+)-4la (major diastereomer)]

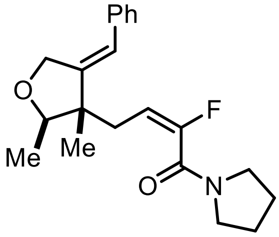

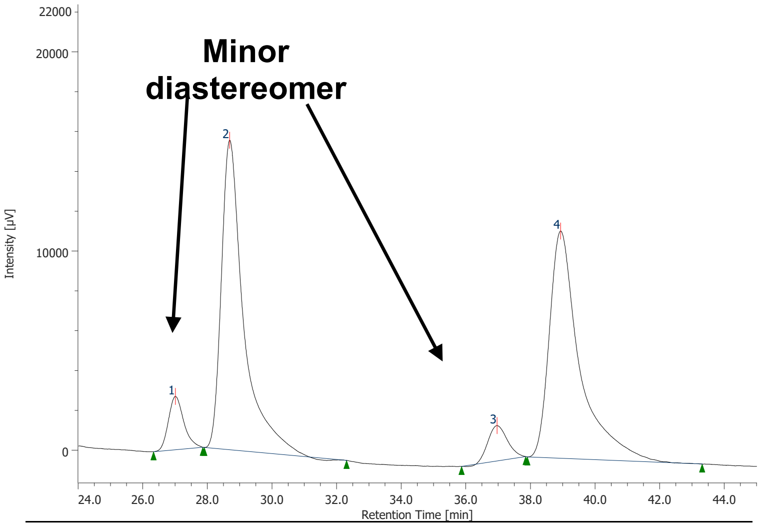

Peak No.	Retention Time (min)	Area (%)
1	27.088	5.341
**2**	**28.692**	**45.234**
3	36.967	4.502
**4**	**38.933**	**44.924**

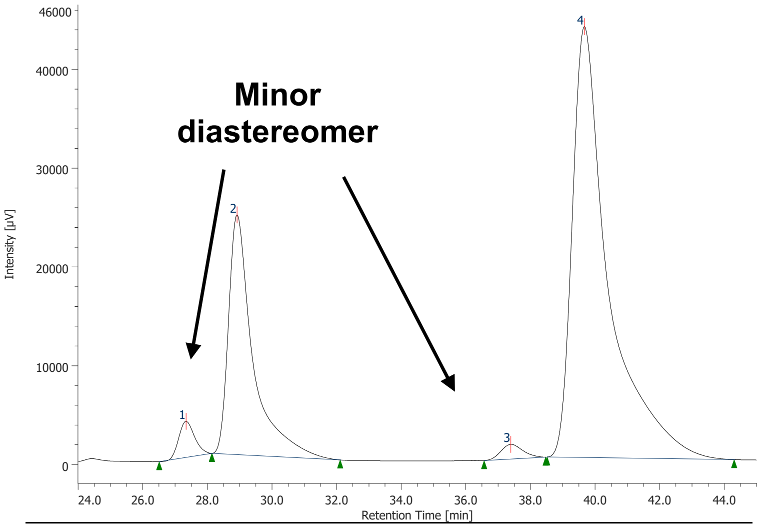

Peak No.	Retention Time (min)	Area (%)
1	27.342	2.554
**2**	**28.917**	**26.279**
3	37.392	1.436
**4**	**39.667**	**69.732**

(+)-(*S*)-3-Phenylbut-3-en-2-yl 3-phenylpropynoate [(+)-1m]

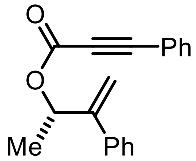

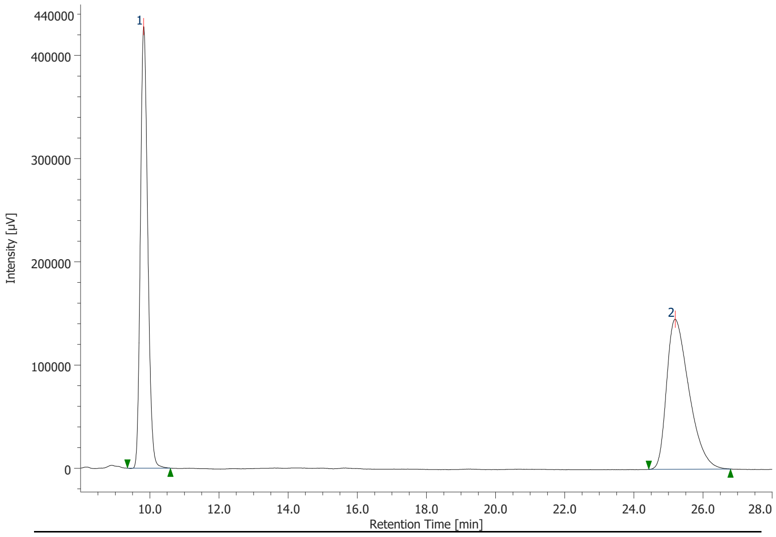

Peak No.	Retention Time (min)	Area (%)
1	9.817	49.075
2	25.192	50.925

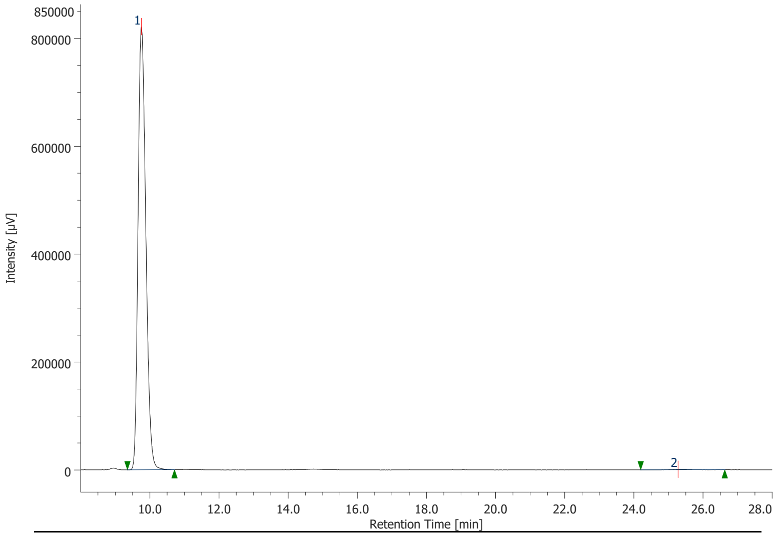

Peak No.	Retention Time (min)	Area (%)
1	9.750	99.716
2	25.275	0.284

(–)-(3*S*,3a*S*,6*R*)-6-Fluoro-3-methyl-3a,7-diphenyl-6-(pyrrolidine-1-carbonyl)-3a,4,5,6-tetrahydroisobenzofuran-1(3*H*)-one [(–)-3ma]

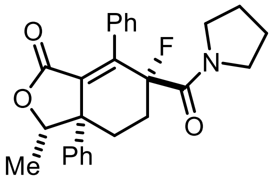

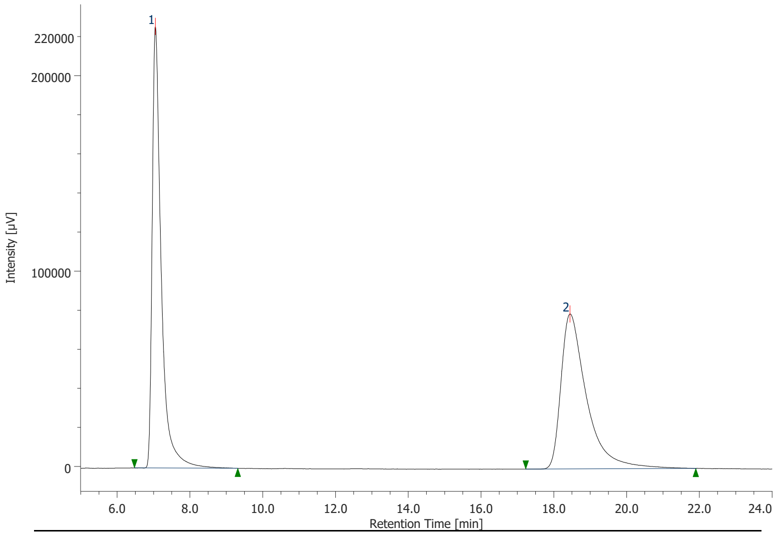

Peak No.	Retention Time (min)	Area (%)
1	7.050	50.067
2	18.442	49.933

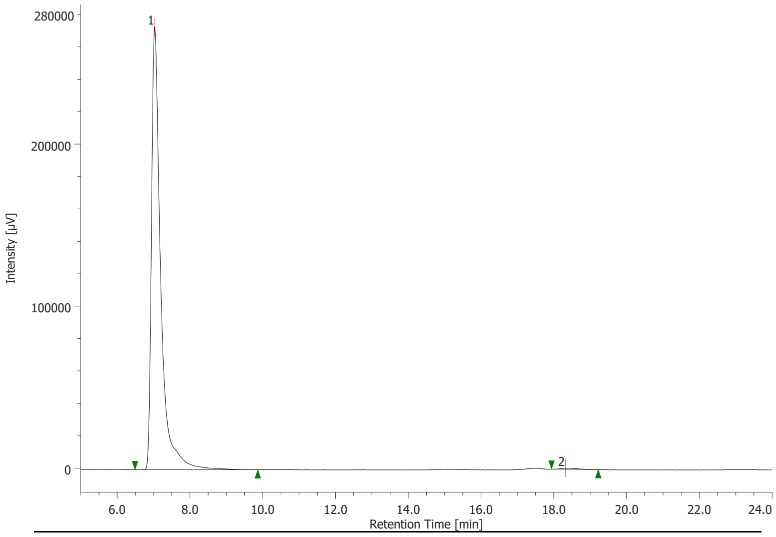

Peak No.	Retention Time (min)	Area (%)
1	7.033	99.573
2	18.317	0.427

(–)-(4*R*,5*R*)-3-(*Z*)-Benzylidene-4-{(*E*)-3-fluoro-4-oxo-4-(pyrrolidin-1-yl)but-2-en-1-yl}-5-methyl-4-phenyldihydrofuran-2(3*H*)-one [(–)-4ma (major diastereomer)]

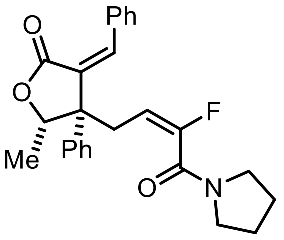

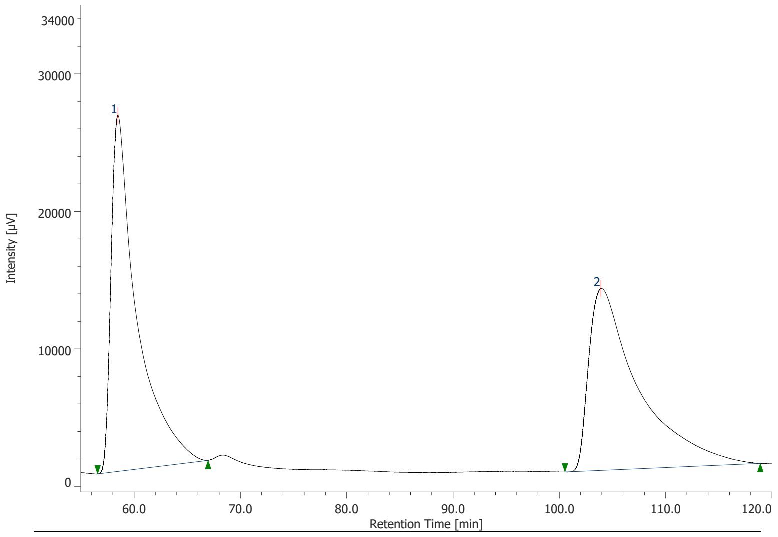

Peak No.	Retention Time (min)	Area (%)
1	58.475	50.281
2	103.908	49.719

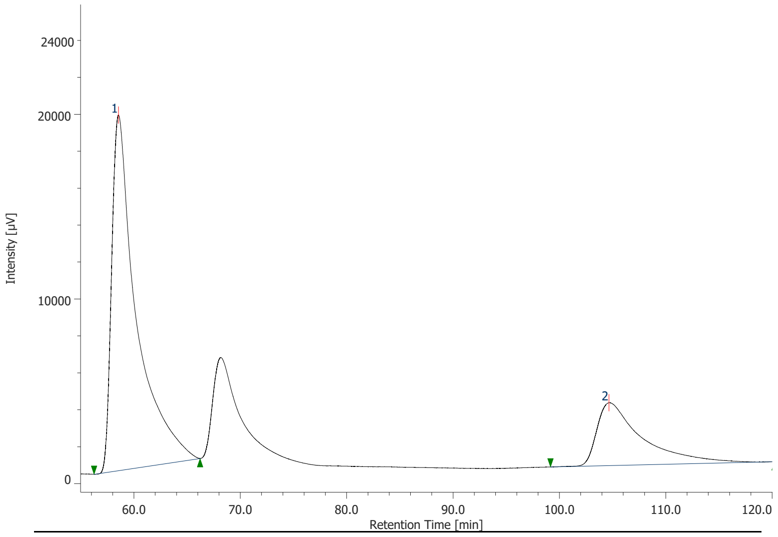

Peak No.	Retention Time (min)	Area (%)
1	58.542	75.540
2	104,642	24.460

7.2. Rh-Catalyzed Enantioselective PKR (type II) of Racemic 1,6-Enynes with Two Different Acrylamide Derivatives

7.2.1. Substituent Effect at α-Position of Acrylamide (Figure 3a)

(–)-Pyrrolidin-1-yl{(5*S*,7a*R*)-1,4,7a-trimethyl-2-(4-methylphenyl)-2,3,5,6,7,7a-hexahydro-1*H*-isoindol-5-yl}methanone [(–)-3ad]

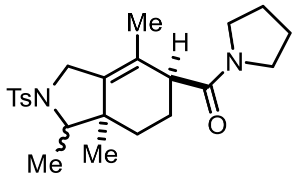

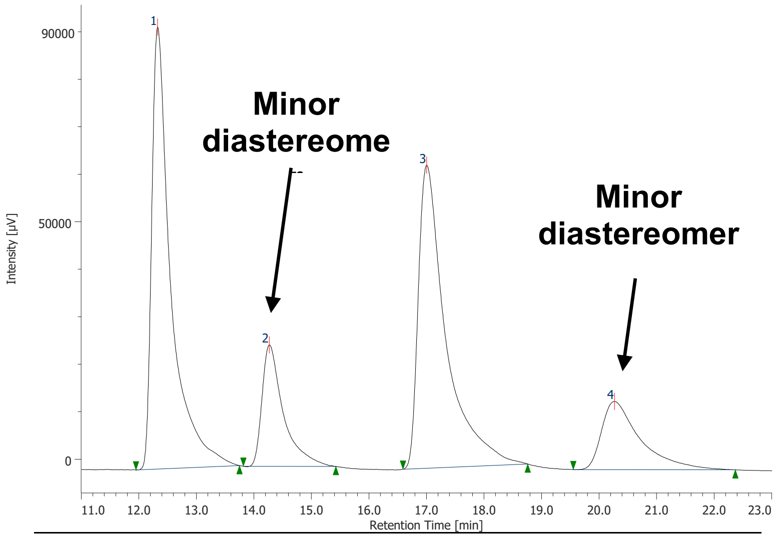

Peak No.	Retention Time (min)	Area (%)
**1**	**12.325**	**38.365**
2	14.267	11.729
**3**	**17.000**	**37.968**
4	20.267	11.939

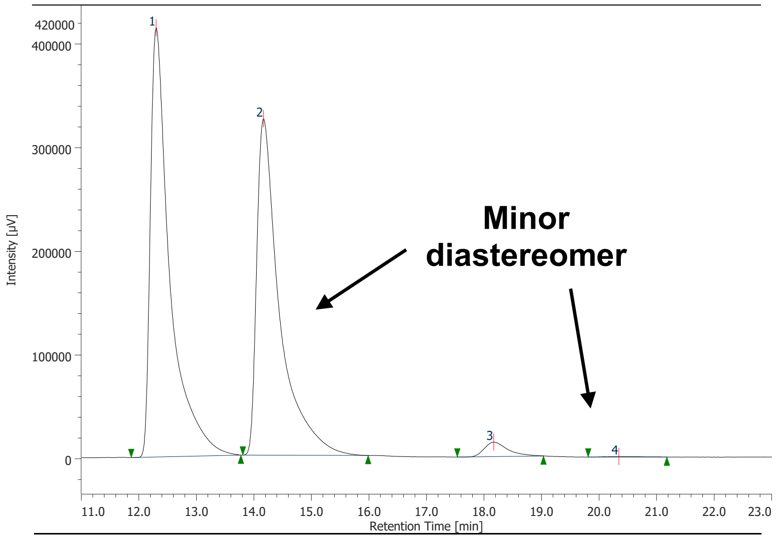

Peak No.	Retention Time (min)	Area (%)
**1**	**12.300**	**50.940**
2	14.167	46.902
**3**	**18.167**	**2.052**
4	20.342	0.106

(+)-(*Z*)-4-{(3*R*,*Z*)-4-Ethylidene-2,3-dimethyl-1-(4-methylphenyl)pyrrolidin-3-yl}-2-methyl-1-(pyrrolidin-1-yl)but-2-en-1-one [(+)-4ae]

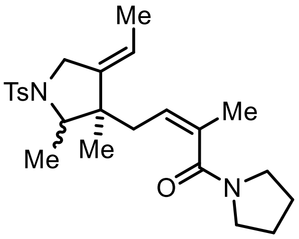

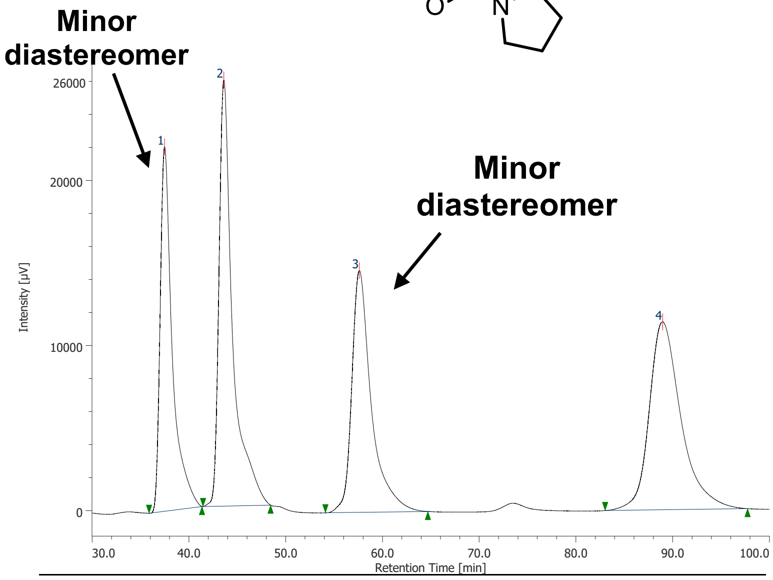

Peak No.	Retention Time (min)	Area (%)
1	37.483	21.100
**2**	**43.608**	**27.895**
3	57.608	22.532
**4**	**88.958**	**28.474**

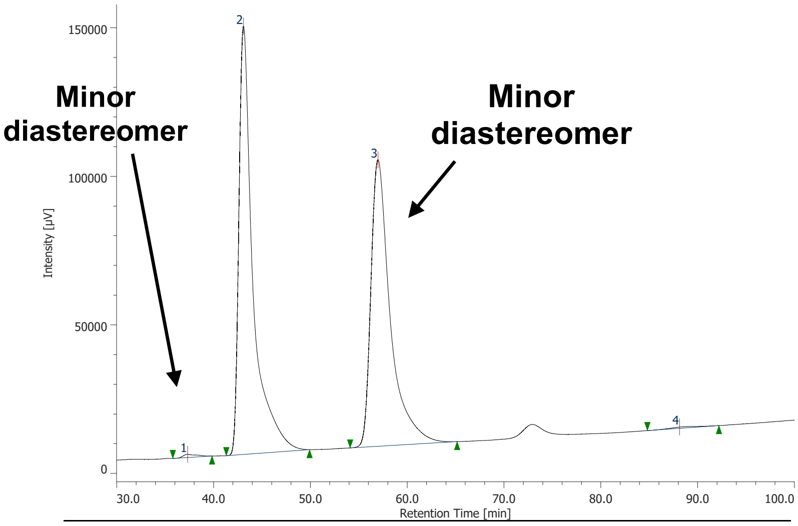

Peak No.	Retention Time (min)	Area (%)
1	37.267	0.397
**2**	**43.092**	**55.762**
3	56.975	43.515
**4**	**88.525**	**0.326**

(+)-(*E*)-2-Chloro-4-{(3*R*,*Z*)-4-ethylidene-2,3-dimethyl-1-(4-methylphenyl)pyrrolidin-3-yl}-1-(pyrrolidin-1-yl)but-2-en-1-one [(+)-4af]

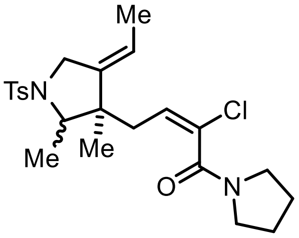

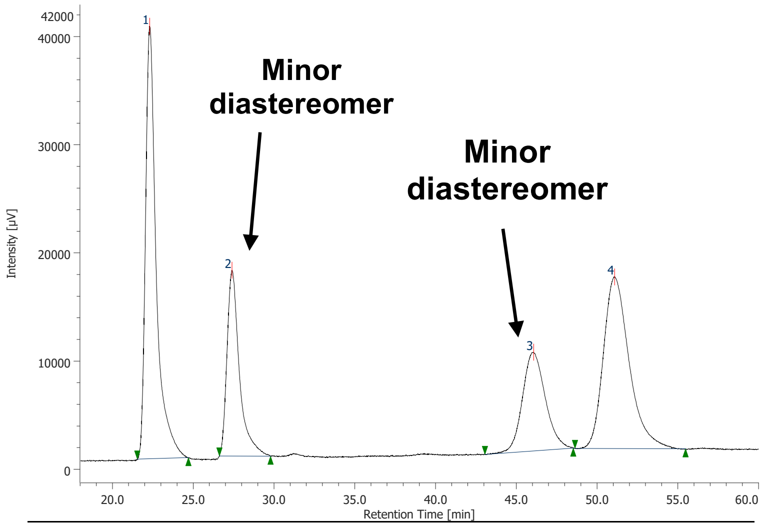

Peak No.	Retention Time (min)	Area (%)
**1**	**22.308**	**34.023**
2	27.400	16.921
3	46.075	11.119
**4**	**51.083**	**32.464**

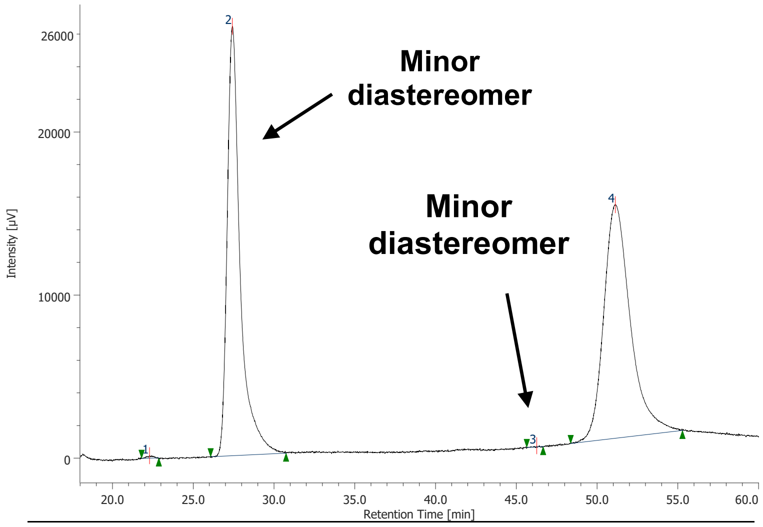

Peak No.	Retention Time (min)	Area (%)
**1**	**22.300**	**0.123**
2	27.425	47.124
3	46.267	0.025
**4**	**51.125**	**52.728**

(–)--(1*S*,5*S*,7a*R*)-*N*,1,7a-Trimethyl-3-oxo-*N*,4-diphenyl-1,3,5,6,7,7a-hexahydro-isobenzofuran-5-carboxamide [(–)-3hg (major diastereomer)]

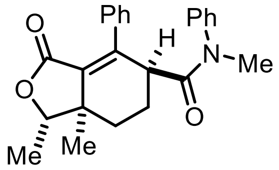

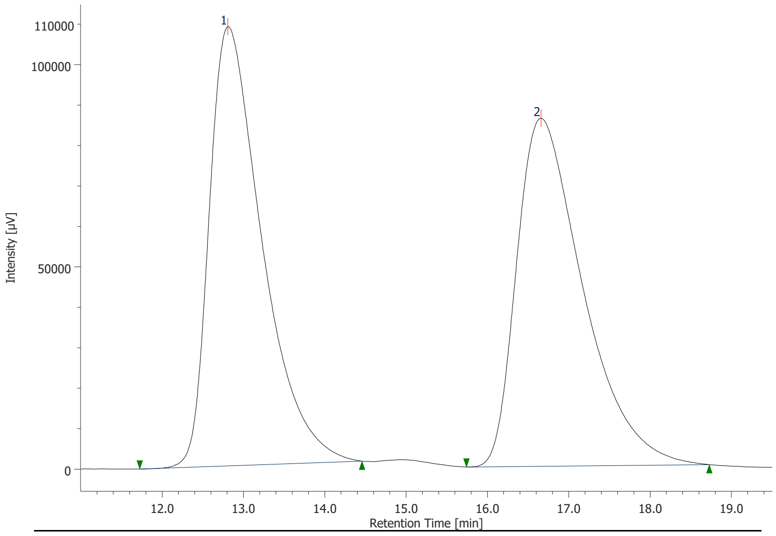

Peak No.	Retention Time (min)	Area (%)
1	12.808	50.146
2	16.658	49.854

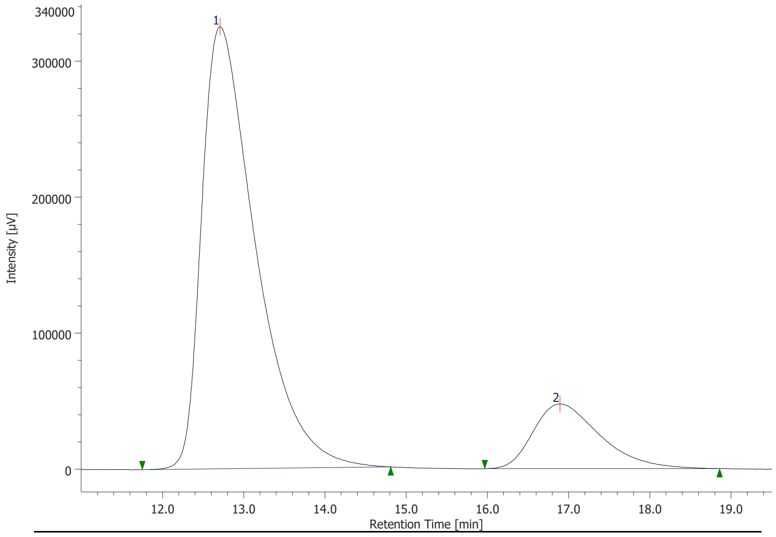

Peak No.	Retention Time (min)	Area (%)
1	12.708	84.699
2	16.892	15.301

(–)-(4*S*,5*R*)-3-{(*Z*)-Benzylidene}-4,5-dimethyl-4-[(*Z*)-3-methyl-4-oxo-4-{pyrrolidin-1-yl}but-2-en-1-yl]dihydrofuran-2(3*H*)-one [(–)-4he]

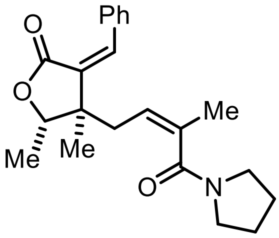

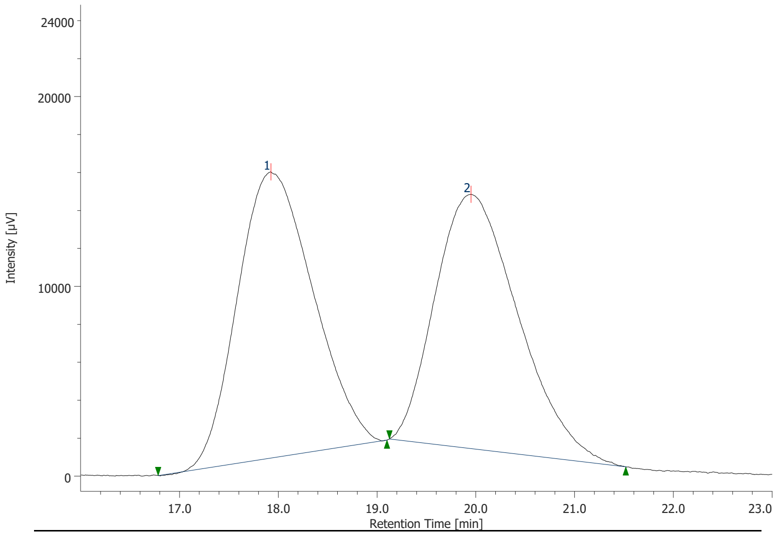

Peak No.	Retention Time (min)	Area (%)
1	17.925	50.574
2	19.950	49.426

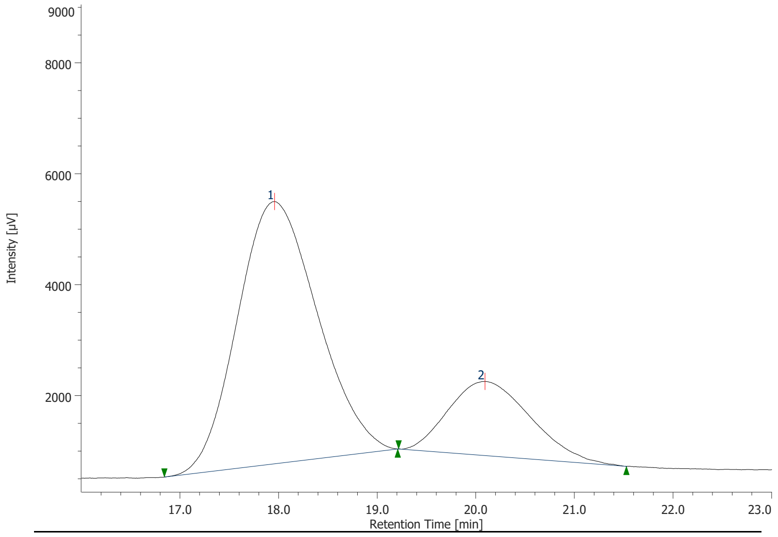

Peak No.	Retention Time (min)	Area (%)
1	17.958	77.509
2	20.092	22.491

7.2.2. PKR (type II) Using Two Different Acrylamide Derivatives (Figure 3b)

(–)-(1*S*,5*S*,7a*R*)-*N*,1,7a-Trimethyl-3-oxo-*N*,4-diphenyl-1,3,5,6,7,7a-hexahydro-isobenzofuran-5-carboxamide [(–)-3hg (major diastereomer)]

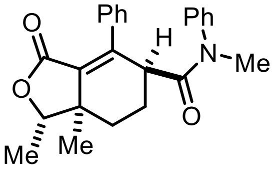

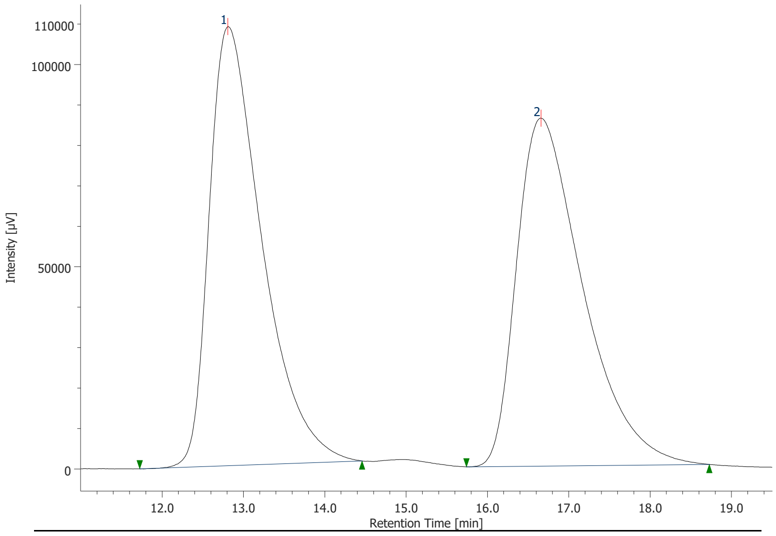

Peak No.	Retention Time (min)	Area (%)
1	12.808	50.146
2	16.658	49.854

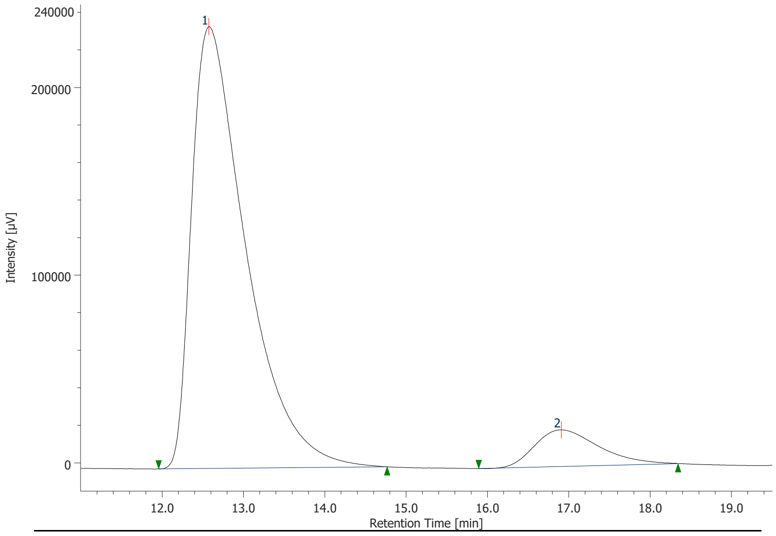

Peak No.	Retention Time (min)	Area (%)
1	12.575	91.073
2	16.908	8.927

(–)-(4*S*,5*R*)-3-(*Z*)-Benzylidene-4-{(*E*)-3-fluoro-4-oxo-4-(pyrrolidin-1-yl)but-2-en-1-yl}-4,5-dimethyldihydrofuran-2(3*H*)-one [(–)-4ha]

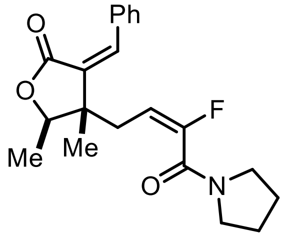

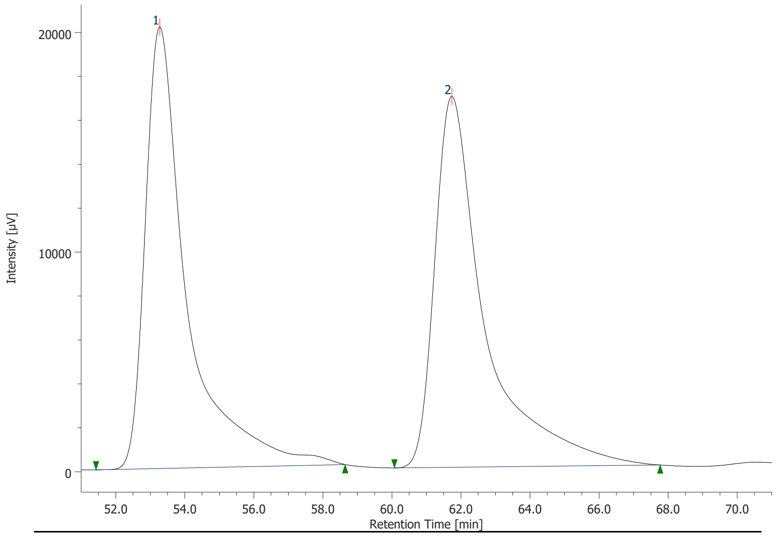

Peak No.	Retention Time (min)	Area (%)
1	53.275	50.651
2	61.733	49.349

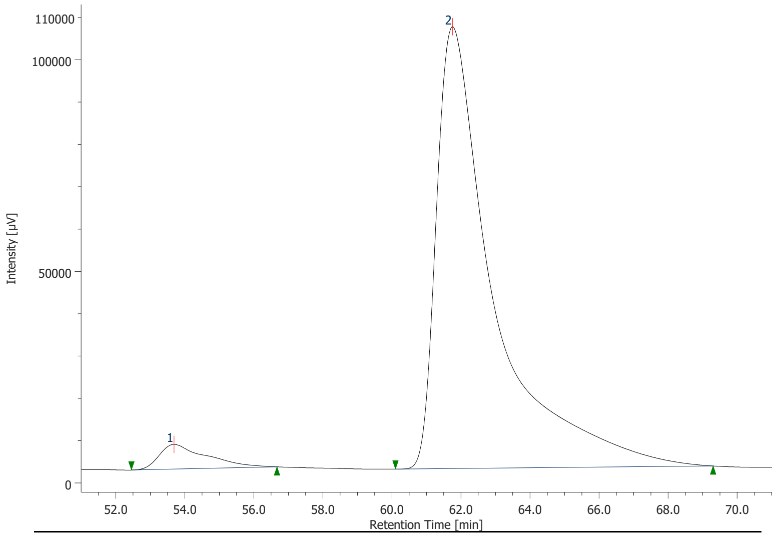

Peak No.	Retention Time (min)	Area (%)
1	53.683	4.388
2	61.750	95.612

(–)-(1*S*,5*S*,7a*R*)-4-(4-Bromophenyl)-*N*,1,7a-trimethyl-3-oxo-*N*-phenyl-1,3,5,6,7,7a-hexahydroisobenzofuran-5-carboxamide [(–)-3ig (major diastereomer)]

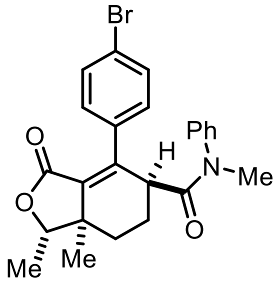

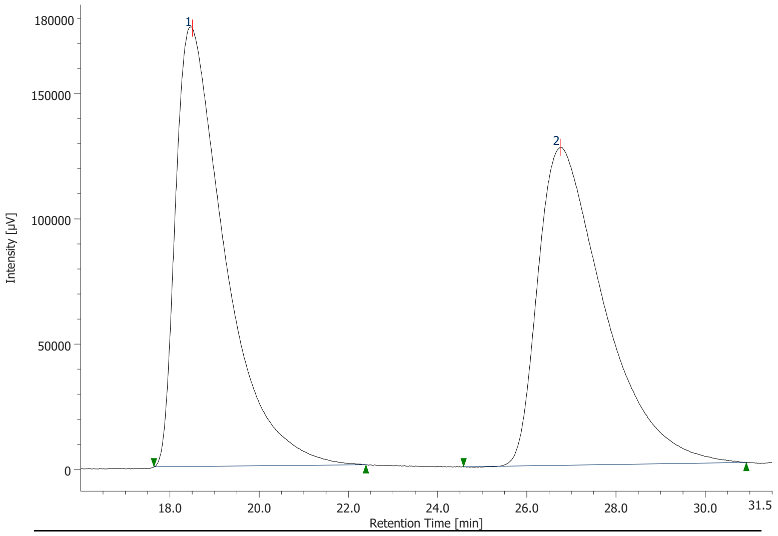

Peak No.	Retention Time (min)	Area (%)
1	18.500	50.663
2	26.750	49.337

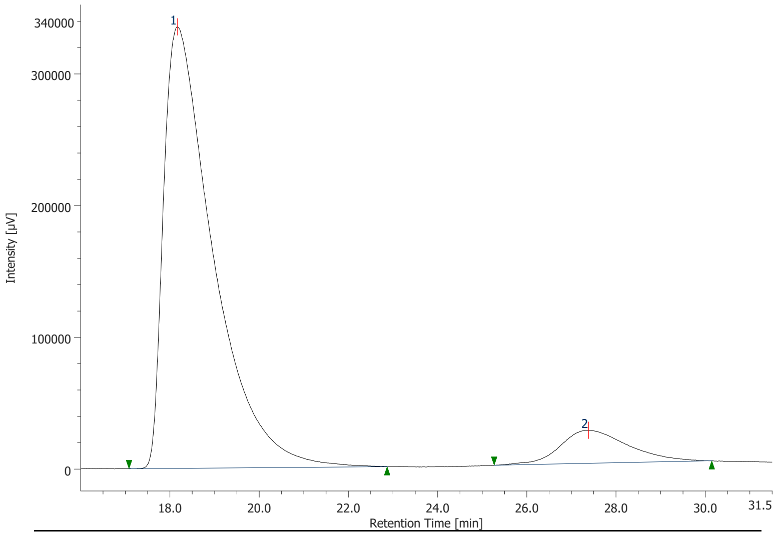

Peak No.	Retention Time (min)	Area (%)
1	18.167	90.765
2	27.383	9.235

(–)-(4*S*,5*R*)-3-(*Z*)-4-Bromobenzylidene-4-{(*E*)-3-fluoro-4-oxo-4-(pyrrolidin-1-yl)but-2-en-1-yl}-4,5-dimethyldihydrofuran-2(3*H*)-one [(–)-4ia]

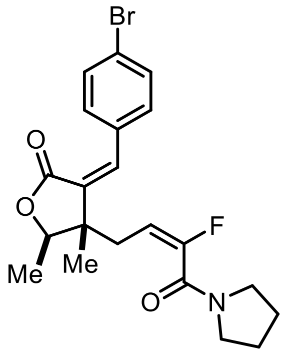

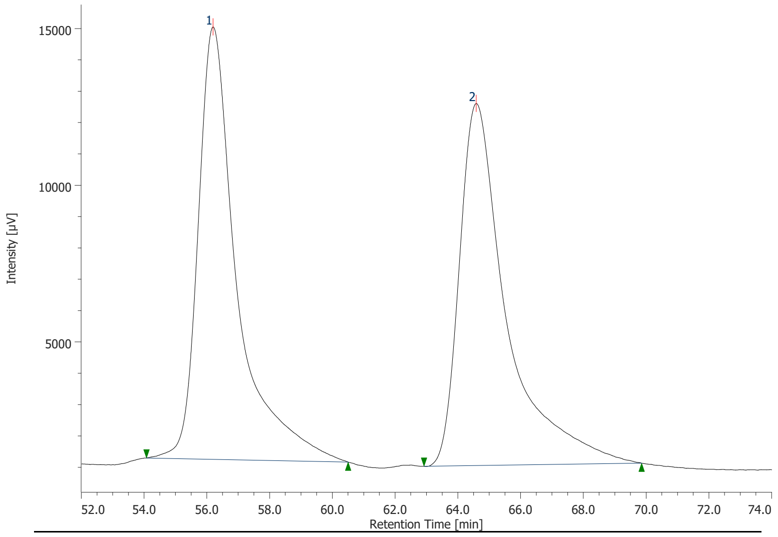

Peak No.	Retention Time (min)	Area (%)
1	56.200	50.494
2	64.583	49.506

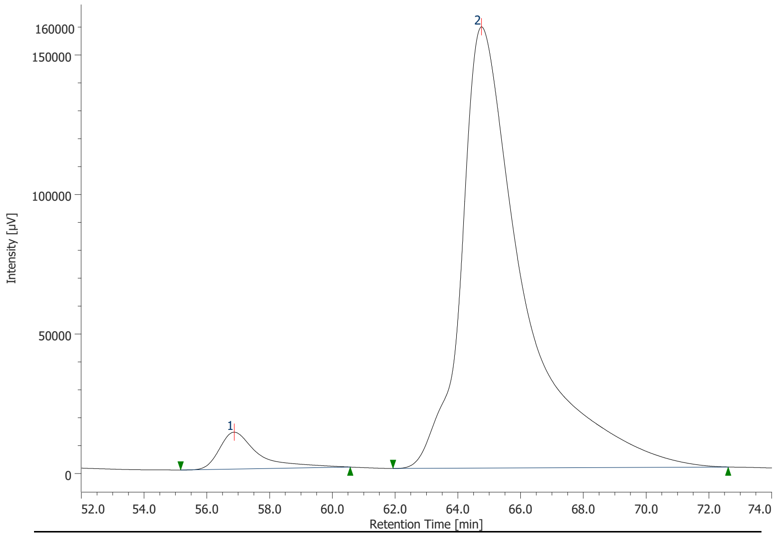

Peak No.	Retention Time (min)	Area (%)
1	56.875	4.963
2	64.750	95.037

(–)-(1*S*,5*S*,7a*R*)-*N*,7a-Dimethyl-3-oxo-*N*,1,4-triphenyl-1,3,5,6,7,7a-hexahydroisobenzofuran-5-carboxamide [(–)-3jg]

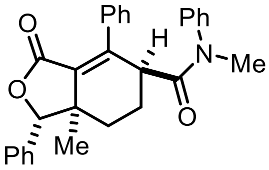

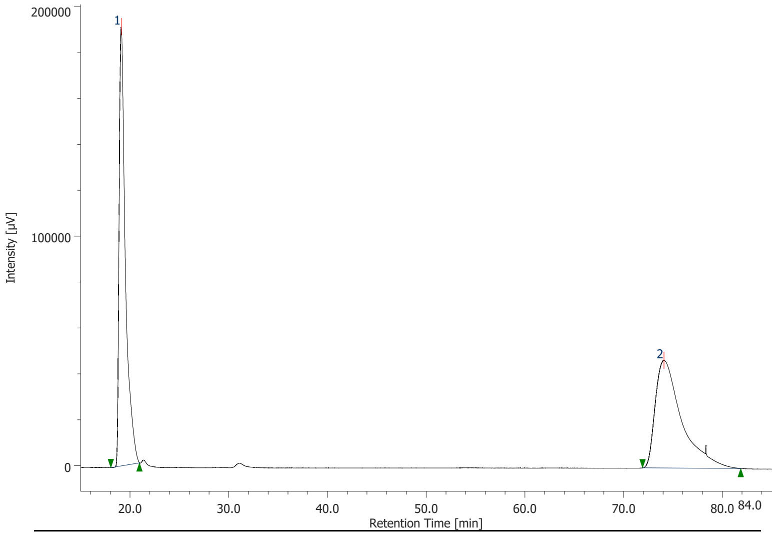

Peak No.	Retention Time (min)	Area (%)
1	19.108	49.513
2	74.083	50.487

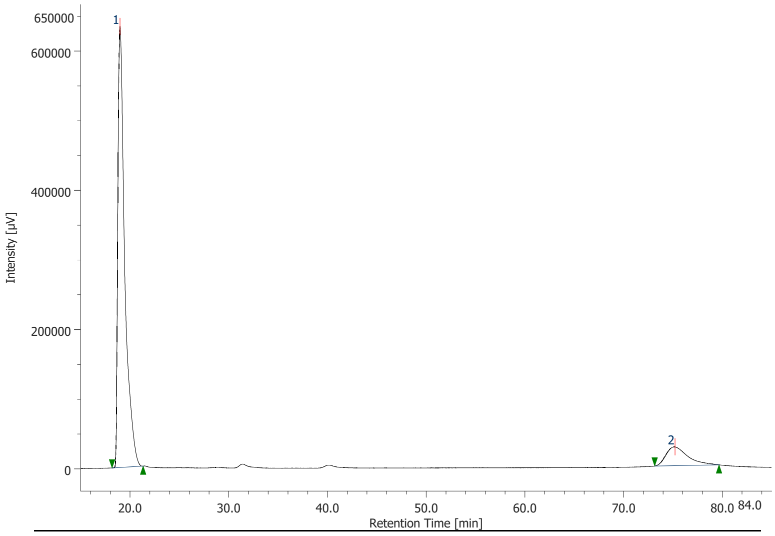

Peak No.	Retention Time (min)	Area (%)
1	18.983	88.201
2	75.192	11.799

(–)-(4*S*,5*R*)-3-(*Z*)-Benzylidene-4-{(*E*)-3-fluoro-4-oxo-4-(pyrrolidin-1-yl)but-2-en-1-yl}-4-methyl-5-phenyldihydrofuran-2(3*H*)-one [(–)-4ja]

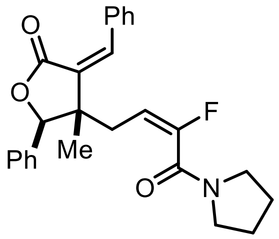

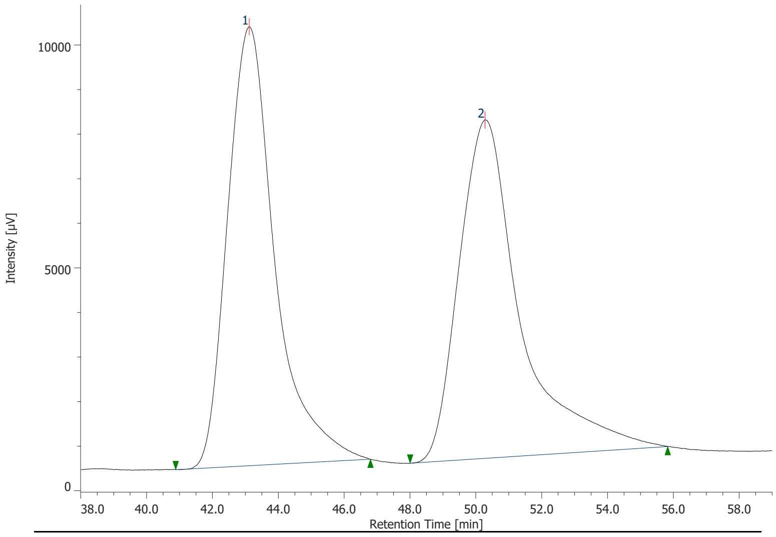

Peak No.	Retention Time (min)	Area (%)
1	43.117	50.055
2	50.275	49.945

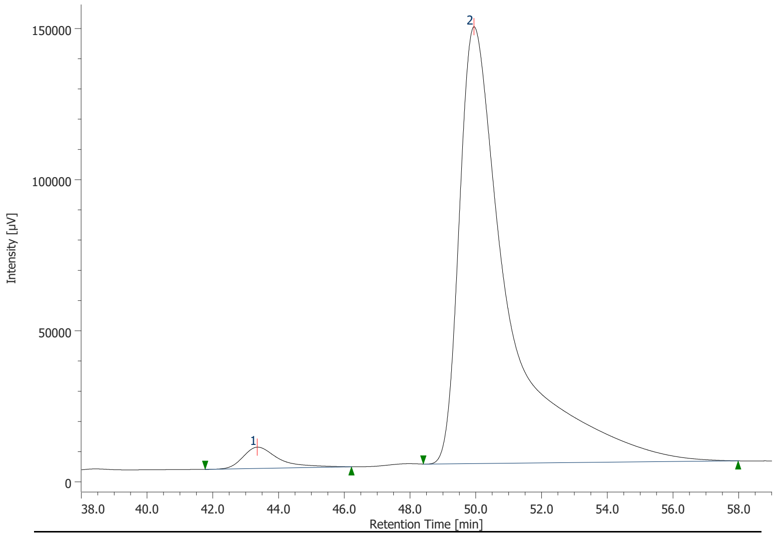

Peak No.	Retention Time (min)	Area (%)
1	43.350	3.376
2	49.942	96.624

(–)-(8*S*,10a*R*,10b*S*)-*N*,10a-Dimethyl-6-oxo-*N*,7-diphenyl-8,9,10,10a,10b,11-hexahydro-6*H*-isoindolo[2,1-a]indole-8-carboxamide [(–)-3kg]

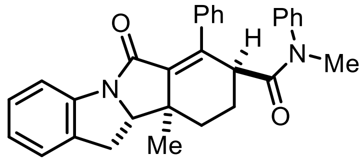

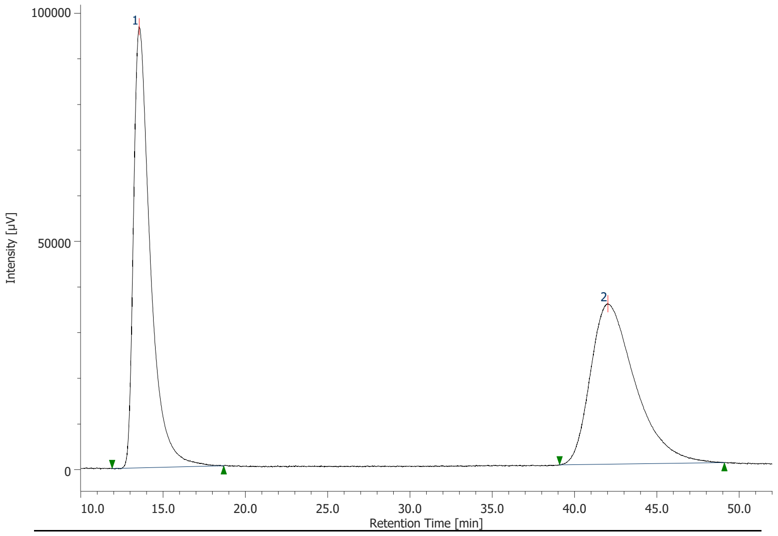

Peak No.	Retention Time (min)	Area (%)
1	13.550	50.932
2	42.017	49.068

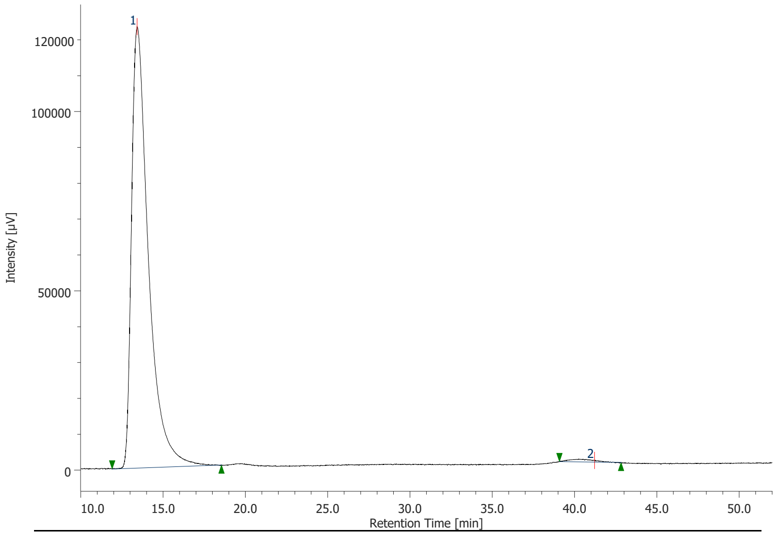

Peak No.	Retention Time (min)	Area (%)
1	13.425	99.108
2	41.200	0.892

(–)-(1*S*,9a*R*)-2-(*Z*)-Benzylidene-1-{(*E*)-3-fluoro-4-oxo-4-(pyrrolidin-1-yl)but-2-en-1-yl}-1-methyl-1,2,9,9a-tetrahydro-3*H*-pyrrolo[1,2-a]indol-3-one [(–)-4ka]

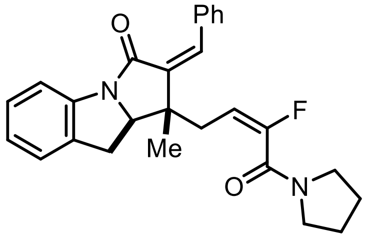

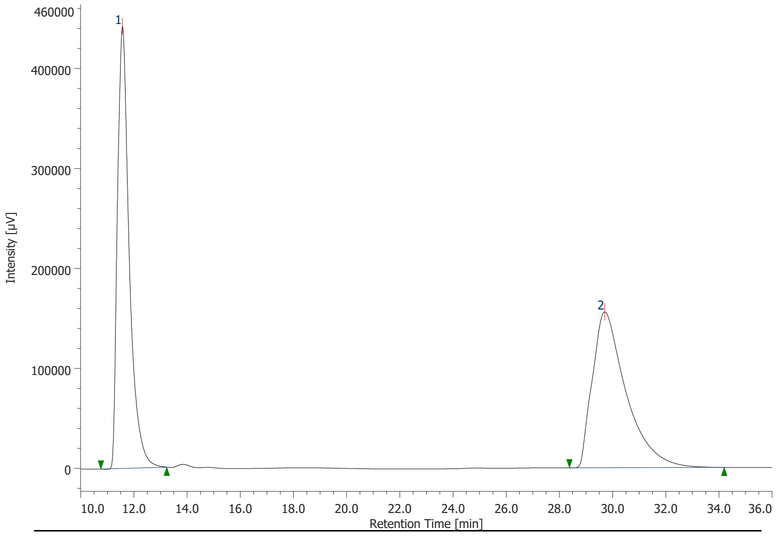

Peak No.	Retention Time (min)	Area (%)
1	11.567	49.950
2	29.700	50.050

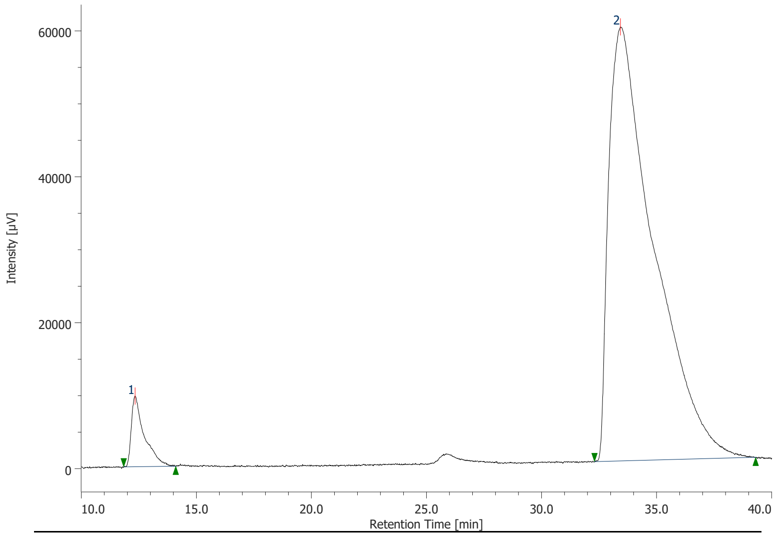

Peak No.	Retention Time (min)	Area (%)
1	12.333	4.493
2	33.425	95.507

(–)-(1*S*,5*S*,7a*R*)-*N*,1,7a-Trimethyl-*N*,4-diphenyl-1,3,5,6,7,7a-hexahydroisobenzofuran-5-carboxamide [(–)-3lg]

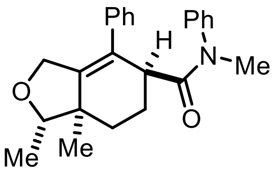

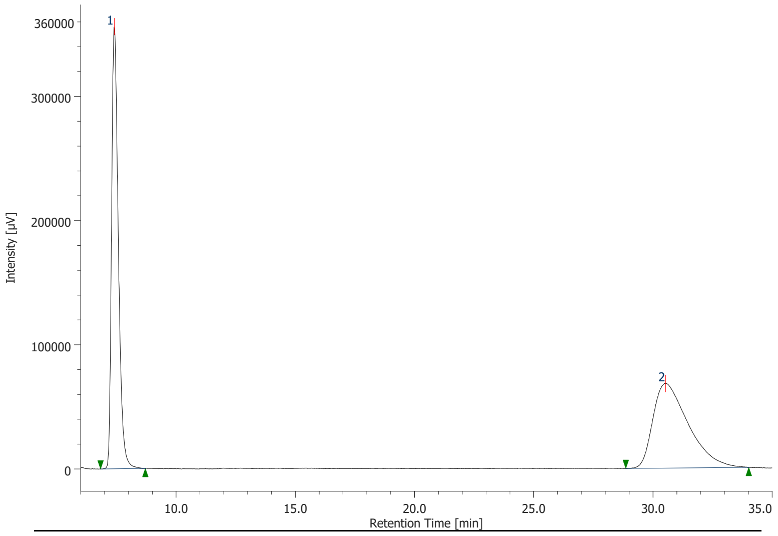

Peak No.	Retention Time (min)	Area (%)
1	7.408	49.913
2	30.525	50.069

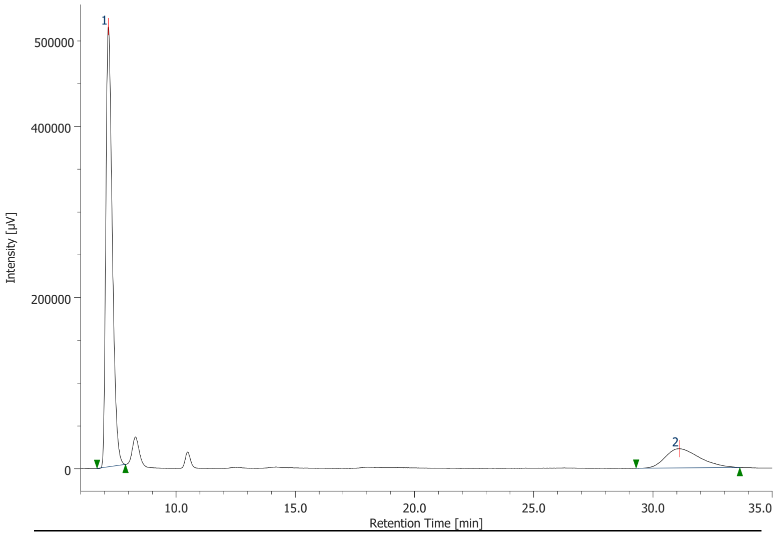

Peak No.	Retention Time (min)	Area (%)
1	7.158	81.116
2	31.100	18.884

(+)-(*E*)-4-{(2*R*,3*S*)-4-(*Z*)-Benzylidene-2,3-dimethyltetrahydrofuran-3-yl}-2-fluoro-1-(pyrrolidin-1-yl)but-2-en-1-one [(+)-4la]

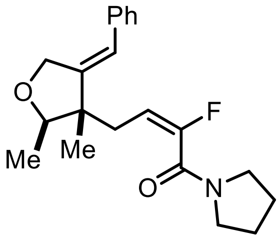

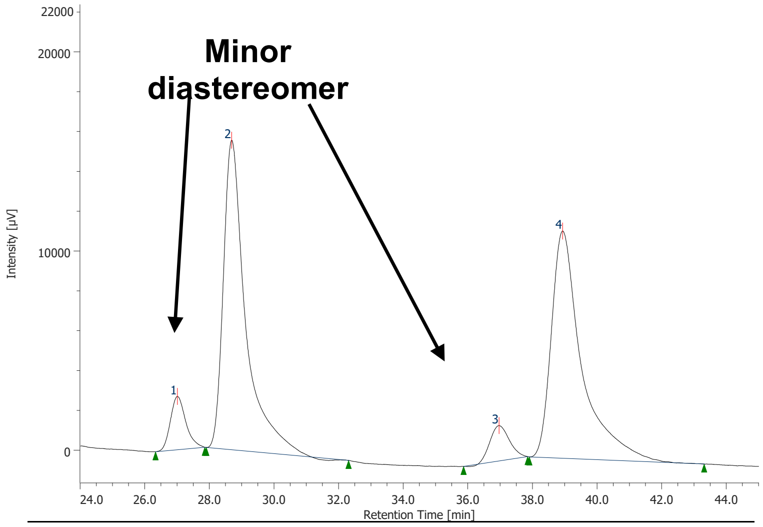

Peak No.	Retention Time (min)	Area (%)
1	27.088	5.341
**2**	**28.692**	**45.234**
3	36.967	4.502
**4**	**38.933**	**44.924**

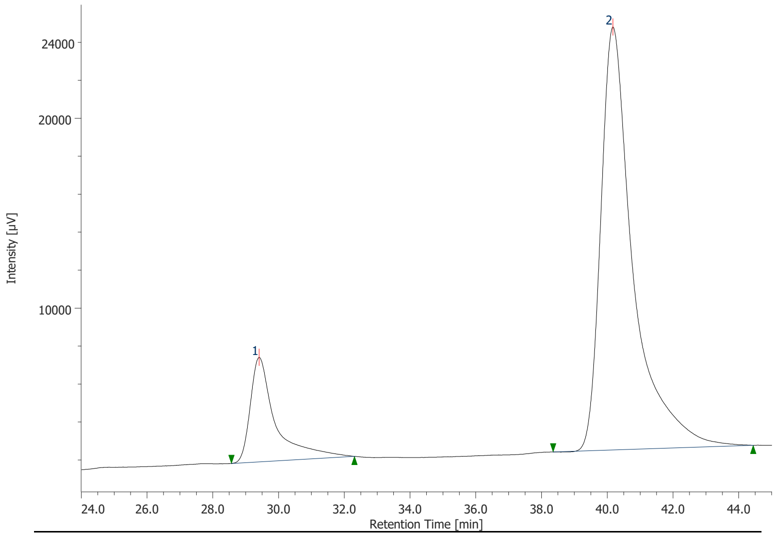

Peak No.	Retention Time (min)	Area (%)
1	29.408	16.185
2	40.167	83.815

(–)-(1*S*,5*S*,7a*R*)-*N*,*N*-Diethyl-1,7a-dimethyl-3-oxo-4-phenyl-1,3,5,6,7,7a-hexahydro-isobenzofuran-5-carboxamide [(–)-3hh]

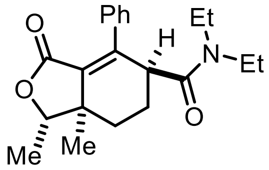

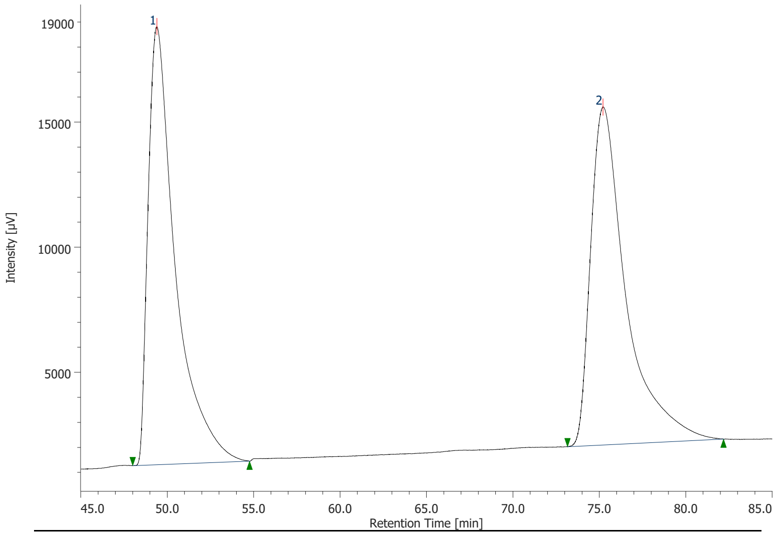

Peak No.	Retention Time (min)	Area (%)
1	49.400	49.934
2	75.208	50.066

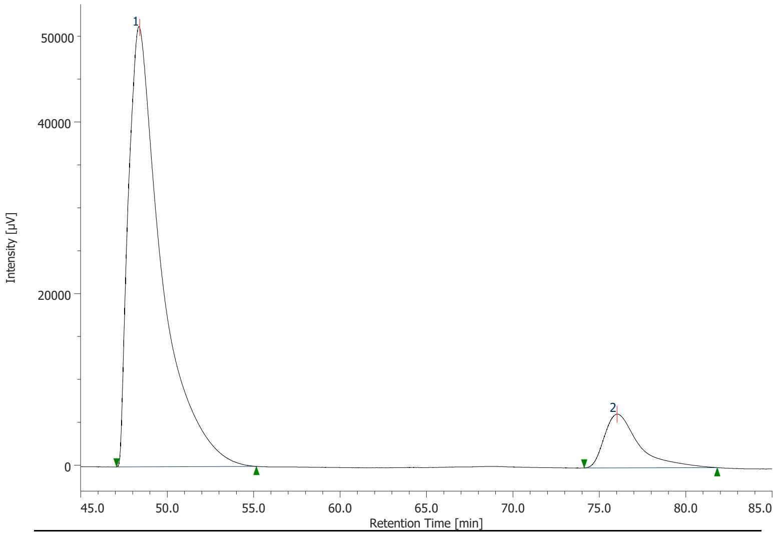

Peak No.	Retention Time (min)	Area (%)
1	48.408	88.665
2	76.017	11.335

(–)-(4*S*,5*R*)-3-(*Z*)-Benzylidene-4-{(*E*)-3-fluoro-4-oxo-4-(pyrrolidin-1-yl)but-2-en-1-yl}-4,5-dimethyldihydrofuran-2(3*H*)-one [(–)-4ha]

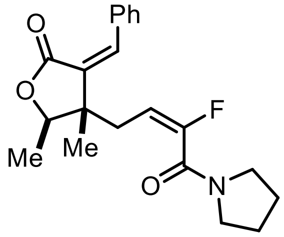

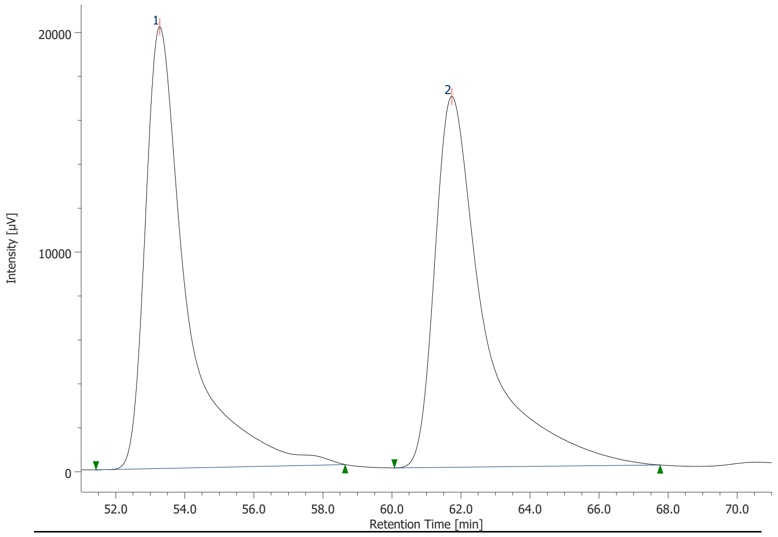

Peak No.	Retention Time (min)	Area (%)
1	53.275	50.651
2	61.733	49.349

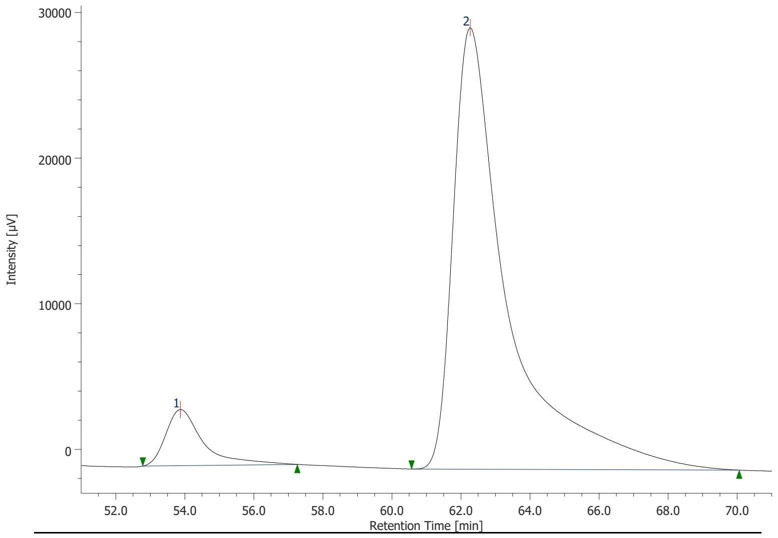

Peak No.	Retention Time (min)	Area (%)
1	53.867	7.983
2	62.267	92.017

(–)-(1*S*,5*S*,7a*R*)-*N*,1,7a-Trimethyl-3-oxo-*N*,4-diphenyl-1,3,5,6,7,7a-hexahydro-isobenzofuran-5-carboxamide [(–)-3hg (major diastereomer)]

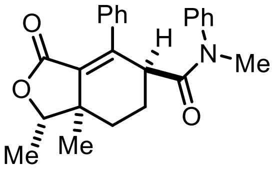

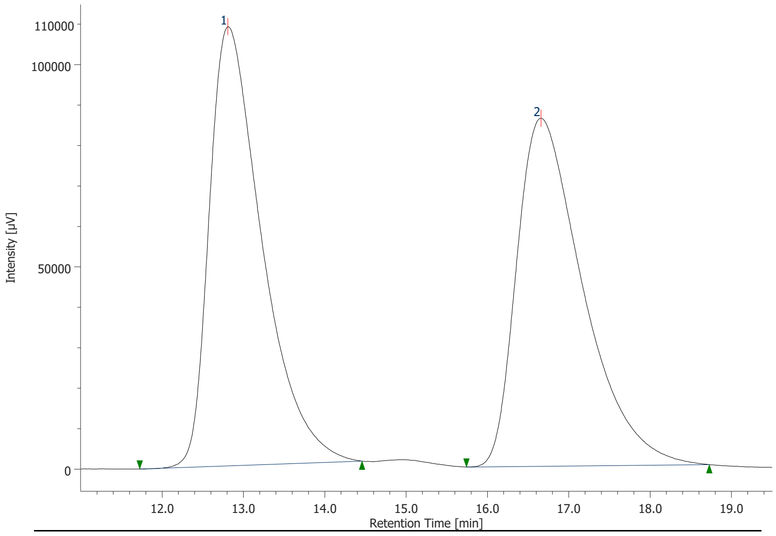

Peak No.	Retention Time (min)	Area (%)
1	12.808	50.146
2	16.658	49.854

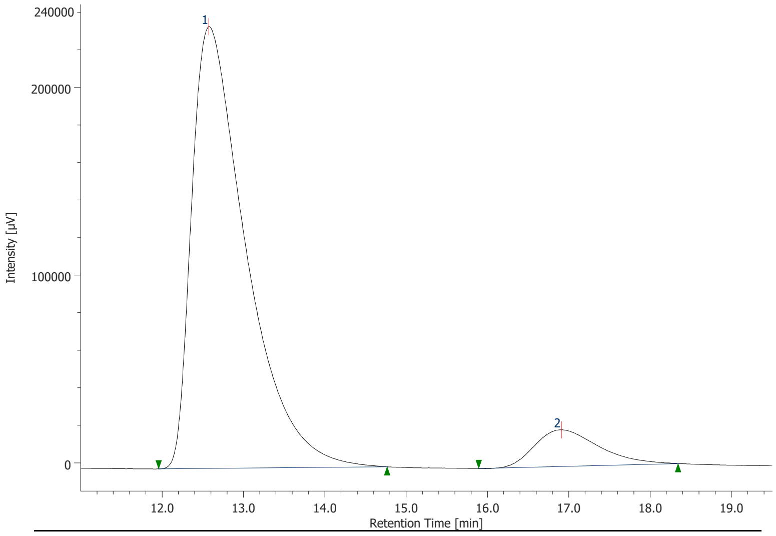

Peak No.	Retention Time (min)	Area (%)
1	12.600	90.071
2	16.875	9.929

(+)-(*E*)-4-{(2*R*,3*S*)-4-(*Z*)-Benzylidene-2,3-dimethyl-5-oxotetrahydrofuran-3-yl}-2-fluoro-*N*-methyl-*N*-phenylbut-2-enamide [(–)-4hi]

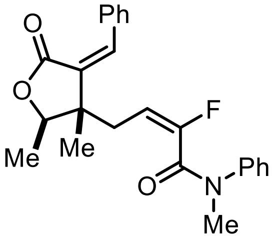

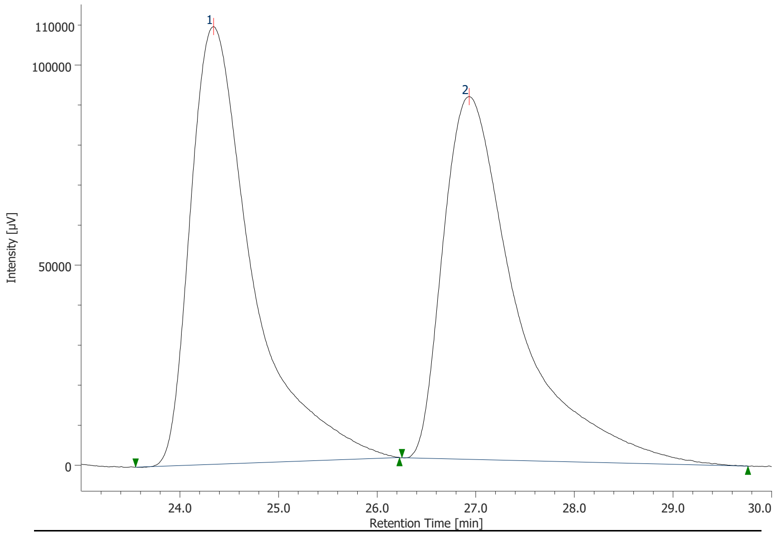

Peak No.	Retention Time (min)	Area (%)
1	24.342	50.251
2	26.933	49.749

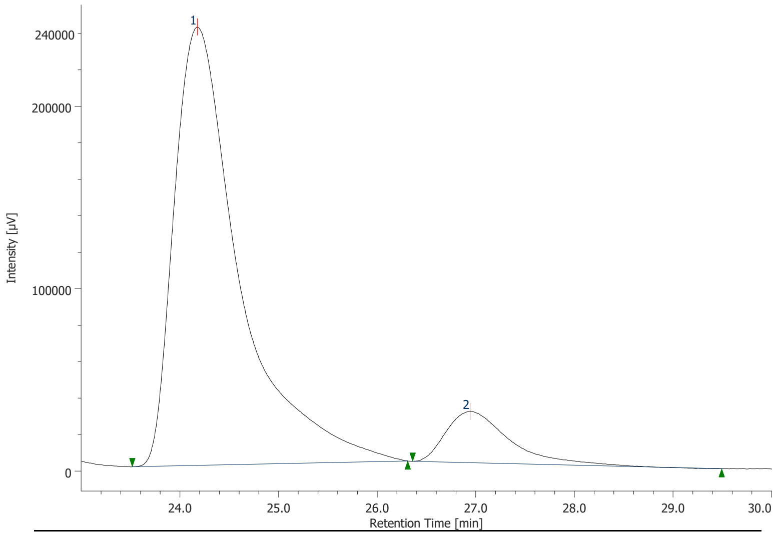

Peak No.	Retention Time (min)	Area (%)
1	24.175	89.781
2	26.942	10.219

(–)-(1*S*,5*S*,7a*R*)-*N*,1,7a-Trimethyl-3-oxo-*N*,4-diphenyl-1,3,5,6,7,7a-hexahydro-isobenzofuran-5-carboxamide [(–)-3hg (major diastereomer)]

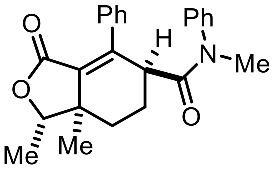

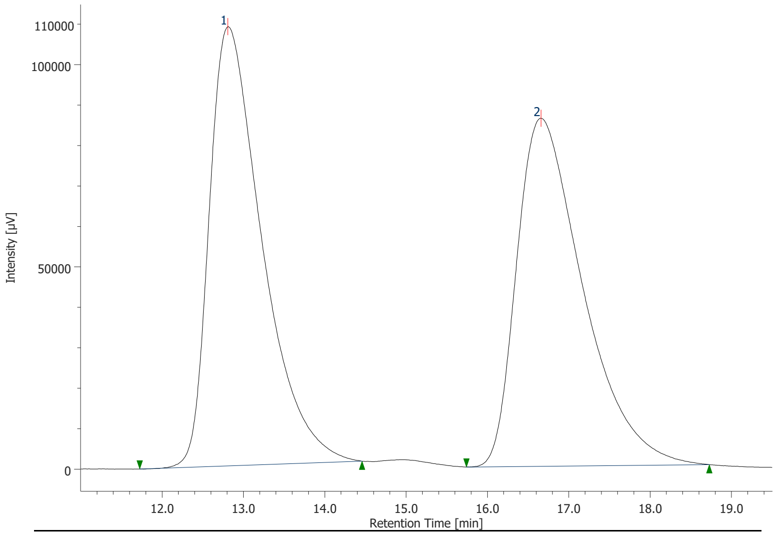

Peak No.	Retention Time (min)	Area (%)
1	12.808	50.146
2	16.658	49.854

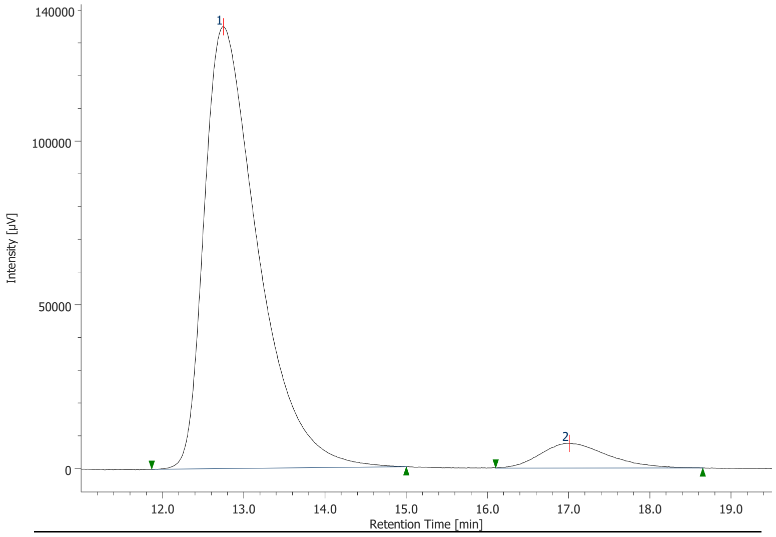

Peak No.	Retention Time (min)	Area (%)
1	12.750	90.071
2	17.008	9.929

(+)-(4*S*,5*R*)-3-{(*Z*)-Benzylidene}-4,5-dimethyl-4-[(*Z*)-3-methyl-4-oxo-4-{pyrrolidin-1-yl}but-2-en-1-yl]dihydrofuran-2(3*H*)-one [(+)-4he]

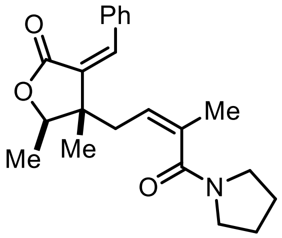

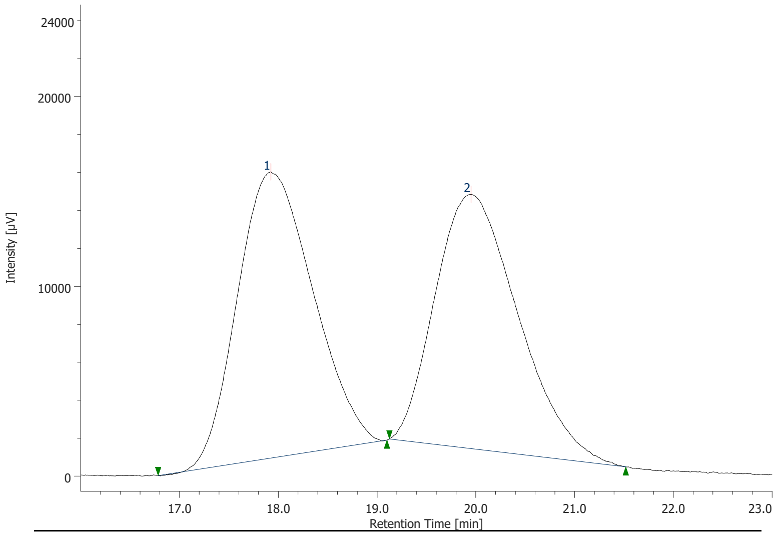

Peak No.	Retention Time (min)	Area (%)
1	17.925	50.574
2	19.950	49.426

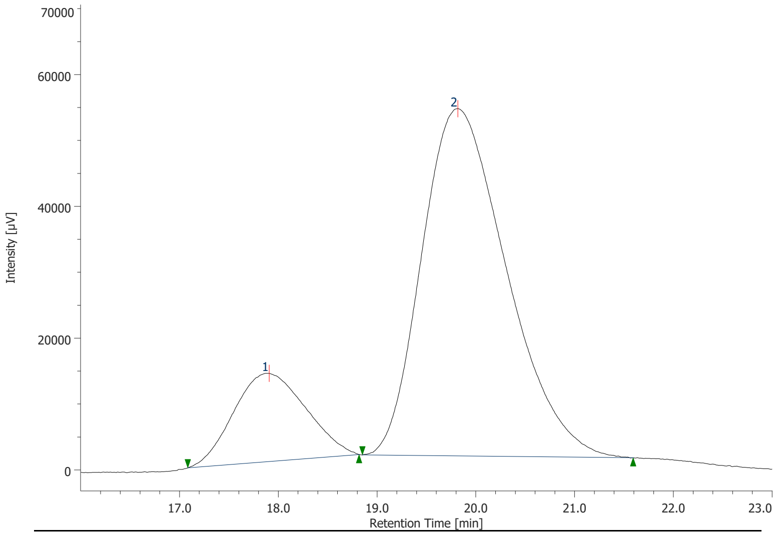

Peak No.	Retention Time (min)	Area (%)
1	17.908	17.762
2	19.817	82.238

(–)-(1*S*,5*S*,7a*R*)-*N*,7a-Dimethyl-3-oxo-*N*,1,4-triphenyl-1,3,5,6,7,7a-hexahydroisobenzofuran-5-carboxamide [(–)-3jg]

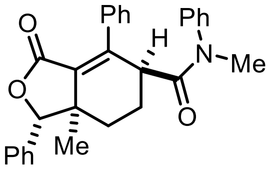

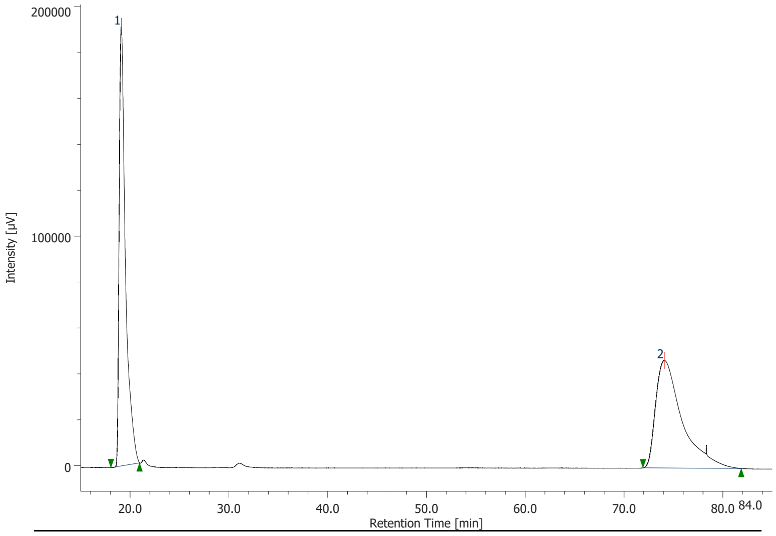

Peak No.	Retention Time (min)	Area (%)
1	19.108	49.513
2	74.083	50.487

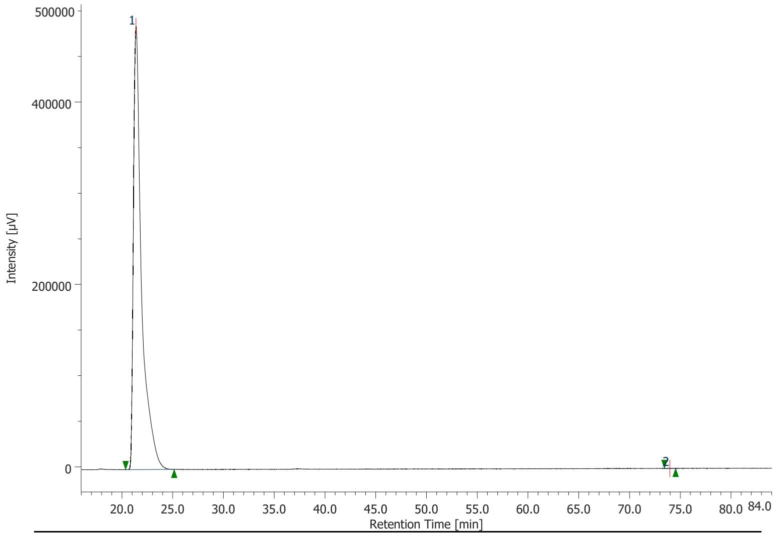

Peak No.	Retention Time (min)	Area (%)
1	20.392	99.998
2	73.950	0.002

(–)-(4*S*,5*R*)-3-{(*Z*)-Benzylidene}-4-methyl-4-[(*Z*)-3-methyl-4-oxo-4-{pyrrolidin-1-yl}but-2-en-1-yl]-5-phenyldihydrofuran-2(3H)-one [(–)-4je]

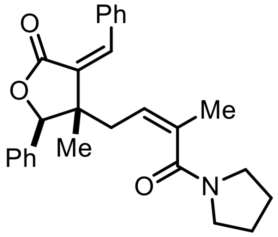

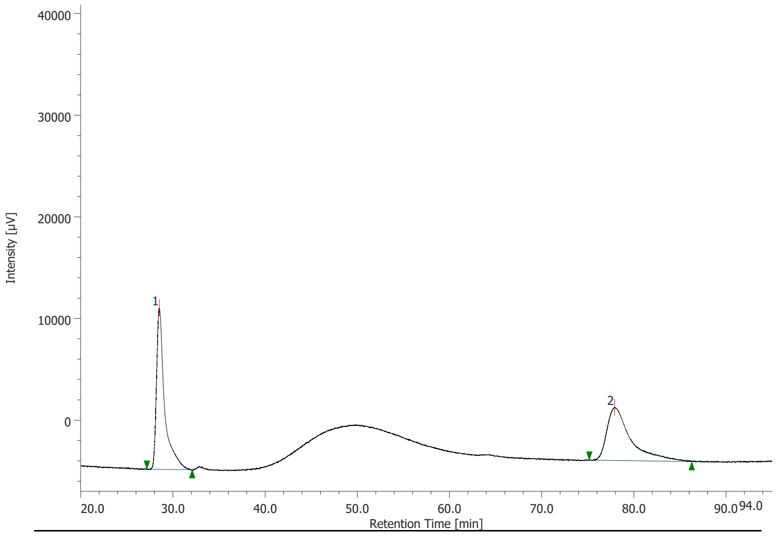

Peak No.	Retention Time (min)	Area (%)
1	28.525	50.791
2	77.892	49.209

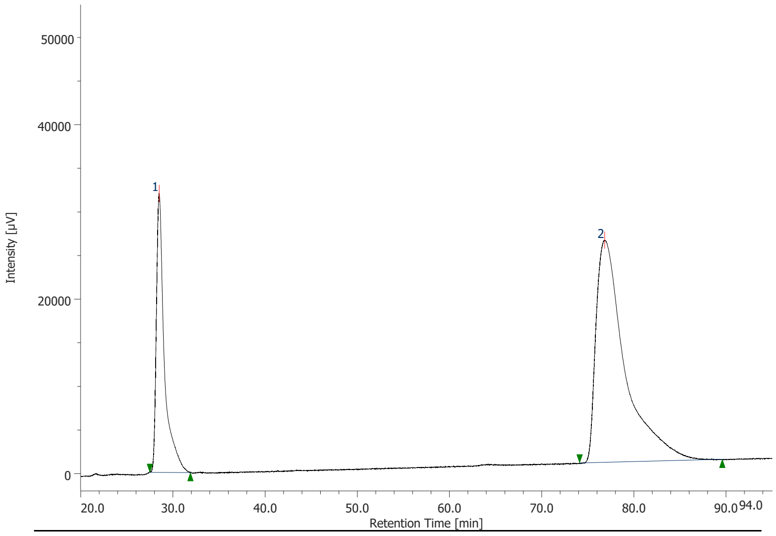

Peak No.	Retention Time (min)	Area (%)
1	28.500	25.073
2	76.833	74.927

7.3. Rh-Catalyzed Enantioselective Desymmetrization of Achiral Dienynes with Acrylamide Derivatives (Figure 4)

(–)-{(1*R*,5*R*,7a*R*)-5-Fluoro-2-(4-methylphenyl)-4-phenyl-1-vinyl-2,3,5,6,7,7a-hexahydro-1*H*-isoindol-5-yl}(pyrrolidin-1-yl)methanone [(–)-6aa (major diastereomer)]

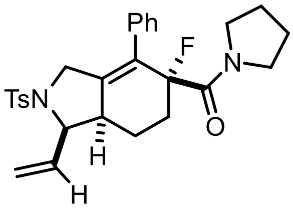

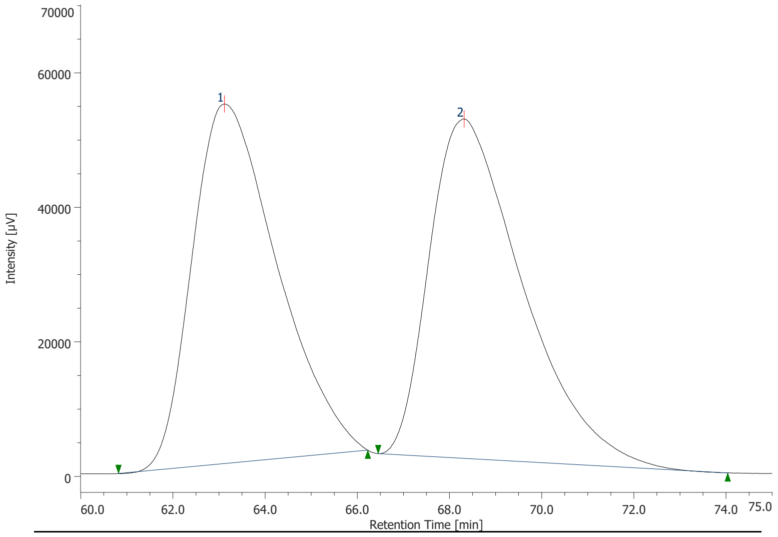

Peak No.	Retention Time (min)	Area (%)
1	63.117	49.052
2	68.317	50.948

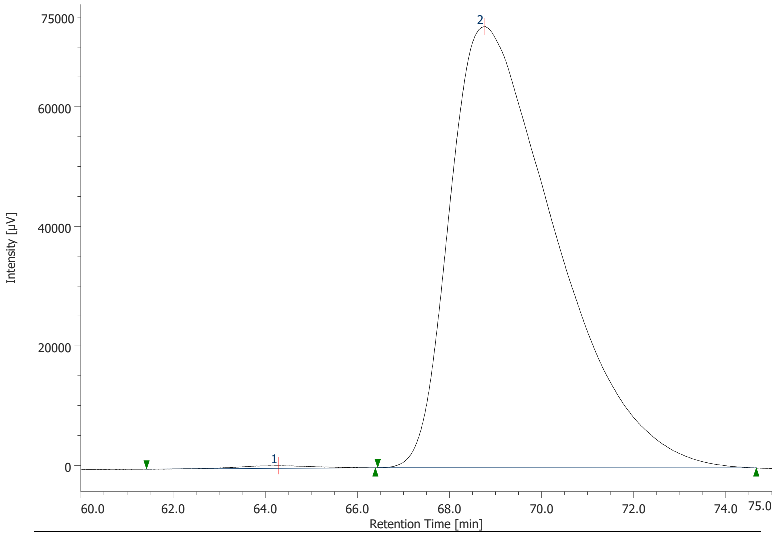

Peak No.	Retention Time (min)	Area (%)
1	64.283	0.447
2	68.750	99.553

(–)-{(1*R*,5*R*,7a*R*)-5-Fluoro-2-(4-methylphenyl)-4-phenyl-1-vinyl-2,3,5,6,7,7a-hexahydro-1*H*-isoindol-5-yl}(morpholino)methanone [(–)-6ab (major diastereomer)]

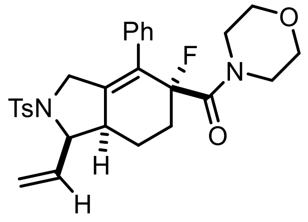

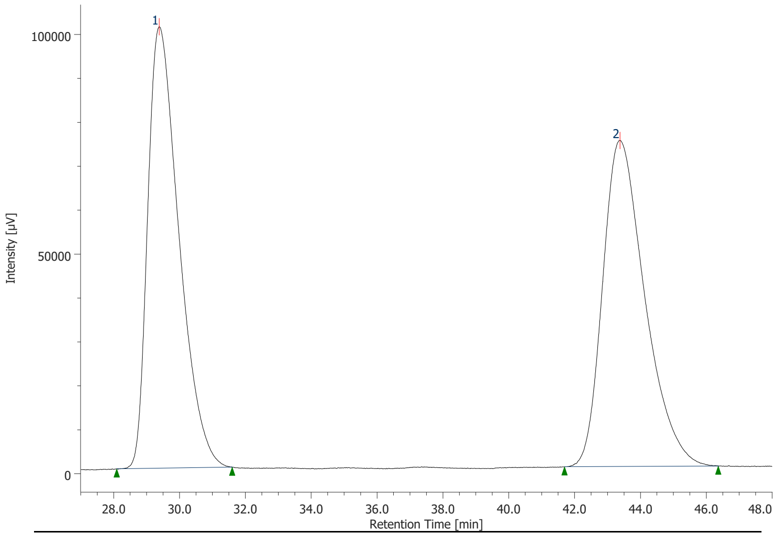

Peak No.	Retention Time (min)	Area (%)
1	29.383	50.067
2	43.375	49.933

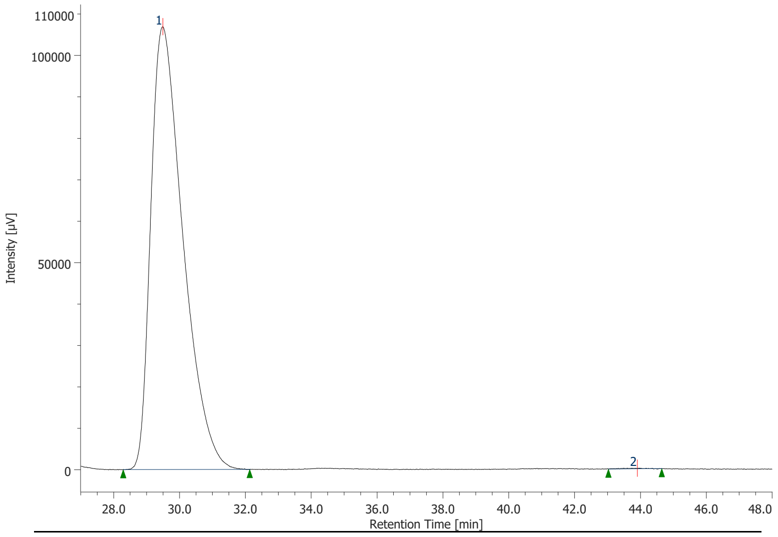

Peak No.	Retention Time (min)	Area (%)
1	29.492	99.901
2	43.900	0.099

(–)-(1*R*,5*R*,7a*R*)-5-Fluoro-*N*-methyl-2-(4-methylphenyl)-*N*,4-diphenyl-1-vinyl-2,3,5,6,7,7a-hexahydro-1*H*-isoindole-5-carboxamide [(–)-6ai]

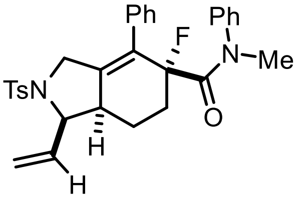

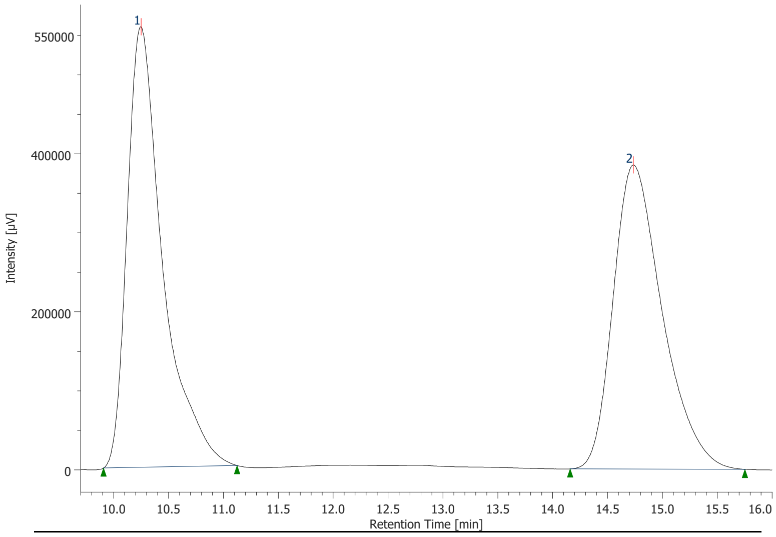

Peak No.	Retention Time (min)	Area (%)
1	10.250	51.233
2	14.733	48.777

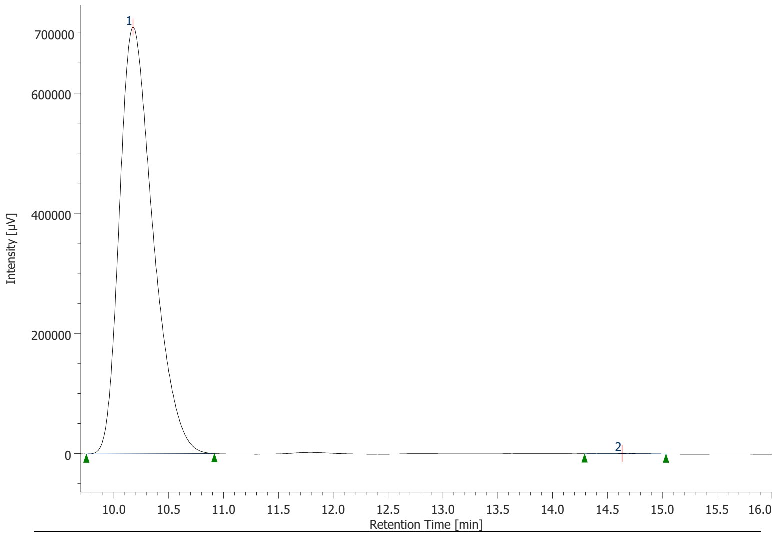

Peak No.	Retention Time (min)	Area (%)
1	10.175	99.898
2	14.633	0.102

(+)-(1*R*,5*R*,7a*R*)-5-Fluoro-2-(4-methylphenyl)-*N*,*N*,4-triphenyl-1-vinyl-2,3,5,6,7,7a-hexahydro-1*H*-isoindole-5-carboxamide [(+)-6ac (major diastereomer)]

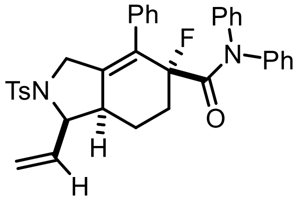

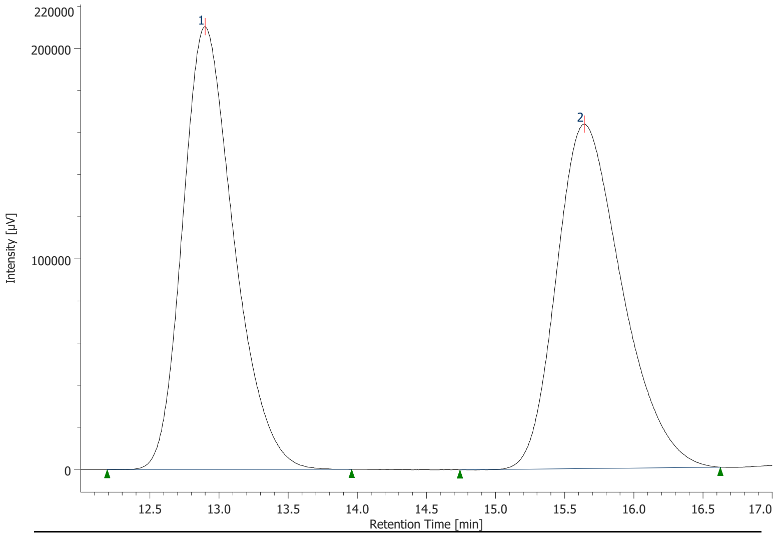

Peak No.	Retention Time (min)	Area (%)
1	12.900	50.181
2	15.642	49.819

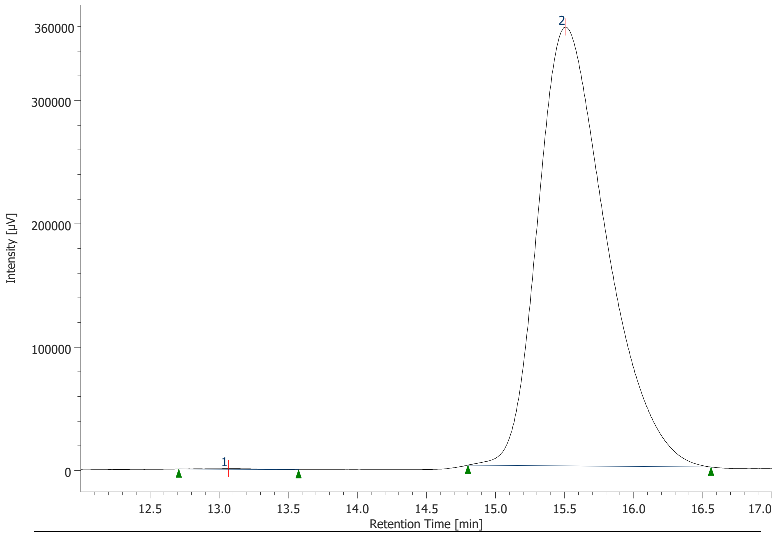

Peak No.	Retention Time (min)	Area (%)
1	13.067	0.120
2	15.508	99.880

(+)-(1*R*,5*R*,7a*R*)-5-Fluoro-*N*-methoxy-*N*-methyl-2-(4-methylphenyl)-4-phenyl-1-vinyl-2,3,5,6,7,7a-hexahydro-1*H*-isoindole-5-carboxamide [(+)-6aj]

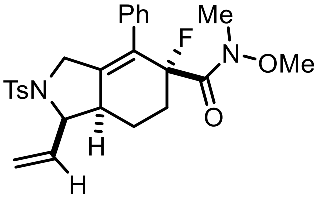

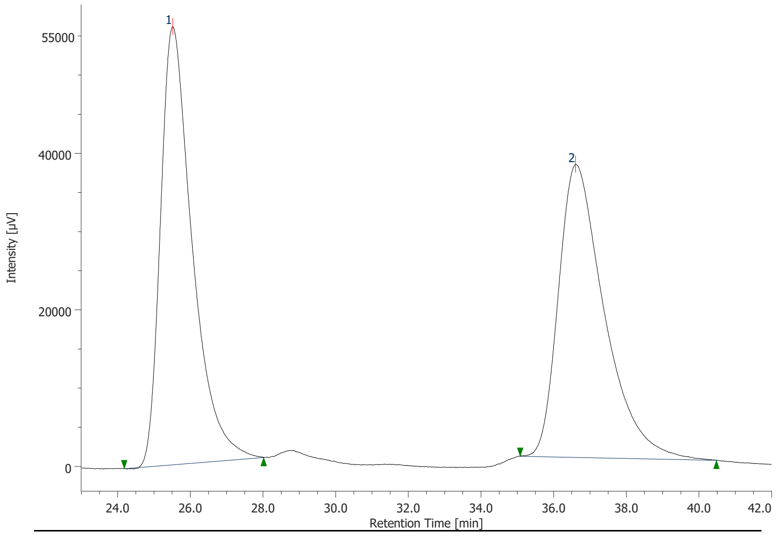

Peak No.	Retention Time (min)	Area (%)
1	25.517	50.933
2	36.600	49.067

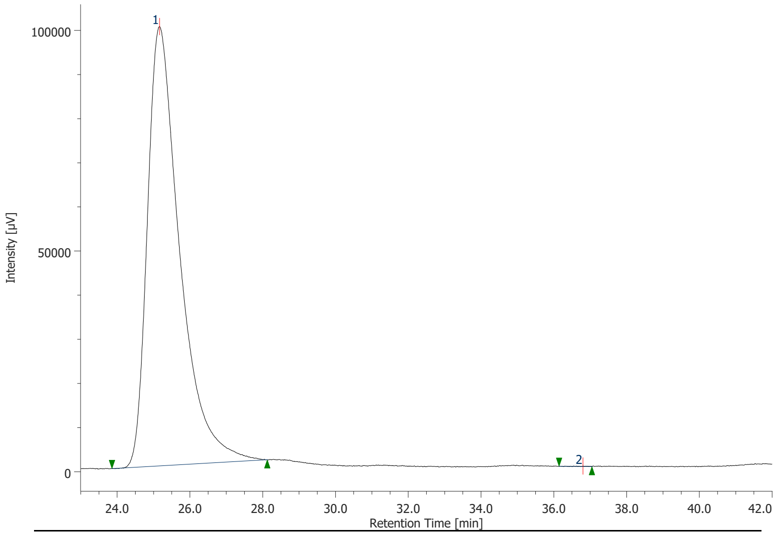

Peak No.	Retention Time (min)	Area (%)
1	25.167	99.975
2	36.800	0.025

(–)-{(1*R*,5*R*,7a*R*)-5-Fluoro-4-(4-methoxyphenyl)-2-(4-methylphenyl)-1-vinyl-2,3,5,6,7,7a-hexahydro-1*H*-isoindol-5-yl}(pyrrolidin-1-yl)methanone [(–)-6ba (major diastereomer)]

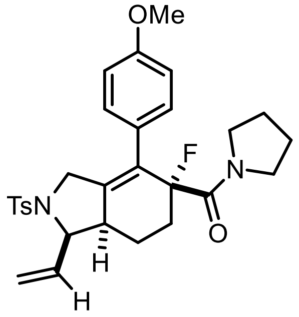

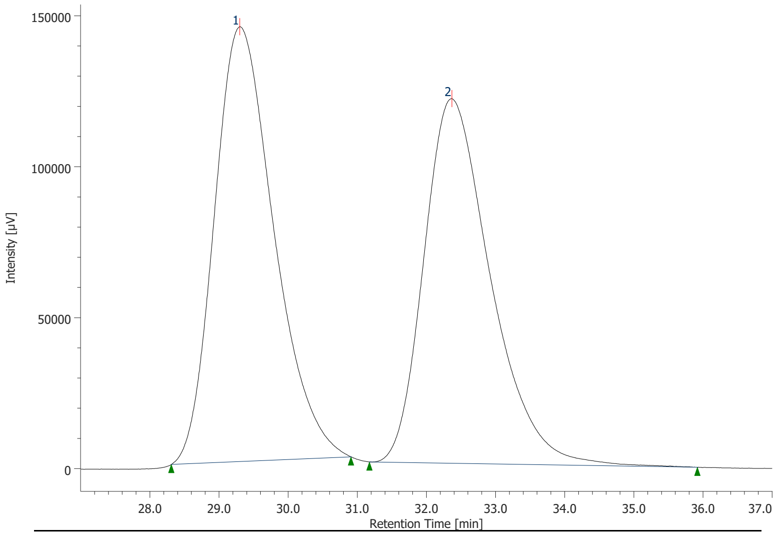

Peak No.	Retention Time (min)	Area (%)
1	29.300	51.686
2	32.367	48.314

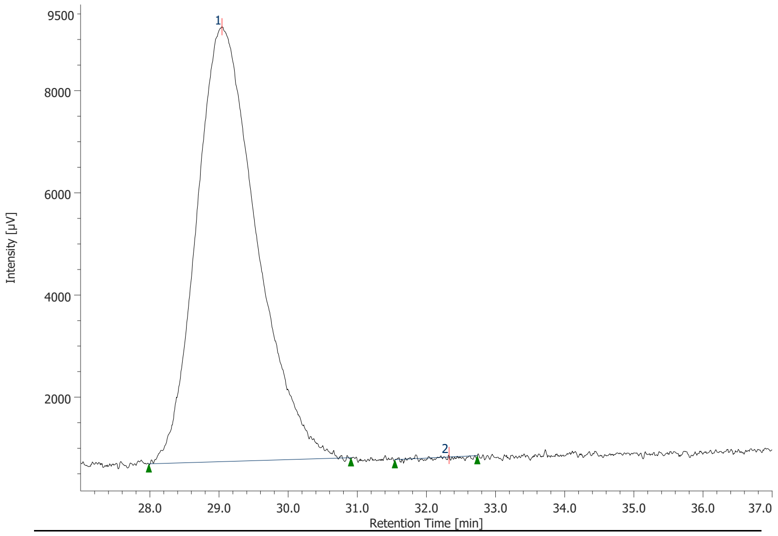

Peak No.	Retention Time (min)	Area (%)
1	29.042	99.915
2	32.325	0.085

(–)-{(1*R*,5*R*,7a*R*)-4-(4-Chlorophenyl)-5-fluoro-2-(4-methylphenyl)-1-vinyl-2,3,5,6,7,7a-hexahydro-1*H*-isoindol-5-yl}(pyrrolidin-1-yl)methanone [(–)-6ca (major diastereomer)]

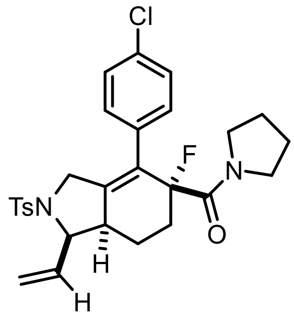

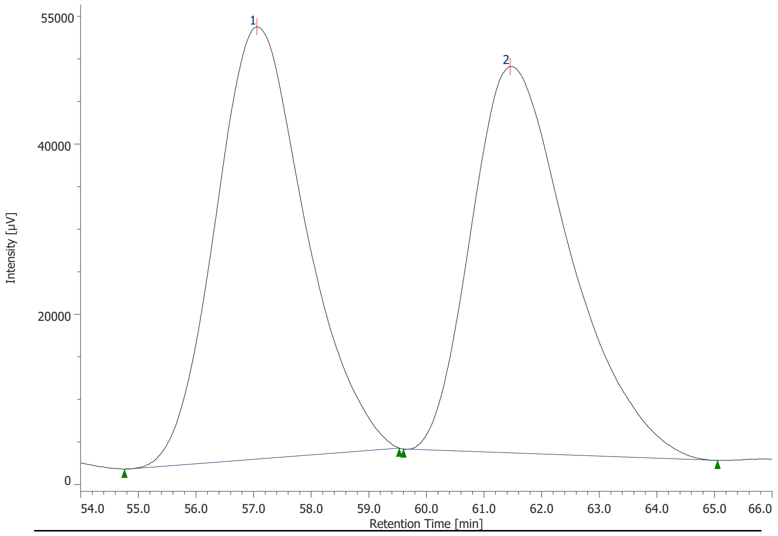

Peak No.	Retention Time (min)	Area (%)
1	57.058	50.358
2	61.450	49.642

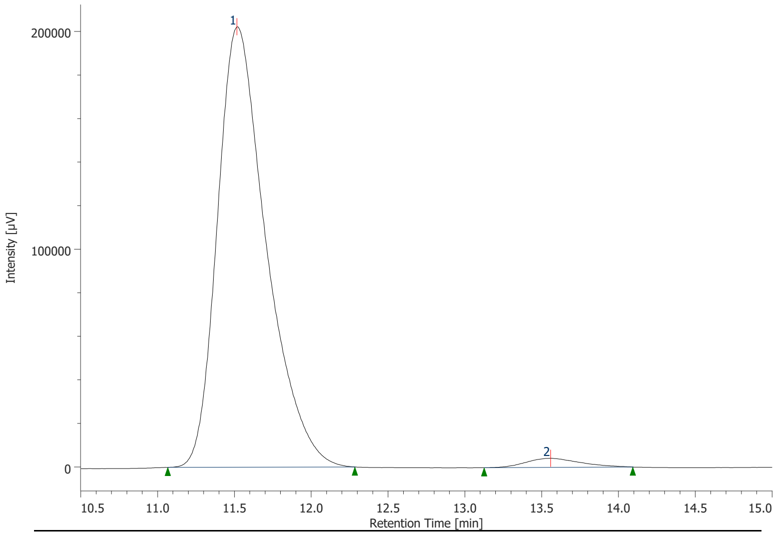

Peak No.	Retention Time (min)	Area (%)
1	56.783	99.978
2	61.467	0.022

(–)-{(1*R*,5*S*,7a*R*)-2-(4-Methylphenyl)-4-phenyl-1-vinyl-2,3,5,6,7,7a-hexahydro-1*H*-isoindol-5-yl}(pyrrolidin-1-yl)methanone [(–)-6ad (major diastereomer)]

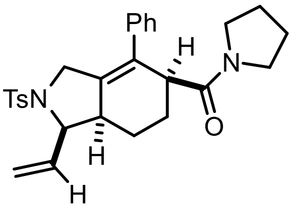

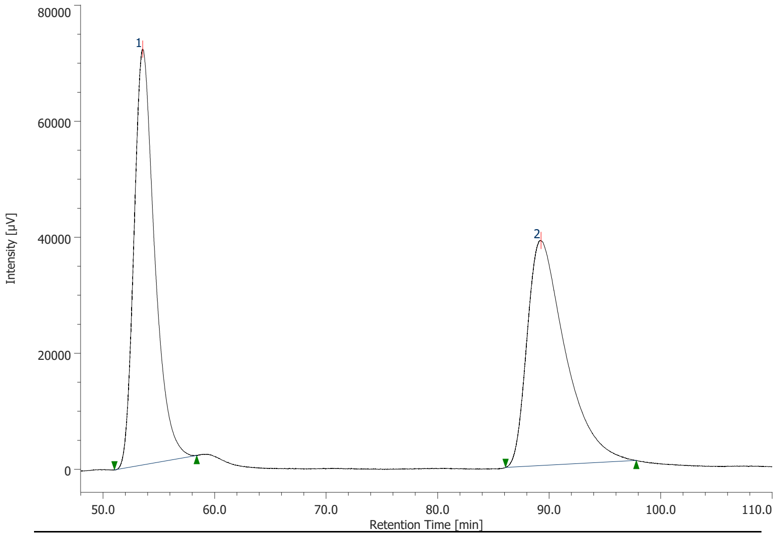

Peak No.	Retention Time (min)	Area (%)
1	53.550	49.976
2	89.267	50.024

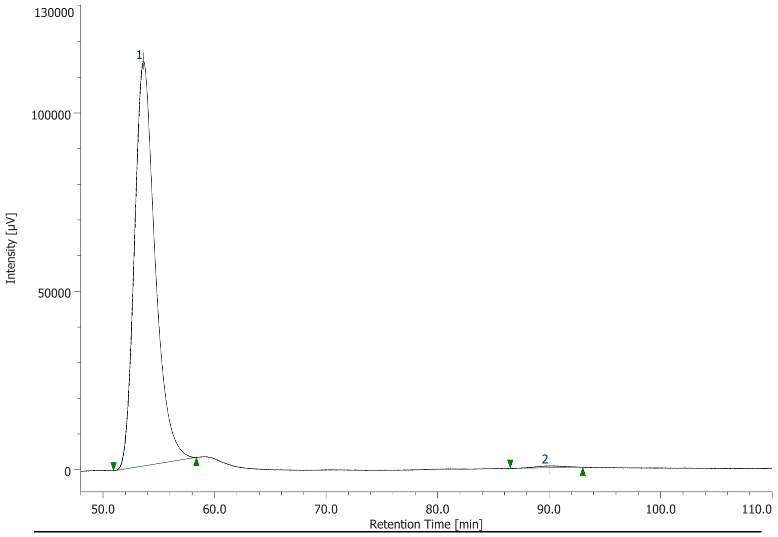

Peak No.	Retention Time (min)	Area (%)
1	53.617	99.394
2	89.992	0.606

(–)-(1*R*,5*S*,7a*R*)-*N*,*N*-Diethyl-2-(4-methylphenyl)-4-phenyl-1-vinyl-2,3,5,6,7,7a-hexahydro-1*H*-isoindole-5-carboxamide [(–)-6ah (major diastereomer)]

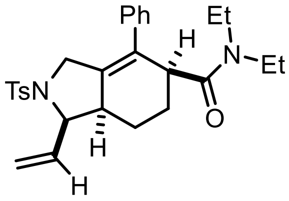

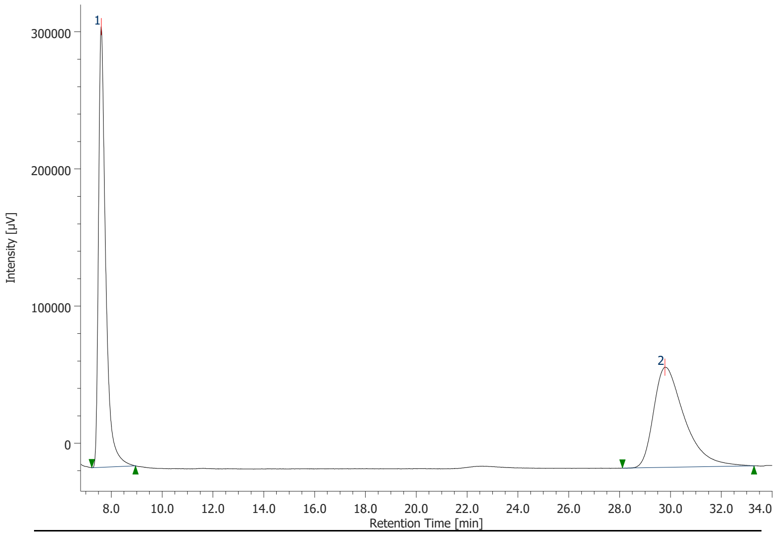

Peak No.	Retention Time (min)	Area (%)
1	7.608	50.582
2	29.775	49.418

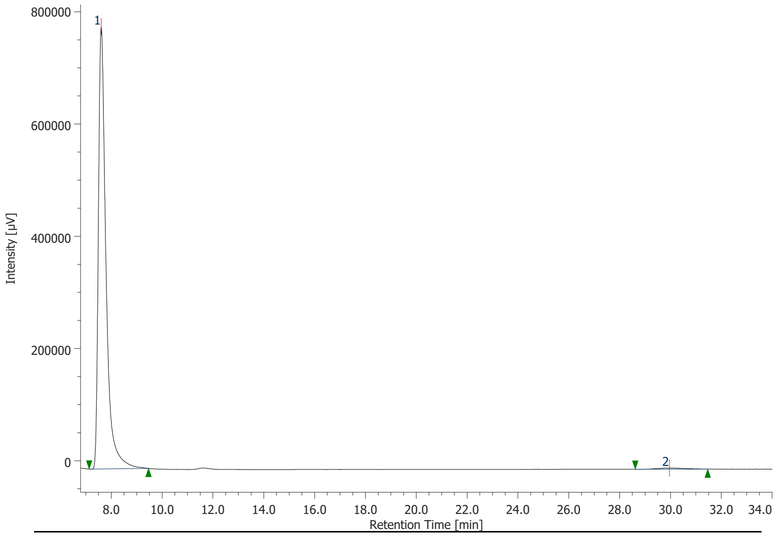

Peak No.	Retention Time (min)	Area (%)
1	7.608	99.076
2	29.958	0.924

(–)-(1*R*,5*S*,7a*R*)-*N*-Methyl-2-(4-methylphenyl)-*N*,4-diphenyl-1-vinyl-2,3,5,6,7,7a-hexahydro-1*H*-isoindole-5-carboxamide [(–)-6ag (major diastereomer)]

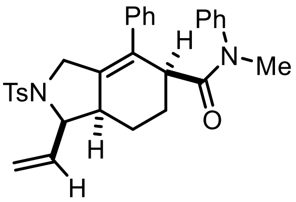

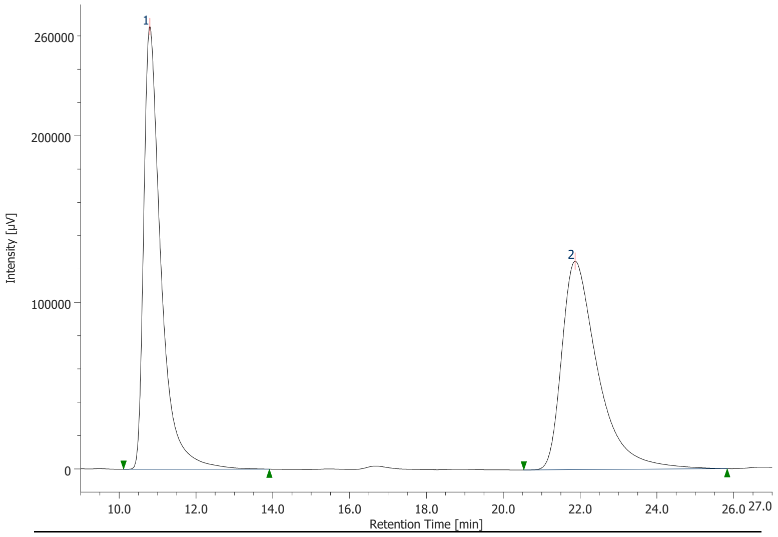

Peak No.	Retention Time (min)	Area (%)
1	10.800	50.431
2	21.867	49.569

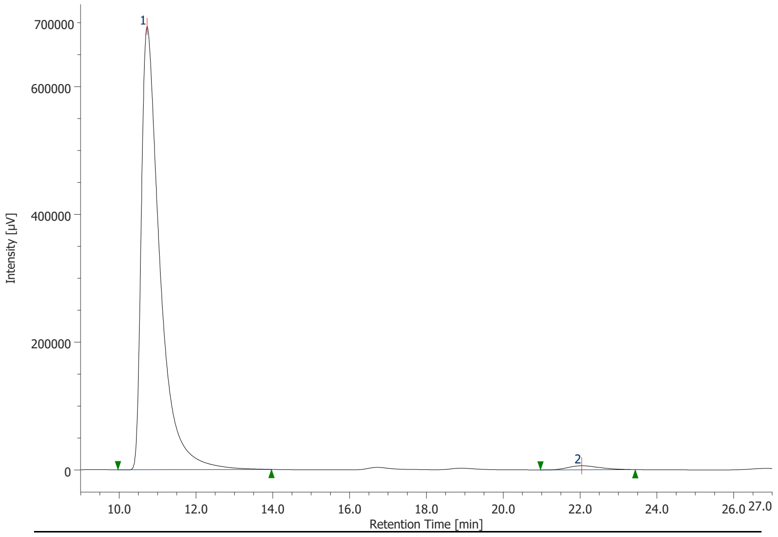

Peak No.	Retention Time (min)	Area (%)
1	10.725	98.474
2	22.042	1.526

(–)-(1*R*,5*S*,7a*R*)-*N*,*N*,4-Triphenyl-2-(4-methylphenyl)-1-vinyl-2,3,5,6,7,7a-hexahydro-1*H*-isoindole-5-carboxamide [(–)-6ak (major diastereomer)]

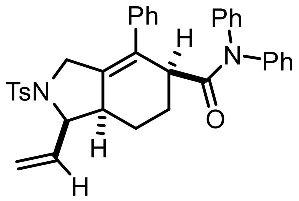

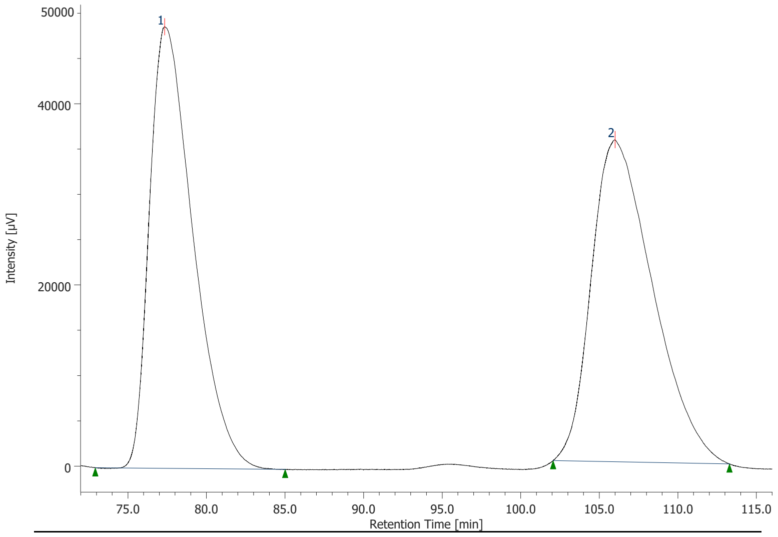

Peak No.	Retention Time (min)	Area (%)
1	77.342	49.354
2	105.992	50.646

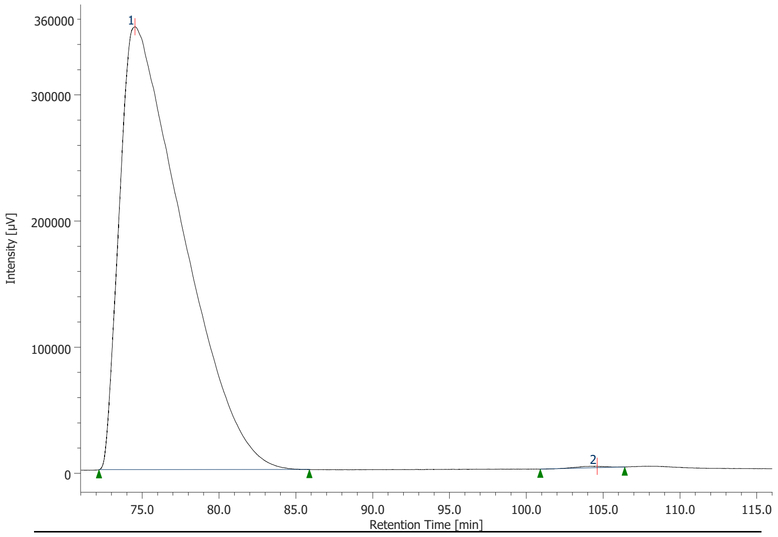

Peak No.	Retention Time (min)	Area (%)
1	74.633	99.848
2	104.600	0.152

(–)-{(1*R*,5*S*,7a*R*)-4-(4-Methoxyphenyl)-2-(4-methylphenyl)-1-vinyl-2,3,5,6,7,7a-hexahydro-1*H*-isoindol-5-yl}(pyrrolidin-1-yl)methanone [(–)-6bd (major diastereomer)]

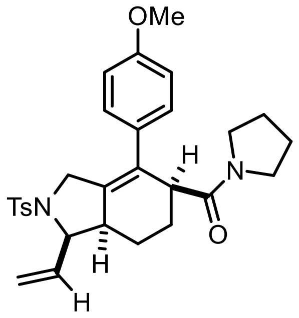

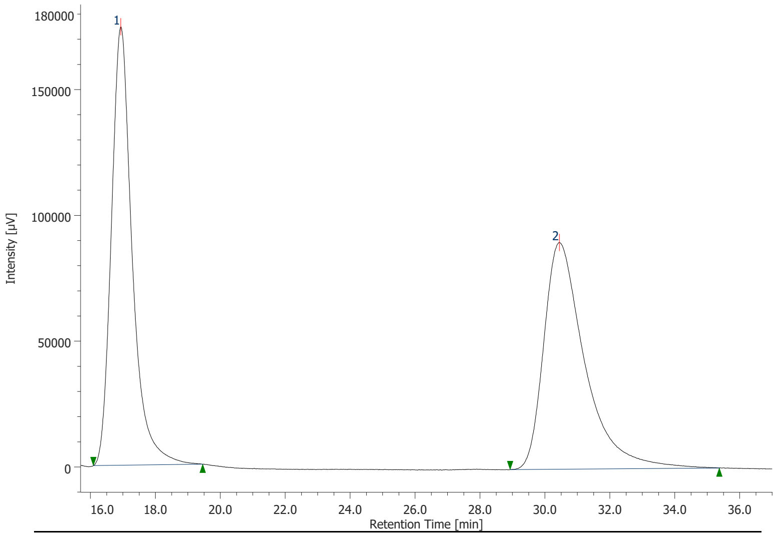

Peak No.	Retention Time (min)	Area (%)
1	16.933	49.532
2	30.442	50.468

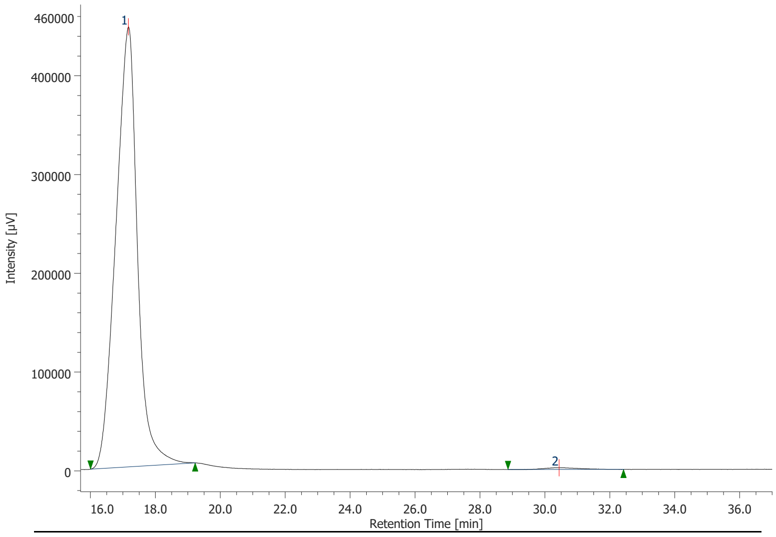

Peak No.	Retention Time (min)	Area (%)
1	17.167	99.389
2	30.433	0.611

(–)-{(1*R*,5*S*,7a*R*)-4-(4-Chlorophenyl)-2-(4-methylphenyl)-1-vinyl-2,3,5,6,7,7a-hexahydro-1*H*-isoindol-5-yl)(pyrrolidin-1-yl)methanone [(–)-6cd (major diastereomer)]

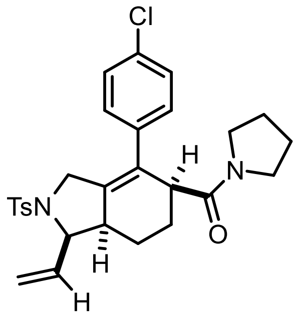

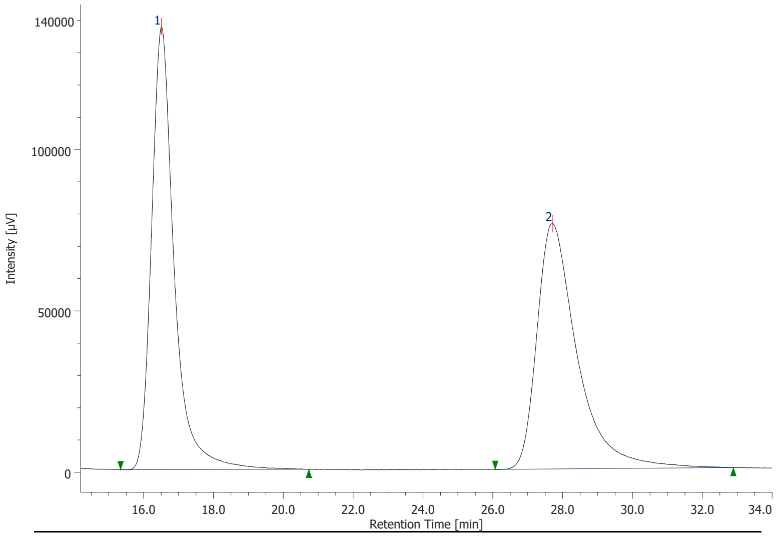

Peak No.	Retention Time (min)	Area (%)
1	16.508	50.416
2	27.717	49.584

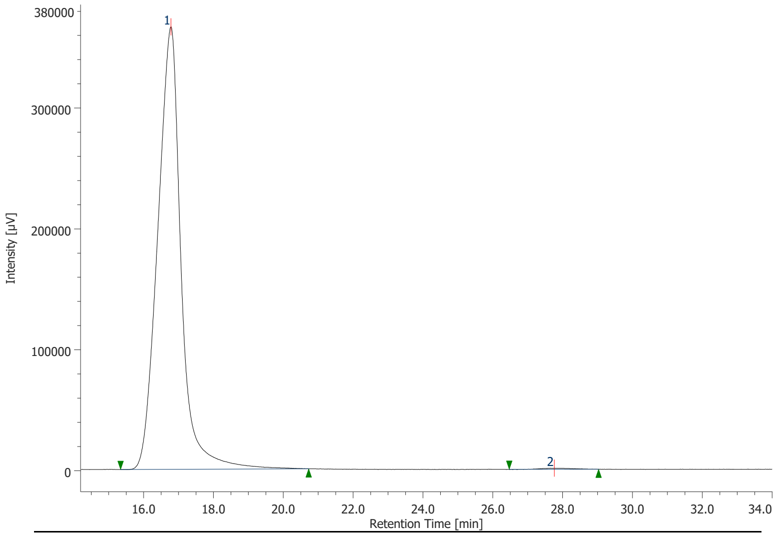

Peak No.	Retention Time (min)	Area (%)
1	16.783	99.679
2	27.758	0.321

(–)-(1*R*,5*S*,7a*R*)-4-(4-Bromophenyl)-2-(4-methylphenyl)-*N*,*N*-diphenyl-1-vinyl-2,3,5,6,7,7a-hexahydro-1*H*-isoindole-5-carboxamide [(–)-6dk]

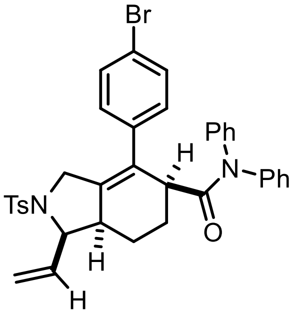

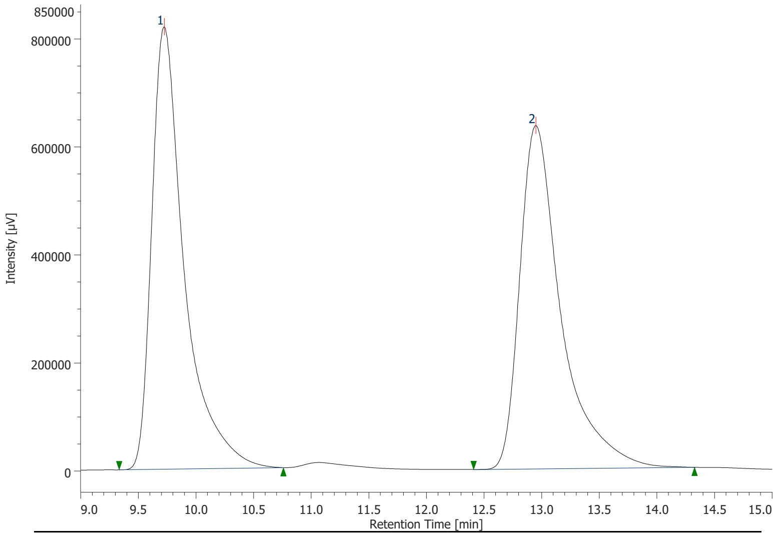

Peak No.	Retention Time (min)	Area (%)
1	9.725	50.420
2	12.950	49.580

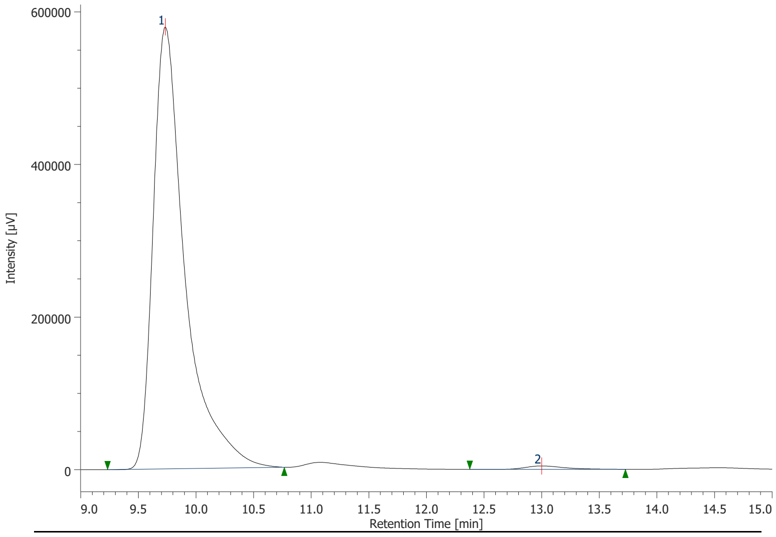

Peak No.	Retention Time (min)	Area (%)
1	9.733	99.077
2	13.000	0.923

(–)-{(1*R*,5*S*,7a*R*)-4-Methyl-2-(4-methylphenyl)-1-vinyl-2,3,5,6,7,7a-hexahydro-1*H*-isoindol-5-yl}(pyrrolidin-1-yl)methanone [(–)-6ed]

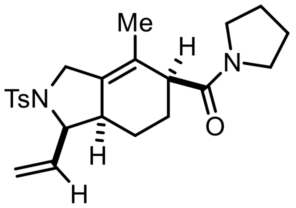

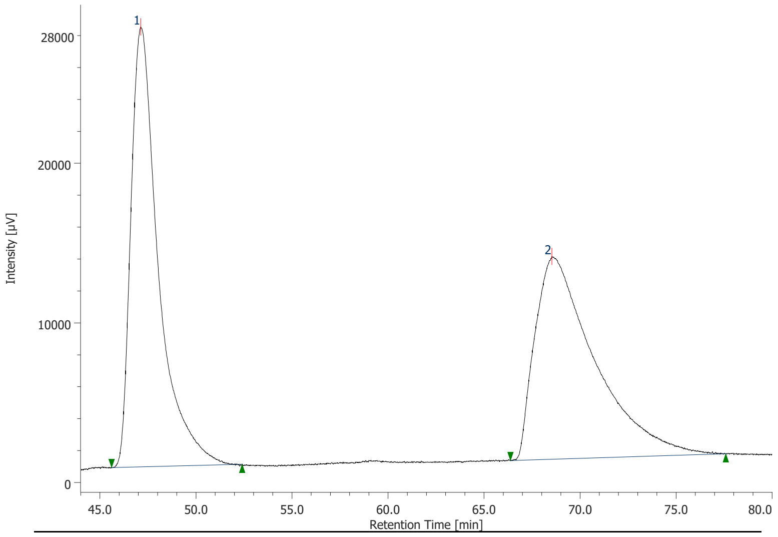

Peak No.	Retention Time (min)	Area (%)
1	47.125	49.987
2	68.525	50.013

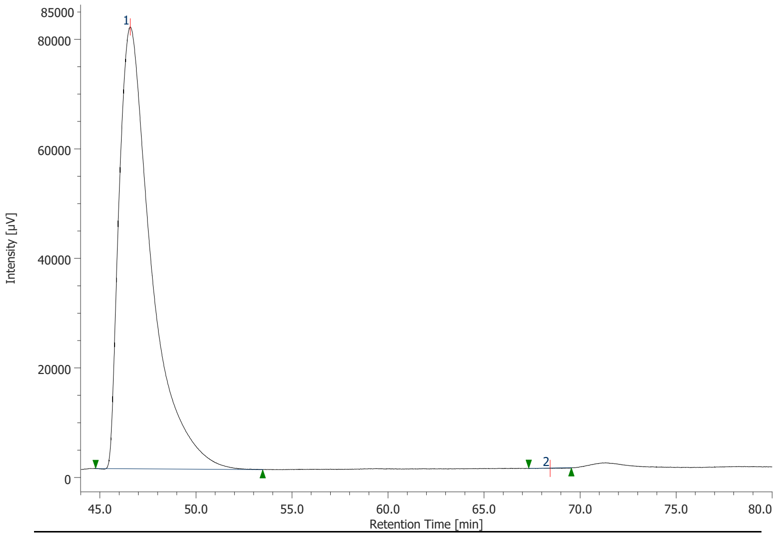

Peak No.	Retention Time (min)	Area (%)
1	46.567	99.932
2	68.433	0.068

(–)-{(1*R*,5*S*,7a*R*)-4-Butyl-2-(4-methylphenyl)-1-vinyl-2,3,5,6,7,7a-hexahydro-1*H*-isoindol-5-yl}(pyrrolidin-1-yl)methanone [(–)-6fd]

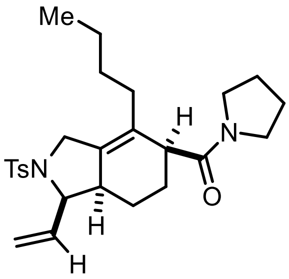

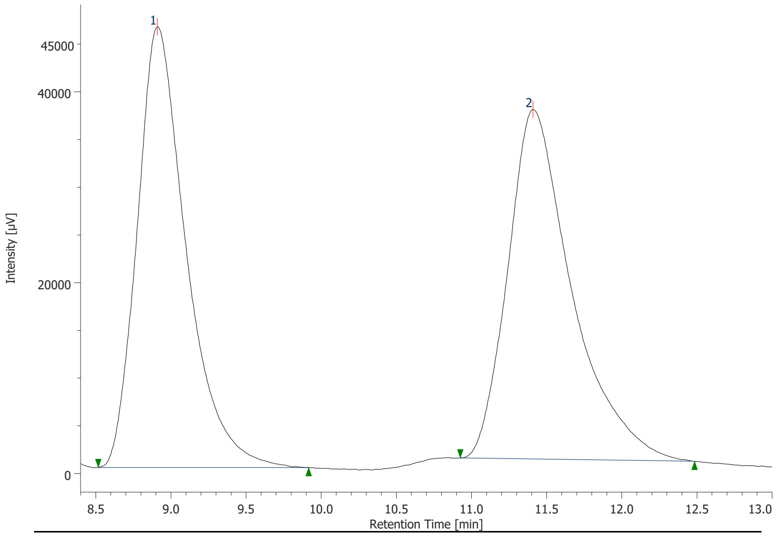

Peak No.	Retention Time (min)	Area (%)
1	8.908	49.120
2	11.408	50.880

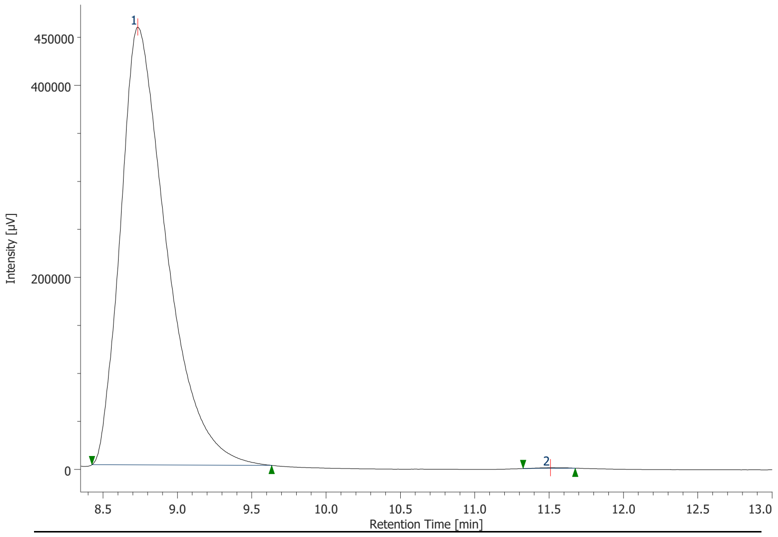

Peak No.	Retention Time (min)	Area (%)
1	8.733	99.912
2	11.508	0.088

(–)-{(1*R*,5*R*,7a*R*)-5-Fluoro-4-phenyl-1-vinyl-1,3,5,6,7,7a-hexahydroisobenzofuran-5-yl}(pyrrolidin-1-yl)methanone [(–)-6ga]

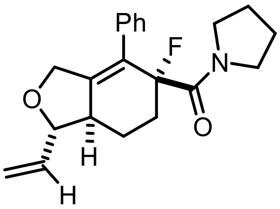

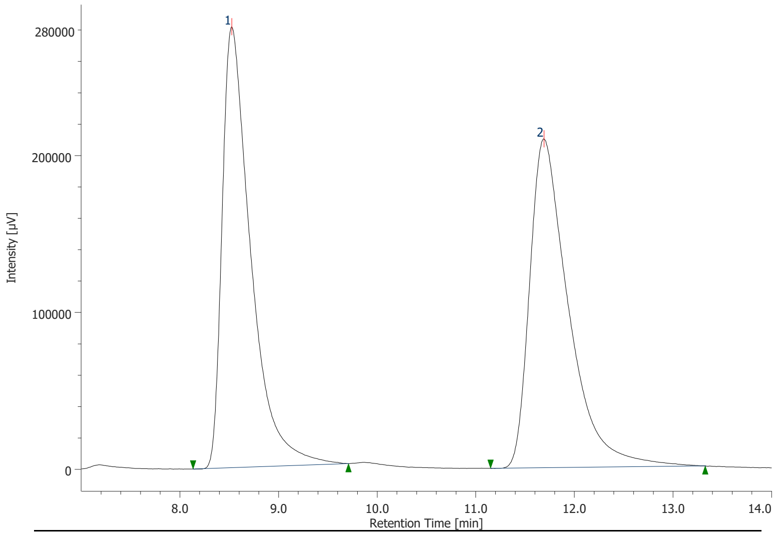

Peak No.	Retention Time (min)	Area (%)
1	8.525	49.678
2	11.692	50.332

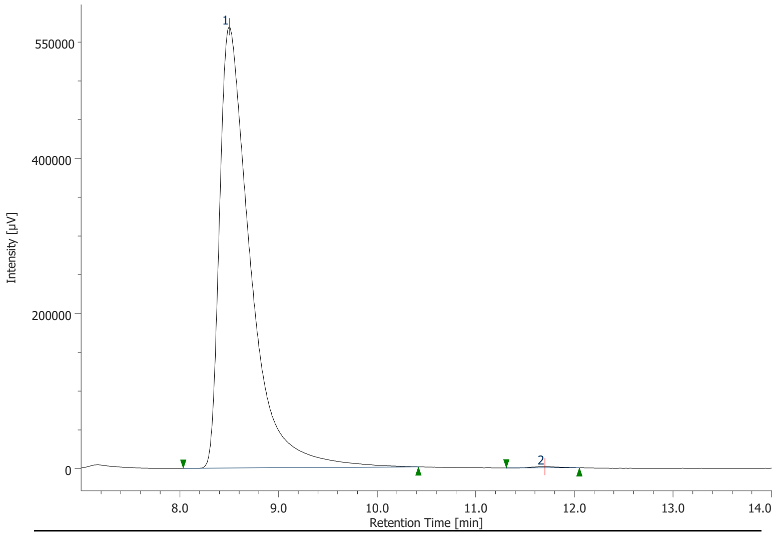

Peak No.	Retention Time (min)	Area (%)
1	8.500	99.754
2	11.700	0.246

(–)-(1*S*,5*S*,7a*R*)-*N*-Methyl-*N*,4-diphenyl-1-vinyl-1,3,5,6,7,7a-hexahydroisobenzofuran-5-carboxamide [(–)-6gg]

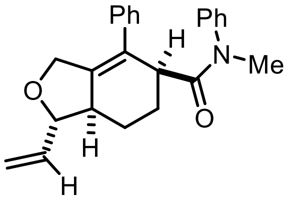

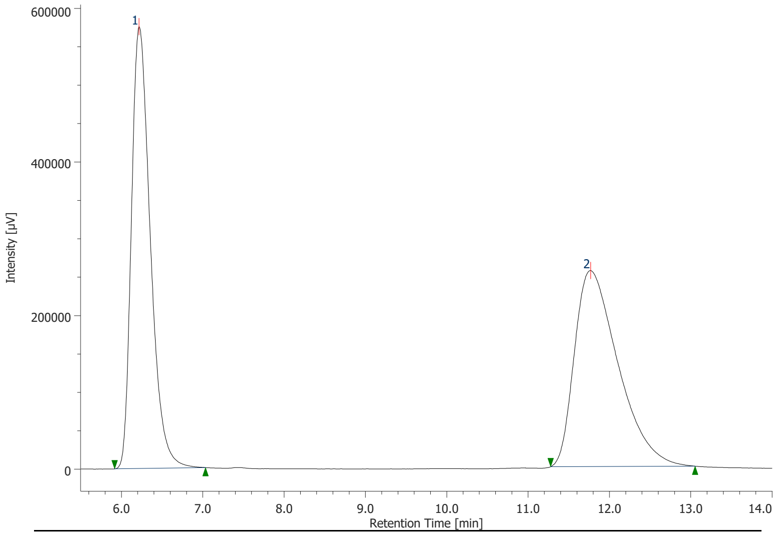

Peak No.	Retention Time (min)	Area (%)
1	6.217	49.441
2	11.767	50.559

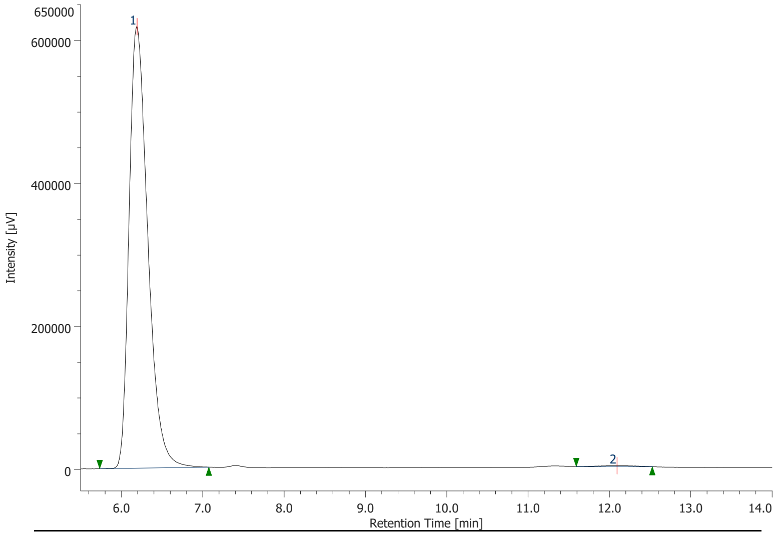

Peak No.	Retention Time (min)	Area (%)
1	6.192	99.591
2	12.092	0.409

(–)-(*E*)-4-{(2*R*,3*R*)-4-(*Z*)-Benzylidene-3-methyl-1-(4-methylphenyl)-2-(prop-1-en-2-yl) pyrrolidin-3-yl}-2-fluoro-1-(pyrrolidin-1-yl)but-2-en-1-one [(–)-7ha (major diastereomer)]

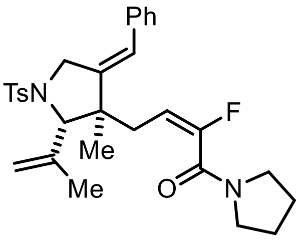

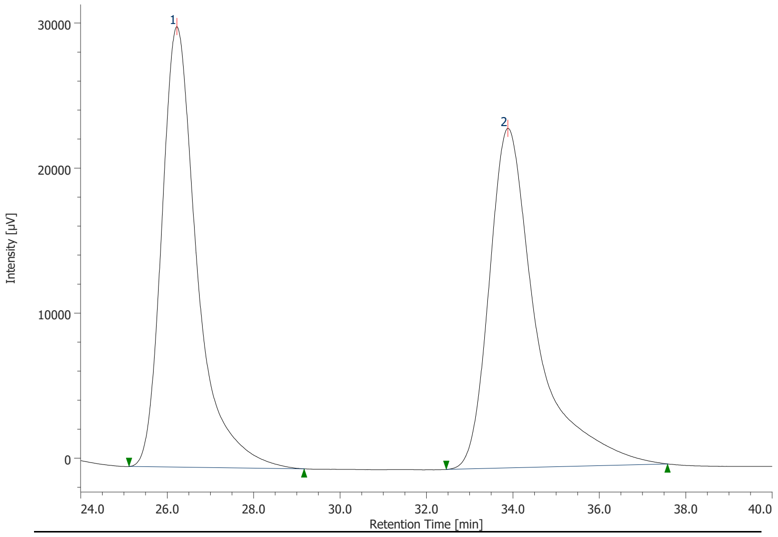

Peak No.	Retention Time (min)	Area (%)
1	26.225	49.239
2	33.883	50.761

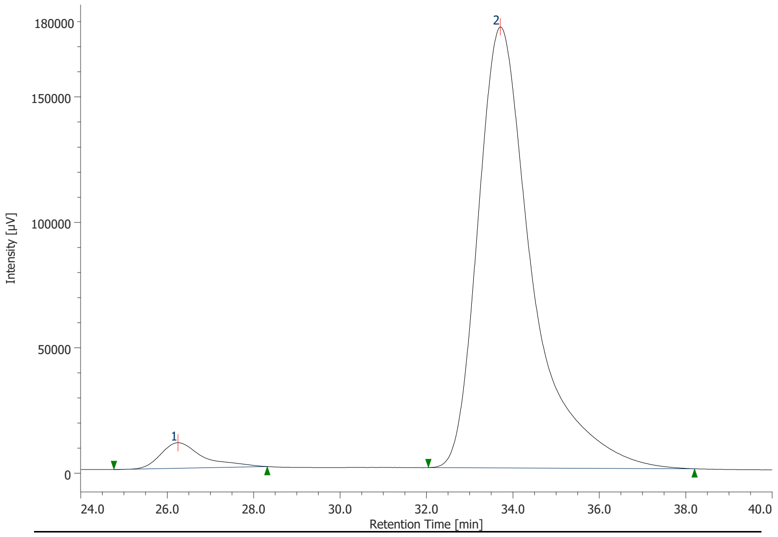

Peak No.	Retention Time (min)	Area (%)
1	26.250	4.357
2	33.708	95.643

(–)-(*E*)-4-[(2*R*,3*R*)-4-{(*Z*)-4-Bromobenzylidene}-3-methyl)-1-(4-methylphenyl)-2-(prop-1-en-2-ylpyrrolidin-3-yl]-2-fluoro-1-(pyrrolidin-1-yl)but-2-en-1-one [(–)-7ia (major diastereomer)]

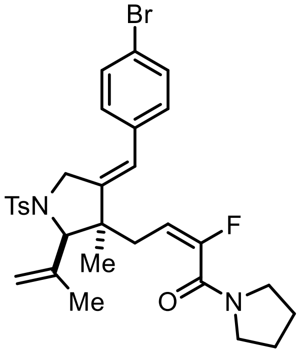

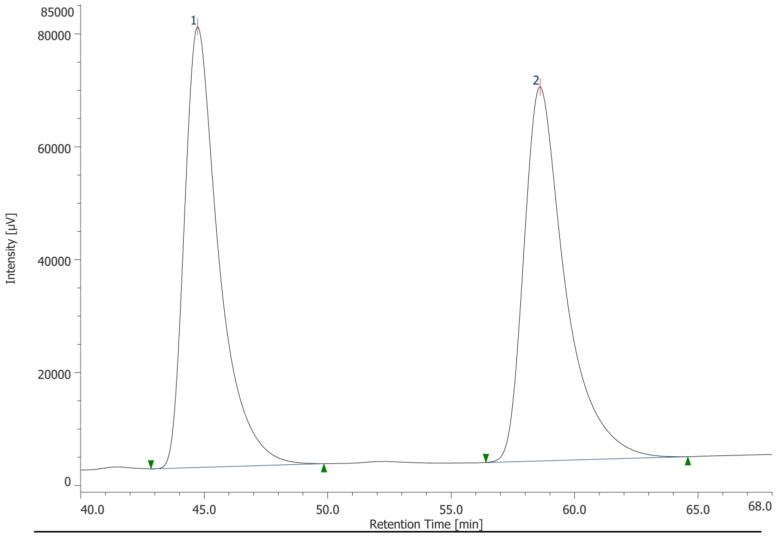

Peak No.	Retention Time (min)	Area (%)
1	44.550	49.482
2	58.250	50.518

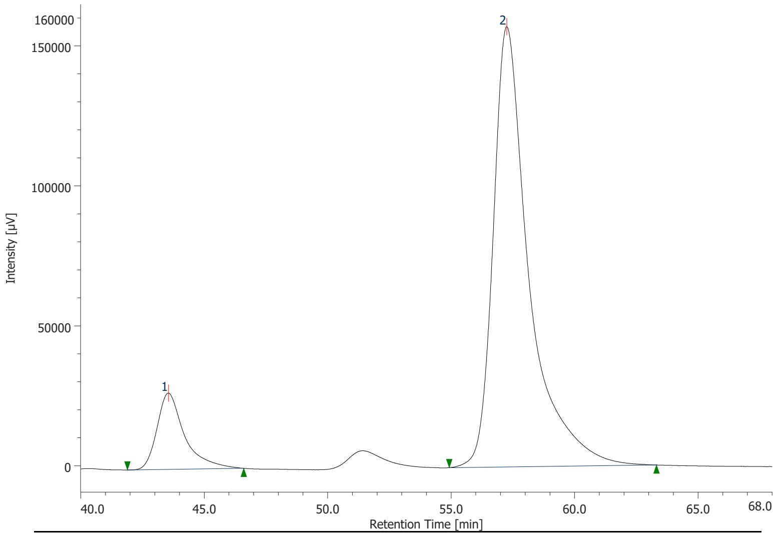

Peak No.	Retention Time (min)	Area (%)
1	43.733	11.751
2	57.600	88.249

(–)-(*E*)-4-{(2*S*,3*R*,*Z*)-4-Ethylidene-3-methyl-1-(4-methylphenyl)-2-(prop-1-en-2-yl) pyrrolidin-3-yl}-2-fluoro-1-(pyrrolidin-1-yl)but-2-en-1-one [(–)-7ja (major diastereomer)]

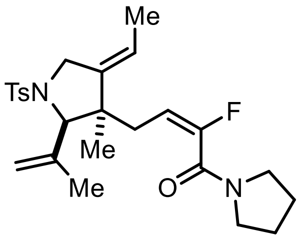

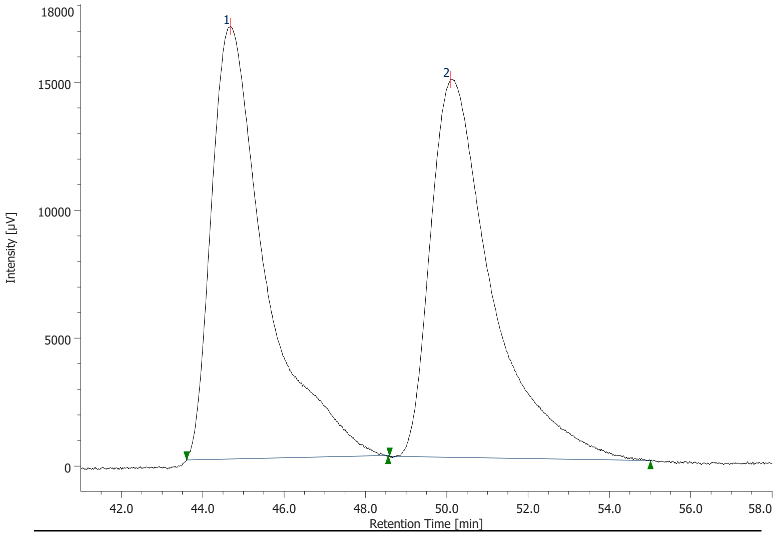

Peak No.	Retention Time (min)	Area (%)
1	44.683	50.256
2	50.083	49.744

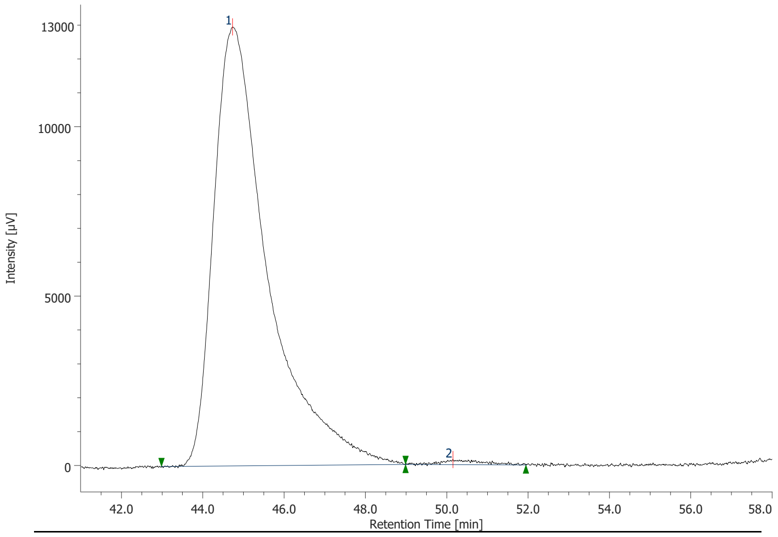

Peak No.	Retention Time (min)	Area (%)
1	44.733	99.141
2	50.150	0.859

(–)-(*Z*)-4-[(2*R*,3*R*)-4-{(*Z*)-4-Bromobenzylidene}-3-methyl-1-(4-methylphenyl)-2-(prop-1-en-2-yl)pyrrolidin-3-yl]-2-methyl-1-(pyrrolidin-1-yl)but-2-en-1-one [(–)-7ie (major diastereomer)]

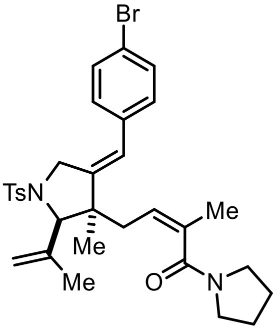

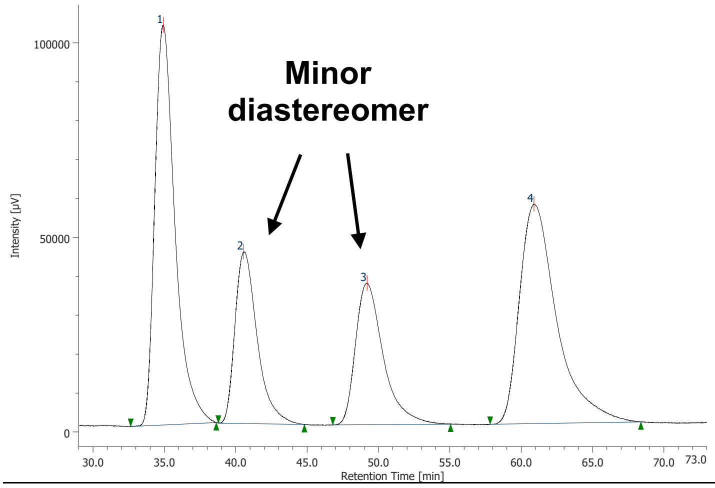

Peak No.	Retention Time (min)	Area (%)
**1**	**34.933**	**33.716**
2	40.550	15.899
3	49.192	16.365
**4**	**60.883**	**34.020**

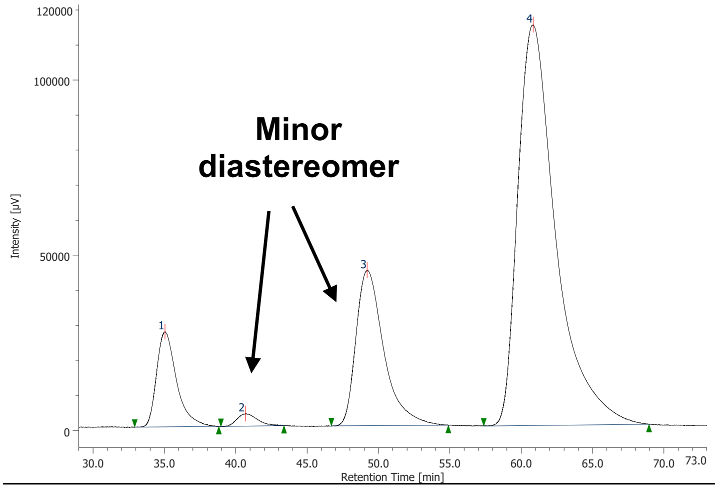

Peak No.	Retention Time (min)	Area (%)
**1**	**35.042**	**8.728**
2	40.667	1.160
3	49.192	19.939
**4**	**60.825**	**70.173**

(–)-(*E*)-4-[(2*R*,3*R*)-4-{(*Z*)-4-Bromobenzylidene}-3-methyl-1-(4-methylphenyl)-2-(prop-1-en-2-yl)pyrrolidin-3-yl]-2-chloro-1-(pyrrolidin-1-yl)but-2-en-1-one [(–)-7if (major diastereomer)]

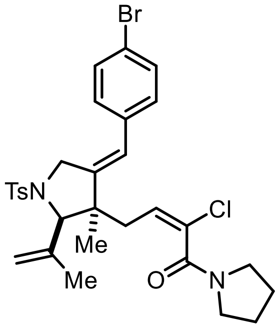

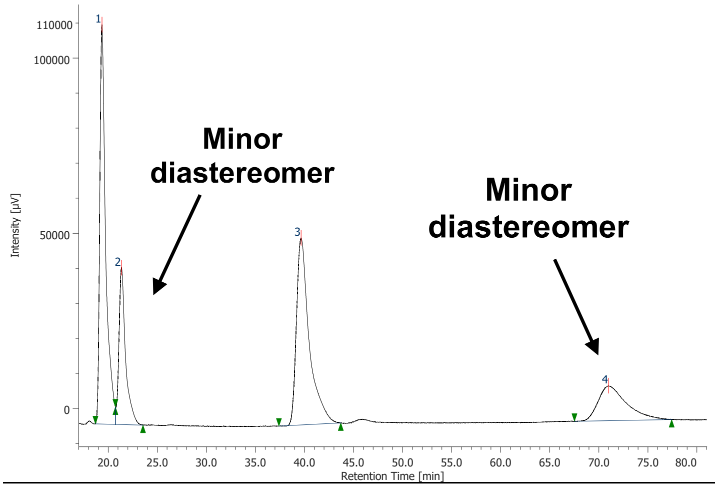

Peak No.	Retention Time (min)	Area (%)
**1**	**19.367**	**35.023**
2	21.358	15.312
**3**	**39.650**	**35.163**
4	70.975	14.502

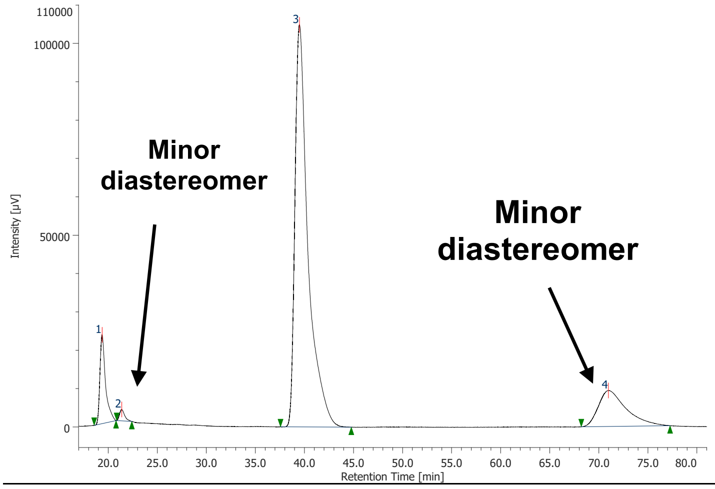

Peak No.	Retention Time (min)	Area (%)
**1**	**19.383**	**7.347**
2	21.392	0.858
**3**	**39.492**	**77.091**
4	70.967	14.703

(+)-(4*R*,5*R*)-3-(*Z*)-Benzylidene-4-{(*E*)-3-fluoro-4-oxo-4-(pyrrolidin-1-yl)but-2-en-1-yl}-4-methyl-5-(prop-1-en-2-yl)dihydrofuran-2(3*H*)-one [(+)-7ka]

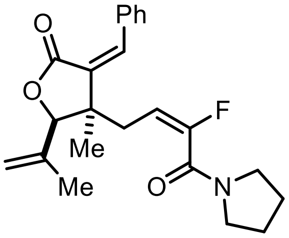

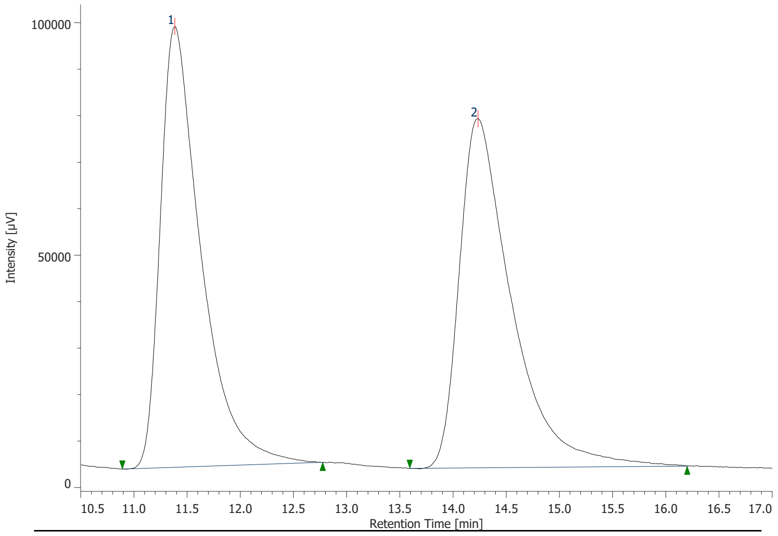

Peak No.	Retention Time (min)	Area (%)
1	11.383	49.555
2	14.233	50.445

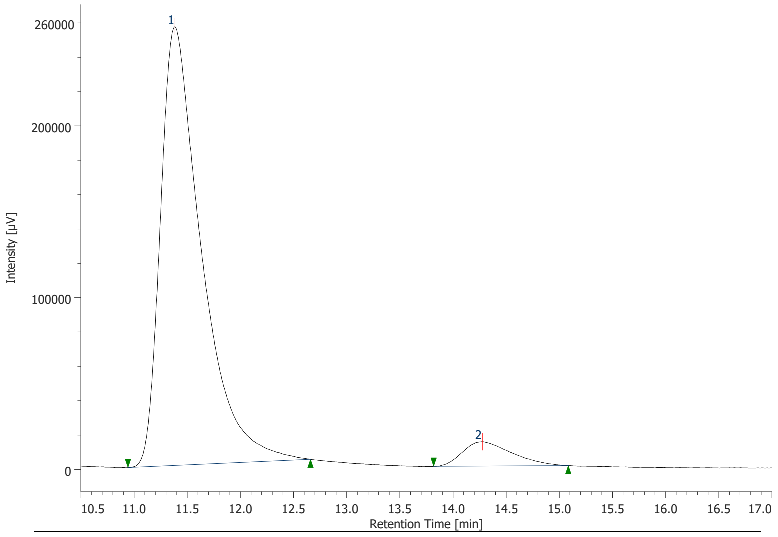

Peak No.	Retention Time (min)	Area (%)
1	11.383	93.798
2	14.275	6.202

7.4. Synthetic Applications (Figure 5a, 5b)

(+)-(3*S*,3a*R*,6*R*,7*S*,7a*S*)-6-Fluoro-3,3a-dimethyl-7-phenyl-6-(pyrrolidine-1-carbonyl) hexahydroisobenzofuran-1(3*H*)-one [(+)-8]

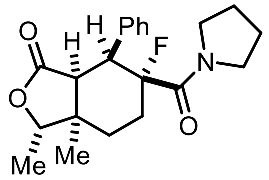

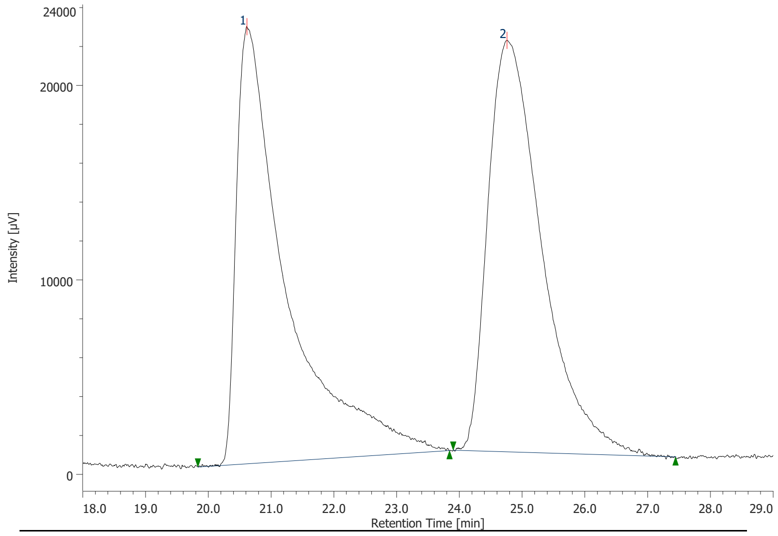

Peak No.	Retention Time (min)	Area (%)
1	20.617	49.463
2	24.758	50.537

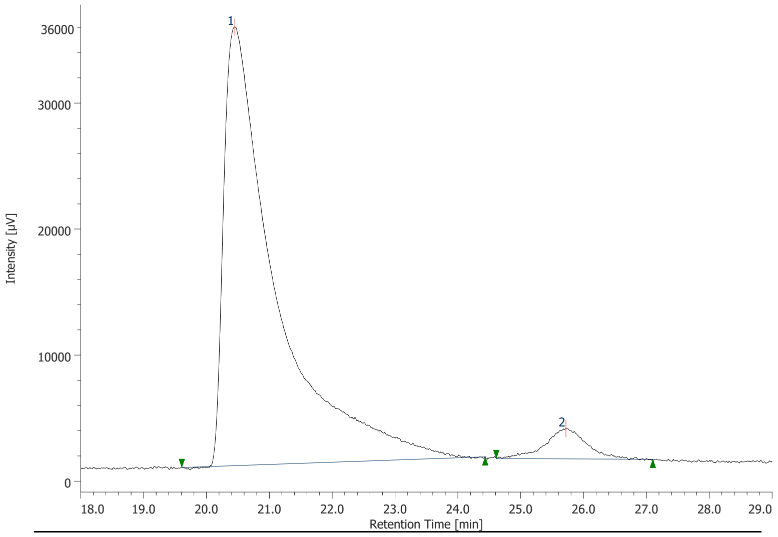

Peak No.	Retention Time (min)	Area (%)
1	20.450	94.727
2	25.717	5.273

(–)-(4*S*,5*R*)-3-Benzyl-4-{(*E*)-3-fluoro-4-oxo-4-(pyrrolidin-1-yl)but-2-en-1-yl}-4,5-dimethyldihydrofuran-2(3*H*)-one [(–)-9 (major diastereomer)]

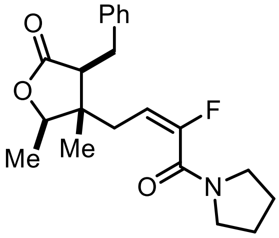

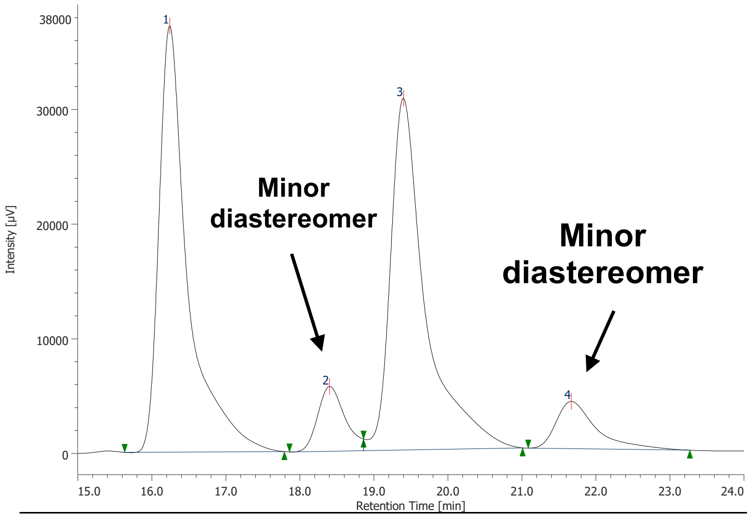

Peak No.	Retention Time (min)	Area (%)
**1**	**16.242**	**43.616**
2	18.400	6.208
**3**	**19.400**	**43.466**
4	21.667	6.710

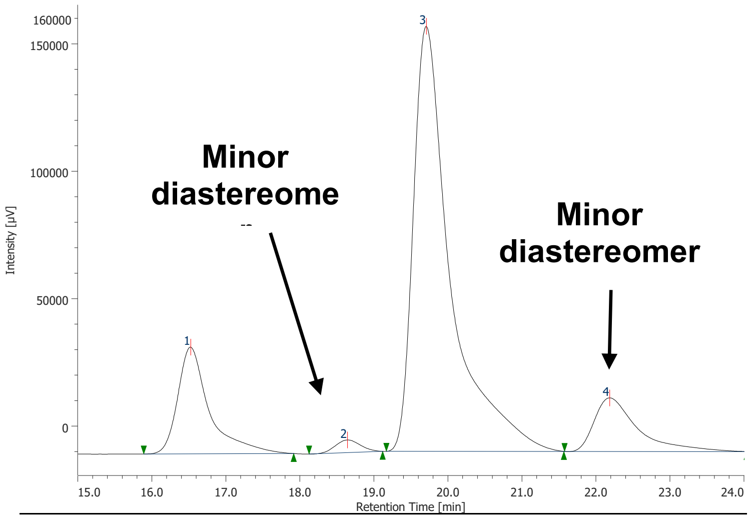

Peak No.	Retention Time (min)	Area (%)
**1**	**16.525**	**15.426**
2	18.642	1.451
**3**	**19.708**	**72.491**
4	22.183	10.631

(–)-(3*S*,4*R*)-2-(*Z*)-Benzylidene-3-{(*E*)-3-fluoro-4-(pyrrolidin-1-yl)but-2-en-1-yl}-3-methylpentane-1,4-diol [(–)-10]

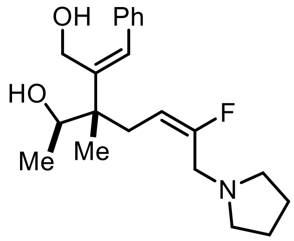

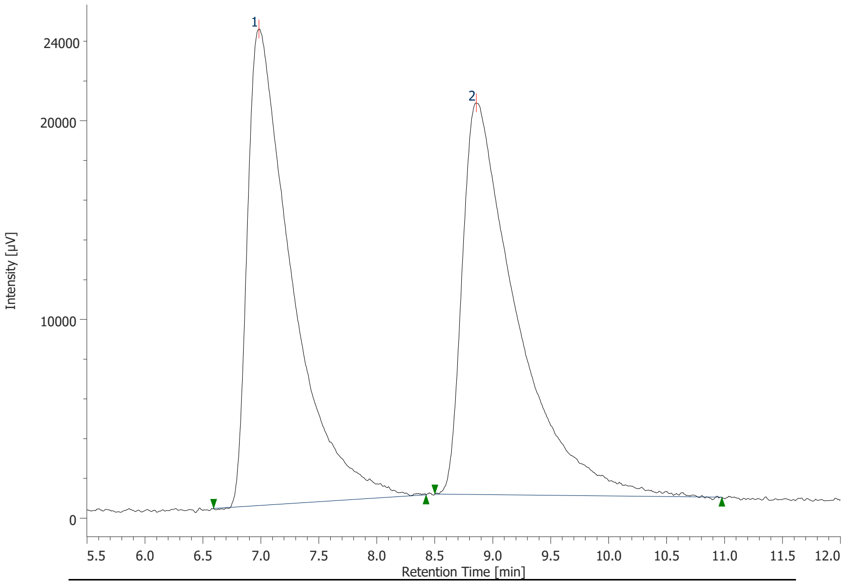

Peak No.	Retention Time (min)	Area (%)
1	6.983	49.853
2	8.858	50.147

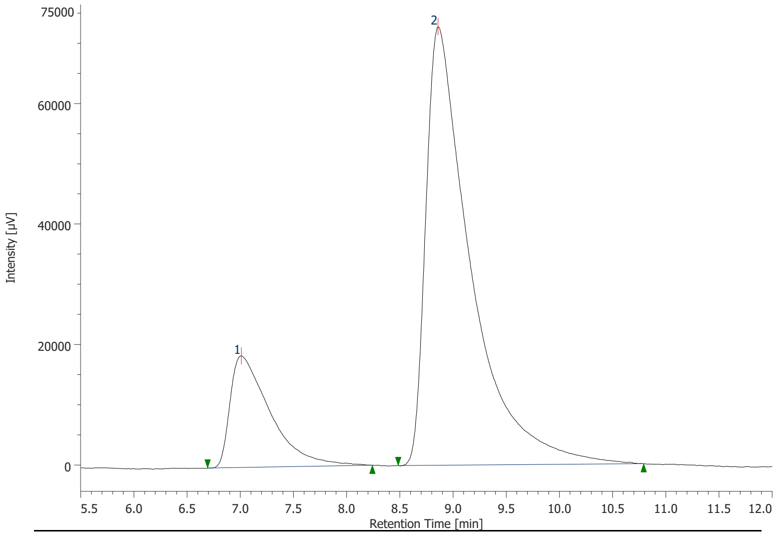

Peak No.	Retention Time (min)	Area (%)
1	7.008	18.474
2	8.858	81.526

(–)-{(1*R*,5*R*,7a*R*)-5-Fluoro-2-(2-iodobenzoyl)-4-phenyl-1-vinyl-2,3,5,6,7,7a-hexahydro-1*H*-isoindol-5-yl}(pyrrolidin-1-yl)methanone [(–)-11 (major diastereomer)]

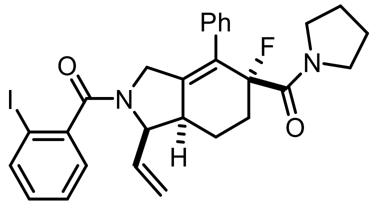

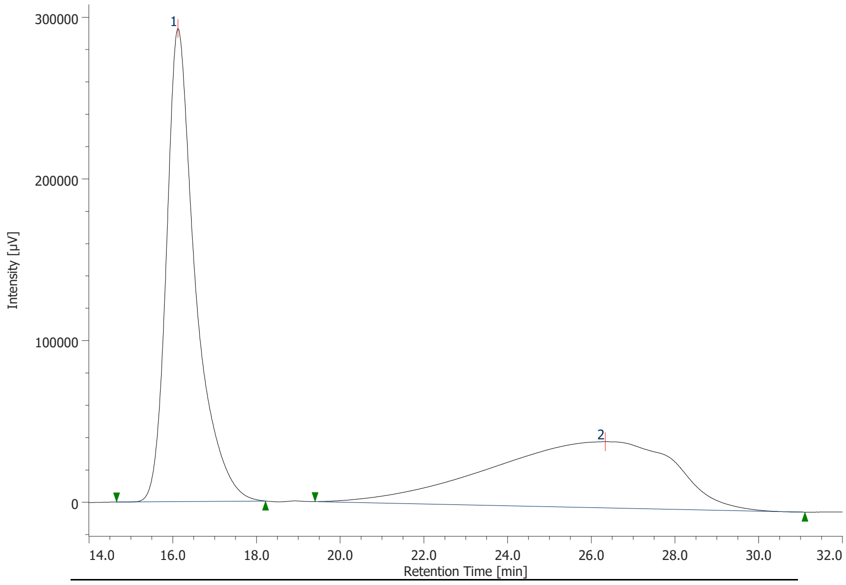

Peak No.	Retention Time (min)	Area (%)
1	16.125	52.810
2	26.333	47.190

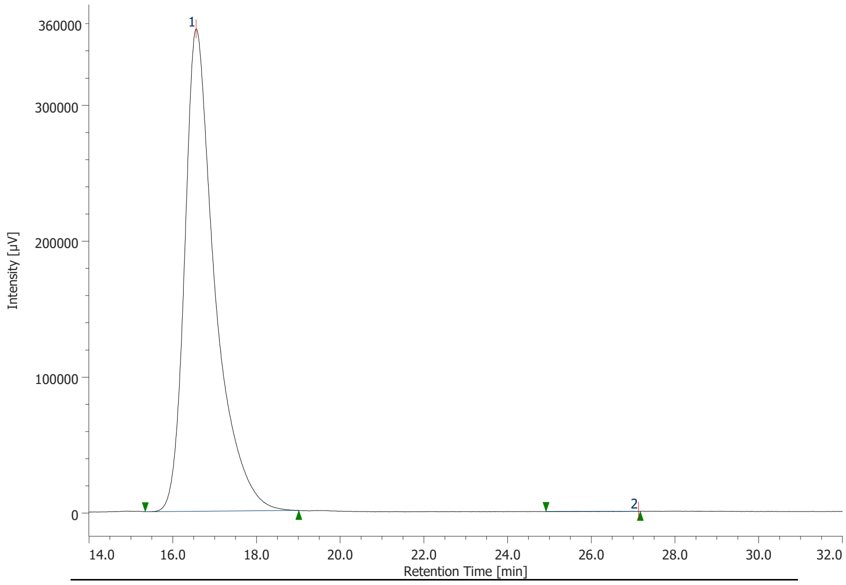

Peak No.	Retention Time (min)	Area (%)
1	16.558	99.980
2	27.125	0.020

(–)-(9*R*,11a*R*,11b*S*)-9-Fluoro-12-methylene-8-phenyl-9-(pyrrolidine-1-carbonyl)-9,10,11,11a,11b,12-hexahydroisoindolo[2,1-b]isoquinolin-5(7*H*)-one [(–)-12]

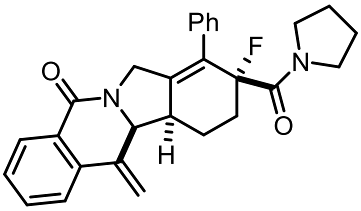

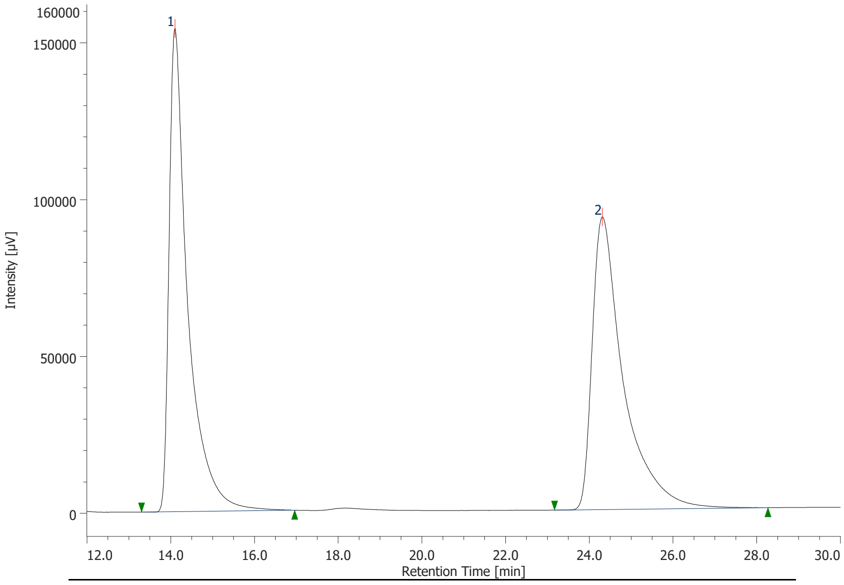

Peak No.	Retention Time (min)	Area (%)
1	14.100	49.822
2	24.308	50.178

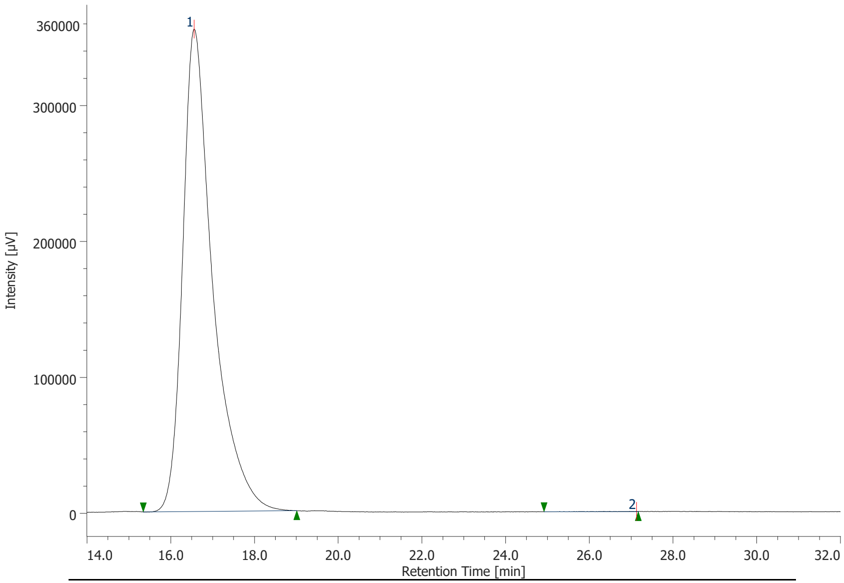

Peak No.	Retention Time (min)	Area (%)
1	14.000	99.915
2	24.592	0.085

7.5. Experimental Mechanistic Studies (Figure 5c)

(–)-{(1*S*,5*R*,7a*R*)-5-Fluoro-1,7a-dimethyl-2-(4-methylphenyl)-4-phenyl-2,3,5,6,7,7a-hexahydro-1*H*-isoindol-5-yl-6,6-*d*₂}(pyrrolidin-1-yl)methanone [(–)-3da-D₂]

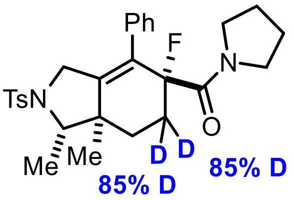

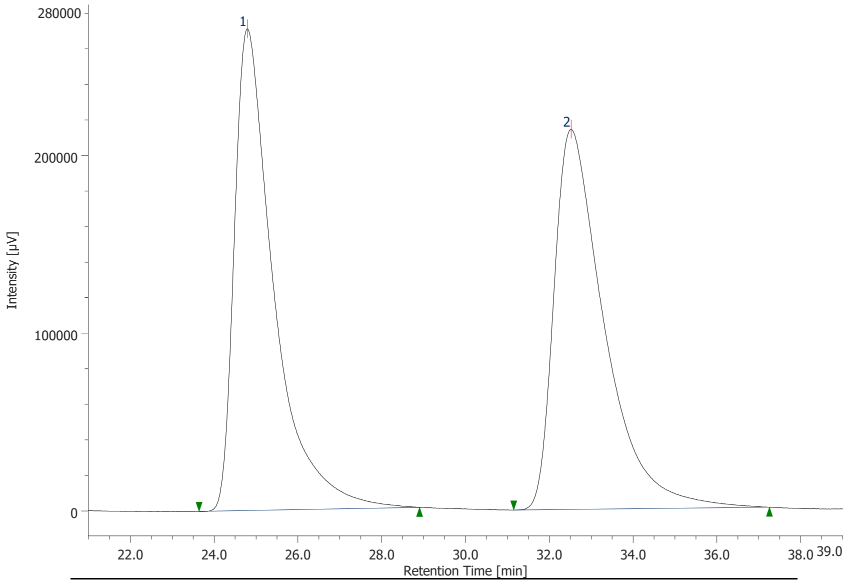

Peak No.	Retention Time (min)	Area (%)
1	24.792	49.838
2	32.517	50.162

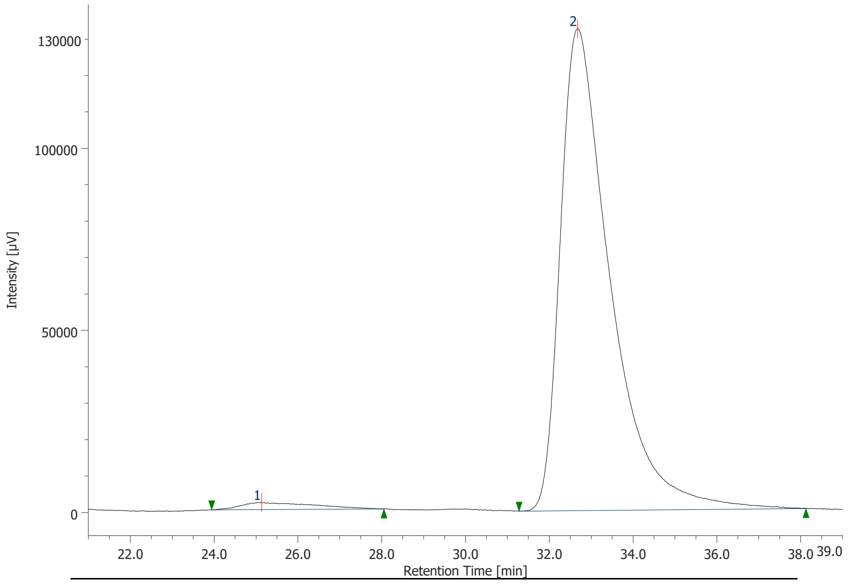

Peak No.	Retention Time (min)	Area (%)
1	25.133	2.076
2	32.675	97.924

(–)-(*E*)-4-{(2*S*,3*R*,*Z*)-2,3-Dimethyl-1-(4-methylphenyl)-4-(phenylmethylene-*d*)pyrrolidin-3-yl}-2-fluoro-1-(pyrrolidin-1-yl)but-2-en-1-one-3-*d* [(–)-4da-D$_2$ (major diastereomer)]

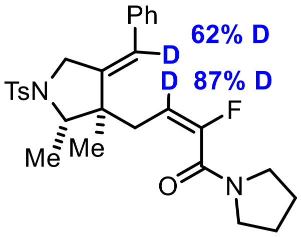

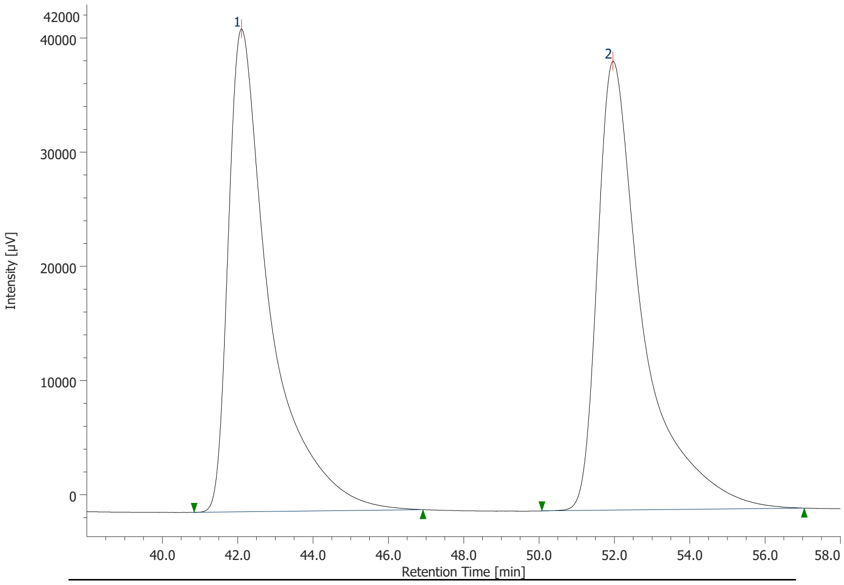

Peak No.	Retention Time (min)	Area (%)
1	42.100	49.632
2	51.958	50.368

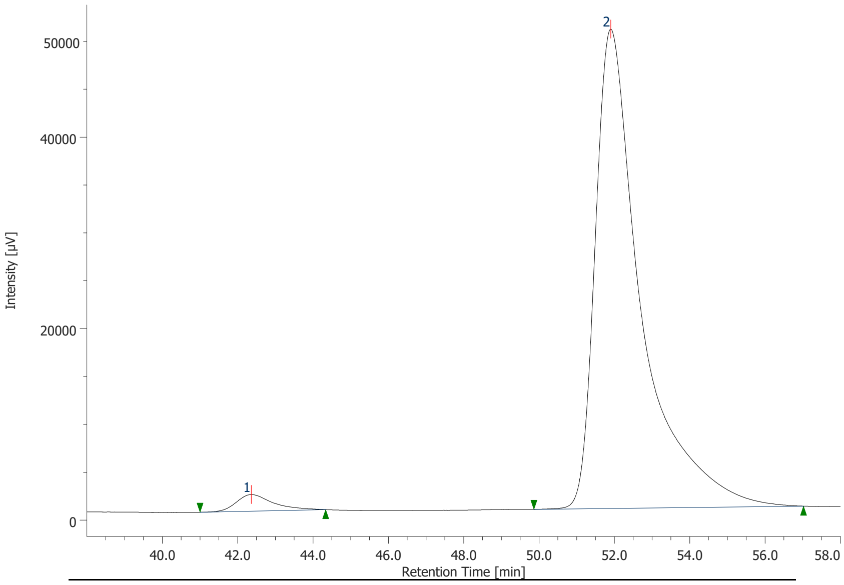

Peak No.	Retention Time (min)	Area (%)
1	42.358	2.704
2	51.900	97.296

(–)-(3*S*,3a*R*,6*R*)-6-Fluoro-3,3a-dimethyl-7-phenyl-6-(pyrrolidine-1-carbonyl)-3a,4,5,6-tetrahydroisobenzofuran-1(3*H*)-one-5,5-d_2 [(–)-3ha-D_2]

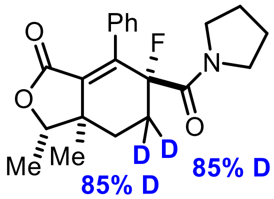

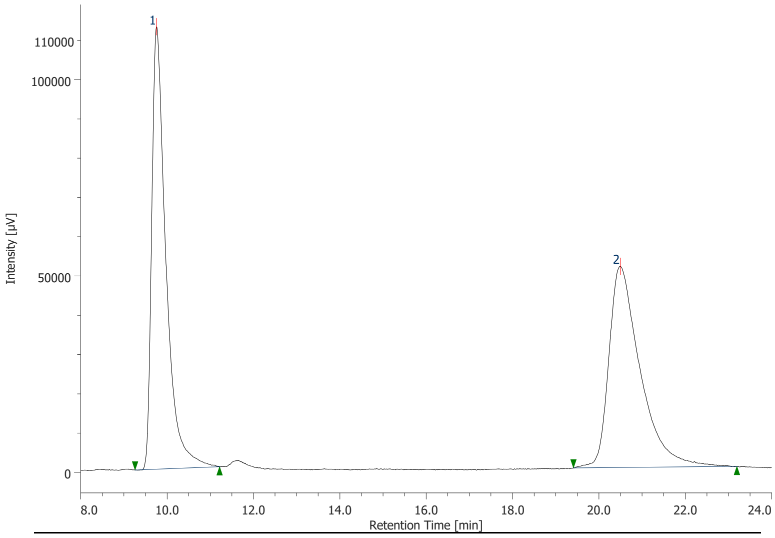

Peak No.	Retention Time (min)	Area (%)
1	9.758	49.931
2	20.492	50.069

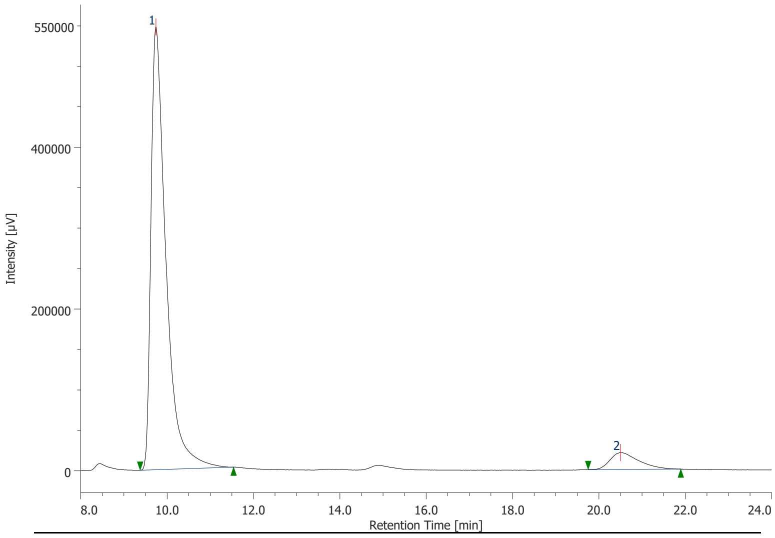

Peak No.	Retention Time (min)	Area (%)
1	9.742	93.221
2	20.500	6.779

(+)-(4*R*,5*S*,*Z*)-4-{(*E*)-3-Fluoro-4-oxo-4-(pyrrolidin-1-yl)but-2-en-1-yl-2-*d*}-4,5-dimethyl-3-(phenylmethylene-*d*)dihydrofuran-2(3*H*)-one [(+)-4ha-D$_2$]

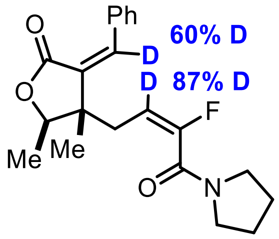

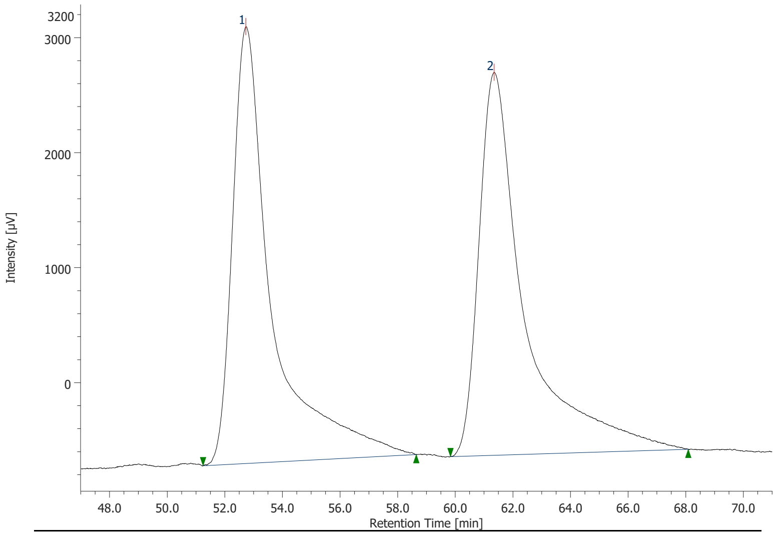

Peak No.	Retention Time (min)	Area (%)
1	52.733	49.490
2	61.350	50.510

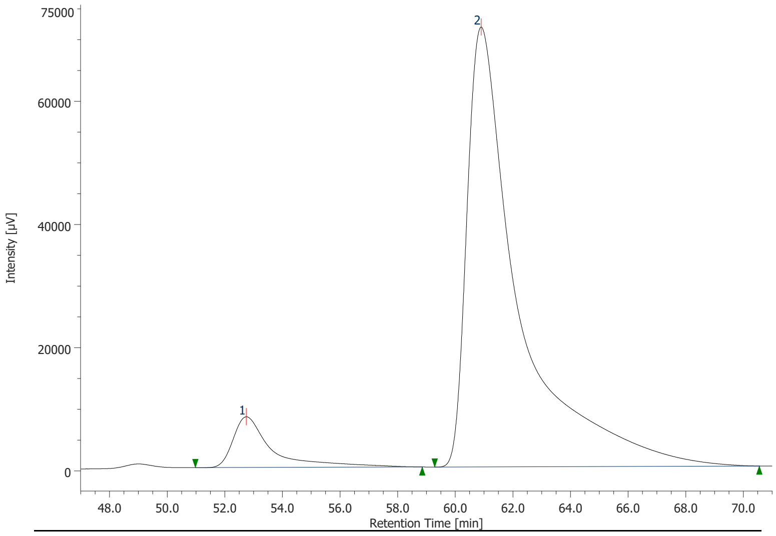

Peak No.	Retention Time (min)	Area (%)
1	52.742	7.990
2	60.900	92.010
